# Genomic analyses of gynaecologic carcinosarcomas reveal frequent mutations in chromatin remodelling genes

**DOI:** 10.1038/ncomms6006

**Published:** 2014-09-19

**Authors:** Siân Jones, Nicolas Stransky, Christine L. McCord, Ethan Cerami, James Lagowski, Devon Kelly, Samuel V. Angiuoli, Mark Sausen, Lisa Kann, Manish Shukla, Rosemary Makar, Laura D. Wood, Luis A. Diaz, Christoph Lengauer, Victor E. Velculescu

**Affiliations:** 1 Personal Genome Diagnostics, Baltimore, Maryland 21224, USA; 2 Blueprint Medicines, Cambridge, Massachusetts 02142, USA; 3 Knight Cancer Institute, Oregon Health and Science University, Portland, Oregon 97239, USA; 4 Department of Pathology, Johns Hopkins University, Baltimore, Maryland 21287, USA; 5 The Sidney Kimmel Comprehensive Cancer Center, Johns Hopkins University School of Medicine, Baltimore, Maryland 21287, USA; 6 Swim Across America Laboratory and the Ludwig Center for Cancer Genetics and Therapeutics at Johns Hopkins, Baltimore, Maryland 21287, USA

## Abstract

Malignant mixed Müllerian tumours, also known as carcinosarcomas, are rare tumours of gynaecological origin. Here we perform whole-exome analyses of 22 tumours using massively parallel sequencing to determine the mutational landscape of this tumour type. On average, we identify 43 mutations per tumour, excluding four cases with a mutator phenotype that harboured inactivating mutations in mismatch repair genes. In addition to mutations in *TP53* and *KRAS*, we identify genetic alterations in chromatin remodelling genes, *ARID1A* and *ARID1B*, in histone methyltransferase *MLL3*, in histone deacetylase modifier *SPOP* and in chromatin assembly factor *BAZ1A*, in nearly two thirds of cases. Alterations in genes with potential clinical utility are observed in more than three quarters of the cases and included members of the PI3-kinase and homologous DNA repair pathways. These findings highlight the importance of the dysregulation of chromatin remodelling in carcinosarcoma tumorigenesis and suggest new avenues for personalized therapy.

## Abstract (web summary)

Malignant mixed Müllerian tumours are a rare and aggressive gynaecological cancer with poor 5-year survival rates. Here, the authors characterize the mutational landscape of carcinosarcomas and highlight the role of chromatin remodelling dysregulation in carcinosarcoma tumorigenesis.

## Graphical abstract

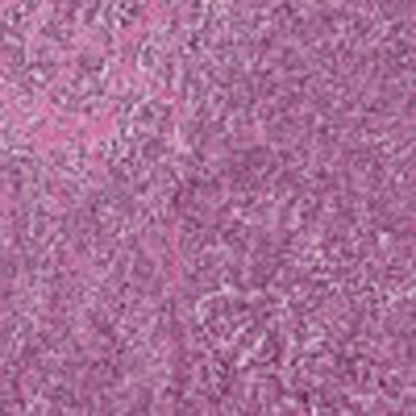

Malignant mixed Müllerian tumours (MMMT) are infrequent gynaecological cancers with histological features of both carcinoma and sarcoma^1^. These tumours arise most commonly in the uterus^2^, but have also been identified in the vagina^3^, cervix^4^, ovary^5^, fallopian tube^6^ and peritoneum^7^. Despite affecting only two per 100,000 US women per year, these tumours are highly aggressive and 5-year survival rates have been reported between 35% and 65% in early-stage carcinosarcoma and only around 10% for stage IV neoplasms^8^.

MMMT typically affect women over the age of 50 years, with a median age of onset of 62 years^9^. Patients may present with vaginal bleeding, abdominal pain and/or mass, but are generally not diagnosed until the disease is in late stage^10^. In addition to advanced age, risk factors include nulliparity, obesity, exogenous oestrogen exposure, pelvic irradiation and treatment with Tamoxifen^9^,^11^,^12^. The primary treatment for MMMT is total abdominal hysterectomy with bilateral salpingo-oophorectomy^13^. In addition to surgery, high rates of recurrence and metastasis necessitate the use of adjuvant chemotherapy and radiation^13^. Despite these interventions, the prognosis is poor and more effective therapeutic options are needed to aid the clinical management of affected patients.

There are three theories for the origin of carcinosarcomas. The collision theory suggests that the tumours are biclonal arising from separate cells that later merge. The combination theory assumes that a common precursor differentiates bidirectionally and the conversion theory posits that a single cell undergoes metaplasmic differentiation^9^,^10^. Molecular and histological evidence supports the conversion hypothesis^14^ and these tumours are now thought to derive from sarcomatous differentiation in a high-grade carcinoma^10^.

Alterations in *TP53*, *KRAS* and the *PIK3CA* pathway have previously been observed in 25%, 15% and 19% of carcinosarcomas, respectively^15^,^16^. Mutations of *CTNNB1* and *NRAS*, as well as mismatch repair deficiency, are known to occur at a lower frequency^15^,^16^,^17^. However, the genetic basis of this tumour type remains largely unexplored. We report here the results of the first unbiased systematic genetic analysis of carcinosarcomas using whole-exome sequencing. These analyses define the mutational landscape of carcinosarcoma and reveal the importance of chromatin remodelling in this tumour type.

## Results

### Overall approach and spectrum of somatic mutations

We used a next-generation sequencing approach to analyse the entire exomes (>20,000 genes) of matched tumour and normal specimens from 22 carcinosarcoma patients for tumour-specific (somatic) mutations. All samples were primary tumours, with the exception of MM02, which was a recurrence at the primary site, and included 17 tumours originating in the uterus and 5 tumours arising in the ovaries (Supplementary Table 1). On average, the sequencing coverage was 191-fold and >90% of bases were covered by at least 10 high-quality reads (Supplementary Table 2). The number of somatic non-synonymous mutations in 18 of these tumours ranged from 4 to 88, with an average of 43 mutations per tumour. The number of somatic mutations per tumour was similar to that of other gynaecological cancers, including ovarian clear cell^18^, high-grade ovarian serous^19^ and uterine serous^20^ carcinomas, but significantly higher than previously observed in low-grade ovarian cancers and pediatric tumours^21^,^22^,^23^. Four tumours harboured a significantly higher number of somatic alterations, ranging from 904 to 5,913. Further analyses revealed somatic mutations in the mismatch repair genes, *MLH1* (MM20T) and *MSH6* (MM04T, MM12T and MM18T) in all cases, suggesting that these defects were responsible for the observed mutator phenotype (Supplementary Table 3). MM12T harboured truncating mutations in *MSH6* in both alleles, whereas single nonsense or frameshift mutations were observed in the other three cases. Microsatellite instability has been previously reported in type I endometrial cancers and low-grade ovarian tumours, as well as in carcinosarcomas^16^,^17^,^21^,^24^.

**Figure 1:**
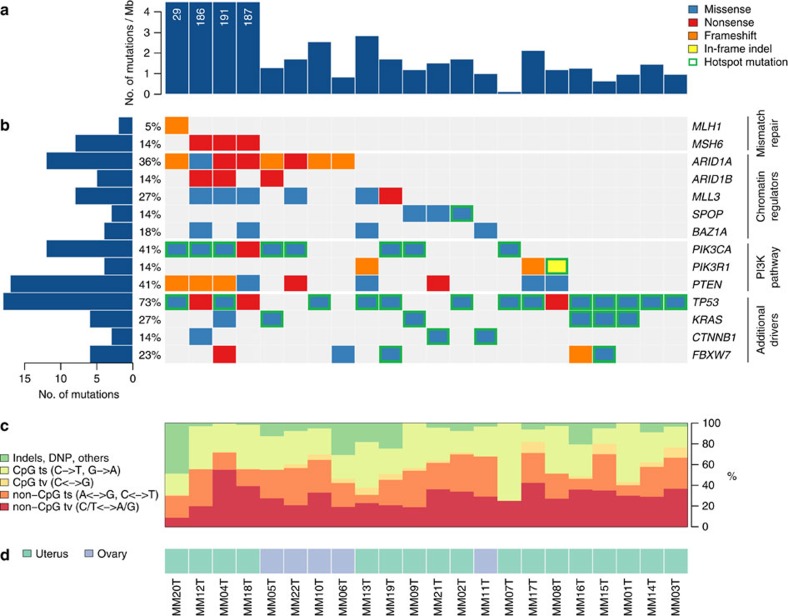
Recurrently mutated genes in gynecological carcinosarcomas. (**a**) The frequency of non-synonymous mutations in each sample of the study is displayed as mutations per megabase. (**b**) The matrix represents individual mutations in 22 patient samples, colour coded by type of mutation. Only the most damaging mutation per gene is shown if multiple mutations were found in a sample. Genes are classified into four functional categories displayed on the right. Mutations occurring at known hotspots are circled in green. Left: bar plot shows the number of mutations in each gene across 22 samples. Percentages represent the fraction of tumours with at least one mutation in the specified gene. (**c**) Base substitution spectrum of individual samples: transitions (ts) and transversions (tv) at CpG and non-CpG sites, indels and dinucleotide substitutions. (**d**) Site of the primary tumour.

The vast majority of somatic alterations observed were single base substitutions, including non-synonymous coding changes, nonsense mutations and splice site alterations, with the remaining mutations as small insertions and deletions (Supplementary Table 3). The non-silent substitutions in the microsatellite stable cases were predominantly C:G to T:A transitions (Fig. 1). These mutations are thought to arise as a result of deamination of 5-methyl cytosine at CpG sites^25^ and are a common feature across cancer types, including other gynaecological tumours^18^,^26^. Mismatch repair-defective cases displayed an atypical mutational signature consistent with previous reports^26^,^27^,^28^. Almost half of the somatic mutations in the tumour harbouring an *MLH1* alteration were small insertions and deletions, compared with only 10% in the microsatellite stable cases. In the *MSH6*-mutated cases, C:G to T:A transitions were the most common, accounting for 53% of somatic mutations, but there was also an overrepresentation of C:G to A:T transversions (30%), probably as a consequence of the inability to repair mismatches arising as a result of oxidative damage^28^ (Fig. 1).

### Recurrently mutated genes

Overall, we identified 777 somatic mutations in 702 genes in the 18 microsatellite stable cancers, as well as 18,371 alterations in 4 mismatch repair-deficient tumours. The analyses of tumour-specific mutations revealed a number of known genes with recurrent alterations. Mutations in *TP53* were observed in 67% (16/22) of cases, a significantly higher fraction than previously appreciated^15^. The phosphoinositide 3-kinase (PI3-kinase) pathway was affected by activating mutations in *PIK3CA* (9/22 cases) and *PTEN* (9/22 cases). We identified mutations in *PIK3R1*, a member of the PI3-kinase pathway that had not been previously implicated in carcinosarcomas, in 3 of 22 cases. As in endometrial tumours^24^, mutations in *PIK3CA* and *PIK3R1* were mutually exclusive, but loss of PTEN function co-occurred with other genes in this pathway. Unlike in previous studies of carcinosarcomas, PI3-kinase pathway mutations were not confined to tumours located in the uterus, but also occurred in ovarian cases^15^. Hotspot mutations in *CTNNB1* were observed in two microsatellite stable cases (Fig. 1).

**Figure 2:**
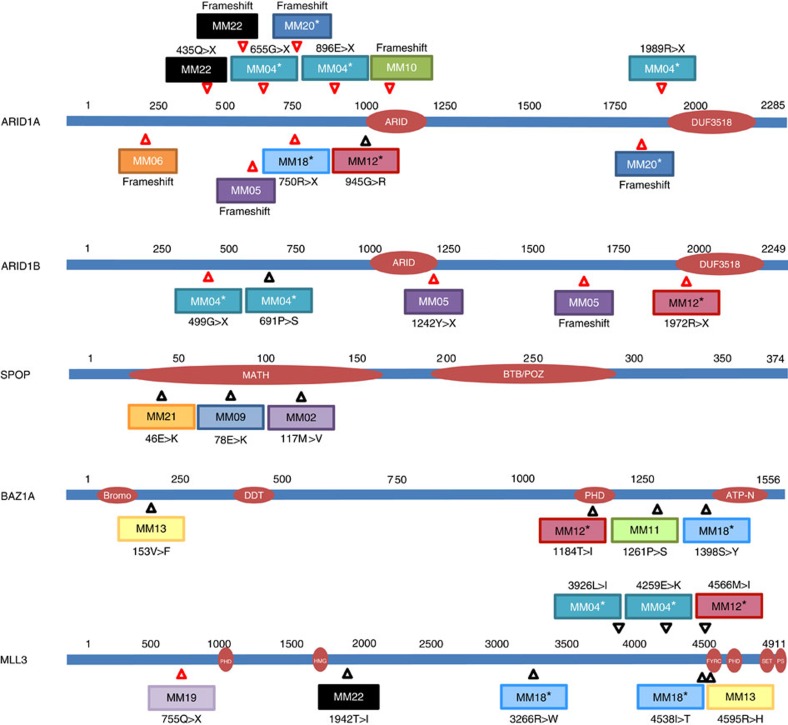
Mutations in chromatin remodelling genes. Locations of mutations in the ARID1A, ARID1B, SPOP, BAZ1A and MLL3 proteins are indicated with arrows. Red arrows indicate mutations predicted to truncate the protein, while black arrows indicate missense alterations. Each tumour sample is depicted in a different colour.

In addition to genes already associated with carcinosarcomas, we identified mutations in genes not previously implicated in this tumour type (Fig. 1). Truncating mutations in *ARID1A* were detected in 32% (7/22) of tumours (Figs 1 and 2). Four of the five ovarian tumours had *ARID1A* mutations and these were not observed in the microsatellite-stable tumours of the uterus. Three of the *ARID1A*-mutated tumours also harboured frameshift and nonsense alterations in *ARID1B*. Alterations in *ARID1A* have been reported in other gynaecological cancers, including a high proportion of ovarian clear cell carcinoma (57%)^18^, endometrioid carcinoma of the ovary^29^ and endometrial tumours^24^, as well as a range of other tumour types such as neuroblastoma, colorectal, gastric, prostate and breast cancers^23^,^30^. Intragenic deletions and point mutations in *ARID1B* were recently discovered in around 10% of neuroblastomas^23^ and have been seen in a small fraction of hepatocellular^31^, breast^32^ and medulloblastoma tumours^33^,^34^.

Alterations of the histone methyltransferase *MLL3* were identified in 27% (6/22) of carcinosarcomas. One of these tumours also harboured a splice site alteration in the related gene, *MLL2*. Mutations in these genes have been observed in ovarian clear cell carcinomas^18^ and are known to be involved in the tumorigenesis of medulloblastoma^22^, lung squamous cell carcinoma^35^ and Mantle cell lymphoma^36^. In addition, *SPOP*, a putative tumour suppressor involved in chromatin remodelling was found to be altered in 3 cases (14%). Mutations in *SPOP* have been previously reported in endometrial^37^, prostate^38^, lung^39^ and colorectal cancers^40^. These mutations fall nearby the reported alterations within the MATH domain and the encoded protein is known to modulate the transcriptional repression activities of DAXX, a component of the histone deacetylase co-repressor complex^41^. *BAZ1A*, an accessory unit of the ATP-dependent chromatin assembly factor^42^ was mutated in 4 of the 22 carcinosarcomas. *BAZ1A* is mutated at a low frequency in ovarian cancer^19^ and deletions of this gene are known to occur in renal papillary carcinoma^43^. Overall, approximately two-thirds of cases harboured mutations in genes involved in chromatin remodelling or modification.

Mutations in the tumour suppressor gene, *FBXW7*, were identified in 23% (5/22) of cases. The encoded protein is a member of the SCF (SKP-cullin-F-box) ubiquitin ligase complex that targets cyclin E for degradation^44^. One mutation was a frameshift predicted to truncate the protein and the other three missense alterations were clustered in the WD repeat domain. Tumour MM19 carried the previously described hotspot mutation, 505R>C. *FBXW7* is mutated in a number of different tumour types, including colorectal and haematopoietic malignancies, and has been recently reported in approximately a third of endometrial cancers^20^,^37^.

The serine/threonine phosphatase, *PPP2R1A*, was mutated in one uterine carcinosarcoma. Alterations in this gene were initially identified in ovarian clear cell carcinomas^18^ and more recently in a range of tumour types, including a number of endometrial cancers and across different ovarian cancer subtypes^24^,^45^. The missense mutation, 180M>R, falls nearby other hotspot mutations within the HEAT domain^18^,^24^.

Consistent with the accepted theory of carcinosarcomas arising from a single cell, mutations in known cancer genes identified through whole-exome analyses appeared to be clonal in nature. Seven of the cases had *TP53* mutation frequencies above 80%, indicating a homozygous change in all tumour cells when adjusted for the presence of contaminating normal tissue, while heterozygous mutations in *TP53* or other known cancer genes in 10 additional cases were present at an average of 49.5%. These analyses suggest that individual carcinosarcomas comprises a population of cells that contain largely identical somatic sequence alterations.

### Clinically actionable alterations

There are currently no targeted therapies for carcinosarcomas and the prognosis for patients with this tumour type is poor. We investigated whether the observed mutations may be clinically actionable using existing or investigational therapies. We examined genetic alterations that were associated with Food and Drug Administration-approved therapies for oncologic indications, therapies in published prospective or retrospective clinical studies and ongoing clinical trials for cancer patients. We also evaluated alterations in 84 well-known cancer predisposing genes in the patients’ germline that may affect cancer susceptibility as detection of such changes have important implications for early detection and intervention. We identified mutations in a number of potentially clinically actionable genes (Table 1). The PI3-kinase pathway was activated in over half of the carcinosarcomas through alterations in *PIK3CA*, *PIK3R1* and *PTEN*. Targeted inhibition of this pathway using PI3K/mammalian target of rapamycin/AKT/MEK inhibitors has been reported and is being studied in ongoing Phase I and II trials in endometrial cancer and other tumour types^46^,^47^,^48^,^49^. A known hotspot missense mutation, 104V>M, in the extracellular domain of the kinase ERBB3 (ref. 50) was observed in a single tumour, and antibodies targeting this protein have been in clinical development, including phase II studies. Similarly, *KRAS* hotspot mutations were identified in five cases and tumours of these patients may respond to MEK/BRAF inhibitors. Pre-clinical evidence suggests that loss of *FBXW7* function, as observed in a number of the carcinosarcomas, may result in sensitivity to HDAC inhibitors^51^. Two somatic nonsense mutations in *BRCA2*, a frameshift in *BRCA1* and a splice site alteration of *FANCM* were also identified providing further evidence for the role of homologous recombination repair defects in carcinosarcomas^52^. In addition, we identified a nonsense mutation (3326K>X) in the *BRCA2* gene in the germline of a patient (MM08) with a family history of cervical (mother) and lung cancer (sister). Tumours of these patients may be sensitive to DNA cross-linking agents and to poly (ADP-ribose) polymerase inhibitors^53^,^54^. The relatively high frequency of mismatch repair-deficient tumours may also be clinically significant, as the efficacy of the antibody, MK-34775, is actively being evaluated in patients with microsatellite unstable tumours.

## Discussion

These analyses have provided the most comprehensive characterization of the mutational landscape of carcinosarcomas to date. Our data show that carcinosarcomas have one of the highest frequencies of chromatin remodelling dysregulation of all tumour types analysed to date. The most highly mutated genes, *ARID1A*, along with *ARID1B*, are key components of the conserved, ATP-dependent SWI/SNF chromatin remodelling complex^55^. This complex uses helicase activity to allow transcription factors access to DNA^56^ and is important in the regulation of multiple cellular processes, including DNA repair, cell cycle progression and cell migration^57^,^58^. Inactivation of the ARID1 complex appears to be particularly important in gynaecological cancers as observed in this study and in other tumours of the female genital tract^18^,^24^,^29^. Among the microsatellite-stable tumours, *ARID1A* mutations were unique to carcinosarcomas originating in the ovaries, which may suggest different mutational signatures in carcinosarcomas of different sites. This is a potentially significant finding as the prognosis for ovarian carcinosarcomas tends to be better than for those of the uterus^8^.

Other genes involved in chromatin modification in this tumour type include *MLL3*, *SPOP* and *BAZ1A*. *MLL3* encodes a histone H3K4 trimethylase that is part of the ASC-2 complex (ASCOM). This complex is a co-activator of *TP53* and regulates the expression of *TP53* target genes in response to DNA damage^59^. Members of the MLL family have also been shown to have a role in *HOX* gene expression^60^ and Wnt signalling^61^. SPOP is a BTB (Bric-a-brac/Tramtrack/Broad complex) protein that recruits DAXX to the ubiquitin ligase Cul3, resulting in ubiquitination and degradation of the DAXX protein^62^. This leads to the reversal of DAXX-mediated repression of ETS1 and p53-dependent transcription^62^. The *BAZ1A* gene encodes the accessory subunit of the ATP-dependent chromatin assembly factor. This complex generates and maintains nucleosome spacing, a function critical for chromatin condensation and appropriate gene silencing^42^. These observations suggest that a variety of different aspects of chromatin regulation and modification are genetically altered in carcinosarcomas. Genomic analyses in other tumour types have shown that somatic mutations of epigenetic regulators can have important clinical consequences, including improved outcome in patients with *DAXX/ATRX* alterations in pancreatic neuroendocrine tumours^63^ and gliomas^64^, and a decreased survival in patients with *ARID1A* and *ARID1B* mutations in neuroblastoma^23^.

The presence of carcinoma and sarcomatous components in MMMTs has hindered the clinical management of patients, as the prognosis and appropriate therapies for carcinomas and sarcomas differ. These molecular data support the idea that carcinosarcomas are most similar to carcinomas, based on the presence of alterations in genes such as *PTEN* that have only rarely been observed in endometrial sarcomas^65^ and uterine sarcomas^66^, but are common in uterine adenocarcinomas^67^. In addition, the number of non-synonymous mutations observed in the carcinosarcomas was similar to that reported in carcinomas and significantly higher than is typical for sarcomas analysed to date^19^,^68^. Our genomic analyses suggest that the unusual pathology of this tumour type could be related to the loss of control of genetic programing as a result of mutations in chromatin remodelling genes in the specific context of progenitor cells that lead to carcinosarcomas.

In addition to providing therapeutic insight, understanding the mutational profile of MMMTs will be beneficial for diagnostic purposes, including early detection, which can immediately have an impact on survival. Recent studies have used mutations as highly specific biomarkers heralding the presence of malignancy even at the earliest stages by sequencing cell-free DNA in the circulation as well as DNA extracted from liquid Papanicolaou smear specimens for the detection of gynaecological cancers^69^,^70^.

Defining the mutational landscape of carcinosarcomas has highlighted alterations in specific genes and pathways that may aid the diagnosis and future clinical management of patients with this malignancy. Alterations of the PI3K and DNA repair pathways have identified specific actionable targets, which have not been previously considered in this tumour type, as well as a frequent dysregulation of chromatin remodelling. These analyses suggest future efforts at defining the downstream targets of chromatin regulators in carcinosarcomas as well as interventional clinical trials based on potentially actionable alterations observed in these cancer patients.

## Methods

### Samples

Twenty-two carcinosarcoma tumour samples and matched normal tissues were obtained with consent from all study participants and approved for research use by the OHSU Institutional Review Board and the Knight BioLibrary. Eleven patients were treated with chemotherapy or radiation after surgery. Samples underwent pathological review to determine tumour cellularity. Tumours were macrodissected to remove contaminating normal tissue. An average of 85% neoplastic cellularity was obtained. DNA was extracted from 10 fresh-frozen tumours, 12 formalin-fixed paraffin embedded tumours and matched normal tissue samples (Supplementary Table 1).

### Library generation and sequencing

Genomic DNA from tumour and normal samples were fragmented and used for Illumina TruSeq library construction (Illumina, San Diego, CA). Exonic regions were captured in solution using the Agilent SureSelect 51 Mb kit (version 4) according to the manufacturer’s instructions (Agilent, Santa Clara, CA). Paired-end sequencing, resulting in 100 bases from each end of the fragments, was performed using a HiSeq 2000 Genome Analyzer (Illumina).

### Bioinformatic analyses

Sequencing reads were analysed and aligned to human genome hg19 with the Eland algorithm in CASAVA 1.7 software (Illumina). Reads were mapped using the default seed-and-extend algorithm, which allowed a maximum of two mismatched bases in the first 32 bp of sequence. Identification of somatic alterations was performed using a next-generation sequencing analysis pipeline that enriched for tumour-specific single-nucleotide alterations and small indels. Briefly, for each position with a mismatch (compared with the hg19 reference sequence using the Eland algorithm), the read coverage of the mismatched and wild-type sequence at that base was calculated. For determination of tumour-specific alterations, tumour and matched normal sequences were compared and known polymorphisms were removed from the analysis. Potential somatic mutations were filtered and visually inspected, and a candidate-mismatched base was identified as a mutation when distinct paired tags contained the mismatched base and the mismatched base was not present in the matched normal sample.

## Additional information

**Accession codes:** Carcinosarcoma exome sequence data has been deposited at the European Genome-phenome Archive ( http://www.ebi.ac.uk/ega/), which is hosted by the EBI, under the accession code EGAS00001000941.

**How to cite this article**: Jones, S. *et al.* Genomic analyses of gynaecologic carcinosarcomas reveal frequent mutations in chromatin remodelling genes. *Nat. Commun.* 5:5006 doi: 10.1038/ncomms6006 (2014).

## Supplementary Material

Supplementary Information Supplementary Tables 1-3

## Figures and Tables

**Table 1:** Alterations in genes with potential clinical actionability.

**Gene symbol**	**Gene description**	**Type of mutation**	**Number of cases mutated**	**Information type**	**Therapy type**
* BRCA1 *	Breast cancer 1; early onset	Frameshift	1	Active clinical trial, published clinical trial	PARP inhibitor
* BRCA2 *	Breast cancer 2; early onset	Nonsense	3	Active clinical trial, published clinical trial	PARP inhibitor
* ERBB3 *	v-erb-b2 erythroblastic leukemia viral oncogene homologue 3	Hotspot	1	Active clinical trial, published clinical trial	ERBB family inhibitors
* FANCM *	Fanconi anaemia, complementation group M	Splice site, frameshift	2	Active clinical trial	Mitomycin
* FBXW7 *	F-box and WD repeat domain containing 7, E3 ubiquitin protein ligase	Frameshift, nonsense, hotspot, missense	5	Preclinical	HDAC inhibitors
* KRAS *	v-Ki-ras2 Kirsten rat sarcoma viral oncogene homologue	Hotspot	5	Active clinical trial, published clinical trial	MEK/BRAF inhibitor
* MLH1 *	mutL homologue 1, colon cancer, non-polyposis type 2 (*Escherichia coli*)	Frameshift	1	Active clinical trial	Anti-PD-1 immunotherapy
* MSH6 *	mutS homologue 6 (*E. coli*)	Nonsense, frameshift	3	Active clinical trial	Anti-PD-1 immunotherapy
* PIK3CA *	Phosphoinositide-3-kinase; catalytic; alpha polypeptide	Hotspot	8	Active clinical trial, published clinical trial	PI3K/mTOR/AKT/MEKi
* PIK3R1 *	Phosphoinositide-3-kinase, regulatory subunit 1 (alpha)	In-frame deletion, in-frame insertion, frameshift	3	Active clinical trial	mTOR inhibitor
* PTEN *	Phosphatase and tensin homologue	Missense, nonsense, frameshift	8	Active clinical trial, published clinical trial	PI3K/AKT inhibitor

HDAC, histone deacetylase; mTOR, mammalian target of rapamycin; PARP, poly (ADP-ribose) polymerase; PI3K, phosphoinositide 3-kinase.
