# Supplementary material for: Genomic analyses of gynaecologic carcinosarcomas reveal frequent mutations in chromatin remodelling genes
Source: Nat Commun. 2014 Sep 19;5:5006. doi: 10.1038/ncomms6006 (PMC4354107; doi:10.1038/ncomms6006)
Supplement: Supplementary Information — Supplementary Tables 1-3 [file ncomms6006-s1.pdf]

Supplementary Table 1. Characteristics of Carcinosarcoma Samples

Sample	Age at Tumor Resection	Adjuvant Therapy	Location	FIGO stage	Sample Type	Pathologic Tumor Cellularity
MM01T	77	Chemotherapy, Radiotherapy	Uterus	IIIA	Frozen	90%
MM02T	59	Chemotherapy	Uterus	IA	Frozen	90%
MM03T	51	None	Uterus	NA	FFPE	70%
MM04T	54	None	Uterus	I	FFPE	90%
MM05T	61	Chemotherapy	Ovary	IIC	FFPE	80%
MM06T	73	None	Ovary	IV	Frozen	90%
MM07T	61	None	Uterus	NA	FFPE	80%
MM08T	74	None	Uterus	IB	FFPE	90%
MM09T	64	Radiotherapy, Chemotherapy	Uterus	III	FFPE	80%
MM10T	84	None	Ovary	IIIC	Frozen	90%
MM11T	64	Chemotherapy	Ovary	IIIA	FFPE	80%
MM12T	55	None	Uterus	IB	FFPE	90%
MM13T	72	Chemotherapy	Uterus	NA	FFPE	70%
MM14T	82	Chemotherapy, Radiotherapy	Uterus	IIIA	Frozen	90%
MM15T	63	None	Uterus	NA	FFPE	90%
MM16T	78	None	Uterus	IIIA	Frozen	90%
MM17T	71	None	Uterus	IIIA	Frozen	90%
MM18T	59	Radiation	Uterus	IB	FFPE	80%
MM19T	68	None	Uterus	II	Frozen	90%
MM20T	44	Radiotherapy	Uterus	IB	FFPE	80%
MM21T	67	Chemotherapy, Radiotherapy	Uterus	IB	Frozen	90%
MM22T	52	Chemotherapy	Ovary	IIC	Frozen	80%

Supplementary Table 2. Next Generation Sequencing Characteristics

Sample	Bases Sequenced	Total Mapped Sequence	Based Mapped to Target Region	Fraction Mapped to Genome	Fraction Mapped to Target Region	Bases in Target Region With at Least 10 Reads	Fraction of Bases in Target Region With at Least 10 Reads	Total Coverage	Distinct Coverage
MM01N	18,963,020,600	18,512,527,600	10,523,589,955	98%	57%	50,619,551	93%	189	129
MM01T	19,930,139,800	19,447,484,900	10,930,949,235	98%	56%	50,495,339	93%	184	106
MM02N	21,885,274,400	17,802,347,700	12,238,609,058	81%	69%	44,312,201	88%	230	127
MM02T	21,229,167,800	20,566,897,300	10,627,258,690	97%	52%	47,154,663	94%	190	156
MM03N	28,905,196,800	17,106,059,900	10,948,811,522	59%	64%	44,173,728	88%	206	70
MM03T	26,784,186,200	20,983,794,400	12,292,403,133	78%	59%	44,988,578	89%	211	140
MM04N	26,806,049,800	24,222,314,400	15,669,280,376	90%	65%	46,594,141	93%	296	220
MM04T	23,457,085,000	20,729,184,400	12,087,655,885	88%	58%	46,591,021	93%	209	149
MM05N	20,765,792,000	12,802,342,300	8,445,580,548	62%	66%	42,646,634	85%	159	70
MM05T	26,451,974,200	21,509,600,800	13,290,987,017	81%	62%	44,240,216	88%	232	167
MM06N	19,486,676,200	8,368,260,600	4,871,252,453	43%	58%	39,999,038	79%	91	17
MM06T	23,555,339,000	22,745,397,800	11,555,196,242	97%	51%	47,289,317	94%	205	151
MM07N	28,301,494,600	25,296,439,200	16,754,453,616	89%	66%	45,825,841	91%	315	244
MM07T	21,247,754,600	17,573,976,900	10,024,976,660	83%	57%	45,351,325	90%	176	138
MM08N	22,292,326,200	17,660,857,700	10,540,875,095	79%	60%	45,890,962	91%	195	123
MM08T	19,375,998,800	17,061,089,000	10,655,290,261	88%	62%	44,425,965	88%	177	139
MM09N	24,060,137,200	16,635,886,600	10,605,738,156	69%	64%	44,799,268	89%	200	128
MM09T	24,840,360,400	19,706,053,100	11,442,733,247	79%	58%	45,992,776	91%	202	144
MM10N	19,253,050,000	15,963,620,700	10,016,985,016	83%	63%	45,751,357	91%	189	125
MM10T	22,798,679,200	22,137,597,100	12,276,948,025	97%	55%	47,066,802	93%	219	146
MM11N	17,962,640,000	10,843,056,600	5,319,327,729	60%	49%	43,180,784	86%	100	48
MM11T	28,468,947,000	18,088,714,700	9,465,616,801	64%	52%	44,746,574	89%	167	59
MM12N	20,513,173,600	16,848,910,900	10,684,330,348	82%	63%	45,742,419	91%	204	133
MM12T	25,842,099,800	18,701,227,700	10,218,870,862	72%	55%	45,495,034	90%	185	129
MM13N	24,738,919,200	22,226,166,700	12,960,565,501	90%	58%	46,953,216	93%	245	197
MM13T	26,006,196,400	23,595,032,100	13,717,824,514	91%	58%	46,664,167	93%	241	207
MM14N	21,970,130,400	18,620,519,900	11,915,475,990	85%	64%	45,539,628	90%	226	179
MM14T	18,422,571,600	17,892,149,700	9,673,788,714	97%	54%	46,952,098	93%	174	134
MM15N	26,627,632,600	21,846,937,500	12,453,164,759	82%	57%	46,233,420	92%	231	146
MM15T	20,260,560,000	16,341,573,800	8,298,190,745	81%	51%	45,650,117	91%	144	90
MM16N	23,780,700,200	20,506,918,700	11,466,432,597	86%	56%	46,665,731	93%	215	151
MM16T	21,073,283,000	20,335,183,900	10,577,443,581	97%	52%	47,194,346	94%	183	152
MM17N	19,665,231,600	16,332,505,900	9,725,070,637	83%	60%	46,467,713	92%	184	136
MM17T	18,626,301,200	18,012,471,600	10,349,944,706	97%	57%	47,070,269	93%	180	129
MM18N	15,990,332,400	12,298,589,200	7,430,514,454	77%	60%	44,364,518	88%	137	99
MM18T	23,319,124,000	19,316,878,000	10,773,762,574	83%	56%	46,458,333	92%	182	134
MM19N	17,196,817,400	15,616,007,100	10,071,602,742	91%	65%	46,329,443	92%	191	139
MM19T	20,820,409,400	20,181,646,000	11,461,608,405	97%	57%	47,100,222	94%	198	159
MM20N	19,456,850,400	17,657,571,400	10,587,349,538	91%	60%	46,728,155	93%	200	164
MM20T	15,066,865,000	13,053,723,900	7,356,653,982	87%	56%	46,038,256	91%	131	100
MM21N	13,953,834,000	12,362,940,000	7,712,602,110	89%	62%	45,783,752	91%	148	113
MM21T	16,380,023,200	15,815,949,400	7,735,258,855	97%	49%	47,147,870	94%	137	116
MM22N	17,297,028,200	14,564,990,800	8,396,701,934	84%	58%	46,268,902	92%	158	118
MM22T	19,222,775,400	18,421,881,200	8,913,252,407	96%	48%	47,227,248	94%	152	106
Average	21,660,276,109	18,098,029,070	10,524,202,924	84%	58%	45,959,340	91%	191	132

Supplementary Table 3. Somatic Mutations Identified in the Carcinosarcoma Samples

Sample	Gene Symbol	Gene Description	Transcript Accession	Nucleotide Position (genomic) hg19	Amino Acid (protein)	Mutation Type	Consequence	% Mutant Tags
MM01T	ABLIM3	actin binding LIM protein family, member 3	CCDS4294.1	chr5_148626050-148626050_C_T	498R>X	Substitution	Nonsense	62%
MM01T	AMOTL2	angiomotin like 2	CCDS33860.1	chr3_134079032-134079032_C_T	600R>Q	Substitution	Nonsynonymous coding	37%
MM01T	ARAF	v-raf murine sarcoma 3611 viral oncogene homolog	CCDS35232.1	chrX_47428124-47428124_C_T	362R>X	Substitution	Nonsense	25%
MM01T	ASTN1	astrotactin 1	ENST00000281881	chr1_176992533-176992533_C_T	482R>Q	Substitution	Nonsynonymous coding	24%
MM01T	BASP1	brain abundant, membrane attached signal protein 1	CCDS3888.1	chr5_17275414-17275414_C_T	30A>V	Substitution	Nonsynonymous coding	16%
MM01T	C12orf44	chromosome 12 open reading frame 44	CCDS8820.1	chr12_52470865-52470865_G_A	183R>Q	Substitution	Nonsynonymous coding	33%
MM01T	CA13	carbonic anhydrase XIII	CCDS6236.1	chr8_86171674-86171674_G_A	87G>E	Substitution	Nonsynonymous coding	54%
MM01T	CACNG3	calcium channel, voltage-dependent, gamma subunit 3	CCDS10620.1	chr16_24372691-24372691_G_C	152G>A	Substitution	Nonsynonymous coding	45%
MM01T	CALCOCO1	calcium binding and coiled-coil domain 1	CCDS8864.1	chr12_54105819-54105819_C_T	662R>H	Substitution	Nonsynonymous coding	31%
MM01T	CERS3	ceramide synthase 3	CCDS10384.1	chr15_101024858-101024858_G_T	102L>M	Substitution	Nonsynonymous coding	26%
MM01T	DOCK3	dedicator of cytokinesis 3	CCDS46835.1	chr3_51386368-51386368_G_A	1334R>Q	Substitution	Nonsynonymous coding	15%
MM01T	EVC	Ellis van Creveld syndrome	CCDS3383.1	chr4_5800320-5800320_G_A	702R>H	Substitution	Nonsynonymous coding	73%
MM01T	FAM135B	family with sequence similarity 135, member B	CCDS6375.2	chr8_139149483-139149483_C_T	1308V>I	Substitution	Nonsynonymous coding	53%
MM01T	FAT4	FAT tumor suppressor homolog 4	CCDS3732.3	chr4_126373056-126373056_C_T	3629P>S	Substitution	Nonsynonymous coding	44%
MM01T	FOXA2	forkhead box A2	CCDS46585.1	chr20_22563360-22563360_T_A	174I>F	Substitution	Nonsynonymous coding	31%
MM01T	G6PD	glucose-6-phosphate dehydrogenase	CCDS14756.2	chrX_153762371-153762371_C_A	247A>S	Substitution	Nonsynonymous coding	27%
MM01T	GLT8D2	glycosyltransferase 8 domain containing 2	CCDS9096.1	chr12_104388251-104388251_C_T	210R>Q	Substitution	Nonsynonymous coding	40%
MM01T	HMCN2	hemicentin 2	ENST00000420499	chr9_133230346-133230346_G_A	346R>Q	Substitution	Nonsynonymous coding	49%
MM01T	KRAS	v-Ki-ras2 Kirsten rat sarcoma viral oncogene homolog	CCDS8703.1	chr12_25398284-25398284_C_A	12G>V	Substitution	Nonsynonymous coding	57%
MM01T	PARD3	par-3 partitioning defective 3 homolog	CCDS7178.1	chr10_34663884-34663884_G_T	529S>X	Substitution	Nonsense	13%
MM01T	PGD	phosphogluconate dehydrogenase	CCDS113.1	chr1_10473287-10473287_G_A	275G>S	Substitution	Nonsynonymous coding	61%
MM01T	POR	P450 (cytochrome) oxidoreductase	ENST00000448410	chr7_75601793-75601793_A_G	84H>R	Substitution	Nonsynonymous coding	56%
MM01T	RBM5	RNA binding motif protein 5	CCDS2810.1	chr3_50155827-50155827_G_A	796A>T	Substitution	Nonsynonymous coding	21%
MM01T	REEP4	receptor accessory protein 4	CCDS6024.1	chr8_21996958-21996958_C_T	130A>T	Substitution	Nonsynonymous coding	21%
MM01T	SH3GL1	SH3-domain GRB2-like 1	CCDS32874.1	chr19_4362367-4362367_C_T	290R>Q	Substitution	Nonsynonymous coding	45%
MM01T	SORCS1	sortilin-related VPS10 domain containing receptor 1	CCDS31283.1	chr10_108924094-108924094_C_A	64R>M	Substitution	Nonsynonymous coding	69%
MM01T	SPTBN1	spectrin, beta, non-erythrocytic 1	CCDS33198.1	chr2_54844824-54844824_C_T	216R>W	Substitution	Nonsynonymous coding	25%
MM01T	TLE4	transducin-like enhancer of split 4	ENST00000376534	chr9_82322044-82322044_G_T	315D>Y	Substitution	Nonsynonymous coding	33%
MM01T	TP53	tumor protein p53	CCDS11118.1	chr17_7578535-7578535_T_A	132K>M	Substitution	Nonsynonymous coding	82%

MM01T	VPS13C	vacuolar protein sorting 13 homolog	CCDS32257.1	chr15_62246704-62246704_A_C	1405D>E	Substitution	Nonsynonymous coding	20%
MM02T	ACVR2A	activin A receptor, type IIA	CCDS33301.1	chr2_148674853-148674856_ACAA_	NA	Deletion	Frameshift	35%
MM02T	C12orf54	chromosome 12 open reading frame 54	CCDS8764.1	chr12_48888682-48888682_C_A	115T>K	Substitution	Nonsynonymous coding	45%
MM02T	CCDC88A	coiled-coil domain containing 88A	CCDS46288.1	chr2_55536123-55536123_C_G	1402L>F	Substitution	Nonsynonymous coding	13%
MM02T	CHD8	chromodomain helicase DNA binding protein 8	CCDS45081.1	chr14_21863171-21863171_G_T	1485Q>K	Substitution	Nonsynonymous coding	41%
MM02T	CYFIP2	cytoplasmic FMR1 interacting protein 2	NM_001037332	chr5_156747710-156747710_G_A	524R>Q	Substitution	Nonsynonymous coding	49%
MM02T	DENND1A	DENN/MADD domain containing 1A	CCDS35133.1	chr9_126531801-126531801_A_T	58Y>X	Substitution	Nonsense	32%
MM02T	DLL3	delta-like 3 (Drosophila)	CCDS12538.1	chr19_39993460-39993460_G_T	139A>S	Substitution	Nonsynonymous coding	91%
MM02T	DLL3	delta-like 3 (Drosophila)	CCDS12538.1	chr19_39993461-39993461_C_T	139A>V	Substitution	Nonsynonymous coding	92%
MM02T	DOCK1	dedicator of cytokinesis 1	NM_001380	chr10_129172446-129172446_G_A	1194E>K	Substitution	Nonsynonymous coding	24%
MM02T	FGA	fibrinogen alpha chain	CCDS3787.1	chr4_155506821-155506821_G_A	587T>M	Substitution	Nonsynonymous coding	90%
MM02T	FRMPD1	FERM and PDZ domain containing 1	CCDS6612.1	chr9_37740320-37740320_G_A	599G>S	Substitution	Nonsynonymous coding	45%
MM02T	GGA2	golgi-associated, gamma adaptin ear containing, ARF binding protein 2	CCDS10611.1	chr16_23489793-23489793_C_G	396K>N	Substitution	Nonsynonymous coding	43%
MM02T	GTPBP5	GTP binding protein 5 (putative)	CCDS13492.1	chr20_60768528-60768528_G_A	18V>M	Substitution	Nonsynonymous coding	47%
MM02T	HERC4	HECT and RLD domain containing E3 ubiquitin protein ligase 4	CCDS41533.1	chr10_69797879-69797879_C_A	145G>V	Substitution	Nonsynonymous coding	33%
MM02T	HRASLS5	HRAS-like suppressor family, member 5	CCDS8044.1	chr11_63258454-63258454_A_G	18I>T	Substitution	Nonsynonymous coding	20%
MM02T	KCNG4	potassium voltage-gated channel, subfamily G, member 4	CCDS10945.1	chr16_84256602-84256602_A_G	261Y>H	Substitution	Nonsynonymous coding	89%
MM02T	KIAA2022	Uncharacterized protein	CCDS35337.1	chrX_73960110-73960110_C_G	1428D>H	Substitution	Nonsynonymous coding	42%
MM02T	KIAA2022	Uncharacterized protein	CCDS35337.1	chrX_73960550-73960550_C_T	1281G>E	Substitution	Nonsynonymous coding	43%
MM02T	LAMA4	laminin, alpha 4	CCDS43491.1	chr6_112457443-112457443_C_T	1099R>K	Substitution	Nonsynonymous coding	41%
MM02T	LPXN	leupaxin	CCDS7969.1	chr11_58295162-58295164_AAG_	NA	Deletion	In-frame deletion	59%
MM02T	MEX3D	mex-3 homolog D (C. elegans)	CCDS32865.2	chr19_1556347-1556347_G_A	391R>C	Substitution	Nonsynonymous coding	37%
MM02T	MTHFR	methylenetetrahydrofolate reductase (NAD(P)H)	ENST00000376583	chr1_11863537-11863537_C_T	12G>E	Substitution	Nonsynonymous coding	41%
MM02T	MYO5A	myosin VA (heavy chain 12, myoxin)	CCDS42037.1	chr15_52720601-52720601_A_T	102Y>N	Substitution	Nonsynonymous coding	51%
MM02T	MYOM1	myomesin 1	CCDS45824.1	chr18_3129419-3129419_A_G	869F>L	Substitution	Nonsynonymous coding	32%
MM02T	NHLRC1	NHL repeat containing 1	CCDS4542.1	chr6_18122185-18122185_G_A	218P>L	Substitution	Nonsynonymous coding	28%
MM02T	NOTCH2	notch 2	ENST00000401649	chr1_120539354-120539357_TTAT_	NA	Deletion	Frameshift	14%
MM02T	NT5DC3	5'-nucleotidase domain containing 3	CCDS41824.1	chr12_104181304-104181304_C_A	368G>V	Substitution	Nonsynonymous coding	49%
MM02T	OR1L4	olfactory receptor, family 1, subfamily L, member 4	CCDS35129.1	chr9_125486717-125486717_G_A	150C>Y	Substitution	Nonsynonymous coding	11%
MM02T	OTOG	otogelin	ENST00000399391	chr11_17614747-17614747_C_G	1017S>X	Substitution	Nonsense	89%
MM02T	OTOG	otogelin	ENST00000399391	chr11_17614822-17614822_C_A	1042S>Y	Substitution	Nonsynonymous coding	85%
MM02T	PCDHB10	protocadherin beta 10	CCDS4252.1	chr5_140572858-140572858_G_T	245A>S	Substitution	Nonsynonymous coding	25%

MM02T	PDGFB	platelet-derived growth factor beta polypeptide	CCDS13987.1	chr22_39627737-39627737_C_T	116A>T	Substitution	Nonsynonymous coding	38%
MM02T	PGK1	phosphoglycerate kinase 1	CCDS14438.1	chrX_77365416-77365416_T_G	_ISV+2>	Substitution	Splice site donor	45%
MM02T	PHF12	PHD finger protein 12	CCDS32598.1	chr17_27233221-27233221_G_A	999R>C	Substitution	Nonsynonymous coding	96%
MM02T	PKD2L1	polycystic kidney disease 2-like 1	CCDS7492.1	chr10_102048788-102048788_G_A	759P>L	Substitution	Nonsynonymous coding	31%
MM02T	PLEC	plectin	CCDS43772.1	chr8_144997729-144997729_T_C	2260E>G	Substitution	Nonsynonymous coding	31%
MM02T	PLEC	plectin	CCDS43772.1	chr8_144997730-144997730_C_T	2260E>K	Substitution	Nonsynonymous coding	31%
MM02T	PTPRA	protein tyrosine phosphatase, receptor type, A	CCDS13038.1	chr20_3002812-3002812_A_G	425K>R	Substitution	Nonsynonymous coding	56%
MM02T	SCN4A	sodium channel, voltage-gated, type IV, alpha subunit	CCDS45761.1	chr17_62018831-62018831_G_A	1604A>V	Substitution	Nonsynonymous coding	62%
MM02T	SEZ6L	seizure related 6 homolog (mouse)-like	CCDS13833.1	chr22_26747038-26747038_G_A	810G>R	Substitution	Nonsynonymous coding	48%
MM02T	SHISA3	shisa homolog 3 (Xenopus laevis)	CCDS33979.1	chr4_42400096-42400096_G_T	8C>F	Substitution	Nonsynonymous coding	88%
MM02T	SNAP25	synaptosomal-associated protein, 25kDa	CCDS13110.1	chr20_10273881-10273881_C_T	79T>M	Substitution	Nonsynonymous coding	53%
MM02T	SPOP	speckle-type POZ protein	CCDS11551.1	chr17_47696599-47696599_T_C	117M>V	Substitution	Nonsynonymous coding	64%
MM02T	THADA	thyroid adenoma associated	CCDS46268.1	chr2_43519337-43519337_C_T	1615D>N	Substitution	Nonsynonymous coding	27%
MM02T	TMEM82	transmembrane protein 82	CCDS30608.1	chr1_16069091-16069091_G_A	13G>S	Substitution	Nonsynonymous coding	48%
MM02T	TP53	tumor protein p53	CCDS11118.1	chr17_7577565-7577565_T_C	239N>S	Substitution	Nonsynonymous coding	88%
MM02T	TPM1	tropomyosin 1 (alpha)	CCDS32262.1	chr15_63353928-63353928_G_A	194E>K	Substitution	Nonsynonymous coding	16%
MM02T	TREML4	triggering receptor expressed on myeloid cells-like 4	CCDS34446.1	chr6_41196683-41196683_C_A	99L>M	Substitution	Nonsynonymous coding	13%
MM02T	UBR2	ubiquitin protein ligase E3 component n-recognin 2	CCDS4870.1	chr6_42647537-42647537_A_	NA	Deletion	Frameshift	21%
MM02T	UNC79	unc-79 homolog (C. elegans)	CCDS9911.2	chr14_94160716-94160716_A_T	2364L>F	Substitution	Nonsynonymous coding	58%
MM02T	VPS13A	vacuolar protein sorting 13 homolog A (S. cerevisiae)	CCDS6655.1	chr9_79862242-79862242_T_G	656I>M	Substitution	Nonsynonymous coding	46%
MM02T	VSTM1	V-set and transmembrane domain containing 1	CCDS12872.1	chr19_54545044-54545044_C_A	194V>L	Substitution	Nonsynonymous coding	40%
MM02T	WDR74	WD repeat domain 74	CCDS44630.1	chr11_62603008-62603008_G_A	_ISV-4>	Substitution	Splice site acceptor	13%
MM03T	APCS	amyloid P component, serum	CCDS1186.1	chr1_159558415-159558415_C_A	197L>I	Substitution	Nonsynonymous coding	19%
MM03T	APH1B	anterior pharynx defective 1 homolog B (C. elegans)	CCDS10184.1	chr15_63569833-63569833_C_T	4A>V	Substitution	Nonsynonymous coding	27%
MM03T	APOB	apolipoprotein B (including Ag(x) antigen)	CCDS1703.1	chr2_21232560-21232560_C_A	2394V>F	Substitution	Nonsynonymous coding	53%
MM03T	CDHR3	cadherin-related family member 3	CCDS47684.1	chr7_105645093-105645093_G_A	338V>I	Substitution	Nonsynonymous coding	12%
MM03T	CMTM6	CKLF-like MARVEL transmembrane domain containing 6	CCDS2653.1	chr3_32529532-32529532_T_C	NA	Substitution	Splice site acceptor	52%
MM03T	COBL	cordon-bleu WH2 repeat protein	CCDS34637.1	chr7_51111341-51111341_G_A	382P>L	Substitution	Nonsynonymous coding	10%
MM03T	DENND5A	DENN/MADD domain containing 5A	CCDS31423.1	chr11_9191476-9191476_G_A	693P>L	Substitution	Nonsynonymous coding	14%
MM03T	DICER1	dicer 1, ribonuclease type III	CCDS9931.1	chr14_95560464-95560464_C_T	1709D>N	Substitution	Nonsynonymous coding	42%
MM03T	DOPEY2	dopey family member 2	CCDS13643.1	chr21_37618389-37618389_G_C	1371V>L	Substitution	Nonsynonymous coding	23%
MM03T	EAF2	ELL associated factor 2	CCDS3006.1	chr3_121575958-121575958_G_A	147E>K	Substitution	Nonsynonymous coding	23%

MM03T	ESCO1	establishment of cohesion 1 homolog 1 (S. cerevisiae)	CCDS32800.1	chr18_19154689-19154689_G_A	39S>L	Substitution	Nonsynonymous coding	10%
MM03T	EXT2	exostosin 2	CCDS7908.1	chr11_44146445-44146445_G_A	284D>N	Substitution	Nonsynonymous coding	11%
MM03T	FAM209A	family with sequence similarity 209, member A	CCDS33493.1	chr20_55100084-55100084_C_A	74Q>K	Substitution	Nonsynonymous coding	16%
MM03T	GPATCH2L	G patch domain containing 2-like	CCDS9848.1	chr14_76621212-76621212_C_G	169S>C	Substitution	Nonsynonymous coding	30%
MM03T	GPR179	G protein-coupled receptor 179	CCDS42308.1	chr17_36485971-36485971_T_C	1161T>A	Substitution	Nonsynonymous coding	49%
MM03T	HSPA12A	heat shock 70kDa protein 12A	CCDS41569.1	chr10_118434798-118434798_G_A	508Q>X	Substitution	Nonsense	32%
MM03T	KCNH3	potassium voltage-gated channel, subfamily H (eag-related), member 3	CCDS8786.1	chr12_49937758-49937758_C_A	295P>Q	Substitution	Nonsynonymous coding	37%
MM03T	KY	kyphoscoliosis peptidase	CCDS46920.1	chr3_134339613-134339613_C_A	190W>C	Substitution	Nonsynonymous coding	28%
MM03T	LAMB3	laminin, beta 3	CCDS1487.1	chr1_209799150-209799150_C_T	607A>T	Substitution	Nonsynonymous coding	39%
MM03T	MUM1L1	melanoma associated antigen (mutated) 1-like 1	ENST00000337685	chrX_105451286-105451286_G_T	621D>Y	Substitution	Nonsynonymous coding	27%
MM03T	OR8G5	olfactory receptor, family 8, subfamily G, member 5	NM_001005198	chr11_124134891-124134891_G_C	57E>Q	Substitution	Nonsynonymous coding	41%
MM03T	OR9G1	olfactory receptor, family 9, subfamily G, member 1	CCDS31536.1	chr11_56468660-56468660_C_A	266S>Y	Substitution	Nonsynonymous coding	17%
MM03T	PRDM15	PR domain containing 15	CCDS13676.1	chr21_43236168-43236168_C_T	1128R>H	Substitution	Nonsynonymous coding	23%
MM03T	PROS1	protein S (alpha)	CCDS2923.1	chr3_93646242-93646242_T_A	29K>M	Substitution	Nonsynonymous coding	21%
MM03T	SH3BP4	SH3-domain binding protein 4	CCDS2513.1	chr2_235951000-235951000_C_A	529F>L	Substitution	Nonsynonymous coding	23%
MM03T	SNX9	sorting nexin 9	CCDS5253.1	chr6_158359770-158359770_G_C	547A>P	Substitution	Nonsynonymous coding	14%
MM03T	STK36	serine/threonine kinase 36	CCDS2421.1	chr2_219564039-219564039_C_T	1258R>W	Substitution	Nonsynonymous coding	14%
MM03T	TBC1D8	TBC1 domain family, member 8 (with GRAM domain)	CCDS46375.1	chr2_101624725-101624725_A_G	994I>T	Substitution	Nonsynonymous coding	53%
MM03T	TDRD6	tudor domain containing 6	CCDS34470.1	chr6_46659722-46659722_A_	NA	Deletion	Frameshift	38%
MM03T	TP53	tumor protein p53	CCDS11118.1	chr17_7578395-7578395_G_T	179H>N	Substitution	Nonsynonymous coding	66%
MM04T	MARCH6	membrane-associated ring finger (C3HC4) 6, E3 ubiquitin protein ligase	CCDS34135.1	chr5_10378897-10378897_G_A	48R>Q	Substitution	Nonsynonymous coding	29%
MM04T	MARCH7	membrane-associated ring finger (C3HC4) 7, E3 ubiquitin protein ligase	CCDS2210.1	chr2_160621182-160621182_G_A	685E>K	Substitution	Nonsynonymous coding	34%
MM04T	MARCH10	membrane-associated ring finger (C3HC4) 10, E3 ubiquitin protein ligase	CCDS11635.1	chr17_60814055-60814055_C_T	392G>S	Substitution	Nonsynonymous coding	27%
MM04T	SEPT14	septin 14	CCDS5519.2	chr7_55872965-55872965_C_A	369E>X	Substitution	Nonsense	33%
MM04T	A1CF	APOBEC1 complementation factor	CCDS7242.1	chr10_52569732-52569732_G_T	519L>M	Substitution	Nonsynonymous coding	18%
MM04T	A2M	alpha-2-macroglobulin	CCDS44827.1	chr12_9225468-9225468_C_T	NA	Substitution	Splice site acceptor	43%
MM04T	A2ML1	alpha-2-macroglobulin-like 1	CCDS8596.2	chr12_8976401-8976401_A_G	111K>R	Substitution	Nonsynonymous coding	32%
MM04T	A2ML1	alpha-2-macroglobulin-like 1	CCDS8596.2	chr12_9006837-9006837_C_A	902L>I	Substitution	Nonsynonymous coding	20%
MM04T	A2ML1	alpha-2-macroglobulin-like 1	CCDS8596.2	chr12_8975302-8975302_G_T	19E>X	Substitution	Nonsense	30%
MM04T	AADACL2	arylacetamide deacetylase-like 2	CCDS3161.2	chr3_151475150-151475150_C_A	325S>Y	Substitution	Nonsynonymous coding	18%
MM04T	AAMDC	adipogenesis associated, Mth938 domain containing	CCDS8254.1	chr11_77583250-77583250_G_T	86K>N	Substitution	Nonsynonymous coding	33%
MM04T	AASDH	aminoadipate-semialdehyde dehydrogenase	CCDS3504.1	chr4_57215746-57215746_G_T	724S>Y	Substitution	Nonsynonymous coding	26%

MM04T	AASDHPPT	aminoadipate-semialdehyde dehydrogenase-phosphopantetheinyl transferase	CCDS31664.1	chr11_105962145-105962145_G_T	212V>F	Substitution	Nonsynonymous coding	24%
MM04T	AASS	aminoadipate-semialdehyde synthase	CCDS5783.1	chr7_121756793-121756793_G_A	263T>M	Substitution	Nonsynonymous coding	22%
MM04T	AATK	apoptosis-associated tyrosine kinase	CCDS45807.1	chr17_79094942-79094942_G_A	932P>S	Substitution	Nonsynonymous coding	31%
MM04T	ABCA1	ATP-binding cassette, sub-family A (ABC1), member 1	CCDS6762.1	chr9_107562184-107562184_T_G	1620K>T	Substitution	Nonsynonymous coding	33%
MM04T	ABCA1	ATP-binding cassette, sub-family A (ABC1), member 1	CCDS6762.1	chr9_107593262-107593262_C_A	612E>D	Substitution	Nonsynonymous coding	24%
MM04T	ABCA12	ATP-binding cassette, sub-family A (ABC1), member 12	CCDS33372.1	chr2_215854093-215854093_A_C	1263I>M	Substitution	Nonsynonymous coding	32%
MM04T	ABCA12	ATP-binding cassette, sub-family A (ABC1), member 12	CCDS33372.1	chr2_215884458-215884458_C_A	450E>D	Substitution	Nonsynonymous coding	29%
MM04T	ABCA12	ATP-binding cassette, sub-family A (ABC1), member 12	CCDS33372.1	chr2_215884496-215884496_C_T	438E>K	Substitution	Nonsynonymous coding	31%
MM04T	ABCA12	ATP-binding cassette, sub-family A (ABC1), member 12	CCDS33372.1	chr2_215917230-215917230_C_T	163R>Q	Substitution	Nonsynonymous coding	27%
MM04T	ABCA12	ATP-binding cassette, sub-family A (ABC1), member 12	CCDS33372.1	chr2_215919325-215919325_T_G	127Q>H	Substitution	Nonsynonymous coding	22%
MM04T	ABCA13	ATP-binding cassette, sub-family A (ABC1), member 13	CCDS47584.1	chr7_48315783-48315783_A_T	2174N>Y	Substitution	Nonsynonymous coding	41%
MM04T	ABCA13	ATP-binding cassette, sub-family A (ABC1), member 13	CCDS47584.1	chr7_48316130-48316130_A_C	2289K>N	Substitution	Nonsynonymous coding	31%
MM04T	ABCA13	ATP-binding cassette, sub-family A (ABC1), member 13	CCDS47584.1	chr7_48318910-48318910_G_T	2707D>Y	Substitution	Nonsynonymous coding	26%
MM04T	ABCA13	ATP-binding cassette, sub-family A (ABC1), member 13	CCDS47584.1	chr7_48319407-48319407_G_T	2872E>D	Substitution	Nonsynonymous coding	25%
MM04T	ABCA13	ATP-binding cassette, sub-family A (ABC1), member 13	CCDS47584.1	chr7_48684325-48684325_C_A	5019S>Y	Substitution	Nonsynonymous coding	24%
MM04T	ABCA4	ATP-binding cassette, sub-family A (ABC1), member 4	CCDS747.1	chr1_94526106-94526106_G_A	716T>M	Substitution	Nonsynonymous coding	18%
MM04T	ABCA5	ATP-binding cassette, sub-family A (ABC1), member 5	CCDS11685.1	chr17_67293434-67293434_C_A	445R>I	Substitution	Nonsynonymous coding	17%
MM04T	ABCA5	ATP-binding cassette, sub-family A (ABC1), member 5	CCDS11685.1	chr17_67300881-67300881_C_T	287A>T	Substitution	Nonsynonymous coding	16%
MM04T	ABCA6	ATP-binding cassette, sub-family A (ABC1), member 6	CCDS11683.1	chr17_67107010-67107010_T_G	735K>T	Substitution	Nonsynonymous coding	26%
MM04T	ABCA6	ATP-binding cassette, sub-family A (ABC1), member 6	CCDS11683.1	chr17_67108407-67108407_T_G	683K>N	Substitution	Nonsynonymous coding	31%
MM04T	ABCA8	ATP-binding cassette, sub-family A (ABC1), member 8	CCDS11680.1	chr17_66879941-66879941_G_T	1193P>H	Substitution	Nonsynonymous coding	33%
MM04T	ABCA8	ATP-binding cassette, sub-family A (ABC1), member 8	CCDS11680.1	chr17_66879986-66879986_C_T	1178R>Q	Substitution	Nonsynonymous coding	42%
MM04T	ABCA8	ATP-binding cassette, sub-family A (ABC1), member 8	CCDS11680.1	chr17_66902188-66902188_A_G	759S>P	Substitution	Nonsynonymous coding	23%
MM04T	ABCB1	ATP-binding cassette, sub-family B (MDR/TAP), member 1	CCDS5608.1	chr7_87170695-87170695_G_T	766S>Y	Substitution	Nonsynonymous coding	34%
MM04T	ABCB11	ATP-binding cassette, sub-family B (MDR/TAP), member 11	CCDS46444.1	chr2_169787308-169787308_G_A	1093S>L	Substitution	Nonsynonymous coding	38%
MM04T	ABCB4	ATP-binding cassette, sub-family B (MDR/TAP), member 4	CCDS5606.1	chr7_87043027-87043027_T_C	897T>A	Substitution	Nonsynonymous coding	16%
MM04T	ABCB4	ATP-binding cassette, sub-family B (MDR/TAP), member 4	CCDS5606.1	chr7_87046661-87046661_T_G	883K>N	Substitution	Nonsynonymous coding	33%
MM04T	ABCB5	ATP-binding cassette, sub-family B (MDR/TAP), member 5	NM_001163941	chr7_20687224-20687224_G_A	350A>T	Substitution	Nonsynonymous coding	26%
MM04T	ABCB5	ATP-binding cassette, sub-family B (MDR/TAP), member 5	NM_001163993	chr7_20706384-20706384_A_C	125K>N	Substitution	Nonsynonymous coding	42%
MM04T	ABCB5	ATP-binding cassette, sub-family B (MDR/TAP), member 5	CCDS5371.1	chr7_20721180-20721180_G_A	142R>Q	Substitution	Nonsynonymous coding	28%
MM04T	ABCB7	ATP-binding cassette, sub-family B (MDR/TAP), member 7	CCDS14428.1	chrX_74273266-74273266_C_A	734R>I	Substitution	Nonsynonymous coding	20%
MM04T	ABCB9	ATP-binding cassette, sub-family B (MDR/TAP), member 9	CCDS9241.1	chr12_123425402-123425402_C_A	507E>D	Substitution	Nonsynonymous coding	27%

MM04T	ABCC11	ATP-binding cassette, sub-family C (CFTR/MRP), member 11	CCDS10732.1	chr16_48239414-48239414_C_T	572G>E	Substitution	Nonsynonymous coding	22%
MM04T	ABCC11	ATP-binding cassette, sub-family C (CFTR/MRP), member 11	CCDS10732.1	chr16_48250116-48250116_G_A	287S>L	Substitution	Nonsynonymous coding	38%
MM04T	ABCC12	ATP-binding cassette, sub-family C (CFTR/MRP), member 12	CCDS10730.1	chr16_48119612-48119612_C_A	1240M>I	Substitution	Nonsynonymous coding	34%
MM04T	ABCC12	ATP-binding cassette, sub-family C (CFTR/MRP), member 12	CCDS10730.1	chr16_48134909-48134909_C_A	971R>I	Substitution	Nonsynonymous coding	20%
MM04T	ABCC2	ATP-binding cassette, sub-family C (CFTR/MRP), member 2	CCDS7484.1	chr10_101563849-101563849_C_T	428A>V	Substitution	Nonsynonymous coding	30%
MM04T	ABCC4	ATP-binding cassette, sub-family C (CFTR/MRP), member 4	CCDS9474.1	chr13_95839079-95839079_C_A	474R>I	Substitution	Nonsynonymous coding	18%
MM04T	ABCC4	ATP-binding cassette, sub-family C (CFTR/MRP), member 4	CCDS9474.1	chr13_95899972-95899972_C_A	37R>L	Substitution	Nonsynonymous coding	24%
MM04T	ABCC4	ATP-binding cassette, sub-family C (CFTR/MRP), member 4	CCDS9474.1	chr13_95673939-95673939_A_	NA	Deletion	Splice site acceptor	11%
MM04T	ABCC8	ATP-binding cassette, sub-family C (CFTR/MRP), member 8	CCDS31437.1	chr11_17424260-17424260_G_T	1200L>I	Substitution	Nonsynonymous coding	28%
MM04T	ABCC9	ATP-binding cassette, sub-family C (CFTR/MRP), member 9	CCDS8693.1	chr12_22005054-22005054_G_A	916R>W	Substitution	Nonsynonymous coding	43%
MM04T	ABCC9	ATP-binding cassette, sub-family C (CFTR/MRP), member 9	CCDS8693.1	chr12_22068777-22068777_C_A	214R>I	Substitution	Nonsynonymous coding	24%
MM04T	ABCD2	ATP-binding cassette, sub-family D (ALD), member 2	CCDS8734.1	chr12_39967522-39967522_G_T	667L>I	Substitution	Nonsynonymous coding	26%
MM04T	ABCD2	ATP-binding cassette, sub-family D (ALD), member 2	CCDS8734.1	chr12_39980008-39980008_G_T	580L>M	Substitution	Nonsynonymous coding	23%
MM04T	ABCD3	ATP-binding cassette, sub-family D (ALD), member 3	CCDS749.1	chr1_94964528-94964528_C_T	520R>X	Substitution	Nonsense	20%
MM04T	ABCF1	ATP-binding cassette, sub-family F (GCN20), member 1	CCDS34380.1	chr6_30552322-30552322_G_A	457R>H	Substitution	Nonsynonymous coding	20%
MM04T	ABCF1	ATP-binding cassette, sub-family F (GCN20), member 1	CCDS34380.1	chr6_30557940-30557940_C_T	750R>X	Substitution	Nonsense	22%
MM04T	ABCF3	ATP-binding cassette, sub-family F (GCN20), member 3	CCDS3254.1	chr3_183906012-183906012_C_T	218T>M	Substitution	Nonsynonymous coding	21%
MM04T	ABHD16B	abhydrolase domain containing 16B	CCDS13539.1	chr20_62493425-62493425_G_A	178E>K	Substitution	Nonsynonymous coding	17%
MM04T	ABHD2	abhydrolase domain containing 2	CCDS10348.1	chr15_89736523-89736523_C_A	352L>I	Substitution	Nonsynonymous coding	12%
MM04T	ABHD4	abhydrolase domain containing 4	CCDS9572.1	chr14_23072978-23072978_C_T	212P>S	Substitution	Nonsynonymous coding	37%
MM04T	ABHD5	abhydrolase domain containing 5	CCDS2711.1	chr3_43756447-43756447_C_A	224L>I	Substitution	Nonsynonymous coding	13%
MM04T	ABI3BP	ABI family, member 3 (NESH) binding protein	CCDS46880.1	chr3_100473530-100473530_T_G	908E>A	Substitution	Nonsynonymous coding	31%
MM04T	ABI3BP	ABI family, member 3 (NESH) binding protein	ENST00000383691	chr3_100539960-100539960_G_A	NA	Substitution	Splice site acceptor	29%
MM04T	ABL2	v-abl Abelson murine leukemia viral oncogene homolog 2	CCDS30947.1	chr1_179077171-179077171_C_A	1077E>D	Substitution	Nonsynonymous coding	34%
MM04T	ABL2	v-abl Abelson murine leukemia viral oncogene homolog 2	CCDS30947.1	chr1_179077544-179077544_G_T	953S>Y	Substitution	Nonsynonymous coding	22%
MM04T	ABL2	v-abl Abelson murine leukemia viral oncogene homolog 2	CCDS30947.1	chr1_179078135-179078135_A_G	756L>S	Substitution	Nonsynonymous coding	26%
MM04T	ABL2	v-abl Abelson murine leukemia viral oncogene homolog 2	CCDS41441.1	chr1_179112145-179112145_C_A	12S>I	Substitution	Nonsynonymous coding	18%
MM04T	ABLIM1	actin binding LIM protein 1	CCDS7590.1	chr10_116361657-116361657_C_A	103G>V	Substitution	Nonsynonymous coding	19%
MM04T	ABLIM2	actin binding LIM protein family, member 2	CCDS47016.1	chr4_8009775-8009775_G_T	469F>L	Substitution	Nonsynonymous coding	31%
MM04T	ABO	ABO blood group (transferase A, alpha 1-3-N-acetylgalactosaminyltransferase; transferase	ENST00000319878	chr9_136131183-136131183_C_T	311R>H	Substitution	Nonsynonymous coding	25%
MM04T	AC002472.13	Leucine-rich repeat-containing protein LOC400891 [Source:UniProtKB/Swiss-	ENST00000342608	chr22_21401752-21401752_G_T	83E>X	Substitution	Nonsense	31%
MM04T	AC005544.1	Uncharacterized protein [Source:UniProtKB/TrEMBL;Acc:H0Y3C3]	ENST00000375684	chr17_65028110-65028110_A_G	188N>S	Substitution	Nonsynonymous coding	25%

MM04T	AC007249.3	-	ENST00000404616	chr2_10595633-10595633_C_T	49S>F	Substitution	Nonsynonymous coding	35%
MM04T	AC018755.11	HCG2008157; Uncharacterized protein; cDNA FLJ30403 fis, clone BRACE2008480	ENST00000301439	chr19_52096058-52096058_G_T	207S>Y	Substitution	Nonsynonymous coding	34%
MM04T	AC074212.3	Uncharacterized protein [Source:UniProtKB/TrEMBL;Acc:C9JSJ3]	ENST00000457052	chr19_46256985-46256985_G_T	165Q>H	Substitution	Nonsynonymous coding	36%
MM04T	AC104809.3	Uncharacterized protein [Source:UniProtKB/TrEMBL;Acc:H7BZ55]	ENST00000430980	chr2_241873875-241873875_C_T	40R>C	Substitution	Nonsynonymous coding	22%
MM04T	AC133919.6	Uncharacterized protein; cDNA FLJ52651 [Source:UniProtKB/TrEMBL;Acc:B7Z8Y8]	ENST00000409768	chr16_90110235-90110235_G_T	91H>N	Substitution	Nonsynonymous coding	37%
MM04T	ACACA	acetyl-CoA carboxylase alpha	CCDS42302.1	chr17_35597396-35597396_C_A	1039Q>H	Substitution	Nonsynonymous coding	31%
MM04T	ACACA	acetyl-CoA carboxylase alpha	CCDS42302.1	chr17_35605558-35605558_C_A	708D>Y	Substitution	Nonsynonymous coding	29%
MM04T	ACACB	acetyl-CoA carboxylase beta	CCDS31898.1	chr12_109644622-109644622_G_T	1007M>I	Substitution	Nonsynonymous coding	23%
MM04T	ACACB	acetyl-CoA carboxylase beta	CCDS31898.1	chr12_109674971-109674971_T_C	1483F>S	Substitution	Nonsynonymous coding	32%
MM04T	ACACB	acetyl-CoA carboxylase beta	CCDS31898.1	chr12_109674982-109674982_C_T	1487R>C	Substitution	Nonsynonymous coding	21%
MM04T	ACAN	aggrecan	NM_013227	chr15_89400588-89400588_C_A	1591S>Y	Substitution	Nonsynonymous coding	23%
MM04T	ACAP1	ArfGAP with coiled-coil, ankyrin repeat and PH domains 1	CCDS11101.1	chr17_7254650-7254650_C_T	720S>F	Substitution	Nonsynonymous coding	26%
MM04T	ACAT2	acetyl-CoA acetyltransferase 2	CCDS5268.1	chr6_160197275-160197275_C_T	243T>M	Substitution	Nonsynonymous coding	25%
MM04T	ACBD3	acyl-CoA binding domain containing 3	CCDS1551.1	chr1_226346962-226346962_G_T	276L>I	Substitution	Nonsynonymous coding	31%
MM04T	ACCS	1-aminocyclopropane-1-carboxylate synthase homolog (Arabidopsis)(non-functional)	CCDS7907.1	chr11_44105064-44105064_G_A	449E>K	Substitution	Nonsynonymous coding	32%
MM04T	ACIN1	apoptotic chromatin condensation inducer 1	CCDS9587.1	chr14_23549036-23549036_G_T	561S>Y	Substitution	Nonsynonymous coding	33%
MM04T	ACMSD	aminocarboxymuconate semialdehyde decarboxylase	CCDS2173.2	chr2_135630167-135630167_C_A	269H>N	Substitution	Nonsynonymous coding	29%
MM04T	ACO1	aconitase 1, soluble	CCDS6525.1	chr9_32418167-32418167_G_T	149R>I	Substitution	Nonsynonymous coding	25%
MM04T	ACOT9	acyl-CoA thioesterase 9	CCDS43924.1	chrX_23723657-23723657_C_A	330E>X	Substitution	Nonsense	33%
MM04T	ACP6	acid phosphatase 6, lysophosphatidic	CCDS928.1	chr1_147131542-147131542_G_T	150L>I	Substitution	Nonsynonymous coding	20%
MM04T	ACPP	acid phosphatase, prostate	CCDS46916.1	chr3_132051046-132051046_G_A	105R>Q	Substitution	Nonsynonymous coding	19%
MM04T	ACPP	acid phosphatase, prostate	CCDS46916.1	chr3_132075555-132075555_C_T	332R>W	Substitution	Nonsynonymous coding	21%
MM04T	ACSBG1	acyl-CoA synthetase bubblegum family member 1	CCDS10298.1	chr15_78472040-78472040_G_T	446Q>K	Substitution	Nonsynonymous coding	30%
MM04T	ACSBG2	acyl-CoA synthetase bubblegum family member 2	CCDS12159.1	chr19_6187410-6187410_C_A	553L>M	Substitution	Nonsynonymous coding	27%
MM04T	ACSL1	acyl-CoA synthetase long-chain family member 1	CCDS3839.1	chr4_185687835-185687835_G_A	402R>C	Substitution	Nonsynonymous coding	19%
MM04T	ACSL1	acyl-CoA synthetase long-chain family member 1	CCDS3839.1	chr4_185724605-185724605_C_T	22V>M	Substitution	Nonsynonymous coding	30%
MM04T	ACSL5	acyl-CoA synthetase long-chain family member 5	CCDS7572.1	chr10_114176679-114176679_T_C	429F>L	Substitution	Nonsynonymous coding	24%
MM04T	ACSM3	acyl-CoA synthetase medium-chain family member 3	CCDS10589.1	chr16_20792355-20792355_C_A	281A>E	Substitution	Nonsynonymous coding	18%
MM04T	ACSM3	acyl-CoA synthetase medium-chain family member 3	CCDS10589.1	chr16_20803358-20803358_G_A	454G>D	Substitution	Nonsynonymous coding	16%
MM04T	ACTN4	actinin, alpha 4	CCDS12518.1	chr19_39200068-39200068_G_A	229E>K	Substitution	Nonsynonymous coding	27%
MM04T	ACTR10	actin-related protein 10 homolog (S. cerevisiae)	CCDS32090.1	chr14_58698938-58698938_G_A	342R>Q	Substitution	Nonsynonymous coding	70%
MM04T	ACTR3	ARP3 actin-related protein 3 homolog (yeast)	CCDS33277.1	chr2_114709367-114709367_G_T	341R>I	Substitution	Nonsynonymous coding	26%

MM04T	ACTR3	ARP3 actin-related protein 3 homolog (yeast)	CCDS33277.1	chr2_114699799-114699799_G_T	241E>X	Substitution	Nonsense	29%
MM04T	ACVR2A	activin A receptor, type IIA	CCDS33301.1	chr2_148657128-148657128_T_C	122V>A	Substitution	Nonsynonymous coding	32%
MM04T	ADAD1	adenosine deaminase domain containing 1 (testis-specific)	CCDS34058.1	chr4_123302222-123302222_C_T	83P>L	Substitution	Nonsynonymous coding	39%
MM04T	ADAD2	adenosine deaminase domain containing 2	CCDS10944.1	chr16_84228698-84228698_C_T	283R>C	Substitution	Nonsynonymous coding	32%
MM04T	ADAL	adenosine deaminase-like	CCDS32214.1	chr15_43627966-43627966_C_A	46L>I	Substitution	Nonsynonymous coding	25%
MM04T	ADAM11	ADAM metallopeptidase domain 11	CCDS11486.1	chr17_42850628-42850628_G_T	NA	Substitution	Splice site acceptor	17%
MM04T	ADAM21	ADAM metallopeptidase domain 21	CCDS9804.1	chr14_70925053-70925053_C_A	279F>L	Substitution	Nonsynonymous coding	27%
MM04T	ADAM21	ADAM metallopeptidase domain 21	CCDS9804.1	chr14_70926127-70926127_G_T	637E>D	Substitution	Nonsynonymous coding	29%
MM04T	ADAM23	ADAM metallopeptidase domain 23	CCDS2369.1	chr2_207457355-207457355_G_T	658G>V	Substitution	Nonsynonymous coding	24%
MM04T	ADAM23	ADAM metallopeptidase domain 23	CCDS2369.1	chr2_207310045-207310045_G_T	77E>X	Substitution	Nonsense	32%
MM04T	ADAM23	ADAM metallopeptidase domain 23	CCDS2369.1	chr2_207454177-207454177_G_T	633E>X	Substitution	Nonsense	24%
MM04T	ADAM28	ADAM metallopeptidase domain 28	CCDS34865.1	chr8_24171006-24171006_G_T	163K>N	Substitution	Nonsynonymous coding	27%
MM04T	ADAM30	ADAM metallopeptidase domain 30	CCDS907.1	chr1_120437861-120437861_G_A	367R>C	Substitution	Nonsynonymous coding	26%
MM04T	ADAM33	ADAM metallopeptidase domain 33	CCDS13058.1	chr20_3649960-3649960_G_A	797S>L	Substitution	Nonsynonymous coding	33%
MM04T	ADAM7	ADAM metallopeptidase domain 7	CCDS6045.1	chr8_24323239-24323239_G_A	114V>I	Substitution	Nonsynonymous coding	18%
MM04T	ADAM7	ADAM metallopeptidase domain 7	CCDS6045.1	chr8_24344778-24344778_G_A	347E>K	Substitution	Nonsynonymous coding	26%
MM04T	ADAM9	ADAM metallopeptidase domain 9	CCDS6112.1	chr8_38880697-38880697_G_A	256R>Q	Substitution	Nonsynonymous coding	26%
MM04T	ADAMTS1	ADAM metallopeptidase with thrombospondin type 1 motif, 1	ENST00000451462	chr21_28215837-28215837_C_	NA	Deletion	Frameshift	67%
MM04T	ADAMTS1	ADAM metallopeptidase with thrombospondin type 1 motif, 1	CCDS33524.1	chr21_28214672-28214672_G_T	355L>I	Substitution	Nonsynonymous coding	41%
MM04T	ADAMTS12	ADAM metallopeptidase with thrombospondin type 1 motif, 12	CCDS34140.1	chr5_33549455-33549455_G_A	1387R>C	Substitution	Nonsynonymous coding	30%
MM04T	ADAMTS16	ADAM metallopeptidase with thrombospondin type 1 motif, 16	CCDS43299.1	chr5_5200357-5200357_C_T	476R>C	Substitution	Nonsynonymous coding	46%
MM04T	ADAMTS16	ADAM metallopeptidase with thrombospondin type 1 motif, 16	CCDS43299.1	chr5_5262767-5262767_C_T	NA	Substitution	Splice site acceptor	32%
MM04T	ADAMTS17	ADAM metallopeptidase with thrombospondin type 1 motif, 17	CCDS10383.1	chr15_100533269-100533269_G_T	978T>N	Substitution	Nonsynonymous coding	34%
MM04T	ADAMTS19	ADAM metallopeptidase with thrombospondin type 1 motif, 19	CCDS4146.1	chr5_128983569-128983569_C_T	656R>X	Substitution	Nonsense	41%
MM04T	ADAMTS2	ADAM metallopeptidase with thrombospondin type 1 motif, 2	CCDS4444.1	chr5_178541020-178541020_C_A	1162D>Y	Substitution	Nonsynonymous coding	22%
MM04T	ADAMTS20	ADAM metallopeptidase with thrombospondin type 1 motif, 20	ENST00000389420	chr12_43896022-43896022_G_A	267A>V	Substitution	Nonsynonymous coding	26%
MM04T	ADAMTS3	ADAM metallopeptidase with thrombospondin type 1 motif, 3	CCDS3553.1	chr4_73434457-73434457_A_G	8L>S	Substitution	Nonsynonymous coding	33%
MM04T	ADAMTS4	ADAM metallopeptidase with thrombospondin type 1 motif, 4	CCDS1223.1	chr1_161160960-161160960_G_T	828L>I	Substitution	Nonsynonymous coding	21%
MM04T	ADAMTS5	ADAM metallopeptidase with thrombospondin type 1 motif, 5	CCDS13579.1	chr21_28306867-28306867_G_A	536A>V	Substitution	Nonsynonymous coding	22%
MM04T	ADAMTS7	ADAM metallopeptidase with thrombospondin type 1 motif, 7	CCDS32303.1	chr15_79092842-79092842_G_A	50R>X	Substitution	Nonsense	30%
MM04T	ADAMTS9	ADAM metallopeptidase with thrombospondin type 1 motif, 9	CCDS2903.1	chr3_64606845-64606845_G_A	920R>W	Substitution	Nonsynonymous coding	26%
MM04T	ADAMTSL2	ADAMTS-like 2	CCDS6976.1	chr9_136420724-136420724_G_A	441E>K	Substitution	Nonsynonymous coding	33%

MM04T	ADAMTSL2	ADAMTS-like 2	CCDS6976.1	chr9_136435468-136435468_G_T	811G>W	Substitution	Nonsynonymous coding	23%
MM04T	ADAMTSL3	ADAMTS-like 3	CCDS10326.1	chr15_84473406-84473406_T_G	108C>G	Substitution	Nonsynonymous coding	33%
MM04T	ADAMTSL3	ADAMTS-like 3	CCDS10326.1	chr15_84705628-84705628_C_T	1620R>W	Substitution	Nonsynonymous coding	20%
MM04T	ADAMTSL4	ADAMTS-like 4	CCDS955.1	chr1_150526447-150526447_G_A	327R>H	Substitution	Nonsynonymous coding	36%
MM04T	ADARB1	adenosine deaminase, RNA-specific, B1	CCDS33589.1	chr21_46604882-46604882_C_T	521R>W	Substitution	Nonsynonymous coding	23%
MM04T	ADC	arginine decarboxylase	CCDS375.1	chr1_33560255-33560255_G_A	232V>I	Substitution	Nonsynonymous coding	32%
MM04T	ADCY1	adenylate cyclase 1 (brain)	CCDS34631.1	chr7_45743334-45743334_G_A	903D>N	Substitution	Nonsynonymous coding	35%
MM04T	ADCY1	adenylate cyclase 1 (brain)	CCDS34631.1	chr7_45750169-45750169_G_A	992R>H	Substitution	Nonsynonymous coding	27%
MM04T	ADCY10	adenylate cyclase 10 (soluble)	CCDS1265.1	chr1_167815331-167815331_A_C	870F>V	Substitution	Nonsynonymous coding	35%
MM04T	ADCY2	adenylate cyclase 2 (brain)	CCDS3872.2	chr5_7727357-7727357_G_T	618Q>H	Substitution	Nonsynonymous coding	28%
MM04T	ADCY2	adenylate cyclase 2 (brain)	CCDS3872.2	chr5_7709330-7709330_C_T	470R>X	Substitution	Nonsense	43%
MM04T	ADCY9	adenylate cyclase 9	CCDS32382.1	chr16_4016512-4016512_G_A	1109T>I	Substitution	Nonsynonymous coding	23%
MM04T	ADCY9	adenylate cyclase 9	CCDS32382.1	chr16_4164654-4164654_G_A	264R>W	Substitution	Nonsynonymous coding	29%
MM04T	ADCYAP1	adenylate cyclase activating polypeptide 1 (pituitary)	CCDS11825.1	chr18_907673-907673_G_A	42A>T	Substitution	Nonsynonymous coding	20%
MM04T	ADCYAP1	adenylate cyclase activating polypeptide 1 (pituitary)	CCDS11825.1	chr18_908267-908267_G_T	82D>Y	Substitution	Nonsynonymous coding	33%
MM04T	ADD2	adducin 2 (beta)	CCDS1906.1	chr2_70901826-70901826_C_A	575K>N	Substitution	Nonsynonymous coding	15%
MM04T	ADD3	adducin 3 (gamma)	CCDS7561.1	chr10_111890220-111890220_G_A	570E>K	Substitution	Nonsynonymous coding	30%
MM04T	ADD3	adducin 3 (gamma)	CCDS7561.1	chr10_111893230-111893230_G_A	659E>K	Substitution	Nonsynonymous coding	30%
MM04T	ADK	adenosine kinase	CCDS7343.1	chr10_76468130-76468130_C_T	339A>V	Substitution	Nonsynonymous coding	29%
MM04T	ADM	adrenomedullin	CCDS7801.1	chr11_10327276-10327276_A_G	10Y>C	Substitution	Nonsynonymous coding	31%
MM04T	ADNP	activity-dependent neuroprotector homeobox	CCDS13433.1	chr20_49510935-49510935_G_T	106L>I	Substitution	Nonsynonymous coding	28%
MM04T	AFF2	AF4/FMR2 family, member 2	CCDS14684.1	chrX_147743630-147743630_G_A	128D>N	Substitution	Nonsynonymous coding	33%
MM04T	AFF2	AF4/FMR2 family, member 2	CCDS14684.1	chrX_148044284-148044284_A_C	910E>D	Substitution	Nonsynonymous coding	28%
MM04T	AFG3L2	AFG3 ATPase family member 3-like 2 (S. cerevisiae)	CCDS11859.1	chr18_12370869-12370869_C_A	91E>X	Substitution	Nonsense	32%
MM04T	AFP	alpha-fetoprotein	CCDS3556.1	chr4_74315801-74315801_C_T	414R>X	Substitution	Nonsense	15%
MM04T	AFTPH	aftiphilin	CCDS46303.1	chr2_64779333-64779333_G_T	242R>M	Substitution	Nonsynonymous coding	24%
MM04T	AFTPH	aftiphilin	CCDS46303.1	chr2_64779656-64779656_G_T	350E>X	Substitution	Nonsense	32%
MM04T	AGA	aspartylglucosaminidase	CCDS3829.1	chr4_178354449-178354449_G_T	287Q>K	Substitution	Nonsynonymous coding	25%
MM04T	AGA	aspartylglucosaminidase	CCDS3829.1	chr4_178355645-178355645_T_G	NA	Substitution	Splice site acceptor	24%
MM04T	AGAP2	ArfGAP with GTPase domain, ankyrin repeat and PH domain 2	CCDS44932.1	chr12_58122118-58122118_C_A	867R>I	Substitution	Nonsynonymous coding	28%
MM04T	AGBL1	ATP/GTP binding protein-like 1	NM_152336	chr15_86822869-86822869_G_T	NA	Substitution	Splice site acceptor	39%
MM04T	AGBL2	ATP/GTP binding protein-like 2	CCDS7944.1	chr11_47711667-47711667_G_T	531S>Y	Substitution	Nonsynonymous coding	18%

MM04T	AGBL3	ATP/GTP binding protein-like 3	CCDS47718.1	chr7_134719115-134719115_G_T	258R>I	Substitution	Nonsynonymous coding	22%
MM04T	AGBL4	ATP/GTP binding protein-like 4	ENST00000334103	chr1_48999797-48999797_G_T	300L>M	Substitution	Nonsynonymous coding	28%
MM04T	AGBL4	ATP/GTP binding protein-like 4	CCDS44137.1	chr1_49100271-49100271_G_T	282S>Y	Substitution	Nonsynonymous coding	21%
MM04T	AGGF1	angiogenic factor with G patch and FHA domains 1	CCDS4035.1	chr5_76330277-76330277_G_T	82R>I	Substitution	Nonsynonymous coding	33%
MM04T	AGL	amylo-alpha-1, 6-glucosidase, 4-alpha-glucanotransferase	CCDS759.1	chr1_100357161-100357161_G_T	NA	Substitution	Splice site acceptor	26%
MM04T	AGMO	alkylglycerol monooxygenase	CCDS34604.1	chr7_15425072-15425072_G_A	358A>V	Substitution	Nonsynonymous coding	33%
MM04T	AGMO	alkylglycerol monooxygenase	CCDS34604.1	chr7_15427047-15427047_C_A	314S>I	Substitution	Nonsynonymous coding	30%
MM04T	AGPAT6	1-acylglycerol-3-phosphate O-acyltransferase 6	CCDS6117.1	chr8_41471931-41471931_G_A	313S>N	Substitution	Nonsynonymous coding	21%
MM04T	AGPAT9	1-acylglycerol-3-phosphate O-acyltransferase 9	CCDS3606.1	chr4_84457914-84457914_G_T	47E>X	Substitution	Nonsense	30%
MM04T	AGPHD1	aminoglycoside phosphotransferase domain containing 1	CCDS42063.1	chr15_78805569-78805569_A_C	47N>H	Substitution	Nonsynonymous coding	35%
MM04T	AGPHD1	aminoglycoside phosphotransferase domain containing 1	CCDS42063.1	chr15_78819794-78819794_C_A	184L>M	Substitution	Nonsynonymous coding	26%
MM04T	AGPS	alkylglycerone phosphate synthase	CCDS2275.1	chr2_178301722-178301722_G_T	193E>X	Substitution	Nonsense	21%
MM04T	AGPS	alkylglycerone phosphate synthase	CCDS2275.1	chr2_178402921-178402921_T_C	659*>Q	Substitution	Nonsynonymous coding	29%
MM04T	AGR2	anterior gradient 2 homolog (Xenopus laevis)	CCDS5364.1	chr7_16841311-16841311_C_A	37R>L	Substitution	Nonsynonymous coding	34%
MM04T	AHCTF1	AT hook containing transcription factor 1	CCDS1629.1	chr1_247027341-247027341_G_T	1142S>Y	Substitution	Nonsynonymous coding	33%
MM04T	AHCYL1	adenosylhomocysteinase-like 1	CCDS818.1	chr1_110561025-110561025_T_C	385L>S	Substitution	Nonsynonymous coding	26%
MM04T	AHCYL1	adenosylhomocysteinase-like 1	CCDS818.1	chr1_110561030-110561030_C_T	387R>C	Substitution	Nonsynonymous coding	35%
MM04T	AHI1	Abelson helper integration site 1	CCDS47483.1	chr6_135787208-135787208_C_T	165V>I	Substitution	Nonsynonymous coding	24%
MM04T	AHI1	Abelson helper integration site 1	CCDS47483.1	chr6_135788719-135788719_A_C	63D>E	Substitution	Nonsynonymous coding	24%
MM04T	AHI1	Abelson helper integration site 1	CCDS47483.1	chr6_135787142-135787142_C_A	187E>X	Substitution	Nonsense	19%
MM04T	AHNAK	AHNAK nucleoprotein	CCDS31584.1	chr11_62294055-62294055_C_T	2612E>K	Substitution	Nonsynonymous coding	20%
MM04T	AHNAK	AHNAK nucleoprotein	CCDS31584.1	chr11_62294871-62294871_C_T	2340E>K	Substitution	Nonsynonymous coding	28%
MM04T	AHNAK	AHNAK nucleoprotein	CCDS31584.1	chr11_62299764-62299764_T_C	709T>A	Substitution	Nonsynonymous coding	22%
MM04T	AHNAK2	AHNAK nucleoprotein 2	CCDS45177.1	chr14_105407866-105407866_G_A	4641S>L	Substitution	Nonsynonymous coding	38%
MM04T	AHNAK2	AHNAK nucleoprotein 2	CCDS45177.1	chr14_105420551-105420551_C_T	413G>R	Substitution	Nonsynonymous coding	32%
MM04T	AIF1	allograft inflammatory factor 1	CCDS4706.1	chr6_31584265-31584265_G_T	114R>I	Substitution	Nonsynonymous coding	23%
MM04T	AK7	adenylate kinase 7	CCDS9945.1	chr14_96875248-96875248_G_T	156M>I	Substitution	Nonsynonymous coding	28%
MM04T	AKAP10	A kinase (PRKA) anchor protein 10	CCDS11214.1	chr17_19835222-19835222_G_A	513R>X	Substitution	Nonsense	23%
MM04T	AKAP11	A kinase (PRKA) anchor protein 11	CCDS9383.1	chr13_42875829-42875829_G_T	983E>X	Substitution	Nonsense	21%
MM04T	AKAP12	A kinase (PRKA) anchor protein 12	CCDS5229.1	chr6_151670744-151670744_G_T	406E>D	Substitution	Nonsynonymous coding	33%
MM04T	AKAP4	A kinase (PRKA) anchor protein 4	CCDS14329.1	chrX_49958792-49958792_G_T	191P>H	Substitution	Nonsynonymous coding	19%
MM04T	AKAP6	A kinase (PRKA) anchor protein 6	CCDS9644.1	chr14_33290957-33290957_G_A	1313G>D	Substitution	Nonsynonymous coding	35%

MM04T	AKAP9	A kinase (PRKA) anchor protein (yotiao) 9	CCDS5622.1	chr7_91631177-91631177_G_A	649R>Q	Substitution	Nonsynonymous coding	26%
MM04T	AKAP9	A kinase (PRKA) anchor protein (yotiao) 9	CCDS5622.1	chr7_91632512-91632512_A_C	1094K>T	Substitution	Nonsynonymous coding	31%
MM04T	AKAP9	A kinase (PRKA) anchor protein (yotiao) 9	CCDS5622.1	chr7_91712715-91712715_C_A	2798L>I	Substitution	Nonsynonymous coding	38%
MM04T	AKAP9	A kinase (PRKA) anchor protein (yotiao) 9	CCDS5622.1	chr7_91631377-91631377_G_T	716E>X	Substitution	Nonsense	37%
MM04T	AKD1	adenylate kinase domain containing 1	CCDS5077.1	chr6_109965891-109965891_C_A	254E>X	Substitution	Nonsense	21%
MM04T	AKNAD1	AKNA domain containing 1	CCDS791.2	chr1_109392165-109392165_G_A	343L>F	Substitution	Nonsynonymous coding	24%
MM04T	AKR1B15	aldo-keto reductase family 1, member B15	CCDS47715.1	chr7_134253038-134253038_G_T	65E>D	Substitution	Nonsynonymous coding	23%
MM04T	AKR1B15	aldo-keto reductase family 1, member B15	CCDS47715.1	chr7_134261167-134261167_A_C	242K>N	Substitution	Nonsynonymous coding	20%
MM04T	AL450307.1	Uncharacterized protein; cDNA FLJ46300 fis, clone TESTI4035989	ENST00000341866	chr10_133608082-133608082_C_A	98E>D	Substitution	Nonsynonymous coding	36%
MM04T	ALB	albumin	CCDS3555.1	chr4_74275129-74275129_C_A	180F>L	Substitution	Nonsynonymous coding	35%
MM04T	ALCAM	activated leukocyte cell adhesion molecule	CCDS33810.1	chr3_105266008-105266008_C_T	374R>X	Substitution	Nonsense	12%
MM04T	ALDH18A1	aldehyde dehydrogenase 18 family, member A1	CCDS7443.1	chr10_97366673-97366673_C_A	745R>I	Substitution	Nonsynonymous coding	46%
MM04T	ALDH1L1	aldehyde dehydrogenase 1 family, member L1	CCDS3034.1	chr3_125877329-125877329_A_C	94I>R	Substitution	Nonsynonymous coding	10%
MM04T	ALDH2	aldehyde dehydrogenase 2 family (mitochondrial)	CCDS9155.1	chr12_112229223-112229223_G_T	265E>D	Substitution	Nonsynonymous coding	22%
MM04T	ALDH3B1	aldehyde dehydrogenase 3 family, member B1	ENST00000007633	chr11_67795258-67795258_G_A	418D>N	Substitution	Nonsynonymous coding	28%
MM04T	ALG11	asparagine-linked glycosylation 11, alpha-1,2-mannosyltransferase homolog (yeast)	CCDS31977.1	chr13_52598341-52598341_C_A	159L>I	Substitution	Nonsynonymous coding	23%
MM04T	ALG13	asparagine-linked glycosylation 13 homolog (S. cerevisiae)	CCDS14559.1	chrX_110925504-110925504_C_A	76L>I	Substitution	Nonsynonymous coding	15%
MM04T	ALG2	asparagine-linked glycosylation 2, alpha-1,3-mannosyltransferase homolog (S. cerevisiae)	CCDS6739.1	chr9_101980242-101980242_G_A	409R>X	Substitution	Nonsense	18%
MM04T	ALG8	asparagine-linked glycosylation 8, alpha-1,3-glucosyltransferase homolog (S. cerevisiae)	CCDS8258.1	chr11_77838456-77838456_C_T	41R>Q	Substitution	Nonsynonymous coding	18%
MM04T	ALK	anaplastic lymphoma receptor tyrosine kinase	CCDS33172.1	chr2_29462691-29462691_G_A	737S>L	Substitution	Nonsynonymous coding	26%
MM04T	ALK	anaplastic lymphoma receptor tyrosine kinase	CCDS33172.1	chr2_29519902-29519902_G_A	557R>C	Substitution	Nonsynonymous coding	21%
MM04T	ALK	anaplastic lymphoma receptor tyrosine kinase	CCDS33172.1	chr2_29606679-29606679_G_A	401R>X	Substitution	Nonsense	26%
MM04T	ALMS1	Alstrom syndrome 1	CCDS42697.1	chr2_73676961-73676961_C_T	1102P>S	Substitution	Nonsynonymous coding	33%
MM04T	ALMS1	Alstrom syndrome 1	CCDS42697.1	chr2_73677211-73677211_G_T	1185R>I	Substitution	Nonsynonymous coding	24%
MM04T	ALMS1	Alstrom syndrome 1	CCDS42697.1	chr2_73827843-73827843_C_T	3902R>X	Substitution	Nonsense	21%
MM04T	ALOX12B	arachidonate 12-lipoxygenase, 12R type	CCDS11129.1	chr17_7983190-7983190_C_T	275G>D	Substitution	Nonsynonymous coding	35%
MM04T	ALOX12B	arachidonate 12-lipoxygenase, 12R type	CCDS11129.1	chr17_7983596-7983596_C_A	237K>N	Substitution	Nonsynonymous coding	18%
MM04T	ALOX15	arachidonate 15-lipoxygenase	CCDS11049.1	chr17_4536765-4536765_C_A	398E>X	Substitution	Nonsense	22%
MM04T	ALOX15B	arachidonate 15-lipoxygenase, type B	CCDS11128.1	chr17_7948570-7948570_C_A	288F>L	Substitution	Nonsynonymous coding	27%
MM04T	ALOXE3	arachidonate lipoxygenase 3	CCDS11130.1	chr17_8020099-8020099_C_A	116G>V	Substitution	Nonsynonymous coding	31%
MM04T	ALPI	alkaline phosphatase, intestinal	CCDS2492.1	chr2_233321109-233321109_G_A	40D>N	Substitution	Nonsynonymous coding	24%
MM04T	ALPK1	alpha-kinase 1	CCDS3697.1	chr4_113352627-113352627_C_T	642H>Y	Substitution	Nonsynonymous coding	33%

MM04T	ALPK1	alpha-kinase 1	CCDS3697.1	chr4_113353573-113353573_C_A	957S>Y	Substitution	Nonsynonymous coding	25%
MM04T	ALPK1	alpha-kinase 1	CCDS3697.1	chr4_113353640-113353640_C_A	979D>E	Substitution	Nonsynonymous coding	29%
MM04T	ALPK1	alpha-kinase 1	CCDS3697.1	chr4_113353005-113353005_G_T	768E>X	Substitution	Nonsense	21%
MM04T	ALPK2	alpha-kinase 2	CCDS11966.2	chr18_56196378-56196378_C_A	1816D>Y	Substitution	Nonsynonymous coding	20%
MM04T	ALPK2	alpha-kinase 2	CCDS11966.2	chr18_56203470-56203470_C_T	1317E>K	Substitution	Nonsynonymous coding	30%
MM04T	ALPK2	alpha-kinase 2	CCDS11966.2	chr18_56247745-56247745_G_A	88S>L	Substitution	Nonsynonymous coding	11%
MM04T	ALPK2	alpha-kinase 2	CCDS11966.2	chr18_56274592-56274592_G_T	63F>L	Substitution	Nonsynonymous coding	34%
MM04T	ALPK2	alpha-kinase 2	CCDS11966.2	chr18_56274605-56274605_G_A	59S>F	Substitution	Nonsynonymous coding	32%
MM04T	ALS2CL	ALS2 C-terminal like	CCDS2743.1	chr3_46713452-46713452_G_A	869S>L	Substitution	Nonsynonymous coding	22%
MM04T	ALS2CL	ALS2 C-terminal like	CCDS2743.1	chr3_46716212-46716212_C_T	758G>E	Substitution	Nonsynonymous coding	25%
MM04T	ALS2CR11	amyotrophic lateral sclerosis 2 (juvenile) chromosome region, candidate 11	NM_001168221	chr2_202357247-202357247_A_G	1273S>P	Substitution	Nonsynonymous coding	34%
MM04T	ALS2CR11	amyotrophic lateral sclerosis 2 (juvenile) chromosome region, candidate 11	NM_001168221	chr2_202358557-202358557_T_G	836K>T	Substitution	Nonsynonymous coding	36%
MM04T	ALS2CR12	amyotrophic lateral sclerosis 2 (juvenile) chromosome region, candidate 12	CCDS2346.1	chr2_202153498-202153498_C_A	406K>N	Substitution	Nonsynonymous coding	23%
MM04T	ALS2CR8	amyotrophic lateral sclerosis 2 (juvenile) chromosome region, candidate 8	CCDS42801.1	chr2_203807573-203807573_A_G	63I>M	Substitution	Nonsynonymous coding	33%
MM04T	ALS2CR8	amyotrophic lateral sclerosis 2 (juvenile) chromosome region, candidate 8	CCDS42801.1	chr2_203836336-203836336_G_T	402E>D	Substitution	Nonsynonymous coding	27%
MM04T	ALS2CR8	amyotrophic lateral sclerosis 2 (juvenile) chromosome region, candidate 8	CCDS42801.1	chr2_203847017-203847017_C_T	638P>S	Substitution	Nonsynonymous coding	31%
MM04T	AMBP	alpha-1-microglobulin/bikunin precursor	CCDS6800.1	chr9_116837291-116837291_C_A	96G>X	Substitution	Nonsense	27%
MM04T	AMER1	APC membrane recruitment protein 1	CCDS14377.2	chrX_63410967-63410967_C_A	734E>X	Substitution	Nonsense	31%
MM04T	AMFR	autocrine motility factor receptor, E3 ubiquitin protein ligase	CCDS10758.1	chr16_56448253-56448253_G_T	87L>I	Substitution	Nonsynonymous coding	28%
MM04T	AMOT	angiomotin	CCDS48154.1	chrX_112048234-112048234_C_T	573E>K	Substitution	Nonsynonymous coding	21%
MM04T	AMPD1	adenosine monophosphate deaminase 1	CCDS876.1	chr1_115218215-115218215_G_A	539P>S	Substitution	Nonsynonymous coding	32%
MM04T	AMPD1	adenosine monophosphate deaminase 1	CCDS876.1	chr1_115218573-115218573_C_A	480E>D	Substitution	Nonsynonymous coding	19%
MM04T	AMPD1	adenosine monophosphate deaminase 1	CCDS876.1	chr1_115223060-115223060_C_T	196R>Q	Substitution	Nonsynonymous coding	24%
MM04T	AMPH	amphiphysin	CCDS5456.1	chr7_38433781-38433781_C_A	478G>X	Substitution	Nonsense	17%
MM04T	AMPH	amphiphysin	CCDS5456.1	chr7_38500909-38500909_C_A	331E>X	Substitution	Nonsense	44%
MM04T	ANAPC4	anaphase promoting complex subunit 4	CCDS3434.1	chr4_25391786-25391786_T_G	182S>A	Substitution	Nonsynonymous coding	28%
MM04T	ANAPC4	anaphase promoting complex subunit 4	CCDS3434.1	chr4_25398299-25398299_T_G	359L>V	Substitution	Nonsynonymous coding	31%
MM04T	ANAPC4	anaphase promoting complex subunit 4	CCDS3434.1	chr4_25417118-25417118_A_C	619K>N	Substitution	Nonsynonymous coding	43%
MM04T	ANGEL1	angel homolog 1 (Drosophila)	CCDS9852.1	chr14_77257038-77257038_C_T	590V>I	Substitution	Nonsynonymous coding	37%
MM04T	ANGPT1	angiopoietin 1	CCDS6306.1	chr8_108348421-108348421_G_T	178L>I	Substitution	Nonsynonymous coding	33%
MM04T	ANGPT2	angiopoietin 2	CCDS5958.1	chr8_6420442-6420442_A_G	5V>A	Substitution	Nonsynonymous coding	24%
MM04T	ANGPT2	angiopoietin 2	CCDS5958.1	chr8_6389975-6389975_C_A	108E>X	Substitution	Nonsense	28%

MM04T	ANGPTL3	angiopoietin-like 3	CCDS622.1	chr1_63063272-63063272_C_A	12P>H	Substitution	Nonsynonymous coding	39%
MM04T	ANGPTL5	angiopoietin-like 5	CCDS8312.1	chr11_101762130-101762130_G_T	349F>L	Substitution	Nonsynonymous coding	25%
MM04T	ANK1	ankyrin 1, erythrocytic	CCDS47849.1	chr8_41580710-41580710_C_T	314R>Q	Substitution	Nonsynonymous coding	32%
MM04T	ANK2	ankyrin 2, neuronal	CCDS3702.1	chr4_114179506-114179506_G_A	442G>D	Substitution	Nonsynonymous coding	40%
MM04T	ANK3	ankyrin 3, node of Ranvier (ankyrin G)	CCDS7258.1	chr10_61831496-61831496_G_T	3048S>Y	Substitution	Nonsynonymous coding	10%
MM04T	ANK3	ankyrin 3, node of Ranvier (ankyrin G)	CCDS7258.1	chr10_61832853-61832853_G_A	2596R>C	Substitution	Nonsynonymous coding	31%
MM04T	ANK3	ankyrin 3, node of Ranvier (ankyrin G)	CCDS7258.1	chr10_61834592-61834592_A_G	2016V>A	Substitution	Nonsynonymous coding	29%
MM04T	ANKEF1	ankyrin repeat and EF-hand domain containing 1	CCDS13108.1	chr20_10019079-10019079_G_A	44E>K	Substitution	Nonsynonymous coding	32%
MM04T	ANKHD1	ankyrin repeat and KH domain containing 1	CCDS4224.1	chr5_139820697-139820697_G_T	295D>Y	Substitution	Nonsynonymous coding	22%
MM04T	ANKHD1	ankyrin repeat and KH domain containing 1	CCDS4224.1	chr5_139928586-139928586_G_A	2592G>D	Substitution	Nonsynonymous coding	40%
MM04T	ANKIB1	ankyrin repeat and IBR domain containing 1	CCDS47639.1	chr7_91924386-91924386_G_T	32E>X	Substitution	Nonsense	28%
MM04T	ANKK1	ankyrin repeat and kinase domain containing 1	CCDS44734.1	chr11_113265784-113265784_C_A	205P>H	Substitution	Nonsynonymous coding	16%
MM04T	ANKRA2	ankyrin repeat, family A (RFXANK-like), 2	CCDS4020.1	chr5_72849278-72849278_G_T	280S>Y	Substitution	Nonsynonymous coding	21%
MM04T	ANKRD10	ankyrin repeat domain 10	CCDS9520.1	chr13_111545432-111545432_G_A	212R>X	Substitution	Nonsense	35%
MM04T	ANKRD12	ankyrin repeat domain 12	CCDS11843.1	chr18_9255009-9255009_C_T	582R>W	Substitution	Nonsynonymous coding	21%
MM04T	ANKRD13A	ankyrin repeat domain 13A	CCDS9140.1	chr12_110471652-110471652_G_A	467E>K	Substitution	Nonsynonymous coding	21%
MM04T	ANKRD17	ankyrin repeat domain 17	CCDS34004.1	chr4_73957361-73957361_C_T	1995R>Q	Substitution	Nonsynonymous coding	34%
MM04T	ANKRD26	ankyrin repeat domain 26	CCDS41499.1	chr10_27324195-27324195_G_T	1062L>I	Substitution	Nonsynonymous coding	36%
MM04T	ANKRD28	ankyrin repeat domain 28	CCDS46769.1	chr3_15755107-15755107_A_C	343L>V	Substitution	Nonsynonymous coding	34%
MM04T	ANKRD30B	ankyrin repeat domain 30B	NM_001145029	chr18_14748527-14748527_G_A	37G>R	Substitution	Nonsynonymous coding	19%
MM04T	ANKRD32	ankyrin repeat domain 32	CCDS4071.2	chr5_94031013-94031013_C_A	1058S>X	Substitution	Nonsense	29%
MM04T	ANKRD33	ankyrin repeat domain 33	CCDS8815.1	chr12_52284460-52284460_C_T	244R>W	Substitution	Nonsynonymous coding	34%
MM04T	ANKRD33B	ankyrin repeat domain 33B	CCDS47191.1	chr5_10638200-10638200_G_T	186R>M	Substitution	Nonsynonymous coding	20%
MM04T	ANKRD34B	ankyrin repeat domain 34B	CCDS34194.1	chr5_79855217-79855217_G_C	208L>V	Substitution	Nonsynonymous coding	31%
MM04T	ANKRD42	ankyrin repeat domain 42	ENST00000260047	chr11_82956623-82956623_G_T	691E>X	Substitution	Nonsense	16%
MM04T	ANKRD44	ankyrin repeat domain 44	CCDS33355.1	chr2_197863678-197863678_T_G	848K>T	Substitution	Nonsynonymous coding	29%
MM04T	ANKRD50	ankyrin repeat domain 50	CCDS34060.1	chr4_125591857-125591857_G_A	859H>Y	Substitution	Nonsynonymous coding	32%
MM04T	ANKS1B	ankyrin repeat and sterile alpha motif domain containing 1B	NM_152788	chr12_99145258-99145258_C_T	1183E>K	Substitution	Nonsynonymous coding	27%
MM04T	ANKS1B	ankyrin repeat and sterile alpha motif domain containing 1B	NM_152788	chr12_99194869-99194869_C_T	1034R>Q	Substitution	Nonsynonymous coding	25%
MM04T	ANO1	anoctamin 1, calcium activated chloride channel	CCDS44663.1	chr11_69970510-69970510_G_A	318D>N	Substitution	Nonsynonymous coding	17%
MM04T	ANO1	anoctamin 1, calcium activated chloride channel	ENST00000316296	chr11_70009453-70009453_G_A	595E>K	Substitution	Nonsynonymous coding	34%
MM04T	ANO2	anoctamin 2	CCDS44807.1	chr12_5744479-5744479_A_G	552I>T	Substitution	Nonsynonymous coding	32%

MM04T	ANO5	anoctamin 5	CCDS31444.1	chr11_22279274-22279274_C_A	461L>I	Substitution	Nonsynonymous coding	40%
MM04T	ANO5	anoctamin 5	CCDS31444.1	chr11_22294375-22294375_T_C	692L>S	Substitution	Nonsynonymous coding	17%
MM04T	ANO5	anoctamin 5	CCDS31444.1	chr11_22301171-22301171_G_T	868E>X	Substitution	Nonsense	26%
MM04T	ANO6	anoctamin 6	CCDS44865.1	chr12_45797291-45797291_T_G	618W>G	Substitution	Nonsynonymous coding	30%
MM04T	ANP32A	acidic (leucine-rich) nuclear phosphoprotein 32 family, member A	CCDS45292.1	chr15_69076826-69076826_C_T	146D>N	Substitution	Nonsynonymous coding	28%
MM04T	ANPEP	alanyl (membrane) aminopeptidase	CCDS10356.1	chr15_90348636-90348636_C_T	225D>N	Substitution	Nonsynonymous coding	38%
MM04T	ANXA4	annexin A4	CCDS1894.1	chr2_70015198-70015198_G_T	8G>C	Substitution	Nonsynonymous coding	12%
MM04T	ANXA5	annexin A5	CCDS3720.1	chr4_122605887-122605887_C_T	45R>Q	Substitution	Nonsynonymous coding	26%
MM04T	AOC2	amine oxidase, copper containing 2 (retina-specific)	CCDS11443.1	chr17_40997293-40997293_G_A	217R>Q	Substitution	Nonsynonymous coding	33%
MM04T	AP1AR	adaptor-related protein complex 1 associated regulatory protein	CCDS3696.1	chr4_113189329-113189329_C_T	225R>C	Substitution	Nonsynonymous coding	18%
MM04T	AP1B1	adaptor-related protein complex 1, beta 1 subunit	CCDS13855.1	chr22_29747781-29747781_G_T	324F>L	Substitution	Nonsynonymous coding	25%
MM04T	AP1G2	adaptor-related protein complex 1, gamma 2 subunit	CCDS9602.1	chr14_24033256-24033256_G_A	364R>W	Substitution	Nonsynonymous coding	28%
MM04T	AP2A2	adaptor-related protein complex 2, alpha 2 subunit	CCDS44512.1	chr11_970203-970203_G_T	57K>N	Substitution	Nonsynonymous coding	17%
MM04T	AP2B1	adaptor-related protein complex 2, beta 1 subunit	CCDS32621.1	chr17_33966715-33966715_G_T	458R>I	Substitution	Nonsynonymous coding	26%
MM04T	AP3B2	adaptor-related protein complex 3, beta 2 subunit	CCDS45331.1	chr15_83345245-83345245_C_A	545A>S	Substitution	Nonsynonymous coding	28%
MM04T	AP3B2	adaptor-related protein complex 3, beta 2 subunit	CCDS45331.1	chr15_83350313-83350313_C_T	127R>H	Substitution	Nonsynonymous coding	27%
MM04T	AP3M1	adaptor-related protein complex 3, mu 1 subunit	CCDS7342.1	chr10_75896398-75896398_G_T	146S>Y	Substitution	Nonsynonymous coding	32%
MM04T	AP3M2	adaptor-related protein complex 3, mu 2 subunit	CCDS6125.1	chr8_42012324-42012324_C_T	40T>I	Substitution	Nonsynonymous coding	30%
MM04T	AP5B1	adaptor-related protein complex 5, beta 1 subunit	NM_138368	chr11_65546585-65546585_G_A	403A>V	Substitution	Nonsynonymous coding	35%
MM04T	APAF1	apoptotic peptidase activating factor 1	CCDS9069.1	chr12_99059537-99059537_A_C	388I>L	Substitution	Nonsynonymous coding	23%
MM04T	APAF1	apoptotic peptidase activating factor 1	CCDS9069.1	chr12_99117046-99117046_A_G	1054K>E	Substitution	Nonsynonymous coding	37%
MM04T	APBB1	amyloid beta (A4) precursor protein-binding, family B, member 1 (Fe65)	CCDS31410.1	chr11_6422650-6422650_C_T	503E>K	Substitution	Nonsynonymous coding	29%
MM04T	APBB1IP	amyloid beta (A4) precursor protein-binding, family B, member 1 interacting protein	CCDS31167.1	chr10_26785232-26785232_G_T	NA	Substitution	Splice site acceptor	10%
MM04T	APBB2	amyloid beta (A4) precursor protein-binding, family B, member 2	CCDS43224.1	chr4_40832589-40832589_A_C	490F>C	Substitution	Nonsynonymous coding	24%
MM04T	APC2	adenomatosis polyposis coli 2	CCDS12068.1	chr19_1466999-1466999_G_T	1233W>C	Substitution	Nonsynonymous coding	24%
MM04T	APCDD1	adenomatosis polyposis coli down-regulated 1	CCDS11849.1	chr18_10487664-10487664_G_A	392A>T	Substitution	Nonsynonymous coding	33%
MM04T	APCDD1	adenomatosis polyposis coli down-regulated 1	CCDS11849.1	chr18_10487905-10487905_G_T	472R>M	Substitution	Nonsynonymous coding	24%
MM04T	APEX2	APEX nuclease (apurinic/apyrimidinic endonuclease) 2	CCDS14365.1	chrX_55032976-55032976_G_A	222R>H	Substitution	Nonsynonymous coding	24%
MM04T	APEX2	APEX nuclease (apurinic/apyrimidinic endonuclease) 2	CCDS14365.1	chrX_55033375-55033375_C_T	355S>L	Substitution	Nonsynonymous coding	26%
MM04T	APEX2	APEX nuclease (apurinic/apyrimidinic endonuclease) 2	CCDS14365.1	chrX_55033844-55033844_C_A	511F>L	Substitution	Nonsynonymous coding	27%
MM04T	APH1B	anterior pharynx defective 1 homolog B (C. elegans)	CCDS10184.1	chr15_63594611-63594611_G_T	182E>D	Substitution	Nonsynonymous coding	20%
MM04T	APIP	APAF1 interacting protein	ENST00000395786	chr11_34912155-34912155_G_T	8L>I	Substitution	Nonsynonymous coding	23%

MM04T	APLP1	amyloid beta (A4) precursor-like protein 1	CCDS32997.1	chr19_36362201-36362201_C_T	163R>C	Substitution	Nonsynonymous coding	19%
MM04T	APLP1	amyloid beta (A4) precursor-like protein 1	CCDS32997.1	chr19_36365671-36365671_G_A	415R>H	Substitution	Nonsynonymous coding	31%
MM04T	APOA1BP	apolipoprotein A-I binding protein	CCDS1145.1	chr1_156563694-156563694_A_G	229N>D	Substitution	Nonsynonymous coding	30%
MM04T	APOA4	apolipoprotein A-IV	CCDS31681.1	chr11_116692026-116692026_C_T	250E>K	Substitution	Nonsynonymous coding	30%
MM04T	APOB	apolipoprotein B (including Ag(x) antigen)	CCDS1703.1	chr2_21231026-21231026_T_C	2905D>G	Substitution	Nonsynonymous coding	25%
MM04T	APOB	apolipoprotein B (including Ag(x) antigen)	CCDS1703.1	chr2_21256244-21256244_G_T	351L>M	Substitution	Nonsynonymous coding	27%
MM04T	APOBEC2	apolipoprotein B mRNA editing enzyme, catalytic polypeptide-like 2	CCDS4848.1	chr6_41029488-41029488_G_A	185E>K	Substitution	Nonsynonymous coding	28%
MM04T	APOH	apolipoprotein H (beta-2-glycoprotein I)	CCDS11663.1	chr17_64210692-64210692_C_A	287K>N	Substitution	Nonsynonymous coding	23%
MM04T	APOL4	apolipoprotein L, 4	ENST00000352371	chr22_36587785-36587785_C_A	131E>X	Substitution	Nonsense	24%
MM04T	APOL5	apolipoprotein L, 5	CCDS13920.1	chr22_36122770-36122770_A_C	219N>H	Substitution	Nonsynonymous coding	33%
MM04T	AQP12A	aquaporin 12A	CCDS2542.1	chr2_241631427-241631427_G_A	33A>T	Substitution	Nonsynonymous coding	29%
MM04T	ARAP2	ArfGAP with RhoGAP domain, ankyrin repeat and PH domain 2	CCDS3441.1	chr4_36069774-36069774_G_A	1624R>X	Substitution	Nonsense	29%
MM04T	ARFGEF1	ADP-ribosylation factor guanine nucleotide-exchange factor 1 (brefeldin A-inhibited)	CCDS6199.1	chr8_68170411-68170411_A_G	784F>L	Substitution	Nonsynonymous coding	32%
MM04T	ARFIP1	ADP-ribosylation factor interacting protein 1	CCDS34080.1	chr4_153784822-153784822_T_C	53F>S	Substitution	Nonsynonymous coding	38%
MM04T	ARHGAP10	Rho GTPase activating protein 10	CCDS34075.1	chr4_148800431-148800431_G_A	294R>Q	Substitution	Nonsynonymous coding	32%
MM04T	ARHGAP10	Rho GTPase activating protein 10	CCDS34075.1	chr4_148744100-148744100_G_T	102E>X	Substitution	Nonsense	30%
MM04T	ARHGAP12	Rho GTPase activating protein 12	CCDS7170.1	chr10_32106793-32106793_C_A	552E>X	Substitution	Nonsense	31%
MM04T	ARHGAP15	Rho GTPase activating protein 15	CCDS2184.1	chr2_144461056-144461056_C_T	403R>C	Substitution	Nonsynonymous coding	23%
MM04T	ARHGAP15	Rho GTPase activating protein 15	CCDS2184.1	chr2_144525678-144525678_G_T	455Q>H	Substitution	Nonsynonymous coding	28%
MM04T	ARHGAP18	Rho GTPase activating protein 18	CCDS34535.1	chr6_129963019-129963019_C_A	86K>N	Substitution	Nonsynonymous coding	29%
MM04T	ARHGAP21	Rho GTPase activating protein 21	CCDS7144.2	chr10_24880217-24880217_G_A	1363P>S	Substitution	Nonsynonymous coding	26%
MM04T	ARHGAP29	Rho GTPase activating protein 29	CCDS748.1	chr1_94650597-94650597_C_T	647R>Q	Substitution	Nonsynonymous coding	13%
MM04T	ARHGAP29	Rho GTPase activating protein 29	CCDS748.1	chr1_94652141-94652141_C_T	565R>Q	Substitution	Nonsynonymous coding	29%
MM04T	ARHGAP30	Rho GTPase activating protein 30	CCDS30918.1	chr1_161029488-161029488_C_T	39S>N	Substitution	Nonsynonymous coding	17%
MM04T	ARHGAP31	Rho GTPase activating protein 31	CCDS43135.1	chr3_119134857-119134857_C_A	1361P>T	Substitution	Nonsynonymous coding	27%
MM04T	ARHGAP32	Rho GTPase activating protein 32	CCDS44769.1	chr11_128840099-128840099_G_T	1656S>Y	Substitution	Nonsynonymous coding	17%
MM04T	ARHGAP4	Rho GTPase activating protein 4	CCDS14736.1	chrX_153174908-153174908_C_A	832E>D	Substitution	Nonsynonymous coding	16%
MM04T	ARHGAP5	Rho GTPase activating protein 5	CCDS32062.1	chr14_32561314-32561314_G_A	480R>Q	Substitution	Nonsynonymous coding	22%
MM04T	ARHGAP5	Rho GTPase activating protein 5	CCDS32062.1	chr14_32562261-32562261_C_A	796L>I	Substitution	Nonsynonymous coding	23%
MM04T	ARHGAP5	Rho GTPase activating protein 5	CCDS32062.1	chr14_32563196-32563196_G_T	1107E>D	Substitution	Nonsynonymous coding	24%
MM04T	ARHGAP6	Rho GTPase activating protein 6	CCDS14142.1	chrX_11162006-11162006_G_A	757S>L	Substitution	Nonsynonymous coding	33%
MM04T	ARHGAP9	Rho GTPase activating protein 9	CCDS8941.2	chr12_57871406-57871406_G_A	198R>C	Substitution	Nonsynonymous coding	52%

MM04T	ARHGEF10	Rho guanine nucleotide exchange factor (GEF) 10	CCDS34794.1	chr8_1824809-1824809_C_T	251S>L	Substitution	Nonsynonymous coding	30%
MM04T	ARHGEF10	Rho guanine nucleotide exchange factor (GEF) 10	CCDS34794.1	chr8_1828252-1828252_G_T	294E>D	Substitution	Nonsynonymous coding	27%
MM04T	ARHGEF10	Rho guanine nucleotide exchange factor (GEF) 10	CCDS34794.1	chr8_1873494-1873494_A_C	845N>T	Substitution	Nonsynonymous coding	15%
MM04T	ARHGEF12	Rho guanine nucleotide exchange factor (GEF) 12	CCDS41727.1	chr11_120317210-120317210_G_T	482D>Y	Substitution	Nonsynonymous coding	27%
MM04T	ARHGEF12	Rho guanine nucleotide exchange factor (GEF) 12	CCDS41727.1	chr11_120328462-120328462_G_A	741R>Q	Substitution	Nonsynonymous coding	20%
MM04T	ARHGEF12	Rho guanine nucleotide exchange factor (GEF) 12	CCDS41727.1	chr11_120331411-120331411_G_A	853R>Q	Substitution	Nonsynonymous coding	19%
MM04T	ARHGEF15	Rho guanine nucleotide exchange factor (GEF) 15	CCDS11139.1	chr17_8215500-8215500_G_A	48R>Q	Substitution	Nonsynonymous coding	27%
MM04T	ARHGEF15	Rho guanine nucleotide exchange factor (GEF) 15	CCDS11139.1	chr17_8218383-8218383_G_T	NA	Substitution	Splice site acceptor	19%
MM04T	ARHGEF25	Rho guanine nucleotide exchange factor (GEF) 25	CCDS44931.1	chr12_58006838-58006838_G_A	114V>I	Substitution	Nonsynonymous coding	40%
MM04T	ARHGEF28	Rho guanine nucleotide exchange factor (GEF) 28	CCDS47231.1	chr5_73207389-73207389_C_A	1646S>Y	Substitution	Nonsynonymous coding	37%
MM04T	ARHGEF39	Rho guanine nucleotide exchange factor (GEF) 39	CCDS6584.2	chr9_35664112-35664112_C_A	122K>N	Substitution	Nonsynonymous coding	27%
MM04T	ARHGEF4	Rho guanine nucleotide exchange factor (GEF) 4	ENST00000409359	chr2_131673020-131673020_A_G	171N>D	Substitution	Nonsynonymous coding	20%
MM04T	ARHGEF4	Rho guanine nucleotide exchange factor (GEF) 4	CCDS2165.1	chr2_131802064-131802064_C_T	598L>F	Substitution	Nonsynonymous coding	23%
MM04T	ARHGEF4	Rho guanine nucleotide exchange factor (GEF) 4	CCDS2165.1	chr2_131802119-131802119_C_T	NA	Substitution	Splice site donor	48%
MM04T	ARHGEF40	Rho guanine nucleotide exchange factor (GEF) 40	CCDS32041.1	chr14_21543302-21543302_G_T	471E>D	Substitution	Nonsynonymous coding	36%
MM04T	ARHGEF40	Rho guanine nucleotide exchange factor (GEF) 40	CCDS32041.1	chr14_21544937-21544937_C_T	641A>V	Substitution	Nonsynonymous coding	32%
MM04T	ARHGEF9	Cdc42 guanine nucleotide exchange factor (GEF) 9	CCDS35315.1	chrX_62898432-62898432_G_T	194Y>X	Substitution	Nonsense	16%
MM04T	ARID1A	AT rich interactive domain 1A (SWI-like)	CCDS285.1	chr1_27087389-27087389_G_T	655G>X	Substitution	Nonsense	23%
MM04T	ARID1A	AT rich interactive domain 1A (SWI-like)	CCDS285.1	chr1_27089730-27089730_G_T	896E>X	Substitution	Nonsense	23%
MM04T	ARID1A	AT rich interactive domain 1A (SWI-like)	CCDS285.1	chr1_27106354-27106354_C_T	1989R>X	Substitution	Nonsense	37%
MM04T	ARID1B	AT rich interactive domain 1B (SWI1-like)	ENST00000354354	chr6_157507638-157507638_C_T	691P>S	Substitution	Nonsynonymous coding	29%
MM04T	ARID1B	AT rich interactive domain 1B (SWI1-like)	CCDS5251.1	chr6_157100582-157100582_G_T	449G>X	Substitution	Nonsense	18%
MM04T	ARID2	AT rich interactive domain 2 (ARID, RFX-like)	CCDS31783.1	chr12_46215258-46215258_A_C	231R>S	Substitution	Nonsynonymous coding	29%
MM04T	ARID2	AT rich interactive domain 2 (ARID, RFX-like)	CCDS31783.1	chr12_46298742-46298742_T_G	1797L>V	Substitution	Nonsynonymous coding	22%
MM04T	ARID4A	AT rich interactive domain 4A (RBP1-like)	CCDS9732.1	chr14_58831193-58831193_C_T	796R>X	Substitution	Nonsense	27%
MM04T	ARID4B	AT rich interactive domain 4B (RBP1-like)	CCDS31061.1	chr1_235357527-235357527_C_A	NA	Substitution	Splice site acceptor	31%
MM04T	ARID4B	AT rich interactive domain 4B (RBP1-like)	CCDS31061.1	chr1_235397750-235397750_G_A	215R>X	Substitution	Nonsense	42%
MM04T	ARID5A	AT rich interactive domain 5A (MRF1-like)	CCDS33251.1	chr2_97216960-97216960_C_T	232A>V	Substitution	Nonsynonymous coding	29%
MM04T	ARID5B	AT rich interactive domain 5B (MRF1-like)	CCDS31208.1	chr10_63851560-63851560_C_T	780R>X	Substitution	Nonsense	26%
MM04T	ARL2BP	ADP-ribosylation factor-like 2 binding protein	CCDS10776.1	chr16_57282473-57282473_G_T	42R>I	Substitution	Nonsynonymous coding	33%
MM04T	ARL6IP4	ADP-ribosylation-like factor 6 interacting protein 4	CCDS31923.1	chr12_123465757-123465757_G_T	147R>I	Substitution	Nonsynonymous coding	19%
MM04T	ARMC1	armadillo repeat containing 1	CCDS6181.1	chr8_66517500-66517500_C_A	219E>X	Substitution	Nonsense	27%

MM04T	ARMC12	armadillo repeat containing 12	CCDS4809.1	chr6_35704964-35704964_G_A	27A>T	Substitution	Nonsynonymous coding	29%
MM04T	ARMC12	armadillo repeat containing 12	CCDS4809.1	chr6_35715059-35715059_G_A	183E>K	Substitution	Nonsynonymous coding	18%
MM04T	ARMC3	armadillo repeat containing 3	CCDS7142.1	chr10_23319593-23319593_T_G	705F>C	Substitution	Nonsynonymous coding	28%
MM04T	ARMC4	armadillo repeat containing 4	CCDS7157.1	chr10_28151419-28151419_A_C	915L>V	Substitution	Nonsynonymous coding	23%
MM04T	ARMC8	armadillo repeat containing 8	CCDS3098.1	chr3_137942253-137942253_G_T	59E>X	Substitution	Nonsense	43%
MM04T	ARMC8	armadillo repeat containing 8	CCDS3098.1	chr3_137960643-137960643_C_T	272R>X	Substitution	Nonsense	22%
MM04T	ARMCX2	armadillo repeat containing, X-linked 2	CCDS14490.1	chrX_100911769-100911769_G_A	269P>L	Substitution	Nonsynonymous coding	28%
MM04T	ARMCX2	armadillo repeat containing, X-linked 2	CCDS14490.1	chrX_100911974-100911974_C_T	201A>T	Substitution	Nonsynonymous coding	18%
MM04T	ARNTL	aryl hydrocarbon receptor nuclear translocator-like	CCDS31430.1	chr11_13381934-13381934_T_G	141F>C	Substitution	Nonsynonymous coding	29%
MM04T	ARNTL2	aryl hydrocarbon receptor nuclear translocator-like 2	CCDS8712.1	chr12_27523120-27523120_C_A	81L>I	Substitution	Nonsynonymous coding	28%
MM04T	ARPC2	actin related protein 2/3 complex, subunit 2, 34kDa	CCDS2410.1	chr2_219099099-219099099_T_G	83F>V	Substitution	Nonsynonymous coding	25%
MM04T	ARPP21	cAMP-regulated phosphoprotein, 21kDa	CCDS2661.1	chr3_35723323-35723323_G_A	27G>D	Substitution	Nonsynonymous coding	35%
MM04T	ARPP21	cAMP-regulated phosphoprotein, 21kDa	CCDS2661.1	chr3_35750523-35750523_G_T	286E>D	Substitution	Nonsynonymous coding	17%
MM04T	ARPP21	cAMP-regulated phosphoprotein, 21kDa	CCDS2661.1	chr3_35833904-35833904_G_T	688G>V	Substitution	Nonsynonymous coding	34%
MM04T	ARPP21	cAMP-regulated phosphoprotein, 21kDa	CCDS2661.1	chr3_35748563-35748563_G_T	262E>X	Substitution	Nonsense	32%
MM04T	ARR3	arrestin 3, retinal (X-arrestin)	CCDS14399.1	chrX_69496001-69496001_G_A	72R>Q	Substitution	Nonsynonymous coding	33%
MM04T	ARR3	arrestin 3, retinal (X-arrestin)	CCDS14399.1	chrX_69496569-69496569_G_T	152E>D	Substitution	Nonsynonymous coding	12%
MM04T	ARR3	arrestin 3, retinal (X-arrestin)	CCDS14399.1	chrX_69498455-69498455_T_G	290L>R	Substitution	Nonsynonymous coding	18%
MM04T	ARSE	arylsulfatase E (chondrodysplasia punctata 1)	CCDS14122.1	chrX_2876450-2876450_G_A	17A>V	Substitution	Nonsynonymous coding	24%
MM04T	ARSF	arylsulfatase F	CCDS14123.1	chrX_2998941-2998941_C_A	98S>Y	Substitution	Nonsynonymous coding	26%
MM04T	ARSF	arylsulfatase F	CCDS14123.1	chrX_3030310-3030310_G_T	496E>X	Substitution	Nonsense	35%
MM04T	ARSI	arylsulfatase family, member I	CCDS34275.1	chr5_149677576-149677576_G_A	304S>L	Substitution	Nonsynonymous coding	36%
MM04T	AS3MT	arsenic (+3 oxidation state) methyltransferase	CCDS41567.1	chr10_104632939-104632939_T_C	136I>T	Substitution	Nonsynonymous coding	25%
MM04T	ASAH1	N-acylsphingosine amidohydrolase (acid ceramidase) 1	CCDS6005.1	chr8_17927368-17927368_G_T	95S>Y	Substitution	Nonsynonymous coding	40%
MM04T	ASAH1	N-acylsphingosine amidohydrolase (acid ceramidase) 1	CCDS6005.1	chr8_17942308-17942308_C_A	1M>I	Substitution	Nonsynonymous coding	36%
MM04T	ASB1	ankyrin repeat and SOCS box containing 1	CCDS33416.1	chr2_239344416-239344416_G_A	86A>T	Substitution	Nonsynonymous coding	29%
MM04T	ASB12	ankyrin repeat and SOCS box containing 12	CCDS14378.1	chrX_63445085-63445085_T_G	140D>A	Substitution	Nonsynonymous coding	19%
MM04T	ASB15	ankyrin repeat and SOCS box containing 15	CCDS34742.1	chr7_123277005-123277005_T_G	579D>E	Substitution	Nonsynonymous coding	46%
MM04T	ASB7	ankyrin repeat and SOCS box containing 7	CCDS10387.1	chr15_101188569-101188569_C_T	287R>X	Substitution	Nonsense	23%
MM04T	ASCC3	activating signal cointegrator 1 complex subunit 3	CCDS5046.1	chr6_101037638-101037638_G_A	1808R>C	Substitution	Nonsynonymous coding	35%
MM04T	ASCC3	activating signal cointegrator 1 complex subunit 3	CCDS5046.1	chr6_101248198-101248198_G_A	369P>S	Substitution	Nonsynonymous coding	13%
MM04T	ASCC3	activating signal cointegrator 1 complex subunit 3	CCDS5046.1	chr6_101296422-101296422_T_G	135N>H	Substitution	Nonsynonymous coding	40%

MM04T	ASCC3	activating signal cointegrator 1 complex subunit 3	CCDS5046.1	chr6_101312026-101312026_T_G	52K>T	Substitution	Nonsynonymous coding	44%
MM04T	ASCL3	achaete-scute complex homolog 3 (Drosophila)	CCDS7795.1	chr11_8959173-8959173_C_A	179R>I	Substitution	Nonsynonymous coding	32%
MM04T	ASH1L	ash1 (absent, small, or homeotic)-like (Drosophila)	CCDS1113.2	chr1_155450767-155450767_C_A	632E>X	Substitution	Nonsense	22%
MM04T	ASH2L	ash2 (absent, small, or homeotic)-like (Drosophila)	CCDS6101.1	chr8_37991002-37991002_T_G	520F>V	Substitution	Nonsynonymous coding	26%
MM04T	ASIC1	acid-sensing (proton-gated) ion channel 1	CCDS8796.1	chr12_50452838-50452838_G_A	97E>K	Substitution	Nonsynonymous coding	30%
MM04T	ASIC3	acid-sensing (proton-gated) ion channel 3	CCDS5915.1	chr7_150747337-150747337_G_A	227D>N	Substitution	Nonsynonymous coding	18%
MM04T	ASNSD1	asparagine synthetase domain containing 1	CCDS2300.1	chr2_190532013-190532013_C_A	385F>L	Substitution	Nonsynonymous coding	31%
MM04T	ASPA	aspartoacylase	CCDS11028.1	chr17_3402321-3402321_C_T	294A>V	Substitution	Nonsynonymous coding	11%
MM04T	ASPM	asp (abnormal spindle) homolog, microcephaly associated (Drosophila)	CCDS1389.1	chr1_197069692-197069692_G_T	2897L>M	Substitution	Nonsynonymous coding	30%
MM04T	ASPM	asp (abnormal spindle) homolog, microcephaly associated (Drosophila)	CCDS1389.1	chr1_197071585-197071585_C_T	2266A>T	Substitution	Nonsynonymous coding	27%
MM04T	ASPM	asp (abnormal spindle) homolog, microcephaly associated (Drosophila)	CCDS1389.1	chr1_197073715-197073715_A_C	1556L>V	Substitution	Nonsynonymous coding	33%
MM04T	ASPM	asp (abnormal spindle) homolog, microcephaly associated (Drosophila)	CCDS1389.1	chr1_197093390-197093390_C_A	1080K>N	Substitution	Nonsynonymous coding	23%
MM04T	ASPM	asp (abnormal spindle) homolog, microcephaly associated (Drosophila)	CCDS1389.1	chr1_197112753-197112753_T_G	210N>T	Substitution	Nonsynonymous coding	35%
MM04T	ASPN	asporin	CCDS6697.1	chr9_95219620-95219620_G_A	365R>C	Substitution	Nonsynonymous coding	29%
MM04T	ASTE1	asteroid homolog 1 (Drosophila)	CCDS3068.1	chr3_130737545-130737545_G_A	440R>W	Substitution	Nonsynonymous coding	18%
MM04T	ASTL	astacin-like metallo-endopeptidase (M12 family)	CCDS33249.1	chr2_96789620-96789620_C_A	422R>I	Substitution	Nonsynonymous coding	30%
MM04T	ASTN1	astrotactin 1	CCDS1319.1	chr1_176857320-176857320_C_T	987M>I	Substitution	Nonsynonymous coding	41%
MM04T	ASTN1	astrotactin 1	CCDS1319.1	chr1_177000058-177000058_T_C	299N>S	Substitution	Nonsynonymous coding	35%
MM04T	ASTN2	astrotactin 2	CCDS48009.1	chr9_120053734-120053734_C_A	167E>D	Substitution	Nonsynonymous coding	25%
MM04T	ASUN	asunder, spermatogenesis regulator	CCDS8708.1	chr12_27067027-27067027_G_T	485S>Y	Substitution	Nonsynonymous coding	33%
MM04T	ASXL3	additional sex combs like 3 (Drosophila)	CCDS45847.1	chr18_31322851-31322851_G_T	NA	Substitution	Splice site acceptor	29%
MM04T	ASXL3	additional sex combs like 3 (Drosophila)	CCDS45847.1	chr18_31187564-31187564_G_T	21E>X	Substitution	Nonsense	36%
MM04T	ASXL3	additional sex combs like 3 (Drosophila)	CCDS45847.1	chr18_31251752-31251752_G_T	213E>X	Substitution	Nonsense	32%
MM04T	ATAD2	ATPase family, AAA domain containing 2	CCDS6343.1	chr8_124348669-124348669_T_A	1052D>V	Substitution	Nonsynonymous coding	33%
MM04T	ATAD2B	ATPase family, AAA domain containing 2B	CCDS46227.1	chr2_24110750-24110750_C_A	175R>I	Substitution	Nonsynonymous coding	25%
MM04T	ATAD2B	ATPase family, AAA domain containing 2B	CCDS46227.1	chr2_24021197-24021197_A_T	NA	Substitution	Splice site acceptor	24%
MM04T	ATAD5	ATPase family, AAA domain containing 5	CCDS11260.1	chr17_29161650-29161650_C_A	184S>Y	Substitution	Nonsynonymous coding	33%
MM04T	ATCAY	ataxia, cerebellar, Cayman type	CCDS45923.1	chr19_3910851-3910851_T_G	277I>S	Substitution	Nonsynonymous coding	28%
MM04T	ATE1	arginyltransferase 1	CCDS31299.1	chr10_123673380-123673380_T_G	88K>Q	Substitution	Nonsynonymous coding	22%
MM04T	ATF7	activating transcription factor 7	CCDS44905.1	chr12_53911105-53911105_G_T	434S>Y	Substitution	Nonsynonymous coding	23%
MM04T	ATF7IP	activating transcription factor 7 interacting protein	CCDS8663.1	chr12_14650839-14650839_G_T	1215K>N	Substitution	Nonsynonymous coding	38%
MM04T	ATF7IP	activating transcription factor 7 interacting protein	CCDS8663.1	chr12_14649176-14649176_C_T	1098R>X	Substitution	Nonsense	36%

MM04T	ATF7IP2	activating transcription factor 7 interacting protein 2	CCDS10540.1	chr16_10575768-10575768_C_T	571R>X	Substitution	Nonsense	20%
MM04T	ATG2A	autophagy related 2A	CCDS31602.1	chr11_64670835-64670835_A_C	1179D>E	Substitution	Nonsynonymous coding	36%
MM04T	ATG2B	autophagy related 2B	CCDS9944.2	chr14_96752156-96752156_C_A	2058R>I	Substitution	Nonsynonymous coding	21%
MM04T	ATG2B	autophagy related 2B	CCDS9944.2	chr14_96797746-96797746_A_C	566V>G	Substitution	Nonsynonymous coding	26%
MM04T	ATG2B	autophagy related 2B	CCDS9944.2	chr14_96800208-96800208_T_G	342N>H	Substitution	Nonsynonymous coding	18%
MM04T	ATG4C	autophagy related 4C, cysteine peptidase	CCDS623.1	chr1_63307175-63307175_G_A	389R>Q	Substitution	Nonsynonymous coding	27%
MM04T	ATG5	autophagy related 5	CCDS5055.1	chr6_106634522-106634522_G_T	241H>N	Substitution	Nonsynonymous coding	29%
MM04T	ATG7	autophagy related 7	CCDS2605.1	chr3_11468391-11468391_A_C	690Q>H	Substitution	Nonsynonymous coding	28%
MM04T	ATIC	5-aminoimidazole-4-carboxamide ribonucleotide formyltransferase/IMP	CCDS2398.1	chr2_216203539-216203539_G_A	379R>Q	Substitution	Nonsynonymous coding	37%
MM04T	ATM	ataxia telangiectasia mutated	CCDS31669.1	chr11_108165729-108165729_C_T	1618R>X	Substitution	Nonsense	26%
MM04T	ATP10B	ATPase, class V, type 10B	CCDS43394.1	chr5_160018118-160018118_G_T	1198S>Y	Substitution	Nonsynonymous coding	32%
MM04T	ATP10B	ATPase, class V, type 10B	CCDS43394.1	chr5_160113098-160113098_C_T	153R>Q	Substitution	Nonsynonymous coding	21%
MM04T	ATP10B	ATPase, class V, type 10B	CCDS43394.1	chr5_160033805-160033805_G_A	1043R>X	Substitution	Nonsense	22%
MM04T	ATP10D	ATPase, class V, type 10D	CCDS3476.1	chr4_47565654-47565654_C_A	909L>I	Substitution	Nonsynonymous coding	23%
MM04T	ATP11A	ATPase, class VI, type 11A	CCDS32011.1	chr13_113530207-113530207_G_T	1093K>N	Substitution	Nonsynonymous coding	18%
MM04T	ATP11B	ATPase, class VI, type 11B	CCDS33896.1	chr3_182553890-182553890_G_A	128R>Q	Substitution	Nonsynonymous coding	35%
MM04T	ATP11B	ATPase, class VI, type 11B	CCDS33896.1	chr3_182591616-182591616_C_T	689R>X	Substitution	Nonsense	26%
MM04T	ATP11C	ATPase, class VI, type 11C	CCDS14668.1	chrX_138845560-138845560_C_A	806K>N	Substitution	Nonsynonymous coding	42%
MM04T	ATP11C	ATPase, class VI, type 11C	CCDS14668.1	chrX_138884391-138884391_T_G	NA	Substitution	Splice site donor	16%
MM04T	ATP13A1	ATPase type 13A1	CCDS32970.2	chr19_19764795-19764795_C_T	658E>K	Substitution	Nonsynonymous coding	33%
MM04T	ATP13A1	ATPase type 13A1	ENST00000291503	chr19_19772920-19772920_C_A	2E>D	Substitution	Nonsynonymous coding	25%
MM04T	ATP13A3	ATPase type 13A3	CCDS43187.1	chr3_194180667-194180667_G_A	87R>C	Substitution	Nonsynonymous coding	24%
MM04T	ATP13A5	ATPase type 13A5	CCDS33914.1	chr3_193028483-193028483__A	NA	Insertion	Frameshift	31%
MM04T	ATP13A5	ATPase type 13A5	CCDS33914.1	chr3_193081129-193081129_A_C	94Y>D	Substitution	Nonsynonymous coding	19%
MM04T	ATP1A2	ATPase, Na+/K+ transporting, alpha 2 polypeptide	CCDS1196.1	chr1_160109762-160109762_C_T	1008R>W	Substitution	Nonsynonymous coding	29%
MM04T	ATP1A4	ATPase, Na+/K+ transporting, alpha 4 polypeptide	CCDS1197.1	chr1_160121879-160121879_C_A	17P>T	Substitution	Nonsynonymous coding	18%
MM04T	ATP1A4	ATPase, Na+/K+ transporting, alpha 4 polypeptide	CCDS1197.1	chr1_160134108-160134108_T_G	314F>C	Substitution	Nonsynonymous coding	22%
MM04T	ATP1A4	ATPase, Na+/K+ transporting, alpha 4 polypeptide	CCDS1197.1	chr1_160144523-160144523_C_T	766T>M	Substitution	Nonsynonymous coding	18%
MM04T	ATP1B4	ATPase, Na+/K+ transporting, beta 4 polypeptide	CCDS48158.1	chrX_119509422-119509422_G_A	253R>Q	Substitution	Nonsynonymous coding	42%
MM04T	ATP5C1	ATP synthase, H+ transporting, mitochondrial F1 complex, gamma polypeptide 1	CCDS31142.1	chr10_7844253-7844253_G_A	220D>N	Substitution	Nonsynonymous coding	19%
MM04T	ATP6AP1	ATPase, H+ transporting, lysosomal accessory protein 1	CCDS35451.1	chrX_153663808-153663808_G_A	387R>H	Substitution	Nonsynonymous coding	23%
MM04T	ATP6AP1L	ATPase, H+ transporting, lysosomal accessory protein 1-like	CCDS34196.1	chr5_81614027-81614027_C_T	195R>X	Substitution	Nonsense	33%

MM04T	ATP6V0A2	ATPase, H+ transporting, lysosomal V0 subunit a2	CCDS9254.1	chr12_124233134-124233134_G_T	579K>N	Substitution	Nonsynonymous coding	27%
MM04T	ATP6V1C2	ATPase, H+ transporting, lysosomal 42kDa, V1 subunit C2	CCDS42653.1	chr2_10912727-10912727_G_A	210R>Q	Substitution	Nonsynonymous coding	14%
MM04T	ATP6V1G1	ATPase, H+ transporting, lysosomal 13kDa, V1 subunit G1	CCDS6807.1	chr9_117354909-117354909_G_T	54E>X	Substitution	Nonsense	32%
MM04T	ATP7A	ATPase, Cu++ transporting, alpha polypeptide	CCDS35339.1	chrX_77284769-77284769_G_A	980R>Q	Substitution	Nonsynonymous coding	46%
MM04T	ATP7A	ATPase, Cu++ transporting, alpha polypeptide	CCDS35339.1	chrX_77286958-77286958_C_A	1058Q>K	Substitution	Nonsynonymous coding	13%
MM04T	ATP7A	ATPase, Cu++ transporting, alpha polypeptide	CCDS35339.1	chrX_77289250-77289250_G_A	1148V>I	Substitution	Nonsynonymous coding	14%
MM04T	ATP7A	ATPase, Cu++ transporting, alpha polypeptide	CCDS35339.1	chrX_77298215-77298215_G_A	1312A>T	Substitution	Nonsynonymous coding	17%
MM04T	ATP7A	ATPase, Cu++ transporting, alpha polypeptide	CCDS35339.1	chrX_77302047-77302047_G_T	1495D>Y	Substitution	Nonsynonymous coding	18%
MM04T	ATP7A	ATPase, Cu++ transporting, alpha polypeptide	CCDS35339.1	chrX_77301004-77301004_G_A	1387W>X	Substitution	Nonsense	15%
MM04T	ATP7B	ATPase, Cu++ transporting, beta polypeptide	CCDS41892.1	chr13_52549254-52549254_C_A	34K>N	Substitution	Nonsynonymous coding	22%
MM04T	ATP8A2	ATPase, aminophospholipid transporter, class I, type 8A, member 2	CCDS41873.1	chr13_26402284-26402284_C_A	903S>Y	Substitution	Nonsynonymous coding	24%
MM04T	ATP8B3	ATPase, aminophospholipid transporter, class I, type 8B, member 3	CCDS45901.1	chr19_1800385-1800385_C_A	406E>X	Substitution	Nonsense	21%
MM04T	ATP9B	ATPase, class II, type 9B	CCDS12014.1	chr18_77104307-77104307_C_T	809R>X	Substitution	Nonsense	32%
MM04T	ATR	ataxia telangiectasia and Rad3 related	CCDS3124.1	chr3_142188397-142188397_G_A	2112R>C	Substitution	Nonsynonymous coding	26%
MM04T	ATRNL1	attractin-like 1	CCDS7592.1	chr10_117061443-117061443_G_T	903G>V	Substitution	Nonsynonymous coding	33%
MM04T	ATRNL1	attractin-like 1	CCDS7592.1	chr10_117309033-117309033_C_T	1261S>F	Substitution	Nonsynonymous coding	15%
MM04T	ATRX	alpha thalassemia/mental retardation syndrome X-linked	CCDS14434.1	chrX_76937885-76937885_T_G	955K>Q	Substitution	Nonsynonymous coding	24%
MM04T	ATRX	alpha thalassemia/mental retardation syndrome X-linked	CCDS14434.1	chrX_76938028-76938028_C_T	907R>Q	Substitution	Nonsynonymous coding	41%
MM04T	ATRX	alpha thalassemia/mental retardation syndrome X-linked	CCDS14434.1	chrX_76939698-76939698_T_G	350K>N	Substitution	Nonsynonymous coding	16%
MM04T	ATXN10	ataxin 10	CCDS14070.1	chr22_46239017-46239017_G_A	462E>K	Substitution	Nonsynonymous coding	33%
MM04T	ATXN3L	ataxin 3-like	CCDS48080.1	chrX_13337680-13337680_T_G	125K>T	Substitution	Nonsynonymous coding	22%
MM04T	ATXN7L1	ataxin 7-like 1	CCDS34727.1	chr7_105401888-105401888_C_A	126R>I	Substitution	Nonsynonymous coding	30%
MM04T	AUH	AU RNA binding protein/enoyl-CoA hydratase	CCDS6689.1	chr9_93976647-93976647_G_A	335R>C	Substitution	Nonsynonymous coding	37%
MM04T	AUNIP	aurora kinase A and ninein interacting protein	CCDS266.1	chr1_26163936-26163936_C_T	63R>K	Substitution	Nonsynonymous coding	32%
MM04T	AUNIP	aurora kinase A and ninein interacting protein	CCDS266.1	chr1_26162338-26162338_C_A	NA	Substitution	Splice site acceptor	40%
MM04T	AURKC	aurora kinase C	CCDS33128.1	chr19_57743516-57743516_C_A	74L>I	Substitution	Nonsynonymous coding	22%
MM04T	AVEN	apoptosis, caspase activation inhibitor	CCDS10030.1	chr15_34295387-34295387_C_A	97E>D	Substitution	Nonsynonymous coding	44%
MM04T	AVL9	AVL9 homolog (S. cerevisiase)	CCDS34613.1	chr7_32590971-32590971_A_C	133K>T	Substitution	Nonsynonymous coding	39%
MM04T	AVPR1A	arginine vasopressin receptor 1A	CCDS8965.1	chr12_63544486-63544486_T_C	44D>G	Substitution	Nonsynonymous coding	29%
MM04T	AXIN1	axin 1	CCDS10405.1	chr16_348179-348179_C_T	443A>T	Substitution	Nonsynonymous coding	38%
MM04T	AXL	AXL receptor tyrosine kinase	CCDS12575.1	chr19_41762374-41762374_C_T	685S>F	Substitution	Nonsynonymous coding	27%
MM04T	AXL	AXL receptor tyrosine kinase	CCDS12575.1	chr19_41765555-41765555_C_T	811P>S	Substitution	Nonsynonymous coding	21%

MM04T	AZGP1	alpha-2-glycoprotein 1, zinc-binding	ENST00000411734	chr7_99565730-99565730_C_T	218D>N	Substitution	Nonsynonymous coding	32%
MM04T	B3GALTL	beta 1,3-galactosyltransferase-like	CCDS9341.1	chr13_31860916-31860916_A_C	342T>P	Substitution	Nonsynonymous coding	20%
MM04T	B3GAT1	beta-1,3-glucuronyltransferase 1 (glucuronosyltransferase P)	CCDS8500.1	chr11_134252846-134252846_G_A	226R>W	Substitution	Nonsynonymous coding	31%
MM04T	B3GAT1	beta-1,3-glucuronyltransferase 1 (glucuronosyltransferase P)	CCDS8500.1	chr11_134257523-134257523_C_T	11V>I	Substitution	Nonsynonymous coding	27%
MM04T	B3GNT6	UDP-GlcNAc:betaGal beta-1,3-N-acetylglucosaminyltransferase 6 (core 3	NM_138706	chr11_76750791-76750791_C_A	66P>T	Substitution	Nonsynonymous coding	26%
MM04T	B4GALNT2	beta-1,4-N-acetyl-galactosaminyl transferase 2	CCDS11544.1	chr17_47246889-47246889_C_A	500F>L	Substitution	Nonsynonymous coding	38%
MM04T	B4GALNT3	beta-1,4-N-acetyl-galactosaminyl transferase 3	CCDS8504.1	chr12_655854-655854_C_T	228R>W	Substitution	Nonsynonymous coding	36%
MM04T	BAAT	bile acid CoA: amino acid N-acyltransferase (glycine N-choloyltransferase)	CCDS6752.1	chr9_104124751-104124751_G_T	406L>I	Substitution	Nonsynonymous coding	28%
MM04T	BAAT	bile acid CoA: amino acid N-acyltransferase (glycine N-choloyltransferase)	CCDS6752.1	chr9_104133260-104133260_G_A	143R>X	Substitution	Nonsense	22%
MM04T	BABAM1	BRISC and BRCA1 A complex member 1	CCDS46012.1	chr19_17379742-17379742_G_T	43A>S	Substitution	Nonsynonymous coding	14%
MM04T	BACE2	beta-site APP-cleaving enzyme 2	CCDS13668.1	chr21_42540421-42540421_C_A	77F>L	Substitution	Nonsynonymous coding	11%
MM04T	BACH1	BTB and CNC homology 1, basic leucine zipper transcription factor 1	CCDS13585.1	chr21_30701851-30701851_G_A	538R>Q	Substitution	Nonsynonymous coding	26%
MM04T	BAI2	brain-specific angiogenesis inhibitor 2	CCDS346.2	chr1_32201949-32201949_C_T	1058R>Q	Substitution	Nonsynonymous coding	18%
MM04T	BAI3	brain-specific angiogenesis inhibitor 3	CCDS4968.1	chr6_69640490-69640490_G_A	266R>Q	Substitution	Nonsynonymous coding	17%
MM04T	BAI3	brain-specific angiogenesis inhibitor 3	CCDS4968.1	chr6_69758097-69758097_C_A	710P>T	Substitution	Nonsynonymous coding	34%
MM04T	BAI3	brain-specific angiogenesis inhibitor 3	CCDS4968.1	chr6_69772894-69772894_C_T	801S>L	Substitution	Nonsynonymous coding	23%
MM04T	BAI3	brain-specific angiogenesis inhibitor 3	CCDS4968.1	chr6_70071264-70071264_C_A	1367L>I	Substitution	Nonsynonymous coding	38%
MM04T	BAI3	brain-specific angiogenesis inhibitor 3	CCDS4968.1	chr6_70092801-70092801_C_T	1452R>W	Substitution	Nonsynonymous coding	27%
MM04T	BAI3	brain-specific angiogenesis inhibitor 3	CCDS4968.1	chr6_69772825-69772825_G_T	NA	Substitution	Splice site acceptor	24%
MM04T	BAI3	brain-specific angiogenesis inhibitor 3	CCDS4968.1	chr6_69665970-69665970_C_A	417S>X	Substitution	Nonsense	32%
MM04T	BAIAP2L2	BAI1-associated protein 2-like 2	CCDS43018.1	chr22_38494133-38494133_C_A	134E>D	Substitution	Nonsynonymous coding	19%
MM04T	BAP1	BRCA1 associated protein-1 (ubiquitin carboxy-terminal hydrolase)	CCDS2853.1	chr3_52442528-52442528_C_A	73D>Y	Substitution	Nonsynonymous coding	22%
MM04T	BARD1	BRCA1 associated RING domain 1	CCDS2397.1	chr2_215646149-215646149_C_T	150R>Q	Substitution	Nonsynonymous coding	28%
MM04T	BARX1	BARX homeobox 1	CCDS35070.2	chr9_96714456-96714456_C_T	252R>H	Substitution	Nonsynonymous coding	14%
MM04T	BAZ1B	bromodomain adjacent to zinc finger domain, 1B	CCDS5549.1	chr7_72891211-72891211_T_G	860E>D	Substitution	Nonsynonymous coding	35%
MM04T	BAZ2A	bromodomain adjacent to zinc finger domain, 2A	CCDS44924.1	chr12_57003698-57003698_C_A	640E>D	Substitution	Nonsynonymous coding	26%
MM04T	BAZ2B	bromodomain adjacent to zinc finger domain, 2B	CCDS2209.2	chr2_160303346-160303346_C_T	215E>K	Substitution	Nonsynonymous coding	23%
MM04T	BBS12	Bardet-Biedl syndrome 12	CCDS3728.1	chr4_123663639-123663639_G_T	198E>X	Substitution	Nonsense	28%
MM04T	BBS2	Bardet-Biedl syndrome 2	CCDS32451.1	chr16_56536710-56536710_C_T	272R>Q	Substitution	Nonsynonymous coding	38%
MM04T	BBS5	Bardet-Biedl syndrome 5	CCDS2233.1	chr2_170344575-170344575_T_G	113L>V	Substitution	Nonsynonymous coding	20%
MM04T	BBS7	Bardet-Biedl syndrome 7	CCDS3724.1	chr4_122765083-122765083_T_G	435E>A	Substitution	Nonsynonymous coding	29%
MM04T	BBS9	Bardet-Biedl syndrome 9	CCDS43566.1	chr7_33185915-33185915_A_C	17K>N	Substitution	Nonsynonymous coding	31%

MM04T	BCAN	brevican	CCDS1149.1	chr1_156618558-156618558_G_A	323G>D	Substitution	Nonsynonymous coding	24%
MM04T	BCAP29	B-cell receptor-associated protein 29	CCDS34730.1	chr7_107254120-107254120_T_G	238L>V	Substitution	Nonsynonymous coding	13%
MM04T	BCAS1	breast carcinoma amplified sequence 1	CCDS13444.1	chr20_52570129-52570129_C_T	508E>K	Substitution	Nonsynonymous coding	31%
MM04T	BCAS1	breast carcinoma amplified sequence 1	CCDS13444.1	chr20_52645344-52645344_G_A	104P>S	Substitution	Nonsynonymous coding	21%
MM04T	BCAT1	branched chain amino-acid transaminase 1, cytosolic	CCDS44845.1	chr12_24985800-24985800_G_A	NA	Substitution	Splice site acceptor	30%
MM04T	BCL11A	B-cell CLL/lymphoma 11A (zinc finger protein)	CCDS1862.1	chr2_60687798-60687798_A_G	750V>A	Substitution	Nonsynonymous coding	33%
MM04T	BCL9	B-cell CLL/lymphoma 9	CCDS30833.1	chr1_147091643-147091643_G_A	561R>H	Substitution	Nonsynonymous coding	18%
MM04T	BCLAF1	BCL2-associated transcription factor 1	CCDS5177.1	chr6_136597297-136597297_C_A	456E>X	Substitution	Nonsense	19%
MM04T	BCO2	beta-carotene oxygenase 2	CCDS8358.2	chr11_112085657-112085657_A_C	502K>T	Substitution	Nonsynonymous coding	37%
MM04T	BCOR	BCL6 corepressor	CCDS48093.1	chrX_39916551-39916551_C_A	1484E>D	Substitution	Nonsynonymous coding	29%
MM04T	BCS1L	BC1 (ubiquinol-cytochrome c reductase) synthesis-like	CCDS2419.1	chr2_219526158-219526158_G_A	117R>Q	Substitution	Nonsynonymous coding	28%
MM04T	BDKRB1	bradykinin receptor B1	CCDS9943.1	chr14_96730268-96730268_T_G	83D>E	Substitution	Nonsynonymous coding	32%
MM04T	BDP1	B double prime 1, subunit of RNA polymerase III transcription initiation factor IIIB	CCDS43328.1	chr5_70782334-70782334_G_A	365D>N	Substitution	Nonsynonymous coding	25%
MM04T	BDP1	B double prime 1, subunit of RNA polymerase III transcription initiation factor IIIB	CCDS43328.1	chr5_70791147-70791147_G_A	571E>K	Substitution	Nonsynonymous coding	22%
MM04T	BDP1	B double prime 1, subunit of RNA polymerase III transcription initiation factor IIIB	ENST00000437938	chr5_70806989-70806989_T_G	1357F>C	Substitution	Nonsynonymous coding	30%
MM04T	BDP1	B double prime 1, subunit of RNA polymerase III transcription initiation factor IIIB	CCDS43328.1	chr5_70819908-70819908_C_T	1844R>C	Substitution	Nonsynonymous coding	32%
MM04T	BEND2	BEN domain containing 2	CCDS14184.1	chrX_18183152-18183152_C_T	793D>N	Substitution	Nonsynonymous coding	36%
MM04T	BEND2	BEN domain containing 2	CCDS14184.1	chrX_18234847-18234847_A_C	11V>G	Substitution	Nonsynonymous coding	29%
MM04T	BEND2	BEN domain containing 2	CCDS14184.1	chrX_18221732-18221732_C_A	266E>X	Substitution	Nonsense	36%
MM04T	BEST2	bestrophin 2	CCDS42506.1	chr19_12864108-12864108_G_A	63E>K	Substitution	Nonsynonymous coding	32%
MM04T	BEST3	bestrophin 3	CCDS8992.2	chr12_70071028-70071028_G_A	216R>X	Substitution	Nonsense	21%
MM04T	BHLHA9	basic helix-loop-helix family, member a9	CCDS45560.1	chr17_1174051-1174051_C_T	65A>V	Substitution	Nonsynonymous coding	34%
MM04T	BIRC6	baculoviral IAP repeat containing 6	CCDS33175.2	chr2_32750029-32750029_G_T	3900E>X	Substitution	Nonsense	24%
MM04T	BLMH	bleomycin hydrolase	CCDS32604.1	chr17_28599913-28599913_C_G	269C>S	Substitution	Nonsynonymous coding	21%
MM04T	BLOC1S5	biogenesis of lysosomal organelles complex-1, subunit 5, muted	CCDS4505.1	chr6_7884636-7884636_C_T	378E>K	Substitution	Nonsynonymous coding	21%
MM04T	BMP10	bone morphogenetic protein 10	CCDS1890.1	chr2_69093441-69093441_C_A	199E>D	Substitution	Nonsynonymous coding	29%
MM04T	BMP2	bone morphogenetic protein 2	CCDS13099.1	chr20_6759270-6759270_G_T	242R>I	Substitution	Nonsynonymous coding	42%
MM04T	BMP5	bone morphogenetic protein 5	CCDS4958.1	chr6_55625290-55625290_C_T	357E>K	Substitution	Nonsynonymous coding	30%
MM04T	BMPR1B	bone morphogenetic protein receptor, type IB	CCDS3642.1	chr4_96036861-96036861_G_A	91R>K	Substitution	Nonsynonymous coding	28%
MM04T	BMPR2	bone morphogenetic protein receptor, type II (serine/threonine kinase)	CCDS33361.1	chr2_203329668-203329668_G_T	71E>D	Substitution	Nonsynonymous coding	21%
MM04T	BNIP1	BCL2/adenovirus E1B 19kDa interacting protein 1	CCDS4385.1	chr5_172590740-172590740_G_A	211R>Q	Substitution	Nonsynonymous coding	23%
MM04T	BOD1L1	biorientation of chromosomes in cell division 1-like 1	CCDS3411.2	chr4_13603977-13603977_G_T	1516S>Y	Substitution	Nonsynonymous coding	38%

MM04T	BRAP	BRCA1 associated protein	CCDS9154.1	chr12_112082234-112082234_C_A	516K>N	Substitution	Nonsynonymous coding	23%
MM04T	BRCA2	breast cancer 2, early onset	CCDS9344.1	chr13_32912628-32912628_A_G	1379Q>R	Substitution	Nonsynonymous coding	21%
MM04T	BRCA2	breast cancer 2, early onset	CCDS9344.1	chr13_32915065-32915065_A_C	2191K>N	Substitution	Nonsynonymous coding	28%
MM04T	BRCA2	breast cancer 2, early onset	CCDS9344.1	chr13_32915088-32915088_C_A	2199T>N	Substitution	Nonsynonymous coding	24%
MM04T	BRCA2	breast cancer 2, early onset	CCDS9344.1	chr13_32921003-32921003_C_A	2326S>Y	Substitution	Nonsynonymous coding	15%
MM04T	BRCA2	breast cancer 2, early onset	CCDS9344.1	chr13_32937562-32937562_G_T	2741K>N	Substitution	Nonsynonymous coding	21%
MM04T	BRCA2	breast cancer 2, early onset	CCDS9344.1	chr13_32913044-32913044_G_T	1518E>X	Substitution	Nonsense	24%
MM04T	BRCC3	BRCA1/BRCA2-containing complex, subunit 3	NM_024332	chrX_154317543-154317543_C_T	137R>C	Substitution	Nonsynonymous coding	17%
MM04T	BRDT	bromodomain, testis-specific	CCDS735.1	chr1_92442676-92442676_C_A	232S>Y	Substitution	Nonsynonymous coding	18%
MM04T	BRPF3	bromodomain and PHD finger containing, 3	CCDS34437.1	chr6_36179240-36179240_G_T	795K>N	Substitution	Nonsynonymous coding	17%
MM04T	BRSK1	BR serine/threonine kinase 1	CCDS12921.1	chr19_55816913-55816913_C_T	617L>F	Substitution	Nonsynonymous coding	25%
MM04T	BRWD1	bromodomain and WD repeat domain containing 1	CCDS13662.1	chr21_40559250-40559250_T_G	2222K>T	Substitution	Nonsynonymous coding	25%
MM04T	BRWD1	bromodomain and WD repeat domain containing 1	CCDS13662.1	chr21_40590484-40590484_C_A	1162R>I	Substitution	Nonsynonymous coding	26%
MM04T	BRWD3	bromodomain and WD repeat domain containing 3	CCDS14447.1	chrX_79936867-79936867_G_A	1543R>W	Substitution	Nonsynonymous coding	26%
MM04T	BRWD3	bromodomain and WD repeat domain containing 3	CCDS14447.1	chrX_79946573-79946573_C_T	1194R>K	Substitution	Nonsynonymous coding	30%
MM04T	BRWD3	bromodomain and WD repeat domain containing 3	CCDS14447.1	chrX_79971732-79971732_C_T	750R>Q	Substitution	Nonsynonymous coding	28%
MM04T	BSN	bassoon presynaptic cytomatrix protein	CCDS2800.1	chr3_49662705-49662705_G_T	174K>N	Substitution	Nonsynonymous coding	27%
MM04T	BSN	bassoon presynaptic cytomatrix protein	CCDS2800.1	chr3_49689040-49689040_G_T	684G>V	Substitution	Nonsynonymous coding	32%
MM04T	BSN	bassoon presynaptic cytomatrix protein	CCDS2800.1	chr3_49694787-49694787_C_T	2600R>W	Substitution	Nonsynonymous coding	24%
MM04T	BST1	bone marrow stromal cell antigen 1	CCDS3416.1	chr4_15733397-15733397_C_A	296L>I	Substitution	Nonsynonymous coding	33%
MM04T	BTAF1	BTAF1 RNA polymerase II, B-TFIID transcription factor-associated, 170kDa (Mot1 homolog, S.	CCDS7419.1	chr10_93754309-93754309_G_A	1073D>N	Substitution	Nonsynonymous coding	28%
MM04T	BTBD7	BTB (POZ) domain containing 7	CCDS32146.1	chr14_93708896-93708896_A_G	1041L>S	Substitution	Nonsynonymous coding	11%
MM04T	BTBD7	BTB (POZ) domain containing 7	CCDS32146.1	chr14_93723596-93723596_G_T	518P>H	Substitution	Nonsynonymous coding	24%
MM04T	BTN1A1	butyrophilin, subfamily 1, member A1	CCDS4614.1	chr6_26507021-26507021_G_A	274E>K	Substitution	Nonsynonymous coding	30%
MM04T	BTN2A2	butyrophilin, subfamily 2, member A2	CCDS4606.1	chr6_26385525-26385525_T_G	126I>S	Substitution	Nonsynonymous coding	22%
MM04T	BTRC	beta-transducin repeat containing E3 ubiquitin protein ligase	CCDS7512.1	chr10_103296370-103296370_C_T	513R>X	Substitution	Nonsense	25%
MM04T	BUB1B	BUB1 mitotic checkpoint serine/threonine kinase B	CCDS10053.1	chr15_40488912-40488912_G_A	409E>K	Substitution	Nonsynonymous coding	31%
MM04T	BVES	blood vessel epicardial substance	CCDS5051.1	chr6_105563646-105563646_T_G	291E>D	Substitution	Nonsynonymous coding	31%
MM04T	BYSL	bystin-like	CCDS34450.1	chr6_41899198-41899198_C_T	257R>X	Substitution	Nonsense	32%
MM04T	C10orf12	chromosome 10 open reading frame 12	CCDS7452.1	chr10_98742998-98742998_T_G	617I>M	Substitution	Nonsynonymous coding	25%
MM04T	C10orf120	chromosome 10 open reading frame 120	CCDS31302.1	chr10_124458855-124458855_G_T	84L>I	Substitution	Nonsynonymous coding	22%
MM04T	C10orf128	chromosome 10 open reading frame 128	CCDS41519.1	chr10_50369652-50369652_G_T	95F>L	Substitution	Nonsynonymous coding	32%

MM04T	C10orf137	chromosome 10 open reading frame 137	CCDS7646.1	chr10_127431786-127431786_G_T	810R>I	Substitution	Nonsynonymous coding	38%
MM04T	C10orf137	chromosome 10 open reading frame 137	CCDS7646.1	chr10_127424429-127424429_G_T	538E>X	Substitution	Nonsense	36%
MM04T	C10orf68	chromosome 10 open reading frame 68	CCDS31177.1	chr10_32978065-32978065_G_T	80R>I	Substitution	Nonsynonymous coding	20%
MM04T	C10orf71	chromosome 10 open reading frame 71	CCDS44387.1	chr10_50532681-50532681_C_A	697F>L	Substitution	Nonsynonymous coding	32%
MM04T	C10orf90	chromosome 10 open reading frame 90	CCDS31310.1	chr10_128153387-128153387_C_A	471R>I	Substitution	Nonsynonymous coding	21%
MM04T	C10orf90	chromosome 10 open reading frame 90	CCDS31310.1	chr10_128193282-128193282_G_T	163L>M	Substitution	Nonsynonymous coding	15%
MM04T	C10orf90	chromosome 10 open reading frame 90	CCDS31310.1	chr10_128202484-128202484_C_A	16R>I	Substitution	Nonsynonymous coding	31%
MM04T	C11orf16	chromosome 11 open reading frame 16	CCDS7794.1	chr11_8947210-8947210_G_T	335S>Y	Substitution	Nonsynonymous coding	24%
MM04T	C11orf65	chromosome 11 open reading frame 65	CCDS8340.1	chr11_108302505-108302505_G_A	48R>C	Substitution	Nonsynonymous coding	15%
MM04T	C12orf40	chromosome 12 open reading frame 40	CCDS41770.1	chr12_40076567-40076567_C_A	281L>I	Substitution	Nonsynonymous coding	31%
MM04T	C12orf40	chromosome 12 open reading frame 40	CCDS41770.1	chr12_40114750-40114750_G_T	552E>D	Substitution	Nonsynonymous coding	23%
MM04T	C12orf42	chromosome 12 open reading frame 42	CCDS44963.1	chr12_103695994-103695994_C_A	325K>N	Substitution	Nonsynonymous coding	38%
MM04T	C12orf42	chromosome 12 open reading frame 42	CCDS44963.1	chr12_103699756-103699756_A_T	209N>K	Substitution	Nonsynonymous coding	42%
MM04T	C12orf50	chromosome 12 open reading frame 50	CCDS9031.1	chr12_88380143-88380143_T_C	290R>G	Substitution	Nonsynonymous coding	17%
MM04T	C12orf55	chromosome 12 open reading frame 55	CCDS9062.1	chr12_97051678-97051678_G_T	132E>X	Substitution	Nonsense	26%
MM04T	C12orf69	chromosome 12 open reading frame 69	CCDS41759.1	chr12_14959262-14959262_G_A	118S>L	Substitution	Nonsynonymous coding	40%
MM04T	C14orf105	chromosome 14 open reading frame 105	CCDS9730.1	chr14_57938195-57938195_G_T	257L>I	Substitution	Nonsynonymous coding	22%
MM04T	C14orf164	chromosome 14 open reading frame 164	ENST00000399910	chr14_23729104-23729104_T_C	119V>A	Substitution	Nonsynonymous coding	26%
MM04T	C14orf37	chromosome 14 open reading frame 37	CCDS32089.1	chr14_58599950-58599950_C_A	493K>N	Substitution	Nonsynonymous coding	27%
MM04T	C14orf37	chromosome 14 open reading frame 37	CCDS32089.1	chr14_58605917-58605917_C_A	54D>Y	Substitution	Nonsynonymous coding	34%
MM04T	C14orf79	chromosome 14 open reading frame 79	CCDS42000.1	chr14_105457918-105457918_G_T	220E>D	Substitution	Nonsynonymous coding	15%
MM04T	C15orf27	chromosome 15 open reading frame 27	CCDS10289.2	chr15_76430170-76430170_C_T	54A>V	Substitution	Nonsynonymous coding	20%
MM04T	C15orf39	chromosome 15 open reading frame 39	CCDS10276.1	chr15_75499036-75499036_A_C	216K>T	Substitution	Nonsynonymous coding	21%
MM04T	C15orf41	chromosome 15 open reading frame 41	CCDS45215.1	chr15_36872166-36872166_A_C	NA	Substitution	Splice site donor	35%
MM04T	C15orf54	chromosome 15 open reading frame 54	CCDS10049.1	chr15_39544348-39544348_A_T	4K>N	Substitution	Nonsynonymous coding	34%
MM04T	C15orf55	chromosome 15 open reading frame 55	CCDS32190.1	chr15_34649305-34649305_G_T	1004E>D	Substitution	Nonsynonymous coding	27%
MM04T	C15orf57	chromosome 15 open reading frame 57	CCDS42022.1	chr15_40854976-40854976_G_T	89S>Y	Substitution	Nonsynonymous coding	27%
MM04T	C16orf59	chromosome 16 open reading frame 59	CCDS10468.2	chr16_2510670-2510670_A_G	58T>A	Substitution	Nonsynonymous coding	25%
MM04T	C16orf87	chromosome 16 open reading frame 87	CCDS10724.1	chr16_46843581-46843581_C_A	94D>Y	Substitution	Nonsynonymous coding	20%
MM04T	C16orf88	chromosome 16 open reading frame 88	CCDS42127.1	chr16_19725580-19725580_C_A	260D>Y	Substitution	Nonsynonymous coding	28%
MM04T	C16orf96	chromosome 16 open reading frame 96	ENST00000382019	chr16_4624984-4624984_G_T	206Q>H	Substitution	Nonsynonymous coding	29%
MM04T	C17orf112	chromosome 17 open reading frame 112	ENST00000441889	chr17_51064055-51064055_G_T	64E>X	Substitution	Nonsense	32%

MM04T	C17orf58	chromosome 17 open reading frame 58	CCDS42375.1	chr17_65989139-65989139_C_T	42D>N	Substitution	Nonsynonymous coding	43%
MM04T	C17orf64	chromosome 17 open reading frame 64	CCDS32698.2	chr17_58506735-58506735_G_A	148A>T	Substitution	Nonsynonymous coding	27%
MM04T	C17orf85	chromosome 17 open reading frame 85	CCDS45578.1	chr17_3743450-3743450_G_A	100S>L	Substitution	Nonsynonymous coding	19%
MM04T	C18orf54	chromosome 18 open reading frame 54	CCDS11956.1	chr18_51886981-51886981_G_T	13Q>H	Substitution	Nonsynonymous coding	21%
MM04T	C18orf63	chromosome 18 open reading frame 63	ENST00000382675	chr18_72009290-72009290_A_C	212N>T	Substitution	Nonsynonymous coding	35%
MM04T	C18orf63	chromosome 18 open reading frame 63	ENST00000382675	chr18_72020580-72020580_C_A	360L>I	Substitution	Nonsynonymous coding	23%
MM04T	C18orf63	chromosome 18 open reading frame 63	ENST00000382675	chr18_72021708-72021708_A_C	673K>T	Substitution	Nonsynonymous coding	28%
MM04T	C18orf8	chromosome 18 open reading frame 8	CCDS32803.1	chr18_21109612-21109612_A_C	477K>T	Substitution	Nonsynonymous coding	21%
MM04T	C19orf38	chromosome 19 open reading frame 38	CCDS45970.1	chr19_10970598-10970598_G_T	157E>X	Substitution	Nonsense	28%
MM04T	C19orf44	chromosome 19 open reading frame 44	CCDS12345.1	chr19_16611746-16611746_G_T	48S>I	Substitution	Nonsynonymous coding	29%
MM04T	C19orf57	chromosome 19 open reading frame 57	CCDS12299.1	chr19_14000064-14000064_G_T	535F>L	Substitution	Nonsynonymous coding	20%
MM04T	C19orf68	chromosome 19 open reading frame 68	ENST00000328759	chr19_48698452-48698452_C_A	377F>L	Substitution	Nonsynonymous coding	27%
MM04T	C19orf70	chromosome 19 open reading frame 70	CCDS12143.1	chr19_5678609-5678609_C_T	104E>K	Substitution	Nonsynonymous coding	38%
MM04T	C1orf101	chromosome 1 open reading frame 101	CCDS44340.1	chr1_244640855-244640855_T_C	43Y>H	Substitution	Nonsynonymous coding	23%
MM04T	C1orf106	chromosome 1 open reading frame 106	CCDS1406.1	chr1_200868629-200868629_G_T	113K>N	Substitution	Nonsynonymous coding	14%
MM04T	C1orf116	chromosome 1 open reading frame 116	CCDS1475.1	chr1_207196264-207196264_G_A	282S>L	Substitution	Nonsynonymous coding	19%
MM04T	C1orf131	chromosome 1 open reading frame 131	CCDS1591.2	chr1_231374800-231374800_T_C	85K>E	Substitution	Nonsynonymous coding	35%
MM04T	C1orf137	chromosome 1 open reading frame 137	ENST00000369482	chr1_117236768-117236768_C_A	6S>Y	Substitution	Nonsynonymous coding	19%
MM04T	C1orf158	chromosome 1 open reading frame 158	CCDS147.1	chr1_12820816-12820816_G_A	173A>T	Substitution	Nonsynonymous coding	19%
MM04T	C1orf168	chromosome 1 open reading frame 168	CCDS30729.1	chr1_57206385-57206385_G_A	563S>L	Substitution	Nonsynonymous coding	14%
MM04T	C1orf170	chromosome 1 open reading frame 170	ENST00000433179	chr1_915823-915823_G_A	102T>I	Substitution	Nonsynonymous coding	46%
MM04T	C1orf173	chromosome 1 open reading frame 173	CCDS30755.1	chr1_75086571-75086571_A_T	283L>I	Substitution	Nonsynonymous coding	33%
MM04T	C1orf180	chromosome 1 open reading frame 180	ENST00000327308	chr1_85096317-85096317_G_T	118L>M	Substitution	Nonsynonymous coding	21%
MM04T	C1orf180	chromosome 1 open reading frame 180	ENST00000327308	chr1_85096451-85096451_C_T	73R>K	Substitution	Nonsynonymous coding	32%
MM04T	C1orf196	chromosome 1 open reading frame 196	ENST00000401953	chr1_14220177-14220177_C_T	16A>V	Substitution	Nonsynonymous coding	23%
MM04T	C1orf228	chromosome 1 open reading frame 228	NM_001145636	chr1_45166669-45166669_G_T	173D>Y	Substitution	Nonsynonymous coding	30%
MM04T	C1orf228	chromosome 1 open reading frame 228	NM_001145636	chr1_45191016-45191016_G_A	422D>N	Substitution	Nonsynonymous coding	35%
MM04T	C1QB	complement component 1, q subcomponent, B chain	CCDS228.1	chr1_22987325-22987325_G_T	70D>Y	Substitution	Nonsynonymous coding	18%
MM04T	C1QL2	complement component 1, q subcomponent-like 2	CCDS42737.1	chr2_119914405-119914405_G_T	279F>L	Substitution	Nonsynonymous coding	29%
MM04T	C1QTNF3	C1q and tumor necrosis factor related protein 3	CCDS3902.1	chr5_33989592-33989592_G_T	252S>Y	Substitution	Nonsynonymous coding	31%
MM04T	C1QTNF3	C1q and tumor necrosis factor related protein 3	CCDS3902.1	chr5_33998765-33998765_G_T	240F>L	Substitution	Nonsynonymous coding	36%
MM04T	C1QTNF3	C1q and tumor necrosis factor related protein 3	CCDS34141.1	chr5_34020767-34020767_C_T	294R>Q	Substitution	Nonsynonymous coding	32%

MM04T	C1QTNF3	C1q and tumor necrosis factor related protein 3	CCDS34141.1	chr5_34035800-34035800_G_A	123R>X	Substitution	Nonsense	32%
MM04T	C1QTNF8	C1q and tumor necrosis factor related protein 8	CCDS32358.1	chr16_1144022-1144022_G_A	80R>W	Substitution	Nonsynonymous coding	48%
MM04T	C20orf194	chromosome 20 open reading frame 194	CCDS42851.1	chr20_3234400-3234400_G_T	1131F>L	Substitution	Nonsynonymous coding	35%
MM04T	C20orf194	chromosome 20 open reading frame 194	CCDS42851.1	chr20_3321242-3321242_C_T	297R>Q	Substitution	Nonsynonymous coding	31%
MM04T	C20orf26	chromosome 20 open reading frame 26	CCDS33447.1	chr20_20232400-20232400_A_G	774Q>R	Substitution	Nonsynonymous coding	44%
MM04T	C21orf128	chromosome 21 open reading frame 128	ENST00000329015	chr21_43523810-43523810_C_A	141Q>H	Substitution	Nonsynonymous coding	34%
MM04T	C21orf59	chromosome 21 open reading frame 59	CCDS13617.1	chr21_33982298-33982298_C_T	53E>K	Substitution	Nonsynonymous coding	23%
MM04T	C21orf67	chromosome 21 open reading frame 67	ENST00000330551	chr21_46354803-46354803_G_T	180S>Y	Substitution	Nonsynonymous coding	21%
MM04T	C21orf91	chromosome 21 open reading frame 91	CCDS42907.1	chr21_19167581-19167581_G_A	224S>L	Substitution	Nonsynonymous coding	25%
MM04T	C22orf31	chromosome 22 open reading frame 31	CCDS13848.1	chr22_29456594-29456594_G_T	81L>I	Substitution	Nonsynonymous coding	21%
MM04T	C2CD3	C2 calcium-dependent domain containing 3	ENST00000334126	chr11_73745122-73745122_G_T	2028S>X	Substitution	Nonsense	34%
MM04T	C2CD4C	C2 calcium-dependent domain containing 4C	CCDS45890.1	chr19_407529-407529_C_T	278R>H	Substitution	Nonsynonymous coding	16%
MM04T	C2orf16	chromosome 2 open reading frame 16	CCDS42666.1	chr2_27800099-27800099_C_A	220F>L	Substitution	Nonsynonymous coding	26%
MM04T	C2orf16	chromosome 2 open reading frame 16	CCDS42666.1	chr2_27801729-27801729_G_T	764A>S	Substitution	Nonsynonymous coding	29%
MM04T	C2orf16	chromosome 2 open reading frame 16	CCDS42666.1	chr2_27803380-27803380_C_A	1314P>H	Substitution	Nonsynonymous coding	26%
MM04T	C2orf16	chromosome 2 open reading frame 16	CCDS42666.1	chr2_27800898-27800898_G_T	487E>X	Substitution	Nonsense	32%
MM04T	C2orf53	chromosome 2 open reading frame 53	CCDS1739.1	chr2_27361187-27361187_T_C	4Q>R	Substitution	Nonsynonymous coding	20%
MM04T	C2orf71	chromosome 2 open reading frame 71	CCDS42669.1	chr2_29295603-29295603_T_C	509T>A	Substitution	Nonsynonymous coding	33%
MM04T	C2orf80	chromosome 2 open reading frame 80	ENST00000451342	chr2_209030493-209030493_G_T	130F>L	Substitution	Nonsynonymous coding	35%
MM04T	C3	complement component 3	CCDS32883.1	chr19_6697782-6697782_C_T	822E>K	Substitution	Nonsynonymous coding	17%
MM04T	C3orf35	chromosome 3 open reading frame 35	CCDS43065.1	chr3_37458846-37458846_G_A	30R>Q	Substitution	Nonsynonymous coding	26%
MM04T	C3orf58	chromosome 3 open reading frame 58	CCDS3130.1	chr3_143704565-143704565_G_T	280E>X	Substitution	Nonsense	35%
MM04T	C3orf70	chromosome 3 open reading frame 70	CCDS33900.1	chr3_184801040-184801040_A_T	170S>T	Substitution	Nonsynonymous coding	39%
MM04T	C4BPA	complement component 4 binding protein, alpha	CCDS1477.1	chr1_207297598-207297598_C_A	198S>Y	Substitution	Nonsynonymous coding	21%
MM04T	C4orf21	chromosome 4 open reading frame 21	NM_018392	chr4_113460725-113460725_G_T	2098S>Y	Substitution	Nonsynonymous coding	33%
MM04T	C4orf21	chromosome 4 open reading frame 21	NM_018392	chr4_113479416-113479416_G_T	1670F>L	Substitution	Nonsynonymous coding	32%
MM04T	C4orf21	chromosome 4 open reading frame 21	NM_018392	chr4_113506809-113506809_A_C	1330F>C	Substitution	Nonsynonymous coding	24%
MM04T	C4orf29	chromosome 4 open reading frame 29	CCDS47131.1	chr4_128949778-128949778_C_A	283S>Y	Substitution	Nonsynonymous coding	26%
MM04T	C5orf34	chromosome 5 open reading frame 34	NM_198566	chr5_43506369-43506369_C_A	138R>I	Substitution	Nonsynonymous coding	23%
MM04T	C5orf34	chromosome 5 open reading frame 34	NM_198566	chr5_43508747-43508747_C_A	73D>Y	Substitution	Nonsynonymous coding	21%
MM04T	C5orf51	chromosome 5 open reading frame 51	CCDS34151.1	chr5_41909953-41909953_G_T	105E>X	Substitution	Nonsense	14%
MM04T	C5orf51	chromosome 5 open reading frame 51	CCDS34151.1	chr5_41917288-41917288_G_T	258E>X	Substitution	Nonsense	24%

MM04T	C6orf118	chromosome 6 open reading frame 118	CCDS5288.1	chr6_165703533-165703533_C_A	382D>Y	Substitution	Nonsynonymous coding	36%
MM04T	C6orf15	chromosome 6 open reading frame 15	CCDS4693.1	chr6_31079444-31079444_G_T	231P>H	Substitution	Nonsynonymous coding	24%
MM04T	C6orf201	chromosome 6 open reading frame 201	CCDS43419.1	chr6_4099276-4099276_G_T	43R>I	Substitution	Nonsynonymous coding	20%
MM04T	C6orf211	chromosome 6 open reading frame 211	CCDS5233.1	chr6_151785698-151785698_C_A	168T>N	Substitution	Nonsynonymous coding	38%
MM04T	C6orf25	chromosome 6 open reading frame 25	CCDS34406.1	chr6_31692529-31692529__C	NA	Insertion	Frameshift	21%
MM04T	C7	complement component 7	CCDS47201.1	chr5_40937769-40937769_A_C	182N>H	Substitution	Nonsynonymous coding	30%
MM04T	C7orf31	chromosome 7 open reading frame 31	CCDS5394.1	chr7_25198153-25198153_G_T	NA	Substitution	Splice site acceptor	25%
MM04T	C7orf49	chromosome 7 open reading frame 49	CCDS5838.2	chr7_134851388-134851388_T_C	150Y>C	Substitution	Nonsynonymous coding	32%
MM04T	C7orf63	chromosome 7 open reading frame 63	CCDS43613.1	chr7_89917641-89917641_C_A	584L>I	Substitution	Nonsynonymous coding	19%
MM04T	C8B	complement component 8, beta polypeptide	CCDS30730.1	chr1_57417816-57417816_G_T	191L>I	Substitution	Nonsynonymous coding	22%
MM04T	C8orf47	chromosome 8 open reading frame 47	CCDS34929.1	chr8_99101635-99101635_G_T	130K>N	Substitution	Nonsynonymous coding	29%
MM04T	C8orf58	chromosome 8 open reading frame 58	CCDS34862.1	chr8_22459512-22459512_G_T	229A>S	Substitution	Nonsynonymous coding	22%
MM04T	C8orf76	chromosome 8 open reading frame 76	CCDS6341.1	chr8_124238781-124238781_C_A	303G>W	Substitution	Nonsynonymous coding	26%
MM04T	C9orf135	chromosome 9 open reading frame 135	CCDS35041.1	chr9_72459484-72459484_G_T	68E>D	Substitution	Nonsynonymous coding	27%
MM04T	C9orf152	chromosome 9 open reading frame 152	CCDS35102.2	chr9_112963361-112963361_A_C	196F>C	Substitution	Nonsynonymous coding	33%
MM04T	C9orf174	chromosome 9 open reading frame 174	ENST00000375202	chr9_100109611-100109611_C_A	882F>L	Substitution	Nonsynonymous coding	36%
MM04T	C9orf174	chromosome 9 open reading frame 174	CCDS35077.1	chr9_100116887-100116887_A_C	1028E>D	Substitution	Nonsynonymous coding	36%
MM04T	C9orf3	chromosome 9 open reading frame 3	ENST00000375316	chr9_97768326-97768326_G_T	139K>N	Substitution	Nonsynonymous coding	40%
MM04T	C9orf41	chromosome 9 open reading frame 41	CCDS6649.1	chr9_77632174-77632174_C_A	141E>X	Substitution	Nonsense	28%
MM04T	C9orf72	chromosome 9 open reading frame 72	CCDS6522.1	chr9_27566800-27566800_G_A	107R>C	Substitution	Nonsynonymous coding	30%
MM04T	C9orf84	chromosome 9 open reading frame 84	CCDS6781.3	chr9_114464413-114464413_A_C	957L>V	Substitution	Nonsynonymous coding	50%
MM04T	C9orf84	chromosome 9 open reading frame 84	CCDS6781.3	chr9_114468869-114468869_G_T	842L>I	Substitution	Nonsynonymous coding	47%
MM04T	C9orf84	chromosome 9 open reading frame 84	CCDS6781.3	chr9_114490010-114490010_C_A	515E>D	Substitution	Nonsynonymous coding	34%
MM04T	C9orf84	chromosome 9 open reading frame 84	CCDS6781.3	chr9_114490190-114490190_G_T	455F>L	Substitution	Nonsynonymous coding	16%
MM04T	C9orf84	chromosome 9 open reading frame 84	ENST00000374283	chr9_114548272-114548272_C_T	30R>Q	Substitution	Nonsynonymous coding	14%
MM04T	C9orf85	chromosome 9 open reading frame 85	CCDS6639.1	chr9_74561973-74561973_C_A	52L>I	Substitution	Nonsynonymous coding	23%
MM04T	CA10	carbonic anhydrase X	CCDS32684.1	chr17_50149693-50149693_C_A	41G>V	Substitution	Nonsynonymous coding	18%
MM04T	CA13	carbonic anhydrase XIII	CCDS6236.1	chr8_86180833-86180833_C_A	216P>T	Substitution	Nonsynonymous coding	26%
MM04T	CA3	carbonic anhydrase III, muscle specific	CCDS6238.1	chr8_86354340-86354340_C_T	91R>C	Substitution	Nonsynonymous coding	35%
MM04T	CACHD1	cache domain containing 1	CCDS628.2	chr1_65107570-65107570_A_C	307K>N	Substitution	Nonsynonymous coding	39%
MM04T	CACHD1	cache domain containing 1	CCDS628.2	chr1_65129446-65129446_A_C	623T>P	Substitution	Nonsynonymous coding	31%
MM04T	CACNA1A	calcium channel, voltage-dependent, P/Q type, alpha 1A subunit	CCDS45998.1	chr19_13345768-13345768_C_A	1739R>L	Substitution	Nonsynonymous coding	23%

MM04T	CACNA1A	calcium channel, voltage-dependent, P/Q type, alpha 1A subunit	CCDS45998.1	chr19_13363807-13363807_G_T	1622L>M	Substitution	Nonsynonymous coding	22%
MM04T	CACNA1A	calcium channel, voltage-dependent, P/Q type, alpha 1A subunit	CCDS45998.1	chr19_13368351-13368351_G_A	1468S>L	Substitution	Nonsynonymous coding	23%
MM04T	CACNA1A	calcium channel, voltage-dependent, P/Q type, alpha 1A subunit	CCDS45998.1	chr19_13411445-13411445_G_A	733A>V	Substitution	Nonsynonymous coding	29%
MM04T	CACNA1B	calcium channel, voltage-dependent, N type, alpha 1B subunit	NM_000718	chr9_140943765-140943765_C_A	1236F>L	Substitution	Nonsynonymous coding	22%
MM04T	CACNA1B	calcium channel, voltage-dependent, N type, alpha 1B subunit	NM_000718	chr9_140954105-140954105_G_T	NA	Substitution	Splice site acceptor	29%
MM04T	CACNA1B	calcium channel, voltage-dependent, N type, alpha 1B subunit	NM_000718	chr9_140850229-140850229_G_T	384E>X	Substitution	Nonsense	24%
MM04T	CACNA1C	calcium channel, voltage-dependent, L type, alpha 1C subunit	CCDS44788.1	chr12_2566785-2566785_C_A	224L>I	Substitution	Nonsynonymous coding	20%
MM04T	CACNA1C	calcium channel, voltage-dependent, L type, alpha 1C subunit	CCDS44788.1	chr12_2783740-2783740_C_T	1635A>V	Substitution	Nonsynonymous coding	20%
MM04T	CACNA1D	calcium channel, voltage-dependent, L type, alpha 1D subunit	CCDS2872.1	chr3_53531413-53531413_G_A	101R>Q	Substitution	Nonsynonymous coding	20%
MM04T	CACNA1D	calcium channel, voltage-dependent, L type, alpha 1D subunit	CCDS2872.1	chr3_53736669-53736669_G_T	408E>X	Substitution	Nonsense	31%
MM04T	CACNA1D	calcium channel, voltage-dependent, L type, alpha 1D subunit	CCDS2872.1	chr3_53845407-53845407_G_T	2174E>X	Substitution	Nonsense	23%
MM04T	CACNA1E	calcium channel, voltage-dependent, R type, alpha 1E subunit	NM_000721	chr1_181702154-181702154_G_T	978A>S	Substitution	Nonsynonymous coding	14%
MM04T	CACNA1F	calcium channel, voltage-dependent, L type, alpha 1F subunit	CCDS35253.1	chrX_49071664-49071664_C_T	1171R>H	Substitution	Nonsynonymous coding	31%
MM04T	CACNA1F	calcium channel, voltage-dependent, L type, alpha 1F subunit	CCDS35253.1	chrX_49075868-49075868_A_G	873F>S	Substitution	Nonsynonymous coding	39%
MM04T	CACNA1F	calcium channel, voltage-dependent, L type, alpha 1F subunit	CCDS35253.1	chrX_49086744-49086744_G_A	252A>V	Substitution	Nonsynonymous coding	20%
MM04T	CACNA1G	calcium channel, voltage-dependent, T type, alpha 1G subunit	CCDS45730.1	chr17_48676991-48676991_C_T	1154A>V	Substitution	Nonsynonymous coding	18%
MM04T	CACNA1I	calcium channel, voltage-dependent, T type, alpha 1I subunit	CCDS46710.1	chr22_40078593-40078593_G_T	1919E>D	Substitution	Nonsynonymous coding	32%
MM04T	CACNA2D3	calcium channel, voltage-dependent, alpha 2/delta subunit 3	NM_018398	chr3_55108143-55108143_G_T	1062E>D	Substitution	Nonsynonymous coding	28%
MM04T	CACNA2D3	calcium channel, voltage-dependent, alpha 2/delta subunit 3	NM_018398	chr3_54921994-54921994_G_T	689E>X	Substitution	Nonsense	30%
MM04T	CACNA2D4	calcium channel, voltage-dependent, alpha 2/delta subunit 4	CCDS44785.1	chr12_1988975-1988975_C_T	520E>K	Substitution	Nonsynonymous coding	45%
MM04T	CACNA2D4	calcium channel, voltage-dependent, alpha 2/delta subunit 4	CCDS44785.1	chr12_1904843-1904843_C_A	1073E>X	Substitution	Nonsense	22%
MM04T	CACNA2D4	calcium channel, voltage-dependent, alpha 2/delta subunit 4	CCDS44785.1	chr12_1967779-1967779_C_A	658E>X	Substitution	Nonsense	26%
MM04T	CACNB2	calcium channel, voltage-dependent, beta 2 subunit	CCDS41494.1	chr10_18629886-18629886_A_G	11T>A	Substitution	Nonsynonymous coding	37%
MM04T	CACNB2	calcium channel, voltage-dependent, beta 2 subunit	CCDS7129.1	chr10_18690019-18690019_C_A	20S>Y	Substitution	Nonsynonymous coding	37%
MM04T	CACNB2	calcium channel, voltage-dependent, beta 2 subunit	CCDS7125.1	chr10_18825052-18825052_C_A	410S>Y	Substitution	Nonsynonymous coding	24%
MM04T	CACNB3	calcium channel, voltage-dependent, beta 3 subunit	CCDS8769.1	chr12_49220561-49220561_G_A	305R>H	Substitution	Nonsynonymous coding	26%
MM04T	CACNB4	calcium channel, voltage-dependent, beta 4 subunit	CCDS46426.1	chr2_152695838-152695838_C_T	453R>Q	Substitution	Nonsynonymous coding	31%
MM04T	CACNG3	calcium channel, voltage-dependent, gamma subunit 3	CCDS10620.1	chr16_24373140-24373140_T_C	302S>P	Substitution	Nonsynonymous coding	37%
MM04T	CACNG4	calcium channel, voltage-dependent, gamma subunit 4	CCDS11667.1	chr17_65026692-65026692_T_C	186Y>H	Substitution	Nonsynonymous coding	21%
MM04T	CACNG5	calcium channel, voltage-dependent, gamma subunit 5	CCDS11666.1	chr17_64880752-64880752_G_A	182A>T	Substitution	Nonsynonymous coding	35%
MM04T	CACUL1	CDK2-associated, cullin domain 1	CCDS41570.1	chr10_120460887-120460887_C_A	243D>Y	Substitution	Nonsynonymous coding	22%
MM04T	CAD	carbamoyl-phosphate synthetase 2, aspartate transcarbamylase, and dihydroorotase	CCDS1742.1	chr2_27465530-27465530_T_G	2089S>A	Substitution	Nonsynonymous coding	21%

MM04T	CADM3	cell adhesion molecule 3	CCDS1182.1	chr1_159162383-159162383_G_A	116R>Q	Substitution	Nonsynonymous coding	34%
MM04T	CADPS2	Ca++-dependent secretion activator 2	CCDS47691.1	chr7_121985675-121985675_G_A	1148L>F	Substitution	Nonsynonymous coding	32%
MM04T	CAGE1	cancer antigen 1	ENST00000379918	chr6_7387309-7387309_G_A	33S>L	Substitution	Nonsynonymous coding	30%
MM04T	CALCR	calcitonin receptor	CCDS5631.1	chr7_93067568-93067568_G_A	273P>S	Substitution	Nonsynonymous coding	31%
MM04T	CALCR	calcitonin receptor	CCDS5631.1	chr7_93073013-93073013_G_T	235F>L	Substitution	Nonsynonymous coding	25%
MM04T	CALR3	calreticulin 3	CCDS12344.1	chr19_16596013-16596013_C_A	151K>N	Substitution	Nonsynonymous coding	52%
MM04T	CALR3	calreticulin 3	CCDS12344.1	chr19_16606654-16606654_C_A	34R>I	Substitution	Nonsynonymous coding	38%
MM04T	CAMK1D	calcium/calmodulin-dependent protein kinase ID	CCDS7091.1	chr10_12870801-12870801_C_A	358S>Y	Substitution	Nonsynonymous coding	27%
MM04T	CAMK2A	calcium/calmodulin-dependent protein kinase II alpha	CCDS43387.1	chr5_149636182-149636182_G_A	122A>V	Substitution	Nonsynonymous coding	13%
MM04T	CAMP	cathelicidin antimicrobial peptide	CCDS2762.1	chr3_48265135-48265135_G_A	45G>D	Substitution	Nonsynonymous coding	25%
MM04T	CAMP	cathelicidin antimicrobial peptide	CCDS2762.1	chr3_48266856-48266856_G_T	152R>I	Substitution	Nonsynonymous coding	31%
MM04T	CAMSAP1	calmodulin regulated spectrin-associated protein 1	CCDS35176.2	chr9_138713151-138713151_C_T	1119R>Q	Substitution	Nonsynonymous coding	23%
MM04T	CAMSAP2	calmodulin regulated spectrin-associated protein family, member 2	CCDS1404.1	chr1_200819006-200819006_G_A	1037E>K	Substitution	Nonsynonymous coding	35%
MM04T	CAMSAP2	calmodulin regulated spectrin-associated protein family, member 2	CCDS1404.1	chr1_200819353-200819353_C_A	1152F>L	Substitution	Nonsynonymous coding	38%
MM04T	CAMSAP2	calmodulin regulated spectrin-associated protein family, member 2	CCDS1404.1	chr1_200822455-200822455_G_T	1219E>X	Substitution	Nonsense	28%
MM04T	CAMTA1	calmodulin binding transcription activator 1	CCDS30576.1	chr1_7151400-7151400_G_A	91E>K	Substitution	Nonsynonymous coding	24%
MM04T	CAMTA1	calmodulin binding transcription activator 1	CCDS30576.1	chr1_7811280-7811280_A_G	1571T>A	Substitution	Nonsynonymous coding	29%
MM04T	CAMTA2	calmodulin binding transcription activator 2	CCDS11063.1	chr17_4872209-4872209_G_A	1180R>X	Substitution	Nonsense	12%
MM04T	CAP2	CAP, adenylate cyclase-associated protein, 2 (yeast)	CCDS4539.1	chr6_17543136-17543136_C_A	357F>L	Substitution	Nonsynonymous coding	27%
MM04T	CAPN12	calpain 12	CCDS12519.1	chr19_39230816-39230816_C_A	202E>X	Substitution	Nonsense	11%
MM04T	CAPN13	calpain 13	CCDS46252.1	chr2_30961140-30961140_G_T	538F>L	Substitution	Nonsynonymous coding	28%
MM04T	CAPN6	calpain 6	CCDS14555.1	chrX_110496247-110496247__T	NA	Insertion	Frameshift	27%
MM04T	CAPN7	calpain 7	CCDS2624.1	chr3_15253671-15253671_G_T	55E>X	Substitution	Nonsense	28%
MM04T	CAPN9	calpain 9	CCDS1586.1	chr1_230916039-230916039_G_A	410R>K	Substitution	Nonsynonymous coding	34%
MM04T	CAPZA2	capping protein (actin filament) muscle Z-line, alpha 2	CCDS5768.1	chr7_116533100-116533100_G_T	NA	Substitution	Splice site donor	13%
MM04T	CARD10	caspase recruitment domain family, member 10	CCDS13948.1	chr22_37891880-37891880_C_A	730E>D	Substitution	Nonsynonymous coding	30%
MM04T	CARD11	caspase recruitment domain family, member 11	CCDS5336.2	chr7_2985525-2985525_C_A	96E>X	Substitution	Nonsense	33%
MM04T	CARD6	caspase recruitment domain family, member 6	CCDS3935.1	chr5_40853491-40853491_G_T	686R>I	Substitution	Nonsynonymous coding	33%
MM04T	CARS2	cysteinyl-tRNA synthetase 2, mitochondrial (putative)	CCDS9514.1	chr13_111296817-111296817_G_A	444P>L	Substitution	Nonsynonymous coding	22%
MM04T	CASC5	cancer susceptibility candidate 5	CCDS42023.1	chr15_40914129-40914129_C_A	582S>Y	Substitution	Nonsynonymous coding	21%
MM04T	CASK	calcium/calmodulin-dependent serine protein kinase (MAGUK family)	CCDS14257.1	chrX_41402003-41402003_G_T	699T>N	Substitution	Nonsynonymous coding	24%
MM04T	CASK	calcium/calmodulin-dependent serine protein kinase (MAGUK family)	CCDS14257.1	chrX_41437631-41437631_G_A	489R>W	Substitution	Nonsynonymous coding	17%

MM04T	CASK	calcium/calmodulin-dependent serine protein kinase (MAGUK family)	CCDS14257.1	chrX_41530756-41530756_C_T	153E>K	Substitution	Nonsynonymous coding	18%
MM04T	CASKIN1	CASK interacting protein 1	CCDS42103.1	chr16_2233846-2233846_G_A	505R>C	Substitution	Nonsynonymous coding	18%
MM04T	CASP10	caspase 10, apoptosis-related cysteine peptidase	CCDS2340.1	chr2_202050731-202050731_C_A	77F>L	Substitution	Nonsynonymous coding	34%
MM04T	CASP10	caspase 10, apoptosis-related cysteine peptidase	CCDS2340.1	chr2_202052468-202052468_G_T	129E>D	Substitution	Nonsynonymous coding	27%
MM04T	CASP10	caspase 10, apoptosis-related cysteine peptidase	CCDS2340.1	chr2_202050503-202050503_G_T	1M>I	Substitution	Nonsynonymous coding	22%
MM04T	CASP5	caspase 5, apoptosis-related cysteine peptidase	CCDS44720.1	chr11_104878041-104878041__T	NA	Insertion	Frameshift	25%
MM04T	CASP5	caspase 5, apoptosis-related cysteine peptidase	CCDS44720.1	chr11_104866562-104866562_T_G	418K>T	Substitution	Nonsynonymous coding	34%
MM04T	CASP8	caspase 8, apoptosis-related cysteine peptidase	CCDS42798.1	chr2_202141636-202141636_G_T	308E>D	Substitution	Nonsynonymous coding	23%
MM04T	CASP8AP2	caspase 8 associated protein 2	NM_001137667	chr6_90572336-90572336_C_T	303S>L	Substitution	Nonsynonymous coding	11%
MM04T	CASP8AP2	caspase 8 associated protein 2	NM_001137667	chr6_90572627-90572627_C_A	400S>Y	Substitution	Nonsynonymous coding	28%
MM04T	CASP8AP2	caspase 8 associated protein 2	NM_001137667	chr6_90575695-90575695_C_A	896H>N	Substitution	Nonsynonymous coding	26%
MM04T	CASP8AP2	caspase 8 associated protein 2	ENST00000455594	chr6_90581120-90581120_T_A	1969F>I	Substitution	Nonsynonymous coding	34%
MM04T	CASQ2	calsequestrin 2 (cardiac muscle)	CCDS884.1	chr1_116280861-116280861_C_A	172K>N	Substitution	Nonsynonymous coding	19%
MM04T	CASS4	Cas scaffolding protein family member 4	CCDS33492.1	chr20_55027640-55027640_G_T	470D>Y	Substitution	Nonsynonymous coding	25%
MM04T	CASZ1	castor zinc finger 1	CCDS41246.1	chr1_10713503-10713503_C_T	871G>S	Substitution	Nonsynonymous coding	29%
MM04T	CATSPER1	cation channel, sperm associated 1	CCDS8127.1	chr11_65789259-65789259_G_T	507F>L	Substitution	Nonsynonymous coding	35%
MM04T	CATSPERB	catsper channel auxiliary subunit beta	CCDS32142.1	chr14_92126306-92126306_C_T	436G>D	Substitution	Nonsynonymous coding	37%
MM04T	CATSPERG	catsper channel auxiliary subunit gamma	CCDS12514.2	chr19_38834650-38834650_G_T	163E>X	Substitution	Nonsense	26%
MM04T	CBFA2T2	core-binding factor, runt domain, alpha subunit 2; translocated to, 2	CCDS13221.1	chr20_32212766-32212766_C_T	306R>W	Substitution	Nonsynonymous coding	35%
MM04T	CBL	Cbl proto-oncogene, E3 ubiquitin protein ligase	CCDS8418.1	chr11_119156172-119156172_G_T	613E>X	Substitution	Nonsense	29%
MM04T	CBLC	Cbl proto-oncogene, E3 ubiquitin protein ligase C	CCDS12643.1	chr19_45296777-45296777_G_A	395G>D	Substitution	Nonsynonymous coding	27%
MM04T	CBR1	carbonyl reductase 1	ENST00000399191	chr21_37444575-37444575_C_A	167F>L	Substitution	Nonsynonymous coding	25%
MM04T	CBR4	carbonyl reductase 4	CCDS3812.1	chr4_169928837-169928837_C_A	71E>D	Substitution	Nonsynonymous coding	18%
MM04T	CBS	cystathionine-beta-synthase	CCDS13693.1	chr21_44476940-44476940_C_T	509A>T	Substitution	Nonsynonymous coding	15%
MM04T	CCAR1	cell division cycle and apoptosis regulator 1	CCDS7282.1	chr10_70507010-70507010_G_T	204R>I	Substitution	Nonsynonymous coding	26%
MM04T	CCDC102A	coiled-coil domain containing 102A	CCDS10784.1	chr16_57552127-57552127_C_A	367E>D	Substitution	Nonsynonymous coding	21%
MM04T	CCDC102B	coiled-coil domain containing 102B	CCDS11996.2	chr18_66504238-66504238_G_A	80E>K	Substitution	Nonsynonymous coding	25%
MM04T	CCDC106	coiled-coil domain containing 106	CCDS33118.1	chr19_56163833-56163833_G_T	188K>N	Substitution	Nonsynonymous coding	22%
MM04T	CCDC108	coiled-coil domain containing 108	CCDS2430.2	chr2_219870245-219870245_C_A	1654E>D	Substitution	Nonsynonymous coding	29%
MM04T	CCDC108	coiled-coil domain containing 108	CCDS2430.2	chr2_219888108-219888108_C_A	881D>Y	Substitution	Nonsynonymous coding	28%
MM04T	CCDC110	coiled-coil domain containing 110	CCDS3843.1	chr4_186379974-186379974_C_A	589E>D	Substitution	Nonsynonymous coding	38%
MM04T	CCDC111	coiled-coil domain containing 111	CCDS3837.1	chr4_185599400-185599400_A_C	287N>H	Substitution	Nonsynonymous coding	28%

MM04T	CCDC112	coiled-coil domain containing 112	CCDS34213.1	chr5_114605499-114605499_A_	NA	Deletion	Splice site acceptor	12%
MM04T	CCDC126	coiled-coil domain containing 126	CCDS5384.1	chr7_23650970-23650970_G_T	12Q>H	Substitution	Nonsynonymous coding	34%
MM04T	CCDC129	coiled-coil domain containing 129	CCDS5435.2	chr7_31617561-31617561_G_A	228R>K	Substitution	Nonsynonymous coding	23%
MM04T	CCDC13	coiled-coil domain containing 13	CCDS2705.1	chr3_42788795-42788795_C_T	225R>Q	Substitution	Nonsynonymous coding	34%
MM04T	CCDC13	coiled-coil domain containing 13	CCDS2705.1	chr3_42799764-42799764_C_A	25R>L	Substitution	Nonsynonymous coding	16%
MM04T	CCDC134	coiled-coil domain containing 134	CCDS33654.1	chr22_42204993-42204993_G_T	33E>D	Substitution	Nonsynonymous coding	57%
MM04T	CCDC136	coiled-coil domain containing 136	CCDS47704.1	chr7_128434614-128434614_G_T	77E>X	Substitution	Nonsense	20%
MM04T	CCDC141	coiled-coil domain containing 141	ENST00000409284	chr2_179749674-179749674_G_A	558S>L	Substitution	Nonsynonymous coding	14%
MM04T	CCDC141	coiled-coil domain containing 141	ENST00000409284	chr2_179751599-179751599_C_T	NA	Substitution	Splice site acceptor	26%
MM04T	CCDC146	coiled-coil domain containing 146	CCDS34671.1	chr7_76889339-76889339__A	NA	Insertion	Frameshift	18%
MM04T	CCDC147	coiled-coil domain containing 147	CCDS31282.1	chr10_106118151-106118151_G_T	21R>I	Substitution	Nonsynonymous coding	24%
MM04T	CCDC147	coiled-coil domain containing 147	CCDS31282.1	chr10_106130713-106130713_G_T	331E>X	Substitution	Nonsense	22%
MM04T	CCDC147	coiled-coil domain containing 147	CCDS31282.1	chr10_106163512-106163512_G_T	689E>X	Substitution	Nonsense	22%
MM04T	CCDC150	coiled-coil domain containing 150	CCDS46478.1	chr2_197584347-197584347_C_T	708L>F	Substitution	Nonsynonymous coding	24%
MM04T	CCDC158	coiled-coil domain containing 158	CCDS43242.1	chr4_77244550-77244550_G_A	1057P>L	Substitution	Nonsynonymous coding	30%
MM04T	CCDC160	coiled-coil domain containing 160	CCDS48171.1	chrX_133378998-133378998_G_T	56Q>H	Substitution	Nonsynonymous coding	13%
MM04T	CCDC164	coiled-coil domain containing 164	CCDS1723.1	chr2_26624860-26624860_G_T	1M>I	Substitution	Nonsynonymous coding	34%
MM04T	CCDC168	coiled-coil domain containing 168	NM_001146197	chr13_103383129-103383129_C_A	6640V>L	Substitution	Nonsynonymous coding	28%
MM04T	CCDC170	coiled-coil domain containing 170	CCDS43515.1	chr6_151859300-151859300_G_T	103D>Y	Substitution	Nonsynonymous coding	37%
MM04T	CCDC173	coiled-coil domain containing 173	CCDS46445.1	chr2_170502397-170502397_T_A	538K>I	Substitution	Nonsynonymous coding	38%
MM04T	CCDC173	coiled-coil domain containing 173	CCDS46445.1	chr2_170505728-170505728_T_G	427E>D	Substitution	Nonsynonymous coding	12%
MM04T	CCDC175	coiled-coil domain containing 175	NM_001164399	chr14_59988313-59988313_G_A	693R>W	Substitution	Nonsynonymous coding	32%
MM04T	CCDC23	coiled-coil domain containing 23	CCDS474.1	chr1_43282209-43282209_G_T	3P>T	Substitution	Nonsynonymous coding	16%
MM04T	CCDC33	coiled-coil domain containing 33	CCDS42058.1	chr15_74623090-74623090_C_T	515R>X	Substitution	Nonsense	36%
MM04T	CCDC38	coiled-coil domain containing 38	CCDS9056.1	chr12_96330256-96330256_G_T	11S>Y	Substitution	Nonsynonymous coding	28%
MM04T	CCDC38	coiled-coil domain containing 38	CCDS9056.1	chr12_96284705-96284705_A_C	259L>X	Substitution	Nonsense	23%
MM04T	CCDC40	coiled-coil domain containing 40	CCDS42395.1	chr17_78071198-78071198_G_A	1059R>Q	Substitution	Nonsynonymous coding	24%
MM04T	CCDC41	coiled-coil domain containing 41	CCDS41820.1	chr12_94769687-94769687_C_T	303R>Q	Substitution	Nonsynonymous coding	48%
MM04T	CCDC42B	coiled-coil domain containing 42B	CCDS44983.1	chr12_113593120-113593120_G_A	249G>E	Substitution	Nonsynonymous coding	20%
MM04T	CCDC54	coiled-coil domain containing 54	CCDS2949.1	chr3_107096927-107096927_G_T	165E>X	Substitution	Nonsense	22%
MM04T	CCDC59	coiled-coil domain containing 59	CCDS9023.1	chr12_82748355-82748355_C_A	161K>N	Substitution	Nonsynonymous coding	38%
MM04T	CCDC59	coiled-coil domain containing 59	CCDS9023.1	chr12_82750999-82750999_C_A	68K>N	Substitution	Nonsynonymous coding	39%

MM04T	CCDC60	coiled-coil domain containing 60	CCDS9190.1	chr12_119937913-119937913_A_C	196K>N	Substitution	Nonsynonymous coding	24%
MM04T	CCDC63	coiled-coil domain containing 63	CCDS9151.1	chr12_111296402-111296402_G_T	64K>N	Substitution	Nonsynonymous coding	34%
MM04T	CCDC65	coiled-coil domain containing 65	CCDS8772.1	chr12_49298822-49298822_G_T	76E>X	Substitution	Nonsense	18%
MM04T	CCDC68	coiled-coil domain containing 68	CCDS11959.1	chr18_52586519-52586519_G_A	258R>C	Substitution	Nonsynonymous coding	31%
MM04T	CCDC68	coiled-coil domain containing 68	CCDS11959.1	chr18_52608292-52608292_A_C	47I>S	Substitution	Nonsynonymous coding	29%
MM04T	CCDC70	coiled-coil domain containing 70	CCDS9431.1	chr13_52439671-52439671_G_A	53E>K	Substitution	Nonsynonymous coding	30%
MM04T	CCDC73	coiled-coil domain containing 73	CCDS41630.1	chr11_32663620-32663620_T_G	316E>D	Substitution	Nonsynonymous coding	31%
MM04T	CCDC77	coiled-coil domain containing 77	CCDS8503.1	chr12_550121-550121_C_A	427L>I	Substitution	Nonsynonymous coding	30%
MM04T	CCDC78	coiled-coil domain containing 78	CCDS32353.1	chr16_776075-776075_C_T	24R>Q	Substitution	Nonsynonymous coding	31%
MM04T	CCDC80	coiled-coil domain containing 80	CCDS2968.1	chr3_112337838-112337838_T_C	717T>A	Substitution	Nonsynonymous coding	29%
MM04T	CCDC80	coiled-coil domain containing 80	ENST00000447230	chr3_112358764-112358764_C_T	8V>M	Substitution	Nonsynonymous coding	22%
MM04T	CCDC83	coiled-coil domain containing 83	CCDS8271.1	chr11_85630543-85630543_C_A	442S>Y	Substitution	Nonsynonymous coding	44%
MM04T	CCDC85A	coiled-coil domain containing 85A	CCDS46290.1	chr2_56420355-56420355_G_T	340Q>H	Substitution	Nonsynonymous coding	30%
MM04T	CCDC88A	coiled-coil domain containing 88A	ENST00000426576	chr2_55522567-55522567_G_A	1081A>V	Substitution	Nonsynonymous coding	33%
MM04T	CCDC88A	coiled-coil domain containing 88A	CCDS46288.1	chr2_55539636-55539636_T_C	1337N>S	Substitution	Nonsynonymous coding	31%
MM04T	CCDS6751.1	-	CCDS6751.1	chr9_104071628-104071628_A_G	174Q>R	Substitution	Nonsynonymous coding	31%
MM04T	CCDS6751.1	-	CCDS6751.1	chr9_104048470-104048470_G_T	113E>X	Substitution	Nonsense	14%
MM04T	CCDS757.1	-	CCDS757.1	chr1_99772517-99772517_G_T	748R>I	Substitution	Nonsynonymous coding	31%
MM04T	CCL11	chemokine (C-C motif) ligand 11	CCDS11279.1	chr17_32614646-32614646_G_T	77K>N	Substitution	Nonsynonymous coding	46%
MM04T	CCL21	chemokine (C-C motif) ligand 21	CCDS6571.1	chr9_34709676-34709676_G_T	64F>L	Substitution	Nonsynonymous coding	37%
MM04T	CCNA1	cyclin A1	CCDS9357.1	chr13_37012807-37012807_C_A	232Y>X	Substitution	Nonsense	26%
MM04T	CCNB1	cyclin B1	CCDS3997.1	chr5_68467184-68467184_C_A	151L>I	Substitution	Nonsynonymous coding	28%
MM04T	CCNB3	cyclin B3	CCDS14331.1	chrX_50054102-50054102_C_A	978S>Y	Substitution	Nonsynonymous coding	22%
MM04T	CCNT1	cyclin T1	CCDS8766.1	chr12_49093563-49093563_C_T	165R>Q	Substitution	Nonsynonymous coding	18%
MM04T	CCNYL2	cyclin Y-like 2	ENST00000426433	chr10_42907224-42907224_G_T	300L>I	Substitution	Nonsynonymous coding	30%
MM04T	CCNYL2	cyclin Y-like 2	ENST00000426433	chr10_42920835-42920835_C_A	192L>F	Substitution	Nonsynonymous coding	33%
MM04T	CCPG1	cell cycle progression 1	CCDS42039.1	chr15_55669255-55669255_C_A	116E>X	Substitution	Nonsense	30%
MM04T	CCR9	chemokine (C-C motif) receptor 9	CCDS2732.1	chr3_45942383-45942383_G_T	35D>Y	Substitution	Nonsynonymous coding	30%
MM04T	CCT4	chaperonin containing TCP1, subunit 4 (delta)	CCDS33206.1	chr2_62106045-62106045_C_A	161E>X	Substitution	Nonsense	21%
MM04T	CCT6B	chaperonin containing TCP1, subunit 6B (zeta 2)	CCDS32617.1	chr17_33255092-33255092_C_T	523R>Q	Substitution	Nonsynonymous coding	27%
MM04T	CCT6B	chaperonin containing TCP1, subunit 6B (zeta 2)	CCDS32617.1	chr17_33256026-33256026_C_T	493G>E	Substitution	Nonsynonymous coding	27%
MM04T	CCT7	chaperonin containing TCP1, subunit 7 (eta)	CCDS46336.1	chr2_73478366-73478366_G_A	406V>M	Substitution	Nonsynonymous coding	44%

MM04T	CD14	CD14 molecule	CCDS4232.1	chr5_140011496-140011496_G_A	358S>L	Substitution	Nonsynonymous coding	30%
MM04T	CD19	CD19 molecule	CCDS10644.1	chr16_28950050-28950050_C_T	514R>C	Substitution	Nonsynonymous coding	33%
MM04T	CD1E	CD1e molecule	CCDS41417.1	chr1_158325323-158325323_C_A	197L>I	Substitution	Nonsynonymous coding	25%
MM04T	CD1E	CD1e molecule	CCDS41417.1	chr1_158326593-158326593_G_T	358K>N	Substitution	Nonsynonymous coding	41%
MM04T	CD200R1	CD200 receptor 1	CCDS2969.1	chr3_112648079-112648079_G_A	160P>S	Substitution	Nonsynonymous coding	15%
MM04T	CD207	CD207 molecule, langerin	NM_015717	chr2_71060874-71060874_C_A	156E>D	Substitution	Nonsynonymous coding	22%
MM04T	CD209	CD209 molecule	CCDS12186.1	chr19_7809892-7809892_C_T	279D>N	Substitution	Nonsynonymous coding	31%
MM04T	CD22	CD22 molecule	CCDS12457.1	chr19_35832370-35832370_G_T	544E>D	Substitution	Nonsynonymous coding	22%
MM04T	CD22	CD22 molecule	CCDS12457.1	chr19_35836617-35836617_G_A	774R>Q	Substitution	Nonsynonymous coding	27%
MM04T	CD36	CD36 molecule (thrombospondin receptor)	CCDS34673.1	chr7_80302097-80302097_C_A	379F>L	Substitution	Nonsynonymous coding	31%
MM04T	CD4	CD4 molecule	CCDS8562.1	chr12_6924121-6924121_G_T	190Q>H	Substitution	Nonsynonymous coding	22%
MM04T	CD40LG	CD40 ligand	CCDS14659.1	chrX_135736565-135736565_G_T	108E>X	Substitution	Nonsense	15%
MM04T	CD48	CD48 molecule	CCDS1208.1	chr1_160651069-160651069_C_A	192R>M	Substitution	Nonsynonymous coding	17%
MM04T	CD53	CD53 molecule	CCDS829.1	chr1_111435078-111435078_G_A	59V>M	Substitution	Nonsynonymous coding	23%
MM04T	CD79A	CD79a molecule, immunoglobulin-associated alpha	CCDS12589.1	chr19_42383220-42383220_C_A	80F>L	Substitution	Nonsynonymous coding	20%
MM04T	CD82	CD82 molecule	CCDS7909.1	chr11_44616262-44616262_A_G	17N>S	Substitution	Nonsynonymous coding	23%
MM04T	CD86	CD86 molecule	CCDS3009.1	chr3_121822581-121822581_G_T	96R>I	Substitution	Nonsynonymous coding	29%
MM04T	CD96	CD96 molecule	CCDS2959.1	chr3_111366453-111366453_T_G	530F>C	Substitution	Nonsynonymous coding	25%
MM04T	CD99L2	CD99 molecule-like 2	CCDS35427.1	chrX_149984543-149984543_C_T	47D>N	Substitution	Nonsynonymous coding	33%
MM04T	CDA	cytidine deaminase	CCDS210.1	chr1_20940377-20940377_G_T	103R>S	Substitution	Nonsynonymous coding	14%
MM04T	CDADC1	cytidine and dCMP deaminase domain containing 1	CCDS9415.1	chr13_49841638-49841638_G_A	148R>Q	Substitution	Nonsynonymous coding	18%
MM04T	CDADC1	cytidine and dCMP deaminase domain containing 1	CCDS9415.1	chr13_49860388-49860388_C_A	481L>I	Substitution	Nonsynonymous coding	26%
MM04T	CDADC1	cytidine and dCMP deaminase domain containing 1	CCDS9415.1	chr13_49842189-49842189_C_T	332R>X	Substitution	Nonsense	25%
MM04T	CDC14A	cell division cycle 14A	ENST00000455467	chr1_100817348-100817348_A_C	12Q>H	Substitution	Nonsynonymous coding	20%
MM04T	CDC14A	cell division cycle 14A	CCDS770.1	chr1_100963699-100963699_T_A	453S>T	Substitution	Nonsynonymous coding	18%
MM04T	CDC23	cell division cycle 23	CCDS4200.2	chr5_137536912-137536912_G_A	180R>X	Substitution	Nonsense	34%
MM04T	CDC26	cell division cycle 26	CCDS6790.1	chr9_116034720-116034720_C_T	23R>Q	Substitution	Nonsynonymous coding	32%
MM04T	CDC27	cell division cycle 27	CCDS45720.1	chr17_45199819-45199819_C_A	801E>X	Substitution	Nonsense	19%
MM04T	CDC37L1	cell division cycle 37-like 1	CCDS6454.1	chr9_4697870-4697870_G_T	246Q>H	Substitution	Nonsynonymous coding	33%
MM04T	CDC42BPA	CDC42 binding protein kinase alpha (DMPK-like)	ENST00000334218	chr1_227198659-227198659_G_T	1620L>I	Substitution	Nonsynonymous coding	22%
MM04T	CDC42BPG	CDC42 binding protein kinase gamma (DMPK-like)	CCDS31601.1	chr11_64604401-64604401_C_A	432K>N	Substitution	Nonsynonymous coding	24%
MM04T	CDC42EP4	CDC42 effector protein (Rho GTPase binding) 4	CCDS11695.1	chr17_71282205-71282205_C_A	145K>N	Substitution	Nonsynonymous coding	27%

MM04T	CDC5L	cell division cycle 5-like	CCDS4912.1	chr6_44376338-44376338_G_A	354R>Q	Substitution	Nonsynonymous coding	35%
MM04T	CDC5L	cell division cycle 5-like	CCDS4912.1	chr6_44413496-44413496_G_T	732M>I	Substitution	Nonsynonymous coding	24%
MM04T	CDC73	cell division cycle 73	CCDS1382.1	chr1_193104712-193104712_G_A	139R>Q	Substitution	Nonsynonymous coding	21%
MM04T	CDC73	cell division cycle 73	CCDS1382.1	chr1_193119509-193119509_G_T	302E>X	Substitution	Nonsense	19%
MM04T	CDCA2	cell division cycle associated 2	CCDS6049.1	chr8_25343279-25343279_A_G	457N>S	Substitution	Nonsynonymous coding	36%
MM04T	CDCA7	cell division cycle associated 7	CCDS2252.1	chr2_174228154-174228154_G_T	195E>D	Substitution	Nonsynonymous coding	27%
MM04T	CDH1	cadherin 1, type 1, E-cadherin (epithelial)	CCDS10869.1	chr16_68855995-68855995_C_A	601F>L	Substitution	Nonsynonymous coding	22%
MM04T	CDH1	cadherin 1, type 1, E-cadherin (epithelial)	CCDS10869.1	chr16_68857443-68857443_G_A	693G>D	Substitution	Nonsynonymous coding	30%
MM04T	CDH1	cadherin 1, type 1, E-cadherin (epithelial)	CCDS10869.1	chr16_68857307-68857307_G_T	648E>X	Substitution	Nonsense	27%
MM04T	CDH10	cadherin 10, type 2 (T2-cadherin)	CCDS3892.1	chr5_24511603-24511603_G_T	279L>I	Substitution	Nonsynonymous coding	40%
MM04T	CDH12	cadherin 12, type 2 (N-cadherin 2)	CCDS3890.1	chr5_21755927-21755927_C_T	553R>Q	Substitution	Nonsynonymous coding	37%
MM04T	CDH18	cadherin 18, type 2	CCDS3889.1	chr5_19612701-19612701_C_A	218R>I	Substitution	Nonsynonymous coding	27%
MM04T	CDH19	cadherin 19, type 2	CCDS11994.1	chr18_64172492-64172492_G_T	626L>I	Substitution	Nonsynonymous coding	39%
MM04T	CDH19	cadherin 19, type 2	CCDS11994.1	chr18_64218463-64218463_C_A	215E>X	Substitution	Nonsense	26%
MM04T	CDH2	cadherin 2, type 1, N-cadherin (neuronal)	CCDS11891.1	chr18_25572735-25572735_G_T	410Q>K	Substitution	Nonsynonymous coding	18%
MM04T	CDH23	cadherin-related 23	NM_022124	chr10_73565657-73565657_C_A	2656A>E	Substitution	Nonsynonymous coding	22%
MM04T	CDH26	cadherin 26	CCDS13485.1	chr20_58563977-58563977_C_T	348R>C	Substitution	Nonsynonymous coding	34%
MM04T	CDH26	cadherin 26	CCDS13485.1	chr20_58571721-58571721_C_A	642L>I	Substitution	Nonsynonymous coding	20%
MM04T	CDH6	cadherin 6, type 2, K-cadherin (fetal kidney)	CCDS3894.1	chr5_31267764-31267764_C_A	62L>I	Substitution	Nonsynonymous coding	34%
MM04T	CDH9	cadherin 9, type 2 (T1-cadherin)	CCDS3893.1	chr5_26915896-26915896_G_T	122S>Y	Substitution	Nonsynonymous coding	44%
MM04T	CDHR3	cadherin-related family member 3	CCDS47684.1	chr7_105635231-105635231_T_G	183M>R	Substitution	Nonsynonymous coding	29%
MM04T	CDK1	cyclin-dependent kinase 1	CCDS44408.1	chr10_62551966-62551966_G_T	238K>N	Substitution	Nonsynonymous coding	25%
MM04T	CDK11A	cyclin-dependent kinase 11A	ENST00000340677	chr1_1588868-1588868_C_A	23R>M	Substitution	Nonsynonymous coding	33%
MM04T	CDK11A	cyclin-dependent kinase 11A	CCDS44042.1	chr1_1635294-1635294_G_A	627S>L	Substitution	Nonsynonymous coding	21%
MM04T	CDK18	cyclin-dependent kinase 18	CCDS1454.1	chr1_205492385-205492385_C_A	30F>L	Substitution	Nonsynonymous coding	19%
MM04T	CDK18	cyclin-dependent kinase 18	CCDS1454.1	chr1_205492772-205492772_C_A	98L>M	Substitution	Nonsynonymous coding	14%
MM04T	CDK20	cyclin-dependent kinase 20	ENST00000286878	chr9_90582206-90582206_G_A	383P>S	Substitution	Nonsynonymous coding	32%
MM04T	CDK5RAP2	CDK5 regulatory subunit associated protein 2	CCDS6823.1	chr9_123290180-123290180_C_A	301E>D	Substitution	Nonsynonymous coding	24%
MM04T	CDK5RAP2	CDK5 regulatory subunit associated protein 2	ENST00000345313	chr9_123298902-123298902_C_A	NA	Substitution	Splice site acceptor	36%
MM04T	CDK8	cyclin-dependent kinase 8	CCDS9317.1	chr13_26911734-26911734_A_C	53Q>H	Substitution	Nonsynonymous coding	32%
MM04T	CDKL1	cyclin-dependent kinase-like 1 (CDC2-related kinase)	ENST00000356146	chr14_50802735-50802735_C_A	986E>D	Substitution	Nonsynonymous coding	25%
MM04T	CDKL2	cyclin-dependent kinase-like 2 (CDC2-related kinase)	CCDS3570.1	chr4_76507101-76507101_C_A	475G>V	Substitution	Nonsynonymous coding	27%

MM04T	CDKL2	cyclin-dependent kinase-like 2 (CDC2-related kinase)	CCDS3570.1	chr4_76551055-76551055_C_T	40D>N	Substitution	Nonsynonymous coding	37%
MM04T	CDKL3	cyclin-dependent kinase-like 3	CCDS47264.1	chr5_133644369-133644369_A_C	311S>A	Substitution	Nonsynonymous coding	32%
MM04T	CDKL5	cyclin-dependent kinase-like 5	CCDS14186.1	chrX_18613531-18613531_C_A	270L>I	Substitution	Nonsynonymous coding	35%
MM04T	CDS2	CDP-diacylglycerol synthase (phosphatidate cytidylyltransferase) 2	CCDS13088.1	chr20_5159565-5159565_A_C	164K>N	Substitution	Nonsynonymous coding	24%
MM04T	CDT1	chromatin licensing and DNA replication factor 1	CCDS32510.1	chr16_88873767-88873767_G_T	452E>X	Substitution	Nonsense	21%
MM04T	CDYL	chromodomain protein, Y-like	CCDS4491.2	chr6_4892414-4892414_G_T	164E>D	Substitution	Nonsynonymous coding	30%
MM04T	CEACAM18	carcinoembryonic antigen-related cell adhesion molecule 18	CCDS46159.1	chr19_51984899-51984899_G_A	279R>H	Substitution	Nonsynonymous coding	33%
MM04T	CEACAM21	carcinoembryonic antigen-related cell adhesion molecule 21	CCDS46086.1	chr19_42083873-42083873_C_A	129S>Y	Substitution	Nonsynonymous coding	21%
MM04T	CEACAM8	carcinoembryonic antigen-related cell adhesion molecule 8	CCDS12610.1	chr19_43087486-43087486_G_A	321A>V	Substitution	Nonsynonymous coding	35%
MM04T	CEBPA	CCAAT/enhancer binding protein (C/EBP), alpha	NM_004364	chr19_33792343-33792343_C_A	326K>N	Substitution	Nonsynonymous coding	20%
MM04T	CEBPZ	CCAAT/enhancer binding protein (C/EBP), zeta	CCDS1787.1	chr2_37456067-37456067_T_G	90N>T	Substitution	Nonsynonymous coding	41%
MM04T	CELF3	CUGBP, Elav-like family member 3	CCDS1002.1	chr1_151680125-151680125_C_T	NA	Substitution	Splice site acceptor	18%
MM04T	CELSR2	cadherin, EGF LAG seven-pass G-type receptor 2 (flamingo homolog, Drosophila)	CCDS796.1	chr1_109793750-109793750_G_A	350R>Q	Substitution	Nonsynonymous coding	33%
MM04T	CELSR2	cadherin, EGF LAG seven-pass G-type receptor 2 (flamingo homolog, Drosophila)	CCDS796.1	chr1_109795811-109795811_G_A	1037R>H	Substitution	Nonsynonymous coding	35%
MM04T	CELSR3	cadherin, EGF LAG seven-pass G-type receptor 3 (flamingo homolog, Drosophila)	CCDS2775.1	chr3_48697393-48697393_C_T	892R>H	Substitution	Nonsynonymous coding	30%
MM04T	CELSR3	cadherin, EGF LAG seven-pass G-type receptor 3 (flamingo homolog, Drosophila)	CCDS2775.1	chr3_48697667-48697667_G_A	801R>C	Substitution	Nonsynonymous coding	21%
MM04T	CENPB	centromere protein B, 80kDa	CCDS13064.1	chr20_3766895-3766895_C_T	79G>D	Substitution	Nonsynonymous coding	25%
MM04T	CENPC1	centromere protein C 1	CCDS47063.1	chr4_68357973-68357973_G_T	814L>I	Substitution	Nonsynonymous coding	31%
MM04T	CENPC1	centromere protein C 1	CCDS47063.1	chr4_68378261-68378261_C_A	491D>Y	Substitution	Nonsynonymous coding	41%
MM04T	CENPF	centromere protein F, 350/400kDa	CCDS31023.1	chr1_214815908-214815908_G_T	1409E>D	Substitution	Nonsynonymous coding	33%
MM04T	CENPF	centromere protein F, 350/400kDa	CCDS31023.1	chr1_214818809-214818809_G_T	1966E>X	Substitution	Nonsense	33%
MM04T	CENPI	centromere protein I	CCDS14479.1	chrX_100364957-100364957_C_A	187F>L	Substitution	Nonsynonymous coding	35%
MM04T	CEP120	centrosomal protein 120kDa	CCDS4134.2	chr5_122720648-122720648_T_C	587Q>R	Substitution	Nonsynonymous coding	34%
MM04T	CEP128	centrosomal protein 128kDa	CCDS32130.1	chr14_80993262-80993262_G_T	1008S>Y	Substitution	Nonsynonymous coding	34%
MM04T	CEP128	centrosomal protein 128kDa	CCDS32130.1	chr14_81297518-81297518_T_C	393K>R	Substitution	Nonsynonymous coding	39%
MM04T	CEP128	centrosomal protein 128kDa	CCDS32130.1	chr14_81380701-81380701_C_A	67E>X	Substitution	Nonsense	15%
MM04T	CEP135	centrosomal protein 135kDa	CCDS33986.1	chr4_56847509-56847509_T_G	581I>M	Substitution	Nonsynonymous coding	46%
MM04T	CEP135	centrosomal protein 135kDa	CCDS33986.1	chr4_56878105-56878105_G_T	919R>I	Substitution	Nonsynonymous coding	17%
MM04T	CEP135	centrosomal protein 135kDa	CCDS33986.1	chr4_56831933-56831933_G_T	318E>X	Substitution	Nonsense	36%
MM04T	CEP135	centrosomal protein 135kDa	CCDS33986.1	chr4_56846345-56846345_G_T	504E>X	Substitution	Nonsense	24%
MM04T	CEP152	centrosomal protein 152kDa	CCDS42033.1	chr15_49089712-49089712_C_A	109R>I	Substitution	Nonsynonymous coding	35%
MM04T	CEP164	centrosomal protein 164kDa	CCDS31683.1	chr11_117222543-117222543_G_A	78A>T	Substitution	Nonsynonymous coding	24%

MM04T	CEP164	centrosomal protein 164kDa	CCDS31683.1	chr11_117263825-117263825_G_A	867E>K	Substitution	Nonsynonymous coding	37%
MM04T	CEP170P1	centrosomal protein 170kDa pseudogene 1	ENST00000412784	chr4_119461525-119461525_G_T	113R>I	Substitution	Nonsynonymous coding	27%
MM04T	CEP19	centrosomal protein 19kDa	CCDS43193.2	chr3_196435420-196435420_G_A	41R>X	Substitution	Nonsense	29%
MM04T	CEP350	centrosomal protein 350kDa	CCDS1336.1	chr1_179966044-179966044_C_T	251A>V	Substitution	Nonsynonymous coding	31%
MM04T	CEP350	centrosomal protein 350kDa	CCDS1336.1	chr1_180010297-180010297_G_T	1379D>Y	Substitution	Nonsynonymous coding	30%
MM04T	CER1	cerberus 1, cysteine knot superfamily, homolog (Xenopus laevis)	CCDS6476.1	chr9_14722585-14722585_G_A	29S>F	Substitution	Nonsynonymous coding	35%
MM04T	CERS2	ceramide synthase 2	CCDS973.1	chr1_150940661-150940661_A_G	103L>S	Substitution	Nonsynonymous coding	23%
MM04T	CETN1	centrin, EF-hand protein, 1	CCDS11820.1	chr18_580508-580508_C_T	34R>W	Substitution	Nonsynonymous coding	30%
MM04T	CFDP1	craniofacial development protein 1	CCDS10916.1	chr16_75338946-75338946_G_A	262A>V	Substitution	Nonsynonymous coding	29%
MM04T	CFDP1	craniofacial development protein 1	CCDS10916.1	chr16_75445788-75445788_C_A	155E>D	Substitution	Nonsynonymous coding	29%
MM04T	CFH	complement factor H	CCDS1385.1	chr1_196646674-196646674_C_T	166R>W	Substitution	Nonsynonymous coding	27%
MM04T	CFI	complement factor I	CCDS34049.1	chr4_110662266-110662266_C_T	512G>D	Substitution	Nonsynonymous coding	36%
MM04T	CFLAR	CASP8 and FADD-like apoptosis regulator	CCDS2337.1	chr2_202010107-202010107_A_C	205N>H	Substitution	Nonsynonymous coding	18%
MM04T	CFTR	cystic fibrosis transmembrane conductance regulator (ATP-binding cassette sub-family C,	CCDS5773.1	chr7_117232404-117232404_C_A	728S>Y	Substitution	Nonsynonymous coding	36%
MM04T	CFTR	cystic fibrosis transmembrane conductance regulator (ATP-binding cassette sub-family C,	CCDS5773.1	chr7_117232688-117232688_G_T	823E>X	Substitution	Nonsense	28%
MM04T	CGN	cingulin	CCDS999.1	chr1_151499465-151499465_G_A	593R>Q	Substitution	Nonsynonymous coding	29%
MM04T	CGN	cingulin	CCDS999.1	chr1_151504980-151504980_C_T	892R>C	Substitution	Nonsynonymous coding	40%
MM04T	CHD1	chromodomain helicase DNA binding protein 1	CCDS34204.1	chr5_98215270-98215270_T_G	1075N>H	Substitution	Nonsynonymous coding	40%
MM04T	CHD1L	chromodomain helicase DNA binding protein 1-like	CCDS927.1	chr1_146757128-146757128_A_C	661K>T	Substitution	Nonsynonymous coding	30%
MM04T	CHD5	chromodomain helicase DNA binding protein 5	CCDS57.1	chr1_6172981-6172981_C_T	1664E>K	Substitution	Nonsynonymous coding	17%
MM04T	CHD8	chromodomain helicase DNA binding protein 8	CCDS45081.1	chr14_21883122-21883122_C_T	388E>K	Substitution	Nonsynonymous coding	31%
MM04T	CHD9	chromodomain helicase DNA binding protein 9	CCDS45485.1	chr16_53260363-53260363_C_A	661S>Y	Substitution	Nonsynonymous coding	38%
MM04T	CHGB	chromogranin B (secretogranin 1)	CCDS13092.1	chr20_5903855-5903855_G_T	355E>D	Substitution	Nonsynonymous coding	24%
MM04T	CHI3L1	chitinase 3-like 1 (cartilage glycoprotein-39)	CCDS1435.1	chr1_203148971-203148971_C_A	310R>I	Substitution	Nonsynonymous coding	35%
MM04T	CHL1	cell adhesion molecule with homology to L1CAM (close homolog of L1)	CCDS2556.1	chr3_369886-369886_C_A	78F>L	Substitution	Nonsynonymous coding	29%
MM04T	CHL1	cell adhesion molecule with homology to L1CAM (close homolog of L1)	CCDS2556.1	chr3_391135-391135_G_T	314E>D	Substitution	Nonsynonymous coding	32%
MM04T	CHL1	cell adhesion molecule with homology to L1CAM (close homolog of L1)	CCDS2556.1	chr3_432722-432722_A_C	891N>H	Substitution	Nonsynonymous coding	12%
MM04T	CHM	choroideremia (Rab escort protein 1)	CCDS14454.1	chrX_85218880-85218880_C_A	164E>D	Substitution	Nonsynonymous coding	24%
MM04T	CHM	choroideremia (Rab escort protein 1)	CCDS14454.1	chrX_85212923-85212923_G_A	293R>X	Substitution	Nonsense	23%
MM04T	CHMP2B	charged multivesicular body protein 2B	CCDS2918.1	chr3_87294976-87294976_C_A	80S>X	Substitution	Nonsense	14%
MM04T	CHMP3	charged multivesicular body protein 3	CCDS33237.1	chr2_86847539-86847539_C_T	94E>K	Substitution	Nonsynonymous coding	33%
MM04T	CHMP4C	charged multivesicular body protein 4C	CCDS6233.1	chr8_82665399-82665399_G_T	97E>D	Substitution	Nonsynonymous coding	23%

MM04T	CHN2	chimerin 2	CCDS5420.1	chr7_29546951-29546951_G_T	367D>Y	Substitution	Nonsynonymous coding	13%
MM04T	CHRDL1	chordin-like 1	CCDS48149.1	chrX_109919523-109919523_C_A	438R>I	Substitution	Nonsynonymous coding	21%
MM04T	CHRDL2	chordin-like 2	CCDS8234.1	chr11_74408272-74408272_T_C	416T>A	Substitution	Nonsynonymous coding	42%
MM04T	CHRNA1	cholinergic receptor, nicotinic, alpha 1 (muscle)	CCDS33331.1	chr2_175614733-175614733_C_T	340V>I	Substitution	Nonsynonymous coding	29%
MM04T	CHRNA5	cholinergic receptor, nicotinic, alpha 5 (neuronal)	CCDS10304.1	chr15_78882291-78882291_G_T	186M>I	Substitution	Nonsynonymous coding	20%
MM04T	CHRNA5	cholinergic receptor, nicotinic, alpha 5 (neuronal)	CCDS10304.1	chr15_78882538-78882538_G_A	269V>I	Substitution	Nonsynonymous coding	31%
MM04T	CHRNB1	cholinergic receptor, nicotinic, beta 1 (muscle)	CCDS11106.1	chr17_7358619-7358619_T_G	354L>R	Substitution	Nonsynonymous coding	28%
MM04T	CHRNB4	cholinergic receptor, nicotinic, beta 4 (neuronal)	CCDS10306.1	chr15_78923451-78923451_C_T	109R>H	Substitution	Nonsynonymous coding	18%
MM04T	CHST10	carbohydrate sulfotransferase 10	CCDS2047.1	chr2_101010210-101010210_C_A	190D>Y	Substitution	Nonsynonymous coding	28%
MM04T	CHST14	carbohydrate (N-acetylgalactosamine 4-0) sulfotransferase 14	CCDS10059.1	chr15_40764038-40764038_T_G	209F>C	Substitution	Nonsynonymous coding	34%
MM04T	CHSY3	chondroitin sulfate synthase 3	CCDS34223.1	chr5_129520570-129520570_G_T	579D>Y	Substitution	Nonsynonymous coding	35%
MM04T	CHTOP	chromatin target of PRMT1	CCDS1048.1	chr1_153610824-153610824_C_T	40S>L	Substitution	Nonsynonymous coding	24%
MM04T	CHUK	conserved helix-loop-helix ubiquitous kinase	CCDS7488.1	chr10_101978817-101978817_C_A	213E>X	Substitution	Nonsense	30%
MM04T	CHURC1-FNTB	CHURC1-FNTB readthrough	CCDS9769.1	chr14_65507588-65507588_C_T	258A>V	Substitution	Nonsynonymous coding	23%
MM04T	CHURC1-FNTB	CHURC1-FNTB readthrough	CCDS9769.1	chr14_65511053-65511053_C_T	283R>X	Substitution	Nonsense	19%
MM04T	CILP	cartilage intermediate layer protein, nucleotide pyrophosphohydrolase	CCDS10203.1	chr15_65490910-65490910_G_A	572R>C	Substitution	Nonsynonymous coding	35%
MM04T	CILP2	cartilage intermediate layer protein 2	CCDS12405.1	chr19_19655276-19655276_G_A	641G>D	Substitution	Nonsynonymous coding	36%
MM04T	CIRH1A	cirrhosis, autosomal recessive 1A (cirhin)	CCDS10872.1	chr16_69188352-69188352_A_G	386K>E	Substitution	Nonsynonymous coding	26%
MM04T	CIZ1	CDKN1A interacting zinc finger protein 1	CCDS6894.1	chr9_130931738-130931738_G_A	698R>C	Substitution	Nonsynonymous coding	21%
MM04T	CKAP2L	cytoskeleton associated protein 2-like	CCDS2100.1	chr2_113496500-113496500_G_T	713S>Y	Substitution	Nonsynonymous coding	34%
MM04T	CKAP2L	cytoskeleton associated protein 2-like	CCDS2100.1	chr2_113509900-113509900_C_T	516E>K	Substitution	Nonsynonymous coding	34%
MM04T	CKAP5	cytoskeleton associated protein 5	CCDS31477.1	chr11_46773021-46773021_G_T	1733L>I	Substitution	Nonsynonymous coding	26%
MM04T	CKAP5	cytoskeleton associated protein 5	CCDS31477.1	chr11_46784660-46784660_A_G	1253F>L	Substitution	Nonsynonymous coding	21%
MM04T	CLASP2	cytoplasmic linker associated protein 2	NM_015097	chr3_33592757-33592757_G_T	1047S>Y	Substitution	Nonsynonymous coding	27%
MM04T	CLASP2	cytoplasmic linker associated protein 2	NM_015097	chr3_33612281-33612281_G_A	901R>X	Substitution	Nonsense	13%
MM04T	CLASRP	CLK4-associating serine/arginine rich protein	CCDS12652.2	chr19_45555366-45555366_G_A	46R>Q	Substitution	Nonsynonymous coding	25%
MM04T	CLCA1	chloride channel accessory 1	CCDS709.1	chr1_86951231-86951231_C_T	314S>F	Substitution	Nonsynonymous coding	18%
MM04T	CLCA2	chloride channel accessory 2	CCDS708.1	chr1_86900235-86900235_A_G	260Q>R	Substitution	Nonsynonymous coding	33%
MM04T	CLCA4	chloride channel accessory 4	CCDS41355.1	chr1_87041178-87041178_A_G	616E>G	Substitution	Nonsynonymous coding	20%
MM04T	CLCN5	chloride channel, voltage-sensitive 5	CCDS48115.1	chrX_49855395-49855395_C_T	738P>S	Substitution	Nonsynonymous coding	24%
MM04T	CLCN5	chloride channel, voltage-sensitive 5	CCDS48115.1	chrX_49855431-49855431_C_T	750R>W	Substitution	Nonsynonymous coding	24%
MM04T	CLCN7	chloride channel, voltage-sensitive 7	CCDS32361.1	chr16_1505209-1505209_G_T	342L>M	Substitution	Nonsynonymous coding	15%

MM04T	CLDN10	claudin 10	CCDS9476.1	chr13_96212438-96212438_C_A	91F>L	Substitution	Nonsynonymous coding	19%
MM04T	CLEC16A	C-type lectin domain family 16, member A	ENST00000428742	chr16_11250936-11250936_T_G	150F>V	Substitution	Nonsynonymous coding	36%
MM04T	CLEC1A	C-type lectin domain family 1, member A	CCDS8612.1	chr12_10225898-10225898_G_T	219S>Y	Substitution	Nonsynonymous coding	21%
MM04T	CLEC7A	C-type lectin domain family 7, member A	CCDS41753.1	chr12_10277925-10277925_G_T	155L>I	Substitution	Nonsynonymous coding	35%
MM04T	CLEC7A	C-type lectin domain family 7, member A	CCDS41753.1	chr12_10271190-10271190_C_T	NA	Substitution	Splice site acceptor	31%
MM04T	CLECL1	C-type lectin-like 1	CCDS8603.1	chr12_9885661-9885661_G_T	67S>X	Substitution	Nonsense	29%
MM04T	CLGN	calmegin	CCDS3751.1	chr4_141317282-141317282_A_C	321F>C	Substitution	Nonsynonymous coding	33%
MM04T	CLHC1	clathrin heavy chain linker domain containing 1	CCDS33201.1	chr2_55439929-55439929_C_T	127E>K	Substitution	Nonsynonymous coding	36%
MM04T	CLHC1	clathrin heavy chain linker domain containing 1	CCDS33201.1	chr2_55449462-55449462_C_A	29R>I	Substitution	Nonsynonymous coding	21%
MM04T	CLIC2	chloride intracellular channel 2	CCDS14767.1	chrX_154508596-154508596_C_A	142E>X	Substitution	Nonsense	31%
MM04T	CLIC4	chloride intracellular channel 4	CCDS256.1	chr1_25167277-25167277_A_C	204K>T	Substitution	Nonsynonymous coding	33%
MM04T	CLIC5	chloride intracellular channel 5	CCDS47438.1	chr6_45909341-45909341_C_T	276D>N	Substitution	Nonsynonymous coding	31%
MM04T	CLIP1	CAP-GLY domain containing linker protein 1	CCDS9232.1	chr12_122825734-122825734_C_T	662E>K	Substitution	Nonsynonymous coding	27%
MM04T	CLIP4	CAP-GLY domain containing linker protein family, member 4	CCDS1770.1	chr2_29375573-29375573_G_T	348K>N	Substitution	Nonsynonymous coding	30%
MM04T	CLK4	CDC-like kinase 4	CCDS4437.1	chr5_178030676-178030676_C_A	463R>I	Substitution	Nonsynonymous coding	26%
MM04T	CLN5	ceroid-lipofuscinosis, neuronal 5	CCDS9456.1	chr13_77574962-77574962_T_G	361F>C	Substitution	Nonsynonymous coding	32%
MM04T	CLNK	cytokine-dependent hematopoietic cell linker	CCDS47024.1	chr4_10542140-10542140_G_T	194P>T	Substitution	Nonsynonymous coding	16%
MM04T	CLNK	cytokine-dependent hematopoietic cell linker	CCDS47024.1	chr4_10599665-10599665_C_T	7R>K	Substitution	Nonsynonymous coding	29%
MM04T	CLRN3	clarin 3	CCDS7656.1	chr10_129676622-129676622_C_T	158E>K	Substitution	Nonsynonymous coding	21%
MM04T	CLSTN1	calsyntenin 1	CCDS30580.1	chr1_9793473-9793473_C_T	805E>K	Substitution	Nonsynonymous coding	19%
MM04T	CLSTN3	calsyntenin 3	ENST00000331148	chr12_7309210-7309210_G_A	80V>M	Substitution	Nonsynonymous coding	35%
MM04T	CLUH	clustered mitochondria (cluA/CLU1) homolog	CCDS45572.1	chr17_2600207-2600207_A_G	626L>S	Substitution	Nonsynonymous coding	35%
MM04T	CLUL1	clusterin-like 1 (retinal)	CCDS42405.1	chr18_627130-627130_C_A	153L>I	Substitution	Nonsynonymous coding	21%
MM04T	CMAHP	cytidine monophospho-N-acetylneuraminic acid hydroxylase, pseudogene	ENST00000377989	chr6_25113968-25113968_C_A	49D>Y	Substitution	Nonsynonymous coding	21%
MM04T	CMTM4	CKLF-like MARVEL transmembrane domain containing 4	CCDS10817.1	chr16_66656125-66656125_T_C	155I>V	Substitution	Nonsynonymous coding	27%
MM04T	CMTM5	CKLF-like MARVEL transmembrane domain containing 5	CCDS9598.1	chr14_23847691-23847691_G_A	87R>Q	Substitution	Nonsynonymous coding	30%
MM04T	CMYA5	cardiomyopathy associated 5	CCDS47238.1	chr5_79031858-79031858_T_G	2424F>V	Substitution	Nonsynonymous coding	30%
MM04T	CNBD1	cyclic nucleotide binding domain containing 1	NM_173538	chr8_88218342-88218342_G_A	185E>K	Substitution	Nonsynonymous coding	32%
MM04T	CNBD2	cyclic nucleotide binding domain containing 2	CCDS13270.1	chr20_34618470-34618470_G_A	540R>H	Substitution	Nonsynonymous coding	33%
MM04T	CNDP1	carnosine dipeptidase 1 (metallopeptidase M20 family)	CCDS12007.1	chr18_72238464-72238464_G_A	267G>D	Substitution	Nonsynonymous coding	35%
MM04T	CNDP2	CNDP dipeptidase 2 (metallopeptidase M20 family)	CCDS12006.1	chr18_72180898-72180898_G_T	283D>Y	Substitution	Nonsynonymous coding	28%
MM04T	CNGA1	cyclic nucleotide gated channel alpha 1	CCDS47050.1	chr4_47938583-47938583_C_T	712R>Q	Substitution	Nonsynonymous coding	33%

MM04T	CNGB1	cyclic nucleotide gated channel beta 1	CCDS42169.1	chr16_57998442-57998442_C_T	56E>K	Substitution	Nonsynonymous coding	39%
MM04T	CNKSR1	connector enhancer of kinase suppressor of Ras 1	CCDS276.1	chr1_26507030-26507030_G_A	47E>K	Substitution	Nonsynonymous coding	30%
MM04T	CNKSR2	connector enhancer of kinase suppressor of Ras 2	CCDS14198.1	chrX_21609167-21609167_G_T	562R>L	Substitution	Nonsynonymous coding	23%
MM04T	CNKSR2	connector enhancer of kinase suppressor of Ras 2	CCDS14198.1	chrX_21627729-21627729_G_A	896E>K	Substitution	Nonsynonymous coding	33%
MM04T	CNNM2	cyclin M2	CCDS44474.1	chr10_104835993-104835993_C_T	795S>L	Substitution	Nonsynonymous coding	39%
MM04T	CNNM3	cyclin M3	CCDS2025.1	chr2_97492600-97492600_C_A	467S>Y	Substitution	Nonsynonymous coding	30%
MM04T	CNOT4	CCR4-NOT transcription complex, subunit 4	CCDS43650.1	chr7_135078728-135078728_G_T	520F>L	Substitution	Nonsynonymous coding	25%
MM04T	CNPY1	canopy 1 homolog (zebrafish)	CCDS43684.1	chr7_155301653-155301653_T_C	27K>R	Substitution	Nonsynonymous coding	31%
MM04T	CNPY4	canopy 4 homolog (zebrafish)	CCDS34701.1	chr7_99720131-99720131_G_T	91E>D	Substitution	Nonsynonymous coding	28%
MM04T	CNR1	cannabinoid receptor 1 (brain)	CCDS5015.1	chr6_88854162-88854162_A_C	278F>V	Substitution	Nonsynonymous coding	38%
MM04T	CNTD1	cyclin N-terminal domain containing 1	CCDS11440.1	chr17_40956407-40956407_G_A	137R>Q	Substitution	Nonsynonymous coding	26%
MM04T	CNTLN	centlein, centrosomal protein	CCDS43789.1	chr9_17366712-17366712_G_T	662E>X	Substitution	Nonsense	20%
MM04T	CNTN1	contactin 1	CCDS8737.1	chr12_41333215-41333215_G_T	436C>F	Substitution	Nonsynonymous coding	37%
MM04T	CNTN2	contactin 2 (axonal)	CCDS1449.1	chr1_205034232-205034232_C_A	513L>I	Substitution	Nonsynonymous coding	22%
MM04T	CNTN5	contactin 5	NM_014361	chr11_99690327-99690327_A_C	36R>S	Substitution	Nonsynonymous coding	27%
MM04T	CNTN6	contactin 6	CCDS2557.1	chr3_1445027-1445027_C_A	1004F>L	Substitution	Nonsynonymous coding	24%
MM04T	CNTNAP1	contactin associated protein 1	CCDS11436.1	chr17_40849644-40849644_G_A	1214R>Q	Substitution	Nonsynonymous coding	30%
MM04T	CNTNAP2	contactin associated protein-like 2	CCDS5889.1	chr7_147259350-147259350_G_A	NA	Substitution	Splice site donor	32%
MM04T	CNTNAP4	contactin associated protein-like 4	NM_138994	chr16_76350369-76350369_G_A	24E>K	Substitution	Nonsynonymous coding	10%
MM04T	CNTNAP4	contactin associated protein-like 4	NM_138994	chr16_76555192-76555192_C_T	768R>W	Substitution	Nonsynonymous coding	23%
MM04T	CNTNAP5	contactin associated protein-like 5	CCDS46401.1	chr2_125282006-125282006_C_A	484S>Y	Substitution	Nonsynonymous coding	33%
MM04T	CNTNAP5	contactin associated protein-like 5	CCDS46401.1	chr2_125405342-125405342_G_T	627K>N	Substitution	Nonsynonymous coding	24%
MM04T	CNTNAP5	contactin associated protein-like 5	CCDS46401.1	chr2_125530547-125530547_C_T	901S>L	Substitution	Nonsynonymous coding	29%
MM04T	COBL	cordon-bleu WH2 repeat protein	CCDS34637.1	chr7_51287451-51287451_C_T	78V>M	Substitution	Nonsynonymous coding	28%
MM04T	COCH	coagulation factor C homolog, cochlin (Limulus polyphemus)	CCDS9640.1	chr14_31344155-31344155_C_T	4A>V	Substitution	Nonsynonymous coding	31%
MM04T	COCH	coagulation factor C homolog, cochlin (Limulus polyphemus)	CCDS9640.1	chr14_31355442-31355442_G_T	467K>N	Substitution	Nonsynonymous coding	28%
MM04T	COL10A1	collagen, type X, alpha 1	CCDS5105.1	chr6_116441561-116441561_T_G	573K>T	Substitution	Nonsynonymous coding	27%
MM04T	COL11A1	collagen, type XI, alpha 1	CCDS779.1	chr1_103343623-103343623_C_A	1803Q>H	Substitution	Nonsynonymous coding	24%
MM04T	COL11A1	collagen, type XI, alpha 1	CCDS779.1	chr1_103412409-103412409_G_T	1103A>D	Substitution	Nonsynonymous coding	33%
MM04T	COL11A1	collagen, type XI, alpha 1	CCDS779.1	chr1_103463886-103463886_G_A	738P>S	Substitution	Nonsynonymous coding	28%
MM04T	COL12A1	collagen, type XII, alpha 1	CCDS43482.1	chr6_75848594-75848594_C_T	1681D>N	Substitution	Nonsynonymous coding	21%
MM04T	COL12A1	collagen, type XII, alpha 1	CCDS43482.1	chr6_75801203-75801203_T_C	NA	Substitution	Splice site donor	39%

MM04T	COL13A1	collagen, type XIII, alpha 1	CCDS44419.1	chr10_71562476-71562476_C_A	NA	Substitution	Splice site donor	15%
MM04T	COL14A1	collagen, type XIV, alpha 1	CCDS34938.1	chr8_121216025-121216025_G_A	319E>K	Substitution	Nonsynonymous coding	34%
MM04T	COL22A1	collagen, type XXII, alpha 1	CCDS6376.1	chr8_139890361-139890361_G_A	97S>L	Substitution	Nonsynonymous coding	16%
MM04T	COL22A1	collagen, type XXII, alpha 1	CCDS6376.1	chr8_139618686-139618686_C_A	1348G>X	Substitution	Nonsense	23%
MM04T	COL23A1	collagen, type XXIII, alpha 1	CCDS4436.1	chr5_177688753-177688753_G_A	NA	Substitution	Splice site acceptor	22%
MM04T	COL23A1	collagen, type XXIII, alpha 1	CCDS4436.1	chr5_177677059-177677059_G_T	NA	Substitution	Splice site donor	20%
MM04T	COL24A1	collagen, type XXIV, alpha 1	CCDS41353.1	chr1_86196234-86196234_G_T	1714L>M	Substitution	Nonsynonymous coding	11%
MM04T	COL27A1	collagen, type XXVII, alpha 1	CCDS6802.1	chr9_116972007-116972007_G_T	774R>L	Substitution	Nonsynonymous coding	18%
MM04T	COL28A1	collagen, type XXVIII, alpha 1	CCDS43553.1	chr7_7398346-7398346_C_T	1099R>Q	Substitution	Nonsynonymous coding	23%
MM04T	COL3A1	collagen, type III, alpha 1	CCDS2297.1	chr2_189857638-189857638_C_T	341A>V	Substitution	Nonsynonymous coding	24%
MM04T	COL4A3BP	collagen, type IV, alpha 3 (Goodpasture antigen) binding protein	CCDS47235.1	chr5_74754985-74754985_G_A	213R>X	Substitution	Nonsense	27%
MM04T	COL4A4	collagen, type IV, alpha 4	CCDS42828.1	chr2_228004894-228004894_G_A	59P>S	Substitution	Nonsynonymous coding	32%
MM04T	COL4A5	collagen, type IV, alpha 5	CCDS35366.1	chrX_107826148-107826148_C_T	324P>L	Substitution	Nonsynonymous coding	15%
MM04T	COL4A6	collagen, type IV, alpha 6	CCDS14541.1	chrX_107446215-107446215_G_A	NA	Substitution	Splice site acceptor	30%
MM04T	COL5A3	collagen, type V, alpha 3	CCDS12222.1	chr19_10108798-10108798_C_A	380E>X	Substitution	Nonsense	28%
MM04T	COL6A1	collagen, type VI, alpha 1	CCDS13727.1	chr21_47421891-47421891_C_T	658S>L	Substitution	Nonsynonymous coding	28%
MM04T	COL6A2	collagen, type VI, alpha 2	CCDS13728.1	chr21_47552261-47552261_C_T	952T>M	Substitution	Nonsynonymous coding	31%
MM04T	COL6A3	collagen, type VI, alpha 3	CCDS33412.1	chr2_238265984-238265984_C_A	2196K>N	Substitution	Nonsynonymous coding	25%
MM04T	COL6A3	collagen, type VI, alpha 3	CCDS33412.1	chr2_238277271-238277271_G_A	1612S>L	Substitution	Nonsynonymous coding	29%
MM04T	COL6A5	collagen, type VI, alpha 5	NM_153264	chr3_130159057-130159057_G_T	1959D>Y	Substitution	Nonsynonymous coding	20%
MM04T	COL6A6	collagen, type VI, alpha 6	CCDS46911.1	chr3_130292929-130292929_G_T	1036R>I	Substitution	Nonsynonymous coding	19%
MM04T	COL6A6	collagen, type VI, alpha 6	CCDS46911.1	chr3_130300451-130300451_G_T	1198E>D	Substitution	Nonsynonymous coding	25%
MM04T	COL6A6	collagen, type VI, alpha 6	CCDS46911.1	chr3_130325771-130325771_T_G	NA	Substitution	Splice site acceptor	32%
MM04T	COL6A6	collagen, type VI, alpha 6	CCDS46911.1	chr3_130313176-130313176_C_T	1508R>X	Substitution	Nonsense	15%
MM04T	COL7A1	collagen, type VII, alpha 1	CCDS2773.1	chr3_48611705-48611705_T_G	2164E>A	Substitution	Nonsynonymous coding	19%
MM04T	COL7A1	collagen, type VII, alpha 1	CCDS2773.1	chr3_48619347-48619347_T_G	1555E>D	Substitution	Nonsynonymous coding	27%
MM04T	COL9A1	collagen, type IX, alpha 1	CCDS4971.1	chr6_70944265-70944265_T_G	764Q>P	Substitution	Nonsynonymous coding	36%
MM04T	COLEC12	collectin sub-family member 12	CCDS32782.1	chr18_347250-347250_C_A	124E>D	Substitution	Nonsynonymous coding	31%
MM04T	COLEC12	collectin sub-family member 12	CCDS32782.1	chr18_357423-357423_G_A	53T>I	Substitution	Nonsynonymous coding	18%
MM04T	COPG2	coatomer protein complex, subunit gamma 2	ENST00000445977	chr7_130301863-130301863_C_T	133D>N	Substitution	Nonsynonymous coding	24%
MM04T	COPZ1	coatomer protein complex, subunit zeta 1	CCDS8877.1	chr12_54736055-54736055_G_T	51K>N	Substitution	Nonsynonymous coding	23%
MM04T	COQ6	coenzyme Q6 homolog, monooxygenase (S. cerevisiae)	CCDS9823.1	chr14_74420205-74420205_G_T	77E>D	Substitution	Nonsynonymous coding	32%

MM04T	CORO1C	coronin, actin binding protein, 1C	CCDS9120.1	chr12_109042747-109042747_G_A	351P>S	Substitution	Nonsynonymous coding	23%
MM04T	CORO1C	coronin, actin binding protein, 1C	CCDS9120.1	chr12_109051189-109051189_T_G	214K>T	Substitution	Nonsynonymous coding	28%
MM04T	CORO1C	coronin, actin binding protein, 1C	CCDS9120.1	chr12_109072107-109072107_C_A	87D>Y	Substitution	Nonsynonymous coding	22%
MM04T	CORO1C	coronin, actin binding protein, 1C	CCDS9120.1	chr12_109072163-109072163_C_T	68R>Q	Substitution	Nonsynonymous coding	33%
MM04T	CORO2B	coronin, actin binding protein, 2B	NM_006091	chr15_69011489-69011489_T_C	363S>P	Substitution	Nonsynonymous coding	13%
MM04T	CORO2B	coronin, actin binding protein, 2B	NM_006091	chr15_69018188-69018188_C_T	440R>X	Substitution	Nonsense	26%
MM04T	CP	ceruloplasmin (ferroxidase)	CCDS3141.1	chr3_148925273-148925273_T_G	305T>P	Substitution	Nonsynonymous coding	24%
MM04T	CPA2	carboxypeptidase A2 (pancreatic)	CCDS5817.2	chr7_129909638-129909638_G_A	95V>M	Substitution	Nonsynonymous coding	40%
MM04T	CPA3	carboxypeptidase A3 (mast cell)	CCDS3138.1	chr3_148601574-148601574_C_A	318S>X	Substitution	Nonsense	33%
MM04T	CPAMD8	C3 and PZP-like, alpha-2-macroglobulin domain containing 8	CCDS42519.1	chr19_17120123-17120123_G_T	212A>D	Substitution	Nonsynonymous coding	18%
MM04T	CPED1	cadherin-like and PC-esterase domain containing 1	CCDS34739.1	chr7_120935617-120935617_C_T	998R>X	Substitution	Nonsense	26%
MM04T	CPNE3	copine III	CCDS6243.1	chr8_87544794-87544794_A_C	149K>Q	Substitution	Nonsynonymous coding	21%
MM04T	CPNE3	copine III	CCDS6243.1	chr8_87560543-87560543_C_A	298F>L	Substitution	Nonsynonymous coding	26%
MM04T	CPNE4	copine IV	CCDS3072.1	chr3_131254061-131254061_G_T	551S>Y	Substitution	Nonsynonymous coding	33%
MM04T	CPNE8	copine VIII	CCDS8733.1	chr12_39242357-39242357_T_G	NA	Substitution	Splice site donor	47%
MM04T	CPOX	coproporphyrinogen oxidase	CCDS2932.1	chr3_98304474-98304474_C_T	328R>H	Substitution	Nonsynonymous coding	32%
MM04T	CPQ	carboxypeptidase Q	CCDS6273.1	chr8_97978196-97978196_G_A	295D>N	Substitution	Nonsynonymous coding	19%
MM04T	CPS1	carbamoyl-phosphate synthase 1, mitochondrial	CCDS46505.1	chr2_211455554-211455554_T_C	297F>L	Substitution	Nonsynonymous coding	29%
MM04T	CPS1	carbamoyl-phosphate synthase 1, mitochondrial	CCDS46505.1	chr2_211465409-211465409_G_T	566K>N	Substitution	Nonsynonymous coding	27%
MM04T	CPS1	carbamoyl-phosphate synthase 1, mitochondrial	CCDS46505.1	chr2_211521333-211521333_A_G	1221I>V	Substitution	Nonsynonymous coding	23%
MM04T	CPS1	carbamoyl-phosphate synthase 1, mitochondrial	CCDS46505.1	chr2_211476973-211476973_G_T	848E>X	Substitution	Nonsense	33%
MM04T	CPSF2	cleavage and polyadenylation specific factor 2, 100kDa	CCDS9902.1	chr14_92627992-92627992_C_A	NA	Substitution	Splice site acceptor	34%
MM04T	CPSF4	cleavage and polyadenylation specific factor 4, 30kDa	CCDS5664.1	chr7_99042437-99042437_C_A	43F>L	Substitution	Nonsynonymous coding	36%
MM04T	CPSF6	cleavage and polyadenylation specific factor 6, 68kDa	CCDS8988.1	chr12_69653297-69653297_C_T	430S>L	Substitution	Nonsynonymous coding	30%
MM04T	CPSF7	cleavage and polyadenylation specific factor 7, 59kDa	CCDS8006.2	chr11_61188974-61188974_G_T	97P>H	Substitution	Nonsynonymous coding	27%
MM04T	CPT1A	carnitine palmitoyltransferase 1A (liver)	CCDS8185.1	chr11_68525188-68525188_C_T	749R>H	Substitution	Nonsynonymous coding	32%
MM04T	CPT1B	carnitine palmitoyltransferase 1B (muscle)	CCDS14098.1	chr22_51015399-51015399_C_T	116V>I	Substitution	Nonsynonymous coding	25%
MM04T	CPVL	carboxypeptidase, vitellogenic-like	CCDS5419.1	chr7_29134713-29134713_T_C	150Y>C	Substitution	Nonsynonymous coding	27%
MM04T	CPXCR1	CPX chromosome region, candidate 1	CCDS14458.1	chrX_88008898-88008898_C_A	161F>L	Substitution	Nonsynonymous coding	28%
MM04T	CPXM2	carboxypeptidase X (M14 family), member 2	CCDS7637.1	chr10_125521676-125521676_C_T	497E>K	Substitution	Nonsynonymous coding	19%
MM04T	CR1	complement component (3b/4b) receptor 1 (Knops blood group)	CCDS44308.1	chr1_207782641-207782641_C_A	1968S>Y	Substitution	Nonsynonymous coding	33%
MM04T	CR1L	complement component (3b/4b) receptor 1-like	CCDS44310.1	chr1_207851630-207851630_C_A	122S>Y	Substitution	Nonsynonymous coding	15%

MM04T	CR2	complement component (3d/Epstein Barr virus) receptor 2	CCDS31007.1	chr1_207643042-207643042_A_T	274I>F	Substitution	Nonsynonymous coding	20%
MM04T	CRB1	crumbs homolog 1 (Drosophila)	CCDS1390.1	chr1_197396899-197396899_G_A	815G>E	Substitution	Nonsynonymous coding	41%
MM04T	CRB1	crumbs homolog 1 (Drosophila)	CCDS1390.1	chr1_197404628-197404628_A_G	1212E>G	Substitution	Nonsynonymous coding	37%
MM04T	CRB1	crumbs homolog 1 (Drosophila)	CCDS1390.1	chr1_197404724-197404724_T_G	1244F>C	Substitution	Nonsynonymous coding	37%
MM04T	CRB2	crumbs homolog 2 (Drosophila)	CCDS6852.2	chr9_126133712-126133712_G_A	764R>Q	Substitution	Nonsynonymous coding	18%
MM04T	CRELD1	cysteine-rich with EGF-like domains 1	CCDS2593.1	chr3_9986110-9986110_C_A	370F>L	Substitution	Nonsynonymous coding	15%
MM04T	CRHR1	corticotropin releasing hormone receptor 1	CCDS45712.1	chr17_43911378-43911378_C_A	386F>L	Substitution	Nonsynonymous coding	26%
MM04T	CRNKL1	crooked neck pre-mRNA splicing factor-like 1 (Drosophila)	CCDS33446.1	chr20_20030037-20030037_A_C	242I>M	Substitution	Nonsynonymous coding	31%
MM04T	CRNKL1	crooked neck pre-mRNA splicing factor-like 1 (Drosophila)	CCDS33446.1	chr20_20031218-20031218_A_C	195L>V	Substitution	Nonsynonymous coding	43%
MM04T	CRTAP	cartilage associated protein	CCDS2657.1	chr3_33174060-33174060_G_T	312K>N	Substitution	Nonsynonymous coding	25%
MM04T	CRY2	cryptochrome 2 (photolyase-like)	CCDS7915.2	chr11_45891090-45891090_T_G	327F>V	Substitution	Nonsynonymous coding	23%
MM04T	CRYBA4	crystallin, beta A4	CCDS13841.1	chr22_27024290-27024290_G_T	113E>D	Substitution	Nonsynonymous coding	19%
MM04T	CSDE1	cold shock domain containing E1, RNA-binding	CCDS44197.1	chr1_115267948-115267948_G_T	564F>L	Substitution	Nonsynonymous coding	22%
MM04T	CSF1R	colony stimulating factor 1 receptor	CCDS4302.1	chr5_149457758-149457758_G_A	216R>X	Substitution	Nonsense	32%
MM04T	CSGALNACT1	chondroitin sulfate N-acetylgalactosaminyltransferase 1	CCDS6010.1	chr8_19316124-19316124_T_C	222T>A	Substitution	Nonsynonymous coding	33%
MM04T	CSMD2	CUB and Sushi multiple domains 2	CCDS380.1	chr1_34003202-34003202_G_T	3069F>L	Substitution	Nonsynonymous coding	31%
MM04T	CSMD2	CUB and Sushi multiple domains 2	CCDS380.1	chr1_34083155-34083155_G_A	1924R>C	Substitution	Nonsynonymous coding	29%
MM04T	CSMD2	CUB and Sushi multiple domains 2	CCDS380.1	chr1_34100838-34100838_A_C	1648F>V	Substitution	Nonsynonymous coding	25%
MM04T	CSMD2	CUB and Sushi multiple domains 2	CCDS380.1	chr1_34181930-34181930_G_T	1018S>Y	Substitution	Nonsynonymous coding	25%
MM04T	CSMD3	CUB and Sushi multiple domains 3	CCDS6315.1	chr8_113293531-113293531_C_T	3127R>Q	Substitution	Nonsynonymous coding	40%
MM04T	CSMD3	CUB and Sushi multiple domains 3	CCDS6315.1	chr8_113331084-113331084_C_T	2448A>T	Substitution	Nonsynonymous coding	30%
MM04T	CSMD3	CUB and Sushi multiple domains 3	CCDS6315.1	chr8_113585840-113585840_G_A	1311T>I	Substitution	Nonsynonymous coding	24%
MM04T	CSMD3	CUB and Sushi multiple domains 3	CCDS6315.1	chr8_113657379-113657379_G_T	1090S>Y	Substitution	Nonsynonymous coding	33%
MM04T	CSMD3	CUB and Sushi multiple domains 3	CCDS6315.1	chr8_113649235-113649235_C_A	NA	Substitution	Splice site acceptor	27%
MM04T	CSMD3	CUB and Sushi multiple domains 3	CCDS6315.1	chr8_113353841-113353841_C_A	2173E>X	Substitution	Nonsense	34%
MM04T	CSN3	casein kappa	CCDS3538.1	chr4_71115153-71115153_G_A	176A>T	Substitution	Nonsynonymous coding	28%
MM04T	CSNK1G1	casein kinase 1, gamma 1	CCDS10192.2	chr15_64543750-64543750_G_T	85H>N	Substitution	Nonsynonymous coding	35%
MM04T	CSRNP2	cysteine-serine-rich nuclear protein 2	CCDS8807.1	chr12_51458222-51458222_G_T	313F>L	Substitution	Nonsynonymous coding	22%
MM04T	CSRNP3	cysteine-serine-rich nuclear protein 3	CCDS2225.1	chr2_166535733-166535733_C_A	410L>I	Substitution	Nonsynonymous coding	21%
MM04T	CSRP2BP	CSRP2 binding protein	CCDS13133.1	chr20_18139878-18139878_G_T	217E>D	Substitution	Nonsynonymous coding	15%
MM04T	CST5	cystatin D	CCDS13162.1	chr20_23856835-23856835_C_T	140R>Q	Substitution	Nonsynonymous coding	14%
MM04T	CSTF2	cleavage stimulation factor, 3' pre-RNA, subunit 2, 64kDa	CCDS14473.1	chrX_100088421-100088421_C_A	487P>Q	Substitution	Nonsynonymous coding	33%

MM04T	CSTL1	cystatin-like 1	CCDS13153.1	chr20_23425407-23425407_G_T	NA	Substitution	Splice site acceptor	22%
MM04T	CTCF	CCCTC-binding factor (zinc finger protein)	CCDS10841.1	chr16_67650677-67650677_G_A	328D>N	Substitution	Nonsynonymous coding	39%
MM04T	CTCFL	CCCTC-binding factor (zinc finger protein)-like	CCDS13459.1	chr20_56083819-56083819_C_T	506R>H	Substitution	Nonsynonymous coding	24%
MM04T	CTCFL	CCCTC-binding factor (zinc finger protein)-like	CCDS13459.1	chr20_56099204-56099204_C_T	20E>K	Substitution	Nonsynonymous coding	35%
MM04T	CTD-2116F7.1	-	ENST00000397549	chr17_77681179-77681179_C_T	35R>C	Substitution	Nonsynonymous coding	28%
MM04T	CTNNA2	catenin (cadherin-associated protein), alpha 2	ENST00000343114	chr2_80540750-80540750_T_C	22S>P	Substitution	Nonsynonymous coding	41%
MM04T	CTNNAL1	catenin (cadherin-associated protein), alpha-like 1	CCDS6775.1	chr9_111727805-111727805_T_C	482I>V	Substitution	Nonsynonymous coding	12%
MM04T	CTNNBL1	catenin, beta like 1	CCDS13298.1	chr20_36365852-36365852_G_T	98E>X	Substitution	Nonsense	29%
MM04T	CTPS2	CTP synthase 2	CCDS14175.1	chrX_16717187-16717187_C_A	66E>X	Substitution	Nonsense	35%
MM04T	CTSC	cathepsin C	CCDS8282.1	chr11_88033775-88033775_T_G	227K>T	Substitution	Nonsynonymous coding	18%
MM04T	CTSL2	cathepsin L2	CCDS6723.1	chr9_99799643-99799643_C_T	96R>Q	Substitution	Nonsynonymous coding	29%
MM04T	CTTNBP2NL	CTTNBP2 N-terminal like	CCDS845.1	chr1_112999126-112999126_C_T	338P>S	Substitution	Nonsynonymous coding	24%
MM04T	CUBN	cubilin (intrinsic factor-cobalamin receptor)	CCDS7113.1	chr10_16877155-16877155_C_A	3407R>I	Substitution	Nonsynonymous coding	25%
MM04T	CUBN	cubilin (intrinsic factor-cobalamin receptor)	CCDS7113.1	chr10_16916380-16916380_C_T	3077E>K	Substitution	Nonsynonymous coding	35%
MM04T	CUBN	cubilin (intrinsic factor-cobalamin receptor)	CCDS7113.1	chr10_16946010-16946010_C_T	2673E>K	Substitution	Nonsynonymous coding	40%
MM04T	CUBN	cubilin (intrinsic factor-cobalamin receptor)	CCDS7113.1	chr10_17026263-17026263_C_T	1456D>N	Substitution	Nonsynonymous coding	32%
MM04T	CUBN	cubilin (intrinsic factor-cobalamin receptor)	CCDS7113.1	chr10_17153023-17153023_C_T	304E>K	Substitution	Nonsynonymous coding	32%
MM04T	CUL4B	cullin 4B	CCDS35379.1	chrX_119674251-119674251_C_A	555R>I	Substitution	Nonsynonymous coding	36%
MM04T	CUL5	cullin 5	CCDS31668.1	chr11_107968435-107968435_T_G	660F>V	Substitution	Nonsynonymous coding	17%
MM04T	CUL7	cullin 7	CCDS4881.1	chr6_43017401-43017401_C_A	NA	Substitution	Splice site acceptor	28%
MM04T	CUX2	cut-like homeobox 2	CCDS41837.1	chr12_111749912-111749912_A_G	637T>A	Substitution	Nonsynonymous coding	24%
MM04T	CUZD1	CUB and zona pellucida-like domains 1	CCDS7631.1	chr10_124593209-124593209_G_A	544R>X	Substitution	Nonsense	34%
MM04T	CWC25	CWC25 spliceosome-associated protein homolog (S. cerevisiae)	CCDS45663.1	chr17_36958958-36958958_G_T	386F>L	Substitution	Nonsynonymous coding	23%
MM04T	CWH43	cell wall biogenesis 43 C-terminal homolog (S. cerevisiae)	CCDS3486.1	chr4_49032943-49032943_G_T	492D>Y	Substitution	Nonsynonymous coding	34%
MM04T	CWH43	cell wall biogenesis 43 C-terminal homolog (S. cerevisiae)	CCDS3486.1	chr4_49005925-49005925_G_T	326E>X	Substitution	Nonsense	27%
MM04T	CXCR1	chemokine (C-X-C motif) receptor 1	CCDS2409.1	chr2_219029355-219029355_C_A	194D>Y	Substitution	Nonsynonymous coding	17%
MM04T	CXorf21	chromosome X open reading frame 21	CCDS14224.1	chrX_30577684-30577684_C_A	263E>D	Substitution	Nonsynonymous coding	21%
MM04T	CXorf22	chromosome X open reading frame 22	CCDS14237.2	chrX_35989861-35989861_C_T	710S>F	Substitution	Nonsynonymous coding	27%
MM04T	CXorf30	chromosome X open reading frame 30	ENST00000378653	chrX_36298626-36298626_G_A	221R>K	Substitution	Nonsynonymous coding	18%
MM04T	CXorf30	chromosome X open reading frame 30	ENST00000378653	chrX_36298629-36298629_G_T	222R>I	Substitution	Nonsynonymous coding	17%
MM04T	CXorf30	chromosome X open reading frame 30	NM_001098843	chrX_36402918-36402918_G_T	567E>X	Substitution	Nonsense	31%
MM04T	CXorf36	chromosome X open reading frame 36	CCDS48096.1	chrX_45059953-45059953_A_C	40L>R	Substitution	Nonsynonymous coding	24%

MM04T	CXorf38	chromosome X open reading frame 38	CCDS14253.1	chrX_40489999-40489999_C_A	276K>N	Substitution	Nonsynonymous coding	21%
MM04T	CXorf38	chromosome X open reading frame 38	CCDS14253.1	chrX_40490015-40490015_C_T	271R>Q	Substitution	Nonsynonymous coding	29%
MM04T	CXorf56	chromosome X open reading frame 56	CCDS14579.1	chrX_118675357-118675357_C_A	180Q>H	Substitution	Nonsynonymous coding	31%
MM04T	CXorf57	chromosome X open reading frame 57	CCDS14519.1	chrX_105883929-105883929_C_A	628L>I	Substitution	Nonsynonymous coding	36%
MM04T	CXorf57	chromosome X open reading frame 57	CCDS14519.1	chrX_105912474-105912474_C_T	784T>I	Substitution	Nonsynonymous coding	30%
MM04T	CYB5R2	cytochrome b5 reductase 2	CCDS7780.1	chr11_7690933-7690933_C_T	61D>N	Substitution	Nonsynonymous coding	17%
MM04T	CYBB	cytochrome b-245, beta polypeptide	CCDS14242.1	chrX_37663335-37663335_C_A	368A>D	Substitution	Nonsynonymous coding	31%
MM04T	CYBRD1	cytochrome b reductase 1	CCDS2244.1	chr2_172411294-172411294_G_T	273R>I	Substitution	Nonsynonymous coding	26%
MM04T	CYP17A1	cytochrome P450, family 17, subfamily A, polypeptide 1	CCDS7541.1	chr10_104593852-104593852_A_C	232L>V	Substitution	Nonsynonymous coding	11%
MM04T	CYP1A1	cytochrome P450, family 1, subfamily A, polypeptide 1	CCDS10268.1	chr15_75015123-75015123_G_A	106R>W	Substitution	Nonsynonymous coding	11%
MM04T	CYP1A1	cytochrome P450, family 1, subfamily A, polypeptide 1	CCDS10268.1	chr15_75015426-75015426_T_C	5I>V	Substitution	Nonsynonymous coding	26%
MM04T	CYP1A2	cytochrome P450, family 1, subfamily A, polypeptide 2	CCDS32293.1	chr15_75042744-75042744_A_G	222N>S	Substitution	Nonsynonymous coding	22%
MM04T	CYP1B1	cytochrome P450, family 1, subfamily B, polypeptide 1	CCDS1793.1	chr2_38302306-38302306_G_A	76R>C	Substitution	Nonsynonymous coding	27%
MM04T	CYP27C1	cytochrome P450, family 27, subfamily C, polypeptide 1	CCDS33285.1	chr2_127961103-127961103_C_A	8R>I	Substitution	Nonsynonymous coding	22%
MM04T	CYP39A1	cytochrome P450, family 39, subfamily A, polypeptide 1	CCDS4916.1	chr6_46604166-46604166_G_T	231P>Q	Substitution	Nonsynonymous coding	29%
MM04T	CYP39A1	cytochrome P450, family 39, subfamily A, polypeptide 1	CCDS4916.1	chr6_46607367-46607367_T_G	118K>Q	Substitution	Nonsynonymous coding	36%
MM04T	CYP3A5	cytochrome P450, family 3, subfamily A, polypeptide 5	CCDS5672.1	chr7_99261673-99261673_G_T	239S>Y	Substitution	Nonsynonymous coding	13%
MM04T	CYP4F8	cytochrome P450, family 4, subfamily F, polypeptide 8	ENST00000441682	chr19_15726598-15726598_G_T	57Q>H	Substitution	Nonsynonymous coding	27%
MM04T	CYP4V2	cytochrome P450, family 4, subfamily V, polypeptide 2	CCDS34119.1	chr4_187131734-187131734_T_C	506I>T	Substitution	Nonsynonymous coding	33%
MM04T	CYP4V2	cytochrome P450, family 4, subfamily V, polypeptide 2	CCDS34119.1	chr4_187115653-187115653_G_T	NA	Substitution	Splice site acceptor	23%
MM04T	CYP4Z1	cytochrome P450, family 4, subfamily Z, polypeptide 1	CCDS545.1	chr1_47583570-47583570_G_T	494K>N	Substitution	Nonsynonymous coding	29%
MM04T	CYP7A1	cytochrome P450, family 7, subfamily A, polypeptide 1	CCDS6171.1	chr8_59404192-59404192_C_T	453E>K	Substitution	Nonsynonymous coding	33%
MM04T	CYP7A1	cytochrome P450, family 7, subfamily A, polypeptide 1	CCDS6171.1	chr8_59407144-59407144_C_A	320E>D	Substitution	Nonsynonymous coding	23%
MM04T	CYSLTR1	cysteinyl leukotriene receptor 1	CCDS14439.1	chrX_77528253-77528253_C_A	331G>X	Substitution	Nonsense	26%
MM04T	CYSLTR2	cysteinyl leukotriene receptor 2	CCDS9412.1	chr13_49281354-49281354_T_C	134V>A	Substitution	Nonsynonymous coding	25%
MM04T	CYTH3	cytohesin 3	CCDS5346.1	chr7_6217465-6217465_G_T	119Y>X	Substitution	Nonsense	28%
MM04T	DAB2	disabled homolog 2, mitogen-responsive phosphoprotein (Drosophila)	CCDS34149.1	chr5_39388915-39388915_T_G	204N>H	Substitution	Nonsynonymous coding	29%
MM04T	DAPK1	death-associated protein kinase 1	CCDS43842.1	chr9_90255268-90255268_G_T	229V>L	Substitution	Nonsynonymous coding	27%
MM04T	DAPK2	death-associated protein kinase 2	CCDS10188.1	chr15_64231485-64231485_C_T	177E>K	Substitution	Nonsynonymous coding	17%
MM04T	DARS	aspartyl-tRNA synthetase	CCDS2180.1	chr2_136680376-136680376_C_A	263E>D	Substitution	Nonsynonymous coding	22%
MM04T	DAXX	death-domain associated protein [Source:HGNC Symbol;Acc:2681]	CCDS4776.1	chr6_33288839-33288839_C_T	238R>Q	Substitution	Nonsynonymous coding	22%
MM04T	DAZL	deleted in azoospermia-like	CCDS43059.1	chr3_16638498-16638498_A_C	119L>V	Substitution	Nonsynonymous coding	26%

MM04T	DBC1	deleted in bladder cancer 1	CCDS6822.1	chr9_121929933-121929933_G_A	572S>L	Substitution	Nonsynonymous coding	37%
MM04T	DBH	dopamine beta-hydroxylase (dopamine beta-monooxygenase)	CCDS6977.2	chr9_136507452-136507452_G_A	204E>K	Substitution	Nonsynonymous coding	25%
MM04T	DCAF12L1	DDB1 and CUL4 associated factor 12-like 1	CCDS14610.1	chrX_125686443-125686443_G_A	50S>L	Substitution	Nonsynonymous coding	33%
MM04T	DCAF12L2	DDB1 and CUL4 associated factor 12-like 2	CCDS43991.1	chrX_125299442-125299442_C_T	156E>K	Substitution	Nonsynonymous coding	17%
MM04T	DCAF13	DDB1 and CUL4 associated factor 13	CCDS34934.1	chr8_104447873-104447873_C_T	421R>C	Substitution	Nonsynonymous coding	45%
MM04T	DCAF17	DDB1 and CUL4 associated factor 17	CCDS2243.2	chr2_172325529-172325529_G_T	324D>Y	Substitution	Nonsynonymous coding	25%
MM04T	DCAF4L1	DDB1 and CUL4 associated factor 4-like 1	CCDS33978.1	chr4_41984570-41984570_G_A	254R>H	Substitution	Nonsynonymous coding	29%
MM04T	DCAF4L2	DDB1 and CUL4 associated factor 4-like 2	CCDS6245.1	chr8_88885230-88885230_C_T	324E>K	Substitution	Nonsynonymous coding	32%
MM04T	DCAF6	DDB1 and CUL4 associated factor 6	CCDS1267.2	chr1_167944141-167944141_A_C	109K>T	Substitution	Nonsynonymous coding	37%
MM04T	DCAF8L2	DDB1 and CUL4 associated factor 8-like 2	CCDS35222.1	chrX_27997862-27997862_C_A	530Q>H	Substitution	Nonsynonymous coding	22%
MM04T	DCBLD1	discoidin, CUB and LCCL domain containing 1	CCDS34522.1	chr6_117841011-117841011_G_T	120E>X	Substitution	Nonsense	27%
MM04T	DCC	deleted in colorectal carcinoma	CCDS11952.1	chr18_50918172-50918172_C_A	868S>Y	Substitution	Nonsynonymous coding	27%
MM04T	DCDC2C	doublecortin domain containing 2C	ENST00000399143	chr2_3802222-3802222_A_C	74K>Q	Substitution	Nonsynonymous coding	29%
MM04T	DCHS1	dachsous 1 (Drosophila)	CCDS7771.1	chr11_6646439-6646439_C_T	2379S>N	Substitution	Nonsynonymous coding	33%
MM04T	DCHS1	dachsous 1 (Drosophila)	CCDS7771.1	chr11_6653626-6653626_C_A	1039E>D	Substitution	Nonsynonymous coding	25%
MM04T	DCHS1	dachsous 1 (Drosophila)	CCDS7771.1	chr11_6653747-6653747_C_T	999R>Q	Substitution	Nonsynonymous coding	35%
MM04T	DCHS2	dachsous 2 (Drosophila)	CCDS3785.1	chr4_155180900-155180900_T_G	1741T>P	Substitution	Nonsynonymous coding	36%
MM04T	DCHS2	dachsous 2 (Drosophila)	CCDS3785.1	chr4_155155756-155155756_C_A	2895E>X	Substitution	Nonsense	40%
MM04T	DCLK1	doublecortin-like kinase 1	CCDS9354.1	chr13_36385090-36385090_C_T	524D>N	Substitution	Nonsynonymous coding	29%
MM04T	DCLK1	doublecortin-like kinase 1	CCDS9354.1	chr13_36401883-36401883_C_A	432M>I	Substitution	Nonsynonymous coding	29%
MM04T	DCLRE1B	DNA cross-link repair 1B	CCDS866.1	chr1_114449747-114449747_C_A	107L>I	Substitution	Nonsynonymous coding	33%
MM04T	DCP2	DCP2 decapping enzyme homolog (S. cerevisiae)	CCDS34210.1	chr5_112336790-112336790_G_T	148E>X	Substitution	Nonsense	39%
MM04T	DCPS	decapping enzyme, scavenger	CCDS8473.1	chr11_126215467-126215467_G_T	325D>Y	Substitution	Nonsynonymous coding	22%
MM04T	DCST1	DC-STAMP domain containing 1	CCDS1083.1	chr1_155006556-155006556_G_T	15R>I	Substitution	Nonsynonymous coding	19%
MM04T	DCT	dopachrome tautomerase	ENST00000377021	chr13_95096685-95096685_G_T	390S>Y	Substitution	Nonsynonymous coding	18%
MM04T	DCT	dopachrome tautomerase	CCDS45060.1	chr13_95131277-95131277_C_T	78R>Q	Substitution	Nonsynonymous coding	32%
MM04T	DCUN1D4	DCN1, defective in cullin neddylation 1, domain containing 4 (S. cerevisiae)	CCDS33982.1	chr4_52777334-52777334_C_A	238F>L	Substitution	Nonsynonymous coding	28%
MM04T	DCUN1D5	DCN1, defective in cullin neddylation 1, domain containing 5 (S. cerevisiae)	CCDS8325.1	chr11_102937272-102937272_T_G	122Q>H	Substitution	Nonsynonymous coding	40%
MM04T	DDB1	damage-specific DNA binding protein 1, 127kDa	CCDS31576.1	chr11_61096945-61096945_G_A	147R>C	Substitution	Nonsynonymous coding	26%
MM04T	DDC	dopa decarboxylase (aromatic L-amino acid decarboxylase)	CCDS5511.1	chr7_50530976-50530976_G_T	466H>N	Substitution	Nonsynonymous coding	27%
MM04T	DDC	dopa decarboxylase (aromatic L-amino acid decarboxylase)	CCDS5511.1	chr7_50605603-50605603_C_A	130K>N	Substitution	Nonsynonymous coding	33%
MM04T	DDIT4L	DNA-damage-inducible transcript 4-like	CCDS34036.1	chr4_101108892-101108892_C_T	175R>Q	Substitution	Nonsynonymous coding	36%

MM04T	DDO	D-aspartate oxidase	CCDS5082.1	chr6_110726086-110726086_G_A	145R>X	Substitution	Nonsense	31%
MM04T	DDX10	DEAD (Asp-Glu-Ala-Asp) box polypeptide 10	CCDS8342.1	chr11_108547918-108547918_G_A	162R>Q	Substitution	Nonsynonymous coding	35%
MM04T	DDX18	DEAD (Asp-Glu-Ala-Asp) box polypeptide 18	CCDS2120.1	chr2_118572395-118572395_G_T	14E>D	Substitution	Nonsynonymous coding	27%
MM04T	DDX21	DEAD (Asp-Glu-Ala-Asp) box helicase 21	CCDS31211.1	chr10_70742333-70742333_C_A	706S>Y	Substitution	Nonsynonymous coding	21%
MM04T	DDX26B	DEAD/H (Asp-Glu-Ala-Asp/His) box polypeptide 26B	CCDS35401.1	chrX_134679384-134679384_G_A	76E>K	Substitution	Nonsynonymous coding	27%
MM04T	DDX26B	DEAD/H (Asp-Glu-Ala-Asp/His) box polypeptide 26B	CCDS35401.1	chrX_134713730-134713730_G_A	676D>N	Substitution	Nonsynonymous coding	46%
MM04T	DDX26B	DEAD/H (Asp-Glu-Ala-Asp/His) box polypeptide 26B	CCDS35401.1	chrX_134715496-134715496_G_A	841E>K	Substitution	Nonsynonymous coding	29%
MM04T	DDX28	DEAD (Asp-Glu-Ala-Asp) box polypeptide 28	CCDS10858.1	chr16_68056940-68056940_G_T	56L>I	Substitution	Nonsynonymous coding	24%
MM04T	DDX52	DEAD (Asp-Glu-Ala-Asp) box polypeptide 52	CCDS11323.1	chr17_35988719-35988719_A_	NA	Deletion	Splice site acceptor	11%
MM04T	DDX53	DEAD (Asp-Glu-Ala-Asp) box polypeptide 53	CCDS35214.1	chrX_23019031-23019031_C_A	286S>Y	Substitution	Nonsynonymous coding	34%
MM04T	DDX53	DEAD (Asp-Glu-Ala-Asp) box polypeptide 53	CCDS35214.1	chrX_23019046-23019046_G_T	291R>I	Substitution	Nonsynonymous coding	16%
MM04T	DDX53	DEAD (Asp-Glu-Ala-Asp) box polypeptide 53	CCDS35214.1	chrX_23019196-23019196_A_G	341D>G	Substitution	Nonsynonymous coding	37%
MM04T	DDX54	DEAD (Asp-Glu-Ala-Asp) box polypeptide 54	CCDS44984.1	chr12_113603517-113603517_G_A	551R>C	Substitution	Nonsynonymous coding	39%
MM04T	DDX59	DEAD (Asp-Glu-Ala-Asp) box polypeptide 59	CCDS30964.1	chr1_200635154-200635154_G_T	239L>M	Substitution	Nonsynonymous coding	29%
MM04T	DDX60	DEAD (Asp-Glu-Ala-Asp) box polypeptide 60	CCDS34097.1	chr4_169157455-169157455_C_A	1494R>I	Substitution	Nonsynonymous coding	18%
MM04T	DDX60	DEAD (Asp-Glu-Ala-Asp) box polypeptide 60	CCDS34097.1	chr4_169196591-169196591_G_A	737R>W	Substitution	Nonsynonymous coding	35%
MM04T	DDX60L	DEAD (Asp-Glu-Ala-Asp) box polypeptide 60-like	CCDS47161.1	chr4_169341441-169341441_C_A	829D>Y	Substitution	Nonsynonymous coding	22%
MM04T	DEF6	differentially expressed in FDCP 6 homolog (mouse)	CCDS4802.1	chr6_35278298-35278298_G_T	100K>N	Substitution	Nonsynonymous coding	35%
MM04T	DEF6	differentially expressed in FDCP 6 homolog (mouse)	CCDS4802.1	chr6_35280507-35280507_G_T	255E>D	Substitution	Nonsynonymous coding	21%
MM04T	DEGS2	delta(4)-desaturase, sphingolipid 2	CCDS9956.1	chr14_100615436-100615436_C_T	232E>K	Substitution	Nonsynonymous coding	19%
MM04T	DENND1C	DENN/MADD domain containing 1C	CCDS45938.1	chr19_6477460-6477460_C_T	126A>T	Substitution	Nonsynonymous coding	14%
MM04T	DENND2C	DENN/MADD domain containing 2C	CCDS875.1	chr1_115130025-115130025_C_A	856R>I	Substitution	Nonsynonymous coding	17%
MM04T	DENND2C	DENN/MADD domain containing 2C	CCDS875.1	chr1_115147585-115147585_A_C	485F>C	Substitution	Nonsynonymous coding	21%
MM04T	DENND4A	DENN/MADD domain containing 4A	CCDS45285.1	chr15_66044862-66044862_C_A	139R>I	Substitution	Nonsynonymous coding	25%
MM04T	DENND5A	DENN/MADD domain containing 5A	CCDS31423.1	chr11_9215135-9215135_G_T	348S>Y	Substitution	Nonsynonymous coding	25%
MM04T	DENND5B	DENN/MADD domain containing 5B	CCDS44857.1	chr12_31586133-31586133_G_T	688R>S	Substitution	Nonsynonymous coding	19%
MM04T	DENND5B	DENN/MADD domain containing 5B	CCDS44857.1	chr12_31586172-31586172_T_C	675S>G	Substitution	Nonsynonymous coding	32%
MM04T	DEPDC1B	DEP domain containing 1B	CCDS3977.1	chr5_59943349-59943349_G_T	109S>Y	Substitution	Nonsynonymous coding	23%
MM04T	DEPDC5	DEP domain containing 5	CCDS46692.1	chr22_32150912-32150912_G_T	2R>I	Substitution	Nonsynonymous coding	23%
MM04T	DEPDC5	DEP domain containing 5	CCDS46692.1	chr22_32180802-32180802_G_T	189D>Y	Substitution	Nonsynonymous coding	17%
MM04T	DEPDC5	DEP domain containing 5	CCDS46692.1	chr22_32188763-32188763_C_T	243R>X	Substitution	Nonsense	31%
MM04T	DGCR14	DiGeorge syndrome critical region gene 14	CCDS13756.1	chr22_19121869-19121869_C_T	424R>H	Substitution	Nonsynonymous coding	33%

MM04T	DGCR6	DiGeorge syndrome critical region gene 6	CCDS13753.1	chr22_18894195-18894195_G_T	76K>N	Substitution	Nonsynonymous coding	42%
MM04T	DGKB	diacylglycerol kinase, beta 90kDa	CCDS47547.1	chr7_14622735-14622735_G_T	488D>E	Substitution	Nonsynonymous coding	21%
MM04T	DGKB	diacylglycerol kinase, beta 90kDa	CCDS47547.1	chr7_14733744-14733744_C_T	223G>R	Substitution	Nonsynonymous coding	28%
MM04T	DGKB	diacylglycerol kinase, beta 90kDa	CCDS47547.1	chr7_14775701-14775701_G_A	96P>L	Substitution	Nonsynonymous coding	20%
MM04T	DGKB	diacylglycerol kinase, beta 90kDa	CCDS47547.1	chr7_14758242-14758242_C_A	131E>X	Substitution	Nonsense	33%
MM04T	DGKG	diacylglycerol kinase, gamma 90kDa	CCDS3274.1	chr3_185978295-185978295_G_A	453R>W	Substitution	Nonsynonymous coding	24%
MM04T	DGKI	diacylglycerol kinase, iota	CCDS5845.1	chr7_137263038-137263038_C_T	559R>Q	Substitution	Nonsynonymous coding	30%
MM04T	DHRS7	dehydrogenase/reductase (SDR family) member 7	CCDS9743.1	chr14_60616887-60616887_G_A	218R>X	Substitution	Nonsense	32%
MM04T	DHX29	DEAH (Asp-Glu-Ala-His) box polypeptide 29	CCDS34158.1	chr5_54585164-54585164_C_T	334E>K	Substitution	Nonsynonymous coding	19%
MM04T	DHX32	DEAH (Asp-Glu-Ala-His) box polypeptide 32	CCDS7652.1	chr10_127542718-127542718_G_T	302L>I	Substitution	Nonsynonymous coding	20%
MM04T	DHX32	DEAH (Asp-Glu-Ala-His) box polypeptide 32	CCDS7652.1	chr10_127555725-127555725_G_T	104L>I	Substitution	Nonsynonymous coding	23%
MM04T	DHX34	DEAH (Asp-Glu-Ala-His) box polypeptide 34	CCDS12700.1	chr19_47856348-47856348_G_A	21E>K	Substitution	Nonsynonymous coding	29%
MM04T	DHX36	DEAH (Asp-Glu-Ala-His) box polypeptide 36	CCDS3171.1	chr3_153998386-153998386_A_G	850L>S	Substitution	Nonsynonymous coding	53%
MM04T	DHX36	DEAH (Asp-Glu-Ala-His) box polypeptide 36	CCDS3171.1	chr3_154032942-154032942_C_A	166D>Y	Substitution	Nonsynonymous coding	26%
MM04T	DIAPH2	diaphanous homolog 2 (Drosophila)	CCDS14467.1	chrX_96185790-96185790_G_A	346R>Q	Substitution	Nonsynonymous coding	28%
MM04T	DIAPH2	diaphanous homolog 2 (Drosophila)	CCDS14467.1	chrX_96330219-96330219_C_A	736L>M	Substitution	Nonsynonymous coding	24%
MM04T	DIAPH3	diaphanous homolog 3 (Drosophila)	CCDS41898.1	chr13_60498968-60498968_G_A	704S>L	Substitution	Nonsynonymous coding	41%
MM04T	DIAPH3	diaphanous homolog 3 (Drosophila)	CCDS41898.1	chr13_60554982-60554982_C_A	498D>Y	Substitution	Nonsynonymous coding	35%
MM04T	DIAPH3	diaphanous homolog 3 (Drosophila)	CCDS41898.1	chr13_60686150-60686150_T_G	128K>N	Substitution	Nonsynonymous coding	45%
MM04T	DICER1	dicer 1, ribonuclease type III	CCDS9931.1	chr14_95562432-95562432_T_G	1609N>H	Substitution	Nonsynonymous coding	33%
MM04T	DIDO1	death inducer-obliterator 1	CCDS33506.1	chr20_61542460-61542460_G_A	169R>C	Substitution	Nonsynonymous coding	33%
MM04T	DIP2A	DIP2 disco-interacting protein 2 homolog A (Drosophila)	CCDS46655.1	chr21_47976927-47976927_G_A	1192A>T	Substitution	Nonsynonymous coding	28%
MM04T	DIP2B	DIP2 disco-interacting protein 2 homolog B (Drosophila)	CCDS31799.1	chr12_51074533-51074533_A_C	398K>T	Substitution	Nonsynonymous coding	21%
MM04T	DIP2C	DIP2 disco-interacting protein 2 homolog C (Drosophila)	CCDS7054.1	chr10_332263-332263_T_G	1357I>L	Substitution	Nonsynonymous coding	30%
MM04T	DIS3	DIS3 mitotic control homolog (S. cerevisiae)	CCDS9447.1	chr13_73337650-73337650_C_T	689R>Q	Substitution	Nonsynonymous coding	31%
MM04T	DISP1	dispatched homolog 1 (Drosophila)	CCDS1536.1	chr1_223163992-223163992_C_T	189P>S	Substitution	Nonsynonymous coding	28%
MM04T	DIXDC1	DIX domain containing 1	ENST00000440460	chr11_111864242-111864242_G_A	431R>H	Substitution	Nonsynonymous coding	32%
MM04T	DKFZP686J19100	Polycystic kidney disease protein 1-like 2 [Source:UniProtKB/Swiss-Prot;Acc:Q7Z442]	NM_052892	chr16_81155263-81155263_G_A	2180T>M	Substitution	Nonsynonymous coding	37%
MM04T	DKFZP686J19100	Polycystic kidney disease protein 1-like 2 [Source:UniProtKB/Swiss-Prot;Acc:Q7Z442]	ENST00000378597	chr16_81208314-81208314_C_A	186G>V	Substitution	Nonsynonymous coding	30%
MM04T	DKK1	dickkopf 1 homolog (Xenopus laevis)	CCDS7246.1	chr10_54074682-54074682_G_A	NA	Substitution	Splice site acceptor	29%
MM04T	DLAT	dihydrolipoamide S-acetyltransferase	CCDS8354.1	chr11_111896426-111896426_T_A	77L>H	Substitution	Nonsynonymous coding	19%
MM04T	DLAT	dihydrolipoamide S-acetyltransferase	CCDS8354.1	chr11_111908137-111908137_G_A	310E>K	Substitution	Nonsynonymous coding	35%

MM04T	DLAT	dihydrolipoamide S-acetyltransferase	CCDS8354.1	chr11_111921971-111921971_G_T	471R>I	Substitution	Nonsynonymous coding	27%
MM04T	DLEU1	deleted in lymphocytic leukemia 1 (non-protein coding)	ENST00000378180	chr13_50656577-50656577_G_A	2R>K	Substitution	Nonsynonymous coding	28%
MM04T	DLG2	discs, large homolog 2 (Drosophila)	CCDS44690.1	chr11_83810057-83810057_C_A	220G>W	Substitution	Nonsynonymous coding	27%
MM04T	DLG3	discs, large homolog 3 (Drosophila)	CCDS14403.1	chrX_69669700-69669700_G_T	232G>W	Substitution	Nonsynonymous coding	21%
MM04T	DLG3	discs, large homolog 3 (Drosophila)	CCDS14403.1	chrX_69719094-69719094_G_A	647E>K	Substitution	Nonsynonymous coding	25%
MM04T	DLG4	discs, large homolog 4 (Drosophila)	CCDS45599.1	chr17_7096835-7096835_C_T	597E>K	Substitution	Nonsynonymous coding	24%
MM04T	DLG4	discs, large homolog 4 (Drosophila)	ENST00000451807	chr17_7111437-7111437_G_A	51A>V	Substitution	Nonsynonymous coding	23%
MM04T	DLL4	delta-like 4 (Drosophila)	CCDS45232.1	chr15_41228465-41228465_G_A	427R>H	Substitution	Nonsynonymous coding	20%
MM04T	DLX1	distal-less homeobox 1	ENST00000409492	chr2_172951750-172951750_G_A	228A>T	Substitution	Nonsynonymous coding	29%
MM04T	DLX6	distal-less homeobox 6	CCDS47647.1	chr7_96636974-96636974_A_C	126E>A	Substitution	Nonsynonymous coding	28%
MM04T	DMAP1	DNA methyltransferase 1 associated protein 1	CCDS509.1	chr1_44684037-44684037_G_T	150D>Y	Substitution	Nonsynonymous coding	31%
MM04T	DMBT1	deleted in malignant brain tumors 1	CCDS44490.1	chr10_124392850-124392850_G_T	2052D>Y	Substitution	Nonsynonymous coding	17%
MM04T	DMD	dystrophin	CCDS14233.1	chrX_32235069-32235069_C_A	2134E>D	Substitution	Nonsynonymous coding	38%
MM04T	DMD	dystrophin	CCDS14233.1	chrX_32456488-32456488_C_T	1314R>Q	Substitution	Nonsynonymous coding	34%
MM04T	DMD	dystrophin	CCDS14233.1	chrX_32466695-32466695_T_G	1222N>H	Substitution	Nonsynonymous coding	27%
MM04T	DMD	dystrophin	CCDS14233.1	chrX_32408299-32408299_C_A	NA	Substitution	Splice site acceptor	34%
MM04T	DMD	dystrophin	CCDS14233.1	chrX_32591951-32591951_G_A	539R>X	Substitution	Nonsense	29%
MM04T	DMKN	dermokine	CCDS46054.1	chr19_35999141-35999141_C_T	347G>D	Substitution	Nonsynonymous coding	26%
MM04T	DMRT3	doublesex and mab-3 related transcription factor 3	CCDS6443.1	chr9_990856-990856_G_A	424V>I	Substitution	Nonsynonymous coding	28%
MM04T	DMXL1	Dmx-like 1	CCDS4125.1	chr5_118465085-118465085_G_T	428D>Y	Substitution	Nonsynonymous coding	34%
MM04T	DMXL1	Dmx-like 1	CCDS4125.1	chr5_118485340-118485340_C_T	1273S>F	Substitution	Nonsynonymous coding	31%
MM04T	DMXL1	Dmx-like 1	CCDS4125.1	chr5_118483114-118483114_G_T	954E>X	Substitution	Nonsense	32%
MM04T	DNA2	DNA replication helicase 2 homolog (yeast)	CCDS44415.1	chr10_70192052-70192052_A_C	681I>S	Substitution	Nonsynonymous coding	13%
MM04T	DNAH1	dynein, axonemal, heavy chain 1	CCDS46842.1	chr3_52429683-52429683_A_G	3750N>D	Substitution	Nonsynonymous coding	42%
MM04T	DNAH1	dynein, axonemal, heavy chain 1	CCDS46842.1	chr3_52417886-52417886_G_T	2721E>X	Substitution	Nonsense	20%
MM04T	DNAH10	dynein, axonemal, heavy chain 10	CCDS9255.2	chr12_124326035-124326035_G_T	1650R>I	Substitution	Nonsynonymous coding	20%
MM04T	DNAH10	dynein, axonemal, heavy chain 10	CCDS9255.2	chr12_124416287-124416287_C_T	4223R>C	Substitution	Nonsynonymous coding	19%
MM04T	DNAH11	dynein, axonemal, heavy chain 11	ENST00000328843	chr7_21695477-21695477_G_A	1663D>N	Substitution	Nonsynonymous coding	29%
MM04T	DNAH11	dynein, axonemal, heavy chain 11	ENST00000328843	chr7_21784597-21784597_C_T	2816T>M	Substitution	Nonsynonymous coding	32%
MM04T	DNAH11	dynein, axonemal, heavy chain 11	ENST00000328843	chr7_21813561-21813561_G_A	3101E>K	Substitution	Nonsynonymous coding	22%
MM04T	DNAH11	dynein, axonemal, heavy chain 11	ENST00000328843	chr7_21939717-21939717_C_T	4435L>F	Substitution	Nonsynonymous coding	41%
MM04T	DNAH17	dynein, axonemal, heavy chain 17	NM_173628	chr17_76475645-76475645_G_T	2608L>M	Substitution	Nonsynonymous coding	17%

MM04T	DNAH17	dynein, axonemal, heavy chain 17	NM_173628	chr17_76488801-76488801_G_A	2152P>L	Substitution	Nonsynonymous coding	29%
MM04T	DNAH17	dynein, axonemal, heavy chain 17	NM_173628	chr17_76528637-76528637_G_A	1014A>V	Substitution	Nonsynonymous coding	24%
MM04T	DNAH2	dynein, axonemal, heavy chain 2	CCDS32551.1	chr17_7637862-7637862_T_G	272L>V	Substitution	Nonsynonymous coding	38%
MM04T	DNAH2	dynein, axonemal, heavy chain 2	CCDS32551.1	chr17_7734992-7734992_G_T	4209D>Y	Substitution	Nonsynonymous coding	21%
MM04T	DNAH2	dynein, axonemal, heavy chain 2	CCDS32551.1	chr17_7630542-7630542_G_T	111E>X	Substitution	Nonsense	27%
MM04T	DNAH3	dynein, axonemal, heavy chain 3	CCDS10594.1	chr16_20996988-20996988_C_A	2359R>L	Substitution	Nonsynonymous coding	31%
MM04T	DNAH3	dynein, axonemal, heavy chain 3	CCDS10594.1	chr16_21147804-21147804_G_A	243R>C	Substitution	Nonsynonymous coding	32%
MM04T	DNAH3	dynein, axonemal, heavy chain 3	CCDS10594.1	chr16_21073847-21073847_C_A	1226E>X	Substitution	Nonsense	36%
MM04T	DNAH5	dynein, axonemal, heavy chain 5	CCDS3882.1	chr5_13729666-13729666_C_T	3922G>D	Substitution	Nonsynonymous coding	48%
MM04T	DNAH5	dynein, axonemal, heavy chain 5	CCDS3882.1	chr5_13807742-13807742_C_A	2615K>N	Substitution	Nonsynonymous coding	24%
MM04T	DNAH5	dynein, axonemal, heavy chain 5	CCDS3882.1	chr5_13913952-13913952_C_T	479R>H	Substitution	Nonsynonymous coding	11%
MM04T	DNAH6	dynein, axonemal, heavy chain 6	CCDS46348.1	chr2_84752842-84752842_G_T	127D>Y	Substitution	Nonsynonymous coding	34%
MM04T	DNAH6	dynein, axonemal, heavy chain 6	CCDS46348.1	chr2_84846859-84846859_G_A	1208R>Q	Substitution	Nonsynonymous coding	31%
MM04T	DNAH7	dynein, axonemal, heavy chain 7	CCDS42794.1	chr2_196737058-196737058_G_T	2183F>L	Substitution	Nonsynonymous coding	32%
MM04T	DNAH7	dynein, axonemal, heavy chain 7	CCDS42794.1	chr2_196883941-196883941_C_A	274M>I	Substitution	Nonsynonymous coding	23%
MM04T	DNAH7	dynein, axonemal, heavy chain 7	CCDS42794.1	chr2_196746611-196746611_G_A	1957R>X	Substitution	Nonsense	32%
MM04T	DNAH8	dynein, axonemal, heavy chain 8	CCDS4838.1	chr6_38704921-38704921_C_T	64P>S	Substitution	Nonsynonymous coding	36%
MM04T	DNAH8	dynein, axonemal, heavy chain 8	CCDS4838.1	chr6_38791412-38791412_G_T	1048E>D	Substitution	Nonsynonymous coding	17%
MM04T	DNAH8	dynein, axonemal, heavy chain 8	CCDS4838.1	chr6_38862522-38862522_C_A	2660L>I	Substitution	Nonsynonymous coding	21%
MM04T	DNAH8	dynein, axonemal, heavy chain 8	CCDS4838.1	chr6_38877508-38877508_G_T	3026S>I	Substitution	Nonsynonymous coding	28%
MM04T	DNAH8	dynein, axonemal, heavy chain 8	CCDS4838.1	chr6_38879251-38879251_C_T	3033R>X	Substitution	Nonsense	26%
MM04T	DNAH9	dynein, axonemal, heavy chain 9	CCDS11160.1	chr17_11687740-11687740_C_T	2649P>S	Substitution	Nonsynonymous coding	26%
MM04T	DNAH9	dynein, axonemal, heavy chain 9	CCDS11160.1	chr17_11696826-11696826_C_A	2690L>I	Substitution	Nonsynonymous coding	20%
MM04T	DNAH9	dynein, axonemal, heavy chain 9	CCDS11160.1	chr17_11738106-11738106_C_T	3133A>V	Substitution	Nonsynonymous coding	41%
MM04T	DNAH9	dynein, axonemal, heavy chain 9	CCDS11160.1	chr17_11784534-11784534_A_C	3537K>T	Substitution	Nonsynonymous coding	24%
MM04T	DNAH9	dynein, axonemal, heavy chain 9	CCDS11160.1	chr17_11775107-11775107_C_T	NA	Substitution	Splice site donor	21%
MM04T	DNAH9	dynein, axonemal, heavy chain 9	CCDS11160.1	chr17_11797742-11797742_C_T	3779R>X	Substitution	Nonsense	22%
MM04T	DNAI2	dynein, axonemal, intermediate chain 2	CCDS11697.1	chr17_72306270-72306270_C_A	488L>I	Substitution	Nonsynonymous coding	13%
MM04T	DNAJA4	DnaJ (Hsp40) homolog, subfamily A, member 4	CCDS10299.2	chr15_78562875-78562875_G_T	86D>Y	Substitution	Nonsynonymous coding	18%
MM04T	DNAJB12	DnaJ (Hsp40) homolog, subfamily B, member 12	CCDS7316.2	chr10_74104738-74104738_T_C	128Y>C	Substitution	Nonsynonymous coding	41%
MM04T	DNAJB4	DnaJ (Hsp40) homolog, subfamily B, member 4	CCDS684.1	chr1_78479031-78479031_G_T	170E>X	Substitution	Nonsense	19%
MM04T	DNAJB8	DnaJ (Hsp40) homolog, subfamily B, member 8	CCDS3048.1	chr3_128181439-128181439_G_A	217S>L	Substitution	Nonsynonymous coding	18%

MM04T	DNAJB9	DnaJ (Hsp40) homolog, subfamily B, member 9	CCDS5752.1	chr7_108213709-108213709_G_T	195R>I	Substitution	Nonsynonymous coding	26%
MM04T	DNAJC10	DnaJ (Hsp40) homolog, subfamily C, member 10	CCDS33345.1	chr2_183593338-183593338_A_C	152N>H	Substitution	Nonsynonymous coding	20%
MM04T	DNAJC10	DnaJ (Hsp40) homolog, subfamily C, member 10	CCDS33345.1	chr2_183594624-183594624_T_G	228F>C	Substitution	Nonsynonymous coding	21%
MM04T	DNAJC11	DnaJ (Hsp40) homolog, subfamily C, member 11	CCDS87.1	chr1_6712998-6712998_G_A	174A>V	Substitution	Nonsynonymous coding	32%
MM04T	DNAJC13	DnaJ (Hsp40) homolog, subfamily C, member 13	CCDS33857.1	chr3_132192037-132192037_A_C	753K>Q	Substitution	Nonsynonymous coding	37%
MM04T	DNAJC18	DnaJ (Hsp40) homolog, subfamily C, member 18	CCDS4214.1	chr5_138760749-138760749_C_T	205R>Q	Substitution	Nonsynonymous coding	32%
MM04T	DNAJC2	DnaJ (Hsp40) homolog, subfamily C, member 2	CCDS43628.1	chr7_102964064-102964064_C_G	234E>Q	Substitution	Nonsynonymous coding	14%
MM04T	DNAJC22	DnaJ (Hsp40) homolog, subfamily C, member 22	CCDS8785.1	chr12_49743127-49743127_G_A	158A>T	Substitution	Nonsynonymous coding	33%
MM04T	DNAJC5G	DnaJ (Hsp40) homolog, subfamily C, member 5 gamma	CCDS1744.1	chr2_27499611-27499611_G_T	5K>N	Substitution	Nonsynonymous coding	18%
MM04T	DNAJC8	DnaJ (Hsp40) homolog, subfamily C, member 8	CCDS41292.1	chr1_28534914-28534914_C_T	137R>Q	Substitution	Nonsynonymous coding	24%
MM04T	DNASE1	deoxyribonuclease I	CCDS10507.1	chr16_3707296-3707296_G_A	220D>N	Substitution	Nonsynonymous coding	13%
MM04T	DNASE1L3	deoxyribonuclease I-like 3	CCDS2886.1	chr3_58190501-58190501_T_C	143H>R	Substitution	Nonsynonymous coding	26%
MM04T	DNASE2	deoxyribonuclease II, lysosomal	CCDS12284.1	chr19_12986861-12986861_C_A	342K>N	Substitution	Nonsynonymous coding	29%
MM04T	DNER	delta/notch-like EGF repeat containing	CCDS33390.1	chr2_230223321-230223321_C_T	717A>T	Substitution	Nonsynonymous coding	23%
MM04T	DNER	delta/notch-like EGF repeat containing	CCDS33390.1	chr2_230253085-230253085_T_G	584E>A	Substitution	Nonsynonymous coding	13%
MM04T	DNER	delta/notch-like EGF repeat containing	CCDS33390.1	chr2_230578964-230578964_C_A	59C>F	Substitution	Nonsynonymous coding	45%
MM04T	DNHD1	dynein heavy chain domain 1	CCDS44532.1	chr11_6588185-6588185_C_A	3816L>M	Substitution	Nonsynonymous coding	19%
MM04T	DNHD1	dynein heavy chain domain 1	CCDS44532.1	chr11_6524151-6524151_C_A	305Y>X	Substitution	Nonsense	23%
MM04T	DNM3	dynamin 3	CCDS44276.1	chr1_172348279-172348279_G_A	668R>Q	Substitution	Nonsynonymous coding	24%
MM04T	DNMT3B	DNA (cytosine-5-)-methyltransferase 3 beta	CCDS13205.1	chr20_31394052-31394052_C_T	780S>L	Substitution	Nonsynonymous coding	22%
MM04T	DNMT3L	DNA (cytosine-5-)-methyltransferase 3-like	CCDS13705.1	chr21_45679355-45679355_C_A	103E>D	Substitution	Nonsynonymous coding	29%
MM04T	DNTTIP2	deoxynucleotidyltransferase, terminal, interacting protein 2	CCDS44174.1	chr1_94342158-94342158_G_T	445L>I	Substitution	Nonsynonymous coding	29%
MM04T	DNTTIP2	deoxynucleotidyltransferase, terminal, interacting protein 2	CCDS44174.1	chr1_94342770-94342770_T_C	241T>A	Substitution	Nonsynonymous coding	24%
MM04T	DOCK10	dedicator of cytokinesis 10	ENST00000373702	chr2_225633635-225633635_G_T	981F>L	Substitution	Nonsynonymous coding	25%
MM04T	DOCK11	dedicator of cytokinesis 11	CCDS35373.1	chrX_117722207-117722207_C_A	635P>T	Substitution	Nonsynonymous coding	55%
MM04T	DOCK11	dedicator of cytokinesis 11	CCDS35373.1	chrX_117742044-117742044_A_C	897L>F	Substitution	Nonsynonymous coding	27%
MM04T	DOCK11	dedicator of cytokinesis 11	CCDS35373.1	chrX_117742076-117742076_T_C	908L>S	Substitution	Nonsynonymous coding	22%
MM04T	DOCK11	dedicator of cytokinesis 11	CCDS35373.1	chrX_117775194-117775194_C_A	1396L>I	Substitution	Nonsynonymous coding	25%
MM04T	DOCK11	dedicator of cytokinesis 11	CCDS35373.1	chrX_117677471-117677471_C_A	NA	Substitution	Splice site acceptor	27%
MM04T	DOCK11	dedicator of cytokinesis 11	CCDS35373.1	chrX_117742034-117742034_G_A	NA	Substitution	Splice site acceptor	32%
MM04T	DOCK11	dedicator of cytokinesis 11	CCDS35373.1	chrX_117722186-117722186_G_T	628E>X	Substitution	Nonsense	56%
MM04T	DOCK2	dedicator of cytokinesis 2	CCDS4371.1	chr5_169098086-169098086_A_G	77T>A	Substitution	Nonsynonymous coding	22%

MM04T	DOCK2	dedicator of cytokinesis 2	CCDS4371.1	chr5_169139142-169139142_A_T	525K>M	Substitution	Nonsynonymous coding	14%
MM04T	DOCK2	dedicator of cytokinesis 2	CCDS4371.1	chr5_169145766-169145766_C_A	746F>L	Substitution	Nonsynonymous coding	39%
MM04T	DOCK2	dedicator of cytokinesis 2	CCDS4371.1	chr5_169446020-169446020_C_T	1097L>F	Substitution	Nonsynonymous coding	13%
MM04T	DOCK2	dedicator of cytokinesis 2	CCDS4371.1	chr5_169506107-169506107_T_C	1708V>A	Substitution	Nonsynonymous coding	22%
MM04T	DOCK3	dedicator of cytokinesis 3	CCDS46835.1	chr3_51352455-51352455_C_A	1100L>M	Substitution	Nonsynonymous coding	22%
MM04T	DOCK4	dedicator of cytokinesis 4	CCDS47688.1	chr7_111512549-111512549_C_A	606E>X	Substitution	Nonsense	21%
MM04T	DOCK5	dedicator of cytokinesis 5	CCDS6047.1	chr8_25200015-25200015_G_A	870R>Q	Substitution	Nonsynonymous coding	22%
MM04T	DOCK7	dedicator of cytokinesis 7	CCDS30734.1	chr1_63100552-63100552_C_A	309M>I	Substitution	Nonsynonymous coding	35%
MM04T	DOCK7	dedicator of cytokinesis 7	CCDS30734.1	chr1_63114085-63114085_C_A	NA	Substitution	Splice site donor	24%
MM04T	DONSON	downstream neighbor of SON	CCDS13632.1	chr21_34959898-34959898_G_T	111S>Y	Substitution	Nonsynonymous coding	21%
MM04T	DONSON	downstream neighbor of SON	CCDS13632.1	chr21_34955824-34955824_G_A	312R>X	Substitution	Nonsense	22%
MM04T	DONSON	downstream neighbor of SON	CCDS13632.1	chr21_34956908-34956908_A_C	258L>X	Substitution	Nonsense	35%
MM04T	DOPEY1	dopey family member 1	CCDS4996.1	chr6_83847733-83847733_C_A	1324F>L	Substitution	Nonsynonymous coding	26%
MM04T	DOPEY2	dopey family member 2	CCDS13643.1	chr21_37583920-37583920_C_A	251L>M	Substitution	Nonsynonymous coding	30%
MM04T	DPF2	D4, zinc and double PHD fingers family 2	CCDS8100.1	chr11_65113468-65113468_G_T	281K>N	Substitution	Nonsynonymous coding	24%
MM04T	DPM1	dolichyl-phosphate mannosyltransferase polypeptide 1, catalytic subunit	CCDS13434.1	chr20_49562280-49562280_A_C	131F>C	Substitution	Nonsynonymous coding	50%
MM04T	DPP4	dipeptidyl-peptidase 4	CCDS2216.1	chr2_162903957-162903957_T_G	50K>T	Substitution	Nonsynonymous coding	36%
MM04T	DPP6	dipeptidyl-peptidase 6	NM_130797	chr7_154593145-154593145_C_A	460F>L	Substitution	Nonsynonymous coding	13%
MM04T	DPP8	dipeptidyl-peptidase 8	CCDS10207.1	chr15_65782575-65782575_C_A	276E>X	Substitution	Nonsense	27%
MM04T	DPPA3	developmental pluripotency associated 3	CCDS8582.1	chr12_7864242-7864242_G_A	26D>N	Substitution	Nonsynonymous coding	29%
MM04T	DPPA3	developmental pluripotency associated 3	CCDS8582.1	chr12_7864224-7864224_G_T	20E>X	Substitution	Nonsense	24%
MM04T	DPPA5	developmental pluripotency associated 5	CCDS34483.1	chr6_74063933-74063933_C_T	6A>T	Substitution	Nonsynonymous coding	18%
MM04T	DPYD	dihydropyrimidine dehydrogenase	CCDS30777.1	chr1_98206012-98206012_G_A	86P>L	Substitution	Nonsynonymous coding	19%
MM04T	DPYS	dihydropyrimidinase	CCDS6302.1	chr8_105440236-105440236_C_A	355R>L	Substitution	Nonsynonymous coding	28%
MM04T	DPYSL2	dihydropyrimidinase-like 2	CCDS6051.1	chr8_26510911-26510911_C_A	542S>Y	Substitution	Nonsynonymous coding	37%
MM04T	DQX1	DEAQ box RNA-dependent ATPase 1	CCDS1949.2	chr2_74749735-74749735_C_A	489E>D	Substitution	Nonsynonymous coding	22%
MM04T	DRAM2	DNA-damage regulated autophagy modulator 2	CCDS30801.1	chr1_111660790-111660790_C_A	265D>Y	Substitution	Nonsynonymous coding	25%
MM04T	DRD1	dopamine receptor D1	CCDS4393.1	chr5_174869018-174869018_C_T	362S>N	Substitution	Nonsynonymous coding	35%
MM04T	DRD3	dopamine receptor D3	CCDS2978.1	chr3_113890695-113890695_G_T	49L>M	Substitution	Nonsynonymous coding	20%
MM04T	DROSHA	drosha, ribonuclease type III	CCDS47195.1	chr5_31521284-31521284_C_T	298R>Q	Substitution	Nonsynonymous coding	26%
MM04T	DRP2	dystrophin related protein 2	CCDS14480.2	chrX_100515124-100515124_G_T	905E>D	Substitution	Nonsynonymous coding	24%
MM04T	DSC2	desmocollin 2	CCDS11892.1	chr18_28667671-28667671_C_T	246E>K	Substitution	Nonsynonymous coding	29%

MM04T	DSC2	desmocollin 2	CCDS11892.1	chr18_28669484-28669484_C_T	183R>Q	Substitution	Nonsynonymous coding	34%
MM04T	DSCAML1	Down syndrome cell adhesion molecule like 1	CCDS8384.1	chr11_117299124-117299124_G_T	2088L>I	Substitution	Nonsynonymous coding	15%
MM04T	DSCR3	Down syndrome critical region gene 3	NM_006052	chr21_38612816-38612816_G_A	61A>V	Substitution	Nonsynonymous coding	30%
MM04T	DSCR3	Down syndrome critical region gene 3	NM_006052	chr21_38600647-38600647_G_A	179R>X	Substitution	Nonsense	29%
MM04T	DSEL	dermatan sulfate epimerase-like	CCDS11995.1	chr18_65178353-65178353_G_T	1175L>I	Substitution	Nonsynonymous coding	36%
MM04T	DSEL	dermatan sulfate epimerase-like	CCDS11995.1	chr18_65178850-65178850_C_T	1009R>H	Substitution	Nonsynonymous coding	32%
MM04T	DSEL	dermatan sulfate epimerase-like	CCDS11995.1	chr18_65179450-65179450_T_G	809K>T	Substitution	Nonsynonymous coding	23%
MM04T	DSG1	desmoglein 1	CCDS11896.1	chr18_28925982-28925982_G_T	641E>X	Substitution	Nonsense	38%
MM04T	DSP	desmoplakin	CCDS4501.1	chr6_7572198-7572198_G_A	676R>H	Substitution	Nonsynonymous coding	33%
MM04T	DSP	desmoplakin	CCDS4501.1	chr6_7576571-7576571_G_A	892R>H	Substitution	Nonsynonymous coding	29%
MM04T	DSPP	dentin sialophosphoprotein	CCDS43248.1	chr4_88533723-88533723_G_T	129E>X	Substitution	Nonsense	30%
MM04T	DST	dystonin	CCDS4959.1	chr6_56480862-56480862_C_A	2468R>I	Substitution	Nonsynonymous coding	22%
MM04T	DST	dystonin	CCDS4959.1	chr6_56480536-56480536_C_A	2577E>X	Substitution	Nonsense	23%
MM04T	DST	dystonin	CCDS4959.1	chr6_56483026-56483026_C_A	1936E>X	Substitution	Nonsense	37%
MM04T	DST	dystonin	CCDS4959.1	chr6_56484238-56484238_C_A	1532E>X	Substitution	Nonsense	36%
MM04T	DSTYK	dual serine/threonine and tyrosine protein kinase	CCDS1451.1	chr1_205132972-205132972_T_G	479K>T	Substitution	Nonsynonymous coding	33%
MM04T	DUOX2	dual oxidase 2	CCDS10117.1	chr15_45389545-45389545_G_T	1246F>L	Substitution	Nonsynonymous coding	10%
MM04T	DUOX2	dual oxidase 2	CCDS10117.1	chr15_45402880-45402880_C_T	304S>N	Substitution	Nonsynonymous coding	58%
MM04T	DUPD1	dual specificity phosphatase and pro isomerase domain containing 1	CCDS31223.1	chr10_76797720-76797720_C_A	179K>N	Substitution	Nonsynonymous coding	39%
MM04T	DUPD1	dual specificity phosphatase and pro isomerase domain containing 1	CCDS31223.1	chr10_76818260-76818260_C_T	5E>K	Substitution	Nonsynonymous coding	30%
MM04T	DUS2L	dihydrouridine synthase 2-like, SMM1 homolog (S. cerevisiae)	CCDS10859.1	chr16_68112404-68112404_G_T	409K>N	Substitution	Nonsynonymous coding	21%
MM04T	DUS4L	dihydrouridine synthase 4-like (S. cerevisiae)	CCDS5745.1	chr7_107216848-107216848_C_T	173Q>X	Substitution	Nonsense	37%
MM04T	DUSP10	dual specificity phosphatase 10	CCDS1528.1	chr1_221879709-221879709_G_A	304S>L	Substitution	Nonsynonymous coding	30%
MM04T	DUSP13	dual specificity phosphatase 13	CCDS31225.1	chr10_76855454-76855454_G_T	184F>L	Substitution	Nonsynonymous coding	31%
MM04T	DUSP13	dual specificity phosphatase 13	CCDS31225.1	chr10_76857554-76857554_G_A	123S>L	Substitution	Nonsynonymous coding	37%
MM04T	DUSP16	dual specificity phosphatase 16	CCDS8650.1	chr12_12630251-12630251_C_A	505R>L	Substitution	Nonsynonymous coding	29%
MM04T	DUSP27	dual specificity phosphatase 27 (putative)	CCDS30932.1	chr1_167096554-167096554_A_G	729N>S	Substitution	Nonsynonymous coding	30%
MM04T	DUSP4	dual specificity phosphatase 4	CCDS6072.1	chr8_29194791-29194791_G_A	313R>C	Substitution	Nonsynonymous coding	25%
MM04T	DUXA	double homeobox A	CCDS33126.1	chr19_57665787-57665787_G_A	203T>M	Substitution	Nonsynonymous coding	16%
MM04T	DYM	dymeclin	CCDS11937.1	chr18_46904958-46904958_C_A	134E>X	Substitution	Nonsense	29%
MM04T	DYNC1I1	dynein, cytoplasmic 1, intermediate chain 1	CCDS5644.1	chr7_95442621-95442621_G_T	113D>Y	Substitution	Nonsynonymous coding	22%
MM04T	DYNC1LI1	dynein, cytoplasmic 1, light intermediate chain 1	CCDS2654.1	chr3_32586454-32586454_G_T	161S>Y	Substitution	Nonsynonymous coding	21%

MM04T	DYNC1LI1	dynein, cytoplasmic 1, light intermediate chain 1	CCDS2654.1	chr3_32582681-32582681_C_A	196E>X	Substitution	Nonsense	22%
MM04T	DYNC2H1	dynein, cytoplasmic 2, heavy chain 1	CCDS44717.1	chr11_102993557-102993557_G_T	497D>Y	Substitution	Nonsynonymous coding	20%
MM04T	DYNC2H1	dynein, cytoplasmic 2, heavy chain 1	CCDS44717.1	chr11_103080663-103080663_G_A	2838R>Q	Substitution	Nonsynonymous coding	40%
MM04T	DYRK1A	dual-specificity tyrosine-(Y)-phosphorylation regulated kinase 1A	CCDS42925.1	chr21_38853118-38853118_C_T	169S>F	Substitution	Nonsynonymous coding	40%
MM04T	DYRK1A	dual-specificity tyrosine-(Y)-phosphorylation regulated kinase 1A	CCDS42925.1	chr21_38877895-38877895_C_A	NA	Substitution	Splice site donor	24%
MM04T	DYRK1B	dual-specificity tyrosine-(Y)-phosphorylation regulated kinase 1B	CCDS12543.1	chr19_40318200-40318200_T_A	302I>F	Substitution	Nonsynonymous coding	34%
MM04T	DYRK4	dual-specificity tyrosine-(Y)-phosphorylation regulated kinase 4	CCDS8530.1	chr12_4705862-4705862_G_A	176R>H	Substitution	Nonsynonymous coding	28%
MM04T	DYRK4	dual-specificity tyrosine-(Y)-phosphorylation regulated kinase 4	CCDS8530.1	chr12_4708255-4708255_C_T	208R>W	Substitution	Nonsynonymous coding	31%
MM04T	DYSF	dysferlin, limb girdle muscular dystrophy 2B (autosomal recessive)	CCDS46328.1	chr2_71909722-71909722_G_T	2079W>L	Substitution	Nonsynonymous coding	20%
MM04T	DYTN	dystrotelin	CCDS46502.1	chr2_207569605-207569605_G_A	149R>X	Substitution	Nonsense	32%
MM04T	DZIP1	DAZ interacting protein 1	CCDS9478.1	chr13_96242593-96242593_G_A	595R>X	Substitution	Nonsense	39%
MM04T	DZIP1	DAZ interacting protein 1	CCDS9478.1	chr13_96242617-96242617_C_A	587E>X	Substitution	Nonsense	39%
MM04T	DZIP1L	DAZ interacting protein 1-like	CCDS3096.1	chr3_137807294-137807294_C_T	309R>Q	Substitution	Nonsynonymous coding	28%
MM04T	E4F1	E4F transcription factor 1	CCDS32370.1	chr16_2283486-2283486_G_T	368E>D	Substitution	Nonsynonymous coding	11%
MM04T	EBF2	early B-cell factor 2	CCDS43726.1	chr8_25745438-25745438_C_A	268G>X	Substitution	Nonsense	33%
MM04T	EBPL	emopamil binding protein-like	CCDS9420.1	chr13_50235209-50235209__A	NA	Insertion	Frameshift	17%
MM04T	ECM2	extracellular matrix protein 2, female organ and adipocyte specific	CCDS6698.1	chr9_95285079-95285079_C_A	24E>X	Substitution	Nonsense	27%
MM04T	ECT2	epithelial cell transforming sequence 2 oncogene	CCDS3220.1	chr3_172473141-172473141_G_T	63E>X	Substitution	Nonsense	25%
MM04T	ECT2	epithelial cell transforming sequence 2 oncogene	ENST00000392692	chr3_172473312-172473312_G_T	84E>X	Substitution	Nonsense	37%
MM04T	EDA	ectodysplasin A	CCDS14394.1	chrX_69176981-69176981_T_G	167D>E	Substitution	Nonsynonymous coding	27%
MM04T	EDA	ectodysplasin A	CCDS14394.1	chrX_69253280-69253280_C_T	276R>C	Substitution	Nonsynonymous coding	32%
MM04T	EDARADD	EDAR-associated death domain	CCDS31065.1	chr1_236558934-236558934_C_A	2A>D	Substitution	Nonsynonymous coding	11%
MM04T	EDEM1	ER degradation enhancer, mannosidase alpha-like 1	CCDS33686.1	chr3_5249893-5249893_T_C	485V>A	Substitution	Nonsynonymous coding	34%
MM04T	EDNRB	endothelin receptor type B	CCDS9461.1	chr13_78477652-78477652_G_T	192L>I	Substitution	Nonsynonymous coding	12%
MM04T	EEF1A2	eukaryotic translation elongation factor 1 alpha 2	CCDS13522.1	chr20_62121873-62121873_T_C	330K>E	Substitution	Nonsynonymous coding	19%
MM04T	EEF1A2	eukaryotic translation elongation factor 1 alpha 2	CCDS13522.1	chr20_62121947-62121947_C_T	305G>D	Substitution	Nonsynonymous coding	28%
MM04T	EFCAB13	EF-hand calcium binding domain 13	CCDS11512.1	chr17_45507328-45507328_G_T	NA	Substitution	Splice site donor	43%
MM04T	EFCAB3	EF-hand calcium binding domain 3	CCDS45751.1	chr17_60484531-60484531_C_A	327F>L	Substitution	Nonsynonymous coding	33%
MM04T	EFCAB4B	EF-hand calcium binding domain 4B	CCDS44803.1	chr12_3763456-3763456_G_A	323S>F	Substitution	Nonsynonymous coding	22%
MM04T	EFCAB5	EF-hand calcium binding domain 5	NM_198529	chr17_28361271-28361271_C_A	356D>E	Substitution	Nonsynonymous coding	31%
MM04T	EFCAB5	EF-hand calcium binding domain 5	NM_198529	chr17_28434854-28434854_C_A	1442H>N	Substitution	Nonsynonymous coding	33%
MM04T	EFCAB6	EF-hand calcium binding domain 6	CCDS14049.1	chr22_43950904-43950904_C_A	1165D>Y	Substitution	Nonsynonymous coding	20%

MM04T	EFCAB6	EF-hand calcium binding domain 6	CCDS14049.1	chr22_44068007-44068007_C_A	496E>X	Substitution	Nonsense	24%
MM04T	EFCAB9	EF-hand calcium binding domain 9	ENST00000398186	chr5_171621276-171621276_C_A	34H>N	Substitution	Nonsynonymous coding	32%
MM04T	EFCAB9	EF-hand calcium binding domain 9	ENST00000398186	chr5_171627714-171627714_C_A	144L>I	Substitution	Nonsynonymous coding	27%
MM04T	EFHB	EF-hand domain family, member B	CCDS33715.2	chr3_19975021-19975021_C_T	164V>I	Substitution	Nonsynonymous coding	18%
MM04T	EFHC1	EF-hand domain (C-terminal) containing 1	CCDS4942.1	chr6_52355035-52355035_C_T	580R>C	Substitution	Nonsynonymous coding	22%
MM04T	EFHC2	EF-hand domain (C-terminal) containing 2	NM_025184	chrX_44091783-44091783_G_A	522R>C	Substitution	Nonsynonymous coding	25%
MM04T	EFHC2	EF-hand domain (C-terminal) containing 2	NM_025184	chrX_44120532-44120532_C_A	132R>L	Substitution	Nonsynonymous coding	30%
MM04T	EFNB2	ephrin-B2	CCDS9507.1	chr13_107164957-107164957_T_G	109K>T	Substitution	Nonsynonymous coding	22%
MM04T	EFTUD2	elongation factor Tu GTP binding domain containing 2	CCDS11489.1	chr17_42962672-42962672_T_G	101K>T	Substitution	Nonsynonymous coding	27%
MM04T	EGF	epidermal growth factor	CCDS3689.1	chr4_110865165-110865165_C_A	226S>Y	Substitution	Nonsynonymous coding	31%
MM04T	EGFLAM	EGF-like, fibronectin type III and laminin G domains	CCDS3924.1	chr5_38407932-38407932_C_A	391F>L	Substitution	Nonsynonymous coding	40%
MM04T	EGFR	epidermal growth factor receptor	CCDS5514.1	chr7_55269478-55269478_A_G	NA	Substitution	Splice site donor	25%
MM04T	EGLN3	egl nine homolog 3 (C. elegans)	CCDS9646.1	chr14_34396185-34396185_C_A	223K>N	Substitution	Nonsynonymous coding	35%
MM04T	EGR1	early growth response 1	CCDS4206.1	chr5_137803327-137803327_G_A	397A>T	Substitution	Nonsynonymous coding	35%
MM04T	EGR2	early growth response 2	CCDS7267.1	chr10_64573428-64573428_G_A	324R>C	Substitution	Nonsynonymous coding	21%
MM04T	EGR4	early growth response 4	NM_001965	chr2_73520425-73520425_A_C	110F>L	Substitution	Nonsynonymous coding	21%
MM04T	EHBP1	EH domain binding protein 1	CCDS1872.1	chr2_63175921-63175921_T_C	682V>A	Substitution	Nonsynonymous coding	33%
MM04T	EHD3	EH-domain containing 3	CCDS1774.1	chr2_31483473-31483473_C_A	200F>L	Substitution	Nonsynonymous coding	22%
MM04T	EIF1B	eukaryotic translation initiation factor 1B	CCDS2690.1	chr3_40352441-40352441_A_G	30Y>C	Substitution	Nonsynonymous coding	41%
MM04T	EIF2B4	eukaryotic translation initiation factor 2B, subunit 4 delta, 67kDa	ENST00000417567	chr2_27590108-27590108_C_A	172K>N	Substitution	Nonsynonymous coding	23%
MM04T	EIF2C2	eukaryotic translation initiation factor 2C, 2	NM_012154	chr8_141569503-141569503_C_A	261E>X	Substitution	Nonsense	33%
MM04T	EIF2S2	eukaryotic translation initiation factor 2, subunit 2 beta, 38kDa	CCDS13231.1	chr20_32684601-32684601_C_T	182R>Q	Substitution	Nonsynonymous coding	24%
MM04T	EIF3B	eukaryotic translation initiation factor 3, subunit B	CCDS5332.1	chr7_2409115-2409115_C_T	471S>F	Substitution	Nonsynonymous coding	20%
MM04T	EIF3H	eukaryotic translation initiation factor 3, subunit H	CCDS6319.1	chr8_117738321-117738321_G_A	75R>W	Substitution	Nonsynonymous coding	21%
MM04T	EIF3I	eukaryotic translation initiation factor 3, subunit I	CCDS357.1	chr1_32696777-32696777_G_A	320E>K	Substitution	Nonsynonymous coding	32%
MM04T	EIF4B	eukaryotic translation initiation factor 4B	CCDS41788.1	chr12_53413718-53413718_G_A	129E>K	Substitution	Nonsynonymous coding	36%
MM04T	EIF4B	eukaryotic translation initiation factor 4B	CCDS41788.1	chr12_53413791-53413791_C_T	153A>V	Substitution	Nonsynonymous coding	38%
MM04T	EIF4G1	eukaryotic translation initiation factor 4 gamma, 1	CCDS3259.1	chr3_184041233-184041233_G_A	709R>Q	Substitution	Nonsynonymous coding	21%
MM04T	EIF4G2	eukaryotic translation initiation factor 4 gamma, 2	ENST00000429377	chr11_10821304-10821304_C_A	NA	Substitution	Splice site acceptor	27%
MM04T	ELAC1	elaC homolog 1 (E. coli)	CCDS11949.1	chr18_48510794-48510794_A_C	162E>D	Substitution	Nonsynonymous coding	25%
MM04T	ELAC2	elaC homolog 2 (E. coli)	CCDS11164.1	chr17_12901819-12901819_C_A	477R>I	Substitution	Nonsynonymous coding	34%
MM04T	ELAVL3	ELAV (embryonic lethal, abnormal vision, Drosophila)-like 3 (Hu antigen C)	CCDS32912.1	chr19_11577045-11577045_T_C	92D>G	Substitution	Nonsynonymous coding	36%

MM04T	ELAVL3	ELAV (embryonic lethal, abnormal vision, Drosophila)-like 3 (Hu antigen C)	CCDS32912.1	chr19_11577086-11577086_C_A	78Q>H	Substitution	Nonsynonymous coding	23%
MM04T	ELL2	elongation factor, RNA polymerase II, 2	CCDS4080.1	chr5_95234462-95234462_C_T	336R>Q	Substitution	Nonsynonymous coding	20%
MM04T	ELL2	elongation factor, RNA polymerase II, 2	CCDS4080.1	chr5_95255174-95255174_C_A	91D>Y	Substitution	Nonsynonymous coding	10%
MM04T	ELMO1	engulfment and cell motility 1	CCDS5449.1	chr7_37283969-37283969_C_A	147M>I	Substitution	Nonsynonymous coding	21%
MM04T	ELMOD2	ELMO/CED-12 domain containing 2	CCDS3752.1	chr4_141471463-141471463_G_T	272E>D	Substitution	Nonsynonymous coding	16%
MM04T	ELN	elastin	CCDS5562.2	chr7_73449737-73449737_T_G	42F>L	Substitution	Nonsynonymous coding	24%
MM04T	ELP3	elongator acetyltransferase complex subunit 3	CCDS6065.1	chr8_28017878-28017878_G_A	464E>K	Substitution	Nonsynonymous coding	23%
MM04T	ELTD1	EGF, latrophilin and seven transmembrane domain containing 1	CCDS41352.1	chr1_79387308-79387308_C_T	416G>D	Substitution	Nonsynonymous coding	27%
MM04T	ELTD1	EGF, latrophilin and seven transmembrane domain containing 1	CCDS41352.1	chr1_79392656-79392656_G_T	333S>Y	Substitution	Nonsynonymous coding	23%
MM04T	EMC1	ER membrane protein complex subunit 1	CCDS190.1	chr1_19557343-19557343_G_A	687R>X	Substitution	Nonsense	36%
MM04T	EME2	essential meiotic endonuclease 1 homolog 2 (S. pombe)	CCDS32364.1	chr16_1825081-1825081_G_A	173E>K	Substitution	Nonsynonymous coding	23%
MM04T	EMILIN1	elastin microfibril interfacer 1	CCDS1733.1	chr2_27306518-27306518_G_T	693Q>H	Substitution	Nonsynonymous coding	27%
MM04T	EMILIN2	elastin microfibril interfacer 2	CCDS11828.1	chr18_2891879-2891879_C_A	585S>Y	Substitution	Nonsynonymous coding	31%
MM04T	EMILIN3	elastin microfibril interfacer 3	CCDS13316.1	chr20_39990951-39990951_G_A	420L>F	Substitution	Nonsynonymous coding	16%
MM04T	EML3	echinoderm microtubule associated protein like 3	CCDS8023.2	chr11_62378456-62378456_C_A	154R>I	Substitution	Nonsynonymous coding	31%
MM04T	EML5	echinoderm microtubule associated protein like 5	CCDS45148.1	chr14_89109311-89109311_A_G	1388V>A	Substitution	Nonsynonymous coding	27%
MM04T	EML5	echinoderm microtubule associated protein like 5	CCDS45148.1	chr14_89172612-89172612_C_A	606A>S	Substitution	Nonsynonymous coding	33%
MM04T	EML5	echinoderm microtubule associated protein like 5	CCDS45148.1	chr14_89258735-89258735_T_C	38Y>C	Substitution	Nonsynonymous coding	24%
MM04T	EML6	echinoderm microtubule associated protein like 6	CCDS46286.1	chr2_55077308-55077308_T_C	466L>S	Substitution	Nonsynonymous coding	24%
MM04T	EML6	echinoderm microtubule associated protein like 6	CCDS46286.1	chr2_55118278-55118278_C_A	842F>L	Substitution	Nonsynonymous coding	19%
MM04T	EMR2	egf-like module containing, mucin-like, hormone receptor-like 2	CCDS32935.1	chr19_14875264-14875264_G_T	355F>L	Substitution	Nonsynonymous coding	28%
MM04T	EMR3	egf-like module containing, mucin-like, hormone receptor-like 3	CCDS12315.1	chr19_14762001-14762001_G_A	198A>V	Substitution	Nonsynonymous coding	33%
MM04T	EN1	engrailed homeobox 1	CCDS2123.1	chr2_119604341-119604341_C_T	135G>S	Substitution	Nonsynonymous coding	13%
MM04T	ENAH	enabled homolog (Drosophila)	ENST00000284563	chr1_225705332-225705332_G_A	407S>L	Substitution	Nonsynonymous coding	22%
MM04T	ENAM	enamelin	CCDS3544.2	chr4_71508219-71508219_C_A	359P>H	Substitution	Nonsynonymous coding	21%
MM04T	ENAM	enamelin	CCDS3544.2	chr4_71509418-71509418_C_A	759L>I	Substitution	Nonsynonymous coding	23%
MM04T	ENC1	ectodermal-neural cortex 1 (with BTB domain)	CCDS4021.1	chr5_73930802-73930802_G_T	503F>L	Substitution	Nonsynonymous coding	38%
MM04T	ENKUR	enkurin, TRPC channel interacting protein	CCDS7146.1	chr10_25284661-25284661_G_A	121P>S	Substitution	Nonsynonymous coding	26%
MM04T	ENO3	enolase 3 (beta, muscle)	CCDS11062.1	chr17_4858481-4858481_G_A	186A>T	Substitution	Nonsynonymous coding	17%
MM04T	ENPEP	glutamyl aminopeptidase (aminopeptidase A)	CCDS3691.1	chr4_111441204-111441204_C_A	517S>Y	Substitution	Nonsynonymous coding	22%
MM04T	ENPEP	glutamyl aminopeptidase (aminopeptidase A)	CCDS3691.1	chr4_111482604-111482604_G_T	922E>X	Substitution	Nonsense	36%
MM04T	ENPP2	ectonucleotide pyrophosphatase/phosphodiesterase 2	CCDS6329.1	chr8_120596006-120596006_C_A	550K>N	Substitution	Nonsynonymous coding	27%

MM04T	ENPP2	ectonucleotide pyrophosphatase/phosphodiesterase 2	CCDS6329.1	chr8_120605998-120605998_C_A	411D>Y	Substitution	Nonsynonymous coding	31%
MM04T	ENPP3	ectonucleotide pyrophosphatase/phosphodiesterase 3	CCDS5148.1	chr6_132061368-132061368_T_G	769L>V	Substitution	Nonsynonymous coding	22%
MM04T	ENPP4	ectonucleotide pyrophosphatase/phosphodiesterase 4	CCDS34468.1	chr6_46107475-46107475_T_G	52F>C	Substitution	Nonsynonymous coding	45%
MM04T	ENSG00000105663	-	CCDS46055.1	chr19_36219939-36219939_T_C	1581C>R	Substitution	Nonsynonymous coding	30%
MM04T	ENSG00000141140	-	CCDS45654.1	chr17_34854347-34854347_G_T	640F>L	Substitution	Nonsynonymous coding	26%
MM04T	ENSG00000155319	-	ENST00000329373	chr16_46587925-46587925_T_A	222E>D	Substitution	Nonsynonymous coding	38%
MM04T	ENSG00000167634	-	CCDS46183.1	chr19_55441952-55441952_C_T	909D>N	Substitution	Nonsynonymous coding	17%
MM04T	ENSG00000172901	-	CCDS4124.1	chr5_115336914-115336914_A_C	600N>H	Substitution	Nonsynonymous coding	35%
MM04T	ENSG00000172901	-	CCDS4124.1	chr5_115350968-115350968_G_T	824D>Y	Substitution	Nonsynonymous coding	34%
MM04T	ENSG00000173517	-	CCDS42062.1	chr15_77471639-77471639_G_T	877S>Y	Substitution	Nonsynonymous coding	20%
MM04T	ENSG00000173517	-	CCDS42062.1	chr15_77473232-77473232_G_T	346S>Y	Substitution	Nonsynonymous coding	32%
MM04T	ENSG00000173517	-	CCDS42062.1	chr15_77473298-77473298_G_A	324S>F	Substitution	Nonsynonymous coding	19%
MM04T	ENSG00000176276	-	ENST00000338626	chr5_75469951-75469951_T_C	20T>A	Substitution	Nonsynonymous coding	17%
MM04T	ENSG00000180923	-	ENST00000316540	chr11_6079337-6079337_G_T	57L>I	Substitution	Nonsynonymous coding	16%
MM04T	ENSG00000180947	-	ENST00000316698	chr11_5922404-5922404_G_T	133R>I	Substitution	Nonsynonymous coding	24%
MM04T	ENSG00000183760	-	CCDS33018.1	chr19_39591613-39591613_C_T	278R>W	Substitution	Nonsynonymous coding	10%
MM04T	ENSG00000186031	-	ENST00000414268	chr15_33445962-33445962_C_T	385R>Q	Substitution	Nonsynonymous coding	20%
MM04T	ENSG00000196763	-	ENST00000359554	chr11_71628623-71628623_C_A	129E>D	Substitution	Nonsynonymous coding	23%
MM04T	ENSG00000196763	-	ENST00000359554	chr11_71634359-71634359_G_T	14S>Y	Substitution	Nonsynonymous coding	18%
MM04T	ENSG00000205141	-	ENST00000378906	chr15_99799696-99799696_C_T	265A>V	Substitution	Nonsynonymous coding	28%
MM04T	ENSG00000205141	-	ENST00000378906	chr15_99799726-99799726_T_C	275V>A	Substitution	Nonsynonymous coding	25%
MM04T	ENSG00000205748	-	ENST00000381510	chr16_17190128-17190128_C_T	51R>Q	Substitution	Nonsynonymous coding	24%
MM04T	ENSG00000214422	-	ENST00000398302	chr15_98417388-98417388_A_G	43V>A	Substitution	Nonsynonymous coding	18%
MM04T	ENSG00000214636	-	ENST00000398711	chr6_52522303-52522303_G_A	34A>T	Substitution	Nonsynonymous coding	30%
MM04T	ENSG00000214877	-	ENST00000399160	chr9_94939981-94939981_G_T	237P>T	Substitution	Nonsynonymous coding	21%
MM04T	ENSG00000215273	-	ENST00000399906	chr4_17875268-17875268_G_T	480S>Y	Substitution	Nonsynonymous coding	28%
MM04T	ENSG00000215273	-	ENST00000399906	chr4_17875248-17875248_C_A	487E>X	Substitution	Nonsense	27%
MM04T	ENSG00000225576	-	ENST00000452141	chr7_38356761-38356761_C_A	75D>Y	Substitution	Nonsynonymous coding	25%
MM04T	ENSG00000226351	-	ENST00000433532	chr16_73127100-73127100_C_T	49R>C	Substitution	Nonsynonymous coding	17%
MM04T	ENSG00000227699	-	ENST00000456709	chrX_101437347-101437347_G_T	20K>N	Substitution	Nonsynonymous coding	25%
MM04T	ENSG00000229070	-	ENST00000450721	chr17_75441393-75441393_G_A	102R>H	Substitution	Nonsynonymous coding	20%
MM04T	ENSG00000230938	-	ENST00000413018	chr9_133057296-133057296_G_T	222D>Y	Substitution	Nonsynonymous coding	41%

MM04T	ENSG00000235766	-	ENST00000440128	chr19_38795444-38795444_G_T	26L>M	Substitution	Nonsynonymous coding	20%
MM04T	ENTPD2	ectonucleoside triphosphate diphosphohydrolase 2	CCDS7026.1	chr9_139945716-139945716_C_A	165E>X	Substitution	Nonsense	17%
MM04T	ENTPD4	ectonucleoside triphosphate diphosphohydrolase 4	CCDS6041.1	chr8_23294537-23294537_G_T	428F>L	Substitution	Nonsynonymous coding	31%
MM04T	ENTPD7	ectonucleoside triphosphate diphosphohydrolase 7	CCDS7480.1	chr10_101460767-101460767_G_T	458R>I	Substitution	Nonsynonymous coding	17%
MM04T	ENY2	enhancer of yellow 2 homolog (Drosophila)	CCDS43762.1	chr8_110346698-110346698_A_G	1M>V	Substitution	Nonsynonymous coding	28%
MM04T	EP300	E1A binding protein p300	CCDS14010.1	chr22_41533773-41533773_G_A	580R>Q	Substitution	Nonsynonymous coding	30%
MM04T	EP400	E1A binding protein p400	CCDS31929.1	chr12_132479459-132479459_G_A	928V>I	Substitution	Nonsynonymous coding	24%
MM04T	EPAS1	endothelial PAS domain protein 1	CCDS1825.1	chr2_46583923-46583923_C_T	144R>C	Substitution	Nonsynonymous coding	28%
MM04T	EPB41L1	erythrocyte membrane protein band 4.1-like 1	ENST00000344237	chr20_34794791-34794791_A_G	506K>E	Substitution	Nonsynonymous coding	25%
MM04T	EPB41L2	erythrocyte membrane protein band 4.1-like 2	ENST00000257986	chr6_131197871-131197871_C_A	41E>D	Substitution	Nonsynonymous coding	25%
MM04T	EPB41L3	erythrocyte membrane protein band 4.1-like 3	CCDS11838.1	chr18_5428360-5428360_C_A	339K>N	Substitution	Nonsynonymous coding	20%
MM04T	EPB41L3	erythrocyte membrane protein band 4.1-like 3	CCDS11838.1	chr18_5428385-5428385_C_A	331R>I	Substitution	Nonsynonymous coding	24%
MM04T	EPB41L3	erythrocyte membrane protein band 4.1-like 3	CCDS11838.1	chr18_5443858-5443858_C_A	170E>X	Substitution	Nonsense	29%
MM04T	EPB41L4B	erythrocyte membrane protein band 4.1 like 4B	ENST00000311609	chr9_112069506-112069506_C_A	15R>S	Substitution	Nonsynonymous coding	42%
MM04T	EPB49	erythrocyte membrane protein band 4.9 (dematin)	CCDS6020.1	chr8_21938878-21938878_G_T	374E>D	Substitution	Nonsynonymous coding	24%
MM04T	EPC1	enhancer of polycomb homolog 1 (Drosophila)	CCDS7172.1	chr10_32561099-32561099_A_C	NA	Substitution	Splice site acceptor	37%
MM04T	EPC2	enhancer of polycomb homolog 2 (Drosophila)	CCDS46422.1	chr2_149528727-149528727_C_A	497F>L	Substitution	Nonsynonymous coding	32%
MM04T	EPG5	ectopic P-granules autophagy protein 5 homolog (C. elegans)	CCDS11926.2	chr18_43446791-43446791_G_T	2198S>Y	Substitution	Nonsynonymous coding	39%
MM04T	EPG5	ectopic P-granules autophagy protein 5 homolog (C. elegans)	CCDS11926.2	chr18_43490649-43490649_A_C	1348L>V	Substitution	Nonsynonymous coding	24%
MM04T	EPG5	ectopic P-granules autophagy protein 5 homolog (C. elegans)	CCDS11926.2	chr18_43532513-43532513_C_T	369D>N	Substitution	Nonsynonymous coding	21%
MM04T	EPHA3	EPH receptor A3	CCDS2922.1	chr3_89259282-89259282_G_T	142K>N	Substitution	Nonsynonymous coding	25%
MM04T	EPHA3	EPH receptor A3	CCDS2922.1	chr3_89456438-89456438_A_C	538E>D	Substitution	Nonsynonymous coding	41%
MM04T	EPHA4	EPH receptor A4	CCDS2447.1	chr2_222307561-222307561_C_T	688V>M	Substitution	Nonsynonymous coding	24%
MM04T	EPHA4	EPH receptor A4	CCDS2447.1	chr2_222428558-222428558_T_G	239E>A	Substitution	Nonsynonymous coding	35%
MM04T	EPHA5	EPH receptor A5	CCDS3513.1	chr4_66230770-66230770_G_T	734P>H	Substitution	Nonsynonymous coding	38%
MM04T	EPHA5	EPH receptor A5	CCDS3513.1	chr4_66242774-66242774_C_T	600E>K	Substitution	Nonsynonymous coding	37%
MM04T	EPHA5	EPH receptor A5	CCDS3513.1	chr4_66467895-66467895_C_A	125R>I	Substitution	Nonsynonymous coding	29%
MM04T	EPHA5	EPH receptor A5	CCDS3513.1	chr4_66468014-66468014_C_A	85E>D	Substitution	Nonsynonymous coding	38%
MM04T	EPHA6	EPH receptor A6	CCDS46876.1	chr3_96962891-96962891_G_T	456D>Y	Substitution	Nonsynonymous coding	36%
MM04T	EPHA7	EPH receptor A7	CCDS5031.1	chr6_94120597-94120597_C_A	152D>Y	Substitution	Nonsynonymous coding	33%
MM04T	EPHA7	EPH receptor A7	CCDS5031.1	chr6_93956553-93956553_G_A	895R>X	Substitution	Nonsense	30%
MM04T	EPHA8	EPH receptor A8	CCDS225.1	chr1_22927836-22927836_G_T	925G>W	Substitution	Nonsynonymous coding	27%

MM04T	EPHB2	EPH receptor B2	CCDS230.1	chr1_23111411-23111411_C_T	218S>L	Substitution	Nonsynonymous coding	34%
MM04T	EPHB6	EPH receptor B6	CCDS5873.2	chr7_142563718-142563718_C_A	369P>H	Substitution	Nonsynonymous coding	25%
MM04T	EPHX4	epoxide hydrolase 4	CCDS736.1	chr1_92508415-92508415_G_T	118S>I	Substitution	Nonsynonymous coding	32%
MM04T	EPRS	glutamyl-prolyl-tRNA synthetase	CCDS31027.1	chr1_220170332-220170332_G_A	845S>F	Substitution	Nonsynonymous coding	27%
MM04T	EPX	eosinophil peroxidase	CCDS11602.1	chr17_56276955-56276955_G_T	446R>I	Substitution	Nonsynonymous coding	24%
MM04T	ERAP2	endoplasmic reticulum aminopeptidase 2	CCDS4086.1	chr5_96215729-96215729_G_T	114D>Y	Substitution	Nonsynonymous coding	24%
MM04T	ERAP2	endoplasmic reticulum aminopeptidase 2	CCDS4086.1	chr5_96228041-96228041_G_A	337E>K	Substitution	Nonsynonymous coding	19%
MM04T	ERBB3	v-erb-b2 erythroblastic leukemia viral oncogene homolog 3 (avian)	CCDS31833.1	chr12_56477581-56477581_G_T	43E>D	Substitution	Nonsynonymous coding	27%
MM04T	ERCC3	excision repair cross-complementing rodent repair deficiency, complementation group 3	CCDS2144.1	chr2_128050287-128050287_A_G	124Y>H	Substitution	Nonsynonymous coding	22%
MM04T	ERCC4	excision repair cross-complementing rodent repair deficiency, complementation group 4	CCDS32390.1	chr16_14028108-14028108_T_G	388L>V	Substitution	Nonsynonymous coding	18%
MM04T	ERCC4	excision repair cross-complementing rodent repair deficiency, complementation group 4	CCDS32390.1	chr16_14029000-14029000_C_T	NA	Substitution	Splice site acceptor	28%
MM04T	ERCC6L	excision repair cross-complementing rodent repair deficiency, complementation group 6-	CCDS35329.1	chrX_71458748-71458748_C_T	5R>Q	Substitution	Nonsynonymous coding	15%
MM04T	ERG	v-ets erythroblastosis virus E26 oncogene homolog (avian)	CCDS46648.1	chr21_39763598-39763598_T_C	292D>G	Substitution	Nonsynonymous coding	26%
MM04T	ERGIC3	ERGIC and golgi 3	CCDS13258.1	chr20_34129891-34129891_G_T	15K>N	Substitution	Nonsynonymous coding	31%
MM04T	ERI2	ERI1 exoribonuclease family member 2	CCDS45436.1	chr16_20814239-20814239_A_C	61F>C	Substitution	Nonsynonymous coding	25%
MM04T	ERI2	ERI1 exoribonuclease family member 2	CCDS45436.1	chr16_20814943-20814943_G_T	25L>I	Substitution	Nonsynonymous coding	21%
MM04T	ERICH1	glutamate-rich 1	CCDS5955.1	chr8_665983-665983_C_A	16R>I	Substitution	Nonsynonymous coding	21%
MM04T	ERMP1	endoplasmic reticulum metallopeptidase 1	CCDS34983.1	chr9_5812195-5812195_C_A	348E>D	Substitution	Nonsynonymous coding	36%
MM04T	ERP27	endoplasmic reticulum protein 27	CCDS8670.1	chr12_15067707-15067707_G_A	262R>C	Substitution	Nonsynonymous coding	33%
MM04T	ESPN	espin	CCDS70.1	chr1_6520072-6520072_C_T	811R>X	Substitution	Nonsense	33%
MM04T	ESRP1	epithelial splicing regulatory protein 1	CCDS47897.1	chr8_95658444-95658444_G_T	142E>X	Substitution	Nonsense	29%
MM04T	ESRP2	epithelial splicing regulatory protein 2	CCDS10863.1	chr16_68267770-68267770_C_T	153D>N	Substitution	Nonsynonymous coding	30%
MM04T	ESYT3	extended synaptotagmin-like protein 3	CCDS3101.2	chr3_138181016-138181016_G_T	295D>Y	Substitution	Nonsynonymous coding	12%
MM04T	ETAA1	Ewing tumor-associated antigen 1	CCDS1882.1	chr2_67631660-67631660_G_A	616D>N	Substitution	Nonsynonymous coding	20%
MM04T	ETF1	eukaryotic translation termination factor 1	CCDS4207.1	chr5_137846307-137846307_G_A	344L>F	Substitution	Nonsynonymous coding	35%
MM04T	EVI2B	ecotropic viral integration site 2B	CCDS11266.1	chr17_29631853-29631853_C_A	259E>X	Substitution	Nonsense	12%
MM04T	EXD1	exonuclease 3'-5' domain containing 1	CCDS10072.1	chr15_41483675-41483675_G_T	219L>I	Substitution	Nonsynonymous coding	36%
MM04T	EXD1	exonuclease 3'-5' domain containing 1	CCDS10072.1	chr15_41488141-41488141_C_A	152R>I	Substitution	Nonsynonymous coding	29%
MM04T	EXO5	exonuclease 5	CCDS453.1	chr1_40980890-40980890_T_G	225F>C	Substitution	Nonsynonymous coding	37%
MM04T	EXOC1	exocyst complex component 1	CCDS3502.1	chr4_56726672-56726672_G_A	74D>N	Substitution	Nonsynonymous coding	24%
MM04T	EXOC1	exocyst complex component 1	CCDS3502.1	chr4_56734600-56734600_G_T	172E>X	Substitution	Nonsense	27%
MM04T	EXOC2	exocyst complex component 2	CCDS34327.1	chr6_564909-564909_C_A	488Q>H	Substitution	Nonsynonymous coding	19%

MM04T	EXOC2	exocyst complex component 2	CCDS34327.1	chr6_619477-619477_C_A	163E>D	Substitution	Nonsynonymous coding	23%
MM04T	EXOC5	exocyst complex component 5	CCDS45111.1	chr14_57710963-57710963_C_T	129E>K	Substitution	Nonsynonymous coding	25%
MM04T	EXOC6B	exocyst complex component 6B	CCDS46333.1	chr2_72740365-72740365_C_A	355D>Y	Substitution	Nonsynonymous coding	36%
MM04T	EXOSC5	exosome component 5	CCDS12580.1	chr19_41898768-41898768_T_G	NA	Substitution	Splice site donor	19%
MM04T	EYA2	eyes absent homolog 2 (Drosophila)	CCDS13403.1	chr20_45717931-45717931_G_A	239D>N	Substitution	Nonsynonymous coding	31%
MM04T	EYA2	eyes absent homolog 2 (Drosophila)	CCDS13403.1	chr20_45717965-45717965_G_A	250R>Q	Substitution	Nonsynonymous coding	35%
MM04T	EYA3	eyes absent homolog 3 (Drosophila)	CCDS316.1	chr1_28300982-28300982_G_A	573L>F	Substitution	Nonsynonymous coding	25%
MM04T	EYS	eyes shut homolog (Drosophila)	CCDS47445.1	chr6_64431603-64431603_T_G	2775K>T	Substitution	Nonsynonymous coding	22%
MM04T	EYS	eyes shut homolog (Drosophila)	CCDS47445.1	chr6_65303162-65303162_C_A	1242R>I	Substitution	Nonsynonymous coding	30%
MM04T	EZR	ezrin	CCDS5258.1	chr6_159206479-159206479_A_C	110I>S	Substitution	Nonsynonymous coding	41%
MM04T	EZR	ezrin	CCDS5258.1	chr6_159205763-159205763_G_A	NA	Substitution	Splice site acceptor	31%
MM04T	F11	coagulation factor XI	CCDS3847.1	chr4_187206855-187206855_G_T	456E>D	Substitution	Nonsynonymous coding	18%
MM04T	F12	coagulation factor XII (Hageman factor)	CCDS34302.1	chr5_176832185-176832185_C_A	133E>D	Substitution	Nonsynonymous coding	28%
MM04T	F13A1	coagulation factor XIII, A1 polypeptide	CCDS4496.1	chr6_6251137-6251137_C_A	199E>D	Substitution	Nonsynonymous coding	13%
MM04T	F2RL1	coagulation factor II (thrombin) receptor-like 1	CCDS4033.1	chr5_76129240-76129240_C_T	270R>X	Substitution	Nonsense	21%
MM04T	F5	coagulation factor V (proaccelerin, labile factor)	CCDS1281.1	chr1_169487731-169487731_C_A	2088K>N	Substitution	Nonsynonymous coding	31%
MM04T	F5	coagulation factor V (proaccelerin, labile factor)	CCDS1281.1	chr1_169511133-169511133_C_A	1065K>N	Substitution	Nonsynonymous coding	31%
MM04T	F8	coagulation factor VIII, procoagulant component	CCDS35457.1	chrX_154124371-154124371_T_G	2137N>T	Substitution	Nonsynonymous coding	32%
MM04T	F8	coagulation factor VIII, procoagulant component	CCDS35457.1	chrX_154133145-154133145_C_A	1843A>S	Substitution	Nonsynonymous coding	11%
MM04T	F8	coagulation factor VIII, procoagulant component	CCDS35457.1	chrX_154158329-154158329_G_T	1246L>I	Substitution	Nonsynonymous coding	28%
MM04T	F8	coagulation factor VIII, procoagulant component	CCDS35457.1	chrX_154159052-154159052_T_G	1005K>Q	Substitution	Nonsynonymous coding	25%
MM04T	F8	coagulation factor VIII, procoagulant component	CCDS35457.1	chrX_154182270-154182270_C_A	600E>D	Substitution	Nonsynonymous coding	28%
MM04T	F9	coagulation factor IX	CCDS14666.1	chrX_138642921-138642921_G_T	249D>Y	Substitution	Nonsynonymous coding	31%
MM04T	FABP3	fatty acid binding protein 3, muscle and heart (mammary-derived growth inhibitor)	CCDS342.1	chr1_31845798-31845798_T_C	22K>E	Substitution	Nonsynonymous coding	38%
MM04T	FABP5	fatty acid binding protein 5 (psoriasis-associated)	CCDS6228.1	chr8_82195744-82195744_G_T	75E>X	Substitution	Nonsense	20%
MM04T	FABP7	fatty acid binding protein 7, brain	CCDS5127.1	chr6_123102318-123102318_T_G	109I>M	Substitution	Nonsynonymous coding	34%
MM04T	FADD	Fas (TNFRSF6)-associated via death domain	CCDS8196.1	chr11_70049703-70049703_C_A	46F>L	Substitution	Nonsynonymous coding	20%
MM04T	FADD	Fas (TNFRSF6)-associated via death domain	CCDS8196.1	chr11_70052414-70052414_G_T	154E>D	Substitution	Nonsynonymous coding	16%
MM04T	FAIM2	Fas apoptotic inhibitory molecule 2	CCDS8791.1	chr12_50284871-50284871_G_A	154T>I	Substitution	Nonsynonymous coding	29%
MM04T	FAM111A	family with sequence similarity 111, member A	CCDS7973.1	chr11_58920169-58920169_C_T	343S>L	Substitution	Nonsynonymous coding	34%
MM04T	FAM111B	family with sequence similarity 111, member B	CCDS7972.1	chr11_58893110-58893110_A_C	514K>Q	Substitution	Nonsynonymous coding	34%
MM04T	FAM111B	family with sequence similarity 111, member B	CCDS7972.1	chr11_58893641-58893641_C_A	691L>I	Substitution	Nonsynonymous coding	32%

MM04T	FAM111B	family with sequence similarity 111, member B	CCDS7972.1	chr11_58892414-58892414_A_T	282K>X	Substitution	Nonsense	39%
MM04T	FAM114A1	family with sequence similarity 114, member A1	CCDS3447.1	chr4_38942622-38942622_A_C	524I>L	Substitution	Nonsynonymous coding	26%
MM04T	FAM120A	family with sequence similarity 120A	CCDS6706.1	chr9_96326639-96326639_G_T	1058E>D	Substitution	Nonsynonymous coding	24%
MM04T	FAM124A	family with sequence similarity 124A	CCDS9427.1	chr13_51855383-51855383_C_A	580F>L	Substitution	Nonsynonymous coding	20%
MM04T	FAM135B	family with sequence similarity 135, member B	CCDS6375.2	chr8_139144887-139144887_T_G	1390E>D	Substitution	Nonsynonymous coding	33%
MM04T	FAM135B	family with sequence similarity 135, member B	CCDS6375.2	chr8_139164319-139164319_T_C	800D>G	Substitution	Nonsynonymous coding	23%
MM04T	FAM135B	family with sequence similarity 135, member B	CCDS6375.2	chr8_139209780-139209780_C_T	268E>K	Substitution	Nonsynonymous coding	17%
MM04T	FAM135B	family with sequence similarity 135, member B	CCDS6375.2	chr8_139255185-139255185_C_A	223E>D	Substitution	Nonsynonymous coding	39%
MM04T	FAM13C	family with sequence similarity 13, member C	CCDS44406.1	chr10_61014160-61014160_C_T	344R>Q	Substitution	Nonsynonymous coding	35%
MM04T	FAM13C	family with sequence similarity 13, member C	CCDS44406.1	chr10_61028336-61028336_A_T	224F>I	Substitution	Nonsynonymous coding	10%
MM04T	FAM149B1	family with sequence similarity 149, member B1	CCDS44435.1	chr10_74968531-74968531_G_A	233D>N	Substitution	Nonsynonymous coding	30%
MM04T	FAM150A	family with sequence similarity 150, member A	CCDS6150.1	chr8_53452415-53452415_T_C	101T>A	Substitution	Nonsynonymous coding	35%
MM04T	FAM154B	family with sequence similarity 154, member B	CCDS32310.1	chr15_82564057-82564057_C_T	56S>L	Substitution	Nonsynonymous coding	24%
MM04T	FAM155A	family with sequence similarity 155, member A	CCDS32006.1	chr13_108518279-108518279_G_T	222Y>X	Substitution	Nonsense	29%
MM04T	FAM161B	family with sequence similarity 161, member B	CCDS9822.1	chr14_74409272-74409272_G_T	358L>M	Substitution	Nonsynonymous coding	24%
MM04T	FAM161B	family with sequence similarity 161, member B	CCDS9822.1	chr14_74404303-74404303_A_G	NA	Substitution	Splice site donor	25%
MM04T	FAM167A	family with sequence similarity 167, member A	CCDS5981.1	chr8_11281901-11281901_C_T	209R>Q	Substitution	Nonsynonymous coding	29%
MM04T	FAM168B	family with sequence similarity 168, member B	CCDS42755.1	chr2_131840165-131840165_C_A	19G>X	Substitution	Nonsense	41%
MM04T	FAM169B	family with sequence similarity 169, member B	CCDS45360.1	chr15_99023958-99023958_T_G	19I>L	Substitution	Nonsynonymous coding	25%
MM04T	FAM172A	family with sequence similarity 172, member A	CCDS4069.1	chr5_93120106-93120106_G_A	336R>X	Substitution	Nonsense	36%
MM04T	FAM172BP	family with sequence similarity 172, member B, pseudogene	ENST00000313598	chr3_101240789-101240789_G_T	19E>X	Substitution	Nonsense	36%
MM04T	FAM179B	family with sequence similarity 179, member B	CCDS9681.1	chr14_45431759-45431759_G_T	45E>D	Substitution	Nonsynonymous coding	29%
MM04T	FAM180A	family with sequence similarity 180, member A	CCDS5841.1	chr7_135418723-135418723_T_G	174*>C	Substitution	Nonsynonymous coding	23%
MM04T	FAM181A	family with sequence similarity 181, member A	CCDS9914.1	chr14_94394744-94394744_G_A	100R>H	Substitution	Nonsynonymous coding	18%
MM04T	FAM181B	family with sequence similarity 181, member B	CCDS31648.1	chr11_82443745-82443745_C_T	343G>S	Substitution	Nonsynonymous coding	27%
MM04T	FAM193A	family with sequence similarity 193, member A	CCDS33943.1	chr4_2691412-2691412_C_A	546F>L	Substitution	Nonsynonymous coding	42%
MM04T	FAM194A	family with sequence similarity 194, member A	CCDS3151.2	chr3_150403561-150403561_C_A	251E>X	Substitution	Nonsense	26%
MM04T	FAM194B	family with sequence similarity 194, member B	CCDS45045.1	chr13_46170992-46170992_G_T	50S>Y	Substitution	Nonsynonymous coding	26%
MM04T	FAM196B	family with sequence similarity 196, member B	CCDS47336.1	chr5_169308432-169308432_C_T	454R>Q	Substitution	Nonsynonymous coding	30%
MM04T	FAM198A	family with sequence similarity 198, member A	CCDS46808.1	chr3_43074761-43074761_C_T	336R>C	Substitution	Nonsynonymous coding	31%
MM04T	FAM19A2	family with sequence similarity 19 (chemokine (C-C motif)-like), member A2	CCDS8962.1	chr12_62261182-62261182_C_T	9A>T	Substitution	Nonsynonymous coding	43%
MM04T	FAM205B	family with sequence similarity 205, member B	ENST00000455647	chr9_34835399-34835399_T_G	30K>N	Substitution	Nonsynonymous coding	50%

MM04T	FAM208B	family with sequence similarity 208, member B	CCDS41485.1	chr10_5762872-5762872_G_A	29D>N	Substitution	Nonsynonymous coding	44%
MM04T	FAM208B	family with sequence similarity 208, member B	CCDS41485.1	chr10_5772538-5772538_G_T	192K>N	Substitution	Nonsynonymous coding	35%
MM04T	FAM208B	family with sequence similarity 208, member B	CCDS41485.1	chr10_5803386-5803386_A_G	2376N>D	Substitution	Nonsynonymous coding	19%
MM04T	FAM208B	family with sequence similarity 208, member B	CCDS41485.1	chr10_5804993-5804993_T_C	2431*>R	Substitution	Nonsynonymous coding	26%
MM04T	FAM213A	family with sequence similarity 213, member A	CCDS7368.1	chr10_82187096-82187096_C_A	140F>L	Substitution	Nonsynonymous coding	30%
MM04T	FAM214A	family with sequence similarity 214, member A	CCDS45263.1	chr15_52877044-52877044_C_A	992R>I	Substitution	Nonsynonymous coding	32%
MM04T	FAM214A	family with sequence similarity 214, member A	CCDS45263.1	chr15_52905953-52905953_G_A	40R>X	Substitution	Nonsense	22%
MM04T	FAM217A	family with sequence similarity 217, member A	CCDS4489.1	chr6_4069066-4069066_C_A	464R>I	Substitution	Nonsynonymous coding	27%
MM04T	FAM218BP	family with sequence similarity 218, member B, pseudogene	CCDS3807.1	chr4_165878426-165878426_C_A	84F>L	Substitution	Nonsynonymous coding	34%
MM04T	FAM3C	family with sequence similarity 3, member C	CCDS5782.1	chr7_120991324-120991324_C_T	NA	Substitution	Splice site acceptor	26%
MM04T	FAM45B	family with sequence similarity 45, member B (pseudogene)	ENST00000408950	chrX_129629329-129629329_G_T	66R>I	Substitution	Nonsynonymous coding	43%
MM04T	FAM45B	family with sequence similarity 45, member B (pseudogene)	ENST00000408950	chrX_129629944-129629944_C_A	271A>E	Substitution	Nonsynonymous coding	32%
MM04T	FAM46A	family with sequence similarity 46, member A	CCDS34489.1	chr6_82459997-82459997_C_A	248E>D	Substitution	Nonsynonymous coding	36%
MM04T	FAM46D	family with sequence similarity 46, member D	CCDS14446.1	chrX_79698342-79698342_G_T	102D>Y	Substitution	Nonsynonymous coding	28%
MM04T	FAM5C	family with sequence similarity 5, member C	CCDS1373.1	chr1_190203552-190203552_G_T	225S>Y	Substitution	Nonsynonymous coding	27%
MM04T	FAM5C	family with sequence similarity 5, member C	CCDS1373.1	chr1_190234101-190234101_G_A	171S>L	Substitution	Nonsynonymous coding	25%
MM04T	FAM5C	family with sequence similarity 5, member C	CCDS1373.1	chr1_190234137-190234137_C_T	159R>Q	Substitution	Nonsynonymous coding	38%
MM04T	FAM5C	family with sequence similarity 5, member C	CCDS1373.1	chr1_190250841-190250841_C_A	92E>D	Substitution	Nonsynonymous coding	23%
MM04T	FAM5C	family with sequence similarity 5, member C	CCDS1373.1	chr1_190424010-190424010_C_T	4R>Q	Substitution	Nonsynonymous coding	30%
MM04T	FAM63B	family with sequence similarity 63, member B	CCDS42046.1	chr15_59124080-59124080_C_T	445R>W	Substitution	Nonsynonymous coding	30%
MM04T	FAM63B	family with sequence similarity 63, member B	CCDS42046.1	chr15_59146766-59146766_A_G	608D>G	Substitution	Nonsynonymous coding	31%
MM04T	FAM65B	family with sequence similarity 65, member B	CCDS47383.1	chr6_24828445-24828445_G_T	883T>N	Substitution	Nonsynonymous coding	35%
MM04T	FAM69A	family with sequence similarity 69, member A	CCDS44173.1	chr1_93309421-93309421_G_A	269A>V	Substitution	Nonsynonymous coding	34%
MM04T	FAM69A	family with sequence similarity 69, member A	CCDS44173.1	chr1_93309074-93309074_C_A	385E>X	Substitution	Nonsense	21%
MM04T	FAM71A	family with sequence similarity 71, member A	CCDS1507.1	chr1_212799958-212799958_C_A	580S>Y	Substitution	Nonsynonymous coding	31%
MM04T	FAM71F1	family with sequence similarity 71, member F1	CCDS5804.1	chr7_128355540-128355540_G_T	15K>N	Substitution	Nonsynonymous coding	31%
MM04T	FAM78A	family with sequence similarity 78, member A	CCDS6941.2	chr9_134151321-134151321_C_A	82K>N	Substitution	Nonsynonymous coding	25%
MM04T	FAM83B	family with sequence similarity 83, member B	CCDS34479.1	chr6_54806160-54806160_T_G	797F>L	Substitution	Nonsynonymous coding	39%
MM04T	FAM8A1	family with sequence similarity 8, member A1	CCDS4540.1	chr6_17605171-17605171_G_T	290E>X	Substitution	Nonsense	21%
MM04T	FAM91A1	family with sequence similarity 91, member A1	CCDS6346.2	chr8_124792783-124792783_G_A	NA	Substitution	Splice site donor	29%
MM04T	FAM92B	family with sequence similarity 92, member B	CCDS32500.1	chr16_85133795-85133795_G_A	235L>F	Substitution	Nonsynonymous coding	19%
MM04T	FAM9A	family with sequence similarity 9, member A	CCDS14131.1	chrX_8763444-8763444_C_T	169R>K	Substitution	Nonsynonymous coding	15%

MM04T	FAM9B	family with sequence similarity 9, member B	CCDS14132.1	chrX_8995995-8995995_C_A	136E>X	Substitution	Nonsense	22%
MM04T	FAN1	FANCD2/FANCI-associated nuclease 1	CCDS32186.1	chr15_31197218-31197218_A_C	118K>Q	Substitution	Nonsynonymous coding	34%
MM04T	FAN1	FANCD2/FANCI-associated nuclease 1	CCDS32186.1	chr15_31197222-31197222_G_T	119R>M	Substitution	Nonsynonymous coding	22%
MM04T	FAN1	FANCD2/FANCI-associated nuclease 1	CCDS32186.1	chr15_31214438-31214438_G_A	685E>K	Substitution	Nonsynonymous coding	27%
MM04T	FANCB	Fanconi anemia, complementation group B	CCDS14161.1	chrX_14883337-14883337__T	NA	Insertion	Frameshift	30%
MM04T	FANCB	Fanconi anemia, complementation group B	CCDS14161.1	chrX_14871234-14871234_A_C	418I>S	Substitution	Nonsynonymous coding	34%
MM04T	FANCF	Fanconi anemia, complementation group F	CCDS7857.1	chr11_22646381-22646381_G_T	326L>I	Substitution	Nonsynonymous coding	34%
MM04T	FANCI	Fanconi anemia, complementation group I	CCDS45346.1	chr15_89819979-89819979_G_T	384E>X	Substitution	Nonsense	27%
MM04T	FANCM	Fanconi anemia, complementation group M	CCDS32070.1	chr14_45658140-45658140__A	NA	Insertion	Frameshift	17%
MM04T	FANCM	Fanconi anemia, complementation group M	CCDS32070.1	chr14_45645960-45645960_A_C	1335K>Q	Substitution	Nonsynonymous coding	25%
MM04T	FAR2	fatty acyl CoA reductase 2	CCDS8717.1	chr12_29463979-29463979_C_T	263R>W	Substitution	Nonsynonymous coding	24%
MM04T	FARSA	phenylalanyl-tRNA synthetase, alpha subunit	CCDS12287.1	chr19_13035466-13035466_G_T	394F>L	Substitution	Nonsynonymous coding	21%
MM04T	FAS	Fas (TNF receptor superfamily, member 6)	CCDS7393.1	chr10_90762789-90762789_C_A	12L>I	Substitution	Nonsynonymous coding	15%
MM04T	FAS	Fas (TNF receptor superfamily, member 6)	CCDS7393.1	chr10_90774060-90774060_G_T	287K>N	Substitution	Nonsynonymous coding	18%
MM04T	FASTKD3	FAST kinase domains 3	CCDS3873.1	chr5_7867125-7867125_G_A	358R>C	Substitution	Nonsynonymous coding	39%
MM04T	FASTKD3	FAST kinase domains 3	CCDS3873.1	chr5_7868054-7868054_C_T	48R>Q	Substitution	Nonsynonymous coding	38%
MM04T	FASTKD5	FAST kinase domains 5	CCDS13048.1	chr20_3128870-3128870_G_T	283H>N	Substitution	Nonsynonymous coding	36%
MM04T	FAT1	FAT tumor suppressor homolog 1 (Drosophila)	CCDS47177.1	chr4_187539573-187539573_C_A	2723D>Y	Substitution	Nonsynonymous coding	26%
MM04T	FAT1	FAT tumor suppressor homolog 1 (Drosophila)	CCDS47177.1	chr4_187629879-187629879_T_C	368E>G	Substitution	Nonsynonymous coding	30%
MM04T	FAT1	FAT tumor suppressor homolog 1 (Drosophila)	CCDS47177.1	chr4_187630240-187630240_C_T	248E>K	Substitution	Nonsynonymous coding	25%
MM04T	FAT4	FAT tumor suppressor homolog 4 (Drosophila)	CCDS3732.3	chr4_126240262-126240262_G_T	899S>I	Substitution	Nonsynonymous coding	27%
MM04T	FAT4	FAT tumor suppressor homolog 4 (Drosophila)	CCDS3732.3	chr4_126370291-126370291_T_G	2707I>S	Substitution	Nonsynonymous coding	29%
MM04T	FAT4	FAT tumor suppressor homolog 4 (Drosophila)	CCDS3732.3	chr4_126370930-126370930_T_G	2920I>S	Substitution	Nonsynonymous coding	28%
MM04T	FAT4	FAT tumor suppressor homolog 4 (Drosophila)	CCDS3732.3	chr4_126371994-126371994_G_A	3275D>N	Substitution	Nonsynonymous coding	27%
MM04T	FAT4	FAT tumor suppressor homolog 4 (Drosophila)	CCDS3732.3	chr4_126329778-126329778_C_T	1917R>X	Substitution	Nonsense	30%
MM04T	FATE1	fetal and adult testis expressed 1	CCDS14700.1	chrX_150890388-150890388_G_T	119D>Y	Substitution	Nonsynonymous coding	20%
MM04T	FBN2	fibrillin 2	CCDS34222.1	chr5_127615955-127615955_T_C	2356N>S	Substitution	Nonsynonymous coding	38%
MM04T	FBN2	fibrillin 2	CCDS34222.1	chr5_127702116-127702116_G_T	752F>L	Substitution	Nonsynonymous coding	24%
MM04T	FBXL16	F-box and leucine-rich repeat protein 16	CCDS10421.1	chr16_747109-747109_C_A	99E>D	Substitution	Nonsynonymous coding	20%
MM04T	FBXL2	F-box and leucine-rich repeat protein 2	CCDS2658.1	chr3_33416874-33416874_C_A	251S>Y	Substitution	Nonsynonymous coding	31%
MM04T	FBXO17	F-box protein 17	CCDS12526.1	chr19_39439234-39439234_G_A	145S>L	Substitution	Nonsynonymous coding	24%
MM04T	FBXO24	F-box protein 24	CCDS5698.1	chr7_100198444-100198444_C_A	555F>L	Substitution	Nonsynonymous coding	37%

MM04T	FBXO3	F-box protein 3	CCDS7887.1	chr11_33763483-33763483_G_A	463R>C	Substitution	Nonsynonymous coding	29%
MM04T	FBXO34	F-box protein 34	CCDS32086.1	chr14_55818887-55818887_G_T	593K>N	Substitution	Nonsynonymous coding	25%
MM04T	FBXO47	F-box protein 47	CCDS32639.1	chr17_37107892-37107892_G_T	186F>L	Substitution	Nonsynonymous coding	16%
MM04T	FBXO6	F-box protein 6	CCDS133.1	chr1_11728959-11728959_C_T	82R>W	Substitution	Nonsynonymous coding	16%
MM04T	FBXO9	F-box protein 9	NM_012347	chr6_52960341-52960341_C_T	372R>C	Substitution	Nonsynonymous coding	31%
MM04T	FBXO9	F-box protein 9	NM_012347	chr6_52962610-52962610_C_A	442F>L	Substitution	Nonsynonymous coding	20%
MM04T	FBXW12	F-box and WD repeat domain containing 12	NM_001159929	chr3_48413802-48413802_G_T	9D>Y	Substitution	Nonsynonymous coding	21%
MM04T	FBXW2	F-box and WD repeat domain containing 2	CCDS43872.1	chr9_123538482-123538482_A_C	236D>E	Substitution	Nonsynonymous coding	17%
MM04T	FBXW7	F-box and WD repeat domain containing 7, E3 ubiquitin protein ligase	CCDS3777.1	chr4_153249457-153249457_G_A	441R>W	Substitution	Nonsynonymous coding	36%
MM04T	FBXW7	F-box and WD repeat domain containing 7, E3 ubiquitin protein ligase	CCDS3777.1	chr4_153332556-153332556_C_A	134E>X	Substitution	Nonsense	33%
MM04T	FCGR2B	Fc fragment of IgG, low affinity IIb, receptor (CD32)	CCDS30924.1	chr1_161647303-161647303_C_T	298P>L	Substitution	Nonsynonymous coding	30%
MM04T	FCHSD2	FCH and double SH3 domains 2	CCDS8218.2	chr11_72553812-72553812_T_G	594I>L	Substitution	Nonsynonymous coding	23%
MM04T	FCRL6	Fc receptor-like 6	CCDS30912.1	chr1_159778787-159778787_C_T	119S>F	Substitution	Nonsynonymous coding	16%
MM04T	FDXACB1	ferredoxin-fold anticodon binding domain containing 1	CCDS44729.1	chr11_111746764-111746764_A_C	253L>V	Substitution	Nonsynonymous coding	29%
MM04T	FEN1	flap structure-specific endonuclease 1	CCDS8010.1	chr11_61563500-61563500_T_C	223F>L	Substitution	Nonsynonymous coding	29%
MM04T	FER1L6	fer-1-like 6 (C. elegans)	CCDS43767.1	chr8_124975580-124975580_G_T	47D>Y	Substitution	Nonsynonymous coding	24%
MM04T	FER1L6	fer-1-like 6 (C. elegans)	CCDS43767.1	chr8_124988139-124988139_A_G	229N>D	Substitution	Nonsynonymous coding	19%
MM04T	FERMT1	fermitin family member 1	CCDS13098.1	chr20_6068464-6068464_A_G	444V>A	Substitution	Nonsynonymous coding	30%
MM04T	FGA	fibrinogen alpha chain	CCDS3787.1	chr4_155507614-155507614_G_A	323P>S	Substitution	Nonsynonymous coding	22%
MM04T	FGD2	FYVE, RhoGEF and PH domain containing 2	CCDS4829.1	chr6_36976732-36976732_A_G	64E>G	Substitution	Nonsynonymous coding	24%
MM04T	FGF19	fibroblast growth factor 19	CCDS8193.1	chr11_69514117-69514117_G_T	188F>L	Substitution	Nonsynonymous coding	23%
MM04T	FGF3	fibroblast growth factor 3	CCDS8195.1	chr11_69631089-69631089_G_A	108S>L	Substitution	Nonsynonymous coding	30%
MM04T	FGL1	fibrinogen-like 1	CCDS6004.1	chr8_17739682-17739682_C_T	24E>K	Substitution	Nonsynonymous coding	32%
MM04T	FH	fumarate hydratase	CCDS1617.1	chr1_241672025-241672025_C_A	206V>L	Substitution	Nonsynonymous coding	32%
MM04T	FHAD1	forkhead-associated (FHA) phosphopeptide binding domain 1	ENST00000401090	chr1_15690249-15690249_G_T	145K>N	Substitution	Nonsynonymous coding	37%
MM04T	FHDC1	FH2 domain containing 1	CCDS34081.1	chr4_153881906-153881906_C_T	254R>W	Substitution	Nonsynonymous coding	48%
MM04T	FIGF	c-fos induced growth factor (vascular endothelial growth factor D)	CCDS14166.1	chrX_15381368-15381368_C_T	55R>Q	Substitution	Nonsynonymous coding	32%
MM04T	FIGF	c-fos induced growth factor (vascular endothelial growth factor D)	CCDS14166.1	chrX_15381416-15381416_C_T	39R>Q	Substitution	Nonsynonymous coding	44%
MM04T	FIGN	fidgetin	CCDS2221.2	chr2_164467599-164467599_G_T	248P>Q	Substitution	Nonsynonymous coding	26%
MM04T	FIGNL1	fidgetin-like 1	CCDS5510.1	chr7_50513740-50513740_T_C	416I>V	Substitution	Nonsynonymous coding	11%
MM04T	FILIP1	filamin A interacting protein 1	CCDS4984.1	chr6_76022810-76022810_C_T	913R>Q	Substitution	Nonsynonymous coding	22%
MM04T	FILIP1	filamin A interacting protein 1	CCDS4984.1	chr6_76023553-76023553_C_A	665Q>H	Substitution	Nonsynonymous coding	32%

MM04T	FILIP1	filamin A interacting protein 1	CCDS4984.1	chr6_76124519-76124519_C_T	57R>Q	Substitution	Nonsynonymous coding	24%
MM04T	FILIP1	filamin A interacting protein 1	CCDS4984.1	chr6_76024677-76024677_C_A	291E>X	Substitution	Nonsense	24%
MM04T	FILIP1L	filamin A interacting protein 1-like	CCDS43117.1	chr3_99568368-99568368_C_A	718E>X	Substitution	Nonsense	28%
MM04T	FJX1	four jointed box 1 (Drosophila)	CCDS44570.1	chr11_35640581-35640581_C_T	133P>S	Substitution	Nonsynonymous coding	22%
MM04T	FKBP15	FK506 binding protein 15, 133kDa	CCDS48007.1	chr9_115931907-115931907_C_T	1028E>K	Substitution	Nonsynonymous coding	23%
MM04T	FKBPL	FK506 binding protein like	CCDS4738.1	chr6_32097328-32097328_G_A	77S>F	Substitution	Nonsynonymous coding	38%
MM04T	FLAD1	FAD1 flavin adenine dinucleotide synthetase homolog (S. cerevisiae)	CCDS1078.1	chr1_154956384-154956384_C_T	72P>S	Substitution	Nonsynonymous coding	42%
MM04T	FLG2	filaggrin family member 2	CCDS30861.1	chr1_152329042-152329042_G_T	407S>Y	Substitution	Nonsynonymous coding	26%
MM04T	FLII	flightless I homolog (Drosophila)	CCDS11192.1	chr17_18150327-18150327_C_A	906G>C	Substitution	Nonsynonymous coding	31%
MM04T	FLII	flightless I homolog (Drosophila)	CCDS11192.1	chr17_18151948-18151948_G_T	702F>L	Substitution	Nonsynonymous coding	26%
MM04T	FLII	flightless I homolog (Drosophila)	CCDS11192.1	chr17_18154609-18154609_G_T	522F>L	Substitution	Nonsynonymous coding	18%
MM04T	FLNA	filamin A, alpha	CCDS48194.1	chrX_153582395-153582395_G_T	1858F>L	Substitution	Nonsynonymous coding	43%
MM04T	FLNB	filamin B, beta	CCDS2885.1	chr3_58131773-58131773_G_T	1851E>X	Substitution	Nonsense	22%
MM04T	FLNC	filamin C, gamma	CCDS43644.1	chr7_128497270-128497270_G_A	2554E>K	Substitution	Nonsynonymous coding	25%
MM04T	FLT1	fms-related tyrosine kinase 1	CCDS9330.1	chr13_28885803-28885803_G_A	1187P>S	Substitution	Nonsynonymous coding	30%
MM04T	FLT3	fms-related tyrosine kinase 3	CCDS31953.1	chr13_28644738-28644738_C_T	19A>T	Substitution	Nonsynonymous coding	62%
MM04T	FLT4	fms-related tyrosine kinase 4	ENST00000376868	chr5_180046652-180046652_G_A	697A>V	Substitution	Nonsynonymous coding	32%
MM04T	FMN1	formin 1	CCDS45209.1	chr15_33300232-33300232_C_A	513E>X	Substitution	Nonsense	19%
MM04T	FMN2	formin 2	CCDS31069.2	chr1_240635737-240635737_G_T	1709R>I	Substitution	Nonsynonymous coding	25%
MM04T	FMNL1	formin-like 1	CCDS11497.1	chr17_43311458-43311458_G_T	169D>Y	Substitution	Nonsynonymous coding	12%
MM04T	FMNL1	formin-like 1	CCDS11497.1	chr17_43323302-43323302_G_A	1018A>T	Substitution	Nonsynonymous coding	34%
MM04T	FMNL2	formin-like 2	CCDS46429.1	chr2_153493095-153493095_G_T	945K>N	Substitution	Nonsynonymous coding	22%
MM04T	FMO2	flavin containing monooxygenase 2 (non-functional)	CCDS1293.1	chr1_171178026-171178026_C_A	450C>X	Substitution	Nonsense	23%
MM04T	FMO3	flavin containing monooxygenase 3	CCDS1292.1	chr1_171080043-171080043_G_T	244K>N	Substitution	Nonsynonymous coding	33%
MM04T	FMO3	flavin containing monooxygenase 3	CCDS1292.1	chr1_171086275-171086275_T_G	431I>S	Substitution	Nonsynonymous coding	43%
MM04T	FMR1NB	fragile X mental retardation 1 neighbor	CCDS14683.1	chrX_147084798-147084798_G_T	119D>Y	Substitution	Nonsynonymous coding	26%
MM04T	FN1	fibronectin 1	CCDS42814.1	chr2_216286864-216286864_A_C	499V>G	Substitution	Nonsynonymous coding	34%
MM04T	FNBP1	formin binding protein 1	CCDS48040.1	chr9_132689480-132689480_C_A	261Q>H	Substitution	Nonsynonymous coding	30%
MM04T	FNBP1L	formin binding protein 1-like	NM_001164473	chr1_94012435-94012435_G_T	437K>N	Substitution	Nonsynonymous coding	39%
MM04T	FNDC1	fibronectin type III domain containing 1	CCDS47512.1	chr6_159660646-159660646_T_G	1426I>M	Substitution	Nonsynonymous coding	39%
MM04T	FNDC3B	fibronectin type III domain containing 3B	ENST00000443501	chr3_171844832-171844832_G_T	5E>D	Substitution	Nonsynonymous coding	32%
MM04T	FNDC7	fibronectin type III domain containing 7	CCDS44185.1	chr1_109261499-109261499_C_A	142F>L	Substitution	Nonsynonymous coding	28%

MM04T	FNIP1	folliculin interacting protein 1	CCDS34227.1	chr5_131007228-131007228_C_A	970W>L	Substitution	Nonsynonymous coding	24%
MM04T	FOCAD	focadhesin	CCDS34993.1	chr9_20720405-20720405_G_T	53E>D	Substitution	Nonsynonymous coding	18%
MM04T	FOCAD	focadhesin	CCDS34993.1	chr9_20981683-20981683_C_T	1546R>W	Substitution	Nonsynonymous coding	20%
MM04T	FOLH1B	folate hydrolase 1B	CCDS8286.1	chr11_89431707-89431707_G_T	423Q>H	Substitution	Nonsynonymous coding	26%
MM04T	FOXA1	forkhead box A1	CCDS9665.1	chr14_38060629-38060629_C_A	454A>S	Substitution	Nonsynonymous coding	23%
MM04T	FOXB2	forkhead box B2	CCDS35045.1	chr9_79634887-79634887_G_A	106R>H	Substitution	Nonsynonymous coding	23%
MM04T	FOXF2	forkhead box F2	CCDS4472.1	chr6_1394981-1394981_G_T	408D>Y	Substitution	Nonsynonymous coding	23%
MM04T	FOXN4	forkhead box N4	CCDS9126.2	chr12_109725673-109725673_G_T	148F>L	Substitution	Nonsynonymous coding	28%
MM04T	FOXN4	forkhead box N4	CCDS9126.2	chr12_109728038-109728038_G_A	47S>L	Substitution	Nonsynonymous coding	31%
MM04T	FOXO1	forkhead box O1	CCDS9371.1	chr13_41134681-41134681_C_T	316R>Q	Substitution	Nonsynonymous coding	18%
MM04T	FOXO1	forkhead box O1	CCDS9371.1	chr13_41134822-41134822_C_T	269R>Q	Substitution	Nonsynonymous coding	21%
MM04T	FOXP2	forkhead box P2	CCDS43635.1	chr7_114066702-114066702_A_G	46T>A	Substitution	Nonsynonymous coding	44%
MM04T	FOXP2	forkhead box P2	CCDS43635.1	chr7_114298221-114298221_C_T	481P>L	Substitution	Nonsynonymous coding	20%
MM04T	FOXP2	forkhead box P2	CCDS43635.1	chr7_114282503-114282503_G_T	297E>X	Substitution	Nonsense	27%
MM04T	FOXP3	forkhead box P3	CCDS14323.1	chrX_49113304-49113304_G_A	184S>L	Substitution	Nonsynonymous coding	24%
MM04T	FOXR1	forkhead box R1	CCDS31688.1	chr11_118849803-118849803_G_T	91K>N	Substitution	Nonsynonymous coding	24%
MM04T	FOXR1	forkhead box R1	CCDS31688.1	chr11_118850239-118850239_C_T	158R>W	Substitution	Nonsynonymous coding	33%
MM04T	FRAS1	Fraser syndrome 1	NM_025074	chr4_79207658-79207658_G_A	500G>E	Substitution	Nonsynonymous coding	35%
MM04T	FREM1	FRAS1 related extracellular matrix 1	CCDS47952.1	chr9_14823326-14823326_C_T	NA	Substitution	Splice site acceptor	54%
MM04T	FREM1	FRAS1 related extracellular matrix 1	CCDS47952.1	chr9_14801851-14801851_C_A	1165E>X	Substitution	Nonsense	35%
MM04T	FREM2	FRAS1 related extracellular matrix protein 2	CCDS31960.1	chr13_39357219-39357219_T_G	1885F>C	Substitution	Nonsynonymous coding	24%
MM04T	FREM3	FRAS1 related extracellular matrix 3	NM_001168235	chr4_144545418-144545418_G_T	1832F>L	Substitution	Nonsynonymous coding	18%
MM04T	FREM3	FRAS1 related extracellular matrix 3	NM_001168235	chr4_144618242-144618242_A_C	1196L>R	Substitution	Nonsynonymous coding	22%
MM04T	FREM3	FRAS1 related extracellular matrix 3	NM_001168235	chr4_144619904-144619904_A_G	642V>A	Substitution	Nonsynonymous coding	20%
MM04T	FRMD7	FERM domain containing 7	CCDS35397.1	chrX_131212303-131212303_T_C	581N>S	Substitution	Nonsynonymous coding	17%
MM04T	FRMD7	FERM domain containing 7	CCDS35397.1	chrX_131212315-131212315_G_A	577A>V	Substitution	Nonsynonymous coding	25%
MM04T	FRMPD1	FERM and PDZ domain containing 1	CCDS6612.1	chr9_37745905-37745905_G_T	1292E>D	Substitution	Nonsynonymous coding	24%
MM04T	FRMPD2	FERM and PDZ domain containing 2	CCDS31195.1	chr10_49409370-49409370_C_A	619D>Y	Substitution	Nonsynonymous coding	35%
MM04T	FRMPD2	FERM and PDZ domain containing 2	CCDS31195.1	chr10_49420125-49420125_C_T	495V>I	Substitution	Nonsynonymous coding	36%
MM04T	FRMPD2	FERM and PDZ domain containing 2	CCDS31195.1	chr10_49392633-49392633_C_A	854E>X	Substitution	Nonsense	25%
MM04T	FRMPD3	FERM and PDZ domain containing 3	ENST00000276185	chrX_106846551-106846551_C_T	1794S>L	Substitution	Nonsynonymous coding	18%
MM04T	FRRS1	ferric-chelate reductase 1	CCDS30780.1	chr1_100181216-100181216_G_A	417L>F	Substitution	Nonsynonymous coding	31%

MM04T	FRY	furry homolog (Drosophila)	ENST00000267067	chr13_32660087-32660087_G_T	36R>I	Substitution	Nonsynonymous coding	19%
MM04T	FRY	furry homolog (Drosophila)	CCDS41875.1	chr13_32721489-32721489_G_A	417R>Q	Substitution	Nonsynonymous coding	29%
MM04T	FRY	furry homolog (Drosophila)	CCDS41875.1	chr13_32863860-32863860_G_A	2854E>K	Substitution	Nonsynonymous coding	33%
MM04T	FRYL	FRY-like	CCDS43227.1	chr4_48542442-48542442_T_G	2075M>L	Substitution	Nonsynonymous coding	31%
MM04T	FRYL	FRY-like	CCDS43227.1	chr4_48549696-48549696_C_T	1660R>Q	Substitution	Nonsynonymous coding	33%
MM04T	FRZB	frizzled-related protein	CCDS2286.1	chr2_183730885-183730885_C_A	132E>D	Substitution	Nonsynonymous coding	27%
MM04T	FSCB	fibrous sheath CABYR binding protein	CCDS9679.1	chr14_44973994-44973994_C_A	733E>X	Substitution	Nonsense	27%
MM04T	FSD1	fibronectin type III and SPRY domain containing 1	CCDS12127.1	chr19_4312033-4312033_G_A	229E>K	Substitution	Nonsynonymous coding	48%
MM04T	FSD2	fibronectin type III and SPRY domain containing 2	CCDS45332.1	chr15_83440960-83440960_T_G	378N>H	Substitution	Nonsynonymous coding	22%
MM04T	FSIP2	fibrous sheath interacting protein 2	ENST00000424728	chr2_186654626-186654626_T_A	921N>K	Substitution	Nonsynonymous coding	38%
MM04T	FSIP2	fibrous sheath interacting protein 2	ENST00000424728	chr2_186654858-186654858_C_A	999L>I	Substitution	Nonsynonymous coding	32%
MM04T	FSIP2	fibrous sheath interacting protein 2	ENST00000424728	chr2_186655381-186655381_C_A	1173S>Y	Substitution	Nonsynonymous coding	27%
MM04T	FSIP2	fibrous sheath interacting protein 2	ENST00000424728	chr2_186656539-186656539_C_A	1559P>H	Substitution	Nonsynonymous coding	27%
MM04T	FSIP2	fibrous sheath interacting protein 2	ENST00000424728	chr2_186659698-186659698_A_C	2612N>T	Substitution	Nonsynonymous coding	25%
MM04T	FSIP2	fibrous sheath interacting protein 2	ENST00000424728	chr2_186664897-186664897_G_T	3622D>Y	Substitution	Nonsynonymous coding	27%
MM04T	FSIP2	fibrous sheath interacting protein 2	ENST00000424728	chr2_186665222-186665222_C_T	3730S>L	Substitution	Nonsynonymous coding	37%
MM04T	FSIP2	fibrous sheath interacting protein 2	ENST00000424728	chr2_186669545-186669545_G_A	5171R>Q	Substitution	Nonsynonymous coding	30%
MM04T	FSIP2	fibrous sheath interacting protein 2	ENST00000424728	chr2_186669641-186669641_A_C	5203E>A	Substitution	Nonsynonymous coding	30%
MM04T	FSIP2	fibrous sheath interacting protein 2	ENST00000424728	chr2_186673138-186673138_G_T	6369D>Y	Substitution	Nonsynonymous coding	24%
MM04T	FSIP2	fibrous sheath interacting protein 2	ENST00000424728	chr2_186667600-186667600_C_T	4523R>X	Substitution	Nonsense	24%
MM04T	FTMT	ferritin mitochondrial	CCDS4128.1	chr5_121188269-121188269_C_T	204S>F	Substitution	Nonsynonymous coding	32%
MM04T	FTSJ1	FtsJ RNA methyltransferase homolog 1 (E. coli)	CCDS14294.1	chrX_48340872-48340872_C_T	246S>L	Substitution	Nonsynonymous coding	22%
MM04T	FTSJ1	FtsJ RNA methyltransferase homolog 1 (E. coli)	CCDS14294.1	chrX_48339813-48339813_G_T	NA	Substitution	Splice site acceptor	22%
MM04T	FUCA1	fucosidase, alpha-L- 1, tissue	CCDS244.2	chr1_24180851-24180851_G_A	323S>L	Substitution	Nonsynonymous coding	24%
MM04T	FUK	fucokinase	CCDS10891.2	chr16_70497526-70497526_A_G	28E>G	Substitution	Nonsynonymous coding	22%
MM04T	FYTTD1	forty-two-three domain containing 1	CCDS3329.1	chr3_197476947-197476947_T_C	NA	Substitution	Splice site donor	33%
MM04T	FZD10	frizzled family receptor 10	CCDS9267.1	chr12_130648529-130648529_G_A	348E>K	Substitution	Nonsynonymous coding	21%
MM04T	FZD3	frizzled family receptor 3	CCDS6069.1	chr8_28385478-28385478_C_T	401R>X	Substitution	Nonsense	37%
MM04T	FZD4	frizzled family receptor 4	CCDS8279.1	chr11_86662230-86662230_C_T	523G>E	Substitution	Nonsynonymous coding	22%
MM04T	FZD4	frizzled family receptor 4	CCDS8279.1	chr11_86662764-86662764_G_A	345S>F	Substitution	Nonsynonymous coding	34%
MM04T	FZD6	frizzled family receptor 6	CCDS6298.1	chr8_104340629-104340629_G_A	509R>Q	Substitution	Nonsynonymous coding	27%
MM04T	FZD9	frizzled family receptor 9	CCDS5548.1	chr7_72848776-72848776_G_A	147D>N	Substitution	Nonsynonymous coding	26%

MM04T	GAA	glucosidase, alpha; acid	CCDS32760.1	chr17_78079554-78079554_G_T	185D>Y	Substitution	Nonsynonymous coding	20%
MM04T	GAB3	GRB2-associated binding protein 3	CCDS48198.1	chrX_153924244-153924244_C_A	493R>I	Substitution	Nonsynonymous coding	30%
MM04T	GAB3	GRB2-associated binding protein 3	CCDS48198.1	chrX_153925432-153925432_G_T	468L>I	Substitution	Nonsynonymous coding	24%
MM04T	GAB4	GRB2-associated binding protein family, member 4	CCDS42976.1	chr22_17468965-17468965_C_A	191E>X	Substitution	Nonsense	27%
MM04T	GABBR1	gamma-aminobutyric acid (GABA) B receptor, 1	CCDS4663.1	chr6_29589581-29589581_C_T	360R>Q	Substitution	Nonsynonymous coding	24%
MM04T	GABPA	GA binding protein transcription factor, alpha subunit 60kDa	CCDS13575.1	chr21_27117599-27117599_G_T	52K>N	Substitution	Nonsynonymous coding	43%
MM04T	GABPB1	GA binding protein transcription factor, beta subunit 1	CCDS32239.1	chr15_50581785-50581785_C_A	272V>F	Substitution	Nonsynonymous coding	20%
MM04T	GABPB1	GA binding protein transcription factor, beta subunit 1	CCDS32239.1	chr15_50596224-50596224_C_T	72R>Q	Substitution	Nonsynonymous coding	32%
MM04T	GABPB1	GA binding protein transcription factor, beta subunit 1	CCDS32239.1	chr15_50578267-50578267_C_A	345E>X	Substitution	Nonsense	21%
MM04T	GABPB2	GA binding protein transcription factor, beta subunit 2	CCDS983.1	chr1_151089933-151089933_T_G	330L>V	Substitution	Nonsynonymous coding	27%
MM04T	GABRA1	gamma-aminobutyric acid (GABA) A receptor, alpha 1	CCDS4357.1	chr5_161292738-161292738_G_A	67E>K	Substitution	Nonsynonymous coding	34%
MM04T	GABRA3	gamma-aminobutyric acid (GABA) A receptor, alpha 3	CCDS14706.1	chrX_151358324-151358324_A_T	341F>I	Substitution	Nonsynonymous coding	32%
MM04T	GABRA4	gamma-aminobutyric acid (GABA) A receptor, alpha 4	CCDS3473.1	chr4_46976343-46976343_C_A	209E>D	Substitution	Nonsynonymous coding	30%
MM04T	GABRA4	gamma-aminobutyric acid (GABA) A receptor, alpha 4	CCDS3473.1	chr4_46979596-46979596_T_G	109K>Q	Substitution	Nonsynonymous coding	28%
MM04T	GABRA6	gamma-aminobutyric acid (GABA) A receptor, alpha 6	CCDS4356.1	chr5_161117246-161117246_G_T	238R>M	Substitution	Nonsynonymous coding	19%
MM04T	GABRE	gamma-aminobutyric acid (GABA) A receptor, epsilon	CCDS14703.1	chrX_151123215-151123215_G_T	493F>L	Substitution	Nonsynonymous coding	21%
MM04T	GABRG2	gamma-aminobutyric acid (GABA) A receptor, gamma 2	CCDS47333.1	chr5_161520910-161520910_C_A	62P>T	Substitution	Nonsynonymous coding	23%
MM04T	GABRG2	gamma-aminobutyric acid (GABA) A receptor, gamma 2	ENST00000393933	chr5_161579447-161579447_G_T	407L>F	Substitution	Nonsynonymous coding	20%
MM04T	GABRG3	gamma-aminobutyric acid (GABA) A receptor, gamma 3	CCDS45195.1	chr15_27572031-27572031_C_A	116L>I	Substitution	Nonsynonymous coding	35%
MM04T	GABRG3	gamma-aminobutyric acid (GABA) A receptor, gamma 3	CCDS45195.1	chr15_27725912-27725912_G_T	231E>X	Substitution	Nonsense	31%
MM04T	GABRQ	gamma-aminobutyric acid (GABA) A receptor, theta	CCDS14707.1	chrX_151806764-151806764_C_A	36F>L	Substitution	Nonsynonymous coding	29%
MM04T	GABRQ	gamma-aminobutyric acid (GABA) A receptor, theta	CCDS14707.1	chrX_151819998-151819998_C_A	304S>X	Substitution	Nonsense	31%
MM04T	GABRR2	gamma-aminobutyric acid (GABA) A receptor, rho 2	CCDS5020.2	chr6_89967684-89967684_C_A	393G>V	Substitution	Nonsynonymous coding	24%
MM04T	GABRR2	gamma-aminobutyric acid (GABA) A receptor, rho 2	CCDS5020.2	chr6_89977482-89977482_T_G	243K>Q	Substitution	Nonsynonymous coding	26%
MM04T	GAL3ST4	galactose-3-O-sulfotransferase 4	CCDS5688.1	chr7_99758553-99758553_G_T	153F>L	Substitution	Nonsynonymous coding	48%
MM04T	GALNT1	UDP-N-acetyl-alpha-D-galactosamine:polypeptide N-	CCDS11915.1	chr18_33243594-33243594_C_A	48L>I	Substitution	Nonsynonymous coding	29%
MM04T	GALNT1	UDP-N-acetyl-alpha-D-galactosamine:polypeptide N-	CCDS11915.1	chr18_33243704-33243704_C_A	84F>L	Substitution	Nonsynonymous coding	34%
MM04T	GALNT12	UDP-N-acetyl-alpha-D-galactosamine:polypeptide N-	CCDS6737.1	chr9_101594229-101594229_G_A	303D>N	Substitution	Nonsynonymous coding	24%
MM04T	GALNT13	UDP-N-acetyl-alpha-D-galactosamine:polypeptide N-	CCDS2199.1	chr2_155252612-155252612_G_T	422Q>H	Substitution	Nonsynonymous coding	38%
MM04T	GALNT15	UDP-N-acetyl-alpha-D-galactosamine:polypeptide N-	CCDS33711.1	chr3_16252702-16252702_C_A	384S>Y	Substitution	Nonsynonymous coding	28%
MM04T	GALNT15	UDP-N-acetyl-alpha-D-galactosamine:polypeptide N-	CCDS33711.1	chr3_16252734-16252734_G_A	395E>K	Substitution	Nonsynonymous coding	29%
MM04T	GALNT3	UDP-N-acetyl-alpha-D-galactosamine:polypeptide N-	CCDS2226.1	chr2_166621450-166621450_G_T	211S>Y	Substitution	Nonsynonymous coding	30%

MM04T	GALNT5	UDP-N-acetyl-alpha-D-galactosamine:polypeptide N-	CCDS2203.1	chr2_158142583-158142583_C_A	560L>I	Substitution	Nonsynonymous coding	26%
MM04T	GALNT5	UDP-N-acetyl-alpha-D-galactosamine:polypeptide N-	CCDS2203.1	chr2_158152285-158152285_G_A	618E>K	Substitution	Nonsynonymous coding	36%
MM04T	GALNT6	UDP-N-acetyl-alpha-D-galactosamine:polypeptide N-	CCDS8813.1	chr12_51749712-51749712_G_A	545R>C	Substitution	Nonsynonymous coding	30%
MM04T	GALNT6	UDP-N-acetyl-alpha-D-galactosamine:polypeptide N-	CCDS8813.1	chr12_51758118-51758118_A_G	279L>P	Substitution	Nonsynonymous coding	39%
MM04T	GALNT8	UDP-N-acetyl-alpha-D-galactosamine:polypeptide N-	CCDS8533.1	chr12_4848451-4848451_G_A	211R>Q	Substitution	Nonsynonymous coding	34%
MM04T	GALNTL6	UDP-N-acetyl-alpha-D-galactosamine:polypeptide N-	CCDS34104.1	chr4_172735776-172735776_C_A	15F>L	Substitution	Nonsynonymous coding	29%
MM04T	GALNTL6	UDP-N-acetyl-alpha-D-galactosamine:polypeptide N-	CCDS34104.1	chr4_173873288-173873288_C_T	417A>V	Substitution	Nonsynonymous coding	23%
MM04T	GANAB	glucosidase, alpha; neutral AB	CCDS41656.1	chr11_62402409-62402409_C_A	148K>N	Substitution	Nonsynonymous coding	15%
MM04T	GANAB	glucosidase, alpha; neutral AB	CCDS41656.1	chr11_62407195-62407195_G_A	16A>V	Substitution	Nonsynonymous coding	28%
MM04T	GANC	glucosidase, alpha; neutral C	CCDS10084.1	chr15_42579971-42579971_G_A	95R>K	Substitution	Nonsynonymous coding	15%
MM04T	GART	phosphoribosylglycinamide formyltransferase, phosphoribosylglycinamide synthetase,	CCDS13627.1	chr21_34889771-34889771_G_T	616S>X	Substitution	Nonsense	25%
MM04T	GAS2L2	growth arrest-specific 2 like 2	CCDS11298.1	chr17_34072695-34072695_C_A	607E>D	Substitution	Nonsynonymous coding	19%
MM04T	GAS2L3	growth arrest-specific 2 like 3	CCDS9079.1	chr12_101016096-101016096_G_A	231R>Q	Substitution	Nonsynonymous coding	30%
MM04T	GAS2L3	growth arrest-specific 2 like 3	CCDS9079.1	chr12_101018661-101018661_G_A	693R>K	Substitution	Nonsynonymous coding	25%
MM04T	GAS8	growth arrest-specific 8	CCDS10992.1	chr16_90097762-90097762_G_A	49R>Q	Substitution	Nonsynonymous coding	19%
MM04T	GATAD2A	GATA zinc finger domain containing 2A	CCDS12402.2	chr19_19606886-19606886_C_A	261S>Y	Substitution	Nonsynonymous coding	18%
MM04T	GATM	glycine amidinotransferase (L-arginine:glycine amidinotransferase)	CCDS10122.1	chr15_45658341-45658341_A_G	294I>T	Substitution	Nonsynonymous coding	20%
MM04T	GBE1	glucan (1,4-alpha-), branching enzyme 1	NM_000158	chr3_81643160-81643160_C_A	336R>I	Substitution	Nonsynonymous coding	17%
MM04T	GBE1	glucan (1,4-alpha-), branching enzyme 1	NM_000158	chr3_81754601-81754601_C_A	103D>Y	Substitution	Nonsynonymous coding	27%
MM04T	GBF1	golgi brefeldin A resistant guanine nucleotide exchange factor 1	CCDS7533.1	chr10_104136781-104136781_C_T	1459L>F	Substitution	Nonsynonymous coding	33%
MM04T	GBP2	guanylate binding protein 2, interferon-inducible	CCDS719.1	chr1_89582672-89582672_C_A	NA	Substitution	Splice site donor	15%
MM04T	GBP5	guanylate binding protein 5	CCDS722.1	chr1_89729555-89729555_A_C	409L>R	Substitution	Nonsynonymous coding	22%
MM04T	GBP6	guanylate binding protein family, member 6	CCDS723.1	chr1_89849675-89849675_G_A	498A>T	Substitution	Nonsynonymous coding	30%
MM04T	GBP7	guanylate binding protein 7	CCDS720.1	chr1_89613339-89613339_A_C	426F>V	Substitution	Nonsynonymous coding	23%
MM04T	GC	group-specific component (vitamin D binding protein)	CCDS3550.1	chr4_72631267-72631267_G_A	119R>X	Substitution	Nonsense	32%
MM04T	GCC1	GRIP and coiled-coil domain containing 1	CCDS5796.1	chr7_127224420-127224420_C_A	273E>X	Substitution	Nonsense	24%
MM04T	GCDH	glutaryl-CoA dehydrogenase	CCDS12286.1	chr19_13008189-13008189_T_G	343I>M	Substitution	Nonsynonymous coding	33%
MM04T	GCLM	glutamate-cysteine ligase, modifier subunit	CCDS746.1	chr1_94362314-94362314_C_A	134D>Y	Substitution	Nonsynonymous coding	32%
MM04T	GCM1	glial cells missing homolog 1 (Drosophila)	CCDS4950.1	chr6_52993105-52993105_G_A	404P>S	Substitution	Nonsynonymous coding	28%
MM04T	GCNT2	glucosaminyl (N-acetyl) transferase 2, I-branching enzyme (I blood group)	ENST00000410107	chr6_10509338-10509338_T_G	5I>S	Substitution	Nonsynonymous coding	16%
MM04T	GCNT3	glucosaminyl (N-acetyl) transferase 3, mucin type	CCDS10172.1	chr15_59910945-59910945_G_T	170E>X	Substitution	Nonsense	35%
MM04T	GCSAML	germinal center-associated, signaling and motility-like	CCDS1635.1	chr1_247712513-247712513_G_A	7R>Q	Substitution	Nonsynonymous coding	21%

MM04T	GDAP2	ganglioside induced differentiation associated protein 2	CCDS897.1	chr1_118462809-118462809_G_T	58L>I	Substitution	Nonsynonymous coding	41%
MM04T	GDE1	glycerophosphodiester phosphodiesterase 1	CCDS10578.1	chr16_19514812-19514812_C_A	326D>Y	Substitution	Nonsynonymous coding	26%
MM04T	GDPD4	glycerophosphodiester phosphodiesterase domain containing 4	CCDS8249.1	chr11_76982226-76982226_C_T	117V>I	Substitution	Nonsynonymous coding	32%
MM04T	GEM	GTP binding protein overexpressed in skeletal muscle	CCDS6261.1	chr8_95264247-95264247_C_A	205E>X	Substitution	Nonsense	23%
MM04T	GEMIN4	gem (nuclear organelle) associated protein 4	CCDS45559.1	chr17_649899-649899_T_C	462I>V	Substitution	Nonsynonymous coding	34%
MM04T	GEMIN6	gem (nuclear organelle) associated protein 6	CCDS1799.1	chr2_39006163-39006163_G_T	11E>X	Substitution	Nonsense	29%
MM04T	gene108171	-	CCDS44779.1	chr11_133790683-133790683_C_A	979E>D	Substitution	Nonsynonymous coding	31%
MM04T	gene110258	-	CCDS47277.1	chr5_139231388-139231388_G_A	533R>C	Substitution	Nonsynonymous coding	29%
MM04T	gene110775	-	CCDS7035.2	chr9_140086965-140086965_G_A	574P>L	Substitution	Nonsynonymous coding	21%
MM04T	gene113070	-	CCDS47498.1	chr6_146978009-146978009_C_A	169L>I	Substitution	Nonsynonymous coding	27%
MM04T	gene113070	-	CCDS47498.1	chr6_146993556-146993556_C_A	347S>Y	Substitution	Nonsynonymous coding	18%
MM04T	gene113070	-	CCDS47498.1	chr6_147012363-147012363_C_A	475P>T	Substitution	Nonsynonymous coding	18%
MM04T	gene113070	-	CCDS47498.1	chr6_147022202-147022202_C_A	568S>Y	Substitution	Nonsynonymous coding	24%
MM04T	gene113070	-	CCDS47498.1	chr6_147105770-147105770_C_T	1351P>L	Substitution	Nonsynonymous coding	26%
MM04T	gene113070	-	CCDS47498.1	chr6_147122375-147122375_C_T	1532R>X	Substitution	Nonsense	20%
MM04T	gene113731	-	CCDS47500.1	chr6_149783017-149783017_A_C	132F>C	Substitution	Nonsynonymous coding	26%
MM04T	gene115326	-	CCDS48188.1	chrX_152612761-152612761_G_T	206E>D	Substitution	Nonsynonymous coding	17%
MM04T	gene119844	-	CCDS47516.1	chr6_168369799-168369799_A_G	1758N>D	Substitution	Nonsynonymous coding	29%
MM04T	gene121477	-	CCDS47351.1	chr5_176959459-176959459_G_T	307F>L	Substitution	Nonsynonymous coding	29%
MM04T	gene13617	-	CCDS47950.1	chr9_5921755-5921755_G_A	1414S>L	Substitution	Nonsynonymous coding	28%
MM04T	gene18417	-	CCDS47541.1	chr7_11078432-11078432_C_T	676R>X	Substitution	Nonsense	34%
MM04T	gene20083	-	CCDS44542.1	chr11_13031489-13031489_G_T	230E>D	Substitution	Nonsynonymous coding	24%
MM04T	gene21742	-	CCDS44062.1	chr1_15578359-15578359_G_T	27D>Y	Substitution	Nonsynonymous coding	14%
MM04T	gene21742	-	CCDS44062.1	chr1_15627867-15627867_C_T	282S>L	Substitution	Nonsynonymous coding	30%
MM04T	gene36146	-	CCDS47569.1	chr7_31015495-31015495_T_C	329F>S	Substitution	Nonsynonymous coding	33%
MM04T	gene37652	-	CCDS47407.1	chr6_32053722-32053722_G_A	985P>S	Substitution	Nonsynonymous coding	30%
MM04T	gene46946	-	CCDS47423.1	chr6_39880742-39880742_T_G	255K>T	Substitution	Nonsynonymous coding	13%
MM04T	gene47378	-	CCDS46074.1	chr19_40276356-40276356_C_T	30R>C	Substitution	Nonsynonymous coding	33%
MM04T	gene49600	-	CCDS45242.1	chr15_42281737-42281737_G_T	504F>L	Substitution	Nonsynonymous coding	17%
MM04T	gene49600	-	CCDS45242.1	chr15_42281631-42281631_G_A	540R>X	Substitution	Nonsense	36%
MM04T	gene53629	-	CCDS44574.1	chr11_44928026-44928026_T_C	20I>T	Substitution	Nonsynonymous coding	29%
MM04T	gene53629	-	CCDS44574.1	chr11_44940825-44940825_G_T	132D>Y	Substitution	Nonsynonymous coding	16%

MM04T	gene53658	-	CCDS46810.1	chr3_44975457-44975457_G_A	151R>W	Substitution	Nonsynonymous coding	25%
MM04T	gene66631	-	CCDS47441.1	chr6_56394248-56394248_G_A	3746R>W	Substitution	Nonsynonymous coding	36%
MM04T	gene66631	-	CCDS47441.1	chr6_56418264-56418264_G_A	2990A>V	Substitution	Nonsynonymous coding	34%
MM04T	gene66631	-	CCDS47441.1	chr6_56497765-56497765_A_T	1198L>Q	Substitution	Nonsynonymous coding	18%
MM04T	gene66631	-	CCDS47441.1	chr6_56505384-56505384_C_T	650E>K	Substitution	Nonsynonymous coding	34%
MM04T	gene66631	-	CCDS47441.1	chr6_56569164-56569164_G_A	231R>X	Substitution	Nonsense	26%
MM04T	gene66761	-	CCDS47444.1	chr6_56470020-56470020_T_C	2599N>D	Substitution	Nonsynonymous coding	24%
MM04T	gene66761	-	CCDS47444.1	chr6_56471108-56471108_A_G	2236V>A	Substitution	Nonsynonymous coding	35%
MM04T	gene66761	-	CCDS47444.1	chr6_56471408-56471408_C_T	2136G>E	Substitution	Nonsynonymous coding	23%
MM04T	gene66761	-	CCDS47444.1	chr6_56473377-56473377_C_A	1480D>Y	Substitution	Nonsynonymous coding	21%
MM04T	gene87951	-	CCDS44706.1	chr11_92543142-92543142__C	NA	Insertion	Frameshift	24%
MM04T	gene87958	-	CCDS47890.1	chr8_92213000-92213000_C_T	305R>X	Substitution	Nonsense	34%
MM04T	gene90027	-	CCDS46363.1	chr2_97315943-97315943_G_A	141A>T	Substitution	Nonsynonymous coding	28%
MM04T	gene98833	-	CCDS44978.1	chr12_112620871-112620871_G_T	3238S>X	Substitution	Nonsense	31%
MM04T	GFM1	G elongation factor, mitochondrial 1	CCDS33885.1	chr3_158371243-158371243_C_A	329L>I	Substitution	Nonsynonymous coding	25%
MM04T	GFRA4	GDNF family receptor alpha 4	CCDS13056.1	chr20_3644024-3644024_G_A	8A>V	Substitution	Nonsynonymous coding	20%
MM04T	GGACT	gamma-glutamylamine cyclotransferase	CCDS45066.1	chr13_101184632-101184632_C_T	72E>K	Substitution	Nonsynonymous coding	38%
MM04T	GGH	gamma-glutamyl hydrolase (conjugase, folylpolygammaglutamyl hydrolase)	CCDS6177.1	chr8_63951232-63951232_C_A	32K>N	Substitution	Nonsynonymous coding	24%
MM04T	GGN	gametogenetin	CCDS12516.1	chr19_38876870-38876870_G_T	344F>L	Substitution	Nonsynonymous coding	24%
MM04T	GGN	gametogenetin	CCDS12516.1	chr19_38877165-38877165_G_A	246T>I	Substitution	Nonsynonymous coding	29%
MM04T	GGNBP2	gametogenetin binding protein 2	CCDS11314.1	chr17_34934591-34934591_C_T	274R>C	Substitution	Nonsynonymous coding	23%
MM04T	GGT5	gamma-glutamyltransferase 5	CCDS42990.1	chr22_24628154-24628154_C_A	207E>X	Substitution	Nonsense	38%
MM04T	GHITM	growth hormone inducible transmembrane protein	CCDS41542.1	chr10_85901297-85901297_C_A	14S>Y	Substitution	Nonsynonymous coding	15%
MM04T	GHR	growth hormone receptor	CCDS3940.1	chr5_42699988-42699988_C_A	168H>N	Substitution	Nonsynonymous coding	31%
MM04T	GIMAP6	GTPase, IMAP family member 6	CCDS34778.1	chr7_150327195-150327195_C_A	12E>D	Substitution	Nonsynonymous coding	17%
MM04T	GIMAP8	GTPase, IMAP family member 8	CCDS34777.1	chr7_150164346-150164346_G_A	187R>H	Substitution	Nonsynonymous coding	19%
MM04T	GIN1	gypsy retrotransposon integrase 1 [Source:HGNC Symbol;Acc:25959]	CCDS43349.1	chr5_102442572-102442572_G_A	61R>C	Substitution	Nonsynonymous coding	34%
MM04T	GINM1	glycoprotein integral membrane 1	CCDS5216.1	chr6_149900009-149900009_T_C	110V>A	Substitution	Nonsynonymous coding	25%
MM04T	GJA1	gap junction protein, alpha 1, 43kDa	CCDS5123.1	chr6_121768036-121768036_C_A	15Q>K	Substitution	Nonsynonymous coding	19%
MM04T	GJA1	gap junction protein, alpha 1, 43kDa	CCDS5123.1	chr6_121768871-121768871_G_T	293R>I	Substitution	Nonsynonymous coding	25%
MM04T	GJA10	gap junction protein, alpha 10, 62kDa	CCDS5025.1	chr6_90605470-90605470_G_A	428R>H	Substitution	Nonsynonymous coding	37%
MM04T	GJA10	gap junction protein, alpha 10, 62kDa	CCDS5025.1	chr6_90605791-90605791_C_T	535S>L	Substitution	Nonsynonymous coding	44%

MM04T	GJA9	gap junction protein, alpha 9, 59kDa	CCDS432.1	chr1_39340657-39340657_C_A	372E>X	Substitution	Nonsense	33%
MM04T	GJB5	gap junction protein, beta 5, 31.1kDa	CCDS382.1	chr1_35223665-35223665_C_A	245S>X	Substitution	Nonsense	32%
MM04T	GJC2	gap junction protein, gamma 2, 47kDa	CCDS1569.1	chr1_228345773-228345773_C_T	105A>V	Substitution	Nonsynonymous coding	35%
MM04T	GJD4	gap junction protein, delta 4, 40.1kDa	CCDS7191.1	chr10_35896538-35896538_C_T	33R>W	Substitution	Nonsynonymous coding	27%
MM04T	GK2	glycerol kinase 2	CCDS3585.1	chr4_80327898-80327898_C_T	486R>Q	Substitution	Nonsynonymous coding	34%
MM04T	GLDC	glycine dehydrogenase (decarboxylating)	CCDS34987.1	chr9_6550844-6550844_C_T	843R>Q	Substitution	Nonsynonymous coding	47%
MM04T	GLI1	GLI family zinc finger 1	CCDS8940.1	chr12_57865444-57865444_T_G	974F>C	Substitution	Nonsynonymous coding	35%
MM04T	GLI2	GLI family zinc finger 2	CCDS33283.1	chr2_121740331-121740331_C_A	520L>M	Substitution	Nonsynonymous coding	36%
MM04T	GLIS3	GLIS family zinc finger 3	CCDS43784.1	chr9_3856125-3856125_G_T	786S>Y	Substitution	Nonsynonymous coding	32%
MM04T	GLIS3	GLIS family zinc finger 3	CCDS43784.1	chr9_4286331-4286331_C_T	32R>Q	Substitution	Nonsynonymous coding	21%
MM04T	GLRA2	glycine receptor, alpha 2	CCDS14160.1	chrX_14748438-14748438_T_C	397L>P	Substitution	Nonsynonymous coding	24%
MM04T	GLRA2	glycine receptor, alpha 2	CCDS14160.1	chrX_14748472-14748472_G_T	408K>N	Substitution	Nonsynonymous coding	34%
MM04T	GLYATL2	glycine-N-acyltransferase-like 2	CCDS41649.1	chr11_58604780-58604780_T_C	93N>D	Substitution	Nonsynonymous coding	38%
MM04T	GMCL1	germ cell-less, spermatogenesis associated 1	CCDS1895.1	chr2_70064682-70064682_A_C	88K>N	Substitution	Nonsynonymous coding	59%
MM04T	GMDS	GDP-mannose 4,6-dehydratase	CCDS4474.1	chr6_2117747-2117747_C_T	64R>Q	Substitution	Nonsynonymous coding	31%
MM04T	GMIP	GEM interacting protein	CCDS12408.1	chr19_19745609-19745609_G_T	627L>I	Substitution	Nonsynonymous coding	21%
MM04T	GMIP	GEM interacting protein	CCDS12408.1	chr19_19747781-19747781_C_A	394E>D	Substitution	Nonsynonymous coding	31%
MM04T	GMPR2	guanosine monophosphate reductase 2	CCDS45087.1	chr14_24702536-24702536_C_T	44S>F	Substitution	Nonsynonymous coding	19%
MM04T	GNAI2	guanine nucleotide binding protein (G protein), alpha inhibiting activity polypeptide 2	ENST00000422163	chr3_50264558-50264558_G_T	1M>I	Substitution	Nonsynonymous coding	19%
MM04T	GNAL	guanine nucleotide binding protein (G protein), alpha activating activity polypeptide, olfactory	CCDS11851.1	chr18_11876666-11876666_C_A	403F>L	Substitution	Nonsynonymous coding	24%
MM04T	GNAT2	guanine nucleotide binding protein (G protein), alpha transducing activity polypeptide 2	CCDS803.1	chr1_110146617-110146617_T_G	277K>T	Substitution	Nonsynonymous coding	35%
MM04T	GNAT2	guanine nucleotide binding protein (G protein), alpha transducing activity polypeptide 2	CCDS803.1	chr1_110149030-110149030_C_A	164D>Y	Substitution	Nonsynonymous coding	21%
MM04T	GNB2	guanine nucleotide binding protein (G protein), beta polypeptide 2	CCDS5703.1	chr7_100276326-100276326_G_A	309A>T	Substitution	Nonsynonymous coding	29%
MM04T	GNB4	guanine nucleotide binding protein (G protein), beta polypeptide 4	CCDS3230.1	chr3_179137294-179137294_C_A	NA	Substitution	Splice site acceptor	37%
MM04T	GNE	glucosamine (UDP-N-acetyl)-2-epimerase/N-acetylmannosamine kinase	CCDS47965.1	chr9_36227371-36227371_C_A	416K>N	Substitution	Nonsynonymous coding	51%
MM04T	GNG11	guanine nucleotide binding protein (G protein), gamma 11	CCDS5634.1	chr7_93555429-93555429_G_T	41K>N	Substitution	Nonsynonymous coding	28%
MM04T	GNL3L	guanine nucleotide binding protein-like 3 (nucleolar)-like	CCDS14360.1	chrX_54566666-54566666_G_T	60K>N	Substitution	Nonsynonymous coding	21%
MM04T	GNPAT	glyceronephosphate O-acyltransferase	CCDS1592.1	chr1_231411898-231411898_T_C	652F>S	Substitution	Nonsynonymous coding	48%
MM04T	GNPDA2	glucosamine-6-phosphate deaminase 2	CCDS3469.1	chr4_44719145-44719145_C_A	132D>Y	Substitution	Nonsynonymous coding	29%
MM04T	GNRHR	gonadotropin-releasing hormone receptor	CCDS3517.1	chr4_68619803-68619803_G_A	84T>I	Substitution	Nonsynonymous coding	31%
MM04T	GOLGA1	golgin A1	CCDS6860.1	chr9_127690532-127690532_T_G	112T>P	Substitution	Nonsynonymous coding	30%
MM04T	GOLGA2B	-	ENST00000397112	chr19_22785631-22785631_G_T	136G>X	Substitution	Nonsense	25%

MM04T	GOLGA4	golgin A4	CCDS2666.1	chr3_37340385-37340385_G_T	292E>D	Substitution	Nonsynonymous coding	28%
MM04T	GOLGA4	golgin A4	CCDS2666.1	chr3_37381630-37381630_G_T	2138D>Y	Substitution	Nonsynonymous coding	31%
MM04T	GOLGA5	golgin A5	CCDS9905.1	chr14_93263907-93263907_A_G	42Y>C	Substitution	Nonsynonymous coding	18%
MM04T	GOLGA5	golgin A5	CCDS9905.1	chr14_93299530-93299530_A_G	595T>A	Substitution	Nonsynonymous coding	22%
MM04T	GOLM1	golgi membrane protein 1	CCDS35054.1	chr9_88648293-88648293_G_T	345L>M	Substitution	Nonsynonymous coding	38%
MM04T	GORAB	golgin, RAB6-interacting	CCDS1289.1	chr1_170521160-170521160_C_T	248R>W	Substitution	Nonsynonymous coding	29%
MM04T	GPAT2	glycerol-3-phosphate acyltransferase 2, mitochondrial	CCDS42714.1	chr2_96690403-96690403_A_G	481F>L	Substitution	Nonsynonymous coding	22%
MM04T	GPATCH1	G patch domain containing 1	CCDS12428.1	chr19_33581757-33581757_T_G	94F>V	Substitution	Nonsynonymous coding	36%
MM04T	GPBAR1	G protein-coupled bile acid receptor 1	CCDS46515.1	chr2_219127819-219127819_T_G	124I>M	Substitution	Nonsynonymous coding	20%
MM04T	GPC4	glypican 4	CCDS14637.1	chrX_132445371-132445371_C_A	264K>N	Substitution	Nonsynonymous coding	24%
MM04T	GPC5	glypican 5	CCDS9468.1	chr13_92345783-92345783_G_A	223R>H	Substitution	Nonsynonymous coding	30%
MM04T	GPC5	glypican 5	CCDS9468.1	chr13_92408643-92408643_C_T	417P>S	Substitution	Nonsynonymous coding	38%
MM04T	GPCPD1	glycerophosphocholine phosphodiesterase GDE1 homolog (S. cerevisiae)	CCDS13090.1	chr20_5566857-5566857_C_T	97R>Q	Substitution	Nonsynonymous coding	32%
MM04T	GPI	glucose-6-phosphate isomerase	CCDS12437.1	chr19_34857754-34857754_G_A	94E>K	Substitution	Nonsynonymous coding	22%
MM04T	GPI	glucose-6-phosphate isomerase	CCDS12437.1	chr19_34859545-34859545_G_T	114D>Y	Substitution	Nonsynonymous coding	24%
MM04T	GPM6A	glycoprotein M6A	CCDS3824.1	chr4_176561305-176561305_C_A	220G>V	Substitution	Nonsynonymous coding	26%
MM04T	GPR110	G protein-coupled receptor 110	ENST00000283297	chr6_46995504-46995504_A_C	NA	Substitution	Splice site acceptor	35%
MM04T	GPR112	G protein-coupled receptor 112	CCDS35409.1	chrX_135430677-135430677_A_C	1604Q>H	Substitution	Nonsynonymous coding	36%
MM04T	GPR112	G protein-coupled receptor 112	CCDS35409.1	chrX_135482015-135482015_G_A	2772R>Q	Substitution	Nonsynonymous coding	36%
MM04T	GPR115	G protein-coupled receptor 115	CCDS4922.2	chr6_47682464-47682464_G_T	495A>S	Substitution	Nonsynonymous coding	28%
MM04T	GPR116	G protein-coupled receptor 116	CCDS4919.1	chr6_46828546-46828546_T_C	762E>G	Substitution	Nonsynonymous coding	26%
MM04T	GPR119	G protein-coupled receptor 119	CCDS14625.1	chrX_129518951-129518951_G_T	157F>L	Substitution	Nonsynonymous coding	29%
MM04T	GPR124	G protein-coupled receptor 124	CCDS6097.2	chr8_37698660-37698660_T_A	935I>N	Substitution	Nonsynonymous coding	11%
MM04T	GPR149	G protein-coupled receptor 149	CCDS43162.1	chr3_154146705-154146705_G_A	234R>C	Substitution	Nonsynonymous coding	14%
MM04T	GPR153	G protein-coupled receptor 153	CCDS64.1	chr1_6311472-6311472_C_T	302R>H	Substitution	Nonsynonymous coding	33%
MM04T	GPR158	G protein-coupled receptor 158	CCDS31166.1	chr10_25888190-25888190_G_A	1212S>N	Substitution	Nonsynonymous coding	30%
MM04T	GPR158	G protein-coupled receptor 158	CCDS31166.1	chr10_25684861-25684861_G_T	344G>X	Substitution	Nonsense	19%
MM04T	GPR158	G protein-coupled receptor 158	CCDS31166.1	chr10_25887373-25887373_G_T	940E>X	Substitution	Nonsense	28%
MM04T	GPR161	G protein-coupled receptor 161	CCDS1268.1	chr1_168073927-168073927_C_A	54K>N	Substitution	Nonsynonymous coding	35%
MM04T	GPR173	G protein-coupled receptor 173	CCDS14349.1	chrX_53106905-53106905_G_T	368E>X	Substitution	Nonsense	23%
MM04T	GPR179	G protein-coupled receptor 179	CCDS42308.1	chr17_36483298-36483298_A_C	2052S>A	Substitution	Nonsynonymous coding	33%
MM04T	GPR179	G protein-coupled receptor 179	CCDS42308.1	chr17_36486010-36486010_C_T	1148D>N	Substitution	Nonsynonymous coding	32%

MM04T	GPR179	G protein-coupled receptor 179	CCDS42308.1	chr17_36491108-36491108_G_T	485L>M	Substitution	Nonsynonymous coding	15%
MM04T	GPR179	G protein-coupled receptor 179	CCDS42308.1	chr17_36499575-36499575_C_T	33R>H	Substitution	Nonsynonymous coding	22%
MM04T	GPR18	G protein-coupled receptor 18	CCDS9491.1	chr13_99907181-99907181_G_A	316R>X	Substitution	Nonsense	11%
MM04T	GPR183	G protein-coupled receptor 183	CCDS9492.1	chr13_99947658-99947658_G_T	248L>I	Substitution	Nonsynonymous coding	34%
MM04T	GPR22	G protein-coupled receptor 22	CCDS5744.1	chr7_107115431-107115431_A_G	309K>R	Substitution	Nonsynonymous coding	21%
MM04T	GPR25	G protein-coupled receptor 25	CCDS1405.1	chr1_200843016-200843016_C_T	284A>V	Substitution	Nonsynonymous coding	32%
MM04T	GPR31	G protein-coupled receptor 31	CCDS5299.1	chr6_167571076-167571076_G_T	82L>M	Substitution	Nonsynonymous coding	15%
MM04T	GPR56	G protein-coupled receptor 56	CCDS32460.1	chr16_57690154-57690154_A_C	345Q>P	Substitution	Nonsynonymous coding	21%
MM04T	GPR64	G protein-coupled receptor 64	CCDS43923.1	chrX_19014241-19014241_C_T	882A>T	Substitution	Nonsynonymous coding	17%
MM04T	GPR65	G protein-coupled receptor 65	CCDS9879.1	chr14_88477859-88477859_G_T	223R>I	Substitution	Nonsynonymous coding	33%
MM04T	GPR78	G protein-coupled receptor 78	CCDS3403.1	chr4_8582878-8582878_G_A	57A>T	Substitution	Nonsynonymous coding	30%
MM04T	GPR84	G protein-coupled receptor 84	CCDS8878.1	chr12_54756785-54756785_T_G	284K>T	Substitution	Nonsynonymous coding	35%
MM04T	GPR98	G protein-coupled receptor 98	CCDS47246.1	chr5_89990200-89990200_C_A	2543L>I	Substitution	Nonsynonymous coding	21%
MM04T	GPR98	G protein-coupled receptor 98	CCDS47246.1	chr5_90055281-90055281_A_C	3999E>A	Substitution	Nonsynonymous coding	23%
MM04T	GPR98	G protein-coupled receptor 98	CCDS47246.1	chr5_90281336-90281336_A_G	6050D>G	Substitution	Nonsynonymous coding	46%
MM04T	GPR98	G protein-coupled receptor 98	CCDS47246.1	chr5_89924386-89924386_G_T	416E>X	Substitution	Nonsense	35%
MM04T	GPR98	G protein-coupled receptor 98	CCDS47246.1	chr5_90046392-90046392_G_T	3667E>X	Substitution	Nonsense	26%
MM04T	GPR98	G protein-coupled receptor 98	CCDS47246.1	chr5_90087050-90087050_C_T	4802R>X	Substitution	Nonsense	36%
MM04T	GPRASP1	G protein-coupled receptor associated sorting protein 1	CCDS35352.1	chrX_101910604-101910604_A_G	588N>S	Substitution	Nonsynonymous coding	26%
MM04T	GPRASP1	G protein-coupled receptor associated sorting protein 1	CCDS35352.1	chrX_101911100-101911100_C_A	753F>L	Substitution	Nonsynonymous coding	42%
MM04T	GPRASP1	G protein-coupled receptor associated sorting protein 1	CCDS35352.1	chrX_101912809-101912809_T_C	1323I>T	Substitution	Nonsynonymous coding	18%
MM04T	GPRASP1	G protein-coupled receptor associated sorting protein 1	CCDS35352.1	chrX_101909853-101909853_G_T	338E>X	Substitution	Nonsense	18%
MM04T	GPRASP1	G protein-coupled receptor associated sorting protein 1	CCDS35352.1	chrX_101912217-101912217_C_T	1126R>X	Substitution	Nonsense	31%
MM04T	GPRC5C	G protein-coupled receptor, family C, group 5, member C	CCDS11699.1	chr17_72435928-72435928_A_C	50K>Q	Substitution	Nonsynonymous coding	10%
MM04T	GPRIN1	G protein regulated inducer of neurite outgrowth 1	CCDS4405.1	chr5_176025989-176025989_G_T	283L>I	Substitution	Nonsynonymous coding	32%
MM04T	GPSM1	G-protein signaling modulator 1	CCDS6996.2	chr9_139235587-139235587_C_A	448F>L	Substitution	Nonsynonymous coding	22%
MM04T	GRAMD3	GRAM domain containing 3	CCDS4136.1	chr5_125808980-125808980_G_T	136E>X	Substitution	Nonsense	17%
MM04T	GRAP2	GRB2-related adaptor protein 2	CCDS13999.1	chr22_40343165-40343165_T_G	19F>V	Substitution	Nonsynonymous coding	26%
MM04T	GRB7	growth factor receptor-bound protein 7	CCDS11345.1	chr17_37898636-37898636_C_A	28P>T	Substitution	Nonsynonymous coding	21%
MM04T	GRB7	growth factor receptor-bound protein 7	CCDS11345.1	chr17_37899534-37899534_G_A	189E>K	Substitution	Nonsynonymous coding	28%
MM04T	GRHL1	grainyhead-like 1 (Drosophila)	CCDS33144.1	chr2_10136459-10136459_G_A	347R>Q	Substitution	Nonsynonymous coding	26%
MM04T	GRHL3	grainyhead-like 3 (Drosophila)	CCDS251.1	chr1_24664178-24664178_G_T	252E>X	Substitution	Nonsense	14%

MM04T	GRIA2	glutamate receptor, ionotropic, AMPA 2	CCDS3797.1	chr4_158255184-158255184_A_G	393D>G	Substitution	Nonsynonymous coding	24%
MM04T	GRIA3	glutamate receptor, ionotropic, AMPA 3	NM_000828	chrX_122536902-122536902_A_G	380N>D	Substitution	Nonsynonymous coding	23%
MM04T	GRID2	glutamate receptor, ionotropic, delta 2	CCDS3637.1	chr4_93225827-93225827_T_C	7L>P	Substitution	Nonsynonymous coding	38%
MM04T	GRIK1	glutamate receptor, ionotropic, kainate 1	CCDS42913.1	chr21_30959782-30959782_C_T	566G>D	Substitution	Nonsynonymous coding	36%
MM04T	GRIK3	glutamate receptor, ionotropic, kainate 3	CCDS416.1	chr1_37291365-37291365_G_T	531F>L	Substitution	Nonsynonymous coding	23%
MM04T	GRIN2A	glutamate receptor, ionotropic, N-methyl D-aspartate 2A	CCDS10539.1	chr16_9858109-9858109_A_C	1098C>G	Substitution	Nonsynonymous coding	38%
MM04T	GRIN2B	glutamate receptor, ionotropic, N-methyl D-aspartate 2B	CCDS8662.1	chr12_13717012-13717012_C_T	1054D>N	Substitution	Nonsynonymous coding	29%
MM04T	GRIN2B	glutamate receptor, ionotropic, N-methyl D-aspartate 2B	CCDS8662.1	chr12_13717395-13717395_C_T	926R>Q	Substitution	Nonsynonymous coding	21%
MM04T	GRIN2B	glutamate receptor, ionotropic, N-methyl D-aspartate 2B	CCDS8662.1	chr12_13768108-13768108_T_C	532T>A	Substitution	Nonsynonymous coding	33%
MM04T	GRIN2C	glutamate receptor, ionotropic, N-methyl D-aspartate 2C	CCDS32724.1	chr17_72850914-72850914_C_A	106Q>H	Substitution	Nonsynonymous coding	32%
MM04T	GRIN3A	glutamate receptor, ionotropic, N-methyl-D-aspartate 3A	CCDS6758.1	chr9_104340125-104340125_C_A	978R>I	Substitution	Nonsynonymous coding	24%
MM04T	GRK7	G protein-coupled receptor kinase 7	CCDS3120.1	chr3_141535792-141535792_T_G	521I>S	Substitution	Nonsynonymous coding	37%
MM04T	GRM1	glutamate receptor, metabotropic 1	CCDS5209.1	chr6_146625808-146625808_A_G	338I>V	Substitution	Nonsynonymous coding	30%
MM04T	GRM3	glutamate receptor, metabotropic 3	CCDS5600.1	chr7_86415763-86415763_G_A	219E>K	Substitution	Nonsynonymous coding	23%
MM04T	GRM3	glutamate receptor, metabotropic 3	CCDS5600.1	chr7_86416261-86416261_G_A	385E>K	Substitution	Nonsynonymous coding	32%
MM04T	GRM5	glutamate receptor, metabotropic 5	CCDS44694.1	chr11_88583093-88583093_C_A	298E>X	Substitution	Nonsense	17%
MM04T	GRM7	glutamate receptor, metabotropic 7	CCDS43042.1	chr3_7188172-7188172_G_A	185E>K	Substitution	Nonsynonymous coding	24%
MM04T	GRM7	glutamate receptor, metabotropic 7	CCDS43042.1	chr3_7620188-7620188_G_T	532R>I	Substitution	Nonsynonymous coding	16%
MM04T	GRM8	glutamate receptor, metabotropic 8	CCDS47696.1	chr7_126746696-126746696_C_T	194R>Q	Substitution	Nonsynonymous coding	27%
MM04T	GRN	granulin	CCDS11483.1	chr17_42427676-42427676_G_A	144D>N	Substitution	Nonsynonymous coding	23%
MM04T	GRPR	gastrin-releasing peptide receptor	CCDS14174.1	chrX_16142263-16142263_A_G	63K>E	Substitution	Nonsynonymous coding	30%
MM04T	GRXCR1	glutaredoxin, cysteine rich 1	CCDS43225.1	chr4_42964963-42964963_C_T	147R>C	Substitution	Nonsynonymous coding	28%
MM04T	GSDMD	gasdermin D	CCDS34956.1	chr8_144644686-144644686_G_A	403E>K	Substitution	Nonsynonymous coding	39%
MM04T	GSG1L	GSG1-like	CCDS45450.1	chr16_28074612-28074612_C_T	45G>D	Substitution	Nonsynonymous coding	56%
MM04T	GSG2	germ cell associated 2 (haspin)	CCDS11036.1	chr17_3628966-3628966_C_A	579H>Q	Substitution	Nonsynonymous coding	11%
MM04T	GSK3A	glycogen synthase kinase 3 alpha	CCDS12599.1	chr19_42744209-42744209_T_G	123K>N	Substitution	Nonsynonymous coding	34%
MM04T	GSPT1	G1 to S phase transition 1	CCDS45412.1	chr16_11980398-11980398_G_T	395H>N	Substitution	Nonsynonymous coding	22%
MM04T	GSPT1	G1 to S phase transition 1	CCDS45412.1	chr16_12009375-12009375_T_C	68Q>R	Substitution	Nonsynonymous coding	21%
MM04T	GSPT1	G1 to S phase transition 1	CCDS45412.1	chr16_11980461-11980461_C_A	374E>X	Substitution	Nonsense	35%
MM04T	GSPT2	G1 to S phase transition 2	CCDS14336.1	chrX_51488590-51488590_T_C	623L>S	Substitution	Nonsynonymous coding	34%
MM04T	GSTCD	glutathione S-transferase, C-terminal domain containing	CCDS43257.1	chr4_106640280-106640280_G_T	164E>X	Substitution	Nonsense	19%
MM04T	GSX2	GS homeobox 2	CCDS3494.1	chr4_54968021-54968021_C_T	283R>C	Substitution	Nonsynonymous coding	33%

MM04T	GTF2H3	general transcription factor IIH, polypeptide 3, 34kDa	CCDS9252.1	chr12_124130104-124130104_G_T	66E>X	Substitution	Nonsense	16%
MM04T	GTF3A	general transcription factor IIIA	CCDS45019.1	chr13_28004752-28004752_T_G	161L>R	Substitution	Nonsynonymous coding	40%
MM04T	GTF3C3	general transcription factor IIIC, polypeptide 3, 102kDa	CCDS2316.1	chr2_197629381-197629381_C_T	856R>Q	Substitution	Nonsynonymous coding	31%
MM04T	GTPBP1	GTP binding protein 1	ENST00000456823	chr22_39130353-39130353_G_A	287E>K	Substitution	Nonsynonymous coding	25%
MM04T	GTPBP3	GTP binding protein 3 (mitochondrial)	CCDS32950.1	chr19_17449057-17449057_G_A	98W>X	Substitution	Nonsense	20%
MM04T	GTPBP5	GTP binding protein 5 (putative)	CCDS13492.1	chr20_60775978-60775978_C_A	356L>M	Substitution	Nonsynonymous coding	11%
MM04T	GTPBP8	GTP-binding protein 8 (putative)	CCDS33820.1	chr3_112715818-112715818_G_T	216E>X	Substitution	Nonsense	33%
MM04T	GUCA1B	guanylate cyclase activator 1B (retina)	CCDS4865.1	chr6_42162391-42162391_C_A	56Q>H	Substitution	Nonsynonymous coding	31%
MM04T	GUCY1B3	guanylate cyclase 1, soluble, beta 3	CCDS47154.1	chr4_156721102-156721102_G_A	351D>N	Substitution	Nonsynonymous coding	29%
MM04T	GUCY1B3	guanylate cyclase 1, soluble, beta 3	CCDS47154.1	chr4_156698783-156698783_G_T	96E>X	Substitution	Nonsense	24%
MM04T	GUCY2F	guanylate cyclase 2F, retinal	CCDS14545.1	chrX_108625355-108625355_C_A	1048E>X	Substitution	Nonsense	30%
MM04T	GUF1	GUF1 GTPase homolog (S. cerevisiae)	CCDS3468.1	chr4_44684393-44684393_G_A	184E>K	Substitution	Nonsynonymous coding	30%
MM04T	GUF1	GUF1 GTPase homolog (S. cerevisiae)	CCDS3468.1	chr4_44688678-44688678_G_A	296E>K	Substitution	Nonsynonymous coding	20%
MM04T	GULP1	GULP, engulfment adaptor PTB domain containing 1	ENST00000451191	chr2_189452778-189452778_C_T	140S>L	Substitution	Nonsynonymous coding	27%
MM04T	GUSB	glucuronidase, beta	CCDS5530.1	chr7_65444847-65444847_C_T	150E>K	Substitution	Nonsynonymous coding	17%
MM04T	GXYLT1	glucoside xylosyltransferase 1	CCDS41772.1	chr12_42499642-42499642_C_T	281R>Q	Substitution	Nonsynonymous coding	25%
MM04T	GXYLT2	glucoside xylosyltransferase 2	CCDS46870.1	chr3_73004303-73004303_C_A	219L>M	Substitution	Nonsynonymous coding	19%
MM04T	GYS2	glycogen synthase 2 (liver)	CCDS8690.1	chr12_21689995-21689995_C_T	669D>N	Substitution	Nonsynonymous coding	28%
MM04T	GYS2	glycogen synthase 2 (liver)	CCDS8690.1	chr12_21721885-21721885_C_T	246R>Q	Substitution	Nonsynonymous coding	28%
MM04T	GYS2	glycogen synthase 2 (liver)	CCDS8690.1	chr12_21757463-21757463_C_A	22E>X	Substitution	Nonsense	35%
MM04T	HAPLN3	hyaluronan and proteoglycan link protein 3	CCDS10346.1	chr15_89424858-89424858_C_T	75E>K	Substitution	Nonsynonymous coding	22%
MM04T	HARBI1	harbinger transposase derived 1	CCDS7920.1	chr11_46637333-46637333_T_C	152H>R	Substitution	Nonsynonymous coding	23%
MM04T	HARS2	histidyl-tRNA synthetase 2, mitochondrial	CCDS4238.1	chr5_140073592-140073592_A_C	86K>Q	Substitution	Nonsynonymous coding	34%
MM04T	HAS1	hyaluronan synthase 1	CCDS12838.1	chr19_52219609-52219609_G_T	321L>I	Substitution	Nonsynonymous coding	26%
MM04T	HAS2	hyaluronan synthase 2	CCDS6335.1	chr8_122641121-122641121_C_T	154E>K	Substitution	Nonsynonymous coding	35%
MM04T	HAT1	histone acetyltransferase 1	CCDS2245.1	chr2_172841226-172841226_A_C	318Q>H	Substitution	Nonsynonymous coding	40%
MM04T	HAUS3	HAUS augmin-like complex, subunit 3	CCDS33941.1	chr4_2241917-2241917_C_A	253E>X	Substitution	Nonsense	39%
MM04T	HBP1	HMG-box transcription factor 1	CCDS5741.1	chr7_106830762-106830762_G_T	356S>I	Substitution	Nonsynonymous coding	38%
MM04T	HBS1L	HBS1-like (S. cerevisiae)	CCDS47480.1	chr6_135359128-135359128_T_G	156N>T	Substitution	Nonsynonymous coding	34%
MM04T	HCCS	holocytochrome c synthase	CCDS14139.1	chrX_11139035-11139035_C_A	177P>H	Substitution	Nonsynonymous coding	27%
MM04T	HCFC2	host cell factor C2	CCDS9097.1	chr12_104474523-104474523_G_T	NA	Substitution	Splice site acceptor	33%
MM04T	HCFC2	host cell factor C2	CCDS9097.1	chr12_104495872-104495872_G_T	669E>X	Substitution	Nonsense	29%

MM04T	HCN1	hyperpolarization activated cyclic nucleotide-gated potassium channel 1	CCDS3952.1	chr5_45303871-45303871_A_C	483F>C	Substitution	Nonsynonymous coding	26%
MM04T	HCN1	hyperpolarization activated cyclic nucleotide-gated potassium channel 1	CCDS3952.1	chr5_45396619-45396619_G_T	402S>Y	Substitution	Nonsynonymous coding	35%
MM04T	HDAC2	histone deacetylase 2	CCDS43493.1	chr6_114264630-114264630_G_T	515F>L	Substitution	Nonsynonymous coding	26%
MM04T	HDAC9	histone deacetylase 9	CCDS47553.1	chr7_18801793-18801793_G_A	686R>Q	Substitution	Nonsynonymous coding	23%
MM04T	HDC	histidine decarboxylase	CCDS10134.1	chr15_50555478-50555478_C_A	53S>I	Substitution	Nonsynonymous coding	20%
MM04T	HDLBP	high density lipoprotein binding protein	CCDS2547.1	chr2_242179456-242179456_G_A	751R>C	Substitution	Nonsynonymous coding	19%
MM04T	HDX	highly divergent homeobox	CCDS35342.1	chrX_83724090-83724090_G_T	214S>Y	Substitution	Nonsynonymous coding	26%
MM04T	HDX	highly divergent homeobox	CCDS35342.1	chrX_83723479-83723479_C_T	NA	Substitution	Splice site donor	57%
MM04T	HDX	highly divergent homeobox	CCDS35342.1	chrX_83723731-83723731_G_A	334R>X	Substitution	Nonsense	26%
MM04T	HEATR1	HEAT repeat containing 1	CCDS31066.1	chr1_236730160-236730160_G_A	1365S>F	Substitution	Nonsynonymous coding	31%
MM04T	HEATR2	HEAT repeat containing 2	CCDS34580.1	chr7_814733-814733_A_G	725I>V	Substitution	Nonsynonymous coding	44%
MM04T	HEATR5B	HEAT repeat containing 5B	CCDS33181.1	chr2_37217868-37217868_C_A	1874E>X	Substitution	Nonsense	32%
MM04T	HEATR6	HEAT repeat containing 6	CCDS11623.1	chr17_58143584-58143584_C_A	468D>Y	Substitution	Nonsynonymous coding	28%
MM04T	HECTD1	HECT domain containing E3 ubiquitin protein ligase 1	CCDS41939.1	chr14_31605847-31605847_G_A	1002R>W	Substitution	Nonsynonymous coding	33%
MM04T	HECTD1	HECT domain containing E3 ubiquitin protein ligase 1	CCDS41939.1	chr14_31638652-31638652_T_G	452N>T	Substitution	Nonsynonymous coding	34%
MM04T	HECW1	HECT, C2 and WW domain containing E3 ubiquitin protein ligase 1	CCDS5469.2	chr7_43495908-43495908_G_A	838R>Q	Substitution	Nonsynonymous coding	27%
MM04T	HECW1	HECT, C2 and WW domain containing E3 ubiquitin protein ligase 1	CCDS5469.2	chr7_43519324-43519324_G_A	1072R>Q	Substitution	Nonsynonymous coding	43%
MM04T	HECW1	HECT, C2 and WW domain containing E3 ubiquitin protein ligase 1	CCDS5469.2	chr7_43547676-43547676_C_T	1271S>L	Substitution	Nonsynonymous coding	15%
MM04T	HECW2	HECT, C2 and WW domain containing E3 ubiquitin protein ligase 2	CCDS33354.1	chr2_197297945-197297945_T_C	68Y>C	Substitution	Nonsynonymous coding	34%
MM04T	HEG1	HEG homolog 1 (zebrafish)	CCDS46898.1	chr3_124748066-124748066_C_T	195A>T	Substitution	Nonsynonymous coding	20%
MM04T	HELB	helicase (DNA) B	CCDS8976.1	chr12_66698787-66698787_A_C	155K>T	Substitution	Nonsynonymous coding	39%
MM04T	HELB	helicase (DNA) B	CCDS8976.1	chr12_66707833-66707833_C_T	583S>L	Substitution	Nonsynonymous coding	19%
MM04T	HELB	helicase (DNA) B	CCDS8976.1	chr12_66725416-66725416_A_C	1051K>N	Substitution	Nonsynonymous coding	20%
MM04T	HELQ	helicase, POLQ-like	CCDS3603.1	chr4_84352867-84352867_C_A	749E>X	Substitution	Nonsense	18%
MM04T	HELZ2	helicase with zinc finger 2, transcriptional coactivator	CCDS33508.1	chr20_62192621-62192621_C_A	2320K>N	Substitution	Nonsynonymous coding	31%
MM04T	HEMGN	hemogen	CCDS6731.1	chr9_100689730-100689730_C_T	464G>E	Substitution	Nonsynonymous coding	33%
MM04T	HEMGN	hemogen	CCDS6731.1	chr9_100692770-100692770_C_A	303E>X	Substitution	Nonsense	35%
MM04T	HEPH	hephaestin	CCDS14384.2	chrX_65423421-65423421_T_A	NA	Substitution	Splice site donor	36%
MM04T	HERC1	HECT and RLD domain containing E3 ubiquitin protein ligase family member 1	CCDS45277.1	chr15_64067184-64067184_C_A	213K>N	Substitution	Nonsynonymous coding	24%
MM04T	HERC1	HECT and RLD domain containing E3 ubiquitin protein ligase family member 1	CCDS45277.1	chr15_64067336-64067336_G_A	163R>X	Substitution	Nonsense	32%
MM04T	HERC2	HECT and RLD domain containing E3 ubiquitin protein ligase 2	CCDS10021.1	chr15_28380691-28380691_A_T	4055S>T	Substitution	Nonsynonymous coding	24%
MM04T	HERC4	HECT and RLD domain containing E3 ubiquitin protein ligase 4	CCDS41533.1	chr10_69714442-69714442_A_C	757F>L	Substitution	Nonsynonymous coding	30%

MM04T	HES3	hairy and enhancer of split 3 (Drosophila)	CCDS41238.1	chr1_6305557-6305557_A_C	184D>A	Substitution	Nonsynonymous coding	28%
MM04T	HES6	hairy and enhancer of split 6 (Drosophila)	CCDS2527.1	chr2_239147539-239147539_C_T	202D>N	Substitution	Nonsynonymous coding	22%
MM04T	HEXB	hexosaminidase B (beta polypeptide)	CCDS4022.1	chr5_74016520-74016520_G_T	521D>Y	Substitution	Nonsynonymous coding	34%
MM04T	HFM1	HFM1, ATP-dependent DNA helicase homolog (S. cerevisiae)	CCDS30769.2	chr1_91739271-91739271_T_G	1257K>T	Substitution	Nonsynonymous coding	18%
MM04T	HFM1	HFM1, ATP-dependent DNA helicase homolog (S. cerevisiae)	CCDS30769.2	chr1_91843622-91843622_C_T	452R>Q	Substitution	Nonsynonymous coding	15%
MM04T	HFM1	HFM1, ATP-dependent DNA helicase homolog (S. cerevisiae)	CCDS30769.2	chr1_91850783-91850783_C_T	255E>K	Substitution	Nonsynonymous coding	22%
MM04T	HGF	hepatocyte growth factor (hepapoietin A; scatter factor)	CCDS5597.1	chr7_81350155-81350155_G_A	393R>C	Substitution	Nonsynonymous coding	34%
MM04T	HHEX	hematopoietically expressed homeobox	CCDS7423.1	chr10_94454401-94454401_C_A	230S>Y	Substitution	Nonsynonymous coding	30%
MM04T	HHIPL1	HHIP-like 1	CCDS45162.1	chr14_100125980-100125980_G_A	421G>D	Substitution	Nonsynonymous coding	24%
MM04T	HHLA2	HERV-H LTR-associating 2	CCDS46883.1	chr3_108072288-108072288_G_A	27A>T	Substitution	Nonsynonymous coding	14%
MM04T	HHLA2	HERV-H LTR-associating 2	CCDS46883.1	chr3_108081216-108081216_A_G	344N>S	Substitution	Nonsynonymous coding	36%
MM04T	HIATL1	hippocampus abundant transcript-like 1	CCDS6710.2	chr9_97207300-97207300_C_A	189L>I	Substitution	Nonsynonymous coding	29%
MM04T	HIBADH	3-hydroxyisobutyrate dehydrogenase	CCDS5414.1	chr7_27565880-27565880_C_A	322D>Y	Substitution	Nonsynonymous coding	25%
MM04T	HIF1A	hypoxia inducible factor 1, alpha subunit (basic helix-loop-helix transcription factor)	CCDS9753.1	chr14_62199143-62199143_G_A	261E>K	Substitution	Nonsynonymous coding	26%
MM04T	HIF1A	hypoxia inducible factor 1, alpha subunit (basic helix-loop-helix transcription factor)	CCDS9753.1	chr14_62188520-62188520_G_T	146E>X	Substitution	Nonsense	25%
MM04T	HIF3A	hypoxia inducible factor 3, alpha subunit	CCDS12681.2	chr19_46815513-46815513_G_T	289S>I	Substitution	Nonsynonymous coding	18%
MM04T	HIRA	HIR histone cell cycle regulation defective homolog A (S. cerevisiae)	CCDS13759.1	chr22_19343313-19343313_G_A	844A>V	Substitution	Nonsynonymous coding	26%
MM04T	HIST3H3	histone cluster 3, H3	CCDS1572.1	chr1_228612911-228612911_G_A	39P>L	Substitution	Nonsynonymous coding	35%
MM04T	HIVEP1	human immunodeficiency virus type I enhancer binding protein 1	CCDS43426.1	chr6_12122336-12122336_C_T	770R>W	Substitution	Nonsynonymous coding	20%
MM04T	HIVEP2	human immunodeficiency virus type I enhancer binding protein 2	CCDS43510.1	chr6_143094230-143094230_G_T	549S>Y	Substitution	Nonsynonymous coding	32%
MM04T	HIVEP2	human immunodeficiency virus type I enhancer binding protein 2	CCDS43510.1	chr6_143091387-143091387_G_A	1497R>X	Substitution	Nonsense	24%
MM04T	HIVEP3	human immunodeficiency virus type I enhancer binding protein 3	CCDS463.1	chr1_41990576-41990576_T_G	1738K>T	Substitution	Nonsynonymous coding	10%
MM04T	HK3	hexokinase 3 (white cell)	CCDS4407.1	chr5_176308744-176308744_C_T	781R>H	Substitution	Nonsynonymous coding	22%
MM04T	HK3	hexokinase 3 (white cell)	CCDS4407.1	chr5_176314494-176314494_G_A	520R>C	Substitution	Nonsynonymous coding	36%
MM04T	HLA-DPB1	major histocompatibility complex, class II, DP beta 1	CCDS4765.1	chr6_33048501-33048501_G_T	51Q>H	Substitution	Nonsynonymous coding	18%
MM04T	HLA-DQB2	major histocompatibility complex, class II, DQ beta 2	ENST00000435145	chr6_32729596-32729596_C_T	69D>N	Substitution	Nonsynonymous coding	22%
MM04T	HLCS	holocarboxylase synthetase (biotin-(proprionyl-CoA-carboxylase (ATP-hydrolysing)) ligase)	CCDS13647.1	chr21_38302646-38302646_C_T	362E>K	Substitution	Nonsynonymous coding	26%
MM04T	HLTF	helicase-like transcription factor	CCDS33875.1	chr3_148781299-148781299_C_A	360C>F	Substitution	Nonsynonymous coding	26%
MM04T	HLX	H2.0-like homeobox	CCDS1527.1	chr1_221053230-221053230_G_A	11A>T	Substitution	Nonsynonymous coding	17%
MM04T	HMCN1	hemicentin 1	CCDS30956.1	chr1_185891543-185891543_T_G	311I>M	Substitution	Nonsynonymous coding	31%
MM04T	HMCN1	hemicentin 1	CCDS30956.1	chr1_185892612-185892612_C_A	371S>Y	Substitution	Nonsynonymous coding	20%
MM04T	HMCN1	hemicentin 1	CCDS30956.1	chr1_185947027-185947027_G_A	827R>Q	Substitution	Nonsynonymous coding	29%

MM04T	HMCN1	hemicentin 1	CCDS30956.1	chr1_185953370-185953370_G_T	954D>Y	Substitution	Nonsynonymous coding	20%
MM04T	HMCN1	hemicentin 1	CCDS30956.1	chr1_185959567-185959567_C_A	1123F>L	Substitution	Nonsynonymous coding	43%
MM04T	HMCN1	hemicentin 1	CCDS30956.1	chr1_186026374-186026374_A_C	2385S>R	Substitution	Nonsynonymous coding	15%
MM04T	HMCN1	hemicentin 1	CCDS30956.1	chr1_186056768-186056768_G_T	3118K>N	Substitution	Nonsynonymous coding	23%
MM04T	HMCN1	hemicentin 1	CCDS30956.1	chr1_186097404-186097404_C_A	4295F>L	Substitution	Nonsynonymous coding	24%
MM04T	HMCN2	hemicentin 2	ENST00000428715	chr9_133302751-133302751_C_T	354A>V	Substitution	Nonsynonymous coding	21%
MM04T	HMGB3	high mobility group box 3	CCDS35428.1	chrX_150154093-150154093_G_T	7K>N	Substitution	Nonsynonymous coding	56%
MM04T	HMGCL	3-hydroxymethyl-3-methylglutaryl-CoA lyase	CCDS243.1	chr1_24130918-24130918_T_C	283Y>C	Substitution	Nonsynonymous coding	26%
MM04T	HMGCLL1	3-hydroxymethyl-3-methylglutaryl-CoA lyase-like 1	CCDS43475.1	chr6_55406573-55406573_G_A	114S>F	Substitution	Nonsynonymous coding	38%
MM04T	HMGCLL1	3-hydroxymethyl-3-methylglutaryl-CoA lyase-like 1	CCDS43475.1	chr6_55381356-55381356_C_A	145G>X	Substitution	Nonsense	31%
MM04T	HNF4G	hepatocyte nuclear factor 4, gamma	CCDS6220.2	chr8_76459877-76459877_C_T	105R>X	Substitution	Nonsense	32%
MM04T	HNRNPD	heterogeneous nuclear ribonucleoprotein D (AU-rich element RNA binding protein 1,	CCDS3592.1	chr4_83280644-83280644_C_A	147E>X	Substitution	Nonsense	40%
MM04T	HNRNPH2	heterogeneous nuclear ribonucleoprotein H2 (H')	ENST00000392994	chrX_100650474-100650474_C_A	110S>Y	Substitution	Nonsynonymous coding	16%
MM04T	HNRNPH2	heterogeneous nuclear ribonucleoprotein H2 (H')	CCDS14485.1	chrX_100667572-100667572_G_A	199R>Q	Substitution	Nonsynonymous coding	19%
MM04T	HOMEZ	homeobox and leucine zipper encoding	CCDS45085.1	chr14_23745179-23745179_G_A	420R>W	Substitution	Nonsynonymous coding	31%
MM04T	HORMAD1	HORMA domain containing 1	CCDS967.1	chr1_150676642-150676642_T_G	300E>D	Substitution	Nonsynonymous coding	12%
MM04T	HORMAD2	HORMA domain containing 2	CCDS46683.1	chr22_30517746-30517746_T_G	179V>G	Substitution	Nonsynonymous coding	36%
MM04T	HOXA5	homeobox A5	CCDS5406.1	chr7_27181514-27181514_T_G	251K>N	Substitution	Nonsynonymous coding	42%
MM04T	HOXA6	homeobox A6	CCDS5407.1	chr7_27187364-27187364_C_A	2S>I	Substitution	Nonsynonymous coding	31%
MM04T	HOXA9	homeobox A9	CCDS5409.1	chr7_27204563-27204563_C_T	172E>K	Substitution	Nonsynonymous coding	26%
MM04T	HOXA9	homeobox A9	CCDS5409.1	chr7_27204614-27204614_T_G	155T>P	Substitution	Nonsynonymous coding	22%
MM04T	HOXB13	homeobox B13	CCDS11536.1	chr17_46805910-46805910_C_T	16E>K	Substitution	Nonsynonymous coding	20%
MM04T	HOXC11	homeobox C11	CCDS8867.1	chr12_54369028-54369028_G_A	249R>Q	Substitution	Nonsynonymous coding	24%
MM04T	HOXD10	homeobox D10	CCDS2266.1	chr2_176981935-176981935_C_T	125S>L	Substitution	Nonsynonymous coding	34%
MM04T	HOXD4	homeobox D4	CCDS2269.1	chr2_177017416-177017416_G_A	172E>K	Substitution	Nonsynonymous coding	35%
MM04T	HP1BP3	heterochromatin protein 1, binding protein 3	CCDS30621.1	chr1_21097473-21097473_G_T	202L>I	Substitution	Nonsynonymous coding	34%
MM04T	HPS3	Hermansky-Pudlak syndrome 3	CCDS3140.1	chr3_148858918-148858918_C_A	276S>Y	Substitution	Nonsynonymous coding	20%
MM04T	HPS3	Hermansky-Pudlak syndrome 3	CCDS3140.1	chr3_148880088-148880088_A_T	754I>F	Substitution	Nonsynonymous coding	22%
MM04T	HPS3	Hermansky-Pudlak syndrome 3	CCDS3140.1	chr3_148885021-148885021_G_T	930E>D	Substitution	Nonsynonymous coding	20%
MM04T	HPSE	heparanase	CCDS3602.1	chr4_84240582-84240582_C_A	138E>D	Substitution	Nonsynonymous coding	19%
MM04T	HPSE2	heparanase 2	CCDS7477.1	chr10_100503657-100503657_G_T	256S>Y	Substitution	Nonsynonymous coding	28%
MM04T	HR	hair growth associated	CCDS6022.1	chr8_21979186-21979186_G_A	715P>S	Substitution	Nonsynonymous coding	14%

MM04T	HRASLS2	HRAS-like suppressor 2	CCDS8046.1	chr11_63326111-63326111_G_A	47A>V	Substitution	Nonsynonymous coding	36%
MM04T	HRASLS5	HRAS-like suppressor family, member 5	CCDS8044.1	chr11_63258364-63258364_T_C	48H>R	Substitution	Nonsynonymous coding	30%
MM04T	HRG	histidine-rich glycoprotein	CCDS3280.1	chr3_186383945-186383945_G_A	42R>Q	Substitution	Nonsynonymous coding	41%
MM04T	HRH3	histamine receptor H3	CCDS13493.1	chr20_60791356-60791356_A_C	348F>L	Substitution	Nonsynonymous coding	17%
MM04T	HRH3	histamine receptor H3	CCDS13493.1	chr20_60791389-60791389_C_A	337M>I	Substitution	Nonsynonymous coding	30%
MM04T	HS6ST2	heparan sulfate 6-O-sulfotransferase 2	CCDS48170.1	chrX_131803228-131803228_C_T	NA	Substitution	Splice site acceptor	10%
MM04T	HSD11B1	hydroxysteroid (11-beta) dehydrogenase 1	CCDS1489.1	chr1_209905783-209905783_A_C	174K>Q	Substitution	Nonsynonymous coding	20%
MM04T	HSD17B11	hydroxysteroid (17-beta) dehydrogenase 11	CCDS3619.1	chr4_88312095-88312095_C_T	43G>E	Substitution	Nonsynonymous coding	34%
MM04T	HSD17B6	hydroxysteroid (17-beta) dehydrogenase 6	CCDS8925.1	chr12_57178787-57178787_G_T	241Q>H	Substitution	Nonsynonymous coding	19%
MM04T	HSPA12A	heat shock 70kDa protein 12A	CCDS41569.1	chr10_118440761-118440761_T_G	310K>T	Substitution	Nonsynonymous coding	33%
MM04T	HSPA13	heat shock protein 70kDa family, member 13	CCDS13567.1	chr21_15753601-15753601_A_G	97Y>H	Substitution	Nonsynonymous coding	33%
MM04T	HSPA14	heat shock 70kDa protein 14	ENST00000309584	chr10_14885802-14885802_G_A	243R>H	Substitution	Nonsynonymous coding	31%
MM04T	HSPA1B	heat shock 70kDa protein 1B	CCDS34415.1	chr6_31797636-31797636_A_G	637I>V	Substitution	Nonsynonymous coding	19%
MM04T	HSPA1L	heat shock 70kDa protein 1-like	CCDS34413.1	chr6_31779419-31779419_C_A	111G>W	Substitution	Nonsynonymous coding	25%
MM04T	HSPA6	heat shock 70kDa protein 6 (HSP70B')	CCDS1231.1	chr1_161495419-161495419_G_A	324R>Q	Substitution	Nonsynonymous coding	29%
MM04T	HSPBP1	HSPA (heat shock 70kDa) binding protein, cytoplasmic cochaperone 1	CCDS33111.1	chr19_55790814-55790814_C_T	55A>T	Substitution	Nonsynonymous coding	32%
MM04T	HSPH1	heat shock 105kDa/110kDa protein 1	CCDS9340.1	chr13_31725127-31725127_C_A	289E>X	Substitution	Nonsense	36%
MM04T	HTATSF1	HIV-1 Tat specific factor 1	CCDS14657.1	chrX_135593147-135593147_G_T	415E>X	Substitution	Nonsense	28%
MM04T	HTR1D	5-hydroxytryptamine (serotonin) receptor 1D, G protein-coupled	CCDS231.1	chr1_23520183-23520183_C_T	177R>Q	Substitution	Nonsynonymous coding	34%
MM04T	HTR1F	5-hydroxytryptamine (serotonin) receptor 1F, G protein-coupled	CCDS2920.1	chr3_88040836-88040836_G_T	313E>X	Substitution	Nonsense	26%
MM04T	HTR3A	5-hydroxytryptamine (serotonin) receptor 3A, ionotropic	CCDS8366.1	chr11_113853966-113853966_G_A	167D>N	Substitution	Nonsynonymous coding	22%
MM04T	HTRA3	HtrA serine peptidase 3	CCDS3400.1	chr4_8293130-8293130_G_A	248A>T	Substitution	Nonsynonymous coding	30%
MM04T	HTT	huntingtin	CCDS43206.1	chr4_3134553-3134553_C_A	803S>Y	Substitution	Nonsynonymous coding	36%
MM04T	HUNK	hormonally up-regulated Neu-associated kinase	CCDS13610.1	chr21_33246160-33246160_T_G	58V>G	Substitution	Nonsynonymous coding	18%
MM04T	HUWE1	HECT, UBA and WWE domain containing 1, E3 ubiquitin protein ligase	CCDS35301.1	chrX_53562429-53562429_G_A	4189L>F	Substitution	Nonsynonymous coding	26%
MM04T	HUWE1	HECT, UBA and WWE domain containing 1, E3 ubiquitin protein ligase	CCDS35301.1	chrX_53613750-53613750_C_A	1578Q>H	Substitution	Nonsynonymous coding	18%
MM04T	HUWE1	HECT, UBA and WWE domain containing 1, E3 ubiquitin protein ligase	CCDS35301.1	chrX_53620565-53620565_A_C	NA	Substitution	Splice site acceptor	24%
MM04T	HYDIN	HYDIN, axonemal central pair apparatus protein	CCDS42189.1	chr16_70841545-70841545_G_T	5101P>T	Substitution	Nonsynonymous coding	21%
MM04T	HYDIN	HYDIN, axonemal central pair apparatus protein	CCDS42189.1	chr16_70996009-70996009_T_C	1940T>A	Substitution	Nonsynonymous coding	20%
MM04T	HYOU1	hypoxia up-regulated 1	CCDS8408.1	chr11_118916313-118916313_C_T	998E>K	Substitution	Nonsynonymous coding	24%
MM04T	IBTK	inhibitor of Bruton agammaglobulinemia tyrosine kinase	CCDS34490.1	chr6_82924385-82924385_A_C	588F>C	Substitution	Nonsynonymous coding	30%
MM04T	IBTK	inhibitor of Bruton agammaglobulinemia tyrosine kinase	CCDS34490.1	chr6_82927697-82927697_C_A	469R>I	Substitution	Nonsynonymous coding	32%

MM04T	IBTK	inhibitor of Bruton agammaglobulinemia tyrosine kinase	CCDS34490.1	chr6_82927763-82927763_C_A	447R>I	Substitution	Nonsynonymous coding	39%
MM04T	ICA1	islet cell autoantigen 1, 69kDa	ENST00000407906	chr7_8196715-8196715_G_T	279L>I	Substitution	Nonsynonymous coding	27%
MM04T	ICAM5	intercellular adhesion molecule 5, telencephalin	CCDS12233.1	chr19_10404565-10404565_G_A	553E>K	Substitution	Nonsynonymous coding	22%
MM04T	ICOS	inducible T-cell co-stimulator	CCDS2363.1	chr2_204822595-204822595_C_A	192S>Y	Substitution	Nonsynonymous coding	33%
MM04T	IDE	insulin-degrading enzyme	CCDS7421.1	chr10_94333743-94333743_C_T	12A>T	Substitution	Nonsynonymous coding	20%
MM04T	IDH1	isocitrate dehydrogenase 1 (NADP+), soluble	CCDS2381.1	chr2_209113362-209113362_G_A	49R>C	Substitution	Nonsynonymous coding	27%
MM04T	IDO1	indoleamine 2,3-dioxygenase 1	CCDS47847.1	chr8_39782263-39782263_T_G	227F>V	Substitution	Nonsynonymous coding	29%
MM04T	IDS	iduronate 2-sulfatase	CCDS14685.1	chrX_148564622-148564622_C_A	436K>N	Substitution	Nonsynonymous coding	28%
MM04T	IDS	iduronate 2-sulfatase	CCDS14685.1	chrX_148568548-148568548_A_G	363V>A	Substitution	Nonsynonymous coding	23%
MM04T	IFI44	interferon-induced protein 44	CCDS688.1	chr1_79125147-79125147_C_T	331R>X	Substitution	Nonsense	38%
MM04T	IFI44L	interferon-induced protein 44-like	CCDS687.2	chr1_79107149-79107149_T_G	393I>M	Substitution	Nonsynonymous coding	22%
MM04T	IFIH1	interferon induced with helicase C domain 1	CCDS2217.1	chr2_163174463-163174463_T_C	119N>D	Substitution	Nonsynonymous coding	23%
MM04T	IFIT1B	interferon-induced protein with tetratricopeptide repeats 1B	CCDS31242.1	chr10_91143083-91143083_T_C	5S>P	Substitution	Nonsynonymous coding	35%
MM04T	IFIT1B	interferon-induced protein with tetratricopeptide repeats 1B	CCDS31242.1	chr10_91144010-91144010_G_T	314D>Y	Substitution	Nonsynonymous coding	23%
MM04T	IFIT3	interferon-induced protein with tetratricopeptide repeats 3	CCDS31241.1	chr10_91098985-91098985_C_A	191F>L	Substitution	Nonsynonymous coding	25%
MM04T	IFIT5	interferon-induced protein with tetratricopeptide repeats 5	CCDS7403.1	chr10_91177745-91177745_T_G	263N>K	Substitution	Nonsynonymous coding	11%
MM04T	IFIT5	interferon-induced protein with tetratricopeptide repeats 5	CCDS7403.1	chr10_91177764-91177764_G_A	270E>K	Substitution	Nonsynonymous coding	38%
MM04T	IFIT5	interferon-induced protein with tetratricopeptide repeats 5	CCDS7403.1	chr10_91178219-91178219_G_T	421K>N	Substitution	Nonsynonymous coding	31%
MM04T	IFLTD1	intermediate filament tail domain containing 1	CCDS44849.1	chr12_25656731-25656731_G_T	398S>Y	Substitution	Nonsynonymous coding	10%
MM04T	IFNAR2	interferon (alpha, beta and omega) receptor 2	CCDS13621.1	chr21_34625004-34625004_T_G	193F>C	Substitution	Nonsynonymous coding	60%
MM04T	IFRD1	interferon-related developmental regulator 1	CCDS34736.1	chr7_112095898-112095898_G_A	59D>N	Substitution	Nonsynonymous coding	30%
MM04T	IFT122	intraflagellar transport 122 homolog (Chlamydomonas)	CCDS3060.1	chr3_129196861-129196861_C_T	435R>W	Substitution	Nonsynonymous coding	28%
MM04T	IFT140	intraflagellar transport 140 homolog (Chlamydomonas)	CCDS10439.1	chr16_1639635-1639635_G_T	261P>T	Substitution	Nonsynonymous coding	31%
MM04T	IFT172	intraflagellar transport 172 homolog (Chlamydomonas)	CCDS1755.1	chr2_27682583-27682583_C_T	879E>K	Substitution	Nonsynonymous coding	31%
MM04T	IFT172	intraflagellar transport 172 homolog (Chlamydomonas)	CCDS1755.1	chr2_27688345-27688345_T_C	624T>A	Substitution	Nonsynonymous coding	32%
MM04T	IFT172	intraflagellar transport 172 homolog (Chlamydomonas)	CCDS1755.1	chr2_27688378-27688378_C_T	613A>T	Substitution	Nonsynonymous coding	25%
MM04T	IFT80	intraflagellar transport 80 homolog (Chlamydomonas)	CCDS3188.1	chr3_160099334-160099334_C_A	72K>N	Substitution	Nonsynonymous coding	37%
MM04T	IGBP1	immunoglobulin (CD79A) binding protein 1	CCDS14396.1	chrX_69354660-69354660_T_G	159I>R	Substitution	Nonsynonymous coding	18%
MM04T	IGF1	insulin-like growth factor 1 (somatomedin C)	CCDS44962.1	chr12_102869478-102869478_C_T	55G>R	Substitution	Nonsynonymous coding	17%
MM04T	IGF1	insulin-like growth factor 1 (somatomedin C)	CCDS44962.1	chr12_102874103-102874103_G_T	19F>L	Substitution	Nonsynonymous coding	23%
MM04T	IGF2BP1	insulin-like growth factor 2 mRNA binding protein 1	CCDS11543.1	chr17_47122352-47122352_G_T	NA	Substitution	Splice site acceptor	40%
MM04T	IGF2BP2	insulin-like growth factor 2 mRNA binding protein 2	CCDS3273.2	chr3_185404958-185404958_C_A	233E>D	Substitution	Nonsynonymous coding	28%

MM04T	IGLC6	immunoglobulin lambda constant 6 (Kern+Oz-marker, gene/pseudogene)	ENST00000441579	chr22_23241847-23241847_C_A	130L>I	Substitution	Nonsynonymous coding	26%
MM04T	IGSF1	immunoglobulin superfamily, member 1	CCDS14629.1	chrX_130419407-130419407_G_C	138A>G	Substitution	Nonsynonymous coding	25%
MM04T	IGSF10	immunoglobulin superfamily, member 10	CCDS3160.1	chr3_151154525-151154525_C_A	2608K>N	Substitution	Nonsynonymous coding	19%
MM04T	IGSF10	immunoglobulin superfamily, member 10	CCDS3160.1	chr3_151171420-151171420_C_T	156R>H	Substitution	Nonsynonymous coding	23%
MM04T	IGSF11	immunoglobulin superfamily, member 11	CCDS46891.1	chr3_118623613-118623613_C_A	246A>S	Substitution	Nonsynonymous coding	32%
MM04T	IHH	Indian hedgehog	CCDS33380.1	chr2_219922296-219922296_A_C	146Y>D	Substitution	Nonsynonymous coding	30%
MM04T	IKBIP	IKBKB interacting protein	CCDS9068.1	chr12_99019992-99019992_G_T	284L>I	Substitution	Nonsynonymous coding	36%
MM04T	IKZF2	IKAROS family zinc finger 2 (Helios)	CCDS2395.1	chr2_213872794-213872794_G_A	291R>X	Substitution	Nonsense	51%
MM04T	IL12A	interleukin 12A (natural killer cell stimulatory factor 1, cytotoxic lymphocyte maturation	CCDS3187.1	chr3_159711381-159711381_G_T	149K>N	Substitution	Nonsynonymous coding	34%
MM04T	IL12RB2	interleukin 12 receptor, beta 2	CCDS638.1	chr1_67795371-67795371_C_T	256R>X	Substitution	Nonsense	26%
MM04T	IL13RA1	interleukin 13 receptor, alpha 1	CCDS14573.1	chrX_117880942-117880942_C_A	85S>X	Substitution	Nonsense	26%
MM04T	IL15	interleukin 15	CCDS3755.1	chr4_142643142-142643142_A_C	59K>T	Substitution	Nonsynonymous coding	26%
MM04T	IL17RB	interleukin 17 receptor B	CCDS2874.1	chr3_53899235-53899235_A_C	470K>T	Substitution	Nonsynonymous coding	40%
MM04T	IL17RD	interleukin 17 receptor D	CCDS2880.2	chr3_57144333-57144333_T_G	106E>A	Substitution	Nonsynonymous coding	18%
MM04T	IL1B	interleukin 1, beta	CCDS2102.1	chr2_113593781-113593781_C_T	9S>N	Substitution	Nonsynonymous coding	49%
MM04T	IL1R1	interleukin 1 receptor, type I	CCDS2055.1	chr2_102793183-102793183_G_T	558E>D	Substitution	Nonsynonymous coding	18%
MM04T	IL1RAPL1	interleukin 1 receptor accessory protein-like 1	CCDS14218.1	chrX_29935666-29935666_A_C	288K>N	Substitution	Nonsynonymous coding	21%
MM04T	IL1RAPL1	interleukin 1 receptor accessory protein-like 1	CCDS14218.1	chrX_29973371-29973371_C_A	509L>I	Substitution	Nonsynonymous coding	34%
MM04T	IL1RAPL2	interleukin 1 receptor accessory protein-like 2	CCDS14517.1	chrX_105011094-105011094_G_T	501E>X	Substitution	Nonsense	34%
MM04T	IL20RB	interleukin 20 receptor beta	CCDS3093.1	chr3_136701165-136701165_C_A	127L>M	Substitution	Nonsynonymous coding	19%
MM04T	IL20RB	interleukin 20 receptor beta	CCDS3093.1	chr3_136701184-136701184_G_T	133R>I	Substitution	Nonsynonymous coding	21%
MM04T	IL20RB	interleukin 20 receptor beta	CCDS3093.1	chr3_136714279-136714279_G_A	236A>T	Substitution	Nonsynonymous coding	37%
MM04T	IL31RA	interleukin 31 receptor A	CCDS3970.2	chr5_55210731-55210731_C_T	598A>V	Substitution	Nonsynonymous coding	29%
MM04T	IL36A	interleukin 36, alpha	CCDS42734.1	chr2_113764264-113764264_C_A	72L>I	Substitution	Nonsynonymous coding	33%
MM04T	IL36B	interleukin 36, beta	CCDS2110.1	chr2_113785523-113785523_C_T	144G>D	Substitution	Nonsynonymous coding	34%
MM04T	IL4	interleukin 4	CCDS4158.1	chr5_132009858-132009858_A_T	39N>I	Substitution	Nonsynonymous coding	18%
MM04T	IL5	interleukin 5 (colony-stimulating factor, eosinophil)	CCDS4156.1	chr5_131877541-131877541_G_A	123L>F	Substitution	Nonsynonymous coding	26%
MM04T	IL7	interleukin 7	CCDS6224.1	chr8_79650870-79650870_C_A	77E>X	Substitution	Nonsense	42%
MM04T	IL7R	interleukin 7 receptor	CCDS3911.1	chr5_35857117-35857117_C_A	13S>Y	Substitution	Nonsynonymous coding	34%
MM04T	IL7R	interleukin 7 receptor	CCDS3911.1	chr5_35871274-35871274_G_A	166D>N	Substitution	Nonsynonymous coding	37%
MM04T	ILF3	interleukin enhancer binding factor 3, 90kDa	CCDS45965.1	chr19_10791772-10791772_G_T	345K>N	Substitution	Nonsynonymous coding	21%
MM04T	ILF3	interleukin enhancer binding factor 3, 90kDa	CCDS45965.1	chr19_10794354-10794354_G_A	636E>K	Substitution	Nonsynonymous coding	32%

MM04T	IMPDH2	IMP (inosine 5'-monophosphate) dehydrogenase 2	CCDS2786.1	chr3_49061827-49061827_G_A	512R>W	Substitution	Nonsynonymous coding	27%
MM04T	IMPG2	interphotoreceptor matrix proteoglycan 2	CCDS2940.1	chr3_100951783-100951783_A_C	1025C>W	Substitution	Nonsynonymous coding	29%
MM04T	IMPG2	interphotoreceptor matrix proteoglycan 2	CCDS2940.1	chr3_100948226-100948226_C_A	1211E>X	Substitution	Nonsense	15%
MM04T	INADL	InaD-like (Drosophila)	CCDS617.2	chr1_62367063-62367063_A_C	1105K>Q	Substitution	Nonsynonymous coding	19%
MM04T	INHBC	inhibin, beta C	CCDS8938.1	chr12_57843436-57843436_A_C	230K>N	Substitution	Nonsynonymous coding	24%
MM04T	INHBE	inhibin, beta E	CCDS8939.1	chr12_57850238-57850238_G_T	220E>D	Substitution	Nonsynonymous coding	26%
MM04T	INMT	indolethylamine N-methyltransferase	CCDS5430.1	chr7_30791866-30791866_G_A	34E>K	Substitution	Nonsynonymous coding	31%
MM04T	INO80	INO80 homolog (S. cerevisiae)	CCDS10071.1	chr15_41387832-41387832_G_T	116F>L	Substitution	Nonsynonymous coding	28%
MM04T	INPP4B	inositol polyphosphate-4-phosphatase, type II, 105kDa	CCDS3757.1	chr4_143045809-143045809_C_A	609E>X	Substitution	Nonsense	27%
MM04T	INPP5D	inositol polyphosphate-5-phosphatase, 145kDa	ENST00000359570	chr2_234078674-234078674_G_A	552R>Q	Substitution	Nonsynonymous coding	33%
MM04T	INTS1	integrator complex subunit 1	CCDS47526.1	chr7_1524745-1524745_C_T	1084D>N	Substitution	Nonsynonymous coding	24%
MM04T	INTS10	integrator complex subunit 10	CCDS6011.2	chr8_19709289-19709289_G_T	702K>N	Substitution	Nonsynonymous coding	16%
MM04T	INTS5	integrator complex subunit 5	CCDS8027.1	chr11_62415457-62415457_G_A	699R>X	Substitution	Nonsense	29%
MM04T	INTS6	integrator complex subunit 6	CCDS9428.1	chr13_51950259-51950259_G_T	552L>I	Substitution	Nonsynonymous coding	36%
MM04T	INTS7	integrator complex subunit 7	CCDS1501.1	chr1_212161258-212161258_C_T	323A>T	Substitution	Nonsynonymous coding	25%
MM04T	INTS9	integrator complex subunit 9	CCDS34873.1	chr8_28633305-28633305_G_A	512R>W	Substitution	Nonsynonymous coding	22%
MM04T	INTU	inturned planar cell polarity effector homolog (Drosophila)	CCDS34061.1	chr4_128590200-128590200_C_A	328L>I	Substitution	Nonsynonymous coding	41%
MM04T	INTU	inturned planar cell polarity effector homolog (Drosophila)	CCDS34061.1	chr4_128625420-128625420_T_G	514I>S	Substitution	Nonsynonymous coding	38%
MM04T	IP6K3	inositol hexakisphosphate kinase 3	CCDS34435.1	chr6_33695969-33695969_G_A	103A>V	Substitution	Nonsynonymous coding	36%
MM04T	IPCEF1	interaction protein for cytohesin exchange factors 1	CCDS47509.1	chr6_154481169-154481169_C_A	371E>X	Substitution	Nonsense	31%
MM04T	IPMK	inositol polyphosphate multikinase	CCDS7250.1	chr10_59986879-59986879_C_A	100D>Y	Substitution	Nonsynonymous coding	18%
MM04T	IPO11	importin 11	CCDS47217.1	chr5_61847150-61847150_A_C	856K>T	Substitution	Nonsynonymous coding	29%
MM04T	IPO8	importin 8	CCDS8719.1	chr12_30819121-30819121_C_A	403K>N	Substitution	Nonsynonymous coding	35%
MM04T	IQCA1	IQ motif containing with AAA domain 1	CCDS46549.1	chr2_237300959-237300959_G_T	415F>L	Substitution	Nonsynonymous coding	19%
MM04T	IQCA1	IQ motif containing with AAA domain 1	CCDS46549.1	chr2_237240016-237240016_C_A	787E>X	Substitution	Nonsense	31%
MM04T	IQCF2	IQ motif containing F2	CCDS2835.1	chr3_51897243-51897243_C_T	118R>X	Substitution	Nonsense	30%
MM04T	IQCH	IQ motif containing H	CCDS32273.1	chr15_67786690-67786690_G_A	986E>K	Substitution	Nonsynonymous coding	35%
MM04T	IQGAP1	IQ motif containing GTPase activating protein 1	CCDS10362.1	chr15_91020020-91020020_A_C	970E>D	Substitution	Nonsynonymous coding	19%
MM04T	IQGAP2	IQ motif containing GTPase activating protein 2	CCDS34188.1	chr5_75993845-75993845_C_T	1414R>X	Substitution	Nonsense	29%
MM04T	IQGAP3	IQ motif containing GTPase activating protein 3	CCDS1144.1	chr1_156534445-156534445_C_A	133K>N	Substitution	Nonsynonymous coding	18%
MM04T	IQGAP3	IQ motif containing GTPase activating protein 3	CCDS1144.1	chr1_156504290-156504290_G_T	NA	Substitution	Splice site donor	31%
MM04T	IQSEC2	IQ motif and Sec7 domain 2	CCDS48130.1	chrX_53265519-53265519_C_A	1146D>Y	Substitution	Nonsynonymous coding	33%

MM04T	IQSEC2	IQ motif and Sec7 domain 2	CCDS48130.1	chrX_53276283-53276283_G_A	873R>C	Substitution	Nonsynonymous coding	26%
MM04T	IQSEC2	IQ motif and Sec7 domain 2	CCDS48130.1	chrX_53271070-53271070_G_A	971R>X	Substitution	Nonsense	30%
MM04T	IQSEC3	IQ motif and Sec7 domain 3 [Source:HGNC Symbol;Acc:29193]	CCDS31725.1	chr12_278208-278208_A_G	698T>A	Substitution	Nonsynonymous coding	43%
MM04T	IQUB	IQ motif and ubiquitin domain containing	CCDS5787.1	chr7_123104958-123104958__T	NA	Insertion	Frameshift	27%
MM04T	IQUB	IQ motif and ubiquitin domain containing	CCDS5787.1	chr7_123101420-123101420_G_T	666F>L	Substitution	Nonsynonymous coding	32%
MM04T	IRAK1BP1	interleukin-1 receptor-associated kinase 1 binding protein 1	CCDS34488.1	chr6_79607867-79607867_C_T	200P>L	Substitution	Nonsynonymous coding	29%
MM04T	IRAK4	interleukin-1 receptor-associated kinase 4	CCDS8744.1	chr12_44171469-44171469_C_A	251F>L	Substitution	Nonsynonymous coding	24%
MM04T	IREB2	iron-responsive element binding protein 2	CCDS10302.1	chr15_78765700-78765700_G_A	334D>N	Substitution	Nonsynonymous coding	23%
MM04T	IREB2	iron-responsive element binding protein 2	CCDS10302.1	chr15_78786378-78786378_C_A	818H>N	Substitution	Nonsynonymous coding	28%
MM04T	IRG1	immunoresponsive 1 homolog (mouse)	ENST00000377462	chr13_77531795-77531795_C_A	374P>H	Substitution	Nonsynonymous coding	24%
MM04T	IRS4	insulin receptor substrate 4	CCDS14544.1	chrX_107976384-107976384_C_T	1064R>Q	Substitution	Nonsynonymous coding	38%
MM04T	IRS4	insulin receptor substrate 4	CCDS14544.1	chrX_107977561-107977561_C_A	672E>X	Substitution	Nonsense	24%
MM04T	ISG20L2	interferon stimulated exonuclease gene 20kDa-like 2	CCDS1153.1	chr1_156697303-156697303_G_A	48P>S	Substitution	Nonsynonymous coding	24%
MM04T	ISL1	ISL LIM homeobox 1	CCDS43314.1	chr5_50680552-50680552_G_T	69R>I	Substitution	Nonsynonymous coding	25%
MM04T	ITGA1	integrin, alpha 1	CCDS3955.1	chr5_52221185-52221185_G_T	827K>N	Substitution	Nonsynonymous coding	26%
MM04T	ITGA1	integrin, alpha 1	CCDS3955.1	chr5_52229746-52229746_T_G	962S>A	Substitution	Nonsynonymous coding	28%
MM04T	ITGA2B	integrin, alpha 2b (platelet glycoprotein IIb of IIb/IIIa complex, antigen CD41)	CCDS32665.1	chr17_42453487-42453487_T_C	805D>G	Substitution	Nonsynonymous coding	18%
MM04T	ITGA5	integrin, alpha 5 (fibronectin receptor, alpha polypeptide)	CCDS8880.1	chr12_54793664-54793664_G_A	904S>L	Substitution	Nonsynonymous coding	24%
MM04T	ITGA6	integrin, alpha 6	CCDS46451.1	chr2_173352185-173352185_A_G	NA	Substitution	Splice site donor	29%
MM04T	ITGA8	integrin, alpha 8	CCDS31155.1	chr10_15650284-15650284_G_T	520S>Y	Substitution	Nonsynonymous coding	28%
MM04T	ITGA8	integrin, alpha 8	CCDS31155.1	chr10_15726101-15726101_C_T	157R>K	Substitution	Nonsynonymous coding	38%
MM04T	ITGA8	integrin, alpha 8	CCDS31155.1	chr10_15649678-15649678_G_A	588R>X	Substitution	Nonsense	21%
MM04T	ITGAE	integrin, alpha E (antigen CD103, human mucosal lymphocyte antigen 1; alpha	CCDS32531.1	chr17_3623608-3623608_C_A	1109R>I	Substitution	Nonsynonymous coding	20%
MM04T	ITGAM	integrin, alpha M (complement component 3 receptor 3 subunit)	CCDS45470.1	chr16_31340592-31340592_A_G	946N>D	Substitution	Nonsynonymous coding	28%
MM04T	ITGAX	integrin, alpha X (complement component 3 receptor 4 subunit)	CCDS10711.1	chr16_31391855-31391855_G_T	1062K>N	Substitution	Nonsynonymous coding	13%
MM04T	ITGB1BP2	integrin beta 1 binding protein (melusin) 2	CCDS14411.1	chrX_70524915-70524915_C_A	306P>H	Substitution	Nonsynonymous coding	32%
MM04T	ITGB3	integrin, beta 3 (platelet glycoprotein IIIa, antigen CD61)	CCDS11511.1	chr17_45360829-45360829_A_G	92D>G	Substitution	Nonsynonymous coding	33%
MM04T	ITIH1	inter-alpha-trypsin inhibitor heavy chain 1	CCDS2864.1	chr3_52817298-52817298_G_T	390E>X	Substitution	Nonsense	25%
MM04T	ITIH2	inter-alpha-trypsin inhibitor heavy chain 2	CCDS31141.1	chr10_7762891-7762891_G_A	235D>N	Substitution	Nonsynonymous coding	20%
MM04T	ITIH2	inter-alpha-trypsin inhibitor heavy chain 2	CCDS31141.1	chr10_7786917-7786917_G_A	858G>R	Substitution	Nonsynonymous coding	39%
MM04T	ITIH5	inter-alpha-trypsin inhibitor heavy chain family, member 5	CCDS31139.1	chr10_7682734-7682734_T_G	128K>N	Substitution	Nonsynonymous coding	19%
MM04T	ITIH6	inter-alpha-trypsin inhibitor heavy chain family, member 6	CCDS14361.1	chrX_54777534-54777534_C_T	1211R>Q	Substitution	Nonsynonymous coding	32%

MM04T	ITIH6	inter-alpha-trypsin inhibitor heavy chain family, member 6	CCDS14361.1	chrX_54817332-54817332_C_T	185G>D	Substitution	Nonsynonymous coding	26%
MM04T	ITLN1	intelectin 1 (galactofuranose binding)	CCDS1211.1	chr1_160854622-160854622_C_A	16G>X	Substitution	Nonsense	21%
MM04T	ITPKB	inositol-trisphosphate 3-kinase B	CCDS1555.1	chr1_226825420-226825420_C_T	862R>Q	Substitution	Nonsynonymous coding	26%
MM04T	ITPR2	inositol 1,4,5-trisphosphate receptor, type 2	CCDS41764.1	chr12_26752221-26752221_C_T	1287E>K	Substitution	Nonsynonymous coding	33%
MM04T	ITPR2	inositol 1,4,5-trisphosphate receptor, type 2	CCDS41764.1	chr12_26875412-26875412_C_T	148R>H	Substitution	Nonsynonymous coding	20%
MM04T	ITSN1	intersectin 1 (SH3 domain protein)	CCDS33545.1	chr21_35258737-35258737_T_C	1664F>L	Substitution	Nonsynonymous coding	19%
MM04T	IVNS1ABP	influenza virus NS1A binding protein	CCDS1368.1	chr1_185269172-185269172_G_T	487P>H	Substitution	Nonsynonymous coding	23%
MM04T	IVNS1ABP	influenza virus NS1A binding protein	CCDS1368.1	chr1_185269663-185269663_C_T	383R>Q	Substitution	Nonsynonymous coding	33%
MM04T	IYD	iodotyrosine deiodinase	CCDS5227.1	chr6_150715251-150715251_G_T	183E>X	Substitution	Nonsense	30%
MM04T	IZUMO1	izumo sperm-egg fusion 1	CCDS12732.1	chr19_49248938-49248938_G_A	60A>V	Substitution	Nonsynonymous coding	30%
MM04T	IZUMO3	IZUMO family member 3	ENST00000380098	chr9_24544986-24544986_C_A	67K>N	Substitution	Nonsynonymous coding	35%
MM04T	JAK1	Janus kinase 1	CCDS41346.1	chr1_65309779-65309779_C_T	791E>K	Substitution	Nonsynonymous coding	24%
MM04T	JAK1	Janus kinase 1	CCDS41346.1	chr1_65325843-65325843_C_T	427V>M	Substitution	Nonsynonymous coding	20%
MM04T	JAK1	Janus kinase 1	CCDS41346.1	chr1_65332689-65332689_C_A	284E>X	Substitution	Nonsense	27%
MM04T	JAK1	Janus kinase 1	CCDS41346.1	chr1_65349005-65349005_C_A	54E>X	Substitution	Nonsense	22%
MM04T	JAK2	Janus kinase 2	CCDS6457.1	chr9_5050746-5050746_G_T	177E>X	Substitution	Nonsense	37%
MM04T	JAK3	Janus kinase 3	CCDS12366.1	chr19_17942524-17942524_C_T	922D>N	Substitution	Nonsynonymous coding	36%
MM04T	JAKMIP2	janus kinase and microtubule interacting protein 2	CCDS4285.1	chr5_147040702-147040702_C_A	146D>Y	Substitution	Nonsynonymous coding	25%
MM04T	JARID2	jumonji, AT rich interactive domain 2	CCDS4533.1	chr6_15501441-15501441_C_A	750P>H	Substitution	Nonsynonymous coding	23%
MM04T	JARID2	jumonji, AT rich interactive domain 2	CCDS4533.1	chr6_15504823-15504823_G_T	847E>D	Substitution	Nonsynonymous coding	31%
MM04T	JARID2	jumonji, AT rich interactive domain 2	CCDS4533.1	chr6_15520412-15520412_C_T	1224A>V	Substitution	Nonsynonymous coding	31%
MM04T	JMJD1C	jumonji domain containing 1C	CCDS41532.1	chr10_64979716-64979716_G_T	159L>I	Substitution	Nonsynonymous coding	28%
MM04T	JMJD7-PLA2G4B	JMJD7-PLA2G4B readthrough	CCDS32202.1	chr15_42133280-42133280_C_T	358R>C	Substitution	Nonsynonymous coding	16%
MM04T	JMY	junction mediating and regulatory protein, p53 cofactor	CCDS4047.3	chr5_78533257-78533257_G_T	262E>X	Substitution	Nonsense	22%
MM04T	JPH2	junctophilin 2	CCDS13325.1	chr20_42744983-42744983_C_A	444E>D	Substitution	Nonsynonymous coding	13%
MM04T	JSRP1	junctional sarcoplasmic reticulum protein 1	CCDS12086.1	chr19_2252725-2252725_T_G	200K>T	Substitution	Nonsynonymous coding	28%
MM04T	KAL1	Kallmann syndrome 1 sequence	CCDS14130.1	chrX_8553424-8553424_C_T	247R>Q	Substitution	Nonsynonymous coding	29%
MM04T	KALRN	kalirin, RhoGEF kinase	CCDS3027.1	chr3_124175535-124175535_G_A	1270E>K	Substitution	Nonsynonymous coding	16%
MM04T	KANK1	KN motif and ankyrin repeat domains 1	CCDS34976.1	chr9_712132-712132_G_T	456E>X	Substitution	Nonsense	32%
MM04T	KANK2	KN motif and ankyrin repeat domains 2	CCDS12255.1	chr19_11283743-11283743_C_T	709A>T	Substitution	Nonsynonymous coding	18%
MM04T	KANK3	KN motif and ankyrin repeat domains 3	CCDS12199.1	chr19_8399715-8399715_C_A	332E>D	Substitution	Nonsynonymous coding	30%
MM04T	KANK4	KN motif and ankyrin repeat domains 4	CCDS620.1	chr1_62739626-62739626_T_G	384I>L	Substitution	Nonsynonymous coding	34%

MM04T	KANK4	KN motif and ankyrin repeat domains 4	CCDS620.1	chr1_62740615-62740615_C_A	54R>I	Substitution	Nonsynonymous coding	22%
MM04T	KANSL1L	KAT8 regulatory NSL complex subunit 1-like	CCDS33370.1	chr2_210888782-210888782_G_T	903S>Y	Substitution	Nonsynonymous coding	35%
MM04T	KAT2B	K(lysine) acetyltransferase 2B	CCDS2634.1	chr3_20189940-20189940_G_A	756E>K	Substitution	Nonsynonymous coding	19%
MM04T	KAT6A	K(lysine) acetyltransferase 6A	CCDS6124.1	chr8_41791026-41791026_G_A	1571S>F	Substitution	Nonsynonymous coding	30%
MM04T	KAT6A	K(lysine) acetyltransferase 6A	CCDS6124.1	chr8_41798947-41798947_A_G	818S>P	Substitution	Nonsynonymous coding	38%
MM04T	KATNAL1	katanin p60 subunit A-like 1	CCDS31956.1	chr13_30784555-30784555_T_G	391K>T	Substitution	Nonsynonymous coding	22%
MM04T	KBTBD3	kelch repeat and BTB (POZ) domain containing 3	CCDS8334.1	chr11_105924202-105924202_A_C	405L>X	Substitution	Nonsense	36%
MM04T	KBTBD7	kelch repeat and BTB (POZ) domain containing 7	CCDS9377.1	chr13_41766628-41766628_T_C	589D>G	Substitution	Nonsynonymous coding	10%
MM04T	KCNA1	potassium voltage-gated channel, shaker-related subfamily, member 1 (episodic ataxia	CCDS8535.1	chr12_5020952-5020952_C_A	136F>L	Substitution	Nonsynonymous coding	30%
MM04T	KCNA10	potassium voltage-gated channel, shaker-related subfamily, member 10	CCDS826.1	chr1_111060030-111060030_G_T	460F>L	Substitution	Nonsynonymous coding	32%
MM04T	KCNA4	potassium voltage-gated channel, shaker-related subfamily, member 4	CCDS41629.1	chr11_30033950-30033950_C_A	92K>N	Substitution	Nonsynonymous coding	31%
MM04T	KCNA5	potassium voltage-gated channel, shaker-related subfamily, member 5	CCDS8536.1	chr12_5153479-5153479_G_A	56A>T	Substitution	Nonsynonymous coding	12%
MM04T	KCNA6	potassium voltage-gated channel, shaker-related subfamily, member 6	CCDS8534.1	chr12_4920318-4920318_G_A	371A>T	Substitution	Nonsynonymous coding	39%
MM04T	KCNAB1	potassium voltage-gated channel, shaker-related subfamily, beta member 1	CCDS3174.1	chr3_156232899-156232899_C_A	252S>Y	Substitution	Nonsynonymous coding	30%
MM04T	KCNAB2	potassium voltage-gated channel, shaker-related subfamily, beta member 2	CCDS55.1	chr1_6156802-6156802_C_T	304P>L	Substitution	Nonsynonymous coding	23%
MM04T	KCNAB3	potassium voltage-gated channel, shaker-related subfamily, beta member 3	CCDS11124.1	chr17_7826850-7826850_C_T	354R>H	Substitution	Nonsynonymous coding	26%
MM04T	KCNB1	potassium voltage-gated channel, Shab-related subfamily, member 1	CCDS13418.1	chr20_48098719-48098719_C_A	100R>L	Substitution	Nonsynonymous coding	19%
MM04T	KCND1	potassium voltage-gated channel, Shal-related subfamily, member 1	CCDS14314.1	chrX_48819857-48819857_C_A	643K>N	Substitution	Nonsynonymous coding	26%
MM04T	KCND1	potassium voltage-gated channel, Shal-related subfamily, member 1	CCDS14314.1	chrX_48826006-48826006_C_T	225E>K	Substitution	Nonsynonymous coding	38%
MM04T	KCND3	potassium voltage-gated channel, Shal-related subfamily, member 3	CCDS843.1	chr1_112524582-112524582_C_T	256R>H	Substitution	Nonsynonymous coding	37%
MM04T	KCND3	potassium voltage-gated channel, Shal-related subfamily, member 3	CCDS843.1	chr1_112321054-112321054_A_G	NA	Substitution	Splice site donor	30%
MM04T	KCNG1	potassium voltage-gated channel, subfamily G, member 1	CCDS13436.1	chr20_49626817-49626817_G_A	20S>L	Substitution	Nonsynonymous coding	36%
MM04T	KCNH1	potassium voltage-gated channel, subfamily H (eag-related), member 1	CCDS1496.1	chr1_211093357-211093357_G_A	363R>X	Substitution	Nonsense	31%
MM04T	KCNH2	potassium voltage-gated channel, subfamily H (eag-related), member 2	CCDS5910.1	chr7_150648025-150648025_C_T	710G>D	Substitution	Nonsynonymous coding	25%
MM04T	KCNH6	potassium voltage-gated channel, subfamily H (eag-related), member 6	CCDS11638.1	chr17_61611277-61611277_G_A	236E>K	Substitution	Nonsynonymous coding	32%
MM04T	KCNH7	potassium voltage-gated channel, subfamily H (eag-related), member 7	CCDS2219.1	chr2_163695019-163695019_G_A	4R>C	Substitution	Nonsynonymous coding	32%
MM04T	KCNH8	potassium voltage-gated channel, subfamily H (eag-related), member 8	CCDS2632.1	chr3_19575403-19575403_C_A	1046L>I	Substitution	Nonsynonymous coding	40%
MM04T	KCNIP1	Kv channel interacting protein 1	CCDS34286.1	chr5_170147349-170147349_G_A	82E>K	Substitution	Nonsynonymous coding	30%
MM04T	KCNIP1	Kv channel interacting protein 1	CCDS34286.1	chr5_170148857-170148857_T_G	104Y>D	Substitution	Nonsynonymous coding	29%
MM04T	KCNIP1	Kv channel interacting protein 1	CCDS34286.1	chr5_170160885-170160885_C_A	207L>I	Substitution	Nonsynonymous coding	28%
MM04T	KCNJ12	potassium inwardly-rectifying channel, subfamily J, member 12	CCDS11219.1	chr17_21318731-21318731_C_T	26S>L	Substitution	Nonsynonymous coding	11%
MM04T	KCNJ15	potassium inwardly-rectifying channel, subfamily J, member 15	CCDS13656.1	chr21_39671299-39671299_G_T	39R>I	Substitution	Nonsynonymous coding	27%

MM04T	KCNJ15	potassium inwardly-rectifying channel, subfamily J, member 15	CCDS13656.1	chr21_39672057-39672057_G_A	292A>T	Substitution	Nonsynonymous coding	33%
MM04T	KCNJ16	potassium inwardly-rectifying channel, subfamily J, member 16	CCDS11687.1	chr17_68128865-68128865_G_A	213E>K	Substitution	Nonsynonymous coding	29%
MM04T	KCNJ2	potassium inwardly-rectifying channel, subfamily J, member 2	CCDS11688.1	chr17_68172272-68172272_G_T	364K>N	Substitution	Nonsynonymous coding	32%
MM04T	KCNJ5	potassium inwardly-rectifying channel, subfamily J, member 5	CCDS8479.1	chr11_128781240-128781240_G_T	24K>N	Substitution	Nonsynonymous coding	18%
MM04T	KCNK13	potassium channel, subfamily K, member 13	CCDS9889.1	chr14_90650596-90650596_C_T	159S>L	Substitution	Nonsynonymous coding	22%
MM04T	KCNK7	potassium channel, subfamily K, member 7	CCDS31608.1	chr11_65361243-65361243_C_T	141R>H	Substitution	Nonsynonymous coding	20%
MM04T	KCNMA1	potassium large conductance calcium-activated channel, subfamily M, alpha member	CCDS7352.1	chr10_78704623-78704623_G_A	879S>L	Substitution	Nonsynonymous coding	31%
MM04T	KCNMB4	potassium large conductance calcium-activated channel, subfamily M, beta member	CCDS8997.1	chr12_70794030-70794030_T_G	126N>K	Substitution	Nonsynonymous coding	21%
MM04T	KCNQ2	potassium voltage-gated channel, KQT-like subfamily, member 2	CCDS13520.1	chr20_62059751-62059751_T_A	396N>Y	Substitution	Nonsynonymous coding	40%
MM04T	KCNQ2	potassium voltage-gated channel, KQT-like subfamily, member 2	CCDS13520.1	chr20_62070074-62070074_C_A	NA	Substitution	Splice site acceptor	34%
MM04T	KCNS3	potassium voltage-gated channel, delayed-rectifier, subfamily S, member 3	CCDS1692.1	chr2_18113019-18113019_G_T	248K>N	Substitution	Nonsynonymous coding	29%
MM04T	KCNS3	potassium voltage-gated channel, delayed-rectifier, subfamily S, member 3	CCDS1692.1	chr2_18113695-18113695_G_T	474E>X	Substitution	Nonsense	33%
MM04T	KCNT2	potassium channel, subfamily T, member 2	CCDS1384.1	chr1_196250075-196250075_C_A	942R>I	Substitution	Nonsynonymous coding	21%
MM04T	KCTD16	potassium channel tetramerisation domain containing 16	CCDS34260.1	chr5_143853255-143853255_G_A	289D>N	Substitution	Nonsynonymous coding	14%
MM04T	KCTD17	potassium channel tetramerisation domain containing 17	CCDS13940.2	chr22_37457600-37457600_G_T	252R>I	Substitution	Nonsynonymous coding	31%
MM04T	KCTD18	potassium channel tetramerisation domain containing 18	CCDS2330.1	chr2_201363707-201363707_C_A	158R>I	Substitution	Nonsynonymous coding	35%
MM04T	KCTD21	potassium channel tetramerisation domain containing 21	CCDS31645.1	chr11_77885516-77885516_G_T	29L>I	Substitution	Nonsynonymous coding	11%
MM04T	KCTD3	potassium channel tetramerisation domain containing 3	CCDS1515.1	chr1_215777597-215777597_G_A	421R>Q	Substitution	Nonsynonymous coding	31%
MM04T	KCTD3	potassium channel tetramerisation domain containing 3	CCDS1515.1	chr1_215749304-215749304_G_T	82E>X	Substitution	Nonsense	52%
MM04T	KCTD8	potassium channel tetramerisation domain containing 8	CCDS3467.1	chr4_44449619-44449619_C_T	308D>N	Substitution	Nonsynonymous coding	23%
MM04T	KDM1B	lysine (K)-specific demethylase 1B	ENST00000388870	chr6_18205779-18205779_G_T	515E>X	Substitution	Nonsense	16%
MM04T	KDM3A	lysine (K)-specific demethylase 3A	CCDS1990.1	chr2_86709598-86709598_G_T	901K>N	Substitution	Nonsynonymous coding	43%
MM04T	KDM5A	lysine (K)-specific demethylase 5A	CCDS41736.1	chr12_406218-406218_C_A	1408S>I	Substitution	Nonsynonymous coding	36%
MM04T	KDM5B	lysine (K)-specific demethylase 5B	CCDS30974.1	chr1_202715432-202715432_C_A	679R>I	Substitution	Nonsynonymous coding	28%
MM04T	KDM6A	lysine (K)-specific demethylase 6A	CCDS14265.1	chrX_44918695-44918695_G_A	393R>Q	Substitution	Nonsynonymous coding	29%
MM04T	KDM6B	lysine (K)-specific demethylase 6B	CCDS32552.1	chr17_7753439-7753439_C_T	1206T>I	Substitution	Nonsynonymous coding	31%
MM04T	KDR	kinase insert domain receptor (a type III receptor tyrosine kinase)	CCDS3497.1	chr4_55972888-55972888_T_G	501K>T	Substitution	Nonsynonymous coding	31%
MM04T	KHDRBS2	KH domain containing, RNA binding, signal transduction associated 2	CCDS4963.1	chr6_62442631-62442631_C_A	283Q>H	Substitution	Nonsynonymous coding	19%
MM04T	KHDRBS2	KH domain containing, RNA binding, signal transduction associated 2	CCDS4963.1	chr6_62611243-62611243_G_A	173R>C	Substitution	Nonsynonymous coding	33%
MM04T	KIAA0020	KIAA0020	CCDS6448.2	chr9_2827069-2827069_T_G	NA	Substitution	Splice site donor	38%
MM04T	KIAA0146	KIAA0146	CCDS43737.1	chr8_48511686-48511686_C_A	491S>Y	Substitution	Nonsynonymous coding	28%
MM04T	KIAA0146	KIAA0146	CCDS43737.1	chr8_48192563-48192563_G_A	49W>X	Substitution	Nonsense	35%

MM04T	KIAA0232	KIAA0232	CCDS43209.1	chr4_6862882-6862882_A_C	258K>T	Substitution	Nonsynonymous coding	19%
MM04T	KIAA0232	KIAA0232	CCDS43209.1	chr4_6863077-6863077_G_A	323R>Q	Substitution	Nonsynonymous coding	32%
MM04T	KIAA0319L	KIAA0319-like	CCDS390.1	chr1_35926039-35926039_C_A	NA	Substitution	Splice site acceptor	22%
MM04T	KIAA0368	KIAA0368	CCDS48006.1	chr9_114206746-114206746_T_G	239K>T	Substitution	Nonsynonymous coding	25%
MM04T	KIAA0495	-	NM_207306	chr1_3662662-3662662_G_T	36L>I	Substitution	Nonsynonymous coding	32%
MM04T	KIAA0586	KIAA0586	CCDS45115.1	chr14_58909588-58909588_C_T	192P>L	Substitution	Nonsynonymous coding	30%
MM04T	KIAA0825	KIAA0825	CCDS4070.1	chr5_93856154-93856154_C_A	257E>X	Substitution	Nonsense	20%
MM04T	KIAA0895	KIAA0895	CCDS43570.1	chr7_36373756-36373756_G_A	339R>X	Substitution	Nonsense	32%
MM04T	KIAA0947	KIAA0947	CCDS47187.1	chr5_5447846-5447846_G_A	174E>K	Substitution	Nonsynonymous coding	44%
MM04T	KIAA0947	KIAA0947	CCDS47187.1	chr5_5464492-5464492_C_A	1682S>Y	Substitution	Nonsynonymous coding	32%
MM04T	KIAA1009	KIAA1009	CCDS34494.2	chr6_84925057-84925057_G_A	149S>L	Substitution	Nonsynonymous coding	24%
MM04T	KIAA1009	KIAA1009	CCDS34494.2	chr6_84836195-84836195_C_A	1303E>X	Substitution	Nonsense	31%
MM04T	KIAA1107	KIAA1107	CCDS44172.1	chr1_92647337-92647337_G_A	928R>Q	Substitution	Nonsynonymous coding	33%
MM04T	KIAA1109	KIAA1109	CCDS43267.1	chr4_123178491-123178491_A_G	2154T>A	Substitution	Nonsynonymous coding	25%
MM04T	KIAA1109	KIAA1109	CCDS43267.1	chr4_123227125-123227125_T_C	3256S>P	Substitution	Nonsynonymous coding	31%
MM04T	KIAA1109	KIAA1109	CCDS43267.1	chr4_123246387-123246387_C_A	3636S>Y	Substitution	Nonsynonymous coding	27%
MM04T	KIAA1109	KIAA1109	CCDS43267.1	chr4_123268917-123268917_C_A	4371S>Y	Substitution	Nonsynonymous coding	33%
MM04T	KIAA1109	KIAA1109	CCDS43267.1	chr4_123264600-123264600_C_T	4130R>X	Substitution	Nonsense	35%
MM04T	KIAA1210	KIAA1210	CCDS48156.1	chrX_118223163-118223163_G_T	677S>Y	Substitution	Nonsynonymous coding	26%
MM04T	KIAA1210	KIAA1210	CCDS48156.1	chrX_118239046-118239046_G_T	326S>Y	Substitution	Nonsynonymous coding	22%
MM04T	KIAA1210	KIAA1210	CCDS48156.1	chrX_118281550-118281550_C_A	99G>V	Substitution	Nonsynonymous coding	36%
MM04T	KIAA1210	KIAA1210	CCDS48156.1	chrX_118284425-118284425_G_A	40R>X	Substitution	Nonsense	28%
MM04T	KIAA1211	KIAA1211	CCDS43230.1	chr4_57181878-57181878_A_G	737K>R	Substitution	Nonsynonymous coding	27%
MM04T	KIAA1239	KIAA1239	CCDS47040.1	chr4_37447791-37447791_G_A	1394R>Q	Substitution	Nonsynonymous coding	32%
MM04T	KIAA1324L	KIAA1324-like	CCDS47632.1	chr7_86544166-86544166_C_A	535R>I	Substitution	Nonsynonymous coding	33%
MM04T	KIAA1324L	KIAA1324-like	CCDS47632.1	chr7_86574241-86574241_C_T	210D>N	Substitution	Nonsynonymous coding	28%
MM04T	KIAA1377	KIAA1377	CCDS31658.1	chr11_101828924-101828924_G_A	178A>T	Substitution	Nonsynonymous coding	20%
MM04T	KIAA1383	KIAA1383	CCDS44334.1	chr1_232943691-232943691_A_T	974K>N	Substitution	Nonsynonymous coding	38%
MM04T	KIAA1407	KIAA1407	CCDS2977.1	chr3_113723549-113723549_C_T	638R>Q	Substitution	Nonsynonymous coding	33%
MM04T	KIAA1429	KIAA1429	CCDS34923.1	chr8_95541498-95541498_G_T	227S>Y	Substitution	Nonsynonymous coding	33%
MM04T	KIAA1432	KIAA1432	CCDS34982.1	chr9_5772740-5772740_C_T	1186R>W	Substitution	Nonsynonymous coding	30%
MM04T	KIAA1462	KIAA1462	CCDS41500.1	chr10_30336573-30336573_G_A	57R>C	Substitution	Nonsynonymous coding	26%

MM04T	KIAA1549	KIAA1549	CCDS47723.1	chr7_138591737-138591737_C_T	1080E>K	Substitution	Nonsynonymous coding	15%
MM04T	KIAA1549L	KIAA1549-like	CCDS44565.1	chr11_33564273-33564273_G_T	91Q>H	Substitution	Nonsynonymous coding	28%
MM04T	KIAA1549L	KIAA1549-like	CCDS44565.1	chr11_33604953-33604953_C_T	1200T>M	Substitution	Nonsynonymous coding	30%
MM04T	KIAA1549L	KIAA1549-like	CCDS44565.1	chr11_33689683-33689683_T_C	1851F>L	Substitution	Nonsynonymous coding	36%
MM04T	KIAA1551	KIAA1551	CCDS8725.2	chr12_32137002-32137002_G_T	1038R>I	Substitution	Nonsynonymous coding	27%
MM04T	KIAA1551	KIAA1551	CCDS8725.2	chr12_32134056-32134056_C_A	56S>X	Substitution	Nonsense	23%
MM04T	KIAA1671	KIAA1671	CCDS46676.1	chr22_25436024-25436024_G_A	976R>Q	Substitution	Nonsynonymous coding	18%
MM04T	KIAA1671	KIAA1671	CCDS46676.1	chr22_25436896-25436896_G_A	1267E>K	Substitution	Nonsynonymous coding	16%
MM04T	KIAA1731	KIAA1731	CCDS44708.1	chr11_93402856-93402856_C_T	150R>X	Substitution	Nonsense	23%
MM04T	KIAA1755	KIAA1755	CCDS33467.1	chr20_36854081-36854081_C_T	719V>I	Substitution	Nonsynonymous coding	19%
MM04T	KIAA1755	KIAA1755	CCDS33467.1	chr20_36870025-36870025_C_A	170E>X	Substitution	Nonsense	22%
MM04T	KIAA1919	KIAA1919	CCDS5090.1	chr6_111583470-111583470_T_G	13V>G	Substitution	Nonsynonymous coding	37%
MM04T	KIAA1984	KIAA1984	CCDS43906.1	chr9_139700974-139700974_G_T	376K>N	Substitution	Nonsynonymous coding	30%
MM04T	KIAA2018	KIAA2018	CCDS43133.1	chr3_113378160-113378160_A_T	790M>K	Substitution	Nonsynonymous coding	24%
MM04T	KIAA2018	KIAA2018	CCDS43133.1	chr3_113379327-113379327_G_T	401S>Y	Substitution	Nonsynonymous coding	27%
MM04T	KIAA2018	KIAA2018	CCDS43133.1	chr3_113379265-113379265_G_A	422R>X	Substitution	Nonsense	29%
MM04T	KIF11	kinesin family member 11	CCDS7422.1	chr10_94369173-94369173_T_G	202I>S	Substitution	Nonsynonymous coding	22%
MM04T	KIF12	kinesin family member 12	CCDS6801.1	chr9_116859619-116859619_A_C	65F>C	Substitution	Nonsynonymous coding	20%
MM04T	KIF13A	kinesin family member 13A	CCDS47381.1	chr6_17764501-17764501_G_T	1753S>Y	Substitution	Nonsynonymous coding	34%
MM04T	KIF13B	kinesin family member 13B	NM_015254	chr8_29013298-29013298_T_G	494I>L	Substitution	Nonsynonymous coding	24%
MM04T	KIF14	kinesin family member 14	CCDS30963.1	chr1_200558349-200558349_T_G	1037K>T	Substitution	Nonsynonymous coding	30%
MM04T	KIF14	kinesin family member 14	CCDS30963.1	chr1_200583453-200583453_G_A	483T>I	Substitution	Nonsynonymous coding	24%
MM04T	KIF15	kinesin family member 15	CCDS33744.1	chr3_44827929-44827929_G_T	168E>X	Substitution	Nonsense	34%
MM04T	KIF16B	kinesin family member 16B	CCDS13122.1	chr20_16360255-16360255_G_A	798R>W	Substitution	Nonsynonymous coding	10%
MM04T	KIF16B	kinesin family member 16B	CCDS13122.1	chr20_16360651-16360651_C_T	666E>K	Substitution	Nonsynonymous coding	26%
MM04T	KIF18A	kinesin family member 18A	CCDS7867.1	chr11_28112183-28112183_C_T	227R>H	Substitution	Nonsynonymous coding	14%
MM04T	KIF1B	kinesin family member 1B	CCDS112.1	chr1_10363524-10363524_G_A	761A>T	Substitution	Nonsynonymous coding	27%
MM04T	KIF20B	kinesin family member 20B	CCDS7407.1	chr10_91497545-91497545_A_C	943K>Q	Substitution	Nonsynonymous coding	32%
MM04T	KIF20B	kinesin family member 20B	CCDS7407.1	chr10_91522554-91522554_C_T	1611P>S	Substitution	Nonsynonymous coding	30%
MM04T	KIF21A	kinesin family member 21A	CCDS31773.1	chr12_39757002-39757002_T_C	306N>S	Substitution	Nonsynonymous coding	24%
MM04T	KIF21A	kinesin family member 21A	CCDS31773.1	chr12_39761758-39761758_C_A	176R>I	Substitution	Nonsynonymous coding	26%
MM04T	KIF23	kinesin family member 23	CCDS32278.1	chr15_69738627-69738627_G_A	907R>Q	Substitution	Nonsynonymous coding	27%

MM04T	KIF2B	kinesin family member 2B	CCDS32685.1	chr17_51900492-51900492_C_T	33A>V	Substitution	Nonsynonymous coding	30%
MM04T	KIF4A	kinesin family member 4A	CCDS14401.1	chrX_69626765-69626765__A	NA	Insertion	Frameshift	17%
MM04T	KIF4A	kinesin family member 4A	CCDS14401.1	chrX_69563819-69563819_T_G	473I>S	Substitution	Nonsynonymous coding	27%
MM04T	KIF4A	kinesin family member 4A	CCDS14401.1	chrX_69596024-69596024_G_T	666Q>H	Substitution	Nonsynonymous coding	25%
MM04T	KIF4B	kinesin family member 4B	CCDS47324.1	chr5_154395396-154395396_G_T	659E>D	Substitution	Nonsynonymous coding	33%
MM04T	KIF5C	kinesin family member 5C	NM_004522	chr2_149829977-149829977_G_T	415E>D	Substitution	Nonsynonymous coding	18%
MM04T	KIR3DX1	killer cell immunoglobulin-like receptor, three domains, X1	ENST00000221567	chr19_55048098-55048098_A_C	222K>T	Substitution	Nonsynonymous coding	23%
MM04T	KLF3	Kruppel-like factor 3 (basic)	CCDS3444.1	chr4_38690369-38690369_C_T	74S>L	Substitution	Nonsynonymous coding	31%
MM04T	KLF7	Kruppel-like factor 7 (ubiquitous)	CCDS2373.1	chr2_207988705-207988705_C_T	176V>I	Substitution	Nonsynonymous coding	27%
MM04T	KLHDC1	kelch domain containing 1	CCDS9692.1	chr14_50199457-50199457_C_A	249S>X	Substitution	Nonsense	15%
MM04T	KLHDC9	kelch domain containing 9	CCDS30919.1	chr1_161068420-161068420_C_A	32S>X	Substitution	Nonsense	19%
MM04T	KLHL1	kelch-like family member 1	CCDS9445.1	chr13_70371068-70371068_G_T	481L>M	Substitution	Nonsynonymous coding	41%
MM04T	KLHL11	kelch-like family member 11	CCDS11411.1	chr17_40010079-40010079_C_A	680W>C	Substitution	Nonsynonymous coding	22%
MM04T	KLHL18	kelch-like family member 18	CCDS33749.1	chr3_47384235-47384235_C_T	418S>L	Substitution	Nonsynonymous coding	26%
MM04T	KLHL22	kelch-like family member 22	CCDS13779.1	chr22_20785808-20785808_G_T	117F>L	Substitution	Nonsynonymous coding	29%
MM04T	KLHL23	kelch-like family member 23	CCDS2236.1	chr2_170592000-170592000_G_A	159R>Q	Substitution	Nonsynonymous coding	63%
MM04T	KLHL24	kelch-like 24 (Drosophila)	CCDS3246.1	chr3_183388894-183388894_C_T	433R>X	Substitution	Nonsense	24%
MM04T	KLHL32	kelch-like 32 (Drosophila)	CCDS5038.1	chr6_97575331-97575331_C_A	469P>H	Substitution	Nonsynonymous coding	26%
MM04T	KLHL33	kelch-like 33 (Drosophila)	NM_001109997	chr14_20897408-20897408_A_G	401L>P	Substitution	Nonsynonymous coding	17%
MM04T	KLHL33	kelch-like 33 (Drosophila)	NM_001109997	chr14_20897516-20897516_G_A	365A>V	Substitution	Nonsynonymous coding	19%
MM04T	KLHL34	kelch-like 34 (Drosophila)	CCDS14199.1	chrX_21674274-21674274_C_T	545D>N	Substitution	Nonsynonymous coding	12%
MM04T	KLHL34	kelch-like 34 (Drosophila)	CCDS14199.1	chrX_21674535-21674535_C_T	458A>T	Substitution	Nonsynonymous coding	26%
MM04T	KLHL41	kelch-like 41 (Drosophila)	CCDS2234.1	chr2_170366764-170366764_C_A	159S>Y	Substitution	Nonsynonymous coding	31%
MM04T	KLK5	kallikrein-related peptidase 5	CCDS12810.1	chr19_51452152-51452152_G_T	185C>X	Substitution	Nonsense	20%
MM04T	KLK6	kallikrein-related peptidase 6	CCDS12811.1	chr19_51466621-51466621_C_A	128E>X	Substitution	Nonsense	22%
MM04T	KLK8	kallikrein-related peptidase 8	CCDS42600.1	chr19_51503365-51503365_C_T	172R>H	Substitution	Nonsynonymous coding	27%
MM04T	KNG1	kininogen 1	CCDS43183.1	chr3_186459351-186459351_G_A	389R>Q	Substitution	Nonsynonymous coding	35%
MM04T	KNG1	kininogen 1	CCDS43183.1	chr3_186459426-186459426_A_C	414D>A	Substitution	Nonsynonymous coding	34%
MM04T	KNTC1	kinetochore associated 1	CCDS45002.1	chr12_123014678-123014678_G_T	23R>I	Substitution	Nonsynonymous coding	26%
MM04T	KPNA1	karyopherin alpha 1 (importin alpha 5)	CCDS3013.1	chr3_122168458-122168458_C_T	294D>N	Substitution	Nonsynonymous coding	26%
MM04T	KPNA1	karyopherin alpha 1 (importin alpha 5)	CCDS3013.1	chr3_122215392-122215392_C_A	7E>D	Substitution	Nonsynonymous coding	20%
MM04T	KRAS	v-Ki-ras2 Kirsten rat sarcoma viral oncogene homolog	CCDS8703.1	chr12_25380181-25380181_T_A	93I>F	Substitution	Nonsynonymous coding	31%

MM04T	KRIT1	KRIT1, ankyrin repeat containing	CCDS5624.1	chr7_91852280-91852280_G_A	423R>X	Substitution	Nonsense	34%
MM04T	KRT1	keratin 1	CCDS8836.1	chr12_53071971-53071971_C_A	281E>D	Substitution	Nonsynonymous coding	33%
MM04T	KRT10	keratin 10	CCDS11377.1	chr17_38978345-38978345_G_A	165R>W	Substitution	Nonsynonymous coding	33%
MM04T	KRT12	keratin 12	CCDS11378.1	chr17_39023102-39023102_G_T	113L>I	Substitution	Nonsynonymous coding	36%
MM04T	KRT28	keratin 28	CCDS11376.1	chr17_38950174-38950174_C_T	368G>D	Substitution	Nonsynonymous coding	32%
MM04T	KRT3	keratin 3	CCDS44895.1	chr12_53186971-53186971_C_A	301E>D	Substitution	Nonsynonymous coding	25%
MM04T	KRT32	keratin 32	CCDS11393.1	chr17_39620542-39620542_T_G	288N>H	Substitution	Nonsynonymous coding	22%
MM04T	KRT36	keratin 36	CCDS11395.1	chr17_39645675-39645675_C_T	148E>K	Substitution	Nonsynonymous coding	30%
MM04T	KRT4	keratin 4	CCDS41787.1	chr12_53205605-53205605_C_A	281G>W	Substitution	Nonsynonymous coding	24%
MM04T	KRT72	keratin 72	CCDS8833.1	chr12_52985254-52985254_C_A	319Q>H	Substitution	Nonsynonymous coding	19%
MM04T	KSR1	kinase suppressor of ras 1	ENST00000398985	chr17_25904817-25904817_G_T	105E>D	Substitution	Nonsynonymous coding	17%
MM04T	KSR2	kinase suppressor of ras 2	NM_173598	chr12_117907466-117907466_C_A	NA	Substitution	Splice site donor	12%
MM04T	KTN1	kinectin 1 (kinesin receptor)	CCDS41957.1	chr14_56117292-56117292_G_T	834L>F	Substitution	Nonsynonymous coding	23%
MM04T	L2HGDH	L-2-hydroxyglutarate dehydrogenase	CCDS9698.1	chr14_50732183-50732183_C_A	363Q>H	Substitution	Nonsynonymous coding	31%
MM04T	L2HGDH	L-2-hydroxyglutarate dehydrogenase	CCDS9698.1	chr14_50760943-50760943_C_A	144E>X	Substitution	Nonsense	23%
MM04T	LAMA1	laminin, alpha 1	CCDS32787.1	chr18_6978221-6978221_T_C	2055Q>R	Substitution	Nonsynonymous coding	35%
MM04T	LAMA1	laminin, alpha 1	CCDS32787.1	chr18_7016579-7016579_C_T	967G>D	Substitution	Nonsynonymous coding	31%
MM04T	LAMA1	laminin, alpha 1	CCDS32787.1	chr18_7080087-7080087_C_A	NA	Substitution	Splice site acceptor	19%
MM04T	LAMA1	laminin, alpha 1	CCDS32787.1	chr18_7040191-7040191_C_A	436E>X	Substitution	Nonsense	25%
MM04T	LAMA2	laminin, alpha 2	CCDS5138.1	chr6_129649451-129649451_G_A	1402R>H	Substitution	Nonsynonymous coding	36%
MM04T	LAMA2	laminin, alpha 2	CCDS5138.1	chr6_129777503-129777503_C_A	2244S>Y	Substitution	Nonsynonymous coding	28%
MM04T	LAMA3	laminin, alpha 3	CCDS42419.1	chr18_21451411-21451411_C_T	1595A>V	Substitution	Nonsynonymous coding	16%
MM04T	LAMA3	laminin, alpha 3	CCDS42419.1	chr18_21479315-21479315_C_A	1967S>Y	Substitution	Nonsynonymous coding	18%
MM04T	LAMA3	laminin, alpha 3	CCDS42419.1	chr18_21478789-21478789_G_T	1901E>X	Substitution	Nonsense	29%
MM04T	LAMB1	laminin, beta 1	CCDS5750.1	chr7_107591768-107591768_C_A	NA	Substitution	Splice site acceptor	32%
MM04T	LAMC3	laminin, gamma 3	CCDS6938.1	chr9_133884784-133884784_C_A	61F>L	Substitution	Nonsynonymous coding	17%
MM04T	LARP1B	La ribonucleoprotein domain family, member 1B	CCDS3738.1	chr4_129035784-129035784_A_C	350T>P	Substitution	Nonsynonymous coding	33%
MM04T	LARP1B	La ribonucleoprotein domain family, member 1B	CCDS3738.1	chr4_128998997-128998997_G_T	33E>X	Substitution	Nonsense	35%
MM04T	LARP1B	La ribonucleoprotein domain family, member 1B	CCDS3738.1	chr4_129043215-129043215_C_T	466R>X	Substitution	Nonsense	30%
MM04T	LARP7	La ribonucleoprotein domain family, member 7	CCDS3701.2	chr4_113574380-113574380_G_A	522E>K	Substitution	Nonsynonymous coding	34%
MM04T	LARS	leucyl-tRNA synthetase	CCDS34265.1	chr5_145529263-145529263_C_A	NA	Substitution	Splice site acceptor	26%
MM04T	LARS	leucyl-tRNA synthetase	CCDS34265.1	chr5_145531439-145531439_C_A	471G>X	Substitution	Nonsense	28%

MM04T	LAX1	lymphocyte transmembrane adaptor 1	CCDS1441.2	chr1_203743181-203743181_C_T	190S>L	Substitution	Nonsynonymous coding	18%
MM04T	LBR	lamin B receptor	CCDS1545.1	chr1_225599097-225599097_C_T	377R>Q	Substitution	Nonsynonymous coding	33%
MM04T	LCA5	Leber congenital amaurosis 5	CCDS4990.1	chr6_80223159-80223159_G_A	164H>Y	Substitution	Nonsynonymous coding	26%
MM04T	LCE3A	late cornified envelope 3A	CCDS1017.1	chr1_152595326-152595326_G_T	85S>Y	Substitution	Nonsynonymous coding	29%
MM04T	LCE4A	late cornified envelope 4A	CCDS1022.1	chr1_152681575-152681575_G_T	8Q>H	Substitution	Nonsynonymous coding	31%
MM04T	LCN2	lipocalin 2	CCDS6892.1	chr9_130913923-130913923_G_T	94K>N	Substitution	Nonsynonymous coding	21%
MM04T	LCORL	ligand dependent nuclear receptor corepressor-like	CCDS3425.1	chr4_17974499-17974499_C_A	55S>I	Substitution	Nonsynonymous coding	40%
MM04T	LCP1	lymphocyte cytosolic protein 1 (L-plastin)	CCDS9403.1	chr13_46704945-46704945_G_A	NA	Substitution	Splice site donor	35%
MM04T	LCP2	lymphocyte cytosolic protein 2 (SH2 domain containing leukocyte protein of 76kDa)	CCDS47339.1	chr5_169688114-169688114_C_A	318E>D	Substitution	Nonsynonymous coding	30%
MM04T	LCP2	lymphocyte cytosolic protein 2 (SH2 domain containing leukocyte protein of 76kDa)	CCDS47339.1	chr5_169685045-169685045_C_A	366E>X	Substitution	Nonsense	29%
MM04T	LCT	lactase	CCDS2178.1	chr2_136564940-136564940_G_A	1311R>X	Substitution	Nonsense	32%
MM04T	LDB3	LIM domain binding 3	CCDS41544.1	chr10_88447002-88447002_C_T	174A>V	Substitution	Nonsynonymous coding	29%
MM04T	LDLRAD4	low density lipoprotein receptor class A domain containing 4	CCDS32793.1	chr18_13438288-13438288_C_T	29S>L	Substitution	Nonsynonymous coding	32%
MM04T	LDLRAP1	low density lipoprotein receptor adaptor protein 1	CCDS30639.1	chr1_25880423-25880423_G_T	33E>D	Substitution	Nonsynonymous coding	22%
MM04T	LEMD3	LEM domain containing 3	CCDS8972.1	chr12_65632546-65632546_C_T	625R>X	Substitution	Nonsense	36%
MM04T	LEPRE1	leucine proline-enriched proteoglycan (leprecan) 1	CCDS472.2	chr1_43213029-43213029_T_G	657N>H	Substitution	Nonsynonymous coding	34%
MM04T	LGI2	leucine-rich repeat LGI family, member 2	CCDS3431.1	chr4_25005110-25005110_T_G	534K>T	Substitution	Nonsynonymous coding	47%
MM04T	LGI2	leucine-rich repeat LGI family, member 2	CCDS3431.1	chr4_25026491-25026491_C_T	122E>K	Substitution	Nonsynonymous coding	27%
MM04T	LGMN	legumain	CCDS9904.1	chr14_93179178-93179178_C_A	194M>I	Substitution	Nonsynonymous coding	27%
MM04T	LGR4	leucine-rich repeat containing G protein-coupled receptor 4	CCDS31449.1	chr11_27390555-27390555_G_A	572S>L	Substitution	Nonsynonymous coding	24%
MM04T	LGR6	leucine-rich repeat containing G protein-coupled receptor 6	ENST00000420582	chr1_202163716-202163716_G_T	31R>I	Substitution	Nonsynonymous coding	13%
MM04T	LHCGR	luteinizing hormone/choriogonadotropin receptor	CCDS1842.1	chr2_48914853-48914853_G_A	695R>C	Substitution	Nonsynonymous coding	34%
MM04T	LHCGR	luteinizing hormone/choriogonadotropin receptor	CCDS1842.1	chr2_48915795-48915795_G_T	381L>I	Substitution	Nonsynonymous coding	33%
MM04T	LHCGR	luteinizing hormone/choriogonadotropin receptor	ENST00000428232	chr2_48948859-48948859_T_C	165N>S	Substitution	Nonsynonymous coding	31%
MM04T	LHCGR	luteinizing hormone/choriogonadotropin receptor	CCDS1842.1	chr2_48950628-48950628_G_T	168A>D	Substitution	Nonsynonymous coding	21%
MM04T	LHFPL5	lipoma HMGIC fusion partner-like 5	CCDS4812.1	chr6_35773483-35773483_G_T	12K>N	Substitution	Nonsynonymous coding	22%
MM04T	LIFR	leukemia inhibitory factor receptor alpha	CCDS3927.1	chr5_38489288-38489288_T_G	743K>Q	Substitution	Nonsynonymous coding	26%
MM04T	LILRA1	leukocyte immunoglobulin-like receptor, subfamily A (with TM domain), member 1	CCDS12901.1	chr19_55110749-55110749_C_T	437A>V	Substitution	Nonsynonymous coding	28%
MM04T	LILRA5	leukocyte immunoglobulin-like receptor, subfamily A (with TM domain), member 5	CCDS12888.1	chr19_54823891-54823891_C_T	2A>T	Substitution	Nonsynonymous coding	39%
MM04T	LILRB5	leukocyte immunoglobulin-like receptor, subfamily B (with TM and ITIM domains),	CCDS46176.1	chr19_54760640-54760640_G_T	NA	Substitution	Splice site acceptor	22%
MM04T	LIMCH1	LIM and calponin homology domains 1	CCDS33977.1	chr4_41496589-41496589_C_T	45R>W	Substitution	Nonsynonymous coding	18%
MM04T	LIMCH1	LIM and calponin homology domains 1	ENST00000313875	chr4_41635727-41635727_A_C	664R>S	Substitution	Nonsynonymous coding	32%

MM04T	LIMCH1	LIM and calponin homology domains 1	CCDS33977.1	chr4_41682098-41682098_C_T	815R>C	Substitution	Nonsynonymous coding	24%
MM04T	LIMCH1	LIM and calponin homology domains 1	CCDS33977.1	chr4_41694357-41694357_G_A	1061R>Q	Substitution	Nonsynonymous coding	31%
MM04T	LIMK2	LIM domain kinase 2	CCDS33637.1	chr22_31663853-31663853_A_G	386Y>C	Substitution	Nonsynonymous coding	30%
MM04T	LIMK2	LIM domain kinase 2	CCDS33637.1	chr22_31663018-31663018_C_T	343R>X	Substitution	Nonsense	31%
MM04T	LIN28B	lin-28 homolog B (C. elegans)	CCDS34504.1	chr6_105526432-105526432_C_A	176S>Y	Substitution	Nonsynonymous coding	37%
MM04T	LIN37	lin-37 homolog (C. elegans)	NM_019104	chr19_36244945-36244945_C_T	158R>C	Substitution	Nonsynonymous coding	30%
MM04T	LIN54	lin-54 homolog (C. elegans)	CCDS3599.1	chr4_83891556-83891556_A_C	292V>G	Substitution	Nonsynonymous coding	34%
MM04T	LIN7C	lin-7 homolog C (C. elegans)	CCDS7864.1	chr11_27523471-27523471_G_T	NA	Substitution	Splice site acceptor	28%
MM04T	LIN9	lin-9 homolog (C. elegans)	CCDS1553.1	chr1_226421127-226421127_G_T	464S>Y	Substitution	Nonsynonymous coding	22%
MM04T	LIN9	lin-9 homolog (C. elegans)	CCDS1553.1	chr1_226474059-226474059_G_A	183R>W	Substitution	Nonsynonymous coding	20%
MM04T	LINC00477	long intergenic non-protein coding RNA 477	ENST00000358374	chr12_24736816-24736816_G_A	80R>C	Substitution	Nonsynonymous coding	20%
MM04T	LIPC	lipase, hepatic	CCDS10166.1	chr15_58830636-58830636_C_T	65R>X	Substitution	Nonsense	30%
MM04T	LIPE	lipase, hormone-sensitive	CCDS12607.1	chr19_42907110-42907110_C_A	872K>N	Substitution	Nonsynonymous coding	34%
MM04T	LIPI	lipase, member I	CCDS13564.1	chr21_15537607-15537607_C_A	301D>Y	Substitution	Nonsynonymous coding	19%
MM04T	LIPI	lipase, member I	CCDS13564.1	chr21_15561394-15561394_T_G	152K>N	Substitution	Nonsynonymous coding	22%
MM04T	LIPM	lipase, family member M	CCDS44457.1	chr10_90574901-90574901_G_T	211A>S	Substitution	Nonsynonymous coding	29%
MM04T	LMAN1	lectin, mannose-binding, 1	CCDS11974.1	chr18_57000356-57000356_C_A	447K>N	Substitution	Nonsynonymous coding	24%
MM04T	LMAN2	lectin, mannose-binding 2	CCDS4417.1	chr5_176764447-176764447_C_T	198E>K	Substitution	Nonsynonymous coding	26%
MM04T	LMLN	leishmanolysin-like (metallopeptidase M8 family)	CCDS46988.1	chr3_197729916-197729916_G_T	421E>D	Substitution	Nonsynonymous coding	18%
MM04T	LMNB1	lamin B1	CCDS4140.1	chr5_126141288-126141288_A_G	181K>R	Substitution	Nonsynonymous coding	38%
MM04T	LMO7	LIM domain 7	CCDS9454.1	chr13_76378506-76378506_C_T	300S>L	Substitution	Nonsynonymous coding	26%
MM04T	LMO7	LIM domain 7	NM_015842	chr13_76382315-76382315_G_T	399K>N	Substitution	Nonsynonymous coding	22%
MM04T	LMO7	LIM domain 7	CCDS9454.1	chr13_76393550-76393550_C_T	414R>C	Substitution	Nonsynonymous coding	39%
MM04T	LMOD2	leiomodin 2 (cardiac)	CCDS47693.1	chr7_123302263-123302263_G_T	208S>I	Substitution	Nonsynonymous coding	32%
MM04T	LMX1B	LIM homeobox transcription factor 1, beta	CCDS6866.1	chr9_129455842-129455842_C_T	238R>C	Substitution	Nonsynonymous coding	17%
MM04T	LONRF3	LON peptidase N-terminal domain and ring finger 3	CCDS35374.1	chrX_118147138-118147138_G_A	650D>N	Substitution	Nonsynonymous coding	33%
MM04T	LONRF3	LON peptidase N-terminal domain and ring finger 3	CCDS35374.1	chrX_118151615-118151615_C_T	748R>X	Substitution	Nonsense	18%
MM04T	LPAR6	lysophosphatidic acid receptor 6	CCDS9410.1	chr13_48986147-48986147_G_A	138T>I	Substitution	Nonsynonymous coding	43%
MM04T	LPCAT4	lysophosphatidylcholine acyltransferase 4	CCDS32191.1	chr15_34653711-34653711_C_T	345A>T	Substitution	Nonsynonymous coding	26%
MM04T	LPIN1	lipin 1	CCDS1682.1	chr2_11964783-11964783_G_A	847D>N	Substitution	Nonsynonymous coding	35%
MM04T	LRBA	LPS-responsive vesicle trafficking, beach and anchor containing	CCDS3773.1	chr4_151827521-151827521_C_A	510K>N	Substitution	Nonsynonymous coding	16%
MM04T	LRCH1	leucine-rich repeats and calponin homology (CH) domain containing 1	CCDS31972.1	chr13_47297460-47297460_C_T	587R>C	Substitution	Nonsynonymous coding	23%

MM04T	LRCH2	leucine-rich repeats and calponin homology (CH) domain containing 2	CCDS48155.1	chrX_114384500-114384500_C_A	529E>X	Substitution	Nonsense	29%
MM04T	LRCH4	leucine-rich repeats and calponin homology (CH) domain containing 4	CCDS34706.1	chr7_100173914-100173914_G_A	529R>W	Substitution	Nonsynonymous coding	18%
MM04T	LRCH4	leucine-rich repeats and calponin homology (CH) domain containing 4	CCDS34706.1	chr7_100175865-100175865_A_C	289F>V	Substitution	Nonsynonymous coding	21%
MM04T	LRIF1	ligand dependent nuclear receptor interacting factor 1	CCDS30800.1	chr1_111490670-111490670_G_A	741R>X	Substitution	Nonsense	22%
MM04T	LRIT2	leucine-rich repeat, immunoglobulin-like and transmembrane domains 2	CCDS31234.1	chr10_85984698-85984698_G_T	95H>N	Substitution	Nonsynonymous coding	34%
MM04T	LRP1B	low density lipoprotein receptor-related protein 1B	ENST00000442974	chr2_141026845-141026845_C_T	183R>Q	Substitution	Nonsynonymous coding	10%
MM04T	LRP1B	low density lipoprotein receptor-related protein 1B	CCDS2182.1	chr2_141201978-141201978_C_A	3405K>N	Substitution	Nonsynonymous coding	32%
MM04T	LRP1B	low density lipoprotein receptor-related protein 1B	CCDS2182.1	chr2_141291694-141291694_C_T	2553R>Q	Substitution	Nonsynonymous coding	28%
MM04T	LRP2	low density lipoprotein receptor-related protein 2	CCDS2232.1	chr2_170011077-170011077_C_T	4063R>Q	Substitution	Nonsynonymous coding	29%
MM04T	LRP2	low density lipoprotein receptor-related protein 2	CCDS2232.1	chr2_170060708-170060708_C_T	2597G>S	Substitution	Nonsynonymous coding	13%
MM04T	LRP2	low density lipoprotein receptor-related protein 2	CCDS2232.1	chr2_170063556-170063556_C_T	2225R>Q	Substitution	Nonsynonymous coding	20%
MM04T	LRP2	low density lipoprotein receptor-related protein 2	CCDS2232.1	chr2_170063656-170063656_G_A	2192P>S	Substitution	Nonsynonymous coding	32%
MM04T	LRP2	low density lipoprotein receptor-related protein 2	CCDS2232.1	chr2_170066037-170066037_G_T	2132S>Y	Substitution	Nonsynonymous coding	28%
MM04T	LRP2	low density lipoprotein receptor-related protein 2	CCDS2232.1	chr2_170089947-170089947_G_A	1691A>V	Substitution	Nonsynonymous coding	31%
MM04T	LRP6	low density lipoprotein receptor-related protein 6	CCDS8647.1	chr12_12315190-12315190_C_T	739R>Q	Substitution	Nonsynonymous coding	24%
MM04T	LRP6	low density lipoprotein receptor-related protein 6	CCDS8647.1	chr12_12334106-12334106_C_T	415R>Q	Substitution	Nonsynonymous coding	33%
MM04T	LRR1	leucine rich repeat protein 1	CCDS9686.1	chr14_50074119-50074119_C_T	95A>V	Substitution	Nonsynonymous coding	26%
MM04T	LRRC10	leucine rich repeat containing 10	CCDS31856.1	chr12_70004573-70004573_G_A	16R>C	Substitution	Nonsynonymous coding	25%
MM04T	LRRC14B	leucine rich repeat containing 14B	CCDS47184.1	chr5_194832-194832_G_T	303Q>H	Substitution	Nonsynonymous coding	39%
MM04T	LRRC16A	leucine rich repeat containing 16A	NM_017640	chr6_25472656-25472656_G_T	261D>Y	Substitution	Nonsynonymous coding	22%
MM04T	LRRC16A	leucine rich repeat containing 16A	NM_017640	chr6_25482569-25482569_A_C	320K>T	Substitution	Nonsynonymous coding	29%
MM04T	LRRC16A	leucine rich repeat containing 16A	NM_017640	chr6_25581521-25581521_G_A	954E>K	Substitution	Nonsynonymous coding	27%
MM04T	LRRC16B	leucine rich repeat containing 16B	CCDS32054.1	chr14_24526433-24526433_A_G	374T>A	Substitution	Nonsynonymous coding	20%
MM04T	LRRC18	leucine rich repeat containing 18	CCDS31197.1	chr10_50122157-50122157_A_G	15L>P	Substitution	Nonsynonymous coding	31%
MM04T	LRRC19	leucine rich repeat containing 19	CCDS6518.1	chr9_26997828-26997828_G_T	165L>I	Substitution	Nonsynonymous coding	22%
MM04T	LRRC2	leucine rich repeat containing 2	CCDS2741.1	chr3_46563069-46563069_G_A	337R>C	Substitution	Nonsynonymous coding	35%
MM04T	LRRC23	leucine rich repeat containing 23	CCDS8569.1	chr12_7022090-7022090_C_T	319R>X	Substitution	Nonsense	36%
MM04T	LRRC27	leucine rich repeat containing 27	CCDS31316.1	chr10_134175022-134175022_C_T	411P>L	Substitution	Nonsynonymous coding	29%
MM04T	LRRC27	leucine rich repeat containing 27	CCDS31316.1	chr10_134188732-134188732_C_T	527R>C	Substitution	Nonsynonymous coding	36%
MM04T	LRRC30	leucine rich repeat containing 30	CCDS42409.1	chr18_7231828-7231828_T_C	231V>A	Substitution	Nonsynonymous coding	20%
MM04T	LRRC39	leucine rich repeat containing 39	CCDS766.1	chr1_100617949-100617949_C_A	315R>I	Substitution	Nonsynonymous coding	28%
MM04T	LRRC3B	leucine rich repeat containing 3B	CCDS2644.1	chr3_26751258-26751258_G_A	32S>N	Substitution	Nonsynonymous coding	35%

MM04T	LRRC49	leucine rich repeat containing 49	CCDS32282.1	chr15_71211448-71211448_G_T	209R>S	Substitution	Nonsynonymous coding	19%
MM04T	LRRC49	leucine rich repeat containing 49	CCDS32282.1	chr15_71276544-71276544_G_T	373D>Y	Substitution	Nonsynonymous coding	32%
MM04T	LRRC49	leucine rich repeat containing 49	CCDS32282.1	chr15_71329531-71329531_C_T	573R>W	Substitution	Nonsynonymous coding	33%
MM04T	LRRC4B	leucine rich repeat containing 4B	CCDS42595.1	chr19_51022603-51022603_G_A	123R>C	Substitution	Nonsynonymous coding	25%
MM04T	LRRC4C	leucine rich repeat containing 4C	CCDS31464.1	chr11_40135960-40135960_C_T	628R>Q	Substitution	Nonsynonymous coding	44%
MM04T	LRRC4C	leucine rich repeat containing 4C	CCDS31464.1	chr11_40137334-40137334_C_A	170R>I	Substitution	Nonsynonymous coding	26%
MM04T	LRRC53	leucine rich repeat containing 53	ENST00000416014	chr1_74946244-74946244_T_C	166N>S	Substitution	Nonsynonymous coding	36%
MM04T	LRRC56	leucine rich repeat containing 56	CCDS7700.1	chr11_541564-541564_C_T	69R>W	Substitution	Nonsynonymous coding	27%
MM04T	LRRC59	leucine rich repeat containing 59	CCDS11566.1	chr17_48462638-48462638_G_A	173R>C	Substitution	Nonsynonymous coding	22%
MM04T	LRRC73	leucine rich repeat containing 73	CCDS34456.1	chr6_43475406-43475406_T_C	223D>G	Substitution	Nonsynonymous coding	33%
MM04T	LRRC8A	leucine rich repeat containing 8 family, member A	CCDS35155.1	chr9_131670170-131670170_C_A	243L>M	Substitution	Nonsynonymous coding	21%
MM04T	LRRC8C	leucine rich repeat containing 8 family, member C	CCDS725.1	chr1_90179249-90179249_C_T	374L>F	Substitution	Nonsynonymous coding	39%
MM04T	LRRCC1	leucine rich repeat and coiled-coil centrosomal protein 1	CCDS43750.1	chr8_86035691-86035691_C_A	325P>Q	Substitution	Nonsynonymous coding	15%
MM04T	LRRFIP1	leucine rich repeat (in FLII) interacting protein 1	CCDS46552.1	chr2_238672520-238672520_G_A	722D>N	Substitution	Nonsynonymous coding	31%
MM04T	LRRFIP2	leucine rich repeat (in FLII) interacting protein 2	CCDS2664.1	chr3_37136305-37136305_T_G	338D>A	Substitution	Nonsynonymous coding	28%
MM04T	LRRIQ1	leucine-rich repeats and IQ motif containing 1	CCDS41816.1	chr12_85450097-85450097_A_C	509K>T	Substitution	Nonsynonymous coding	34%
MM04T	LRRIQ1	leucine-rich repeats and IQ motif containing 1	CCDS41816.1	chr12_85450583-85450583_G_T	671R>I	Substitution	Nonsynonymous coding	36%
MM04T	LRRIQ1	leucine-rich repeats and IQ motif containing 1	CCDS41816.1	chr12_85459103-85459103_A_C	819N>H	Substitution	Nonsynonymous coding	29%
MM04T	LRRIQ1	leucine-rich repeats and IQ motif containing 1	CCDS41816.1	chr12_85450348-85450348_G_T	593E>X	Substitution	Nonsense	28%
MM04T	LRRIQ1	leucine-rich repeats and IQ motif containing 1	CCDS41816.1	chr12_85450933-85450933_C_T	788R>X	Substitution	Nonsense	34%
MM04T	LRRIQ1	leucine-rich repeats and IQ motif containing 1	CCDS41816.1	chr12_85459124-85459124_C_T	826R>X	Substitution	Nonsense	34%
MM04T	LRRIQ3	leucine-rich repeats and IQ motif containing 3	CCDS41350.1	chr1_74506974-74506974_C_A	547E>D	Substitution	Nonsynonymous coding	28%
MM04T	LRRIQ4	leucine-rich repeats and IQ motif containing 4	CCDS46951.1	chr3_169539767-169539767_C_T	20P>S	Substitution	Nonsynonymous coding	32%
MM04T	LRRK1	leucine-rich repeat kinase 1	CCDS42086.1	chr15_101562761-101562761_A_G	676T>A	Substitution	Nonsynonymous coding	31%
MM04T	LRRK2	leucine-rich repeat kinase 2	CCDS31774.1	chr12_40693001-40693001_G_T	1146E>D	Substitution	Nonsynonymous coding	19%
MM04T	LRRK2	leucine-rich repeat kinase 2	CCDS31774.1	chr12_40713792-40713792_T_G	1610I>M	Substitution	Nonsynonymous coding	28%
MM04T	LRRK2	leucine-rich repeat kinase 2	CCDS31774.1	chr12_40716230-40716230_G_T	1809K>N	Substitution	Nonsynonymous coding	31%
MM04T	LRRK2	leucine-rich repeat kinase 2	CCDS31774.1	chr12_40697937-40697937_G_A	NA	Substitution	Splice site donor	28%
MM04T	LRRK2	leucine-rich repeat kinase 2	CCDS31774.1	chr12_40677866-40677866_G_T	811G>X	Substitution	Nonsense	26%
MM04T	LRRK2	leucine-rich repeat kinase 2	CCDS31774.1	chr12_40697829-40697829_G_T	1224E>X	Substitution	Nonsense	27%
MM04T	LRRN1	leucine rich repeat neuronal 1	CCDS33685.1	chr3_3887610-3887610_G_A	429D>N	Substitution	Nonsynonymous coding	22%
MM04T	LRRN1	leucine rich repeat neuronal 1	CCDS33685.1	chr3_3888129-3888129_C_A	602H>N	Substitution	Nonsynonymous coding	32%

MM04T	LRRN3	leucine rich repeat neuronal 3	CCDS5754.1	chr7_110763046-110763046_T_G	73I>S	Substitution	Nonsynonymous coding	23%
MM04T	LRRN3	leucine rich repeat neuronal 3	CCDS5754.1	chr7_110763912-110763912_G_T	362E>X	Substitution	Nonsense	21%
MM04T	LRRTM3	leucine rich repeat transmembrane neuronal 3	CCDS7270.1	chr10_68687114-68687114_C_A	147S>Y	Substitution	Nonsynonymous coding	28%
MM04T	LRTOMT	leucine rich transmembrane and 0-methyltransferase domain containing	CCDS44668.1	chr11_71817157-71817157_C_A	87L>I	Substitution	Nonsynonymous coding	51%
MM04T	LSG1	large subunit GTPase 1 homolog (S. cerevisiae)	CCDS33922.1	chr3_194373592-194373592_G_A	347R>W	Substitution	Nonsynonymous coding	24%
MM04T	LSG1	large subunit GTPase 1 homolog (S. cerevisiae)	CCDS33922.1	chr3_194387300-194387300_C_A	76E>D	Substitution	Nonsynonymous coding	24%
MM04T	LSP1	lymphocyte-specific protein 1	CCDS31334.1	chr11_1905219-1905219_G_A	186E>K	Substitution	Nonsynonymous coding	11%
MM04T	LTA4H	leukotriene A4 hydrolase	CCDS9059.1	chr12_96394808-96394808_C_T	599V>M	Substitution	Nonsynonymous coding	27%
MM04T	LTBP2	latent transforming growth factor beta binding protein 2	CCDS9831.1	chr14_74995798-74995798_C_T	672G>E	Substitution	Nonsynonymous coding	35%
MM04T	LTBP4	latent transforming growth factor beta binding protein 4	ENST00000308370	chr19_41133107-41133107_G_A	1471D>N	Substitution	Nonsynonymous coding	24%
MM04T	LTN1	listerin E3 ubiquitin protein ligase 1	CCDS33527.1	chr21_30318561-30318561_C_A	1179S>I	Substitution	Nonsynonymous coding	21%
MM04T	LTN1	listerin E3 ubiquitin protein ligase 1	CCDS33527.1	chr21_30343035-30343035_C_A	338K>N	Substitution	Nonsynonymous coding	22%
MM04T	LUC7L2	LUC7-like 2 (S. cerevisiae)	CCDS43656.1	chr7_139106937-139106937_G_T	344D>Y	Substitution	Nonsynonymous coding	26%
MM04T	LUM	lumican	CCDS9038.1	chr12_91502012-91502012_T_G	249N>H	Substitution	Nonsynonymous coding	36%
MM04T	LUM	lumican	CCDS9038.1	chr12_91502496-91502496_C_A	87E>D	Substitution	Nonsynonymous coding	26%
MM04T	LUZP2	leucine zipper protein 2	CCDS31446.1	chr11_25100105-25100105_G_T	314L>F	Substitution	Nonsynonymous coding	35%
MM04T	LUZP4	leucine zipper protein 4	CCDS14567.1	chrX_114536641-114536641_C_T	59S>L	Substitution	Nonsynonymous coding	20%
MM04T	LY6E	lymphocyte antigen 6 complex, locus E	CCDS6394.1	chr8_144102737-144102737_C_T	20S>L	Substitution	Nonsynonymous coding	30%
MM04T	LY75	lymphocyte antigen 75	CCDS2211.1	chr2_160692081-160692081_C_T	1195E>K	Substitution	Nonsynonymous coding	22%
MM04T	LY75	lymphocyte antigen 75	CCDS2211.1	chr2_160737752-160737752_C_A	NA	Substitution	Splice site acceptor	45%
MM04T	LYAR	Ly1 antibody reactive	CCDS3374.1	chr4_4283564-4283564__T	NA	Insertion	Frameshift	36%
MM04T	LYPLAL1	lysophospholipase-like 1	CCDS1522.1	chr1_219352563-219352563_A_C	56I>L	Substitution	Nonsynonymous coding	19%
MM04T	LYRM5	LYR motif containing 5	NM_001001660	chr12_25357133-25357133_G_T	54E>X	Substitution	Nonsense	27%
MM04T	LYST	lysosomal trafficking regulator	CCDS31062.1	chr1_235937215-235937215_G_T	1904S>Y	Substitution	Nonsynonymous coding	26%
MM04T	LYST	lysosomal trafficking regulator	CCDS31062.1	chr1_235964271-235964271_G_A	1280S>F	Substitution	Nonsynonymous coding	27%
MM04T	MAB21L3	mab-21-like 3 (C. elegans)	CCDS886.1	chr1_116670245-116670245_G_T	214E>X	Substitution	Nonsense	21%
MM04T	MACF1	microtubule-actin crosslinking factor 1	CCDS436.1	chr1_39797308-39797308_C_T	123A>V	Substitution	Nonsynonymous coding	37%
MM04T	MACF1	microtubule-actin crosslinking factor 1	CCDS436.1	chr1_39800390-39800390_G_T	1150E>D	Substitution	Nonsynonymous coding	19%
MM04T	MACF1	microtubule-actin crosslinking factor 1	CCDS436.1	chr1_39918368-39918368_C_T	5319R>X	Substitution	Nonsense	28%
MM04T	MADD	MAP-kinase activating death domain	CCDS7930.1	chr11_47311028-47311028_C_T	934R>X	Substitution	Nonsense	22%
MM04T	MAGEA10	melanoma antigen family A, 10	CCDS14705.1	chrX_151303041-151303041_G_A	351T>I	Substitution	Nonsynonymous coding	23%
MM04T	MAGEB18	melanoma antigen family B, 18	CCDS14216.1	chrX_26157161-26157161_G_T	20C>F	Substitution	Nonsynonymous coding	31%

MM04T	MAGEB2	melanoma antigen family B, 2	CCDS14219.1	chrX_30236803-30236803_G_A	36E>K	Substitution	Nonsynonymous coding	40%
MM04T	MAGEB4	melanoma antigen family B, 4	CCDS14221.1	chrX_30260427-30260427_C_A	59P>T	Substitution	Nonsynonymous coding	19%
MM04T	MAGEC1	melanoma antigen family C, 1	CCDS35417.1	chrX_140995658-140995658_C_T	823S>L	Substitution	Nonsynonymous coding	26%
MM04T	MAGEC1	melanoma antigen family C, 1	CCDS35417.1	chrX_140996050-140996050_C_T	954R>C	Substitution	Nonsynonymous coding	21%
MM04T	MAGEC2	melanoma antigen family C, 2	CCDS14678.1	chrX_141291178-141291178_T_C	199E>G	Substitution	Nonsynonymous coding	24%
MM04T	MAGED1	melanoma antigen family D, 1	CCDS35279.1	chrX_51639876-51639876_G_T	431Q>H	Substitution	Nonsynonymous coding	31%
MM04T	MAGED1	melanoma antigen family D, 1	CCDS35279.1	chrX_51640912-51640912_G_T	586E>X	Substitution	Nonsense	22%
MM04T	MAGEE1	melanoma antigen family E, 1	CCDS14433.1	chrX_75649870-75649870_A_G	516E>G	Substitution	Nonsynonymous coding	39%
MM04T	MAGEE1	melanoma antigen family E, 1	CCDS14433.1	chrX_75650296-75650296_G_T	658R>I	Substitution	Nonsynonymous coding	17%
MM04T	MAGEE1	melanoma antigen family E, 1	CCDS14433.1	chrX_75651135-75651135_G_A	938E>K	Substitution	Nonsynonymous coding	29%
MM04T	MAGEE2	melanoma antigen family E, 2	CCDS14431.1	chrX_75003449-75003449_C_A	480V>F	Substitution	Nonsynonymous coding	28%
MM04T	MAGEL2	MAGE-like 2	NM_019066	chr15_23890547-23890547_C_A	781E>D	Substitution	Nonsynonymous coding	18%
MM04T	MAGI1	membrane associated guanylate kinase, WW and PDZ domain containing 1	CCDS33780.1	chr3_65342147-65342147_G_A	1432A>V	Substitution	Nonsynonymous coding	29%
MM04T	MAGI2	membrane associated guanylate kinase, WW and PDZ domain containing 2	CCDS5594.1	chr7_77708284-77708284_G_A	1229T>M	Substitution	Nonsynonymous coding	21%
MM04T	MAGI2	membrane associated guanylate kinase, WW and PDZ domain containing 2	CCDS5594.1	chr7_77824214-77824214_G_T	749S>Y	Substitution	Nonsynonymous coding	30%
MM04T	MAGI2	membrane associated guanylate kinase, WW and PDZ domain containing 2	CCDS5594.1	chr7_77998488-77998488_C_T	363G>D	Substitution	Nonsynonymous coding	40%
MM04T	MAGI2	membrane associated guanylate kinase, WW and PDZ domain containing 2	CCDS5594.1	chr7_78256462-78256462_G_A	171A>V	Substitution	Nonsynonymous coding	32%
MM04T	MAGI3	membrane associated guanylate kinase, WW and PDZ domain containing 3	CCDS860.1	chr1_114224842-114224842_C_A	1113P>T	Substitution	Nonsynonymous coding	24%
MM04T	MAGT1	magnesium transporter 1	CCDS14436.2	chrX_77096749-77096749_G_A	331R>X	Substitution	Nonsense	35%
MM04T	MAK	male germ cell-associated kinase	CCDS4516.1	chr6_10819153-10819153_G_T	41S>Y	Substitution	Nonsynonymous coding	37%
MM04T	MAL	mal, T-cell differentiation protein	CCDS2006.1	chr2_95719196-95719196_C_A	153S>X	Substitution	Nonsense	18%
MM04T	MAMDC2	MAM domain containing 2	CCDS6631.1	chr9_72727944-72727944_T_C	180F>S	Substitution	Nonsynonymous coding	25%
MM04T	MAMLD1	mastermind-like domain containing 1	CCDS14693.2	chrX_149638364-149638364_C_A	173D>E	Substitution	Nonsynonymous coding	13%
MM04T	MAMLD1	mastermind-like domain containing 1	CCDS14693.2	chrX_149638416-149638416_C_A	191P>T	Substitution	Nonsynonymous coding	31%
MM04T	MAN2A2	mannosidase, alpha, class 2A, member 2	CCDS32332.1	chr15_91450624-91450624_G_T	365K>N	Substitution	Nonsynonymous coding	19%
MM04T	MANBA	mannosidase, beta A, lysosomal	CCDS3658.1	chr4_103644033-103644033_G_A	182R>W	Substitution	Nonsynonymous coding	40%
MM04T	MANBA	mannosidase, beta A, lysosomal	CCDS3658.1	chr4_103610842-103610842_C_A	NA	Substitution	Splice site acceptor	26%
MM04T	MAOA	monoamine oxidase A	CCDS14260.1	chrX_43590966-43590966_C_A	274P>H	Substitution	Nonsynonymous coding	26%
MM04T	MAP1A	microtubule-associated protein 1A	CCDS42031.1	chr15_43819880-43819880_G_T	2070S>I	Substitution	Nonsynonymous coding	32%
MM04T	MAP1B	microtubule-associated protein 1B	CCDS4012.1	chr5_71489773-71489773_G_T	197K>N	Substitution	Nonsynonymous coding	25%
MM04T	MAP1B	microtubule-associated protein 1B	CCDS4012.1	chr5_71491465-71491465_G_T	761K>N	Substitution	Nonsynonymous coding	18%
MM04T	MAP1B	microtubule-associated protein 1B	CCDS4012.1	chr5_71491652-71491652_G_A	824E>K	Substitution	Nonsynonymous coding	35%

MM04T	MAP1B	microtubule-associated protein 1B	CCDS4012.1	chr5_71491772-71491772_G_A	864E>K	Substitution	Nonsynonymous coding	31%
MM04T	MAP1B	microtubule-associated protein 1B	CCDS4012.1	chr5_71489692-71489692_G_A	NA	Substitution	Splice site acceptor	29%
MM04T	MAP2	microtubule-associated protein 2	ENST00000445941	chr2_210555364-210555364_G_T	164E>D	Substitution	Nonsynonymous coding	34%
MM04T	MAP2	microtubule-associated protein 2	CCDS2384.1	chr2_210570347-210570347_G_T	1543R>I	Substitution	Nonsynonymous coding	29%
MM04T	MAP2K4	mitogen-activated protein kinase kinase 4	CCDS11162.1	chr17_11984773-11984773_G_T	107E>X	Substitution	Nonsense	21%
MM04T	MAP2K4	mitogen-activated protein kinase kinase 4	CCDS11162.1	chr17_12011200-12011200_G_T	203E>X	Substitution	Nonsense	35%
MM04T	MAP2K6	mitogen-activated protein kinase kinase 6	CCDS11686.1	chr17_67515559-67515559_G_A	118A>T	Substitution	Nonsynonymous coding	30%
MM04T	MAP2K7	mitogen-activated protein kinase kinase 7	CCDS42491.1	chr19_7968853-7968853_G_T	8Q>H	Substitution	Nonsynonymous coding	19%
MM04T	MAP2K7	mitogen-activated protein kinase kinase 7	ENST00000425613	chr19_7975124-7975124_C_A	147Q>K	Substitution	Nonsynonymous coding	26%
MM04T	MAP3K15	mitogen-activated protein kinase kinase kinase 15	CCDS35212.1	chrX_19418711-19418711_C_T	114D>N	Substitution	Nonsynonymous coding	34%
MM04T	MAP3K15	mitogen-activated protein kinase kinase kinase 15	CCDS35212.1	chrX_19418775-19418775_G_T	92F>L	Substitution	Nonsynonymous coding	28%
MM04T	MAP3K19	mitogen-activated protein kinase kinase kinase 19	CCDS2176.2	chr2_135743936-135743936_C_A	836D>Y	Substitution	Nonsynonymous coding	23%
MM04T	MAP3K19	mitogen-activated protein kinase kinase kinase 19	CCDS2176.2	chr2_135744808-135744808_C_A	545S>I	Substitution	Nonsynonymous coding	28%
MM04T	MAP3K19	mitogen-activated protein kinase kinase kinase 19	CCDS2176.2	chr2_135745210-135745210_C_A	411R>I	Substitution	Nonsynonymous coding	33%
MM04T	MAP3K19	mitogen-activated protein kinase kinase kinase 19	CCDS2176.2	chr2_135745593-135745593_G_T	283F>L	Substitution	Nonsynonymous coding	29%
MM04T	MAP3K19	mitogen-activated protein kinase kinase kinase 19	CCDS2176.2	chr2_135745711-135745711_A_C	244F>C	Substitution	Nonsynonymous coding	35%
MM04T	MAP3K2	mitogen-activated protein kinase kinase kinase 2	CCDS46404.1	chr2_128072339-128072339_C_A	483D>Y	Substitution	Nonsynonymous coding	36%
MM04T	MAP3K5	mitogen-activated protein kinase kinase kinase 5	CCDS5179.1	chr6_136935369-136935369_T_G	736N>H	Substitution	Nonsynonymous coding	28%
MM04T	MAP3K5	mitogen-activated protein kinase kinase kinase 5	CCDS5179.1	chr6_137017107-137017107_T_C	355Y>C	Substitution	Nonsynonymous coding	23%
MM04T	MAP3K6	mitogen-activated protein kinase kinase kinase 6	CCDS299.1	chr1_27687856-27687856_C_T	573A>T	Substitution	Nonsynonymous coding	27%
MM04T	MAP3K7	mitogen-activated protein kinase kinase kinase 7	CCDS5028.1	chr6_91278339-91278339_G_A	79R>W	Substitution	Nonsynonymous coding	30%
MM04T	MAP3K8	mitogen-activated protein kinase kinase kinase 8	CCDS7166.1	chr10_30728021-30728021_G_T	52D>Y	Substitution	Nonsynonymous coding	31%
MM04T	MAP4K1	mitogen-activated protein kinase kinase kinase kinase 1	CCDS42564.1	chr19_39086348-39086348_G_A	734P>L	Substitution	Nonsynonymous coding	20%
MM04T	MAP6	microtubule-associated protein 6	CCDS31641.1	chr11_75298132-75298132_T_C	805H>R	Substitution	Nonsynonymous coding	24%
MM04T	MAP7	microtubule-associated protein 7	CCDS5178.1	chr6_136704883-136704883_G_A	188S>L	Substitution	Nonsynonymous coding	34%
MM04T	MAP7D3	MAP7 domain containing 3	CCDS44004.1	chrX_135313984-135313984_G_A	378P>S	Substitution	Nonsynonymous coding	23%
MM04T	MAP7D3	MAP7 domain containing 3	CCDS44004.1	chrX_135322636-135322636_C_T	182R>Q	Substitution	Nonsynonymous coding	37%
MM04T	MAP7D3	MAP7 domain containing 3	CCDS44004.1	chrX_135328491-135328491_G_A	27A>V	Substitution	Nonsynonymous coding	18%
MM04T	MAP9	microtubule-associated protein 9	CCDS35493.1	chr4_156281568-156281568_C_A	NA	Substitution	Splice site acceptor	40%
MM04T	MAPK10	mitogen-activated protein kinase 10	CCDS34026.1	chr4_87080614-87080614_C_T	25D>N	Substitution	Nonsynonymous coding	35%
MM04T	MAPK12	mitogen-activated protein kinase 12	CCDS14089.1	chr22_50693933-50693933_C_T	267E>K	Substitution	Nonsynonymous coding	33%
MM04T	MAPK8	mitogen-activated protein kinase 8	CCDS7223.1	chr10_49632554-49632554_C_A	NA	Substitution	Splice site acceptor	20%

MM04T	MAPK8IP2	mitogen-activated protein kinase 8 interacting protein 2	ENST00000329492	chr22_51042329-51042329_C_T	201R>C	Substitution	Nonsynonymous coding	20%
MM04T	MAPKAPK3	mitogen-activated protein kinase-activated protein kinase 3	CCDS2832.1	chr3_50683163-50683163_G_T	217E>D	Substitution	Nonsynonymous coding	16%
MM04T	MAPRE1	microtubule-associated protein, RP/EB family, member 1	CCDS13208.1	chr20_31424651-31424651_A_C	NA	Substitution	Splice site donor	19%
MM04T	MAPT	microtubule-associated protein tau	CCDS45715.1	chr17_44061192-44061192_C_A	341S>Y	Substitution	Nonsynonymous coding	25%
MM04T	MAPT	microtubule-associated protein tau	CCDS45715.1	chr17_44067392-44067392_C_A	444S>Y	Substitution	Nonsynonymous coding	25%
MM04T	MARCKS	myristoylated alanine-rich protein kinase C substrate	CCDS5101.1	chr6_114181513-114181513_G_A	253D>N	Substitution	Nonsynonymous coding	26%
MM04T	MARK2	MAP/microtubule affinity-regulating kinase 2	CCDS41665.1	chr11_63676642-63676642_G_A	724R>Q	Substitution	Nonsynonymous coding	35%
MM04T	MARK2	MAP/microtubule affinity-regulating kinase 2	NM_001039469	chr11_63662630-63662630_G_A	NA	Substitution	Splice site acceptor	61%
MM04T	MARK3	MAP/microtubule affinity-regulating kinase 3	CCDS45165.1	chr14_103932133-103932133_A_T	NA	Substitution	Splice site donor	32%
MM04T	MARK4	MAP/microtubule affinity-regulating kinase 4	CCDS12658.1	chr19_45801448-45801448_C_T	642S>L	Substitution	Nonsynonymous coding	30%
MM04T	MARS2	methionyl-tRNA synthetase 2, mitochondrial	CCDS33358.1	chr2_198570529-198570529_G_T	134D>Y	Substitution	Nonsynonymous coding	25%
MM04T	MAS1L	MAS1 oncogene-like	CCDS4661.1	chr6_29455628-29455628_A_G	18F>L	Substitution	Nonsynonymous coding	18%
MM04T	MASP1	mannan-binding lectin serine peptidase 1 (C4/C2 activating component of Ra-reactive	CCDS33908.1	chr3_186969513-186969513_G_T	307P>H	Substitution	Nonsynonymous coding	22%
MM04T	MASP2	mannan-binding lectin serine peptidase 2	CCDS123.1	chr1_11087510-11087510_C_T	498R>Q	Substitution	Nonsynonymous coding	34%
MM04T	MAST2	microtubule associated serine/threonine kinase 2	CCDS41326.1	chr1_46348061-46348061_G_A	165G>D	Substitution	Nonsynonymous coding	27%
MM04T	MAST4	microtubule associated serine/threonine kinase family member 4	CCDS47225.1	chr5_66461609-66461609_G_A	2012G>D	Substitution	Nonsynonymous coding	19%
MM04T	MASTL	microtubule associated serine/threonine kinase-like	CCDS7153.1	chr10_27459657-27459657_C_A	590S>X	Substitution	Nonsense	25%
MM04T	MAT2A	methionine adenosyltransferase II, alpha	CCDS1977.1	chr2_85768837-85768837_G_T	125R>I	Substitution	Nonsynonymous coding	18%
MM04T	MATN3	matrilin 3	CCDS46226.1	chr2_20202946-20202946_T_C	298N>D	Substitution	Nonsynonymous coding	18%
MM04T	MB21D2	Mab-21 domain containing 2	CCDS3302.2	chr3_192516963-192516963_G_A	230R>C	Substitution	Nonsynonymous coding	40%
MM04T	MBD4	methyl-CpG binding domain protein 4	CCDS3058.1	chr3_129155864-129155864_G_T	208T>N	Substitution	Nonsynonymous coding	34%
MM04T	MBD5	methyl-CpG binding domain protein 5	CCDS33302.1	chr2_149247795-149247795_C_T	1299R>W	Substitution	Nonsynonymous coding	24%
MM04T	MBOAT2	membrane bound O-acyltransferase domain containing 2	CCDS1660.1	chr2_8998822-8998822_G_T	517S>X	Substitution	Nonsense	21%
MM04T	MBOAT4	membrane bound O-acyltransferase domain containing 4	CCDS47835.1	chr8_29989535-29989535_G_A	411S>L	Substitution	Nonsynonymous coding	24%
MM04T	MBP	myelin basic protein	ENST00000397869	chr18_74700368-74700368_G_A	145S>L	Substitution	Nonsynonymous coding	12%
MM04T	MBTPS1	membrane-bound transcription factor peptidase, site 1	CCDS10941.1	chr16_84132690-84132690_C_T	130R>Q	Substitution	Nonsynonymous coding	34%
MM04T	MBTPS1	membrane-bound transcription factor peptidase, site 1	CCDS10941.1	chr16_84132822-84132822_C_A	86R>I	Substitution	Nonsynonymous coding	28%
MM04T	MBTPS2	membrane-bound transcription factor peptidase, site 2	CCDS14201.1	chrX_21869702-21869702_G_T	172E>X	Substitution	Nonsense	21%
MM04T	MC1R	melanocortin 1 receptor (alpha melanocyte stimulating hormone receptor)	NM_002386	chr16_89986439-89986439_T_C	258F>S	Substitution	Nonsynonymous coding	32%
MM04T	MC4R	melanocortin 4 receptor	CCDS11976.1	chr18_58039473-58039473_T_C	37D>G	Substitution	Nonsynonymous coding	21%
MM04T	MCF2	MCF.2 cell line derived transforming sequence	CCDS48175.1	chrX_138669893-138669893_G_T	895S>Y	Substitution	Nonsynonymous coding	15%
MM04T	MCF2	MCF.2 cell line derived transforming sequence	CCDS48175.1	chrX_138698599-138698599_G_T	405L>I	Substitution	Nonsynonymous coding	18%

MM04T	MCF2	MCF.2 cell line derived transforming sequence	CCDS48175.1	chrX_138711973-138711973_C_A	167E>X	Substitution	Nonsense	32%
MM04T	MCF2L	MCF.2 cell line derived transforming sequence-like	CCDS45070.1	chr13_113724410-113724410_G_A	364D>N	Substitution	Nonsynonymous coding	30%
MM04T	MCF2L2	MCF.2 cell line derived transforming sequence-like 2	CCDS3243.1	chr3_183027524-183027524_T_G	365K>Q	Substitution	Nonsynonymous coding	41%
MM04T	MCF2L2	MCF.2 cell line derived transforming sequence-like 2	CCDS3243.1	chr3_183097151-183097151_G_A	70S>L	Substitution	Nonsynonymous coding	33%
MM04T	MCF2L2	MCF.2 cell line derived transforming sequence-like 2	CCDS3243.1	chr3_183028701-183028701_A_G	NA	Substitution	Splice site donor	51%
MM04T	MCM10	minichromosome maintenance complex component 10	CCDS7096.1	chr10_13237180-13237180_C_T	630R>X	Substitution	Nonsense	25%
MM04T	MCM3AP	minichromosome maintenance complex component 3 associated protein	CCDS13734.1	chr21_47695227-47695227_C_A	624R>I	Substitution	Nonsynonymous coding	22%
MM04T	MCM3AP	minichromosome maintenance complex component 3 associated protein	CCDS13734.1	chr21_47704855-47704855_T_C	116T>A	Substitution	Nonsynonymous coding	41%
MM04T	MCM4	minichromosome maintenance complex component 4	CCDS6143.1	chr8_48878971-48878971_C_T	NA	Substitution	Splice site donor	38%
MM04T	MCM8	minichromosome maintenance complex component 8	CCDS13094.1	chr20_5974327-5974327_G_T	806E>X	Substitution	Nonsense	63%
MM04T	MCOLN1	mucolipin 1	CCDS12180.1	chr19_7593569-7593569_C_T	322R>X	Substitution	Nonsense	33%
MM04T	MCOLN3	mucolipin 3	CCDS701.1	chr1_85510812-85510812_T_G	NA	Substitution	Splice site donor	31%
MM04T	MCTP1	multiple C2 domains, transmembrane 1	CCDS34203.1	chr5_94289024-94289024_C_A	294E>X	Substitution	Nonsense	23%
MM04T	MCTP2	multiple C2 domains, transmembrane 2	CCDS32338.1	chr15_95001462-95001462_G_A	783E>K	Substitution	Nonsynonymous coding	36%
MM04T	MDGA2	MAM domain containing glycosylphosphatidylinositol anchor 2	ENST00000399222	chr14_47566339-47566339_A_C	NA	Substitution	Splice site acceptor	31%
MM04T	MDGA2	MAM domain containing glycosylphosphatidylinositol anchor 2	CCDS45098.1	chr14_47343401-47343401_C_A	745E>X	Substitution	Nonsense	47%
MM04T	MDN1	MDN1, midasin homolog (yeast)	CCDS5024.1	chr6_90372673-90372673_C_A	4750E>D	Substitution	Nonsynonymous coding	32%
MM04T	MDN1	MDN1, midasin homolog (yeast)	CCDS5024.1	chr6_90450000-90450000_G_A	1516R>C	Substitution	Nonsynonymous coding	33%
MM04T	MED12L	mediator complex subunit 12-like	CCDS33876.1	chr3_151082934-151082934_G_A	1007R>H	Substitution	Nonsynonymous coding	34%
MM04T	MED13	mediator complex subunit 13	CCDS42366.1	chr17_60039090-60039090_T_G	1705E>D	Substitution	Nonsynonymous coding	36%
MM04T	MED13	mediator complex subunit 13	CCDS42366.1	chr17_60050234-60050234_C_T	1274C>Y	Substitution	Nonsynonymous coding	28%
MM04T	MED13	mediator complex subunit 13	CCDS42366.1	chr17_60050217-60050217_G_A	1280R>X	Substitution	Nonsense	31%
MM04T	MED13L	mediator complex subunit 13-like	CCDS9177.1	chr12_116446456-116446456_C_A	588E>X	Substitution	Nonsense	35%
MM04T	MED25	mediator complex subunit 25	CCDS33075.1	chr19_50321714-50321714_A_G	39Y>C	Substitution	Nonsynonymous coding	25%
MM04T	MED6	mediator complex subunit 6	CCDS9805.1	chr14_71059612-71059612_C_A	151D>Y	Substitution	Nonsynonymous coding	28%
MM04T	MEDAG	mesenteric estrogen-dependent adipogenesis	CCDS9338.1	chr13_31480737-31480737_G_A	29D>N	Substitution	Nonsynonymous coding	24%
MM04T	MEF2A	myocyte enhancer factor 2A	CCDS45362.1	chr15_100185767-100185767_T_C	19V>A	Substitution	Nonsynonymous coding	67%
MM04T	MEF2C	myocyte enhancer factor 2C	CCDS47245.1	chr5_88024415-88024415_G_A	332S>F	Substitution	Nonsynonymous coding	23%
MM04T	MEFV	Mediterranean fever	CCDS10498.1	chr16_3304380-3304380_C_T	230E>K	Substitution	Nonsynonymous coding	23%
MM04T	MEGF10	multiple EGF-like-domains 10	CCDS4142.1	chr5_126791189-126791189_C_T	1041P>L	Substitution	Nonsynonymous coding	20%
MM04T	MEGF11	multiple EGF-like-domains 11	CCDS10213.2	chr15_66210355-66210355_C_T	679D>N	Substitution	Nonsynonymous coding	33%
MM04T	MEGF11	multiple EGF-like-domains 11	CCDS10213.2	chr15_66191072-66191072_C_A	990E>X	Substitution	Nonsense	61%

MM04T	MEGF8	multiple EGF-like-domains 8	ENST00000251268	chr19_42852556-42852556_G_A	744V>M	Substitution	Nonsynonymous coding	43%
MM04T	MEIS1	Meis homeobox 1	CCDS46309.1	chr2_66667693-66667693_G_T	NA	Substitution	Splice site acceptor	24%
MM04T	MEP1A	meprin A, alpha (PABA peptide hydrolase)	CCDS4918.1	chr6_46787435-46787435_C_A	184L>I	Substitution	Nonsynonymous coding	48%
MM04T	MEP1B	meprin A, beta	CCDS45846.1	chr18_29784236-29784236_T_C	154F>L	Substitution	Nonsynonymous coding	31%
MM04T	MEP1B	meprin A, beta	CCDS45846.1	chr18_29787247-29787247_G_A	194D>N	Substitution	Nonsynonymous coding	38%
MM04T	MERTK	c-mer proto-oncogene tyrosine kinase	CCDS2094.1	chr2_112760755-112760755_C_A	593L>M	Substitution	Nonsynonymous coding	24%
MM04T	METTL10	methyltransferase like 10	CCDS31307.1	chr10_126478849-126478849_C_A	57E>X	Substitution	Nonsense	18%
MM04T	METTL17	methyltransferase like 17	CCDS41913.1	chr14_21464712-21464712_C_A	369F>L	Substitution	Nonsynonymous coding	39%
MM04T	METTL4	methyltransferase like 4	CCDS11826.1	chr18_2566965-2566965_C_T	84R>Q	Substitution	Nonsynonymous coding	34%
MM04T	MFSD11	major facilitator superfamily domain containing 11	CCDS11750.1	chr17_74738319-74738319_G_T	134R>I	Substitution	Nonsynonymous coding	21%
MM04T	MFSD6	major facilitator superfamily domain containing 6	CCDS2306.1	chr2_191301650-191301650_G_T	299E>X	Substitution	Nonsense	39%
MM04T	MGA	MGA, MAX dimerization protein	NM_001164273	chr15_42050033-42050033_G_A	2396R>Q	Substitution	Nonsynonymous coding	32%
MM04T	MGA	MGA, MAX dimerization protein	NM_001164273	chr15_42052602-42052602_C_T	2425R>C	Substitution	Nonsynonymous coding	33%
MM04T	MGA	MGA, MAX dimerization protein	NM_001164273	chr15_42005688-42005688_G_T	1142E>X	Substitution	Nonsense	11%
MM04T	MGAM	maltase-glucoamylase (alpha-glucosidase)	CCDS47727.1	chr7_141747643-141747643_G_T	853E>X	Substitution	Nonsense	15%
MM04T	MGAT1	mannosyl (alpha-1,3-)-glycoprotein beta-1,2-N-acetylglucosaminyltransferase	CCDS4458.1	chr5_180219314-180219314_C_T	220E>K	Substitution	Nonsynonymous coding	23%
MM04T	MGAT4A	mannosyl (alpha-1,3-)-glycoprotein beta-1,4-N-acetylglucosaminyltransferase, isozyme A	CCDS2036.1	chr2_99242211-99242211_G_T	519S>Y	Substitution	Nonsynonymous coding	15%
MM04T	MGAT4C	mannosyl (alpha-1,3-)-glycoprotein beta-1,4-N-acetylglucosaminyltransferase, isozyme C	CCDS9030.1	chr12_86373822-86373822_G_A	228R>X	Substitution	Nonsense	31%
MM04T	MGEA5	meningioma expressed antigen 5 (hyaluronidase)	CCDS7520.1	chr10_103563740-103563740_G_T	263S>Y	Substitution	Nonsynonymous coding	22%
MM04T	MGME1	mitochondrial genome maintenance exonuclease 1	CCDS13131.1	chr20_17950518-17950518_T_C	6F>L	Substitution	Nonsynonymous coding	33%
MM04T	MI0003758	-	hsa-mir-1264	chrX_113887182-113887182_T_G	18L>W	Substitution	Nonsynonymous coding	33%
MM04T	MIA2	melanoma inhibitory activity 2	CCDS9672.1	chr14_39716960-39716960_A_C	394K>N	Substitution	Nonsynonymous coding	32%
MM04T	MIA2	melanoma inhibitory activity 2	CCDS9672.1	chr14_39717203-39717203_G_T	475K>N	Substitution	Nonsynonymous coding	25%
MM04T	MIA3	melanoma inhibitory activity family, member 3	CCDS41470.1	chr1_222803042-222803042_G_A	827R>Q	Substitution	Nonsynonymous coding	30%
MM04T	MICAL1	microtubule associated monoxygenase, calponin and LIM domain containing 1	CCDS5076.1	chr6_109769493-109769493_C_T	590A>T	Substitution	Nonsynonymous coding	35%
MM04T	MICAL3	microtubule associated monoxygenase, calponin and LIM domain containing 3	CCDS46659.1	chr22_18379714-18379714_C_T	328E>K	Substitution	Nonsynonymous coding	40%
MM04T	MICALCL	MICAL C-terminal like	CCDS41620.1	chr11_12316174-12316174_C_A	399S>Y	Substitution	Nonsynonymous coding	23%
MM04T	MICALCL	MICAL C-terminal like	CCDS41620.1	chr11_12315873-12315873_G_T	299E>X	Substitution	Nonsense	33%
MM04T	MID1	midline 1 (Opitz/BBB syndrome)	CCDS14138.1	chrX_10535512-10535512_C_T	26A>T	Substitution	Nonsynonymous coding	29%
MM04T	MID1IP1	MID1 interacting protein 1	CCDS14249.1	chrX_38664503-38664503_G_A	102E>K	Substitution	Nonsynonymous coding	23%
MM04T	MIER1	mesoderm induction early response 1 homolog (Xenopus laevis)	CCDS41348.1	chr1_67423799-67423799_C_T	80R>X	Substitution	Nonsense	21%
MM04T	MINK1	misshapen-like kinase 1	CCDS45588.1	chr17_4789457-4789457_C_A	246P>H	Substitution	Nonsynonymous coding	36%

MM04T	MINK1	misshapen-like kinase 1	CCDS45588.1	chr17_4799301-4799301_A_C	1123K>T	Substitution	Nonsynonymous coding	31%
MM04T	MINK1	misshapen-like kinase 1	CCDS45588.1	chr17_4788301-4788301_C_T	NA	Substitution	Splice site donor	16%
MM04T	MIR10B	microRNA 10b	hsa-mir-10b	chr2_177015125-177015125_C_T	32S>L	Substitution	Nonsynonymous coding	36%
MM04T	MIR133B	microRNA 133b	hsa-mir-133b	chr6_52013755-52013755_C_T	12S>L	Substitution	Nonsynonymous coding	21%
MM04T	MIR146B	microRNA 146b	hsa-mir-146b	chr10_104196331-104196331_C_A	21F>L	Substitution	Nonsynonymous coding	32%
MM04T	MIR409	microRNA 409	hsa-mir-409	chr14_101531680-101531680_G_A	15R>Q	Substitution	Nonsynonymous coding	14%
MM04T	MIR448	microRNA 448	hsa-mir-448	chrX_114058123-114058123_C_T	36S>L	Substitution	Nonsynonymous coding	34%
MM04T	MIR499A	microRNA 499a	hsa-mir-499	chr20_33578215-33578215_G_A	13D>N	Substitution	Nonsynonymous coding	26%
MM04T	MIR595	microRNA 595	hsa-mir-595	chr7_158325421-158325421_C_A	29G>X	Substitution	Nonsense	31%
MM04T	MIR657	microRNA 657	hsa-mir-657	chr17_79099165-79099165_C_A	3*>Y	Substitution	Nonsynonymous coding	19%
MM04T	MIS18BP1	MIS18 binding protein 1	CCDS9684.1	chr14_45687589-45687589_C_T	913R>Q	Substitution	Nonsynonymous coding	24%
MM04T	MIS18BP1	MIS18 binding protein 1	CCDS9684.1	chr14_45700468-45700468_T_G	490Q>H	Substitution	Nonsynonymous coding	19%
MM04T	MKI67	antigen identified by monoclonal antibody Ki-67	CCDS7659.1	chr10_129900863-129900863_C_A	3081D>Y	Substitution	Nonsynonymous coding	24%
MM04T	MKI67	antigen identified by monoclonal antibody Ki-67	CCDS7659.1	chr10_129905984-129905984_C_T	1374E>K	Substitution	Nonsynonymous coding	28%
MM04T	MKI67	antigen identified by monoclonal antibody Ki-67	CCDS7659.1	chr10_129910636-129910636_A_G	577V>A	Substitution	Nonsynonymous coding	32%
MM04T	MKI67	antigen identified by monoclonal antibody Ki-67	CCDS7659.1	chr10_129913277-129913277_C_A	465E>D	Substitution	Nonsynonymous coding	23%
MM04T	MKI67	antigen identified by monoclonal antibody Ki-67	CCDS7659.1	chr10_129907286-129907286_C_A	940E>X	Substitution	Nonsense	23%
MM04T	MKLN1	muskelin 1, intracellular mediator containing kelch motifs	CCDS34754.1	chr7_131122670-131122670_C_T	363R>C	Substitution	Nonsynonymous coding	30%
MM04T	MKRN3	makorin ring finger protein 3	CCDS10013.1	chr15_23812057-23812057_C_A	376F>L	Substitution	Nonsynonymous coding	22%
MM04T	MKRN3	makorin ring finger protein 3	CCDS10013.1	chr15_23812278-23812278_G_A	450R>Q	Substitution	Nonsynonymous coding	29%
MM04T	MLF1	myeloid leukemia factor 1	CCDS3182.1	chr3_158314716-158314716_G_T	87Q>H	Substitution	Nonsynonymous coding	21%
MM04T	MLH3	mutL homolog 3 (E. coli)	CCDS32123.1	chr14_75513186-75513186_A_G	1058V>A	Substitution	Nonsynonymous coding	28%
MM04T	MLKL	mixed lineage kinase domain-like	CCDS32487.1	chr16_74706405-74706405_C_A	471K>N	Substitution	Nonsynonymous coding	38%
MM04T	MLL	myeloid/lymphoid or mixed-lineage leukemia (trithorax homolog, Drosophila)	ENST00000328469	chr11_118309767-118309767_G_T	154R>I	Substitution	Nonsynonymous coding	24%
MM04T	MLL3	myeloid/lymphoid or mixed-lineage leukemia 3	CCDS5931.1	chr7_151846237-151846237_C_T	4259E>K	Substitution	Nonsynonymous coding	33%
MM04T	MLL3	myeloid/lymphoid or mixed-lineage leukemia 3	ENST00000355193	chr7_151854911-151854911_G_T	3926L>I	Substitution	Nonsynonymous coding	40%
MM04T	MLXIP	MLX interacting protein	NM_014938	chr12_122517176-122517176_G_T	NA	Substitution	Splice site donor	25%
MM04T	MLXIP	MLX interacting protein	ENST00000377037	chr12_122618672-122618672_G_T	214E>X	Substitution	Nonsense	22%
MM04T	MLXIPL	MLX interacting protein-like	CCDS5553.1	chr7_73021731-73021731_T_G	168N>T	Substitution	Nonsynonymous coding	37%
MM04T	MMEL1	membrane metallo-endopeptidase-like 1	CCDS30569.1	chr1_2537716-2537716_C_A	232D>Y	Substitution	Nonsynonymous coding	23%
MM04T	MMP1	matrix metallopeptidase 1 (interstitial collagenase)	CCDS8322.1	chr11_102667505-102667505_G_T	172S>Y	Substitution	Nonsynonymous coding	25%
MM04T	MMP20	matrix metallopeptidase 20	CCDS8318.1	chr11_102449795-102449795_T_G	442Q>H	Substitution	Nonsynonymous coding	35%

MM04T	MMP24	matrix metallopeptidase 24 (membrane-inserted)	CCDS46593.1	chr20_33842305-33842305_C_T	189R>C	Substitution	Nonsynonymous coding	32%
MM04T	MMP24	matrix metallopeptidase 24 (membrane-inserted)	CCDS46593.1	chr20_33851731-33851731_C_A	319L>I	Substitution	Nonsynonymous coding	16%
MM04T	MMP27	matrix metallopeptidase 27	CCDS8319.1	chr11_102564733-102564733_G_A	366T>I	Substitution	Nonsynonymous coding	36%
MM04T	MMP7	matrix metallopeptidase 7 (matrilysin, uterine)	CCDS8317.1	chr11_102398389-102398389_G_T	117T>N	Substitution	Nonsynonymous coding	23%
MM04T	MMP9	matrix metallopeptidase 9 (gelatinase B, 92kDa gelatinase, 92kDa type IV collagenase)	CCDS13390.1	chr20_44637665-44637665_G_T	34D>Y	Substitution	Nonsynonymous coding	12%
MM04T	MMRN2	multimerin 2	CCDS7379.1	chr10_88696562-88696562_A_G	930S>P	Substitution	Nonsynonymous coding	24%
MM04T	MNAT1	menage a trois homolog 1, cyclin H assembly factor (Xenopus laevis)	CCDS9750.1	chr14_61264824-61264824_G_T	88D>Y	Substitution	Nonsynonymous coding	26%
MM04T	MNDA	myeloid cell nuclear differentiation antigen	CCDS1177.1	chr1_158811968-158811968_C_A	9L>I	Substitution	Nonsynonymous coding	24%
MM04T	MNDA	myeloid cell nuclear differentiation antigen	CCDS1177.1	chr1_158817632-158817632_C_T	368R>X	Substitution	Nonsense	32%
MM04T	MOB3C	MOB kinase activator 3C	CCDS539.1	chr1_47075706-47075706_T_C	249S>G	Substitution	Nonsynonymous coding	24%
MM04T	MOGAT1	monoacylglycerol O-acyltransferase 1	CCDS46524.1	chr2_223559222-223559222_G_A	207R>Q	Substitution	Nonsynonymous coding	26%
MM04T	MOGS	mannosyl-oligosaccharide glucosidase	CCDS42700.1	chr2_74689935-74689935_T_G	327K>N	Substitution	Nonsynonymous coding	23%
MM04T	MON2	MON2 homolog (S. cerevisiae)	CCDS31849.1	chr12_62943462-62943462_G_A	923R>Q	Substitution	Nonsynonymous coding	24%
MM04T	MORC1	MORC family CW-type zinc finger 1	CCDS2955.1	chr3_108682389-108682389_C_A	891D>Y	Substitution	Nonsynonymous coding	23%
MM04T	MORC1	MORC family CW-type zinc finger 1	CCDS2955.1	chr3_108780857-108780857_C_A	315R>I	Substitution	Nonsynonymous coding	17%
MM04T	MORC1	MORC family CW-type zinc finger 1	CCDS2955.1	chr3_108819264-108819264_C_A	105S>I	Substitution	Nonsynonymous coding	15%
MM04T	MORC4	MORC family CW-type zinc finger 4	CCDS14525.2	chrX_106184843-106184843_G_A	894R>W	Substitution	Nonsynonymous coding	25%
MM04T	MORC4	MORC family CW-type zinc finger 4	CCDS14525.2	chrX_106186115-106186115_A_C	669L>W	Substitution	Nonsynonymous coding	41%
MM04T	MORC4	MORC family CW-type zinc finger 4	CCDS14525.2	chrX_106224218-106224218_C_T	280R>H	Substitution	Nonsynonymous coding	27%
MM04T	MORN1	MORN repeat containing 1	CCDS40.1	chr1_2268221-2268221_C_A	369D>Y	Substitution	Nonsynonymous coding	24%
MM04T	MORN1	MORN repeat containing 1	CCDS40.1	chr1_2321404-2321404_G_T	36F>L	Substitution	Nonsynonymous coding	16%
MM04T	MPDZ	multiple PDZ domain protein	CCDS47951.1	chr9_13205053-13205053_A_C	510L>V	Substitution	Nonsynonymous coding	18%
MM04T	MPDZ	multiple PDZ domain protein	CCDS47951.1	chr9_13216779-13216779_A_C	428I>M	Substitution	Nonsynonymous coding	26%
MM04T	MPDZ	multiple PDZ domain protein	CCDS47951.1	chr9_13221472-13221472_C_T	259E>K	Substitution	Nonsynonymous coding	29%
MM04T	MPDZ	multiple PDZ domain protein	CCDS47951.1	chr9_13247653-13247653_A_G	55V>A	Substitution	Nonsynonymous coding	30%
MM04T	MPDZ	multiple PDZ domain protein	CCDS47951.1	chr9_13175799-13175799_C_A	1003E>X	Substitution	Nonsense	43%
MM04T	MPEG1	macrophage expressed 1	CCDS41650.1	chr11_58978816-58978816_T_G	508E>A	Substitution	Nonsynonymous coding	30%
MM04T	MPEG1	macrophage expressed 1	CCDS41650.1	chr11_58979143-58979143_T_G	399K>T	Substitution	Nonsynonymous coding	28%
MM04T	MPEG1	macrophage expressed 1	CCDS41650.1	chr11_58980056-58980056_C_A	95E>X	Substitution	Nonsense	30%
MM04T	MPHOSPH10	M-phase phosphoprotein 10 (U3 small nucleolar ribonucleoprotein)	CCDS1916.1	chr2_71371596-71371596_A_C	495E>D	Substitution	Nonsynonymous coding	25%
MM04T	MPHOSPH10	M-phase phosphoprotein 10 (U3 small nucleolar ribonucleoprotein)	CCDS1916.1	chr2_71376534-71376534_C_T	616S>L	Substitution	Nonsynonymous coding	22%
MM04T	MPP4	membrane protein, palmitoylated 4 (MAGUK p55 subfamily member 4)	CCDS46491.1	chr2_202521082-202521082_C_T	380R>H	Substitution	Nonsynonymous coding	24%

MM04T	MPP5	membrane protein, palmitoylated 5 (MAGUK p55 subfamily member 5)	CCDS9779.1	chr14_67790494-67790494_C_T	606R>W	Substitution	Nonsynonymous coding	36%
MM04T	MPPED1	metallophosphoesterase domain containing 1	CCDS46723.1	chr22_43870746-43870746_G_T	179E>D	Substitution	Nonsynonymous coding	26%
MM04T	MPRIP	myosin phosphatase Rho interacting protein	ENST00000395806	chr17_16961002-16961002_G_A	59R>Q	Substitution	Nonsynonymous coding	28%
MM04T	MRAP2	melanocortin 2 receptor accessory protein 2	CCDS5001.1	chr6_84798828-84798828_G_T	82E>D	Substitution	Nonsynonymous coding	31%
MM04T	MRC2	mannose receptor, C type 2	CCDS11634.1	chr17_60753790-60753790_G_A	578E>K	Substitution	Nonsynonymous coding	11%
MM04T	MRC2	mannose receptor, C type 2	CCDS11634.1	chr17_60754694-60754694_G_T	633K>N	Substitution	Nonsynonymous coding	13%
MM04T	MRC2	mannose receptor, C type 2	CCDS11634.1	chr17_60768086-60768086_C_T	1326R>W	Substitution	Nonsynonymous coding	25%
MM04T	MRE11A	MRE11 meiotic recombination 11 homolog A (S. cerevisiae)	CCDS8299.1	chr11_94192676-94192676_C_A	466E>D	Substitution	Nonsynonymous coding	29%
MM04T	MRFAP1	Morf4 family associated protein 1	CCDS3389.1	chr4_6642794-6642794_A_G	69T>A	Substitution	Nonsynonymous coding	29%
MM04T	MRGPRD	MAS-related GPR, member D	CCDS31625.1	chr11_68747623-68747623_A_G	278L>P	Substitution	Nonsynonymous coding	28%
MM04T	MRGPRD	MAS-related GPR, member D	CCDS31625.1	chr11_68747955-68747955_G_T	167F>L	Substitution	Nonsynonymous coding	33%
MM04T	MRGPRD	MAS-related GPR, member D	CCDS31625.1	chr11_68748293-68748293_G_A	55R>X	Substitution	Nonsense	31%
MM04T	MRO	maestro	CCDS45868.1	chr18_48333129-48333129_T_G	78K>T	Substitution	Nonsynonymous coding	33%
MM04T	MRPL39	mitochondrial ribosomal protein L39	CCDS33522.1	chr21_26972176-26972176_T_G	175I>L	Substitution	Nonsynonymous coding	27%
MM04T	MRPS18A	mitochondrial ribosomal protein S18A	CCDS4906.1	chr6_43643331-43643331_G_T	92F>L	Substitution	Nonsynonymous coding	23%
MM04T	MRPS35	mitochondrial ribosomal protein S35	CCDS8714.1	chr12_27908243-27908243_A_G	278I>V	Substitution	Nonsynonymous coding	32%
MM04T	MRPS5	mitochondrial ribosomal protein S5	CCDS2010.1	chr2_95756241-95756241_T_C	320I>V	Substitution	Nonsynonymous coding	29%
MM04T	MRS2	MRS2 magnesium homeostasis factor homolog (S. cerevisiae)	CCDS4552.1	chr6_24423172-24423172_G_T	372R>I	Substitution	Nonsynonymous coding	29%
MM04T	MRVI1	murine retrovirus integration site 1 homolog	CCDS44538.1	chr11_10615134-10615134_G_A	685R>W	Substitution	Nonsynonymous coding	24%
MM04T	MS4A13	membrane-spanning 4-domains, subfamily A, member 13	CCDS31571.1	chr11_60291422-60291422_C_A	61S>Y	Substitution	Nonsynonymous coding	38%
MM04T	MS4A14	membrane-spanning 4-domains, subfamily A, member 14	CCDS31569.1	chr11_60167883-60167883_C_A	92L>I	Substitution	Nonsynonymous coding	21%
MM04T	MS4A14	membrane-spanning 4-domains, subfamily A, member 14	CCDS31569.1	chr11_60184332-60184332_G_T	631A>S	Substitution	Nonsynonymous coding	30%
MM04T	MS4A14	membrane-spanning 4-domains, subfamily A, member 14	CCDS31569.1	chr11_60183849-60183849_G_T	470E>X	Substitution	Nonsense	31%
MM04T	MS4A15	membrane-spanning 4-domains, subfamily A, member 15	CCDS44617.1	chr11_60531343-60531343_C_A	46P>Q	Substitution	Nonsynonymous coding	24%
MM04T	MSH2	mutS homolog 2, colon cancer, nonpolyposis type 1 (E. coli)	CCDS1834.1	chr2_47690242-47690242_G_T	487D>Y	Substitution	Nonsynonymous coding	22%
MM04T	MSH4	mutS homolog 4 (E. coli)	CCDS670.1	chr1_76344775-76344775_G_T	547A>S	Substitution	Nonsynonymous coding	18%
MM04T	MSH5	mutS homolog 5 (E. coli)	CCDS34409.1	chr6_31729649-31729649_G_A	763E>K	Substitution	Nonsynonymous coding	19%
MM04T	MSH6	mutS homolog 6 (E. coli)	CCDS1836.1	chr2_48027494-48027494_G_A	791R>H	Substitution	Nonsynonymous coding	25%
MM04T	MSH6	mutS homolog 6 (E. coli)	CCDS1836.1	chr2_48027887-48027887_G_A	922R>Q	Substitution	Nonsynonymous coding	30%
MM04T	MSH6	mutS homolog 6 (E. coli)	CCDS1836.1	chr2_48027844-48027844_G_T	908E>X	Substitution	Nonsense	26%
MM04T	MSL2	male-specific lethal 2 homolog (Drosophila)	CCDS33861.1	chr3_135870293-135870293_C_T	477R>Q	Substitution	Nonsynonymous coding	36%
MM04T	MSL2	male-specific lethal 2 homolog (Drosophila)	CCDS33861.1	chr3_135870326-135870326_C_T	466R>H	Substitution	Nonsynonymous coding	36%

MM04T	MSLN	mesothelin	CCDS32356.1	chr16_814646-814646_G_A	NA	Substitution	Splice site acceptor	17%
MM04T	MSLNL	mesothelin-like	CCDS42093.1	chr16_830088-830088_G_T	305L>I	Substitution	Nonsynonymous coding	33%
MM04T	MSRB1	methionine sulfoxide reductase B1	ENST00000399753	chr16_1991235-1991235_C_T	76G>E	Substitution	Nonsynonymous coding	12%
MM04T	MSS51	MSS51 mitochondrial translational activator	CCDS31221.1	chr10_75188010-75188010_C_A	11K>N	Substitution	Nonsynonymous coding	42%
MM04T	MSX1	msh homeobox 1	CCDS3378.2	chr4_4864605-4864605_T_C	216V>A	Substitution	Nonsynonymous coding	32%
MM04T	MT4	metallothionein 4	CCDS42165.1	chr16_56601719-56601719_T_C	30Y>H	Substitution	Nonsynonymous coding	19%
MM04T	MTA2	metastasis associated 1 family, member 2	CCDS8022.1	chr11_62363550-62363550_G_T	349S>Y	Substitution	Nonsynonymous coding	24%
MM04T	MTA3	metastasis associated 1 family, member 3	ENST00000405094	chr2_42950146-42950146_G_T	577R>I	Substitution	Nonsynonymous coding	28%
MM04T	MTDH	metadherin	CCDS6274.1	chr8_98699667-98699667_A_C	193E>D	Substitution	Nonsynonymous coding	28%
MM04T	MTERF	mitochondrial transcription termination factor	CCDS5621.1	chr7_91503069-91503069_C_T	347E>K	Substitution	Nonsynonymous coding	44%
MM04T	MTERF	mitochondrial transcription termination factor	CCDS5621.1	chr7_91503498-91503498_A_C	204F>V	Substitution	Nonsynonymous coding	35%
MM04T	MTFR1L	mitochondrial fission regulator 1-like	CCDS41284.1	chr1_26153194-26153194_T_C	110S>P	Substitution	Nonsynonymous coding	25%
MM04T	MTHFD1	methylenetetrahydrofolate dehydrogenase (NADP+ dependent) 1,	CCDS9763.1	chr14_64884651-64884651_A_C	175K>T	Substitution	Nonsynonymous coding	34%
MM04T	MTHFD1	methylenetetrahydrofolate dehydrogenase (NADP+ dependent) 1,	CCDS9763.1	chr14_64916319-64916319_C_A	812F>L	Substitution	Nonsynonymous coding	35%
MM04T	MTHFD1	methylenetetrahydrofolate dehydrogenase (NADP+ dependent) 1,	CCDS9763.1	chr14_64894077-64894077_G_T	384E>X	Substitution	Nonsense	35%
MM04T	MTHFD1L	methylenetetrahydrofolate dehydrogenase (NADP+ dependent) 1-like	CCDS5228.1	chr6_151336798-151336798_G_T	852R>I	Substitution	Nonsynonymous coding	34%
MM04T	MTIF2	mitochondrial translational initiation factor 2	CCDS1853.1	chr2_55464464-55464464_T_G	657K>N	Substitution	Nonsynonymous coding	44%
MM04T	MTIF2	mitochondrial translational initiation factor 2	CCDS1853.1	chr2_55476590-55476590_C_T	308D>N	Substitution	Nonsynonymous coding	25%
MM04T	MTIF2	mitochondrial translational initiation factor 2	CCDS1853.1	chr2_55476628-55476628_G_A	295A>V	Substitution	Nonsynonymous coding	29%
MM04T	MTIF2	mitochondrial translational initiation factor 2	CCDS1853.1	chr2_55490957-55490957_C_T	13R>Q	Substitution	Nonsynonymous coding	13%
MM04T	MTIF3	mitochondrial translational initiation factor 3	CCDS9322.1	chr13_28014341-28014341_A_C	82I>S	Substitution	Nonsynonymous coding	17%
MM04T	MTM1	myotubularin 1	CCDS14694.1	chrX_149818280-149818280_C_A	320S>Y	Substitution	Nonsynonymous coding	16%
MM04T	MTM1	myotubularin 1	CCDS14694.1	chrX_149831960-149831960_A_T	508K>X	Substitution	Nonsense	20%
MM04T	MTMR10	myotubularin related protein 10	CCDS45204.1	chr15_31282989-31282989_G_T	38L>I	Substitution	Nonsynonymous coding	33%
MM04T	MTMR3	myotubularin related protein 3	CCDS13870.1	chr22_30403294-30403294_C_A	288S>Y	Substitution	Nonsynonymous coding	23%
MM04T	MTMR3	myotubularin related protein 3	CCDS13870.1	chr22_30415971-30415971_C_A	775L>I	Substitution	Nonsynonymous coding	35%
MM04T	MTMR6	myotubularin related protein 6	CCDS9313.1	chr13_25828535-25828535_T_G	378K>T	Substitution	Nonsynonymous coding	36%
MM04T	MTMR7	myotubularin related protein 7	CCDS34851.1	chr8_17166826-17166826_C_A	371E>X	Substitution	Nonsense	28%
MM04T	MTMR8	myotubularin related protein 8	CCDS14379.1	chrX_63551450-63551450_G_A	447R>W	Substitution	Nonsynonymous coding	31%
MM04T	MTMR8	myotubularin related protein 8	CCDS14379.1	chrX_63557180-63557180_C_A	357D>Y	Substitution	Nonsynonymous coding	21%
MM04T	MTMR8	myotubularin related protein 8	CCDS14379.1	chrX_63488418-63488418_C_A	705*>L	Substitution	Nonsynonymous coding	14%
MM04T	MTOR	mechanistic target of rapamycin (serine/threonine kinase)	CCDS127.1	chr1_11190630-11190630_C_T	1857E>K	Substitution	Nonsynonymous coding	23%

MM04T	MTOR	mechanistic target of rapamycin (serine/threonine kinase)	CCDS127.1	chr1_11273473-11273473_G_A	1090R>C	Substitution	Nonsynonymous coding	23%
MM04T	MTR	5-methyltetrahydrofolate-homocysteine methyltransferase	CCDS1614.1	chr1_236972052-236972052_G_A	130E>K	Substitution	Nonsynonymous coding	33%
MM04T	MTR	5-methyltetrahydrofolate-homocysteine methyltransferase	CCDS1614.1	chr1_237044084-237044084_G_T	875R>I	Substitution	Nonsynonymous coding	22%
MM04T	MTUS1	microtubule associated tumor suppressor 1	CCDS43717.1	chr8_17581243-17581243_C_A	796R>M	Substitution	Nonsynonymous coding	29%
MM04T	MUC16	mucin 16, cell surface associated	NM_024690	chr19_9047532-9047532_T_G	11367N>H	Substitution	Nonsynonymous coding	36%
MM04T	MUC16	mucin 16, cell surface associated	NM_024690	chr19_9060072-9060072_C_A	9125R>I	Substitution	Nonsynonymous coding	18%
MM04T	MUC16	mucin 16, cell surface associated	NM_024690	chr19_9060192-9060192_G_T	9085S>Y	Substitution	Nonsynonymous coding	33%
MM04T	MUC16	mucin 16, cell surface associated	NM_024690	chr19_9069388-9069388_T_A	6020N>Y	Substitution	Nonsynonymous coding	21%
MM04T	MUC16	mucin 16, cell surface associated	NM_024690	chr19_9083766-9083766_C_A	2683E>D	Substitution	Nonsynonymous coding	23%
MM04T	MUC16	mucin 16, cell surface associated	NM_024690	chr19_9090941-9090941_C_T	292D>N	Substitution	Nonsynonymous coding	37%
MM04T	MUC16	mucin 16, cell surface associated	NM_024690	chr19_9049935-9049935_C_A	10566E>X	Substitution	Nonsense	22%
MM04T	MUC17	mucin 17, cell surface associated	CCDS34711.1	chr7_100675256-100675256_C_T	187P>S	Substitution	Nonsynonymous coding	27%
MM04T	MUC17	mucin 17, cell surface associated	CCDS34711.1	chr7_100675296-100675296_C_A	200T>N	Substitution	Nonsynonymous coding	30%
MM04T	MUC2	mucin 2, oligomeric mucus/gel-forming	NM_002457	chr11_1095207-1095207_C_A	2005F>L	Substitution	Nonsynonymous coding	23%
MM04T	MUC2	mucin 2, oligomeric mucus/gel-forming	NM_002457	chr11_1099512-1099512_G_T	2447K>N	Substitution	Nonsynonymous coding	15%
MM04T	MUC5B	mucin 5B, oligomeric mucus/gel-forming	CCDS44515.1	chr11_1262666-1262666_A_C	1522K>T	Substitution	Nonsynonymous coding	23%
MM04T	MUL1	mitochondrial E3 ubiquitin protein ligase 1	CCDS208.1	chr1_20827284-20827284_C_T	320V>I	Substitution	Nonsynonymous coding	22%
MM04T	MUL1	mitochondrial E3 ubiquitin protein ligase 1	CCDS208.1	chr1_20834462-20834462_G_T	19S>Y	Substitution	Nonsynonymous coding	26%
MM04T	MUM1L1	melanoma associated antigen (mutated) 1-like 1	ENST00000337685	chrX_105450752-105450752_T_G	443L>V	Substitution	Nonsynonymous coding	47%
MM04T	MUT	methylmalonyl CoA mutase	CCDS4924.1	chr6_49425654-49425654_T_C	168E>G	Substitution	Nonsynonymous coding	26%
MM04T	MUT	methylmalonyl CoA mutase	CCDS4924.1	chr6_49425418-49425418_C_A	247E>X	Substitution	Nonsense	44%
MM04T	MYBPC1	myosin binding protein C, slow type	CCDS9083.1	chr12_102040632-102040632_G_T	353D>Y	Substitution	Nonsynonymous coding	25%
MM04T	MYBPC1	myosin binding protein C, slow type	CCDS9083.1	chr12_102061599-102061599_C_T	816R>C	Substitution	Nonsynonymous coding	30%
MM04T	MYBPC1	myosin binding protein C, slow type	CCDS9083.1	chr12_102071026-102071026_T_C	988V>A	Substitution	Nonsynonymous coding	37%
MM04T	MYBPC1	myosin binding protein C, slow type	CCDS9083.1	chr12_102028331-102028331_G_A	NA	Substitution	Splice site acceptor	20%
MM04T	MYBPC2	myosin binding protein C, fast type	CCDS46152.1	chr19_50946847-50946847_G_T	333E>D	Substitution	Nonsynonymous coding	26%
MM04T	MYCBP2	MYC binding protein 2, E3 ubiquitin protein ligase	CCDS31994.1	chr13_77672270-77672270_G_T	2969L>I	Substitution	Nonsynonymous coding	24%
MM04T	MYCBP2	MYC binding protein 2, E3 ubiquitin protein ligase	CCDS31994.1	chr13_77779656-77779656_T_G	1185M>L	Substitution	Nonsynonymous coding	33%
MM04T	MYH14	myosin, heavy chain 14, non-muscle	CCDS46151.1	chr19_50766627-50766627_G_A	849A>T	Substitution	Nonsynonymous coding	22%
MM04T	MYH15	myosin, heavy chain 15	CCDS43127.1	chr3_108127129-108127129_C_T	1560E>K	Substitution	Nonsynonymous coding	18%
MM04T	MYH15	myosin, heavy chain 15	CCDS43127.1	chr3_108163501-108163501_G_T	901L>I	Substitution	Nonsynonymous coding	31%
MM04T	MYH15	myosin, heavy chain 15	CCDS43127.1	chr3_108188894-108188894_C_A	537E>X	Substitution	Nonsense	24%

MM04T	MYH2	myosin, heavy chain 2, skeletal muscle, adult	CCDS11156.1	chr17_10438680-10438680_C_A	659E>D	Substitution	Nonsynonymous coding	39%
MM04T	MYH6	myosin, heavy chain 6, cardiac muscle, alpha	CCDS9600.1	chr14_23866232-23866232_C_T	703G>D	Substitution	Nonsynonymous coding	25%
MM04T	MYH7	myosin, heavy chain 7, cardiac muscle, beta	CCDS9601.1	chr14_23885392-23885392_G_A	1592R>W	Substitution	Nonsynonymous coding	46%
MM04T	MYH7B	myosin, heavy chain 7B, cardiac muscle, beta	CCDS42869.1	chr20_33588569-33588569_A_G	NA	Substitution	Splice site acceptor	20%
MM04T	MYH9	myosin, heavy chain 9, non-muscle	CCDS13927.1	chr22_36689402-36689402_C_A	1356E>D	Substitution	Nonsynonymous coding	19%
MM04T	MYLK	myosin light chain kinase	CCDS46896.1	chr3_123457901-123457901_A_C	144F>C	Substitution	Nonsynonymous coding	20%
MM04T	MYLK3	myosin light chain kinase 3	CCDS10723.2	chr16_46781973-46781973_C_A	45D>Y	Substitution	Nonsynonymous coding	32%
MM04T	MYLK4	myosin light chain kinase family, member 4	CCDS34330.1	chr6_2671569-2671569_C_T	378R>H	Substitution	Nonsynonymous coding	35%
MM04T	MYO15A	myosin XVA	CCDS42271.1	chr17_18058038-18058038_G_T	2731E>D	Substitution	Nonsynonymous coding	21%
MM04T	MYO16	myosin XVI	CCDS32008.1	chr13_109379923-109379923_C_T	145L>F	Substitution	Nonsynonymous coding	41%
MM04T	MYO16	myosin XVI	CCDS32008.1	chr13_109792641-109792641_G_A	1339D>N	Substitution	Nonsynonymous coding	26%
MM04T	MYO16	myosin XVI	CCDS32008.1	chr13_109859049-109859049_C_A	1814D>E	Substitution	Nonsynonymous coding	32%
MM04T	MYO16	myosin XVI	CCDS32008.1	chr13_109817299-109817299_G_T	1717E>X	Substitution	Nonsense	16%
MM04T	MYO18A	myosin XVIIIA	CCDS45642.1	chr17_27443474-27443474_C_T	NA	Substitution	Splice site acceptor	28%
MM04T	MYO18B	myosin XVIIIB	NM_032608	chr22_26176035-26176035_G_A	694R>Q	Substitution	Nonsynonymous coding	39%
MM04T	MYO1A	myosin IA	CCDS8929.1	chr12_57430850-57430850_C_T	694R>H	Substitution	Nonsynonymous coding	29%
MM04T	MYO1A	myosin IA	CCDS8929.1	chr12_57435261-57435261_C_A	373K>N	Substitution	Nonsynonymous coding	11%
MM04T	MYO1H	myosin IH	NM_001101421	chr12_109834312-109834312_T_G	122F>L	Substitution	Nonsynonymous coding	39%
MM04T	MYO3A	myosin IIIA	CCDS7148.1	chr10_26357713-26357713_A_G	357Q>R	Substitution	Nonsynonymous coding	38%
MM04T	MYO3A	myosin IIIA	CCDS7148.1	chr10_26414440-26414440_G_A	673D>N	Substitution	Nonsynonymous coding	29%
MM04T	MYO3B	myosin IIIB	CCDS42773.1	chr2_171356234-171356234_G_A	1069A>T	Substitution	Nonsynonymous coding	40%
MM04T	MYO5B	myosin VB	CCDS42436.1	chr18_47488689-47488689_C_T	498D>N	Substitution	Nonsynonymous coding	32%
MM04T	MYO5B	myosin VB	CCDS42436.1	chr18_47500907-47500907_G_A	379R>C	Substitution	Nonsynonymous coding	29%
MM04T	MYO7A	myosin VIIA	NM_000260	chr11_76924066-76924066_G_A	2142D>N	Substitution	Nonsynonymous coding	25%
MM04T	MYO7A	myosin VIIA	NM_000260	chr11_76903216-76903216_G_T	1349E>X	Substitution	Nonsense	19%
MM04T	MYO7B	myosin VIIB	CCDS46405.1	chr2_128346090-128346090_G_T	605G>V	Substitution	Nonsynonymous coding	22%
MM04T	MYO9A	myosin IXA	CCDS10239.1	chr15_72192073-72192073_A_C	1142I>S	Substitution	Nonsynonymous coding	33%
MM04T	MYO9A	myosin IXA	CCDS10239.1	chr15_72300243-72300243_T_G	435K>T	Substitution	Nonsynonymous coding	31%
MM04T	MYOC	myocilin, trabecular meshwork inducible glucocorticoid response	CCDS1297.1	chr1_171605340-171605340_C_T	414E>K	Substitution	Nonsynonymous coding	31%
MM04T	MYOC	myocilin, trabecular meshwork inducible glucocorticoid response	CCDS1297.1	chr1_171605841-171605841_C_A	247E>X	Substitution	Nonsense	23%
MM04T	MYOCD	myocardin	CCDS11163.1	chr17_12655788-12655788_A_G	395T>A	Substitution	Nonsynonymous coding	30%
MM04T	MYOCD	myocardin	CCDS11163.1	chr17_12666670-12666670_C_A	842F>L	Substitution	Nonsynonymous coding	22%

MM04T	MYOF	myoferlin	CCDS41551.1	chr10_95085712-95085712_T_G	1714K>N	Substitution	Nonsynonymous coding	32%
MM04T	MYOM1	myomesin 1	CCDS45824.1	chr18_3134759-3134759_G_A	758S>L	Substitution	Nonsynonymous coding	35%
MM04T	MYOM3	myomesin 3	CCDS41281.1	chr1_24400669-24400669_G_T	983S>R	Substitution	Nonsynonymous coding	21%
MM04T	MYOZ1	myozenin 1	CCDS7330.1	chr10_75397517-75397517_G_T	79F>L	Substitution	Nonsynonymous coding	25%
MM04T	MYOZ2	myozenin 2	CCDS3711.1	chr4_120072138-120072138_G_T	63R>I	Substitution	Nonsynonymous coding	30%
MM04T	MYRFL	myelin regulatory factor-like	ENST00000299350	chr12_70351699-70351699_G_T	215E>X	Substitution	Nonsense	36%
MM04T	MYSM1	Myb-like, SWIRM and MPN domains 1	CCDS41343.1	chr1_59125714-59125714_C_A	814E>D	Substitution	Nonsynonymous coding	31%
MM04T	MYSM1	Myb-like, SWIRM and MPN domains 1	CCDS41343.1	chr1_59154736-59154736_C_A	125E>X	Substitution	Nonsense	27%
MM04T	MYT1	myelin transcription factor 1	CCDS13558.1	chr20_62839732-62839732_G_A	395V>I	Substitution	Nonsynonymous coding	32%
MM04T	MYT1	myelin transcription factor 1	CCDS13558.1	chr20_62839824-62839824_G_T	425K>N	Substitution	Nonsynonymous coding	15%
MM04T	MYT1L	myelin transcription factor 1-like	CCDS46222.1	chr2_1893150-1893150_G_A	793P>S	Substitution	Nonsynonymous coding	21%
MM04T	N4BP1	NEDD4 binding protein 1	CCDS45479.1	chr16_48595270-48595270_C_A	428K>N	Substitution	Nonsynonymous coding	16%
MM04T	N4BP2	NEDD4 binding protein 2	CCDS3457.1	chr4_40098977-40098977_A_C	6K>T	Substitution	Nonsynonymous coding	35%
MM04T	N4BP2L1	NEDD4 binding protein 2-like 1	CCDS9345.2	chr13_32981411-32981411_C_A	124W>C	Substitution	Nonsynonymous coding	23%
MM04T	N4BP2L2	NEDD4 binding protein 2-like 2	CCDS45024.1	chr13_33096393-33096393_C_T	38D>N	Substitution	Nonsynonymous coding	29%
MM04T	N6AMT2	N-6 adenine-specific DNA methyltransferase 2 (putative)	CCDS9293.1	chr13_21306195-21306195_G_A	98S>L	Substitution	Nonsynonymous coding	28%
MM04T	NAA40	N(alpha)-acetyltransferase 40, NatD catalytic subunit, homolog (S. cerevisiae)	CCDS8053.1	chr11_63719915-63719915_G_T	96E>D	Substitution	Nonsynonymous coding	30%
MM04T	NAA40	N(alpha)-acetyltransferase 40, NatD catalytic subunit, homolog (S. cerevisiae)	CCDS8053.1	chr11_63721500-63721500_A_C	184Q>P	Substitution	Nonsynonymous coding	27%
MM04T	NAGK	N-acetylglucosamine kinase	CCDS33220.1	chr2_71302694-71302694_C_T	197R>W	Substitution	Nonsynonymous coding	35%
MM04T	NALCN	sodium leak channel, non-selective	CCDS9498.1	chr13_101753209-101753209_C_T	1030V>I	Substitution	Nonsynonymous coding	24%
MM04T	NAMPTL	nicotinamide phosphoribosyltransferase-like	ENST00000440465	chr10_36812223-36812223_G_A	314P>S	Substitution	Nonsynonymous coding	20%
MM04T	NAP1L2	nucleosome assembly protein 1-like 2	CCDS14423.1	chrX_72433253-72433253_C_T	359R>Q	Substitution	Nonsynonymous coding	23%
MM04T	NAP1L2	nucleosome assembly protein 1-like 2	CCDS14423.1	chrX_72433810-72433810_C_A	173E>D	Substitution	Nonsynonymous coding	26%
MM04T	NAPA	N-ethylmaleimide-sensitive factor attachment protein, alpha	CCDS12702.1	chr19_47991634-47991634_C_T	267D>N	Substitution	Nonsynonymous coding	27%
MM04T	NARFL	nuclear prelamin A recognition factor-like	CCDS10425.1	chr16_789681-789681_G_A	42R>C	Substitution	Nonsynonymous coding	24%
MM04T	NAT8	N-acetyltransferase 8 (GCN5-related, putative)	NM_016347	chr2_73927876-73927876_C_T	186G>D	Substitution	Nonsynonymous coding	32%
MM04T	NAT8	N-acetyltransferase 8 (GCN5-related, putative)	NM_016347	chr2_73928311-73928311_C_T	41R>Q	Substitution	Nonsynonymous coding	32%
MM04T	NAV1	neuron navigator 1	CCDS1414.1	chr1_201779200-201779200_C_T	1507R>X	Substitution	Nonsense	39%
MM04T	NAV2	neuron navigator 2	CCDS7851.2	chr11_19955292-19955292_G_T	501S>I	Substitution	Nonsynonymous coding	25%
MM04T	NAV3	neuron navigator 3	CCDS41815.1	chr12_78400684-78400684_C_A	456L>I	Substitution	Nonsynonymous coding	27%
MM04T	NBEA	neurobeachin	CCDS45026.1	chr13_35751142-35751142_G_A	1522E>K	Substitution	Nonsynonymous coding	39%
MM04T	NBEA	neurobeachin	CCDS45026.1	chr13_35770192-35770192_T_A	1707L>M	Substitution	Nonsynonymous coding	33%

MM04T	NBEA	neurobeachin	CCDS45026.1	chr13_36223874-36223874_C_T	2597S>F	Substitution	Nonsynonymous coding	24%
MM04T	NBEA	neurobeachin	CCDS45026.1	chr13_35751132-35751132_G_T	NA	Substitution	Splice site acceptor	42%
MM04T	NBEA	neurobeachin	CCDS45026.1	chr13_36026354-36026354_G_T	2144E>X	Substitution	Nonsense	34%
MM04T	NBEAL1	neurobeachin-like 1	CCDS46495.1	chr2_203922167-203922167_G_T	169R>I	Substitution	Nonsynonymous coding	30%
MM04T	NBEAL1	neurobeachin-like 1	CCDS46495.1	chr2_203972697-203972697_G_A	550V>M	Substitution	Nonsynonymous coding	32%
MM04T	NBEAL1	neurobeachin-like 1	CCDS46495.1	chr2_204045201-204045201_C_A	2158F>L	Substitution	Nonsynonymous coding	25%
MM04T	NBEAL1	neurobeachin-like 1	CCDS46495.1	chr2_204045150-204045150_G_A	2141W>X	Substitution	Nonsense	27%
MM04T	NBEAL2	neurobeachin-like 2	CCDS46817.1	chr3_47045750-47045750_C_A	2022P>H	Substitution	Nonsynonymous coding	20%
MM04T	NBEAL2	neurobeachin-like 2	CCDS46817.1	chr3_47046576-47046576_G_A	2137V>M	Substitution	Nonsynonymous coding	17%
MM04T	NBL1	neuroblastoma, suppression of tumorigenicity 1	CCDS41278.1	chr1_19981611-19981611_G_A	65D>N	Substitution	Nonsynonymous coding	33%
MM04T	NBL1	neuroblastoma, suppression of tumorigenicity 1	CCDS41278.1	chr1_19981611-19981611_G_T	65D>Y	Substitution	Nonsynonymous coding	16%
MM04T	NCAM1	neural cell adhesion molecule 1	ENST00000401611	chr11_113103048-113103048_A_G	338Y>C	Substitution	Nonsynonymous coding	34%
MM04T	NCAM1	neural cell adhesion molecule 1	ENST00000433634	chr11_113142516-113142516_C_T	247R>X	Substitution	Nonsense	24%
MM04T	NCAM2	neural cell adhesion molecule 2	ENST00000284894	chr21_22644156-22644156_C_A	16S>Y	Substitution	Nonsynonymous coding	36%
MM04T	NCAM2	neural cell adhesion molecule 2	CCDS42910.1	chr21_22782769-22782769_G_T	457K>N	Substitution	Nonsynonymous coding	43%
MM04T	NCAM2	neural cell adhesion molecule 2	CCDS42910.1	chr21_22849638-22849638_G_T	641E>D	Substitution	Nonsynonymous coding	20%
MM04T	NCAM2	neural cell adhesion molecule 2	CCDS42910.1	chr21_22849760-22849760_T_G	682F>C	Substitution	Nonsynonymous coding	41%
MM04T	NCAM2	neural cell adhesion molecule 2	CCDS42910.1	chr21_22707880-22707880_G_T	265E>X	Substitution	Nonsense	32%
MM04T	NCAN	neurocan	CCDS12397.1	chr19_19360589-19360589_C_T	1279R>W	Substitution	Nonsynonymous coding	18%
MM04T	NCAPD2	non-SMC condensin I complex, subunit D2	CCDS8548.1	chr12_6632090-6632090_C_A	684S>Y	Substitution	Nonsynonymous coding	27%
MM04T	NCAPH	non-SMC condensin I complex, subunit H	CCDS2021.1	chr2_97025928-97025928_G_T	469D>Y	Substitution	Nonsynonymous coding	42%
MM04T	NCDN	neurochondrin	CCDS392.1	chr1_36028038-36028038_C_T	397R>W	Substitution	Nonsynonymous coding	28%
MM04T	NCKAP5	NCK-associated protein 5	CCDS46418.1	chr2_133538711-133538711_G_T	1655L>I	Substitution	Nonsynonymous coding	21%
MM04T	NCOA1	nuclear receptor coactivator 1	CCDS1712.1	chr2_24929877-24929877_C_T	513S>L	Substitution	Nonsynonymous coding	33%
MM04T	NCOA2	nuclear receptor coactivator 2	CCDS47872.1	chr8_71044086-71044086_C_T	1104E>K	Substitution	Nonsynonymous coding	40%
MM04T	NCOA5	nuclear receptor coactivator 5	CCDS13392.1	chr20_44691286-44691286_A_C	465F>V	Substitution	Nonsynonymous coding	24%
MM04T	NCOA7	nuclear receptor coactivator 7	CCDS5132.1	chr6_126210307-126210307_G_T	369E>D	Substitution	Nonsynonymous coding	27%
MM04T	NCOR1	nuclear receptor corepressor 1	CCDS11175.1	chr17_15965510-15965510_T_C	1766T>A	Substitution	Nonsynonymous coding	26%
MM04T	NCOR1	nuclear receptor corepressor 1	CCDS11175.1	chr17_16052775-16052775_C_A	300R>I	Substitution	Nonsynonymous coding	16%
MM04T	NCOR1	nuclear receptor corepressor 1	CCDS11175.1	chr17_16056708-16056708_T_G	245K>T	Substitution	Nonsynonymous coding	13%
MM04T	NCR1	natural cytotoxicity triggering receptor 1 [Source:HGNC Symbol;Acc:6731]	CCDS12911.1	chr19_55424096-55424096_C_T	258R>W	Substitution	Nonsynonymous coding	22%
MM04T	NCR3LG1	natural killer cell cytotoxicity receptor 3 ligand 1	ENST00000338965	chr11_17393815-17393815_G_A	374R>Q	Substitution	Nonsynonymous coding	36%

MM04T	NDNF	neuron-derived neurotrophic factor	CCDS3717.2	chr4_121958456-121958456_G_T	224L>I	Substitution	Nonsynonymous coding	21%
MM04T	NDRG3	NDRG family member 3	CCDS13285.1	chr20_35315993-35315993_G_T	74F>L	Substitution	Nonsynonymous coding	29%
MM04T	NDST1	N-deacetylase/N-sulfotransferase (heparan glucosaminyl) 1	CCDS34277.1	chr5_149927883-149927883_G_A	750R>H	Substitution	Nonsynonymous coding	23%
MM04T	NDST3	N-deacetylase/N-sulfotransferase (heparan glucosaminyl) 3	CCDS3708.1	chr4_119174662-119174662_C_A	803S>Y	Substitution	Nonsynonymous coding	31%
MM04T	NDST4	N-deacetylase/N-sulfotransferase (heparan glucosaminyl) 4	CCDS3706.1	chr4_115998171-115998171_G_A	8R>W	Substitution	Nonsynonymous coding	34%
MM04T	NDUFA9	NADH dehydrogenase (ubiquinone) 1 alpha subcomplex, 9, 39kDa	CCDS8532.1	chr12_4763595-4763595_G_T	63G>X	Substitution	Nonsense	20%
MM04T	NDUFB11	NADH dehydrogenase (ubiquinone) 1 beta subcomplex, 11, 17.3kDa	CCDS14273.1	chrX_47002088-47002088_C_T	88R>Q	Substitution	Nonsynonymous coding	49%
MM04T	NDUFB5	NADH dehydrogenase (ubiquinone) 1 beta subcomplex, 5, 16kDa	CCDS3234.1	chr3_179334788-179334788_G_T	100E>X	Substitution	Nonsense	27%
MM04T	NDUFS1	NADH dehydrogenase (ubiquinone) Fe-S protein 1, 75kDa (NADH-coenzyme Q	CCDS2366.1	chr2_206997826-206997826_G_T	466L>I	Substitution	Nonsynonymous coding	21%
MM04T	NDUFS1	NADH dehydrogenase (ubiquinone) Fe-S protein 1, 75kDa (NADH-coenzyme Q	CCDS2366.1	chr2_206991490-206991490_G_A	655R>X	Substitution	Nonsense	39%
MM04T	NDUFS2	NADH dehydrogenase (ubiquinone) Fe-S protein 2, 49kDa (NADH-coenzyme Q	CCDS1224.1	chr1_161179344-161179344_G_A	196A>T	Substitution	Nonsynonymous coding	34%
MM04T	NEB	nebulin	CCDS46424.1	chr2_152373001-152373001_G_A	5925A>V	Substitution	Nonsynonymous coding	27%
MM04T	NEB	nebulin	CCDS46424.1	chr2_152380907-152380907_C_T	5765R>H	Substitution	Nonsynonymous coding	32%
MM04T	NEB	nebulin	CCDS46424.1	chr2_152392251-152392251_G_T	5388S>Y	Substitution	Nonsynonymous coding	24%
MM04T	NEB	nebulin	CCDS46424.1	chr2_152534219-152534219_C_T	1212D>N	Substitution	Nonsynonymous coding	30%
MM04T	NEB	nebulin	CCDS46424.1	chr2_152553658-152553658_T_G	NA	Substitution	Splice site donor	29%
MM04T	NEBL	nebulette	CCDS7134.1	chr10_21120156-21120156_C_T	547R>Q	Substitution	Nonsynonymous coding	25%
MM04T	NEFH	neurofilament, heavy polypeptide	CCDS13858.1	chr22_29876807-29876807_G_T	186D>Y	Substitution	Nonsynonymous coding	28%
MM04T	NEFL	neurofilament, light polypeptide	ENST00000221169	chr8_24811806-24811806_T_C	353K>R	Substitution	Nonsynonymous coding	38%
MM04T	NEFL	neurofilament, light polypeptide	ENST00000221169	chr8_24813333-24813333_C_T	233E>K	Substitution	Nonsynonymous coding	36%
MM04T	NEK10	NIMA-related kinase 10	CCDS46781.1	chr3_27333024-27333024_C_T	476R>H	Substitution	Nonsynonymous coding	29%
MM04T	NEK10	NIMA-related kinase 10	CCDS46781.1	chr3_27393996-27393996_C_A	31R>I	Substitution	Nonsynonymous coding	24%
MM04T	NEK5	NIMA-related kinase 5	CCDS31979.1	chr13_52639709-52639709_C_T	654R>K	Substitution	Nonsynonymous coding	23%
MM04T	NEK5	NIMA-related kinase 5	CCDS31979.1	chr13_52682523-52682523_C_T	162R>Q	Substitution	Nonsynonymous coding	26%
MM04T	NEK5	NIMA-related kinase 5	CCDS31979.1	chr13_52686454-52686454_G_T	88L>I	Substitution	Nonsynonymous coding	18%
MM04T	NELL1	NEL-like 1 (chicken)	CCDS7855.1	chr11_21135237-21135237_G_A	468R>H	Substitution	Nonsynonymous coding	25%
MM04T	NELL1	NEL-like 1 (chicken)	CCDS7855.1	chr11_21594834-21594834_G_A	754C>Y	Substitution	Nonsynonymous coding	34%
MM04T	NELL1	NEL-like 1 (chicken)	CCDS7855.1	chr11_21594864-21594864_G_T	764R>I	Substitution	Nonsynonymous coding	22%
MM04T	NELL2	NEL-like 2 (chicken)	CCDS44863.1	chr12_44917145-44917145_C_A	693D>Y	Substitution	Nonsynonymous coding	39%
MM04T	NELL2	NEL-like 2 (chicken)	CCDS44863.1	chr12_45108447-45108447_G_T	408L>I	Substitution	Nonsynonymous coding	30%
MM04T	NEO1	neogenin 1	CCDS10247.1	chr15_73590697-73590697_C_T	1304R>X	Substitution	Nonsense	29%
MM04T	NES	nestin	CCDS1151.1	chr1_156641223-156641223_C_A	919E>D	Substitution	Nonsynonymous coding	35%

MM04T	NES	nestin	CCDS1151.1	chr1_156641450-156641450_C_A	844E>X	Substitution	Nonsense	21%
MM04T	NES	nestin	CCDS1151.1	chr1_156641546-156641546_C_A	812E>X	Substitution	Nonsense	23%
MM04T	NETO1	neuropilin (NRP) and tolloid (TLL)-like 1	CCDS12000.1	chr18_70417352-70417352_C_T	496E>K	Substitution	Nonsynonymous coding	40%
MM04T	NEU3	sialidase 3 (membrane sialidase)	CCDS44682.1	chr11_74716917-74716917_A_G	256T>A	Substitution	Nonsynonymous coding	30%
MM04T	NEU4	sialidase 4	CCDS2553.1	chr2_242757862-242757862_G_A	327E>K	Substitution	Nonsynonymous coding	10%
MM04T	NEXN	nexilin (F actin binding protein)	CCDS41351.1	chr1_78392170-78392170_G_T	187K>N	Substitution	Nonsynonymous coding	33%
MM04T	NEXN	nexilin (F actin binding protein)	CCDS41351.1	chr1_78407840-78407840_A_C	536K>Q	Substitution	Nonsynonymous coding	25%
MM04T	NEXN	nexilin (F actin binding protein)	CCDS41351.1	chr1_78392300-78392300_A_C	NA	Substitution	Splice site donor	34%
MM04T	NEXN	nexilin (F actin binding protein)	CCDS41351.1	chr1_78399012-78399012_G_T	367E>X	Substitution	Nonsense	15%
MM04T	NF1	neurofibromin 1	CCDS42292.1	chr17_29677227-29677227_C_T	2450R>X	Substitution	Nonsense	28%
MM04T	NFASC	neurofascin	NM_001005388	chr1_204971834-204971834_G_A	1083E>K	Substitution	Nonsynonymous coding	29%
MM04T	NFAT5	nuclear factor of activated T-cells 5, tonicity-responsive	CCDS45518.1	chr16_69726291-69726291_A_T	855I>F	Substitution	Nonsynonymous coding	35%
MM04T	NFAT5	nuclear factor of activated T-cells 5, tonicity-responsive	CCDS45518.1	chr16_69660356-69660356_G_T	60E>X	Substitution	Nonsense	32%
MM04T	NFATC1	nuclear factor of activated T-cells, cytoplasmic, calcineurin-dependent 1	CCDS32850.1	chr18_77208926-77208926_C_A	498L>I	Substitution	Nonsynonymous coding	28%
MM04T	NFATC1	nuclear factor of activated T-cells, cytoplasmic, calcineurin-dependent 1	CCDS32850.1	chr18_77246463-77246463_G_A	757D>N	Substitution	Nonsynonymous coding	21%
MM04T	NFATC3	nuclear factor of activated T-cells, cytoplasmic, calcineurin-dependent 3	CCDS10860.1	chr16_68200824-68200824_G_T	560K>N	Substitution	Nonsynonymous coding	38%
MM04T	NFIA	nuclear factor I/A	CCDS44156.1	chr1_61554118-61554118_G_T	109D>Y	Substitution	Nonsynonymous coding	25%
MM04T	NFIB	nuclear factor I/B	CCDS6474.1	chr9_14307303-14307303_C_A	83D>Y	Substitution	Nonsynonymous coding	25%
MM04T	NFKB1	nuclear factor of kappa light polypeptide gene enhancer in B-cells 1	CCDS3657.1	chr4_103534603-103534603_G_T	872E>X	Substitution	Nonsense	27%
MM04T	NFKB2	nuclear factor of kappa light polypeptide gene enhancer in B-cells 2 (p49/p100)	CCDS41564.1	chr10_104158227-104158227_G_A	313R>Q	Substitution	Nonsynonymous coding	21%
MM04T	NFKB2	nuclear factor of kappa light polypeptide gene enhancer in B-cells 2 (p49/p100)	CCDS41564.1	chr10_104161804-104161804_G_A	NA	Substitution	Splice site acceptor	27%
MM04T	NFRKB	nuclear factor related to kappaB binding protein	CCDS8483.1	chr11_129744712-129744712_G_A	677R>C	Substitution	Nonsynonymous coding	42%
MM04T	NFYB	nuclear transcription factor Y, beta	CCDS9098.1	chr12_104517171-104517171_C_A	88E>X	Substitution	Nonsense	47%
MM04T	NHLRC1	NHL repeat containing 1	CCDS4542.1	chr6_18122084-18122084_G_A	252R>W	Substitution	Nonsynonymous coding	24%
MM04T	NHLRC2	NHL repeat containing 2	CCDS7585.1	chr10_115636505-115636505_A_C	186K>T	Substitution	Nonsynonymous coding	37%
MM04T	NHS	Nance-Horan syndrome (congenital cataracts and dental anomalies)	CCDS14181.1	chrX_17750121-17750121_G_A	1477R>K	Substitution	Nonsynonymous coding	17%
MM04T	NHSL2	NHS-like 2	CCDS35328.1	chrX_71360222-71360222_A_G	576T>A	Substitution	Nonsynonymous coding	26%
MM04T	NID1	nidogen 1	CCDS1608.1	chr1_236201550-236201550_A_G	380F>S	Substitution	Nonsynonymous coding	28%
MM04T	NIN	ninein (GSK3B interacting protein)	CCDS41952.1	chr14_51239725-51239725_G_A	252S>F	Substitution	Nonsynonymous coding	39%
MM04T	NKAPL	NFKB activating protein-like	CCDS34353.1	chr6_28227812-28227812_G_T	221K>N	Substitution	Nonsynonymous coding	29%
MM04T	NKPD1	NTPase, KAP family P-loop domain containing 1	CCDS42577.1	chr19_45655876-45655876_C_T	385E>K	Substitution	Nonsynonymous coding	40%
MM04T	NKPD1	NTPase, KAP family P-loop domain containing 1	CCDS42577.1	chr19_45656133-45656133_C_T	299R>H	Substitution	Nonsynonymous coding	35%

MM04T	NKRF	NFKB repressing factor	CCDS35375.1	chrX_118724901-118724901_G_A	163P>S	Substitution	Nonsynonymous coding	15%
MM04T	NKRF	NFKB repressing factor	CCDS35375.1	chrX_118726451-118726451_T_G	4I>L	Substitution	Nonsynonymous coding	20%
MM04T	NKX2-5	NK2 homeobox 5	CCDS4387.1	chr5_172659609-172659609_G_A	313S>L	Substitution	Nonsynonymous coding	32%
MM04T	NLE1	notchless homolog 1 (Drosophila)	CCDS11291.1	chr17_33462389-33462389_G_T	365L>I	Substitution	Nonsynonymous coding	19%
MM04T	NLGN1	neuroligin 1	CCDS3222.1	chr3_173998399-173998399_G_T	593R>I	Substitution	Nonsynonymous coding	31%
MM04T	NLGN2	neuroligin 2	CCDS11103.1	chr17_7320493-7320493_G_A	628R>H	Substitution	Nonsynonymous coding	29%
MM04T	NLGN3	neuroligin 3	CCDS14407.1	chrX_70384026-70384026_G_A	234G>D	Substitution	Nonsynonymous coding	33%
MM04T	NLGN4X	neuroligin 4, X-linked	CCDS14126.1	chrX_6069100-6069100_C_A	136M>I	Substitution	Nonsynonymous coding	28%
MM04T	NLRC3	NLR family, CARD domain containing 3	ENST00000448023	chr16_3591858-3591858_C_A	1101K>N	Substitution	Nonsynonymous coding	19%
MM04T	NLRC3	NLR family, CARD domain containing 3	ENST00000448023	chr16_3611719-3611719_G_A	714R>C	Substitution	Nonsynonymous coding	19%
MM04T	NLRC4	NLR family, CARD domain containing 4	CCDS33174.1	chr2_32460499-32460499_C_T	918R>K	Substitution	Nonsynonymous coding	30%
MM04T	NLRC4	NLR family, CARD domain containing 4	CCDS33174.1	chr2_32476391-32476391_C_T	181R>Q	Substitution	Nonsynonymous coding	31%
MM04T	NLRC4	NLR family, CARD domain containing 4	CCDS33174.1	chr2_32463252-32463252_C_A	824E>X	Substitution	Nonsense	30%
MM04T	NLRP1	NLR family, pyrin domain containing 1	CCDS42246.1	chr17_5418184-5418184_G_A	1438R>W	Substitution	Nonsynonymous coding	33%
MM04T	NLRP10	NLR family, pyrin domain containing 10	CCDS7784.1	chr11_7981362-7981362_C_A	599Q>H	Substitution	Nonsynonymous coding	28%
MM04T	NLRP12	NLR family, pyrin domain containing 12	CCDS12864.1	chr19_54314024-54314024_C_T	297D>N	Substitution	Nonsynonymous coding	34%
MM04T	NLRP14	NLR family, pyrin domain containing 14	CCDS7776.1	chr11_7065038-7065038_T_G	594F>C	Substitution	Nonsynonymous coding	39%
MM04T	NLRP14	NLR family, pyrin domain containing 14	CCDS7776.1	chr11_7070949-7070949_C_A	724S>Y	Substitution	Nonsynonymous coding	19%
MM04T	NLRP4	NLR family, pyrin domain containing 4	CCDS12936.1	chr19_56363463-56363463_T_G	6F>C	Substitution	Nonsynonymous coding	31%
MM04T	NLRP4	NLR family, pyrin domain containing 4	CCDS12936.1	chr19_56369103-56369103_C_T	115S>F	Substitution	Nonsynonymous coding	24%
MM04T	NLRP4	NLR family, pyrin domain containing 4	CCDS12936.1	chr19_56379115-56379115_C_A	743L>I	Substitution	Nonsynonymous coding	22%
MM04T	NLRP5	NLR family, pyrin domain containing 5	CCDS12938.1	chr19_56539737-56539737_C_T	713P>L	Substitution	Nonsynonymous coding	21%
MM04T	NLRP9	NLR family, pyrin domain containing 9	CCDS12934.1	chr19_56244577-56244577_G_A	207P>L	Substitution	Nonsynonymous coding	21%
MM04T	NLRP9	NLR family, pyrin domain containing 9	CCDS12934.1	chr19_56249632-56249632_A_C	37F>V	Substitution	Nonsynonymous coding	29%
MM04T	NLRX1	NLR family member X1	CCDS8416.1	chr11_119054038-119054038_C_A	940L>M	Substitution	Nonsynonymous coding	21%
MM04T	NM_001037228	-	NM_001037228	chr2_96906138-96906138_G_A	26R>Q	Substitution	Nonsynonymous coding	30%
MM04T	NM_001037228	-	NM_001037228	chr2_96906231-96906231_C_A	57S>Y	Substitution	Nonsynonymous coding	24%
MM04T	NMB	neuromedin B	CCDS42076.1	chr15_85198619-85198619_T_G	116N>T	Substitution	Nonsynonymous coding	40%
MM04T	NME8	NME/NM23 family member 8	CCDS5452.1	chr7_37936660-37936660_T_C	578V>A	Substitution	Nonsynonymous coding	28%
MM04T	NME9	NME/NM23 family member 9	CCDS3099.1	chr3_138022463-138022463_C_A	211E>X	Substitution	Nonsense	22%
MM04T	NMT2	N-myristoyltransferase 2	CCDS7109.1	chr10_15154910-15154910_G_A	408T>M	Substitution	Nonsynonymous coding	24%
MM04T	NMT2	N-myristoyltransferase 2	CCDS7109.1	chr10_15170410-15170410_A_C	313F>C	Substitution	Nonsynonymous coding	23%

MM04T	NNT	nicotinamide nucleotide transhydrogenase	CCDS3949.1	chr5_43655941-43655941_G_T	NA	Substitution	Splice site acceptor	23%
MM04T	NOA1	nitric oxide associated 1	CCDS3510.1	chr4_57829786-57829786_G_T	643L>I	Substitution	Nonsynonymous coding	18%
MM04T	NOBOX	NOBOX oogenesis homeobox	NM_001080413	chr7_144095402-144095402_G_T	466L>M	Substitution	Nonsynonymous coding	19%
MM04T	NOBOX	NOBOX oogenesis homeobox	NM_001080413	chr7_144095513-144095513_G_A	429P>S	Substitution	Nonsynonymous coding	18%
MM04T	NOC3L	nucleolar complex associated 3 homolog (S. cerevisiae)	CCDS7433.1	chr10_96112709-96112709_C_A	268D>Y	Substitution	Nonsynonymous coding	24%
MM04T	NOL7	nucleolar protein 7, 27kDa	CCDS4528.1	chr6_13615949-13615949_A_C	91K>T	Substitution	Nonsynonymous coding	33%
MM04T	NOM1	nucleolar protein with MIF4G domain 1	CCDS34787.1	chr7_156756692-156756692_T_G	669F>V	Substitution	Nonsynonymous coding	39%
MM04T	NOP56	NOP56 ribonucleoprotein	CCDS13030.1	chr20_2637727-2637727_G_A	428A>T	Substitution	Nonsynonymous coding	24%
MM04T	NOS2	nitric oxide synthase 2, inducible	CCDS11223.1	chr17_26096177-26096177_C_A	620R>S	Substitution	Nonsynonymous coding	30%
MM04T	NOS2	nitric oxide synthase 2, inducible	CCDS11223.1	chr17_26105938-26105938_G_T	383F>L	Substitution	Nonsynonymous coding	21%
MM04T	NOS3	nitric oxide synthase 3 (endothelial cell)	CCDS5912.1	chr7_150709464-150709464_G_A	1004D>N	Substitution	Nonsynonymous coding	37%
MM04T	NOSTRIN	nitric oxide synthase trafficker	CCDS42771.1	chr2_169699615-169699615_G_T	206E>D	Substitution	Nonsynonymous coding	18%
MM04T	NOSTRIN	nitric oxide synthase trafficker	CCDS42771.1	chr2_169717361-169717361_G_T	412E>D	Substitution	Nonsynonymous coding	19%
MM04T	NOTCH2	notch 2	CCDS908.1	chr1_120458509-120458509_T_C	2279H>R	Substitution	Nonsynonymous coding	29%
MM04T	NOTCH2	notch 2	CCDS908.1	chr1_120458746-120458746_G_T	2200S>Y	Substitution	Nonsynonymous coding	30%
MM04T	NOTCH2	notch 2	CCDS908.1	chr1_120461166-120461166_C_T	1931R>H	Substitution	Nonsynonymous coding	32%
MM04T	NOTCH2	notch 2	CCDS908.1	chr1_120479946-120479946_C_T	1161G>R	Substitution	Nonsynonymous coding	30%
MM04T	NOTO	notochord homeobox	CCDS46335.1	chr2_73430015-73430015_T_C	74V>A	Substitution	Nonsynonymous coding	37%
MM04T	NOTUM	notum pectinacetylesterase homolog (Drosophila)	CCDS32771.2	chr17_79915727-79915727_C_T	217G>D	Substitution	Nonsynonymous coding	24%
MM04T	NOX1	NADPH oxidase 1	CCDS14474.1	chrX_100129086-100129086_G_T	15L>M	Substitution	Nonsynonymous coding	27%
MM04T	NOX4	NADPH oxidase 4	CCDS8285.1	chr11_89135534-89135534_A_T	269I>N	Substitution	Nonsynonymous coding	36%
MM04T	NOXO1	NADPH oxidase organizer 1	CCDS42101.1	chr16_2030686-2030686_C_A	65R>I	Substitution	Nonsynonymous coding	35%
MM04T	NPAP1	nuclear pore associated protein 1	CCDS10015.1	chr15_24923194-24923194_T_G	727L>R	Substitution	Nonsynonymous coding	19%
MM04T	NPAS3	neuronal PAS domain protein 3	CCDS9645.1	chr14_34243634-34243634_C_T	283A>V	Substitution	Nonsynonymous coding	27%
MM04T	NPAS4	neuronal PAS domain protein 4	CCDS8138.1	chr11_66191619-66191619_C_A	420L>I	Substitution	Nonsynonymous coding	29%
MM04T	NPAT	nuclear protein, ataxia-telangiectasia locus	CCDS41710.1	chr11_108043828-108043828_G_A	628S>L	Substitution	Nonsynonymous coding	27%
MM04T	NPAT	nuclear protein, ataxia-telangiectasia locus	CCDS41710.1	chr11_108044135-108044135_G_T	526L>I	Substitution	Nonsynonymous coding	37%
MM04T	NPAT	nuclear protein, ataxia-telangiectasia locus	CCDS41710.1	chr11_108043970-108043970_C_A	581E>X	Substitution	Nonsense	53%
MM04T	NPC1	Niemann-Pick disease, type C1	CCDS11878.1	chr18_21121069-21121069_G_T	826S>Y	Substitution	Nonsynonymous coding	28%
MM04T	NPEPL1	aminopeptidase-like 1	CCDS46621.1	chr20_57287576-57287576_G_T	314E>D	Substitution	Nonsynonymous coding	21%
MM04T	NPHP3	nephronophthisis 3 (adolescent)	CCDS3078.1	chr3_132424596-132424596_C_A	446K>N	Substitution	Nonsynonymous coding	22%
MM04T	NPHP3	nephronophthisis 3 (adolescent)	CCDS3078.1	chr3_132402320-132402320_G_A	1207R>X	Substitution	Nonsense	27%

MM04T	NPHS2	nephrosis 2, idiopathic, steroid-resistant (podocin)	CCDS1331.1	chr1_179520321-179520321_G_T	380S>Y	Substitution	Nonsynonymous coding	24%
MM04T	NPL	N-acetylneuraminate pyruvate lyase (dihydrodipicolinate synthase)	CCDS1350.1	chr1_182798000-182798000_C_A	307S>Y	Substitution	Nonsynonymous coding	31%
MM04T	NPR1	natriuretic peptide receptor A/guanylate cyclase A (atrionatriuretic peptide receptor A)	CCDS1051.1	chr1_153661467-153661467_G_A	819R>H	Substitution	Nonsynonymous coding	31%
MM04T	NPSR1	neuropeptide S receptor 1	CCDS5443.1	chr7_34698098-34698098_C_A	25A>D	Substitution	Nonsynonymous coding	25%
MM04T	NPSR1	neuropeptide S receptor 1	CCDS5443.1	chr7_34867065-34867065_C_A	177F>L	Substitution	Nonsynonymous coding	29%
MM04T	NPTN	neuroplastin	CCDS10249.1	chr15_73862668-73862668_C_T	313E>K	Substitution	Nonsynonymous coding	25%
MM04T	NPTX1	neuronal pentraxin I	CCDS32762.1	chr17_78447131-78447131_A_C	256C>G	Substitution	Nonsynonymous coding	22%
MM04T	NPY1R	neuropeptide Y receptor Y1	CCDS34089.1	chr4_164246862-164246862_C_A	250D>Y	Substitution	Nonsynonymous coding	33%
MM04T	NPY5R	neuropeptide Y receptor Y5	CCDS3804.1	chr4_164272343-164272343_G_T	306E>D	Substitution	Nonsynonymous coding	27%
MM04T	NR0B2	nuclear receptor subfamily 0, group B, member 2	CCDS291.1	chr1_27239964-27239964_G_T	156F>L	Substitution	Nonsynonymous coding	22%
MM04T	NR1H4	nuclear receptor subfamily 1, group H, member 4	CCDS9078.1	chr12_100904888-100904888_C_A	138H>N	Substitution	Nonsynonymous coding	19%
MM04T	NR1H4	nuclear receptor subfamily 1, group H, member 4	CCDS9078.1	chr12_100930735-100930735_G_T	277E>X	Substitution	Nonsense	15%
MM04T	NR1I3	nuclear receptor subfamily 1, group I, member 3	CCDS41429.1	chr1_161205738-161205738_G_A	46T>I	Substitution	Nonsynonymous coding	34%
MM04T	NR2C1	nuclear receptor subfamily 2, group C, member 1	CCDS9051.1	chr12_95456378-95456378_G_A	64S>F	Substitution	Nonsynonymous coding	30%
MM04T	NR2E1	nuclear receptor subfamily 2, group E, member 1	CCDS5063.1	chr6_108508587-108508587_G_T	360E>X	Substitution	Nonsense	38%
MM04T	NR4A2	nuclear receptor subfamily 4, group A, member 2	CCDS2201.1	chr2_157182372-157182372_C_A	561E>X	Substitution	Nonsense	32%
MM04T	NRCAM	neuronal cell adhesion molecule	CCDS47686.1	chr7_107834834-107834834_A_G	501I>T	Substitution	Nonsynonymous coding	13%
MM04T	NRD1	nardilysin (N-arginine dibasic convertase)	CCDS559.1	chr1_52272559-52272559_C_T	741D>N	Substitution	Nonsynonymous coding	28%
MM04T	NRG1	neuregulin 1	CCDS6083.1	chr8_32607084-32607084_C_T	233L>F	Substitution	Nonsynonymous coding	23%
MM04T	NRG3	neuregulin 3	CCDS31233.1	chr10_83635427-83635427_G_T	111D>Y	Substitution	Nonsynonymous coding	30%
MM04T	NRIP2	nuclear receptor interacting protein 2	CCDS8514.1	chr12_2944031-2944031_C_A	40S>I	Substitution	Nonsynonymous coding	20%
MM04T	NRK	Nik related kinase	NM_198465	chrX_105142622-105142622_G_T	209R>M	Substitution	Nonsynonymous coding	23%
MM04T	NRK	Nik related kinase	NM_198465	chrX_105161586-105161586_C_A	812L>I	Substitution	Nonsynonymous coding	24%
MM04T	NRK	Nik related kinase	NM_198465	chrX_105168859-105168859_A_C	1050I>L	Substitution	Nonsynonymous coding	36%
MM04T	NRK	Nik related kinase	NM_198465	chrX_105193708-105193708_G_T	1499D>Y	Substitution	Nonsynonymous coding	26%
MM04T	NRK	Nik related kinase	NM_198465	chrX_105152231-105152231_G_T	NA	Substitution	Splice site acceptor	24%
MM04T	NRXN1	neurexin 1	CCDS46282.1	chr2_50758472-50758472_C_T	787R>Q	Substitution	Nonsynonymous coding	34%
MM04T	NRXN3	neurexin 3	ENST00000330071	chr14_79454510-79454510_G_T	857W>C	Substitution	Nonsynonymous coding	20%
MM04T	NRXN3	neurexin 3	CCDS9870.1	chr14_80130277-80130277_G_T	828E>X	Substitution	Nonsense	27%
MM04T	NSD1	nuclear receptor binding SET domain protein 1	CCDS4412.1	chr5_176637437-176637437_G_T	679Q>H	Substitution	Nonsynonymous coding	29%
MM04T	NSD1	nuclear receptor binding SET domain protein 1	CCDS4412.1	chr5_176631273-176631273_G_T	406E>X	Substitution	Nonsense	17%
MM04T	NSD1	nuclear receptor binding SET domain protein 1	CCDS4412.1	chr5_176700702-176700702_G_T	1847E>X	Substitution	Nonsense	19%

MM04T	NSUN6	NOP2/Sun domain family, member 6	CCDS7130.1	chr10_18834992-18834992_G_A	427S>L	Substitution	Nonsynonymous coding	23%
MM04T	NT5C1A	5'-nucleotidase, cytosolic IA	CCDS440.1	chr1_40125038-40125038_A_G	288S>P	Substitution	Nonsynonymous coding	32%
MM04T	NT5C1B	5'-nucleotidase, cytosolic IB	CCDS33150.1	chr2_18766033-18766033_G_A	217A>V	Substitution	Nonsynonymous coding	18%
MM04T	NT5C3B	5'-nucleotidase, cytosolic IIIB	CCDS11410.1	chr17_39983831-39983831_C_A	197K>N	Substitution	Nonsynonymous coding	33%
MM04T	NTF3	neurotrophin 3	CCDS44806.1	chr12_5603687-5603687_C_T	116R>W	Substitution	Nonsynonymous coding	32%
MM04T	NTN1	netrin 1	CCDS11148.1	chr17_8926181-8926181_C_A	164S>Y	Substitution	Nonsynonymous coding	32%
MM04T	NTN4	netrin 4	CCDS9054.1	chr12_96180959-96180959_C_A	115E>X	Substitution	Nonsense	31%
MM04T	NTRK3	neurotrophic tyrosine kinase, receptor, type 3	CCDS32322.1	chr15_88476407-88476407_C_A	575K>N	Substitution	Nonsynonymous coding	30%
MM04T	NTS	neurotensin	CCDS9029.1	chr12_86276116-86276116_G_T	159R>I	Substitution	Nonsynonymous coding	13%
MM04T	NUDCD3	NudC domain containing 3	CCDS5490.2	chr7_44425668-44425668_C_T	343R>Q	Substitution	Nonsynonymous coding	36%
MM04T	NUDT18	nudix (nucleoside diphosphate linked moiety X)-type motif 18	ENST00000309188	chr8_21965319-21965319_C_T	155R>Q	Substitution	Nonsynonymous coding	28%
MM04T	NUDT5	nudix (nucleoside diphosphate linked moiety X)-type motif 5	ENST00000444732	chr10_12221028-12221028_G_A	79R>C	Substitution	Nonsynonymous coding	23%
MM04T	NUDT7	nudix (nucleoside diphosphate linked moiety X)-type motif 7	CCDS42195.1	chr16_77775490-77775490_G_T	120L>F	Substitution	Nonsynonymous coding	25%
MM04T	NUF2	NUF2, NDC80 kinetochore complex component, homolog (S. cerevisiae)	CCDS1245.1	chr1_163297332-163297332_C_T	60R>X	Substitution	Nonsense	27%
MM04T	NUMBL	numb homolog (Drosophila)-like	CCDS12561.1	chr19_41173827-41173827_G_A	459A>V	Substitution	Nonsynonymous coding	33%
MM04T	NUP133	nucleoporin 133kDa	ENST00000366678	chr1_229634958-229634958_C_A	220E>X	Substitution	Nonsense	26%
MM04T	NUP153	nucleoporin 153kDa	CCDS4541.1	chr6_17629522-17629522_C_T	970E>K	Substitution	Nonsynonymous coding	31%
MM04T	NUP155	nucleoporin 155kDa	CCDS3921.1	chr5_37314344-37314344_G_T	798L>I	Substitution	Nonsynonymous coding	20%
MM04T	NUP205	nucleoporin 205kDa	CCDS34759.1	chr7_135287723-135287723_A_C	895N>H	Substitution	Nonsynonymous coding	28%
MM04T	NUP205	nucleoporin 205kDa	CCDS34759.1	chr7_135289102-135289102_G_T	906G>V	Substitution	Nonsynonymous coding	24%
MM04T	NUP210	nucleoporin 210kDa	CCDS33704.1	chr3_13395119-13395119_C_T	855A>T	Substitution	Nonsynonymous coding	35%
MM04T	NUP210L	nucleoporin 210kDa-like	CCDS41399.1	chr1_154101806-154101806_G_T	342S>Y	Substitution	Nonsynonymous coding	17%
MM04T	NUP210L	nucleoporin 210kDa-like	CCDS41399.1	chr1_154108372-154108372_C_A	309E>D	Substitution	Nonsynonymous coding	17%
MM04T	NUP210L	nucleoporin 210kDa-like	CCDS41399.1	chr1_154125221-154125221_G_A	111R>X	Substitution	Nonsense	20%
MM04T	NUP37	nucleoporin 37kDa	CCDS9089.1	chr12_102492897-102492897_C_T	146D>N	Substitution	Nonsynonymous coding	22%
MM04T	NUP88	nucleoporin 88kDa	CCDS11070.1	chr17_5294966-5294966_G_A	467R>X	Substitution	Nonsense	25%
MM04T	NVL	nuclear VCP-like	CCDS1541.1	chr1_224424257-224424257_C_T	773D>N	Substitution	Nonsynonymous coding	27%
MM04T	NXF1	nuclear RNA export factor 1	CCDS8037.1	chr11_62570989-62570989_G_A	91R>C	Substitution	Nonsynonymous coding	43%
MM04T	NXF1	nuclear RNA export factor 1	CCDS8037.1	chr11_62571417-62571417_C_A	21R>I	Substitution	Nonsynonymous coding	22%
MM04T	NXF3	nuclear RNA export factor 3	CCDS14503.1	chrX_102334524-102334524_G_T	403L>I	Substitution	Nonsynonymous coding	34%
MM04T	NXPE2	neurexophilin and PC-esterase domain family, member 2	CCDS44738.1	chr11_114576510-114576510_G_T	312E>D	Substitution	Nonsynonymous coding	17%
MM04T	NXPE3	neurexophilin and PC-esterase domain family, member 3	CCDS2945.1	chr3_101520161-101520161_G_A	59R>Q	Substitution	Nonsynonymous coding	34%

MM04T	NXPE4	neurexophilin and PC-esterase domain family, member 4	CCDS41718.1	chr11_114441872-114441872_C_A	475E>X	Substitution	Nonsense	25%
MM04T	NYAP2	neuronal tyrosine-phosphorylated phosphoinositide-3-kinase adaptor 2	CCDS46529.1	chr2_226447697-226447697_C_T	522R>C	Substitution	Nonsynonymous coding	29%
MM04T	OAS2	2'-5'-oligoadenylate synthetase 2, 69/71kDa	CCDS31906.1	chr12_113435557-113435557_C_T	287A>V	Substitution	Nonsynonymous coding	42%
MM04T	OBSCN	obscurin, cytoskeletal calmodulin and titin-interacting RhoGEF	NM_001098623	chr1_228402664-228402664_G_A	565E>K	Substitution	Nonsynonymous coding	29%
MM04T	OBSCN	obscurin, cytoskeletal calmodulin and titin-interacting RhoGEF	NM_001098623	chr1_228554845-228554845_A_G	6533T>A	Substitution	Nonsynonymous coding	34%
MM04T	OBSCN	obscurin, cytoskeletal calmodulin and titin-interacting RhoGEF	NM_001098623	chr1_228559766-228559766_C_A	7096S>Y	Substitution	Nonsynonymous coding	20%
MM04T	OC90	otoconin 90	CCDS47919.1	chr8_133041405-133041405_G_T	351C>X	Substitution	Nonsense	33%
MM04T	OCRL	oculocerebrorenal syndrome of Lowe	CCDS35393.1	chrX_128722235-128722235_C_A	779T>K	Substitution	Nonsynonymous coding	29%
MM04T	ODF2L	outer dense fiber of sperm tails 2-like	CCDS30763.1	chr1_86850427-86850427_C_A	102E>X	Substitution	Nonsense	28%
MM04T	OGDH	oxoglutarate (alpha-ketoglutarate) dehydrogenase (lipoamide)	CCDS34627.1	chr7_44736650-44736650_C_T	680R>W	Substitution	Nonsynonymous coding	29%
MM04T	OGT	O-linked N-acetylglucosamine (GlcNAc) transferase	CCDS14414.1	chrX_70782762-70782762_G_T	681Q>H	Substitution	Nonsynonymous coding	30%
MM04T	OGT	O-linked N-acetylglucosamine (GlcNAc) transferase	CCDS14414.1	chrX_70782770-70782770_C_T	684S>L	Substitution	Nonsynonymous coding	27%
MM04T	OLFM3	olfactomedin 3	ENST00000338858	chr1_102312522-102312522_G_T	3P>H	Substitution	Nonsynonymous coding	22%
MM04T	OLFM4	olfactomedin 4	CCDS9440.1	chr13_53624884-53624884_C_A	504S>Y	Substitution	Nonsynonymous coding	21%
MM04T	OLFML1	olfactomedin-like 1	CCDS7779.1	chr11_7509403-7509403_G_T	59E>X	Substitution	Nonsense	17%
MM04T	OLFML2B	olfactomedin-like 2B	CCDS1236.1	chr1_161953871-161953871_C_T	616G>D	Substitution	Nonsynonymous coding	34%
MM04T	OPA1	optic atrophy 1 (autosomal dominant)	CCDS33917.1	chr3_193382781-193382781_C_A	907S>Y	Substitution	Nonsynonymous coding	29%
MM04T	OPN1SW	opsin 1 (cone pigments), short-wave-sensitive	CCDS5806.1	chr7_128414556-128414556_T_G	228K>T	Substitution	Nonsynonymous coding	27%
MM04T	OPTN	optineurin	CCDS7094.1	chr10_13152292-13152292_A_T	62N>I	Substitution	Nonsynonymous coding	34%
MM04T	OR10A7	olfactory receptor, family 10, subfamily A, member 7	CCDS31815.1	chr12_55614848-55614848_C_A	14L>I	Substitution	Nonsynonymous coding	32%
MM04T	OR10AB1P	olfactory receptor, family 10, subfamily AB, member 1 pseudogene	ENST00000317359	chr11_7750304-7750304_G_A	130R>H	Substitution	Nonsynonymous coding	30%
MM04T	OR10D3	olfactory receptor, family 10, subfamily D, member 3 (non-functional)	ENST00000318666	chr11_124056323-124056323_C_T	116T>M	Substitution	Nonsynonymous coding	31%
MM04T	OR10G7	olfactory receptor, family 10, subfamily G, member 7	CCDS31705.1	chr11_123908824-123908824_C_A	295K>N	Substitution	Nonsynonymous coding	32%
MM04T	OR10J1	olfactory receptor, family 10, subfamily J, member 1	CCDS1185.1	chr1_159410402-159410402_C_T	285S>L	Substitution	Nonsynonymous coding	10%
MM04T	OR10Q1	olfactory receptor, family 10, subfamily Q, member 1	CCDS31547.1	chr11_57996257-57996257_G_T	31L>I	Substitution	Nonsynonymous coding	26%
MM04T	OR10R2	olfactory receptor, family 10, subfamily R, member 2	CCDS30898.1	chr1_158450217-158450217_T_A	184F>I	Substitution	Nonsynonymous coding	35%
MM04T	OR10X1	olfactory receptor, family 10, subfamily X, member 1	CCDS30900.1	chr1_158548845-158548845_C_T	282G>E	Substitution	Nonsynonymous coding	23%
MM04T	OR10Z1	olfactory receptor, family 10, subfamily Z, member 1	CCDS30901.1	chr1_158577122-158577122_G_T	298Q>H	Substitution	Nonsynonymous coding	17%
MM04T	OR11H4	olfactory receptor, family 11, subfamily H, member 4	CCDS32034.1	chr14_20711694-20711694_C_A	248F>L	Substitution	Nonsynonymous coding	34%
MM04T	OR11H6	olfactory receptor, family 11, subfamily H, member 6	CCDS32033.1	chr14_20691973-20691973_C_A	35F>L	Substitution	Nonsynonymous coding	30%
MM04T	OR12D3	olfactory receptor, family 12, subfamily D, member 3	ENST00000396806	chr6_29236265-29236265_C_A	308D>Y	Substitution	Nonsynonymous coding	26%
MM04T	OR14J1	olfactory receptor, family 14, subfamily J, member 1	CCDS34362.1	chr6_29274738-29274738_C_A	91S>Y	Substitution	Nonsynonymous coding	30%

MM04T	OR1L1	olfactory receptor, family 1, subfamily L, member 1	CCDS35127.1	chr9_125424594-125424594_G_T	250M>I	Substitution	Nonsynonymous coding	35%
MM04T	OR1L3	olfactory receptor, family 1, subfamily L, member 3	CCDS35128.1	chr9_125437431-125437431_G_T	8R>I	Substitution	Nonsynonymous coding	19%
MM04T	OR1L8	olfactory receptor, family 1, subfamily L, member 8	CCDS35124.1	chr9_125330302-125330302_A_C	152F>C	Substitution	Nonsynonymous coding	35%
MM04T	OR1L8	olfactory receptor, family 1, subfamily L, member 8	CCDS35124.1	chr9_125330591-125330591_G_T	56L>I	Substitution	Nonsynonymous coding	18%
MM04T	OR1M1	olfactory receptor, family 1, subfamily M, member 1	CCDS32896.1	chr19_9204414-9204414_G_A	165R>H	Substitution	Nonsynonymous coding	34%
MM04T	OR1N1	olfactory receptor, family 1, subfamily N, member 1	CCDS6844.1	chr9_125289393-125289393_G_T	60F>L	Substitution	Nonsynonymous coding	17%
MM04T	OR2A12	olfactory receptor, family 2, subfamily A, member 12	CCDS43670.1	chr7_143792299-143792299_C_A	33F>L	Substitution	Nonsynonymous coding	26%
MM04T	OR2A2	olfactory receptor, family 2, subfamily A, member 2	CCDS43671.1	chr7_143807220-143807220_C_T	182S>F	Substitution	Nonsynonymous coding	21%
MM04T	OR2B2	olfactory receptor, family 2, subfamily B, member 2	CCDS4641.1	chr6_27879581-27879581_C_T	173E>K	Substitution	Nonsynonymous coding	31%
MM04T	OR2B3	olfactory receptor, family 2, subfamily B, member 3	CCDS34358.1	chr6_29054524-29054524_G_A	168R>C	Substitution	Nonsynonymous coding	25%
MM04T	OR2B3	olfactory receptor, family 2, subfamily B, member 3	CCDS34358.1	chr6_29054693-29054693_C_G	111E>D	Substitution	Nonsynonymous coding	26%
MM04T	OR2D2	olfactory receptor, family 2, subfamily D, member 2	CCDS31416.1	chr11_6913115-6913115_A_C	206I>S	Substitution	Nonsynonymous coding	25%
MM04T	OR2D2	olfactory receptor, family 2, subfamily D, member 2	CCDS31416.1	chr11_6913436-6913436_G_C	99A>G	Substitution	Nonsynonymous coding	25%
MM04T	OR2F2	olfactory receptor, family 2, subfamily F, member 2	CCDS43666.1	chr7_143632612-143632612_G_A	96S>N	Substitution	Nonsynonymous coding	15%
MM04T	OR2F2	olfactory receptor, family 2, subfamily F, member 2	CCDS43666.1	chr7_143632949-143632949_G_T	208M>I	Substitution	Nonsynonymous coding	29%
MM04T	OR2G3	olfactory receptor, family 2, subfamily G, member 3	CCDS31093.1	chr1_247769133-247769133_A_C	82L>F	Substitution	Nonsynonymous coding	27%
MM04T	OR2W1	olfactory receptor, family 2, subfamily W, member 1	CCDS4656.1	chr6_29012422-29012422_G_T	177F>L	Substitution	Nonsynonymous coding	32%
MM04T	OR2W5	olfactory receptor, family 2, subfamily W, member 5	NM_001004698	chr1_247654969-247654969_G_T	180E>D	Substitution	Nonsynonymous coding	20%
MM04T	OR2Y1	olfactory receptor, family 2, subfamily Y, member 1	CCDS34323.1	chr5_180167052-180167052_T_G	3S>R	Substitution	Nonsynonymous coding	33%
MM04T	OR3A1	olfactory receptor, family 3, subfamily A, member 1	CCDS11023.1	chr17_3195618-3195618_G_A	87R>C	Substitution	Nonsynonymous coding	33%
MM04T	OR4A15	olfactory receptor, family 4, subfamily A, member 15	CCDS31500.1	chr11_55135537-55135537_T_G	60L>V	Substitution	Nonsynonymous coding	33%
MM04T	OR4A15	olfactory receptor, family 4, subfamily A, member 15	CCDS31500.1	chr11_55135924-55135924_C_T	189L>F	Substitution	Nonsynonymous coding	20%
MM04T	OR4C3	olfactory receptor, family 4, subfamily C, member 3	CCDS31489.1	chr11_48347310-48347310_C_T	273A>V	Substitution	Nonsynonymous coding	11%
MM04T	OR4C5	olfactory receptor, family 4, subfamily C, member 5	ENST00000319813	chr11_48387495-48387495_G_T	175L>I	Substitution	Nonsynonymous coding	21%
MM04T	OR4C6	olfactory receptor, family 4, subfamily C, member 6	CCDS31506.1	chr11_55433463-55433463_C_A	274S>X	Substitution	Nonsense	35%
MM04T	OR4D11	olfactory receptor, family 4, subfamily D, member 11	CCDS31563.1	chr11_59271968-59271968_C_A	307P>H	Substitution	Nonsynonymous coding	20%
MM04T	OR4K13	olfactory receptor, family 4, subfamily K, member 13	CCDS32028.1	chr14_20502003-20502003_T_G	305*>Y	Substitution	Nonsynonymous coding	27%
MM04T	OR4M2	olfactory receptor, family 4, subfamily M, member 2	CCDS32172.1	chr15_22369507-22369507_A_C	311K>T	Substitution	Nonsynonymous coding	11%
MM04T	OR4N5	olfactory receptor, family 4, subfamily N, member 5	CCDS32031.1	chr14_20612709-20612709_C_A	272S>Y	Substitution	Nonsynonymous coding	26%
MM04T	OR4Q3	olfactory receptor, family 4, subfamily Q, member 3	CCDS32020.1	chr14_20215840-20215840_A_G	85D>G	Substitution	Nonsynonymous coding	35%
MM04T	OR51B5	olfactory receptor, family 51, subfamily B, member 5	CCDS31378.1	chr11_5364553-5364553_C_A	68D>Y	Substitution	Nonsynonymous coding	20%
MM04T	OR51G1	olfactory receptor, family 51, subfamily G, member 1	CCDS31366.1	chr11_4944776-4944776_C_T	265R>H	Substitution	Nonsynonymous coding	29%

MM04T	OR51G1	olfactory receptor, family 51, subfamily G, member 1	CCDS31366.1	chr11_4945104-4945104_G_T	156L>I	Substitution	Nonsynonymous coding	24%
MM04T	OR51J1	olfactory receptor, family 51, subfamily J, member 1 (gene/pseudogene)	ENST00000332043	chr11_5424719-5424719_C_T	298T>I	Substitution	Nonsynonymous coding	24%
MM04T	OR51S1	olfactory receptor, family 51, subfamily S, member 1	CCDS31362.1	chr11_4869964-4869964_G_A	159R>X	Substitution	Nonsense	36%
MM04T	OR51T1	olfactory receptor, family 51, subfamily T, member 1	CCDS31363.1	chr11_4903053-4903053_C_T	2T>I	Substitution	Nonsynonymous coding	33%
MM04T	OR52A5	olfactory receptor, family 52, subfamily A, member 5	CCDS31373.1	chr11_5153355-5153355_C_A	173R>L	Substitution	Nonsynonymous coding	22%
MM04T	OR52B4	olfactory receptor, family 52, subfamily B, member 4	CCDS41609.1	chr11_4389363-4389363_G_A	55R>C	Substitution	Nonsynonymous coding	33%
MM04T	OR52D1	olfactory receptor, family 52, subfamily D, member 1	CCDS31384.1	chr11_5510109-5510109_T_G	58L>R	Substitution	Nonsynonymous coding	31%
MM04T	OR52E8	olfactory receptor, family 52, subfamily E, member 8	CCDS31400.1	chr11_5878464-5878464_T_C	157R>G	Substitution	Nonsynonymous coding	28%
MM04T	OR52H1	olfactory receptor, family 52, subfamily H, member 1	CCDS31386.1	chr11_5565829-5565829_C_A	309D>Y	Substitution	Nonsynonymous coding	14%
MM04T	OR52I1	olfactory receptor, family 52, subfamily I, member 1	ENST00000450052	chr11_4615383-4615383_T_C	64Y>H	Substitution	Nonsynonymous coding	28%
MM04T	OR52K3P	olfactory receptor, family 52, subfamily K, member 3 pseudogene	ENST00000416879	chr11_4496780-4496780_C_A	31A>D	Substitution	Nonsynonymous coding	25%
MM04T	OR52N1	olfactory receptor, family 52, subfamily N, member 1	CCDS31398.1	chr11_5809377-5809377_G_T	224L>I	Substitution	Nonsynonymous coding	29%
MM04T	OR52N2	olfactory receptor, family 52, subfamily N, member 2	CCDS31399.1	chr11_5842124-5842124_G_A	187A>T	Substitution	Nonsynonymous coding	30%
MM04T	OR56A1	olfactory receptor, family 56, subfamily A, member 1	CCDS31405.1	chr11_6048170-6048170_A_C	255I>M	Substitution	Nonsynonymous coding	32%
MM04T	OR5C1	olfactory receptor, family 5, subfamily C, member 1	CCDS35131.1	chr9_125551770-125551770_C_T	187P>S	Substitution	Nonsynonymous coding	32%
MM04T	OR5D14	olfactory receptor, family 5, subfamily D, member 14	CCDS31508.1	chr11_55563147-55563147_T_G	39I>S	Substitution	Nonsynonymous coding	23%
MM04T	OR5D16	olfactory receptor, family 5, subfamily D, member 16	CCDS31512.1	chr11_55607198-55607198_T_G	324F>C	Substitution	Nonsynonymous coding	33%
MM04T	OR5H2	olfactory receptor, family 5, subfamily H, member 2	CCDS33801.1	chr3_98001970-98001970_C_T	80S>F	Substitution	Nonsynonymous coding	36%
MM04T	OR5H2	olfactory receptor, family 5, subfamily H, member 2	CCDS33801.1	chr3_98002350-98002350_T_G	207L>V	Substitution	Nonsynonymous coding	30%
MM04T	OR5K1	olfactory receptor, family 5, subfamily K, member 1	CCDS43115.1	chr3_98189280-98189280_C_A	287P>H	Substitution	Nonsynonymous coding	38%
MM04T	OR5K2	olfactory receptor, family 5, subfamily K, member 2	CCDS33804.1	chr3_98217174-98217174_C_A	217S>Y	Substitution	Nonsynonymous coding	19%
MM04T	OR5K3	olfactory receptor, family 5, subfamily K, member 3	CCDS33803.1	chr3_98110127-98110127_T_G	206I>M	Substitution	Nonsynonymous coding	39%
MM04T	OR5M11	olfactory receptor, family 5, subfamily M, member 11	NM_001005245	chr11_56310406-56310406_C_A	110E>X	Substitution	Nonsense	18%
MM04T	OR5T1	olfactory receptor, family 5, subfamily T, member 1	CCDS31525.1	chr11_56043272-56043272_G_A	53G>D	Substitution	Nonsynonymous coding	30%
MM04T	OR5T2	olfactory receptor, family 5, subfamily T, member 2	CCDS31523.1	chr11_55999864-55999864_C_A	266K>N	Substitution	Nonsynonymous coding	21%
MM04T	OR5T3	olfactory receptor, family 5, subfamily T, member 3	CCDS31524.1	chr11_56020307-56020307_T_C	211M>T	Substitution	Nonsynonymous coding	29%
MM04T	OR5W2	olfactory receptor, family 5, subfamily W, member 2	CCDS31513.1	chr11_55681928-55681928_C_T	44G>E	Substitution	Nonsynonymous coding	26%
MM04T	OR6B1	olfactory receptor, family 6, subfamily B, member 1	CCDS43667.1	chr7_143701101-143701101_G_T	4E>D	Substitution	Nonsynonymous coding	22%
MM04T	OR6B2	olfactory receptor, family 6, subfamily B, member 2	CCDS46559.1	chr2_240968930-240968930_C_T	306G>D	Substitution	Nonsynonymous coding	24%
MM04T	OR6C1	olfactory receptor, family 6, subfamily C, member 1	CCDS31818.1	chr12_55715066-55715066_C_A	228S>Y	Substitution	Nonsynonymous coding	30%
MM04T	OR6C4	olfactory receptor, family 6, subfamily C, member 4	CCDS31827.1	chr12_55945308-55945308_T_A	100F>I	Substitution	Nonsynonymous coding	23%
MM04T	OR6C74	olfactory receptor, family 6, subfamily C, member 74	CCDS31816.1	chr12_55641774-55641774_G_T	235A>S	Substitution	Nonsynonymous coding	31%

MM04T	OR6C75	olfactory receptor, family 6, subfamily C, member 75	CCDS31820.1	chr12_55758982-55758982_C_A	30L>I	Substitution	Nonsynonymous coding	25%
MM04T	OR6C75	olfactory receptor, family 6, subfamily C, member 75	CCDS31820.1	chr12_55759820-55759820_C_A	309S>Y	Substitution	Nonsynonymous coding	21%
MM04T	OR6K2	olfactory receptor, family 6, subfamily K, member 2	CCDS30902.1	chr1_158670028-158670028_G_T	139L>I	Substitution	Nonsynonymous coding	35%
MM04T	OR6M1	olfactory receptor, family 6, subfamily M, member 1	CCDS31696.1	chr11_123676797-123676797_C_A	87E>D	Substitution	Nonsynonymous coding	30%
MM04T	OR6N2	olfactory receptor, family 6, subfamily N, member 2	CCDS30906.1	chr1_158747097-158747097_G_T	110S>Y	Substitution	Nonsynonymous coding	32%
MM04T	OR6S1	olfactory receptor, family 6, subfamily S, member 1	CCDS32038.1	chr14_21109625-21109625_T_C	76T>A	Substitution	Nonsynonymous coding	33%
MM04T	OR6V1	olfactory receptor, family 6, subfamily V, member 1	CCDS47728.1	chr7_142749540-142749540_C_A	35L>I	Substitution	Nonsynonymous coding	32%
MM04T	OR7D4	olfactory receptor, family 7, subfamily D, member 4	CCDS32901.1	chr19_9324983-9324983_G_T	177F>L	Substitution	Nonsynonymous coding	18%
MM04T	OR8F1P	olfactory receptor, family 8, subfamily F, member 1 pseudogene	ENST00000318460	chr11_124078772-124078772_G_T	253D>Y	Substitution	Nonsynonymous coding	33%
MM04T	OR8G1	olfactory receptor, family 8, subfamily G, member 1	ENST00000341493	chr11_124120956-124120956_C_A	178F>L	Substitution	Nonsynonymous coding	37%
MM04T	OR8G1	olfactory receptor, family 8, subfamily G, member 1	ENST00000341493	chr11_124121015-124121015_T_G	198L>R	Substitution	Nonsynonymous coding	34%
MM04T	OR8U1	olfactory receptor, family 8, subfamily U, member 1	CCDS41647.1	chr11_56143446-56143446_C_A	116A>D	Substitution	Nonsynonymous coding	21%
MM04T	OR9I1	olfactory receptor, family 9, subfamily I, member 1	CCDS31542.1	chr11_57886203-57886203_G_T	238F>L	Substitution	Nonsynonymous coding	38%
MM04T	OSBPL10	oxysterol binding protein-like 10	CCDS2651.1	chr3_31921248-31921248_C_T	119R>Q	Substitution	Nonsynonymous coding	31%
MM04T	OSBPL11	oxysterol binding protein-like 11	CCDS3033.1	chr3_125286396-125286396_C_T	237R>Q	Substitution	Nonsynonymous coding	28%
MM04T	OSBPL1A	oxysterol binding protein-like 1A	CCDS11884.1	chr18_21759703-21759703_G_A	637R>X	Substitution	Nonsense	23%
MM04T	OSBPL6	oxysterol binding protein-like 6	CCDS2278.1	chr2_179238741-179238741_C_T	511S>L	Substitution	Nonsynonymous coding	42%
MM04T	OSBPL8	oxysterol binding protein-like 8	CCDS31862.1	chr12_76765242-76765242_A_C	680D>E	Substitution	Nonsynonymous coding	29%
MM04T	OSGEP	O-sialoglycoprotein endopeptidase	CCDS9549.1	chr14_20917350-20917350_G_A	163R>C	Substitution	Nonsynonymous coding	28%
MM04T	OSGIN1	oxidative stress induced growth inhibitor 1	CCDS10938.1	chr16_83998674-83998674_C_T	249R>C	Substitution	Nonsynonymous coding	27%
MM04T	OTOA	otoancorin	CCDS10600.2	chr16_21693112-21693112_C_T	78S>F	Substitution	Nonsynonymous coding	32%
MM04T	OTOA	otoancorin	CCDS10600.2	chr16_21693118-21693118_A_G	80N>S	Substitution	Nonsynonymous coding	33%
MM04T	OTOA	otoancorin	CCDS10600.2	chr16_21728281-21728281_C_A	514F>L	Substitution	Nonsynonymous coding	26%
MM04T	OTOG	otogelin	ENST00000399391	chr11_17577421-17577421_G_T	224K>N	Substitution	Nonsynonymous coding	22%
MM04T	OTOG	otogelin	ENST00000399391	chr11_17650784-17650784_G_T	2223Q>H	Substitution	Nonsynonymous coding	28%
MM04T	OTOG	otogelin	ENST00000399391	chr11_17656626-17656626_G_T	NA	Substitution	Splice site acceptor	13%
MM04T	OTOGL	otogelin-like	ENST00000458043	chr12_80732934-80732934_T_C	3V>A	Substitution	Nonsynonymous coding	24%
MM04T	OTOGL	otogelin-like	ENST00000458043	chr12_80749626-80749626_G_A	258E>K	Substitution	Nonsynonymous coding	32%
MM04T	OTUD3	OTU domain containing 3	CCDS41279.1	chr1_20232935-20232935_G_T	282E>D	Substitution	Nonsynonymous coding	30%
MM04T	OTUD4	OTU domain containing 4	CCDS47139.1	chr4_146072045-146072045_C_A	262K>N	Substitution	Nonsynonymous coding	23%
MM04T	OTUD4	OTU domain containing 4	CCDS47139.1	chr4_146077114-146077114_C_T	157G>R	Substitution	Nonsynonymous coding	18%
MM04T	OTUD4	OTU domain containing 4	CCDS47139.1	chr4_146095825-146095825_C_A	12E>D	Substitution	Nonsynonymous coding	32%

MM04T	OTUD7B	OTU domain containing 7B	CCDS41389.1	chr1_149936181-149936181_C_T	233R>H	Substitution	Nonsynonymous coding	30%
MM04T	OVGP1	oviductal glycoprotein 1, 120kDa	CCDS834.1	chr1_111965561-111965561_C_T	199R>H	Substitution	Nonsynonymous coding	29%
MM04T	OXCT1	3-oxoacid CoA transferase 1	CCDS3937.1	chr5_41861444-41861444_C_A	84G>X	Substitution	Nonsense	21%
MM04T	OXCT2	3-oxoacid CoA transferase 2	CCDS445.1	chr1_40236336-40236336_G_T	198L>I	Substitution	Nonsynonymous coding	21%
MM04T	P2RX1	purinergic receptor P2X, ligand-gated ion channel, 1	CCDS11040.1	chr17_3819446-3819446_C_T	25R>H	Substitution	Nonsynonymous coding	33%
MM04T	P2RX7	purinergic receptor P2X, ligand-gated ion channel, 7	CCDS9213.1	chr12_121622580-121622580_A_G	588Y>C	Substitution	Nonsynonymous coding	47%
MM04T	P2RY10	purinergic receptor P2Y, G-protein coupled, 10	CCDS14442.1	chrX_78216286-78216286_G_A	90R>Q	Substitution	Nonsynonymous coding	22%
MM04T	P2RY10	purinergic receptor P2Y, G-protein coupled, 10	CCDS14442.1	chrX_78216809-78216809_T_G	264I>M	Substitution	Nonsynonymous coding	29%
MM04T	P2RY10	purinergic receptor P2Y, G-protein coupled, 10	CCDS14442.1	chrX_78216997-78216997_G_A	327R>H	Substitution	Nonsynonymous coding	25%
MM04T	P2RY13	purinergic receptor P2Y, G-protein coupled, 13	CCDS3158.2	chr3_151045787-151045787_A_C	353L>V	Substitution	Nonsynonymous coding	37%
MM04T	P2RY4	pyrimidinergic receptor P2Y, G-protein coupled, 4	CCDS14398.1	chrX_69479323-69479323_C_T	51G>D	Substitution	Nonsynonymous coding	36%
MM04T	PABPC3	poly(A) binding protein, cytoplasmic 3	CCDS9311.1	chr13_25670617-25670617_G_A	94R>Q	Substitution	Nonsynonymous coding	26%
MM04T	PAG1	phosphoprotein associated with glycosphingolipid microdomains 1	CCDS6227.1	chr8_81889006-81889006_G_T	358L>I	Substitution	Nonsynonymous coding	25%
MM04T	PAG1	phosphoprotein associated with glycosphingolipid microdomains 1	CCDS6227.1	chr8_81899700-81899700_G_A	60P>L	Substitution	Nonsynonymous coding	20%
MM04T	PAGE3	P antigen family, member 3 (prostate associated)	CCDS35307.1	chrX_55290320-55290320_C_A	6R>I	Substitution	Nonsynonymous coding	45%
MM04T	PAK7	p21 protein (Cdc42/Rac)-activated kinase 7	CCDS13107.1	chr20_9561331-9561331_G_T	151L>I	Substitution	Nonsynonymous coding	14%
MM04T	PALB2	partner and localizer of BRCA2	CCDS32406.1	chr16_23646440-23646440_C_A	476R>I	Substitution	Nonsynonymous coding	24%
MM04T	PALM2-AKAP2	PALM2-AKAP2 readthrough	CCDS35100.1	chr9_112900331-112900331_C_T	836A>V	Substitution	Nonsynonymous coding	19%
MM04T	PALM2-AKAP2	PALM2-AKAP2 readthrough	CCDS35100.1	chr9_112918757-112918757_G_A	1052E>K	Substitution	Nonsynonymous coding	35%
MM04T	PAMR1	peptidase domain containing associated with muscle regeneration 1	CCDS7898.1	chr11_35461197-35461197_G_T	377L>I	Substitution	Nonsynonymous coding	11%
MM04T	PAPD4	PAP associated domain containing 4	CCDS4048.1	chr5_78919307-78919307_G_T	154D>Y	Substitution	Nonsynonymous coding	26%
MM04T	PAPLN	papilin, proteoglycan-like sulfated glycoprotein	CCDS32114.1	chr14_73726043-73726043_G_A	565R>Q	Substitution	Nonsynonymous coding	21%
MM04T	PAPLN	papilin, proteoglycan-like sulfated glycoprotein	CCDS32114.1	chr14_73729508-73729508_C_T	872A>V	Substitution	Nonsynonymous coding	25%
MM04T	PAPLN	papilin, proteoglycan-like sulfated glycoprotein	CCDS32114.1	chr14_73731024-73731024_G_T	962K>N	Substitution	Nonsynonymous coding	20%
MM04T	PAPLN	papilin, proteoglycan-like sulfated glycoprotein	CCDS32114.1	chr14_73731338-73731338_T_G	983F>C	Substitution	Nonsynonymous coding	29%
MM04T	PAPOLA	poly(A) polymerase alpha	CCDS9946.1	chr14_96997889-96997889_A_C	191K>N	Substitution	Nonsynonymous coding	34%
MM04T	PAPOLA	poly(A) polymerase alpha	CCDS9946.1	chr14_96998962-96998962_A_C	271K>T	Substitution	Nonsynonymous coding	41%
MM04T	PAPOLA	poly(A) polymerase alpha	CCDS9946.1	chr14_97009137-97009137_G_A	399R>Q	Substitution	Nonsynonymous coding	40%
MM04T	PAPOLB	poly(A) polymerase beta (testis specific)	NM_020144	chr7_4899813-4899813_C_T	543M>I	Substitution	Nonsynonymous coding	31%
MM04T	PAPOLB	poly(A) polymerase beta (testis specific)	NM_020144	chr7_4900274-4900274_C_T	390E>K	Substitution	Nonsynonymous coding	32%
MM04T	PAPOLB	poly(A) polymerase beta (testis specific)	NM_020144	chr7_4900623-4900623_G_T	273F>L	Substitution	Nonsynonymous coding	20%
MM04T	PAPOLB	poly(A) polymerase beta (testis specific)	NM_020144	chr7_4900859-4900859_C_A	195D>Y	Substitution	Nonsynonymous coding	21%

MM04T	PAPOLB	poly(A) polymerase beta (testis specific)	NM_020144	chr7_4901340-4901340_C_A	34E>D	Substitution	Nonsynonymous coding	37%
MM04T	PAPPA	pregnancy-associated plasma protein A, pappalysin 1	CCDS6813.1	chr9_119033623-119033623_G_A	961D>N	Substitution	Nonsynonymous coding	22%
MM04T	PAPPA	pregnancy-associated plasma protein A, pappalysin 1	CCDS6813.1	chr9_119115026-119115026_T_C	1336S>P	Substitution	Nonsynonymous coding	31%
MM04T	PAPPA2	pappalysin 2	CCDS41438.1	chr1_176738873-176738873_T_G	1485F>C	Substitution	Nonsynonymous coding	38%
MM04T	PAPSS2	3'-phosphoadenosine 5'-phosphosulfate synthase 2	CCDS44453.1	chr10_89501057-89501057_G_T	384W>C	Substitution	Nonsynonymous coding	28%
MM04T	PAQR7	progestin and adipoQ receptor family member VII	CCDS267.1	chr1_26189844-26189844_C_T	163E>K	Substitution	Nonsynonymous coding	22%
MM04T	PARD3	par-3 partitioning defective 3 homolog (C. elegans)	CCDS7178.1	chr10_34626229-34626229_C_T	848R>Q	Substitution	Nonsynonymous coding	38%
MM04T	PARD3B	par-3 partitioning defective 3 homolog B (C. elegans)	CCDS42806.1	chr2_206480337-206480337_C_T	1078P>S	Substitution	Nonsynonymous coding	18%
MM04T	PARD6B	par-6 partitioning defective 6 homolog beta (C. elegans)	CCDS33485.1	chr20_49366780-49366780_G_A	292E>K	Substitution	Nonsynonymous coding	30%
MM04T	PARL	presenilin associated, rhomboid-like	CCDS3248.1	chr3_183584427-183584427_C_A	150D>Y	Substitution	Nonsynonymous coding	27%
MM04T	PARN	poly(A)-specific ribonuclease	CCDS45419.1	chr16_14530620-14530620_G_A	625S>L	Substitution	Nonsynonymous coding	20%
MM04T	PARP11	poly (ADP-ribose) polymerase family, member 11	CCDS8523.2	chr12_3938154-3938154_C_T	64E>K	Substitution	Nonsynonymous coding	21%
MM04T	PARP12	poly (ADP-ribose) polymerase family, member 12	CCDS5857.1	chr7_139737542-139737542_T_G	433K>Q	Substitution	Nonsynonymous coding	17%
MM04T	PARP14	poly (ADP-ribose) polymerase family, member 14	CCDS46894.1	chr3_122420126-122420126_G_A	909E>K	Substitution	Nonsynonymous coding	27%
MM04T	PARP14	poly (ADP-ribose) polymerase family, member 14	CCDS46894.1	chr3_122437275-122437275_C_A	1426S>X	Substitution	Nonsense	31%
MM04T	PARP15	poly (ADP-ribose) polymerase family, member 15	CCDS46893.1	chr3_122335871-122335871_G_T	287G>V	Substitution	Nonsynonymous coding	16%
MM04T	PARP15	poly (ADP-ribose) polymerase family, member 15	CCDS46893.1	chr3_122340438-122340438_C_A	404S>Y	Substitution	Nonsynonymous coding	26%
MM04T	PARP16	poly (ADP-ribose) polymerase family, member 16	CCDS10204.1	chr15_65551837-65551837_C_T	294V>I	Substitution	Nonsynonymous coding	22%
MM04T	PARP2	poly (ADP-ribose) polymerase 2	CCDS41910.1	chr14_20813166-20813166_G_T	41R>I	Substitution	Nonsynonymous coding	21%
MM04T	PARVA	parvin, alpha	CCDS44541.1	chr11_12518069-12518069_A_C	155Q>H	Substitution	Nonsynonymous coding	38%
MM04T	PASD1	PAS domain containing 1	CCDS35431.1	chrX_150828252-150828252_C_A	262S>Y	Substitution	Nonsynonymous coding	27%
MM04T	PASD1	PAS domain containing 1	CCDS35431.1	chrX_150832727-150832727_G_T	326E>D	Substitution	Nonsynonymous coding	18%
MM04T	PASK	PAS domain containing serine/threonine kinase	CCDS2545.1	chr2_242066688-242066688_C_A	548D>Y	Substitution	Nonsynonymous coding	29%
MM04T	PATZ1	POZ (BTB) and AT hook containing zinc finger 1	CCDS13894.1	chr22_31731824-31731824_C_T	454R>Q	Substitution	Nonsynonymous coding	30%
MM04T	PAX7	paired box 7	CCDS186.1	chr1_18962743-18962743_C_T	155S>L	Substitution	Nonsynonymous coding	26%
MM04T	PAXBP1	PAX3 and PAX7 binding protein 1	CCDS13619.1	chr21_34132284-34132284_C_A	333E>X	Substitution	Nonsense	24%
MM04T	PBRM1	polybromo 1	CCDS2859.1	chr3_52661328-52661328_G_T	469S>Y	Substitution	Nonsynonymous coding	21%
MM04T	PBX1	pre-B-cell leukemia homeobox 1	CCDS1246.1	chr1_164769105-164769105_G_A	227R>H	Substitution	Nonsynonymous coding	14%
MM04T	PBX1	pre-B-cell leukemia homeobox 1	CCDS1246.1	chr1_164532474-164532474_G_T	NA	Substitution	Splice site acceptor	22%
MM04T	PBX3	pre-B-cell leukemia homeobox 3	CCDS6865.1	chr9_128692127-128692127_A_G	NA	Substitution	Splice site donor	31%
MM04T	PCBP1	poly(rC) binding protein 1	CCDS1898.1	chr2_70315136-70315136_G_A	87M>I	Substitution	Nonsynonymous coding	23%
MM04T	PCBP1	poly(rC) binding protein 1	CCDS1898.1	chr2_70315660-70315660_C_T	262S>F	Substitution	Nonsynonymous coding	23%

MM04T	PCDH1	protocadherin 1	CCDS4267.1	chr5_141236997-141236997_C_T	1047A>T	Substitution	Nonsynonymous coding	27%
MM04T	PCDH1	protocadherin 1	CCDS4267.1	chr5_141243872-141243872_C_T	675R>Q	Substitution	Nonsynonymous coding	33%
MM04T	PCDH1	protocadherin 1	CCDS4267.1	chr5_141244631-141244631_C_T	422R>Q	Substitution	Nonsynonymous coding	21%
MM04T	PCDH10	protocadherin 10	CCDS34063.1	chr4_134076104-134076104_C_T	908S>F	Substitution	Nonsynonymous coding	27%
MM04T	PCDH15	protocadherin-related 15	CCDS44404.1	chr10_55581920-55581920_C_T	1858E>K	Substitution	Nonsynonymous coding	27%
MM04T	PCDH15	protocadherin-related 15	CCDS44404.1	chr10_55582522-55582522_G_T	1657S>Y	Substitution	Nonsynonymous coding	23%
MM04T	PCDH15	protocadherin-related 15	CCDS44404.1	chr10_55721637-55721637_G_A	962R>C	Substitution	Nonsynonymous coding	34%
MM04T	PCDH15	protocadherin-related 15	CCDS44404.1	chr10_56138539-56138539_A_C	NA	Substitution	Splice site donor	33%
MM04T	PCDH15	protocadherin-related 15	CCDS44404.1	chr10_55955586-55955586_C_A	388E>X	Substitution	Nonsense	24%
MM04T	PCDH18	protocadherin 18	CCDS34064.1	chr4_138452153-138452153_C_A	364E>X	Substitution	Nonsense	40%
MM04T	PCDH19	protocadherin 19	CCDS43976.1	chrX_99661554-99661554_A_G	681F>S	Substitution	Nonsynonymous coding	15%
MM04T	PCDH20	protocadherin 20	CCDS9442.2	chr13_61986429-61986429_C_A	601E>D	Substitution	Nonsynonymous coding	33%
MM04T	PCDH20	protocadherin 20	CCDS9442.2	chr13_61987750-61987750_G_A	161A>V	Substitution	Nonsynonymous coding	33%
MM04T	PCDH9	protocadherin 9	CCDS9444.1	chr13_67477703-67477703_A_G	1024V>A	Substitution	Nonsynonymous coding	27%
MM04T	PCDH9	protocadherin 9	CCDS9444.1	chr13_67800886-67800886_G_T	563L>M	Substitution	Nonsynonymous coding	35%
MM04T	PCDH9	protocadherin 9	CCDS9444.1	chr13_67802500-67802500_C_A	25E>X	Substitution	Nonsense	36%
MM04T	PCDHA6	protocadherin alpha 6	CCDS4241.1	chr5_140307263-140307263_A_C	262E>D	Substitution	Nonsynonymous coding	36%
MM04T	PCDHA6	protocadherin alpha 6	CCDS47281.1	chr5_140208487-140208487_G_T	271E>X	Substitution	Nonsense	25%
MM04T	PCDHA8	protocadherin alpha 8	CCDS34253.1	chr5_140221633-140221633_G_A	243E>K	Substitution	Nonsynonymous coding	38%
MM04T	PCDHB10	protocadherin beta 10	CCDS4252.1	chr5_140572744-140572744_G_A	207E>K	Substitution	Nonsynonymous coding	23%
MM04T	PCDHB12	protocadherin beta 12	CCDS4254.1	chr5_140589590-140589590_C_T	371R>X	Substitution	Nonsense	28%
MM04T	PCDHB16	protocadherin beta 16	CCDS4251.1	chr5_140562262-140562262_C_A	43S>Y	Substitution	Nonsynonymous coding	42%
MM04T	PCDHB3	protocadherin beta 3	CCDS4245.1	chr5_140480573-140480573_T_G	114L>V	Substitution	Nonsynonymous coding	37%
MM04T	PCDHB4	protocadherin beta 4	CCDS4246.1	chr5_140502262-140502262_C_T	228R>X	Substitution	Nonsense	31%
MM04T	PCDHB4	protocadherin beta 4	CCDS4246.1	chr5_140502688-140502688_C_T	370R>X	Substitution	Nonsense	33%
MM04T	PCDHB5	protocadherin beta 5	CCDS4247.1	chr5_140515911-140515911_G_A	299E>K	Substitution	Nonsynonymous coding	34%
MM04T	PCDHB5	protocadherin beta 5	CCDS4247.1	chr5_140516154-140516154_A_G	380M>V	Substitution	Nonsynonymous coding	27%
MM04T	PCDHB6	protocadherin beta 6	CCDS4248.1	chr5_140530293-140530293_C_A	152P>H	Substitution	Nonsynonymous coding	31%
MM04T	PCDHB6	protocadherin beta 6	CCDS4248.1	chr5_140530394-140530394_G_A	186E>K	Substitution	Nonsynonymous coding	32%
MM04T	PCDHB7	protocadherin beta 7	CCDS4249.1	chr5_140552873-140552873_C_A	153L>I	Substitution	Nonsynonymous coding	33%
MM04T	PCDHGA1	protocadherin gamma subfamily A, 1	CCDS34256.1	chr5_140710873-140710873_C_A	208H>N	Substitution	Nonsynonymous coding	31%
MM04T	PCDHGA1	protocadherin gamma subfamily A, 1	CCDS34256.1	chr5_140712719-140712719_C_T	823S>F	Substitution	Nonsynonymous coding	37%

MM04T	PCDHGA2	protocadherin gamma subfamily A, 2	CCDS47289.1	chr5_140719944-140719944_C_A	469A>D	Substitution	Nonsynonymous coding	24%
MM04T	PCDHGA5	protocadherin gamma subfamily A, 5	NM_018918	chr5_140744334-140744334_C_T	146A>V	Substitution	Nonsynonymous coding	40%
MM04T	PCDHGA6	protocadherin gamma subfamily A, 6	NM_018919	chr5_140753958-140753958_T_G	103L>R	Substitution	Nonsynonymous coding	30%
MM04T	PCDHGA7	protocadherin gamma subfamily A, 7	NM_018920	chr5_140763408-140763408_C_A	314F>L	Substitution	Nonsynonymous coding	34%
MM04T	PCDHGA8	protocadherin gamma subfamily A, 8	CCDS47291.1	chr5_140773387-140773387_C_T	336S>L	Substitution	Nonsynonymous coding	33%
MM04T	PCDHGA8	protocadherin gamma subfamily A, 8	CCDS47291.1	chr5_140773459-140773459_C_T	360S>F	Substitution	Nonsynonymous coding	29%
MM04T	PCDHGA9	protocadherin gamma subfamily A, 9	NM_018921	chr5_140783768-140783768_G_T	417E>X	Substitution	Nonsense	26%
MM04T	PCDHGB1	protocadherin gamma subfamily B, 1	NM_018922	chr5_140730684-140730684_G_A	286S>N	Substitution	Nonsynonymous coding	30%
MM04T	PCF11	PCF11, cleavage and polyadenylation factor subunit, homolog (S. cerevisiae)	CCDS44689.1	chr11_82877688-82877688_G_T	583E>D	Substitution	Nonsynonymous coding	23%
MM04T	PCF11	PCF11, cleavage and polyadenylation factor subunit, homolog (S. cerevisiae)	CCDS44689.1	chr11_82878277-82878277_G_A	643R>Q	Substitution	Nonsynonymous coding	38%
MM04T	PCGF6	polycomb group ring finger 6	CCDS31275.1	chr10_105073971-105073971_C_T	323R>Q	Substitution	Nonsynonymous coding	46%
MM04T	PCID2	PCI domain containing 2	CCDS9532.2	chr13_113838710-113838710_T_C	212Y>C	Substitution	Nonsynonymous coding	29%
MM04T	PCID2	PCI domain containing 2	CCDS9532.2	chr13_113854748-113854748_C_T	40R>Q	Substitution	Nonsynonymous coding	25%
MM04T	PCK1	phosphoenolpyruvate carboxykinase 1 (soluble)	CCDS13460.1	chr20_56139310-56139310_G_T	349K>N	Substitution	Nonsynonymous coding	22%
MM04T	PCK2	phosphoenolpyruvate carboxykinase 2 (mitochondrial)	CCDS9609.1	chr14_24566148-24566148_G_A	26S>N	Substitution	Nonsynonymous coding	34%
MM04T	PCLO	piccolo presynaptic cytomatrix protein	CCDS47630.1	chr7_82579090-82579090_C_T	3605R>Q	Substitution	Nonsynonymous coding	32%
MM04T	PCLO	piccolo presynaptic cytomatrix protein	CCDS47630.1	chr7_82580431-82580431_C_T	3158G>D	Substitution	Nonsynonymous coding	23%
MM04T	PCLO	piccolo presynaptic cytomatrix protein	CCDS47630.1	chr7_82581462-82581462_C_A	2936R>I	Substitution	Nonsynonymous coding	28%
MM04T	PCLO	piccolo presynaptic cytomatrix protein	CCDS47630.1	chr7_82583362-82583362_T_C	2303K>E	Substitution	Nonsynonymous coding	29%
MM04T	PCLO	piccolo presynaptic cytomatrix protein	CCDS47630.1	chr7_82585937-82585937_T_G	1444E>D	Substitution	Nonsynonymous coding	29%
MM04T	PCLO	piccolo presynaptic cytomatrix protein	CCDS47630.1	chr7_82763915-82763915_C_T	984G>D	Substitution	Nonsynonymous coding	28%
MM04T	PCLO	piccolo presynaptic cytomatrix protein	CCDS47630.1	chr7_82764966-82764966_C_A	634E>X	Substitution	Nonsense	21%
MM04T	PCM1	pericentriolar material 1	CCDS47812.1	chr8_17817827-17817827_G_T	732E>D	Substitution	Nonsynonymous coding	32%
MM04T	PCM1	pericentriolar material 1	CCDS47812.1	chr8_17867210-17867210_G_T	1706R>M	Substitution	Nonsynonymous coding	30%
MM04T	PCMTD1	protein-L-isoaspartate (D-aspartate) O-methyltransferase domain containing 1	CCDS6148.1	chr8_52733230-52733230_C_T	252R>Q	Substitution	Nonsynonymous coding	18%
MM04T	PCNT	pericentrin	CCDS33592.1	chr21_47836119-47836119_C_A	2096S>Y	Substitution	Nonsynonymous coding	21%
MM04T	PCNT	pericentrin	CCDS33592.1	chr21_47819606-47819606_G_T	1563E>X	Substitution	Nonsense	24%
MM04T	PCNX	pecanex homolog (Drosophila)	CCDS9806.1	chr14_71576190-71576190_C_A	2256L>M	Substitution	Nonsynonymous coding	31%
MM04T	PCNXL4	pecanex-like 4 (Drosophila)	ENST00000404681	chr14_60574744-60574744_C_A	130L>I	Substitution	Nonsynonymous coding	32%
MM04T	PCOLCE2	procollagen C-endopeptidase enhancer 2	CCDS3127.1	chr3_142537266-142537266_G_A	387R>X	Substitution	Nonsense	22%
MM04T	PCSK4	proprotein convertase subtilisin/kexin type 4	CCDS12069.2	chr19_1482903-1482903_A_G	563F>S	Substitution	Nonsynonymous coding	14%
MM04T	PCYOX1L	prenylcysteine oxidase 1 like	CCDS4296.1	chr5_148745538-148745538_C_A	168F>L	Substitution	Nonsynonymous coding	23%

MM04T	PCYOX1L	prenylcysteine oxidase 1 like	CCDS4296.1	chr5_148746962-148746962_C_A	241S>Y	Substitution	Nonsynonymous coding	18%
MM04T	PDCD4	programmed cell death 4 (neoplastic transformation inhibitor)	CCDS7567.1	chr10_112650336-112650336_G_A	300V>M	Substitution	Nonsynonymous coding	24%
MM04T	PDCD4	programmed cell death 4 (neoplastic transformation inhibitor)	CCDS7567.1	chr10_112654200-112654200_G_T	377E>X	Substitution	Nonsense	34%
MM04T	PDDC1	Parkinson disease 7 domain containing 1	CCDS7713.1	chr11_774059-774059_C_A	66D>Y	Substitution	Nonsynonymous coding	27%
MM04T	PDE11A	phosphodiesterase 11A	CCDS33334.1	chr2_178545616-178545616_G_T	787F>L	Substitution	Nonsynonymous coding	17%
MM04T	PDE11A	phosphodiesterase 11A	CCDS33334.1	chr2_178545555-178545555_G_A	808R>X	Substitution	Nonsense	36%
MM04T	PDE1B	phosphodiesterase 1B, calmodulin-dependent	CCDS8882.1	chr12_54963341-54963341_G_T	141R>I	Substitution	Nonsynonymous coding	22%
MM04T	PDE1C	phosphodiesterase 1C, calmodulin-dependent 70kDa	CCDS5437.1	chr7_32091190-32091190_C_T	35R>K	Substitution	Nonsynonymous coding	11%
MM04T	PDE1C	phosphodiesterase 1C, calmodulin-dependent 70kDa	CCDS5437.1	chr7_31920454-31920454_G_A	50Q>X	Substitution	Nonsense	21%
MM04T	PDE2A	phosphodiesterase 2A, cGMP-stimulated	CCDS8216.1	chr11_72297120-72297120_C_A	392E>D	Substitution	Nonsynonymous coding	19%
MM04T	PDE4A	phosphodiesterase 4A, cAMP-specific	CCDS45961.1	chr19_10578223-10578223_G_A	863A>T	Substitution	Nonsynonymous coding	22%
MM04T	PDE6C	phosphodiesterase 6C, cGMP-specific, cone, alpha prime	CCDS7429.1	chr10_95418670-95418670_G_T	683M>I	Substitution	Nonsynonymous coding	26%
MM04T	PDE8B	phosphodiesterase 8B	CCDS4037.1	chr5_76700580-76700580_G_T	416E>X	Substitution	Nonsense	24%
MM04T	PDE9A	phosphodiesterase 9A	CCDS13690.1	chr21_44171250-44171250__T	NA	Insertion	Frameshift	17%
MM04T	PDE9A	phosphodiesterase 9A	CCDS13690.1	chr21_44185538-44185538_A_C	430E>D	Substitution	Nonsynonymous coding	34%
MM04T	PDGFRB	platelet-derived growth factor receptor, beta polypeptide	CCDS4303.1	chr5_149495372-149495372_G_A	1092S>L	Substitution	Nonsynonymous coding	33%
MM04T	PDGFRL	platelet-derived growth factor receptor-like	CCDS6003.1	chr8_17447089-17447089_C_A	56D>E	Substitution	Nonsynonymous coding	34%
MM04T	PDGFRL	platelet-derived growth factor receptor-like	CCDS6003.1	chr8_17447147-17447147_C_T	76R>C	Substitution	Nonsynonymous coding	41%
MM04T	PDHA1	pyruvate dehydrogenase (lipoamide) alpha 1	CCDS14192.1	chrX_19369513-19369513_G_A	136A>T	Substitution	Nonsynonymous coding	27%
MM04T	PDHA1	pyruvate dehydrogenase (lipoamide) alpha 1	CCDS14192.1	chrX_19373832-19373832_G_A	263R>Q	Substitution	Nonsynonymous coding	23%
MM04T	PDIA3	protein disulfide isomerase family A, member 3	CCDS10101.1	chr15_44063379-44063379_A_C	494K>T	Substitution	Nonsynonymous coding	32%
MM04T	PDILT	protein disulfide isomerase-like, testis expressed	CCDS10584.1	chr16_20370649-20370649_C_A	583E>X	Substitution	Nonsense	32%
MM04T	PDK4	pyruvate dehydrogenase kinase, isozyme 4	CCDS5643.1	chr7_95222231-95222231_G_A	124R>X	Substitution	Nonsense	29%
MM04T	PDLIM5	PDZ and LIM domain 5	CCDS3641.1	chr4_95444909-95444909_T_C	44V>A	Substitution	Nonsynonymous coding	19%
MM04T	PDPR	pyruvate dehydrogenase phosphatase regulatory subunit	CCDS45520.1	chr16_70166086-70166086_C_T	294R>W	Substitution	Nonsynonymous coding	19%
MM04T	PDS5A	PDS5, regulator of cohesion maintenance, homolog A (S. cerevisiae)	CCDS47045.1	chr4_39850493-39850493_T_G	1106K>T	Substitution	Nonsynonymous coding	32%
MM04T	PDSS1	prenyl (decaprenyl) diphosphate synthase, subunit 1	CCDS31168.1	chr10_27012996-27012996_T_G	259Y>D	Substitution	Nonsynonymous coding	28%
MM04T	PDZD2	PDZ domain containing 2	CCDS34137.1	chr5_32087876-32087876_C_A	1441S>Y	Substitution	Nonsynonymous coding	25%
MM04T	PDZD2	PDZ domain containing 2	CCDS34137.1	chr5_32090553-32090553_C_A	2333F>L	Substitution	Nonsynonymous coding	21%
MM04T	PDZD7	PDZ domain containing 7	ENST00000238965	chr10_102770123-102770123_C_A	202E>D	Substitution	Nonsynonymous coding	23%
MM04T	PDZD8	PDZ domain containing 8	CCDS7600.1	chr10_119049846-119049846_T_G	371K>T	Substitution	Nonsynonymous coding	22%
MM04T	PDZK1	PDZ domain containing 1	CCDS924.1	chr1_145747242-145747242_G_T	67E>X	Substitution	Nonsense	50%

MM04T	PDZRN4	PDZ domain containing ring finger 4	CCDS8739.1	chr12_41900312-41900312_C_T	42R>C	Substitution	Nonsynonymous coding	26%
MM04T	PDZRN4	PDZ domain containing ring finger 4	CCDS8739.1	chr12_41966493-41966493_C_T	380R>X	Substitution	Nonsense	31%
MM04T	PEAR1	platelet endothelial aggregation receptor 1	CCDS30892.1	chr1_156876621-156876621_G_A	198C>Y	Substitution	Nonsynonymous coding	29%
MM04T	PEAR1	platelet endothelial aggregation receptor 1	CCDS30892.1	chr1_156877492-156877492_G_T	245Q>H	Substitution	Nonsynonymous coding	22%
MM04T	PEG3	paternally expressed 3	CCDS33123.1	chr19_57286489-57286489_G_A	384S>F	Substitution	Nonsynonymous coding	28%
MM04T	PEG3	paternally expressed 3	CCDS33123.1	chr19_57286654-57286654_C_A	329R>I	Substitution	Nonsynonymous coding	22%
MM04T	PEG3	paternally expressed 3	CCDS12948.1	chr19_57327687-57327687_G_T	708S>Y	Substitution	Nonsynonymous coding	33%
MM04T	PEG3	paternally expressed 3	CCDS12948.1	chr19_57329140-57329140_C_A	279R>I	Substitution	Nonsynonymous coding	24%
MM04T	PEG3	paternally expressed 3	CCDS12948.1	chr19_57327880-57327880_C_A	644E>X	Substitution	Nonsense	20%
MM04T	PENK	proenkephalin	CCDS6168.1	chr8_57354237-57354237_G_A	133A>V	Substitution	Nonsynonymous coding	23%
MM04T	PER2	period circadian clock 2	CCDS2528.1	chr2_239174207-239174207_C_A	331R>I	Substitution	Nonsynonymous coding	29%
MM04T	PEX1	peroxisomal biogenesis factor 1	CCDS5627.1	chr7_92140328-92140328_G_T	506S>Y	Substitution	Nonsynonymous coding	15%
MM04T	PEX5L	peroxisomal biogenesis factor 5-like	CCDS3236.1	chr3_179593209-179593209_T_A	188T>S	Substitution	Nonsynonymous coding	19%
MM04T	PFKFB1	6-phosphofructo-2-kinase/fructose-2,6-biphosphatase 1	CCDS14364.1	chrX_54971885-54971885_T_C	362Y>C	Substitution	Nonsynonymous coding	20%
MM04T	PFKFB1	6-phosphofructo-2-kinase/fructose-2,6-biphosphatase 1	CCDS14364.1	chrX_54978349-54978349_G_A	279R>C	Substitution	Nonsynonymous coding	17%
MM04T	PFKFB3	6-phosphofructo-2-kinase/fructose-2,6-biphosphatase 3	CCDS7078.1	chr10_6259110-6259110_G_A	152E>K	Substitution	Nonsynonymous coding	20%
MM04T	PFKP	phosphofructokinase, platelet	CCDS7059.1	chr10_3178716-3178716_G_T	767S>I	Substitution	Nonsynonymous coding	20%
MM04T	PFN2	profilin 2	CCDS46934.1	chr3_149683989-149683989_C_T	130E>K	Substitution	Nonsynonymous coding	34%
MM04T	PFN3	profilin 3	CCDS34301.1	chr5_176827199-176827199_C_T	127E>K	Substitution	Nonsynonymous coding	20%
MM04T	PGBD1	piggyBac transposable element derived 1	CCDS4648.1	chr6_28268861-28268861_G_T	410L>F	Substitution	Nonsynonymous coding	33%
MM04T	PGBD1	piggyBac transposable element derived 1	CCDS4648.1	chr6_28269108-28269108_A_C	493I>L	Substitution	Nonsynonymous coding	38%
MM04T	PGBD1	piggyBac transposable element derived 1	CCDS4648.1	chr6_28269498-28269498_C_A	623H>N	Substitution	Nonsynonymous coding	30%
MM04T	PGBD1	piggyBac transposable element derived 1	CCDS4648.1	chr6_28269661-28269661_G_A	677R>Q	Substitution	Nonsynonymous coding	35%
MM04T	PGBD1	piggyBac transposable element derived 1	CCDS4648.1	chr6_28269396-28269396_G_T	589E>X	Substitution	Nonsense	36%
MM04T	PGBD2	piggyBac transposable element derived 2	CCDS31128.1	chr1_249211486-249211486_G_A	235E>K	Substitution	Nonsynonymous coding	38%
MM04T	PGBD4	piggyBac transposable element derived 4	CCDS10033.1	chr15_34394857-34394857_C_T	42S>L	Substitution	Nonsynonymous coding	27%
MM04T	PGBD4	piggyBac transposable element derived 4	CCDS10033.1	chr15_34395812-34395812_G_T	360Q>H	Substitution	Nonsynonymous coding	40%
MM04T	PGF	placental growth factor	CCDS9835.1	chr14_75413091-75413091_T_G	138K>T	Substitution	Nonsynonymous coding	30%
MM04T	PGK1	phosphoglycerate kinase 1	CCDS14438.1	chrX_77372837-77372837_C_A	149A>D	Substitution	Nonsynonymous coding	29%
MM04T	PGLYRP2	peptidoglycan recognition protein 2	ENST00000292609	chr19_15580316-15580316_G_A	590R>W	Substitution	Nonsynonymous coding	21%
MM04T	PGM1	phosphoglucomutase 1	CCDS625.1	chr1_64117392-64117392_T_G	445L>V	Substitution	Nonsynonymous coding	41%
MM04T	PGPEP1L	pyroglutamyl-peptidase I-like	NM_001102612	chr15_99511748-99511748_C_T	184E>K	Substitution	Nonsynonymous coding	12%

MM04T	PGPEP1L	pyroglutamyl-peptidase I-like	NM_001102612	chr15_99511874-99511874_C_T	142A>T	Substitution	Nonsynonymous coding	17%
MM04T	PGR	progesterone receptor	CCDS8310.1	chr11_100922293-100922293_C_T	740R>Q	Substitution	Nonsynonymous coding	42%
MM04T	PHACTR1	phosphatase and actin regulator 1	NM_030948	chr6_13053737-13053737_G_A	131E>K	Substitution	Nonsynonymous coding	29%
MM04T	PHACTR3	phosphatase and actin regulator 3	CCDS13480.1	chr20_58415459-58415459_G_T	474E>X	Substitution	Nonsense	20%
MM04T	PHEX	phosphate regulating endopeptidase homolog, X-linked	CCDS14204.1	chrX_22094544-22094544_G_A	130E>K	Substitution	Nonsynonymous coding	27%
MM04T	PHF12	PHD finger protein 12	CCDS32598.1	chr17_27233328-27233328_C_A	963S>I	Substitution	Nonsynonymous coding	28%
MM04T	PHF16	PHD finger protein 16	CCDS14271.1	chrX_46918360-46918360_G_A	785E>K	Substitution	Nonsynonymous coding	26%
MM04T	PHF17	PHD finger protein 17	CCDS34062.1	chr4_129770271-129770271_G_T	145D>Y	Substitution	Nonsynonymous coding	31%
MM04T	PHF17	PHD finger protein 17	CCDS34062.1	chr4_129792876-129792876_G_A	663R>H	Substitution	Nonsynonymous coding	36%
MM04T	PHF3	PHD finger protein 3	CCDS4966.1	chr6_64423604-64423604_A_C	2040*>Y	Substitution	Nonsynonymous coding	34%
MM04T	PHF7	PHD finger protein 7	CCDS2854.1	chr3_52455015-52455015_G_T	170Q>H	Substitution	Nonsynonymous coding	19%
MM04T	PHIP	pleckstrin homology domain interacting protein	CCDS4987.1	chr6_79655843-79655843_G_T	1502S>Y	Substitution	Nonsynonymous coding	37%
MM04T	PHKA1	phosphorylase kinase, alpha 1 (muscle)	CCDS14421.1	chrX_71825186-71825186_C_T	917R>Q	Substitution	Nonsynonymous coding	31%
MM04T	PHKA1	phosphorylase kinase, alpha 1 (muscle)	CCDS14421.1	chrX_71838672-71838672_G_T	753L>I	Substitution	Nonsynonymous coding	24%
MM04T	PHKA1	phosphorylase kinase, alpha 1 (muscle)	CCDS14421.1	chrX_71870254-71870254_T_C	437D>G	Substitution	Nonsynonymous coding	20%
MM04T	PHKA1	phosphorylase kinase, alpha 1 (muscle)	CCDS14421.1	chrX_71825181-71825181_G_A	919R>X	Substitution	Nonsense	27%
MM04T	PHKA2	phosphorylase kinase, alpha 2 (liver)	CCDS14190.1	chrX_18936961-18936961_C_T	659E>K	Substitution	Nonsynonymous coding	29%
MM04T	PHKA2	phosphorylase kinase, alpha 2 (liver)	CCDS14190.1	chrX_18943856-18943856_C_T	500R>Q	Substitution	Nonsynonymous coding	15%
MM04T	PHKA2	phosphorylase kinase, alpha 2 (liver)	CCDS14190.1	chrX_18958138-18958138_C_T	298R>Q	Substitution	Nonsynonymous coding	17%
MM04T	PHKA2	phosphorylase kinase, alpha 2 (liver)	CCDS14190.1	chrX_18924728-18924728_G_T	897Y>X	Substitution	Nonsense	26%
MM04T	PHLDB2	pleckstrin homology-like domain, family B, member 2	CCDS46886.1	chr3_111688708-111688708_C_T	1163R>C	Substitution	Nonsynonymous coding	27%
MM04T	PHLPP2	PH domain and leucine rich repeat protein phosphatase 2	CCDS32479.1	chr16_71683148-71683148_C_T	1206R>Q	Substitution	Nonsynonymous coding	25%
MM04T	PHTF1	putative homeodomain transcription factor 1	CCDS861.1	chr1_114243561-114243561_C_T	634G>E	Substitution	Nonsynonymous coding	38%
MM04T	PI4KA	phosphatidylinositol 4-kinase, catalytic, alpha	CCDS33603.1	chr22_21107187-21107187_T_G	NA	Substitution	Splice site donor	31%
MM04T	PI4KB	phosphatidylinositol 4-kinase, catalytic, beta	CCDS993.1	chr1_151278752-151278752_G_A	436R>W	Substitution	Nonsynonymous coding	29%
MM04T	PIAS2	protein inhibitor of activated STAT, 2	CCDS32824.1	chr18_44470595-44470595_T_A	149K>N	Substitution	Nonsynonymous coding	25%
MM04T	PIEZO2	piezo-type mechanosensitive ion channel component 2	NM_022068	chr18_10758077-10758077_C_A	1246E>D	Substitution	Nonsynonymous coding	16%
MM04T	PIFO	primary cilia formation	CCDS833.1	chr1_111891171-111891171_G_T	98V>L	Substitution	Nonsynonymous coding	19%
MM04T	PIGK	phosphatidylinositol glycan anchor biosynthesis, class K	CCDS674.1	chr1_77627340-77627340_C_T	214R>Q	Substitution	Nonsynonymous coding	30%
MM04T	PIGO	phosphatidylinositol glycan anchor biosynthesis, class O	CCDS6575.1	chr9_35091479-35091479_C_T	802R>Q	Substitution	Nonsynonymous coding	34%
MM04T	PIGX	phosphatidylinositol glycan anchor biosynthesis, class X	CCDS3320.1	chr3_196449285-196449285_G_T	NA	Substitution	Splice site acceptor	23%
MM04T	PIK3C2A	phosphatidylinositol-4-phosphate 3-kinase, catalytic subunit type 2 alpha	CCDS7824.1	chr11_17158085-17158085_C_A	598E>X	Substitution	Nonsense	21%

MM04T	PIK3C2B	phosphatidylinositol-4-phosphate 3-kinase, catalytic subunit type 2 beta	CCDS1446.1	chr1_204411723-204411723_G_T	1029F>L	Substitution	Nonsynonymous coding	12%
MM04T	PIK3C2B	phosphatidylinositol-4-phosphate 3-kinase, catalytic subunit type 2 beta	ENST00000438854	chr1_204436071-204436071_C_T	32R>H	Substitution	Nonsynonymous coding	25%
MM04T	PIK3CA	phosphatidylinositol-4,5-bisphosphate 3-kinase, catalytic subunit alpha	CCDS43171.1	chr3_178916876-178916876_G_A	88R>Q	Substitution	Nonsynonymous coding	32%
MM04T	PIK3CA	phosphatidylinositol-4,5-bisphosphate 3-kinase, catalytic subunit alpha	CCDS43171.1	chr3_178922301-178922301_G_A	357R>Q	Substitution	Nonsynonymous coding	32%
MM04T	PIK3CD	phosphatidylinositol-4,5-bisphosphate 3-kinase, catalytic subunit delta	CCDS104.1	chr1_9778918-9778918_C_T	396A>V	Substitution	Nonsynonymous coding	13%
MM04T	PIK3CG	phosphatidylinositol-4,5-bisphosphate 3-kinase, catalytic subunit gamma	CCDS5739.1	chr7_106509747-106509747_G_A	581E>K	Substitution	Nonsynonymous coding	27%
MM04T	PIK3CG	phosphatidylinositol-4,5-bisphosphate 3-kinase, catalytic subunit gamma	CCDS5739.1	chr7_106515198-106515198_G_A	781E>K	Substitution	Nonsynonymous coding	16%
MM04T	PIK3CG	phosphatidylinositol-4,5-bisphosphate 3-kinase, catalytic subunit gamma	CCDS5739.1	chr7_106523554-106523554_T_G	902F>L	Substitution	Nonsynonymous coding	29%
MM04T	PIKFYVE	phosphoinositide kinase, FYVE finger containing	CCDS2382.1	chr2_209141530-209141530_G_T	139K>N	Substitution	Nonsynonymous coding	28%
MM04T	PIKFYVE	phosphoinositide kinase, FYVE finger containing	CCDS2382.1	chr2_209215599-209215599_C_A	1847R>S	Substitution	Nonsynonymous coding	30%
MM04T	PIKFYVE	phosphoinositide kinase, FYVE finger containing	CCDS2382.1	chr2_209218628-209218628_G_T	1951D>Y	Substitution	Nonsynonymous coding	24%
MM04T	PIP5K1A	phosphatidylinositol-4-phosphate 5-kinase, type I, alpha	CCDS44219.1	chr1_151196848-151196848_T_C	41V>A	Substitution	Nonsynonymous coding	27%
MM04T	PITPNM2	phosphatidylinositol transfer protein, membrane-associated 2	CCDS9242.1	chr12_123488954-123488954_G_T	345F>L	Substitution	Nonsynonymous coding	28%
MM04T	PIWIL1	piwi-like 1 (Drosophila)	CCDS9268.1	chr12_130839140-130839140_G_T	368R>I	Substitution	Nonsynonymous coding	17%
MM04T	PIWIL2	piwi-like 2 (Drosophila)	CCDS6029.1	chr8_22138920-22138920_C_T	106A>V	Substitution	Nonsynonymous coding	31%
MM04T	PIWIL2	piwi-like 2 (Drosophila)	CCDS6029.1	chr8_22168710-22168710_C_T	629A>V	Substitution	Nonsynonymous coding	33%
MM04T	PIWIL3	piwi-like 3 (Drosophila)	CCDS33623.1	chr22_25131750-25131750_C_A	520R>I	Substitution	Nonsynonymous coding	27%
MM04T	PIWIL3	piwi-like 3 (Drosophila)	CCDS33623.1	chr22_25145740-25145740_C_A	379S>I	Substitution	Nonsynonymous coding	23%
MM04T	PIWIL3	piwi-like 3 (Drosophila)	CCDS33623.1	chr22_25123969-25123969_G_A	662R>X	Substitution	Nonsense	38%
MM04T	PIWIL4	piwi-like 4 (Drosophila)	CCDS31656.1	chr11_94335100-94335100_G_T	507R>I	Substitution	Nonsynonymous coding	26%
MM04T	PJA1	praja ring finger 1, E3 ubiquitin protein ligase	CCDS14393.1	chrX_68381320-68381320_C_T	588A>T	Substitution	Nonsynonymous coding	30%
MM04T	PKD1L1	polycystic kidney disease 1 like 1	CCDS34633.1	chr7_47835708-47835708_G_T	2745S>Y	Substitution	Nonsynonymous coding	23%
MM04T	PKD1L1	polycystic kidney disease 1 like 1	CCDS34633.1	chr7_47886498-47886498_G_A	1711S>F	Substitution	Nonsynonymous coding	23%
MM04T	PKD1L2	polycystic kidney disease 1-like 2	CCDS42202.1	chr16_81204660-81204660_C_T	933G>E	Substitution	Nonsynonymous coding	18%
MM04T	PKD1L3	polycystic kidney disease 1-like 3	ENST00000335106	chr16_71971315-71971315_G_T	1442F>L	Substitution	Nonsynonymous coding	34%
MM04T	PKD2	polycystic kidney disease 2 (autosomal dominant)	CCDS3627.1	chr4_88959641-88959641_G_A	361R>Q	Substitution	Nonsynonymous coding	27%
MM04T	PKDREJ	polycystic kidney disease (polycystin) and REJ homolog (sperm receptor for egg jelly	CCDS14073.1	chr22_46653425-46653425_G_A	1932S>L	Substitution	Nonsynonymous coding	33%
MM04T	PKHD1	polycystic kidney and hepatic disease 1 (autosomal recessive)	CCDS4935.1	chr6_51640627-51640627_G_T	2845H>N	Substitution	Nonsynonymous coding	33%
MM04T	PKHD1	polycystic kidney and hepatic disease 1 (autosomal recessive)	CCDS4935.1	chr6_51750747-51750747_T_G	2378K>T	Substitution	Nonsynonymous coding	30%
MM04T	PKHD1	polycystic kidney and hepatic disease 1 (autosomal recessive)	CCDS4935.1	chr6_51897852-51897852_T_A	1114S>C	Substitution	Nonsynonymous coding	34%
MM04T	PKHD1	polycystic kidney and hepatic disease 1 (autosomal recessive)	CCDS4935.1	chr6_51913401-51913401_C_A	766G>X	Substitution	Nonsense	31%
MM04T	PKNOX2	PBX/knotted 1 homeobox 2	CCDS41730.1	chr11_125299990-125299990_C_T	382A>V	Substitution	Nonsynonymous coding	33%

MM04T	PKP2	plakophilin 2	CCDS8731.1	chr12_32945618-32945618_G_T	846S>Y	Substitution	Nonsynonymous coding	20%
MM04T	PKP3	plakophilin 3	CCDS7695.1	chr11_400543-400543_G_T	525E>D	Substitution	Nonsynonymous coding	25%
MM04T	PKP4	plakophilin 4	CCDS33305.1	chr2_159523057-159523057_C_T	904R>C	Substitution	Nonsynonymous coding	24%
MM04T	PLA2G4F	phospholipase A2, group IVF	CCDS32204.1	chr15_42446648-42446648_C_A	65A>S	Substitution	Nonsynonymous coding	12%
MM04T	PLAUR	plasminogen activator, urokinase receptor	CCDS12628.1	chr19_44160711-44160711_C_T	131R>Q	Substitution	Nonsynonymous coding	26%
MM04T	PLCB1	phospholipase C, beta 1 (phosphoinositide-specific)	CCDS13102.1	chr20_8609067-8609067_G_T	125E>X	Substitution	Nonsense	34%
MM04T	PLCE1	phospholipase C, epsilon 1	CCDS41552.1	chr10_95791870-95791870_T_A	356I>N	Substitution	Nonsynonymous coding	20%
MM04T	PLCE1	phospholipase C, epsilon 1	CCDS41552.1	chr10_95993845-95993845_G_A	664D>N	Substitution	Nonsynonymous coding	31%
MM04T	PLCG2	phospholipase C, gamma 2 (phosphatidylinositol-specific)	CCDS42204.1	chr16_81888120-81888120_C_T	89R>C	Substitution	Nonsynonymous coding	23%
MM04T	PLCG2	phospholipase C, gamma 2 (phosphatidylinositol-specific)	CCDS42204.1	chr16_81953246-81953246_G_A	738E>K	Substitution	Nonsynonymous coding	21%
MM04T	PLCH1	phospholipase C, eta 1	CCDS46939.1	chr3_155203396-155203396_C_A	916R>I	Substitution	Nonsynonymous coding	37%
MM04T	PLCL1	phospholipase C-like 1	CCDS2326.1	chr2_198949838-198949838_T_A	435L>I	Substitution	Nonsynonymous coding	29%
MM04T	PLCL1	phospholipase C-like 1	CCDS2326.1	chr2_198949867-198949867_G_T	444K>N	Substitution	Nonsynonymous coding	30%
MM04T	PLCL1	phospholipase C-like 1	CCDS2326.1	chr2_198968608-198968608_G_T	920R>I	Substitution	Nonsynonymous coding	16%
MM04T	PLCL2	phospholipase C-like 2	CCDS33713.1	chr3_17052053-17052053_C_A	279F>L	Substitution	Nonsynonymous coding	32%
MM04T	PLCZ1	phospholipase C, zeta 1	CCDS8680.1	chr12_18876418-18876418_C_T	65R>Q	Substitution	Nonsynonymous coding	39%
MM04T	PLD1	phospholipase D1, phosphatidylcholine-specific	CCDS3216.1	chr3_171330172-171330172_T_G	927M>L	Substitution	Nonsynonymous coding	26%
MM04T	PLD1	phospholipase D1, phosphatidylcholine-specific	CCDS3216.1	chr3_171405244-171405244_A_G	557F>S	Substitution	Nonsynonymous coding	20%
MM04T	PLEC	plectin	CCDS43772.1	chr8_144993091-144993091_C_T	3770R>H	Substitution	Nonsynonymous coding	20%
MM04T	PLEKHA1	pleckstrin homology domain containing, family A (phosphoinositide binding specific) member	CCDS7629.1	chr10_124152787-124152787_A_C	24K>T	Substitution	Nonsynonymous coding	15%
MM04T	PLEKHA6	pleckstrin homology domain containing, family A member 6	CCDS1444.1	chr1_204228847-204228847_C_A	182E>D	Substitution	Nonsynonymous coding	18%
MM04T	PLEKHA8P1	pleckstrin homology domain containing, family A member 8 pseudogene 1	CCDS8747.1	chr12_45567149-45567149_G_A	334R>X	Substitution	Nonsense	31%
MM04T	PLEKHG1	pleckstrin homology domain containing, family G (with RhoGef domain) member 1	CCDS34552.1	chr6_151139352-151139352_C_T	438H>Y	Substitution	Nonsynonymous coding	35%
MM04T	PLEKHG1	pleckstrin homology domain containing, family G (with RhoGef domain) member 1	CCDS34552.1	chr6_151151900-151151900_G_T	551E>D	Substitution	Nonsynonymous coding	23%
MM04T	PLEKHG4	pleckstrin homology domain containing, family G (with RhoGef domain) member 4	CCDS32466.1	chr16_67315972-67315972_G_A	356E>K	Substitution	Nonsynonymous coding	32%
MM04T	PLEKHG5	pleckstrin homology domain containing, family G (with RhoGef domain) member 5	CCDS41240.1	chr1_6530630-6530630_G_A	616A>V	Substitution	Nonsynonymous coding	36%
MM04T	PLEKHH1	pleckstrin homology domain containing, family H (with MyTH4 domain) member 1	CCDS45128.1	chr14_68029207-68029207_G_A	287V>I	Substitution	Nonsynonymous coding	21%
MM04T	PLEKHH2	pleckstrin homology domain containing, family H (with MyTH4 domain) member 2	CCDS1812.1	chr2_43953473-43953473_T_G	868D>E	Substitution	Nonsynonymous coding	23%
MM04T	PLEKHH2	pleckstrin homology domain containing, family H (with MyTH4 domain) member 2	CCDS1812.1	chr2_43980760-43980760_T_G	1219L>R	Substitution	Nonsynonymous coding	46%
MM04T	PLG	plasminogen	CCDS5279.1	chr6_161139332-161139332_C_A	265P>H	Substitution	Nonsynonymous coding	32%
MM04T	PLK3	polo-like kinase 3	CCDS515.1	chr1_45266820-45266820_G_A	144R>Q	Substitution	Nonsynonymous coding	20%
MM04T	PLK3	polo-like kinase 3	CCDS515.1	chr1_45267520-45267520_A_T	190N>I	Substitution	Nonsynonymous coding	22%

MM04T	PLOD2	procollagen-lysine, 2-oxoglutarate 5-dioxygenase 2	CCDS3132.1	chr3_145824381-145824381_G_T	185L>I	Substitution	Nonsynonymous coding	28%
MM04T	PLOD2	procollagen-lysine, 2-oxoglutarate 5-dioxygenase 2	CCDS3132.1	chr3_145841984-145841984_C_A	48E>X	Substitution	Nonsense	26%
MM04T	PLOD3	procollagen-lysine, 2-oxoglutarate 5-dioxygenase 3	CCDS5715.1	chr7_100850158-100850158_G_A	655R>C	Substitution	Nonsynonymous coding	25%
MM04T	PLRG1	pleiotropic regulator 1	CCDS34083.1	chr4_155457844-155457844_C_A	513R>I	Substitution	Nonsynonymous coding	18%
MM04T	PLRG1	pleiotropic regulator 1	CCDS34083.1	chr4_155458497-155458497_G_A	476R>X	Substitution	Nonsense	44%
MM04T	PLSCR2	phospholipid scramblase 2	CCDS3134.1	chr3_146173150-146173150_C_T	66R>Q	Substitution	Nonsynonymous coding	28%
MM04T	PLSCR5	phospholipid scramblase family, member 5	CCDS46931.1	chr3_146303888-146303888_G_T	263F>L	Substitution	Nonsynonymous coding	44%
MM04T	PLVAP	plasmalemma vesicle associated protein	CCDS32952.1	chr19_17476402-17476402_C_T	291R>Q	Substitution	Nonsynonymous coding	37%
MM04T	PLXDC2	plexin domain containing 2	CCDS7132.1	chr10_20436835-20436835_A_C	NA	Substitution	Splice site donor	25%
MM04T	PLXDC2	plexin domain containing 2	CCDS7132.1	chr10_20335828-20335828_C_T	119R>X	Substitution	Nonsense	29%
MM04T	PLXNA1	plexin A1	CCDS33847.1	chr3_126735843-126735843_G_A	1057R>Q	Substitution	Nonsynonymous coding	30%
MM04T	PLXNA1	plexin A1	CCDS33847.1	chr3_126737291-126737291_G_A	1249R>Q	Substitution	Nonsynonymous coding	22%
MM04T	PLXNA2	plexin A2	CCDS31013.1	chr1_208276539-208276539_G_T	520C>X	Substitution	Nonsense	30%
MM04T	PLXNA3	plexin A3	CCDS14752.1	chrX_153697505-153697505_T_C	1514V>A	Substitution	Nonsynonymous coding	28%
MM04T	PLXNA4	plexin A4	CCDS43646.1	chr7_131815337-131815337_C_T	NA	Substitution	Splice site acceptor	33%
MM04T	PLXNB3	plexin B3	CCDS14729.1	chrX_153041609-153041609_G_A	1557D>N	Substitution	Nonsynonymous coding	36%
MM04T	PLXNB3	plexin B3	CCDS14729.1	chrX_153044439-153044439_C_T	1892A>V	Substitution	Nonsynonymous coding	39%
MM04T	PLXNC1	plexin C1	CCDS9049.1	chr12_94658989-94658989_A_C	1195E>D	Substitution	Nonsynonymous coding	22%
MM04T	PLXND1	plexin D1	CCDS33854.1	chr3_129279561-129279561_C_A	1667D>Y	Substitution	Nonsynonymous coding	28%
MM04T	PMEL	premelanosome protein	CCDS8897.1	chr12_56351351-56351351_C_A	246D>Y	Substitution	Nonsynonymous coding	24%
MM04T	PMPCB	peptidase (mitochondrial processing) beta	CCDS5730.1	chr7_102939929-102939929_G_T	94E>X	Substitution	Nonsense	31%
MM04T	PMS1	PMS1 postmeiotic segregation increased 1 (S. cerevisiae)	ENST00000409985	chr2_190670540-190670540__AA	NA	Insertion	Frameshift	11%
MM04T	PMS1	PMS1 postmeiotic segregation increased 1 (S. cerevisiae)	CCDS2302.1	chr2_190660497-190660497_G_T	45E>D	Substitution	Nonsynonymous coding	25%
MM04T	PNISR	PNN-interacting serine/arginine-rich protein	CCDS5043.1	chr6_99849452-99849452_T_G	461K>T	Substitution	Nonsynonymous coding	32%
MM04T	PNMA5	paraneoplastic Ma antigen family member 5	CCDS14718.1	chrX_152159655-152159655_G_A	163S>L	Substitution	Nonsynonymous coding	35%
MM04T	PNPLA4	patatin-like phospholipase domain containing 4	CCDS14129.1	chrX_7890090-7890090_G_T	77S>Y	Substitution	Nonsynonymous coding	33%
MM04T	PNPLA6	patatin-like phospholipase domain containing 6	CCDS32891.1	chr19_7626154-7626154_A_G	1287D>G	Substitution	Nonsynonymous coding	35%
MM04T	PNPLA8	patatin-like phospholipase domain containing 8	CCDS34733.1	chr7_108119814-108119814_A_C	630L>V	Substitution	Nonsynonymous coding	36%
MM04T	PNRC1	proline-rich nuclear receptor coactivator 1	CCDS5018.1	chr6_89793662-89793662_G_T	244R>I	Substitution	Nonsynonymous coding	34%
MM04T	PODXL	podocalyxin-like	CCDS34755.1	chr7_131191465-131191465_C_A	374E>D	Substitution	Nonsynonymous coding	37%
MM04T	POF1B	premature ovarian failure, 1B	CCDS14452.1	chrX_84559349-84559349_C_A	522E>D	Substitution	Nonsynonymous coding	16%
MM04T	POF1B	premature ovarian failure, 1B	CCDS14452.1	chrX_84560831-84560831_C_A	468R>I	Substitution	Nonsynonymous coding	30%

MM04T	POF1B	premature ovarian failure, 1B	CCDS14452.1	chrX_84560848-84560848_C_A	462E>D	Substitution	Nonsynonymous coding	30%
MM04T	POF1B	premature ovarian failure, 1B	CCDS14452.1	chrX_84634253-84634253_G_T	69F>L	Substitution	Nonsynonymous coding	26%
MM04T	POF1B	premature ovarian failure, 1B	CCDS14452.1	chrX_84634345-84634345_C_A	39A>S	Substitution	Nonsynonymous coding	24%
MM04T	POGK	pogo transposable element with KRAB domain	CCDS1254.1	chr1_166818709-166818709_T_G	298I>S	Substitution	Nonsynonymous coding	36%
MM04T	POLA1	polymerase (DNA directed), alpha 1, catalytic subunit	CCDS14214.1	chrX_24906160-24906160_G_A	1356R>Q	Substitution	Nonsynonymous coding	21%
MM04T	POLE	polymerase (DNA directed), epsilon, catalytic subunit	CCDS9278.1	chr12_133225540-133225540_G_A	1375A>V	Substitution	Nonsynonymous coding	34%
MM04T	POLE	polymerase (DNA directed), epsilon, catalytic subunit	CCDS9278.1	chr12_133253184-133253184_G_C	286P>R	Substitution	Nonsynonymous coding	27%
MM04T	POLG2	polymerase (DNA directed), gamma 2, accessory subunit	CCDS32706.1	chr17_62492686-62492686_T_C	134K>R	Substitution	Nonsynonymous coding	36%
MM04T	POLI	polymerase (DNA directed) iota	CCDS11954.2	chr18_51820556-51820556_A_C	648N>H	Substitution	Nonsynonymous coding	33%
MM04T	POLN	polymerase (DNA directed) nu	CCDS3360.1	chr4_2230823-2230823_T_C	NA	Substitution	Splice site donor	41%
MM04T	POLQ	polymerase (DNA directed), theta	CCDS33833.1	chr3_121168251-121168251_C_T	2392G>E	Substitution	Nonsynonymous coding	11%
MM04T	POLQ	polymerase (DNA directed), theta	CCDS33833.1	chr3_121228428-121228428_C_A	647D>Y	Substitution	Nonsynonymous coding	39%
MM04T	POLQ	polymerase (DNA directed), theta	ENST00000393672	chr3_121264772-121264772_T_C	118R>G	Substitution	Nonsynonymous coding	29%
MM04T	POLQ	polymerase (DNA directed), theta	ENST00000393672	chr3_121265230-121265230_C_A	87G>X	Substitution	Nonsense	24%
MM04T	POLR1B	polymerase (RNA) I polypeptide B, 128kDa	CCDS2097.1	chr2_113333247-113333247_C_T	1117R>W	Substitution	Nonsynonymous coding	37%
MM04T	POLR1E	polymerase (RNA) I polypeptide E, 53kDa	CCDS6611.1	chr9_37495252-37495252_G_A	212D>N	Substitution	Nonsynonymous coding	19%
MM04T	POLR2A	polymerase (RNA) II (DNA directed) polypeptide A, 220kDa	CCDS32548.1	chr17_7402674-7402674_G_A	512R>Q	Substitution	Nonsynonymous coding	33%
MM04T	POLR3B	polymerase (RNA) III (DNA directed) polypeptide B	CCDS9105.1	chr12_106897967-106897967_G_T	1069M>I	Substitution	Nonsynonymous coding	32%
MM04T	POM121L2	POM121 transmembrane nucleoporin-like 2	NM_033482	chr6_27278315-27278315_C_A	545E>D	Substitution	Nonsynonymous coding	26%
MM04T	POM121L2	POM121 transmembrane nucleoporin-like 2	NM_033482	chr6_27278909-27278909_C_A	347E>D	Substitution	Nonsynonymous coding	30%
MM04T	POM121L2	POM121 transmembrane nucleoporin-like 2	NM_033482	chr6_27279093-27279093_A_T	286I>K	Substitution	Nonsynonymous coding	22%
MM04T	PON2	paraoxonase 2	CCDS5640.1	chr7_95045580-95045580_G_A	59P>S	Substitution	Nonsynonymous coding	26%
MM04T	POSTN	periostin, osteoblast specific factor	CCDS9364.1	chr13_38172796-38172796_T_G	23N>T	Substitution	Nonsynonymous coding	20%
MM04T	POU2F2	POU class 2 homeobox 2	CCDS33035.1	chr19_42626697-42626697_C_A	20E>D	Substitution	Nonsynonymous coding	26%
MM04T	POU4F3	POU class 4 homeobox 3	CCDS4281.1	chr5_145719823-145719823_G_A	278R>H	Substitution	Nonsynonymous coding	36%
MM04T	POU6F1	POU class 6 homeobox 1	CCDS31803.1	chr12_51589905-51589905_C_A	33A>S	Substitution	Nonsynonymous coding	30%
MM04T	PP13004	-	ENST00000381493	chr7_36124078-36124078_C_T	NA	Substitution	Splice site acceptor	29%
MM04T	PP2D1	protein phosphatase 2C-like domain containing 1	ENST00000389050	chr3_20042611-20042611_C_A	334R>I	Substitution	Nonsynonymous coding	35%
MM04T	PP2D1	protein phosphatase 2C-like domain containing 1	ENST00000389050	chr3_20043127-20043127_T_G	162K>T	Substitution	Nonsynonymous coding	53%
MM04T	PPAN-P2RY11	PPAN-P2RY11 readthrough	CCDS42498.1	chr19_10224334-10224334_C_A	435F>L	Substitution	Nonsynonymous coding	20%
MM04T	PPARA	peroxisome proliferator-activated receptor alpha	CCDS33669.1	chr22_46615873-46615873_G_A	225A>T	Substitution	Nonsynonymous coding	14%
MM04T	PPARG	peroxisome proliferator-activated receptor gamma	CCDS2609.1	chr3_12458537-12458537_G_T	385R>L	Substitution	Nonsynonymous coding	28%

MM04T	PPARG	peroxisome proliferator-activated receptor gamma	CCDS2609.1	chr3_12458536-12458536_C_T	385R>X	Substitution	Nonsense	29%
MM04T	PPARGC1A	peroxisome proliferator-activated receptor gamma, coactivator 1 alpha	CCDS3429.1	chr4_23803892-23803892_A_G	699V>A	Substitution	Nonsynonymous coding	18%
MM04T	PPEF1	protein phosphatase, EF-hand calcium binding domain 1	CCDS14188.1	chrX_18824657-18824657_G_A	463R>H	Substitution	Nonsynonymous coding	33%
MM04T	PPEF1	protein phosphatase, EF-hand calcium binding domain 1	CCDS14188.1	chrX_18845417-18845417_C_T	592R>C	Substitution	Nonsynonymous coding	22%
MM04T	PPEF2	protein phosphatase, EF-hand calcium binding domain 2	CCDS34013.1	chr4_76812859-76812859_C_T	NA	Substitution	Splice site acceptor	23%
MM04T	PPFIA2	protein tyrosine phosphatase, receptor type, f polypeptide (PTPRF), interacting protein	NM_003625	chr12_81655778-81655778_G_A	1253R>C	Substitution	Nonsynonymous coding	33%
MM04T	PPFIA2	protein tyrosine phosphatase, receptor type, f polypeptide (PTPRF), interacting protein	NM_003625	chr12_81769690-81769690_C_A	339R>I	Substitution	Nonsynonymous coding	36%
MM04T	PPFIA2	protein tyrosine phosphatase, receptor type, f polypeptide (PTPRF), interacting protein	NM_003625	chr12_82147853-82147853_G_A	50R>W	Substitution	Nonsynonymous coding	23%
MM04T	PPFIA3	protein tyrosine phosphatase, receptor type, f polypeptide (PTPRF), interacting protein	CCDS12758.1	chr19_49652893-49652893_G_T	1148E>D	Substitution	Nonsynonymous coding	18%
MM04T	PPFIA4	protein tyrosine phosphatase, receptor type, f polypeptide (PTPRF), interacting protein	CCDS44296.1	chr1_203028323-203028323_G_A	303E>K	Substitution	Nonsynonymous coding	23%
MM04T	PPFIBP1	PTPRF interacting protein, binding protein 1 (liprin beta 1)	CCDS8713.1	chr12_27820104-27820104_G_T	333D>Y	Substitution	Nonsynonymous coding	13%
MM04T	PPFIBP1	PTPRF interacting protein, binding protein 1 (liprin beta 1)	CCDS8713.1	chr12_27830006-27830006_G_A	531R>Q	Substitution	Nonsynonymous coding	22%
MM04T	PPFIBP2	PTPRF interacting protein, binding protein 2 (liprin beta 2)	CCDS31419.1	chr11_7647047-7647047_G_T	251D>Y	Substitution	Nonsynonymous coding	15%
MM04T	PPID	peptidylprolyl isomerase D	CCDS3801.1	chr4_159640431-159640431_C_A	78K>N	Substitution	Nonsynonymous coding	32%
MM04T	PPIL4	peptidylprolyl isomerase (cyclophilin)-like 4	CCDS34550.1	chr6_149826734-149826734_C_T	445R>Q	Substitution	Nonsynonymous coding	30%
MM04T	PPIP5K2	diphosphoinositol pentakisphosphate kinase 2	CCDS34207.1	chr5_102484977-102484977_G_A	289R>Q	Substitution	Nonsynonymous coding	29%
MM04T	PPM1J	protein phosphatase, Mg2+/Mn2+ dependent, 1J	CCDS855.2	chr1_113255392-113255392_C_T	235A>T	Substitution	Nonsynonymous coding	31%
MM04T	PPM1N	protein phosphatase, Mg2+/Mn2+ dependent, 1N (putative)	ENST00000401593	chr19_46005399-46005399_A_C	122K>Q	Substitution	Nonsynonymous coding	28%
MM04T	PPP1R12B	protein phosphatase 1, regulatory subunit 12B	CCDS30973.1	chr1_202396302-202396302_G_A	279R>Q	Substitution	Nonsynonymous coding	28%
MM04T	PPP1R15B	protein phosphatase 1, regulatory subunit 15B	CCDS1445.1	chr1_204379897-204379897_T_G	215N>H	Substitution	Nonsynonymous coding	25%
MM04T	PPP1R17	protein phosphatase 1, regulatory subunit 17	CCDS5436.1	chr7_31736606-31736606_G_T	88R>I	Substitution	Nonsynonymous coding	27%
MM04T	PPP1R36	protein phosphatase 1, regulatory subunit 36	CCDS9767.1	chr14_65032134-65032134_G_A	110R>Q	Substitution	Nonsynonymous coding	28%
MM04T	PPP1R3A	protein phosphatase 1, regulatory subunit 3A	CCDS5759.1	chr7_113518676-113518676_G_T	824S>Y	Substitution	Nonsynonymous coding	28%
MM04T	PPP1R3A	protein phosphatase 1, regulatory subunit 3A	CCDS5759.1	chr7_113519690-113519690_C_T	486R>Q	Substitution	Nonsynonymous coding	40%
MM04T	PPP1R3A	protein phosphatase 1, regulatory subunit 3A	CCDS5759.1	chr7_113519706-113519706_C_T	481D>N	Substitution	Nonsynonymous coding	19%
MM04T	PPP1R3A	protein phosphatase 1, regulatory subunit 3A	CCDS5759.1	chr7_113520131-113520131_C_A	339R>M	Substitution	Nonsynonymous coding	44%
MM04T	PPP1R3A	protein phosphatase 1, regulatory subunit 3A	CCDS5759.1	chr7_113522156-113522156_G_A	302R>X	Substitution	Nonsense	36%
MM04T	PPP1R9A	protein phosphatase 1, regulatory subunit 9A	CCDS34683.1	chr7_94539793-94539793_G_A	123R>Q	Substitution	Nonsynonymous coding	34%
MM04T	PPP2R1B	protein phosphatase 2, regulatory subunit A, beta	CCDS8348.1	chr11_111631757-111631757_C_T	109A>T	Substitution	Nonsynonymous coding	21%
MM04T	PPP2R3C	protein phosphatase 2, regulatory subunit B'', gamma	CCDS9654.1	chr14_35579823-35579823_C_A	67D>Y	Substitution	Nonsynonymous coding	36%
MM04T	PPP2R5D	protein phosphatase 2, regulatory subunit B', delta	CCDS4878.1	chr6_42977093-42977093_G_T	429E>X	Substitution	Nonsense	28%
MM04T	PPP3R1	protein phosphatase 3, regulatory subunit B, alpha	CCDS46310.1	chr2_68414394-68414394_T_G	91K>N	Substitution	Nonsynonymous coding	25%

MM04T	PPP4R1	protein phosphatase 4, regulatory subunit 1	CCDS42412.1	chr18_9563462-9563462_A_C	554L>V	Substitution	Nonsynonymous coding	36%
MM04T	PPP4R4	protein phosphatase 4, regulatory subunit 4	CCDS9921.1	chr14_94703970-94703970_T_C	267V>A	Substitution	Nonsynonymous coding	23%
MM04T	PPP6C	protein phosphatase 6, catalytic subunit	CCDS48018.1	chr9_127915943-127915943_C_T	217E>K	Substitution	Nonsynonymous coding	18%
MM04T	PPYR1	pancreatic polypeptide receptor 1	CCDS31193.1	chr10_47087154-47087154_C_T	124S>L	Substitution	Nonsynonymous coding	36%
MM04T	PRDM10	PR domain containing 10	CCDS44771.1	chr11_129807524-129807524_T_G	336K>N	Substitution	Nonsynonymous coding	32%
MM04T	PRDM2	PR domain containing 2, with ZNF domain	CCDS150.1	chr1_14107541-14107541_T_C	1084V>A	Substitution	Nonsynonymous coding	37%
MM04T	PRDM2	PR domain containing 2, with ZNF domain	CCDS150.1	chr1_14108110-14108110_A_C	1274K>Q	Substitution	Nonsynonymous coding	26%
MM04T	PRDM5	PR domain containing 5	CCDS3716.1	chr4_121738035-121738035_C_T	232R>Q	Substitution	Nonsynonymous coding	29%
MM04T	PRDM6	PR domain containing 6	CCDS47259.1	chr5_122491677-122491677_G_A	334E>K	Substitution	Nonsynonymous coding	30%
MM04T	PRELP	proline/arginine-rich end leucine-rich repeat protein	CCDS1438.1	chr1_203455863-203455863_G_A	335D>N	Substitution	Nonsynonymous coding	32%
MM04T	PRELP	proline/arginine-rich end leucine-rich repeat protein	CCDS1438.1	chr1_203455917-203455917_C_T	353R>C	Substitution	Nonsynonymous coding	23%
MM04T	PREX2	phosphatidylinositol-3,4,5-trisphosphate-dependent Rac exchange factor 2	CCDS6201.1	chr8_68981315-68981315_C_T	463R>C	Substitution	Nonsynonymous coding	30%
MM04T	PREX2	phosphatidylinositol-3,4,5-trisphosphate-dependent Rac exchange factor 2	CCDS6201.1	chr8_68995501-68995501_G_T	635K>N	Substitution	Nonsynonymous coding	28%
MM04T	PRF1	perforin 1 (pore forming protein)	CCDS7305.1	chr10_72358485-72358485_G_A	331S>L	Substitution	Nonsynonymous coding	21%
MM04T	PRG4	proteoglycan 4	CCDS1369.1	chr1_186276573-186276573_G_T	574K>N	Substitution	Nonsynonymous coding	13%
MM04T	PRG4	proteoglycan 4	CCDS1369.1	chr1_186281350-186281350_G_T	1279Q>H	Substitution	Nonsynonymous coding	31%
MM04T	PRICKLE3	prickle homolog 3 (Drosophila)	CCDS14320.1	chrX_49032171-49032171_G_A	567R>C	Substitution	Nonsynonymous coding	42%
MM04T	PRICKLE3	prickle homolog 3 (Drosophila)	CCDS14320.1	chrX_49032543-49032543_G_A	443L>F	Substitution	Nonsynonymous coding	27%
MM04T	PRICKLE4	prickle homolog 4 (Drosophila)	CCDS34449.1	chr6_41752810-41752810_G_T	126K>N	Substitution	Nonsynonymous coding	19%
MM04T	PRKAA2	protein kinase, AMP-activated, alpha 2 catalytic subunit	CCDS605.1	chr1_57173166-57173166_G_T	480R>I	Substitution	Nonsynonymous coding	17%
MM04T	PRKAB2	protein kinase, AMP-activated, beta 2 non-catalytic subunit	CCDS925.1	chr1_146639497-146639497_C_A	58E>X	Substitution	Nonsense	28%
MM04T	PRKACB	protein kinase, cAMP-dependent, catalytic, beta	CCDS693.1	chr1_84649758-84649758_G_T	139E>D	Substitution	Nonsynonymous coding	19%
MM04T	PRKAR2A	protein kinase, cAMP-dependent, regulatory, type II, alpha	CCDS2778.1	chr3_48828006-48828006_C_A	136D>Y	Substitution	Nonsynonymous coding	17%
MM04T	PRKCG	protein kinase C, gamma	CCDS12867.1	chr19_54387447-54387447_G_A	79E>K	Substitution	Nonsynonymous coding	30%
MM04T	PRKCG	protein kinase C, gamma	CCDS12867.1	chr19_54395836-54395836_G_A	254D>N	Substitution	Nonsynonymous coding	27%
MM04T	PRKCG	protein kinase C, gamma	CCDS12867.1	chr19_54401775-54401775_G_A	392D>N	Substitution	Nonsynonymous coding	35%
MM04T	PRKCG	protein kinase C, gamma	CCDS12867.1	chr19_54403487-54403487_G_A	428D>N	Substitution	Nonsynonymous coding	26%
MM04T	PRKCI	protein kinase C, iota	CCDS3212.2	chr3_169999037-169999037_T_G	322F>L	Substitution	Nonsynonymous coding	19%
MM04T	PRKCSH	protein kinase C substrate 80K-H	CCDS32911.1	chr19_11559370-11559370_G_T	397E>D	Substitution	Nonsynonymous coding	21%
MM04T	PRKDC	protein kinase, DNA-activated, catalytic polypeptide	ENST00000314191	chr8_48868484-48868484_T_G	117K>Q	Substitution	Nonsynonymous coding	38%
MM04T	PRKG1	protein kinase, cGMP-dependent, type I	CCDS7244.1	chr10_54041996-54041996_G_T	543E>D	Substitution	Nonsynonymous coding	29%
MM04T	PRKG2	protein kinase, cGMP-dependent, type II	CCDS3589.1	chr4_82013542-82013542_C_T	731E>K	Substitution	Nonsynonymous coding	27%

MM04T	PROCA1	protein interacting with cyclin A1	CCDS11239.1	chr17_27031166-27031166_C_T	141A>T	Substitution	Nonsynonymous coding	33%
MM04T	PROM2	prominin 2	CCDS2012.1	chr2_95940430-95940430_C_A	33L>I	Substitution	Nonsynonymous coding	19%
MM04T	PROX2	prospero homeobox 2	CCDS45136.1	chr14_75321927-75321927_C_T	563D>N	Substitution	Nonsynonymous coding	30%
MM04T	PRPF3	PRP3 pre-mRNA processing factor 3 homolog (S. cerevisiae)	CCDS951.1	chr1_150305616-150305616_G_A	225G>D	Substitution	Nonsynonymous coding	29%
MM04T	PRPF39	PRP39 pre-mRNA processing factor 39 homolog (S. cerevisiae)	CCDS9682.2	chr14_45576688-45576688_G_T	258D>Y	Substitution	Nonsynonymous coding	31%
MM04T	PRPF40A	PRP40 pre-mRNA processing factor 40 homolog A (S. cerevisiae)	CCDS46430.1	chr2_153514443-153514443_G_T	887S>X	Substitution	Nonsense	32%
MM04T	PRPF40B	PRP40 pre-mRNA processing factor 40 homolog B (S. cerevisiae)	CCDS31796.1	chr12_50027835-50027835_G_T	236E>X	Substitution	Nonsense	25%
MM04T	PRPH	peripherin	CCDS8783.1	chr12_49690803-49690803_G_T	278K>N	Substitution	Nonsynonymous coding	32%
MM04T	PRR14L	proline rich 14-like	CCDS13900.2	chr22_32112479-32112479_T_C	449D>G	Substitution	Nonsynonymous coding	37%
MM04T	PRR16	proline rich 16	CCDS4127.1	chr5_120021677-120021677_C_A	40S>Y	Substitution	Nonsynonymous coding	29%
MM04T	PRR25	proline rich 25	CCDS45372.1	chr16_855450-855450_G_A	3R>Q	Substitution	Nonsynonymous coding	38%
MM04T	PRR25	proline rich 25	CCDS45372.1	chr16_855555-855555_G_A	38R>K	Substitution	Nonsynonymous coding	32%
MM04T	PRR9	proline rich 9	ENST00000368744	chr1_153190680-153190680_G_T	20K>N	Substitution	Nonsynonymous coding	19%
MM04T	PRRC2C	proline-rich coiled-coil 2C	NM_015172	chr1_171501615-171501615_G_A	461R>H	Substitution	Nonsynonymous coding	31%
MM04T	PRRC2C	proline-rich coiled-coil 2C	NM_015172	chr1_171511058-171511058_T_G	1483L>V	Substitution	Nonsynonymous coding	31%
MM04T	PRRC2C	proline-rich coiled-coil 2C	NM_015172	chr1_171535513-171535513_G_T	2085E>X	Substitution	Nonsense	28%
MM04T	PRSS12	protease, serine, 12 (neurotrypsin, motopsin)	CCDS3709.1	chr4_119204044-119204044_C_A	754W>C	Substitution	Nonsynonymous coding	35%
MM04T	PRSS38	protease, serine, 38	CCDS1563.1	chr1_228033748-228033748_T_C	274Y>H	Substitution	Nonsynonymous coding	22%
MM04T	PRSS58	protease, serine, 58	CCDS5871.1	chr7_141954995-141954995_T_A	106K>X	Substitution	Nonsense	31%
MM04T	PRTG	protogenin	CCDS42040.1	chr15_55930750-55930750_C_A	817E>X	Substitution	Nonsense	25%
MM04T	PRTG	protogenin	CCDS42040.1	chr15_55965839-55965839_G_A	528R>X	Substitution	Nonsense	34%
MM04T	PRUNE2	prune homolog 2 (Drosophila)	CCDS47982.1	chr9_79324132-79324132_G_A	1020R>X	Substitution	Nonsense	53%
MM04T	PSMA3	proteasome (prosome, macropain) subunit, alpha type, 3	CCDS9731.1	chr14_58737704-58737704_G_T	237E>D	Substitution	Nonsynonymous coding	19%
MM04T	PSMA8	proteasome (prosome, macropain) subunit, alpha type, 8	CCDS32808.1	chr18_23758836-23758836_C_A	180L>I	Substitution	Nonsynonymous coding	28%
MM04T	PSMB2	proteasome (prosome, macropain) subunit, beta type, 2	CCDS394.1	chr1_36101921-36101921_C_A	68K>N	Substitution	Nonsynonymous coding	19%
MM04T	PSMB4	proteasome (prosome, macropain) subunit, beta type, 4	CCDS996.1	chr1_151372596-151372596_A_G	94T>A	Substitution	Nonsynonymous coding	28%
MM04T	PSMB4	proteasome (prosome, macropain) subunit, beta type, 4	CCDS996.1	chr1_151372118-151372118_G_T	19G>X	Substitution	Nonsense	20%
MM04T	PSMC3IP	PSMC3 interacting protein	CCDS45688.1	chr17_40729568-40729568_G_A	16L>F	Substitution	Nonsynonymous coding	34%
MM04T	PSMD12	proteasome (prosome, macropain) 26S subunit, non-ATPase, 12	CCDS11669.1	chr17_65344682-65344682_A_C	165I>S	Substitution	Nonsynonymous coding	29%
MM04T	PSMD2	proteasome (prosome, macropain) 26S subunit, non-ATPase, 2	CCDS3258.1	chr3_184019678-184019678_G_T	175D>Y	Substitution	Nonsynonymous coding	25%
MM04T	PSMD2	proteasome (prosome, macropain) 26S subunit, non-ATPase, 2	CCDS3258.1	chr3_184025298-184025298_G_A	730G>S	Substitution	Nonsynonymous coding	27%
MM04T	PSMD5	proteasome (prosome, macropain) 26S subunit, non-ATPase, 5	CCDS6824.1	chr9_123591452-123591452_G_T	199S>Y	Substitution	Nonsynonymous coding	36%

MM04T	PSME4	proteasome (prosome, macropain) activator subunit 4	CCDS33197.2	chr2_54122789-54122789_C_A	1258R>I	Substitution	Nonsynonymous coding	28%
MM04T	PSTPIP2	proline-serine-threonine phosphatase interacting protein 2	CCDS32820.2	chr18_43572128-43572128_A_C	261I>S	Substitution	Nonsynonymous coding	32%
MM04T	PTAR1	protein prenyltransferase alpha subunit repeat containing 1	CCDS47978.1	chr9_72338300-72338300_C_A	297E>X	Substitution	Nonsense	29%
MM04T	PTBP3	polypyrimidine tract binding protein 3	CCDS6784.1	chr9_115092735-115092735_T_G	10T>P	Substitution	Nonsynonymous coding	17%
MM04T	PTCD1	pentatricopeptide repeat domain 1	CCDS34691.1	chr7_99027402-99027402_C_T	208D>N	Substitution	Nonsynonymous coding	33%
MM04T	PTCD2	pentatricopeptide repeat domain 2	CCDS4014.2	chr5_71634515-71634515_C_A	236L>I	Substitution	Nonsynonymous coding	23%
MM04T	PTCD3	pentatricopeptide repeat domain 3	CCDS33235.1	chr2_86352138-86352138_G_T	246R>I	Substitution	Nonsynonymous coding	24%
MM04T	PTCD3	pentatricopeptide repeat domain 3	CCDS33235.1	chr2_86344286-86344286_A_C	NA	Substitution	Splice site donor	40%
MM04T	PTCH1	patched 1	CCDS6714.1	chr9_98231138-98231138_G_T	715F>L	Substitution	Nonsynonymous coding	23%
MM04T	PTCH1	patched 1	CCDS6714.1	chr9_98239054-98239054_C_A	530R>I	Substitution	Nonsynonymous coding	30%
MM04T	PTCH2	patched 2	CCDS516.1	chr1_45293243-45293243_G_T	734F>L	Substitution	Nonsynonymous coding	17%
MM04T	PTCH2	patched 2	CCDS516.1	chr1_45297836-45297836_G_T	148S>X	Substitution	Nonsense	28%
MM04T	PTCHD3	patched domain containing 3	NM_001034842	chr10_27687454-27687454_T_G	691K>N	Substitution	Nonsynonymous coding	30%
MM04T	PTDSS1	phosphatidylserine synthase 1	CCDS6271.1	chr8_97299342-97299342_C_A	137L>I	Substitution	Nonsynonymous coding	37%
MM04T	PTEN	phosphatase and tensin homolog	NM_000314	chr10_89692954-89692954__A	NA	Insertion	Frameshift	30%
MM04T	PTEN	phosphatase and tensin homolog	NM_000314	chr10_89692905-89692905_G_A	130R>Q	Substitution	Nonsynonymous coding	34%
MM04T	PTEN	phosphatase and tensin homolog	NM_000314	chr10_89692940-89692940_C_T	142R>W	Substitution	Nonsynonymous coding	38%
MM04T	PTGDR	prostaglandin D2 receptor (DP)	CCDS9707.1	chr14_52741617-52741617_T_G	339F>V	Substitution	Nonsynonymous coding	21%
MM04T	PTGES2	prostaglandin E synthase 2	CCDS6891.1	chr9_130883542-130883542_C_T	339G>D	Substitution	Nonsynonymous coding	32%
MM04T	PTGR1	prostaglandin reductase 1	CCDS6779.1	chr9_114332454-114332454_G_A	266R>C	Substitution	Nonsynonymous coding	43%
MM04T	PTH1R	parathyroid hormone 1 receptor	ENST00000313063	chr3_46930456-46930456_A_C	61K>T	Substitution	Nonsynonymous coding	29%
MM04T	PTK2	PTK2 protein tyrosine kinase 2	CCDS6381.1	chr8_141829042-141829042_T_A	242K>N	Substitution	Nonsynonymous coding	25%
MM04T	PTMA	prothymosin, alpha	ENST00000412128	chr2_232575138-232575138_C_T	1A>V	Substitution	Nonsynonymous coding	18%
MM04T	PTPDC1	protein tyrosine phosphatase domain containing 1	CCDS6708.1	chr9_96857730-96857730_C_T	248R>X	Substitution	Nonsense	34%
MM04T	PTPN22	protein tyrosine phosphatase, non-receptor type 22 (lymphoid)	CCDS863.1	chr1_114380728-114380728_T_G	432N>H	Substitution	Nonsynonymous coding	25%
MM04T	PTPN3	protein tyrosine phosphatase, non-receptor type 3	CCDS6776.1	chr9_112182737-112182737_G_T	427S>Y	Substitution	Nonsynonymous coding	16%
MM04T	PTPN4	protein tyrosine phosphatase, non-receptor type 4 (megakaryocyte)	CCDS2129.1	chr2_120677790-120677790_T_G	325F>C	Substitution	Nonsynonymous coding	30%
MM04T	PTPN5	protein tyrosine phosphatase, non-receptor type 5 (striatum-enriched)	CCDS7845.1	chr11_18751236-18751236_G_A	487P>S	Substitution	Nonsynonymous coding	33%
MM04T	PTPN9	protein tyrosine phosphatase, non-receptor type 9	CCDS10280.1	chr15_75763038-75763038_C_A	448E>X	Substitution	Nonsense	35%
MM04T	PTPRA	protein tyrosine phosphatase, receptor type, A	CCDS13038.1	chr20_3008428-3008428_A_G	621I>V	Substitution	Nonsynonymous coding	34%
MM04T	PTPRB	protein tyrosine phosphatase, receptor type, B	CCDS44943.1	chr12_70965609-70965609_C_A	1034R>I	Substitution	Nonsynonymous coding	35%
MM04T	PTPRB	protein tyrosine phosphatase, receptor type, B	CCDS44943.1	chr12_70989867-70989867_C_T	407G>E	Substitution	Nonsynonymous coding	15%

MM04T	PTPRC	protein tyrosine phosphatase, receptor type, C	CCDS1397.1	chr1_198665947-198665947_C_A	67F>L	Substitution	Nonsynonymous coding	36%
MM04T	PTPRD	protein tyrosine phosphatase, receptor type, D	CCDS43786.1	chr9_8389296-8389296_C_A	1441R>I	Substitution	Nonsynonymous coding	21%
MM04T	PTPRD	protein tyrosine phosphatase, receptor type, D	CCDS43786.1	chr9_8518099-8518099_G_A	431S>L	Substitution	Nonsynonymous coding	33%
MM04T	PTPRD	protein tyrosine phosphatase, receptor type, D	CCDS43786.1	chr9_8633353-8633353_C_T	106E>K	Substitution	Nonsynonymous coding	26%
MM04T	PTPRF	protein tyrosine phosphatase, receptor type, F	CCDS489.2	chr1_44070892-44070892_A_G	1056D>G	Substitution	Nonsynonymous coding	21%
MM04T	PTPRG	protein tyrosine phosphatase, receptor type, G	CCDS2895.1	chr3_62177276-62177276_G_T	389K>N	Substitution	Nonsynonymous coding	31%
MM04T	PTPRN	protein tyrosine phosphatase, receptor type, N	CCDS2440.1	chr2_220172550-220172550_G_A	44R>C	Substitution	Nonsynonymous coding	30%
MM04T	PTPRN2	protein tyrosine phosphatase, receptor type, N polypeptide 2	CCDS5947.1	chr7_157475569-157475569_C_T	617A>T	Substitution	Nonsynonymous coding	24%
MM04T	PTPRN2	protein tyrosine phosphatase, receptor type, N polypeptide 2	CCDS5947.1	chr7_157926608-157926608_C_A	439E>D	Substitution	Nonsynonymous coding	24%
MM04T	PTPRQ	protein tyrosine phosphatase, receptor type, Q	NM_001145026	chr12_81062846-81062846_G_T	1913D>Y	Substitution	Nonsynonymous coding	23%
MM04T	PTPRT	protein tyrosine phosphatase, receptor type, T	CCDS42874.1	chr20_41306771-41306771_C_A	296E>D	Substitution	Nonsynonymous coding	31%
MM04T	PTPRU	protein tyrosine phosphatase, receptor type, U	CCDS334.1	chr1_29611288-29611288_C_T	742S>L	Substitution	Nonsynonymous coding	26%
MM04T	PTPRZ1	protein tyrosine phosphatase, receptor-type, Z polypeptide 1	CCDS34740.1	chr7_121612642-121612642_G_A	118E>K	Substitution	Nonsynonymous coding	34%
MM04T	PTX3	pentraxin 3, long	CCDS3180.1	chr3_157160598-157160598_A_G	326T>A	Substitution	Nonsynonymous coding	29%
MM04T	PTX3	pentraxin 3, long	CCDS3180.1	chr3_157160764-157160764_C_A	381S>X	Substitution	Nonsense	23%
MM04T	PUS7	pseudouridylate synthase 7 homolog (S. cerevisiae)	CCDS34725.1	chr7_105142959-105142959_G_T	213S>Y	Substitution	Nonsynonymous coding	26%
MM04T	PVRL1	poliovirus receptor-related 1 (herpesvirus entry mediator C)	CCDS8426.1	chr11_119548526-119548526_G_A	158R>X	Substitution	Nonsense	19%
MM04T	PXDN	peroxidasin homolog (Drosophila)	CCDS46221.1	chr2_1696905-1696905_C_A	68D>Y	Substitution	Nonsynonymous coding	19%
MM04T	PXDNL	peroxidasin homolog (Drosophila)-like	CCDS47855.1	chr8_52321347-52321347_C_T	946R>Q	Substitution	Nonsynonymous coding	20%
MM04T	PYHIN1	pyrin and HIN domain family, member 1	CCDS1178.1	chr1_158908874-158908874_G_T	139R>I	Substitution	Nonsynonymous coding	23%
MM04T	PZP	pregnancy-zone protein	CCDS8600.1	chr12_9346686-9346686_T_G	414K>T	Substitution	Nonsynonymous coding	29%
MM04T	PZP	pregnancy-zone protein	ENST00000381997	chr12_9354982-9354982_C_A	7R>I	Substitution	Nonsynonymous coding	30%
MM04T	QKI	QKI, KH domain containing, RNA binding	CCDS5285.1	chr6_163876310-163876310_G_T	NA	Substitution	Splice site acceptor	33%
MM04T	QPCT	glutaminyl-peptide cyclotransferase	CCDS1790.1	chr2_37596827-37596827_G_T	NA	Substitution	Splice site acceptor	24%
MM04T	QPRT	quinolinate phosphoribosyltransferase	CCDS10651.1	chr16_29706458-29706458_G_A	163D>N	Substitution	Nonsynonymous coding	24%
MM04T	QRICH2	glutamine rich 2	CCDS32741.1	chr17_74300527-74300527_G_A	59A>V	Substitution	Nonsynonymous coding	31%
MM04T	QRICH2	glutamine rich 2	CCDS32741.1	chr17_74300552-74300552_C_T	51V>I	Substitution	Nonsynonymous coding	24%
MM04T	QRSL1	glutaminyl-tRNA synthase (glutamine-hydrolyzing)-like 1	CCDS5057.1	chr6_107090946-107090946_T_G	121L>V	Substitution	Nonsynonymous coding	40%
MM04T	QSOX1	quiescin Q6 sulfhydryl oxidase 1	CCDS1337.1	chr1_180158779-180158779_C_A	370F>L	Substitution	Nonsynonymous coding	32%
MM04T	QSOX1	quiescin Q6 sulfhydryl oxidase 1	CCDS1337.1	chr1_180163428-180163428_G_A	457E>K	Substitution	Nonsynonymous coding	20%
MM04T	QSOX2	quiescin Q6 sulfhydryl oxidase 2	CCDS35178.1	chr9_139113660-139113660_G_A	268S>L	Substitution	Nonsynonymous coding	31%
MM04T	R3HDM1	R3H domain containing 1	ENST00000410054	chr2_136360239-136360239_T_G	32F>L	Substitution	Nonsynonymous coding	38%

MM04T	R3HDM4	R3H domain containing 4	CCDS12048.1	chr19_899617-899617_C_A	211A>S	Substitution	Nonsynonymous coding	22%
MM04T	R3HDML	R3H domain containing-like	CCDS13329.1	chr20_42979340-42979340_C_T	224P>S	Substitution	Nonsynonymous coding	31%
MM04T	RAB11A	RAB11A, member RAS oncogene family	CCDS10212.1	chr15_66169850-66169850_G_A	74R>Q	Substitution	Nonsynonymous coding	27%
MM04T	RAB11FIP1	RAB11 family interacting protein 1 (class I)	CCDS34882.1	chr8_37729433-37729433_G_T	963P>T	Substitution	Nonsynonymous coding	36%
MM04T	RAB38	RAB38, member RAS oncogene family	CCDS8281.1	chr11_87883055-87883055_C_A	91D>Y	Substitution	Nonsynonymous coding	21%
MM04T	RAB3A	RAB3A, member RAS oncogene family	CCDS12372.1	chr19_18308460-18308460_G_T	161F>L	Substitution	Nonsynonymous coding	20%
MM04T	RAB3GAP1	RAB3 GTPase activating protein subunit 1 (catalytic)	CCDS33294.1	chr2_135911363-135911363_T_C	736W>R	Substitution	Nonsynonymous coding	38%
MM04T	RAB3GAP2	RAB3 GTPase activating protein subunit 2 (non-catalytic)	CCDS31028.1	chr1_220330761-220330761_C_A	1136G>X	Substitution	Nonsense	18%
MM04T	RAB43	RAB43, member RAS oncogene family	CCDS33850.1	chr3_128813982-128813982_G_A	79R>W	Substitution	Nonsynonymous coding	31%
MM04T	RAB4B-EGLN2	RAB4B-EGLN2 readthrough (non-protein coding)	CCDS12567.1	chr19_41307031-41307031_G_A	185C>Y	Substitution	Nonsynonymous coding	28%
MM04T	RAB9B	RAB9B, member RAS oncogene family	CCDS14515.1	chrX_103080122-103080122_G_T	198S>Y	Substitution	Nonsynonymous coding	25%
MM04T	RABEPK	Rab9 effector protein with kelch motifs	ENST00000373544	chr9_127982615-127982615_C_A	125S>Y	Substitution	Nonsynonymous coding	32%
MM04T	RABGAP1	RAB GTPase activating protein 1	CCDS6848.2	chr9_125782716-125782716_A_C	591K>T	Substitution	Nonsynonymous coding	21%
MM04T	RAD18	RAD18 homolog (S. cerevisiae)	CCDS2571.1	chr3_8990217-8990217_T_G	57K>N	Substitution	Nonsynonymous coding	32%
MM04T	RAD18	RAD18 homolog (S. cerevisiae)	CCDS2571.1	chr3_8944103-8944103_C_A	377E>X	Substitution	Nonsense	28%
MM04T	RAD23A	RAD23 homolog A (S. cerevisiae)	CCDS12289.1	chr19_13059354-13059354_G_A	154A>T	Substitution	Nonsynonymous coding	19%
MM04T	RAD23B	RAD23 homolog B (S. cerevisiae)	CCDS6769.1	chr9_110087229-110087229_A_C	354S>R	Substitution	Nonsynonymous coding	34%
MM04T	RAD50	RAD50 homolog (S. cerevisiae)	CCDS34233.1	chr5_131923370-131923370_G_T	291E>D	Substitution	Nonsynonymous coding	21%
MM04T	RAD51	RAD51 homolog (S. cerevisiae)	CCDS10062.1	chr15_41001241-41001241_C_A	121S>Y	Substitution	Nonsynonymous coding	23%
MM04T	RAD54B	RAD54 homolog B (S. cerevisiae)	CCDS6262.1	chr8_95392411-95392411_G_T	737L>I	Substitution	Nonsynonymous coding	21%
MM04T	RAD54L2	RAD54-like 2 (S. cerevisiae)	CCDS33765.2	chr3_51624446-51624446_G_A	4E>K	Substitution	Nonsynonymous coding	30%
MM04T	RAI1	retinoic acid induced 1	CCDS11188.1	chr17_17698804-17698804_G_A	848A>T	Substitution	Nonsynonymous coding	18%
MM04T	RAI14	retinoic acid induced 14	CCDS34142.1	chr5_34823188-34823188_C_A	414S>Y	Substitution	Nonsynonymous coding	23%
MM04T	RALGAPA1	Ral GTPase activating protein, alpha subunit 1 (catalytic)	CCDS32064.1	chr14_36039919-36039919_G_A	1961A>V	Substitution	Nonsynonymous coding	37%
MM04T	RALGAPA2	Ral GTPase activating protein, alpha subunit 2 (catalytic)	CCDS46584.1	chr20_20634206-20634206_C_T	99R>Q	Substitution	Nonsynonymous coding	28%
MM04T	RALGAPA2	Ral GTPase activating protein, alpha subunit 2 (catalytic)	CCDS46584.1	chr20_20493548-20493548_C_A	1489G>X	Substitution	Nonsense	29%
MM04T	RALGAPB	Ral GTPase activating protein, beta subunit (non-catalytic)	CCDS13305.1	chr20_37144845-37144845_G_A	295D>N	Substitution	Nonsynonymous coding	37%
MM04T	RALGAPB	Ral GTPase activating protein, beta subunit (non-catalytic)	CCDS13305.1	chr20_37144923-37144923_G_T	321E>X	Substitution	Nonsense	32%
MM04T	RALGDS	ral guanine nucleotide dissociation stimulator	CCDS6959.1	chr9_135981370-135981370_C_A	517E>D	Substitution	Nonsynonymous coding	31%
MM04T	RANBP10	RAN binding protein 10	CCDS32469.1	chr16_67763669-67763669_C_T	354R>Q	Substitution	Nonsynonymous coding	33%
MM04T	RANBP2	RAN binding protein 2	CCDS2079.1	chr2_109389483-109389483__A	NA	Insertion	Frameshift	45%
MM04T	RANBP2	RAN binding protein 2	CCDS2079.1	chr2_109382596-109382596_A_C	1867E>D	Substitution	Nonsynonymous coding	36%

MM04T	RANBP6	RAN binding protein 6	CCDS6467.1	chr9_6013918-6013918_T_C	564K>E	Substitution	Nonsynonymous coding	24%
MM04T	RANGAP1	Ran GTPase activating protein 1	CCDS14012.1	chr22_41650357-41650357_C_A	405E>D	Substitution	Nonsynonymous coding	19%
MM04T	RAP2A	RAP2A, member of RAS oncogene family	CCDS9485.1	chr13_98116524-98116524_C_T	127S>L	Substitution	Nonsynonymous coding	32%
MM04T	RAPGEF2	Rap guanine nucleotide exchange factor (GEF) 2	CCDS43277.1	chr4_160262864-160262864_C_T	734R>C	Substitution	Nonsynonymous coding	32%
MM04T	RAPGEF3	Rap guanine nucleotide exchange factor (GEF) 3	CCDS41775.1	chr12_48131835-48131835_C_T	875E>K	Substitution	Nonsynonymous coding	18%
MM04T	RAPGEF4	Rap guanine nucleotide exchange factor (GEF) 4	CCDS42775.1	chr2_173913371-173913371_G_A	966S>N	Substitution	Nonsynonymous coding	34%
MM04T	RAPGEF5	Rap guanine nucleotide exchange factor (GEF) 5	NM_012294	chr7_22184702-22184702_G_A	563R>X	Substitution	Nonsense	29%
MM04T	RAPGEF6	Rap guanine nucleotide exchange factor (GEF) 6	CCDS34225.1	chr5_130797548-130797548_T_C	905D>G	Substitution	Nonsynonymous coding	36%
MM04T	RAPGEF6	Rap guanine nucleotide exchange factor (GEF) 6	CCDS34225.1	chr5_130788793-130788793_G_A	1052R>X	Substitution	Nonsense	25%
MM04T	RASA1	RAS p21 protein activator (GTPase activating protein) 1	CCDS34200.1	chr5_86645121-86645121_G_A	398R>Q	Substitution	Nonsynonymous coding	23%
MM04T	RASA2	RAS p21 protein activator 2	CCDS3117.1	chr3_141328315-141328315_G_T	760R>I	Substitution	Nonsynonymous coding	37%
MM04T	RASAL3	RAS protein activator like 3	CCDS46006.1	chr19_15562679-15562679_G_T	988S>Y	Substitution	Nonsynonymous coding	27%
MM04T	RASAL3	RAS protein activator like 3	CCDS46006.1	chr19_15567042-15567042_G_T	532L>I	Substitution	Nonsynonymous coding	18%
MM04T	RASAL3	RAS protein activator like 3	CCDS46006.1	chr19_15562879-15562879_C_A	957E>X	Substitution	Nonsense	17%
MM04T	RASGEF1B	RasGEF domain family, member 1B	CCDS34022.1	chr4_82353495-82353495_C_T	452G>E	Substitution	Nonsynonymous coding	28%
MM04T	RASGEF1C	RasGEF domain family, member 1C	CCDS4452.1	chr5_179528507-179528507_C_A	465K>N	Substitution	Nonsynonymous coding	17%
MM04T	RASGEF1C	RasGEF domain family, member 1C	CCDS4452.1	chr5_179529141-179529141_G_T	436L>I	Substitution	Nonsynonymous coding	33%
MM04T	RASGEF1C	RasGEF domain family, member 1C	CCDS4452.1	chr5_179546410-179546410_G_T	281F>L	Substitution	Nonsynonymous coding	19%
MM04T	RASGRP2	RAS guanyl releasing protein 2 (calcium and DAG-regulated)	CCDS31598.1	chr11_64503129-64503129_C_T	394S>N	Substitution	Nonsynonymous coding	37%
MM04T	RASGRP3	RAS guanyl releasing protein 3 (calcium and DAG-regulated)	CCDS46256.1	chr2_33741645-33741645_G_T	36R>I	Substitution	Nonsynonymous coding	14%
MM04T	RASIP1	Ras interacting protein 1	CCDS12731.1	chr19_49227988-49227988_G_A	786S>L	Substitution	Nonsynonymous coding	33%
MM04T	RASIP1	Ras interacting protein 1	CCDS12731.1	chr19_49238667-49238667_G_T	322S>Y	Substitution	Nonsynonymous coding	29%
MM04T	RASIP1	Ras interacting protein 1	CCDS12731.1	chr19_49243400-49243400_C_A	NA	Substitution	Splice site donor	16%
MM04T	RASSF2	Ras association (RalGDS/AF-6) domain family member 2	CCDS13083.1	chr20_4764939-4764939_C_T	321E>K	Substitution	Nonsynonymous coding	23%
MM04T	RASSF4	Ras association (RalGDS/AF-6) domain family member 4	ENST00000374414	chr10_45478435-45478435_C_T	120S>F	Substitution	Nonsynonymous coding	38%
MM04T	RASSF9	Ras association (RalGDS/AF-6) domain family (N-terminal) member 9	CCDS44950.1	chr12_86199529-86199529_C_T	87E>K	Substitution	Nonsynonymous coding	32%
MM04T	RASSF9	Ras association (RalGDS/AF-6) domain family (N-terminal) member 9	CCDS44950.1	chr12_86199738-86199738_G_A	17S>F	Substitution	Nonsynonymous coding	29%
MM04T	RASSF9	Ras association (RalGDS/AF-6) domain family (N-terminal) member 9	CCDS44950.1	chr12_86198722-86198722_C_A	356E>X	Substitution	Nonsense	28%
MM04T	RB1	retinoblastoma 1	CCDS31973.1	chr13_48921968-48921968_G_A	170E>K	Substitution	Nonsynonymous coding	33%
MM04T	RB1	retinoblastoma 1	CCDS31973.1	chr13_49039375-49039375_G_A	787R>Q	Substitution	Nonsynonymous coding	22%
MM04T	RBBP6	retinoblastoma binding protein 6	CCDS10621.1	chr16_24581699-24581699_G_T	1230E>X	Substitution	Nonsense	35%
MM04T	RBFOX2	RNA binding protein, fox-1 homolog (C. elegans) 2	ENST00000359369	chr22_36146497-36146497_G_T	291S>Y	Substitution	Nonsynonymous coding	32%

MM04T	RBL1	retinoblastoma-like 1 (p107)	CCDS13289.1	chr20_35663765-35663765_C_T	684A>T	Substitution	Nonsynonymous coding	24%
MM04T	RBL2	retinoblastoma-like 2 (p130)	CCDS10748.1	chr16_53515716-53515716_T_G	1073I>S	Substitution	Nonsynonymous coding	20%
MM04T	RBM12B	RNA binding motif protein 12B	CCDS43755.1	chr8_94747425-94747425_C_A	405R>I	Substitution	Nonsynonymous coding	30%
MM04T	RBM26	RNA binding motif protein 26	CCDS9462.1	chr13_79945244-79945244_C_T	157R>Q	Substitution	Nonsynonymous coding	30%
MM04T	RBM39	RNA binding motif protein 39	ENST00000397370	chr20_34322866-34322866_A_G	80V>A	Substitution	Nonsynonymous coding	20%
MM04T	RBM41	RNA binding motif protein 41	CCDS14526.1	chrX_106310812-106310812_G_A	396A>V	Substitution	Nonsynonymous coding	22%
MM04T	RBM45	RNA binding motif protein 45	CCDS33335.1	chr2_178990778-178990778_G_A	434D>N	Substitution	Nonsynonymous coding	21%
MM04T	RBM46	RNA binding motif protein 46	CCDS3790.1	chr4_155720090-155720090_T_G	259F>C	Substitution	Nonsynonymous coding	33%
MM04T	RBM48	RNA binding motif protein 48	CCDS43615.1	chr7_92163778-92163778_G_T	171D>Y	Substitution	Nonsynonymous coding	26%
MM04T	RBMS2	RNA binding motif, single stranded interacting protein 2	CCDS8923.1	chr12_56965488-56965488_G_A	131E>K	Substitution	Nonsynonymous coding	30%
MM04T	RBMX	RNA binding motif protein, X-linked	CCDS14661.1	chrX_135961586-135961586__T	NA	Insertion	Frameshift	12%
MM04T	RBP7	retinol binding protein 7, cellular	CCDS109.1	chr1_10067689-10067689_G_T	45Q>H	Substitution	Nonsynonymous coding	24%
MM04T	RBPMS	RNA binding protein with multiple splicing	CCDS34876.1	chr8_30404816-30404816_C_T	179S>F	Substitution	Nonsynonymous coding	39%
MM04T	RC3H1	ring finger and CCCH-type domains 1	CCDS30940.1	chr1_173921242-173921242_T_C	802Y>C	Substitution	Nonsynonymous coding	29%
MM04T	RC3H2	ring finger and CCCH-type domains 2	CCDS43874.1	chr9_125621084-125621084_G_T	716S>Y	Substitution	Nonsynonymous coding	23%
MM04T	RCBTB1	regulator of chromosome condensation (RCC1) and BTB (POZ) domain containing protein 1	CCDS9418.1	chr13_50108264-50108264_C_A	530K>N	Substitution	Nonsynonymous coding	31%
MM04T	RCBTB2	regulator of chromosome condensation (RCC1) and BTB (POZ) domain containing protein 2	CCDS9411.1	chr13_49085916-49085916_C_T	258R>H	Substitution	Nonsynonymous coding	38%
MM04T	RCHY1	ring finger and CHY zinc finger domain containing 1, E3 ubiquitin protein ligase	CCDS3567.1	chr4_76419321-76419321_C_T	132G>E	Substitution	Nonsynonymous coding	31%
MM04T	RCSD1	RCSD domain containing 1	CCDS1263.1	chr1_167653222-167653222_C_T	31A>V	Substitution	Nonsynonymous coding	19%
MM04T	RDH11	retinol dehydrogenase 11 (all-trans/9-cis/11-cis)	CCDS32104.1	chr14_68151909-68151909_G_A	226T>M	Substitution	Nonsynonymous coding	42%
MM04T	RDH8	retinol dehydrogenase 8 (all-trans)	CCDS12223.1	chr19_10132074-10132074_T_G	227L>R	Substitution	Nonsynonymous coding	28%
MM04T	RDX	radixin	CCDS8343.1	chr11_110108287-110108287_C_T	394R>Q	Substitution	Nonsynonymous coding	48%
MM04T	RECQL	RecQ protein-like (DNA helicase Q1-like)	CCDS31756.1	chr12_21639489-21639489_G_T	142S>Y	Substitution	Nonsynonymous coding	24%
MM04T	RECQL4	RecQ protein-like 4	ENST00000428558	chr8_145738802-145738802_G_A	755R>W	Substitution	Nonsynonymous coding	25%
MM04T	RECQL5	RecQ protein-like 5	CCDS42380.1	chr17_73657697-73657697_G_A	288A>V	Substitution	Nonsynonymous coding	30%
MM04T	REN	renin	CCDS30981.1	chr1_204124205-204124205_C_T	387R>Q	Substitution	Nonsynonymous coding	31%
MM04T	REN	renin	CCDS30981.1	chr1_204130528-204130528_C_T	89E>K	Substitution	Nonsynonymous coding	30%
MM04T	REN	renin	CCDS30981.1	chr1_204131277-204131277_C_A	38R>I	Substitution	Nonsynonymous coding	28%
MM04T	REPS1	RALBP1 associated Eps domain containing 1	CCDS5193.2	chr6_139232480-139232480_G_T	666S>Y	Substitution	Nonsynonymous coding	29%
MM04T	REPS1	RALBP1 associated Eps domain containing 1	CCDS5193.2	chr6_139264669-139264669_G_T	321L>I	Substitution	Nonsynonymous coding	25%
MM04T	REPS1	RALBP1 associated Eps domain containing 1	CCDS5193.2	chr6_139232475-139232475_G_A	668R>X	Substitution	Nonsense	29%
MM04T	REPS2	RALBP1 associated Eps domain containing 2	CCDS14180.2	chrX_17080633-17080633_C_A	396P>H	Substitution	Nonsynonymous coding	18%

MM04T	RERE	arginine-glutamic acid dipeptide (RE) repeats	CCDS95.1	chr1_8426017-8426017_G_T	434F>L	Substitution	Nonsynonymous coding	16%
MM04T	REST	RE1-silencing transcription factor	CCDS3509.1	chr4_57797638-57797638_T_G	872L>V	Substitution	Nonsynonymous coding	34%
MM04T	REV1	REV1, polymerase (DNA directed)	CCDS2045.1	chr2_100055239-100055239_C_T	346C>Y	Substitution	Nonsynonymous coding	27%
MM04T	REV3L	REV3-like, polymerase (DNA directed), zeta, catalytic subunit	CCDS5091.2	chr6_111694785-111694785_T_G	1591Q>H	Substitution	Nonsynonymous coding	27%
MM04T	REV3L	REV3-like, polymerase (DNA directed), zeta, catalytic subunit	CCDS5091.2	chr6_111696908-111696908_C_T	884A>T	Substitution	Nonsynonymous coding	27%
MM04T	REXO1	REX1, RNA exonuclease 1 homolog (S. cerevisiae)	CCDS32866.1	chr19_1816761-1816761_C_T	1085D>N	Substitution	Nonsynonymous coding	20%
MM04T	REXO1	REX1, RNA exonuclease 1 homolog (S. cerevisiae)	CCDS32866.1	chr19_1828421-1828421_C_T	123E>K	Substitution	Nonsynonymous coding	33%
MM04T	RFC1	replication factor C (activator 1) 1, 145kDa	CCDS3450.1	chr4_39329146-39329146_C_A	188E>X	Substitution	Nonsense	35%
MM04T	RFC2	replication factor C (activator 1) 2, 40kDa	CCDS5568.1	chr7_73654342-73654342_C_T	207E>K	Substitution	Nonsynonymous coding	19%
MM04T	RFC3	replication factor C (activator 1) 3, 38kDa	CCDS9352.1	chr13_34399927-34399927_G_T	99D>Y	Substitution	Nonsynonymous coding	35%
MM04T	RFTN1	raftlin, lipid raft linker 1	CCDS33712.1	chr3_16358410-16358410_G_T	554H>Q	Substitution	Nonsynonymous coding	21%
MM04T	RFTN2	raftlin family member 2	CCDS2323.1	chr2_198495775-198495775_C_G	296L>F	Substitution	Nonsynonymous coding	34%
MM04T	RFX6	regulatory factor X, 6	CCDS5113.1	chr6_117237440-117237440_T_G	312I>S	Substitution	Nonsynonymous coding	39%
MM04T	RFX8	RFX family member 8, lacking RFX DNA binding domain	CCDS46376.1	chr2_102027101-102027101_G_T	265L>I	Substitution	Nonsynonymous coding	14%
MM04T	RFXAP	regulatory factor X-associated protein	CCDS9359.1	chr13_37401818-37401818_G_T	249Q>H	Substitution	Nonsynonymous coding	19%
MM04T	RGAG4	retrotransposon gag domain containing 4	NM_001024455	chrX_71350454-71350454_C_T	313V>I	Substitution	Nonsynonymous coding	20%
MM04T	RGAG4	retrotransposon gag domain containing 4	NM_001024455	chrX_71351330-71351330_C_A	21E>X	Substitution	Nonsense	21%
MM04T	RGR	retinal G protein coupled receptor	CCDS7374.1	chr10_86018368-86018368_G_T	287Q>H	Substitution	Nonsynonymous coding	24%
MM04T	RGR	retinal G protein coupled receptor	CCDS7374.1	chr10_86018377-86018377_G_T	290E>D	Substitution	Nonsynonymous coding	29%
MM04T	RGS12	regulator of G-protein signaling 12	CCDS3366.1	chr4_3318204-3318204_G_A	103E>K	Substitution	Nonsynonymous coding	33%
MM04T	RGS14	regulator of G-protein signaling 14	CCDS43405.1	chr5_176798489-176798489_G_T	466R>I	Substitution	Nonsynonymous coding	20%
MM04T	RGS18	regulator of G-protein signaling 18	CCDS1374.1	chr1_192153451-192153451_G_T	159E>X	Substitution	Nonsense	34%
MM04T	RGS2	regulator of G-protein signaling 2, 24kDa	CCDS1377.1	chr1_192780144-192780144_C_T	103S>L	Substitution	Nonsynonymous coding	18%
MM04T	RGS3	regulator of G-protein signaling 3	CCDS43869.1	chr9_116224127-116224127_G_A	74R>Q	Substitution	Nonsynonymous coding	32%
MM04T	RGS3	regulator of G-protein signaling 3	CCDS43869.1	chr9_116345947-116345947_C_A	752S>Y	Substitution	Nonsynonymous coding	17%
MM04T	RGS5	regulator of G-protein signaling 5	CCDS1244.1	chr1_163122475-163122475_C_A	83K>N	Substitution	Nonsynonymous coding	32%
MM04T	RGS6	regulator of G-protein signaling 6	CCDS9808.1	chr14_72985078-72985078_G_T	371D>Y	Substitution	Nonsynonymous coding	61%
MM04T	RGS7BP	regulator of G-protein signaling 7 binding protein	CCDS34170.1	chr5_63894255-63894255_A_C	225K>N	Substitution	Nonsynonymous coding	23%
MM04T	RGSL1	regulator of G-protein signaling like 1	NM_001137669	chr1_182429135-182429135_C_A	NA	Substitution	Splice site acceptor	22%
MM04T	RGSL1	regulator of G-protein signaling like 1	NM_001137669	chr1_182499375-182499375_C_A	NA	Substitution	Splice site acceptor	26%
MM04T	RHOD	ras homolog family member D	CCDS8155.1	chr11_66834253-66834253_G_A	89A>T	Substitution	Nonsynonymous coding	30%
MM04T	RHOT2	ras homolog family member T2	CCDS10417.1	chr16_721127-721127_G_A	265E>K	Substitution	Nonsynonymous coding	18%

MM04T	RHOXF1	Rhox homeobox family, member 1	CCDS14593.1	chrX_119249445-119249445_A_C	110F>V	Substitution	Nonsynonymous coding	31%
MM04T	RIC8B	resistance to inhibitors of cholinesterase 8 homolog B (C. elegans)	ENST00000436486	chr12_107264731-107264731_C_T	528P>S	Substitution	Nonsynonymous coding	37%
MM04T	RICTOR	RPTOR independent companion of MTOR, complex 2	CCDS34148.1	chr5_38953633-38953633_C_T	907R>H	Substitution	Nonsynonymous coding	26%
MM04T	RICTOR	RPTOR independent companion of MTOR, complex 2	CCDS34148.1	chr5_38957819-38957819_G_A	812P>S	Substitution	Nonsynonymous coding	21%
MM04T	RICTOR	RPTOR independent companion of MTOR, complex 2	CCDS34148.1	chr5_39002633-39002633_T_C	NA	Substitution	Splice site donor	26%
MM04T	RIMBP2	RIMS binding protein 2	CCDS31925.1	chr12_130898831-130898831_G_T	831L>I	Substitution	Nonsynonymous coding	11%
MM04T	RIMBP2	RIMS binding protein 2	CCDS31925.1	chr12_130921748-130921748_C_A	565R>L	Substitution	Nonsynonymous coding	19%
MM04T	RIMS1	regulating synaptic membrane exocytosis 1	CCDS47449.1	chr6_72943483-72943483_G_T	562L>F	Substitution	Nonsynonymous coding	23%
MM04T	RIMS2	regulating synaptic membrane exocytosis 2	CCDS43761.1	chr8_105025783-105025783_A_G	867N>S	Substitution	Nonsynonymous coding	34%
MM04T	RINT1	RAD50 interactor 1	CCDS34726.1	chr7_105204357-105204357_G_T	617E>X	Substitution	Nonsense	41%
MM04T	RIPK2	receptor-interacting serine-threonine kinase 2	CCDS6247.1	chr8_90802523-90802523_T_G	501F>C	Substitution	Nonsynonymous coding	37%
MM04T	RIPK3	receptor-interacting serine-threonine kinase 3	CCDS9628.1	chr14_24805602-24805602_C_T	NA	Substitution	Splice site acceptor	33%
MM04T	RLIM	ring finger protein, LIM domain interacting	CCDS14427.1	chrX_73812275-73812275_C_A	292R>I	Substitution	Nonsynonymous coding	25%
MM04T	RMDN2	regulator of microtubule dynamics 2	CCDS1792.1	chr2_38178836-38178836_G_A	160E>K	Substitution	Nonsynonymous coding	28%
MM04T	RMDN2	regulator of microtubule dynamics 2	CCDS1792.1	chr2_38178975-38178975_C_T	206A>V	Substitution	Nonsynonymous coding	35%
MM04T	RMDN2	regulator of microtubule dynamics 2	ENST00000406963	chr2_38244343-38244343_G_T	487Q>H	Substitution	Nonsynonymous coding	19%
MM04T	RMND5B	required for meiotic nuclear division 5 homolog B (S. cerevisiae)	CCDS4431.1	chr5_177573133-177573133_G_A	238G>D	Substitution	Nonsynonymous coding	27%
MM04T	RNASE9	ribonuclease, RNase A family, 9 (non-active)	CCDS32036.1	chr14_21024970-21024970_T_G	87I>L	Substitution	Nonsynonymous coding	24%
MM04T	RNASEL	ribonuclease L (2',5'-oligoisoadenylate synthetase-dependent)	CCDS1347.1	chr1_182544637-182544637_T_G	706K>Q	Substitution	Nonsynonymous coding	35%
MM04T	RNF128	ring finger protein 128, E3 ubiquitin protein ligase	CCDS14520.1	chrX_105937275-105937275_A_C	15I>L	Substitution	Nonsynonymous coding	30%
MM04T	RNF128	ring finger protein 128, E3 ubiquitin protein ligase	CCDS14521.1	chrX_105970341-105970341_C_A	66S>R	Substitution	Nonsynonymous coding	33%
MM04T	RNF138	ring finger protein 138, E3 ubiquitin protein ligase	CCDS11903.1	chr18_29691863-29691863_G_T	86C>F	Substitution	Nonsynonymous coding	16%
MM04T	RNF139	ring finger protein 139	CCDS6350.1	chr8_125499506-125499506_G_A	539R>H	Substitution	Nonsynonymous coding	32%
MM04T	RNF148	ring finger protein 148	CCDS47692.1	chr7_122342273-122342273_C_A	178E>X	Substitution	Nonsense	38%
MM04T	RNF167	ring finger protein 167	CCDS11060.1	chr17_4845732-4845732_C_T	88R>X	Substitution	Nonsense	23%
MM04T	RNF168	ring finger protein 168, E3 ubiquitin protein ligase	CCDS3317.1	chr3_196199531-196199531_G_T	292S>Y	Substitution	Nonsynonymous coding	18%
MM04T	RNF17	ring finger protein 17	CCDS9308.2	chr13_25352439-25352439_G_T	108K>N	Substitution	Nonsynonymous coding	27%
MM04T	RNF17	ring finger protein 17	CCDS9308.2	chr13_25362269-25362269_C_A	252S>Y	Substitution	Nonsynonymous coding	25%
MM04T	RNF17	ring finger protein 17	CCDS9308.2	chr13_25418929-25418929_A_G	991T>A	Substitution	Nonsynonymous coding	22%
MM04T	RNF175	ring finger protein 175	CCDS47149.1	chr4_154672600-154672600_C_A	32D>Y	Substitution	Nonsynonymous coding	31%
MM04T	RNF207	ring finger protein 207	CCDS59.2	chr1_6266716-6266716_T_G	41F>V	Substitution	Nonsynonymous coding	22%
MM04T	RNF213	ring finger protein 213	CCDS11772.1	chr17_78263601-78263601_A_C	359K>N	Substitution	Nonsynonymous coding	14%

MM04T	RNF219	ring finger protein 219	CCDS31997.1	chr13_79191201-79191201_T_C	232E>G	Substitution	Nonsynonymous coding	31%
MM04T	RNF219	ring finger protein 219	CCDS31997.1	chr13_79219006-79219006_C_A	67E>X	Substitution	Nonsense	25%
MM04T	RNF32	ring finger protein 32	CCDS5944.1	chr7_156451242-156451242_G_T	221R>I	Substitution	Nonsynonymous coding	26%
MM04T	RNF6	ring finger protein (C3H2C3 type) 6	CCDS9316.1	chr13_26788214-26788214_C_T	602R>Q	Substitution	Nonsynonymous coding	35%
MM04T	RNF6	ring finger protein (C3H2C3 type) 6	CCDS9316.1	chr13_26788215-26788215_G_A	602R>X	Substitution	Nonsense	32%
MM04T	RNF8	ring finger protein 8, E3 ubiquitin protein ligase	CCDS4834.1	chr6_37336452-37336452_G_T	145E>X	Substitution	Nonsense	12%
MM04T	RNFT1	ring finger protein, transmembrane 1	CCDS11622.2	chr17_58034719-58034719_C_A	291E>X	Substitution	Nonsense	32%
MM04T	RNGTT	RNA guanylyltransferase and 5'-phosphatase	CCDS5017.1	chr6_89638724-89638724_C_A	116R>I	Substitution	Nonsynonymous coding	27%
MM04T	ROBO1	roundabout, axon guidance receptor, homolog 1 (Drosophila)	CCDS46872.1	chr3_78676616-78676616_G_A	1205R>W	Substitution	Nonsynonymous coding	29%
MM04T	ROBO2	roundabout, axon guidance receptor, homolog 2 (Drosophila)	CCDS43109.1	chr3_77607107-77607107_G_T	415R>I	Substitution	Nonsynonymous coding	41%
MM04T	ROBO2	roundabout, axon guidance receptor, homolog 2 (Drosophila)	CCDS43109.1	chr3_77671487-77671487_G_A	1222D>N	Substitution	Nonsynonymous coding	27%
MM04T	ROBO4	roundabout, axon guidance receptor, homolog 4 (Drosophila)	CCDS8455.1	chr11_124766503-124766503_G_A	155T>I	Substitution	Nonsynonymous coding	17%
MM04T	ROR1	receptor tyrosine kinase-like orphan receptor 1	CCDS626.1	chr1_64624793-64624793_C_T	435S>L	Substitution	Nonsynonymous coding	31%
MM04T	ROS1	c-ros oncogene 1 , receptor tyrosine kinase	CCDS5116.1	chr6_117639383-117639383_C_A	1991K>N	Substitution	Nonsynonymous coding	40%
MM04T	ROS1	c-ros oncogene 1 , receptor tyrosine kinase	CCDS5116.1	chr6_117674241-117674241_C_A	1411K>N	Substitution	Nonsynonymous coding	25%
MM04T	RP1	retinitis pigmentosa 1 (autosomal dominant)	CCDS6160.1	chr8_55538363-55538363_A_G	641R>G	Substitution	Nonsynonymous coding	14%
MM04T	RP1	retinitis pigmentosa 1 (autosomal dominant)	CCDS6160.1	chr8_55538595-55538595_A_C	718D>A	Substitution	Nonsynonymous coding	38%
MM04T	RP1	retinitis pigmentosa 1 (autosomal dominant)	CCDS6160.1	chr8_55540850-55540850_G_T	1470E>X	Substitution	Nonsense	41%
MM04T	RP11-108K14.4	Putative scavenger receptor cysteine-rich domain-containing protein LOC619207	ENST00000333815	chr10_135269966-135269966_G_A	98R>Q	Substitution	Nonsynonymous coding	32%
MM04T	RP11-108K14.4	Putative scavenger receptor cysteine-rich domain-containing protein LOC619207	ENST00000333815	chr10_135279785-135279785_C_A	902F>L	Substitution	Nonsynonymous coding	31%
MM04T	RP11-121L10.3	-	ENST00000318973	chr11_90016189-90016189_C_T	122R>C	Substitution	Nonsynonymous coding	26%
MM04T	RP11-351E7.2	-	ENST00000391672	chr8_73342131-73342131_G_T	92K>N	Substitution	Nonsynonymous coding	31%
MM04T	RP11-455G16.1	Uncharacterized protein [Source:UniProtKB/TrEMBL;Acc:E7EQP2]	ENST00000326780	chr4_120116700-120116700_G_T	91L>I	Substitution	Nonsynonymous coding	18%
MM04T	RP11-707M1.1	-	ENST00000357667	chr11_49597952-49597952_T_C	51V>A	Substitution	Nonsynonymous coding	41%
MM04T	RP11-722P11.6	-	ENST00000328207	chr12_48954387-48954387_C_A	236C>X	Substitution	Nonsense	32%
MM04T	RP11-723O4.6	Uncharacterized protein FLJ43738 [Source:UniProtKB/Swiss-Prot;Acc:Q6ZUG5]	ENST00000344062	chr3_128664589-128664589_A_G	74F>S	Substitution	Nonsynonymous coding	23%
MM04T	RP1L1	retinitis pigmentosa 1-like 1	CCDS43708.1	chr8_10480645-10480645_G_A	23R>C	Substitution	Nonsynonymous coding	24%
MM04T	RP3-368B9.1	Uncharacterized protein [Source:UniProtKB/TrEMBL;Acc:E7ETJ0]	ENST00000404649	chr4_3589701-3589701_G_T	23Q>H	Substitution	Nonsynonymous coding	11%
MM04T	RPE65	retinal pigment epithelium-specific protein 65kDa	CCDS643.1	chr1_68896769-68896769_C_A	477D>Y	Substitution	Nonsynonymous coding	17%
MM04T	RPGRIP1L	RPGRIP1-like	CCDS32447.1	chr16_53653105-53653105_G_A	1150R>W	Substitution	Nonsynonymous coding	36%
MM04T	RPGRIP1L	RPGRIP1-like	CCDS32447.1	chr16_53691371-53691371_A_C	525D>E	Substitution	Nonsynonymous coding	33%
MM04T	RPL17	ribosomal protein L17	CCDS45865.1	chr18_47016847-47016847_C_T	98A>T	Substitution	Nonsynonymous coding	29%

MM04T	RPL27	ribosomal protein L27	CCDS11449.1	chr17_41154904-41154904_G_T	126K>N	Substitution	Nonsynonymous coding	43%
MM04T	RPL3L	ribosomal protein L3-like	CCDS10450.1	chr16_2000865-2000865_G_A	161R>W	Substitution	Nonsynonymous coding	32%
MM04T	RPN2	ribophorin II	CCDS13291.1	chr20_35860711-35860711_G_A	532E>K	Substitution	Nonsynonymous coding	29%
MM04T	RPP14	ribonuclease P/MRP 14kDa subunit	CCDS2888.1	chr3_58296303-58296303_G_T	49K>N	Substitution	Nonsynonymous coding	19%
MM04T	RPP14	ribonuclease P/MRP 14kDa subunit	CCDS2888.1	chr3_58302306-58302306_G_T	76R>I	Substitution	Nonsynonymous coding	24%
MM04T	RPP25L	ribonuclease P/MRP 25kDa subunit-like	CCDS6559.1	chr9_34610819-34610819_G_A	159R>X	Substitution	Nonsense	23%
MM04T	RPP40	ribonuclease P/MRP 40kDa subunit	CCDS34333.1	chr6_5002341-5002341_T_C	88K>E	Substitution	Nonsynonymous coding	36%
MM04T	RPS6KA1	ribosomal protein S6 kinase, 90kDa, polypeptide 1	CCDS30649.1	chr1_26873692-26873692_C_T	89R>W	Substitution	Nonsynonymous coding	25%
MM04T	RPS6KA1	ribosomal protein S6 kinase, 90kDa, polypeptide 1	CCDS30649.1	chr1_26887302-26887302_G_A	443R>H	Substitution	Nonsynonymous coding	21%
MM04T	RPS6KA3	ribosomal protein S6 kinase, 90kDa, polypeptide 3	CCDS14197.1	chrX_20179781-20179781_G_T	647S>Y	Substitution	Nonsynonymous coding	39%
MM04T	RPS6KA3	ribosomal protein S6 kinase, 90kDa, polypeptide 3	CCDS14197.1	chrX_20183109-20183109_G_A	558R>X	Substitution	Nonsense	16%
MM04T	RPS6KA6	ribosomal protein S6 kinase, 90kDa, polypeptide 6	CCDS14451.1	chrX_83403078-83403078_C_A	104M>I	Substitution	Nonsynonymous coding	33%
MM04T	RPS6KL1	ribosomal protein S6 kinase-like 1	CCDS9834.1	chr14_75388145-75388145_G_A	34R>C	Substitution	Nonsynonymous coding	22%
MM04T	RPTOR	regulatory associated protein of MTOR, complex 1	CCDS11773.1	chr17_78882621-78882621_T_G	804F>L	Substitution	Nonsynonymous coding	26%
MM04T	RRAGB	Ras-related GTP binding B	CCDS14372.1	chrX_55744846-55744846_C_T	29S>L	Substitution	Nonsynonymous coding	25%
MM04T	RRBP1	ribosome binding protein 1	CCDS13128.1	chr20_17595521-17595521_C_A	919R>I	Substitution	Nonsynonymous coding	19%
MM04T	RRBP1	ribosome binding protein 1	ENST00000246043	chr20_17639379-17639379_C_T	592A>T	Substitution	Nonsynonymous coding	25%
MM04T	RRBP1	ribosome binding protein 1	CCDS13128.1	chr20_17640733-17640733_C_A	140K>N	Substitution	Nonsynonymous coding	37%
MM04T	RSAD2	radical S-adenosyl methionine domain containing 2	CCDS1656.1	chr2_7033818-7033818_T_G	302L>R	Substitution	Nonsynonymous coding	31%
MM04T	RSBN1	round spermatid basic protein 1	CCDS862.1	chr1_114308864-114308864_C_A	716S>I	Substitution	Nonsynonymous coding	21%
MM04T	RSF1	remodeling and spacing factor 1	CCDS8253.1	chr11_77396175-77396175_C_A	977S>I	Substitution	Nonsynonymous coding	35%
MM04T	RSRC1	arginine/serine-rich coiled-coil 1	CCDS3181.1	chr3_157841711-157841711_G_A	84R>Q	Substitution	Nonsynonymous coding	27%
MM04T	RTCA	RNA 3'-terminal phosphate cyclase	CCDS44178.1	chr1_100738975-100738975_T_G	160F>C	Substitution	Nonsynonymous coding	42%
MM04T	RTEL1-TNFRSF6B	RTEL1-TNFRSF6B readthrough (non-protein coding)	CCDS13530.2	chr20_62319098-62319098_T_C	486S>P	Substitution	Nonsynonymous coding	15%
MM04T	RTF1	Rtf1, Paf1/RNA polymerase II complex component, homolog (S. cerevisiae)	CCDS32200.2	chr15_41772424-41772424_G_T	643D>Y	Substitution	Nonsynonymous coding	30%
MM04T	RTL1	retrotransposon-like 1	NM_001134888	chr14_101347575-101347575_C_T	1184R>Q	Substitution	Nonsynonymous coding	24%
MM04T	RTP1	receptor (chemosensory) transporter protein 1	CCDS3287.2	chr3_186917626-186917626_G_A	187R>H	Substitution	Nonsynonymous coding	32%
MM04T	RTP2	receptor (chemosensory) transporter protein 2	CCDS33911.1	chr3_187419779-187419779_C_A	46Q>H	Substitution	Nonsynonymous coding	42%
MM04T	RTTN	rotatin	CCDS42443.1	chr18_67759986-67759986_T_G	1320K>Q	Substitution	Nonsynonymous coding	35%
MM04T	RUFY2	RUN and FYVE domain containing 2	CCDS44414.1	chr10_70138333-70138333_C_A	400D>Y	Substitution	Nonsynonymous coding	33%
MM04T	RUFY3	RUN and FYVE domain containing 3	CCDS34001.1	chr4_71650576-71650576_G_T	351E>X	Substitution	Nonsense	30%
MM04T	RUNDC1	RUN domain containing 1	CCDS11448.1	chr17_41139324-41139324_G_T	184E>X	Substitution	Nonsense	29%

MM04T	RUNDC3A	RUN domain containing 3A	CCDS45698.1	chr17_42389983-42389983_C_T	48A>V	Substitution	Nonsynonymous coding	17%
MM04T	RUNDC3B	RUN domain containing 3B	CCDS5609.1	chr7_87280163-87280163_C_T	50R>W	Substitution	Nonsynonymous coding	23%
MM04T	RUNX1T1	runt-related transcription factor 1; translocated to, 1 (cyclin D-related)	CCDS6256.1	chr8_92998514-92998514_G_A	373R>X	Substitution	Nonsense	18%
MM04T	RUSC2	RUN and SH3 domain containing 2	CCDS35008.1	chr9_35548205-35548205_C_T	563R>C	Substitution	Nonsynonymous coding	29%
MM04T	RUSC2	RUN and SH3 domain containing 2	CCDS35008.1	chr9_35558533-35558533_C_T	1104R>C	Substitution	Nonsynonymous coding	19%
MM04T	RUSC2	RUN and SH3 domain containing 2	CCDS35008.1	chr9_35560815-35560815_G_A	1393R>Q	Substitution	Nonsynonymous coding	35%
MM04T	RXFP2	relaxin/insulin-like family peptide receptor 2	CCDS9342.1	chr13_32355863-32355863_G_A	281D>N	Substitution	Nonsynonymous coding	23%
MM04T	RXRG	retinoid X receptor, gamma	CCDS1248.1	chr1_165398114-165398114_C_A	47D>Y	Substitution	Nonsynonymous coding	21%
MM04T	RYR1	ryanodine receptor 1 (skeletal)	CCDS33011.1	chr19_38951046-38951046_G_T	798E>X	Substitution	Nonsense	28%
MM04T	RYR2	ryanodine receptor 2 (cardiac)	NM_001035	chr1_237777638-237777638_C_T	1737T>I	Substitution	Nonsynonymous coding	19%
MM04T	RYR2	ryanodine receptor 2 (cardiac)	NM_001035	chr1_237787107-237787107_C_T	1987P>S	Substitution	Nonsynonymous coding	30%
MM04T	RYR2	ryanodine receptor 2 (cardiac)	NM_001035	chr1_237841403-237841403_C_A	2962F>L	Substitution	Nonsynonymous coding	28%
MM04T	RYR3	ryanodine receptor 3	CCDS45210.1	chr15_33765676-33765676_C_A	36F>L	Substitution	Nonsynonymous coding	26%
MM04T	S100A5	S100 calcium binding protein A5	CCDS1041.1	chr1_153509894-153509894_C_T	71D>N	Substitution	Nonsynonymous coding	34%
MM04T	S100Z	S100 calcium binding protein Z	CCDS43333.1	chr5_76173562-76173562_G_A	69E>K	Substitution	Nonsynonymous coding	21%
MM04T	SAAL1	serum amyloid A-like 1	CCDS31439.1	chr11_18111002-18111002_C_A	215E>D	Substitution	Nonsynonymous coding	24%
MM04T	SACM1L	SAC1 suppressor of actin mutations 1-like (yeast)	CCDS33745.1	chr3_45785084-45785084_A_C	563T>P	Substitution	Nonsynonymous coding	20%
MM04T	SACS	spastic ataxia of Charlevoix-Saguenay (sacsin)	CCDS9300.2	chr13_23904307-23904307_T_G	4570K>Q	Substitution	Nonsynonymous coding	36%
MM04T	SACS	spastic ataxia of Charlevoix-Saguenay (sacsin)	CCDS9300.2	chr13_23905152-23905152_G_T	4288T>N	Substitution	Nonsynonymous coding	25%
MM04T	SACS	spastic ataxia of Charlevoix-Saguenay (sacsin)	CCDS9300.2	chr13_23909485-23909485_C_T	2844D>N	Substitution	Nonsynonymous coding	21%
MM04T	SACS	spastic ataxia of Charlevoix-Saguenay (sacsin)	CCDS9300.2	chr13_23909751-23909751_C_A	2755S>I	Substitution	Nonsynonymous coding	18%
MM04T	SACS	spastic ataxia of Charlevoix-Saguenay (sacsin)	CCDS9300.2	chr13_23913271-23913271_C_T	1582D>N	Substitution	Nonsynonymous coding	32%
MM04T	SAG	S-antigen; retina and pineal gland (arrestin)	CCDS46545.1	chr2_234255535-234255535_G_A	399D>N	Substitution	Nonsynonymous coding	19%
MM04T	SALL1	sal-like 1 (Drosophila)	CCDS10747.1	chr16_51172696-51172696_G_A	1146S>L	Substitution	Nonsynonymous coding	23%
MM04T	SAMD15	sterile alpha motif domain containing 15	CCDS32126.1	chr14_77844665-77844665_A_G	302T>A	Substitution	Nonsynonymous coding	23%
MM04T	SAMD15	sterile alpha motif domain containing 15	CCDS32126.1	chr14_77845256-77845256_G_T	499E>X	Substitution	Nonsense	21%
MM04T	SAMD3	sterile alpha motif domain containing 3	CCDS34539.1	chr6_130497110-130497110_C_T	233R>Q	Substitution	Nonsynonymous coding	35%
MM04T	SAMD3	sterile alpha motif domain containing 3	CCDS34539.1	chr6_130530683-130530683_T_G	114N>H	Substitution	Nonsynonymous coding	28%
MM04T	SAMD5	sterile alpha motif domain containing 5	CCDS34548.1	chr6_147830474-147830474_A_C	137K>T	Substitution	Nonsynonymous coding	14%
MM04T	SAMD9	sterile alpha motif domain containing 9	CCDS34680.1	chr7_92733282-92733282_T_G	710K>T	Substitution	Nonsynonymous coding	21%
MM04T	SAMD9L	sterile alpha motif domain containing 9-like	CCDS34681.1	chr7_92762496-92762496_G_T	930S>Y	Substitution	Nonsynonymous coding	22%
MM04T	SAMD9L	sterile alpha motif domain containing 9-like	CCDS34681.1	chr7_92763831-92763831_G_A	485S>F	Substitution	Nonsynonymous coding	22%

MM04T	SAMM50	sorting and assembly machinery component 50 homolog (S. cerevisiae)	CCDS14055.1	chr22_44372633-44372633_G_A	261A>T	Substitution	Nonsynonymous coding	36%
MM04T	SAMSN1	SAM domain, SH3 domain and nuclear localization signals 1	CCDS42906.1	chr21_15872989-15872989_T_G	210K>T	Substitution	Nonsynonymous coding	25%
MM04T	SAMSN1	SAM domain, SH3 domain and nuclear localization signals 1	CCDS42906.1	chr21_15893509-15893509_G_A	31R>W	Substitution	Nonsynonymous coding	18%
MM04T	SARDH	sarcosine dehydrogenase	CCDS6978.1	chr9_136573504-136573504_G_A	459R>X	Substitution	Nonsense	17%
MM04T	SASH1	SAM and SH3 domain containing 1	CCDS5212.1	chr6_148795275-148795275_A_C	182I>L	Substitution	Nonsynonymous coding	29%
MM04T	SASS6	spindle assembly 6 homolog (C. elegans)	CCDS764.1	chr1_100573257-100573257_T_G	358N>T	Substitution	Nonsynonymous coding	36%
MM04T	SASS6	spindle assembly 6 homolog (C. elegans)	CCDS764.1	chr1_100575922-100575922_C_A	263D>Y	Substitution	Nonsynonymous coding	25%
MM04T	SAT1	spermidine/spermine N1-acetyltransferase 1	CCDS14207.1	chrX_23803899-23803899_C_A	148L>M	Substitution	Nonsynonymous coding	18%
MM04T	SATB1	SATB homeobox 1	CCDS2631.1	chr3_18428081-18428081_C_T	410R>Q	Substitution	Nonsynonymous coding	30%
MM04T	SBDS	Shwachman-Bodian-Diamond syndrome	CCDS5537.1	chr7_66453479-66453479_A_G	211L>P	Substitution	Nonsynonymous coding	16%
MM04T	SBK2	SH3-binding domain kinase family, member 2	CCDS42631.1	chr19_56041257-56041257_G_A	297A>V	Substitution	Nonsynonymous coding	16%
MM04T	SCAF11	SR-related CTD-associated factor 11	CCDS8748.2	chr12_46320342-46320342_C_T	1048E>K	Substitution	Nonsynonymous coding	29%
MM04T	SCAF11	SR-related CTD-associated factor 11	CCDS8748.2	chr12_46325298-46325298_C_T	278E>K	Substitution	Nonsynonymous coding	22%
MM04T	SCAF4	SR-related CTD-associated factor 4	CCDS33537.1	chr21_33044070-33044070_T_G	1029D>A	Substitution	Nonsynonymous coding	31%
MM04T	SCAF4	SR-related CTD-associated factor 4	CCDS33537.1	chr21_33064672-33064672_T_G	535E>A	Substitution	Nonsynonymous coding	27%
MM04T	SCAF4	SR-related CTD-associated factor 4	CCDS33537.1	chr21_33067122-33067122_T_G	NA	Substitution	Splice site donor	47%
MM04T	SCAF8	SR-related CTD-associated factor 8	CCDS5247.1	chr6_155153171-155153171_G_A	820V>I	Substitution	Nonsynonymous coding	26%
MM04T	SCAF8	SR-related CTD-associated factor 8	CCDS5247.1	chr6_155148378-155148378_A_C	NA	Substitution	Splice site donor	27%
MM04T	SCAMP1	secretory carrier membrane protein 1	ENST00000451676	chr5_77771406-77771406_A_C	311K>T	Substitution	Nonsynonymous coding	29%
MM04T	SCAND3	SCAN domain containing 3	CCDS34355.1	chr6_28542738-28542738_G_T	582L>I	Substitution	Nonsynonymous coding	15%
MM04T	SCAP	SREBF chaperone	CCDS2755.2	chr3_47462120-47462120_C_T	496R>Q	Substitution	Nonsynonymous coding	18%
MM04T	SCARA5	scavenger receptor class A, member 5 (putative)	CCDS6064.1	chr8_27737093-27737093_G_T	448F>L	Substitution	Nonsynonymous coding	21%
MM04T	SCCPDH	saccharopine dehydrogenase (putative)	CCDS31084.1	chr1_246921655-246921655_G_T	231R>I	Substitution	Nonsynonymous coding	26%
MM04T	SCEL	sciellin	CCDS9459.1	chr13_78176806-78176806_C_T	332S>F	Substitution	Nonsynonymous coding	25%
MM04T	SCG2	secretogranin II	CCDS2457.1	chr2_224462560-224462560_C_A	481A>S	Substitution	Nonsynonymous coding	25%
MM04T	SCGB1D1	secretoglobin, family 1D, member 1	CCDS8015.1	chr11_61959570-61959570_G_A	33G>D	Substitution	Nonsynonymous coding	34%
MM04T	SCGB1D1	secretoglobin, family 1D, member 1	CCDS8015.1	chr11_61959698-61959698_C_A	76L>I	Substitution	Nonsynonymous coding	21%
MM04T	SCGB1D4	secretoglobin, family 1D, member 4	CCDS31583.1	chr11_62065064-62065064_G_A	41A>V	Substitution	Nonsynonymous coding	36%
MM04T	SCLT1	sodium channel and clathrin linker 1	CCDS3740.1	chr4_129878248-129878248_T_C	360D>G	Substitution	Nonsynonymous coding	39%
MM04T	SCML1	sex comb on midleg-like 1 (Drosophila)	CCDS35210.1	chrX_17768103-17768103_C_A	131F>L	Substitution	Nonsynonymous coding	33%
MM04T	SCML2	sex comb on midleg-like 2 (Drosophila)	CCDS14185.1	chrX_18259388-18259388_C_A	696E>X	Substitution	Nonsense	33%
MM04T	SCN10A	sodium channel, voltage-gated, type X, alpha subunit	CCDS33736.1	chr3_38738884-38738884_C_A	1943E>X	Substitution	Nonsense	28%

MM04T	SCN10A	sodium channel, voltage-gated, type X, alpha subunit	CCDS33736.1	chr3_38743480-38743480_C_A	1503E>X	Substitution	Nonsense	28%
MM04T	SCN11A	sodium channel, voltage-gated, type XI, alpha subunit	CCDS33737.1	chr3_38889102-38889102_G_T	1487L>I	Substitution	Nonsynonymous coding	29%
MM04T	SCN11A	sodium channel, voltage-gated, type XI, alpha subunit	CCDS33737.1	chr3_38945472-38945472_C_T	576D>N	Substitution	Nonsynonymous coding	16%
MM04T	SCN1B	sodium channel, voltage-gated, type I, beta subunit	CCDS46047.1	chr19_35523506-35523506_C_A	39L>I	Substitution	Nonsynonymous coding	29%
MM04T	SCN2A	sodium channel, voltage-gated, type II, alpha subunit	CCDS33313.1	chr2_166245943-166245943_G_A	1876R>Q	Substitution	Nonsynonymous coding	23%
MM04T	SCN3A	sodium channel, voltage-gated, type III, alpha subunit	CCDS33312.1	chr2_165946813-165946813_A_C	1950N>K	Substitution	Nonsynonymous coding	47%
MM04T	SCN4A	sodium channel, voltage-gated, type IV, alpha subunit	CCDS45761.1	chr17_62019110-62019110_G_A	1511S>L	Substitution	Nonsynonymous coding	20%
MM04T	SCN4A	sodium channel, voltage-gated, type IV, alpha subunit	CCDS45761.1	chr17_62034853-62034853_G_A	682S>L	Substitution	Nonsynonymous coding	30%
MM04T	SCN5A	sodium channel, voltage-gated, type V, alpha subunit	CCDS46796.1	chr3_38628908-38628908_G_T	807L>M	Substitution	Nonsynonymous coding	23%
MM04T	SCN5A	sodium channel, voltage-gated, type V, alpha subunit	CCDS46796.1	chr3_38646368-38646368_G_T	457S>Y	Substitution	Nonsynonymous coding	18%
MM04T	SCN5A	sodium channel, voltage-gated, type V, alpha subunit	CCDS46796.1	chr3_38663915-38663915_G_T	153P>H	Substitution	Nonsynonymous coding	21%
MM04T	SCN7A	sodium channel, voltage-gated, type VII, alpha subunit	CCDS46442.1	chr2_167289066-167289066_T_C	785Y>C	Substitution	Nonsynonymous coding	25%
MM04T	SCN7A	sodium channel, voltage-gated, type VII, alpha subunit	CCDS46442.1	chr2_167289247-167289247_G_T	725L>M	Substitution	Nonsynonymous coding	19%
MM04T	SCN7A	sodium channel, voltage-gated, type VII, alpha subunit	CCDS46442.1	chr2_167313475-167313475_C_A	399E>X	Substitution	Nonsense	16%
MM04T	SCN9A	sodium channel, voltage-gated, type IX, alpha subunit	CCDS46441.1	chr2_167168182-167168182_C_T	29E>K	Substitution	Nonsynonymous coding	28%
MM04T	SCN9A	sodium channel, voltage-gated, type IX, alpha subunit	CCDS46441.1	chr2_167168211-167168211_G_T	19S>Y	Substitution	Nonsynonymous coding	22%
MM04T	SCNN1B	sodium channel, non-voltage-gated 1, beta subunit	CCDS10609.1	chr16_23391473-23391473_G_T	509E>X	Substitution	Nonsense	17%
MM04T	SCNN1G	sodium channel, non-voltage-gated 1, gamma subunit	CCDS10608.1	chr16_23224023-23224023_G_A	440R>Q	Substitution	Nonsynonymous coding	18%
MM04T	SCRN1	secernin 1	CCDS47567.1	chr7_29963582-29963582_G_T	432F>L	Substitution	Nonsynonymous coding	27%
MM04T	SCUBE1	signal peptide, CUB domain, EGF-like 1	CCDS14048.1	chr22_43610257-43610257_A_C	631V>G	Substitution	Nonsynonymous coding	17%
MM04T	SCUBE1	signal peptide, CUB domain, EGF-like 1	CCDS14048.1	chr22_43614370-43614370_G_T	594F>L	Substitution	Nonsynonymous coding	22%
MM04T	SCUBE3	signal peptide, CUB domain, EGF-like 3	CCDS4800.1	chr6_35210053-35210053_G_A	497R>Q	Substitution	Nonsynonymous coding	19%
MM04T	SEBOX	SEBOX homeobox	CCDS11229.1	chr17_26694479-26694479_G_A	450R>C	Substitution	Nonsynonymous coding	16%
MM04T	SEC14L1	SEC14-like 1 (S. cerevisiae)	CCDS42385.1	chr17_75202817-75202817_C_T	450P>L	Substitution	Nonsynonymous coding	26%
MM04T	SEC14L4	SEC14-like 4 (S. cerevisiae)	CCDS13878.1	chr22_30886146-30886146_G_T	390S>Y	Substitution	Nonsynonymous coding	26%
MM04T	SEC14L4	SEC14-like 4 (S. cerevisiae)	CCDS13878.1	chr22_30887605-30887605_T_C	346M>V	Substitution	Nonsynonymous coding	31%
MM04T	SEC61A1	Sec61 alpha 1 subunit (S. cerevisiae)	CCDS3046.1	chr3_127783794-127783794_C_T	231R>W	Substitution	Nonsynonymous coding	24%
MM04T	SEC63	SEC63 homolog (S. cerevisiae)	CCDS5061.1	chr6_108227773-108227773_T_G	280E>D	Substitution	Nonsynonymous coding	29%
MM04T	SEC63	SEC63 homolog (S. cerevisiae)	CCDS5061.1	chr6_108246033-108246033_T_G	110N>H	Substitution	Nonsynonymous coding	38%
MM04T	SEC63	SEC63 homolog (S. cerevisiae)	CCDS5061.1	chr6_108250710-108250710_G_A	45R>X	Substitution	Nonsense	35%
MM04T	SECISBP2L	SECIS binding protein 2-like	ENST00000380927	chr15_49311644-49311644_C_T	381D>N	Substitution	Nonsynonymous coding	40%
MM04T	SEL1L	sel-1 suppressor of lin-12-like (C. elegans)	CCDS9876.1	chr14_81972537-81972537_G_T	130P>H	Substitution	Nonsynonymous coding	27%

MM04T	SEL1L3	sel-1 suppressor of lin-12-like 3 (C. elegans)	CCDS47037.1	chr4_25834646-25834646_G_A	357R>X	Substitution	Nonsense	22%
MM04T	SELENBP1	selenium binding protein 1	CCDS995.1	chr1_151338253-151338253_C_T	305E>K	Substitution	Nonsynonymous coding	27%
MM04T	SELRC1	Sel1 repeat containing 1	CCDS570.1	chr1_53158480-53158480_T_C	56K>E	Substitution	Nonsynonymous coding	41%
MM04T	SEMA3A	sema domain, immunoglobulin domain (Ig), short basic domain, secreted, (semaphorin) 3A	CCDS5599.1	chr7_83764206-83764206_A_T	58H>Q	Substitution	Nonsynonymous coding	10%
MM04T	SEMA3C	sema domain, immunoglobulin domain (Ig), short basic domain, secreted, (semaphorin) 3C	CCDS5596.1	chr7_80457917-80457917_C_A	83S>I	Substitution	Nonsynonymous coding	28%
MM04T	SEMA3G	sema domain, immunoglobulin domain (Ig), short basic domain, secreted, (semaphorin) 3G	CCDS2856.1	chr3_52469816-52469816_T_C	718N>D	Substitution	Nonsynonymous coding	38%
MM04T	SEMA5B	sema domain, seven thrombospondin repeats (type 1 and type 1-like), transmembrane	CCDS35491.1	chr3_122632466-122632466_C_T	732S>N	Substitution	Nonsynonymous coding	21%
MM04T	SEMA6A	sema domain, transmembrane domain (TM), and cytoplasmic domain, (semaphorin) 6A	CCDS47256.1	chr5_115840552-115840552_G_A	30S>L	Substitution	Nonsynonymous coding	28%
MM04T	SEMA6C	sema domain, transmembrane domain (TM), and cytoplasmic domain, (semaphorin) 6C	CCDS984.1	chr1_151104982-151104982_T_C	924N>S	Substitution	Nonsynonymous coding	40%
MM04T	SEPHS2	selenophosphate synthetase 2	ENST00000418751	chr16_30456632-30456632_G_T	21F>L	Substitution	Nonsynonymous coding	31%
MM04T	SERINC5	serine incorporator 5	NM_178276	chr5_79473787-79473787_C_T	99A>T	Substitution	Nonsynonymous coding	20%
MM04T	SERPINA6	serpin peptidase inhibitor, clade A (alpha-1 antiproteinase, antitrypsin), member 6	CCDS9924.1	chr14_94780532-94780532_C_A	152E>X	Substitution	Nonsense	24%
MM04T	SERPINB5	serpin peptidase inhibitor, clade B (ovalbumin), member 5	CCDS32839.1	chr18_61170754-61170754_G_T	309E>D	Substitution	Nonsynonymous coding	31%
MM04T	SERPINB6	serpin peptidase inhibitor, clade B (ovalbumin), member 6	CCDS4479.1	chr6_2953355-2953355_C_A	166A>S	Substitution	Nonsynonymous coding	19%
MM04T	SERPINB7	serpin peptidase inhibitor, clade B (ovalbumin), member 7	CCDS11988.1	chr18_61463524-61463524_T_G	121L>V	Substitution	Nonsynonymous coding	34%
MM04T	SERPINB7	serpin peptidase inhibitor, clade B (ovalbumin), member 7	CCDS11988.1	chr18_61471552-61471552_T_G	276F>V	Substitution	Nonsynonymous coding	29%
MM04T	SERPINB8	serpin peptidase inhibitor, clade B (ovalbumin), member 8	CCDS11991.1	chr18_61650848-61650848_G_A	154D>N	Substitution	Nonsynonymous coding	26%
MM04T	SERPINB8	serpin peptidase inhibitor, clade B (ovalbumin), member 8	CCDS11991.1	chr18_61652468-61652468_G_A	237D>N	Substitution	Nonsynonymous coding	16%
MM04T	SERPINE3	serpin peptidase inhibitor, clade E (nexin, plasminogen activator inhibitor type 1),	NM_001101320	chr13_51922447-51922447_G_T	267D>Y	Substitution	Nonsynonymous coding	31%
MM04T	SESN1	sestrin 1	CCDS5070.1	chr6_109311999-109311999_G_A	425R>C	Substitution	Nonsynonymous coding	38%
MM04T	SETD1A	SET domain containing 1A	CCDS32435.1	chr16_30982716-30982716_G_A	1012D>N	Substitution	Nonsynonymous coding	37%
MM04T	SETD2	SET domain containing 2	CCDS2749.2	chr3_47098523-47098523_C_A	2251D>Y	Substitution	Nonsynonymous coding	20%
MM04T	SETD2	SET domain containing 2	CCDS2749.2	chr3_47098631-47098631_C_T	2215E>K	Substitution	Nonsynonymous coding	28%
MM04T	SETD2	SET domain containing 2	CCDS2749.2	chr3_47163667-47163667_G_T	820S>Y	Substitution	Nonsynonymous coding	42%
MM04T	SETD2	SET domain containing 2	CCDS2749.2	chr3_47163941-47163941_C_A	729E>X	Substitution	Nonsense	28%
MM04T	SETD3	SET domain containing 3	CCDS9951.1	chr14_99925516-99925516_C_A	118E>X	Substitution	Nonsense	34%
MM04T	SETD5	SET domain containing 5	ENST00000421188	chr3_9510257-9510257_G_A	238R>Q	Substitution	Nonsynonymous coding	28%
MM04T	SETD5	SET domain containing 5	CCDS46741.1	chr3_9517339-9517339_C_T	1298S>F	Substitution	Nonsynonymous coding	21%
MM04T	SETDB1	SET domain, bifurcated 1	CCDS44217.1	chr1_150923391-150923391_T_G	680L>V	Substitution	Nonsynonymous coding	32%
MM04T	SETX	senataxin	CCDS6947.1	chr9_135203690-135203690_C_T	1099D>N	Substitution	Nonsynonymous coding	30%
MM04T	SETX	senataxin	CCDS6947.1	chr9_135204164-135204164_C_A	941E>X	Substitution	Nonsense	28%
MM04T	SF1	splicing factor 1	CCDS31599.1	chr11_64535714-64535714_T_G	311K>T	Substitution	Nonsynonymous coding	23%

MM04T	SF3A1	splicing factor 3a, subunit 1, 120kDa	CCDS13875.1	chr22_30736338-30736338_C_A	408D>Y	Substitution	Nonsynonymous coding	34%
MM04T	SF3B1	splicing factor 3b, subunit 1, 155kDa	CCDS33356.1	chr2_198285764-198285764_A_G	97S>P	Substitution	Nonsynonymous coding	34%
MM04T	SFPQ	splicing factor proline/glutamine-rich	CCDS388.1	chr1_35652831-35652831_C_T	613G>S	Substitution	Nonsynonymous coding	35%
MM04T	SFTPA1	surfactant protein A1	CCDS44445.1	chr10_81372088-81372088_G_T	65E>X	Substitution	Nonsense	35%
MM04T	SFTPD	surfactant protein D	CCDS7362.1	chr10_81697815-81697815_C_A	307K>N	Substitution	Nonsynonymous coding	16%
MM04T	SGCG	sarcoglycan, gamma (35kDa dystrophin-associated glycoprotein)	CCDS9299.1	chr13_23777928-23777928_G_T	32R>I	Substitution	Nonsynonymous coding	16%
MM04T	SGCG	sarcoglycan, gamma (35kDa dystrophin-associated glycoprotein)	CCDS9299.1	chr13_23894851-23894851_T_G	218I>M	Substitution	Nonsynonymous coding	38%
MM04T	SGIP1	SH3-domain GRB2-like (endophilin) interacting protein 1	ENST00000237247	chr1_67099823-67099823_G_A	54A>T	Substitution	Nonsynonymous coding	19%
MM04T	SGIP1	SH3-domain GRB2-like (endophilin) interacting protein 1	ENST00000371039	chr1_67160169-67160169_C_T	341R>C	Substitution	Nonsynonymous coding	28%
MM04T	SGK110	putative uncharacterized serine/threonine-protein kinase SgK110 [Source:RefSeq	ENST00000429736	chr19_56052745-56052745_C_A	183D>Y	Substitution	Nonsynonymous coding	23%
MM04T	SGPL1	sphingosine-1-phosphate lyase 1	CCDS31216.1	chr10_72628151-72628151_G_A	222R>Q	Substitution	Nonsynonymous coding	25%
MM04T	SGSM1	small G protein signaling modulator 1	CCDS46674.1	chr22_25294076-25294076_C_A	775F>L	Substitution	Nonsynonymous coding	19%
MM04T	SGSM2	small G protein signaling modulator 2	CCDS32526.1	chr17_2267414-2267414_C_A	290T>N	Substitution	Nonsynonymous coding	31%
MM04T	SH2D1A	SH2 domain containing 1A	CCDS14608.1	chrX_123504164-123504164_A_G	114T>A	Substitution	Nonsynonymous coding	35%
MM04T	SH2D3C	SH2 domain containing 3C	CCDS6877.1	chr9_130507114-130507114_G_A	510A>V	Substitution	Nonsynonymous coding	20%
MM04T	SH2D4A	SH2 domain containing 4A	CCDS6009.1	chr8_19231137-19231137_G_T	338Q>H	Substitution	Nonsynonymous coding	29%
MM04T	SH2D4A	SH2 domain containing 4A	CCDS6009.1	chr8_19231040-19231040_G_T	NA	Substitution	Splice site acceptor	29%
MM04T	SH2D7	SH2 domain containing 7	CCDS45315.1	chr15_78390312-78390312_G_A	103R>Q	Substitution	Nonsynonymous coding	30%
MM04T	SH3BP5	SH3-domain binding protein 5 (BTK-associated)	CCDS2625.2	chr3_15298407-15298407_C_T	368R>Q	Substitution	Nonsynonymous coding	39%
MM04T	SH3D21	SH3 domain containing 21	NM_001162530	chr1_36772798-36772798_G_T	86R>I	Substitution	Nonsynonymous coding	28%
MM04T	SH3GL3	SH3-domain GRB2-like 3	CCDS10325.2	chr15_84286902-84286902_G_T	303E>X	Substitution	Nonsense	24%
MM04T	SH3GLB2	SH3-domain GRB2-like endophilin B2	CCDS6916.1	chr9_131784604-131784604_C_A	54E>D	Substitution	Nonsynonymous coding	30%
MM04T	SH3PXD2A	SH3 and PX domains 2A	ENST00000369774	chr10_105382229-105382229_C_T	267R>Q	Substitution	Nonsynonymous coding	28%
MM04T	SH3TC2	SH3 domain and tetratricopeptide repeats 2	CCDS4293.1	chr5_148406657-148406657_C_T	880A>T	Substitution	Nonsynonymous coding	19%
MM04T	SH3YL1	SH3 domain containing, Ysc84-like 1 (S. cerevisiae)	CCDS42646.1	chr2_218858-218858_G_A	232R>X	Substitution	Nonsense	14%
MM04T	SHANK1	SH3 and multiple ankyrin repeat domains 1	CCDS12799.1	chr19_51175280-51175280_G_T	890S>Y	Substitution	Nonsynonymous coding	11%
MM04T	SHC2	SHC (Src homology 2 domain containing) transforming protein 2	CCDS45891.1	chr19_438791-438791_C_T	216R>H	Substitution	Nonsynonymous coding	15%
MM04T	SHC4	SHC (Src homology 2 domain containing) family, member 4	CCDS10130.1	chr15_49217091-49217091_C_A	214R>I	Substitution	Nonsynonymous coding	27%
MM04T	SHKBP1	SH3KBP1 binding protein 1	CCDS12560.1	chr19_41084099-41084099_C_A	100F>L	Substitution	Nonsynonymous coding	32%
MM04T	SHPRH	SNF2 histone linker PHD RING helicase, E3 ubiquitin protein ligase	CCDS43513.1	chr6_146268712-146268712_G_T	377L>I	Substitution	Nonsynonymous coding	25%
MM04T	SHPRH	SNF2 histone linker PHD RING helicase, E3 ubiquitin protein ligase	CCDS43513.1	chr6_146276148-146276148_G_A	104S>F	Substitution	Nonsynonymous coding	42%
MM04T	SHROOM2	shroom family member 2	CCDS14135.1	chrX_9866242-9866242_G_A	935E>K	Substitution	Nonsynonymous coding	32%

MM04T	SHROOM3	shroom family member 3	CCDS3579.2	chr4_77662204-77662204_C_T	960R>W	Substitution	Nonsynonymous coding	36%
MM04T	SHROOM4	shroom family member 4	CCDS35277.1	chrX_50377340-50377340_G_A	578S>L	Substitution	Nonsynonymous coding	33%
MM04T	SI	sucrase-isomaltase (alpha-glucosidase)	CCDS3196.1	chr3_164767021-164767021_T_C	537K>E	Substitution	Nonsynonymous coding	29%
MM04T	SI	sucrase-isomaltase (alpha-glucosidase)	CCDS3196.1	chr3_164700787-164700787_T_A	NA	Substitution	Splice site donor	30%
MM04T	SIAH3	siah E3 ubiquitin protein ligase family member 3	CCDS41883.1	chr13_46358026-46358026_G_A	101P>L	Substitution	Nonsynonymous coding	34%
MM04T	SIGLEC12	sialic acid binding Ig-like lectin 12 (gene/pseudogene)	CCDS12833.1	chr19_52003244-52003244_G_T	246Y>X	Substitution	Nonsense	34%
MM04T	SIGLEC14	sialic acid binding Ig-like lectin 14	CCDS42604.1	chr19_52147129-52147129_C_A	305E>D	Substitution	Nonsynonymous coding	18%
MM04T	SIK3	SIK family kinase 3	CCDS8379.1	chr11_116732585-116732585_G_A	657P>S	Substitution	Nonsynonymous coding	28%
MM04T	SIM1	single-minded homolog 1 (Drosophila)	CCDS5045.1	chr6_100838268-100838268_C_A	757G>V	Substitution	Nonsynonymous coding	23%
MM04T	SIN3A	SIN3 transcription regulator homolog A (yeast)	CCDS10279.1	chr15_75699454-75699454_G_T	450S>Y	Substitution	Nonsynonymous coding	18%
MM04T	SIN3B	SIN3 transcription regulator homolog B (yeast)	CCDS32946.1	chr19_16952581-16952581_G_T	128E>D	Substitution	Nonsynonymous coding	25%
MM04T	SIPA1	signal-induced proliferation-associated 1	CCDS8108.1	chr11_65413719-65413719_G_A	431D>N	Substitution	Nonsynonymous coding	34%
MM04T	SIPA1	signal-induced proliferation-associated 1	CCDS8108.1	chr11_65408999-65408999_C_T	203R>X	Substitution	Nonsense	20%
MM04T	SIPA1L1	signal-induced proliferation-associated 1 like 1	CCDS9807.1	chr14_72117181-72117181_C_T	650R>W	Substitution	Nonsynonymous coding	23%
MM04T	SIPA1L2	signal-induced proliferation-associated 1 like 2	CCDS41474.1	chr1_232615429-232615429_C_T	677E>K	Substitution	Nonsynonymous coding	39%
MM04T	SIRT1	sirtuin 1	CCDS7273.1	chr10_69667863-69667863_G_A	384R>Q	Substitution	Nonsynonymous coding	28%
MM04T	SIX3	SIX homeobox 3	CCDS1821.1	chr2_45169658-45169658_G_A	139A>T	Substitution	Nonsynonymous coding	38%
MM04T	SKIDA1	SKI/DACH domain containing 1	CCDS44363.1	chr10_21804695-21804695_T_G	686K>T	Substitution	Nonsynonymous coding	33%
MM04T	SKIDA1	SKI/DACH domain containing 1	CCDS44363.1	chr10_21805096-21805096_C_A	552E>D	Substitution	Nonsynonymous coding	34%
MM04T	SKIL	SKI-like oncogene	CCDS33890.1	chr3_170078648-170078648_G_T	177E>X	Substitution	Nonsense	30%
MM04T	SKOR2	SKI family transcriptional corepressor 2	ENST00000425639	chr18_44775120-44775120_G_T	145F>L	Substitution	Nonsynonymous coding	29%
MM04T	SLA	Src-like-adaptor	CCDS47923.1	chr8_134060122-134060122_T_C	142D>G	Substitution	Nonsynonymous coding	30%
MM04T	SLAIN1	SLAIN motif family, member 1	CCDS31995.1	chr13_78335026-78335026_G_A	252R>Q	Substitution	Nonsynonymous coding	32%
MM04T	SLAMF1	signaling lymphocytic activation molecule family member 1	CCDS1207.1	chr1_160607157-160607157_G_T	80S>Y	Substitution	Nonsynonymous coding	20%
MM04T	SLBP	stem-loop binding protein	CCDS3350.1	chr4_1701772-1701772_C_A	103D>Y	Substitution	Nonsynonymous coding	25%
MM04T	SLC12A1	solute carrier family 12 (sodium/potassium/chloride transporters),	CCDS10129.2	chr15_48541810-48541810_T_A	575F>I	Substitution	Nonsynonymous coding	23%
MM04T	SLC12A1	solute carrier family 12 (sodium/potassium/chloride transporters),	CCDS10129.2	chr15_48593563-48593563_G_T	1050A>S	Substitution	Nonsynonymous coding	26%
MM04T	SLC12A2	solute carrier family 12 (sodium/potassium/chloride transporters),	CCDS4144.1	chr5_127516587-127516587_T_C	1038L>S	Substitution	Nonsynonymous coding	32%
MM04T	SLC12A5	solute carrier family 12 (potassium/chloride transporter), member 5	CCDS46610.1	chr20_44684896-44684896_G_T	988E>D	Substitution	Nonsynonymous coding	30%
MM04T	SLC12A6	solute carrier family 12 (potassium/chloride transporters), member 6	CCDS42011.1	chr15_34628815-34628815_C_T	14D>N	Substitution	Nonsynonymous coding	24%
MM04T	SLC12A7	solute carrier family 12 (potassium/chloride transporters), member 7	CCDS34129.1	chr5_1060557-1060557_C_A	917D>Y	Substitution	Nonsynonymous coding	29%
MM04T	SLC12A7	solute carrier family 12 (potassium/chloride transporters), member 7	CCDS34129.1	chr5_1077994-1077994_C_T	528R>H	Substitution	Nonsynonymous coding	23%

MM04T	SLC15A1	solute carrier family 15 (oligopeptide transporter), member 1	CCDS9489.1	chr13_99337070-99337070_C_T	679E>K	Substitution	Nonsynonymous coding	24%
MM04T	SLC15A1	solute carrier family 15 (oligopeptide transporter), member 1	ENST00000376494	chr13_99373370-99373370_G_T	280S>Y	Substitution	Nonsynonymous coding	30%
MM04T	SLC15A5	solute carrier family 15, member 5	ENST00000344941	chr12_16410689-16410689_C_T	234A>T	Substitution	Nonsynonymous coding	33%
MM04T	SLC16A4	solute carrier family 16, member 4 (monocarboxylic acid transporter 5)	CCDS823.1	chr1_110921789-110921789_T_C	239D>G	Substitution	Nonsynonymous coding	35%
MM04T	SLC16A9	solute carrier family 16, member 9 (monocarboxylic acid transporter 9)	CCDS7256.1	chr10_61424014-61424014_C_T	136R>Q	Substitution	Nonsynonymous coding	27%
MM04T	SLC17A1	solute carrier family 17 (sodium phosphate), member 1	CCDS4565.1	chr6_25813426-25813426_G_A	211A>V	Substitution	Nonsynonymous coding	21%
MM04T	SLC17A2	solute carrier family 17 (sodium phosphate), member 2	CCDS4567.1	chr6_25916044-25916044_A_G	328L>S	Substitution	Nonsynonymous coding	26%
MM04T	SLC17A2	solute carrier family 17 (sodium phosphate), member 2	CCDS4567.1	chr6_25924138-25924138_G_T	NA	Substitution	Splice site acceptor	41%
MM04T	SLC17A3	solute carrier family 17 (sodium phosphate), member 3	CCDS47385.1	chr6_25850279-25850279_A_C	374L>V	Substitution	Nonsynonymous coding	24%
MM04T	SLC17A6	solute carrier family 17 (sodium-dependent inorganic phosphate cotransporter), member 6	CCDS7856.1	chr11_22396395-22396395_T_G	379L>R	Substitution	Nonsynonymous coding	35%
MM04T	SLC18A1	solute carrier family 18 (vesicular monoamine), member 1	CCDS6013.1	chr8_20022397-20022397_G_A	333S>F	Substitution	Nonsynonymous coding	36%
MM04T	SLC18A1	solute carrier family 18 (vesicular monoamine), member 1	CCDS6013.1	chr8_20022617-20022617_C_T	287S>N	Substitution	Nonsynonymous coding	26%
MM04T	SLC18A1	solute carrier family 18 (vesicular monoamine), member 1	CCDS6013.1	chr8_20031905-20031905_G_T	200Q>K	Substitution	Nonsynonymous coding	27%
MM04T	SLC18A1	solute carrier family 18 (vesicular monoamine), member 1	CCDS6013.1	chr8_20036715-20036715_C_A	135E>D	Substitution	Nonsynonymous coding	31%
MM04T	SLC18A1	solute carrier family 18 (vesicular monoamine), member 1	CCDS6013.1	chr8_20036941-20036941_G_T	60S>Y	Substitution	Nonsynonymous coding	16%
MM04T	SLC18A2	solute carrier family 18 (vesicular monoamine), member 2	CCDS7599.1	chr10_119003781-119003781_G_A	141V>I	Substitution	Nonsynonymous coding	22%
MM04T	SLC19A1	solute carrier family 19 (folate transporter), member 1	CCDS13725.1	chr21_46945809-46945809_G_T	405F>L	Substitution	Nonsynonymous coding	28%
MM04T	SLC19A3	solute carrier family 19, member 3	CCDS2468.1	chr2_228564031-228564031_C_T	134E>K	Substitution	Nonsynonymous coding	18%
MM04T	SLC19A3	solute carrier family 19, member 3	CCDS2468.1	chr2_228564208-228564208_C_T	75D>N	Substitution	Nonsynonymous coding	22%
MM04T	SLC1A1	solute carrier family 1 (neuronal/epithelial high affinity glutamate transporter, system Xag),	CCDS6452.1	chr9_4576026-4576026_C_A	301L>I	Substitution	Nonsynonymous coding	16%
MM04T	SLC1A3	solute carrier family 1 (glial high affinity glutamate transporter), member 3	CCDS3919.1	chr5_36676998-36676998_A_C	191K>T	Substitution	Nonsynonymous coding	44%
MM04T	SLC1A6	solute carrier family 1 (high affinity aspartate/glutamate transporter), member 6	CCDS12321.1	chr19_15083562-15083562_C_T	54R>H	Substitution	Nonsynonymous coding	28%
MM04T	SLC20A1	solute carrier family 20 (phosphate transporter), member 1	CCDS2099.1	chr2_113416967-113416967_T_A	412L>X	Substitution	Nonsense	23%
MM04T	SLC22A10	solute carrier family 22, member 10	CCDS41661.1	chr11_63067096-63067096_T_A	355F>L	Substitution	Nonsynonymous coding	24%
MM04T	SLC22A10	solute carrier family 22, member 10	CCDS41661.1	chr11_63078489-63078489_C_A	537L>I	Substitution	Nonsynonymous coding	39%
MM04T	SLC22A15	solute carrier family 22, member 15	CCDS44198.1	chr1_116534775-116534775_G_A	71A>T	Substitution	Nonsynonymous coding	22%
MM04T	SLC22A15	solute carrier family 22, member 15	CCDS44198.1	chr1_116574156-116574156_C_T	300R>C	Substitution	Nonsynonymous coding	18%
MM04T	SLC22A16	solute carrier family 22 (organic cation/carnitine transporter), member 16	CCDS5084.1	chr6_110746104-110746104_G_A	569T>I	Substitution	Nonsynonymous coding	25%
MM04T	SLC22A20	solute carrier family 22, member 20	NM_001004326	chr11_65000815-65000815_C_T	413A>V	Substitution	Nonsynonymous coding	36%
MM04T	SLC22A24	solute carrier family 22, member 24	ENST00000417740	chr11_62902206-62902206_G_T	146S>X	Substitution	Nonsense	34%
MM04T	SLC22A3	solute carrier family 22 (extraneuronal monoamine transporter), member 3	CCDS5277.1	chr6_160857854-160857854_G_A	340D>N	Substitution	Nonsynonymous coding	30%
MM04T	SLC22A3	solute carrier family 22 (extraneuronal monoamine transporter), member 3	CCDS5277.1	chr6_160864676-160864676_C_T	471S>L	Substitution	Nonsynonymous coding	26%

MM04T	SLC24A2	solute carrier family 24 (sodium/potassium/calcium exchanger),	CCDS6493.1	chr9_19786675-19786675_T_C	64T>A	Substitution	Nonsynonymous coding	26%
MM04T	SLC24A2	solute carrier family 24 (sodium/potassium/calcium exchanger),	CCDS6493.1	chr9_19786147-19786147_G_A	240R>X	Substitution	Nonsense	33%
MM04T	SLC25A12	solute carrier family 25 (aspartate/glutamate carrier), member 12	CCDS33327.1	chr2_172641873-172641873_C_T	650E>K	Substitution	Nonsynonymous coding	34%
MM04T	SLC25A13	solute carrier family 25 (aspartate/glutamate carrier), member 13	CCDS5645.1	chr7_95864126-95864126_C_A	106E>X	Substitution	Nonsense	29%
MM04T	SLC25A21	solute carrier family 25 (mitochondrial oxoadipate carrier), member 21	CCDS9663.1	chr14_37283181-37283181_C_T	49D>N	Substitution	Nonsynonymous coding	14%
MM04T	SLC25A23	solute carrier family 25 (mitochondrial carrier; phosphate carrier), member 23	CCDS32882.1	chr19_6454349-6454349_G_T	260F>L	Substitution	Nonsynonymous coding	27%
MM04T	SLC25A24	solute carrier family 25 (mitochondrial carrier; phosphate carrier), member 24	CCDS41361.1	chr1_108724600-108724600_T_G	121T>P	Substitution	Nonsynonymous coding	27%
MM04T	SLC25A27	solute carrier family 25, member 27	CCDS43470.1	chr6_46644184-46644184_C_T	322P>L	Substitution	Nonsynonymous coding	31%
MM04T	SLC25A3	solute carrier family 25 (mitochondrial carrier; phosphate carrier), member 3	CCDS9066.1	chr12_98993807-98993807_G_T	240R>L	Substitution	Nonsynonymous coding	27%
MM04T	SLC25A30	solute carrier family 25, member 30	CCDS31967.1	chr13_45983133-45983133_A_C	47I>M	Substitution	Nonsynonymous coding	35%
MM04T	SLC25A31	solute carrier family 25 (mitochondrial carrier; adenine nucleotide translocator), member 31	CCDS3733.1	chr4_128694702-128694702_C_A	307F>L	Substitution	Nonsynonymous coding	32%
MM04T	SLC25A48	solute carrier family 25, member 48	ENST00000420621	chr5_135215704-135215704_G_T	287M>I	Substitution	Nonsynonymous coding	12%
MM04T	SLC26A10	solute carrier family 26, member 10	CCDS8949.2	chr12_58017819-58017819_C_T	389R>C	Substitution	Nonsynonymous coding	30%
MM04T	SLC26A11	solute carrier family 26, member 11	CCDS11771.2	chr17_78215614-78215614_G_A	344A>T	Substitution	Nonsynonymous coding	35%
MM04T	SLC26A3	solute carrier family 26, member 3	CCDS5748.1	chr7_107408083-107408083_C_T	738D>N	Substitution	Nonsynonymous coding	38%
MM04T	SLC26A3	solute carrier family 26, member 3	CCDS5748.1	chr7_107431609-107431609_G_A	152R>C	Substitution	Nonsynonymous coding	29%
MM04T	SLC26A4	solute carrier family 26, member 4	CCDS5746.1	chr7_107350568-107350568_T_G	720L>W	Substitution	Nonsynonymous coding	16%
MM04T	SLC26A7	solute carrier family 26, member 7	CCDS6255.1	chr8_92346655-92346655_C_A	259L>I	Substitution	Nonsynonymous coding	39%
MM04T	SLC26A7	solute carrier family 26, member 7	CCDS6255.1	chr8_92350421-92350421_A_G	280N>S	Substitution	Nonsynonymous coding	22%
MM04T	SLC26A7	solute carrier family 26, member 7	CCDS6255.1	chr8_92365219-92365219_G_A	437D>N	Substitution	Nonsynonymous coding	24%
MM04T	SLC26A8	solute carrier family 26, member 8	CCDS4813.1	chr6_35980127-35980127_G_A	71R>X	Substitution	Nonsense	45%
MM04T	SLC27A6	solute carrier family 27 (fatty acid transporter), member 6	CCDS4145.1	chr5_128301957-128301957_C_T	43R>W	Substitution	Nonsynonymous coding	26%
MM04T	SLC28A1	solute carrier family 28 (sodium-coupled nucleoside transporter), member 1	CCDS10334.1	chr15_85433676-85433676_G_T	63R>M	Substitution	Nonsynonymous coding	23%
MM04T	SLC28A2	solute carrier family 28 (sodium-coupled nucleoside transporter), member 2	CCDS10121.1	chr15_45559675-45559675_G_A	322R>H	Substitution	Nonsynonymous coding	34%
MM04T	SLC29A3	solute carrier family 29 (nucleoside transporters), member 3	CCDS7310.1	chr10_73121811-73121811_G_A	292D>N	Substitution	Nonsynonymous coding	20%
MM04T	SLC2A2	solute carrier family 2 (facilitated glucose transporter), member 2	CCDS3215.1	chr3_170715887-170715887_G_T	460F>L	Substitution	Nonsynonymous coding	30%
MM04T	SLC2A2	solute carrier family 2 (facilitated glucose transporter), member 2	CCDS3215.1	chr3_170723124-170723124_G_T	305L>I	Substitution	Nonsynonymous coding	29%
MM04T	SLC34A3	solute carrier family 34 (sodium phosphate), member 3	CCDS7038.1	chr9_140128130-140128130_A_C	268N>H	Substitution	Nonsynonymous coding	36%
MM04T	SLC35A2	solute carrier family 35 (UDP-galactose transporter), member A2	CCDS14311.1	chrX_48762108-48762108_C_T	360V>I	Substitution	Nonsynonymous coding	38%
MM04T	SLC35D3	solute carrier family 35, member D3	CCDS34544.1	chr6_137245335-137245335_C_T	251S>L	Substitution	Nonsynonymous coding	31%
MM04T	SLC35E4	solute carrier family 35, member E4	CCDS13882.1	chr22_31032486-31032486_G_A	17E>K	Substitution	Nonsynonymous coding	31%
MM04T	SLC35F1	solute carrier family 35, member F1	CCDS34524.1	chr6_118556782-118556782_A_G	154T>A	Substitution	Nonsynonymous coding	29%

MM04T	SLC35F3	solute carrier family 35, member F3	CCDS1600.1	chr1_234040861-234040861_G_A	13G>D	Substitution	Nonsynonymous coding	38%
MM04T	SLC35F3	solute carrier family 35, member F3	CCDS1600.1	chr1_234454551-234454551_T_G	337L>V	Substitution	Nonsynonymous coding	20%
MM04T	SLC36A2	solute carrier family 36 (proton/amino acid symporter), member 2	CCDS4315.1	chr5_150726859-150726859_T_C	55T>A	Substitution	Nonsynonymous coding	20%
MM04T	SLC36A4	solute carrier family 36 (proton/amino acid symporter), member 4	CCDS8291.1	chr11_92881996-92881996_G_T	408L>I	Substitution	Nonsynonymous coding	34%
MM04T	SLC37A2	solute carrier family 37 (glycerol-3-phosphate transporter), member 2	CCDS31714.1	chr11_124952154-124952154_C_A	300F>L	Substitution	Nonsynonymous coding	30%
MM04T	SLC38A1	solute carrier family 38, member 1	CCDS41774.1	chr12_46623362-46623362_C_A	61K>N	Substitution	Nonsynonymous coding	29%
MM04T	SLC38A1	solute carrier family 38, member 1	CCDS41774.1	chr12_46633559-46633559_C_T	9E>K	Substitution	Nonsynonymous coding	20%
MM04T	SLC38A11	solute carrier family 38, member 11	CCDS2224.1	chr2_165771647-165771647_G_T	197Y>X	Substitution	Nonsense	24%
MM04T	SLC38A6	solute carrier family 38, member 6	CCDS9751.1	chr14_61449304-61449304_G_T	62G>C	Substitution	Nonsynonymous coding	19%
MM04T	SLC39A10	solute carrier family 39 (zinc transporter), member 10	CCDS33353.1	chr2_196545017-196545017_A_C	84K>T	Substitution	Nonsynonymous coding	36%
MM04T	SLC39A12	solute carrier family 39 (zinc transporter), member 12	CCDS44362.1	chr10_18242415-18242415_G_T	70E>D	Substitution	Nonsynonymous coding	28%
MM04T	SLC39A5	solute carrier family 39 (metal ion transporter), member 5	CCDS8912.2	chr12_56630210-56630210_G_A	326E>K	Substitution	Nonsynonymous coding	26%
MM04T	SLC39A5	solute carrier family 39 (metal ion transporter), member 5	CCDS8912.2	chr12_56630789-56630789_G_A	428E>K	Substitution	Nonsynonymous coding	28%
MM04T	SLC39A9	solute carrier family 39 (zinc transporter), member 9	CCDS9795.1	chr14_69922500-69922500_C_T	204R>W	Substitution	Nonsynonymous coding	24%
MM04T	SLC39A9	solute carrier family 39 (zinc transporter), member 9	CCDS9795.1	chr14_69908806-69908806_G_T	76E>X	Substitution	Nonsense	40%
MM04T	SLC3A1	solute carrier family 3 (cystine, dibasic and neutral amino acid transporters, activator of	CCDS1819.1	chr2_44502902-44502902_C_A	76F>L	Substitution	Nonsynonymous coding	34%
MM04T	SLC44A1	solute carrier family 44, member 1	CCDS6763.1	chr9_108125227-108125227_C_A	342F>L	Substitution	Nonsynonymous coding	22%
MM04T	SLC44A5	solute carrier family 44, member 5	CCDS667.1	chr1_75681455-75681455_C_A	571R>I	Substitution	Nonsynonymous coding	28%
MM04T	SLC44A5	solute carrier family 44, member 5	CCDS667.1	chr1_75704306-75704306_A_G	183F>S	Substitution	Nonsynonymous coding	29%
MM04T	SLC44A5	solute carrier family 44, member 5	CCDS667.1	chr1_75683575-75683575_C_A	534E>X	Substitution	Nonsense	40%
MM04T	SLC45A3	solute carrier family 45, member 3	CCDS1458.1	chr1_205631156-205631156_C_T	353A>T	Substitution	Nonsynonymous coding	33%
MM04T	SLC45A4	solute carrier family 45, member 4	CCDS34948.1	chr8_142221718-142221718_C_A	740M>I	Substitution	Nonsynonymous coding	26%
MM04T	SLC46A3	solute carrier family 46, member 3	CCDS45021.1	chr13_29287127-29287127_C_A	250K>N	Substitution	Nonsynonymous coding	34%
MM04T	SLC46A3	solute carrier family 46, member 3	CCDS45021.1	chr13_29287217-29287217_A_C	220D>E	Substitution	Nonsynonymous coding	24%
MM04T	SLC48A1	solute carrier family 48 (heme transporter), member 1	CCDS8755.2	chr12_48172961-48172961_C_A	96A>D	Substitution	Nonsynonymous coding	26%
MM04T	SLC4A2	solute carrier family 4, anion exchanger, member 2 (erythrocyte membrane protein	CCDS5917.1	chr7_150765106-150765106_G_A	371R>H	Substitution	Nonsynonymous coding	41%
MM04T	SLC4A4	solute carrier family 4, sodium bicarbonate cotransporter, member 4	CCDS47071.1	chr4_72121049-72121049_G_T	62E>D	Substitution	Nonsynonymous coding	30%
MM04T	SLC4A4	solute carrier family 4, sodium bicarbonate cotransporter, member 4	CCDS47071.1	chr4_72316110-72316110_T_C	353F>S	Substitution	Nonsynonymous coding	41%
MM04T	SLC4A5	solute carrier family 4, sodium bicarbonate cotransporter, member 5	CCDS1936.1	chr2_74474397-74474397_T_C	609I>V	Substitution	Nonsynonymous coding	37%
MM04T	SLC4A5	solute carrier family 4, sodium bicarbonate cotransporter, member 5	CCDS1936.1	chr2_74477476-74477476_A_C	549N>K	Substitution	Nonsynonymous coding	40%
MM04T	SLC51B	solute carrier family 51, beta subunit	CCDS10199.1	chr15_65345313-65345313_G_T	66K>N	Substitution	Nonsynonymous coding	34%
MM04T	SLC5A1	solute carrier family 5 (sodium/glucose cotransporter), member 1	CCDS13902.1	chr22_32495249-32495249_G_A	454D>N	Substitution	Nonsynonymous coding	16%

MM04T	SLC5A10	solute carrier family 5 (sodium/glucose cotransporter), member 10	CCDS11201.2	chr17_18916717-18916717_G_A	342R>Q	Substitution	Nonsynonymous coding	19%
MM04T	SLC5A8	solute carrier family 5 (iodide transporter), member 8	CCDS9080.1	chr12_101552060-101552060_C_A	559E>D	Substitution	Nonsynonymous coding	25%
MM04T	SLC6A12	solute carrier family 6 (neurotransmitter transporter, betaine/GABA), member 12	CCDS8501.1	chr12_319093-319093_C_A	20E>D	Substitution	Nonsynonymous coding	18%
MM04T	SLC6A18	solute carrier family 6, member 18	CCDS3860.1	chr5_1244860-1244860_A_G	545Y>C	Substitution	Nonsynonymous coding	21%
MM04T	SLC7A6OS	solute carrier family 7, member 6 opposite strand	CCDS10865.1	chr16_68335245-68335245_C_A	288S>I	Substitution	Nonsynonymous coding	29%
MM04T	SLC7A7	solute carrier family 7 (amino acid transporter light chain, y+L system), member 7	CCDS9574.1	chr14_23282105-23282105_T_G	NA	Substitution	Splice site donor	25%
MM04T	SLC7A8	solute carrier family 7 (amino acid transporter light chain, L system), member 8	CCDS9590.1	chr14_23652012-23652012_T_C	38K>E	Substitution	Nonsynonymous coding	33%
MM04T	SLC8A1	solute carrier family 8 (sodium/calcium exchanger), member 1	CCDS1806.1	chr2_40656657-40656657_C_A	255R>I	Substitution	Nonsynonymous coding	25%
MM04T	SLC8A3	solute carrier family 8 (sodium/calcium exchanger), member 3	CCDS35498.1	chr14_70512693-70512693_C_A	919E>X	Substitution	Nonsense	16%
MM04T	SLC9A2	solute carrier family 9, subfamily A (NHE2, cation proton antiporter 2), member 2	CCDS2062.1	chr2_103324731-103324731_G_A	741R>Q	Substitution	Nonsynonymous coding	33%
MM04T	SLC9A4	solute carrier family 9, subfamily A (NHE4, cation proton antiporter 4), member 4	CCDS33264.1	chr2_103125355-103125355_A_C	484N>T	Substitution	Nonsynonymous coding	32%
MM04T	SLC9A6	solute carrier family 9, subfamily A (NHE6, cation proton antiporter 6), member 6	CCDS44003.1	chrX_135084290-135084290_G_A	273E>K	Substitution	Nonsynonymous coding	20%
MM04T	SLC9A6	solute carrier family 9, subfamily A (NHE6, cation proton antiporter 6), member 6	CCDS44003.1	chrX_135092669-135092669_C_A	323S>Y	Substitution	Nonsynonymous coding	18%
MM04T	SLC9A7	solute carrier family 9, subfamily A (NHE7, cation proton antiporter 7), member 7	CCDS14269.1	chrX_46466477-46466477_C_A	696E>D	Substitution	Nonsynonymous coding	32%
MM04T	SLC9A7	solute carrier family 9, subfamily A (NHE7, cation proton antiporter 7), member 7	CCDS14269.1	chrX_46495061-46495061_C_T	546E>K	Substitution	Nonsynonymous coding	38%
MM04T	SLC9B2	solute carrier family 9, subfamily B (NHA2, cation proton antiporter 2), member 2	CCDS3662.1	chr4_103968330-103968330__A	NA	Insertion	Frameshift	31%
MM04T	SLC9B2	solute carrier family 9, subfamily B (NHA2, cation proton antiporter 2), member 2	CCDS3662.1	chr4_103968343-103968343_A_G	252S>P	Substitution	Nonsynonymous coding	28%
MM04T	SLC9C1	solute carrier family 9, subfamily C (Na+-transporting carboxylic acid decarboxylase),	CCDS33817.1	chr3_111958848-111958848_G_A	429R>C	Substitution	Nonsynonymous coding	33%
MM04T	SLC9C2	solute carrier family 9, member C2 (putative)	CCDS1308.1	chr1_173472431-173472431_C_A	1115K>N	Substitution	Nonsynonymous coding	36%
MM04T	SLC9C2	solute carrier family 9, member C2 (putative)	CCDS1308.1	chr1_173569302-173569302_G_A	61S>F	Substitution	Nonsynonymous coding	28%
MM04T	SLCO1A2	solute carrier organic anion transporter family, member 1A2	CCDS8686.1	chr12_21467610-21467610_G_T	70L>I	Substitution	Nonsynonymous coding	40%
MM04T	SLCO1A2	solute carrier organic anion transporter family, member 1A2	CCDS8686.1	chr12_21487575-21487575_C_A	3E>X	Substitution	Nonsense	19%
MM04T	SLCO1B1	solute carrier organic anion transporter family, member 1B1	CCDS8685.1	chr12_21370153-21370153_A_C	533K>T	Substitution	Nonsynonymous coding	43%
MM04T	SLCO1B3	solute carrier organic anion transporter family, member 1B3	CCDS8684.1	chr12_21033796-21033796_T_G	447S>A	Substitution	Nonsynonymous coding	40%
MM04T	SLCO2A1	solute carrier organic anion transporter family, member 2A1	CCDS3084.1	chr3_133673955-133673955_C_A	160Q>H	Substitution	Nonsynonymous coding	23%
MM04T	SLFN5	schlafen family member 5	CCDS32619.1	chr17_33592836-33592836_G_C	869A>P	Substitution	Nonsynonymous coding	30%
MM04T	SLIT2	slit homolog 2 (Drosophila)	CCDS3426.1	chr4_20620402-20620402_C_T	1454R>X	Substitution	Nonsense	32%
MM04T	SLITRK1	SLIT and NTRK-like family, member 1	CCDS9464.1	chr13_84453961-84453961_G_A	561P>L	Substitution	Nonsynonymous coding	28%
MM04T	SLITRK1	SLIT and NTRK-like family, member 1	CCDS9464.1	chr13_84454735-84454735_G_T	303S>Y	Substitution	Nonsynonymous coding	34%
MM04T	SLITRK1	SLIT and NTRK-like family, member 1	CCDS9464.1	chr13_84455278-84455278_C_T	122R>Q	Substitution	Nonsynonymous coding	40%
MM04T	SLITRK2	SLIT and NTRK-like family, member 2	CCDS14680.1	chrX_144904986-144904986_C_T	348S>F	Substitution	Nonsynonymous coding	19%
MM04T	SLITRK3	SLIT and NTRK-like family, member 3	CCDS3197.1	chr3_164907463-164907463_G_T	386L>I	Substitution	Nonsynonymous coding	32%

MM04T	SLITRK4	SLIT and NTRK-like family, member 4	CCDS14679.1	chrX_142717102-142717102_C_T	608R>Q	Substitution	Nonsynonymous coding	31%
MM04T	SLITRK5	SLIT and NTRK-like family, member 5	CCDS9465.1	chr13_88328511-88328511_T_C	290S>P	Substitution	Nonsynonymous coding	40%
MM04T	SLITRK5	SLIT and NTRK-like family, member 5	CCDS9465.1	chr13_88329213-88329213_G_A	524V>I	Substitution	Nonsynonymous coding	28%
MM04T	SLITRK6	SLIT and NTRK-like family, member 6	CCDS41903.1	chr13_86368645-86368645_G_T	667H>N	Substitution	Nonsynonymous coding	36%
MM04T	SLK	STE20-like kinase	CCDS7553.1	chr10_105762811-105762811_G_T	625E>D	Substitution	Nonsynonymous coding	41%
MM04T	SLK	STE20-like kinase	CCDS7553.1	chr10_105768032-105768032_G_A	901R>Q	Substitution	Nonsynonymous coding	28%
MM04T	SLMAP	sarcolemma associated protein	CCDS33774.1	chr3_57846448-57846448_G_A	237R>Q	Substitution	Nonsynonymous coding	72%
MM04T	SLTM	SAFB-like, transcription modulator	CCDS10168.2	chr15_59185133-59185133_G_A	621R>C	Substitution	Nonsynonymous coding	21%
MM04T	SMAP2	small ArfGAP2	CCDS451.1	chr1_40872452-40872452_C_T	50R>X	Substitution	Nonsense	33%
MM04T	SMARCA1	SWI/SNF related, matrix associated, actin dependent regulator of chromatin, subfamily	CCDS14612.1	chrX_128626006-128626006_G_A	576A>V	Substitution	Nonsynonymous coding	21%
MM04T	SMARCA2	SWI/SNF related, matrix associated, actin dependent regulator of chromatin, subfamily	CCDS34977.1	chr9_2029101-2029101_C_A	27L>I	Substitution	Nonsynonymous coding	33%
MM04T	SMARCA5	SWI/SNF related, matrix associated, actin dependent regulator of chromatin, subfamily	CCDS3761.1	chr4_144442700-144442700_G_A	124R>H	Substitution	Nonsynonymous coding	22%
MM04T	SMARCA5	SWI/SNF related, matrix associated, actin dependent regulator of chromatin, subfamily	CCDS3761.1	chr4_144466678-144466678_G_A	780R>H	Substitution	Nonsynonymous coding	23%
MM04T	SMARCB1	SWI/SNF related, matrix associated, actin dependent regulator of chromatin, subfamily	CCDS13817.1	chr22_24129381-24129381_A_G	9T>A	Substitution	Nonsynonymous coding	31%
MM04T	SMARCC2	SWI/SNF related, matrix associated, actin dependent regulator of chromatin, subfamily c,	CCDS8907.1	chr12_56574823-56574823_G_C	340T>R	Substitution	Nonsynonymous coding	23%
MM04T	SMARCD1	SWI/SNF related, matrix associated, actin dependent regulator of chromatin, subfamily	CCDS8797.2	chr12_50482348-50482348_C_A	233F>L	Substitution	Nonsynonymous coding	22%
MM04T	SMC1A	structural maintenance of chromosomes 1A	CCDS14352.1	chrX_53409534-53409534_C_T	1060E>K	Substitution	Nonsynonymous coding	34%
MM04T	SMC1A	structural maintenance of chromosomes 1A	CCDS14352.1	chrX_53436051-53436051_C_T	496R>H	Substitution	Nonsynonymous coding	29%
MM04T	SMC1B	structural maintenance of chromosomes 1B	CCDS43027.1	chr22_45758768-45758768_C_A	853K>N	Substitution	Nonsynonymous coding	31%
MM04T	SMC1B	structural maintenance of chromosomes 1B	CCDS43027.1	chr22_45758812-45758812_T_G	839K>Q	Substitution	Nonsynonymous coding	45%
MM04T	SMC3	structural maintenance of chromosomes 3	CCDS31285.1	chr10_112349729-112349729_C_A	497L>I	Substitution	Nonsynonymous coding	21%
MM04T	SMC5	structural maintenance of chromosomes 5	CCDS6632.1	chr9_72892229-72892229_C_A	128F>L	Substitution	Nonsynonymous coding	28%
MM04T	SMC6	structural maintenance of chromosomes 6	CCDS1690.1	chr2_17888487-17888487_T_G	669S>R	Substitution	Nonsynonymous coding	35%
MM04T	SMC6	structural maintenance of chromosomes 6	CCDS1690.1	chr2_17876489-17876489_C_A	870E>X	Substitution	Nonsense	21%
MM04T	SMCHD1	structural maintenance of chromosomes flexible hinge domain containing 1	CCDS45822.1	chr18_2763774-2763774_C_T	1569S>L	Substitution	Nonsynonymous coding	31%
MM04T	SMCHD1	structural maintenance of chromosomes flexible hinge domain containing 1	CCDS45822.1	chr18_2784503-2784503_G_A	1868R>Q	Substitution	Nonsynonymous coding	29%
MM04T	SMCR7	Smith-Magenis syndrome chromosome region, candidate 7	CCDS45624.1	chr17_18168025-18168025_G_A	449D>N	Substitution	Nonsynonymous coding	28%
MM04T	SMG1	smg-1 homolog, phosphatidylinositol 3-kinase-related kinase (C. elegans)	CCDS45430.1	chr16_18849370-18849370_C_T	2460R>H	Substitution	Nonsynonymous coding	24%
MM04T	SMG1	smg-1 homolog, phosphatidylinositol 3-kinase-related kinase (C. elegans)	CCDS45430.1	chr16_18849969-18849969_T_C	2330I>V	Substitution	Nonsynonymous coding	24%
MM04T	SMG8	smg-8 homolog, nonsense mediated mRNA decay factor (C. elegans)	CCDS11615.1	chr17_57288085-57288085_C_T	225R>X	Substitution	Nonsense	39%
MM04T	SMIM10	small integral membrane protein 10	NM_001163438	chrX_134125354-134125354_G_A	77V>I	Substitution	Nonsynonymous coding	33%
MM04T	SMO	smoothened, frizzled family receptor	CCDS5811.1	chr7_128850335-128850335_G_A	533S>N	Substitution	Nonsynonymous coding	36%

MM04T	SMPX	small muscle protein, X-linked	CCDS14200.1	chrX_21772372-21772372_C_T	13A>T	Substitution	Nonsynonymous coding	31%
MM04T	SNAP91	synaptosomal-associated protein, 91kDa	CCDS47455.1	chr6_84417533-84417533_C_A	38K>N	Substitution	Nonsynonymous coding	19%
MM04T	SNAPC4	small nuclear RNA activating complex, polypeptide 4, 190kDa	CCDS6998.1	chr9_139275241-139275241_G_A	817P>L	Substitution	Nonsynonymous coding	25%
MM04T	SNCA	synuclein, alpha (non A4 component of amyloid precursor)	CCDS3634.1	chr4_90743425-90743425_C_T	93G>D	Substitution	Nonsynonymous coding	24%
MM04T	SNCAIP	synuclein, alpha interacting protein	CCDS4131.1	chr5_121786396-121786396_A_C	618K>N	Substitution	Nonsynonymous coding	33%
MM04T	SND1	staphylococcal nuclease and tudor domain containing 1	CCDS34747.1	chr7_127729577-127729577_C_T	819R>W	Substitution	Nonsynonymous coding	27%
MM04T	SNRK	SNF related kinase	CCDS43075.1	chr3_43345245-43345245_G_T	184E>X	Substitution	Nonsense	33%
MM04T	SNRNP200	small nuclear ribonucleoprotein 200kDa (U5)	CCDS2020.1	chr2_96944592-96944592_G_T	1760L>M	Substitution	Nonsynonymous coding	22%
MM04T	SNRNP40	small nuclear ribonucleoprotein 40kDa (U5)	CCDS340.1	chr1_31754229-31754229_C_A	216D>Y	Substitution	Nonsynonymous coding	31%
MM04T	SNRPB	small nuclear ribonucleoprotein polypeptides B and B1	CCDS13026.1	chr20_2444500-2444500_C_G	105G>R	Substitution	Nonsynonymous coding	25%
MM04T	SNRPC	small nuclear ribonucleoprotein polypeptide C	CCDS34436.1	chr6_34730416-34730416_G_T	32E>D	Substitution	Nonsynonymous coding	30%
MM04T	SNRPN	small nuclear ribonucleoprotein polypeptide N	CCDS10017.1	chr15_25221458-25221458_G_T	54K>N	Substitution	Nonsynonymous coding	18%
MM04T	SNTG1	syntrophin, gamma 1	CCDS6147.1	chr8_51705372-51705372_G_T	513A>S	Substitution	Nonsynonymous coding	20%
MM04T	SNX11	sorting nexin 11	CCDS11526.1	chr17_46196467-46196467_G_A	153R>Q	Substitution	Nonsynonymous coding	19%
MM04T	SNX19	sorting nexin 19	CCDS31721.1	chr11_130784870-130784870_G_T	322S>Y	Substitution	Nonsynonymous coding	17%
MM04T	SNX20	sorting nexin 20	CCDS10745.1	chr16_50707913-50707913_C_T	119D>N	Substitution	Nonsynonymous coding	19%
MM04T	SNX25	sorting nexin 25	CCDS34116.1	chr4_186244723-186244723_C_A	342F>L	Substitution	Nonsynonymous coding	50%
MM04T	SNX7	sorting nexin 7	CCDS755.2	chr1_99203886-99203886_A_C	407N>H	Substitution	Nonsynonymous coding	14%
MM04T	SOAT2	sterol O-acyltransferase 2	CCDS8847.1	chr12_53517635-53517635_G_A	499R>Q	Substitution	Nonsynonymous coding	40%
MM04T	SOGA2	SOGA family member 2	CCDS11841.1	chr18_8720483-8720483_A_G	116R>G	Substitution	Nonsynonymous coding	22%
MM04T	SOGA3	SOGA family member 3	CCDS43505.1	chr6_127794511-127794511_A_C	920I>S	Substitution	Nonsynonymous coding	27%
MM04T	SOGA3	SOGA family member 3	CCDS43505.1	chr6_127797352-127797352_C_T	607E>K	Substitution	Nonsynonymous coding	23%
MM04T	SON	SON DNA binding protein	CCDS13629.1	chr21_34924937-34924937_G_A	1134D>N	Substitution	Nonsynonymous coding	33%
MM04T	SORCS1	sortilin-related VPS10 domain containing receptor 1	CCDS31283.1	chr10_108371764-108371764_G_A	980R>C	Substitution	Nonsynonymous coding	32%
MM04T	SORCS1	sortilin-related VPS10 domain containing receptor 1	CCDS31283.1	chr10_108521351-108521351_G_T	318F>L	Substitution	Nonsynonymous coding	33%
MM04T	SORCS2	sortilin-related VPS10 domain containing receptor 2	CCDS47008.1	chr4_7668894-7668894_G_A	372R>Q	Substitution	Nonsynonymous coding	20%
MM04T	SORCS3	sortilin-related VPS10 domain containing receptor 3	CCDS7558.1	chr10_106937871-106937871_C_T	650S>L	Substitution	Nonsynonymous coding	25%
MM04T	SORCS3	sortilin-related VPS10 domain containing receptor 3	CCDS7558.1	chr10_107005354-107005354_G_A	975D>N	Substitution	Nonsynonymous coding	22%
MM04T	SORCS3	sortilin-related VPS10 domain containing receptor 3	CCDS7558.1	chr10_107012619-107012619_G_T	1064K>N	Substitution	Nonsynonymous coding	26%
MM04T	SORL1	sortilin-related receptor, L(DLR class) A repeats containing	CCDS8436.1	chr11_121495874-121495874_T_G	2084I>M	Substitution	Nonsynonymous coding	20%
MM04T	SOS2	son of sevenless homolog 2 (Drosophila)	CCDS9697.1	chr14_50611968-50611968_A_C	863L>V	Substitution	Nonsynonymous coding	25%
MM04T	SOS2	son of sevenless homolog 2 (Drosophila)	CCDS9697.1	chr14_50612269-50612269_T_G	810E>D	Substitution	Nonsynonymous coding	28%

MM04T	SOWAHA	sosondowah ankyrin repeat domain family member A	CCDS43361.1	chr5_132150335-132150335_G_A	341R>H	Substitution	Nonsynonymous coding	25%
MM04T	SOWAHB	sosondowah ankyrin repeat domain family member B	CCDS34017.1	chr4_77816759-77816759_C_A	748K>N	Substitution	Nonsynonymous coding	23%
MM04T	SOX6	SRY (sex determining region Y)-box 6	CCDS7821.1	chr11_16340191-16340191_C_A	82E>D	Substitution	Nonsynonymous coding	30%
MM04T	SP1	Sp1 transcription factor	CCDS8857.1	chr12_53804718-53804718_G_T	684K>N	Substitution	Nonsynonymous coding	24%
MM04T	SPAG17	sperm associated antigen 17	CCDS899.1	chr1_118693253-118693253_C_A	NA	Substitution	Splice site acceptor	60%
MM04T	SPAG9	sperm associated antigen 9	CCDS45740.1	chr17_49057274-49057274_G_T	1081S>Y	Substitution	Nonsynonymous coding	31%
MM04T	SPAG9	sperm associated antigen 9	CCDS45740.1	chr17_49197752-49197752_T_C	89Y>C	Substitution	Nonsynonymous coding	31%
MM04T	SPAG9	sperm associated antigen 9	CCDS45740.1	chr17_49119008-49119008_C_A	199E>X	Substitution	Nonsense	38%
MM04T	SPAST	spastin	CCDS1778.1	chr2_32379535-32379535_G_A	607W>X	Substitution	Nonsense	21%
MM04T	SPATA16	spermatogenesis associated 16	CCDS3221.1	chr3_172766761-172766761_G_T	246L>I	Substitution	Nonsynonymous coding	40%
MM04T	SPATA17	spermatogenesis associated 17	CCDS1519.1	chr1_217915399-217915399_T_G	160Y>D	Substitution	Nonsynonymous coding	32%
MM04T	SPATA18	spermatogenesis associated 18	CCDS3489.1	chr4_52917949-52917949_G_T	27E>X	Substitution	Nonsense	22%
MM04T	SPATA20	spermatogenesis associated 20	CCDS11571.1	chr17_48625670-48625670_G_A	51R>Q	Substitution	Nonsynonymous coding	33%
MM04T	SPATA21	spermatogenesis associated 21	CCDS172.1	chr1_16730506-16730506_G_T	280F>L	Substitution	Nonsynonymous coding	24%
MM04T	SPATA24	spermatogenesis associated 24	ENST00000302091	chr5_138732481-138732481_C_T	218A>T	Substitution	Nonsynonymous coding	15%
MM04T	SPATA32	spermatogenesis associated 32	CCDS32669.1	chr17_43332623-43332623_G_A	309S>F	Substitution	Nonsynonymous coding	20%
MM04T	SPATA4	spermatogenesis associated 4	CCDS3826.1	chr4_177113804-177113804_C_A	221R>I	Substitution	Nonsynonymous coding	30%
MM04T	SPATA5L1	spermatogenesis associated 5-like 1	CCDS10123.1	chr15_45695046-45695046_G_T	140R>I	Substitution	Nonsynonymous coding	22%
MM04T	SPATA9	spermatogenesis associated 9	CCDS4076.1	chr5_95011330-95011330_G_A	55A>V	Substitution	Nonsynonymous coding	33%
MM04T	SPDYA	speedy homolog A (Xenopus laevis)	CCDS1767.2	chr2_29038938-29038938_T_C	20S>P	Substitution	Nonsynonymous coding	23%
MM04T	SPDYC	speedy homolog C (Xenopus laevis)	CCDS31606.1	chr11_64939990-64939990_C_T	144R>X	Substitution	Nonsense	32%
MM04T	SPECC1	sperm antigen with calponin homology and coiled-coil domains 1	CCDS32590.1	chr17_20108118-20108118_G_T	252E>D	Substitution	Nonsynonymous coding	31%
MM04T	SPECC1	sperm antigen with calponin homology and coiled-coil domains 1	CCDS32590.1	chr17_20108619-20108619_G_T	419K>N	Substitution	Nonsynonymous coding	21%
MM04T	SPECC1	sperm antigen with calponin homology and coiled-coil domains 1	CCDS32590.1	chr17_20108551-20108551_G_T	397E>X	Substitution	Nonsense	31%
MM04T	SPEF2	sperm flagellar 2	CCDS43309.1	chr5_35700675-35700675_C_T	740A>V	Substitution	Nonsynonymous coding	28%
MM04T	SPEF2	sperm flagellar 2	CCDS43309.1	chr5_35753775-35753775_C_T	1127A>V	Substitution	Nonsynonymous coding	25%
MM04T	SPEG	SPEG complex locus	ENST00000452101	chr2_220308758-220308758_G_A	30A>T	Substitution	Nonsynonymous coding	32%
MM04T	SPEG	SPEG complex locus	CCDS42824.1	chr2_220344574-220344574_G_A	1719D>N	Substitution	Nonsynonymous coding	31%
MM04T	SPERT	spermatid associated	CCDS9399.1	chr13_46287329-46287329_G_A	57E>K	Substitution	Nonsynonymous coding	35%
MM04T	SPG11	spastic paraplegia 11 (autosomal recessive)	CCDS10112.1	chr15_44856800-44856800_C_T	2366E>K	Substitution	Nonsynonymous coding	40%
MM04T	SPG11	spastic paraplegia 11 (autosomal recessive)	CCDS10112.1	chr15_44914034-44914034_C_A	848R>I	Substitution	Nonsynonymous coding	39%
MM04T	SPG11	spastic paraplegia 11 (autosomal recessive)	CCDS10112.1	chr15_44925802-44925802_T_G	546N>H	Substitution	Nonsynonymous coding	27%

MM04T	SPG11	spastic paraplegia 11 (autosomal recessive)	CCDS10112.1	chr15_44890524-44890524_C_A	1314E>X	Substitution	Nonsense	28%
MM04T	SPG20	spastic paraplegia 20 (Troyer syndrome)	CCDS9356.1	chr13_36888421-36888421_C_T	476G>R	Substitution	Nonsynonymous coding	21%
MM04T	SPINK5	serine peptidase inhibitor, Kazal type 5	CCDS47300.1	chr5_147481366-147481366_A_C	442K>T	Substitution	Nonsynonymous coding	19%
MM04T	SPINT1	serine peptidase inhibitor, Kunitz type 1	CCDS10067.1	chr15_41145694-41145694_A_C	204D>A	Substitution	Nonsynonymous coding	19%
MM04T	SPO11	SPO11 meiotic protein covalently bound to DSB homolog (S. cerevisiae)	CCDS13456.1	chr20_55909114-55909114_C_A	158S>Y	Substitution	Nonsynonymous coding	38%
MM04T	SPO11	SPO11 meiotic protein covalently bound to DSB homolog (S. cerevisiae)	CCDS13456.1	chr20_55915179-55915179_C_A	302H>N	Substitution	Nonsynonymous coding	31%
MM04T	SPOCD1	SPOC domain containing 1	CCDS347.1	chr1_32258314-32258314_C_T	914D>N	Substitution	Nonsynonymous coding	11%
MM04T	SPOCK3	sparc/osteonectin, cwcv and kazal-like domains proteoglycan (testican) 3	CCDS34095.1	chr4_167675776-167675776_T_C	272S>G	Substitution	Nonsynonymous coding	32%
MM04T	SPON1	spondin 1, extracellular matrix protein	ENST00000310358	chr11_14281225-14281225_G_T	602E>D	Substitution	Nonsynonymous coding	15%
MM04T	SPON1	spondin 1, extracellular matrix protein	ENST00000310358	chr11_14284396-14284396_G_A	711R>Q	Substitution	Nonsynonymous coding	19%
MM04T	SPON1	spondin 1, extracellular matrix protein	ENST00000310358	chr11_14157106-14157106_C_T	272R>X	Substitution	Nonsense	33%
MM04T	SPON1	spondin 1, extracellular matrix protein	ENST00000310358	chr11_14284425-14284425_C_T	721R>X	Substitution	Nonsense	22%
MM04T	SPOPL	speckle-type POZ protein-like	CCDS33298.1	chr2_139310141-139310141_C_T	124R>X	Substitution	Nonsense	30%
MM04T	SPPL2A	signal peptide peptidase like 2A	CCDS10138.1	chr15_51000038-51000038_C_A	508E>X	Substitution	Nonsense	21%
MM04T	SPR	sepiapterin reductase (7,8-dihydrobiopterin:NADP+ oxidoreductase)	CCDS1920.1	chr2_73118536-73118536_G_A	219R>Q	Substitution	Nonsynonymous coding	21%
MM04T	SPTA1	spectrin, alpha, erythrocytic 1 (elliptocytosis 2)	CCDS41423.1	chr1_158617358-158617358_C_A	1289Q>H	Substitution	Nonsynonymous coding	15%
MM04T	SPTA1	spectrin, alpha, erythrocytic 1 (elliptocytosis 2)	CCDS41423.1	chr1_158622400-158622400_G_T	1078L>I	Substitution	Nonsynonymous coding	18%
MM04T	SPTA1	spectrin, alpha, erythrocytic 1 (elliptocytosis 2)	CCDS41423.1	chr1_158647588-158647588_C_A	283K>N	Substitution	Nonsynonymous coding	25%
MM04T	SPTBN2	spectrin, beta, non-erythrocytic 2	CCDS8150.1	chr11_66453351-66453351_C_A	2388K>N	Substitution	Nonsynonymous coding	32%
MM04T	SPTBN2	spectrin, beta, non-erythrocytic 2	CCDS8150.1	chr11_66472475-66472475_C_T	758D>N	Substitution	Nonsynonymous coding	31%
MM04T	SPTBN5	spectrin, beta, non-erythrocytic 5	NM_016642	chr15_42167712-42167712_G_A	1376R>C	Substitution	Nonsynonymous coding	19%
MM04T	SPTBN5	spectrin, beta, non-erythrocytic 5	NM_016642	chr15_42170740-42170740_T_G	1055Q>H	Substitution	Nonsynonymous coding	32%
MM04T	SPZ1	spermatogenic leucine zipper 1	CCDS43336.1	chr5_79616598-79616598_G_T	188K>N	Substitution	Nonsynonymous coding	18%
MM04T	SQLE	squalene epoxidase	CCDS47918.1	chr8_126015427-126015427_G_T	101G>X	Substitution	Nonsense	23%
MM04T	SREK1	splicing regulatory glutamine/lysine-rich protein 1	CCDS43323.1	chr5_65458364-65458364_T_G	233N>K	Substitution	Nonsynonymous coding	33%
MM04T	SRFBP1	serum response factor binding protein 1	CCDS43354.1	chr5_121309905-121309905_A_C	17R>S	Substitution	Nonsynonymous coding	31%
MM04T	SRGAP1	SLIT-ROBO Rho GTPase activating protein 1	CCDS8967.1	chr12_64536208-64536208_C_T	1005S>F	Substitution	Nonsynonymous coding	33%
MM04T	SRGAP3	SLIT-ROBO Rho GTPase activating protein 3	CCDS2572.1	chr3_9166406-9166406_T_C	NA	Substitution	Splice site donor	24%
MM04T	SRL	sarcalumenin	CCDS42113.1	chr16_4242450-4242450_C_T	376D>N	Substitution	Nonsynonymous coding	33%
MM04T	SRPK1	SRSF protein kinase 1	CCDS47415.1	chr6_35806186-35806186_C_A	571E>X	Substitution	Nonsense	34%
MM04T	SRPK3	SRSF protein kinase 3	CCDS35441.1	chrX_153050847-153050847_G_A	526E>K	Substitution	Nonsynonymous coding	25%
MM04T	SRPR	signal recognition particle receptor (docking protein)	CCDS31717.1	chr11_126134389-126134389_C_T	524R>H	Substitution	Nonsynonymous coding	28%

MM04T	SRRM2	serine/arginine repetitive matrix 2	ENST00000382300	chr16_2818898-2818898_C_A	139F>L	Substitution	Nonsynonymous coding	22%
MM04T	SRRM3	serine/arginine repetitive matrix 3	ENST00000388802	chr7_75914933-75914933_C_T	579R>C	Substitution	Nonsynonymous coding	24%
MM04T	SRRT	serrate RNA effector molecule homolog (Arabidopsis)	CCDS34709.1	chr7_100482545-100482545_G_T	348S>I	Substitution	Nonsynonymous coding	34%
MM04T	SSPO	SCO-spondin homolog (Bos taurus)	ENST00000378016	chr7_149482693-149482693_G_A	1037E>K	Substitution	Nonsynonymous coding	33%
MM04T	SSX2IP	synovial sarcoma, X breakpoint 2 interacting protein	CCDS699.1	chr1_85128033-85128033_G_T	259L>I	Substitution	Nonsynonymous coding	34%
MM04T	ST14	suppression of tumorigenicity 14 (colon carcinoma)	CCDS8487.1	chr11_130029916-130029916_G_T	14K>N	Substitution	Nonsynonymous coding	20%
MM04T	ST18	suppression of tumorigenicity 18 (breast carcinoma) (zinc finger protein)	CCDS6149.1	chr8_53045680-53045680_C_T	794R>H	Substitution	Nonsynonymous coding	23%
MM04T	ST3GAL4	ST3 beta-galactoside alpha-2,3-sialyltransferase 4	ENST00000239597	chr11_126310163-126310163_G_A	234R>K	Substitution	Nonsynonymous coding	30%
MM04T	ST3GAL4	ST3 beta-galactoside alpha-2,3-sialyltransferase 4	ENST00000239597	chr11_126310230-126310230_G_T	256Q>H	Substitution	Nonsynonymous coding	20%
MM04T	ST3GAL6	ST3 beta-galactoside alpha-2,3-sialyltransferase 6	CCDS2933.1	chr3_98491728-98491728_A_C	80K>T	Substitution	Nonsynonymous coding	27%
MM04T	ST6GAL2	ST6 beta-galactosamide alpha-2,6-sialyltranferase 2	CCDS2073.1	chr2_107423304-107423304_C_T	474D>N	Substitution	Nonsynonymous coding	17%
MM04T	ST6GALNAC2	ST6 (alpha-N-acetyl-neuraminyl-2,3-beta-galactosyl-1,3)-N-acetylgalactosaminide alpha-	CCDS11747.1	chr17_74562190-74562190_C_T	374R>H	Substitution	Nonsynonymous coding	29%
MM04T	ST6GALNAC6	ST6 (alpha-N-acetyl-neuraminyl-2,3-beta-galactosyl-1,3)-N-acetylgalactosaminide alpha-	CCDS6882.1	chr9_130656897-130656897_G_A	64A>V	Substitution	Nonsynonymous coding	20%
MM04T	ST8SIA3	ST8 alpha-N-acetyl-neuraminide alpha-2,8-sialyltransferase 3	CCDS32834.1	chr18_55027275-55027275_C_A	304L>I	Substitution	Nonsynonymous coding	30%
MM04T	STAB1	stabilin 1	CCDS33768.1	chr3_52558227-52558227_G_T	2552D>Y	Substitution	Nonsynonymous coding	28%
MM04T	STAB2	stabilin 2	CCDS31888.1	chr12_104048394-104048394_A_G	490D>G	Substitution	Nonsynonymous coding	34%
MM04T	STAG1	stromal antigen 1	CCDS3090.1	chr3_136323304-136323304_C_A	48K>N	Substitution	Nonsynonymous coding	32%
MM04T	STAG2	stromal antigen 2	CCDS43990.1	chrX_123179106-123179106_A_T	185Q>H	Substitution	Nonsynonymous coding	14%
MM04T	STAG2	stromal antigen 2	CCDS43990.1	chrX_123185201-123185201_G_T	385D>Y	Substitution	Nonsynonymous coding	63%
MM04T	STAG2	stromal antigen 2	CCDS43990.1	chrX_123190015-123190015_A_C	412N>H	Substitution	Nonsynonymous coding	20%
MM04T	STAM2	signal transducing adaptor molecule (SH3 domain and ITAM motif) 2	CCDS2196.1	chr2_152982850-152982850_C_T	357A>T	Substitution	Nonsynonymous coding	48%
MM04T	STAM2	signal transducing adaptor molecule (SH3 domain and ITAM motif) 2	CCDS2196.1	chr2_153004633-153004633_G_T	69L>I	Substitution	Nonsynonymous coding	31%
MM04T	STAMBPL1	STAM binding protein-like 1	CCDS7391.1	chr10_90674348-90674348_T_A	279F>Y	Substitution	Nonsynonymous coding	19%
MM04T	STAR	steroidogenic acute regulatory protein	CCDS6102.1	chr8_38003614-38003614_C_T	173E>K	Substitution	Nonsynonymous coding	19%
MM04T	STARD4	StAR-related lipid transfer (START) domain containing 4	CCDS4104.1	chr5_110835805-110835805_C_A	NA	Substitution	Splice site acceptor	27%
MM04T	STARD8	StAR-related lipid transfer (START) domain containing 8	CCDS48134.1	chrX_67943870-67943870_G_A	1034G>D	Substitution	Nonsynonymous coding	18%
MM04T	STARD9	StAR-related lipid transfer (START) domain containing 9	NM_020759	chr15_42977311-42977311_A_C	1179T>P	Substitution	Nonsynonymous coding	33%
MM04T	STARD9	StAR-related lipid transfer (START) domain containing 9	NM_020759	chr15_42978575-42978575_C_A	1600S>Y	Substitution	Nonsynonymous coding	39%
MM04T	STAU2	staufen, RNA binding protein, homolog 2 (Drosophila)	NM_001164380	chr8_74334858-74334858_G_T	537S>Y	Substitution	Nonsynonymous coding	27%
MM04T	STIL	SCL/TAL1 interrupting locus	CCDS41329.1	chr1_47765576-47765576_C_T	NA	Substitution	Splice site donor	41%
MM04T	STIM1	stromal interaction molecule 1	CCDS7749.1	chr11_4112889-4112889_G_A	640R>Q	Substitution	Nonsynonymous coding	19%
MM04T	STK17A	serine/threonine kinase 17a	CCDS5470.1	chr7_43664298-43664298_G_A	368E>K	Substitution	Nonsynonymous coding	31%

MM04T	STK17B	serine/threonine kinase 17b	CCDS2315.1	chr2_197002227-197002227_G_A	355R>C	Substitution	Nonsynonymous coding	22%
MM04T	STK31	serine/threonine kinase 31	CCDS5386.1	chr7_23826152-23826152_A_G	767E>G	Substitution	Nonsynonymous coding	22%
MM04T	STON1-GTF2A1L	STON1-GTF2A1L readthrough	CCDS1840.1	chr2_48809217-48809217_T_C	482I>T	Substitution	Nonsynonymous coding	20%
MM04T	STPG1	sperm-tail PG-rich repeat containing 1	CCDS253.1	chr1_24696175-24696175_C_A	195K>N	Substitution	Nonsynonymous coding	32%
MM04T	STRBP	spermatid perinuclear RNA binding protein	CCDS6851.1	chr9_125936024-125936024_T_G	108M>L	Substitution	Nonsynonymous coding	32%
MM04T	STRIP1	striatin interacting protein 1	CCDS30798.1	chr1_110582071-110582071_A_C	172N>T	Substitution	Nonsynonymous coding	33%
MM04T	STX10	syntaxin 10	CCDS32922.1	chr19_13259856-13259856_C_A	117R>M	Substitution	Nonsynonymous coding	22%
MM04T	STX10	syntaxin 10	CCDS32922.1	chr19_13260350-13260350_C_A	88R>I	Substitution	Nonsynonymous coding	18%
MM04T	STX17	syntaxin 17	CCDS6745.1	chr9_102713550-102713550_G_T	133R>I	Substitution	Nonsynonymous coding	26%
MM04T	STX7	syntaxin 7	CCDS5153.1	chr6_132791127-132791127_A_C	167L>R	Substitution	Nonsynonymous coding	29%
MM04T	STXBP1	syntaxin binding protein 1	CCDS6874.1	chr9_130420666-130420666_T_G	61I>S	Substitution	Nonsynonymous coding	29%
MM04T	STYK1	serine/threonine/tyrosine kinase 1	CCDS8629.1	chr12_10772871-10772871_C_A	381E>X	Substitution	Nonsense	20%
MM04T	SUCO	SUN domain containing ossification factor	CCDS1303.1	chr1_172579251-172579251_G_A	1206R>Q	Substitution	Nonsynonymous coding	29%
MM04T	SUDS3	suppressor of defective silencing 3 homolog (S. cerevisiae)	CCDS44993.1	chr12_118828982-118828982_G_T	138E>X	Substitution	Nonsense	34%
MM04T	SUGP1	SURP and G patch domain containing 1	CCDS12399.1	chr19_19427311-19427311_C_A	42K>N	Substitution	Nonsynonymous coding	24%
MM04T	SUGP2	SURP and G patch domain containing 2	CCDS12392.1	chr19_19115419-19115419_G_T	829F>L	Substitution	Nonsynonymous coding	25%
MM04T	SULF1	sulfatase 1	CCDS6204.1	chr8_70476278-70476278_C_T	23S>L	Substitution	Nonsynonymous coding	33%
MM04T	SULT1C3	sulfotransferase family, cytosolic, 1C, member 3	CCDS33267.1	chr2_108869887-108869887_G_T	130E>X	Substitution	Nonsense	24%
MM04T	SULT1E1	sulfotransferase family 1E, estrogen-preferring, member 1	CCDS3531.1	chr4_70713477-70713477_G_T	177S>Y	Substitution	Nonsynonymous coding	18%
MM04T	SULT4A1	sulfotransferase family 4A, member 1	CCDS14051.1	chr22_44258124-44258124_C_T	47D>N	Substitution	Nonsynonymous coding	18%
MM04T	SULT6B1	sulfotransferase family, cytosolic, 6B, member 1	CCDS33182.1	chr2_37398583-37398583_G_A	221R>C	Substitution	Nonsynonymous coding	24%
MM04T	SUN1	Sad1 and UNC84 domain containing 1	CCDS47525.1	chr7_912948-912948_C_A	780H>N	Substitution	Nonsynonymous coding	33%
MM04T	SUPT20HL2	suppressor of Ty 20 homolog (S. cerevisiae)-like 2	NM_001136233	chrX_24330937-24330937_G_T	166L>I	Substitution	Nonsynonymous coding	16%
MM04T	SUPV3L1	suppressor of var1, 3-like 1 (S. cerevisiae)	CCDS7287.1	chr10_70940175-70940175_C_A	43S>Y	Substitution	Nonsynonymous coding	30%
MM04T	SUSD5	sushi domain containing 5	CCDS46787.1	chr3_33194501-33194501_G_T	541F>L	Substitution	Nonsynonymous coding	24%
MM04T	SUV39H1	suppressor of variegation 3-9 homolog 1 (Drosophila)	CCDS14304.1	chrX_48557311-48557311_A_C	13K>T	Substitution	Nonsynonymous coding	11%
MM04T	SUV420H1	suppressor of variegation 4-20 homolog 1 (Drosophila)	CCDS31623.1	chr11_67925352-67925352_A_G	821Y>H	Substitution	Nonsynonymous coding	27%
MM04T	SUV420H1	suppressor of variegation 4-20 homolog 1 (Drosophila)	CCDS31623.1	chr11_67925927-67925927_G_T	629S>Y	Substitution	Nonsynonymous coding	25%
MM04T	SVEP1	sushi, von Willebrand factor type A, EGF and pentraxin domain containing 1	CCDS48004.1	chr9_113139637-113139637_C_T	3473R>Q	Substitution	Nonsynonymous coding	29%
MM04T	SVIL	supervillin	CCDS7164.1	chr10_29822347-29822347_G_A	317R>X	Substitution	Nonsense	39%
MM04T	SVOPL	SVOP-like	CCDS47721.1	chr7_138344730-138344730_G_T	117L>M	Substitution	Nonsynonymous coding	20%
MM04T	SWT1	SWT1 RNA endoribonuclease homolog (S. cerevisiae)	CCDS1367.1	chr1_185129981-185129981_G_T	3S>I	Substitution	Nonsynonymous coding	27%

MM04T	SWT1	SWT1 RNA endoribonuclease homolog (S. cerevisiae)	CCDS1367.1	chr1_185183670-185183670_A_C	668K>N	Substitution	Nonsynonymous coding	33%
MM04T	SYAP1	synapse associated protein 1	CCDS14177.1	chrX_16754348-16754348_G_T	118K>N	Substitution	Nonsynonymous coding	23%
MM04T	SYCE1L	synaptonemal complex central element protein 1-like	CCDS45533.1	chr16_77242167-77242167_G_A	NA	Substitution	Splice site donor	19%
MM04T	SYCE2	synaptonemal complex central element protein 2	CCDS42509.1	chr19_13030056-13030056_C_T	NA	Substitution	Splice site donor	31%
MM04T	SYCP2	synaptonemal complex protein 2	CCDS13482.1	chr20_58476852-58476852_C_A	349K>N	Substitution	Nonsynonymous coding	17%
MM04T	SYCP2	synaptonemal complex protein 2	CCDS13482.1	chr20_58490599-58490599_T_G	174K>Q	Substitution	Nonsynonymous coding	27%
MM04T	SYCP2	synaptonemal complex protein 2	CCDS13482.1	chr20_58461886-58461886_C_A	790E>X	Substitution	Nonsense	19%
MM04T	SYDE2	synapse defective 1, Rho GTPase, homolog 2 (C. elegans)	CCDS44169.1	chr1_85648754-85648754_C_T	524R>Q	Substitution	Nonsynonymous coding	27%
MM04T	SYDE2	synapse defective 1, Rho GTPase, homolog 2 (C. elegans)	CCDS44169.1	chr1_85655833-85655833_C_A	450E>X	Substitution	Nonsense	23%
MM04T	SYN1	synapsin I	CCDS14280.1	chrX_47435923-47435923_C_A	318K>N	Substitution	Nonsynonymous coding	37%
MM04T	SYN2	synapsin II	ENST00000341648	chr3_12208742-12208742_C_T	261S>L	Substitution	Nonsynonymous coding	19%
MM04T	SYN3	synapsin III	CCDS13908.1	chr22_33265081-33265081_G_A	165R>C	Substitution	Nonsynonymous coding	31%
MM04T	SYNCRIP	synaptotagmin binding, cytoplasmic RNA interacting protein	CCDS5005.1	chr6_86325058-86325058_C_T	430D>N	Substitution	Nonsynonymous coding	41%
MM04T	SYNCRIP	synaptotagmin binding, cytoplasmic RNA interacting protein	CCDS5005.1	chr6_86332226-86332226_C_A	328D>Y	Substitution	Nonsynonymous coding	40%
MM04T	SYNE1	spectrin repeat containing, nuclear envelope 1	CCDS5236.1	chr6_152485317-152485317_C_A	7924R>I	Substitution	Nonsynonymous coding	30%
MM04T	SYNE1	spectrin repeat containing, nuclear envelope 1	CCDS5236.1	chr6_152642435-152642435_A_C	5392L>V	Substitution	Nonsynonymous coding	25%
MM04T	SYNE1	spectrin repeat containing, nuclear envelope 1	CCDS5236.1	chr6_152644767-152644767_C_T	5255D>N	Substitution	Nonsynonymous coding	20%
MM04T	SYNE1	spectrin repeat containing, nuclear envelope 1	CCDS5236.1	chr6_152647610-152647610_A_C	5038F>L	Substitution	Nonsynonymous coding	14%
MM04T	SYNE1	spectrin repeat containing, nuclear envelope 1	CCDS5236.1	chr6_152671455-152671455_G_T	3917L>M	Substitution	Nonsynonymous coding	19%
MM04T	SYNE1	spectrin repeat containing, nuclear envelope 1	CCDS5236.1	chr6_152804341-152804341_G_T	410P>H	Substitution	Nonsynonymous coding	27%
MM04T	SYNE1	spectrin repeat containing, nuclear envelope 1	CCDS5236.1	chr6_152774744-152774744_G_A	1002R>X	Substitution	Nonsense	25%
MM04T	SYNE2	spectrin repeat containing, nuclear envelope 2	CCDS9761.2	chr14_64519708-64519708__A	NA	Insertion	Frameshift	24%
MM04T	SYNE2	spectrin repeat containing, nuclear envelope 2	CCDS9761.2	chr14_64488613-64488613_G_T	1797M>I	Substitution	Nonsynonymous coding	29%
MM04T	SYNE2	spectrin repeat containing, nuclear envelope 2	CCDS9761.2	chr14_64492071-64492071_G_T	2062E>X	Substitution	Nonsense	23%
MM04T	SYNJ1	synaptojanin 1	CCDS33539.1	chr21_34037251-34037251_T_C	759Q>R	Substitution	Nonsynonymous coding	10%
MM04T	SYNJ1	synaptojanin 1	CCDS33539.1	chr21_34038374-34038374_C_T	675R>Q	Substitution	Nonsynonymous coding	27%
MM04T	SYNJ1	synaptojanin 1	CCDS33539.1	chr21_34025669-34025669_C_A	935E>X	Substitution	Nonsense	32%
MM04T	SYNJ2	synaptojanin 2	CCDS5254.1	chr6_158507892-158507892_G_A	1072A>T	Substitution	Nonsynonymous coding	33%
MM04T	SYNM	synemin, intermediate filament protein	ENST00000336292	chr15_99672165-99672165_G_T	1199W>C	Substitution	Nonsynonymous coding	23%
MM04T	SYNPO2	synaptopodin 2	CCDS34054.1	chr4_119953152-119953152_C_A	1074F>L	Substitution	Nonsynonymous coding	36%
MM04T	SYT14	synaptotagmin XIV	ENST00000399639	chr4_68928550-68928550_G_T	93L>I	Substitution	Nonsynonymous coding	41%
MM04T	SYT15	synaptotagmin XV	CCDS44376.1	chr10_46969397-46969397_T_C	22I>V	Substitution	Nonsynonymous coding	18%

MM04T	SYTL2	synaptotagmin-like 2	ENST00000359152	chr11_85439058-85439058_G_T	5S>Y	Substitution	Nonsynonymous coding	49%
MM04T	SYTL2	synaptotagmin-like 2	CCDS31651.1	chr11_85436218-85436218_C_A	428E>X	Substitution	Nonsense	22%
MM04T	SYTL2	synaptotagmin-like 2	CCDS31651.1	chr11_85437493-85437493_C_A	3E>X	Substitution	Nonsense	28%
MM04T	SYTL2	synaptotagmin-like 2	ENST00000359152	chr11_85438237-85438237_C_A	279E>X	Substitution	Nonsense	32%
MM04T	SYTL3	synaptotagmin-like 3	CCDS34563.1	chr6_159086482-159086482_T_C	56W>R	Substitution	Nonsynonymous coding	18%
MM04T	TAB2	TGF-beta activated kinase 1/MAP3K7 binding protein 2	CCDS5214.1	chr6_149730820-149730820_C_T	683R>C	Substitution	Nonsynonymous coding	34%
MM04T	TAB3	TGF-beta activated kinase 1/MAP3K7 binding protein 3	CCDS14226.1	chrX_30861155-30861155_G_A	606P>S	Substitution	Nonsynonymous coding	34%
MM04T	TADA1	transcriptional adaptor 1	CCDS1255.1	chr1_166826906-166826906_T_G	302E>D	Substitution	Nonsynonymous coding	31%
MM04T	TAF1	TAF1 RNA polymerase II, TATA box binding protein (TBP)-associated factor, 250kDa	CCDS14412.1	chrX_70626501-70626501_C_T	1358R>C	Substitution	Nonsynonymous coding	39%
MM04T	TAF1A	TATA box binding protein (TBP)-associated factor, RNA polymerase I, A, 48kDa	CCDS1531.1	chr1_222750874-222750874_C_T	173E>K	Substitution	Nonsynonymous coding	32%
MM04T	TAF1B	TATA box binding protein (TBP)-associated factor, RNA polymerase I, B, 63kDa	CCDS33143.1	chr2_9989571-9989571__A	NA	Insertion	Frameshift	12%
MM04T	TAF1B	TATA box binding protein (TBP)-associated factor, RNA polymerase I, B, 63kDa	CCDS33143.1	chr2_9989503-9989503_G_T	40R>I	Substitution	Nonsynonymous coding	13%
MM04T	TAF1L	TAF1 RNA polymerase II, TATA box binding protein (TBP)-associated factor, 210kDa-like	NM_153809	chr9_32631814-32631814_C_T	1255R>Q	Substitution	Nonsynonymous coding	25%
MM04T	TAF1L	TAF1 RNA polymerase II, TATA box binding protein (TBP)-associated factor, 210kDa-like	NM_153809	chr9_32633029-32633029_T_A	850K>I	Substitution	Nonsynonymous coding	38%
MM04T	TAF5	TAF5 RNA polymerase II, TATA box binding protein (TBP)-associated factor, 100kDa	CCDS7547.1	chr10_105147440-105147440_G_T	724E>X	Substitution	Nonsense	45%
MM04T	TAF9	TAF9 RNA polymerase II, TATA box binding protein (TBP)-associated factor, 32kDa	CCDS4001.1	chr5_68662387-68662387_C_A	22E>X	Substitution	Nonsense	35%
MM04T	TAF9B	TAF9B RNA polymerase II, TATA box binding protein (TBP)-associated factor, 31kDa	CCDS35340.1	chrX_77394369-77394369_A_T	35V>D	Substitution	Nonsynonymous coding	52%
MM04T	TAMM41	TAM41, mitochondrial translocator assembly and maintenance protein, homolog (S.	ENST00000444133	chr3_11849223-11849223_G_T	341L>I	Substitution	Nonsynonymous coding	23%
MM04T	TAMM41	TAM41, mitochondrial translocator assembly and maintenance protein, homolog (S.	CCDS2607.1	chr3_11858803-11858803_G_A	191R>W	Substitution	Nonsynonymous coding	24%
MM04T	TANC1	tetratricopeptide repeat, ankyrin repeat and coiled-coil containing 1	CCDS42766.1	chr2_160043438-160043438_C_A	882S>Y	Substitution	Nonsynonymous coding	31%
MM04T	TAP1	transporter 1, ATP-binding cassette, sub-family B (MDR/TAP)	CCDS4758.1	chr6_32818742-32818742_C_A	403K>N	Substitution	Nonsynonymous coding	54%
MM04T	TARS	threonyl-tRNA synthetase	CCDS3899.1	chr5_33445480-33445480_A_C	36K>N	Substitution	Nonsynonymous coding	24%
MM04T	TAS1R3	taste receptor, type 1, member 3	CCDS30556.1	chr1_1269769-1269769_C_A	828F>L	Substitution	Nonsynonymous coding	23%
MM04T	TAS2R16	taste receptor, type 2, member 16	CCDS5785.1	chr7_122635289-122635289_G_T	134L>M	Substitution	Nonsynonymous coding	37%
MM04T	TAS2R42	taste receptor, type 2, member 42	CCDS31747.1	chr12_11339039-11339039_C_A	169D>Y	Substitution	Nonsynonymous coding	41%
MM04T	TAS2R7	taste receptor, type 2, member 7	CCDS8631.1	chr12_10954430-10954430_T_C	247Y>C	Substitution	Nonsynonymous coding	35%
MM04T	TAS2R7	taste receptor, type 2, member 7	CCDS8631.1	chr12_10954796-10954796_C_A	125R>I	Substitution	Nonsynonymous coding	28%
MM04T	TBC1D2	TBC1 domain family, member 2	CCDS35080.1	chr9_100971314-100971314_C_T	596E>K	Substitution	Nonsynonymous coding	28%
MM04T	TBC1D22B	TBC1 domain family, member 22B	CCDS4832.1	chr6_37298920-37298920_C_T	497A>V	Substitution	Nonsynonymous coding	31%
MM04T	TBC1D23	TBC1 domain family, member 23	CCDS2936.1	chr3_100039661-100039661_G_T	607E>X	Substitution	Nonsense	21%
MM04T	TBC1D30	TBC1 domain family, member 30	ENST00000229088	chr12_65268826-65268826_C_A	678S>Y	Substitution	Nonsynonymous coding	31%
MM04T	TBC1D4	TBC1 domain family, member 4	CCDS41901.1	chr13_76055721-76055721_C_A	61E>D	Substitution	Nonsynonymous coding	22%

MM04T	TBC1D8	TBC1 domain family, member 8 (with GRAM domain)	CCDS46375.1	chr2_101667028-101667028_C_T	221R>Q	Substitution	Nonsynonymous coding	35%
MM04T	TBC1D8B	TBC1 domain family, member 8B (with GRAM domain)	CCDS14522.1	chrX_106066630-106066630_G_T	254R>I	Substitution	Nonsynonymous coding	16%
MM04T	TBCE	tubulin folding cofactor E	CCDS1605.1	chr1_235577788-235577788_A_C	76N>H	Substitution	Nonsynonymous coding	32%
MM04T	TBCEL	tubulin folding cofactor E-like	CCDS31692.1	chr11_120925829-120925829_C_A	175S>Y	Substitution	Nonsynonymous coding	28%
MM04T	TBCK	TBC1 domain containing kinase	CCDS3673.1	chr4_107173088-107173088_G_T	115P>T	Substitution	Nonsynonymous coding	26%
MM04T	TBL1XR1	transducin (beta)-like 1 X-linked receptor 1	CCDS46961.1	chr3_176767924-176767924_G_A	188S>F	Substitution	Nonsynonymous coding	23%
MM04T	TBL3	transducin (beta)-like 3	CCDS10453.1	chr16_2025538-2025538_C_T	272R>C	Substitution	Nonsynonymous coding	26%
MM04T	TBP	TATA box binding protein	CCDS5315.1	chr6_170873706-170873706_G_A	191E>K	Substitution	Nonsynonymous coding	35%
MM04T	TBP	TATA box binding protein	CCDS5315.1	chr6_170881327-170881327_C_A	332L>I	Substitution	Nonsynonymous coding	24%
MM04T	TBR1	T-box, brain, 1	CCDS33310.1	chr2_162280636-162280636_G_T	649E>D	Substitution	Nonsynonymous coding	22%
MM04T	TBX19	T-box 19	CCDS1272.1	chr1_168260583-168260583_A_C	130N>T	Substitution	Nonsynonymous coding	33%
MM04T	TBX19	T-box 19	CCDS1272.1	chr1_168262449-168262449_G_A	179R>Q	Substitution	Nonsynonymous coding	22%
MM04T	TCEA1	transcription elongation factor A (SII), 1	CCDS47858.1	chr8_54897066-54897066_C_A	179E>X	Substitution	Nonsense	28%
MM04T	TCEAL1	transcription elongation factor A (SII)-like 1	CCDS35358.1	chrX_102885100-102885100_G_A	86E>K	Substitution	Nonsynonymous coding	21%
MM04T	TCEAL2	transcription elongation factor A (SII)-like 2	CCDS14496.1	chrX_101381954-101381954_G_A	51G>D	Substitution	Nonsynonymous coding	29%
MM04T	TCEAL4	transcription elongation factor A (SII)-like 4	CCDS14510.2	chrX_102841986-102841986_G_T	128R>I	Substitution	Nonsynonymous coding	20%
MM04T	TCEAL4	transcription elongation factor A (SII)-like 4	CCDS14510.2	chrX_102842075-102842075_G_T	158E>X	Substitution	Nonsense	14%
MM04T	TCEANC	transcription elongation factor A (SII) N-terminal and central domain containing	CCDS48081.1	chrX_13681370-13681370_C_T	278T>M	Substitution	Nonsynonymous coding	18%
MM04T	TCERG1	transcription elongation regulator 1	CCDS4282.1	chr5_145883501-145883501_G_T	888D>Y	Substitution	Nonsynonymous coding	17%
MM04T	TCERG1	transcription elongation regulator 1	CCDS4282.1	chr5_145886637-145886637_G_A	926R>Q	Substitution	Nonsynonymous coding	16%
MM04T	TCF20	transcription factor 20 (AR1)	CCDS14033.1	chr22_42565883-42565883_C_A	1923E>D	Substitution	Nonsynonymous coding	24%
MM04T	TCF4	transcription factor 4	CCDS42438.1	chr18_53128297-53128297_C_T	86G>E	Substitution	Nonsynonymous coding	24%
MM04T	TCF4	transcription factor 4	CCDS42438.1	chr18_53254287-53254287_C_T	21D>N	Substitution	Nonsynonymous coding	25%
MM04T	TCF7L2	transcription factor 7-like 2 (T-cell specific, HMG-box)	CCDS7576.1	chr10_114711306-114711306_G_T	107M>I	Substitution	Nonsynonymous coding	22%
MM04T	TCF7L2	transcription factor 7-like 2 (T-cell specific, HMG-box)	CCDS7576.1	chr10_114911535-114911535_G_T	328K>N	Substitution	Nonsynonymous coding	21%
MM04T	TCHH	trichohyalin	CCDS41396.1	chr1_152081894-152081894_G_T	1267L>M	Substitution	Nonsynonymous coding	32%
MM04T	TCHH	trichohyalin	CCDS41396.1	chr1_152086421-152086421_G_A	46R>W	Substitution	Nonsynonymous coding	35%
MM04T	TCP11L2	t-complex 11, testis-specific-like 2	CCDS9104.1	chr12_106715304-106715304_G_A	152R>H	Substitution	Nonsynonymous coding	27%
MM04T	TCTEX1D2	Tctex1 domain containing 2	CCDS33929.1	chr3_196033829-196033829_C_A	101R>I	Substitution	Nonsynonymous coding	28%
MM04T	TDRD1	tudor domain containing 1	CCDS7588.1	chr10_115947709-115947709_C_A	40S>Y	Substitution	Nonsynonymous coding	25%
MM04T	TDRD1	tudor domain containing 1	CCDS7588.1	chr10_115970346-115970346_C_A	464P>H	Substitution	Nonsynonymous coding	19%
MM04T	TDRD15	tudor domain containing 15	ENST00000405799	chr2_21361432-21361432_G_A	365D>N	Substitution	Nonsynonymous coding	28%

MM04T	TDRD15	tudor domain containing 15	ENST00000405799	chr2_21362186-21362186_T_A	616F>Y	Substitution	Nonsynonymous coding	33%
MM04T	TDRD15	tudor domain containing 15	ENST00000405799	chr2_21364080-21364080_G_T	1247E>D	Substitution	Nonsynonymous coding	28%
MM04T	TDRD7	tudor domain containing 7	CCDS6725.1	chr9_100193285-100193285_C_A	93S>Y	Substitution	Nonsynonymous coding	29%
MM04T	TDRD7	tudor domain containing 7	CCDS6725.1	chr9_100194358-100194358_T_C	134V>A	Substitution	Nonsynonymous coding	21%
MM04T	TEAD2	TEA domain family member 2	CCDS12761.1	chr19_49858456-49858456_T_G	159E>A	Substitution	Nonsynonymous coding	31%
MM04T	TEAD4	TEA domain family member 4	CCDS41737.1	chr12_3149557-3149557_G_T	277E>D	Substitution	Nonsynonymous coding	26%
MM04T	TEC	tec protein tyrosine kinase	CCDS3481.1	chr4_48139450-48139450_C_A	627E>X	Substitution	Nonsense	25%
MM04T	TECPR1	tectonin beta-propeller repeat containing 1	CCDS47648.1	chr7_97875349-97875349_C_T	37R>H	Substitution	Nonsynonymous coding	18%
MM04T	TECTA	tectorin alpha	CCDS8434.1	chr11_121000423-121000423_C_T	815T>M	Substitution	Nonsynonymous coding	23%
MM04T	TECTA	tectorin alpha	CCDS8434.1	chr11_121028872-121028872_C_T	1543S>L	Substitution	Nonsynonymous coding	20%
MM04T	TEDDM1	transmembrane epididymal protein 1	CCDS30953.1	chr1_182368964-182368964_G_T	219F>L	Substitution	Nonsynonymous coding	24%
MM04T	TEFM	transcription elongation factor, mitochondrial	CCDS42291.1	chr17_29226197-29226197_G_T	358S>Y	Substitution	Nonsynonymous coding	19%
MM04T	TENM1	teneurin transmembrane protein 1	CCDS14609.1	chrX_123517636-123517636_T_C	2375Q>R	Substitution	Nonsynonymous coding	20%
MM04T	TENM1	teneurin transmembrane protein 1	CCDS14609.1	chrX_123518682-123518682_A_C	2026I>M	Substitution	Nonsynonymous coding	12%
MM04T	TENM1	teneurin transmembrane protein 1	CCDS14609.1	chrX_123539096-123539096_G_A	1719R>W	Substitution	Nonsynonymous coding	35%
MM04T	TENM1	teneurin transmembrane protein 1	CCDS14609.1	chrX_123554354-123554354_G_A	1590R>C	Substitution	Nonsynonymous coding	32%
MM04T	TENM1	teneurin transmembrane protein 1	CCDS14609.1	chrX_123654439-123654439_G_T	1077L>I	Substitution	Nonsynonymous coding	34%
MM04T	TENM2	teneurin transmembrane protein 2	NM_001122679	chr5_167545300-167545300_C_T	606A>V	Substitution	Nonsynonymous coding	33%
MM04T	TENM2	teneurin transmembrane protein 2	NM_001122679	chr5_167645405-167645405_G_T	1494Q>H	Substitution	Nonsynonymous coding	28%
MM04T	TENM2	teneurin transmembrane protein 2	NM_001122679	chr5_167638746-167638746_G_T	1269E>X	Substitution	Nonsense	44%
MM04T	TENM3	teneurin transmembrane protein 3	CCDS47165.1	chr4_183267917-183267917_T_G	116S>A	Substitution	Nonsynonymous coding	38%
MM04T	TENM3	teneurin transmembrane protein 3	CCDS47165.1	chr4_183717814-183717814_T_G	2413F>C	Substitution	Nonsynonymous coding	36%
MM04T	TENM4	teneurin transmembrane protein 4	CCDS44688.1	chr11_78423578-78423578_G_A	1335R>C	Substitution	Nonsynonymous coding	36%
MM04T	TEP1	telomerase-associated protein 1	CCDS9548.1	chr14_20853258-20853258_T_C	998Y>C	Substitution	Nonsynonymous coding	38%
MM04T	TEP1	telomerase-associated protein 1	CCDS9548.1	chr14_20869271-20869271_C_T	474R>Q	Substitution	Nonsynonymous coding	35%
MM04T	TES	testis derived transcript (3 LIM domains)	CCDS5763.1	chr7_115850785-115850785_G_T	8K>N	Substitution	Nonsynonymous coding	24%
MM04T	TESC	tescalcin	CCDS9183.2	chr12_117513077-117513077_G_A	96R>C	Substitution	Nonsynonymous coding	25%
MM04T	TET1	tet methylcytosine dioxygenase 1	CCDS7281.1	chr10_70332695-70332695_G_T	200K>N	Substitution	Nonsynonymous coding	36%
MM04T	TET1	tet methylcytosine dioxygenase 1	CCDS7281.1	chr10_70332922-70332922_G_A	276R>Q	Substitution	Nonsynonymous coding	33%
MM04T	TET1	tet methylcytosine dioxygenase 1	CCDS7281.1	chr10_70405912-70405912_A_C	1142Q>H	Substitution	Nonsynonymous coding	37%
MM04T	TET1	tet methylcytosine dioxygenase 1	CCDS7281.1	chr10_70441163-70441163_A_G	1611N>S	Substitution	Nonsynonymous coding	19%
MM04T	TET2	tet methylcytosine dioxygenase 2	CCDS47120.1	chr4_106197675-106197675_G_T	2003*>L	Substitution	Nonsynonymous coding	31%

MM04T	TEX10	testis expressed 10	CCDS6748.1	chr9_103102539-103102539_C_A	417S>I	Substitution	Nonsynonymous coding	26%
MM04T	TEX11	testis expressed 11	CCDS35323.1	chrX_69826850-69826850_C_A	652D>Y	Substitution	Nonsynonymous coding	19%
MM04T	TEX11	testis expressed 11	CCDS35323.1	chrX_69945124-69945124_C_A	320E>X	Substitution	Nonsense	16%
MM04T	TEX13A	testis expressed 13A	ENST00000413579	chrX_104464065-104464065_C_T	271E>K	Substitution	Nonsynonymous coding	22%
MM04T	TEX14	testis expressed 14	CCDS32693.1	chr17_56649433-56649433_A_C	1229F>C	Substitution	Nonsynonymous coding	20%
MM04T	TEX14	testis expressed 14	CCDS32693.1	chr17_56688673-56688673_C_A	345E>X	Substitution	Nonsense	21%
MM04T	TEX15	testis expressed 15	CCDS6080.1	chr8_30694699-30694699_A_G	2651L>S	Substitution	Nonsynonymous coding	33%
MM04T	TEX15	testis expressed 15	CCDS6080.1	chr8_30695125-30695125_G_A	2509S>L	Substitution	Nonsynonymous coding	27%
MM04T	TEX19	testis expressed 19	CCDS11809.1	chr17_80320120-80320120_A_C	32S>R	Substitution	Nonsynonymous coding	18%
MM04T	TEX2	testis expressed 2 [Source:HGNC Symbol;Acc:30884]	CCDS11658.1	chr17_62265791-62265791_C_T	728V>I	Substitution	Nonsynonymous coding	31%
MM04T	TEX35	testis expressed 35	CCDS1323.1	chr1_178489560-178489560_G_T	110K>N	Substitution	Nonsynonymous coding	22%
MM04T	TFAP2C	transcription factor AP-2 gamma (activating enhancer binding protein 2 gamma)	CCDS13454.1	chr20_55209214-55209214_C_T	271S>L	Substitution	Nonsynonymous coding	36%
MM04T	TFAP2D	transcription factor AP-2 delta (activating enhancer binding protein 2 delta)	CCDS4933.1	chr6_50712897-50712897_C_T	321L>F	Substitution	Nonsynonymous coding	18%
MM04T	TFAP2D	transcription factor AP-2 delta (activating enhancer binding protein 2 delta)	CCDS4933.1	chr6_50712891-50712891_G_T	319E>X	Substitution	Nonsense	18%
MM04T	TFAP2E	transcription factor AP-2 epsilon (activating enhancer binding protein 2 epsilon)	CCDS393.2	chr1_36053963-36053963_G_T	199A>S	Substitution	Nonsynonymous coding	20%
MM04T	TFB2M	transcription factor B2, mitochondrial	CCDS1627.1	chr1_246720809-246720809_G_T	144L>I	Substitution	Nonsynonymous coding	20%
MM04T	TFCP2	transcription factor CP2	CCDS8808.1	chr12_51500392-51500392_G_T	278S>Y	Substitution	Nonsynonymous coding	21%
MM04T	TFCP2L1	transcription factor CP2-like 1	CCDS2134.1	chr2_121991760-121991760_G_A	369P>S	Substitution	Nonsynonymous coding	39%
MM04T	TFDP3	transcription factor Dp family, member 3	CCDS14636.2	chrX_132352030-132352030_C_A	86Q>H	Substitution	Nonsynonymous coding	31%
MM04T	TFE3	transcription factor binding to IGHM enhancer 3	CCDS14315.3	chrX_48898016-48898016_C_T	66E>K	Substitution	Nonsynonymous coding	39%
MM04T	TFEC	transcription factor EC	CCDS5762.1	chr7_115580925-115580925_C_T	242D>N	Substitution	Nonsynonymous coding	33%
MM04T	TFPI	tissue factor pathway inhibitor (lipoprotein-associated coagulation inhibitor)	CCDS2294.1	chr2_188332523-188332523_G_T	255N>K	Substitution	Nonsynonymous coding	39%
MM04T	TFPI2	tissue factor pathway inhibitor 2	CCDS5632.1	chr7_93518351-93518351_C_A	152K>N	Substitution	Nonsynonymous coding	35%
MM04T	TG	thyroglobulin	CCDS34944.1	chr8_133898716-133898716_G_A	367E>K	Substitution	Nonsynonymous coding	20%
MM04T	TGFB1I1	transforming growth factor beta 1 induced transcript 1	CCDS42156.1	chr16_31485002-31485002_G_T	NA	Substitution	Splice site acceptor	36%
MM04T	TGFBI	transforming growth factor, beta-induced, 68kDa	CCDS47266.1	chr5_135391405-135391405_G_A	483D>N	Substitution	Nonsynonymous coding	19%
MM04T	TGFBR3	transforming growth factor, beta receptor III	CCDS30770.1	chr1_92181891-92181891_T_G	590N>H	Substitution	Nonsynonymous coding	31%
MM04T	TGFBRAP1	transforming growth factor, beta receptor associated protein 1	CCDS2067.1	chr2_105885896-105885896_C_T	747E>K	Substitution	Nonsynonymous coding	32%
MM04T	TGIF1	TGFB-induced factor homeobox 1	CCDS11834.1	chr18_3456370-3456370_G_A	141G>D	Substitution	Nonsynonymous coding	28%
MM04T	TGM1	transglutaminase 1 (K polypeptide epidermal type I, protein-glutamine-gamma-	CCDS9622.1	chr14_24729217-24729217_G_T	269L>I	Substitution	Nonsynonymous coding	20%
MM04T	TGM3	transglutaminase 3 (E polypeptide, protein-glutamine-gamma-glutamyltransferase)	CCDS33435.1	chr20_2308885-2308885_G_A	403D>N	Substitution	Nonsynonymous coding	34%
MM04T	TGM3	transglutaminase 3 (E polypeptide, protein-glutamine-gamma-glutamyltransferase)	CCDS33435.1	chr20_2315797-2315797_T_C	560Y>H	Substitution	Nonsynonymous coding	17%

MM04T	TGM4	transglutaminase 4 (prostate)	CCDS2723.1	chr3_44951595-44951595_G_T	447E>D	Substitution	Nonsynonymous coding	20%
MM04T	TGM5	transglutaminase 5	CCDS32212.1	chr15_43531034-43531034_C_A	442E>D	Substitution	Nonsynonymous coding	37%
MM04T	TGM7	transglutaminase 7	CCDS32213.1	chr15_43571350-43571350_C_A	602D>Y	Substitution	Nonsynonymous coding	11%
MM04T	TH	tyrosine hydroxylase	CCDS7731.1	chr11_2189843-2189843_C_T	153R>Q	Substitution	Nonsynonymous coding	14%
MM04T	THAP6	THAP domain containing 6	CCDS3568.1	chr4_76440715-76440715_C_T	18S>L	Substitution	Nonsynonymous coding	30%
MM04T	THBS2	thrombospondin 2	CCDS34574.1	chr6_169632281-169632281_C_T	649E>K	Substitution	Nonsynonymous coding	32%
MM04T	THBS3	thrombospondin 3	CCDS1099.1	chr1_155173028-155173028_C_A	248D>Y	Substitution	Nonsynonymous coding	32%
MM04T	THEM4	thioesterase superfamily member 4	CCDS1006.1	chr1_151867487-151867487_G_T	95L>I	Substitution	Nonsynonymous coding	15%
MM04T	THEMIS	thymocyte selection associated	CCDS34534.1	chr6_128150816-128150816_C_A	172E>X	Substitution	Nonsense	31%
MM04T	THEMIS	thymocyte selection associated	CCDS34534.1	chr6_128150906-128150906_C_A	142E>X	Substitution	Nonsense	26%
MM04T	THNSL1	threonine synthase-like 1 (S. cerevisiae)	CCDS7147.1	chr10_25312197-25312197_G_T	15Q>H	Substitution	Nonsynonymous coding	20%
MM04T	THNSL2	threonine synthase-like 2 (S. cerevisiae)	CCDS2002.2	chr2_88482310-88482310_G_A	299D>N	Substitution	Nonsynonymous coding	31%
MM04T	THOC2	THO complex 2	CCDS43988.1	chrX_122805548-122805548_A_G	278L>P	Substitution	Nonsynonymous coding	17%
MM04T	THOC7	THO complex 7 homolog (Drosophila)	CCDS2900.1	chr3_63824159-63824159_G_A	52R>C	Substitution	Nonsynonymous coding	16%
MM04T	THRAP3	thyroid hormone receptor associated protein 3	CCDS405.1	chr1_36754784-36754784_G_T	388M>I	Substitution	Nonsynonymous coding	30%
MM04T	THRB	thyroid hormone receptor, beta	CCDS2641.1	chr3_24164533-24164533_G_A	410R>X	Substitution	Nonsense	36%
MM04T	THSD7B	thrombospondin, type I, domain containing 7B	ENST00000272643	chr2_137814764-137814764_C_T	305S>L	Substitution	Nonsynonymous coding	19%
MM04T	THSD7B	thrombospondin, type I, domain containing 7B	ENST00000272643	chr2_138000044-138000044_G_A	723R>Q	Substitution	Nonsynonymous coding	30%
MM04T	THUMPD3	THUMP domain containing 3	CCDS2573.1	chr3_9406812-9406812_G_T	20Q>H	Substitution	Nonsynonymous coding	25%
MM04T	TIA1	TIA1 cytotoxic granule-associated RNA binding protein	CCDS1901.1	chr2_70457941-70457941_G_A	57R>C	Substitution	Nonsynonymous coding	23%
MM04T	TIA1	TIA1 cytotoxic granule-associated RNA binding protein	CCDS1901.1	chr2_70443374-70443374_G_A	244R>X	Substitution	Nonsense	23%
MM04T	TIA1	TIA1 cytotoxic granule-associated RNA binding protein	CCDS1901.1	chr2_70454919-70454919_C_A	116E>X	Substitution	Nonsense	22%
MM04T	TIAL1	TIA1 cytotoxic granule-associated RNA binding protein-like 1	CCDS31295.1	chr10_121339503-121339503_C_A	148D>Y	Substitution	Nonsynonymous coding	23%
MM04T	TIAM1	T-cell lymphoma invasion and metastasis 1	CCDS13609.1	chr21_32526629-32526629_G_A	1036S>L	Substitution	Nonsynonymous coding	30%
MM04T	TIAM1	T-cell lymphoma invasion and metastasis 1	CCDS13609.1	chr21_32559367-32559367_C_T	871R>Q	Substitution	Nonsynonymous coding	24%
MM04T	TIAM1	T-cell lymphoma invasion and metastasis 1	CCDS13609.1	chr21_32638679-32638679_C_T	204E>K	Substitution	Nonsynonymous coding	22%
MM04T	TIAM2	T-cell lymphoma invasion and metastasis 2	CCDS34558.1	chr6_155465774-155465774_G_T	555K>N	Substitution	Nonsynonymous coding	40%
MM04T	TIAM2	T-cell lymphoma invasion and metastasis 2	CCDS34558.1	chr6_155565208-155565208_G_T	1138D>Y	Substitution	Nonsynonymous coding	22%
MM04T	TIAM2	T-cell lymphoma invasion and metastasis 2	CCDS34558.1	chr6_155565808-155565808_G_A	1145G>E	Substitution	Nonsynonymous coding	23%
MM04T	TICRR	TOPBP1-interacting checkpoint and replication regulator	CCDS10352.2	chr15_90152048-90152048_C_T	913R>X	Substitution	Nonsense	37%
MM04T	TIGD4	tigger transposable element derived 4	CCDS34079.1	chr4_153691967-153691967_G_T	64L>I	Substitution	Nonsynonymous coding	41%
MM04T	TIGD6	tigger transposable element derived 6	CCDS4301.1	chr5_149375524-149375524_G_A	130R>C	Substitution	Nonsynonymous coding	30%

MM04T	TIGD7	tigger transposable element derived 7	CCDS10500.1	chr16_3349411-3349411_G_T	402L>I	Substitution	Nonsynonymous coding	33%
MM04T	TIGD7	tigger transposable element derived 7	CCDS10500.1	chr16_3350489-3350489_A_C	42F>L	Substitution	Nonsynonymous coding	34%
MM04T	TIMELESS	timeless circadian clock	CCDS8918.1	chr12_56822139-56822139_G_A	487R>X	Substitution	Nonsense	29%
MM04T	TIRAP	toll-interleukin 1 receptor (TIR) domain containing adaptor protein	CCDS41731.1	chr11_126162698-126162698_G_A	132E>K	Substitution	Nonsynonymous coding	31%
MM04T	TJP1	tight junction protein 1	CCDS42007.1	chr15_30011117-30011117_G_A	1077R>C	Substitution	Nonsynonymous coding	30%
MM04T	TKT	transketolase	CCDS2871.1	chr3_53262293-53262293_T_G	493K>T	Substitution	Nonsynonymous coding	18%
MM04T	TKTL1	transketolase-like 1	CCDS35448.1	chrX_153549231-153549231_T_G	386I>S	Substitution	Nonsynonymous coding	27%
MM04T	TLE2	transducin-like enhancer of split 2 (E(sp1) homolog, Drosophila)	CCDS45911.1	chr19_3028735-3028735_C_A	31E>X	Substitution	Nonsense	13%
MM04T	TLE3	transducin-like enhancer of split 3 (E(sp1) homolog, Drosophila)	CCDS45293.1	chr15_70356794-70356794_G_A	198A>V	Substitution	Nonsynonymous coding	25%
MM04T	TLL1	tolloid-like 1	CCDS3811.1	chr4_166986953-166986953_G_T	709R>I	Substitution	Nonsynonymous coding	30%
MM04T	TLN1	talin 1	CCDS35009.1	chr9_35707366-35707366_A_C	1584I>M	Substitution	Nonsynonymous coding	34%
MM04T	TLN2	talin 2	CCDS32261.1	chr15_63084892-63084892_T_C	1930F>S	Substitution	Nonsynonymous coding	28%
MM04T	TLR10	toll-like receptor 10	CCDS3445.1	chr4_38775618-38775618_T_G	532N>H	Substitution	Nonsynonymous coding	35%
MM04T	TLR10	toll-like receptor 10	CCDS3445.1	chr4_38774898-38774898_G_A	772R>X	Substitution	Nonsense	30%
MM04T	TLR3	toll-like receptor 3	CCDS3846.1	chr4_187004021-187004021_G_A	394R>Q	Substitution	Nonsynonymous coding	39%
MM04T	TLR4	toll-like receptor 4	CCDS6818.1	chr9_120475851-120475851_C_A	482S>Y	Substitution	Nonsynonymous coding	32%
MM04T	TLR7	toll-like receptor 7	CCDS14151.1	chrX_12905805-12905805_G_T	726K>N	Substitution	Nonsynonymous coding	19%
MM04T	TLR8	toll-like receptor 8	CCDS14152.1	chrX_12937997-12937997_C_T	280R>C	Substitution	Nonsynonymous coding	32%
MM04T	TLR8	toll-like receptor 8	CCDS14152.1	chrX_12940280-12940280_T_C	1041Y>H	Substitution	Nonsynonymous coding	38%
MM04T	TLR8	toll-like receptor 8	CCDS14152.1	chrX_12937491-12937491_C_A	111S>X	Substitution	Nonsense	18%
MM04T	TM4SF1	transmembrane 4 L six family member 1	ENST00000383054	chr3_149087582-149087582_T_G	226I>L	Substitution	Nonsynonymous coding	31%
MM04T	TM9SF1	transmembrane 9 superfamily member 1	CCDS9617.1	chr14_24663920-24663920_C_A	102E>D	Substitution	Nonsynonymous coding	21%
MM04T	TM9SF4	transmembrane 9 superfamily protein member 4	CCDS13196.2	chr20_30733188-30733188_C_A	282S>Y	Substitution	Nonsynonymous coding	33%
MM04T	TMA16	translation machinery associated 16 homolog (S. cerevisiae)	CCDS43278.1	chr4_164440490-164440490_T_C	146W>R	Substitution	Nonsynonymous coding	44%
MM04T	TMA16	translation machinery associated 16 homolog (S. cerevisiae)	CCDS43278.1	chr4_164440507-164440507_G_T	151K>N	Substitution	Nonsynonymous coding	38%
MM04T	TMC3	transmembrane channel-like 3	CCDS45324.1	chr15_81627313-81627313_C_T	736R>Q	Substitution	Nonsynonymous coding	34%
MM04T	TMC4	transmembrane channel-like 4	CCDS46174.1	chr19_54666840-54666840_T_A	450N>Y	Substitution	Nonsynonymous coding	24%
MM04T	TMC7	transmembrane channel-like 7	CCDS10573.1	chr16_19058543-19058543_A_G	571K>R	Substitution	Nonsynonymous coding	30%
MM04T	TMCC3	transmembrane and coiled-coil domain family 3	CCDS31877.1	chr12_94976130-94976130_G_A	88S>L	Substitution	Nonsynonymous coding	19%
MM04T	TMCO2	transmembrane and coiled-coil domains 2	CCDS30684.1	chr1_40717133-40717133_A_C	139K>T	Substitution	Nonsynonymous coding	29%
MM04T	TMEFF2	transmembrane protein with EGF-like and two follistatin-like domains 2	CCDS2314.1	chr2_193056685-193056685_A_G	68L>P	Substitution	Nonsynonymous coding	30%
MM04T	TMEM108	transmembrane protein 108	CCDS33858.1	chr3_133109181-133109181_A_G	v	Substitution	Splice site donor	38%

MM04T	TMEM132B	transmembrane protein 132B	CCDS41859.1	chr12_125811224-125811224_C_T	NA	Substitution	Splice site donor	34%
MM04T	TMEM132B	transmembrane protein 132B	CCDS41859.1	chr12_125834714-125834714_G_T	257E>X	Substitution	Nonsense	30%
MM04T	TMEM132D	transmembrane protein 132D	CCDS9266.1	chr12_129559527-129559527_G_T	731F>L	Substitution	Nonsynonymous coding	27%
MM04T	TMEM132D	transmembrane protein 132D	CCDS9266.1	chr12_130184644-130184644_A_T	227Y>N	Substitution	Nonsynonymous coding	14%
MM04T	TMEM132E	transmembrane protein 132E	CCDS11283.1	chr17_32953155-32953155_G_A	26R>H	Substitution	Nonsynonymous coding	32%
MM04T	TMEM133	transmembrane protein 133	CCDS8309.1	chr11_100863094-100863094_G_A	19E>K	Substitution	Nonsynonymous coding	32%
MM04T	TMEM133	transmembrane protein 133	CCDS8309.1	chr11_100863245-100863245_T_G	69F>C	Substitution	Nonsynonymous coding	35%
MM04T	TMEM164	transmembrane protein 164	CCDS14550.2	chrX_109310581-109310581_C_A	133L>I	Substitution	Nonsynonymous coding	20%
MM04T	TMEM17	transmembrane protein 17	CCDS1871.1	chr2_62728565-62728565_G_T	126L>I	Substitution	Nonsynonymous coding	43%
MM04T	TMEM17	transmembrane protein 17	CCDS1871.1	chr2_62729884-62729884_A_C	49F>C	Substitution	Nonsynonymous coding	26%
MM04T	TMEM19	transmembrane protein 19	CCDS9002.1	chr12_72094678-72094678_A_C	305K>T	Substitution	Nonsynonymous coding	26%
MM04T	TMEM190	transmembrane protein 190	CCDS33113.1	chr19_55889488-55889488_G_T	151E>X	Substitution	Nonsense	13%
MM04T	TMEM194B	transmembrane protein 194B	CCDS46476.1	chr2_191390102-191390102_G_A	34R>C	Substitution	Nonsynonymous coding	27%
MM04T	TMEM198	transmembrane protein 198	CCDS33385.1	chr2_220412363-220412363_G_A	101G>D	Substitution	Nonsynonymous coding	21%
MM04T	TMEM209	transmembrane protein 209	CCDS47712.1	chr7_129841775-129841775_C_T	163S>N	Substitution	Nonsynonymous coding	26%
MM04T	TMEM212	transmembrane protein 212	CCDS46958.1	chr3_171571420-171571420_C_A	108S>X	Substitution	Nonsense	34%
MM04T	TMEM232	transmembrane protein 232	CCDS47253.1	chr5_109963542-109963542_G_T	181L>M	Substitution	Nonsynonymous coding	25%
MM04T	TMEM244	transmembrane protein 244	CCDS34536.1	chr6_130166959-130166959_G_T	24F>L	Substitution	Nonsynonymous coding	21%
MM04T	TMEM255A	transmembrane protein 255A	CCDS14597.1	chrX_119445116-119445116_C_A	17D>Y	Substitution	Nonsynonymous coding	18%
MM04T	TMEM257	transmembrane protein 257	CCDS14681.1	chrX_144909473-144909473_G_T	93R>I	Substitution	Nonsynonymous coding	24%
MM04T	TMEM35	transmembrane protein 35	CCDS14478.1	chrX_100334017-100334017_T_G	9I>S	Substitution	Nonsynonymous coding	28%
MM04T	TMEM45A	transmembrane protein 45A	CCDS2937.1	chr3_100295842-100295842_G_T	270E>X	Substitution	Nonsense	33%
MM04T	TMEM66	transmembrane protein 66	CCDS6074.1	chr8_29927560-29927560_C_T	100D>N	Substitution	Nonsynonymous coding	29%
MM04T	TMEM72	transmembrane protein 72	CCDS41504.1	chr10_45430317-45430317_G_T	188S>I	Substitution	Nonsynonymous coding	27%
MM04T	TMEM87A	transmembrane protein 87A	CCDS32205.1	chr15_42531881-42531881_T_G	224Y>S	Substitution	Nonsynonymous coding	11%
MM04T	TMPRSS11A	transmembrane protease, serine 11A	CCDS3519.1	chr4_68784711-68784711_A_C	314F>C	Substitution	Nonsynonymous coding	20%
MM04T	TMPRSS11A	transmembrane protease, serine 11A	CCDS3519.1	chr4_68784857-68784857_C_A	265E>D	Substitution	Nonsynonymous coding	35%
MM04T	TMPRSS11D	transmembrane protease, serine 11D	CCDS3518.1	chr4_68693020-68693020_G_T	304S>Y	Substitution	Nonsynonymous coding	35%
MM04T	TMPRSS11E	transmembrane protease, serine 11E	CCDS33993.1	chr4_69342129-69342129_T_C	227L>P	Substitution	Nonsynonymous coding	17%
MM04T	TMPRSS12	transmembrane (C-terminal) protease, serine 12	CCDS44881.1	chr12_51237824-51237824_C_T	NA	Substitution	Splice site donor	29%
MM04T	TMPRSS15	transmembrane protease, serine 15	CCDS13571.1	chr21_19725360-19725360_T_G	344K>T	Substitution	Nonsynonymous coding	58%
MM04T	TMPRSS15	transmembrane protease, serine 15	CCDS13571.1	chr21_19726177-19726177_G_T	295S>Y	Substitution	Nonsynonymous coding	29%

MM04T	TMPRSS7	transmembrane protease, serine 7	CCDS43129.2	chr3_111795940-111795940_C_T	599R>C	Substitution	Nonsynonymous coding	28%
MM04T	TMSB4X	thymosin beta 4, X-linked	CCDS35202.1	chrX_12994399-12994399_A_G	7M>V	Substitution	Nonsynonymous coding	22%
MM04T	TMTC1	transmembrane and tetratricopeptide repeat containing 1	CCDS8718.1	chr12_29911671-29911671_G_T	66L>M	Substitution	Nonsynonymous coding	23%
MM04T	TMTC3	transmembrane and tetratricopeptide repeat containing 3	CCDS9032.1	chr12_88547174-88547174_T_G	99F>C	Substitution	Nonsynonymous coding	29%
MM04T	TNFAIP2	tumor necrosis factor, alpha-induced protein 2	CCDS9979.1	chr14_103593952-103593952_G_T	282Q>H	Substitution	Nonsynonymous coding	14%
MM04T	TNFRSF11A	tumor necrosis factor receptor superfamily, member 11a, NFKB activator	CCDS11980.1	chr18_60036114-60036114_G_A	322E>K	Substitution	Nonsynonymous coding	30%
MM04T	TNFRSF8	tumor necrosis factor receptor superfamily, member 8	CCDS144.1	chr1_12157271-12157271_C_T	89R>X	Substitution	Nonsense	25%
MM04T	TNFSF11	tumor necrosis factor (ligand) superfamily, member 11	CCDS9384.1	chr13_43181035-43181035_A_C	312K>T	Substitution	Nonsynonymous coding	22%
MM04T	TNFSF4	tumor necrosis factor (ligand) superfamily, member 4	CCDS1306.1	chr1_173155702-173155702_C_A	169E>X	Substitution	Nonsense	33%
MM04T	TNFSF8	tumor necrosis factor (ligand) superfamily, member 8	CCDS6810.1	chr9_117666452-117666452_G_T	155S>Y	Substitution	Nonsynonymous coding	38%
MM04T	TNIK	TRAF2 and NCK interacting kinase	CCDS46956.1	chr3_170797434-170797434_C_A	1107K>N	Substitution	Nonsynonymous coding	45%
MM04T	TNKS	tankyrase, TRF1-interacting ankyrin-related ADP-ribose polymerase	CCDS5974.1	chr8_9564457-9564457_A_T	469K>I	Substitution	Nonsynonymous coding	30%
MM04T	TNKS	tankyrase, TRF1-interacting ankyrin-related ADP-ribose polymerase	CCDS5974.1	chr8_9620680-9620680_C_A	1100H>N	Substitution	Nonsynonymous coding	23%
MM04T	TNKS2	tankyrase, TRF1-interacting ankyrin-related ADP-ribose polymerase 2	CCDS7417.1	chr10_93593641-93593641_C_A	436S>Y	Substitution	Nonsynonymous coding	25%
MM04T	TNMD	tenomodulin	CCDS14469.1	chrX_99854520-99854520_G_T	254E>X	Substitution	Nonsense	41%
MM04T	TNNC1	troponin C type 1 (slow)	CCDS2857.1	chr3_52485544-52485544_C_A	NA	Substitution	Splice site acceptor	20%
MM04T	TNNI3K	TNNI3 interacting kinase	CCDS44161.1	chr1_75009601-75009601_G_A	916D>N	Substitution	Nonsynonymous coding	35%
MM04T	TNR	tenascin R	CCDS1318.1	chr1_175334385-175334385_G_T	783S>Y	Substitution	Nonsynonymous coding	14%
MM04T	TNRC6B	trinucleotide repeat containing 6B	CCDS46713.1	chr22_40696978-40696978_C_A	1192S>Y	Substitution	Nonsynonymous coding	34%
MM04T	TNS1	tensin 1	CCDS2407.1	chr2_218682432-218682432_G_T	1437F>L	Substitution	Nonsynonymous coding	21%
MM04T	TNS1	tensin 1	CCDS2407.1	chr2_218700826-218700826_C_T	914R>H	Substitution	Nonsynonymous coding	25%
MM04T	TNS3	tensin 3	ENST00000450444	chr7_47481640-47481640_G_T	69S>Y	Substitution	Nonsynonymous coding	17%
MM04T	TONSL	tonsoku-like, DNA repair protein	CCDS34968.2	chr8_145660903-145660903_G_A	936R>X	Substitution	Nonsense	37%
MM04T	TOP2B	topoisomerase (DNA) II beta 180kDa	CCDS46776.1	chr3_25673982-25673982_T_G	402K>N	Substitution	Nonsynonymous coding	31%
MM04T	TOPAZ1	testis and ovary specific PAZ domain containing 1	CCDS46809.1	chr3_44285704-44285704_C_A	569S>Y	Substitution	Nonsynonymous coding	10%
MM04T	TOPBP1	topoisomerase (DNA) II binding protein 1	CCDS46919.1	chr3_133372224-133372224_G_T	296S>Y	Substitution	Nonsynonymous coding	24%
MM04T	TP53	tumor protein p53	CCDS11118.1	chr17_7578182-7578182_G_A	223P>S	Substitution	Nonsynonymous coding	42%
MM04T	TP53BP1	tumor protein p53 binding protein 1	CCDS45250.1	chr15_43748187-43748187_T_G	873K>N	Substitution	Nonsynonymous coding	26%
MM04T	TP53BP2	tumor protein p53 binding protein, 2	CCDS44319.1	chr1_223985988-223985988_C_T	626G>E	Substitution	Nonsynonymous coding	22%
MM04T	TPCN2	two pore segment channel 2	CCDS8189.1	chr11_68853209-68853209_G_A	637D>N	Substitution	Nonsynonymous coding	27%
MM04T	TPD52L3	tumor protein D52-like 3	CCDS34986.1	chr9_6328778-6328778_G_T	61K>N	Substitution	Nonsynonymous coding	23%
MM04T	TPO	thyroid peroxidase	CCDS1643.1	chr2_1459947-1459947_G_A	238D>N	Substitution	Nonsynonymous coding	27%

MM04T	TPP1	tripeptidyl peptidase I	CCDS7770.1	chr11_6638228-6638228_T_G	222N>T	Substitution	Nonsynonymous coding	36%
MM04T	TPP2	tripeptidyl peptidase II	CCDS9502.1	chr13_103266499-103266499_A_C	115K>Q	Substitution	Nonsynonymous coding	33%
MM04T	TPPP2	tubulin polymerization-promoting protein family member 2	CCDS9566.1	chr14_21498830-21498830_C_A	30F>L	Substitution	Nonsynonymous coding	21%
MM04T	TPPP2	tubulin polymerization-promoting protein family member 2	CCDS9566.1	chr14_21499256-21499256_C_A	87P>T	Substitution	Nonsynonymous coding	22%
MM04T	TPTE	transmembrane phosphatase with tensin homology	CCDS13560.2	chr21_10910388-10910388_G_T	456N>K	Substitution	Nonsynonymous coding	17%
MM04T	TPX2	TPX2, microtubule-associated, homolog (Xenopus laevis)	CCDS13190.1	chr20_30366672-30366672_G_T	313K>N	Substitution	Nonsynonymous coding	28%
MM04T	TPX2	TPX2, microtubule-associated, homolog (Xenopus laevis)	CCDS13190.1	chr20_30381800-30381800_G_T	553K>N	Substitution	Nonsynonymous coding	30%
MM04T	TPX2	TPX2, microtubule-associated, homolog (Xenopus laevis)	CCDS13190.1	chr20_30382201-30382201_T_C	NA	Substitution	Splice site acceptor	21%
MM04T	TRAC	T cell receptor alpha constant	ENST00000454915	chr14_22539456-22539456_T_G	106F>V	Substitution	Nonsynonymous coding	34%
MM04T	TRAFD1	TRAF-type zinc finger domain containing 1	CCDS9160.1	chr12_112589641-112589641_A_C	439Q>P	Substitution	Nonsynonymous coding	28%
MM04T	TRAIP	TRAF interacting protein	CCDS2806.1	chr3_49869449-49869449_C_A	313D>Y	Substitution	Nonsynonymous coding	23%
MM04T	TRAM1	translocation associated membrane protein 1	CCDS6207.1	chr8_71499188-71499188_C_A	230E>X	Substitution	Nonsense	30%
MM04T	TRANK1	tetratricopeptide repeat and ankyrin repeat containing 1	CCDS46789.1	chr3_36872731-36872731_C_A	2187E>D	Substitution	Nonsynonymous coding	35%
MM04T	TRANK1	tetratricopeptide repeat and ankyrin repeat containing 1	CCDS46789.1	chr3_36873463-36873463_C_A	1943E>D	Substitution	Nonsynonymous coding	22%
MM04T	TRANK1	tetratricopeptide repeat and ankyrin repeat containing 1	CCDS46789.1	chr3_36887775-36887775_C_T	1125D>N	Substitution	Nonsynonymous coding	32%
MM04T	TRANK1	tetratricopeptide repeat and ankyrin repeat containing 1	NM_014831	chr3_36915727-36915727_C_T	279R>Q	Substitution	Nonsynonymous coding	28%
MM04T	TRAPPC11	trafficking protein particle complex 11	CCDS34112.1	chr4_184598642-184598642_G_A	256E>K	Substitution	Nonsynonymous coding	27%
MM04T	TRAPPC8	trafficking protein particle complex 8	CCDS11901.1	chr18_29493468-29493468_T_C	212E>G	Substitution	Nonsynonymous coding	29%
MM04T	TRBV27	T cell receptor beta variable 27	ENST00000390399	chr7_142423246-142423246_C_A	11L>I	Substitution	Nonsynonymous coding	25%
MM04T	TRBV29-1	T cell receptor beta variable 29-1	ENST00000422143	chr7_142448725-142448725_C_T	107L>F	Substitution	Nonsynonymous coding	35%
MM04T	TRBV30	T cell receptor beta variable 30 (gene/pseudogene)	ENST00000417977	chr7_142510553-142510553_G_T	18S>Y	Substitution	Nonsynonymous coding	34%
MM04T	TRDN	triadin	NM_006073	chr6_123759249-123759249__T	NA	Insertion	Frameshift	37%
MM04T	TREML4	triggering receptor expressed on myeloid cells-like 4	CCDS34446.1	chr6_41197799-41197799_G_T	NA	Substitution	Splice site acceptor	16%
MM04T	TRHDE	thyrotropin-releasing hormone degrading enzyme	CCDS9004.1	chr12_73012702-73012702_G_A	740E>K	Substitution	Nonsynonymous coding	33%
MM04T	TRHDE	thyrotropin-releasing hormone degrading enzyme	CCDS9004.1	chr12_73046918-73046918_G_T	944W>L	Substitution	Nonsynonymous coding	31%
MM04T	TRIB3	tribbles homolog 3 (Drosophila)	CCDS12997.1	chr20_368826-368826_C_T	58R>C	Substitution	Nonsynonymous coding	33%
MM04T	TRIM14	tripartite motif containing 14	CCDS6734.1	chr9_100850257-100850257_G_A	275T>M	Substitution	Nonsynonymous coding	11%
MM04T	TRIM14	tripartite motif containing 14	CCDS6734.1	chr9_100872180-100872180_C_A	98E>D	Substitution	Nonsynonymous coding	34%
MM04T	TRIM17	tripartite motif containing 17	CCDS1571.1	chr1_228602453-228602453_C_A	107K>N	Substitution	Nonsynonymous coding	11%
MM04T	TRIM22	tripartite motif containing 22	CCDS41612.1	chr11_5730331-5730331_C_A	317S>Y	Substitution	Nonsynonymous coding	33%
MM04T	TRIM22	tripartite motif containing 22	CCDS41612.1	chr11_5730723-5730723_G_T	448D>Y	Substitution	Nonsynonymous coding	21%
MM04T	TRIM23	tripartite motif containing 23	CCDS3987.1	chr5_64905082-64905082_C_A	344K>N	Substitution	Nonsynonymous coding	35%

MM04T	TRIM3	tripartite motif containing 3	CCDS7764.1	chr11_6470404-6470404_C_T	697D>N	Substitution	Nonsynonymous coding	30%
MM04T	TRIM36	tripartite motif containing 36	CCDS4115.1	chr5_114499263-114499263_G_A	84R>W	Substitution	Nonsynonymous coding	38%
MM04T	TRIM36	tripartite motif containing 36	CCDS34211.1	chr5_114506903-114506903_C_T	27R>Q	Substitution	Nonsynonymous coding	37%
MM04T	TRIM5	tripartite motif containing 5	CCDS31393.1	chr11_5699637-5699637_C_T	181V>I	Substitution	Nonsynonymous coding	26%
MM04T	TRIM56	tripartite motif containing 56	CCDS43625.1	chr7_100732682-100732682_C_T	697R>X	Substitution	Nonsense	35%
MM04T	TRIM59	tripartite motif containing 59	CCDS3190.1	chr3_160156780-160156780_T_G	64E>D	Substitution	Nonsynonymous coding	30%
MM04T	TRIM6	tripartite motif containing 6	CCDS31388.1	chr11_5631371-5631371_C_A	NA	Substitution	Splice site acceptor	28%
MM04T	TRIML2	tripartite motif family-like 2	CCDS3850.1	chr4_189026051-189026051_C_A	25E>D	Substitution	Nonsynonymous coding	26%
MM04T	TRIO	trio Rho guanine nucleotide exchange factor	CCDS3883.1	chr5_14297367-14297367_G_A	455E>K	Substitution	Nonsynonymous coding	37%
MM04T	TRIP10	thyroid hormone receptor interactor 10	CCDS12172.1	chr19_6743760-6743760_A_C	185E>D	Substitution	Nonsynonymous coding	32%
MM04T	TRIP12	thyroid hormone receptor interactor 12	CCDS33391.1	chr2_230633371-230633371_G_T	1915L>I	Substitution	Nonsynonymous coding	29%
MM04T	TRIP12	thyroid hormone receptor interactor 12	CCDS33391.1	chr2_230654457-230654457_G_T	1447S>X	Substitution	Nonsense	20%
MM04T	TRIP4	thyroid hormone receptor interactor 4	CCDS10194.1	chr15_64686239-64686239_G_T	66E>X	Substitution	Nonsense	30%
MM04T	TRMT10A	tRNA methyltransferase 10 homolog A (S. cerevisiae)	CCDS3650.1	chr4_100479277-100479277_G_A	93R>X	Substitution	Nonsense	33%
MM04T	TRMT5	tRNA methyltransferase 5 homolog (S. cerevisiae)	CCDS32092.1	chr14_61446449-61446449_C_T	56R>K	Substitution	Nonsynonymous coding	33%
MM04T	TRNAU1AP	tRNA selenocysteine 1 associated protein 1	CCDS324.1	chr1_28887170-28887170_G_T	51E>X	Substitution	Nonsense	22%
MM04T	TRO	trophinin	CCDS43959.1	chrX_54955714-54955714_C_T	853L>F	Substitution	Nonsynonymous coding	31%
MM04T	TROAP	trophinin associated protein	CCDS8784.1	chr12_49722962-49722962_C_T	347R>W	Substitution	Nonsynonymous coding	19%
MM04T	TROVE2	TROVE domain family, member 2	CCDS1379.1	chr1_193053835-193053835_C_T	531R>X	Substitution	Nonsense	25%
MM04T	TRPA1	transient receptor potential cation channel, subfamily A, member 1	CCDS34908.1	chr8_72966005-72966005_G_A	543R>C	Substitution	Nonsynonymous coding	41%
MM04T	TRPC4	transient receptor potential cation channel, subfamily C, member 4	CCDS45037.1	chr13_38225565-38225565_C_T	639R>Q	Substitution	Nonsynonymous coding	29%
MM04T	TRPC5	transient receptor potential cation channel, subfamily C, member 5	CCDS14561.1	chrX_111019934-111019934_C_A	843K>N	Substitution	Nonsynonymous coding	33%
MM04T	TRPC5	transient receptor potential cation channel, subfamily C, member 5	CCDS14561.1	chrX_111095658-111095658_A_C	415I>M	Substitution	Nonsynonymous coding	24%
MM04T	TRPC7	transient receptor potential cation channel, subfamily C, member 7	NM_020389	chr5_135567128-135567128_G_A	652L>F	Substitution	Nonsynonymous coding	32%
MM04T	TRPC7	transient receptor potential cation channel, subfamily C, member 7	NM_020389	chr5_135610523-135610523_G_T	322F>L	Substitution	Nonsynonymous coding	19%
MM04T	TRPM1	transient receptor potential cation channel, subfamily M, member 1	CCDS10024.2	chr15_31319298-31319298_A_C	1084S>A	Substitution	Nonsynonymous coding	21%
MM04T	TRPM1	transient receptor potential cation channel, subfamily M, member 1	CCDS10024.2	chr15_31295173-31295173_C_A	1222E>X	Substitution	Nonsense	22%
MM04T	TRPM3	transient receptor potential cation channel, subfamily M, member 3	CCDS43835.1	chr9_73152136-73152136_G_A	1286S>L	Substitution	Nonsynonymous coding	29%
MM04T	TRPM3	transient receptor potential cation channel, subfamily M, member 3	CCDS43835.1	chr9_73235035-73235035_C_T	684D>N	Substitution	Nonsynonymous coding	44%
MM04T	TRPM3	transient receptor potential cation channel, subfamily M, member 3	ENST00000377101	chr9_73376427-73376427_G_T	301F>L	Substitution	Nonsynonymous coding	31%
MM04T	TRPM3	transient receptor potential cation channel, subfamily M, member 3	CCDS43835.1	chr9_73736193-73736193_C_A	26L>F	Substitution	Nonsynonymous coding	35%
MM04T	TRPM3	transient receptor potential cation channel, subfamily M, member 3	CCDS43835.1	chr9_73399028-73399028_C_A	381E>X	Substitution	Nonsense	28%

MM04T	TRPM4	transient receptor potential cation channel, subfamily M, member 4	CCDS33073.1	chr19_49714028-49714028_G_T	1130E>D	Substitution	Nonsynonymous coding	21%
MM04T	TRPM6	transient receptor potential cation channel, subfamily M, member 6	CCDS6647.1	chr9_77339653-77339653_C_A	1982R>I	Substitution	Nonsynonymous coding	35%
MM04T	TRPM6	transient receptor potential cation channel, subfamily M, member 6	CCDS6647.1	chr9_77400924-77400924_A_G	929W>R	Substitution	Nonsynonymous coding	27%
MM04T	TRPM6	transient receptor potential cation channel, subfamily M, member 6	CCDS6647.1	chr9_77400980-77400980_C_A	910W>L	Substitution	Nonsynonymous coding	22%
MM04T	TRPM6	transient receptor potential cation channel, subfamily M, member 6	CCDS6647.1	chr9_77411760-77411760_T_G	763K>T	Substitution	Nonsynonymous coding	29%
MM04T	TRPS1	trichorhinophalangeal syndrome I	CCDS6318.2	chr8_116631590-116631590_G_C	245Y>X	Substitution	Nonsense	32%
MM04T	TRPV4	transient receptor potential cation channel, subfamily V, member 4	CCDS9134.1	chr12_110231365-110231365_G_A	542S>F	Substitution	Nonsynonymous coding	28%
MM04T	TRPV4	transient receptor potential cation channel, subfamily V, member 4	CCDS9134.1	chr12_110236550-110236550_A_G	341F>L	Substitution	Nonsynonymous coding	21%
MM04T	TRPV4	transient receptor potential cation channel, subfamily V, member 4	CCDS9134.1	chr12_110252352-110252352_C_T	84D>N	Substitution	Nonsynonymous coding	35%
MM04T	TRPV5	transient receptor potential cation channel, subfamily V, member 5	CCDS5875.1	chr7_142605914-142605914_C_A	652K>N	Substitution	Nonsynonymous coding	36%
MM04T	TSC1	tuberous sclerosis 1	CCDS6956.1	chr9_135781467-135781467_G_A	500R>X	Substitution	Nonsense	29%
MM04T	TSC1	tuberous sclerosis 1	CCDS6956.1	chr9_135782124-135782124_C_A	478E>X	Substitution	Nonsense	23%
MM04T	TSC2	tuberous sclerosis 2	CCDS10458.1	chr16_2134305-2134305_G_A	1361R>Q	Substitution	Nonsynonymous coding	20%
MM04T	TSC22D4	TSC22 domain family, member 4	CCDS5695.1	chr7_100075102-100075102_G_A	187S>L	Substitution	Nonsynonymous coding	41%
MM04T	TSEN34	tRNA splicing endonuclease 34 homolog (S. cerevisiae)	CCDS42609.1	chr19_54695638-54695638_G_A	104A>T	Substitution	Nonsynonymous coding	25%
MM04T	TSEN34	tRNA splicing endonuclease 34 homolog (S. cerevisiae)	CCDS42609.1	chr19_54696138-54696138_G_A	220R>H	Substitution	Nonsynonymous coding	34%
MM04T	TSG101	tumor susceptibility gene 101	CCDS7842.1	chr11_18505518-18505518_G_A	248R>C	Substitution	Nonsynonymous coding	21%
MM04T	TSGA10	testis specific, 10	CCDS2037.1	chr2_99722030-99722030_C_A	114R>I	Substitution	Nonsynonymous coding	31%
MM04T	TSGA10IP	testis specific, 10 interacting protein	NM_152762	chr11_65715262-65715262_G_T	322E>D	Substitution	Nonsynonymous coding	18%
MM04T	TSGA13	testis specific, 13	CCDS5824.1	chr7_130357652-130357652__T	NA	Insertion	Frameshift	32%
MM04T	TSHZ3	teashirt zinc finger homeobox 3	CCDS12421.2	chr19_31770413-31770413_C_T	96E>K	Substitution	Nonsynonymous coding	30%
MM04T	TSPAN19	tetraspanin 19	CCDS44949.1	chr12_85423524-85423524_C_A	38D>Y	Substitution	Nonsynonymous coding	36%
MM04T	TSPAN32	tetraspanin 32	CCDS7733.1	chr11_2324141-2324141_G_A	45R>H	Substitution	Nonsynonymous coding	16%
MM04T	TSPAN8	tetraspanin 8	CCDS8999.1	chr12_71537954-71537954_G_A	34R>X	Substitution	Nonsense	19%
MM04T	TSPYL5	TSPY-like 5	CCDS34927.1	chr8_98289523-98289523_C_T	184E>K	Substitution	Nonsynonymous coding	30%
MM04T	TSPYL6	TSPY-like 6	NM_001003937	chr2_54482390-54482390_C_A	300R>I	Substitution	Nonsynonymous coding	27%
MM04T	TTBK2	tau tubulin kinase 2	CCDS42029.1	chr15_43103902-43103902_C_A	227R>I	Substitution	Nonsynonymous coding	23%
MM04T	TTC14	tetratricopeptide repeat domain 14	CCDS3237.1	chr3_180326556-180326556_C_A	453T>K	Substitution	Nonsynonymous coding	36%
MM04T	TTC16	tetratricopeptide repeat domain 16	CCDS6875.1	chr9_130479634-130479634_C_A	71C>X	Substitution	Nonsense	26%
MM04T	TTC21B	tetratricopeptide repeat domain 21B	CCDS33315.1	chr2_166758275-166758275_T_G	905K>T	Substitution	Nonsynonymous coding	29%
MM04T	TTC30A	tetratricopeptide repeat domain 30A	CCDS2276.1	chr2_178481692-178481692_T_C	580T>A	Substitution	Nonsynonymous coding	35%
MM04T	TTC30B	tetratricopeptide repeat domain 30B	CCDS42784.1	chr2_178416302-178416302_A_G	397V>A	Substitution	Nonsynonymous coding	28%

MM04T	TTC30B	tetratricopeptide repeat domain 30B	CCDS42784.1	chr2_178416698-178416698_C_A	265R>I	Substitution	Nonsynonymous coding	22%
MM04T	TTC34	tetratricopeptide repeat domain 34	ENST00000401095	chr1_2700243-2700243_C_T	200D>N	Substitution	Nonsynonymous coding	18%
MM04T	TTC34	tetratricopeptide repeat domain 34	ENST00000401095	chr1_2700297-2700297_G_T	182L>I	Substitution	Nonsynonymous coding	20%
MM04T	TTC38	tetratricopeptide repeat domain 38	CCDS43030.1	chr22_46671259-46671259_G_T	160Q>H	Substitution	Nonsynonymous coding	31%
MM04T	TTC39A	tetratricopeptide repeat domain 39A	CCDS44143.1	chr1_51753908-51753908_C_T	556R>Q	Substitution	Nonsynonymous coding	38%
MM04T	TTC39A	tetratricopeptide repeat domain 39A	CCDS44143.1	chr1_51756207-51756207_C_A	457E>X	Substitution	Nonsense	33%
MM04T	TTC6	tetratricopeptide repeat domain 6	ENST00000382320	chr14_38266134-38266134_G_T	51M>I	Substitution	Nonsynonymous coding	22%
MM04T	TTF1	transcription termination factor, RNA polymerase I	CCDS6948.1	chr9_135277775-135277775_A_C	145F>C	Substitution	Nonsynonymous coding	36%
MM04T	TTF1	transcription termination factor, RNA polymerase I	CCDS6948.1	chr9_135278196-135278196_A_C	5S>A	Substitution	Nonsynonymous coding	22%
MM04T	TTL	tubulin tyrosine ligase	CCDS2096.1	chr2_113260572-113260572_T_C	230V>A	Substitution	Nonsynonymous coding	23%
MM04T	TTLL11	tubulin tyrosine ligase-like family, member 11	CCDS48012.1	chr9_124801608-124801608_G_A	258H>Y	Substitution	Nonsynonymous coding	22%
MM04T	TTLL2	tubulin tyrosine ligase-like family, member 2	CCDS5301.1	chr6_167754076-167754076_G_T	230D>Y	Substitution	Nonsynonymous coding	15%
MM04T	TTLL5	tubulin tyrosine ligase-like family, member 5	CCDS32124.1	chr14_76243122-76243122_G_T	772K>N	Substitution	Nonsynonymous coding	18%
MM04T	TTLL7	tubulin tyrosine ligase-like family, member 7	CCDS690.2	chr1_84356079-84356079_C_T	765R>Q	Substitution	Nonsynonymous coding	23%
MM04T	TTN	titin	NM_133379	chr2_179614443-179614443__T	NA	Insertion	Frameshift	23%
MM04T	TTN	titin	ENST00000356127	chr2_179402490-179402490_T_G	30578K>N	Substitution	Nonsynonymous coding	40%
MM04T	TTN	titin	ENST00000356127	chr2_179414931-179414931_C_A	27975R>I	Substitution	Nonsynonymous coding	27%
MM04T	TTN	titin	ENST00000356127	chr2_179427758-179427758_C_A	25131G>C	Substitution	Nonsynonymous coding	24%
MM04T	TTN	titin	ENST00000356127	chr2_179436214-179436214_C_A	22312R>I	Substitution	Nonsynonymous coding	26%
MM04T	TTN	titin	ENST00000356127	chr2_179436760-179436760_G_T	22130P>H	Substitution	Nonsynonymous coding	26%
MM04T	TTN	titin	ENST00000356127	chr2_179457152-179457152_C_A	17290E>D	Substitution	Nonsynonymous coding	26%
MM04T	TTN	titin	ENST00000356127	chr2_179473500-179473500_T_G	14845K>T	Substitution	Nonsynonymous coding	50%
MM04T	TTN	titin	ENST00000356127	chr2_179474899-179474899_G_T	14550F>L	Substitution	Nonsynonymous coding	22%
MM04T	TTN	titin	ENST00000356127	chr2_179495762-179495762_C_A	12103E>D	Substitution	Nonsynonymous coding	23%
MM04T	TTN	titin	ENST00000356127	chr2_179498238-179498238_C_T	11715R>H	Substitution	Nonsynonymous coding	26%
MM04T	TTN	titin	ENST00000356127	chr2_179500275-179500275_C_A	11358D>Y	Substitution	Nonsynonymous coding	21%
MM04T	TTN	titin	ENST00000356127	chr2_179558686-179558686_G_T	9248F>L	Substitution	Nonsynonymous coding	24%
MM04T	TTN	titin	ENST00000356127	chr2_179584926-179584926_G_A	6571R>W	Substitution	Nonsynonymous coding	28%
MM04T	TTN	titin	ENST00000356127	chr2_179587827-179587827_C_T	6059E>K	Substitution	Nonsynonymous coding	32%
MM04T	TTN	titin	ENST00000356127	chr2_179593664-179593664_C_A	5123K>N	Substitution	Nonsynonymous coding	36%
MM04T	TTN	titin	ENST00000356127	chr2_179603052-179603052_C_T	3466E>K	Substitution	Nonsynonymous coding	37%
MM04T	TTN	titin	ENST00000342175	chr2_179603970-179603970_C_T	4493D>N	Substitution	Nonsynonymous coding	30%

MM04T	TTN	titin	ENST00000342175	chr2_179606495-179606495_A_C	3651F>C	Substitution	Nonsynonymous coding	10%
MM04T	TTN	titin	NM_133379	chr2_179610805-179610805_C_T	5441R>Q	Substitution	Nonsynonymous coding	38%
MM04T	TTN	titin	NM_133379	chr2_179612897-179612897_C_T	4744E>K	Substitution	Nonsynonymous coding	32%
MM04T	TTN	titin	NM_133379	chr2_179613073-179613073_C_A	4685R>I	Substitution	Nonsynonymous coding	23%
MM04T	TTN	titin	NM_133379	chr2_179613394-179613394_T_G	4578Q>P	Substitution	Nonsynonymous coding	27%
MM04T	TTN	titin	NM_133379	chr2_179638266-179638266_C_T	2506R>Q	Substitution	Nonsynonymous coding	39%
MM04T	TTN	titin	NM_133379	chr2_179639138-179639138_C_T	2285E>K	Substitution	Nonsynonymous coding	24%
MM04T	TTN	titin	NM_133379	chr2_179641543-179641543_C_T	1683R>Q	Substitution	Nonsynonymous coding	35%
MM04T	TTN	titin	ENST00000356127	chr2_179476482-179476482_T_G	NA	Substitution	Splice site donor	39%
MM04T	TTN	titin	ENST00000356127	chr2_179416861-179416861_C_A	27686E>X	Substitution	Nonsense	34%
MM04T	TTN	titin	ENST00000356127	chr2_179432768-179432768_C_A	23461E>X	Substitution	Nonsense	17%
MM04T	TTN	titin	ENST00000356127	chr2_179579178-179579178_C_A	7531E>X	Substitution	Nonsense	24%
MM04T	TTN	titin	NM_133379	chr2_179613626-179613626_C_A	4501E>X	Substitution	Nonsense	18%
MM04T	TTN	titin	NM_133379	chr2_179615321-179615321_G_A	3936R>X	Substitution	Nonsense	21%
MM04T	TTPA	tocopherol (alpha) transfer protein	CCDS6178.1	chr8_63985509-63985509_G_T	115L>I	Substitution	Nonsynonymous coding	27%
MM04T	TTYH2	tweety homolog 2 (Drosophila)	CCDS32717.1	chr17_72249971-72249971_C_T	508T>M	Substitution	Nonsynonymous coding	15%
MM04T	TUBAL3	tubulin, alpha-like 3	CCDS7066.2	chr10_5442918-5442918_T_G	46N>H	Substitution	Nonsynonymous coding	27%
MM04T	TUBAL3	tubulin, alpha-like 3	CCDS7066.2	chr10_5435503-5435503_C_A	440E>X	Substitution	Nonsense	21%
MM04T	TUBGCP2	tubulin, gamma complex associated protein 2	CCDS7676.1	chr10_135106730-135106730_C_A	279E>D	Substitution	Nonsynonymous coding	30%
MM04T	TUBGCP5	tubulin, gamma complex associated protein 5	CCDS10008.1	chr15_22867481-22867481_C_A	853P>T	Substitution	Nonsynonymous coding	27%
MM04T	TUBGCP6	tubulin, gamma complex associated protein 6	CCDS14087.1	chr22_50682689-50682689_C_T	67R>K	Substitution	Nonsynonymous coding	28%
MM04T	TUFM	Tu translation elongation factor, mitochondrial	CCDS10642.1	chr16_28856770-28856770_C_A	93K>N	Substitution	Nonsynonymous coding	40%
MM04T	TUSC3	tumor suppressor candidate 3	CCDS5994.1	chr8_15517143-15517143_G_T	185R>I	Substitution	Nonsynonymous coding	27%
MM04T	TUSC3	tumor suppressor candidate 3	CCDS5994.1	chr8_15480694-15480694_C_T	82R>X	Substitution	Nonsense	24%
MM04T	TXNRD1	thioredoxin reductase 1	ENST00000429002	chr12_104742156-104742156_A_C	640K>T	Substitution	Nonsynonymous coding	26%
MM04T	TXNRD3	thioredoxin reductase 3	ENST00000450785	chr3_126341346-126341346_T_G	555K>T	Substitution	Nonsynonymous coding	28%
MM04T	TYMS	thymidylate synthetase	CCDS11821.1	chr18_672903-672903_G_A	283R>Q	Substitution	Nonsynonymous coding	39%
MM04T	TYR	tyrosinase	CCDS8284.1	chr11_88911770-88911770_C_T	217R>W	Substitution	Nonsynonymous coding	19%
MM04T	TYW1B	tRNA-yW synthesizing protein 1 homolog B (S. cerevisiae)	ENST00000438904	chr7_72286030-72286030_T_C	13T>A	Substitution	Nonsynonymous coding	15%
MM04T	U2AF1L4	U2 small nuclear RNA auxiliary factor 1-like 4	CCDS42551.1	chr19_36234923-36234923_A_G	119Y>H	Substitution	Nonsynonymous coding	21%
MM04T	U2SURP	U2 snRNP-associated SURP domain containing	CCDS46928.1	chr3_142761969-142761969_G_T	799E>X	Substitution	Nonsense	45%
MM04T	UACA	uveal autoantigen with coiled-coil domains and ankyrin repeats	CCDS10235.1	chr15_70960151-70960151_C_A	958E>X	Substitution	Nonsense	42%

MM04T	UBAP2	ubiquitin associated protein 2	CCDS6547.1	chr9_33989092-33989092_C_A	107K>N	Substitution	Nonsynonymous coding	39%
MM04T	UBASH3A	ubiquitin associated and SH3 domain containing A	CCDS13687.1	chr21_43833289-43833289_G_A	171E>K	Substitution	Nonsynonymous coding	26%
MM04T	UBE2A	ubiquitin-conjugating enzyme E2A	CCDS14580.1	chrX_118717207-118717207_C_T	150R>C	Substitution	Nonsynonymous coding	43%
MM04T	UBE2D2	ubiquitin-conjugating enzyme E2D 2	CCDS43369.1	chr5_138994337-138994337_A_C	59D>A	Substitution	Nonsynonymous coding	29%
MM04T	UBE2E2	ubiquitin-conjugating enzyme E2E 2	CCDS2637.1	chr3_23541203-23541203_C_A	111S>X	Substitution	Nonsense	27%
MM04T	UBE2O	ubiquitin-conjugating enzyme E2O	CCDS32742.1	chr17_74394407-74394407_T_C	652R>G	Substitution	Nonsynonymous coding	29%
MM04T	UBE2Q1	ubiquitin-conjugating enzyme E2Q family member 1	CCDS1069.1	chr1_154527246-154527246_C_T	185V>M	Substitution	Nonsynonymous coding	28%
MM04T	UBE2U	ubiquitin-conjugating enzyme E2U (putative)	CCDS627.1	chr1_64698264-64698264_T_G	176F>C	Substitution	Nonsynonymous coding	33%
MM04T	UBE3A	ubiquitin protein ligase E3A	CCDS45192.1	chr15_25616927-25616927_C_A	135E>X	Substitution	Nonsense	29%
MM04T	UBE3B	ubiquitin protein ligase E3B	CCDS9129.1	chr12_109928889-109928889_C_A	224L>I	Substitution	Nonsynonymous coding	21%
MM04T	UBE3D	ubiquitin protein ligase E3D	CCDS34491.1	chr6_83763906-83763906_A_C	109F>C	Substitution	Nonsynonymous coding	23%
MM04T	UBIAD1	UbiA prenyltransferase domain containing 1	CCDS129.1	chr1_11346073-11346073_G_A	301R>Q	Substitution	Nonsynonymous coding	38%
MM04T	UBQLN3	ubiquilin 3	CCDS7758.1	chr11_5529898-5529898_C_A	297E>D	Substitution	Nonsynonymous coding	22%
MM04T	UBQLNL	ubiquilin-like	CCDS31385.1	chr11_5537652-5537652_C_T	7R>Q	Substitution	Nonsynonymous coding	28%
MM04T	UBR1	ubiquitin protein ligase E3 component n-recognin 1	CCDS10091.1	chr15_43348619-43348619_C_A	402E>X	Substitution	Nonsense	45%
MM04T	UBR3	ubiquitin protein ligase E3 component n-recognin 3 (putative)	CCDS2238.2	chr2_170885888-170885888_C_T	1496R>W	Substitution	Nonsynonymous coding	36%
MM04T	UBR4	ubiquitin protein ligase E3 component n-recognin 4	CCDS189.1	chr1_19493699-19493699_T_G	1309Q>P	Substitution	Nonsynonymous coding	24%
MM04T	UBR5	ubiquitin protein ligase E3 component n-recognin 5	CCDS34933.1	chr8_103305990-103305990_C_T	1478V>I	Substitution	Nonsynonymous coding	28%
MM04T	UBR5	ubiquitin protein ligase E3 component n-recognin 5	CCDS34933.1	chr8_103326139-103326139_G_A	634R>X	Substitution	Nonsense	33%
MM04T	UBR7	ubiquitin protein ligase E3 component n-recognin 7 (putative)	CCDS9909.1	chr14_93693318-93693318_C_A	405F>L	Substitution	Nonsynonymous coding	25%
MM04T	UBTF	upstream binding transcription factor, RNA polymerase I	CCDS11480.1	chr17_42290650-42290650_T_A	159M>L	Substitution	Nonsynonymous coding	21%
MM04T	UBXN2A	UBX domain protein 2A	CCDS1704.1	chr2_24207608-24207608_C_A	164A>D	Substitution	Nonsynonymous coding	22%
MM04T	UBXN7	UBX domain protein 7	CCDS43191.1	chr3_196083647-196083647_C_T	460R>Q	Substitution	Nonsynonymous coding	33%
MM04T	UBXN7	UBX domain protein 7	CCDS43191.1	chr3_196118794-196118794_G_A	120R>W	Substitution	Nonsynonymous coding	33%
MM04T	UBXN7	UBX domain protein 7	CCDS43191.1	chr3_196129863-196129863_T_G	83Q>H	Substitution	Nonsynonymous coding	26%
MM04T	UBXN7	UBX domain protein 7	CCDS43191.1	chr3_196089444-196089444_C_A	317E>X	Substitution	Nonsense	30%
MM04T	UBXN8	UBX domain protein 8	ENST00000265616	chr8_30608999-30608999_C_T	59L>F	Substitution	Nonsynonymous coding	18%
MM04T	UCHL5	ubiquitin carboxyl-terminal hydrolase L5	CCDS1378.1	chr1_192992054-192992054_A_G	NA	Substitution	Splice site donor	38%
MM04T	UCN2	urocortin 2	CCDS2772.1	chr3_48600436-48600436_C_T	41R>Q	Substitution	Nonsynonymous coding	29%
MM04T	UFSP2	UFM1-specific peptidase 2	CCDS3842.1	chr4_186339638-186339638_T_G	97K>T	Substitution	Nonsynonymous coding	40%
MM04T	UGDH	UDP-glucose 6-dehydrogenase	CCDS3455.1	chr4_39515753-39515753__A	NA	Insertion	Frameshift	24%
MM04T	UGDH	UDP-glucose 6-dehydrogenase	CCDS3455.1	chr4_39511412-39511412_C_A	260R>I	Substitution	Nonsynonymous coding	40%

MM04T	UGGT1	UDP-glucose glycoprotein glucosyltransferase 1	CCDS2154.1	chr2_128865608-128865608_G_A	125R>H	Substitution	Nonsynonymous coding	34%
MM04T	UGGT1	UDP-glucose glycoprotein glucosyltransferase 1	CCDS2154.1	chr2_128878857-128878857_T_G	353F>C	Substitution	Nonsynonymous coding	10%
MM04T	UGGT2	UDP-glucose glycoprotein glucosyltransferase 2	CCDS9480.1	chr13_96513048-96513048_A_C	1245F>C	Substitution	Nonsynonymous coding	30%
MM04T	UGT1A6	UDP glucuronosyltransferase 1 family, polypeptide A6	CCDS33402.1	chr2_234526691-234526691_C_A	113S>Y	Substitution	Nonsynonymous coding	21%
MM04T	UGT1A6	UDP glucuronosyltransferase 1 family, polypeptide A6	CCDS2507.1	chr2_234601822-234601822_G_A	58V>M	Substitution	Nonsynonymous coding	16%
MM04T	UGT2A1	UDP glucuronosyltransferase 2 family, polypeptide A1, complex locus	CCDS3529.1	chr4_70512737-70512737_C_A	209R>I	Substitution	Nonsynonymous coding	23%
MM04T	UGT2B15	UDP glucuronosyltransferase 2 family, polypeptide B15	CCDS3524.1	chr4_69512834-69512834_C_A	527K>N	Substitution	Nonsynonymous coding	19%
MM04T	UHMK1	U2AF homology motif (UHM) kinase 1	CCDS1239.1	chr1_162470849-162470849_C_A	236F>L	Substitution	Nonsynonymous coding	26%
MM04T	ULK2	unc-51-like kinase 2 (C. elegans)	CCDS11213.1	chr17_19713705-19713705_G_T	381F>L	Substitution	Nonsynonymous coding	26%
MM04T	ULK4	unc-51-like kinase 4 (C. elegans)	CCDS43071.1	chr3_41973499-41973499_C_A	NA	Substitution	Splice site acceptor	33%
MM04T	UMODL1	uromodulin-like 1	CCDS42935.1	chr21_43524033-43524033_G_T	452R>I	Substitution	Nonsynonymous coding	19%
MM04T	UNC13B	unc-13 homolog B (C. elegans)	CCDS6579.1	chr9_35376184-35376184_G_A	510E>K	Substitution	Nonsynonymous coding	28%
MM04T	UNC13C	unc-13 homolog C (C. elegans)	CCDS45264.1	chr15_54308000-54308000_G_T	967R>I	Substitution	Nonsynonymous coding	32%
MM04T	UNC13C	unc-13 homolog C (C. elegans)	CCDS45264.1	chr15_54527286-54527286_T_G	1044L>V	Substitution	Nonsynonymous coding	32%
MM04T	UNC13C	unc-13 homolog C (C. elegans)	CCDS45264.1	chr15_54860065-54860065_C_A	2009S>Y	Substitution	Nonsynonymous coding	17%
MM04T	UNC13C	unc-13 homolog C (C. elegans)	CCDS45264.1	chr15_54305200-54305200_G_T	34E>X	Substitution	Nonsense	24%
MM04T	UNC5A	unc-5 homolog A (C. elegans)	CCDS34299.1	chr5_176295233-176295233_C_T	132S>L	Substitution	Nonsynonymous coding	21%
MM04T	UNC5D	unc-5 homolog D (C. elegans)	CCDS6093.2	chr8_35542256-35542256_C_T	303S>F	Substitution	Nonsynonymous coding	30%
MM04T	UNC5D	unc-5 homolog D (C. elegans)	CCDS6093.2	chr8_35583878-35583878_G_T	504K>N	Substitution	Nonsynonymous coding	29%
MM04T	UNC79	unc-79 homolog (C. elegans)	CCDS9911.2	chr14_94088209-94088209_G_A	1367D>N	Substitution	Nonsynonymous coding	37%
MM04T	UNC80	unc-80 homolog (C. elegans)	CCDS46504.1	chr2_210690774-210690774_G_T	825E>D	Substitution	Nonsynonymous coding	38%
MM04T	UNC80	unc-80 homolog (C. elegans)	CCDS46504.1	chr2_210761087-210761087_A_C	1445S>R	Substitution	Nonsynonymous coding	31%
MM04T	UNC80	unc-80 homolog (C. elegans)	CCDS46504.1	chr2_210834512-210834512_C_A	2610P>H	Substitution	Nonsynonymous coding	23%
MM04T	UPF2	UPF2 regulator of nonsense transcripts homolog (yeast)	CCDS7086.1	chr10_11994078-11994078_G_T	1007F>L	Substitution	Nonsynonymous coding	23%
MM04T	UPF2	UPF2 regulator of nonsense transcripts homolog (yeast)	CCDS7086.1	chr10_12071315-12071315_C_A	192D>Y	Substitution	Nonsynonymous coding	22%
MM04T	UPP2	uridine phosphorylase 2	CCDS46435.1	chr2_158977970-158977970_C_A	225F>L	Substitution	Nonsynonymous coding	23%
MM04T	UQCRH	ubiquinol-cytochrome c reductase hinge protein	CCDS30704.1	chr1_46769462-46769462_G_T	8K>N	Substitution	Nonsynonymous coding	31%
MM04T	URB1	URB1 ribosome biogenesis 1 homolog (S. cerevisiae)	CCDS46645.1	chr21_33719513-33719514_TC_	NA	Deletion	Frameshift	34%
MM04T	URB2	URB2 ribosome biogenesis 2 homolog (S. cerevisiae)	CCDS31052.1	chr1_229768082-229768082_G_T	65D>Y	Substitution	Nonsynonymous coding	39%
MM04T	URB2	URB2 ribosome biogenesis 2 homolog (S. cerevisiae)	CCDS31052.1	chr1_229772495-229772495_G_A	712R>K	Substitution	Nonsynonymous coding	26%
MM04T	URGCP	upregulator of cell proliferation	CCDS47578.1	chr7_43917113-43917113_G_A	650A>V	Substitution	Nonsynonymous coding	27%
MM04T	URGCP	upregulator of cell proliferation	CCDS47578.1	chr7_43921560-43921560_G_T	NA	Substitution	Splice site acceptor	24%

MM04T	USH2A	Usher syndrome 2A (autosomal recessive, mild)	CCDS31025.1	chr1_215901469-215901469_G_T	3990S>Y	Substitution	Nonsynonymous coding	28%
MM04T	USH2A	Usher syndrome 2A (autosomal recessive, mild)	CCDS31025.1	chr1_215901695-215901695_A_C	3915L>V	Substitution	Nonsynonymous coding	48%
MM04T	USH2A	Usher syndrome 2A (autosomal recessive, mild)	CCDS31025.1	chr1_215914813-215914813_G_T	3872S>Y	Substitution	Nonsynonymous coding	14%
MM04T	USH2A	Usher syndrome 2A (autosomal recessive, mild)	CCDS31025.1	chr1_215953252-215953252_C_A	3624Q>H	Substitution	Nonsynonymous coding	20%
MM04T	USH2A	Usher syndrome 2A (autosomal recessive, mild)	CCDS31025.1	chr1_215987234-215987234_C_T	3195G>R	Substitution	Nonsynonymous coding	36%
MM04T	USH2A	Usher syndrome 2A (autosomal recessive, mild)	CCDS31025.1	chr1_216074101-216074101_G_T	2483L>I	Substitution	Nonsynonymous coding	21%
MM04T	USH2A	Usher syndrome 2A (autosomal recessive, mild)	CCDS31025.1	chr1_216144031-216144031_G_T	2298P>H	Substitution	Nonsynonymous coding	24%
MM04T	USH2A	Usher syndrome 2A (autosomal recessive, mild)	CCDS31025.1	chr1_216144105-216144105_A_C	2273S>R	Substitution	Nonsynonymous coding	20%
MM04T	USH2A	Usher syndrome 2A (autosomal recessive, mild)	CCDS31025.1	chr1_216258043-216258043_C_A	1722A>S	Substitution	Nonsynonymous coding	11%
MM04T	USH2A	Usher syndrome 2A (autosomal recessive, mild)	CCDS31025.1	chr1_216424349-216424349_G_T	688P>H	Substitution	Nonsynonymous coding	18%
MM04T	USH2A	Usher syndrome 2A (autosomal recessive, mild)	CCDS31025.1	chr1_216595264-216595264_G_T	139P>T	Substitution	Nonsynonymous coding	30%
MM04T	USH2A	Usher syndrome 2A (autosomal recessive, mild)	CCDS31025.1	chr1_215960054-215960054_C_A	3449E>X	Substitution	Nonsense	23%
MM04T	USP2	ubiquitin specific peptidase 2	CCDS8423.1	chr11_119234579-119234579_G_A	43L>F	Substitution	Nonsynonymous coding	11%
MM04T	USP21	ubiquitin specific peptidase 21	CCDS30920.1	chr1_161132774-161132774_G_A	320R>Q	Substitution	Nonsynonymous coding	33%
MM04T	USP21	ubiquitin specific peptidase 21	CCDS30920.1	chr1_161134633-161134633_C_T	465R>X	Substitution	Nonsense	36%
MM04T	USP24	ubiquitin specific peptidase 24	CCDS44154.1	chr1_55562760-55562760_C_T	1774R>Q	Substitution	Nonsynonymous coding	33%
MM04T	USP24	ubiquitin specific peptidase 24	CCDS44154.1	chr1_55563691-55563691_A_C	1680I>M	Substitution	Nonsynonymous coding	25%
MM04T	USP24	ubiquitin specific peptidase 24	CCDS44154.1	chr1_55603315-55603315_G_T	865S>Y	Substitution	Nonsynonymous coding	25%
MM04T	USP25	ubiquitin specific peptidase 25	CCDS33515.1	chr21_17202880-17202880_G_A	575R>Q	Substitution	Nonsynonymous coding	37%
MM04T	USP26	ubiquitin specific peptidase 26	CCDS14635.1	chrX_132160312-132160312_C_T	646R>Q	Substitution	Nonsynonymous coding	19%
MM04T	USP26	ubiquitin specific peptidase 26	CCDS14635.1	chrX_132161539-132161539_G_A	237S>F	Substitution	Nonsynonymous coding	33%
MM04T	USP29	ubiquitin specific peptidase 29	CCDS33124.1	chr19_57641850-57641850_C_A	603L>I	Substitution	Nonsynonymous coding	22%
MM04T	USP31	ubiquitin specific peptidase 31	ENST00000381162	chr16_23085253-23085253_C_A	12E>X	Substitution	Nonsense	22%
MM04T	USP33	ubiquitin specific peptidase 33	CCDS678.1	chr1_78177475-78177475_T_G	819K>T	Substitution	Nonsynonymous coding	21%
MM04T	USP34	ubiquitin specific peptidase 34	CCDS42686.1	chr2_61522395-61522395_G_T	1429L>I	Substitution	Nonsynonymous coding	34%
MM04T	USP34	ubiquitin specific peptidase 34	CCDS42686.1	chr2_61561087-61561087_G_A	922R>C	Substitution	Nonsynonymous coding	22%
MM04T	USP34	ubiquitin specific peptidase 34	CCDS42686.1	chr2_61566531-61566531_C_T	900R>Q	Substitution	Nonsynonymous coding	33%
MM04T	USP34	ubiquitin specific peptidase 34	CCDS42686.1	chr2_61607492-61607492_A_C	276L>V	Substitution	Nonsynonymous coding	25%
MM04T	USP39	ubiquitin specific peptidase 39	CCDS33234.1	chr2_85857985-85857985_A_C	289N>H	Substitution	Nonsynonymous coding	24%
MM04T	USP39	ubiquitin specific peptidase 39	CCDS33234.1	chr2_85866511-85866511_G_T	427E>D	Substitution	Nonsynonymous coding	35%
MM04T	USP40	ubiquitin specific peptidase 40	CCDS46547.1	chr2_234396895-234396895_C_T	1074E>K	Substitution	Nonsynonymous coding	27%
MM04T	USP40	ubiquitin specific peptidase 40	CCDS46547.1	chr2_234450938-234450938_C_A	358M>I	Substitution	Nonsynonymous coding	21%

MM04T	USP45	ubiquitin specific peptidase 45	CCDS34501.1	chr6_99885135-99885135_C_A	767K>N	Substitution	Nonsynonymous coding	29%
MM04T	USP47	ubiquitin specific peptidase 47	CCDS41619.1	chr11_11924385-11924385_C_A	171S>R	Substitution	Nonsynonymous coding	23%
MM04T	USP5	ubiquitin specific peptidase 5 (isopeptidase T)	CCDS41743.1	chr12_6971715-6971715_G_T	585K>N	Substitution	Nonsynonymous coding	19%
MM04T	USP50	ubiquitin specific peptidase 50	NM_203494	chr15_50833415-50833415_C_A	169R>I	Substitution	Nonsynonymous coding	21%
MM04T	USP7	ubiquitin specific peptidase 7 (herpes virus-associated)	CCDS32385.1	chr16_9009359-9009359_C_A	310K>N	Substitution	Nonsynonymous coding	16%
MM04T	USP8	ubiquitin specific peptidase 8	CCDS10137.1	chr15_50776523-50776523_T_G	619F>V	Substitution	Nonsynonymous coding	27%
MM04T	USP8	ubiquitin specific peptidase 8	CCDS10137.1	chr15_50786365-50786365_A_G	849D>G	Substitution	Nonsynonymous coding	18%
MM04T	USP8	ubiquitin specific peptidase 8	CCDS10137.1	chr15_50791241-50791241_T_G	1105F>V	Substitution	Nonsynonymous coding	38%
MM04T	USP9X	ubiquitin specific peptidase 9, X-linked	CCDS43930.1	chrX_41055914-41055914_G_A	1386A>T	Substitution	Nonsynonymous coding	39%
MM04T	USP9X	ubiquitin specific peptidase 9, X-linked	CCDS43930.1	chrX_41089790-41089790_T_C	2506S>P	Substitution	Nonsynonymous coding	23%
MM04T	USP9X	ubiquitin specific peptidase 9, X-linked	CCDS43930.1	chrX_41048729-41048729_G_A	NA	Substitution	Splice site donor	34%
MM04T	USPL1	ubiquitin specific peptidase like 1	CCDS9336.1	chr13_31216849-31216849_C_A	356S>Y	Substitution	Nonsynonymous coding	20%
MM04T	UTP20	UTP20, small subunit (SSU) processome component, homolog (yeast)	CCDS9081.1	chr12_101674925-101674925_T_C	26V>A	Substitution	Nonsynonymous coding	12%
MM04T	UTP20	UTP20, small subunit (SSU) processome component, homolog (yeast)	CCDS9081.1	chr12_101736316-101736316_C_A	1398F>L	Substitution	Nonsynonymous coding	15%
MM04T	UTP20	UTP20, small subunit (SSU) processome component, homolog (yeast)	CCDS9081.1	chr12_101750747-101750747_G_T	1860E>X	Substitution	Nonsense	35%
MM04T	UTP6	UTP6, small subunit (SSU) processome component, homolog (yeast)	CCDS11269.1	chr17_30226727-30226727_C_T	39D>N	Substitution	Nonsynonymous coding	35%
MM04T	UTRN	utrophin	CCDS34547.1	chr6_144780323-144780323_A_C	847N>T	Substitution	Nonsynonymous coding	24%
MM04T	UTRN	utrophin	CCDS34547.1	chr6_145124220-145124220_C_A	3098F>L	Substitution	Nonsynonymous coding	31%
MM04T	UTRN	utrophin	CCDS34547.1	chr6_144760023-144760023_G_T	462E>X	Substitution	Nonsense	19%
MM04T	UVSSA	UV-stimulated scaffold protein A	CCDS33938.1	chr4_1343584-1343584_T_C	124F>S	Substitution	Nonsynonymous coding	40%
MM04T	VAPB	VAMP (vesicle-associated membrane protein)-associated protein B and C	CCDS33498.1	chr20_57009697-57009697_G_T	84S>I	Substitution	Nonsynonymous coding	21%
MM04T	VASH1	vasohibin 1	CCDS9851.1	chr14_77229344-77229344_C_A	60F>L	Substitution	Nonsynonymous coding	23%
MM04T	VAV1	vav 1 guanine nucleotide exchange factor	CCDS12174.1	chr19_6828670-6828670_A_G	377N>S	Substitution	Nonsynonymous coding	19%
MM04T	VAV3	vav 3 guanine nucleotide exchange factor	CCDS785.1	chr1_108145740-108145740_A_C	687I>M	Substitution	Nonsynonymous coding	14%
MM04T	VAV3	vav 3 guanine nucleotide exchange factor	CCDS785.1	chr1_108507486-108507486_C_A	2E>D	Substitution	Nonsynonymous coding	24%
MM04T	VCAM1	vascular cell adhesion molecule 1	CCDS773.1	chr1_101188822-101188822_G_A	196R>Q	Substitution	Nonsynonymous coding	37%
MM04T	VCAN	versican	CCDS4060.1	chr5_82815877-82815877_G_T	584K>N	Substitution	Nonsynonymous coding	32%
MM04T	VCAN	versican	CCDS4060.1	chr5_82816470-82816470_A_C	782N>T	Substitution	Nonsynonymous coding	40%
MM04T	VCAN	versican	CCDS4060.1	chr5_82834562-82834562_G_A	1914E>K	Substitution	Nonsynonymous coding	31%
MM04T	VCAN	versican	CCDS4060.1	chr5_82815233-82815233_G_T	370E>X	Substitution	Nonsense	31%
MM04T	VCAN	versican	CCDS4060.1	chr5_82817699-82817699_G_T	1192E>X	Substitution	Nonsense	23%
MM04T	VCL	vinculin	CCDS7341.1	chr10_75830502-75830502_G_A	105R>Q	Substitution	Nonsynonymous coding	32%

MM04T	VEPH1	ventricular zone expressed PH domain-containing 1	CCDS3179.1	chr3_157131731-157131731_C_A	282R>I	Substitution	Nonsynonymous coding	30%
MM04T	VGLL2	vestigial like 2 (Drosophila)	CCDS5115.1	chr6_117589429-117589429_T_C	56S>P	Substitution	Nonsynonymous coding	38%
MM04T	VIM	vimentin	CCDS7120.1	chr10_17271679-17271679_C_A	86F>L	Substitution	Nonsynonymous coding	22%
MM04T	VIPR1	vasoactive intestinal peptide receptor 1	CCDS2698.1	chr3_42569614-42569614_C_T	212S>L	Substitution	Nonsynonymous coding	31%
MM04T	VN1R2	vomeronasal 1 receptor 2	CCDS12862.1	chr19_53762779-53762779_G_A	384R>K	Substitution	Nonsynonymous coding	32%
MM04T	VNN1	vanin 1	CCDS5159.1	chr6_133013693-133013693_C_A	286R>I	Substitution	Nonsynonymous coding	25%
MM04T	VNN2	vanin 2	CCDS5161.1	chr6_133078886-133078886_G_T	46S>Y	Substitution	Nonsynonymous coding	26%
MM04T	VPS13A	vacuolar protein sorting 13 homolog A (S. cerevisiae)	CCDS6655.1	chr9_79834915-79834915_G_A	267R>Q	Substitution	Nonsynonymous coding	24%
MM04T	VPS13A	vacuolar protein sorting 13 homolog A (S. cerevisiae)	CCDS6655.1	chr9_79836192-79836192_A_C	361K>Q	Substitution	Nonsynonymous coding	15%
MM04T	VPS13A	vacuolar protein sorting 13 homolog A (S. cerevisiae)	CCDS6655.1	chr9_79836210-79836210_G_T	367E>X	Substitution	Nonsense	34%
MM04T	VPS13B	vacuolar protein sorting 13 homolog B (yeast)	CCDS6280.1	chr8_100454826-100454826_G_T	1136E>D	Substitution	Nonsynonymous coding	36%
MM04T	VPS13B	vacuolar protein sorting 13 homolog B (yeast)	CCDS6280.1	chr8_100829870-100829870_C_T	2759R>W	Substitution	Nonsynonymous coding	33%
MM04T	VPS13B	vacuolar protein sorting 13 homolog B (yeast)	CCDS6280.1	chr8_100832183-100832183_C_T	2968R>X	Substitution	Nonsense	17%
MM04T	VPS13C	vacuolar protein sorting 13 homolog C (S. cerevisiae)	CCDS32257.1	chr15_62266517-62266517_C_A	836Q>H	Substitution	Nonsynonymous coding	19%
MM04T	VPS13C	vacuolar protein sorting 13 homolog C (S. cerevisiae)	CCDS32257.1	chr15_62228795-62228795_C_T	NA	Substitution	Splice site donor	32%
MM04T	VPS13C	vacuolar protein sorting 13 homolog C (S. cerevisiae)	CCDS32257.1	chr15_62254995-62254995_C_A	1130E>X	Substitution	Nonsense	21%
MM04T	VPS13D	vacuolar protein sorting 13 homolog D (S. cerevisiae)	CCDS30588.1	chr1_12313784-12313784_G_T	190Q>H	Substitution	Nonsynonymous coding	32%
MM04T	VPS35	vacuolar protein sorting 35 homolog (S. cerevisiae)	CCDS10721.1	chr16_46702948-46702948_C_T	514R>Q	Substitution	Nonsynonymous coding	29%
MM04T	VPS35	vacuolar protein sorting 35 homolog (S. cerevisiae)	CCDS10721.1	chr16_46708575-46708575_A_	NA	Deletion	Splice site acceptor	13%
MM04T	VPS39	vacuolar protein sorting 39 homolog (S. cerevisiae)	CCDS10083.1	chr15_42483712-42483712_G_T	62F>L	Substitution	Nonsynonymous coding	35%
MM04T	VPS41	vacuolar protein sorting 41 homolog (S. cerevisiae)	CCDS5457.1	chr7_38813779-38813779_C_A	316E>D	Substitution	Nonsynonymous coding	17%
MM04T	VPS41	vacuolar protein sorting 41 homolog (S. cerevisiae)	CCDS5457.1	chr7_38829415-38829415_C_T	257R>Q	Substitution	Nonsynonymous coding	15%
MM04T	VPS4A	vacuolar protein sorting 4 homolog A (S. cerevisiae)	CCDS45517.1	chr16_69354656-69354656_T_G	279S>A	Substitution	Nonsynonymous coding	36%
MM04T	VPS8	vacuolar protein sorting 8 homolog (S. cerevisiae)	CCDS46971.1	chr3_184612645-184612645__A	NA	Insertion	Frameshift	19%
MM04T	VRK2	vaccinia related kinase 2	CCDS1859.1	chr2_58313555-58313555_G_A	113G>E	Substitution	Nonsynonymous coding	29%
MM04T	VRTN	vertebrae development associated	CCDS9830.1	chr14_74823964-74823964_G_A	160D>N	Substitution	Nonsynonymous coding	29%
MM04T	VSIG4	V-set and immunoglobulin domain containing 4	CCDS14383.1	chrX_65253346-65253346_C_A	128D>Y	Substitution	Nonsynonymous coding	18%
MM04T	VTCN1	V-set domain containing T cell activation inhibitor 1	CCDS894.1	chr1_117695864-117695864_G_T	191F>L	Substitution	Nonsynonymous coding	40%
MM04T	VTCN1	V-set domain containing T cell activation inhibitor 1	CCDS894.1	chr1_117712771-117712771_G_T	19L>M	Substitution	Nonsynonymous coding	33%
MM04T	VWA5B2	von Willebrand factor A domain containing 5B2	NM_138345	chr3_183959518-183959518_G_A	1141D>N	Substitution	Nonsynonymous coding	38%
MM04T	VWA8	von Willebrand factor A domain containing 8	CCDS41881.1	chr13_42465570-42465570_C_T	213D>N	Substitution	Nonsynonymous coding	23%
MM04T	VWCE	von Willebrand factor C and EGF domains	CCDS8002.1	chr11_61044140-61044140_A_G	474S>P	Substitution	Nonsynonymous coding	27%

MM04T	VWCE	von Willebrand factor C and EGF domains	CCDS8002.1	chr11_61050302-61050302_C_T	206R>Q	Substitution	Nonsynonymous coding	32%
MM04T	VWF	von Willebrand factor	CCDS8539.1	chr12_6219561-6219561_C_A	171D>Y	Substitution	Nonsynonymous coding	16%
MM04T	WAC	WW domain containing adaptor with coiled-coil	CCDS7159.1	chr10_28903608-28903608_G_A	517R>H	Substitution	Nonsynonymous coding	36%
MM04T	WARS2	tryptophanyl tRNA synthetase 2, mitochondrial	CCDS900.1	chr1_119576721-119576721_G_T	211L>I	Substitution	Nonsynonymous coding	19%
MM04T	WAS	Wiskott-Aldrich syndrome	CCDS14303.1	chrX_48549521-48549521_G_T	493D>Y	Substitution	Nonsynonymous coding	28%
MM04T	WASF3	WAS protein family, member 3	CCDS9318.1	chr13_27256847-27256847_C_T	363L>F	Substitution	Nonsynonymous coding	28%
MM04T	WBP4	WW domain binding protein 4	CCDS9375.1	chr13_41654879-41654879_C_T	285S>L	Substitution	Nonsynonymous coding	21%
MM04T	WBP5	WW domain binding protein 5	CCDS14507.1	chrX_102612767-102612767_C_A	52S>Y	Substitution	Nonsynonymous coding	24%
MM04T	WDFY3	WD repeat and FYVE domain containing 3	CCDS3609.1	chr4_85656266-85656266_G_A	2308S>L	Substitution	Nonsynonymous coding	26%
MM04T	WDFY3	WD repeat and FYVE domain containing 3	CCDS3609.1	chr4_85617299-85617299_C_A	2909E>X	Substitution	Nonsense	18%
MM04T	WDFY4	WDFY family member 4	CCDS44385.1	chr10_49937550-49937550_A_G	349T>A	Substitution	Nonsynonymous coding	35%
MM04T	WDFY4	WDFY family member 4	CCDS44385.1	chr10_49939431-49939431_A_C	469K>T	Substitution	Nonsynonymous coding	30%
MM04T	WDFY4	WDFY family member 4	CCDS44385.1	chr10_49983792-49983792_C_T	935S>L	Substitution	Nonsynonymous coding	19%
MM04T	WDFY4	WDFY family member 4	CCDS44385.1	chr10_50108280-50108280_G_T	2484E>D	Substitution	Nonsynonymous coding	32%
MM04T	WDR19	WD repeat domain 19	CCDS47042.1	chr4_39188187-39188187_C_T	43R>C	Substitution	Nonsynonymous coding	44%
MM04T	WDR19	WD repeat domain 19	CCDS47042.1	chr4_39245931-39245931_C_T	829R>X	Substitution	Nonsense	36%
MM04T	WDR26	WD repeat domain 26	NM_025160	chr1_224621600-224621600_T_G	70N>H	Substitution	Nonsynonymous coding	24%
MM04T	WDR3	WD repeat domain 3	CCDS898.1	chr1_118477264-118477264_A_G	114K>E	Substitution	Nonsynonymous coding	29%
MM04T	WDR3	WD repeat domain 3	CCDS898.1	chr1_118499701-118499701_G_T	822E>X	Substitution	Nonsense	34%
MM04T	WDR35	WD repeat domain 35	CCDS33152.1	chr2_20141566-20141566_A_C	638F>C	Substitution	Nonsynonymous coding	34%
MM04T	WDR35	WD repeat domain 35	CCDS33152.1	chr2_20169254-20169254_C_T	332R>Q	Substitution	Nonsynonymous coding	30%
MM04T	WDR35	WD repeat domain 35	CCDS33152.1	chr2_20169282-20169282_C_T	323D>N	Substitution	Nonsynonymous coding	25%
MM04T	WDR37	WD repeat domain 37	CCDS7057.1	chr10_1132292-1132292_G_A	200G>D	Substitution	Nonsynonymous coding	26%
MM04T	WDR43	WD repeat domain 43	CCDS46251.1	chr2_29124983-29124983_A_C	119K>T	Substitution	Nonsynonymous coding	35%
MM04T	WDR44	WD repeat domain 44	CCDS14572.1	chrX_117543570-117543570_G_A	551R>Q	Substitution	Nonsynonymous coding	42%
MM04T	WDR45B	WD repeat domain 45B	CCDS11815.2	chr17_80601838-80601838_T_G	43E>D	Substitution	Nonsynonymous coding	40%
MM04T	WDR49	WD repeat domain 49	CCDS3201.1	chr3_167196724-167196724_C_T	679R>Q	Substitution	Nonsynonymous coding	30%
MM04T	WDR52	WD repeat domain 52	CCDS2972.1	chr3_113115522-113115522_C_T	541R>Q	Substitution	Nonsynonymous coding	34%
MM04T	WDR5B	WD repeat domain 5B	CCDS3012.1	chr3_122134035-122134035_G_T	114S>Y	Substitution	Nonsynonymous coding	37%
MM04T	WDR6	WD repeat domain 6	CCDS2782.2	chr3_49052661-49052661_G_T	1132E>D	Substitution	Nonsynonymous coding	27%
MM04T	WDR63	WD repeat domain 63	CCDS702.1	chr1_85551539-85551539_G_A	189R>Q	Substitution	Nonsynonymous coding	49%
MM04T	WDR63	WD repeat domain 63	CCDS702.1	chr1_85564222-85564222_C_A	454L>I	Substitution	Nonsynonymous coding	43%

MM04T	WDR64	WD repeat domain 64	CCDS1619.1	chr1_241912851-241912851_A_G	243T>A	Substitution	Nonsynonymous coding	31%
MM04T	WDR64	WD repeat domain 64	NM_144625	chr1_241934960-241934960_C_A	NA	Substitution	Splice site acceptor	18%
MM04T	WDR66	WD repeat domain 66	CCDS41853.1	chr12_122361742-122361742_G_T	198R>I	Substitution	Nonsynonymous coding	22%
MM04T	WDR66	WD repeat domain 66	CCDS41853.1	chr12_122398591-122398591_G_A	745R>Q	Substitution	Nonsynonymous coding	27%
MM04T	WDR67	WD repeat domain 67	CCDS6338.1	chr8_124132346-124132346_T_G	496Y>X	Substitution	Nonsense	14%
MM04T	WDR69	WD repeat domain 69	CCDS2470.1	chr2_228750117-228750117_G_A	31D>N	Substitution	Nonsynonymous coding	28%
MM04T	WDR72	WD repeat domain 72	CCDS10151.1	chr15_53992005-53992005_C_A	569E>D	Substitution	Nonsynonymous coding	35%
MM04T	WDR75	WD repeat domain 75	CCDS2298.1	chr2_190338930-190338930_C_A	690L>I	Substitution	Nonsynonymous coding	32%
MM04T	WDR76	WD repeat domain 76	CCDS10106.1	chr15_44134682-44134682_G_T	268G>X	Substitution	Nonsense	23%
MM04T	WDR87	WD repeat domain 87	CCDS46063.1	chr19_38377263-38377263_C_T	2311E>K	Substitution	Nonsynonymous coding	33%
MM04T	WDR87	WD repeat domain 87	CCDS46063.1	chr19_38380612-38380612_C_A	1194K>N	Substitution	Nonsynonymous coding	25%
MM04T	WDR87	WD repeat domain 87	CCDS46063.1	chr19_38384165-38384165_C_A	687Q>H	Substitution	Nonsynonymous coding	22%
MM04T	WDR87	WD repeat domain 87	CCDS46063.1	chr19_38383342-38383342_C_A	962G>X	Substitution	Nonsense	33%
MM04T	WDR88	WD repeat domain 88	CCDS12429.1	chr19_33647413-33647413_A_C	321E>A	Substitution	Nonsynonymous coding	33%
MM04T	WDR96	WD repeat domain 96	CCDS31281.1	chr10_105948072-105948072_C_A	548R>I	Substitution	Nonsynonymous coding	19%
MM04T	WDR96	WD repeat domain 96	CCDS31281.1	chr10_105922119-105922119_C_A	1097E>X	Substitution	Nonsense	29%
MM04T	WFDC1	WAP four-disulfide core domain 1	CCDS10946.1	chr16_84358066-84358066_G_T	202E>X	Substitution	Nonsense	21%
MM04T	WHSC1	Wolf-Hirschhorn syndrome candidate 1	ENST00000350915	chr4_1955003-1955003_C_A	45S>Y	Substitution	Nonsynonymous coding	31%
MM04T	WHSC1L1	Wolf-Hirschhorn syndrome candidate 1-like 1	CCDS43729.1	chr8_38162828-38162828_C_T	793R>H	Substitution	Nonsynonymous coding	22%
MM04T	WIZ	widely interspaced zinc finger motifs	ENST00000389282	chr19_15550582-15550582_A_G	360F>S	Substitution	Nonsynonymous coding	18%
MM04T	WNK2	WNK lysine deficient protein kinase 2	NM_006648	chr9_96051652-96051652_C_G	1539P>R	Substitution	Nonsynonymous coding	36%
MM04T	WNK3	WNK lysine deficient protein kinase 3	CCDS14357.1	chrX_54224858-54224858_G_A	1768P>S	Substitution	Nonsynonymous coding	44%
MM04T	WNK3	WNK lysine deficient protein kinase 3	CCDS14357.1	chrX_54263906-54263906_T_G	1365N>H	Substitution	Nonsynonymous coding	34%
MM04T	WNK3	WNK lysine deficient protein kinase 3	CCDS14357.1	chrX_54263777-54263777_C_A	1408E>X	Substitution	Nonsense	27%
MM04T	WNT11	wingless-type MMTV integration site family, member 11	CCDS8242.1	chr11_75902637-75902637_C_A	287E>D	Substitution	Nonsynonymous coding	20%
MM04T	WNT2B	wingless-type MMTV integration site family, member 2B	CCDS847.1	chr1_113057714-113057714_G_T	134R>I	Substitution	Nonsynonymous coding	25%
MM04T	WNT3	wingless-type MMTV integration site family, member 3	CCDS11505.1	chr17_44847360-44847360_G_A	126A>V	Substitution	Nonsynonymous coding	26%
MM04T	WNT3A	wingless-type MMTV integration site family, member 3A	CCDS1564.1	chr1_228194857-228194857_C_A	10L>I	Substitution	Nonsynonymous coding	17%
MM04T	WNT4	wingless-type MMTV integration site family, member 4	ENST00000374655	chr1_22453936-22453936_G_T	118L>I	Substitution	Nonsynonymous coding	30%
MM04T	WNT7A	wingless-type MMTV integration site family, member 7A	CCDS2616.1	chr3_13896057-13896057_T_C	181N>S	Substitution	Nonsynonymous coding	31%
MM04T	WNT8A	wingless-type MMTV integration site family, member 8A	CCDS43368.1	chr5_137420328-137420328_A_G	NA	Substitution	Splice site donor	19%
MM04T	WRN	Werner syndrome, RecQ helicase-like	CCDS6082.1	chr8_30938628-30938628_T_C	362V>A	Substitution	Nonsynonymous coding	30%

MM04T	WT1-AS	WT1 antisense RNA	ENST00000395900	chr11_32460485-32460485_G_A	20A>T	Substitution	Nonsynonymous coding	17%
MM04T	WT1-AS	WT1 antisense RNA	ENST00000395900	chr11_32460534-32460534_C_T	36S>L	Substitution	Nonsynonymous coding	19%
MM04T	WWC1	WW and C2 domain containing 1	CCDS4366.1	chr5_167857217-167857217_A_C	692Q>H	Substitution	Nonsynonymous coding	35%
MM04T	WWC3	WWC family member 3	CCDS14136.1	chrX_10106932-10106932_C_T	1014R>W	Substitution	Nonsynonymous coding	37%
MM04T	WWP2	WW domain containing E3 ubiquitin protein ligase 2	CCDS10885.1	chr16_69973017-69973017_G_A	811E>K	Substitution	Nonsynonymous coding	30%
MM04T	XBP1	X-box binding protein 1	NM_001079539	chr22_29191342-29191342_T_G	318N>H	Substitution	Nonsynonymous coding	26%
MM04T	XIAP	X-linked inhibitor of apoptosis	CCDS14606.1	chrX_123019646-123019646_C_T	45S>L	Substitution	Nonsynonymous coding	31%
MM04T	XIRP1	xin actin-binding repeat containing 1	CCDS2683.1	chr3_39226726-39226726_C_A	1404S>I	Substitution	Nonsynonymous coding	18%
MM04T	XIRP1	xin actin-binding repeat containing 1	CCDS2683.1	chr3_39225947-39225947_G_A	1664R>X	Substitution	Nonsense	17%
MM04T	XIRP2	xin actin-binding repeat containing 2	CCDS42769.1	chr2_167760210-167760210_C_T	73S>L	Substitution	Nonsynonymous coding	26%
MM04T	XIRP2	xin actin-binding repeat containing 2	CCDS42769.1	chr2_168067335-168067335_C_T	218S>F	Substitution	Nonsynonymous coding	20%
MM04T	XIRP2	xin actin-binding repeat containing 2	CCDS42769.1	chr2_168099972-168099972_G_T	690K>N	Substitution	Nonsynonymous coding	31%
MM04T	XIRP2	xin actin-binding repeat containing 2	CCDS42769.1	chr2_168101234-168101234_C_T	1111S>L	Substitution	Nonsynonymous coding	40%
MM04T	XIRP2	xin actin-binding repeat containing 2	CCDS42769.1	chr2_168104508-168104508_G_T	2202K>N	Substitution	Nonsynonymous coding	27%
MM04T	XIRP2	xin actin-binding repeat containing 2	CCDS42769.1	chr2_168105113-168105113_C_A	2404S>Y	Substitution	Nonsynonymous coding	23%
MM04T	XIRP2	xin actin-binding repeat containing 2	CCDS42769.1	chr2_168108332-168108332_G_T	3477R>I	Substitution	Nonsynonymous coding	27%
MM04T	XIRP2	xin actin-binding repeat containing 2	CCDS42768.1	chr2_168115020-168115020_C_T	655A>V	Substitution	Nonsynonymous coding	32%
MM04T	XIRP2	xin actin-binding repeat containing 2	CCDS42768.1	chr2_168115189-168115189_T_G	711N>K	Substitution	Nonsynonymous coding	27%
MM04T	XIRP2	xin actin-binding repeat containing 2	CCDS42769.1	chr2_168099925-168099925_G_T	675E>X	Substitution	Nonsense	23%
MM04T	XIRP2	xin actin-binding repeat containing 2	CCDS42769.1	chr2_168104212-168104212_G_T	2104E>X	Substitution	Nonsense	27%
MM04T	XIRP2	xin actin-binding repeat containing 2	CCDS42769.1	chr2_168105142-168105142_G_T	2414G>X	Substitution	Nonsense	19%
MM04T	XKR3	XK, Kell blood group complex subunit-related family, member 3	CCDS42975.1	chr22_17280897-17280897_C_A	118R>I	Substitution	Nonsynonymous coding	42%
MM04T	XKR6	XK, Kell blood group complex subunit-related family, member 6	CCDS5978.2	chr8_10756032-10756032_C_A	452E>D	Substitution	Nonsynonymous coding	17%
MM04T	XKR9	XK, Kell blood group complex subunit-related family, member 9	CCDS34905.1	chr8_71646348-71646348_C_A	271L>I	Substitution	Nonsynonymous coding	36%
MM04T	XPA	xeroderma pigmentosum, complementation group A	CCDS6729.1	chr9_100447259-100447259_G_A	207R>X	Substitution	Nonsense	15%
MM04T	XPC	xeroderma pigmentosum, complementation group C	CCDS46763.1	chr3_14207048-14207048_C_T	220R>Q	Substitution	Nonsynonymous coding	30%
MM04T	XPO1	exportin 1 (CRM1 homolog, yeast)	CCDS33205.1	chr2_61717776-61717776_C_A	NA	Substitution	Splice site donor	19%
MM04T	XPO5	exportin 5	CCDS47430.1	chr6_43541337-43541337_A_C	36F>C	Substitution	Nonsynonymous coding	26%
MM04T	XRCC1	X-ray repair complementing defective repair in Chinese hamster cells 1	CCDS12624.1	chr19_44057007-44057007_C_A	255K>N	Substitution	Nonsynonymous coding	34%
MM04T	XRCC1	X-ray repair complementing defective repair in Chinese hamster cells 1	CCDS12624.1	chr19_44058887-44058887_G_A	109R>C	Substitution	Nonsynonymous coding	30%
MM04T	XRN2	5'-3' exoribonuclease 2	CCDS13144.1	chr20_21314470-21314470_G_T	354E>D	Substitution	Nonsynonymous coding	28%
MM04T	XRN2	5'-3' exoribonuclease 2	CCDS13144.1	chr20_21327175-21327175_G_T	551R>I	Substitution	Nonsynonymous coding	40%

MM04T	XYLT1	xylosyltransferase I	CCDS10569.1	chr16_17232365-17232365_C_A	537E>D	Substitution	Nonsynonymous coding	31%
MM04T	XYLT1	xylosyltransferase I	CCDS10569.1	chr16_17353100-17353100_C_T	220G>R	Substitution	Nonsynonymous coding	20%
MM04T	YAF2	YY1 associated factor 2	CCDS31775.1	chr12_42555495-42555495_C_A	75K>N	Substitution	Nonsynonymous coding	24%
MM04T	YIPF7	Yip1 domain family, member 7	NM_182592	chr4_44631527-44631527_A_C	131L>V	Substitution	Nonsynonymous coding	16%
MM04T	YLPM1	YLP motif containing 1	CCDS45135.1	chr14_75283313-75283313_C_A	1869L>I	Substitution	Nonsynonymous coding	31%
MM04T	YOD1	YOD1 OTU deubiquinating enzyme 1 homolog (S. cerevisiae)	CCDS31002.1	chr1_207224035-207224035_G_T	114S>Y	Substitution	Nonsynonymous coding	36%
MM04T	YTHDC1	YTH domain containing 1	CCDS33992.1	chr4_69203501-69203501_C_A	83R>I	Substitution	Nonsynonymous coding	36%
MM04T	YTHDC2	YTH domain containing 2	CCDS4113.1	chr5_112868649-112868649_G_A	250R>Q	Substitution	Nonsynonymous coding	23%
MM04T	YTHDC2	YTH domain containing 2	CCDS4113.1	chr5_112889544-112889544_G_A	682R>Q	Substitution	Nonsynonymous coding	34%
MM04T	YWHAB	tyrosine 3-monooxygenase/tryptophan 5-monooxygenase activation protein, beta	CCDS13339.1	chr20_43533730-43533730_G_T	182E>D	Substitution	Nonsynonymous coding	21%
MM04T	YY1AP1	YY1 associated protein 1	CCDS1115.1	chr1_155630514-155630514_G_A	442A>V	Substitution	Nonsynonymous coding	21%
MM04T	ZAN	zonadhesin	ENST00000419263	chr7_100348394-100348394_G_A	466E>K	Substitution	Nonsynonymous coding	22%
MM04T	ZAN	zonadhesin	ENST00000419263	chr7_100388611-100388611_G_A	2468G>R	Substitution	Nonsynonymous coding	28%
MM04T	ZAN	zonadhesin	ENST00000425997	chr7_100390155-100390155_G_A	2613R>H	Substitution	Nonsynonymous coding	23%
MM04T	ZAR1L	zygote arrest 1-like	CCDS45023.1	chr13_32878092-32878092_C_T	297R>Q	Substitution	Nonsynonymous coding	18%
MM04T	ZBBX	zinc finger, B-box domain containing	CCDS3199.2	chr3_167090661-167090661_G_A	10P>L	Substitution	Nonsynonymous coding	31%
MM04T	ZBP1	Z-DNA binding protein 1	CCDS13461.1	chr20_56190072-56190072_C_T	125A>T	Substitution	Nonsynonymous coding	21%
MM04T	ZBTB1	zinc finger and BTB domain containing 1	CCDS45126.1	chr14_64989726-64989726_G_T	502D>Y	Substitution	Nonsynonymous coding	24%
MM04T	ZBTB10	zinc finger and BTB domain containing 10	CCDS47880.1	chr8_81412123-81412123_G_A	456R>Q	Substitution	Nonsynonymous coding	30%
MM04T	ZBTB10	zinc finger and BTB domain containing 10	CCDS47880.1	chr8_81412490-81412490_C_A	578N>K	Substitution	Nonsynonymous coding	34%
MM04T	ZBTB10	zinc finger and BTB domain containing 10	ENST00000379091	chr8_81398088-81398088_G_T	24E>X	Substitution	Nonsense	21%
MM04T	ZBTB11	zinc finger and BTB domain containing 11	CCDS2943.1	chr3_101370309-101370309_G_T	955H>N	Substitution	Nonsynonymous coding	22%
MM04T	ZBTB14	zinc finger and BTB domain containing 14	CCDS11837.1	chr18_5291160-5291160_C_A	349K>N	Substitution	Nonsynonymous coding	29%
MM04T	ZBTB16	zinc finger and BTB domain containing 16	CCDS8367.1	chr11_114112922-114112922_G_T	496G>V	Substitution	Nonsynonymous coding	19%
MM04T	ZBTB18	zinc finger and BTB domain containing 18	CCDS1622.1	chr1_244218015-244218015_T_G	313N>K	Substitution	Nonsynonymous coding	35%
MM04T	ZBTB21	zinc finger and BTB domain containing 21	CCDS13678.1	chr21_43412136-43412136_T_G	690K>T	Substitution	Nonsynonymous coding	35%
MM04T	ZBTB26	zinc finger and BTB domain containing 26	CCDS6847.1	chr9_125682119-125682119_A_C	32F>C	Substitution	Nonsynonymous coding	33%
MM04T	ZBTB43	zinc finger and BTB domain containing 43	CCDS6867.1	chr9_129595642-129595642_C_T	285S>L	Substitution	Nonsynonymous coding	23%
MM04T	ZBTB43	zinc finger and BTB domain containing 43	CCDS6867.1	chr9_129596134-129596134_C_A	449S>Y	Substitution	Nonsynonymous coding	23%
MM04T	ZBTB46	zinc finger and BTB domain containing 46	CCDS13538.1	chr20_62421590-62421590_C_T	174R>Q	Substitution	Nonsynonymous coding	34%
MM04T	ZBTB49	zinc finger and BTB domain containing 49	CCDS3375.1	chr4_4317658-4317658_C_T	528R>W	Substitution	Nonsynonymous coding	25%
MM04T	ZBTB7C	zinc finger and BTB domain containing 7C	CCDS32830.1	chr18_45567285-45567285_G_A	65A>V	Substitution	Nonsynonymous coding	32%

MM04T	ZC3H10	zinc finger CCCH-type containing 10	CCDS8903.1	chr12_56515090-56515090_G_T	248R>S	Substitution	Nonsynonymous coding	27%
MM04T	ZC3H12C	zinc finger CCCH-type containing 12C	CCDS44727.1	chr11_110035605-110035605_C_A	599L>M	Substitution	Nonsynonymous coding	37%
MM04T	ZC3H13	zinc finger CCCH-type containing 13	ENST00000242848	chr13_46533748-46533748_G_A	1569A>V	Substitution	Nonsynonymous coding	29%
MM04T	ZC3H18	zinc finger CCCH-type containing 18	CCDS10967.1	chr16_88665046-88665046_G_T	286E>X	Substitution	Nonsense	20%
MM04T	ZC3H6	zinc finger CCCH-type containing 6	CCDS46393.1	chr2_113060830-113060830_A_C	80S>R	Substitution	Nonsynonymous coding	32%
MM04T	ZC3HAV1	zinc finger CCCH-type, antiviral 1	CCDS5851.1	chr7_138732434-138732434_G_A	872S>L	Substitution	Nonsynonymous coding	19%
MM04T	ZC3HC1	zinc finger, C3HC-type containing 1	CCDS34753.1	chr7_129666019-129666019_G_C	252S>C	Substitution	Nonsynonymous coding	35%
MM04T	ZC4H2	zinc finger, C4H2 domain containing	CCDS14380.1	chrX_64139983-64139983_C_A	126E>X	Substitution	Nonsense	33%
MM04T	ZCCHC14	zinc finger, CCHC domain containing 14	ENST00000412336	chr16_87445046-87445046_C_A	957S>I	Substitution	Nonsynonymous coding	31%
MM04T	ZCCHC14	zinc finger, CCHC domain containing 14	CCDS10961.1	chr16_87457471-87457471_G_T	125S>Y	Substitution	Nonsynonymous coding	28%
MM04T	ZCCHC18	zinc finger, CCHC domain containing 18	NM_001143978	chrX_103359553-103359553_G_A	251A>T	Substitution	Nonsynonymous coding	23%
MM04T	ZCCHC2	zinc finger, CCHC domain containing 2	CCDS45880.1	chr18_60241584-60241584_C_T	757A>V	Substitution	Nonsynonymous coding	37%
MM04T	ZCCHC5	zinc finger, CCHC domain containing 5	CCDS14440.1	chrX_77912999-77912999_T_G	307S>R	Substitution	Nonsynonymous coding	34%
MM04T	ZCCHC6	zinc finger, CCHC domain containing 6	CCDS35057.1	chr9_88940303-88940303_C_A	579E>X	Substitution	Nonsense	24%
MM04T	ZCCHC7	zinc finger, CCHC domain containing 7	CCDS6608.2	chr9_37126547-37126547_A_C	73K>T	Substitution	Nonsynonymous coding	16%
MM04T	ZCCHC7	zinc finger, CCHC domain containing 7	CCDS6608.2	chr9_37126821-37126821_G_T	164E>D	Substitution	Nonsynonymous coding	30%
MM04T	ZCCHC8	zinc finger, CCHC domain containing 8	NM_017612	chr12_122964834-122964834_A_C	348V>G	Substitution	Nonsynonymous coding	34%
MM04T	ZDBF2	zinc finger, DBF-type containing 2	CCDS46501.1	chr2_207174995-207174995_A_C	1915K>Q	Substitution	Nonsynonymous coding	34%
MM04T	ZDBF2	zinc finger, DBF-type containing 2	CCDS46501.1	chr2_207170390-207170390_G_T	380E>X	Substitution	Nonsense	31%
MM04T	ZDBF2	zinc finger, DBF-type containing 2	CCDS46501.1	chr2_207171569-207171569_G_T	773E>X	Substitution	Nonsense	21%
MM04T	ZDHHC15	zinc finger, DHHC-type containing 15	NM_001146257	chrX_74670594-74670594_T_C	132Y>C	Substitution	Nonsynonymous coding	29%
MM04T	ZDHHC17	zinc finger, DHHC-type containing 17	CCDS44946.1	chr12_77220694-77220694_C_T	302R>W	Substitution	Nonsynonymous coding	33%
MM04T	ZDHHC19	zinc finger, DHHC-type containing 19	CCDS43190.1	chr3_195938150-195938150_C_T	13E>K	Substitution	Nonsynonymous coding	19%
MM04T	ZDHHC23	zinc finger, DHHC-type containing 23	CCDS33827.1	chr3_113672618-113672618_G_A	78R>Q	Substitution	Nonsynonymous coding	31%
MM04T	ZDHHC5	zinc finger, DHHC-type containing 5	CCDS7965.1	chr11_57457837-57457837_C_A	196F>L	Substitution	Nonsynonymous coding	24%
MM04T	ZEB2	zinc finger E-box binding homeobox 2	CCDS2186.1	chr2_145156336-145156336_G_T	806F>L	Substitution	Nonsynonymous coding	23%
MM04T	ZEB2	zinc finger E-box binding homeobox 2	CCDS2186.1	chr2_145157774-145157774_G_A	327S>L	Substitution	Nonsynonymous coding	24%
MM04T	ZFAND4	zinc finger, AN1-type domain 4	CCDS7214.1	chr10_46121400-46121400__A	NA	Insertion	Frameshift	27%
MM04T	ZFC3H1	zinc finger, C3H1-type containing	CCDS41813.1	chr12_72023470-72023470_A_C	1249F>V	Substitution	Nonsynonymous coding	32%
MM04T	ZFC3H1	zinc finger, C3H1-type containing	CCDS41813.1	chr12_72024652-72024652_A_C	1185F>V	Substitution	Nonsynonymous coding	32%
MM04T	ZFC3H1	zinc finger, C3H1-type containing	CCDS41813.1	chr12_72056981-72056981_C_T	137S>N	Substitution	Nonsynonymous coding	34%
MM04T	ZFHX2	zinc finger homeobox 2	ENST00000419474	chr14_23993066-23993066_C_T	2029D>N	Substitution	Nonsynonymous coding	10%

MM04T	ZFHX3	zinc finger homeobox 3	CCDS10908.1	chr16_72822481-72822481_G_A	3232R>C	Substitution	Nonsynonymous coding	39%
MM04T	ZFHX3	zinc finger homeobox 3	CCDS10908.1	chr16_72830958-72830958_C_T	1875E>K	Substitution	Nonsynonymous coding	35%
MM04T	ZFHX3	zinc finger homeobox 3	CCDS10908.1	chr16_72993903-72993903_C_T	48G>R	Substitution	Nonsynonymous coding	46%
MM04T	ZFHX4	zinc finger homeobox 4	CCDS47878.1	chr8_77761750-77761750_C_A	1171F>L	Substitution	Nonsynonymous coding	37%
MM04T	ZFP1	ZFP1 zinc finger protein	CCDS10914.2	chr16_75200769-75200769_G_T	39E>D	Substitution	Nonsynonymous coding	22%
MM04T	ZFP14	ZFP14 zinc finger protein	CCDS33002.1	chr19_36832078-36832078_C_T	217R>Q	Substitution	Nonsynonymous coding	28%
MM04T	ZFP14	ZFP14 zinc finger protein	CCDS33002.1	chr19_36832177-36832177_C_T	184R>H	Substitution	Nonsynonymous coding	31%
MM04T	ZFP28	ZFP28 zinc finger protein	CCDS12946.1	chr19_57066400-57066400_G_T	749R>I	Substitution	Nonsynonymous coding	39%
MM04T	ZFP28	ZFP28 zinc finger protein	CCDS12946.1	chr19_57065826-57065826_G_T	558E>X	Substitution	Nonsense	17%
MM04T	ZFP30	ZFP30 zinc finger protein	CCDS33005.1	chr19_38126825-38126825_C_A	206R>I	Substitution	Nonsynonymous coding	31%
MM04T	ZFP30	ZFP30 zinc finger protein	CCDS33005.1	chr19_38126256-38126256_C_A	396E>X	Substitution	Nonsense	27%
MM04T	ZFP41	ZFP41 zinc finger protein	ENST00000344692	chr8_144357014-144357014_C_T	12R>C	Substitution	Nonsynonymous coding	27%
MM04T	ZFP42	ZFP42 zinc finger protein	CCDS3849.1	chr4_188924375-188924375_G_T	138E>D	Substitution	Nonsynonymous coding	34%
MM04T	ZFP42	ZFP42 zinc finger protein	CCDS3849.1	chr4_188924520-188924520_G_A	187A>T	Substitution	Nonsynonymous coding	28%
MM04T	ZFP57	ZFP57 zinc finger protein	CCDS43436.1	chr6_29643715-29643715_G_T	62S>Y	Substitution	Nonsynonymous coding	20%
MM04T	ZFP69B	ZFP69 zinc finger protein B	CCDS452.1	chr1_40928144-40928144_C_T	61S>L	Substitution	Nonsynonymous coding	28%
MM04T	ZFP82	ZFP82 zinc finger protein	CCDS12493.1	chr19_36884673-36884673_C_A	190R>I	Substitution	Nonsynonymous coding	27%
MM04T	ZFP90	ZFP90 zinc finger protein	CCDS42183.1	chr16_68597006-68597006_T_C	106S>P	Substitution	Nonsynonymous coding	26%
MM04T	ZFPM2	zinc finger protein, FOG family member 2	CCDS47908.1	chr8_106814591-106814591_C_A	761L>I	Substitution	Nonsynonymous coding	32%
MM04T	ZFR	zinc finger RNA binding protein	CCDS34139.1	chr5_32400255-32400255_C_T	524E>K	Substitution	Nonsynonymous coding	18%
MM04T	ZFR	zinc finger RNA binding protein	CCDS34139.1	chr5_32415076-32415076_G_T	261S>Y	Substitution	Nonsynonymous coding	28%
MM04T	ZFYVE20	zinc finger, FYVE domain containing 20	CCDS2623.1	chr3_15122310-15122310_C_A	NA	Substitution	Splice site acceptor	19%
MM04T	ZFYVE26	zinc finger, FYVE domain containing 26	CCDS9788.1	chr14_68251891-68251891_C_A	1136K>N	Substitution	Nonsynonymous coding	23%
MM04T	ZHX1	zinc fingers and homeoboxes 1	CCDS6342.1	chr8_124267871-124267871_C_T	106E>K	Substitution	Nonsynonymous coding	20%
MM04T	ZIC4	Zic family member 4	CCDS43160.1	chr3_147114121-147114121_C_T	69G>E	Substitution	Nonsynonymous coding	30%
MM04T	ZIM3	zinc finger, imprinted 3	CCDS33125.1	chr19_57646623-57646623_C_A	361R>I	Substitution	Nonsynonymous coding	23%
MM04T	ZKSCAN2	zinc finger with KRAB and SCAN domains 2	CCDS32410.1	chr16_25258304-25258304_G_A	405R>X	Substitution	Nonsense	25%
MM04T	ZMAT1	zinc finger, matrin-type 1	CCDS35348.1	chrX_101139356-101139356_C_A	348R>I	Substitution	Nonsynonymous coding	29%
MM04T	ZMIZ1	zinc finger, MIZ-type containing 1	CCDS7357.1	chr10_81072478-81072478_A_G	1059D>G	Substitution	Nonsynonymous coding	13%
MM04T	ZMYND15	zinc finger, MYND-type containing 15	CCDS45584.1	chr17_4644406-4644406_G_T	188R>I	Substitution	Nonsynonymous coding	27%
MM04T	ZNF101	zinc finger protein 101	CCDS32971.1	chr19_19791027-19791027_G_T	410R>I	Substitution	Nonsynonymous coding	35%
MM04T	ZNF106	zinc finger protein 106	CCDS32208.1	chr15_42731790-42731790_C_T	1306A>T	Substitution	Nonsynonymous coding	16%

MM04T	ZNF107	zinc finger protein 107	CCDS5527.1	chr7_64167508-64167508_G_T	276E>X	Substitution	Nonsense	27%
MM04T	ZNF12	zinc finger protein 12	CCDS47538.1	chr7_6736988-6736988_G_T	74L>I	Substitution	Nonsynonymous coding	29%
MM04T	ZNF132	zinc finger protein 132	CCDS12980.1	chr19_58944706-58944706_T_G	702K>T	Substitution	Nonsynonymous coding	22%
MM04T	ZNF132	zinc finger protein 132	CCDS12980.1	chr19_58944797-58944797_G_A	672R>W	Substitution	Nonsynonymous coding	35%
MM04T	ZNF134	zinc finger protein 134	CCDS42638.1	chr19_58131918-58131918_C_T	144T>I	Substitution	Nonsynonymous coding	30%
MM04T	ZNF138	zinc finger protein 138	CCDS34645.1	chr7_64292015-64292015_A_C	75K>T	Substitution	Nonsynonymous coding	18%
MM04T	ZNF14	zinc finger protein 14	CCDS12409.1	chr19_19822697-19822697_G_T	465H>N	Substitution	Nonsynonymous coding	28%
MM04T	ZNF143	zinc finger protein 143	CCDS7799.2	chr11_9516259-9516259_C_T	238R>X	Substitution	Nonsense	28%
MM04T	ZNF157	zinc finger protein 157	CCDS14278.1	chrX_47272941-47272941_G_T	490R>I	Substitution	Nonsynonymous coding	41%
MM04T	ZNF160	zinc finger protein 160	CCDS12859.1	chr19_53572226-53572226_G_A	521R>C	Substitution	Nonsynonymous coding	24%
MM04T	ZNF18	zinc finger protein 18	CCDS32568.1	chr17_11886683-11886683_C_A	265E>X	Substitution	Nonsense	28%
MM04T	ZNF180	zinc finger protein 180	CCDS12639.1	chr19_44980992-44980992_C_T	569R>K	Substitution	Nonsynonymous coding	38%
MM04T	ZNF180	zinc finger protein 180	CCDS12639.1	chr19_44981032-44981032_T_G	556K>Q	Substitution	Nonsynonymous coding	30%
MM04T	ZNF182	zinc finger protein 182	CCDS35236.1	chrX_47835717-47835717_C_A	590R>I	Substitution	Nonsynonymous coding	32%
MM04T	ZNF184	zinc finger protein 184	CCDS4624.1	chr6_27419689-27419689_C_A	550R>I	Substitution	Nonsynonymous coding	34%
MM04T	ZNF189	zinc finger protein 189	CCDS6754.1	chr9_104162267-104162267_G_T	45E>D	Substitution	Nonsynonymous coding	40%
MM04T	ZNF189	zinc finger protein 189	CCDS6754.1	chr9_104170946-104170946_G_A	299R>Q	Substitution	Nonsynonymous coding	29%
MM04T	ZNF202	zinc finger protein 202	CCDS8443.1	chr11_123598960-123598960_G_T	238T>K	Substitution	Nonsynonymous coding	37%
MM04T	ZNF212	zinc finger protein 212	CCDS5896.1	chr7_148947280-148947280_A_C	19T>P	Substitution	Nonsynonymous coding	26%
MM04T	ZNF212	zinc finger protein 212	CCDS5896.1	chr7_148950883-148950883_A_C	289K>Q	Substitution	Nonsynonymous coding	33%
MM04T	ZNF217	zinc finger protein 217	CCDS13443.1	chr20_52199220-52199220_C_T	49R>Q	Substitution	Nonsynonymous coding	33%
MM04T	ZNF222	zinc finger protein 222	CCDS46098.1	chr19_44536198-44536198_A_C	164Q>P	Substitution	Nonsynonymous coding	36%
MM04T	ZNF224	zinc finger protein 224	CCDS33046.1	chr19_44612400-44612400_G_A	696R>Q	Substitution	Nonsynonymous coding	39%
MM04T	ZNF229	zinc finger protein 229	CCDS42574.1	chr19_44933626-44933626_C_T	444A>T	Substitution	Nonsynonymous coding	29%
MM04T	ZNF23	zinc finger protein 23	CCDS10900.1	chr16_71482273-71482273_C_A	552R>I	Substitution	Nonsynonymous coding	29%
MM04T	ZNF234	zinc finger protein 234	CCDS46101.1	chr19_44660632-44660632_A_C	155K>Q	Substitution	Nonsynonymous coding	37%
MM04T	ZNF234	zinc finger protein 234	CCDS46101.1	chr19_44661119-44661119_A_C	317K>T	Substitution	Nonsynonymous coding	31%
MM04T	ZNF236	zinc finger protein 236	CCDS42447.1	chr18_74561507-74561507_G_T	25E>D	Substitution	Nonsynonymous coding	22%
MM04T	ZNF236	zinc finger protein 236	CCDS42447.1	chr18_74635034-74635034_C_T	1187R>X	Substitution	Nonsense	28%
MM04T	ZNF236	zinc finger protein 236	CCDS42447.1	chr18_74639913-74639913_C_A	1480S>X	Substitution	Nonsense	29%
MM04T	ZNF248	zinc finger protein 248	CCDS7194.1	chr10_38121149-38121149_T_G	378K>N	Substitution	Nonsynonymous coding	29%
MM04T	ZNF248	zinc finger protein 248	CCDS7194.1	chr10_38121555-38121555_T_G	243K>T	Substitution	Nonsynonymous coding	33%

MM04T	ZNF257	zinc finger protein 257	CCDS46030.1	chr19_22271382-22271382_G_T	277R>I	Substitution	Nonsynonymous coding	27%
MM04T	ZNF26	zinc finger protein 26	CCDS31939.1	chr12_133586978-133586978_A_T	171E>D	Substitution	Nonsynonymous coding	27%
MM04T	ZNF264	zinc finger protein 264	CCDS33127.1	chr19_57723746-57723746_C_A	427F>L	Substitution	Nonsynonymous coding	38%
MM04T	ZNF267	zinc finger protein 267	CCDS32440.1	chr16_31927189-31927189_G_T	540R>I	Substitution	Nonsynonymous coding	33%
MM04T	ZNF268	zinc finger protein 268	CCDS9283.1	chr12_133732665-133732665_C_A	278S>Y	Substitution	Nonsynonymous coding	25%
MM04T	ZNF268	zinc finger protein 268	CCDS45012.1	chr12_133764497-133764497_A_T	25I>F	Substitution	Nonsynonymous coding	29%
MM04T	ZNF280B	zinc finger protein 280B	CCDS13799.1	chr22_22842434-22842434_C_A	430K>N	Substitution	Nonsynonymous coding	39%
MM04T	ZNF280B	zinc finger protein 280B	CCDS13799.1	chr22_22842893-22842893_C_A	277K>N	Substitution	Nonsynonymous coding	23%
MM04T	ZNF280B	zinc finger protein 280B	CCDS13799.1	chr22_22843648-22843648_C_T	26E>K	Substitution	Nonsynonymous coding	22%
MM04T	ZNF281	zinc finger protein 281	CCDS1402.1	chr1_200376556-200376556_T_C	760T>A	Substitution	Nonsynonymous coding	27%
MM04T	ZNF281	zinc finger protein 281	CCDS1402.1	chr1_200378085-200378085_T_C	250E>G	Substitution	Nonsynonymous coding	23%
MM04T	ZNF281	zinc finger protein 281	CCDS1402.1	chr1_200378380-200378380_G_C	152L>V	Substitution	Nonsynonymous coding	30%
MM04T	ZNF283	zinc finger protein 283	CCDS12497.1	chr19_37368220-37368220_G_A	163R>Q	Substitution	Nonsynonymous coding	39%
MM04T	ZNF283	zinc finger protein 283	CCDS46097.1	chr19_44351583-44351583_G_A	277S>N	Substitution	Nonsynonymous coding	40%
MM04T	ZNF283	zinc finger protein 283	CCDS46097.1	chr19_44352021-44352021_G_T	423R>I	Substitution	Nonsynonymous coding	27%
MM04T	ZNF284	zinc finger protein 284	CCDS46099.1	chr19_44591055-44591055_G_T	475R>I	Substitution	Nonsynonymous coding	21%
MM04T	ZNF292	zinc finger protein 292	CCDS47457.1	chr6_87968866-87968866_T_G	1840I>S	Substitution	Nonsynonymous coding	36%
MM04T	ZNF292	zinc finger protein 292	CCDS47457.1	chr6_87969864-87969864_C_T	2173R>X	Substitution	Nonsense	33%
MM04T	ZNF300	zinc finger protein 300	NM_052860	chr5_150274997-150274997_C_T	602V>I	Substitution	Nonsynonymous coding	23%
MM04T	ZNF318	zinc finger protein 318	CCDS4895.2	chr6_43323460-43323460_G_T	538L>I	Substitution	Nonsynonymous coding	21%
MM04T	ZNF324	zinc finger protein 324	CCDS12981.1	chr19_58981097-58981097_C_A	42L>I	Substitution	Nonsynonymous coding	29%
MM04T	ZNF329	zinc finger protein 329	CCDS12972.1	chr19_58640119-58640119_C_A	251R>I	Substitution	Nonsynonymous coding	35%
MM04T	ZNF334	zinc finger protein 334	CCDS33480.1	chr20_45130604-45130604_C_A	458K>N	Substitution	Nonsynonymous coding	13%
MM04T	ZNF34	zinc finger protein 34	CCDS47945.1	chr8_145999657-145999657_C_T	226R>H	Substitution	Nonsynonymous coding	21%
MM04T	ZNF354A	zinc finger protein 354A	CCDS4438.1	chr5_178139322-178139322_C_A	519E>D	Substitution	Nonsynonymous coding	25%
MM04T	ZNF354A	zinc finger protein 354A	CCDS4438.1	chr5_178139506-178139506_C_T	458R>Q	Substitution	Nonsynonymous coding	20%
MM04T	ZNF354B	zinc finger protein 354B	CCDS4439.1	chr5_178311162-178311162_G_T	570R>I	Substitution	Nonsynonymous coding	26%
MM04T	ZNF37A	zinc finger protein 37A	CCDS31183.1	chr10_38407009-38407009_G_T	310K>N	Substitution	Nonsynonymous coding	34%
MM04T	ZNF391	zinc finger protein 391	CCDS43429.1	chr6_27368787-27368787_G_T	213R>L	Substitution	Nonsynonymous coding	26%
MM04T	ZNF404	zinc finger protein 404	NM_001033719	chr19_44384224-44384224_G_A	22S>L	Substitution	Nonsynonymous coding	21%
MM04T	ZNF407	zinc finger protein 407	CCDS45885.1	chr18_72343443-72343443_A_C	156E>D	Substitution	Nonsynonymous coding	25%
MM04T	ZNF420	zinc finger protein 420	CCDS12498.1	chr19_37619629-37619629_G_A	579R>Q	Substitution	Nonsynonymous coding	36%

MM04T	ZNF420	zinc finger protein 420	CCDS12498.1	chr19_37619853-37619853_C_T	654R>C	Substitution	Nonsynonymous coding	28%
MM04T	ZNF420	zinc finger protein 420	CCDS12498.1	chr19_37619904-37619904_T_G	671F>V	Substitution	Nonsynonymous coding	30%
MM04T	ZNF420	zinc finger protein 420	CCDS12498.1	chr19_37619660-37619660_C_A	589C>X	Substitution	Nonsense	28%
MM04T	ZNF426	zinc finger protein 426	CCDS12215.1	chr19_9639711-9639711_A_G	337V>A	Substitution	Nonsynonymous coding	33%
MM04T	ZNF429	zinc finger protein 429	CCDS42537.1	chr19_21719517-21719517_G_T	221R>I	Substitution	Nonsynonymous coding	59%
MM04T	ZNF432	zinc finger protein 432	CCDS12848.1	chr19_52537754-52537754_C_T	393R>Q	Substitution	Nonsynonymous coding	46%
MM04T	ZNF44	zinc finger protein 44	ENST00000426973	chr19_12358183-12358183_C_T	511G>E	Substitution	Nonsynonymous coding	28%
MM04T	ZNF44	zinc finger protein 44	CCDS45988.1	chr19_12383841-12383841_C_A	410R>I	Substitution	Nonsynonymous coding	25%
MM04T	ZNF44	zinc finger protein 44	CCDS45988.1	chr19_12384168-12384168_C_A	301R>I	Substitution	Nonsynonymous coding	33%
MM04T	ZNF451	zinc finger protein 451	CCDS43477.1	chr6_57006862-57006862_C_T	325R>C	Substitution	Nonsynonymous coding	18%
MM04T	ZNF454	zinc finger protein 454	CCDS4441.1	chr5_178373962-178373962_G_T	75E>D	Substitution	Nonsynonymous coding	34%
MM04T	ZNF454	zinc finger protein 454	CCDS4441.1	chr5_178392612-178392612_C_T	403R>W	Substitution	Nonsynonymous coding	22%
MM04T	ZNF460	zinc finger protein 460	CCDS12949.1	chr19_57803422-57803422_G_A	505E>K	Substitution	Nonsynonymous coding	31%
MM04T	ZNF462	zinc finger protein 462	CCDS35096.1	chr9_109689356-109689356_G_A	1055E>K	Substitution	Nonsynonymous coding	23%
MM04T	ZNF462	zinc finger protein 462	CCDS35096.1	chr9_109691465-109691465_G_A	1758E>K	Substitution	Nonsynonymous coding	30%
MM04T	ZNF467	zinc finger protein 467	ENST00000378031	chr7_149461579-149461579_G_T	192F>L	Substitution	Nonsynonymous coding	17%
MM04T	ZNF467	zinc finger protein 467	ENST00000378031	chr7_149461611-149461611_C_T	182E>K	Substitution	Nonsynonymous coding	22%
MM04T	ZNF467	zinc finger protein 467	ENST00000378031	chr7_149461670-149461670_G_A	162P>L	Substitution	Nonsynonymous coding	30%
MM04T	ZNF470	zinc finger protein 470	CCDS33122.1	chr19_57088960-57088960_G_A	388R>Q	Substitution	Nonsynonymous coding	27%
MM04T	ZNF470	zinc finger protein 470	CCDS33122.1	chr19_57089047-57089047_G_T	417R>I	Substitution	Nonsynonymous coding	23%
MM04T	ZNF470	zinc finger protein 470	CCDS33122.1	chr19_57089229-57089229_G_T	478E>X	Substitution	Nonsense	28%
MM04T	ZNF473	zinc finger protein 473	CCDS33077.1	chr19_50549318-50549318_G_T	540E>X	Substitution	Nonsense	23%
MM04T	ZNF480	zinc finger protein 480	CCDS12850.2	chr19_52825759-52825759_G_T	419R>I	Substitution	Nonsynonymous coding	68%
MM04T	ZNF480	zinc finger protein 480	CCDS12850.2	chr19_52825859-52825859_G_T	452E>D	Substitution	Nonsynonymous coding	19%
MM04T	ZNF490	zinc finger protein 490	CCDS12272.1	chr19_12691588-12691588_C_T	434R>Q	Substitution	Nonsynonymous coding	29%
MM04T	ZNF490	zinc finger protein 490	CCDS12272.1	chr19_12692539-12692539_C_A	NA	Substitution	Splice site acceptor	29%
MM04T	ZNF496	zinc finger protein 496	CCDS1631.1	chr1_247492570-247492570_G_A	104A>V	Substitution	Nonsynonymous coding	20%
MM04T	ZNF497	zinc finger protein 497	CCDS12977.1	chr19_58868527-58868527_C_T	159E>K	Substitution	Nonsynonymous coding	23%
MM04T	ZNF510	zinc finger protein 510	CCDS35074.1	chr9_99522488-99522488_C_A	208E>D	Substitution	Nonsynonymous coding	22%
MM04T	ZNF510	zinc finger protein 510	CCDS35074.1	chr9_99525400-99525400_T_G	118K>Q	Substitution	Nonsynonymous coding	32%
MM04T	ZNF521	zinc finger protein 521	CCDS32806.1	chr18_22805376-22805376_C_T	836E>K	Substitution	Nonsynonymous coding	24%
MM04T	ZNF521	zinc finger protein 521	CCDS32806.1	chr18_22806432-22806432_C_T	484E>K	Substitution	Nonsynonymous coding	20%

MM04T	ZNF527	zinc finger protein 527	CCDS42559.1	chr19_37880084-37880084_G_T	378R>I	Substitution	Nonsynonymous coding	33%
MM04T	ZNF527	zinc finger protein 527	CCDS42559.1	chr19_37880641-37880641_C_A	564H>N	Substitution	Nonsynonymous coding	32%
MM04T	ZNF527	zinc finger protein 527	CCDS42559.1	chr19_37880756-37880756_G_T	602R>I	Substitution	Nonsynonymous coding	17%
MM04T	ZNF530	zinc finger protein 530	CCDS12955.1	chr19_58117197-58117197_G_T	102D>Y	Substitution	Nonsynonymous coding	30%
MM04T	ZNF541	zinc finger protein 541	CCDS46133.1	chr19_48043509-48043509_G_A	849T>M	Substitution	Nonsynonymous coding	38%
MM04T	ZNF541	zinc finger protein 541	CCDS46133.1	chr19_48052589-48052589_G_T	154S>Y	Substitution	Nonsynonymous coding	21%
MM04T	ZNF546	zinc finger protein 546	CCDS12548.1	chr19_40521024-40521024_A_C	616K>T	Substitution	Nonsynonymous coding	40%
MM04T	ZNF546	zinc finger protein 546	CCDS12548.1	chr19_40521348-40521348_A_C	724K>T	Substitution	Nonsynonymous coding	33%
MM04T	ZNF550	zinc finger protein 550	CCDS35500.1	chr19_58058953-58058953_C_T	179R>Q	Substitution	Nonsynonymous coding	24%
MM04T	ZNF560	zinc finger protein 560	CCDS12214.1	chr19_9577577-9577577_C_A	682K>N	Substitution	Nonsynonymous coding	24%
MM04T	ZNF560	zinc finger protein 560	CCDS12214.1	chr19_9578954-9578954_G_T	223F>L	Substitution	Nonsynonymous coding	22%
MM04T	ZNF564	zinc finger protein 564	CCDS42505.1	chr19_12639164-12639164_C_A	49D>Y	Substitution	Nonsynonymous coding	21%
MM04T	ZNF567	zinc finger protein 567	CCDS12495.1	chr19_37210694-37210694_G_T	325Q>H	Substitution	Nonsynonymous coding	24%
MM04T	ZNF577	zinc finger protein 577	CCDS12842.2	chr19_52375864-52375864_A_G	460I>T	Substitution	Nonsynonymous coding	29%
MM04T	ZNF585A	zinc finger protein 585A	CCDS12499.1	chr19_37643813-37643813_C_T	275E>K	Substitution	Nonsynonymous coding	23%
MM04T	ZNF585A	zinc finger protein 585A	CCDS12499.1	chr19_37644184-37644184_C_A	151R>I	Substitution	Nonsynonymous coding	25%
MM04T	ZNF585A	zinc finger protein 585A	CCDS12499.1	chr19_37644294-37644294_C_A	114Q>H	Substitution	Nonsynonymous coding	36%
MM04T	ZNF585B	zinc finger protein 585B	CCDS12500.1	chr19_37676966-37676966_C_A	491E>D	Substitution	Nonsynonymous coding	38%
MM04T	ZNF594	zinc finger protein 594	CCDS42241.1	chr17_5087028-5087028_C_A	175R>I	Substitution	Nonsynonymous coding	37%
MM04T	ZNF594	zinc finger protein 594	ENST00000381752	chr17_5084766-5084766_C_A	141E>X	Substitution	Nonsense	22%
MM04T	ZNF599	zinc finger protein 599	CCDS32991.1	chr19_35250055-35250055_T_C	551T>A	Substitution	Nonsynonymous coding	29%
MM04T	ZNF607	zinc finger protein 607	CCDS33006.1	chr19_38190577-38190577_C_T	152S>N	Substitution	Nonsynonymous coding	28%
MM04T	ZNF608	zinc finger protein 608	CCDS34219.1	chr5_123983198-123983198_G_T	960S>X	Substitution	Nonsense	37%
MM04T	ZNF610	zinc finger protein 610	CCDS12851.1	chr19_52869129-52869129_A_C	166K>N	Substitution	Nonsynonymous coding	44%
MM04T	ZNF613	zinc finger protein 613	CCDS33089.1	chr19_52447399-52447399_A_C	88Q>P	Substitution	Nonsynonymous coding	32%
MM04T	ZNF614	zinc finger protein 614	CCDS12847.1	chr19_52520442-52520442_A_C	137L>V	Substitution	Nonsynonymous coding	40%
MM04T	ZNF615	zinc finger protein 615	CCDS12846.1	chr19_52497222-52497222_C_A	369E>D	Substitution	Nonsynonymous coding	21%
MM04T	ZNF616	zinc finger protein 616	CCDS33090.1	chr19_52618611-52618611_C_A	602E>D	Substitution	Nonsynonymous coding	29%
MM04T	ZNF616	zinc finger protein 616	CCDS33090.1	chr19_52619215-52619215_C_T	401R>Q	Substitution	Nonsynonymous coding	34%
MM04T	ZNF641	zinc finger protein 641	CCDS8763.1	chr12_48737297-48737297_G_T	259S>Y	Substitution	Nonsynonymous coding	20%
MM04T	ZNF641	zinc finger protein 641	CCDS8763.1	chr12_48739220-48739220_G_T	119S>Y	Substitution	Nonsynonymous coding	22%
MM04T	ZNF644	zinc finger protein 644	CCDS731.1	chr1_91404144-91404144_C_T	923D>N	Substitution	Nonsynonymous coding	23%

MM04T	ZNF644	zinc finger protein 644	CCDS731.1	chr1_91404171-91404171_C_T	914D>N	Substitution	Nonsynonymous coding	24%
MM04T	ZNF644	zinc finger protein 644	CCDS731.1	chr1_91405467-91405467_G_A	482R>C	Substitution	Nonsynonymous coding	28%
MM04T	ZNF645	zinc finger protein 645	CCDS14205.1	chrX_22291313-22291313_C_T	69R>X	Substitution	Nonsense	33%
MM04T	ZNF649	zinc finger protein 649	CCDS12843.1	chr19_52394392-52394392_G_A	333L>F	Substitution	Nonsynonymous coding	25%
MM04T	ZNF655	zinc finger protein 655	CCDS47655.1	chr7_99170501-99170501_G_A	292R>Q	Substitution	Nonsynonymous coding	30%
MM04T	ZNF660	zinc finger protein 660	CCDS2716.1	chr3_44635761-44635761_G_T	26E>X	Substitution	Nonsense	37%
MM04T	ZNF662	zinc finger protein 662	CCDS46807.1	chr3_42956007-42956007_G_A	174E>K	Substitution	Nonsynonymous coding	30%
MM04T	ZNF664	zinc finger protein 664	CCDS9257.1	chr12_124496915-124496915_A_C	75N>T	Substitution	Nonsynonymous coding	39%
MM04T	ZNF670	zinc finger protein 670	CCDS31087.1	chr1_247201284-247201284_G_A	213R>C	Substitution	Nonsynonymous coding	31%
MM04T	ZNF671	zinc finger protein 671	CCDS12961.1	chr19_58232730-58232730_T_C	242T>A	Substitution	Nonsynonymous coding	38%
MM04T	ZNF674	zinc finger protein 674	CCDS48099.1	chrX_46359490-46359490_G_T	512L>I	Substitution	Nonsynonymous coding	23%
MM04T	ZNF675	zinc finger protein 675	CCDS32981.1	chr19_23836070-23836070_T_G	555K>N	Substitution	Nonsynonymous coding	26%
MM04T	ZNF677	zinc finger protein 677	CCDS12861.1	chr19_53741551-53741551_G_T	143F>L	Substitution	Nonsynonymous coding	42%
MM04T	ZNF680	zinc finger protein 680	CCDS34644.1	chr7_63981755-63981755_C_A	459E>D	Substitution	Nonsynonymous coding	39%
MM04T	ZNF684	zinc finger protein 684	CCDS454.1	chr1_41013103-41013103_C_A	370L>I	Substitution	Nonsynonymous coding	42%
MM04T	ZNF7	zinc finger protein 7	CCDS6435.1	chr8_146067431-146067431_A_C	313K>N	Substitution	Nonsynonymous coding	31%
MM04T	ZNF7	zinc finger protein 7	CCDS6435.1	chr8_146068538-146068538_A_C	682K>N	Substitution	Nonsynonymous coding	22%
MM04T	ZNF709	zinc finger protein 709	CCDS42504.1	chr19_12577292-12577292_G_T	58Q>K	Substitution	Nonsynonymous coding	24%
MM04T	ZNF709	zinc finger protein 709	CCDS42504.1	chr19_12576018-12576018_G_A	240R>X	Substitution	Nonsense	31%
MM04T	ZNF711	zinc finger protein 711	CCDS35344.1	chrX_84525909-84525909_G_T	454R>I	Substitution	Nonsynonymous coding	47%
MM04T	ZNF711	zinc finger protein 711	CCDS35344.1	chrX_84525917-84525917_G_A	457E>K	Substitution	Nonsynonymous coding	19%
MM04T	ZNF713	zinc finger protein 713	CCDS34639.1	chr7_56007670-56007670_C_T	422R>C	Substitution	Nonsynonymous coding	30%
MM04T	ZNF718	zinc finger protein 718	NM_001039127	chr4_155047-155047_G_T	191R>I	Substitution	Nonsynonymous coding	24%
MM04T	ZNF729	zinc finger protein 729	ENST00000357491	chr19_22496538-22496538_G_T	107V>L	Substitution	Nonsynonymous coding	21%
MM04T	ZNF730	zinc finger protein 730	ENST00000327867	chr19_23328173-23328173_G_T	109E>D	Substitution	Nonsynonymous coding	31%
MM04T	ZNF730	zinc finger protein 730	ENST00000327867	chr19_23328594-23328594_C_A	250H>N	Substitution	Nonsynonymous coding	19%
MM04T	ZNF746	zinc finger protein 746	CCDS5897.1	chr7_149189981-149189981_C_A	163K>N	Substitution	Nonsynonymous coding	30%
MM04T	ZNF76	zinc finger protein 76	CCDS4801.1	chr6_35259095-35259095_G_T	222E>D	Substitution	Nonsynonymous coding	35%
MM04T	ZNF767	zinc finger family member 767	ENST00000335215	chr7_149318543-149318543_C_A	44K>N	Substitution	Nonsynonymous coding	16%
MM04T	ZNF770	zinc finger protein 770	CCDS10042.1	chr15_35273570-35273570_G_A	689S>L	Substitution	Nonsynonymous coding	44%
MM04T	ZNF780B	zinc finger protein 780B	ENST00000388801	chr19_40539612-40539612_T_C	582K>R	Substitution	Nonsynonymous coding	36%
MM04T	ZNF781	zinc finger protein 781	CCDS12507.1	chr19_38160195-38160195_C_A	285M>I	Substitution	Nonsynonymous coding	23%

MM04T	ZNF781	zinc finger protein 781	CCDS12507.1	chr19_38160641-38160641_T_C	137K>E	Substitution	Nonsynonymous coding	35%
MM04T	ZNF786	zinc finger protein 786	CCDS47738.1	chr7_148769515-148769515_C_T	117D>N	Substitution	Nonsynonymous coding	25%
MM04T	ZNF788	zinc finger family member 788	ENST00000339302	chr19_12222997-12222997_G_A	212R>Q	Substitution	Nonsynonymous coding	24%
MM04T	ZNF790	zinc finger protein 790	CCDS12496.1	chr19_37310425-37310425_C_T	274R>Q	Substitution	Nonsynonymous coding	46%
MM04T	ZNF791	zinc finger protein 791	CCDS12273.1	chr19_12739148-12739148_G_T	269D>Y	Substitution	Nonsynonymous coding	28%
MM04T	ZNF791	zinc finger protein 791	CCDS12273.1	chr19_12739709-12739709_C_T	456R>X	Substitution	Nonsense	28%
MM04T	ZNF804A	zinc finger protein 804A	CCDS2291.1	chr2_185801081-185801081_T_C	320S>P	Substitution	Nonsynonymous coding	27%
MM04T	ZNF804A	zinc finger protein 804A	CCDS2291.1	chr2_185802954-185802954_A_C	944N>T	Substitution	Nonsynonymous coding	33%
MM04T	ZNF804B	zinc finger protein 804B	CCDS5613.1	chr7_88965709-88965709_C_A	1138S>Y	Substitution	Nonsynonymous coding	26%
MM04T	ZNF804B	zinc finger protein 804B	CCDS5613.1	chr7_88962858-88962858_C_T	188R>X	Substitution	Nonsense	25%
MM04T	ZNF823	zinc finger protein 823	CCDS45981.1	chr19_11833762-11833762_A_G	196L>S	Substitution	Nonsynonymous coding	26%
MM04T	ZNF823	zinc finger protein 823	CCDS45981.1	chr19_11833810-11833810_C_T	180R>Q	Substitution	Nonsynonymous coding	23%
MM04T	ZNF823	zinc finger protein 823	CCDS45981.1	chr19_11833391-11833391_G_A	320R>X	Substitution	Nonsense	36%
MM04T	ZNF827	zinc finger protein 827	CCDS34072.1	chr4_146791459-146791459_C_T	640G>E	Substitution	Nonsynonymous coding	29%
MM04T	ZNF83	zinc finger protein 83	CCDS12854.1	chr19_53117566-53117566_C_A	84E>D	Substitution	Nonsynonymous coding	30%
MM04T	ZNF831	zinc finger protein 831	CCDS42894.1	chr20_57829431-57829431_C_T	1556S>L	Substitution	Nonsynonymous coding	37%
MM04T	ZNF836	zinc finger protein 836	CCDS46162.1	chr19_52659551-52659551_C_A	462R>I	Substitution	Nonsynonymous coding	25%
MM04T	ZNF84	zinc finger protein 84	CCDS31940.1	chr12_133635409-133635409_G_T	703R>I	Substitution	Nonsynonymous coding	34%
MM04T	ZNF841	zinc finger protein 841	CCDS46161.1	chr19_52570041-52570041_C_A	365R>I	Substitution	Nonsynonymous coding	35%
MM04T	ZNF844	zinc finger protein 844	CCDS45985.1	chr19_12187353-12187353_G_T	473R>I	Substitution	Nonsynonymous coding	15%
MM04T	ZNF852	zinc finger protein 852	ENST00000436261	chr3_44541145-44541145_C_T	375R>Q	Substitution	Nonsynonymous coding	23%
MM04T	ZNF883	zinc finger protein 883	NM_001101338	chr9_115760310-115760310_G_A	77A>V	Substitution	Nonsynonymous coding	34%
MM04T	ZNF91	zinc finger protein 91	CCDS42541.1	chr19_23545244-23545244_C_A	179K>N	Substitution	Nonsynonymous coding	46%
MM04T	ZNF92	zinc finger protein 92	CCDS34646.1	chr7_64863354-64863354_G_T	109E>D	Substitution	Nonsynonymous coding	27%
MM04T	ZNFX1	zinc finger, NFX1-type containing 1	CCDS13417.1	chr20_47864358-47864358_C_A	1735E>X	Substitution	Nonsense	33%
MM04T	ZNHIT3	zinc finger, HIT-type containing 3	CCDS11312.1	chr17_34842547-34842547_G_A	2A>T	Substitution	Nonsynonymous coding	18%
MM04T	ZNRF2	zinc and ring finger 2	CCDS5426.1	chr7_30402015-30402015_G_T	232V>L	Substitution	Nonsynonymous coding	17%
MM04T	ZNRF4	zinc and ring finger 4	CCDS42475.1	chr19_5455788-5455788_G_A	96A>T	Substitution	Nonsynonymous coding	16%
MM04T	ZP1	zona pellucida glycoprotein 1 (sperm receptor)	CCDS31572.1	chr11_60637374-60637374_G_A	NA	Substitution	Splice site donor	35%
MM04T	ZP2	zona pellucida glycoprotein 2 (sperm receptor)	CCDS10596.1	chr16_21215415-21215415_C_A	303G>V	Substitution	Nonsynonymous coding	21%
MM04T	ZP4	zona pellucida glycoprotein 4	CCDS1615.1	chr1_238053227-238053227_C_T	114A>T	Substitution	Nonsynonymous coding	38%
MM04T	ZPBP	zona pellucida binding protein	CCDS5509.1	chr7_50023098-50023098_C_A	267E>D	Substitution	Nonsynonymous coding	38%

MM04T	ZRANB3	zinc finger, RAN-binding domain containing 3	CCDS46419.1	chr2_135985423-135985423_T_G	706K>T	Substitution	Nonsynonymous coding	42%
MM04T	ZRANB3	zinc finger, RAN-binding domain containing 3	CCDS46419.1	chr2_136111090-136111090_C_A	97E>X	Substitution	Nonsense	28%
MM04T	ZSCAN20	zinc finger and SCAN domain containing 20	CCDS41300.1	chr1_33954169-33954169_G_T	174Q>H	Substitution	Nonsynonymous coding	14%
MM04T	ZSCAN20	zinc finger and SCAN domain containing 20	CCDS41300.1	chr1_33960020-33960020_G_T	692E>D	Substitution	Nonsynonymous coding	16%
MM04T	ZSCAN30	zinc finger and SCAN domain containing 30	CCDS42427.1	chr18_32833517-32833517_C_A	461R>I	Substitution	Nonsynonymous coding	26%
MM04T	ZSCAN4	zinc finger and SCAN domain containing 4	CCDS12958.1	chr19_58187767-58187767_C_A	85S>Y	Substitution	Nonsynonymous coding	38%
MM04T	ZSWIM2	zinc finger, SWIM-type containing 2	CCDS33348.1	chr2_187703837-187703837_G_A	115R>X	Substitution	Nonsense	31%
MM04T	ZSWIM6	zinc finger, SWIM-type containing 6	CCDS47215.1	chr5_60835431-60835431_C_T	850R>W	Substitution	Nonsynonymous coding	30%
MM04T	ZUFSP	zinc finger with UFM1-specific peptidase domain	CCDS5110.1	chr6_116967088-116967088_C_T	493R>Q	Substitution	Nonsynonymous coding	46%
MM04T	ZXDC	ZXD family zinc finger C	CCDS43145.1	chr3_126191110-126191110_A_G	316S>P	Substitution	Nonsynonymous coding	29%
MM04T	ZYG11A	zyg-11 family member A, cell cycle regulator	CCDS44148.1	chr1_53329764-53329764_C_T	421R>C	Substitution	Nonsynonymous coding	23%
MM04T	ZZZ3	zinc finger, ZZ-type containing 3	CCDS677.1	chr1_78046723-78046723_T_G	647K>T	Substitution	Nonsynonymous coding	33%
MM04T	ZZZ3	zinc finger, ZZ-type containing 3	CCDS677.1	chr1_78097945-78097945_C_A	365M>I	Substitution	Nonsynonymous coding	28%
MM05T	ABCA7	ATP-binding cassette, sub-family A (ABC1), member 7	CCDS12055.1	chr19_1047226-1047226_C_T	639T>M	Substitution	Nonsynonymous coding	23%
MM05T	ABCB1	ATP-binding cassette, sub-family B (MDR/TAP), member 1	CCDS5608.1	chr7_87148690-87148690_C_T	960G>E	Substitution	Nonsynonymous coding	14%
MM05T	ANKIB1	ankyrin repeat and IBR domain containing 1	CCDS47639.1	chr7_92027526-92027526_G_T	845A>S	Substitution	Nonsynonymous coding	10%
MM05T	ARHGAP20	Rho GTPase activating protein 20	CCDS31673.1	chr11_110450636-110450636_G_A	1012R>C	Substitution	Nonsynonymous coding	22%
MM05T	ARID1A	AT rich interactive domain 1A (SWI-like)	CCDS285.1	chr1_27059260-27059261_CA_	NA	Deletion	Frameshift	42%
MM05T	ARID1B	AT rich interactive domain 1B (SWI1-like)	CCDS5251.1	chr6_157527324-157527324_A_	NA	Deletion	Frameshift	21%
MM05T	ARID1B	AT rich interactive domain 1B (SWI1-like)	CCDS5251.1	chr6_157511262-157511262_C_A	1242Y>X	Substitution	Nonsense	15%
MM05T	ASXL2	additional sex combs like 2 (Drosophila)	NM_018263	chr2_25965531-25965531_G_C	1225C>W	Substitution	Nonsynonymous coding	20%
MM05T	AXIN1	axin 1	CCDS10405.1	chr16_396257-396257_C_T	257D>N	Substitution	Nonsynonymous coding	21%
MM05T	AZIN1	antizyme inhibitor 1	CCDS6295.1	chr8_103851964-103851964_G_A	64P>S	Substitution	Nonsynonymous coding	11%
MM05T	C1orf147	chromosome 1 open reading frame 147	ENST00000367119	chr1_206666268-206666268_C_T	89G>E	Substitution	Nonsynonymous coding	22%
MM05T	CASC5	cancer susceptibility candidate 5	CCDS42023.1	chr15_40916868-40916868_A_C	1495K>T	Substitution	Nonsynonymous coding	16%
MM05T	DCP2	DCP2 decapping enzyme homolog (S. cerevisiae)	CCDS34210.1	chr5_112337135-112337135_A_G	223N>D	Substitution	Nonsynonymous coding	22%
MM05T	DGAT2L7P	diacylglycerol O-acyltransferase 2-like 7, pseudogene	ENST00000379421	chr7_100845569-100845569_G_T	138L>I	Substitution	Nonsynonymous coding	19%
MM05T	DGKI	diacylglycerol kinase, iota	CCDS5845.1	chr7_137374705-137374705_G_T	149H>N	Substitution	Nonsynonymous coding	27%
MM05T	DNM1P46	DNM1 pseudogene 46	ENST00000423248	chr15_100332842-100332842__T	NA	Insertion	Frameshift	12%
MM05T	FSTL5	follistatin-like 5	CCDS3802.1	chr4_163032431-163032431_G_A	40R>X	Substitution	Nonsense	18%
MM05T	GYG2	glycogenin 2	CCDS14121.1	chrX_2773052-2773052_G_T	146E>X	Substitution	Nonsense	22%
MM05T	HEXA	hexosaminidase A (alpha polypeptide)	CCDS10243.1	chr15_72640388-72640388_C_T	NA	Substitution	Splice site donor	14%

MM05T	HRNR	hornerin	CCDS30859.1	chr1_152191074-152191074_G_A	1011R>C	Substitution	Nonsynonymous coding	24%
MM05T	IGLV5-48	immunoglobulin lambda variable 5-48 (non-functional)	ENST00000390293	chr22_22707728-22707728_C_T	106Q>X	Substitution	Nonsense	22%
MM05T	ITGA9	integrin, alpha 9	CCDS2669.1	chr3_37555284-37555284_G_A	310A>T	Substitution	Nonsynonymous coding	18%
MM05T	JAK1	Janus kinase 1	CCDS41346.1	chr1_65312421-65312421_T_G	NA	Substitution	Splice site acceptor	25%
MM05T	KCNQ2	potassium voltage-gated channel, KQT-like subfamily, member 2	ENST00000370222	chr20_62044647-62044647_T_C	612Q>R	Substitution	Nonsynonymous coding	27%
MM05T	KRAS	v-Ki-ras2 Kirsten rat sarcoma viral oncogene homolog	CCDS8703.1	chr12_25398284-25398284_C_A	12G>V	Substitution	Nonsynonymous coding	14%
MM05T	LRRC10	leucine rich repeat containing 10	CCDS31856.1	chr12_70003915-70003915_C_T	235R>H	Substitution	Nonsynonymous coding	17%
MM05T	MBNL2	muscleblind-like splicing regulator 2	CCDS9484.1	chr13_97986632-97986632_G_A	93A>T	Substitution	Nonsynonymous coding	14%
MM05T	NSUN2	NOP2/Sun RNA methyltransferase family, member 2	CCDS3869.1	chr5_6609961-6609961_G_A	434P>L	Substitution	Nonsynonymous coding	19%
MM05T	NUP153	nucleoporin 153kDa	CCDS4541.1	chr6_17675809-17675809_T_C	176H>R	Substitution	Nonsynonymous coding	20%
MM05T	PIK3CA	phosphatidylinositol-4,5-bisphosphate 3-kinase, catalytic subunit alpha	CCDS43171.1	chr3_178936091-178936091_G_A	545E>K	Substitution	Nonsynonymous coding	13%
MM05T	RASSF4	Ras association (RalGDS/AF-6) domain family member 4	ENST00000334940	chr10_45479390-45479390_G_A	77G>S	Substitution	Nonsynonymous coding	20%
MM05T	RGS20	regulator of G-protein signaling 20	CCDS6155.1	chr8_54866739-54866739_G_T	283E>X	Substitution	Nonsense	27%
MM05T	SLC5A3	solute carrier family 5 (sodium/myo-inositol cotransporter), member 3	CCDS33549.1	chr21_35469455-35469455_G_A	653R>Q	Substitution	Nonsynonymous coding	23%
MM05T	SLC5A4	solute carrier family 5 (low affinity glucose cotransporter), member 4	CCDS13903.1	chr22_32643472-32643472_G_A	135R>W	Substitution	Nonsynonymous coding	18%
MM05T	SLC7A14	solute carrier family 7 (orphan transporter), member 14	CCDS33892.1	chr3_170201305-170201305_C_T	305V>M	Substitution	Nonsynonymous coding	18%
MM05T	TAS2R39	taste receptor, type 2, member 39	CCDS47729.1	chr7_142881325-142881325_A_C	272I>L	Substitution	Nonsynonymous coding	28%
MM05T	THOC3	THO complex 3	CCDS4397.1	chr5_175395058-175395058_G_	NA	Deletion	Frameshift	13%
MM05T	WDR45B	WD repeat domain 45B	CCDS11815.2	chr17_80585167-80585170_ACTG_	NA	Deletion	Splice site acceptor	20%
MM05T	ZFYVE26	zinc finger, FYVE domain containing 26	CCDS9788.1	chr14_68229394-68229394_C_T	2052V>I	Substitution	Nonsynonymous coding	27%
MM05T	ZMIZ2	zinc finger, MIZ-type containing 2	CCDS43576.1	chr7_44798880-44798880_G_A	272V>M	Substitution	Nonsynonymous coding	20%
MM06T	ADAMTS10	ADAM metallopeptidase with thrombospondin type 1 motif, 10	CCDS12206.1	chr19_8670129-8670129_G_A	68T>M	Substitution	Nonsynonymous coding	17%
MM06T	ARHGAP32	Rho GTPase activating protein 32	CCDS44769.1	chr11_128840478-128840478_T_C	1530M>V	Substitution	Nonsynonymous coding	34%
MM06T	ARID1A	AT rich interactive domain 1A (SWI-like)	CCDS285.1	chr1_27023560-27023560_C_	NA	Deletion	Frameshift	26%
MM06T	BCL2L11	BCL2-like 11 (apoptosis facilitator)	CCDS2089.1	chr2_111907671-111907671__G	NA	Insertion	Frameshift	27%
MM06T	DNAH6	dynein, axonemal, heavy chain 6	CCDS46348.1	chr2_84756035-84756035_G_A	136R>Q	Substitution	Nonsynonymous coding	55%
MM06T	EGFR	epidermal growth factor receptor	CCDS5515.1	chr7_55238153-55238153_G_	NA	Deletion	Frameshift	17%
MM06T	FBXW7	F-box and WD repeat domain containing 7, E3 ubiquitin protein ligase	CCDS3777.1	chr4_153247294-153247294_G_A	503A>V	Substitution	Nonsynonymous coding	26%
MM06T	HMCN1	hemicentin 1	CCDS30956.1	chr1_186057077-186057077_G_T	3126G>V	Substitution	Nonsynonymous coding	40%
MM06T	HYDIN	HYDIN, axonemal central pair apparatus protein	CCDS42189.1	chr16_70900066-70900069_ACTC_	NA	Deletion	Splice site donor	20%
MM06T	KCNJ12	potassium inwardly-rectifying channel, subfamily J, member 12	CCDS11219.1	chr17_21319651-21319653_GAG_	NA	Deletion	In-frame deletion	14%
MM06T	KIAA0195	uncharacterized protein	CCDS32732.1	chr17_73485372-73485372_C_T	264R>X	Substitution	Nonsense	16%

MM06T	LAX1	lymphocyte transmembrane adaptor 1	CCDS1441.2	chr1_203743084-203743084_G_A	158A>T	Substitution	Nonsynonymous coding	19%
MM06T	LRRC7	leucine rich repeat containing 7	CCDS645.1	chr1_70504419-70504419_C_	NA	Deletion	Frameshift	26%
MM06T	LRRTM2	leucine rich repeat transmembrane neuronal 2	CCDS47272.1	chr5_138209522-138209522_T_C	243N>S	Substitution	Nonsynonymous coding	11%
MM06T	MEP1B	meprin A, beta	CCDS45846.1	chr18_29797032-29797032_G_A	613C>Y	Substitution	Nonsynonymous coding	28%
MM06T	NSD1	nuclear receptor binding SET domain protein 1	CCDS4412.1	chr5_176562442-176562442_G_T	113C>F	Substitution	Nonsynonymous coding	27%
MM06T	OGFRL1	opioid growth factor receptor-like 1	CCDS34482.1	chr6_72006438-72006438__T	NA	Insertion	Frameshift	18%
MM06T	PPP1R13L	protein phosphatase 1, regulatory subunit 13 like	CCDS33050.1	chr19_45899997-45899997_G_A	173T>M	Substitution	Nonsynonymous coding	13%
MM06T	RING1	ring finger protein 1	CCDS34424.1	chr6_33179576-33179576_G_C	306E>Q	Substitution	Nonsynonymous coding	13%
MM06T	RPP25	ribonuclease P/MRP 25kDa subunit	CCDS10274.1	chr15_75248695-75248695_G_C	77T>S	Substitution	Nonsynonymous coding	29%
MM06T	SETD1A	SET domain containing 1A	CCDS32435.1	chr16_30972664-30972664_C_A	108T>N	Substitution	Nonsynonymous coding	10%
MM06T	SLC51A	solute carrier family 51, alpha subunit	CCDS3314.1	chr3_195955038-195955038__G	NA	Insertion	Frameshift	12%
MM06T	SPHK2	sphingosine kinase 2	CCDS12727.1	chr19_49131521-49131521_A_G	287N>D	Substitution	Nonsynonymous coding	11%
MM06T	SPINT1	serine peptidase inhibitor, Kunitz type 1	CCDS10067.1	chr15_41136925-41136925_T_A	58V>E	Substitution	Nonsynonymous coding	21%
MM06T	UBAP2L	ubiquitin associated protein 2-like	CCDS1063.1	chr1_154227337-154227337_C_T	627T>M	Substitution	Nonsynonymous coding	10%
MM06T	ZNF521	zinc finger protein 521	CCDS32806.1	chr18_22804538-22804538_C_T	1115R>K	Substitution	Nonsynonymous coding	20%
MM08T	ANO2	anoctamin 2	CCDS44807.1	chr12_6030411-6030411_C_T	106R>H	Substitution	Nonsynonymous coding	25%
MM08T	ASXL3	additional sex combs like 3 (Drosophila)	CCDS45847.1	chr18_31324343-31324343_G_A	1511V>I	Substitution	Nonsynonymous coding	29%
MM08T	CASKIN2	CASK interacting protein 2	CCDS11723.1	chr17_73499255-73499255_C_T	634E>K	Substitution	Nonsynonymous coding	15%
MM08T	CCNT2	cyclin T2	CCDS2174.1	chr2_135677462-135677462_G_T	NA	Substitution	Splice site donor	40%
MM08T	CDC42BPB	CDC42 binding protein kinase beta (DMPK-like)	CCDS9978.1	chr14_103430882-103430882_C_T	895E>K	Substitution	Nonsynonymous coding	20%
MM08T	COPZ1	coatomer protein complex, subunit zeta 1	CCDS8877.1	chr12_54743423-54743423_C_T	156T>M	Substitution	Nonsynonymous coding	17%
MM08T	CYP2W1	cytochrome P450, family 2, subfamily W, polypeptide 1	CCDS5319.2	chr7_1022905-1022905_G_A	20A>T	Substitution	Nonsynonymous coding	31%
MM08T	DCLRE1C	DNA cross-link repair 1C	CCDS31149.1	chr10_14961793-14961793_G_C	367P>R	Substitution	Nonsynonymous coding	19%
MM08T	DHTKD1	dehydrogenase E1 and transketolase domain containing 1	CCDS7087.1	chr10_12129595-12129595_C_A	195A>D	Substitution	Nonsynonymous coding	20%
MM08T	DOCK7	dedicator of cytokinesis 7	CCDS30734.1	chr1_62970446-62970446_T_A	1509E>V	Substitution	Nonsynonymous coding	18%
MM08T	DSEL	dermatan sulfate epimerase-like	CCDS11995.1	chr18_65180719-65180719_G_T	386P>H	Substitution	Nonsynonymous coding	37%
MM08T	FAM167B	family with sequence similarity 167, member B	CCDS358.2	chr1_32714006-32714006_G_C	99G>A	Substitution	Nonsynonymous coding	67%
MM08T	ING4	inhibitor of growth family, member 4	CCDS44813.1	chr12_6761835-6761835_C_T	165V>M	Substitution	Nonsynonymous coding	18%
MM08T	ITPRIPL1	inositol 1,4,5-trisphosphate receptor interacting protein-like 1	CCDS33250.1	chr2_96992429-96992429_G_A	28M>I	Substitution	Nonsynonymous coding	24%
MM08T	LACTBL1	lactamase, beta-like 1	ENST00000401081	chr1_23285322-23285322_G_A	108R>W	Substitution	Nonsynonymous coding	27%
MM08T	LAMA3	laminin, alpha 3	CCDS42419.1	chr18_21396442-21396442_T_G	713V>G	Substitution	Nonsynonymous coding	32%
MM08T	LCE3A	late cornified envelope 3A	CCDS1017.1	chr1_152595351-152595351_C_T	77G>R	Substitution	Nonsynonymous coding	26%

MM08T	MARK4	MAP/microtubule affinity-regulating kinase 4	CCDS12658.1	chr19_45783851-45783851_G_A	379A>T	Substitution	Nonsynonymous coding	10%
MM08T	MUSK	muscle, skeletal, receptor tyrosine kinase	CCDS48005.1	chr9_113549969-113549969_G_A	NA	Substitution	Splice site acceptor	50%
MM08T	MVP	major vault protein	CCDS10656.1	chr16_29851667-29851667_C_T	360R>C	Substitution	Nonsynonymous coding	24%
MM08T	NID1	nidogen 1	CCDS1608.1	chr1_236176788-236176788_T_C	776Q>R	Substitution	Nonsynonymous coding	27%
MM08T	PCDHA1	protocadherin alpha 1	CCDS34246.1	chr5_140166698-140166698_G_A	275G>S	Substitution	Nonsynonymous coding	25%
MM08T	PIK3R1	phosphoinositide-3-kinase, regulatory subunit 1 (alpha)	CCDS3993.1	chr5_67591116-67591116__TTA	570L>LI	Insertion	In-frame insertion	27%
MM08T	PIK3R5	phosphoinositide-3-kinase, regulatory subunit 5	CCDS11147.1	chr17_8791741-8791741_G_A	455R>W	Substitution	Nonsynonymous coding	60%
MM08T	PLEKHG3	pleckstrin homology domain containing, family G; member 3	CCDS32098.1	chr14_65207884-65207884_G_A	494G>D	Substitution	Nonsynonymous coding	21%
MM08T	PROX2	prospero homeobox 2	CCDS45136.1	chr14_75329616-75329616_G_A	308R>C	Substitution	Nonsynonymous coding	26%
MM08T	PTEN	phosphatase and tensin homolog	NM_000314	chr10_89624263-89624263_A_G	13K>E	Substitution	Nonsynonymous coding	30%
MM08T	PXDNL	peroxidasin homolog (Drosophila)-like	CCDS47855.1	chr8_52336137-52336137_G_A	598T>M	Substitution	Nonsynonymous coding	25%
MM08T	SCNN1B	sodium channel, non-voltage-gated 1, beta subunit	CCDS10609.1	chr16_23364399-23364399_A_C	NA	Substitution	Splice site donor	26%
MM08T	SERPINB7	serpin peptidase inhibitor, clade B (ovalbumin), member 7	CCDS11988.1	chr18_61471670-61471670_G_A	315R>H	Substitution	Nonsynonymous coding	43%
MM08T	SORCS1	sortilin-related VPS10 domain containing receptor 1	CCDS31283.1	chr10_108924224-108924224_C_T	21A>T	Substitution	Nonsynonymous coding	30%
MM08T	TP53	tumor protein p53	CCDS11118.1	chr17_7578212-7578212_G_A	213R>X	Substitution	Nonsense	56%
MM08T	UBA7	ubiquitin-like modifier activating enzyme 7	CCDS2805.1	chr3_49846592-49846592_C_A	722D>Y	Substitution	Nonsynonymous coding	33%
MM08T	UNKL	unkempt homolog (Drosophila)-like	CCDS32359.1	chr16_1453320-1453320_C_T	105G>R	Substitution	Nonsynonymous coding	22%
MM08T	WDR67	WD repeat domain 67	CCDS6338.1	chr8_124138303-124138303_G_A	526W>X	Substitution	Nonsense	47%
MM08T	ZC4H2	zinc finger, C4H2 domain containing	CCDS14380.1	chrX_64141723-64141723_G_A	67R>X	Substitution	Nonsense	12%
MM08T	ZFP28	ZFP28 zinc finger protein	CCDS12946.1	chr19_57061469-57061469_T_A	241F>I	Substitution	Nonsynonymous coding	22%
MM09T	AOC2	amine oxidase, copper containing 2 (retina-specific)	CCDS11443.1	chr17_40997271-40997271_C_T	210R>W	Substitution	Nonsynonymous coding	21%
MM09T	APOB	apolipoprotein B (including Ag(x) antigen)	CCDS1703.1	chr2_21237353-21237353_T_C	1270D>G	Substitution	Nonsynonymous coding	21%
MM09T	ASAP3	ArfGAP with SH3 domain, ankyrin repeat and PH domain 3	CCDS235.1	chr1_23763782-23763782_C_T	395E>K	Substitution	Nonsynonymous coding	19%
MM09T	BRD9	bromodomain containing 9	CCDS34127.1	chr5_878576-878576_C_T	293A>T	Substitution	Nonsynonymous coding	16%
MM09T	C6	complement component 6	CCDS3936.1	chr5_41154097-41154097_G_A	702T>M	Substitution	Nonsynonymous coding	23%
MM09T	CAD	carbamoyl-phosphate synthetase 2, aspartate transcarbamylase, and dihydroorotase	CCDS1742.1	chr2_27440438-27440438_G_C	7E>Q	Substitution	Nonsynonymous coding	27%
MM09T	CFH	complement factor H	CCDS1385.1	chr1_196696029-196696029_C_T	732T>M	Substitution	Nonsynonymous coding	14%
MM09T	DOK2	docking protein 2, 56kDa	CCDS6016.1	chr8_21767240-21767240_G_C	274P>R	Substitution	Nonsynonymous coding	28%
MM09T	ERAP2	endoplasmic reticulum aminopeptidase 2	CCDS4086.1	chr5_96228110-96228110_G_A	360D>N	Substitution	Nonsynonymous coding	19%
MM09T	ERBB3	v-erb-b2 erythroblastic leukemia viral oncogene homolog 3 (avian)	CCDS31833.1	chr12_56478854-56478854_G_A	104V>M	Substitution	Nonsynonymous coding	44%
MM09T	FANCD2	Fanconi anemia, complementation group D2	CCDS2595.1	chr3_10088343-10088343_A_G	405N>S	Substitution	Nonsynonymous coding	26%
MM09T	KCNJ14	potassium inwardly-rectifying channel, subfamily J, member 14	CCDS12721.1	chr19_48967528-48967528_G_A	269V>M	Substitution	Nonsynonymous coding	22%

MM09T	KLHL36	kelch-like 36 (Drosophila)	CCDS10948.1	chr16_84695417-84695417_G_A	510R>H	Substitution	Nonsynonymous coding	21%
MM09T	KRAS	v-Ki-ras2 Kirsten rat sarcoma viral oncogene homolog	CCDS8703.1	chr12_25398284-25398284_C_A	12G>V	Substitution	Nonsynonymous coding	52%
MM09T	LIG3	ligase III, DNA, ATP-dependent	CCDS11284.2	chr17_33310077-33310077_G_A	18R>Q	Substitution	Nonsynonymous coding	20%
MM09T	LYZL2	lysozyme-like 2	CCDS7167.2	chr10_30918597-30918597_T_C	13K>R	Substitution	Nonsynonymous coding	13%
MM09T	MBD1	methyl-CpG binding domain protein 1	CCDS11943.1	chr18_47803109-47803109_C_T	133C>Y	Substitution	Nonsynonymous coding	19%
MM09T	NEIL1	nei endonuclease VIII-like 1 (E. coli)	CCDS10278.1	chr15_75646604-75646604_C_T	287P>L	Substitution	Nonsynonymous coding	30%
MM09T	NLRP5	NLR family, pyrin domain containing 5	CCDS12938.1	chr19_56520199-56520199_C_A	163P>Q	Substitution	Nonsynonymous coding	15%
MM09T	PIK3CA	phosphatidylinositol-4,5-bisphosphate 3-kinase, catalytic subunit alpha	CCDS43171.1	chr3_178952085-178952085_A_G	1047H>R	Substitution	Nonsynonymous coding	39%
MM09T	PLCE1	phospholipase C, epsilon 1	CCDS41552.1	chr10_96028768-96028768_C_T	1455T>M	Substitution	Nonsynonymous coding	16%
MM09T	PPP1R9A	protein phosphatase 1, regulatory subunit 9A	NM_001166160	chr7_94833011-94833011_A_G	642N>S	Substitution	Nonsynonymous coding	13%
MM09T	PRRT4	proline-rich transmembrane protein 4	CCDS47698.1	chr7_127991680-127991680_G_A	644H>Y	Substitution	Nonsynonymous coding	13%
MM09T	PXDNL	peroxidasin homolog (Drosophila)-like	CCDS47855.1	chr8_52412283-52412283_C_T	143G>E	Substitution	Nonsynonymous coding	19%
MM09T	RBL1	retinoblastoma-like 1 (p107)	CCDS13289.1	chr20_35651201-35651201_C_T	804R>H	Substitution	Nonsynonymous coding	15%
MM09T	RNF112	ring finger protein 112	NM_007148	chr17_19318369-19318369_G_A	382R>H	Substitution	Nonsynonymous coding	18%
MM09T	RNF123	ring finger protein 123	CCDS33758.1	chr3_49758068-49758068_C_T	1209R>C	Substitution	Nonsynonymous coding	16%
MM09T	RXFP1	relaxin/insulin-like family peptide receptor 1	CCDS43276.1	chr4_159566061-159566061_A_G	372I>M	Substitution	Nonsynonymous coding	21%
MM09T	SHE	Src homology 2 domain containing E	CCDS30877.1	chr1_154456625-154456625_T_G	496*>Y	Substitution	Nonsynonymous coding	14%
MM09T	SIGLEC1	sialic acid binding Ig-like lectin 1, sialoadhesin	CCDS13060.1	chr20_3672185-3672185_A_G	1465C>R	Substitution	Nonsynonymous coding	13%
MM09T	SLC38A5	solute carrier family 38, member 5	CCDS14293.1	chrX_48319088-48319088_G_A	339R>C	Substitution	Nonsynonymous coding	18%
MM09T	SPOP	speckle-type POZ protein	CCDS11551.1	chr17_47696716-47696716_C_T	78E>K	Substitution	Nonsynonymous coding	22%
MM09T	TIMM21	translocase of inner mitochondrial membrane 21 homolog (yeast)	CCDS12003.1	chr18_71816122-71816122_T_G	27Y>D	Substitution	Nonsynonymous coding	18%
MM09T	TRAM1L1	translocation associated membrane protein 1-like 1	CCDS3707.1	chr4_118006500-118006500_T_A	17E>V	Substitution	Nonsynonymous coding	14%
MM09T	UBAC1	UBA domain containing 1	CCDS35177.1	chr9_138839666-138839666_G_A	140A>V	Substitution	Nonsynonymous coding	12%
MM09T	VARS	valyl-tRNA synthetase	CCDS34412.1	chr6_31760236-31760236_T_C	240K>R	Substitution	Nonsynonymous coding	15%
MM09T	ZNF800	zinc finger protein 800	CCDS5795.1	chr7_127017355-127017355_T_A	62L>F	Substitution	Nonsynonymous coding	16%
MM10T	MARCH8	membrane-associated ring finger (C3HC4) 8, E3 ubiquitin protein ligase	CCDS7213.1	chr10_45953829-45953829_C_T	245S>N	Substitution	Nonsynonymous coding	83%
MM10T	A2M	alpha-2-macroglobulin	CCDS44827.1	chr12_9248167-9248167_T_C	661T>A	Substitution	Nonsynonymous coding	13%
MM10T	AKAP6	A kinase (PRKA) anchor protein 6	CCDS9644.1	chr14_33292146-33292146_C_A	1709N>K	Substitution	Nonsynonymous coding	17%
MM10T	ARID1A	AT rich interactive domain 1A (SWI-like)	CCDS285.1	chr1_27094424-27094424_G_	NA	Deletion	Frameshift	68%
MM10T	ARMC4	armadillo repeat containing 4	CCDS7157.1	chr10_28229688-28229688_G_A	597S>L	Substitution	Nonsynonymous coding	12%
MM10T	ARPC1B	actin related protein 2/3 complex, subunit 1B, 41kDa	CCDS5661.1	chr7_98988655-98988655_G_A	214G>R	Substitution	Nonsynonymous coding	16%
MM10T	ASTN2	astrotactin 2	CCDS48009.1	chr9_119976961-119976961_G_A	231R>X	Substitution	Nonsense	61%

MM10T	ATP6V1A	ATPase, H+ transporting, lysosomal 70kDa, V1 subunit A	CCDS2976.1	chr3_113507661-113507661_T_C	273I>T	Substitution	Nonsynonymous coding	20%
MM10T	B4GALNT3	beta-1,4-N-acetyl-galactosaminyl transferase 3	CCDS8504.1	chr12_645453-645453_C_G	115L>V	Substitution	Nonsynonymous coding	18%
MM10T	CCKAR	cholecystokinin A receptor	CCDS3438.1	chr4_26484867-26484867_C_T	222G>E	Substitution	Nonsynonymous coding	23%
MM10T	CD22	CD22 molecule	CCDS12457.1	chr19_35832462-35832462_A_G	575N>S	Substitution	Nonsynonymous coding	18%
MM10T	CDH8	cadherin 8, type 2	CCDS10802.1	chr16_61851408-61851408_G_T	418P>T	Substitution	Nonsynonymous coding	27%
MM10T	CELSR1	cadherin, EGF LAG seven-pass G-type receptor 1 (flamingo homolog, Drosophila)	CCDS14076.1	chr22_46931003-46931003_G_A	689Q>X	Substitution	Nonsense	28%
MM10T	CENPE	centromere protein E, 312kDa	CCDS34042.1	chr4_104030043-104030043_C_A	2643C>F	Substitution	Nonsynonymous coding	17%
MM10T	CHD1	chromodomain helicase DNA binding protein 1	CCDS34204.1	chr5_98223925-98223925_C_A	788G>V	Substitution	Nonsynonymous coding	24%
MM10T	CLIC5	chloride intracellular channel 5	CCDS47438.1	chr6_45870960-45870960_G_C	366I>M	Substitution	Nonsynonymous coding	34%
MM10T	COL4A3	collagen, type IV, alpha 3 (Goodpasture antigen)	CCDS42829.1	chr2_228172428-228172428_C_T	1419P>S	Substitution	Nonsynonymous coding	14%
MM10T	CTD-3064H18.4	uncharacterized protein	ENST00000316807	chr19_38039944-38039944_G_A	20L>F	Substitution	Nonsynonymous coding	13%
MM10T	DEK	DEK oncogene	CCDS34344.1	chr6_18249962-18249962_C_G	228D>H	Substitution	Nonsynonymous coding	18%
MM10T	DOPEY1	dopey family member 1	CCDS4996.1	chr6_83847881-83847881_C_T	1374Q>X	Substitution	Nonsense	12%
MM10T	DUOXA1	dual oxidase maturation factor 1	CCDS10119.1	chr15_45410147-45410147_C_T	371R>Q	Substitution	Nonsynonymous coding	28%
MM10T	EFR3B	EFR3 homolog B (S. cerevisiae)	CCDS46231.1	chr2_25353467-25353467_C_A	273R>S	Substitution	Nonsynonymous coding	32%
MM10T	ELSPBP1	epididymal sperm binding protein 1	CCDS12708.1	chr19_48519260-48519260_G_A	107D>N	Substitution	Nonsynonymous coding	56%
MM10T	EMC4	ER membrane protein complex subunit 4	CCDS10035.1	chr15_34520687-34520687_T_C	138I>T	Substitution	Nonsynonymous coding	20%
MM10T	FAM214B	family with sequence similarity 214, member B	CCDS6578.1	chr9_35107475-35107475_C_T	266R>Q	Substitution	Nonsynonymous coding	29%
MM10T	FN3KRP	fructosamine 3 kinase related protein	CCDS11817.1	chr17_80684712-80684712_A_C	199K>Q	Substitution	Nonsynonymous coding	17%
MM10T	FREM1	FRAS1 related extracellular matrix 1	CCDS47952.1	chr9_14775989-14775989_T_C	1552H>R	Substitution	Nonsynonymous coding	47%
MM10T	GCOM1	GRINL1A complex locus 1	CCDS32247.1	chr15_57925820-57925820_A_G	272N>D	Substitution	Nonsynonymous coding	12%
MM10T	GOLGA3	golgin A3	CCDS9281.1	chr12_133373163-133373163_C_G	688A>P	Substitution	Nonsynonymous coding	25%
MM10T	GRIN2D	glutamate receptor, ionotropic, N-methyl D-aspartate 2D	CCDS12719.1	chr19_48925066-48925066_C_A	706P>T	Substitution	Nonsynonymous coding	15%
MM10T	GRM8	glutamate receptor, metabotropic 8	CCDS47696.1	chr7_126173250-126173250_C_T	729R>Q	Substitution	Nonsynonymous coding	20%
MM10T	HDAC2	histone deacetylase 2	CCDS43493.1	chr6_114292207-114292207_C_A	50A>S	Substitution	Nonsynonymous coding	13%
MM10T	HNRNPA2B1	heterogeneous nuclear ribonucleoprotein A2/B1	CCDS43557.1	chr7_26237273-26237273_T_C	41Y>C	Substitution	Nonsynonymous coding	32%
MM10T	ICAM5	intercellular adhesion molecule 5, telencephalin	CCDS12233.1	chr19_10404578-10404578_C_T	557P>L	Substitution	Nonsynonymous coding	13%
MM10T	IK	IK cytokine, down-regulator of HLA II	CCDS47280.1	chr5_140037169-140037169_G_T	278V>F	Substitution	Nonsynonymous coding	17%
MM10T	IRS1	insulin receptor substrate 1	CCDS2463.1	chr2_227662199-227662199_G_A	419S>F	Substitution	Nonsynonymous coding	20%
MM10T	ITGB4	integrin, beta 4	CCDS11727.1	chr17_73732406-73732406_G_A	600R>H	Substitution	Nonsynonymous coding	21%
MM10T	KANK1	KN motif and ankyrin repeat domains 1	CCDS34976.1	chr9_711409-711409_G_A	215G>R	Substitution	Nonsynonymous coding	30%
MM10T	KIAA1324L	uncharacterized protein	CCDS47632.1	chr7_86509836-86509838_TCT_	NA	Deletion	In-frame deletion	13%

MM10T	KIF15	kinesin family member 15	CCDS33744.1	chr3_44893430-44893430_G_C	1320E>Q	Substitution	Nonsynonymous coding	34%
MM10T	LPIN1	lipin 1	CCDS1682.1	chr2_11935605-11935605_G_T	590L>F	Substitution	Nonsynonymous coding	37%
MM10T	MAGEE1	melanoma antigen family E, 1	CCDS14433.1	chrX_75649080-75649080_G_A	253D>N	Substitution	Nonsynonymous coding	38%
MM10T	MAMLD1	mastermind-like domain containing 1	CCDS14693.2	chrX_149613877-149613877_C_T	32S>L	Substitution	Nonsynonymous coding	25%
MM10T	MAN1A1	mannosidase, alpha, class 1A, member 1	CCDS5122.1	chr6_119509710-119509710_C_T	527D>N	Substitution	Nonsynonymous coding	26%
MM10T	MASP1	mannan-binding lectin serine peptidase 1 (C4/C2 activating component of Ra-reactive	CCDS33907.1	chr3_186938886-186938886_T_A	616K>X	Substitution	Nonsense	11%
MM10T	MCMBP	minichromosome maintenance complex binding protein	CCDS7617.1	chr10_121602814-121602814_T_G	318I>L	Substitution	Nonsynonymous coding	23%
MM10T	MTMR8	myotubularin related protein 8	CCDS14379.1	chrX_63488736-63488736_T_C	599D>G	Substitution	Nonsynonymous coding	39%
MM10T	N4BP1	NEDD4 binding protein 1	CCDS45479.1	chr16_48595127-48595127__C	NA	Insertion	Frameshift	21%
MM10T	NASP	nuclear autoantigenic sperm protein (histone-binding)	CCDS526.1	chr1_46073604-46073604_G_A	343A>T	Substitution	Nonsynonymous coding	17%
MM10T	NTRK2	neurotrophic tyrosine kinase, receptor, type 2	CCDS6671.1	chr9_87285764-87285764_C_T	34T>M	Substitution	Nonsynonymous coding	28%
MM10T	PCDHGA7	protocadherin gamma subfamily A, 7	NM_018920	chr5_140764319-140764319_C_T	618A>V	Substitution	Nonsynonymous coding	18%
MM10T	PIK3R2	phosphoinositide-3-kinase, regulatory subunit 2 (beta)	CCDS12371.1	chr19_18273884-18273884_G_A	406R>H	Substitution	Nonsynonymous coding	18%
MM10T	PLK1	polo-like kinase 1	CCDS10616.1	chr16_23691564-23691564_G_A	190V>M	Substitution	Nonsynonymous coding	22%
MM10T	RASL12	RAS-like, family 12	CCDS10200.1	chr15_65347311-65347311_C_T	243V>M	Substitution	Nonsynonymous coding	24%
MM10T	RB1	retinoblastoma 1	CCDS31973.1	chr13_49039213-49039213_T_A	764L>Q	Substitution	Nonsynonymous coding	24%
MM10T	RBM4B	RNA binding motif protein 4B	CCDS8149.1	chr11_66436178-66436178_C_A	333A>S	Substitution	Nonsynonymous coding	23%
MM10T	RIPPLY2	ripply2 homolog (zebrafish)	CCDS34493.1	chr6_84563182-84563182_C_T	16A>V	Substitution	Nonsynonymous coding	14%
MM10T	SAMD15	sterile alpha motif domain containing 15	CCDS32126.1	chr14_77844219-77844219_C_A	153S>X	Substitution	Nonsense	18%
MM10T	SDK2	sidekick cell adhesion molecule 2	CCDS45769.1	chr17_71420083-71420083_C_T	578D>N	Substitution	Nonsynonymous coding	38%
MM10T	SH3RF3	SH3 domain containing ring finger 3	NM_001099289	chr2_109746153-109746153_G_T	53D>Y	Substitution	Nonsynonymous coding	13%
MM10T	SLC25A13	solute carrier family 25 (aspartate/glutamate carrier), member 13	CCDS5645.1	chr7_95775937-95775937_T_A	461E>D	Substitution	Nonsynonymous coding	19%
MM10T	SNHG14	small nucleolar RNA host gene 14 (non-protein coding)	ENST00000383025	chr15_25328597-25328597_C_T	80A>V	Substitution	Nonsynonymous coding	47%
MM10T	SPHKAP	SPHK1 interactor, AKAP domain containing	CCDS46537.1	chr2_228881796-228881796_G_T	1258N>K	Substitution	Nonsynonymous coding	17%
MM10T	SPTBN5	spectrin, beta, non-erythrocytic 5	NM_016642	chr15_42173303-42173303_C_A	828D>Y	Substitution	Nonsynonymous coding	40%
MM10T	SUPT6H	suppressor of Ty 6 homolog (S. cerevisiae)	CCDS32596.1	chr17_27002000-27002000_C_T	120R>C	Substitution	Nonsynonymous coding	14%
MM10T	SYNE1	spectrin repeat containing, nuclear envelope 1	CCDS5236.1	chr6_152631535-152631535_C_G	5672R>P	Substitution	Nonsynonymous coding	13%
MM10T	TM9SF1	transmembrane 9 superfamily member 1	CCDS9617.1	chr14_24664158-24664158_C_	NA	Deletion	Frameshift	23%
MM10T	TMLHE	trimethyllysine hydroxylase, epsilon	CCDS14768.1	chrX_154741409-154741409_G_A	228S>F	Substitution	Nonsynonymous coding	17%
MM10T	TMPRSS9	transmembrane protease, serine 9	CCDS12088.1	chr19_2415703-2415703_C_T	503R>W	Substitution	Nonsynonymous coding	12%
MM10T	TP53	tumor protein p53	CCDS11118.1	chr17_7577566-7577566_T_C	239N>D	Substitution	Nonsynonymous coding	89%
MM10T	TRHDE	thyrotropin-releasing hormone degrading enzyme	CCDS9004.1	chr12_72936078-72936078_C_A	532A>D	Substitution	Nonsynonymous coding	22%

MM10T	TRMT11	tRNA methyltransferase 11 homolog (S. cerevisiae)	CCDS35496.1	chr6_126314956-126314956_A_T	42E>D	Substitution	Nonsynonymous coding	11%
MM10T	TRPV4	transient receptor potential cation channel, subfamily V, member 4	CCDS9134.1	chr12_110224623-110224623_T_C	743D>G	Substitution	Nonsynonymous coding	68%
MM10T	TTN	titin	ENST00000356127	chr2_179516471-179516471_C_A	10695V>L	Substitution	Nonsynonymous coding	15%
MM10T	TUFT1	tuftelin 1	CCDS1000.1	chr1_151551303-151551303_G_T	_ISV+1>	Substitution	Splice site donor	10%
MM10T	VGLL3	vestigial like 3 (Drosophila)	CCDS43110.1	chr3_87018204-87018204_G_T	158P>Q	Substitution	Nonsynonymous coding	11%
MM10T	WDFY4	WDFY family member 4	CCDS44385.1	chr10_50151420-50151420_G_A	2559A>T	Substitution	Nonsynonymous coding	35%
MM10T	ZNF451	zinc finger protein 451	ENST00000370711	chr6_56963946-56963946_A_G	65T>A	Substitution	Nonsynonymous coding	21%
MM10T	ZNF648	zinc finger protein 648	CCDS30952.1	chr1_182025929-182025929_C_T	406R>H	Substitution	Nonsynonymous coding	16%
MM11T	ACTL6B	actin-like 6B	CCDS5702.1	chr7_100252740-100252740_C_T	91E>K	Substitution	Nonsynonymous coding	19%
MM11T	BAZ1A	bromodomain adjacent to zinc finger domain, 1A	CCDS9651.1	chr14_35231425-35231425_G_A	1261P>S	Substitution	Nonsynonymous coding	13%
MM11T	BEST3	bestrophin 3	CCDS8992.2	chr12_70070852-70070852_G_A	NA	Substitution	Splice site acceptor	18%
MM11T	C14orf93	chromosome 14 open reading frame 93	CCDS9583.1	chr14_23465326-23465326_C_T	250R>H	Substitution	Nonsynonymous coding	23%
MM11T	CCDC146	coiled-coil domain containing 146	CCDS34671.1	chr7_76916813-76916813_T_A	778Y>X	Substitution	Nonsense	13%
MM11T	COL5A3	collagen, type V, alpha 3	CCDS12222.1	chr19_10081321-10081321_C_T	1305A>T	Substitution	Nonsynonymous coding	31%
MM11T	CTNNB1	catenin (cadherin-associated protein), beta 1, 88kDa	CCDS2694.1	chr3_41266101-41266101_C_G	33S>C	Substitution	Nonsynonymous coding	35%
MM11T	DPF2	D4, zinc and double PHD fingers family 2	CCDS8100.1	chr11_65107954-65107954_C_T	44S>L	Substitution	Nonsynonymous coding	13%
MM11T	DRD4	dopamine receptor D4	CCDS7710.1	chr11_640535-640535_G_	NA	Deletion	Frameshift	21%
MM11T	E2F7	E2F transcription factor 7	CCDS9016.1	chr12_77423772-77423772_C_T	575D>N	Substitution	Nonsynonymous coding	30%
MM11T	ESR1	estrogen receptor 1	CCDS5234.1	chr6_152419923-152419923_A_C	537Y>S	Substitution	Nonsynonymous coding	32%
MM11T	FAM133A	family with sequence similarity 133, member A	CCDS14466.1	chrX_92964582-92964582_C_A	55S>X	Substitution	Nonsense	17%
MM11T	FARSA	phenylalanyl-tRNA synthetase, alpha subunit	CCDS12287.1	chr19_13041256-13041256_C_T	124R>Q	Substitution	Nonsynonymous coding	13%
MM11T	FAT1	FAT tumor suppressor homolog 1 (Drosophila)	CCDS47177.1	chr4_187541847-187541847_C_T	1965V>I	Substitution	Nonsynonymous coding	12%
MM11T	FREM2	FRAS1 related extracellular matrix protein 2	CCDS31960.1	chr13_39452996-39452996_C_T	2963T>I	Substitution	Nonsynonymous coding	15%
MM11T	GPR162	G-protein coupled receptor 162	CCDS8563.1	chr12_6933731-6933731_G_T	223G>W	Substitution	Nonsynonymous coding	31%
MM11T	HUWE1	HECT, UBA and WWE domain containing 1, E3 ubiquitin protein ligase	CCDS35301.1	chrX_53564560-53564560_G_A	4032R>C	Substitution	Nonsynonymous coding	24%
MM11T	MARVELD2	MARVEL domain containing 2	CCDS34175.1	chr5_68715276-68715276_C_T	22P>S	Substitution	Nonsynonymous coding	31%
MM11T	NLRP6	NLR family, pyrin domain containing 6	CCDS7693.1	chr11_281571-281571_G_A	613E>K	Substitution	Nonsynonymous coding	44%
MM11T	PARP10	poly (ADP-ribose) polymerase family, member 10 [Source:HGNC Symbol;Acc:25895]	CCDS34960.1	chr8_145051803-145051803_T_G	976D>A	Substitution	Nonsynonymous coding	19%
MM11T	PLXNA3	plexin A3	CCDS14752.1	chrX_153699557-153699557_A_G	1756M>V	Substitution	Nonsynonymous coding	23%
MM11T	PRDM10	PR domain containing 10	CCDS44771.1	chr11_129802076-129802076_C_T	403R>Q	Substitution	Nonsynonymous coding	19%
MM11T	PRKCB	protein kinase C, beta	CCDS10619.1	chr16_24202490-24202490_G_A	601R>Q	Substitution	Nonsynonymous coding	11%
MM11T	PXDNL	peroxidasin homolog (Drosophila)-like	CCDS47855.1	chr8_52321257-52321257_C_T	976R>Q	Substitution	Nonsynonymous coding	17%

MM11T	RNASEH2B	ribonuclease H2, subunit B	CCDS9425.1	chr13_51528056-51528056_G_A	253D>N	Substitution	Nonsynonymous coding	11%
MM11T	SLC39A6	solute carrier family 39 (zinc transporter), member 6	CCDS42428.1	chr18_33691209-33691209_T_A	643D>V	Substitution	Nonsynonymous coding	38%
MM11T	SPAG17	sperm associated antigen 17	CCDS899.1	chr1_118514636-118514636_G_A	2059S>F	Substitution	Nonsynonymous coding	17%
MM11T	SPEF2	sperm flagellar 2	ENST00000303129	chr5_35789448-35789448_G_A	97M>I	Substitution	Nonsynonymous coding	13%
MM11T	SSPO	SCO-spondin homolog (Bos taurus)	ENST00000262089	chr7_149492787-149492787_G_T	1233Q>H	Substitution	Nonsynonymous coding	25%
MM11T	TBL3	transducin (beta)-like 3	CCDS10453.1	chr16_2024784-2024784_C_T	134R>C	Substitution	Nonsynonymous coding	19%
MM11T	TUBD1	tubulin, delta 1	CCDS11620.1	chr17_57944063-57944063_T_A	328S>C	Substitution	Nonsynonymous coding	11%
MM12T	MARCH7	membrane-associated ring finger (C3HC4) 7, E3 ubiquitin protein ligase	CCDS2210.1	chr2_160602422-160602422_G_A	163R>Q	Substitution	Nonsynonymous coding	21%
MM12T	SEPT1	septin 1	CCDS10678.1	chr16_30393191-30393191_C_A	65Q>H	Substitution	Nonsynonymous coding	41%
MM12T	SEPT3	septin 3	CCDS14026.2	chr22_42390641-42390641_C_A	312L>I	Substitution	Nonsynonymous coding	52%
MM12T	SEPT6	septin 6	CCDS14584.1	chrX_118827061-118827061_G_A	3A>V	Substitution	Nonsynonymous coding	15%
MM12T	SEPT7	septin 7	NM_001011553	chr7_35872496-35872496_C_T	51T>M	Substitution	Nonsynonymous coding	24%
MM12T	SEPT8	septin 8	CCDS43358.1	chr5_132096610-132096610_T_G	390E>D	Substitution	Nonsynonymous coding	37%
MM12T	SEPT9	septin 9	CCDS45790.1	chr17_75398315-75398315_G_A	84R>H	Substitution	Nonsynonymous coding	43%
MM12T	A1BG	alpha-1-B glycoprotein	CCDS12976.1	chr19_58862986-58862986_C_A	227K>N	Substitution	Nonsynonymous coding	16%
MM12T	A2ML1	alpha-2-macroglobulin-like 1	CCDS8596.2	chr12_9010659-9010659_C_A	1075C>X	Substitution	Nonsense	11%
MM12T	AAK1	AP2 associated kinase 1	CCDS1893.2	chr2_69784051-69784051_G_A	75R>C	Substitution	Nonsynonymous coding	15%
MM12T	AAK1	AP2 associated kinase 1	CCDS1893.2	chr2_69752245-69752245_C_T	ISV-1>	Substitution	Splice site acceptor	10%
MM12T	AAR2	AAR2 splicing factor homolog (S. cerevisiae)	CCDS13273.1	chr20_34828410-34828410_G_A	207R>H	Substitution	Nonsynonymous coding	31%
MM12T	AASS	aminoadipate-semialdehyde synthase	CCDS5783.1	chr7_121773650-121773650_G_T	44P>H	Substitution	Nonsynonymous coding	30%
MM12T	ABAT	4-aminobutyrate aminotransferase	CCDS10534.1	chr16_8866733-8866733_T_C	305S>P	Substitution	Nonsynonymous coding	33%
MM12T	ABCA1	ATP-binding cassette, sub-family A (ABC1), member 1	CCDS6762.1	chr9_107645412-107645412_C_T	110R>Q	Substitution	Nonsynonymous coding	22%
MM12T	ABCA1	ATP-binding cassette, sub-family A (ABC1), member 1	CCDS6762.1	chr9_107556794-107556794_A_	NA	Deletion	Splice site acceptor	16%
MM12T	ABCA1	ATP-binding cassette, sub-family A (ABC1), member 1	CCDS6762.1	chr9_107556794-107556795_AA_	NA	Deletion	Splice site acceptor	11%
MM12T	ABCA13	ATP-binding cassette, sub-family A (ABC1), member 13	CCDS47584.1	chr7_48411808-48411808_C_T	3616A>V	Substitution	Nonsynonymous coding	14%
MM12T	ABCA2	ATP-binding cassette, sub-family A (ABC1), member 2	CCDS43909.1	chr9_139909991-139909991_C_T	1191R>H	Substitution	Nonsynonymous coding	12%
MM12T	ABCA2	ATP-binding cassette, sub-family A (ABC1), member 2	CCDS43909.1	chr9_139914880-139914880_G_A	445R>W	Substitution	Nonsynonymous coding	11%
MM12T	ABCA2	ATP-binding cassette, sub-family A (ABC1), member 2	CCDS43909.1	chr9_139917508-139917508_G_A	ISV-4>	Substitution	Splice site acceptor	12%
MM12T	ABCA2	ATP-binding cassette, sub-family A (ABC1), member 2	CCDS43909.1	chr9_139911099-139911099_C_T	950W>X	Substitution	Nonsense	34%
MM12T	ABCA3	ATP-binding cassette, sub-family A (ABC1), member 3	CCDS10466.1	chr16_2347480-2347480_C_T	705D>N	Substitution	Nonsynonymous coding	27%
MM12T	ABCA3	ATP-binding cassette, sub-family A (ABC1), member 3	CCDS10466.1	chr16_2369817-2369817_G_A	213A>V	Substitution	Nonsynonymous coding	29%
MM12T	ABCA3	ATP-binding cassette, sub-family A (ABC1), member 3	CCDS10466.1	chr16_2373544-2373544_G_T	198S>Y	Substitution	Nonsynonymous coding	29%

MM12T	ABCA7	ATP-binding cassette, sub-family A (ABC1), member 7	CCDS12055.1	chr19_1047375-1047375_G_A	689A>T	Substitution	Nonsynonymous coding	45%
MM12T	ABCA7	ATP-binding cassette, sub-family A (ABC1), member 7	CCDS12055.1	chr19_1049327-1049327_C_T	815R>C	Substitution	Nonsynonymous coding	11%
MM12T	ABCA9	ATP-binding cassette, sub-family A (ABC1), member 9	CCDS11681.1	chr17_67045483-67045483_G_T	82P>H	Substitution	Nonsynonymous coding	20%
MM12T	ABCB1	ATP-binding cassette, sub-family B (MDR/TAP), member 1	CCDS5608.1	chr7_87178770-87178770_G_A	540A>V	Substitution	Nonsynonymous coding	32%
MM12T	ABCB4	ATP-binding cassette, sub-family B (MDR/TAP), member 4	CCDS5606.1	chr7_87074282-87074282_A_	NA	Deletion	Frameshift	15%
MM12T	ABCB4	ATP-binding cassette, sub-family B (MDR/TAP), member 4	CCDS5606.1	chr7_87079399-87079399_C_T	240A>T	Substitution	Nonsynonymous coding	12%
MM12T	ABCB5	ATP-binding cassette, sub-family B (MDR/TAP), member 5	NM_001163941	chr7_20668418-20668418_G_A	72M>I	Substitution	Nonsynonymous coding	20%
MM12T	ABCB5	ATP-binding cassette, sub-family B (MDR/TAP), member 5	NM_001163941	chr7_20682869-20682869_C_T	126S>F	Substitution	Nonsynonymous coding	31%
MM12T	ABCB5	ATP-binding cassette, sub-family B (MDR/TAP), member 5	CCDS5371.1	chr7_20762730-20762730_C_T	393T>I	Substitution	Nonsynonymous coding	23%
MM12T	ABCB5	ATP-binding cassette, sub-family B (MDR/TAP), member 5	CCDS5371.1	chr7_20778692-20778692_C_A	540A>D	Substitution	Nonsynonymous coding	38%
MM12T	ABCB6	ATP-binding cassette, sub-family B (MDR/TAP), member 6	CCDS2436.1	chr2_220075737-220075737_G_A	688R>C	Substitution	Nonsynonymous coding	21%
MM12T	ABCB6	ATP-binding cassette, sub-family B (MDR/TAP), member 6	CCDS2436.1	chr2_220078366-220078366_C_T	534G>D	Substitution	Nonsynonymous coding	13%
MM12T	ABCB9	ATP-binding cassette, sub-family B (MDR/TAP), member 9	CCDS9241.1	chr12_123414652-123414652_T_C	703T>A	Substitution	Nonsynonymous coding	30%
MM12T	ABCC10	ATP-binding cassette, sub-family C (CFTR/MRP), member 10	CCDS4896.1	chr6_43413520-43413520_C_T	1044P>S	Substitution	Nonsynonymous coding	12%
MM12T	ABCC12	ATP-binding cassette, sub-family C (CFTR/MRP), member 12	CCDS10730.1	chr16_48180280-48180280_C_A	19R>I	Substitution	Nonsynonymous coding	36%
MM12T	ABCC12	ATP-binding cassette, sub-family C (CFTR/MRP), member 12	CCDS10730.1	chr16_48145704-48145704_G_A	703R>X	Substitution	Nonsense	11%
MM12T	ABCC2	ATP-binding cassette, sub-family C (CFTR/MRP), member 2	CCDS7484.1	chr10_101604157-101604157_C_T	1308R>W	Substitution	Nonsynonymous coding	14%
MM12T	ABCC3	ATP-binding cassette, sub-family C (CFTR/MRP), member 3	CCDS32681.1	chr17_48746589-48746589_G_A	676G>R	Substitution	Nonsynonymous coding	32%
MM12T	ABCC5	ATP-binding cassette, sub-family C (CFTR/MRP), member 5	CCDS43176.1	chr3_183689395-183689395_G_A	573R>C	Substitution	Nonsynonymous coding	10%
MM12T	ABCC6	ATP-binding cassette, sub-family C (CFTR/MRP), member 6	CCDS10568.1	chr16_16256898-16256898_C_T	1153R>H	Substitution	Nonsynonymous coding	17%
MM12T	ABCD1	ATP-binding cassette, sub-family D (ALD), member 1	CCDS14728.1	chrX_152990830-152990830_C_T	37R>C	Substitution	Nonsynonymous coding	40%
MM12T	ABCD1	ATP-binding cassette, sub-family D (ALD), member 1	CCDS14728.1	chrX_152991454-152991454_G_A	245A>T	Substitution	Nonsynonymous coding	36%
MM12T	ABCD4	ATP-binding cassette, sub-family D (ALD), member 4	CCDS9828.1	chr14_74759920-74759920_G_A	251R>C	Substitution	Nonsynonymous coding	31%
MM12T	ABCF1	ATP-binding cassette, sub-family F (GCN20), member 1	CCDS34380.1	chr6_30545854-30545854__AA	NA	Insertion	Frameshift	13%
MM12T	ABCF1	ATP-binding cassette, sub-family F (GCN20), member 1	CCDS34380.1	chr6_30553124-30553124_C_T	ISV+4>	Substitution	Splice site donor	21%
MM12T	ABCG1	ATP-binding cassette, sub-family G (WHITE), member 1	CCDS13682.1	chr21_43708097-43708097_G_A	358G>R	Substitution	Nonsynonymous coding	13%
MM12T	ABCG4	ATP-binding cassette, sub-family G (WHITE), member 4	CCDS8415.1	chr11_119031698-119031698_C_T	608A>V	Substitution	Nonsynonymous coding	12%
MM12T	ABCG5	ATP-binding cassette, sub-family G (WHITE), member 5	CCDS1814.1	chr2_44051091-44051091_C_T	429A>T	Substitution	Nonsynonymous coding	16%
MM12T	ABCG8	ATP-binding cassette, sub-family G (WHITE), member 8	CCDS1815.1	chr2_44078920-44078920_C_T	174R>W	Substitution	Nonsynonymous coding	31%
MM12T	ABHD12	abhydrolase domain containing 12	CCDS13172.1	chr20_25290170-25290170_T_C	221T>A	Substitution	Nonsynonymous coding	32%
MM12T	ABHD16B	abhydrolase domain containing 16B	CCDS13539.1	chr20_62493870-62493870_G_A	326S>N	Substitution	Nonsynonymous coding	11%
MM12T	ABHD3	abhydrolase domain containing 3	CCDS32802.1	chr18_19263897-19263897_G_A	180A>V	Substitution	Nonsynonymous coding	13%

MM12T	ABI1	abl-interactor 1	CCDS7150.1	chr10_27059246-27059246_C_A	169G>V	Substitution	Nonsynonymous coding	17%
MM12T	ABI3BP	ABI family, member 3 (NESH) binding protein	CCDS46880.1	chr3_100523693-100523693_G_A	564R>C	Substitution	Nonsynonymous coding	38%
MM12T	ABI3BP	ABI family, member 3 (NESH) binding protein	CCDS46880.1	chr3_100527008-100527008_G_T	557P>T	Substitution	Nonsynonymous coding	16%
MM12T	ABI3BP	ABI family, member 3 (NESH) binding protein	CCDS46880.1	chr3_100605164-100605164_A_C	162F>L	Substitution	Nonsynonymous coding	44%
MM12T	ABL2	v-abl Abelson murine leukemia viral oncogene homolog 2	CCDS30947.1	chr1_179090747-179090747_C_T	315A>T	Substitution	Nonsynonymous coding	31%
MM12T	ABL2	v-abl Abelson murine leukemia viral oncogene homolog 2	CCDS30947.1	chr1_179079591-179079591_C_A	ISV-1>	Substitution	Splice site acceptor	35%
MM12T	ABLIM1	actin binding LIM protein 1	CCDS7590.1	chr10_116203893-116203893_G_A	610L>F	Substitution	Nonsynonymous coding	37%
MM12T	ABLIM3	actin binding LIM protein family, member 3	CCDS4294.1	chr5_148630979-148630979_C_T	616R>X	Substitution	Nonsense	16%
MM12T	ABP1	amiloride binding protein 1 (amine oxidase (copper-containing))	CCDS43679.1	chr7_150557660-150557660_C_T	643P>L	Substitution	Nonsynonymous coding	26%
MM12T	ABRA	actin-binding Rho activating protein	CCDS6305.1	chr8_107781766-107781766_C_T	218R>H	Substitution	Nonsynonymous coding	37%
MM12T	AC006547.14	uncharacterized protein LOC388849 [Source:RefSeq peptide;Acc:NP_001230466]	ENST00000439765	chr22_20138044-20138044_G_T	119A>D	Substitution	Nonsynonymous coding	13%
MM12T	AC009403.2	Uncharacterized protein [Source:UniProtKB/TrEMBL;Acc:B5MCR4]	ENST00000401694	chr7_155404105-155404105_C_A	36E>D	Substitution	Nonsynonymous coding	30%
MM12T	AC016251.1	-	ENST00000378936	chr15_96831407-96831407_G_A	85T>M	Substitution	Nonsynonymous coding	22%
MM12T	AC079612.1	Uncharacterized protein; cDNA FLJ45964 fis, clone PLACE7014396	ENST00000358775	chr2_240500540-240500540_G_A	141A>T	Substitution	Nonsynonymous coding	32%
MM12T	AC093802.1	Uncharacterized protein [Source:UniProtKB/TrEMBL;Acc:B5MD18]	ENST00000407524	chr2_240702099-240702099_G_A	50R>H	Substitution	Nonsynonymous coding	50%
MM12T	AC114273.1	-	ENST00000397365	chr19_16146326-16146326_C_T	123R>C	Substitution	Nonsynonymous coding	14%
MM12T	AC115618.1	Uncharacterized protein; cDNA FLJ26048 fis, clone PRS02384	ENST00000376775	chrX_48433140-48433140_C_T	37A>T	Substitution	Nonsynonymous coding	30%
MM12T	AC133919.6	Uncharacterized protein; cDNA FLJ52651 [Source:UniProtKB/TrEMBL;Acc:B7Z8Y8]	ENST00000409768	chr16_90110975-90110975_G_A	15L>F	Substitution	Nonsynonymous coding	25%
MM12T	AC138647.1	Uncharacterized protein [Source:UniProtKB/TrEMBL;Acc:C9IYH1]	ENST00000427937	chr8_142528537-142528537_G_A	97D>N	Substitution	Nonsynonymous coding	17%
MM12T	ACAA1	acetyl-CoA acyltransferase 1	CCDS2673.1	chr3_38178412-38178412_C_T	39V>M	Substitution	Nonsynonymous coding	20%
MM12T	ACAA2	acetyl-CoA acyltransferase 2	CCDS11939.1	chr18_47318689-47318689_C_T	196A>T	Substitution	Nonsynonymous coding	15%
MM12T	ACACB	acetyl-CoA carboxylase beta	CCDS31898.1	chr12_109623408-109623408_C_T	615R>W	Substitution	Nonsynonymous coding	31%
MM12T	ACACB	acetyl-CoA carboxylase beta	CCDS31898.1	chr12_109654684-109654684_G_A	1175A>T	Substitution	Nonsynonymous coding	11%
MM12T	ACAD10	acyl-CoA dehydrogenase family, member 10	CCDS44973.1	chr12_112165918-112165918_C_T	436A>V	Substitution	Nonsynonymous coding	13%
MM12T	ACADL	acyl-CoA dehydrogenase, long chain	CCDS2389.1	chr2_211082758-211082758_A_G	101V>A	Substitution	Nonsynonymous coding	19%
MM12T	ACCS	1-aminocyclopropane-1-carboxylate synthase homolog (Arabidopsis)(non-functional)	CCDS7907.1	chr11_44102787-44102787_C_T	343A>V	Substitution	Nonsynonymous coding	26%
MM12T	ACE	angiotensin I converting enzyme (peptidyl-dipeptidase A) 1	CCDS11637.1	chr17_61560847-61560847_C_T	505P>L	Substitution	Nonsynonymous coding	19%
MM12T	ACE	angiotensin I converting enzyme (peptidyl-dipeptidase A) 1	CCDS11637.1	chr17_61560877-61560877_C_T	515A>V	Substitution	Nonsynonymous coding	25%
MM12T	ACE	angiotensin I converting enzyme (peptidyl-dipeptidase A) 1	CCDS11637.1	chr17_61573795-61573795_G_A	1141A>T	Substitution	Nonsynonymous coding	37%
MM12T	ACOX2	acyl-CoA oxidase 2, branched chain	CCDS33775.1	chr3_58520148-58520148_G_A	88R>W	Substitution	Nonsynonymous coding	27%
MM12T	ACP2	acid phosphatase 2, lysosomal	CCDS7928.1	chr11_47261698-47261698_C_T	414R>H	Substitution	Nonsynonymous coding	10%
MM12T	ACPL2	acid phosphatase-like 2	CCDS3116.1	chr3_140998312-140998312_C_T	111R>X	Substitution	Nonsense	33%

MM12T	ACRC	acidic repeat containing	CCDS35326.1	chrX_70824284-70824284_C_A	386P>H	Substitution	Nonsynonymous coding	36%
MM12T	ACSL4	acyl-CoA synthetase long-chain family member 4	CCDS14548.1	chrX_108911458-108911458_C_T	437R>H	Substitution	Nonsynonymous coding	22%
MM12T	ACSL5	acyl-CoA synthetase long-chain family member 5	CCDS7572.1	chr10_114187105-114187105_A_G	737I>V	Substitution	Nonsynonymous coding	32%
MM12T	ACSL6	acyl-CoA synthetase long-chain family member 6	CCDS34228.1	chr5_131308972-131308972_C_T	397R>Q	Substitution	Nonsynonymous coding	14%
MM12T	ACSL6	acyl-CoA synthetase long-chain family member 6	CCDS34228.1	chr5_131323864-131323864_C_A	236E>D	Substitution	Nonsynonymous coding	13%
MM12T	ACSM2B	acyl-CoA synthetase medium-chain family member 2B	CCDS10586.1	chr16_20563515-20563515_C_T	282C>Y	Substitution	Nonsynonymous coding	17%
MM12T	ACSM4	acyl-CoA synthetase medium-chain family member 4	CCDS44825.1	chr12_7473398-7473398_G_T	333K>N	Substitution	Nonsynonymous coding	10%
MM12T	ACSS2	acyl-CoA synthetase short-chain family member 2	CCDS13243.1	chr20_33507322-33507322_G_A	307A>T	Substitution	Nonsynonymous coding	25%
MM12T	ACTL7A	actin-like 7A	CCDS6772.1	chr9_111625501-111625501_G_A	300R>H	Substitution	Nonsynonymous coding	10%
MM12T	ACTL7B	actin-like 7B	CCDS6771.1	chr9_111617720-111617720_G_A	164A>V	Substitution	Nonsynonymous coding	31%
MM12T	ACTL8	actin-like 8	CCDS183.1	chr1_18152404-18152404_G_A	164R>H	Substitution	Nonsynonymous coding	11%
MM12T	ACTN1	actinin, alpha 1	CCDS45130.1	chr14_69349672-69349672_A_T	579V>D	Substitution	Nonsynonymous coding	27%
MM12T	ACTN1	actinin, alpha 1	CCDS45130.1	chr14_69369214-69369214_C_T	248A>T	Substitution	Nonsynonymous coding	32%
MM12T	ACTN2	actinin, alpha 2	CCDS1613.1	chr1_236881198-236881198_G_A	56G>D	Substitution	Nonsynonymous coding	23%
MM12T	ACTN2	actinin, alpha 2	CCDS1613.1	chr1_236906196-236906196_G_A	370D>N	Substitution	Nonsynonymous coding	16%
MM12T	ACTN4	actinin, alpha 4	CCDS12518.1	chr19_39214259-39214259_T_C	483L>P	Substitution	Nonsynonymous coding	33%
MM12T	ACTR1A	ARP1 actin-related protein 1 homolog A, centractin alpha (yeast)	CCDS7536.1	chr10_104242820-104242820_G_A	233T>M	Substitution	Nonsynonymous coding	33%
MM12T	ACTR6	ARP6 actin-related protein 6 homolog (yeast)	CCDS9074.1	chr12_100613790-100613790_G_C	309M>I	Substitution	Nonsynonymous coding	50%
MM12T	ACTRT3	actin-related protein T3	CCDS3206.1	chr3_169486049-169486049_G_A	97P>L	Substitution	Nonsynonymous coding	36%
MM12T	ACVR2B	activin A receptor, type IIB	CCDS2679.1	chr3_38518844-38518844_G_A	40R>H	Substitution	Nonsynonymous coding	13%
MM12T	ADAD2	adenosine deaminase domain containing 2	CCDS10944.1	chr16_84228102-84228102_C_T	230A>V	Substitution	Nonsynonymous coding	35%
MM12T	ADAM15	ADAM metallopeptidase domain 15	CCDS1087.1	chr1_155029568-155029568_C_T	380A>V	Substitution	Nonsynonymous coding	34%
MM12T	ADAM18	ADAM metallopeptidase domain 18	CCDS6113.1	chr8_39525629-39525629_G_A	480G>D	Substitution	Nonsynonymous coding	14%
MM12T	ADAM19	ADAM metallopeptidase domain 19	CCDS4338.1	chr5_156991409-156991409_C_T	75E>K	Substitution	Nonsynonymous coding	33%
MM12T	ADAM23	ADAM metallopeptidase domain 23	CCDS2369.1	chr2_207422202-207422202_G_A	329V>M	Substitution	Nonsynonymous coding	24%
MM12T	ADAM7	ADAM metallopeptidase domain 7	CCDS6045.1	chr8_24342794-24342794_A_G	294K>E	Substitution	Nonsynonymous coding	14%
MM12T	ADAM8	ADAM metallopeptidase domain 8	NM_001109	chr10_135085374-135085374_C_T	348V>I	Substitution	Nonsynonymous coding	36%
MM12T	ADAM9	ADAM metallopeptidase domain 9	CCDS6112.1	chr8_38899526-38899526_G_A	398G>R	Substitution	Nonsynonymous coding	12%
MM12T	ADAMTS10	ADAM metallopeptidase with thrombospondin type 1 motif, 10 [Source:HGNC	CCDS12206.1	chr19_8651744-8651744_A_G	732V>A	Substitution	Nonsynonymous coding	36%
MM12T	ADAMTS12	ADAM metallopeptidase with thrombospondin type 1 motif, 12	CCDS34140.1	chr5_33576635-33576635_G_A	1166P>S	Substitution	Nonsynonymous coding	31%
MM12T	ADAMTS13	ADAM metallopeptidase with thrombospondin type 1 motif, 13	CCDS6970.1	chr9_136295180-136295180_G_A	316G>S	Substitution	Nonsynonymous coding	29%
MM12T	ADAMTS2	ADAM metallopeptidase with thrombospondin type 1 motif, 2	CCDS4444.1	chr5_178564928-178564928_C_T	598R>H	Substitution	Nonsynonymous coding	14%

MM12T	ADAMTS3	ADAM metallopeptidase with thrombospondin type 1 motif, 3	CCDS3553.1	chr4_73188757-73188757_G_A	307R>C	Substitution	Nonsynonymous coding	13%
MM12T	ADAMTS6	ADAM metallopeptidase with thrombospondin type 1 motif, 6	CCDS3983.2	chr5_64558659-64558659_T_C	584H>R	Substitution	Nonsynonymous coding	40%
MM12T	ADAMTS6	ADAM metallopeptidase with thrombospondin type 1 motif, 6	CCDS3983.2	chr5_64748662-64748662_G_T	239H>N	Substitution	Nonsynonymous coding	23%
MM12T	ADAMTS6	ADAM metallopeptidase with thrombospondin type 1 motif, 6	CCDS3983.2	chr5_64511309-64511309_T_A	760K>X	Substitution	Nonsense	11%
MM12T	ADAMTSL2	ADAMTS-like 2	CCDS6976.1	chr9_136412178-136412178_G_A	261G>D	Substitution	Nonsynonymous coding	34%
MM12T	ADAMTSL2	ADAMTS-like 2	CCDS6976.1	chr9_136426348-136426348_G_A	560V>M	Substitution	Nonsynonymous coding	28%
MM12T	ADAMTSL3	ADAMTS-like 3	CCDS10326.1	chr15_84442329-84442329_G_A	82G>S	Substitution	Nonsynonymous coding	24%
MM12T	ADAMTSL3	ADAMTS-like 3	CCDS10326.1	chr15_84611704-84611704_G_A	787R>Q	Substitution	Nonsynonymous coding	28%
MM12T	ADAMTSL4	ADAMTS-like 4	CCDS955.1	chr1_150530506-150530506__G	NA	Insertion	Frameshift	28%
MM12T	ADAP1	ArfGAP with dual PH domains 1	CCDS5318.1	chr7_938673-938673_C_T	365A>T	Substitution	Nonsynonymous coding	35%
MM12T	ADARB2	adenosine deaminase, RNA-specific, B2	CCDS7058.1	chr10_1263027-1263027_G_A	516R>C	Substitution	Nonsynonymous coding	21%
MM12T	ADARB2	adenosine deaminase, RNA-specific, B2	CCDS7058.1	chr10_1313222-1313222_G_A	374R>C	Substitution	Nonsynonymous coding	40%
MM12T	ADARB2	adenosine deaminase, RNA-specific, B2	CCDS7058.1	chr10_1405345-1405345_C_A	319G>C	Substitution	Nonsynonymous coding	35%
MM12T	ADAT3	adenosine deaminase, tRNA-specific 3	CCDS12076.1	chr19_1912408-1912408_C_T	105S>L	Substitution	Nonsynonymous coding	32%
MM12T	ADCK3	aarF domain containing kinase 3	CCDS1557.1	chr1_227174371-227174371_G_A	626R>H	Substitution	Nonsynonymous coding	33%
MM12T	ADCK5	aarF domain containing kinase 5	CCDS34965.1	chr8_145618058-145618058_C_T	530R>C	Substitution	Nonsynonymous coding	33%
MM12T	ADCY1	adenylate cyclase 1 (brain)	CCDS34631.1	chr7_45632413-45632413_G_A	232R>Q	Substitution	Nonsynonymous coding	11%
MM12T	ADCY10	adenylate cyclase 10 (soluble)	CCDS1265.1	chr1_167815477-167815477_C_A	821S>I	Substitution	Nonsynonymous coding	32%
MM12T	ADCY2	adenylate cyclase 2 (brain)	CCDS3872.2	chr5_7690820-7690820_C_A	246S>Y	Substitution	Nonsynonymous coding	10%
MM12T	ADCY3	adenylate cyclase 3	CCDS1715.1	chr2_25061487-25061487_C_T	454V>M	Substitution	Nonsynonymous coding	13%
MM12T	ADCY4	adenylate cyclase 4	CCDS9627.1	chr14_24789040-24789040_C_A	881D>Y	Substitution	Nonsynonymous coding	24%
MM12T	ADCY4	adenylate cyclase 4	CCDS9627.1	chr14_24798723-24798723_C_A	412G>W	Substitution	Nonsynonymous coding	36%
MM12T	ADCY5	adenylate cyclase 5	CCDS3022.1	chr3_123016228-123016228_C_A	968D>Y	Substitution	Nonsynonymous coding	30%
MM12T	ADCY9	adenylate cyclase 9	CCDS32382.1	chr16_4164885-4164885_T_A	187T>S	Substitution	Nonsynonymous coding	14%
MM12T	ADCY9	adenylate cyclase 9	CCDS32382.1	chr16_4164935-4164935_C_T	170R>Q	Substitution	Nonsynonymous coding	13%
MM12T	ADCY9	adenylate cyclase 9	CCDS32382.1	chr16_4165403-4165403_C_T	14S>N	Substitution	Nonsynonymous coding	44%
MM12T	ADD1	adducin 1 (alpha)	CCDS3363.1	chr4_2906581-2906581_G_T	418G>C	Substitution	Nonsynonymous coding	12%
MM12T	ADH6	alcohol dehydrogenase 6 (class V)	CCDS43255.1	chr4_100126172-100126172_G_A	338A>V	Substitution	Nonsynonymous coding	13%
MM12T	ADNP	activity-dependent neuroprotector homeobox	CCDS13433.1	chr20_49510514-49510514_C_T	246R>H	Substitution	Nonsynonymous coding	21%
MM12T	ADRA1D	adrenoceptor alpha 1D	CCDS13079.1	chr20_4228545-4228545_C_G	354V>L	Substitution	Nonsynonymous coding	29%
MM12T	ADRA1D	adrenoceptor alpha 1D	CCDS13079.1	chr20_4228719-4228719_G_A	296R>X	Substitution	Nonsense	19%
MM12T	ADRB2	adrenoceptor beta 2, surface	CCDS4292.1	chr5_148206743-148206743_G_A	117V>I	Substitution	Nonsynonymous coding	36%

MM12T	ADRB2	adrenoceptor beta 2, surface	CCDS4292.1	chr5_148207518-148207518_A_G	375K>R	Substitution	Nonsynonymous coding	24%
MM12T	ADRBK2	adrenergic, beta, receptor kinase 2	CCDS13832.1	chr22_26086237-26086237_C_T	347P>S	Substitution	Nonsynonymous coding	33%
MM12T	ADRM1	adhesion regulating molecule 1	CCDS13496.1	chr20_60882875-60882875_G_A	283G>S	Substitution	Nonsynonymous coding	30%
MM12T	ADSSL1	adenylosuccinate synthase like 1	CCDS9991.1	chr14_105206132-105206132_G_A	195R>H	Substitution	Nonsynonymous coding	13%
MM12T	ADSSL1	adenylosuccinate synthase like 1	CCDS9991.1	chr14_105208301-105208301_C_T	347R>C	Substitution	Nonsynonymous coding	31%
MM12T	AES	amino-terminal enhancer of split	CCDS12101.1	chr19_3057690-3057690_C_T	126R>H	Substitution	Nonsynonymous coding	33%
MM12T	AFF2	AF4/FMR2 family, member 2	CCDS14684.1	chrX_147924511-147924511_G_A	399G>R	Substitution	Nonsynonymous coding	28%
MM12T	AFTPH	aftiphilin	CCDS46303.1	chr2_64779291-64779291_C_A	228A>D	Substitution	Nonsynonymous coding	21%
MM12T	AGAP1	ArfGAP with GTPase domain, ankyrin repeat and PH domain 1	ENST00000409538	chr2_236579396-236579396_G_T	218S>I	Substitution	Nonsynonymous coding	19%
MM12T	AGAP3	ArfGAP with GTPase domain, ankyrin repeat and PH domain 3	CCDS43681.1	chr7_150820954-150820954_G_A	401R>H	Substitution	Nonsynonymous coding	38%
MM12T	AGAP3	ArfGAP with GTPase domain, ankyrin repeat and PH domain 3	CCDS43681.1	chr7_150835251-150835251_G_T	506S>I	Substitution	Nonsynonymous coding	33%
MM12T	AGBL3	ATP/GTP binding protein-like 3	CCDS47718.1	chr7_134719318-134719318_C_T	326R>C	Substitution	Nonsynonymous coding	35%
MM12T	AGBL4	ATP/GTP binding protein-like 4	CCDS44137.1	chr1_49100240-49100240_C_T	292W>X	Substitution	Nonsense	39%
MM12T	AGL	amylo-alpha-1, 6-glucosidase, 4-alpha-glucanotransferase	CCDS759.1	chr1_100378028-100378028__A	NA	Insertion	Frameshift	25%
MM12T	AGRN	agrin	CCDS30551.1	chr1_978822-978822_C_T	530R>C	Substitution	Nonsynonymous coding	13%
MM12T	AGRN	agrin	CCDS30551.1	chr1_984732-984732_G_A	1472G>D	Substitution	Nonsynonymous coding	34%
MM12T	AGRN	agrin	CCDS30551.1	chr1_989927-989927_G_A	1992W>X	Substitution	Nonsense	11%
MM12T	AGTPBP1	ATP/GTP binding protein 1	CCDS6672.1	chr9_88193951-88193951_C_T	1036V>I	Substitution	Nonsynonymous coding	30%
MM12T	AGXT	alanine-glyoxylate aminotransferase	CCDS2543.1	chr2_241815404-241815404_G_A	277A>T	Substitution	Nonsynonymous coding	17%
MM12T	AHCTF1	AT hook containing transcription factor 1	CCDS1629.1	chr1_247059155-247059155_G_A	566R>X	Substitution	Nonsense	11%
MM12T	AHCY	adenosylhomocysteinase	CCDS13233.1	chr20_32873350-32873350_T_C	355S>G	Substitution	Nonsynonymous coding	31%
MM12T	AHCY	adenosylhomocysteinase	CCDS13233.1	chr20_32878558-32878558_C_T	249A>T	Substitution	Nonsynonymous coding	11%
MM12T	AHCYL2	adenosylhomocysteinase-like 2	CCDS5812.1	chr7_129028981-129028981_G_A	187C>Y	Substitution	Nonsynonymous coding	25%
MM12T	AHDC1	AT hook, DNA binding motif, containing 1	CCDS30652.1	chr1_27876688-27876688_G_A	647H>Y	Substitution	Nonsynonymous coding	33%
MM12T	AHDC1	AT hook, DNA binding motif, containing 1	CCDS30652.1	chr1_27878430-27878430_C_T	66R>H	Substitution	Nonsynonymous coding	24%
MM12T	AHNAK	AHNAK nucleoprotein	CCDS31584.1	chr11_62285796-62285796_G_T	5365L>M	Substitution	Nonsynonymous coding	11%
MM12T	AHNAK	AHNAK nucleoprotein	CCDS31584.1	chr11_62291642-62291642_G_T	3416S>Y	Substitution	Nonsynonymous coding	26%
MM12T	AHNAK	AHNAK nucleoprotein	CCDS31584.1	chr11_62300333-62300333_T_G	519K>T	Substitution	Nonsynonymous coding	34%
MM12T	AHNAK	AHNAK nucleoprotein	CCDS31584.1	chr11_62300931-62300931_G_A	320R>C	Substitution	Nonsynonymous coding	10%
MM12T	AHNAK2	AHNAK nucleoprotein 2	CCDS45177.1	chr14_105404639-105404639_C_T	5717D>N	Substitution	Nonsynonymous coding	31%
MM12T	AK5	adenylate kinase 5	CCDS675.1	chr1_77759510-77759510_C_T	94R>W	Substitution	Nonsynonymous coding	10%
MM12T	AKAP12	A kinase (PRKA) anchor protein 12	CCDS5229.1	chr6_151673894-151673894_A_C	1456K>N	Substitution	Nonsynonymous coding	25%

MM12T	AKAP3	A kinase (PRKA) anchor protein 3	CCDS8531.1	chr12_4735823-4735823_G_T	749P>T	Substitution	Nonsynonymous coding	29%
MM12T	AKAP4	A kinase (PRKA) anchor protein 4	CCDS14329.1	chrX_49958916-49958916_C_A	150A>S	Substitution	Nonsynonymous coding	27%
MM12T	AKAP6	A kinase (PRKA) anchor protein 6	CCDS9644.1	chr14_33014655-33014655_C_T	266P>S	Substitution	Nonsynonymous coding	27%
MM12T	AKAP6	A kinase (PRKA) anchor protein 6	CCDS9644.1	chr14_33293591-33293591_C_T	2191A>V	Substitution	Nonsynonymous coding	23%
MM12T	AKAP8	A kinase (PRKA) anchor protein 8	CCDS12329.1	chr19_15469876-15469876_G_A	ISV-3>	Substitution	Splice site acceptor	14%
MM12T	AKAP9	A kinase (PRKA) anchor protein (yotiao) 9	CCDS5622.1	chr7_91630493-91630493_G_A	421R>Q	Substitution	Nonsynonymous coding	16%
MM12T	AKAP9	A kinase (PRKA) anchor protein (yotiao) 9	CCDS5622.1	chr7_91691662-91691662_C_T	1947R>C	Substitution	Nonsynonymous coding	15%
MM12T	AKD1	adenylate kinase domain containing 1	NM_001145128	chr6_109863332-109863332_A_G	1090S>P	Substitution	Nonsynonymous coding	22%
MM12T	AKR1E2	aldo-keto reductase family 1, member E2	CCDS31134.1	chr10_4875555-4875555_G_A	74C>Y	Substitution	Nonsynonymous coding	28%
MM12T	AKT1	v-akt murine thymoma viral oncogene homolog 1	CCDS9994.1	chr14_105240286-105240286_C_T	222R>H	Substitution	Nonsynonymous coding	10%
MM12T	AKT2	v-akt murine thymoma viral oncogene homolog 2	ENST00000391845	chr19_40746192-40746192_C_T	4V>I	Substitution	Nonsynonymous coding	13%
MM12T	AL033381.1	Uncharacterized protein; cDNA FLJ34594 fis, clone KIDNE2009109	ENST00000314040	chr6_1102380-1102380_G_A	122D>N	Substitution	Nonsynonymous coding	22%
MM12T	AL359195.1	Uncharacterized protein; cDNA FLJ46261 fis, clone TESTI4025062	ENST00000356374	chr10_82012502-82012502_G_A	7G>D	Substitution	Nonsynonymous coding	35%
MM12T	ALCAM	activated leukocyte cell adhesion molecule	CCDS33810.1	chr3_105260570-105260570__A	NA	Insertion	Frameshift	24%
MM12T	ALDH1A2	aldehyde dehydrogenase 1 family, member A2	CCDS10163.1	chr15_58256142-58256142_G_A	343R>W	Substitution	Nonsynonymous coding	15%
MM12T	ALDH1B1	aldehyde dehydrogenase 1 family, member B1	CCDS6615.1	chr9_38396764-38396764_T_C	340V>A	Substitution	Nonsynonymous coding	11%
MM12T	ALDH1L1	aldehyde dehydrogenase 1 family, member L1	CCDS3034.1	chr3_125869343-125869343_C_T	297G>D	Substitution	Nonsynonymous coding	35%
MM12T	ALDH6A1	aldehyde dehydrogenase 6 family, member A1	CCDS9826.1	chr14_74531957-74531957_C_T	444G>E	Substitution	Nonsynonymous coding	17%
MM12T	ALDH7A1	aldehyde dehydrogenase 7 family, member A1	CCDS4137.2	chr5_125930773-125930773_C_T	40A>T	Substitution	Nonsynonymous coding	29%
MM12T	ALDH8A1	aldehyde dehydrogenase 8 family, member A1	CCDS5171.1	chr6_135239923-135239923_G_A	365A>V	Substitution	Nonsynonymous coding	48%
MM12T	ALG1	asparagine-linked glycosylation 1, beta-1,4-mannosyltransferase homolog (S. cerevisiae)	CCDS10528.1	chr16_5125468-5125468_A_G	157H>R	Substitution	Nonsynonymous coding	31%
MM12T	ALG12	asparagine-linked glycosylation 12, alpha-1,6-mannosyltransferase homolog (S. cerevisiae)	CCDS14081.1	chr22_50302920-50302920_G_T	247T>N	Substitution	Nonsynonymous coding	32%
MM12T	ALG3	asparagine-linked glycosylation 3, alpha-1,3-mannosyltransferase homolog (S. cerevisiae)	CCDS46968.1	chr3_183963113-183963113_C_T	160A>T	Substitution	Nonsynonymous coding	12%
MM12T	ALK	anaplastic lymphoma receptor tyrosine kinase	CCDS33172.1	chr2_29420442-29420442_G_A	1347R>W	Substitution	Nonsynonymous coding	21%
MM12T	ALK	anaplastic lymphoma receptor tyrosine kinase	CCDS33172.1	chr2_29430100-29430100_G_A	1292P>L	Substitution	Nonsynonymous coding	29%
MM12T	ALK	anaplastic lymphoma receptor tyrosine kinase	CCDS33172.1	chr2_29917827-29917827_G_A	281H>Y	Substitution	Nonsynonymous coding	27%
MM12T	ALK	anaplastic lymphoma receptor tyrosine kinase	CCDS33172.1	chr2_30142900-30142900_C_T	209R>H	Substitution	Nonsynonymous coding	36%
MM12T	ALK	anaplastic lymphoma receptor tyrosine kinase	CCDS33172.1	chr2_29551349-29551349_T_C	ISV-2>	Substitution	Splice site acceptor	32%
MM12T	ALKBH6	alkB, alkylation repair homolog 6 (E. coli)	CCDS12485.2	chr19_36501849-36501849_G_T	123L>I	Substitution	Nonsynonymous coding	17%
MM12T	ALKBH6	alkB, alkylation repair homolog 6 (E. coli)	CCDS12485.2	chr19_36505076-36505076_C_T	ISV+1>	Substitution	Splice site donor	28%
MM12T	ALMS1	Alstrom syndrome 1	CCDS42697.1	chr2_73717497-73717497_G_A	2803R>H	Substitution	Nonsynonymous coding	33%
MM12T	ALOX12	arachidonate 12-lipoxygenase	CCDS11084.1	chr17_6901831-6901831_G_A	114R>H	Substitution	Nonsynonymous coding	11%

MM12T	ALOX12B	arachidonate 12-lipoxygenase, 12R type	CCDS11129.1	chr17_7976159-7976159_C_T	679R>H	Substitution	Nonsynonymous coding	14%
MM12T	ALOX15B	arachidonate 15-lipoxygenase, type B	CCDS11128.1	chr17_7948885-7948885_C_T	361R>C	Substitution	Nonsynonymous coding	24%
MM12T	ALPK2	alpha-kinase 2	CCDS11966.2	chr18_56247178-56247178_G_T	277S>Y	Substitution	Nonsynonymous coding	18%
MM12T	ALPPL2	alkaline phosphatase, placental-like 2	CCDS2491.1	chr2_233274523-233274523_G_A	514A>T	Substitution	Nonsynonymous coding	18%
MM12T	ALS2	amyotrophic lateral sclerosis 2 (juvenile)	CCDS42800.1	chr2_202574700-202574700_C_T	1395R>K	Substitution	Nonsynonymous coding	31%
MM12T	ALS2	amyotrophic lateral sclerosis 2 (juvenile)	CCDS42800.1	chr2_202575815-202575815_G_A	1341R>C	Substitution	Nonsynonymous coding	11%
MM12T	ALS2CL	ALS2 C-terminal like	CCDS2743.1	chr3_46727071-46727071_C_T	244A>T	Substitution	Nonsynonymous coding	28%
MM12T	ALS2CL	ALS2 C-terminal like	CCDS2743.1	chr3_46730870-46730870_C_T	21A>T	Substitution	Nonsynonymous coding	16%
MM12T	ALS2CR11	amyotrophic lateral sclerosis 2 (juvenile) chromosome region, candidate 11	NM_001168221	chr2_202359041-202359041_G_A	675L>F	Substitution	Nonsynonymous coding	24%
MM12T	AMBN	ameloblastin (enamel matrix protein)	CCDS3543.1	chr4_71472346-71472346_G_T	415D>Y	Substitution	Nonsynonymous coding	12%
MM12T	AMER3	APC membrane recruitment protein 3	CCDS2164.1	chr2_131519883-131519883_G_T	80A>S	Substitution	Nonsynonymous coding	13%
MM12T	AMFR	autocrine motility factor receptor, E3 ubiquitin protein ligase	ENST00000314566	chr16_56396202-56396202_T_C	269T>A	Substitution	Nonsynonymous coding	35%
MM12T	AMH	anti-Mullerian hormone	CCDS12085.1	chr19_2251576-2251576_C_T	435R>C	Substitution	Nonsynonymous coding	36%
MM12T	AMIGO1	adhesion molecule with Ig-like domain 1	CCDS30795.1	chr1_110050345-110050345_C_T	397R>H	Substitution	Nonsynonymous coding	22%
MM12T	AMIGO3	adhesion molecule with Ig-like domain 3	CCDS33759.1	chr3_49756430-49756430_G_A	157R>C	Substitution	Nonsynonymous coding	13%
MM12T	AMN	amnion associated transmembrane protein	CCDS9977.1	chr14_103395095-103395095_G_A	99G>D	Substitution	Nonsynonymous coding	38%
MM12T	AMOT	angiomotin	CCDS48154.1	chrX_112024240-112024240_G_A	783R>W	Substitution	Nonsynonymous coding	29%
MM12T	AMOT	angiomotin	CCDS48154.1	chrX_112024304-112024304_C_T	761M>I	Substitution	Nonsynonymous coding	13%
MM12T	AMOTL1	angiomotin like 1	CCDS44712.1	chr11_94554844-94554844_G_A	424A>T	Substitution	Nonsynonymous coding	24%
MM12T	AMPD3	adenosine monophosphate deaminase 3	CCDS7802.1	chr11_10523021-10523021_C_T	585R>C	Substitution	Nonsynonymous coding	29%
MM12T	AMPH	amphiphysin	CCDS5456.1	chr7_38505073-38505073_G_A	248A>V	Substitution	Nonsynonymous coding	27%
MM12T	AMT	aminomethyltransferase	CCDS2797.1	chr3_49456476-49456476_C_A	269G>C	Substitution	Nonsynonymous coding	17%
MM12T	AMZ2	archaelysin family metallopeptidase 2	CCDS11674.1	chr17_66246588-66246588_G_A	87R>H	Substitution	Nonsynonymous coding	11%
MM12T	ANAPC2	anaphase promoting complex subunit 2	CCDS7033.1	chr9_140069837-140069837_C_T	703G>D	Substitution	Nonsynonymous coding	30%
MM12T	ANAPC5	anaphase promoting complex subunit 5	CCDS9220.1	chr12_121790029-121790029_C_T	39V>M	Substitution	Nonsynonymous coding	12%
MM12T	ANAPC7	anaphase promoting complex subunit 7	CCDS9145.2	chr12_110824131-110824131_C_T	ISV+1>	Substitution	Splice site donor	11%
MM12T	ANGPT4	angiopoietin 4	CCDS13009.1	chr20_860486-860486_G_T	319F>L	Substitution	Nonsynonymous coding	28%
MM12T	ANGPT4	angiopoietin 4	CCDS13009.1	chr20_865780-865780_C_T	259R>H	Substitution	Nonsynonymous coding	16%
MM12T	ANGPTL4	angiopoietin-like 4	CCDS12200.1	chr19_8436182-8436182_G_A	272R>H	Substitution	Nonsynonymous coding	26%
MM12T	ANK1	ankyrin 1, erythrocytic	CCDS6122.1	chr8_41522337-41522337_G_A	69R>W	Substitution	Nonsynonymous coding	50%
MM12T	ANK3	ankyrin 3, node of Ranvier (ankyrin G)	CCDS7258.1	chr10_61815519-61815519_C_T	4321C>Y	Substitution	Nonsynonymous coding	19%
MM12T	ANK3	ankyrin 3, node of Ranvier (ankyrin G)	CCDS7258.1	chr10_61829264-61829264_A_G	3792L>S	Substitution	Nonsynonymous coding	45%

MM12T	ANK3	ankyrin 3, node of Ranvier (ankyrin G)	CCDS7258.1	chr10_61836099-61836099_G_A	1514P>S	Substitution	Nonsynonymous coding	18%
MM12T	ANK3	ankyrin 3, node of Ranvier (ankyrin G)	CCDS7258.1	chr10_61932907-61932907_C_T	741G>E	Substitution	Nonsynonymous coding	39%
MM12T	ANKAR	ankyrin and armadillo repeat containing	CCDS33351.1	chr2_190593419-190593419_G_A	951S>N	Substitution	Nonsynonymous coding	15%
MM12T	ANKDD1A	ankyrin repeat and death domain containing 1A	CCDS10197.2	chr15_65209705-65209705_C_T	87R>C	Substitution	Nonsynonymous coding	32%
MM12T	ANKDD1B	ankyrin repeat and death domain containing 1B	ENST00000344149	chr5_74948998-74948998_G_A	216R>K	Substitution	Nonsynonymous coding	20%
MM12T	ANKDD1B	ankyrin repeat and death domain containing 1B	ENST00000344149	chr5_74955142-74955142_G_A	283R>H	Substitution	Nonsynonymous coding	31%
MM12T	ANKK1	ankyrin repeat and kinase domain containing 1	CCDS44734.1	chr11_113258739-113258739_C_T	45R>W	Substitution	Nonsynonymous coding	19%
MM12T	ANKMY1	ankyrin repeat and MYND domain containing 1	CCDS2536.1	chr2_241465178-241465178_C_A	331S>I	Substitution	Nonsynonymous coding	40%
MM12T	ANKRD11	ankyrin repeat domain 11	CCDS32513.1	chr16_89346682-89346682_C_T	2090A>T	Substitution	Nonsynonymous coding	36%
MM12T	ANKRD11	ankyrin repeat domain 11	CCDS32513.1	chr16_89350738-89350738_G_A	738R>C	Substitution	Nonsynonymous coding	13%
MM12T	ANKRD12	ankyrin repeat domain 12	CCDS11843.1	chr18_9255948-9255948_A_	NA	Deletion	Frameshift	34%
MM12T	ANKRD12	ankyrin repeat domain 12	CCDS11843.1	chr18_9256062-9256062_A_	NA	Deletion	Frameshift	31%
MM12T	ANKRD13A	ankyrin repeat domain 13A	CCDS9140.1	chr12_110457001-110457001_G_A	201R>H	Substitution	Nonsynonymous coding	33%
MM12T	ANKRD13D	ankyrin repeat domain 13 family, member D	CCDS31616.1	chr11_67059154-67059154_C_A	73L>I	Substitution	Nonsynonymous coding	11%
MM12T	ANKRD17	ankyrin repeat domain 17	CCDS34004.1	chr4_73968230-73968230_G_A	1479A>V	Substitution	Nonsynonymous coding	33%
MM12T	ANKRD2	ankyrin repeat domain 2 (stretch responsive muscle)	CCDS7466.1	chr10_99338081-99338081_C_T	119R>W	Substitution	Nonsynonymous coding	11%
MM12T	ANKRD23	ankyrin repeat domain 23	CCDS2027.1	chr2_97505740-97505740_C_A	239Q>H	Substitution	Nonsynonymous coding	32%
MM12T	ANKRD24	ankyrin repeat domain 24	CCDS45925.1	chr19_4222673-4222673_G_T	1060E>X	Substitution	Nonsense	14%
MM12T	ANKRD26	ankyrin repeat domain 26	CCDS41499.1	chr10_27326148-27326148_C_A	888E>D	Substitution	Nonsynonymous coding	55%
MM12T	ANKRD27	ankyrin repeat domain 27 (VPS9 domain)	CCDS32986.1	chr19_33089107-33089107_G_A	1033P>S	Substitution	Nonsynonymous coding	34%
MM12T	ANKRD27	ankyrin repeat domain 27 (VPS9 domain)	CCDS32986.1	chr19_33106643-33106643_G_A	699A>V	Substitution	Nonsynonymous coding	15%
MM12T	ANKRD27	ankyrin repeat domain 27 (VPS9 domain)	CCDS32986.1	chr19_33130321-33130321_T_C	353T>A	Substitution	Nonsynonymous coding	30%
MM12T	ANKRD27	ankyrin repeat domain 27 (VPS9 domain)	CCDS32986.1	chr19_33137429-33137429_A_C	102N>K	Substitution	Nonsynonymous coding	33%
MM12T	ANKRD33	ankyrin repeat domain 33	CCDS44892.1	chr12_52283300-52283300_T_C	ISV+4>	Substitution	Splice site donor	47%
MM12T	ANKRD34B	ankyrin repeat domain 34B	CCDS34194.1	chr5_79855012-79855012_T_C	276E>G	Substitution	Nonsynonymous coding	30%
MM12T	ANKRD35	ankyrin repeat domain 35	CCDS919.1	chr1_145562930-145562930_C_T	873A>V	Substitution	Nonsynonymous coding	33%
MM12T	ANKRD44	ankyrin repeat domain 44	CCDS33355.1	chr2_197990118-197990118_C_T	154W>X	Substitution	Nonsense	25%
MM12T	ANKRD50	ankyrin repeat domain 50	CCDS34060.1	chr4_125591940-125591940_C_T	831R>H	Substitution	Nonsynonymous coding	10%
MM12T	ANKRD52	ankyrin repeat domain 52	CCDS44920.1	chr12_56642565-56642565_C_T	561G>D	Substitution	Nonsynonymous coding	30%
MM12T	ANKRD60	ankyrin repeat domain 60	ENST00000457363	chr20_56803414-56803414_C_T	99R>Q	Substitution	Nonsynonymous coding	30%
MM12T	ANKRD60	ankyrin repeat domain 60	ENST00000457363	chr20_56803471-56803471_G_A	80P>L	Substitution	Nonsynonymous coding	36%
MM12T	ANKRD62	ankyrin repeat domain 62	ENST00000314074	chr18_12094158-12094158_G_A	34A>T	Substitution	Nonsynonymous coding	33%

MM12T	ANKS1A	ankyrin repeat and sterile alpha motif domain containing 1A	CCDS4798.1	chr6_35051200-35051200_A_G	972T>A	Substitution	Nonsynonymous coding	20%
MM12T	ANKS6	ankyrin repeat and sterile alpha motif domain containing 6	CCDS43856.1	chr9_101530476-101530476_C_T	677G>R	Substitution	Nonsynonymous coding	35%
MM12T	ANKS6	ankyrin repeat and sterile alpha motif domain containing 6	CCDS43856.1	chr9_101540676-101540676_G_A	467R>X	Substitution	Nonsense	34%
MM12T	ANKZF1	ankyrin repeat and zinc finger domain containing 1	CCDS42821.1	chr2_220098123-220098123_C_A	263L>M	Substitution	Nonsynonymous coding	41%
MM12T	ANKZF1	ankyrin repeat and zinc finger domain containing 1	CCDS42821.1	chr2_220098551-220098551_G_A	312A>T	Substitution	Nonsynonymous coding	14%
MM12T	ANO1	anoctamin 1, calcium activated chloride channel	CCDS44663.1	chr11_69995812-69995812_G_A	ISV-4>	Substitution	Splice site acceptor	11%
MM12T	ANO5	anoctamin 5	CCDS31444.1	chr11_22239808-22239808_A_T	52N>I	Substitution	Nonsynonymous coding	23%
MM12T	ANO6	anoctamin 6	CCDS31782.1	chr12_45822911-45822911_T_A	850F>L	Substitution	Nonsynonymous coding	14%
MM12T	ANO9	anoctamin 9	CCDS31326.1	chr11_418734-418734_C_T	706V>I	Substitution	Nonsynonymous coding	25%
MM12T	ANO9	anoctamin 9	CCDS31326.1	chr11_418941-418941_C_A	661K>N	Substitution	Nonsynonymous coding	30%
MM12T	ANPEP	alanyl (membrane) aminopeptidase	CCDS10356.1	chr15_90334299-90334299_C_T	852D>N	Substitution	Nonsynonymous coding	11%
MM12T	ANXA1	annexin A1	CCDS6645.1	chr9_75780077-75780077_G_A	220V>M	Substitution	Nonsynonymous coding	10%
MM12T	AP001631.10	-	ENST00000411787	chr21_44581295-44581295_G_T	23S>Y	Substitution	Nonsynonymous coding	25%
MM12T	AP1B1	adaptor-related protein complex 1, beta 1 subunit	ENST00000415756	chr22_29747363-29747363_C_T	30G>D	Substitution	Nonsynonymous coding	14%
MM12T	AP1G2	adaptor-related protein complex 1, gamma 2 subunit	CCDS9602.1	chr14_24032654-24032654__C	NA	Insertion	Frameshift	31%
MM12T	AP1G2	adaptor-related protein complex 1, gamma 2 subunit	CCDS9602.1	chr14_24035494-24035494_C_T	155R>H	Substitution	Nonsynonymous coding	27%
MM12T	AP2A1	adaptor-related protein complex 2, alpha 1 subunit	CCDS46148.1	chr19_50309957-50309957_G_A	959R>H	Substitution	Nonsynonymous coding	12%
MM12T	AP3B2	adaptor-related protein complex 3, beta 2 subunit	CCDS45331.1	chr15_83350280-83350280_C_T	138R>H	Substitution	Nonsynonymous coding	26%
MM12T	AP3S1	adaptor-related protein complex 3, sigma 1 subunit	CCDS4123.1	chr5_115238605-115238605_G_A	123M>I	Substitution	Nonsynonymous coding	25%
MM12T	AP4B1	adaptor-related protein complex 4, beta 1 subunit	CCDS865.1	chr1_114447238-114447238_C_A	34Q>H	Substitution	Nonsynonymous coding	11%
MM12T	AP4E1	adaptor-related protein complex 4, epsilon 1 subunit	CCDS32240.1	chr15_51221214-51221214_T_C	184V>A	Substitution	Nonsynonymous coding	40%
MM12T	AP5Z1	adaptor-related protein complex 5, zeta 1 subunit	CCDS47528.1	chr7_4824622-4824622_C_T	292R>W	Substitution	Nonsynonymous coding	30%
MM12T	APBA3	amyloid beta (A4) precursor protein-binding, family A, member 3	CCDS12110.1	chr19_3753849-3753849_G_A	309R>W	Substitution	Nonsynonymous coding	27%
MM12T	APBB2	amyloid beta (A4) precursor protein-binding, family B, member 2	CCDS43224.1	chr4_40824034-40824034_G_A	633R>X	Substitution	Nonsense	17%
MM12T	APBB3	amyloid beta (A4) precursor protein-binding, family B, member 3	CCDS4227.1	chr5_139941969-139941969_C_T	156R>Q	Substitution	Nonsynonymous coding	24%
MM12T	APC	adenomatous polyposis coli	CCDS4107.1	chr5_112154998-112154998_G_A	423W>X	Substitution	Nonsense	17%
MM12T	APC	adenomatous polyposis coli	CCDS4107.1	chr5_112175639-112175639_C_T	1450R>X	Substitution	Nonsense	40%
MM12T	APC2	adenomatosis polyposis coli 2	CCDS12068.1	chr19_1466287-1466287_C_T	996S>L	Substitution	Nonsynonymous coding	16%
MM12T	APC2	adenomatosis polyposis coli 2	CCDS12068.1	chr19_1468686-1468686_G_A	1796V>I	Substitution	Nonsynonymous coding	33%
MM12T	APLN	apelin	ENST00000427399	chrX_128781732-128781732_G_A	97A>V	Substitution	Nonsynonymous coding	15%
MM12T	APLNR	apelin receptor	CCDS7950.1	chr11_57003416-57003416_C_T	355G>S	Substitution	Nonsynonymous coding	27%
MM12T	APLP1	amyloid beta (A4) precursor-like protein 1	CCDS32997.1	chr19_36360590-36360590_G_A	57A>T	Substitution	Nonsynonymous coding	29%

MM12T	APOA4	apolipoprotein A-IV	CCDS31681.1	chr11_116692310-116692310_C_T	155R>H	Substitution	Nonsynonymous coding	36%
MM12T	APOB	apolipoprotein B (including Ag(x) antigen)	CCDS1703.1	chr2_21233757-21233757_C_T	1995D>N	Substitution	Nonsynonymous coding	12%
MM12T	APOBEC1	apolipoprotein B mRNA editing enzyme, catalytic polypeptide 1	CCDS8579.1	chr12_7803644-7803644_G_A	179A>V	Substitution	Nonsynonymous coding	39%
MM12T	APOBEC3D	apolipoprotein B mRNA editing enzyme, catalytic polypeptide-like 3D	CCDS46709.1	chr22_39421265-39421265_G_A	134R>H	Substitution	Nonsynonymous coding	11%
MM12T	APOBEC4	apolipoprotein B mRNA editing enzyme, catalytic polypeptide-like 4 (putative)	CCDS1358.1	chr1_183617843-183617843_G_A	25S>F	Substitution	Nonsynonymous coding	36%
MM12T	APOBR	apolipoprotein B receptor	NM_018690	chr16_28507078-28507078_C_A	239P>H	Substitution	Nonsynonymous coding	36%
MM12T	APOBR	apolipoprotein B receptor	NM_018690	chr16_28509481-28509481_G_A	1003R>H	Substitution	Nonsynonymous coding	37%
MM12T	APOBR	apolipoprotein B receptor	NM_018690	chr16_28509674-28509674_G_T	ISV+4>	Substitution	Splice site donor	30%
MM12T	APOE	apolipoprotein E	CCDS12647.1	chr19_45412041-45412041_G_A	163R>H	Substitution	Nonsynonymous coding	29%
MM12T	APOL5	apolipoprotein L, 5	CCDS13920.1	chr22_36122590-36122590_G_A	159A>T	Substitution	Nonsynonymous coding	17%
MM12T	APRT	adenine phosphoribosyltransferase	CCDS32511.1	chr16_88878234-88878234_A_G	25V>A	Substitution	Nonsynonymous coding	28%
MM12T	AQP2	aquaporin 2 (collecting duct)	CCDS8792.1	chr12_50348441-50348441_C_A	185P>H	Substitution	Nonsynonymous coding	27%
MM12T	AQP5	aquaporin 5	CCDS8793.1	chr12_50357353-50357353_G_A	148A>T	Substitution	Nonsynonymous coding	27%
MM12T	AQP8	aquaporin 8	CCDS10626.1	chr16_25228522-25228522_G_T	6A>S	Substitution	Nonsynonymous coding	12%
MM12T	AR	androgen receptor	CCDS14387.1	chrX_66942750-66942750_C_T	844A>V	Substitution	Nonsynonymous coding	19%
MM12T	ARAP1	ArfGAP with RhoGAP domain, ankyrin repeat and PH domain 1	CCDS41687.1	chr11_72409075-72409075_C_T	873G>D	Substitution	Nonsynonymous coding	26%
MM12T	ARAP1	ArfGAP with RhoGAP domain, ankyrin repeat and PH domain 1	CCDS41687.1	chr11_72437890-72437890_G_A	95P>L	Substitution	Nonsynonymous coding	14%
MM12T	ARAP3	ArfGAP with RhoGAP domain, ankyrin repeat and PH domain 3	CCDS4266.1	chr5_141050147-141050147_C_A	680S>I	Substitution	Nonsynonymous coding	31%
MM12T	ARCN1	archain 1	CCDS8400.1	chr11_118463431-118463431_C_T	331P>L	Substitution	Nonsynonymous coding	11%
MM12T	ARFGAP1	ADP-ribosylation factor GTPase activating protein 1	ENST00000370275	chr20_61917513-61917513_G_A	339R>H	Substitution	Nonsynonymous coding	31%
MM12T	ARFGEF2	ADP-ribosylation factor guanine nucleotide-exchange factor 2 (brefeldin A-inhibited)	CCDS13411.1	chr20_47607573-47607573_A_G	ISV-4>	Substitution	Splice site acceptor	45%
MM12T	ARFRP1	ADP-ribosylation factor related protein 1	ENST00000303260	chr20_62337070-62337070_C_A	101D>Y	Substitution	Nonsynonymous coding	11%
MM12T	ARHGAP1	Rho GTPase activating protein 1	CCDS7922.1	chr11_46700940-46700940_C_T	369R>H	Substitution	Nonsynonymous coding	22%
MM12T	ARHGAP10	Rho GTPase activating protein 10	CCDS34075.1	chr4_148944432-148944432_G_A	579D>N	Substitution	Nonsynonymous coding	18%
MM12T	ARHGAP11A	Rho GTPase activating protein 11A	CCDS10028.1	chr15_32929599-32929599_C_A	875S>R	Substitution	Nonsynonymous coding	14%
MM12T	ARHGAP19	Rho GTPase activating protein 19	CCDS7454.2	chr10_99019348-99019348_A_T	217N>K	Substitution	Nonsynonymous coding	12%
MM12T	ARHGAP25	Rho GTPase activating protein 25	CCDS46312.1	chr2_69002329-69002329_C_T	6S>L	Substitution	Nonsynonymous coding	31%
MM12T	ARHGAP25	Rho GTPase activating protein 25	CCDS33214.1	chr2_69049745-69049745_C_T	491R>C	Substitution	Nonsynonymous coding	24%
MM12T	ARHGAP30	Rho GTPase activating protein 30	CCDS30918.1	chr1_161022229-161022229_C_T	ISV+1>	Substitution	Splice site donor	36%
MM12T	ARHGAP30	Rho GTPase activating protein 30	CCDS30918.1	chr1_161021311-161021311_C_A	405E>X	Substitution	Nonsense	30%
MM12T	ARHGAP31	Rho GTPase activating protein 31	CCDS43135.1	chr3_119133892-119133892_G_A	1039S>N	Substitution	Nonsynonymous coding	31%
MM12T	ARHGAP31	Rho GTPase activating protein 31	CCDS43135.1	chr3_119134692-119134692_C_T	1306Q>X	Substitution	Nonsense	11%

MM12T	ARHGAP32	Rho GTPase activating protein 32	CCDS44769.1	chr11_128842868-128842868_G_A	1164A>V	Substitution	Nonsynonymous coding	15%
MM12T	ARHGAP33	Rho GTPase activating protein 33	ENST00000007510	chr19_36277338-36277338_C_T	656R>W	Substitution	Nonsynonymous coding	48%
MM12T	ARHGAP35	Rho GTPase activating protein 35	CCDS46127.1	chr19_47422965-47422965_C_T	345P>S	Substitution	Nonsynonymous coding	17%
MM12T	ARHGAP36	Rho GTPase activating protein 36	CCDS14628.1	chrX_130217932-130217932_C_T	182R>C	Substitution	Nonsynonymous coding	63%
MM12T	ARHGAP39	Rho GTPase activating protein 39	CCDS34971.1	chr8_145756217-145756217_G_A	1007P>S	Substitution	Nonsynonymous coding	45%
MM12T	ARHGAP40	Rho GTPase activating protein 40	NM_001164431	chr20_37266404-37266404_G_A	334V>I	Substitution	Nonsynonymous coding	10%
MM12T	ARHGAP40	Rho GTPase activating protein 40	NM_001164431	chr20_37274763-37274763_C_T	547A>V	Substitution	Nonsynonymous coding	31%
MM12T	ARHGAP5	Rho GTPase activating protein 5	CCDS32062.1	chr14_32563048-32563048__T	NA	Insertion	Frameshift	27%
MM12T	ARHGAP5	Rho GTPase activating protein 5	CCDS32062.1	chr14_32563413-32563413_G_A	1180A>T	Substitution	Nonsynonymous coding	21%
MM12T	ARHGAP6	Rho GTPase activating protein 6	CCDS14140.1	chrX_11157302-11157302_C_A	869R>I	Substitution	Nonsynonymous coding	26%
MM12T	ARHGAP6	Rho GTPase activating protein 6	CCDS14140.1	chrX_11204397-11204397_G_T	411P>H	Substitution	Nonsynonymous coding	11%
MM12T	ARHGEF10	Rho guanine nucleotide exchange factor (GEF) 10	CCDS34794.1	chr8_1833845-1833845_C_T	385P>L	Substitution	Nonsynonymous coding	25%
MM12T	ARHGEF10L	Rho guanine nucleotide exchange factor (GEF) 10-like	CCDS182.1	chr1_18014167-18014167_C_T	1037R>C	Substitution	Nonsynonymous coding	41%
MM12T	ARHGEF10L	Rho guanine nucleotide exchange factor (GEF) 10-like	CCDS182.1	chr1_17907130-17907130_A_G	ISV+3>	Substitution	Splice site donor	36%
MM12T	ARHGEF11	Rho guanine nucleotide exchange factor (GEF) 11	CCDS1163.1	chr1_156917153-156917153_G_A	811P>S	Substitution	Nonsynonymous coding	11%
MM12T	ARHGEF11	Rho guanine nucleotide exchange factor (GEF) 11	CCDS1163.1	chr1_156931470-156931470_G_A	413A>V	Substitution	Nonsynonymous coding	20%
MM12T	ARHGEF11	Rho guanine nucleotide exchange factor (GEF) 11	CCDS1163.1	chr1_156955955-156955955_G_T	15L>M	Substitution	Nonsynonymous coding	24%
MM12T	ARHGEF17	Rho guanine nucleotide exchange factor (GEF) 17	CCDS8221.1	chr11_73021058-73021058_C_T	459R>W	Substitution	Nonsynonymous coding	37%
MM12T	ARHGEF17	Rho guanine nucleotide exchange factor (GEF) 17	CCDS8221.1	chr11_73073191-73073191_G_A	1534R>H	Substitution	Nonsynonymous coding	10%
MM12T	ARHGEF18	Rho/Rac guanine nucleotide exchange factor (GEF) 18	CCDS45946.1	chr19_7532428-7532428_G_A	925R>H	Substitution	Nonsynonymous coding	13%
MM12T	ARHGEF19	Rho guanine nucleotide exchange factor (GEF) 19	CCDS170.1	chr1_16532780-16532780_G_A	398A>V	Substitution	Nonsynonymous coding	37%
MM12T	ARHGEF3	Rho guanine nucleotide exchange factor (GEF) 3	CCDS46854.1	chr3_56779397-56779397_C_A	268D>Y	Substitution	Nonsynonymous coding	25%
MM12T	ARHGEF3	Rho guanine nucleotide exchange factor (GEF) 3	CCDS46854.1	chr3_56807811-56807811_G_A	76R>X	Substitution	Nonsense	29%
MM12T	ARHGEF37	Rho guanine nucleotide exchange factor (GEF) 37	CCDS43385.1	chr5_148999959-148999959_C_A	313P>T	Substitution	Nonsynonymous coding	41%
MM12T	ARHGEF38	Rho guanine nucleotide exchange factor (GEF) 38	ENST00000420470	chr4_106566370-106566370_G_A	34G>S	Substitution	Nonsynonymous coding	15%
MM12T	ARHGEF4	Rho guanine nucleotide exchange factor (GEF) 4	ENST00000409359	chr2_131672915-131672915_C_G	136P>A	Substitution	Nonsynonymous coding	33%
MM12T	ARHGEF7	Rho guanine nucleotide exchange factor (GEF) 7	CCDS45068.1	chr13_111862350-111862350_G_C	ISV+1>	Substitution	Splice site donor	29%
MM12T	ARHGEF9	Cdc42 guanine nucleotide exchange factor (GEF) 9	CCDS35315.1	chrX_62863892-62863892_G_A	446A>V	Substitution	Nonsynonymous coding	24%
MM12T	ARID1A	AT rich interactive domain 1A (SWI-like)	CCDS285.1	chr1_27092812-27092812_G_A	945G>R	Substitution	Nonsynonymous coding	13%
MM12T	ARID1B	AT rich interactive domain 1B (SWI1-like)	CCDS5251.1	chr6_157528243-157528243_C_T	1972R>X	Substitution	Nonsense	23%
MM12T	ARID4B	AT rich interactive domain 4B (RBP1-like)	CCDS31061.1	chr1_235344908-235344908_G_T	1109P>H	Substitution	Nonsynonymous coding	17%
MM12T	ARID5A	AT rich interactive domain 5A (MRF1-like)	CCDS33251.1	chr2_97215934-97215934_C_T	108R>C	Substitution	Nonsynonymous coding	38%

MM12T	ARID5A	AT rich interactive domain 5A (MRF1-like)	CCDS33251.1	chr2_97216960-97216960_C_T	232A>V	Substitution	Nonsynonymous coding	12%
MM12T	ARIH2	ariadne homolog 2 (Drosophila)	CCDS2780.1	chr3_49020686-49020686_G_A	489D>N	Substitution	Nonsynonymous coding	35%
MM12T	ARL1	ADP-ribosylation factor-like 1	CCDS44958.1	chr12_101790198-101790198_C_A	165G>V	Substitution	Nonsynonymous coding	18%
MM12T	ARL11	ADP-ribosylation factor-like 11	CCDS9419.1	chr13_50204958-50204958_G_T	125E>D	Substitution	Nonsynonymous coding	16%
MM12T	ARL13B	ADP-ribosylation factor-like 13B	CCDS2925.1	chr3_93754216-93754216_C_T	141A>V	Substitution	Nonsynonymous coding	13%
MM12T	ARMC4	armadillo repeat containing 4	CCDS7157.1	chr10_28228838-28228838_C_T	695M>I	Substitution	Nonsynonymous coding	12%
MM12T	ARMC7	armadillo repeat containing 7	CCDS11714.1	chr17_73124952-73124952_C_T	139T>M	Substitution	Nonsynonymous coding	34%
MM12T	ARMCX5	armadillo repeat containing, X-linked 5	CCDS14500.1	chrX_101857581-101857581_G_A	171C>Y	Substitution	Nonsynonymous coding	21%
MM12T	ARPC1A	actin related protein 2/3 complex, subunit 1A, 41kDa	CCDS5660.1	chr7_98961185-98961185_G_A	335V>M	Substitution	Nonsynonymous coding	38%
MM12T	ARPC1B	actin related protein 2/3 complex, subunit 1B, 41kDa	CCDS5661.1	chr7_98985689-98985689_G_A	66R>H	Substitution	Nonsynonymous coding	18%
MM12T	ARPC5L	actin related protein 2/3 complex, subunit 5-like	CCDS6859.1	chr9_127636005-127636005_C_T	62R>W	Substitution	Nonsynonymous coding	31%
MM12T	ARPP21	cAMP-regulated phosphoprotein, 21kDa	CCDS2661.1	chr3_35748578-35748578_G_A	ISV+4>	Substitution	Splice site donor	30%
MM12T	ARRB1	arrestin, beta 1	CCDS44684.1	chr11_75001070-75001070_C_T	12A>T	Substitution	Nonsynonymous coding	24%
MM12T	ARRDC1	arrestin domain containing 1	CCDS7049.1	chr9_140508108-140508108_G_A	108V>M	Substitution	Nonsynonymous coding	28%
MM12T	ARSB	arylsulfatase B	CCDS43334.1	chr5_78111924-78111924_G_T	409C>X	Substitution	Nonsense	20%
MM12T	ARSD	arylsulfatase D	CCDS35196.1	chrX_2825619-2825619_G_A	492A>V	Substitution	Nonsynonymous coding	17%
MM12T	ARSI	arylsulfatase family, member I	CCDS34275.1	chr5_149677471-149677471_C_T	339R>Q	Substitution	Nonsynonymous coding	11%
MM12T	ARVCF	armadillo repeat gene deleted in velocardiofacial syndrome	CCDS13771.1	chr22_19960652-19960652_C_T	810V>M	Substitution	Nonsynonymous coding	34%
MM12T	ARVCF	armadillo repeat gene deleted in velocardiofacial syndrome	CCDS13771.1	chr22_19966469-19966469_G_A	511R>W	Substitution	Nonsynonymous coding	17%
MM12T	ARVCF	armadillo repeat gene deleted in velocardiofacial syndrome	CCDS13771.1	chr22_19968887-19968887_C_T	248R>H	Substitution	Nonsynonymous coding	18%
MM12T	ARX	aristaless related homeobox	CCDS14215.1	chrX_25022849-25022849_C_T	543A>T	Substitution	Nonsynonymous coding	11%
MM12T	ASAP1	ArfGAP with SH3 domain, ankyrin repeat and PH domain 1	CCDS6362.1	chr8_131073147-131073147_G_T	957P>H	Substitution	Nonsynonymous coding	28%
MM12T	ASAP2	ArfGAP with SH3 domain, ankyrin repeat and PH domain 2	CCDS1661.1	chr2_9463282-9463282_C_T	168A>V	Substitution	Nonsynonymous coding	12%
MM12T	ASB16	ankyrin repeat and SOCS box containing 16	CCDS11478.1	chr17_42254285-42254285_C_T	250A>V	Substitution	Nonsynonymous coding	11%
MM12T	ASB18	ankyrin repeat and SOCS box containing 18	CCDS46548.1	chr2_237123016-237123016_C_T	364A>T	Substitution	Nonsynonymous coding	21%
MM12T	ASB4	ankyrin repeat and SOCS box containing 4	CCDS5641.1	chr7_95157275-95157275_C_T	213A>V	Substitution	Nonsynonymous coding	37%
MM12T	ASB6	ankyrin repeat and SOCS box containing 6	CCDS6924.1	chr9_132400416-132400416_G_T	307L>I	Substitution	Nonsynonymous coding	30%
MM12T	ASB7	ankyrin repeat and SOCS box containing 7	CCDS10387.1	chr15_101169983-101169983_C_T	185R>X	Substitution	Nonsense	11%
MM12T	ASCC2	activating signal cointegrator 1 complex subunit 2	CCDS13869.1	chr22_30197067-30197067_G_A	534T>M	Substitution	Nonsynonymous coding	18%
MM12T	ASCC3	activating signal cointegrator 1 complex subunit 3	CCDS5046.1	chr6_101109882-101109882_T_	NA	Deletion	Frameshift	14%
MM12T	ASCL1	achaete-scute complex homolog 1 (Drosophila)	CCDS31886.1	chr12_103352525-103352525_G_A	168R>H	Substitution	Nonsynonymous coding	18%
MM12T	ASF1A	ASF1 anti-silencing function 1 homolog A (S. cerevisiae)	CCDS47469.1	chr6_119228600-119228600_G_A	146V>I	Substitution	Nonsynonymous coding	20%

MM12T	ASF1B	ASF1 anti-silencing function 1 homolog B (S. cerevisiae)	CCDS12306.1	chr19_14247162-14247162_T_C	36D>G	Substitution	Nonsynonymous coding	10%
MM12T	ASH1L	ash1 (absent, small, or homeotic)-like (Drosophila)	CCDS1113.2	chr1_155429621-155429621_T_C	1685K>E	Substitution	Nonsynonymous coding	32%
MM12T	ASH1L	ash1 (absent, small, or homeotic)-like (Drosophila)	CCDS1113.2	chr1_155449035-155449035_C_T	1209S>N	Substitution	Nonsynonymous coding	29%
MM12T	ASIC1	acid-sensing (proton-gated) ion channel 1	CCDS8796.1	chr12_50472298-50472298_G_A	311R>H	Substitution	Nonsynonymous coding	34%
MM12T	ASIC3	acid-sensing (proton-gated) ion channel 3	CCDS5915.1	chr7_150749251-150749251_A_G	ISV-2>	Substitution	Splice site acceptor	32%
MM12T	ASIC4	acid-sensing (proton-gated) ion channel family member 4	CCDS33384.1	chr2_220379541-220379541_C_T	159A>V	Substitution	Nonsynonymous coding	10%
MM12T	ASPH	aspartate beta-hydroxylase	CCDS34898.1	chr8_62489337-62489337_C_A	381K>N	Substitution	Nonsynonymous coding	25%
MM12T	ASPHD2	aspartate beta-hydroxylase domain containing 2	CCDS13834.2	chr22_26838512-26838512_C_T	325S>F	Substitution	Nonsynonymous coding	25%
MM12T	ASPN	asporin	CCDS6697.1	chr9_95227296-95227296_G_T	206P>H	Substitution	Nonsynonymous coding	19%
MM12T	ASPSCR1	alveolar soft part sarcoma chromosome region, candidate 1	CCDS11796.1	chr17_79941524-79941524_C_T	85R>C	Substitution	Nonsynonymous coding	26%
MM12T	ASPSCR1	alveolar soft part sarcoma chromosome region, candidate 1	CCDS11796.1	chr17_79954495-79954495_G_A	236A>T	Substitution	Nonsynonymous coding	21%
MM12T	ASTN1	astrotactin 1	CCDS1319.1	chr1_177001804-177001804_C_T	218R>H	Substitution	Nonsynonymous coding	16%
MM12T	ASTN2	astrotactin 2	CCDS48009.1	chr9_119188319-119188319_G_T	1273C>X	Substitution	Nonsense	31%
MM12T	ASXL1	additional sex combs like 1 (Drosophila)	CCDS13201.1	chr20_31022231-31022231_G_A	ISV-4>	Substitution	Splice site acceptor	31%
MM12T	ASXL3	additional sex combs like 3 (Drosophila)	CCDS45847.1	chr18_31324170-31324170_C_A	1453P>H	Substitution	Nonsynonymous coding	33%
MM12T	ATAD2B	ATPase family, AAA domain containing 2B	CCDS46227.1	chr2_24046305-24046305_C_T	652D>N	Substitution	Nonsynonymous coding	15%
MM12T	ATE1	arginyltransferase 1	CCDS31299.1	chr10_123600655-123600655_A_G	367Y>H	Substitution	Nonsynonymous coding	24%
MM12T	ATE1	arginyltransferase 1	CCDS31299.1	chr10_123658406-123658406_A_G	298Y>H	Substitution	Nonsynonymous coding	11%
MM12T	ATF7	activating transcription factor 7	CCDS44905.1	chr12_53918499-53918499_C_T	336R>H	Substitution	Nonsynonymous coding	12%
MM12T	ATF7IP	activating transcription factor 7 interacting protein	CCDS8663.1	chr12_14577118-14577118_A_	NA	Deletion	Frameshift	12%
MM12T	ATG13	autophagy related 13	CCDS44582.1	chr11_46671756-46671756_C_T	116T>M	Substitution	Nonsynonymous coding	28%
MM12T	ATG2A	autophagy related 2A	CCDS31602.1	chr11_64676761-64676761_C_T	729R>H	Substitution	Nonsynonymous coding	11%
MM12T	ATG4B	autophagy related 4B, cysteine peptidase	CCDS46564.1	chr2_242607996-242607996_G_A	285A>T	Substitution	Nonsynonymous coding	46%
MM12T	ATG7	autophagy related 7	CCDS2605.1	chr3_11402068-11402068_C_T	498A>V	Substitution	Nonsynonymous coding	22%
MM12T	ATHL1	ATH1, acid trehalase-like 1 (yeast)	CCDS31322.2	chr11_294180-294180_G_A	598G>R	Substitution	Nonsynonymous coding	34%
MM12T	ATIC	5-aminoimidazole-4-carboxamide ribonucleotide formyltransferase/IMP	CCDS2398.1	chr2_216182924-216182924_G_A	64R>H	Substitution	Nonsynonymous coding	15%
MM12T	ATL2	atlastin GTPase 2	CCDS46260.1	chr2_38570444-38570444_A_G	110L>P	Substitution	Nonsynonymous coding	14%
MM12T	ATL3	atlastin GTPase 3	ENST00000332645	chr11_63438813-63438813_C_A	51G>C	Substitution	Nonsynonymous coding	21%
MM12T	ATN1	atrophin 1 [Source:HGNC Symbol;Acc:3033]	CCDS31734.1	chr12_7045241-7045241_C_A	271P>T	Substitution	Nonsynonymous coding	39%
MM12T	ATN1	atrophin 1 [Source:HGNC Symbol;Acc:3033]	CCDS31734.1	chr12_7046127-7046127_C_A	566P>H	Substitution	Nonsynonymous coding	20%
MM12T	ATP10A	ATPase, class V, type 10A	CCDS32178.1	chr15_25924948-25924948_G_T	1347S>Y	Substitution	Nonsynonymous coding	34%
MM12T	ATP10A	ATPase, class V, type 10A	CCDS32178.1	chr15_25940196-25940196_C_A	953R>M	Substitution	Nonsynonymous coding	31%

MM12T	ATP10A	ATPase, class V, type 10A	CCDS32178.1	chr15_25958905-25958905_G_A	754R>W	Substitution	Nonsynonymous coding	32%
MM12T	ATP10A	ATPase, class V, type 10A	CCDS32178.1	chr15_25959081-25959081_G_A	695A>V	Substitution	Nonsynonymous coding	39%
MM12T	ATP10D	ATPase, class V, type 10D	CCDS3476.1	chr4_47560276-47560276_C_T	807S>L	Substitution	Nonsynonymous coding	34%
MM12T	ATP11A	ATPase, class VI, type 11A	CCDS32011.1	chr13_113510224-113510224_G_T	ISV-1>	Substitution	Splice site acceptor	30%
MM12T	ATP11B	ATPase, class VI, type 11B	CCDS33896.1	chr3_182607205-182607205_A_T	951S>C	Substitution	Nonsynonymous coding	14%
MM12T	ATP11B	ATPase, class VI, type 11B	CCDS33896.1	chr3_182554963-182554963_G_A	ISV+1>	Substitution	Splice site donor	20%
MM12T	ATP11C	ATPase, class VI, type 11C	CCDS14668.1	chrX_138840030-138840030_A_	NA	Deletion	Splice site acceptor	43%
MM12T	ATP13A1	ATPase type 13A1	CCDS32970.2	chr19_19764607-19764607_G_T	696L>I	Substitution	Nonsynonymous coding	29%
MM12T	ATP13A2	ATPase type 13A2	CCDS175.1	chr1_17313384-17313384_A_G	1051F>L	Substitution	Nonsynonymous coding	30%
MM12T	ATP13A4	ATPase type 13A4	CCDS3304.2	chr3_193182764-193182764_C_T	476V>I	Substitution	Nonsynonymous coding	26%
MM12T	ATP1A1	ATPase, Na+/K+ transporting, alpha 1 polypeptide	CCDS887.1	chr1_116930114-116930114_G_A	ISV+1>	Substitution	Splice site donor	11%
MM12T	ATP1A2	ATPase, Na+/K+ transporting, alpha 2 polypeptide	CCDS1196.1	chr1_160097414-160097414_G_A	274G>D	Substitution	Nonsynonymous coding	15%
MM12T	ATP1A2	ATPase, Na+/K+ transporting, alpha 2 polypeptide	CCDS1196.1	chr1_160104982-160104982_C_T	671T>I	Substitution	Nonsynonymous coding	32%
MM12T	ATP1A4	ATPase, Na+/K+ transporting, alpha 4 polypeptide	CCDS1197.1	chr1_160146396-160146396_C_T	ISV+4>	Substitution	Splice site donor	24%
MM12T	ATP1B2	ATPase, Na+/K+ transporting, beta 2 polypeptide	CCDS32550.1	chr17_7556803-7556803_A_G	75D>G	Substitution	Nonsynonymous coding	46%
MM12T	ATP2A1	ATPase, Ca++ transporting, cardiac muscle, fast twitch 1	CCDS10643.1	chr16_28893848-28893848_G_A	134R>H	Substitution	Nonsynonymous coding	14%
MM12T	ATP2A1	ATPase, Ca++ transporting, cardiac muscle, fast twitch 1	CCDS10643.1	chr16_28911986-28911986_G_A	617A>T	Substitution	Nonsynonymous coding	13%
MM12T	ATP2C2	ATPase, Ca++ transporting, type 2C, member 2	CCDS42207.1	chr16_84472842-84472842_C_T	353R>W	Substitution	Nonsynonymous coding	10%
MM12T	ATP5C1	ATP synthase, H+ transporting, mitochondrial F1 complex, gamma polypeptide 1	CCDS31142.1	chr10_7844783-7844783_G_T	286E>X	Substitution	Nonsense	31%
MM12T	ATP5G3	ATP synthase, H+ transporting, mitochondrial Fo complex, subunit C3 (subunit 9)	CCDS2263.1	chr2_176044851-176044851_C_T	32R>Q	Substitution	Nonsynonymous coding	23%
MM12T	ATP6V0A4	ATPase, H+ transporting, lysosomal V0 subunit a4	CCDS5849.1	chr7_138417717-138417717_C_T	605V>I	Substitution	Nonsynonymous coding	22%
MM12T	ATP6V0A4	ATPase, H+ transporting, lysosomal V0 subunit a4	CCDS5849.1	chr7_138429870-138429870_C_T	492W>X	Substitution	Nonsense	39%
MM12T	ATP6V1A	ATPase, H+ transporting, lysosomal 70kDa, V1 subunit A	CCDS2976.1	chr3_113503628-113503628_G_A	171R>Q	Substitution	Nonsynonymous coding	32%
MM12T	ATP7A	ATPase, Cu++ transporting, alpha polypeptide	CCDS35339.1	chrX_77302032-77302032_G_A	1490G>R	Substitution	Nonsynonymous coding	29%
MM12T	ATP7B	ATPase, Cu++ transporting, beta polypeptide	CCDS41892.1	chr13_52513207-52513207_C_T	1227A>T	Substitution	Nonsynonymous coding	12%
MM12T	ATP7B	ATPase, Cu++ transporting, beta polypeptide	CCDS41892.1	chr13_52520556-52520556_G_A	975S>F	Substitution	Nonsynonymous coding	30%
MM12T	ATP7B	ATPase, Cu++ transporting, beta polypeptide	CCDS41892.1	chr13_52532535-52532535_G_A	756A>V	Substitution	Nonsynonymous coding	29%
MM12T	ATP8A1	ATPase, aminophospholipid transporter (APLT), class I, type 8A, member 1	CCDS3466.1	chr4_42416721-42416721_G_A	1107A>V	Substitution	Nonsynonymous coding	13%
MM12T	ATR	ataxia telangiectasia and Rad3 related	CCDS3124.1	chr3_142232468-142232468_G_T	1506L>I	Substitution	Nonsynonymous coding	13%
MM12T	ATXN10	ataxin 10	CCDS14070.1	chr22_46088876-46088876_G_T	103R>S	Substitution	Nonsynonymous coding	19%
MM12T	ATXN10	ataxin 10	CCDS14070.1	chr22_46088913-46088913_C_T	116L>F	Substitution	Nonsynonymous coding	14%
MM12T	ATXN2	ataxin 2	ENST00000389154	chr12_111890598-111890598_G_A	405P>L	Substitution	Nonsynonymous coding	33%

MM12T	ATXN2	ataxin 2	CCDS31902.1	chr12_111926303-111926303_C_A	899K>N	Substitution	Nonsynonymous coding	23%
MM12T	ATXN2L	ataxin 2-like	CCDS10640.1	chr16_28847369-28847369_C_T	1004A>V	Substitution	Nonsynonymous coding	12%
MM12T	ATXN7L1	ataxin 7-like 1	CCDS47682.1	chr7_105254395-105254395_G_A	796P>S	Substitution	Nonsynonymous coding	11%
MM12T	AURKC	aurora kinase C	CCDS33128.1	chr19_57743402-57743402_C_T	36R>W	Substitution	Nonsynonymous coding	23%
MM12T	AVPR1A	arginine vasopressin receptor 1A	CCDS8965.1	chr12_63541343-63541343_A_	NA	Deletion	Frameshift	33%
MM12T	AVPR2	arginine vasopressin receptor 2	CCDS14735.1	chrX_153172010-153172010_G_A	315S>N	Substitution	Nonsynonymous coding	10%
MM12T	AXIN2	axin 2	CCDS11662.1	chr17_63545734-63545734_G_A	287S>F	Substitution	Nonsynonymous coding	14%
MM12T	AXL	AXL receptor tyrosine kinase	CCDS12575.1	chr19_41748902-41748902_A_C	476K>T	Substitution	Nonsynonymous coding	37%
MM12T	AXL	AXL receptor tyrosine kinase	CCDS12575.1	chr19_41758798-41758798_G_A	618V>I	Substitution	Nonsynonymous coding	19%
MM12T	AZI1	5-azacytidine induced 1	CCDS45808.1	chr17_79170789-79170789_G_A	572R>W	Substitution	Nonsynonymous coding	25%
MM12T	B3GALT6	UDP-Gal:betaGal beta 1,3-galactosyltransferase polypeptide 6	CCDS13.1	chr1_1168512-1168512_A_G	285D>G	Substitution	Nonsynonymous coding	36%
MM12T	B3GNT2	UDP-GlcNAc:betaGal beta-1,3-N-acetylglucosaminyltransferase 2	CCDS1870.1	chr2_62450470-62450470_T_C	372L>P	Substitution	Nonsynonymous coding	33%
MM12T	B3GNT9	UDP-GlcNAc:betaGal beta-1,3-N-acetylglucosaminyltransferase 9	CCDS45509.1	chr16_67183440-67183440_C_T	317V>I	Substitution	Nonsynonymous coding	28%
MM12T	B4GALNT2	beta-1,4-N-acetyl-galactosaminyl transferase 2	CCDS11544.1	chr17_47246123-47246123_G_T	452K>N	Substitution	Nonsynonymous coding	31%
MM12T	B4GALNT4	beta-1,4-N-acetyl-galactosaminyl transferase 4	CCDS7694.1	chr11_380019-380019_G_A	881R>Q	Substitution	Nonsynonymous coding	35%
MM12T	B4GALT7	xylosylprotein beta 1,4-galactosyltransferase, polypeptide 7	CCDS4429.1	chr5_177036001-177036001_G_A	272A>T	Substitution	Nonsynonymous coding	13%
MM12T	BAAT	bile acid CoA: amino acid N-acyltransferase (glycine N-choloyltransferase)	CCDS6752.1	chr9_104125077-104125077_C_T	297R>H	Substitution	Nonsynonymous coding	26%
MM12T	BACE1	beta-site APP-cleaving enzyme 1	CCDS8383.1	chr11_117164718-117164718_C_T	192D>N	Substitution	Nonsynonymous coding	13%
MM12T	BACH1	BTB and CNC homology 1, basic leucine zipper transcription factor 1	ENST00000422809	chr21_30969991-30969991_G_T	205R>I	Substitution	Nonsynonymous coding	15%
MM12T	BAG1	BCL2-associated athanogene	CCDS35004.1	chr9_33264527-33264527_G_A	49A>V	Substitution	Nonsynonymous coding	17%
MM12T	BAG3	BCL2-associated athanogene 3	CCDS7615.1	chr10_121436172-121436172_C_T	369A>V	Substitution	Nonsynonymous coding	15%
MM12T	BAHCC1	BAH domain and coiled-coil containing 1 [Source:HGNC Symbol;Acc:29279]	ENST00000436173	chr17_79414665-79414665_C_T	1256A>V	Substitution	Nonsynonymous coding	24%
MM12T	BAHD1	bromo adjacent homology domain containing 1	CCDS10058.1	chr15_40751416-40751416_G_T	251K>N	Substitution	Nonsynonymous coding	31%
MM12T	BAI1	brain-specific angiogenesis inhibitor 1	NM_001702	chr8_143602200-143602200_C_T	980R>C	Substitution	Nonsynonymous coding	28%
MM12T	BAI1	brain-specific angiogenesis inhibitor 1	NM_001702	chr8_143618427-143618427_G_A	1217G>D	Substitution	Nonsynonymous coding	35%
MM12T	BAI1	brain-specific angiogenesis inhibitor 1	NM_001702	chr8_143623950-143623950_G_T	1452S>I	Substitution	Nonsynonymous coding	28%
MM12T	BAI3	brain-specific angiogenesis inhibitor 3	CCDS4968.1	chr6_69665964-69665964_C_T	415T>M	Substitution	Nonsynonymous coding	39%
MM12T	BAI3	brain-specific angiogenesis inhibitor 3	CCDS4968.1	chr6_69723957-69723957_C_A	653L>I	Substitution	Nonsynonymous coding	48%
MM12T	BAIAP2L2	BAI1-associated protein 2-like 2	CCDS43018.1	chr22_38482343-38482343_C_T	458R>Q	Substitution	Nonsynonymous coding	68%
MM12T	BAIAP3	BAI1-associated protein 3	CCDS10434.1	chr16_1391434-1391434_G_T	260Q>H	Substitution	Nonsynonymous coding	10%
MM12T	BAMBI	BMP and activin membrane-bound inhibitor homolog (Xenopus laevis)	CCDS7162.1	chr10_28970385-28970385_C_T	92T>I	Substitution	Nonsynonymous coding	27%
MM12T	BANP	BTG3 associated nuclear protein	CCDS10966.2	chr16_88039844-88039844_G_A	171V>I	Substitution	Nonsynonymous coding	34%

MM12T	BARD1	BRCA1 associated RING domain 1	CCDS2397.1	chr2_215609847-215609847_C_T	616S>N	Substitution	Nonsynonymous coding	21%
MM12T	BAZ1A	bromodomain adjacent to zinc finger domain, 1A	CCDS9651.1	chr14_35234138-35234138_G_A	1184T>I	Substitution	Nonsynonymous coding	31%
MM12T	BAZ1B	bromodomain adjacent to zinc finger domain, 1B	CCDS5549.1	chr7_72873957-72873957_G_A	1114A>V	Substitution	Nonsynonymous coding	24%
MM12T	BAZ1B	bromodomain adjacent to zinc finger domain, 1B	CCDS5549.1	chr7_72891811-72891811_C_A	660Q>H	Substitution	Nonsynonymous coding	11%
MM12T	BAZ1B	bromodomain adjacent to zinc finger domain, 1B	CCDS5549.1	chr7_72856543-72856543_G_A	1479R>X	Substitution	Nonsense	35%
MM12T	BAZ2A	bromodomain adjacent to zinc finger domain, 2A	CCDS44924.1	chr12_56992674-56992674_G_A	1844R>W	Substitution	Nonsynonymous coding	11%
MM12T	BBC3	BCL2 binding component 3	CCDS46129.1	chr19_47725082-47725082_C_T	221S>N	Substitution	Nonsynonymous coding	28%
MM12T	BBS5	Bardet-Biedl syndrome 5	CCDS2233.1	chr2_170359660-170359660_A_G	291D>G	Substitution	Nonsynonymous coding	15%
MM12T	BBX	bobby sox homolog (Drosophila)	CCDS46881.1	chr3_107435563-107435563_G_A	91C>Y	Substitution	Nonsynonymous coding	30%
MM12T	BBX	bobby sox homolog (Drosophila)	CCDS46881.1	chr3_107524293-107524293_G_A	939A>T	Substitution	Nonsynonymous coding	24%
MM12T	BCAR1	breast cancer anti-estrogen resistance 1	CCDS10915.1	chr16_75263657-75263657_C_T	789A>T	Substitution	Nonsynonymous coding	32%
MM12T	BCAS3	breast carcinoma amplified sequence 3	CCDS45749.1	chr17_59155805-59155805_T_G	763S>A	Substitution	Nonsynonymous coding	30%
MM12T	BCAS3	breast carcinoma amplified sequence 3	CCDS45749.1	chr17_59469335-59469335_C_T	ISV-3>	Substitution	Splice site acceptor	26%
MM12T	BCHE	butyrylcholinesterase	CCDS3198.1	chr3_165548191-165548191_C_A	211A>S	Substitution	Nonsynonymous coding	25%
MM12T	BCKDHB	branched chain keto acid dehydrogenase E1, beta polypeptide	CCDS4994.1	chr6_80878617-80878617_G_A	168R>H	Substitution	Nonsynonymous coding	20%
MM12T	BCKDHB	branched chain keto acid dehydrogenase E1, beta polypeptide	CCDS4994.1	chr6_80881032-80881032_G_T	223G>X	Substitution	Nonsense	37%
MM12T	BCKDK	branched chain ketoacid dehydrogenase kinase	CCDS10705.1	chr16_31122419-31122419_T_C	242C>R	Substitution	Nonsynonymous coding	10%
MM12T	BCL11B	B-cell CLL/lymphoma 11B (zinc finger protein)	CCDS9950.1	chr14_99641309-99641309_C_T	622A>T	Substitution	Nonsynonymous coding	38%
MM12T	BCL2	B-cell CLL/lymphoma 2	CCDS11981.1	chr18_60795908-60795908_C_T	224A>T	Substitution	Nonsynonymous coding	21%
MM12T	BCL3	B-cell CLL/lymphoma 3	CCDS12642.2	chr19_45260462-45260462_G_T	236E>D	Substitution	Nonsynonymous coding	34%
MM12T	BCL6B	B-cell CLL/lymphoma 6, member B	CCDS42248.1	chr17_6927428-6927428_G_A	69G>D	Substitution	Nonsynonymous coding	26%
MM12T	BCL6B	B-cell CLL/lymphoma 6, member B	CCDS42248.1	chr17_6927800-6927800_C_T	161P>L	Substitution	Nonsynonymous coding	13%
MM12T	BCL9	B-cell CLL/lymphoma 9	CCDS30833.1	chr1_147086310-147086310_G_T	152R>M	Substitution	Nonsynonymous coding	29%
MM12T	BCL9	B-cell CLL/lymphoma 9	CCDS30833.1	chr1_147092737-147092737_C_T	926L>F	Substitution	Nonsynonymous coding	17%
MM12T	BCL9	B-cell CLL/lymphoma 9	CCDS30833.1	chr1_147095697-147095697_C_T	1073P>L	Substitution	Nonsynonymous coding	12%
MM12T	BCL9L	B-cell CLL/lymphoma 9-like	CCDS8403.1	chr11_118771672-118771672_G_A	927P>L	Substitution	Nonsynonymous coding	24%
MM12T	BCMO1	beta-carotene 15,15'-monooxygenase 1	CCDS10934.1	chr16_81301680-81301680_G_A	263A>T	Substitution	Nonsynonymous coding	10%
MM12T	BCO2	beta-carotene oxygenase 2	CCDS8358.2	chr11_112050117-112050117_T_G	69S>A	Substitution	Nonsynonymous coding	36%
MM12T	BCOR	BCL6 corepressor	CCDS48093.1	chrX_39932749-39932749_G_A	617A>V	Substitution	Nonsynonymous coding	21%
MM12T	BCORL1	BCL6 corepressor-like 1	CCDS14616.1	chrX_129149524-129149524_C_A	926L>I	Substitution	Nonsynonymous coding	35%
MM12T	BCORL1	BCL6 corepressor-like 1	CCDS14616.1	chrX_129189993-129189993_G_A	1673G>E	Substitution	Nonsynonymous coding	15%
MM12T	BCR	breakpoint cluster region	CCDS13806.1	chr22_23524040-23524040_G_A	298R>H	Substitution	Nonsynonymous coding	28%

MM12T	BDKRB1	bradykinin receptor B1	CCDS9943.1	chr14_96730497-96730497_C_A	160L>I	Substitution	Nonsynonymous coding	20%
MM12T	BDKRB2	bradykinin receptor B2	CCDS9942.1	chr14_96706784-96706784_G_A	40G>E	Substitution	Nonsynonymous coding	19%
MM12T	BDP1	B double prime 1, subunit of RNA polymerase III transcription initiation factor IIIB	CCDS43328.1	chr5_70782320-70782320_G_A	360R>H	Substitution	Nonsynonymous coding	56%
MM12T	BDP1	B double prime 1, subunit of RNA polymerase III transcription initiation factor IIIB	CCDS43328.1	chr5_70785377-70785377_G_A	454A>T	Substitution	Nonsynonymous coding	35%
MM12T	BDP1	B double prime 1, subunit of RNA polymerase III transcription initiation factor IIIB	CCDS43328.1	chr5_70845290-70845290_A_C	2284Q>H	Substitution	Nonsynonymous coding	47%
MM12T	BDP1	B double prime 1, subunit of RNA polymerase III transcription initiation factor IIIB	CCDS43328.1	chr5_70860660-70860660_T_C	2608V>A	Substitution	Nonsynonymous coding	14%
MM12T	BEND2	BEN domain containing 2	CCDS14184.1	chrX_18192212-18192212_G_A	640A>V	Substitution	Nonsynonymous coding	28%
MM12T	BEST3	bestrophin 3	CCDS8992.2	chr12_70091461-70091461_C_T	40A>T	Substitution	Nonsynonymous coding	28%
MM12T	BEX5	brain expressed, X-linked 5	CCDS35350.1	chrX_101408913-101408913_G_T	109L>I	Substitution	Nonsynonymous coding	34%
MM12T	BFSP1	beaded filament structural protein 1, filensin	CCDS13126.1	chr20_17474952-17474952_G_A	589P>S	Substitution	Nonsynonymous coding	11%
MM12T	BFSP1	beaded filament structural protein 1, filensin	CCDS13126.1	chr20_17511691-17511691_T_A	95N>I	Substitution	Nonsynonymous coding	26%
MM12T	BGN	biglycan	CCDS14721.1	chrX_152772061-152772061_C_T	214R>C	Substitution	Nonsynonymous coding	10%
MM12T	BHLHA9	basic helix-loop-helix family, member a9	CCDS45560.1	chr17_1174057-1174057_G_A	67R>H	Substitution	Nonsynonymous coding	17%
MM12T	BHMT2	betaine--homocysteine S-methyltransferase 2	CCDS4045.1	chr5_78376543-78376543_G_A	98A>T	Substitution	Nonsynonymous coding	10%
MM12T	BID	BH3 interacting domain death agonist	CCDS13747.1	chr22_18232885-18232885_C_T	46A>T	Substitution	Nonsynonymous coding	25%
MM12T	BIN3	bridging integrator 3	CCDS47825.1	chr8_22487971-22487971_G_A	94A>V	Substitution	Nonsynonymous coding	23%
MM12T	BIRC5	baculoviral IAP repeat containing 5	CCDS32752.1	chr17_76219600-76219600_C_T	155R>C	Substitution	Nonsynonymous coding	35%
MM12T	BIRC6	baculoviral IAP repeat containing 6	CCDS33175.2	chr2_32692736-32692736_G_A	1834A>T	Substitution	Nonsynonymous coding	16%
MM12T	BIRC6	baculoviral IAP repeat containing 6	CCDS33175.2	chr2_32702593-32702593_T_C	2337L>P	Substitution	Nonsynonymous coding	28%
MM12T	BIRC6	baculoviral IAP repeat containing 6	CCDS33175.2	chr2_32750551-32750551_C_T	3926R>C	Substitution	Nonsynonymous coding	23%
MM12T	BIRC6	baculoviral IAP repeat containing 6	CCDS33175.2	chr2_32774414-32774414_C_T	4337A>V	Substitution	Nonsynonymous coding	11%
MM12T	BIRC7	baculoviral IAP repeat containing 7	ENST00000395306	chr20_61869599-61869599_C_T	14R>C	Substitution	Nonsynonymous coding	14%
MM12T	BIRC8	baculoviral IAP repeat containing 8	CCDS12863.1	chr19_53793489-53793489_C_A	47A>S	Substitution	Nonsynonymous coding	26%
MM12T	BLID	BH3-like motif containing, cell death inducer	CCDS31693.1	chr11_121986441-121986441_C_A	64V>L	Substitution	Nonsynonymous coding	35%
MM12T	BLM	Bloom syndrome, RecQ helicase-like	CCDS10363.1	chr15_91304139-91304139_A_	NA	Deletion	Frameshift	19%
MM12T	BLMH	bleomycin hydrolase	CCDS32604.1	chr17_28616498-28616498_G_A	72R>X	Substitution	Nonsense	31%
MM12T	BLNK	B-cell linker	CCDS7446.1	chr10_97951784-97951784_C_T	439S>N	Substitution	Nonsynonymous coding	15%
MM12T	BMP1	bone morphogenetic protein 1	CCDS6026.1	chr8_22052406-22052406_C_T	538A>V	Substitution	Nonsynonymous coding	31%
MM12T	BMP6	bone morphogenetic protein 6	CCDS4503.1	chr6_7862638-7862638_C_T	371R>C	Substitution	Nonsynonymous coding	10%
MM12T	BMP7	bone morphogenetic protein 7	CCDS13455.1	chr20_55841160-55841160_G_A	7R>X	Substitution	Nonsense	10%
MM12T	BMPR1B	bone morphogenetic protein receptor, type IB	CCDS3642.1	chr4_96052665-96052665_T_C	ISV+2>	Substitution	Splice site donor	21%
MM12T	BMS1	BMS1 homolog, ribosome assembly protein (yeast)	CCDS7199.1	chr10_43292494-43292494_G_C	601S>T	Substitution	Nonsynonymous coding	40%

MM12T	BNC1	basonuclin 1	CCDS10324.1	chr15_83926489-83926489_G_A	897S>L	Substitution	Nonsynonymous coding	31%
MM12T	BNIP1	BCL2/adenovirus E1B 19kDa interacting protein 1	CCDS4385.1	chr5_172578611-172578611_T_	NA	Deletion	Frameshift	35%
MM12T	BNIPL	BCL2/adenovirus E1B 19kD interacting protein like	CCDS978.2	chr1_151018347-151018347_G_A	309R>Q	Substitution	Nonsynonymous coding	33%
MM12T	BOC	BOC cell adhesion associated, oncogene regulated	CCDS2971.1	chr3_112991398-112991398_G_A	270S>N	Substitution	Nonsynonymous coding	31%
MM12T	BOK	BCL2-related ovarian killer	CCDS2550.1	chr2_242509659-242509659_G_A	157V>M	Substitution	Nonsynonymous coding	49%
MM12T	BORA	bora, aurora kinase A activator	CCDS9446.1	chr13_73321193-73321193_T_C	476C>R	Substitution	Nonsynonymous coding	24%
MM12T	BPIFA2	BPI fold containing family A, member 2	CCDS13214.1	chr20_31760858-31760858_T_G	93L>R	Substitution	Nonsynonymous coding	28%
MM12T	BPIFB1	BPI fold containing family B, member 1	CCDS13218.1	chr20_31878894-31878894_G_A	166R>H	Substitution	Nonsynonymous coding	11%
MM12T	BPIFB1	BPI fold containing family B, member 1	CCDS13218.1	chr20_31890779-31890779_G_A	347E>K	Substitution	Nonsynonymous coding	17%
MM12T	BPIFB1	BPI fold containing family B, member 1	CCDS13218.1	chr20_31894729-31894729_C_T	444S>F	Substitution	Nonsynonymous coding	15%
MM12T	BPIFB4	BPI fold containing family B, member 4	CCDS13213.2	chr20_31671236-31671236_G_A	78G>D	Substitution	Nonsynonymous coding	13%
MM12T	BPIFC	BPI fold containing family C	CCDS13906.1	chr22_32815359-32815359_C_A	417S>I	Substitution	Nonsynonymous coding	35%
MM12T	BRAT1	BRCA1-associated ATM activator 1	ENST00000382467	chr7_2582524-2582524_C_T	49R>H	Substitution	Nonsynonymous coding	14%
MM12T	BRCA2	breast cancer 2, early onset	CCDS9344.1	chr13_32899249-32899249_G_A	118R>H	Substitution	Nonsynonymous coding	17%
MM12T	BRCA2	breast cancer 2, early onset	CCDS9344.1	chr13_32915198-32915198_G_A	2236E>K	Substitution	Nonsynonymous coding	11%
MM12T	BRD3	bromodomain containing 3	CCDS6980.1	chr9_136905225-136905225_G_A	525P>L	Substitution	Nonsynonymous coding	33%
MM12T	BRD8	bromodomain containing 8	CCDS4198.1	chr5_137500779-137500779_G_T	452P>H	Substitution	Nonsynonymous coding	34%
MM12T	BRD9	bromodomain containing 9	CCDS34127.1	chr5_881266-881266_C_A	237S>I	Substitution	Nonsynonymous coding	32%
MM12T	BRSK1	BR serine/threonine kinase 1	CCDS12921.1	chr19_55814112-55814112_G_A	302R>H	Substitution	Nonsynonymous coding	31%
MM12T	BRSK2	BR serine/threonine kinase 2	CCDS41590.1	chr11_1480430-1480430_A_G	650N>D	Substitution	Nonsynonymous coding	44%
MM12T	BRSK2	BR serine/threonine kinase 2	ENST00000308230	chr11_1469027-1469027_G_A	ISV-1>	Substitution	Splice site acceptor	29%
MM12T	BRWD1	bromodomain and WD repeat domain containing 1	CCDS13662.1	chr21_40581991-40581991__A	NA	Insertion	Frameshift	25%
MM12T	BRWD3	bromodomain and WD repeat domain containing 3	CCDS14447.1	chrX_79991591-79991591_A_	NA	Deletion	Splice site acceptor	14%
MM12T	BRWD3	bromodomain and WD repeat domain containing 3	CCDS14447.1	chrX_79984250-79984250_C_T	ISV+1>	Substitution	Splice site donor	20%
MM12T	BRWD3	bromodomain and WD repeat domain containing 3	CCDS14447.1	chrX_80064508-80064508_G_A	ISV+4>	Substitution	Splice site donor	31%
MM12T	BTAF1	BTAF1 RNA polymerase II, B-TFIID transcription factor-associated, 170kDa (Mot1 homolog, S.	CCDS7419.1	chr10_93702328-93702328_C_A	ISV+3>	Substitution	Splice site donor	23%
MM12T	BTBD3	BTB (POZ) domain containing 3	CCDS13113.1	chr20_11903690-11903690_G_T	315K>N	Substitution	Nonsynonymous coding	40%
MM12T	BTBD7	BTB (POZ) domain containing 7	CCDS32146.1	chr14_93761193-93761193_T_	NA	Deletion	Frameshift	29%
MM12T	BTBD7	BTB (POZ) domain containing 7	CCDS32146.1	chr14_93717955-93717955_C_T	599R>H	Substitution	Nonsynonymous coding	22%
MM12T	BTK	Bruton agammaglobulinemia tyrosine kinase	CCDS14482.1	chrX_100630190-100630190_C_T	28R>H	Substitution	Nonsynonymous coding	33%
MM12T	BTN2A2	butyrophilin, subfamily 2, member A2	CCDS4606.1	chr6_26388406-26388406_G_A	203G>D	Substitution	Nonsynonymous coding	19%
MM12T	BTNL9	butyrophilin-like 9	CCDS4460.2	chr5_180486432-180486432_G_A	393R>H	Substitution	Nonsynonymous coding	14%

MM12T	BZRAP1	benzodiazapine receptor (peripheral) associated protein 1	CCDS11605.1	chr17_56405253-56405253_G_T	10P>H	Substitution	Nonsynonymous coding	24%
MM12T	C10orf112	chromosome 10 open reading frame 112	ENST00000454679	chr10_19569027-19569027_C_T	96T>I	Substitution	Nonsynonymous coding	13%
MM12T	C10orf112	chromosome 10 open reading frame 112	ENST00000454679	chr10_19616510-19616510_A_C	251N>H	Substitution	Nonsynonymous coding	28%
MM12T	C10orf118	chromosome 10 open reading frame 118	CCDS7587.1	chr10_115891005-115891005_C_T	668A>T	Substitution	Nonsynonymous coding	13%
MM12T	C10orf118	chromosome 10 open reading frame 118	CCDS7587.1	chr10_115894744-115894744_C_T	528R>H	Substitution	Nonsynonymous coding	21%
MM12T	C10orf137	chromosome 10 open reading frame 137	CCDS7646.1	chr10_127426521-127426521_A_C	564E>A	Substitution	Nonsynonymous coding	13%
MM12T	C10orf137	chromosome 10 open reading frame 137	CCDS7646.1	chr10_127426535-127426535_C_T	569L>F	Substitution	Nonsynonymous coding	35%
MM12T	C10orf68	chromosome 10 open reading frame 68	CCDS31177.1	chr10_33137556-33137556_G_A	512V>I	Substitution	Nonsynonymous coding	36%
MM12T	C11orf21	chromosome 11 open reading frame 21	CCDS44518.1	chr11_2323001-2323001_C_T	31A>T	Substitution	Nonsynonymous coding	19%
MM12T	C11orf24	chromosome 11 open reading frame 24	CCDS8180.1	chr11_68029865-68029865_C_T	200A>T	Substitution	Nonsynonymous coding	32%
MM12T	C11orf49	chromosome 11 open reading frame 49	CCDS31480.1	chr11_47176747-47176747_C_T	129A>V	Substitution	Nonsynonymous coding	31%
MM12T	C11orf89	chromosome 11 open reading frame 89	ENST00000391480	chr11_1911646-1911646_C_T	57A>T	Substitution	Nonsynonymous coding	36%
MM12T	C12orf4	chromosome 12 open reading frame 4	CCDS8528.1	chr12_4598983-4598983_G_A	550S>F	Substitution	Nonsynonymous coding	27%
MM12T	C12orf56	chromosome 12 open reading frame 56	CCDS44935.1	chr12_64664484-64664484_A_G	372V>A	Substitution	Nonsynonymous coding	32%
MM12T	C12orf60	chromosome 12 open reading frame 60	CCDS8667.1	chr12_14976237-14976237_T_C	123I>T	Substitution	Nonsynonymous coding	27%
MM12T	C12orf74	chromosome 12 open reading frame 74	CCDS41819.1	chr12_93101441-93101441_C_A	175P>T	Substitution	Nonsynonymous coding	28%
MM12T	C13orf35	chromosome 13 open reading frame 35	CCDS9526.1	chr13_113333775-113333775_G_A	28A>T	Substitution	Nonsynonymous coding	30%
MM12T	C14orf159	chromosome 14 open reading frame 159	ENST00000298858	chr14_91633917-91633917_G_A	121R>H	Substitution	Nonsynonymous coding	32%
MM12T	C14orf159	chromosome 14 open reading frame 159	CCDS41979.1	chr14_91647656-91647656_C_A	286P>H	Substitution	Nonsynonymous coding	21%
MM12T	C14orf93	chromosome 14 open reading frame 93	CCDS9583.1	chr14_23456769-23456769_C_A	424E>D	Substitution	Nonsynonymous coding	35%
MM12T	C15orf39	chromosome 15 open reading frame 39	CCDS10276.1	chr15_75498687-75498687_G_T	100G>C	Substitution	Nonsynonymous coding	32%
MM12T	C15orf39	chromosome 15 open reading frame 39	CCDS10276.1	chr15_75500086-75500086_G_A	566G>D	Substitution	Nonsynonymous coding	11%
MM12T	C15orf39	chromosome 15 open reading frame 39	CCDS10276.1	chr15_75500958-75500958_C_A	857L>M	Substitution	Nonsynonymous coding	12%
MM12T	C15orf39	chromosome 15 open reading frame 39	CCDS10276.1	chr15_75503327-75503327_G_A	1005R>H	Substitution	Nonsynonymous coding	20%
MM12T	C15orf48	chromosome 15 open reading frame 48	CCDS10124.1	chr15_45723033-45723033_A_G	ISV+3>	Substitution	Splice site donor	39%
MM12T	C15orf52	chromosome 15 open reading frame 52	CCDS10055.2	chr15_40631782-40631782_C_	NA	Deletion	Frameshift	35%
MM12T	C15orf54	chromosome 15 open reading frame 54	CCDS10049.1	chr15_39544541-39544541_G_A	69A>T	Substitution	Nonsynonymous coding	11%
MM12T	C15orf55	chromosome 15 open reading frame 55	CCDS32190.1	chr15_34642912-34642912_C_T	245L>F	Substitution	Nonsynonymous coding	37%
MM12T	C16orf11	chromosome 16 open reading frame 11	CCDS45365.1	chr16_613631-613631_G_A	113A>T	Substitution	Nonsynonymous coding	25%
MM12T	C16orf11	chromosome 16 open reading frame 11	CCDS45365.1	chr16_614276-614276_C_T	328R>W	Substitution	Nonsynonymous coding	13%
MM12T	C16orf54	chromosome 16 open reading frame 54	CCDS10652.1	chr16_29756203-29756203_G_A	24P>S	Substitution	Nonsynonymous coding	11%
MM12T	C16orf86	chromosome 16 open reading frame 86	CCDS32468.2	chr16_67701331-67701331_C_T	79R>W	Substitution	Nonsynonymous coding	16%

MM12T	C16orf91	chromosome 16 open reading frame 91	CCDS32360.1	chr16_1478443-1478443_A_G	70S>P	Substitution	Nonsynonymous coding	33%
MM12T	C17orf47	chromosome 17 open reading frame 47	CCDS32691.1	chr17_56621358-56621358_C_T	64A>T	Substitution	Nonsynonymous coding	12%
MM12T	C17orf62	chromosome 17 open reading frame 62	ENST00000342572	chr17_80401431-80401431_G_A	191P>S	Substitution	Nonsynonymous coding	14%
MM12T	C17orf70	chromosome 17 open reading frame 70	CCDS32765.1	chr17_79514274-79514274_C_T	461G>R	Substitution	Nonsynonymous coding	31%
MM12T	C17orf75	chromosome 17 open reading frame 75	NM_022344	chr17_30658936-30658936_G_T	346A>D	Substitution	Nonsynonymous coding	16%
MM12T	C17orf97	chromosome 17 open reading frame 97	CCDS32519.2	chr17_260197-260197_C_T	22P>S	Substitution	Nonsynonymous coding	45%
MM12T	C17orf98	chromosome 17 open reading frame 98	CCDS42310.1	chr17_36997620-36997620_C_T	8R>H	Substitution	Nonsynonymous coding	25%
MM12T	C17orf99	chromosome 17 open reading frame 99	NM_001163075	chr17_76160284-76160284_G_A	160S>N	Substitution	Nonsynonymous coding	10%
MM12T	C18orf32	chromosome 18 open reading frame 32	CCDS32831.1	chr18_47010001-47010001_C_T	40A>T	Substitution	Nonsynonymous coding	32%
MM12T	C18orf8	chromosome 18 open reading frame 8	CCDS32803.1	chr18_21109195-21109195_G_A	450R>Q	Substitution	Nonsynonymous coding	34%
MM12T	C19orf24	chromosome 19 open reading frame 24	ENST00000416408	chr19_1278902-1278902_C_G	31H>Q	Substitution	Nonsynonymous coding	35%
MM12T	C19orf25	chromosome 19 open reading frame 25	CCDS45898.1	chr19_1478808-1478808_G_A	32A>V	Substitution	Nonsynonymous coding	45%
MM12T	C19orf38	chromosome 19 open reading frame 38	CCDS45970.1	chr19_10959188-10959188_C_T	2P>S	Substitution	Nonsynonymous coding	33%
MM12T	C19orf38	chromosome 19 open reading frame 38	CCDS45970.1	chr19_10961021-10961021_G_A	40A>T	Substitution	Nonsynonymous coding	43%
MM12T	C19orf68	chromosome 19 open reading frame 68	ENST00000328759	chr19_48698592-48698592_C_T	424A>V	Substitution	Nonsynonymous coding	11%
MM12T	C1GALT1C1	C1GALT1-specific chaperone 1	CCDS14602.1	chrX_119760552-119760552_A_C	157F>C	Substitution	Nonsynonymous coding	31%
MM12T	C1orf110	chromosome 1 open reading frame 110	CCDS44269.1	chr1_162824700-162824700_T_G	255N>T	Substitution	Nonsynonymous coding	36%
MM12T	C1orf114	chromosome 1 open reading frame 114	CCDS1279.1	chr1_169390795-169390795_C_T	292A>T	Substitution	Nonsynonymous coding	13%
MM12T	C1orf168	chromosome 1 open reading frame 168	CCDS30729.1	chr1_57192215-57192215_G_A	612T>I	Substitution	Nonsynonymous coding	13%
MM12T	C1orf173	chromosome 1 open reading frame 173	CCDS30755.1	chr1_75038564-75038564_C_A	944E>X	Substitution	Nonsense	16%
MM12T	C1orf200	chromosome 1 open reading frame 200	ENST00000377320	chr1_9714413-9714413_C_T	28V>I	Substitution	Nonsynonymous coding	12%
MM12T	C1ORF224	-	ENST00000414055	chr1_16543026-16543026_G_A	38R>X	Substitution	Nonsense	15%
MM12T	C1orf228	chromosome 1 open reading frame 228	NM_001145636	chr1_45162839-45162839_G_A	91S>N	Substitution	Nonsynonymous coding	11%
MM12T	C1orf229	chromosome 1 open reading frame 229	CCDS1630.1	chr1_247275319-247275319_G_A	70P>S	Substitution	Nonsynonymous coding	40%
MM12T	C1orf50	chromosome 1 open reading frame 50	CCDS473.1	chr1_43233248-43233248_G_A	50A>T	Substitution	Nonsynonymous coding	11%
MM12T	C1orf65	chromosome 1 open reading frame 65	CCDS1537.1	chr1_223568328-223568328_G_T	504R>I	Substitution	Nonsynonymous coding	12%
MM12T	C1orf95	chromosome 1 open reading frame 95	CCDS31044.1	chr1_226784565-226784565_G_A	89V>M	Substitution	Nonsynonymous coding	31%
MM12T	C1QTNF5	C1q and tumor necrosis factor related protein 5	CCDS8420.1	chr11_119210099-119210099_C_T	225S>N	Substitution	Nonsynonymous coding	11%
MM12T	C1RL-AS1	C1RL antisense RNA 1	ENST00000382215	chr12_7272127-7272127_G_A	3A>T	Substitution	Nonsynonymous coding	32%
MM12T	C1RL-AS1	C1RL antisense RNA 1	ENST00000382215	chr12_7274419-7274419_G_A	87W>X	Substitution	Nonsense	12%
MM12T	C20orf112	chromosome 20 open reading frame 112	ENST00000201961	chr20_31108722-31108722_C_T	67A>T	Substitution	Nonsynonymous coding	11%
MM12T	C20orf112	chromosome 20 open reading frame 112	ENST00000201961	chr20_31115598-31115598_G_A	53A>V	Substitution	Nonsynonymous coding	33%

MM12T	C20orf194	chromosome 20 open reading frame 194	CCDS42851.1	chr20_3234371-3234371_C_T	ISV+1>	Substitution	Splice site donor	38%
MM12T	C20orf26	chromosome 20 open reading frame 26	CCDS33447.1	chr20_20079311-20079311_G_T	238A>S	Substitution	Nonsynonymous coding	33%
MM12T	C21orf59	chromosome 21 open reading frame 59	CCDS13617.1	chr21_33974665-33974665_C_T	227A>T	Substitution	Nonsynonymous coding	13%
MM12T	C21orf59	chromosome 21 open reading frame 59	CCDS13617.1	chr21_33982189-33982189_G_A	89A>V	Substitution	Nonsynonymous coding	39%
MM12T	C21orf91	chromosome 21 open reading frame 91	CCDS42907.1	chr21_19169024-19169024_G_A	180A>V	Substitution	Nonsynonymous coding	13%
MM12T	C22orf28	chromosome 22 open reading frame 28	CCDS13905.1	chr22_32792233-32792233_G_A	273A>V	Substitution	Nonsynonymous coding	34%
MM12T	C22orf31	chromosome 22 open reading frame 31	CCDS13848.1	chr22_29455064-29455064_G_A	180T>I	Substitution	Nonsynonymous coding	21%
MM12T	C2CD2	C2 calcium-dependent domain containing 2	CCDS42933.1	chr21_43321727-43321727_G_A	ISV+4>	Substitution	Splice site donor	42%
MM12T	C2CD4B	C2 calcium-dependent domain containing 4B	CCDS32259.1	chr15_62457174-62457174_G_T	4L>I	Substitution	Nonsynonymous coding	31%
MM12T	C2orf16	chromosome 2 open reading frame 16	CCDS42666.1	chr2_27800626-27800626_G_A	396G>E	Substitution	Nonsynonymous coding	20%
MM12T	C2orf16	chromosome 2 open reading frame 16	CCDS42666.1	chr2_27802186-27802186_G_A	916R>K	Substitution	Nonsynonymous coding	15%
MM12T	C2orf40	chromosome 2 open reading frame 40	CCDS2072.1	chr2_106690369-106690369_C_T	52A>V	Substitution	Nonsynonymous coding	11%
MM12T	C2orf61	chromosome 2 open reading frame 61	CCDS1831.1	chr2_47380109-47380109_C_A	43R>I	Substitution	Nonsynonymous coding	30%
MM12T	C2orf66	chromosome 2 open reading frame 66	CCDS2317.1	chr2_197674092-197674092_C_T	7R>H	Substitution	Nonsynonymous coding	35%
MM12T	C3orf36	chromosome 3 open reading frame 36	CCDS3083.1	chr3_133647302-133647302_C_T	116A>T	Substitution	Nonsynonymous coding	19%
MM12T	C3orf36	chromosome 3 open reading frame 36	CCDS3083.1	chr3_133647521-133647521_C_A	43G>X	Substitution	Nonsense	33%
MM12T	C3orf58	chromosome 3 open reading frame 58	CCDS3130.1	chr3_143708657-143708657_G_T	423A>S	Substitution	Nonsynonymous coding	37%
MM12T	C3orf58	chromosome 3 open reading frame 58	CCDS3130.1	chr3_143708675-143708675_G_A	429V>M	Substitution	Nonsynonymous coding	29%
MM12T	C4B	complement component 4B (Chido blood group)	CCDS47405.1	chr6_31996582-31996582_G_A	1115G>S	Substitution	Nonsynonymous coding	13%
MM12T	C5	complement component 5	CCDS6826.1	chr9_123779729-123779729_C_T	593A>T	Substitution	Nonsynonymous coding	15%
MM12T	C5AR2	complement component 5a receptor 2	CCDS12699.1	chr19_47844955-47844955_G_A	300R>H	Substitution	Nonsynonymous coding	31%
MM12T	C5orf45	chromosome 5 open reading frame 45	CCDS34319.1	chr5_179271150-179271150_A_G	ISV+2>	Substitution	Splice site donor	13%
MM12T	C5orf54	chromosome 5 open reading frame 54	CCDS34283.1	chr5_159822202-159822202_G_A	99A>V	Substitution	Nonsynonymous coding	11%
MM12T	C5orf54	chromosome 5 open reading frame 54	CCDS34283.1	chr5_159822419-159822419_G_A	27R>C	Substitution	Nonsynonymous coding	31%
MM12T	C5orf56	chromosome 5 open reading frame 56	ENST00000378947	chr5_131792784-131792784_C_T	57P>S	Substitution	Nonsynonymous coding	36%
MM12T	C6	complement component 6	CCDS3936.1	chr5_41172398-41172398_C_T	407R>H	Substitution	Nonsynonymous coding	18%
MM12T	C6	complement component 6	CCDS3936.1	chr5_41195957-41195957_C_A	175R>M	Substitution	Nonsynonymous coding	23%
MM12T	C6orf15	chromosome 6 open reading frame 15	CCDS4693.1	chr6_31080293-31080293_G_T	14L>M	Substitution	Nonsynonymous coding	23%
MM12T	C6orf211	chromosome 6 open reading frame 211	CCDS5233.1	chr6_151785651-151785651_G_T	152Q>H	Substitution	Nonsynonymous coding	10%
MM12T	C6orf48	chromosome 6 open reading frame 48	ENST00000375637	chr6_31803208-31803208_C_T	91A>V	Substitution	Nonsynonymous coding	35%
MM12T	C7orf10	chromosome 7 open reading frame 10	NM_024728	chr7_40314173-40314173_A_G	183Y>C	Substitution	Nonsynonymous coding	33%
MM12T	C7orf57	chromosome 7 open reading frame 57	CCDS47583.1	chr7_48092305-48092305_A_	NA	Deletion	Frameshift	37%

MM12T	C8orf33	chromosome 8 open reading frame 33	CCDS34974.1	chr8_146278786-146278786_G_T	169Q>H	Substitution	Nonsynonymous coding	31%
MM12T	C8orf34	chromosome 8 open reading frame 34	ENST00000325233	chr8_69537840-69537840_C_T	10T>I	Substitution	Nonsynonymous coding	32%
MM12T	C8orf4	chromosome 8 open reading frame 4	CCDS6115.1	chr8_40011077-40011077_C_T	9A>V	Substitution	Nonsynonymous coding	28%
MM12T	C8orf46	chromosome 8 open reading frame 46	CCDS6191.2	chr8_67428268-67428268_C_A	194S>Y	Substitution	Nonsynonymous coding	32%
MM12T	C8orf82	chromosome 8 open reading frame 82	CCDS34970.1	chr8_145752824-145752824_C_T	185G>S	Substitution	Nonsynonymous coding	41%
MM12T	C8orf86	chromosome 8 open reading frame 86	CCDS6108.1	chr8_38385956-38385956_C_T	67G>D	Substitution	Nonsynonymous coding	16%
MM12T	C9orf142	chromosome 9 open reading frame 142	CCDS7020.1	chr9_139887666-139887666_G_A	131R>H	Substitution	Nonsynonymous coding	31%
MM12T	C9orf169	chromosome 9 open reading frame 169	CCDS48064.1	chr9_140120281-140120281_A_G	70K>E	Substitution	Nonsynonymous coding	31%
MM12T	C9orf3	chromosome 9 open reading frame 3	CCDS6713.1	chr9_97522520-97522520_C_T	152S>F	Substitution	Nonsynonymous coding	32%
MM12T	C9orf3	chromosome 9 open reading frame 3	CCDS6713.1	chr9_97535446-97535446_G_A	320W>X	Substitution	Nonsense	35%
MM12T	CA10	carbonic anhydrase X	CCDS32684.1	chr17_49726552-49726552_T_C	209T>A	Substitution	Nonsynonymous coding	31%
MM12T	CA11	carbonic anhydrase XI	CCDS12729.1	chr19_49143106-49143106_C_T	169G>E	Substitution	Nonsynonymous coding	32%
MM12T	CA12	carbonic anhydrase XII	CCDS10185.1	chr15_63673861-63673861_T_C	20Q>R	Substitution	Nonsynonymous coding	25%
MM12T	CA14	carbonic anhydrase XIV	CCDS947.1	chr1_150232570-150232570_A_G	ISV+4>	Substitution	Splice site donor	60%
MM12T	CA8	carbonic anhydrase VIII	CCDS6174.1	chr8_61144920-61144920_T_C	146N>D	Substitution	Nonsynonymous coding	37%
MM12T	CABIN1	calcineurin binding protein 1	CCDS13823.1	chr22_24458422-24458422_A_G	544M>V	Substitution	Nonsynonymous coding	31%
MM12T	CABP2	calcium binding protein 2	CCDS8170.1	chr11_67287533-67287533_C_T	160A>T	Substitution	Nonsynonymous coding	11%
MM12T	CACNA1B	calcium channel, voltage-dependent, N type, alpha 1B subunit	NM_000718	chr9_140952560-140952560_G_A	1389R>H	Substitution	Nonsynonymous coding	28%
MM12T	CACNA1C	calcium channel, voltage-dependent, L type, alpha 1C subunit	CCDS44788.1	chr12_2719707-2719707_C_T	1207R>X	Substitution	Nonsense	44%
MM12T	CACNA1D	calcium channel, voltage-dependent, L type, alpha 1D subunit	CCDS2872.1	chr3_53757478-53757478_C_A	582L>I	Substitution	Nonsynonymous coding	22%
MM12T	CACNA1G	calcium channel, voltage-dependent, T type, alpha 1G subunit	CCDS45730.1	chr17_48669350-48669350_C_T	936T>M	Substitution	Nonsynonymous coding	23%
MM12T	CACNA1G	calcium channel, voltage-dependent, T type, alpha 1G subunit	CCDS45730.1	chr17_48701762-48701762_T_C	2091Y>H	Substitution	Nonsynonymous coding	35%
MM12T	CACNA1G	calcium channel, voltage-dependent, T type, alpha 1G subunit	CCDS45730.1	chr17_48704012-48704012_C_T	2345A>V	Substitution	Nonsynonymous coding	26%
MM12T	CACNA1I	calcium channel, voltage-dependent, T type, alpha 1I subunit	CCDS46710.1	chr22_40037071-40037071_G_A	314G>S	Substitution	Nonsynonymous coding	34%
MM12T	CACNA1I	calcium channel, voltage-dependent, T type, alpha 1I subunit	CCDS46710.1	chr22_40069076-40069076_G_A	1591R>H	Substitution	Nonsynonymous coding	26%
MM12T	CACNA1S	calcium channel, voltage-dependent, L type, alpha 1S subunit	CCDS1407.1	chr1_201022685-201022685_C_T	1233A>T	Substitution	Nonsynonymous coding	28%
MM12T	CACNA1S	calcium channel, voltage-dependent, L type, alpha 1S subunit	CCDS1407.1	chr1_201047160-201047160_C_T	489R>H	Substitution	Nonsynonymous coding	30%
MM12T	CACNA1S	calcium channel, voltage-dependent, L type, alpha 1S subunit	CCDS1407.1	chr1_201060864-201060864_C_T	200A>T	Substitution	Nonsynonymous coding	10%
MM12T	CACNA2D2	calcium channel, voltage-dependent, alpha 2/delta subunit 2	CCDS33763.1	chr3_50416524-50416524_G_A	420T>M	Substitution	Nonsynonymous coding	12%
MM12T	CACNA2D2	calcium channel, voltage-dependent, alpha 2/delta subunit 2	CCDS33763.1	chr3_50416963-50416963_C_T	351R>H	Substitution	Nonsynonymous coding	13%
MM12T	CACNA2D3	calcium channel, voltage-dependent, alpha 2/delta subunit 3	NM_018398	chr3_54596915-54596915_G_A	211W>X	Substitution	Nonsense	16%
MM12T	CACNA2D4	calcium channel, voltage-dependent, alpha 2/delta subunit 4	CCDS44785.1	chr12_1904848-1904848_G_A	1071A>V	Substitution	Nonsynonymous coding	11%

MM12T	CACNA2D4	calcium channel, voltage-dependent, alpha 2/delta subunit 4	CCDS44785.1	chr12_1993423-1993423_G_A	446A>V	Substitution	Nonsynonymous coding	32%
MM12T	CACNB1	calcium channel, voltage-dependent, beta 1 subunit	CCDS42311.1	chr17_37343848-37343848_G_A	100P>S	Substitution	Nonsynonymous coding	34%
MM12T	CACNB3	calcium channel, voltage-dependent, beta 3 subunit	CCDS8769.1	chr12_49220834-49220834_C_A	356Y>X	Substitution	Nonsense	32%
MM12T	CACNG3	calcium channel, voltage-dependent, gamma subunit 3	CCDS10620.1	chr16_24372936-24372936_C_T	234R>W	Substitution	Nonsynonymous coding	30%
MM12T	CACNG5	calcium channel, voltage-dependent, gamma subunit 5	CCDS11666.1	chr17_64880774-64880774_C_T	189T>M	Substitution	Nonsynonymous coding	26%
MM12T	CAD	carbamoyl-phosphate synthetase 2, aspartate transcarbamylase, and dihydroorotase	CCDS1742.1	chr2_27455949-27455949_A_G	978T>A	Substitution	Nonsynonymous coding	34%
MM12T	CAD	carbamoyl-phosphate synthetase 2, aspartate transcarbamylase, and dihydroorotase	CCDS1742.1	chr2_27461033-27461033_G_A	1613C>Y	Substitution	Nonsynonymous coding	43%
MM12T	CAD	carbamoyl-phosphate synthetase 2, aspartate transcarbamylase, and dihydroorotase	CCDS1742.1	chr2_27449521-27449521_C_T	ISV+4>	Substitution	Splice site donor	36%
MM12T	CAD	carbamoyl-phosphate synthetase 2, aspartate transcarbamylase, and dihydroorotase	CCDS1742.1	chr2_27445832-27445832_C_T	246R>X	Substitution	Nonsense	29%
MM12T	CADM4	cell adhesion molecule 4	CCDS12627.1	chr19_44130371-44130371_C_T	190R>H	Substitution	Nonsynonymous coding	32%
MM12T	CADPS	Ca++-dependent secretion activator	CCDS46858.1	chr3_62484866-62484866_G_T	893L>I	Substitution	Nonsynonymous coding	31%
MM12T	CALB1	calbindin 1, 28kDa	CCDS6251.1	chr8_91072928-91072928_C_A	201D>Y	Substitution	Nonsynonymous coding	25%
MM12T	CALCOCO1	calcium binding and coiled-coil domain 1	CCDS8864.1	chr12_54110058-54110058_C_T	331A>T	Substitution	Nonsynonymous coding	13%
MM12T	CALD1	caldesmon 1	CCDS5835.1	chr7_134617816-134617816_G_A	99R>H	Substitution	Nonsynonymous coding	33%
MM12T	CALHM3	calcium homeostasis modulator 3	CCDS44476.1	chr10_105236136-105236136_G_A	153A>V	Substitution	Nonsynonymous coding	17%
MM12T	CALML3-AS1	CALML3 antisense RNA 1	ENST00000380330	chr10_5558054-5558054_G_A	61P>S	Substitution	Nonsynonymous coding	11%
MM12T	CALN1	calneuron 1	CCDS47603.1	chr7_71868322-71868322_C_A	11K>N	Substitution	Nonsynonymous coding	40%
MM12T	CALR	calreticulin	CCDS12288.1	chr19_13054364-13054364_C_T	325T>I	Substitution	Nonsynonymous coding	24%
MM12T	CAMKK2	calcium/calmodulin-dependent protein kinase kinase 2, beta	CCDS9216.1	chr12_121678660-121678660_G_A	537R>X	Substitution	Nonsense	28%
MM12T	CAMKV	CaM kinase-like vesicle-associated	CCDS33762.1	chr3_49897686-49897686_G_T	287S>Y	Substitution	Nonsynonymous coding	38%
MM12T	CAMSAP2	calmodulin regulated spectrin-associated protein family, member 2	CCDS1404.1	chr1_200811194-200811194_C_T	334R>C	Substitution	Nonsynonymous coding	33%
MM12T	CAMTA1	calmodulin binding transcription activator 1	CCDS30576.1	chr1_6885274-6885274_G_A	ISV+4>	Substitution	Splice site donor	15%
MM12T	CAND2	cullin-associated and neddylation-dissociated 2 (putative)	CCDS43053.1	chr3_12858079-12858079_C_T	457R>W	Substitution	Nonsynonymous coding	36%
MM12T	CAPN12	calpain 12	CCDS12519.1	chr19_39228979-39228979_C_T	300R>H	Substitution	Nonsynonymous coding	33%
MM12T	CAPN5	calpain 5 [Source:HGNC Symbol;Acc:1482]	CCDS8248.1	chr11_76823833-76823833_G_A	166A>T	Substitution	Nonsynonymous coding	27%
MM12T	CAPZA3	capping protein (actin filament) muscle Z-line, alpha 3	CCDS8681.1	chr12_18891987-18891987_T_A	262V>E	Substitution	Nonsynonymous coding	11%
MM12T	CAPZB	capping protein (actin filament) muscle Z-line, beta	NM_004930	chr1_19746205-19746205_G_A	15R>C	Substitution	Nonsynonymous coding	27%
MM12T	CARD6	caspase recruitment domain family, member 6	CCDS3935.1	chr5_40843608-40843608_A_T	213E>V	Substitution	Nonsynonymous coding	17%
MM12T	CARNS1	carnosine synthase 1	CCDS44658.1	chr11_67191779-67191779_C_T	731R>C	Substitution	Nonsynonymous coding	69%
MM12T	CARS	cysteinyl-tRNA synthetase	CCDS41600.1	chr11_3059352-3059352_C_A	243Q>H	Substitution	Nonsynonymous coding	40%
MM12T	CASD1	CAS1 domain containing 1	CCDS5636.1	chr7_94183816-94183816_A_T	686R>X	Substitution	Nonsense	12%
MM12T	CASK	calcium/calmodulin-dependent serine protein kinase (MAGUK family)	CCDS14257.1	chrX_41394037-41394037_C_T	742A>T	Substitution	Nonsynonymous coding	11%

MM12T	CASKIN1	CASK interacting protein 1	CCDS42103.1	chr16_2229851-2229851_C_A	1173G>V	Substitution	Nonsynonymous coding	10%
MM12T	CASKIN1	CASK interacting protein 1	CCDS42103.1	chr16_2230128-2230128_G_A	1081P>S	Substitution	Nonsynonymous coding	32%
MM12T	CASKIN2	CASK interacting protein 2	CCDS11723.1	chr17_73498339-73498339_G_A	939T>M	Substitution	Nonsynonymous coding	29%
MM12T	CASKIN2	CASK interacting protein 2	CCDS11723.1	chr17_73499831-73499831_G_A	530A>V	Substitution	Nonsynonymous coding	39%
MM12T	CASP8AP2	caspase 8 associated protein 2	NM_001137667	chr6_90577649-90577649_G_A	1547R>H	Substitution	Nonsynonymous coding	14%
MM12T	CASR	calcium-sensing receptor	CCDS3010.1	chr3_122003398-122003398_G_A	866R>H	Substitution	Nonsynonymous coding	32%
MM12T	CAST	calpastatin	CCDS43342.1	chr5_96084271-96084271_G_A	461R>Q	Substitution	Nonsynonymous coding	34%
MM12T	CAT	catalase	CCDS7891.1	chr11_34477715-34477715_A_T	290E>V	Substitution	Nonsynonymous coding	10%
MM12T	CATSPER1	cation channel, sperm associated 1	CCDS8127.1	chr11_65788380-65788380_G_A	610P>L	Substitution	Nonsynonymous coding	16%
MM12T	CATSPERD	catsper channel auxiliary subunit delta	CCDS12149.2	chr19_5739383-5739383_C_T	169S>F	Substitution	Nonsynonymous coding	26%
MM12T	CBFA2T3	core-binding factor, runt domain, alpha subunit 2; translocated to, 3	CCDS10972.1	chr16_88943469-88943469_G_A	626A>V	Substitution	Nonsynonymous coding	15%
MM12T	CBL	Cbl proto-oncogene, E3 ubiquitin protein ligase	CCDS8418.1	chr11_119149290-119149290_C_T	433P>L	Substitution	Nonsynonymous coding	25%
MM12T	CBLN3	cerebellin 3 precursor	CCDS32057.1	chr14_24897550-24897550_C_T	121R>Q	Substitution	Nonsynonymous coding	29%
MM12T	CBR1	carbonyl reductase 1	ENST00000399191	chr21_37444556-37444556_C_T	161S>L	Substitution	Nonsynonymous coding	32%
MM12T	CBX4	chromobox homolog 4	CCDS32758.1	chr17_77808836-77808836_G_A	202A>V	Substitution	Nonsynonymous coding	12%
MM12T	CC2D1A	coiled-coil and C2 domain containing 1A	CCDS42512.1	chr19_14023203-14023203_G_T	88E>D	Substitution	Nonsynonymous coding	38%
MM12T	CC2D1B	coiled-coil and C2 domain containing 1B	ENST00000371573	chr1_52818889-52818889_C_T	519A>T	Substitution	Nonsynonymous coding	25%
MM12T	CC2D2A	coiled-coil and C2 domain containing 2A	CCDS47026.1	chr4_15517577-15517577_G_A	323A>T	Substitution	Nonsynonymous coding	13%
MM12T	CC2D2B	coiled-coil and C2 domain containing 2B	CCDS41555.1	chr10_97772440-97772440_C_A	91P>H	Substitution	Nonsynonymous coding	27%
MM12T	CCDC106	coiled-coil domain containing 106	CCDS33118.1	chr19_56163879-56163879_G_A	204E>K	Substitution	Nonsynonymous coding	32%
MM12T	CCDC108	coiled-coil domain containing 108	CCDS2430.2	chr2_219874072-219874072_G_C	1521C>W	Substitution	Nonsynonymous coding	15%
MM12T	CCDC108	coiled-coil domain containing 108	CCDS2430.2	chr2_219885905-219885905_C_T	1076W>X	Substitution	Nonsense	40%
MM12T	CCDC112	coiled-coil domain containing 112	CCDS34213.1	chr5_114607059-114607059_A_G	395F>L	Substitution	Nonsynonymous coding	13%
MM12T	CCDC113	coiled-coil domain containing 113	CCDS10795.1	chr16_58292357-58292357_C_T	159S>F	Substitution	Nonsynonymous coding	10%
MM12T	CCDC116	coiled-coil domain containing 116	CCDS13791.1	chr22_21987538-21987538_C_T	23A>V	Substitution	Nonsynonymous coding	48%
MM12T	CCDC116	coiled-coil domain containing 116	CCDS13791.1	chr22_21988641-21988641_C_T	135R>W	Substitution	Nonsynonymous coding	34%
MM12T	CCDC116	coiled-coil domain containing 116	CCDS13791.1	chr22_21988657-21988657_C_T	140P>L	Substitution	Nonsynonymous coding	23%
MM12T	CCDC116	coiled-coil domain containing 116	CCDS13791.1	chr22_21988734-21988734_G_A	166A>T	Substitution	Nonsynonymous coding	15%
MM12T	CCDC127	coiled-coil domain containing 127	CCDS3852.1	chr5_205864-205864_G_A	111R>C	Substitution	Nonsynonymous coding	35%
MM12T	CCDC137	coiled-coil domain containing 137	CCDS42400.1	chr17_79637467-79637467__A	NA	Insertion	Frameshift	15%
MM12T	CCDC141	coiled-coil domain containing 141	CCDS2281.1	chr2_179736886-179736886_C_T	110A>T	Substitution	Nonsynonymous coding	21%
MM12T	CCDC147	coiled-coil domain containing 147	CCDS31282.1	chr10_106166524-106166524_G_T	743E>D	Substitution	Nonsynonymous coding	17%

MM12T	CCDC147	coiled-coil domain containing 147	CCDS31282.1	chr10_106214226-106214226_C_T	853P>S	Substitution	Nonsynonymous coding	24%
MM12T	CCDC147	coiled-coil domain containing 147	CCDS31282.1	chr10_106128310-106128310_G_T	308E>X	Substitution	Nonsense	28%
MM12T	CCDC148	coiled-coil domain containing 148	CCDS33304.1	chr2_159077168-159077168_G_A	437L>F	Substitution	Nonsynonymous coding	13%
MM12T	CCDC158	coiled-coil domain containing 158	CCDS43242.1	chr4_77250101-77250101_C_T	984A>T	Substitution	Nonsynonymous coding	17%
MM12T	CCDC158	coiled-coil domain containing 158	CCDS43242.1	chr4_77252521-77252521_C_T	969S>N	Substitution	Nonsynonymous coding	11%
MM12T	CCDC164	coiled-coil domain containing 164	ENST00000439066	chr2_26671259-26671259_G_A	11V>I	Substitution	Nonsynonymous coding	38%
MM12T	CCDC17	coiled-coil domain containing 17	CCDS44131.1	chr1_46088296-46088296_C_A	230G>C	Substitution	Nonsynonymous coding	34%
MM12T	CCDC174	coiled-coil domain containing 174	CCDS2620.2	chr3_14708937-14708937_G_A	244R>Q	Substitution	Nonsynonymous coding	36%
MM12T	CCDC176	coiled-coil domain containing 176	CCDS32119.2	chr14_74486219-74486219_G_A	13S>N	Substitution	Nonsynonymous coding	10%
MM12T	CCDC24	coiled-coil domain containing 24	CCDS507.1	chr1_44457899-44457899_G_A	48A>T	Substitution	Nonsynonymous coding	11%
MM12T	CCDC34	coiled-coil domain containing 34	CCDS31448.1	chr11_27362359-27362359_T_A	264E>V	Substitution	Nonsynonymous coding	14%
MM12T	CCDC40	coiled-coil domain containing 40	CCDS42395.1	chr17_78069153-78069153_C_T	975A>V	Substitution	Nonsynonymous coding	20%
MM12T	CCDC40	coiled-coil domain containing 40	CCDS42395.1	chr17_78073389-78073389_C_T	1082R>C	Substitution	Nonsynonymous coding	12%
MM12T	CCDC47	coiled-coil domain containing 47	CCDS11643.1	chr17_61842197-61842197_G_A	92T>I	Substitution	Nonsynonymous coding	19%
MM12T	CCDC50	coiled-coil domain containing 50	CCDS33912.1	chr3_191078926-191078926_G_A	103R>H	Substitution	Nonsynonymous coding	12%
MM12T	CCDC64B	coiled-coil domain containing 64B	CCDS45393.1	chr16_3081028-3081028_G_A	136R>C	Substitution	Nonsynonymous coding	29%
MM12T	CCDC70	coiled-coil domain containing 70	CCDS9431.1	chr13_52440019-52440019_G_A	169A>T	Substitution	Nonsynonymous coding	13%
MM12T	CCDC78	coiled-coil domain containing 78	CCDS32353.1	chr16_774695-774695_C_T	251A>T	Substitution	Nonsynonymous coding	10%
MM12T	CCDC85C	coiled-coil domain containing 85C	CCDS45161.1	chr14_99983505-99983505_C_T	331A>T	Substitution	Nonsynonymous coding	38%
MM12T	CCDC88B	coiled-coil domain containing 88B	CCDS8072.2	chr11_64118723-64118723_G_A	985R>H	Substitution	Nonsynonymous coding	18%
MM12T	CCDC88B	coiled-coil domain containing 88B	CCDS8072.2	chr11_64119821-64119821_C_T	1107R>W	Substitution	Nonsynonymous coding	20%
MM12T	CCDC88B	coiled-coil domain containing 88B	CCDS8072.2	chr11_64120704-64120704_C_G	1227Q>E	Substitution	Nonsynonymous coding	12%
MM12T	CCDC88B	coiled-coil domain containing 88B	CCDS8072.2	chr11_64121210-64121210_G_A	1286R>H	Substitution	Nonsynonymous coding	12%
MM12T	CCDC88C	coiled-coil domain containing 88C	CCDS45151.1	chr14_91739847-91739847_C_T	1737A>T	Substitution	Nonsynonymous coding	29%
MM12T	CCDC88C	coiled-coil domain containing 88C	CCDS45151.1	chr14_91808751-91808751_C_A	152Q>H	Substitution	Nonsynonymous coding	32%
MM12T	CCDC92	coiled-coil domain containing 92	CCDS9256.1	chr12_124427914-124427914_C_T	54R>Q	Substitution	Nonsynonymous coding	26%
MM12T	CCDC93	coiled-coil domain containing 93	CCDS2121.2	chr2_118704407-118704407_C_T	426A>T	Substitution	Nonsynonymous coding	25%
MM12T	CCL17	chemokine (C-C motif) ligand 17	CCDS10780.1	chr16_57449049-57449049_C_T	43P>S	Substitution	Nonsynonymous coding	31%
MM12T	CCM2	cerebral cavernous malformation 2	CCDS34630.1	chr7_45115589-45115589_G_A	444R>H	Substitution	Nonsynonymous coding	11%
MM12T	CCM2L	cerebral cavernous malformation 2-like	ENST00000300415	chr20_30618809-30618809_C_T	470R>W	Substitution	Nonsynonymous coding	36%
MM12T	CCNA1	cyclin A1	CCDS9357.1	chr13_37012842-37012842_G_A	244R>H	Substitution	Nonsynonymous coding	18%
MM12T	CCNDBP1	cyclin D-type binding-protein 1	CCDS10092.1	chr15_43486617-43486617_C_T	309A>V	Substitution	Nonsynonymous coding	39%

MM12T	CCNF	cyclin F	CCDS10467.1	chr16_2487219-2487219_G_A	146A>T	Substitution	Nonsynonymous coding	34%
MM12T	CCP110	centriolar coiled coil protein 110kDa	CCDS10579.1	chr16_19548126-19548126_C_T	379R>C	Substitution	Nonsynonymous coding	17%
MM12T	CCR10	chemokine (C-C motif) receptor 10	CCDS11435.1	chr17_40832247-40832247_C_T	138R>H	Substitution	Nonsynonymous coding	22%
MM12T	CCR10	chemokine (C-C motif) receptor 10	CCDS11435.1	chr17_40832502-40832502_G_A	53A>V	Substitution	Nonsynonymous coding	30%
MM12T	CCR9	chemokine (C-C motif) receptor 9	CCDS2732.1	chr3_45943064-45943064_G_A	262V>I	Substitution	Nonsynonymous coding	12%
MM12T	CCSER1	coiled-coil serine-rich protein 1	CCDS47099.1	chr4_91321226-91321226_G_T	517D>Y	Substitution	Nonsynonymous coding	33%
MM12T	CCT3	chaperonin containing TCP1, subunit 3 (gamma)	CCDS1140.2	chr1_156286993-156286993_C_T	313R>H	Substitution	Nonsynonymous coding	17%
MM12T	CCT5	chaperonin containing TCP1, subunit 5 (epsilon)	CCDS3877.1	chr5_10260992-10260992_C_T	321A>V	Substitution	Nonsynonymous coding	40%
MM12T	CCT6B	chaperonin containing TCP1, subunit 6B (zeta 2)	CCDS32617.1	chr17_33255086-33255086_C_T	525G>E	Substitution	Nonsynonymous coding	13%
MM12T	CCT8L1P	chaperonin containing TCP1, subunit 8 (theta)-like 1, pseudogene	ENST00000021776	chr7_152143294-152143294_G_A	245A>T	Substitution	Nonsynonymous coding	44%
MM12T	CD163L1	CD163 molecule-like 1	CCDS8577.1	chr12_7548829-7548829_C_T	638A>T	Substitution	Nonsynonymous coding	12%
MM12T	CD19	CD19 molecule	CCDS10644.1	chr16_28948588-28948588_C_A	ISV-4>	Substitution	Splice site acceptor	24%
MM12T	CD248	CD248 molecule, endosialin	CCDS8134.1	chr11_66083430-66083430_C_T	357G>S	Substitution	Nonsynonymous coding	36%
MM12T	CD300LB	CD300 molecule-like family member b	CCDS11700.1	chr17_72522003-72522003_C_T	122R>H	Substitution	Nonsynonymous coding	16%
MM12T	CD300LG	CD300 molecule-like family member g	CCDS11470.1	chr17_41930333-41930333_C_T	145R>C	Substitution	Nonsynonymous coding	34%
MM12T	CD34	CD34 molecule	ENST00000367036	chr1_208063101-208063101_A_	NA	Deletion	Frameshift	28%
MM12T	CD37	CD37 molecule	CCDS12760.1	chr19_49838840-49838840_C_A	15L>I	Substitution	Nonsynonymous coding	31%
MM12T	CD38	CD38 molecule	CCDS3417.1	chr4_15818213-15818213_G_T	105D>Y	Substitution	Nonsynonymous coding	31%
MM12T	CD82	CD82 molecule	CCDS7909.1	chr11_44640638-44640638_G_A	256V>I	Substitution	Nonsynonymous coding	39%
MM12T	CD97	CD97 molecule	CCDS32929.1	chr19_14512503-14512503_G_A	372V>I	Substitution	Nonsynonymous coding	12%
MM12T	CDAN1	codanin 1	CCDS32209.1	chr15_43028229-43028229_C_T	206R>Q	Substitution	Nonsynonymous coding	33%
MM12T	CDC14A	cell division cycle 14A	CCDS770.1	chr1_100889815-100889815_G_T	116R>I	Substitution	Nonsynonymous coding	29%
MM12T	CDC14B	cell division cycle 14B	ENST00000375236	chr9_99278021-99278021_A_C	466I>S	Substitution	Nonsynonymous coding	37%
MM12T	CDC23	cell division cycle 23	CCDS4200.2	chr5_137542340-137542340_C_T	90A>T	Substitution	Nonsynonymous coding	22%
MM12T	CDC34	cell division cycle 34	CCDS12030.1	chr19_541398-541398_C_T	186T>M	Substitution	Nonsynonymous coding	47%
MM12T	CDC42BPB	CDC42 binding protein kinase beta (DMPK-like)	CCDS9978.1	chr14_103406195-103406195_G_A	1561P>S	Substitution	Nonsynonymous coding	12%
MM12T	CDC42BPB	CDC42 binding protein kinase beta (DMPK-like)	CCDS9978.1	chr14_103447355-103447355_G_A	299R>X	Substitution	Nonsense	27%
MM12T	CDC42BPG	CDC42 binding protein kinase gamma (DMPK-like)	CCDS31601.1	chr11_64600744-64600744_G_A	897S>L	Substitution	Nonsynonymous coding	24%
MM12T	CDC42BPG	CDC42 binding protein kinase gamma (DMPK-like)	CCDS31601.1	chr11_64606214-64606214_G_A	346A>V	Substitution	Nonsynonymous coding	24%
MM12T	CDC42EP1	CDC42 effector protein (Rho GTPase binding) 1	CCDS13949.1	chr22_37962639-37962639_C_	NA	Deletion	Frameshift	25%
MM12T	CDC42EP1	CDC42 effector protein (Rho GTPase binding) 1	CCDS13949.1	chr22_37964544-37964544_C_T	298A>V	Substitution	Nonsynonymous coding	23%
MM12T	CDC7	cell division cycle 7	CCDS734.1	chr1_91985737-91985737_C_T	411R>X	Substitution	Nonsense	25%

MM12T	CDCA2	cell division cycle associated 2	CCDS6049.1	chr8_25343318-25343318_C_A	470S>Y	Substitution	Nonsynonymous coding	32%
MM12T	CDCA2	cell division cycle associated 2	CCDS6049.1	chr8_25364551-25364551_G_A	790C>Y	Substitution	Nonsynonymous coding	33%
MM12T	CDCP1	CUB domain containing protein 1	CCDS2727.1	chr3_45136887-45136887_G_T	400L>I	Substitution	Nonsynonymous coding	31%
MM12T	CDCP2	CUB domain containing protein 2	CCDS588.2	chr1_54605319-54605319__GGG	NA	Insertion	In-frame insertion	13%
MM12T	CDH12	cadherin 12, type 2 (N-cadherin 2)	CCDS3890.1	chr5_21755942-21755942_G_A	548A>V	Substitution	Nonsynonymous coding	42%
MM12T	CDH13	cadherin 13, H-cadherin (heart)	NM_001257	chr16_83520164-83520164_G_T	288Q>H	Substitution	Nonsynonymous coding	16%
MM12T	CDH15	cadherin 15, type 1, M-cadherin (myotubule)	CCDS10976.1	chr16_89257781-89257781_G_A	426A>T	Substitution	Nonsynonymous coding	30%
MM12T	CDH16	cadherin 16, KSP-cadherin	CCDS10823.1	chr16_66946676-66946676_G_T	391F>L	Substitution	Nonsynonymous coding	32%
MM12T	CDH17	cadherin 17, LI cadherin (liver-intestine)	CCDS6260.1	chr8_95143154-95143154_C_T	745R>H	Substitution	Nonsynonymous coding	21%
MM12T	CDH2	cadherin 2, type 1, N-cadherin (neuronal)	CCDS11891.1	chr18_25593816-25593816_G_A	77A>V	Substitution	Nonsynonymous coding	21%
MM12T	CDH22	cadherin 22, type 2	CCDS13395.1	chr20_44803572-44803572_G_A	687A>V	Substitution	Nonsynonymous coding	32%
MM12T	CDH23	cadherin-related 23	NM_022124	chr10_73567275-73567275_G_T	2771G>C	Substitution	Nonsynonymous coding	22%
MM12T	CDH23	cadherin-related 23	NM_022124	chr10_73567389-73567389_C_T	2809R>C	Substitution	Nonsynonymous coding	31%
MM12T	CDH26	cadherin 26	CCDS13485.1	chr20_58587784-58587784_A_	NA	Deletion	Frameshift	21%
MM12T	CDH26	cadherin 26	CCDS13485.1	chr20_58569512-58569512_C_T	545A>V	Substitution	Nonsynonymous coding	24%
MM12T	CDH3	cadherin 3, type 1, P-cadherin (placental)	CCDS10868.1	chr16_68713873-68713873_G_A	288R>Q	Substitution	Nonsynonymous coding	49%
MM12T	CDH3	cadherin 3, type 1, P-cadherin (placental)	CCDS10868.1	chr16_68732130-68732130_C_T	773P>S	Substitution	Nonsynonymous coding	32%
MM12T	CDH5	cadherin 5, type 2 (vascular endothelium)	CCDS10804.1	chr16_66413272-66413272_C_T	11S>L	Substitution	Nonsynonymous coding	13%
MM12T	CDH7	cadherin 7, type 2	CCDS11993.1	chr18_63511085-63511085_G_A	340R>Q	Substitution	Nonsynonymous coding	21%
MM12T	CDH9	cadherin 9, type 2 (T1-cadherin)	CCDS3893.1	chr5_26915852-26915852_G_A	137R>W	Substitution	Nonsynonymous coding	12%
MM12T	CDHR3	cadherin-related family member 3	CCDS47684.1	chr7_105645064-105645064_G_A	328R>H	Substitution	Nonsynonymous coding	11%
MM12T	CDIPT	CDP-diacylglycerol--inositol 3-phosphatidyltransferase	ENST00000403894	chr16_29872418-29872418_G_A	167A>V	Substitution	Nonsynonymous coding	11%
MM12T	CDK12	cyclin-dependent kinase 12	CCDS11337.1	chr17_37619337-37619337_G_A	338S>N	Substitution	Nonsynonymous coding	34%
MM12T	CDK12	cyclin-dependent kinase 12	CCDS11337.1	chr17_37687463-37687463_G_A	1456G>D	Substitution	Nonsynonymous coding	27%
MM12T	CDK18	cyclin-dependent kinase 18	CCDS1454.1	chr1_205492351-205492351_G_A	19R>H	Substitution	Nonsynonymous coding	29%
MM12T	CDK2	cyclin-dependent kinase 2	CCDS8898.1	chr12_56365333-56365333_G_A	274R>Q	Substitution	Nonsynonymous coding	28%
MM12T	CDK20	cyclin-dependent kinase 20	CCDS35060.1	chr9_90586201-90586201_G_A	80A>V	Substitution	Nonsynonymous coding	32%
MM12T	CDK5RAP1	CDK5 regulatory subunit associated protein 1	CCDS13219.1	chr20_31958339-31958339_T_G	453N>T	Substitution	Nonsynonymous coding	12%
MM12T	CDK5RAP1	CDK5 regulatory subunit associated protein 1	CCDS13219.1	chr20_31984670-31984670_A_T	67H>Q	Substitution	Nonsynonymous coding	11%
MM12T	CDK8	cyclin-dependent kinase 8	CCDS9317.1	chr13_26967528-26967528_T_C	224F>S	Substitution	Nonsynonymous coding	11%
MM12T	CDK9	cyclin-dependent kinase 9	CCDS6879.1	chr9_130549830-130549830_C_A	70L>I	Substitution	Nonsynonymous coding	38%
MM12T	CDKL3	cyclin-dependent kinase-like 3	CCDS47264.1	chr5_133685943-133685943_C_T	179G>E	Substitution	Nonsynonymous coding	12%

MM12T	CDKL5	cyclin-dependent kinase-like 5	CCDS14186.1	chrX_18622500-18622500_G_A	486A>T	Substitution	Nonsynonymous coding	38%
MM12T	CDKL5	cyclin-dependent kinase-like 5	CCDS14186.1	chrX_18622570-18622570_C_T	509A>V	Substitution	Nonsynonymous coding	12%
MM12T	CDKL5	cyclin-dependent kinase-like 5	CCDS14186.1	chrX_18582644-18582644_T_C	ISV+2>	Substitution	Splice site donor	26%
MM12T	CDO1	cysteine dioxygenase, type I	CCDS4121.1	chr5_115142159-115142159_G_T	154L>I	Substitution	Nonsynonymous coding	34%
MM12T	CDS1	CDP-diacylglycerol synthase (phosphatidate cytidylyltransferase) 1	CCDS3608.1	chr4_85564231-85564231_G_A	363A>T	Substitution	Nonsynonymous coding	37%
MM12T	CEBPZ	CCAAT/enhancer binding protein (C/EBP), zeta	CCDS1787.1	chr2_37455465-37455465_C_T	291E>K	Substitution	Nonsynonymous coding	28%
MM12T	CECR6	cat eye syndrome chromosome region, candidate 6	CCDS13740.1	chr22_17600744-17600744_C_T	425R>H	Substitution	Nonsynonymous coding	33%
MM12T	CECR6	cat eye syndrome chromosome region, candidate 6	CCDS13740.1	chr22_17600843-17600843_G_A	392A>V	Substitution	Nonsynonymous coding	41%
MM12T	CEL	carboxyl ester lipase (bile salt-stimulated lipase)	CCDS43896.1	chr9_135940612-135940612_G_A	179A>T	Substitution	Nonsynonymous coding	12%
MM12T	CELF3	CUGBP, Elav-like family member 3	CCDS1002.1	chr1_151680363-151680363_G_A	179R>X	Substitution	Nonsense	32%
MM12T	CELSR1	cadherin, EGF LAG seven-pass G-type receptor 1 (flamingo homolog, Drosophila)	CCDS14076.1	chr22_46859880-46859880_C_T	1303D>N	Substitution	Nonsynonymous coding	13%
MM12T	CELSR1	cadherin, EGF LAG seven-pass G-type receptor 1 (flamingo homolog, Drosophila)	CCDS14076.1	chr22_46931873-46931873_C_T	399V>I	Substitution	Nonsynonymous coding	15%
MM12T	CELSR2	cadherin, EGF LAG seven-pass G-type receptor 2 (flamingo homolog, Drosophila)	CCDS796.1	chr1_109794842-109794842_G_A	714S>N	Substitution	Nonsynonymous coding	31%
MM12T	CELSR2	cadherin, EGF LAG seven-pass G-type receptor 2 (flamingo homolog, Drosophila)	CCDS796.1	chr1_109801524-109801524_G_A	1261V>M	Substitution	Nonsynonymous coding	36%
MM12T	CELSR3	cadherin, EGF LAG seven-pass G-type receptor 3 (flamingo homolog, Drosophila)	CCDS2775.1	chr3_48681013-48681013_G_T	2755L>I	Substitution	Nonsynonymous coding	37%
MM12T	CELSR3	cadherin, EGF LAG seven-pass G-type receptor 3 (flamingo homolog, Drosophila)	CCDS2775.1	chr3_48687984-48687984_C_T	2134R>K	Substitution	Nonsynonymous coding	16%
MM12T	CELSR3	cadherin, EGF LAG seven-pass G-type receptor 3 (flamingo homolog, Drosophila)	CCDS2775.1	chr3_48697594-48697594_C_T	825R>H	Substitution	Nonsynonymous coding	11%
MM12T	CELSR3	cadherin, EGF LAG seven-pass G-type receptor 3 (flamingo homolog, Drosophila)	CCDS2775.1	chr3_48698662-48698662_C_T	469R>H	Substitution	Nonsynonymous coding	11%
MM12T	CELSR3	cadherin, EGF LAG seven-pass G-type receptor 3 (flamingo homolog, Drosophila)	CCDS2775.1	chr3_48698740-48698740_C_T	443R>H	Substitution	Nonsynonymous coding	30%
MM12T	CELSR3	cadherin, EGF LAG seven-pass G-type receptor 3 (flamingo homolog, Drosophila)	CCDS2775.1	chr3_48698791-48698791_C_T	426R>H	Substitution	Nonsynonymous coding	32%
MM12T	CENPB	centromere protein B, 80kDa	CCDS13064.1	chr20_3766572-3766572_C_T	187A>T	Substitution	Nonsynonymous coding	29%
MM12T	CENPE	centromere protein E, 312kDa	CCDS34042.1	chr4_104030070-104030070_G_A	2634P>L	Substitution	Nonsynonymous coding	26%
MM12T	CENPE	centromere protein E, 312kDa	CCDS34042.1	chr4_104117997-104117997_G_T	23L>I	Substitution	Nonsynonymous coding	32%
MM12T	CENPF	centromere protein F, 350/400kDa	CCDS31023.1	chr1_214813419-214813419_C_T	580H>Y	Substitution	Nonsynonymous coding	44%
MM12T	CENPF	centromere protein F, 350/400kDa	CCDS31023.1	chr1_214819533-214819533_C_T	2207T>M	Substitution	Nonsynonymous coding	38%
MM12T	CENPI	centromere protein I	CCDS14479.1	chrX_100382190-100382190_C_T	281R>W	Substitution	Nonsynonymous coding	30%
MM12T	CENPJ	centromere protein J	CCDS9310.1	chr13_25466782-25466782_G_A	1072A>V	Substitution	Nonsynonymous coding	13%
MM12T	CEP128	centrosomal protein 128kDa	CCDS32130.1	chr14_81304618-81304618_T_G	285E>A	Substitution	Nonsynonymous coding	18%
MM12T	CEP128	centrosomal protein 128kDa	CCDS32130.1	chr14_81329104-81329104_C_A	253Q>H	Substitution	Nonsynonymous coding	38%
MM12T	CEP164	centrosomal protein 164kDa	CCDS31683.1	chr11_117222543-117222543_G_A	78A>T	Substitution	Nonsynonymous coding	24%
MM12T	CEP164	centrosomal protein 164kDa	CCDS31683.1	chr11_117263749-117263749_G_T	841Q>H	Substitution	Nonsynonymous coding	12%
MM12T	CEP170B	centrosomal protein 170B	CCDS45175.1	chr14_105353812-105353812_G_A	1079R>H	Substitution	Nonsynonymous coding	32%

MM12T	CEP170B	centrosomal protein 170B	CCDS45175.1	chr14_105353976-105353976_G_A	1134A>T	Substitution	Nonsynonymous coding	35%
MM12T	CEP192	centrosomal protein 192kDa	CCDS32792.1	chr18_13068895-13068895_G_A	1027E>K	Substitution	Nonsynonymous coding	15%
MM12T	CEP192	centrosomal protein 192kDa	CCDS32792.1	chr18_13095603-13095603_C_T	1523P>L	Substitution	Nonsynonymous coding	28%
MM12T	CEP250	centrosomal protein 250kDa	CCDS13255.1	chr20_34060643-34060643_G_A	399R>H	Substitution	Nonsynonymous coding	24%
MM12T	CEP250	centrosomal protein 250kDa	CCDS13255.1	chr20_34090717-34090717_G_T	1507R>M	Substitution	Nonsynonymous coding	32%
MM12T	CEP250	centrosomal protein 250kDa	CCDS13255.1	chr20_34090455-34090455_C_T	1420Q>X	Substitution	Nonsense	15%
MM12T	CEP290	centrosomal protein 290kDa	NM_025114	chr12_88465149-88465149_C_T	1978R>Q	Substitution	Nonsynonymous coding	26%
MM12T	CEP350	centrosomal protein 350kDa	CCDS1336.1	chr1_179965891-179965891_G_A	200R>Q	Substitution	Nonsynonymous coding	20%
MM12T	CEP350	centrosomal protein 350kDa	CCDS1336.1	chr1_179989356-179989356_G_A	816S>N	Substitution	Nonsynonymous coding	21%
MM12T	CEP44	centrosomal protein 44kDa	CCDS47163.1	chr4_175225516-175225516_G_A	168G>E	Substitution	Nonsynonymous coding	15%
MM12T	CEP57L1	centrosomal protein 57kDa-like 1	CCDS5071.1	chr6_109475148-109475148_C_T	192A>V	Substitution	Nonsynonymous coding	15%
MM12T	CEP72	centrosomal protein 72kDa	CCDS34126.1	chr5_624695-624695_G_A	ISV+1>	Substitution	Splice site donor	12%
MM12T	CEP78	centrosomal protein 78kDa	CCDS47984.1	chr9_80854969-80854969_G_A	95R>H	Substitution	Nonsynonymous coding	18%
MM12T	CEP97	centrosomal protein 97kDa	CCDS2944.1	chr3_101481355-101481355_G_A	615R>H	Substitution	Nonsynonymous coding	17%
MM12T	CEPT1	choline/ethanolamine phosphotransferase 1	CCDS830.1	chr1_111690484-111690484_C_T	50R>W	Substitution	Nonsynonymous coding	27%
MM12T	CERS6	ceramide synthase 6	CCDS2228.1	chr2_169626102-169626102_C_T	362A>V	Substitution	Nonsynonymous coding	15%
MM12T	CES3	carboxylesterase 3	CCDS10826.1	chr16_67006810-67006810_A_G	525Q>R	Substitution	Nonsynonymous coding	13%
MM12T	CES5A	carboxylesterase 5A	CCDS45490.1	chr16_55886822-55886822_G_T	415P>H	Substitution	Nonsynonymous coding	25%
MM12T	CFB	complement factor B [Source:HGNC Symbol;Acc:1037]	CCDS4729.1	chr6_31916712-31916712_G_A	381R>H	Substitution	Nonsynonymous coding	23%
MM12T	CFH	complement factor H	CCDS1385.1	chr1_196695660-196695660_C_T	645T>M	Substitution	Nonsynonymous coding	17%
MM12T	CGNL1	cingulin-like 1	CCDS10161.1	chr15_57731032-57731032_C_T	279R>W	Substitution	Nonsynonymous coding	26%
MM12T	CGNL1	cingulin-like 1	CCDS10161.1	chr15_57745911-57745911_G_T	695Q>H	Substitution	Nonsynonymous coding	28%
MM12T	CGNL1	cingulin-like 1	CCDS10161.1	chr15_57816877-57816877_G_A	989M>I	Substitution	Nonsynonymous coding	16%
MM12T	CHAF1A	chromatin assembly factor 1, subunit A (p150)	CCDS32875.1	chr19_4422768-4422768_G_A	408R>H	Substitution	Nonsynonymous coding	12%
MM12T	CHAMP1	chromosome alignment maintaining phosphoprotein 1	CCDS9545.1	chr13_115090092-115090092_C_T	259P>S	Substitution	Nonsynonymous coding	33%
MM12T	CHD1	chromodomain helicase DNA binding protein 1	CCDS34204.1	chr5_98221325-98221325_A_C	842L>R	Substitution	Nonsynonymous coding	16%
MM12T	CHD2	chromodomain helicase DNA binding protein 2	CCDS10374.2	chr15_93496673-93496673_A_G	530H>R	Substitution	Nonsynonymous coding	29%
MM12T	CHD3	chromodomain helicase DNA binding protein 3	CCDS32553.2	chr17_7797799-7797799_A_G	440Y>C	Substitution	Nonsynonymous coding	13%
MM12T	CHD3	chromodomain helicase DNA binding protein 3	CCDS32553.2	chr17_7810786-7810786_G_A	1694R>H	Substitution	Nonsynonymous coding	28%
MM12T	CHD4	chromodomain helicase DNA binding protein 4	CCDS8552.1	chr12_6707111-6707111_C_T	614R>H	Substitution	Nonsynonymous coding	13%
MM12T	CHD4	chromodomain helicase DNA binding protein 4	CCDS8552.1	chr12_6707509-6707509_G_T	522P>H	Substitution	Nonsynonymous coding	28%
MM12T	CHD4	chromodomain helicase DNA binding protein 4	CCDS8552.1	chr12_6709792-6709792_G_A	324A>V	Substitution	Nonsynonymous coding	13%

MM12T	CHD4	chromodomain helicase DNA binding protein 4	CCDS8552.1	chr12_6680190-6680190_G_A	1856Q>X	Substitution	Nonsense	36%
MM12T	CHD6	chromodomain helicase DNA binding protein 6	CCDS13317.1	chr20_40034047-40034047_C_T	2445R>Q	Substitution	Nonsynonymous coding	36%
MM12T	CHD6	chromodomain helicase DNA binding protein 6	CCDS13317.1	chr20_40081497-40081497_G_A	1069S>L	Substitution	Nonsynonymous coding	33%
MM12T	CHD7	chromodomain helicase DNA binding protein 7	CCDS47865.1	chr8_61655180-61655180_T_C	397S>P	Substitution	Nonsynonymous coding	32%
MM12T	CHD7	chromodomain helicase DNA binding protein 7	CCDS47865.1	chr8_61765633-61765633_C_A	2117L>I	Substitution	Nonsynonymous coding	12%
MM12T	CHD7	chromodomain helicase DNA binding protein 7	CCDS47865.1	chr8_61778271-61778271_G_A	2925A>T	Substitution	Nonsynonymous coding	37%
MM12T	CHD9	chromodomain helicase DNA binding protein 9	CCDS45485.1	chr16_53276755-53276755_C_T	961R>C	Substitution	Nonsynonymous coding	33%
MM12T	CHD9	chromodomain helicase DNA binding protein 9	CCDS45485.1	chr16_53326813-53326813_C_T	1787R>C	Substitution	Nonsynonymous coding	35%
MM12T	CHD9	chromodomain helicase DNA binding protein 9	CCDS45485.1	chr16_53337698-53337698_G_A	1927R>H	Substitution	Nonsynonymous coding	31%
MM12T	CHD9	chromodomain helicase DNA binding protein 9	CCDS45485.1	chr16_53337880-53337880_C_T	1988R>C	Substitution	Nonsynonymous coding	31%
MM12T	CHDH	choline dehydrogenase	CCDS2873.1	chr3_53856621-53856621_C_T	251R>H	Substitution	Nonsynonymous coding	30%
MM12T	CHGA	chromogranin A (parathyroid secretory protein 1)	CCDS9906.1	chr14_93392955-93392955_G_A	33M>I	Substitution	Nonsynonymous coding	18%
MM12T	CHI3L1	chitinase 3-like 1 (cartilage glycoprotein-39)	CCDS1435.1	chr1_203150364-203150364_G_A	213R>C	Substitution	Nonsynonymous coding	16%
MM12T	CHI3L2	chitinase 3-like 2	CCDS30802.1	chr1_111778330-111778330_G_A	184V>I	Substitution	Nonsynonymous coding	20%
MM12T	CHID1	chitinase domain containing 1	CCDS44511.1	chr11_883199-883199_C_T	328G>D	Substitution	Nonsynonymous coding	30%
MM12T	CHKA	choline kinase alpha	CCDS8178.1	chr11_67842221-67842221_C_T	198G>D	Substitution	Nonsynonymous coding	11%
MM12T	CHMP2A	charged multivesicular body protein 2A	CCDS12986.1	chr19_59065534-59065534_G_A	16R>W	Substitution	Nonsynonymous coding	16%
MM12T	CHODL	chondrolectin	CCDS13570.1	chr21_19635132-19635132_T_C	220V>A	Substitution	Nonsynonymous coding	56%
MM12T	CHP2	calcineurin-like EF-hand protein 2	CCDS10617.1	chr16_23767029-23767029_G_A	39D>N	Substitution	Nonsynonymous coding	14%
MM12T	CHPF2	chondroitin polymerizing factor 2	CCDS34779.1	chr7_150934469-150934469_C_T	341R>W	Substitution	Nonsynonymous coding	40%
MM12T	CHRD	chordin	CCDS3266.1	chr3_184099345-184099345_G_T	148E>D	Substitution	Nonsynonymous coding	15%
MM12T	CHRD	chordin	CCDS3266.1	chr3_184102469-184102469_G_A	529A>T	Substitution	Nonsynonymous coding	13%
MM12T	CHRM1	cholinergic receptor, muscarinic 1	CCDS8040.1	chr11_62678100-62678100_G_A	158A>V	Substitution	Nonsynonymous coding	12%
MM12T	CHRNA1	cholinergic receptor, nicotinic, alpha 1 (muscle)	CCDS33331.1	chr2_175612880-175612880_C_T	474R>Q	Substitution	Nonsynonymous coding	12%
MM12T	CHRNA2	cholinergic receptor, nicotinic, alpha 2 (neuronal)	CCDS6059.1	chr8_27320798-27320798_C_T	388E>K	Substitution	Nonsynonymous coding	41%
MM12T	CHRNB2	cholinergic receptor, nicotinic, beta 2 (neuronal)	CCDS1070.1	chr1_154541989-154541989_C_A	39P>H	Substitution	Nonsynonymous coding	31%
MM12T	CHRNB3	cholinergic receptor, nicotinic, beta 3 (neuronal)	CCDS6134.1	chr8_42587393-42587393_G_A	315V>M	Substitution	Nonsynonymous coding	18%
MM12T	CHRND	cholinergic receptor, nicotinic, delta (muscle)	CCDS2494.1	chr2_233390969-233390969_C_T	15A>V	Substitution	Nonsynonymous coding	30%
MM12T	CHRND	cholinergic receptor, nicotinic, delta (muscle)	CCDS2494.1	chr2_233396161-233396161_C_T	307P>L	Substitution	Nonsynonymous coding	34%
MM12T	CHRNE	cholinergic receptor, nicotinic, epsilon (muscle)	CCDS11058.1	chr17_4802840-4802840_C_T	319V>I	Substitution	Nonsynonymous coding	16%
MM12T	CHRNE	cholinergic receptor, nicotinic, epsilon (muscle)	CCDS11058.1	chr17_4804426-4804426_C_T	221A>T	Substitution	Nonsynonymous coding	26%
MM12T	CHRNE	cholinergic receptor, nicotinic, epsilon (muscle)	CCDS11058.1	chr17_4804465-4804465_C_T	208D>N	Substitution	Nonsynonymous coding	18%

MM12T	CHRNE	cholinergic receptor, nicotinic, epsilon (muscle)	CCDS11058.1	chr17_4805293-4805293_C_T	145R>H	Substitution	Nonsynonymous coding	12%
MM12T	CHST10	carbohydrate sulfotransferase 10	CCDS2047.1	chr2_101009786-101009786_T_C	331D>G	Substitution	Nonsynonymous coding	40%
MM12T	CHST10	carbohydrate sulfotransferase 10	CCDS2047.1	chr2_101012038-101012038_C_T	156V>M	Substitution	Nonsynonymous coding	41%
MM12T	CHST2	carbohydrate (N-acetylglucosamine-6-O) sulfotransferase 2	CCDS3129.1	chr3_142840706-142840706_C_T	350R>C	Substitution	Nonsynonymous coding	29%
MM12T	CHST6	carbohydrate (N-acetylglucosamine 6-O) sulfotransferase 6	CCDS10918.1	chr16_75513690-75513690_C_T	13A>T	Substitution	Nonsynonymous coding	13%
MM12T	CHST7	carbohydrate (N-acetylglucosamine 6-O) sulfotransferase 7	CCDS14268.1	chrX_46434226-46434226_G_A	287R>H	Substitution	Nonsynonymous coding	33%
MM12T	CHSY1	chondroitin sulfate synthase 1	NM_014918	chr15_101718260-101718260_G_T	581A>D	Substitution	Nonsynonymous coding	13%
MM12T	CHTF18	CTF18, chromosome transmission fidelity factor 18 homolog (S. cerevisiae)	CCDS45371.1	chr16_843011-843011_G_A	540A>T	Substitution	Nonsynonymous coding	34%
MM12T	CIDEC	cell death-inducing DFFA-like effector c	CCDS2587.1	chr3_9918821-9918821_G_A	46R>C	Substitution	Nonsynonymous coding	38%
MM12T	CILP2	cartilage intermediate layer protein 2	CCDS12405.1	chr19_19655248-19655248_G_A	632G>S	Substitution	Nonsynonymous coding	25%
MM12T	CKAP2	cytoskeleton associated protein 2	CCDS41893.1	chr13_53048165-53048165_C_T	584T>M	Substitution	Nonsynonymous coding	34%
MM12T	CKAP5	cytoskeleton associated protein 5	CCDS31477.1	chr11_46780906-46780906_T_C	1494D>G	Substitution	Nonsynonymous coding	22%
MM12T	CKMT1B	creatine kinase, mitochondrial 1B	CCDS10097.1	chr15_43891412-43891412_C_T	399R>C	Substitution	Nonsynonymous coding	15%
MM12T	CKMT2	creatine kinase, mitochondrial 2 (sarcomeric)	CCDS4053.1	chr5_80559317-80559317_G_A	341R>H	Substitution	Nonsynonymous coding	34%
MM12T	CKMT2	creatine kinase, mitochondrial 2 (sarcomeric)	CCDS4053.1	chr5_80554980-80554980_G_A	307W>X	Substitution	Nonsense	11%
MM12T	CLASP1	cytoplasmic linker associated protein 1	NM_015282	chr2_122154739-122154739_G_A	1034P>S	Substitution	Nonsynonymous coding	31%
MM12T	CLCN1	chloride channel, voltage-sensitive 1	CCDS5881.1	chr7_143036399-143036399_G_A	485M>I	Substitution	Nonsynonymous coding	12%
MM12T	CLCN7	chloride channel, voltage-sensitive 7	CCDS32361.1	chr16_1505730-1505730_A_G	ISV+2>	Substitution	Splice site donor	30%
MM12T	CLDN1	claudin 1	CCDS3295.1	chr3_190030678-190030678_G_A	124A>V	Substitution	Nonsynonymous coding	21%
MM12T	CLDN1	claudin 1	CCDS3295.1	chr3_190030789-190030789_C_T	87G>D	Substitution	Nonsynonymous coding	10%
MM12T	CLDN11	claudin 11	CCDS3213.1	chr3_170150320-170150320_G_A	134A>T	Substitution	Nonsynonymous coding	31%
MM12T	CLDN12	claudin 12	CCDS5618.1	chr7_90042213-90042213_T_C	75Y>H	Substitution	Nonsynonymous coding	16%
MM12T	CLDN15	claudin 15	CCDS5717.1	chr7_100880816-100880816_C_T	16G>E	Substitution	Nonsynonymous coding	30%
MM12T	CLDN22	claudin 22	CCDS43286.1	chr4_184240941-184240941_G_A	144T>M	Substitution	Nonsynonymous coding	22%
MM12T	CLDN9	claudin 9	CCDS10487.1	chr16_3063605-3063605_G_A	81R>H	Substitution	Nonsynonymous coding	39%
MM12T	CLEC2L	C-type lectin domain family 2, member L	CCDS47724.1	chr7_139225199-139225199_C_T	133A>V	Substitution	Nonsynonymous coding	10%
MM12T	CLEC3A	C-type lectin domain family 3, member A	CCDS10927.1	chr16_78064556-78064556_G_A	138E>K	Substitution	Nonsynonymous coding	30%
MM12T	CLEC4G	C-type lectin domain family 4, member G	CCDS12185.1	chr19_7794325-7794325_G_A	270T>M	Substitution	Nonsynonymous coding	32%
MM12T	CLEC9A	C-type lectin domain family 9, member A	CCDS8611.1	chr12_10217364-10217364_G_T	169G>X	Substitution	Nonsense	11%
MM12T	CLIC6	chloride intracellular channel 6	CCDS13638.1	chr21_36042291-36042291_G_A	202A>T	Substitution	Nonsynonymous coding	13%
MM12T	CLIC6	chloride intracellular channel 6	CCDS13638.1	chr21_36042366-36042366_G_T	227G>W	Substitution	Nonsynonymous coding	45%
MM12T	CLIP2	CAP-GLY domain containing linker protein 2	CCDS5569.1	chr7_73814758-73814758_C_T	980A>V	Substitution	Nonsynonymous coding	29%

MM12T	CLIP3	CAP-GLY domain containing linker protein 3	CCDS12486.1	chr19_36508803-36508803_G_A	425A>V	Substitution	Nonsynonymous coding	30%
MM12T	CLIP3	CAP-GLY domain containing linker protein 3	CCDS12486.1	chr19_36517518-36517518_G_A	178R>C	Substitution	Nonsynonymous coding	30%
MM12T	CLIP4	CAP-GLY domain containing linker protein family, member 4	CCDS1770.1	chr2_29366574-29366574_G_A	ISV-1>	Substitution	Splice site acceptor	20%
MM12T	CLK2	CDC-like kinase 2	CCDS1107.1	chr1_155237856-155237856_G_A	205R>X	Substitution	Nonsense	28%
MM12T	CLK3	CDC-like kinase 3	CCDS45304.1	chr15_74911641-74911641_G_A	183R>Q	Substitution	Nonsynonymous coding	11%
MM12T	CLK4	CDC-like kinase 4	CCDS4437.1	chr5_178039855-178039855_T_C	288K>R	Substitution	Nonsynonymous coding	33%
MM12T	CLMN	calmin (calponin-like, transmembrane)	CCDS9933.1	chr14_95679587-95679587_G_T	193L>M	Substitution	Nonsynonymous coding	32%
MM12T	CLN6	ceroid-lipofuscinosis, neuronal 6, late infantile, variant	CCDS10227.1	chr15_68504170-68504170_C_T	110R>H	Substitution	Nonsynonymous coding	13%
MM12T	CLP1	cleavage and polyadenylation factor I subunit 1	CCDS7964.1	chr11_57427444-57427444_C_A	166L>I	Substitution	Nonsynonymous coding	31%
MM12T	CLPP	ClpP caseinolytic peptidase, ATP-dependent, proteolytic subunit homolog (E. coli)	CCDS12162.1	chr19_6362482-6362482_T_C	99V>A	Substitution	Nonsynonymous coding	27%
MM12T	CLPTM1	cleft lip and palate associated transmembrane protein 1	CCDS12651.1	chr19_45477753-45477753_G_A	123A>T	Substitution	Nonsynonymous coding	12%
MM12T	CLTC	clathrin, heavy chain (Hc)	CCDS32696.1	chr17_57743594-57743594_C_T	592A>V	Substitution	Nonsynonymous coding	25%
MM12T	CLTC	clathrin, heavy chain (Hc)	CCDS32696.1	chr17_57758765-57758765_G_A	1059A>T	Substitution	Nonsynonymous coding	34%
MM12T	CLTCL1	clathrin, heavy chain-like 1	CCDS46662.1	chr22_19226794-19226794_G_A	ISV+4>	Substitution	Splice site donor	19%
MM12T	CLVS1	clavesin 1	CCDS6176.1	chr8_62289226-62289226_C_T	173P>L	Substitution	Nonsynonymous coding	26%
MM12T	CMYA5	cardiomyopathy associated 5	CCDS47238.1	chr5_79029567-79029567_C_T	1660P>L	Substitution	Nonsynonymous coding	28%
MM12T	CNDP2	CNDP dipeptidase 2 (metallopeptidase M20 family)	CCDS12006.1	chr18_72167213-72167213_C_T	2A>V	Substitution	Nonsynonymous coding	30%
MM12T	CNGA2	cyclic nucleotide gated channel alpha 2	CCDS14701.1	chrX_150911771-150911771_C_T	266R>C	Substitution	Nonsynonymous coding	29%
MM12T	CNGB1	cyclic nucleotide gated channel beta 1	CCDS42169.1	chr16_57957192-57957192_G_A	543T>I	Substitution	Nonsynonymous coding	34%
MM12T	CNNM2	cyclin M2	CCDS44474.1	chr10_104679100-104679100_G_A	288R>H	Substitution	Nonsynonymous coding	12%
MM12T	CNNM3	cyclin M3	CCDS2025.1	chr2_97492600-97492600_C_T	467S>F	Substitution	Nonsynonymous coding	29%
MM12T	CNOT11	CCR4-NOT transcription complex, subunit 11	CCDS2050.1	chr2_101883235-101883235_G_A	378A>T	Substitution	Nonsynonymous coding	34%
MM12T	CNOT4	CCR4-NOT transcription complex, subunit 4	CCDS43650.1	chr7_135078937-135078937_G_T	451L>M	Substitution	Nonsynonymous coding	12%
MM12T	CNOT6L	CCR4-NOT transcription complex, subunit 6-like	CCDS47082.1	chr4_78647371-78647371_C_T	469A>T	Substitution	Nonsynonymous coding	35%
MM12T	CNPY1	canopy 1 homolog (zebrafish)	CCDS43684.1	chr7_155301605-155301605_C_A	43R>I	Substitution	Nonsynonymous coding	24%
MM12T	CNPY2	canopy 2 homolog (zebrafish)	CCDS8914.1	chr12_56704996-56704996_G_A	136A>V	Substitution	Nonsynonymous coding	13%
MM12T	CNTFR	ciliary neurotrophic factor receptor	CCDS6558.1	chr9_34552831-34552831_C_T	264A>T	Substitution	Nonsynonymous coding	27%
MM12T	CNTLN	centlein, centrosomal protein	CCDS43789.1	chr9_17135271-17135271_G_	NA	Deletion	Frameshift	34%
MM12T	CNTN1	contactin 1	CCDS8737.1	chr12_41337923-41337923_A_T	545Y>F	Substitution	Nonsynonymous coding	38%
MM12T	CNTN2	contactin 2 (axonal)	CCDS1449.1	chr1_205042817-205042817_G_A	1016R>H	Substitution	Nonsynonymous coding	31%
MM12T	CNTN2	contactin 2 (axonal)	CCDS1449.1	chr1_205027722-205027722_C_T	140R>X	Substitution	Nonsense	12%
MM12T	CNTN3	contactin 3 (plasmacytoma associated)	CCDS33790.1	chr3_74334506-74334506_G_A	885A>V	Substitution	Nonsynonymous coding	10%

MM12T	CNTN6	contactin 6	CCDS2557.1	chr3_1427365-1427365_C_T	863T>I	Substitution	Nonsynonymous coding	10%
MM12T	CNTNAP2	contactin associated protein-like 2	CCDS5889.1	chr7_148112674-148112674_C_T	1321T>I	Substitution	Nonsynonymous coding	13%
MM12T	CNTNAP4	contactin associated protein-like 4	NM_138994	chr16_76592533-76592533_A_G	1221N>D	Substitution	Nonsynonymous coding	26%
MM12T	CNTNAP5	contactin associated protein-like 5	CCDS46401.1	chr2_125281937-125281937_G_A	461R>H	Substitution	Nonsynonymous coding	16%
MM12T	CNTRL	centriolin	CCDS35118.1	chr9_123931925-123931925_A_T	2036E>V	Substitution	Nonsynonymous coding	12%
MM12T	COCH	coagulation factor C homolog, cochlin (Limulus polyphemus)	CCDS9640.1	chr14_31349847-31349847_G_A	179R>H	Substitution	Nonsynonymous coding	11%
MM12T	COG3	component of oligomeric golgi complex 3	CCDS9398.1	chr13_46065596-46065596_G_A	340E>K	Substitution	Nonsynonymous coding	30%
MM12T	COG4	component of oligomeric golgi complex 4	CCDS10892.2	chr16_70515300-70515300_G_A	733R>W	Substitution	Nonsynonymous coding	26%
MM12T	COG7	component of oligomeric golgi complex 7	CCDS10610.1	chr16_23428324-23428324_C_T	419C>Y	Substitution	Nonsynonymous coding	37%
MM12T	COL11A1	collagen, type XI, alpha 1	CCDS779.1	chr1_103355065-103355065_T_	NA	Deletion	Frameshift	28%
MM12T	COL11A2	collagen, type XI, alpha 2	CCDS43452.1	chr6_33154556-33154556_C_T	216A>T	Substitution	Nonsynonymous coding	15%
MM12T	COL15A1	collagen, type XV, alpha 1	CCDS35081.1	chr9_101749637-101749637_C_A	237P>H	Substitution	Nonsynonymous coding	37%
MM12T	COL16A1	collagen, type XVI, alpha 1	CCDS41297.1	chr1_32131521-32131521_G_A	1140R>X	Substitution	Nonsense	10%
MM12T	COL17A1	collagen, type XVII, alpha 1	CCDS7554.1	chr10_105794468-105794468_C_T	1226R>Q	Substitution	Nonsynonymous coding	31%
MM12T	COL17A1	collagen, type XVII, alpha 1	CCDS7554.1	chr10_105809853-105809853_G_T	720L>I	Substitution	Nonsynonymous coding	33%
MM12T	COL18A1	-	NM_130444	chr21_46897667-46897667_G_T	752G>C	Substitution	Nonsynonymous coding	29%
MM12T	COL18A1-AS1	COL18A1 antisense RNA 1	ENST00000330082	chr21_46841314-46841314_G_A	125S>F	Substitution	Nonsynonymous coding	33%
MM12T	COL1A2	collagen, type I, alpha 2	CCDS34682.1	chr7_94029581-94029581_G_C	69G>A	Substitution	Nonsynonymous coding	31%
MM12T	COL20A1	collagen, type XX, alpha 1	CCDS46628.1	chr20_61938964-61938964_G_A	207A>T	Substitution	Nonsynonymous coding	12%
MM12T	COL20A1	collagen, type XX, alpha 1	CCDS46628.1	chr20_61948034-61948034_G_T	885R>I	Substitution	Nonsynonymous coding	39%
MM12T	COL20A1	collagen, type XX, alpha 1	CCDS46628.1	chr20_61956797-61956797_C_T	1100P>L	Substitution	Nonsynonymous coding	28%
MM12T	COL20A1	collagen, type XX, alpha 1	CCDS46628.1	chr20_61959785-61959785_G_A	1239G>D	Substitution	Nonsynonymous coding	12%
MM12T	COL22A1	collagen, type XXII, alpha 1	CCDS6376.1	chr8_139697533-139697533_C_A	962S>I	Substitution	Nonsynonymous coding	26%
MM12T	COL23A1	collagen, type XXIII, alpha 1	CCDS4436.1	chr5_177675209-177675209_C_T	399S>N	Substitution	Nonsynonymous coding	36%
MM12T	COL27A1	collagen, type XXVII, alpha 1	CCDS6802.1	chr9_117054471-117054471_C_A	1522P>H	Substitution	Nonsynonymous coding	14%
MM12T	COL28A1	collagen, type XXVIII, alpha 1	CCDS43553.1	chr7_7410432-7410432_G_A	997P>L	Substitution	Nonsynonymous coding	18%
MM12T	COL28A1	collagen, type XXVIII, alpha 1	CCDS43553.1	chr7_7516743-7516743_C_A	411K>N	Substitution	Nonsynonymous coding	30%
MM12T	COL4A2	collagen, type IV, alpha 2	CCDS41907.1	chr13_111143678-111143678_G_T	1149G>X	Substitution	Nonsense	31%
MM12T	COL4A3	collagen, type IV, alpha 3 (Goodpasture antigen)	CCDS42829.1	chr2_228169778-228169778__A	NA	Insertion	Frameshift	24%
MM12T	COL4A4	collagen, type IV, alpha 4	CCDS42828.1	chr2_227942732-227942732_C_T	622G>D	Substitution	Nonsynonymous coding	28%
MM12T	COL4A5	collagen, type IV, alpha 5	CCDS35366.1	chrX_107938509-107938509_G_T	1612G>W	Substitution	Nonsynonymous coding	25%
MM12T	COL4A6	collagen, type IV, alpha 6	CCDS14541.1	chrX_107422033-107422033_C_T	800R>Q	Substitution	Nonsynonymous coding	28%

MM12T	COL4A6	collagen, type IV, alpha 6	CCDS14541.1	chrX_107431769-107431769_G_A	523A>V	Substitution	Nonsynonymous coding	13%
MM12T	COL5A1	collagen, type V, alpha 1	CCDS6982.1	chr9_137717709-137717709_G_	NA	Deletion	Frameshift	33%
MM12T	COL5A1	collagen, type V, alpha 1	CCDS6982.1	chr9_137620531-137620531_G_A	268G>S	Substitution	Nonsynonymous coding	29%
MM12T	COL5A2	collagen, type V, alpha 2	CCDS33350.1	chr2_189921719-189921719_C_T	791A>T	Substitution	Nonsynonymous coding	20%
MM12T	COL5A3	collagen, type V, alpha 3	CCDS12222.1	chr19_10073514-10073514_G_A	1611T>I	Substitution	Nonsynonymous coding	31%
MM12T	COL5A3	collagen, type V, alpha 3	CCDS12222.1	chr19_10116662-10116662_C_A	ISV-1>	Substitution	Splice site acceptor	15%
MM12T	COL6A1	collagen, type VI, alpha 1	CCDS13727.1	chr21_47421187-47421187_G_A	615D>N	Substitution	Nonsynonymous coding	14%
MM12T	COL6A1	collagen, type VI, alpha 1	CCDS13727.1	chr21_47423917-47423917_C_T	1026A>V	Substitution	Nonsynonymous coding	10%
MM12T	COL6A2	collagen, type VI, alpha 2	CCDS13728.1	chr21_47545474-47545474_G_A	638V>I	Substitution	Nonsynonymous coding	31%
MM12T	COL6A3	collagen, type VI, alpha 3	CCDS33412.1	chr2_238244972-238244972_G_A	2924A>V	Substitution	Nonsynonymous coding	14%
MM12T	COL6A6	collagen, type VI, alpha 6	CCDS46911.1	chr3_130282110-130282110_G_A	88S>N	Substitution	Nonsynonymous coding	33%
MM12T	COL6A6	collagen, type VI, alpha 6	CCDS46911.1	chr3_130284144-130284144_G_A	323R>Q	Substitution	Nonsynonymous coding	29%
MM12T	COL7A1	collagen, type VII, alpha 1	CCDS2773.1	chr3_48628126-48628126_C_A	587S>I	Substitution	Nonsynonymous coding	31%
MM12T	COL7A1	collagen, type VII, alpha 1	CCDS2773.1	chr3_48612975-48612975_G_A	ISV-3>	Substitution	Splice site acceptor	32%
MM12T	COL7A1	collagen, type VII, alpha 1	CCDS2773.1	chr3_48622196-48622196_G_A	1340R>X	Substitution	Nonsense	31%
MM12T	COL9A1	collagen, type IX, alpha 1	CCDS4971.1	chr6_71011713-71011713_G_A	27R>C	Substitution	Nonsynonymous coding	17%
MM12T	COLEC12	collectin sub-family member 12	CCDS32782.1	chr18_347104-347104_G_A	173A>V	Substitution	Nonsynonymous coding	37%
MM12T	COPA	coatomer protein complex, subunit alpha	CCDS41424.1	chr1_160302240-160302240_G_T	165S>Y	Substitution	Nonsynonymous coding	23%
MM12T	COPB1	coatomer protein complex, subunit beta 1	CCDS7815.1	chr11_14507924-14507924_T_C	276T>A	Substitution	Nonsynonymous coding	28%
MM12T	COPG1	coatomer protein complex, subunit gamma 1	CCDS33851.1	chr3_128985870-128985870_C_T	491A>V	Substitution	Nonsynonymous coding	33%
MM12T	COPG2	coatomer protein complex, subunit gamma 2	NM_012133	chr7_130148405-130148405_C_T	279C>Y	Substitution	Nonsynonymous coding	31%
MM12T	COPS7A	COP9 constitutive photomorphogenic homolog subunit 7A (Arabidopsis)	CCDS8558.1	chr12_6838452-6838452_C_T	123R>C	Substitution	Nonsynonymous coding	11%
MM12T	COQ3	coenzyme Q3 homolog, methyltransferase (S. cerevisiae)	CCDS5042.1	chr6_99817700-99817700_A_	NA	Deletion	Splice site acceptor	13%
MM12T	COQ9	coenzyme Q9 homolog (S. cerevisiae)	CCDS32459.1	chr16_57493636-57493636_G_A	ISV+4>	Substitution	Splice site donor	34%
MM12T	CORIN	corin, serine peptidase	CCDS3477.1	chr4_47644064-47644064_T_C	691T>A	Substitution	Nonsynonymous coding	40%
MM12T	CORIN	corin, serine peptidase	CCDS3477.1	chr4_47765474-47765474_C_T	180R>H	Substitution	Nonsynonymous coding	11%
MM12T	CORIN	corin, serine peptidase	CCDS3477.1	chr4_47746603-47746603_A_	NA	Deletion	Splice site acceptor	33%
MM12T	CORO1B	coronin, actin binding protein, 1B	CCDS8164.1	chr11_67208688-67208688_G_A	222R>W	Substitution	Nonsynonymous coding	31%
MM12T	CORO2B	coronin, actin binding protein, 2B	NM_006091	chr15_68937691-68937691_C_A	70L>M	Substitution	Nonsynonymous coding	13%
MM12T	CORO7-PAM16	CORO7-PAM16 readthrough	CCDS10513.1	chr16_4455536-4455536_C_T	174A>T	Substitution	Nonsynonymous coding	28%
MM12T	COTL1	coactosin-like 1 (Dictyostelium)	CCDS10947.1	chr16_84623863-84623863_C_T	56V>I	Substitution	Nonsynonymous coding	11%
MM12T	COX18	cytochrome c oxidase assembly homolog 18 (yeast)	CCDS3554.1	chr4_73935080-73935080_G_A	96A>V	Substitution	Nonsynonymous coding	14%

MM12T	COX18	cytochrome c oxidase assembly homolog 18 (yeast)	CCDS3554.1	chr4_73931081-73931081_G_A	162R>X	Substitution	Nonsense	13%
MM12T	CP	ceruloplasmin (ferroxidase)	CCDS3141.1	chr3_148903178-148903178_T_G	711Q>H	Substitution	Nonsynonymous coding	23%
MM12T	CPA5	carboxypeptidase A5	CCDS5819.1	chr7_130007228-130007228_G_A	285S>N	Substitution	Nonsynonymous coding	53%
MM12T	CPEB1	cytoplasmic polyadenylation element binding protein 1	CCDS45329.1	chr15_83221363-83221363_T_A	356Y>F	Substitution	Nonsynonymous coding	35%
MM12T	CPEB2	cytoplasmic polyadenylation element binding protein 2	CCDS3412.1	chr4_15063798-15063798_C_T	486R>X	Substitution	Nonsense	11%
MM12T	CPEB3	cytoplasmic polyadenylation element binding protein 3	CCDS31246.1	chr10_93812185-93812185_C_A	627K>N	Substitution	Nonsynonymous coding	33%
MM12T	CPLX1	complexin 1	CCDS46995.1	chr4_780462-780462_G_A	78R>C	Substitution	Nonsynonymous coding	15%
MM12T	CPNE1	copine I	CCDS46595.1	chr20_34215288-34215288_G_A	389R>C	Substitution	Nonsynonymous coding	41%
MM12T	CPNE4	copine IV	CCDS3072.1	chr3_131442447-131442447_C_T	68R>H	Substitution	Nonsynonymous coding	26%
MM12T	CPNE7	copine VII	CCDS10980.1	chr16_89657640-89657640_C_T	500A>V	Substitution	Nonsynonymous coding	11%
MM12T	CPO	carboxypeptidase O	CCDS2372.1	chr2_207814396-207814396_C_A	42L>M	Substitution	Nonsynonymous coding	10%
MM12T	CPOX	coproporphyrinogen oxidase	CCDS2932.1	chr3_98307633-98307633_C_T	293A>T	Substitution	Nonsynonymous coding	16%
MM12T	CPS1	carbamoyl-phosphate synthase 1, mitochondrial	CCDS46505.1	chr2_211442196-211442196_G_A	151A>T	Substitution	Nonsynonymous coding	33%
MM12T	CPSF1	cleavage and polyadenylation specific factor 1, 160kDa	CCDS34966.1	chr8_145618997-145618997_C_T	1344G>S	Substitution	Nonsynonymous coding	37%
MM12T	CPSF4	cleavage and polyadenylation specific factor 4, 30kDa	CCDS5664.1	chr7_99047957-99047957_G_T	122K>N	Substitution	Nonsynonymous coding	33%
MM12T	CPT1B	carnitine palmitoyltransferase 1B (muscle)	CCDS14098.1	chr22_51015350-51015350_A_G	132L>P	Substitution	Nonsynonymous coding	11%
MM12T	CPT1C	carnitine palmitoyltransferase 1C	CCDS12779.1	chr19_50208355-50208355_G_A	288R>H	Substitution	Nonsynonymous coding	26%
MM12T	CPXM1	carboxypeptidase X (M14 family), member 1	CCDS13033.1	chr20_2775056-2775056_C_T	662R>H	Substitution	Nonsynonymous coding	19%
MM12T	CPXM2	carboxypeptidase X (M14 family), member 2	CCDS7637.1	chr10_125639816-125639816_C_T	105S>N	Substitution	Nonsynonymous coding	28%
MM12T	CPXM2	carboxypeptidase X (M14 family), member 2	CCDS7637.1	chr10_125651123-125651123_A_G	18V>A	Substitution	Nonsynonymous coding	12%
MM12T	CRADD	CASP2 and RIPK1 domain containing adaptor with death domain	CCDS9048.1	chr12_94072579-94072579_G_A	10R>H	Substitution	Nonsynonymous coding	14%
MM12T	CRAMP1L	Crm, cramped-like (Drosophila)	CCDS10440.2	chr16_1706446-1706446_G_A	563R>H	Substitution	Nonsynonymous coding	38%
MM12T	CRAMP1L	Crm, cramped-like (Drosophila)	CCDS10440.2	chr16_1706786-1706786_G_T	676E>D	Substitution	Nonsynonymous coding	30%
MM12T	CRB1	crumbs homolog 1 (Drosophila)	CCDS1390.1	chr1_197326065-197326065_C_T	365R>C	Substitution	Nonsynonymous coding	26%
MM12T	CRB2	crumbs homolog 2 (Drosophila)	CCDS6852.2	chr9_126135424-126135424_G_A	872A>T	Substitution	Nonsynonymous coding	12%
MM12T	CREBBP	CREB binding protein	CCDS10509.1	chr16_3779340-3779340_G_A	1903P>L	Substitution	Nonsynonymous coding	14%
MM12T	CREBBP	CREB binding protein	CCDS10509.1	chr16_3808953-3808953_G_A	1091R>C	Substitution	Nonsynonymous coding	39%
MM12T	CREBBP	CREB binding protein	CCDS10509.1	chr16_3823801-3823801_G_A	805A>V	Substitution	Nonsynonymous coding	20%
MM12T	CREBBP	CREB binding protein	CCDS10509.1	chr16_3842041-3842041_C_T	424R>Q	Substitution	Nonsynonymous coding	26%
MM12T	CRELD1	cysteine-rich with EGF-like domains 1	CCDS33693.1	chr3_9982533-9982533_G_A	ISV-1>	Substitution	Splice site acceptor	25%
MM12T	CREM	cAMP responsive element modulator	CCDS7180.1	chr10_35467886-35467886_G_T	80E>X	Substitution	Nonsense	25%
MM12T	CRIM1	cysteine rich transmembrane BMP regulator 1 (chordin-like)	CCDS1783.1	chr2_36740819-36740819_G_A	634G>E	Substitution	Nonsynonymous coding	14%

MM12T	CRIP2	cysteine-rich protein 2	CCDS10003.1	chr14_105945155-105945155_C_T	95A>V	Substitution	Nonsynonymous coding	21%
MM12T	CRNKL1	crooked neck pre-mRNA splicing factor-like 1 (Drosophila)	CCDS33446.1	chr20_20019096-20019096_T_C	ISV-2>	Substitution	Splice site acceptor	23%
MM12T	CROCC	ciliary rootlet coiled-coil, rootletin	CCDS30616.1	chr1_17296329-17296329_G_A	1784S>N	Substitution	Nonsynonymous coding	34%
MM12T	CRP	C-reactive protein, pentraxin-related	CCDS30911.1	chr1_159684241-159684241_C_T	12S>N	Substitution	Nonsynonymous coding	29%
MM12T	CRTAC1	cartilage acidic protein 1	CCDS31266.1	chr10_99625444-99625444_C_T	616R>H	Substitution	Nonsynonymous coding	15%
MM12T	CRTAC1	cartilage acidic protein 1	CCDS31266.1	chr10_99677284-99677284_C_T	230A>T	Substitution	Nonsynonymous coding	32%
MM12T	CRTAC1	cartilage acidic protein 1	CCDS31266.1	chr10_99770988-99770988_G_T	44P>H	Substitution	Nonsynonymous coding	11%
MM12T	CRTC2	CREB regulated transcription coactivator 2	CCDS30875.1	chr1_153924538-153924538_C_T	318G>D	Substitution	Nonsynonymous coding	28%
MM12T	CRYBA4	crystallin, beta A4	CCDS13841.1	chr22_27024263-27024263_C_A	104D>E	Substitution	Nonsynonymous coding	24%
MM12T	CRYBA4	crystallin, beta A4	CCDS13841.1	chr22_27026429-27026429_G_T	190S>I	Substitution	Nonsynonymous coding	34%
MM12T	CRYBA4	crystallin, beta A4	CCDS13841.1	chr22_27021497-27021497_C_T	71R>X	Substitution	Nonsense	19%
MM12T	CRYBB1	crystallin, beta B1	CCDS13840.1	chr22_26995521-26995521_C_T	231R>H	Substitution	Nonsynonymous coding	20%
MM12T	CRYBG3	beta-gamma crystallin domain containing 3	CCDS43113.1	chr3_97596589-97596589_C_A	236P>H	Substitution	Nonsynonymous coding	24%
MM12T	CRYGB	crystallin, gamma B	CCDS2380.1	chr2_209010536-209010536_G_T	72L>I	Substitution	Nonsynonymous coding	36%
MM12T	CRYL1	crystallin, lambda 1	CCDS41871.1	chr13_21006252-21006252_G_A	208R>W	Substitution	Nonsynonymous coding	30%
MM12T	CRYM	crystallin, mu	CCDS10597.1	chr16_21281185-21281185_G_T	139L>M	Substitution	Nonsynonymous coding	13%
MM12T	CSF1	colony stimulating factor 1 (macrophage)	CCDS816.1	chr1_110459960-110459960_G_A	91D>N	Substitution	Nonsynonymous coding	15%
MM12T	CSF1	colony stimulating factor 1 (macrophage)	CCDS816.1	chr1_110459981-110459981_C_T	98R>C	Substitution	Nonsynonymous coding	28%
MM12T	CSF1	colony stimulating factor 1 (macrophage)	CCDS816.1	chr1_110466319-110466319_C_T	359P>L	Substitution	Nonsynonymous coding	30%
MM12T	CSF3R	colony stimulating factor 3 receptor (granulocyte)	CCDS414.1	chr1_36939086-36939086_G_A	208A>V	Substitution	Nonsynonymous coding	37%
MM12T	CSMD1	CUB and Sushi multiple domains 1	NM_033225	chr8_2808725-2808725_G_A	3371A>V	Substitution	Nonsynonymous coding	47%
MM12T	CSMD1	CUB and Sushi multiple domains 1	NM_033225	chr8_3265447-3265447_C_T	682G>D	Substitution	Nonsynonymous coding	33%
MM12T	CSMD2	CUB and Sushi multiple domains 2	CCDS380.1	chr1_34034979-34034979_C_T	2711C>Y	Substitution	Nonsynonymous coding	30%
MM12T	CSMD2	CUB and Sushi multiple domains 2	CCDS380.1	chr1_34180240-34180240_G_A	1078T>M	Substitution	Nonsynonymous coding	38%
MM12T	CSMD3	CUB and Sushi multiple domains 3	CCDS6315.1	chr8_113314166-113314166_G_A	2766P>S	Substitution	Nonsynonymous coding	35%
MM12T	CSMD3	CUB and Sushi multiple domains 3	CCDS6315.1	chr8_113402916-113402916_C_A	1971V>L	Substitution	Nonsynonymous coding	21%
MM12T	CSMD3	CUB and Sushi multiple domains 3	CCDS6315.1	chr8_113293532-113293532_G_A	3127R>X	Substitution	Nonsense	23%
MM12T	CSNK1A1P1	casein kinase 1, alpha 1 pseudogene 1	ENST00000430593	chr15_37110596-37110596_G_A	22A>V	Substitution	Nonsynonymous coding	40%
MM12T	CSNK1G2	casein kinase 1, gamma 2	CCDS12077.1	chr19_1980183-1980183_C_T	410S>L	Substitution	Nonsynonymous coding	16%
MM12T	CSNK2A1	casein kinase 2, alpha 1 polypeptide	CCDS13003.1	chr20_468128-468128_G_A	306R>X	Substitution	Nonsense	11%
MM12T	CSNK2A2	casein kinase 2, alpha prime polypeptide	CCDS10794.1	chr16_58200581-58200581_C_T	245R>H	Substitution	Nonsynonymous coding	20%
MM12T	CSPG5	chondroitin sulfate proteoglycan 5 (neuroglycan C)	CCDS2757.1	chr3_47618438-47618438_G_A	360R>C	Substitution	Nonsynonymous coding	11%

MM12T	CSPG5	chondroitin sulfate proteoglycan 5 (neuroglycan C)	CCDS2757.1	chr3_47619097-47619097_G_A	140P>L	Substitution	Nonsynonymous coding	33%
MM12T	CSRP2BP	CSRP2 binding protein	CCDS13133.1	chr20_18163773-18163773_T_A	605D>E	Substitution	Nonsynonymous coding	31%
MM12T	CST4	cystatin S	CCDS13159.1	chr20_23666550-23666550_G_T	136S>Y	Substitution	Nonsynonymous coding	35%
MM12T	CST8	cystatin 8 (cystatin-related epididymal specific)	CCDS13156.1	chr20_23473616-23473616_C_A	85L>I	Substitution	Nonsynonymous coding	22%
MM12T	CST9LP1	cystatin 9-like pseudogene 1	ENST00000376987	chr20_23531135-23531135_T_C	52E>G	Substitution	Nonsynonymous coding	39%
MM12T	CSTL1	cystatin-like 1	CCDS13153.1	chr20_23421076-23421076_G_A	58D>N	Substitution	Nonsynonymous coding	29%
MM12T	CTBP1-AS1	CTBP1 antisense RNA 1	CCDS33935.1	chr4_1244378-1244378_G_A	6C>Y	Substitution	Nonsynonymous coding	21%
MM12T	CTBP2	C-terminal binding protein 2	CCDS7644.1	chr10_126715658-126715658_G_T	224A>D	Substitution	Nonsynonymous coding	13%
MM12T	CTBS	chitobiase, di-N-acetyl-	CCDS698.1	chr1_85020853-85020853_C_A	329W>C	Substitution	Nonsynonymous coding	17%
MM12T	CTCF	CCCTC-binding factor (zinc finger protein)	CCDS10841.1	chr16_67650720-67650720_G_A	342R>H	Substitution	Nonsynonymous coding	19%
MM12T	CTD-2373H9.6	differential display clone 8 isoform 2 [Source:RefSeq peptide;Acc:NP_001230469]	ENST00000322630	chr17_76887261-76887261_G_A	442T>M	Substitution	Nonsynonymous coding	28%
MM12T	CTD-2593A12.4	-	ENST00000423805	chr5_180409426-180409426_C_T	52R>X	Substitution	Nonsense	14%
MM12T	CTD-3064H18.4	-	ENST00000316807	chr19_38039839-38039839_G_A	55P>S	Substitution	Nonsynonymous coding	35%
MM12T	CTD-3088G3.8	Uncharacterized protein [Source:UniProtKB/TrEMBL;Acc:I3L099]	ENST00000344649	chr16_11554895-11554895_G_A	91L>F	Substitution	Nonsynonymous coding	35%
MM12T	CTGF	connective tissue growth factor	CCDS5151.1	chr6_132270489-132270489_G_A	322T>I	Substitution	Nonsynonymous coding	23%
MM12T	CTIF	CBP80/20-dependent translation initiation factor	CCDS45864.1	chr18_46284714-46284714_C_T	337R>W	Substitution	Nonsynonymous coding	12%
MM12T	CTIF	CBP80/20-dependent translation initiation factor	CCDS45864.1	chr18_46287933-46287933_G_A	417R>H	Substitution	Nonsynonymous coding	11%
MM12T	CTNNB1	catenin (cadherin-associated protein), beta 1, 88kDa	CCDS2694.1	chr3_41265563-41265563_G_A	2A>T	Substitution	Nonsynonymous coding	12%
MM12T	CTNNBL1	catenin, beta like 1	CCDS13298.1	chr20_36361299-36361299_C_T	17R>C	Substitution	Nonsynonymous coding	29%
MM12T	CTNNBL1	catenin, beta like 1	CCDS13298.1	chr20_36361369-36361369_G_A	40R>H	Substitution	Nonsynonymous coding	10%
MM12T	CTNND1	catenin (cadherin-associated protein), delta 1	CCDS44604.1	chr11_57559054-57559054_G_A	35R>H	Substitution	Nonsynonymous coding	12%
MM12T	CTNND1	catenin (cadherin-associated protein), delta 1	CCDS44604.1	chr11_57572248-57572248_G_A	573S>N	Substitution	Nonsynonymous coding	12%
MM12T	CTR9	Ctr9, Paf1/RNA polymerase II complex component, homolog (S. cerevisiae)	CCDS7805.1	chr11_10788028-10788028_G_T	559Q>H	Substitution	Nonsynonymous coding	21%
MM12T	CTR9	Ctr9, Paf1/RNA polymerase II complex component, homolog (S. cerevisiae)	CCDS7805.1	chr11_10797171-10797171_C_A	1029A>D	Substitution	Nonsynonymous coding	16%
MM12T	CTRC	chymotrypsin C (caldecrin)	CCDS156.1	chr1_15772117-15772117_G_A	222C>Y	Substitution	Nonsynonymous coding	35%
MM12T	CTSC	cathepsin C	CCDS8282.1	chr11_88033712-88033712_G_T	248P>H	Substitution	Nonsynonymous coding	23%
MM12T	CTSH	cathepsin H	CCDS10308.1	chr15_79220087-79220087_C_T	223G>S	Substitution	Nonsynonymous coding	24%
MM12T	CTSK	cathepsin K	CCDS969.1	chr1_150771704-150771704_G_A	277A>V	Substitution	Nonsynonymous coding	14%
MM12T	CTSW	cathepsin W	CCDS8117.1	chr11_65650098-65650098_G_A	186R>H	Substitution	Nonsynonymous coding	31%
MM12T	CTTN	cortactin	CCDS41680.1	chr11_70279381-70279381_G_A	481A>T	Substitution	Nonsynonymous coding	29%
MM12T	CTU1	cytosolic thiouridylase subunit 1 homolog (S. pombe)	CCDS12824.1	chr19_51602106-51602106_C_T	267V>M	Substitution	Nonsynonymous coding	13%
MM12T	CTU1	cytosolic thiouridylase subunit 1 homolog (S. pombe)	CCDS12824.1	chr19_51607612-51607612_C_T	72R>H	Substitution	Nonsynonymous coding	32%

MM12T	CTU2	cytosolic thiouridylase subunit 2 homolog (S. pombe)	CCDS45545.1	chr16_88778074-88778074_G_A	105R>H	Substitution	Nonsynonymous coding	16%
MM12T	CUBN	cubilin (intrinsic factor-cobalamin receptor)	CCDS7113.1	chr10_16932429-16932429_G_A	2899T>I	Substitution	Nonsynonymous coding	31%
MM12T	CUBN	cubilin (intrinsic factor-cobalamin receptor)	CCDS7113.1	chr10_17024583-17024583_C_A	1532S>I	Substitution	Nonsynonymous coding	11%
MM12T	CUL4A	cullin 4A	CCDS41908.1	chr13_113899501-113899501_A_G	494M>V	Substitution	Nonsynonymous coding	36%
MM12T	CUL5	cullin 5	CCDS31668.1	chr11_107960218-107960218_G_A	ISV-1>	Substitution	Splice site acceptor	21%
MM12T	CUTC	cutC copper transporter homolog (E. coli)	CCDS7483.1	chr10_101515471-101515471_C_A	266A>D	Substitution	Nonsynonymous coding	44%
MM12T	CUX1	cut-like homeobox 1	CCDS5721.1	chr7_101844738-101844738_C_T	721Q>X	Substitution	Nonsense	32%
MM12T	CUX2	cut-like homeobox 2	CCDS41837.1	chr12_111729249-111729249_G_A	110R>H	Substitution	Nonsynonymous coding	15%
MM12T	CUX2	cut-like homeobox 2	CCDS41837.1	chr12_111776191-111776191_C_T	1100R>W	Substitution	Nonsynonymous coding	13%
MM12T	CWF19L2	CWF19-like 2, cell cycle control (S. pombe)	CCDS8336.2	chr11_107197711-107197711_C_A	870E>D	Substitution	Nonsynonymous coding	27%
MM12T	CWH43	cell wall biogenesis 43 C-terminal homolog (S. cerevisiae)	CCDS3486.1	chr4_48996709-48996709_G_A	195W>X	Substitution	Nonsense	12%
MM12T	CXCL14	chemokine (C-X-C motif) ligand 14	CCDS4188.1	chr5_134914191-134914191_G_A	47R>C	Substitution	Nonsynonymous coding	31%
MM12T	CXCR2	chemokine (C-X-C motif) receptor 2	CCDS2408.1	chr2_219000386-219000386_C_T	288R>C	Substitution	Nonsynonymous coding	36%
MM12T	CXCR7	chemokine (C-X-C motif) receptor 7	CCDS2516.1	chr2_237490076-237490076_G_T	323R>M	Substitution	Nonsynonymous coding	26%
MM12T	CXorf21	chromosome X open reading frame 21	CCDS14224.1	chrX_30577593-30577593_A_G	294S>P	Substitution	Nonsynonymous coding	13%
MM12T	CXorf30	chromosome X open reading frame 30	ENST00000378653	chrX_36254939-36254939_C_A	163T>K	Substitution	Nonsynonymous coding	24%
MM12T	CXorf56	chromosome X open reading frame 56	CCDS14579.1	chrX_118694231-118694231_C_A	81R>I	Substitution	Nonsynonymous coding	32%
MM12T	CXorf67	chromosome X open reading frame 67	CCDS14335.1	chrX_51150515-51150515_G_A	216R>H	Substitution	Nonsynonymous coding	35%
MM12T	CXorf67	chromosome X open reading frame 67	CCDS14335.1	chrX_51151196-51151196_C_T	443S>F	Substitution	Nonsynonymous coding	28%
MM12T	CYB5D2	cytochrome b5 domain containing 2	CCDS11044.1	chr17_4053193-4053193_G_A	87A>T	Substitution	Nonsynonymous coding	11%
MM12T	CYB5R1	cytochrome b5 reductase 1	CCDS1431.1	chr1_202935015-202935015_C_A	115K>N	Substitution	Nonsynonymous coding	13%
MM12T	CYFIP2	cytoplasmic FMR1 interacting protein 2	NM_001037332	chr5_156750965-156750965_G_A	570A>T	Substitution	Nonsynonymous coding	38%
MM12T	CYFIP2	cytoplasmic FMR1 interacting protein 2	NM_001037332	chr5_156752587-156752587_G_A	641M>I	Substitution	Nonsynonymous coding	29%
MM12T	CYFIP2	cytoplasmic FMR1 interacting protein 2	NM_001037332	chr5_156816347-156816347_G_A	1120V>I	Substitution	Nonsynonymous coding	14%
MM12T	CYFIP2	cytoplasmic FMR1 interacting protein 2	NM_001037332	chr5_156766244-156766244_C_A	855C>X	Substitution	Nonsense	38%
MM12T	CYGB	cytoglobin	CCDS11746.1	chr17_74527753-74527753_G_A	55S>L	Substitution	Nonsynonymous coding	27%
MM12T	CYHR1	cysteine/histidine-rich 1	CCDS47943.1	chr8_145677871-145677871_C_T	190A>T	Substitution	Nonsynonymous coding	12%
MM12T	CYP19A1	cytochrome P450, family 19, subfamily A, polypeptide 1	CCDS10139.1	chr15_51529174-51529174_T_C	60I>V	Substitution	Nonsynonymous coding	29%
MM12T	CYP1B1	cytochrome P450, family 1, subfamily B, polypeptide 1	CCDS1793.1	chr2_38301976-38301976_C_T	186A>T	Substitution	Nonsynonymous coding	32%
MM12T	CYP1B1	cytochrome P450, family 1, subfamily B, polypeptide 1	CCDS1793.1	chr2_38302093-38302093_C_T	147A>T	Substitution	Nonsynonymous coding	12%
MM12T	CYP1B1	cytochrome P450, family 1, subfamily B, polypeptide 1	CCDS1793.1	chr2_38302338-38302338_G_A	65A>V	Substitution	Nonsynonymous coding	33%
MM12T	CYP24A1	cytochrome P450, family 24, subfamily A, polypeptide 1	CCDS33491.1	chr20_52773755-52773755_G_T	503P>H	Substitution	Nonsynonymous coding	43%

MM12T	CYP26B1	cytochrome P450, family 26, subfamily B, polypeptide 1	CCDS1919.1	chr2_72360330-72360330_C_T	323R>Q	Substitution	Nonsynonymous coding	13%
MM12T	CYP27A1	cytochrome P450, family 27, subfamily A, polypeptide 1	CCDS2423.1	chr2_219677823-219677823_C_T	ISV+4>	Substitution	Splice site donor	55%
MM12T	CYP27B1	cytochrome P450, family 27, subfamily B, polypeptide 1	CCDS8954.1	chr12_58157931-58157931_G_A	389R>C	Substitution	Nonsynonymous coding	27%
MM12T	CYP2A13	cytochrome P450, family 2, subfamily A, polypeptide 13	CCDS12571.1	chr19_41596035-41596035_C_T	143R>C	Substitution	Nonsynonymous coding	16%
MM12T	CYP2A6	cytochrome P450, family 2, subfamily A, polypeptide 6	CCDS12568.1	chr19_41354570-41354570_G_A	148R>C	Substitution	Nonsynonymous coding	11%
MM12T	CYP2C19	cytochrome P450, family 2, subfamily C, polypeptide 19	CCDS7436.1	chr10_96540336-96540336_G_A	188D>N	Substitution	Nonsynonymous coding	36%
MM12T	CYP2C8	cytochrome P450, family 2, subfamily C, polypeptide 8	CCDS7438.1	chr10_96818190-96818190_G_A	241R>X	Substitution	Nonsense	32%
MM12T	CYP2D7P1	cytochrome P450, family 2, subfamily D, polypeptide 7 pseudogene 1	ENST00000436260	chr22_42539579-42539579_G_A	62R>W	Substitution	Nonsynonymous coding	12%
MM12T	CYP2W1	cytochrome P450, family 2, subfamily W, polypeptide 1	ENST00000415893	chr7_1028257-1028257_G_A	236G>E	Substitution	Nonsynonymous coding	33%
MM12T	CYP3A5	cytochrome P450, family 3, subfamily A, polypeptide 5	ENST00000439761	chr7_99269491-99269491_G_A	110T>M	Substitution	Nonsynonymous coding	30%
MM12T	CYP4F12	cytochrome P450, family 4, subfamily F, polypeptide 12	CCDS42517.1	chr19_15794431-15794431_C_T	259A>V	Substitution	Nonsynonymous coding	30%
MM12T	CYP4V2	cytochrome P450, family 4, subfamily V, polypeptide 2	CCDS34119.1	chr4_187130367-187130367_C_T	449A>V	Substitution	Nonsynonymous coding	25%
MM12T	CYP51A1	cytochrome P450, family 51, subfamily A, polypeptide 1	CCDS5623.1	chr7_91747904-91747904_C_T	371R>H	Substitution	Nonsynonymous coding	33%
MM12T	CYP8B1	cytochrome P450, family 8, subfamily B, polypeptide 1	CCDS2707.1	chr3_42917161-42917161_G_A	50R>W	Substitution	Nonsynonymous coding	33%
MM12T	CYSLTR1	cysteinyl leukotriene receptor 1	CCDS14439.1	chrX_77528587-77528587_T_	NA	Deletion	Frameshift	24%
MM12T	CYTH1	cytohesin 1	CCDS42392.2	chr17_76705788-76705788_G_A	17R>C	Substitution	Nonsynonymous coding	27%
MM12T	CYTH4	cytohesin 4	CCDS13946.1	chr22_37708164-37708164_G_A	354R>H	Substitution	Nonsynonymous coding	39%
MM12T	CYTIP	cytohesin 1 interacting protein	CCDS2204.1	chr2_158300424-158300424_C_A	37D>Y	Substitution	Nonsynonymous coding	42%
MM12T	D2HGDH	D-2-hydroxyglutarate dehydrogenase	CCDS33426.1	chr2_242683092-242683092_G_T	182E>D	Substitution	Nonsynonymous coding	22%
MM12T	DAAM2	dishevelled associated activator of morphogenesis 2	NM_015345	chr6_39832234-39832234_C_T	95A>V	Substitution	Nonsynonymous coding	27%
MM12T	DAAM2	dishevelled associated activator of morphogenesis 2	NM_015345	chr6_39869698-39869698_G_A	1030R>H	Substitution	Nonsynonymous coding	12%
MM12T	DACH2	dachshund homolog 2 (Drosophila)	CCDS14455.1	chrX_85403763-85403763_G_A	47V>M	Substitution	Nonsynonymous coding	40%
MM12T	DACT1	dapper, antagonist of beta-catenin, homolog 1 (Xenopus laevis)	CCDS9736.1	chr14_59113155-59113155_C_T	605T>M	Substitution	Nonsynonymous coding	19%
MM12T	DAGLB	diacylglycerol lipase, beta	CCDS5350.1	chr7_6449582-6449582_C_T	635M>I	Substitution	Nonsynonymous coding	32%
MM12T	DAO	D-amino-acid oxidase	CCDS9122.1	chr12_109278787-109278787_G_A	2R>H	Substitution	Nonsynonymous coding	27%
MM12T	DAO	D-amino-acid oxidase	CCDS9122.1	chr12_109278847-109278847_G_A	22R>H	Substitution	Nonsynonymous coding	10%
MM12T	DAPK1	death-associated protein kinase 1	CCDS43842.1	chr9_90296434-90296434_C_A	706S>Y	Substitution	Nonsynonymous coding	33%
MM12T	DAPK3	death-associated protein kinase 3	CCDS12116.1	chr19_3961115-3961115_G_A	225T>M	Substitution	Nonsynonymous coding	34%
MM12T	DAPK3	death-associated protein kinase 3	CCDS12116.1	chr19_3964259-3964259_C_T	179G>D	Substitution	Nonsynonymous coding	36%
MM12T	DAPP1	dual adaptor of phosphotyrosine and 3-phosphoinositides	CCDS47112.1	chr4_100774375-100774375_G_A	120G>D	Substitution	Nonsynonymous coding	12%
MM12T	DAZAP1	DAZ associated protein 1	CCDS12065.1	chr19_1434765-1434765_G_A	360G>R	Substitution	Nonsynonymous coding	39%
MM12T	DAZAP2	DAZ associated protein 2	CCDS44885.1	chr12_51634141-51634141_C_T	10Q>X	Substitution	Nonsense	43%

MM12T	DBC1	deleted in bladder cancer 1	CCDS6822.1	chr9_121976221-121976221_C_A	300A>S	Substitution	Nonsynonymous coding	42%
MM12T	DBF4B	DBF4 homolog B (S. cerevisiae)	CCDS11485.1	chr17_42828026-42828026_C_A	418P>H	Substitution	Nonsynonymous coding	36%
MM12T	DBN1	drebrin 1	CCDS4421.1	chr5_176884688-176884688_G_A	618A>V	Substitution	Nonsynonymous coding	36%
MM12T	DBT	dihydrolipoamide branched chain transacylase E2	CCDS767.1	chr1_100696363-100696363_T_C	120K>R	Substitution	Nonsynonymous coding	18%
MM12T	DBX2	developing brain homeobox 2	CCDS31781.1	chr12_45410358-45410358_G_A	244S>F	Substitution	Nonsynonymous coding	22%
MM12T	DCAF12L1	DDB1 and CUL4 associated factor 12-like 1	CCDS14610.1	chrX_125686039-125686039_G_T	185L>M	Substitution	Nonsynonymous coding	14%
MM12T	DCAF12L2	DDB1 and CUL4 associated factor 12-like 2	CCDS43991.1	chrX_125299307-125299307_C_T	201A>T	Substitution	Nonsynonymous coding	28%
MM12T	DCAF12L2	DDB1 and CUL4 associated factor 12-like 2	CCDS43991.1	chrX_125299870-125299870_G_A	13A>V	Substitution	Nonsynonymous coding	33%
MM12T	DCAF13	DDB1 and CUL4 associated factor 13	CCDS34934.1	chr8_104427474-104427474_G_A	86A>T	Substitution	Nonsynonymous coding	37%
MM12T	DCAKD	dephospho-CoA kinase domain containing	CCDS11493.1	chr17_43102070-43102070_G_A	143R>W	Substitution	Nonsynonymous coding	13%
MM12T	DCC	deleted in colorectal carcinoma	CCDS11952.1	chr18_50278453-50278453_C_T	41R>C	Substitution	Nonsynonymous coding	11%
MM12T	DCDC5	doublecortin domain containing 5	ENST00000406071	chr11_30928254-30928254_G_A	423P>S	Substitution	Nonsynonymous coding	30%
MM12T	DCHS1	dachsous 1 (Drosophila)	CCDS7771.1	chr11_6648170-6648170_G_A	2034R>C	Substitution	Nonsynonymous coding	12%
MM12T	DCHS1	dachsous 1 (Drosophila)	CCDS7771.1	chr11_6648895-6648895_C_A	1792G>V	Substitution	Nonsynonymous coding	27%
MM12T	DCHS1	dachsous 1 (Drosophila)	CCDS7771.1	chr11_6652894-6652894_T_C	1210T>A	Substitution	Nonsynonymous coding	21%
MM12T	DCHS2	dachsous 2 (Drosophila)	CCDS3785.1	chr4_155158119-155158119_G_A	2107A>V	Substitution	Nonsynonymous coding	22%
MM12T	DCLK2	doublecortin-like kinase 2	CCDS34076.1	chr4_151141860-151141860_T_G	354I>M	Substitution	Nonsynonymous coding	34%
MM12T	DCLK3	doublecortin-like kinase 3	CCDS43064.1	chr3_36780096-36780096_A_G	19Y>H	Substitution	Nonsynonymous coding	10%
MM12T	DCX	doublecortin	CCDS14556.1	chrX_110574264-110574264_G_A	353R>X	Substitution	Nonsense	17%
MM12T	DDB1	damage-specific DNA binding protein 1, 127kDa	CCDS31576.1	chr11_61070068-61070068_A_G	1033V>A	Substitution	Nonsynonymous coding	15%
MM12T	DDB1	damage-specific DNA binding protein 1, 127kDa	CCDS31576.1	chr11_61083841-61083841_C_A	442E>D	Substitution	Nonsynonymous coding	32%
MM12T	DDB1	damage-specific DNA binding protein 1, 127kDa	CCDS31576.1	chr11_61094254-61094254_C_T	221A>T	Substitution	Nonsynonymous coding	30%
MM12T	DDR1	discoidin domain receptor tyrosine kinase 1	CCDS47396.1	chr6_30865941-30865941_C_T	776R>W	Substitution	Nonsynonymous coding	22%
MM12T	DDX20	DEAD (Asp-Glu-Ala-Asp) box polypeptide 20	CCDS842.1	chr1_112305407-112305407_A_	NA	Deletion	Splice site donor	21%
MM12T	DDX23	DEAD (Asp-Glu-Ala-Asp) box polypeptide 23	CCDS8770.1	chr12_49231357-49231357_G_A	235Q>X	Substitution	Nonsense	29%
MM12T	DDX24	DEAD (Asp-Glu-Ala-Asp) box polypeptide 24	CCDS9918.1	chr14_94528925-94528925_G_A	254A>V	Substitution	Nonsynonymous coding	17%
MM12T	DDX26B	DEAD/H (Asp-Glu-Ala-Asp/His) box polypeptide 26B	CCDS35401.1	chrX_134715497-134715497_A_	NA	Deletion	Frameshift	36%
MM12T	DDX26B	DEAD/H (Asp-Glu-Ala-Asp/His) box polypeptide 26B	CCDS35401.1	chrX_134706867-134706867_T_C	472L>P	Substitution	Nonsynonymous coding	18%
MM12T	DDX26B	DEAD/H (Asp-Glu-Ala-Asp/His) box polypeptide 26B	CCDS35401.1	chrX_134707863-134707863_G_T	ISV-1>	Substitution	Splice site acceptor	24%
MM12T	DDX39A	DEAD (Asp-Glu-Ala-Asp) box polypeptide 39A	CCDS12308.1	chr19_14523449-14523449_C_T	84V>I	Substitution	Nonsynonymous coding	34%
MM12T	DDX3X	DEAD (Asp-Glu-Ala-Asp) box polypeptide 3, X-linked	CCDS43931.1	chrX_41196698-41196698_G_A	28S>N	Substitution	Nonsynonymous coding	16%
MM12T	DDX4	DEAD (Asp-Glu-Ala-Asp) box polypeptide 4	CCDS3969.1	chr5_55056058-55056058_A_T	53H>L	Substitution	Nonsynonymous coding	11%

MM12T	DDX4	DEAD (Asp-Glu-Ala-Asp) box polypeptide 4	CCDS3969.1	chr5_55076927-55076927_G_A	177R>H	Substitution	Nonsynonymous coding	17%
MM12T	DDX41	DEAD (Asp-Glu-Ala-Asp) box polypeptide 41	CCDS4427.1	chr5_176941760-176941760_G_A	293R>C	Substitution	Nonsynonymous coding	37%
MM12T	DDX42	DEAD (Asp-Glu-Ala-Asp) box polypeptide 42	CCDS32704.1	chr17_61883905-61883905_C_A	246A>D	Substitution	Nonsynonymous coding	12%
MM12T	DDX42	DEAD (Asp-Glu-Ala-Asp) box polypeptide 42	CCDS32704.1	chr17_61890641-61890641_C_T	577R>X	Substitution	Nonsense	11%
MM12T	DDX50	DEAD (Asp-Glu-Ala-Asp) box polypeptide 50	CCDS7283.1	chr10_70673300-70673300_T_A	304V>D	Substitution	Nonsynonymous coding	22%
MM12T	DDX53	DEAD (Asp-Glu-Ala-Asp) box polypeptide 53	CCDS35214.1	chrX_23018203-23018203_G_T	10R>M	Substitution	Nonsynonymous coding	22%
MM12T	DDX53	DEAD (Asp-Glu-Ala-Asp) box polypeptide 53	CCDS35214.1	chrX_23019613-23019613_C_T	480A>V	Substitution	Nonsynonymous coding	25%
MM12T	DDX53	DEAD (Asp-Glu-Ala-Asp) box polypeptide 53	CCDS35214.1	chrX_23019628-23019628_G_A	485S>N	Substitution	Nonsynonymous coding	29%
MM12T	DDX54	DEAD (Asp-Glu-Ala-Asp) box polypeptide 54	CCDS44984.1	chr12_113617007-113617007_C_T	169A>T	Substitution	Nonsynonymous coding	12%
MM12T	DDX56	DEAD (Asp-Glu-Ala-Asp) box helicase 56	ENST00000448192	chr7_44605970-44605970_G_A	153A>V	Substitution	Nonsynonymous coding	12%
MM12T	DDX60	DEAD (Asp-Glu-Ala-Asp) box polypeptide 60	CCDS34097.1	chr4_169183889-169183889_C_T	1030S>N	Substitution	Nonsynonymous coding	15%
MM12T	DDX60	DEAD (Asp-Glu-Ala-Asp) box polypeptide 60	CCDS34097.1	chr4_169227552-169227552_C_A	195R>I	Substitution	Nonsynonymous coding	35%
MM12T	DDX60	DEAD (Asp-Glu-Ala-Asp) box polypeptide 60	CCDS34097.1	chr4_169206026-169206026_C_T	502W>X	Substitution	Nonsense	31%
MM12T	DEAF1	DEAF1 transcription factor	CCDS31327.1	chr11_679706-679706_C_T	370V>I	Substitution	Nonsynonymous coding	12%
MM12T	DEDD2	death effector domain containing 2	CCDS12597.1	chr19_42703613-42703613_G_A	320R>W	Substitution	Nonsynonymous coding	12%
MM12T	DEFB118	defensin, beta 118	CCDS13177.1	chr20_29956497-29956497_C_T	15P>L	Substitution	Nonsynonymous coding	15%
MM12T	DEGS1	delta(4)-desaturase, sphingolipid 1	CCDS1540.1	chr1_224377387-224377387_T_C	64L>S	Substitution	Nonsynonymous coding	25%
MM12T	DEK	DEK oncogene	CCDS34344.1	chr6_18237651-18237651_T_G	287S>R	Substitution	Nonsynonymous coding	12%
MM12T	DENND2C	DENN/MADD domain containing 2C	CCDS875.1	chr1_115167854-115167854_G_A	251S>F	Substitution	Nonsynonymous coding	26%
MM12T	DEPDC1	DEP domain containing 1	CCDS44159.1	chr1_68948460-68948460_C_T	344C>Y	Substitution	Nonsynonymous coding	21%
MM12T	DEPDC1B	DEP domain containing 1B	CCDS3977.1	chr5_59899273-59899273_G_T	396P>H	Substitution	Nonsynonymous coding	37%
MM12T	DERL3	derlin 3	CCDS46672.1	chr22_24181111-24181111_C_T	22A>T	Substitution	Nonsynonymous coding	20%
MM12T	DESI2	desumoylating isopeptidase 2	CCDS1626.1	chr1_244868954-244868954_C_T	150L>F	Substitution	Nonsynonymous coding	28%
MM12T	DET1	de-etiolated homolog 1 (Arabidopsis)	CCDS45343.1	chr15_89074905-89074905_C_T	22R>Q	Substitution	Nonsynonymous coding	31%
MM12T	DFNB31	deafness, autosomal recessive 31	CCDS6806.1	chr9_117185746-117185746_C_T	492D>N	Substitution	Nonsynonymous coding	13%
MM12T	DFNB31	deafness, autosomal recessive 31	CCDS6806.1	chr9_117228647-117228647_C_T	288R>Q	Substitution	Nonsynonymous coding	31%
MM12T	DGAT2	diacylglycerol O-acyltransferase 2	CCDS31642.1	chr11_75507547-75507547_C_T	202P>S	Substitution	Nonsynonymous coding	15%
MM12T	DGAT2L6	diacylglycerol O-acyltransferase 2-like 6	CCDS14397.1	chrX_69419117-69419117_C_T	ISV-4>	Substitution	Splice site acceptor	26%
MM12T	DGCR8	DiGeorge syndrome critical region gene 8	CCDS13773.1	chr22_20073737-20073737_G_A	84R>H	Substitution	Nonsynonymous coding	11%
MM12T	DGKB	diacylglycerol kinase, beta 90kDa	CCDS47547.1	chr7_14722443-14722443_G_A	285R>C	Substitution	Nonsynonymous coding	31%
MM12T	DGKG	diacylglycerol kinase, gamma 90kDa	CCDS3274.1	chr3_186002489-186002489_C_T	185R>H	Substitution	Nonsynonymous coding	29%
MM12T	DGKI	diacylglycerol kinase, iota	ENST00000424189	chr7_137230658-137230658_C_T	733G>D	Substitution	Nonsynonymous coding	18%

MM12T	DGKQ	diacylglycerol kinase, theta 110kDa	CCDS3342.1	chr4_956248-956248_T_C	730D>G	Substitution	Nonsynonymous coding	22%
MM12T	DHCR7	7-dehydrocholesterol reductase	CCDS8200.1	chr11_71150122-71150122_T_C	212T>A	Substitution	Nonsynonymous coding	36%
MM12T	DHODH	dihydroorotate dehydrogenase (quinone)	CCDS42192.1	chr16_72055088-72055088_G_A	195A>T	Substitution	Nonsynonymous coding	32%
MM12T	DHODH	dihydroorotate dehydrogenase (quinone)	CCDS42192.1	chr16_72058069-72058069_G_A	387D>N	Substitution	Nonsynonymous coding	25%
MM12T	DHRS12	dehydrogenase/reductase (SDR family) member 12	CCDS31976.1	chr13_52346002-52346002_C_T	172A>T	Substitution	Nonsynonymous coding	14%
MM12T	DHRS7	dehydrogenase/reductase (SDR family) member 7	CCDS9743.1	chr14_60616897-60616897_A_	NA	Deletion	Frameshift	18%
MM12T	DHRS7C	dehydrogenase/reductase (SDR family) member 7C	NM_001105571	chr17_9676237-9676237_C_T	192A>T	Substitution	Nonsynonymous coding	45%
MM12T	DHTKD1	dehydrogenase E1 and transketolase domain containing 1	CCDS7087.1	chr10_12160908-12160908_C_A	855H>N	Substitution	Nonsynonymous coding	24%
MM12T	DHTKD1	dehydrogenase E1 and transketolase domain containing 1	CCDS7087.1	chr10_12162882-12162882_G_A	919A>T	Substitution	Nonsynonymous coding	47%
MM12T	DHX15	DEAH (Asp-Glu-Ala-His) box polypeptide 15	CCDS33966.1	chr4_24538752-24538752_C_A	611D>Y	Substitution	Nonsynonymous coding	19%
MM12T	DHX16	DEAH (Asp-Glu-Ala-His) box polypeptide 16	CCDS4685.1	chr6_30627338-30627338_G_A	640R>W	Substitution	Nonsynonymous coding	47%
MM12T	DHX29	DEAH (Asp-Glu-Ala-His) box polypeptide 29	CCDS34158.1	chr5_54603295-54603295_G_A	36A>V	Substitution	Nonsynonymous coding	28%
MM12T	DHX30	DEAH (Asp-Glu-Ala-His) box polypeptide 30	CCDS2759.1	chr3_47859514-47859514_C_T	11R>W	Substitution	Nonsynonymous coding	26%
MM12T	DHX30	DEAH (Asp-Glu-Ala-His) box polypeptide 30	CCDS2759.1	chr3_47890503-47890503_C_T	955R>C	Substitution	Nonsynonymous coding	32%
MM12T	DHX34	DEAH (Asp-Glu-Ala-His) box polypeptide 34	CCDS12700.1	chr19_47856393-47856393_C_T	36R>C	Substitution	Nonsynonymous coding	29%
MM12T	DHX36	DEAH (Asp-Glu-Ala-His) box polypeptide 36	CCDS3171.1	chr3_154006687-154006687_C_A	667V>L	Substitution	Nonsynonymous coding	38%
MM12T	DHX38	DEAH (Asp-Glu-Ala-His) box polypeptide 38	CCDS10907.1	chr16_72131671-72131671_C_T	203R>X	Substitution	Nonsense	10%
MM12T	DHX40	DEAH (Asp-Glu-Ala-His) box polypeptide 40	CCDS11617.1	chr17_57663597-57663600_AAAA_	NA	Deletion	Splice site donor	14%
MM12T	DHX40	DEAH (Asp-Glu-Ala-His) box polypeptide 40	CCDS11617.1	chr17_57663597-57663599_AAA_	NA	Deletion	Splice site donor	31%
MM12T	DHX57	DEAH (Asp-Glu-Ala-Asp/His) box polypeptide 57	CCDS1800.1	chr2_39050139-39050139_G_A	1096P>L	Substitution	Nonsynonymous coding	11%
MM12T	DHX57	DEAH (Asp-Glu-Ala-Asp/His) box polypeptide 57	CCDS1800.1	chr2_39065023-39065023_A_G	831I>T	Substitution	Nonsynonymous coding	24%
MM12T	DHX8	DEAH (Asp-Glu-Ala-His) box polypeptide 8	CCDS11464.1	chr17_41571096-41571096_C_T	352R>X	Substitution	Nonsense	13%
MM12T	DIAPH3	diaphanous homolog 3 (Drosophila)	CCDS41898.1	chr13_60348873-60348873_G_A	1083P>L	Substitution	Nonsynonymous coding	30%
MM12T	DIAPH3	diaphanous homolog 3 (Drosophila)	CCDS41898.1	chr13_60566229-60566229_C_T	406R>H	Substitution	Nonsynonymous coding	28%
MM12T	DICER1	dicer 1, ribonuclease type III	CCDS9931.1	chr14_95570243-95570243_G_A	1164L>F	Substitution	Nonsynonymous coding	30%
MM12T	DICER1	dicer 1, ribonuclease type III	CCDS9931.1	chr14_95572409-95572409_G_A	986P>S	Substitution	Nonsynonymous coding	10%
MM12T	DIP2A	DIP2 disco-interacting protein 2 homolog A (Drosophila)	CCDS46655.1	chr21_47918542-47918542_C_T	151R>X	Substitution	Nonsense	29%
MM12T	DIP2B	DIP2 disco-interacting protein 2 homolog B (Drosophila)	CCDS31799.1	chr12_51122442-51122442_G_A	1208A>T	Substitution	Nonsynonymous coding	26%
MM12T	DIP2C	DIP2 disco-interacting protein 2 homolog C (Drosophila)	CCDS7054.1	chr10_412344-412344_C_T	713M>I	Substitution	Nonsynonymous coding	32%
MM12T	DIP2C	DIP2 disco-interacting protein 2 homolog C (Drosophila)	CCDS7054.1	chr10_518493-518493_G_A	ISV-4>	Substitution	Splice site acceptor	12%
MM12T	DIRAS1	DIRAS family, GTP-binding RAS-like 1	CCDS12092.1	chr19_2717474-2717474_C_T	111V>M	Substitution	Nonsynonymous coding	36%
MM12T	DISC1	disrupted in schizophrenia 1	CCDS31055.1	chr1_231906668-231906668_C_T	496Q>X	Substitution	Nonsense	25%

MM12T	DISP2	dispatched homolog 2 (Drosophila)	CCDS10056.1	chr15_40657910-40657910_G_A	310R>H	Substitution	Nonsynonymous coding	13%
MM12T	DIXDC1	DIX domain containing 1	ENST00000440460	chr11_111866266-111866266_C_T	555T>M	Substitution	Nonsynonymous coding	29%
MM12T	DKFZP686J19100	Polycystic kidney disease protein 1-like 2 [Source:UniProtKB/Swiss-Prot;Acc:Q7Z442]	NM_052892	chr16_81197214-81197214_C_A	1156E>D	Substitution	Nonsynonymous coding	27%
MM12T	DLEC1	deleted in lung and esophageal cancer 1	NM_007337	chr3_38163974-38163974_C_A	1739L>I	Substitution	Nonsynonymous coding	27%
MM12T	DLEU7	deleted in lymphocytic leukemia, 7	NM_198989	chr13_51417662-51417662_G_A	41R>W	Substitution	Nonsynonymous coding	27%
MM12T	DLG5	discs, large homolog 5 (Drosophila)	CCDS7353.2	chr10_79571809-79571809_C_T	1399G>S	Substitution	Nonsynonymous coding	26%
MM12T	DLST	dihydrolipoamide S-succinyltransferase (E2 component of 2-oxo-glutarate complex)	CCDS9833.1	chr14_75360125-75360125_C_T	224R>W	Substitution	Nonsynonymous coding	29%
MM12T	DLX3	distal-less homeobox 3	CCDS11556.1	chr17_48069105-48069105_C_T	214A>T	Substitution	Nonsynonymous coding	19%
MM12T	DMBT1	deleted in malignant brain tumors 1	CCDS44490.1	chr10_124396826-124396826_C_T	2185Q>X	Substitution	Nonsense	15%
MM12T	DMD	dystrophin	ENST00000378682	chrX_31132811-31132813_GGT_	NA	Deletion	In-frame deletion	25%
MM12T	DMD	dystrophin	ENST00000378682	chrX_31132810-31132813_GGGT_	NA	Deletion	Frameshift	29%
MM12T	DMD	dystrophin	CCDS14233.1	chrX_31191720-31191720_G_A	3422P>S	Substitution	Nonsynonymous coding	30%
MM12T	DMD	dystrophin	CCDS14233.1	chrX_32404497-32404497_G_A	1535T>M	Substitution	Nonsynonymous coding	31%
MM12T	DMGDH	dimethylglycine dehydrogenase	CCDS4044.1	chr5_78340250-78340250_G_T	291L>I	Substitution	Nonsynonymous coding	26%
MM12T	DMPK	dystrophia myotonica-protein kinase	CCDS46119.1	chr19_46274309-46274309_G_A	563P>S	Substitution	Nonsynonymous coding	23%
MM12T	DMRTA2	DMRT-like family A2	CCDS44141.1	chr1_50885295-50885295_G_A	224S>L	Substitution	Nonsynonymous coding	10%
MM12T	DMTF1	cyclin D binding myb-like transcription factor 1	CCDS5601.1	chr7_86824066-86824066_G_A	699V>I	Substitution	Nonsynonymous coding	32%
MM12T	DMXL1	Dmx-like 1	CCDS4125.1	chr5_118513772-118513772_T_A	2323L>H	Substitution	Nonsynonymous coding	40%
MM12T	DMXL2	Dmx-like 2	CCDS10141.1	chr15_51742457-51742457_C_T	2924V>I	Substitution	Nonsynonymous coding	29%
MM12T	DMXL2	Dmx-like 2	CCDS10141.1	chr15_51791855-51791855_G_A	1189A>V	Substitution	Nonsynonymous coding	26%
MM12T	DMXL2	Dmx-like 2	CCDS10141.1	chr15_51792203-51792203_G_A	1073A>V	Substitution	Nonsynonymous coding	16%
MM12T	DMXL2	Dmx-like 2	CCDS10141.1	chr15_51829921-51829921_C_A	461G>X	Substitution	Nonsense	32%
MM12T	DNA2	DNA replication helicase 2 homolog (yeast)	CCDS44415.1	chr10_70204814-70204814_C_T	448A>T	Substitution	Nonsynonymous coding	36%
MM12T	DNAAF3	dynein, axonemal, assembly factor 3	CCDS12918.2	chr19_55677965-55677965_A_G	18C>R	Substitution	Nonsynonymous coding	30%
MM12T	DNAH1	dynein, axonemal, heavy chain 1	CCDS46842.1	chr3_52404002-52404002_G_T	2035E>D	Substitution	Nonsynonymous coding	39%
MM12T	DNAH1	dynein, axonemal, heavy chain 1	CCDS46842.1	chr3_52425019-52425019_G_A	3230W>X	Substitution	Nonsense	21%
MM12T	DNAH1	dynein, axonemal, heavy chain 1	CCDS46842.1	chr3_52434358-52434358_G_T	4232G>X	Substitution	Nonsense	14%
MM12T	DNAH10	dynein, axonemal, heavy chain 10	CCDS9255.2	chr12_124285848-124285848_G_A	710R>H	Substitution	Nonsynonymous coding	11%
MM12T	DNAH10	dynein, axonemal, heavy chain 10	CCDS9255.2	chr12_124414986-124414986_G_A	4099R>H	Substitution	Nonsynonymous coding	38%
MM12T	DNAH11	dynein, axonemal, heavy chain 11	ENST00000328843	chr7_21639510-21639510_C_T	925H>Y	Substitution	Nonsynonymous coding	30%
MM12T	DNAH11	dynein, axonemal, heavy chain 11	ENST00000328843	chr7_21646120-21646120_C_T	1242A>V	Substitution	Nonsynonymous coding	19%
MM12T	DNAH11	dynein, axonemal, heavy chain 11	ENST00000328843	chr7_21788212-21788212_G_A	2849R>Q	Substitution	Nonsynonymous coding	29%

MM12T	DNAH11	dynein, axonemal, heavy chain 11	ENST00000328843	chr7_21904268-21904268_C_A	3837A>D	Substitution	Nonsynonymous coding	12%
MM12T	DNAH11	dynein, axonemal, heavy chain 11	ENST00000328843	chr7_21938973-21938973_C_T	4364R>X	Substitution	Nonsense	13%
MM12T	DNAH17	dynein, axonemal, heavy chain 17	NM_173628	chr17_76475573-76475573_C_T	2632A>T	Substitution	Nonsynonymous coding	15%
MM12T	DNAH17	dynein, axonemal, heavy chain 17	NM_173628	chr17_76487605-76487605_C_T	2197G>S	Substitution	Nonsynonymous coding	29%
MM12T	DNAH17	dynein, axonemal, heavy chain 17	NM_173628	chr17_76501520-76501520_C_T	1604R>H	Substitution	Nonsynonymous coding	16%
MM12T	DNAH17	dynein, axonemal, heavy chain 17	NM_173628	chr17_76521225-76521225_C_T	1244A>T	Substitution	Nonsynonymous coding	12%
MM12T	DNAH2	dynein, axonemal, heavy chain 2	CCDS32551.1	chr17_7643075-7643075_G_A	399A>T	Substitution	Nonsynonymous coding	11%
MM12T	DNAH2	dynein, axonemal, heavy chain 2	CCDS32551.1	chr17_7681423-7681423_C_T	1759T>I	Substitution	Nonsynonymous coding	31%
MM12T	DNAH2	dynein, axonemal, heavy chain 2	CCDS32551.1	chr17_7727166-7727166_G_A	3782A>T	Substitution	Nonsynonymous coding	27%
MM12T	DNAH2	dynein, axonemal, heavy chain 2	CCDS32551.1	chr17_7734465-7734465_T_C	4098Y>H	Substitution	Nonsynonymous coding	19%
MM12T	DNAH3	dynein, axonemal, heavy chain 3	CCDS10594.1	chr16_21011834-21011834_C_A	2045E>X	Substitution	Nonsense	33%
MM12T	DNAH5	dynein, axonemal, heavy chain 5	CCDS3882.1	chr5_13762979-13762979_A_G	3378V>A	Substitution	Nonsynonymous coding	42%
MM12T	DNAH5	dynein, axonemal, heavy chain 5	CCDS3882.1	chr5_13901422-13901422_T_C	664N>S	Substitution	Nonsynonymous coding	36%
MM12T	DNAH5	dynein, axonemal, heavy chain 5	CCDS3882.1	chr5_13919404-13919404_C_T	286E>K	Substitution	Nonsynonymous coding	18%
MM12T	DNAH6	dynein, axonemal, heavy chain 6	CCDS46348.1	chr2_84777072-84777072_C_T	459A>V	Substitution	Nonsynonymous coding	24%
MM12T	DNAH6	dynein, axonemal, heavy chain 6	CCDS46348.1	chr2_84852135-84852135_C_T	1488A>V	Substitution	Nonsynonymous coding	20%
MM12T	DNAH6	dynein, axonemal, heavy chain 6	CCDS46348.1	chr2_84934679-84934679_C_T	2963R>C	Substitution	Nonsynonymous coding	18%
MM12T	DNAH6	dynein, axonemal, heavy chain 6	CCDS46348.1	chr2_84913685-84913685_A_G	ISV+4>	Substitution	Splice site donor	27%
MM12T	DNAH6	dynein, axonemal, heavy chain 6	CCDS46348.1	chr2_84804521-84804521_C_T	689R>X	Substitution	Nonsense	15%
MM12T	DNAH7	dynein, axonemal, heavy chain 7	CCDS42794.1	chr2_196837170-196837170_C_A	618E>D	Substitution	Nonsynonymous coding	36%
MM12T	DNAH8	dynein, axonemal, heavy chain 8	CCDS4838.1	chr6_38818110-38818110_C_G	1544C>W	Substitution	Nonsynonymous coding	18%
MM12T	DNAH8	dynein, axonemal, heavy chain 8	CCDS4838.1	chr6_38885870-38885870_C_G	3276A>G	Substitution	Nonsynonymous coding	16%
MM12T	DNAH8	dynein, axonemal, heavy chain 8	CCDS4838.1	chr6_38749029-38749029_G_T	ISV-1>	Substitution	Splice site acceptor	23%
MM12T	DNAH8	dynein, axonemal, heavy chain 8	CCDS4838.1	chr6_38723858-38723858_A_G	ISV+3>	Substitution	Splice site donor	16%
MM12T	DNAH9	dynein, axonemal, heavy chain 9	CCDS11160.1	chr17_11583197-11583197_G_T	1159Q>H	Substitution	Nonsynonymous coding	18%
MM12T	DNAH9	dynein, axonemal, heavy chain 9	CCDS11160.1	chr17_11795157-11795157_C_T	3726R>W	Substitution	Nonsynonymous coding	16%
MM12T	DNAJB12	DnaJ (Hsp40) homolog, subfamily B, member 12	CCDS7316.2	chr10_74100910-74100910_G_A	193A>V	Substitution	Nonsynonymous coding	11%
MM12T	DNAJB13	DnaJ (Hsp40) homolog, subfamily B, member 13	CCDS8227.1	chr11_73677186-73677186_C_A	166L>M	Substitution	Nonsynonymous coding	14%
MM12T	DNAJB8	DnaJ (Hsp40) homolog, subfamily B, member 8	CCDS3048.1	chr3_128181482-128181482_C_T	203G>R	Substitution	Nonsynonymous coding	29%
MM12T	DNAJC16	DnaJ (Hsp40) homolog, subfamily C, member 16	CCDS30606.1	chr1_15890420-15890420_C_T	ISV-4>	Substitution	Splice site acceptor	15%
MM12T	DNAJC2	DnaJ (Hsp40) homolog, subfamily C, member 2	CCDS43628.1	chr7_102964064-102964064_C_G	234E>Q	Substitution	Nonsynonymous coding	20%
MM12T	DNAJC21	DnaJ (Hsp40) homolog, subfamily C, member 21	CCDS3907.2	chr5_34937654-34937654_G_A	221R>Q	Substitution	Nonsynonymous coding	16%

MM12T	DNAJC5B	DnaJ (Hsp40) homolog, subfamily C, member 5 beta	CCDS6183.1	chr8_66988980-66988980_G_A	69A>T	Substitution	Nonsynonymous coding	15%
MM12T	DNASE1	deoxyribonuclease I	CCDS10507.1	chr16_3707065-3707065_G_A	168A>T	Substitution	Nonsynonymous coding	16%
MM12T	DNASE1L2	deoxyribonuclease I-like 2	CCDS42105.1	chr16_2287373-2287373_C_T	ISV-4>	Substitution	Splice site acceptor	27%
MM12T	DNM1	dynamin 1	CCDS6895.1	chr9_130981471-130981471_G_A	177A>T	Substitution	Nonsynonymous coding	18%
MM12T	DNM1L	dynamin 1-like	CCDS8729.1	chr12_32854424-32854424_C_T	60R>W	Substitution	Nonsynonymous coding	26%
MM12T	DNM2	dynamin 2	CCDS45968.1	chr19_10939878-10939878_G_T	742S>I	Substitution	Nonsynonymous coding	15%
MM12T	DNMT3A	DNA (cytosine-5-)-methyltransferase 3 alpha	CCDS33157.1	chr2_25458591-25458591_C_T	861C>Y	Substitution	Nonsynonymous coding	23%
MM12T	DNMT3A	DNA (cytosine-5-)-methyltransferase 3 alpha	CCDS33157.1	chr2_25469629-25469629_G_A	380A>V	Substitution	Nonsynonymous coding	12%
MM12T	DNMT3B	DNA (cytosine-5-)-methyltransferase 3 beta	CCDS13205.1	chr20_31379479-31379479_G_A	296V>I	Substitution	Nonsynonymous coding	31%
MM12T	DNMT3B	DNA (cytosine-5-)-methyltransferase 3 beta	CCDS13205.1	chr20_31383238-31383238_G_A	384A>T	Substitution	Nonsynonymous coding	12%
MM12T	DNMT3B	DNA (cytosine-5-)-methyltransferase 3 beta	CCDS13205.1	chr20_31395599-31395599_G_A	818V>M	Substitution	Nonsynonymous coding	29%
MM12T	DNPEP	aspartyl aminopeptidase	CCDS42823.1	chr2_220246865-220246865_G_A	ISV-4>	Substitution	Splice site acceptor	14%
MM12T	DNPH1	2'-deoxynucleoside 5'-phosphate N-hydrolase 1	CCDS4891.1	chr6_43193610-43193610_G_A	129A>V	Substitution	Nonsynonymous coding	40%
MM12T	DOCK1	dedicator of cytokinesis 1	NM_001380	chr10_128796461-128796461_G_T	239D>Y	Substitution	Nonsynonymous coding	28%
MM12T	DOCK1	dedicator of cytokinesis 1	NM_001380	chr10_129224184-129224184_C_T	1587T>M	Substitution	Nonsynonymous coding	22%
MM12T	DOCK1	dedicator of cytokinesis 1	NM_001380	chr10_129231622-129231622_C_T	1643R>C	Substitution	Nonsynonymous coding	29%
MM12T	DOCK10	dedicator of cytokinesis 10	CCDS46528.1	chr2_225907069-225907069_C_A	8R>M	Substitution	Nonsynonymous coding	27%
MM12T	DOCK11	dedicator of cytokinesis 11	CCDS35373.1	chrX_117815681-117815681_G_A	1963A>T	Substitution	Nonsynonymous coding	28%
MM12T	DOCK2	dedicator of cytokinesis 2	CCDS4371.1	chr5_169507194-169507194_G_A	1732D>N	Substitution	Nonsynonymous coding	28%
MM12T	DOCK3	dedicator of cytokinesis 3	CCDS46835.1	chr3_51400085-51400085_C_A	1758S>Y	Substitution	Nonsynonymous coding	36%
MM12T	DOCK4	dedicator of cytokinesis 4	CCDS47688.1	chr7_111405269-111405269_C_A	1300A>S	Substitution	Nonsynonymous coding	24%
MM12T	DOCK4	dedicator of cytokinesis 4	CCDS47688.1	chr7_111462476-111462476_A_C	958L>V	Substitution	Nonsynonymous coding	23%
MM12T	DOCK6	dedicator of cytokinesis 6	CCDS45975.1	chr19_11352859-11352859_G_A	502P>L	Substitution	Nonsynonymous coding	32%
MM12T	DOCK8	dedicator of cytokinesis 8	CCDS6440.1	chr9_340223-340223_G_A	459M>I	Substitution	Nonsynonymous coding	24%
MM12T	DOCK9	dedicator of cytokinesis 9	CCDS45062.1	chr13_99476756-99476756_C_T	1676V>M	Substitution	Nonsynonymous coding	33%
MM12T	DOCK9	dedicator of cytokinesis 9	CCDS45062.1	chr13_99483762-99483762_C_T	1469G>E	Substitution	Nonsynonymous coding	11%
MM12T	DOHH	deoxyhypusine hydroxylase/monooxygenase	CCDS12108.1	chr19_3491802-3491802_G_T	199H>Q	Substitution	Nonsynonymous coding	11%
MM12T	DOHH	deoxyhypusine hydroxylase/monooxygenase	CCDS12108.1	chr19_3496809-3496809_C_T	2V>M	Substitution	Nonsynonymous coding	30%
MM12T	DOK7	docking protein 7	ENST00000389653	chr4_3502427-3502427_G_A	568A>T	Substitution	Nonsynonymous coding	13%
MM12T	DOLK	dolichol kinase	CCDS6915.1	chr9_131708138-131708138_G_A	482A>V	Substitution	Nonsynonymous coding	38%
MM12T	DOPEY2	dopey family member 2	CCDS13643.1	chr21_37603097-37603097_C_T	672A>V	Substitution	Nonsynonymous coding	31%
MM12T	DOPEY2	dopey family member 2	CCDS13643.1	chr21_37623584-37623584_T_C	ISV+2>	Substitution	Splice site donor	45%

MM12T	DOT1L	DOT1-like, histone H3 methyltransferase (S. cerevisiae)	CCDS42460.1	chr19_2210451-2210451_C_T	353A>V	Substitution	Nonsynonymous coding	26%
MM12T	DOT1L	DOT1-like, histone H3 methyltransferase (S. cerevisiae)	CCDS42460.1	chr19_2226905-2226905_G_A	1462R>Q	Substitution	Nonsynonymous coding	35%
MM12T	DPF2	D4, zinc and double PHD fingers family 2	CCDS8100.1	chr11_65113714-65113714_A_G	ISV-4>	Substitution	Splice site acceptor	42%
MM12T	DPH2	DPH2 homolog (S. cerevisiae)	CCDS504.1	chr1_44436744-44436744_G_A	123V>M	Substitution	Nonsynonymous coding	25%
MM12T	DPP10	dipeptidyl-peptidase 10 (non-functional)	CCDS46400.1	chr2_116101420-116101420_G_A	68R>K	Substitution	Nonsynonymous coding	30%
MM12T	DPP3	dipeptidyl-peptidase 3	CCDS8141.1	chr11_66254795-66254795_G_T	185Q>H	Substitution	Nonsynonymous coding	34%
MM12T	DPP6	dipeptidyl-peptidase 6	ENST00000406326	chr7_154379585-154379585_C_T	285P>S	Substitution	Nonsynonymous coding	20%
MM12T	DPP8	dipeptidyl-peptidase 8	CCDS10207.1	chr15_65744385-65744385_G_A	792T>M	Substitution	Nonsynonymous coding	32%
MM12T	DPP9	dipeptidyl-peptidase 9	CCDS45928.1	chr19_4704017-4704017_C_T	217R>Q	Substitution	Nonsynonymous coding	16%
MM12T	DPRX	divergent-paired related homeobox	CCDS33103.1	chr19_54140183-54140183_G_A	173A>T	Substitution	Nonsynonymous coding	37%
MM12T	DPT	dermatopontin	CCDS1275.1	chr1_168698178-168698178_G_A	79P>S	Substitution	Nonsynonymous coding	26%
MM12T	DPYD	dihydropyrimidine dehydrogenase	CCDS30777.1	chr1_97544542-97544542_G_A	1023P>L	Substitution	Nonsynonymous coding	26%
MM12T	DPYD	dihydropyrimidine dehydrogenase	CCDS30777.1	chr1_97658735-97658735_G_A	838L>F	Substitution	Nonsynonymous coding	48%
MM12T	DPYS	dihydropyrimidinase	CCDS6302.1	chr8_105441903-105441903_C_T	274A>T	Substitution	Nonsynonymous coding	14%
MM12T	DPYSL3	dihydropyrimidinase-like 3	CCDS43381.1	chr5_146773647-146773647_C_T	555R>H	Substitution	Nonsynonymous coding	29%
MM12T	DQX1	DEAQ box RNA-dependent ATPase 1	CCDS1949.2	chr2_74752178-74752178_C_A	130S>I	Substitution	Nonsynonymous coding	11%
MM12T	DRAXIN	dorsal inhibitory axon guidance protein	CCDS135.1	chr1_11766394-11766394_G_A	27A>T	Substitution	Nonsynonymous coding	30%
MM12T	DRAXIN	dorsal inhibitory axon guidance protein	CCDS135.1	chr1_11766448-11766448_C_T	45P>S	Substitution	Nonsynonymous coding	35%
MM12T	DROSHA	drosha, ribonuclease type III	CCDS47195.1	chr5_31409179-31409179_C_A	1280D>Y	Substitution	Nonsynonymous coding	14%
MM12T	DSC1	desmocollin 1	CCDS11894.1	chr18_28739496-28739496__A	NA	Insertion	Splice site acceptor	14%
MM12T	DSC3	desmocollin 3	CCDS32810.1	chr18_28588431-28588431_G_A	442P>S	Substitution	Nonsynonymous coding	12%
MM12T	DSCAM	Down syndrome cell adhesion molecule	CCDS42929.1	chr21_41414441-41414441_G_A	1848T>M	Substitution	Nonsynonymous coding	30%
MM12T	DSCAM	Down syndrome cell adhesion molecule	CCDS42929.1	chr21_41561090-41561090_G_A	811A>V	Substitution	Nonsynonymous coding	27%
MM12T	DSCAML1	Down syndrome cell adhesion molecule like 1	CCDS8384.1	chr11_117389187-117389187_C_T	562V>I	Substitution	Nonsynonymous coding	12%
MM12T	DSCAML1	Down syndrome cell adhesion molecule like 1	CCDS8384.1	chr11_117667953-117667953_T_A	8R>W	Substitution	Nonsynonymous coding	28%
MM12T	DSE	dermatan sulfate epimerase	CCDS5107.1	chr6_116754682-116754682_C_T	363R>C	Substitution	Nonsynonymous coding	12%
MM12T	DSPP	dentin sialophosphoprotein	CCDS43248.1	chr4_88534319-88534319_G_T	327E>D	Substitution	Nonsynonymous coding	42%
MM12T	DTX3L	deltex 3-like (Drosophila)	CCDS3015.1	chr3_122288300-122288300_T_C	455L>P	Substitution	Nonsynonymous coding	27%
MM12T	DUOX2	dual oxidase 2	CCDS10117.1	chr15_45405539-45405539_C_T	24G>D	Substitution	Nonsynonymous coding	47%
MM12T	DUS4L	dihydrouridine synthase 4-like (S. cerevisiae)	CCDS5745.1	chr7_107215721-107215721_G_A	149E>K	Substitution	Nonsynonymous coding	10%
MM12T	DUSP11	dual specificity phosphatase 11 (RNA/RNP complex 1-interacting)	CCDS1928.2	chr2_74005465-74005465_C_A	94G>V	Substitution	Nonsynonymous coding	16%
MM12T	DUSP12	dual specificity phosphatase 12	CCDS1234.1	chr1_161719661-161719661_G_A	24A>T	Substitution	Nonsynonymous coding	39%

MM12T	DUSP15	dual specificity phosphatase 15	CCDS13193.1	chr20_30450475-30450475_G_A	112R>W	Substitution	Nonsynonymous coding	12%
MM12T	DUSP22	dual specificity phosphatase 22	CCDS4468.1	chr6_348790-348790_G_A	153E>K	Substitution	Nonsynonymous coding	15%
MM12T	DUSP22	dual specificity phosphatase 22	CCDS4468.1	chr6_348099-348099_G_A	ISV-4>	Substitution	Splice site acceptor	13%
MM12T	DUSP22	dual specificity phosphatase 22	CCDS4468.1	chr6_345932-345932_C_T	ISV+4>	Substitution	Splice site donor	16%
MM12T	DUSP27	dual specificity phosphatase 27 (putative)	CCDS30932.1	chr1_167082952-167082952_C_T	39S>L	Substitution	Nonsynonymous coding	19%
MM12T	DUSP27	dual specificity phosphatase 27 (putative)	CCDS30932.1	chr1_167097384-167097384_C_T	1006R>W	Substitution	Nonsynonymous coding	29%
MM12T	DUSP4	dual specificity phosphatase 4	CCDS6072.1	chr8_29194790-29194790_C_T	313R>H	Substitution	Nonsynonymous coding	11%
MM12T	DUSP8	dual specificity phosphatase 8	CCDS7724.1	chr11_1580277-1580277_C_T	127A>T	Substitution	Nonsynonymous coding	15%
MM12T	DYM	dymeclin	CCDS11937.1	chr18_46812951-46812951_C_T	267G>S	Substitution	Nonsynonymous coding	40%
MM12T	DYNC1H1	dynein, cytoplasmic 1, heavy chain 1	CCDS9966.1	chr14_102449797-102449797_G_A	438V>I	Substitution	Nonsynonymous coding	12%
MM12T	DYNC1H1	dynein, cytoplasmic 1, heavy chain 1	CCDS9966.1	chr14_102471170-102471170_G_T	1707K>N	Substitution	Nonsynonymous coding	28%
MM12T	DYNC1H1	dynein, cytoplasmic 1, heavy chain 1	CCDS9966.1	chr14_102496216-102496216_G_A	3235A>T	Substitution	Nonsynonymous coding	28%
MM12T	DYNC1H1	dynein, cytoplasmic 1, heavy chain 1	CCDS9966.1	chr14_102505353-102505353_G_A	3741R>H	Substitution	Nonsynonymous coding	11%
MM12T	DYNC1H1	dynein, cytoplasmic 1, heavy chain 1	CCDS9966.1	chr14_102478454-102478454_C_T	ISV+4>	Substitution	Splice site donor	14%
MM12T	DYNLL2	dynein, light chain, LC8-type 2	CCDS11601.1	chr17_56164482-56164482_G_A	11A>T	Substitution	Nonsynonymous coding	11%
MM12T	DZANK1	double zinc ribbon and ankyrin repeat domains 1	CCDS46582.1	chr20_18395083-18395083_C_T	384G>D	Substitution	Nonsynonymous coding	13%
MM12T	DZIP1	DAZ interacting protein 1	CCDS9478.1	chr13_96285515-96285515_G_T	220R>S	Substitution	Nonsynonymous coding	32%
MM12T	E2F5	E2F transcription factor 5, p130-binding	CCDS47885.1	chr8_86114484-86114484_C_A	113Q>K	Substitution	Nonsynonymous coding	16%
MM12T	E4F1	E4F transcription factor 1	CCDS32370.1	chr16_2284376-2284376_G_A	527R>H	Substitution	Nonsynonymous coding	23%
MM12T	EBF3	early B-cell factor 3	CCDS31314.1	chr10_131757270-131757270_G_A	138A>V	Substitution	Nonsynonymous coding	31%
MM12T	ECD	ecdysoneless homolog (Drosophila)	CCDS44434.1	chr10_74906123-74906123__A	NA	Insertion	Splice site acceptor	16%
MM12T	ECEL1	endothelin converting enzyme-like 1	CCDS2493.1	chr2_233349303-233349303_G_A	355R>W	Substitution	Nonsynonymous coding	17%
MM12T	ECHDC2	enoyl CoA hydratase domain containing 2	CCDS571.1	chr1_53364881-53364881_G_A	209R>W	Substitution	Nonsynonymous coding	36%
MM12T	EDA	ectodysplasin A	CCDS14394.1	chrX_69253319-69253319_C_T	289R>C	Substitution	Nonsynonymous coding	31%
MM12T	EDC3	enhancer of mRNA decapping 3 homolog (S. cerevisiae)	CCDS10267.1	chr15_74932913-74932913_G_T	290L>M	Substitution	Nonsynonymous coding	31%
MM12T	EDC4	enhancer of mRNA decapping 4	CCDS10849.1	chr16_67915241-67915241_C_A	904L>M	Substitution	Nonsynonymous coding	28%
MM12T	EDEM2	ER degradation enhancer, mannosidase alpha-like 2	CCDS13247.1	chr20_33734720-33734720_C_A	42M>I	Substitution	Nonsynonymous coding	12%
MM12T	EEA1	early endosome antigen 1	CCDS31874.1	chr12_93206833-93206833_C_T	656A>T	Substitution	Nonsynonymous coding	13%
MM12T	EED	embryonic ectoderm development	CCDS8273.1	chr11_85963275-85963275_G_A	118S>N	Substitution	Nonsynonymous coding	12%
MM12T	EEF2	eukaryotic translation elongation factor 2	CCDS12117.1	chr19_3977334-3977334_C_A	754Q>H	Substitution	Nonsynonymous coding	32%
MM12T	EFCAB13	EF-hand calcium binding domain 13	CCDS11512.1	chr17_45456584-45456584_C_T	516A>V	Substitution	Nonsynonymous coding	35%
MM12T	EFCAB4A	EF-hand calcium binding domain 4A	CCDS41588.1	chr11_829356-829356_G_A	ISV-4>	Substitution	Splice site acceptor	25%

MM12T	EFCAB4B	EF-hand calcium binding domain 4B	CCDS44803.1	chr12_3757704-3757704_G_A	ISV+4>	Substitution	Splice site donor	32%
MM12T	EFCAB6	EF-hand calcium binding domain 6	CCDS14049.1	chr22_43926760-43926760_G_A	1440R>W	Substitution	Nonsynonymous coding	17%
MM12T	EFHB	EF-hand domain family, member B	CCDS33715.2	chr3_19975387-19975387_G_A	42R>X	Substitution	Nonsense	26%
MM12T	EFHC1	EF-hand domain (C-terminal) containing 1	CCDS4942.1	chr6_52303292-52303292_G_A	159R>Q	Substitution	Nonsynonymous coding	19%
MM12T	EFTUD1	elongation factor Tu GTP binding domain containing 1	CCDS42071.1	chr15_82456236-82456236_C_T	614A>T	Substitution	Nonsynonymous coding	24%
MM12T	EFTUD1	elongation factor Tu GTP binding domain containing 1	CCDS42071.1	chr15_82519875-82519875_T_C	370T>A	Substitution	Nonsynonymous coding	21%
MM12T	EGF	epidermal growth factor	CCDS3689.1	chr4_110834498-110834498_C_T	3L>F	Substitution	Nonsynonymous coding	39%
MM12T	EGF	epidermal growth factor	CCDS3689.1	chr4_110932474-110932474_C_T	1163R>X	Substitution	Nonsense	11%
MM12T	EGFL8	EGF-like-domain, multiple 8	CCDS4743.1	chr6_32135717-32135717_G_A	289G>D	Substitution	Nonsynonymous coding	11%
MM12T	EGFLAM	EGF-like, fibronectin type III and laminin G domains	CCDS3924.1	chr5_38427153-38427153_C_T	618S>F	Substitution	Nonsynonymous coding	31%
MM12T	EGR2	early growth response 2	CCDS7267.1	chr10_64573446-64573446_G_A	318R>W	Substitution	Nonsynonymous coding	32%
MM12T	EGR4	early growth response 4	NM_001965	chr2_73518709-73518709_C_T	549R>H	Substitution	Nonsynonymous coding	33%
MM12T	EGR4	early growth response 4	NM_001965	chr2_73519576-73519576_G_A	260A>V	Substitution	Nonsynonymous coding	29%
MM12T	EHBP1L1	EH domain binding protein 1-like 1	CCDS44649.1	chr11_65350045-65350045_G_T	634Q>H	Substitution	Nonsynonymous coding	33%
MM12T	EHD2	EH-domain containing 2	CCDS12704.1	chr19_48219991-48219991_G_A	41R>H	Substitution	Nonsynonymous coding	38%
MM12T	EHD3	EH-domain containing 3	CCDS1774.1	chr2_31489054-31489054_G_T	364Q>H	Substitution	Nonsynonymous coding	31%
MM12T	EHMT1	euchromatic histone-lysine N-methyltransferase 1	NM_024757	chr9_140648636-140648636_G_T	421S>I	Substitution	Nonsynonymous coding	25%
MM12T	EHMT1	euchromatic histone-lysine N-methyltransferase 1	NM_024757	chr9_140707606-140707606_G_A	1006V>M	Substitution	Nonsynonymous coding	45%
MM12T	EIF1	eukaryotic translation initiation factor 1	CCDS11403.1	chr17_39846413-39846413_G_A	90R>H	Substitution	Nonsynonymous coding	17%
MM12T	EIF2B5	eukaryotic translation initiation factor 2B, subunit 5 epsilon, 82kDa	CCDS3252.1	chr3_183858233-183858233_G_A	291V>I	Substitution	Nonsynonymous coding	31%
MM12T	EIF2B5	eukaryotic translation initiation factor 2B, subunit 5 epsilon, 82kDa	CCDS3252.1	chr3_183861235-183861235_C_T	584A>V	Substitution	Nonsynonymous coding	12%
MM12T	EIF2S1	eukaryotic translation initiation factor 2, subunit 1 alpha, 35kDa	CCDS9781.1	chr14_67849201-67849201_C_T	239T>M	Substitution	Nonsynonymous coding	12%
MM12T	EIF3A	eukaryotic translation initiation factor 3, subunit A	CCDS7608.1	chr10_120795600-120795600_C_T	1367R>H	Substitution	Nonsynonymous coding	18%
MM12T	EIF3G	eukaryotic translation initiation factor 3, subunit G	CCDS12227.1	chr19_10229396-10229396_C_T	83R>H	Substitution	Nonsynonymous coding	29%
MM12T	EIF3G	eukaryotic translation initiation factor 3, subunit G	CCDS12227.1	chr19_10227865-10227865_C_A	ISV-1>	Substitution	Splice site acceptor	14%
MM12T	EIF4E1B	eukaryotic translation initiation factor 4E family member 1B	CCDS47345.1	chr5_176072494-176072494_C_A	197N>K	Substitution	Nonsynonymous coding	30%
MM12T	EIF4G1	eukaryotic translation initiation factor 4 gamma, 1	CCDS3259.1	chr3_184039259-184039259_C_A	296S>Y	Substitution	Nonsynonymous coding	35%
MM12T	EIF4G1	eukaryotic translation initiation factor 4 gamma, 1	CCDS3259.1	chr3_184039307-184039307_G_A	312R>H	Substitution	Nonsynonymous coding	17%
MM12T	EIF4G1	eukaryotic translation initiation factor 4 gamma, 1	CCDS3259.1	chr3_184039660-184039660_G_T	430A>S	Substitution	Nonsynonymous coding	29%
MM12T	EIF4G2	eukaryotic translation initiation factor 4 gamma, 2	ENST00000429377	chr11_10826471-10826471_C_T	187V>I	Substitution	Nonsynonymous coding	18%
MM12T	EIF4G3	eukaryotic translation initiation factor 4 gamma, 3	CCDS214.1	chr1_21191610-21191610_G_A	867A>V	Substitution	Nonsynonymous coding	10%
MM12T	EIF5B	eukaryotic translation initiation factor 5B	CCDS42721.1	chr2_99977939-99977939_A_G	192E>G	Substitution	Nonsynonymous coding	43%

MM12T	ELFN1	extracellular leucine-rich repeat and fibronectin type III domain containing 1	NM_001128636	chr7_1784905-1784905_G_A	225V>I	Substitution	Nonsynonymous coding	31%
MM12T	ELFN1	extracellular leucine-rich repeat and fibronectin type III domain containing 1	NM_001128636	chr7_1785023-1785023_G_A	264R>H	Substitution	Nonsynonymous coding	28%
MM12T	ELFN1	extracellular leucine-rich repeat and fibronectin type III domain containing 1	NM_001128636	chr7_1786672-1786672_G_A	814D>N	Substitution	Nonsynonymous coding	28%
MM12T	ELFN2	extracellular leucine-rich repeat and fibronectin type III domain containing 2	CCDS33642.1	chr22_37770317-37770317_G_A	420R>C	Substitution	Nonsynonymous coding	19%
MM12T	ELL	elongation factor RNA polymerase II	CCDS12380.1	chr19_18561307-18561307_C_T	482G>E	Substitution	Nonsynonymous coding	12%
MM12T	ELMO3	engulfment and cell motility 3	CCDS10833.2	chr16_67233457-67233457_C_T	80A>V	Substitution	Nonsynonymous coding	22%
MM12T	ELMO3	engulfment and cell motility 3	CCDS10833.2	chr16_67236847-67236847_C_T	592R>C	Substitution	Nonsynonymous coding	32%
MM12T	ELMSAN1	ELM2 and Myb/SANT-like domain containing 1	CCDS9819.1	chr14_74205678-74205678_C_T	345R>H	Substitution	Nonsynonymous coding	11%
MM12T	ELN	elastin	ENST00000358929	chr7_73474852-73474852_C_T	625R>W	Substitution	Nonsynonymous coding	37%
MM12T	ELP2	elongator acetyltransferase complex subunit 2	CCDS11918.1	chr18_33740902-33740902_C_T	566H>Y	Substitution	Nonsynonymous coding	27%
MM12T	EME1	essential meiotic endonuclease 1 homolog 1 (S. pombe)	CCDS11565.1	chr17_48456864-48456864_A_G	428S>G	Substitution	Nonsynonymous coding	28%
MM12T	EMILIN1	elastin microfibril interfacer 1	CCDS1733.1	chr2_27305025-27305025_C_T	196R>W	Substitution	Nonsynonymous coding	34%
MM12T	EMILIN1	elastin microfibril interfacer 1	CCDS1733.1	chr2_27305731-27305731_C_T	431A>V	Substitution	Nonsynonymous coding	26%
MM12T	EMILIN3	elastin microfibril interfacer 3	CCDS13316.1	chr20_39990977-39990977_C_T	411G>D	Substitution	Nonsynonymous coding	32%
MM12T	EML5	echinoderm microtubule associated protein like 5	CCDS45148.1	chr14_89171833-89171833_C_T	642R>H	Substitution	Nonsynonymous coding	16%
MM12T	EML5	echinoderm microtubule associated protein like 5	CCDS45148.1	chr14_89202849-89202849_C_T	303S>N	Substitution	Nonsynonymous coding	28%
MM12T	EML6	echinoderm microtubule associated protein like 6	CCDS46286.1	chr2_55106696-55106696_G_A	786C>Y	Substitution	Nonsynonymous coding	22%
MM12T	EMP1	epithelial membrane protein 1	CCDS8660.1	chr12_13366647-13366647_C_A	70L>I	Substitution	Nonsynonymous coding	14%
MM12T	EMR1	egf-like module containing, mucin-like, hormone receptor-like 1	CCDS12175.1	chr19_6906515-6906515_G_A	341A>T	Substitution	Nonsynonymous coding	33%
MM12T	EMR2	egf-like module containing, mucin-like, hormone receptor-like 2	CCDS32935.1	chr19_14884819-14884819_C_T	44A>T	Substitution	Nonsynonymous coding	38%
MM12T	ENO3	enolase 3 (beta, muscle)	CCDS11062.1	chr17_4860156-4860156_C_T	403R>C	Substitution	Nonsynonymous coding	25%
MM12T	ENO4	enolase family member 4	ENST00000341276	chr10_118633618-118633618_C_T	419T>I	Substitution	Nonsynonymous coding	27%
MM12T	ENPP2	ectonucleotide pyrophosphatase/phosphodiesterase 2	CCDS6329.1	chr8_120596231-120596231_C_T	528G>R	Substitution	Nonsynonymous coding	10%
MM12T	ENPP3	ectonucleotide pyrophosphatase/phosphodiesterase 3	CCDS5148.1	chr6_132006596-132006596_C_T	405R>C	Substitution	Nonsynonymous coding	40%
MM12T	ENSG00000005189	-	CCDS10591.1	chr16_20857622-20857622_C_T	735P>L	Substitution	Nonsynonymous coding	29%
MM12T	ENSG00000105520	-	CCDS12258.1	chr19_11473201-11473201_C_A	226L>I	Substitution	Nonsynonymous coding	26%
MM12T	ENSG00000105663	-	CCDS46055.1	chr19_36212263-36212263_G_A	672G>R	Substitution	Nonsynonymous coding	35%
MM12T	ENSG00000105663	-	CCDS46055.1	chr19_36213366-36213366_C_T	855R>W	Substitution	Nonsynonymous coding	32%
MM12T	ENSG00000129951	-	CCDS12043.1	chr19_812877-812877_C_T	645G>D	Substitution	Nonsynonymous coding	47%
MM12T	ENSG00000129951	-	CCDS12043.1	chr19_812889-812889_G_A	641A>V	Substitution	Nonsynonymous coding	47%
MM12T	ENSG00000129951	-	CCDS12043.1	chr19_812910-812910_G_A	634A>V	Substitution	Nonsynonymous coding	11%
MM12T	ENSG00000143674	-	CCDS1598.1	chr1_233497907-233497907_G_A	474V>M	Substitution	Nonsynonymous coding	18%

MM12T	ENSG00000163526	-	ENST00000413793	chr2_220136156-220136156_G_A	179C>Y	Substitution	Nonsynonymous coding	14%
MM12T	ENSG00000172901	-	CCDS4124.1	chr5_115298438-115298438_C_T	42R>C	Substitution	Nonsynonymous coding	13%
MM12T	ENSG00000173517	-	CCDS42062.1	chr15_77472837-77472837_C_T	478V>M	Substitution	Nonsynonymous coding	21%
MM12T	ENSG00000175901	-	ENST00000321795	chr17_79005001-79005001_G_A	91A>V	Substitution	Nonsynonymous coding	11%
MM12T	ENSG00000196624	-	ENST00000457893	chr6_36690394-36690394_C_T	531A>V	Substitution	Nonsynonymous coding	10%
MM12T	ENSG00000196624	-	ENST00000457893	chr6_36699781-36699781_T_G	696S>A	Substitution	Nonsynonymous coding	28%
MM12T	ENSG00000202582	-	ENST00000365712	chr12_50271495-50271495_C_T	401R>H	Substitution	Nonsynonymous coding	29%
MM12T	ENSG00000204325	-	ENST00000226258	chr17_73620880-73620880_G_A	249E>K	Substitution	Nonsynonymous coding	31%
MM12T	ENSG00000204987	-	ENST00000378229	chr7_141883072-141883072_G_T	202A>S	Substitution	Nonsynonymous coding	11%
MM12T	ENSG00000204987	-	ENST00000378229	chr7_141901351-141901351_C_T	642T>I	Substitution	Nonsynonymous coding	15%
MM12T	ENSG00000206016	-	ENST00000382619	chr7_821821-821821_C_T	29R>W	Substitution	Nonsynonymous coding	16%
MM12T	ENSG00000206016	-	ENST00000382619	chr7_822008-822008_G_A	91R>H	Substitution	Nonsynonymous coding	13%
MM12T	ENSG00000214088	-	ENST00000397504	chr7_141837298-141837298_G_A	305V>I	Substitution	Nonsynonymous coding	15%
MM12T	ENSG00000214395	-	ENST00000398271	chr6_116947208-116947208_G_A	1G>E	Substitution	Nonsynonymous coding	24%
MM12T	ENSG00000214877	-	ENST00000399160	chr9_94949164-94949164_G_A	174A>V	Substitution	Nonsynonymous coding	31%
MM12T	ENSG00000220191	-	ENST00000404353	chr10_81680172-81680172_G_T	30K>N	Substitution	Nonsynonymous coding	10%
MM12T	ENSG00000223354	-	ENST00000412854	chr13_25715858-25715858__A	NA	Insertion	Frameshift	23%
MM12T	ENSG00000226190	-	ENST00000437239	chr14_107078537-107078537_G_A	63R>C	Substitution	Nonsynonymous coding	13%
MM12T	ENSG00000226778	-	ENST00000412457	chr8_67525327-67525327_T_C	34D>G	Substitution	Nonsynonymous coding	35%
MM12T	ENSG00000231657	-	ENST00000441104	chr11_558069-558069_G_	NA	Deletion	Frameshift	36%
MM12T	ENSG00000233530	-	ENST00000419858	chr1_31902395-31902395_G_T	74L>M	Substitution	Nonsynonymous coding	32%
MM12T	ENSG00000233855	-	ENST00000426581	chr14_106322218-106322218_C_T	182D>N	Substitution	Nonsynonymous coding	15%
MM12T	ENSG00000236410	-	ENST00000412560	chr16_10960301-10960301_C_T	52A>V	Substitution	Nonsynonymous coding	10%
MM12T	ENSG00000236585	-	ENST00000451999	chr17_76422530-76422530_G_A	41G>D	Substitution	Nonsynonymous coding	15%
MM12T	ENTPD2	ectonucleoside triphosphate diphosphohydrolase 2	CCDS7026.1	chr9_139948421-139948421_G_T	10P>Q	Substitution	Nonsynonymous coding	32%
MM12T	ENTPD3	ectonucleoside triphosphate diphosphohydrolase 3	CCDS2691.1	chr3_40456261-40456261_T_C	176I>T	Substitution	Nonsynonymous coding	31%
MM12T	ENTPD4	ectonucleoside triphosphate diphosphohydrolase 4	CCDS6041.1	chr8_23301467-23301467_C_T	ISV-1>	Substitution	Splice site acceptor	19%
MM12T	ENTPD6	ectonucleoside triphosphate diphosphohydrolase 6 (putative)	CCDS13170.1	chr20_25204788-25204788_A_C	413K>T	Substitution	Nonsynonymous coding	27%
MM12T	ENTPD7	ectonucleoside triphosphate diphosphohydrolase 7	CCDS7480.1	chr10_101421342-101421342_C_T	50R>X	Substitution	Nonsense	11%
MM12T	ENY2	enhancer of yellow 2 homolog (Drosophila)	CCDS43762.1	chr8_110348432-110348432_C_T	28R>C	Substitution	Nonsynonymous coding	34%
MM12T	EP300	E1A binding protein p300	CCDS14010.1	chr22_41542746-41542746_G_A	686G>D	Substitution	Nonsynonymous coding	12%
MM12T	EP400	E1A binding protein p400	CCDS31929.1	chr12_132551915-132551915_G_A	2919V>M	Substitution	Nonsynonymous coding	28%

MM12T	EP400	E1A binding protein p400	CCDS31929.1	chr12_132464311-132464311_C_T	469Q>X	Substitution	Nonsense	38%
MM12T	EPB41L1	erythrocyte membrane protein band 4.1-like 1	ENST00000344237	chr20_34794302-34794302_G_T	343G>X	Substitution	Nonsense	29%
MM12T	EPB41L3	erythrocyte membrane protein band 4.1-like 3	CCDS11838.1	chr18_5395621-5395621_G_A	1020T>M	Substitution	Nonsynonymous coding	25%
MM12T	EPB41L3	erythrocyte membrane protein band 4.1-like 3	CCDS11838.1	chr18_5428360-5428360_C_A	339K>N	Substitution	Nonsynonymous coding	27%
MM12T	EPB41L3	erythrocyte membrane protein band 4.1-like 3	CCDS11838.1	chr18_5478291-5478291_C_T	110M>I	Substitution	Nonsynonymous coding	22%
MM12T	EPB41L5	erythrocyte membrane protein band 4.1 like 5	CCDS2130.1	chr2_120932419-120932419_C_T	713S>F	Substitution	Nonsynonymous coding	14%
MM12T	EPB49	erythrocyte membrane protein band 4.9 (dematin)	CCDS6020.1	chr8_21926526-21926526_G_T	ISV-1>	Substitution	Splice site acceptor	28%
MM12T	EPC1	enhancer of polycomb homolog 1 (Drosophila)	CCDS7172.1	chr10_32582026-32582026_G_A	186R>X	Substitution	Nonsense	16%
MM12T	EPG5	ectopic P-granules autophagy protein 5 homolog (C. elegans)	CCDS11926.2	chr18_43526703-43526703_G_A	535P>S	Substitution	Nonsynonymous coding	28%
MM12T	EPHA3	EPH receptor A3	CCDS2922.1	chr3_89468516-89468516_C_T	684R>X	Substitution	Nonsense	20%
MM12T	EPHA6	EPH receptor A6	CCDS46876.1	chr3_96706463-96706463_C_T	247A>V	Substitution	Nonsynonymous coding	26%
MM12T	EPHA7	EPH receptor A7	CCDS5031.1	chr6_93969173-93969173_T_C	608Y>C	Substitution	Nonsynonymous coding	20%
MM12T	EPHA7	EPH receptor A7	CCDS5031.1	chr6_94068079-94068079_G_A	295R>C	Substitution	Nonsynonymous coding	49%
MM12T	EPHB2	EPH receptor B2	CCDS230.1	chr1_23189599-23189599_G_A	294R>Q	Substitution	Nonsynonymous coding	22%
MM12T	EPHB3	EPH receptor B3	CCDS3268.1	chr3_184298829-184298829_C_A	870L>I	Substitution	Nonsynonymous coding	31%
MM12T	EPM2AIP1	EPM2A (laforin) interacting protein 1	CCDS46790.1	chr3_37033763-37033763_C_T	269S>N	Substitution	Nonsynonymous coding	11%
MM12T	EPN2	epsin 2	CCDS11203.1	chr17_19186703-19186703_C_T	91R>C	Substitution	Nonsynonymous coding	28%
MM12T	EPN3	epsin 3	CCDS11570.1	chr17_48614374-48614374_C_T	153R>C	Substitution	Nonsynonymous coding	28%
MM12T	EPPK1	epiplakin 1	NM_031308	chr8_144945063-144945063_G_A	787R>C	Substitution	Nonsynonymous coding	36%
MM12T	EPS8	epidermal growth factor receptor pathway substrate 8	CCDS31753.1	chr12_15776224-15776224__A	NA	Insertion	Splice site acceptor	17%
MM12T	EPS8L1	EPS8-like 1 [Source:HGNC Symbol;Acc:21295]	CCDS12914.1	chr19_55597298-55597298_G_A	492R>H	Substitution	Nonsynonymous coding	19%
MM12T	EPYC	epiphycan	CCDS31870.1	chr12_91372043-91372044_AA_	NA	Deletion	Splice site acceptor	20%
MM12T	ERBB2	v-erb-b2 erythroblastic leukemia viral oncogene homolog 2, neuro/glioblastoma	CCDS32642.1	chr17_37866135-37866135_G_A	ISV+1>	Substitution	Splice site donor	19%
MM12T	ERBB3	v-erb-b2 erythroblastic leukemia viral oncogene homolog 3 (avian)	CCDS31833.1	chr12_56474091-56474091_G_A	3A>T	Substitution	Nonsynonymous coding	28%
MM12T	ERBB3	v-erb-b2 erythroblastic leukemia viral oncogene homolog 3 (avian)	CCDS31833.1	chr12_56493456-56493456_G_A	955R>H	Substitution	Nonsynonymous coding	11%
MM12T	ERC1	ELKS/RAB6-interacting/CAST family member 1	CCDS8508.1	chr12_1137085-1137085_C_T	6R>C	Substitution	Nonsynonymous coding	24%
MM12T	ERCC1	excision repair cross-complementing rodent repair deficiency, complementation group 1	CCDS12663.1	chr19_45923683-45923683_C_A	108R>S	Substitution	Nonsynonymous coding	32%
MM12T	ERCC2	excision repair cross-complementing rodent repair deficiency, complementation group 2	CCDS33049.1	chr19_45856014-45856014_C_T	631R>H	Substitution	Nonsynonymous coding	31%
MM12T	ERCC2	excision repair cross-complementing rodent repair deficiency, complementation group 2	CCDS33049.1	chr19_45858067-45858067_G_T	529A>D	Substitution	Nonsynonymous coding	28%
MM12T	ERCC6L	excision repair cross-complementing rodent repair deficiency, complementation group 6-like	CCDS35329.1	chrX_71425591-71425591_T_G	1009E>A	Substitution	Nonsynonymous coding	11%
MM12T	ERGIC1	endoplasmic reticulum-golgi intermediate compartment (ERGIC) 1	ENST00000265089	chr5_172342557-172342557_C_T	138R>C	Substitution	Nonsynonymous coding	31%
MM12T	ERGIC3	ERGIC and golgi 3	CCDS13258.1	chr20_34144868-34144868_C_T	340T>M	Substitution	Nonsynonymous coding	28%

MM12T	ERN1	endoplasmic reticulum to nucleus signaling 1	CCDS45762.1	chr17_62144065-62144065_A_G	270F>L	Substitution	Nonsynonymous coding	13%
MM12T	ERN2	endoplasmic reticulum to nucleus signaling 2	CCDS32407.1	chr16_23702279-23702279_C_T	933R>H	Substitution	Nonsynonymous coding	14%
MM12T	ERN2	endoplasmic reticulum to nucleus signaling 2	CCDS32407.1	chr16_23716337-23716337_G_A	289R>C	Substitution	Nonsynonymous coding	19%
MM12T	ERP27	endoplasmic reticulum protein 27	CCDS8670.1	chr12_15090917-15090917_G_A	55A>V	Substitution	Nonsynonymous coding	14%
MM12T	ERV3-1	endogenous retrovirus group 3, member 1	CCDS47595.1	chr7_64453199-64453199_G_A	69S>L	Substitution	Nonsynonymous coding	25%
MM12T	ESCO2	establishment of cohesion 1 homolog 2 (S. cerevisiae)	CCDS34872.1	chr8_27634330-27634330_C_T	169R>X	Substitution	Nonsense	13%
MM12T	ESPL1	extra spindle pole bodies homolog 1 (S. cerevisiae)	CCDS8852.1	chr12_53664188-53664188_G_T	390Q>H	Substitution	Nonsynonymous coding	32%
MM12T	ESPL1	extra spindle pole bodies homolog 1 (S. cerevisiae)	CCDS8852.1	chr12_53687185-53687185_G_A	2097R>H	Substitution	Nonsynonymous coding	14%
MM12T	ESPNL	espin-like	CCDS2525.1	chr2_239037448-239037448_G_A	439R>Q	Substitution	Nonsynonymous coding	13%
MM12T	ESRP1	epithelial splicing regulatory protein 1	CCDS47897.1	chr8_95686621-95686621_T_C	513M>T	Substitution	Nonsynonymous coding	29%
MM12T	ESRRG	estrogen-related receptor gamma	CCDS41468.1	chr1_216692665-216692665_C_T	321E>K	Substitution	Nonsynonymous coding	29%
MM12T	ESYT1	extended synaptotagmin-like protein 1	CCDS8904.1	chr12_56526521-56526521_C_T	394P>S	Substitution	Nonsynonymous coding	35%
MM12T	ESYT1	extended synaptotagmin-like protein 1	CCDS8904.1	chr12_56531820-56531820_C_T	737R>C	Substitution	Nonsynonymous coding	32%
MM12T	ESYT1	extended synaptotagmin-like protein 1	CCDS8904.1	chr12_56532216-56532216_C_T	789T>I	Substitution	Nonsynonymous coding	26%
MM12T	ETAA1	Ewing tumor-associated antigen 1	CCDS1882.1	chr2_67630904-67630904_C_T	364P>S	Substitution	Nonsynonymous coding	30%
MM12T	ETFDH	electron-transferring-flavoprotein dehydrogenase	CCDS3800.1	chr4_159627385-159627385_G_A	444V>I	Substitution	Nonsynonymous coding	18%
MM12T	ETS1	v-ets erythroblastosis virus E26 oncogene homolog 1 (avian)	CCDS44767.1	chr11_128350152-128350152_G_A	353R>W	Substitution	Nonsynonymous coding	13%
MM12T	ETS1	v-ets erythroblastosis virus E26 oncogene homolog 1 (avian)	CCDS44767.1	chr11_128360403-128360403_C_T	95A>T	Substitution	Nonsynonymous coding	25%
MM12T	ETV5	ets variant 5	CCDS33906.1	chr3_185766567-185766567_G_A	465P>L	Substitution	Nonsynonymous coding	17%
MM12T	EVA1C	eva-1 homolog C (C. elegans)	CCDS13614.1	chr21_33887382-33887382_C_T	403S>F	Substitution	Nonsynonymous coding	36%
MM12T	EVA1C	eva-1 homolog C (C. elegans)	CCDS13614.1	chr21_33887264-33887264_G_T	364G>X	Substitution	Nonsense	15%
MM12T	EVI2A	ecotropic viral integration site 2A	CCDS32608.1	chr17_29645706-29645706_C_A	132S>I	Substitution	Nonsynonymous coding	32%
MM12T	EVI2B	ecotropic viral integration site 2B	CCDS11266.1	chr17_29631642-29631642_G_A	329A>V	Substitution	Nonsynonymous coding	35%
MM12T	EVPL	envoplakin	CCDS11737.1	chr17_74005000-74005000_C_T	1429R>H	Substitution	Nonsynonymous coding	13%
MM12T	EVPL	envoplakin	CCDS11737.1	chr17_74005615-74005615_C_A	1224R>M	Substitution	Nonsynonymous coding	37%
MM12T	EVPLL	envoplakin-like	CCDS45626.1	chr17_18285949-18285949_G_A	157A>T	Substitution	Nonsynonymous coding	10%
MM12T	EVX2	even-skipped homeobox 2	CCDS33333.1	chr2_176948128-176948128_C_T	126R>H	Substitution	Nonsynonymous coding	36%
MM12T	EVX2	even-skipped homeobox 2	CCDS33333.1	chr2_176948384-176948384_C_T	41E>K	Substitution	Nonsynonymous coding	13%
MM12T	EXD2	exonuclease 3'-5' domain containing 2	ENST00000312994	chr14_69676833-69676833_G_A	113R>H	Substitution	Nonsynonymous coding	33%
MM12T	EXO1	exonuclease 1	CCDS1620.1	chr1_242042480-242042480_G_A	648M>I	Substitution	Nonsynonymous coding	36%
MM12T	EXOC1	exocyst complex component 1	CCDS3502.1	chr4_56768600-56768600_C_T	810R>C	Substitution	Nonsynonymous coding	17%
MM12T	EXOC2	exocyst complex component 2	CCDS34327.1	chr6_491138-491138_T_C	870T>A	Substitution	Nonsynonymous coding	23%

MM12T	EXOC3	exocyst complex component 3	NM_007277	chr5_453602-453602_G_A	161R>H	Substitution	Nonsynonymous coding	14%
MM12T	EXOC6	exocyst complex component 6	CCDS7424.2	chr10_94653132-94653132_C_T	43A>V	Substitution	Nonsynonymous coding	30%
MM12T	EXOC6	exocyst complex component 6	CCDS7424.2	chr10_94757290-94757290_G_A	671M>I	Substitution	Nonsynonymous coding	11%
MM12T	EXOC6	exocyst complex component 6	CCDS7424.2	chr10_94688093-94688093_T_C	ISV-3>	Substitution	Splice site acceptor	11%
MM12T	EXOC8	exocyst complex component 8	CCDS1593.1	chr1_231471733-231471733_C_A	587G>C	Substitution	Nonsynonymous coding	26%
MM12T	EXOC8	exocyst complex component 8	CCDS1593.1	chr1_231472161-231472161_C_T	444R>H	Substitution	Nonsynonymous coding	30%
MM12T	EXPH5	exophilin 5	CCDS8341.1	chr11_108383613-108383613_C_T	874C>Y	Substitution	Nonsynonymous coding	28%
MM12T	EXT1	exostosin 1	CCDS6324.1	chr8_118847712-118847712_C_T	379V>I	Substitution	Nonsynonymous coding	38%
MM12T	EXT2	exostosin 2	CCDS7908.1	chr11_44129790-44129790_G_T	176Q>H	Substitution	Nonsynonymous coding	15%
MM12T	EXTL3	exostoses (multiple)-like 3	CCDS6070.1	chr8_28608217-28608217_C_T	865A>V	Substitution	Nonsynonymous coding	11%
MM12T	EYA1	eyes absent homolog 1 (Drosophila)	CCDS34906.1	chr8_72234013-72234013_G_A	125A>V	Substitution	Nonsynonymous coding	36%
MM12T	EYS	eyes shut homolog (Drosophila)	CCDS47445.1	chr6_66094317-66094317_T_C	421N>D	Substitution	Nonsynonymous coding	21%
MM12T	F2RL3	coagulation factor II (thrombin) receptor-like 3	CCDS12350.1	chr19_17001115-17001115_G_A	281A>T	Substitution	Nonsynonymous coding	14%
MM12T	F5	coagulation factor V (proaccelerin, labile factor)	CCDS1281.1	chr1_169494129-169494129_C_T	1912G>R	Substitution	Nonsynonymous coding	32%
MM12T	FADS2	fatty acid desaturase 2	CCDS8012.1	chr11_61631185-61631185_G_A	362A>T	Substitution	Nonsynonymous coding	25%
MM12T	FADS3	fatty acid desaturase 3	CCDS8013.1	chr11_61647584-61647584_G_A	85R>C	Substitution	Nonsynonymous coding	13%
MM12T	FADS3	fatty acid desaturase 3	CCDS8013.1	chr11_61643451-61643451_G_	NA	Deletion	Splice site acceptor	20%
MM12T	FAIM3	Fas apoptotic inhibitory molecule 3	CCDS1473.1	chr1_207083120-207083120_T_G	312N>T	Substitution	Nonsynonymous coding	29%
MM12T	FAM101A	family with sequence similarity 101, member A	CCDS9258.1	chr12_124796421-124796421_G_A	8V>M	Substitution	Nonsynonymous coding	14%
MM12T	FAM102B	family with sequence similarity 102, member B	CCDS30786.2	chr1_109170872-109170872_T_C	214S>P	Substitution	Nonsynonymous coding	11%
MM12T	FAM108B1	family with sequence similarity 108, member B1	CCDS35042.1	chr9_74485173-74485173_C_T	158G>D	Substitution	Nonsynonymous coding	22%
MM12T	FAM109B	family with sequence similarity 109, member B	CCDS33655.1	chr22_42473469-42473469_C_T	58R>C	Substitution	Nonsynonymous coding	33%
MM12T	FAM110A	family with sequence similarity 110, member A	CCDS13008.1	chr20_826222-826222_C_T	259R>W	Substitution	Nonsynonymous coding	33%
MM12T	FAM111A	family with sequence similarity 111, member A	CCDS7973.1	chr11_58919526-58919526_C_T	129R>C	Substitution	Nonsynonymous coding	11%
MM12T	FAM120C	family with sequence similarity 120C	CCDS14356.1	chrX_54107891-54107891_G_T	948L>I	Substitution	Nonsynonymous coding	41%
MM12T	FAM120C	family with sequence similarity 120C	CCDS14356.1	chrX_54209433-54209433_G_A	67L>F	Substitution	Nonsynonymous coding	28%
MM12T	FAM124A	family with sequence similarity 124A	CCDS9427.1	chr13_51825816-51825816_C_A	141L>M	Substitution	Nonsynonymous coding	20%
MM12T	FAM124B	family with sequence similarity 124B	CCDS46527.1	chr2_225266204-225266204_C_T	94W>X	Substitution	Nonsense	15%
MM12T	FAM129A	family with sequence similarity 129, member A	CCDS1364.1	chr1_184764173-184764173_C_T	909D>N	Substitution	Nonsynonymous coding	13%
MM12T	FAM129A	family with sequence similarity 129, member A	CCDS1364.1	chr1_184777288-184777288_G_A	419R>C	Substitution	Nonsynonymous coding	24%
MM12T	FAM129B	family with sequence similarity 129, member B	CCDS35145.1	chr9_130271282-130271282_C_A	430K>N	Substitution	Nonsynonymous coding	10%
MM12T	FAM132A	family with sequence similarity 132, member A	CCDS30554.1	chr1_1177932-1177932_G_A	302T>M	Substitution	Nonsynonymous coding	37%

MM12T	FAM135B	family with sequence similarity 135, member B	CCDS6375.2	chr8_139164067-139164067_C_T	884R>H	Substitution	Nonsynonymous coding	35%
MM12T	FAM135B	family with sequence similarity 135, member B	CCDS6375.2	chr8_139164731-139164731_T_C	663T>A	Substitution	Nonsynonymous coding	19%
MM12T	FAM135B	family with sequence similarity 135, member B	CCDS6375.2	chr8_139207501-139207501_C_A	291Q>H	Substitution	Nonsynonymous coding	42%
MM12T	FAM13C	family with sequence similarity 13, member C	ENST00000435852	chr10_61014086-61014086_G_T	452L>M	Substitution	Nonsynonymous coding	31%
MM12T	FAM151A	family with sequence similarity 151, member A	CCDS594.1	chr1_55075512-55075512_T_C	396E>G	Substitution	Nonsynonymous coding	30%
MM12T	FAM151A	family with sequence similarity 151, member A	CCDS594.1	chr1_55088986-55088986_G_A	28A>V	Substitution	Nonsynonymous coding	21%
MM12T	FAM159A	family with sequence similarity 159, member A	NM_001042693	chr1_53108540-53108540_G_T	63G>V	Substitution	Nonsynonymous coding	42%
MM12T	FAM159A	family with sequence similarity 159, member A	NM_001042693	chr1_53122629-53122629_G_A	164A>T	Substitution	Nonsynonymous coding	28%
MM12T	FAM160B2	family with sequence similarity 160, member B2	CCDS6021.2	chr8_21958960-21958960_C_A	542C>X	Substitution	Nonsense	12%
MM12T	FAM161A	family with sequence similarity 161, member A	CCDS42687.2	chr2_62067439-62067439_G_A	234P>S	Substitution	Nonsynonymous coding	20%
MM12T	FAM162B	family with sequence similarity 162, member B	CCDS43497.1	chr6_117086580-117086580_G_A	54P>S	Substitution	Nonsynonymous coding	26%
MM12T	FAM170A	family with sequence similarity 170, member A	CCDS43353.1	chr5_118969778-118969778_C_T	112T>I	Substitution	Nonsynonymous coding	21%
MM12T	FAM171B	family with sequence similarity 171, member B	CCDS33347.1	chr2_187626468-187626468_G_A	467V>I	Substitution	Nonsynonymous coding	26%
MM12T	FAM172A	family with sequence similarity 172, member A	CCDS4069.1	chr5_93217382-93217382_C_T	194V>I	Substitution	Nonsynonymous coding	28%
MM12T	FAM177A1	family with sequence similarity 177, member A1	CCDS9653.2	chr14_35546395-35546395_C_A	127L>I	Substitution	Nonsynonymous coding	20%
MM12T	FAM179B	family with sequence similarity 179, member B	CCDS9681.1	chr14_45431853-45431853_G_A	77A>T	Substitution	Nonsynonymous coding	31%
MM12T	FAM180A	family with sequence similarity 180, member A	CCDS5841.1	chr7_135433281-135433281_C_A	16E>D	Substitution	Nonsynonymous coding	31%
MM12T	FAM184A	family with sequence similarity 184, member A	CCDS43499.1	chr6_119399367-119399367_C_T	33S>N	Substitution	Nonsynonymous coding	53%
MM12T	FAM184A	family with sequence similarity 184, member A	CCDS43499.1	chr6_119345660-119345660_C_A	160E>X	Substitution	Nonsense	16%
MM12T	FAM184B	family with sequence similarity 184, member B	CCDS47033.1	chr4_17710846-17710846_C_T	188G>D	Substitution	Nonsynonymous coding	31%
MM12T	FAM186B	family with sequence similarity 186, member B	CCDS8788.1	chr12_49998291-49998291_C_T	43D>N	Substitution	Nonsynonymous coding	13%
MM12T	FAM187A	family with sequence similarity 187, member A	ENST00000331733	chr17_42981561-42981561_C_A	122L>M	Substitution	Nonsynonymous coding	35%
MM12T	FAM188B	family with sequence similarity 188, member B	CCDS5431.1	chr7_30951846-30951846_G_A	108A>T	Substitution	Nonsynonymous coding	11%
MM12T	FAM198A	family with sequence similarity 198, member A	CCDS46808.1	chr3_43073865-43073865_G_A	37R>H	Substitution	Nonsynonymous coding	31%
MM12T	FAM198A	family with sequence similarity 198, member A	CCDS46808.1	chr3_43074608-43074608_G_A	285A>T	Substitution	Nonsynonymous coding	33%
MM12T	FAM198A	family with sequence similarity 198, member A	CCDS46808.1	chr3_43097682-43097682_C_T	511A>V	Substitution	Nonsynonymous coding	33%
MM12T	FAM208A	family with sequence similarity 208, member A	CCDS46853.1	chr3_56667772-56667772_G_A	1016T>I	Substitution	Nonsynonymous coding	26%
MM12T	FAM208A	family with sequence similarity 208, member A	CCDS46853.1	chr3_56667833-56667833_C_A	996G>C	Substitution	Nonsynonymous coding	29%
MM12T	FAM208B	family with sequence similarity 208, member B	CCDS41485.1	chr10_5789777-5789777_G_T	1465D>Y	Substitution	Nonsynonymous coding	29%
MM12T	FAM211A	family with sequence similarity 211, member A	CCDS45620.1	chr17_16365615-16365615_T_C	111D>G	Substitution	Nonsynonymous coding	28%
MM12T	FAM213A	family with sequence similarity 213, member A	CCDS7368.1	chr10_82182195-82182195_G_T	67K>N	Substitution	Nonsynonymous coding	28%
MM12T	FAM217A	family with sequence similarity 217, member A	CCDS4489.1	chr6_4069320-4069320_C_T	379W>X	Substitution	Nonsense	11%

MM12T	FAM221B	family with sequence similarity 221, member B	CCDS43799.1	chr9_35826097-35826097_G_	NA	Deletion	Frameshift	27%
MM12T	FAM227A	family with sequence similarity 227, member A	NM_001013647	chr22_39035671-39035671_G_A	95P>L	Substitution	Nonsynonymous coding	32%
MM12T	FAM3B	family with sequence similarity 3, member B	CCDS13671.1	chr21_42720607-42720607_G_A	192A>T	Substitution	Nonsynonymous coding	27%
MM12T	FAM46B	family with sequence similarity 46, member B	CCDS294.2	chr1_27332740-27332740_G_A	325R>C	Substitution	Nonsynonymous coding	35%
MM12T	FAM46B	family with sequence similarity 46, member B	CCDS294.2	chr1_27333442-27333442_G_A	91R>C	Substitution	Nonsynonymous coding	34%
MM12T	FAM47A	family with sequence similarity 47, member A	CCDS43926.1	chrX_34149902-34149902_C_T	165C>Y	Substitution	Nonsynonymous coding	28%
MM12T	FAM47E	family with sequence similarity 47, member E	CCDS47081.1	chr4_77192742-77192742_G_T	231E>X	Substitution	Nonsense	16%
MM12T	FAM50B	family with sequence similarity 50, member B	CCDS4487.1	chr6_3850692-3850692_C_T	216A>V	Substitution	Nonsynonymous coding	24%
MM12T	FAM50B	family with sequence similarity 50, member B	CCDS4487.1	chr6_3850883-3850883_G_A	280A>T	Substitution	Nonsynonymous coding	12%
MM12T	FAM53B	family with sequence similarity 53, member B	CCDS7641.1	chr10_126370235-126370235_G_A	283R>W	Substitution	Nonsynonymous coding	11%
MM12T	FAM53C	family with sequence similarity 53, member C	CCDS4204.1	chr5_137680606-137680606_C_T	77H>Y	Substitution	Nonsynonymous coding	11%
MM12T	FAM53C	family with sequence similarity 53, member C	CCDS4204.1	chr5_137681236-137681236_C_T	287R>W	Substitution	Nonsynonymous coding	30%
MM12T	FAM5B	family with sequence similarity 5, member B	CCDS1320.1	chr1_177247786-177247786_G_A	367S>N	Substitution	Nonsynonymous coding	27%
MM12T	FAM65A	family with sequence similarity 65, member A	CCDS10840.1	chr16_67576850-67576850_G_A	721A>T	Substitution	Nonsynonymous coding	30%
MM12T	FAM65C	family with sequence similarity 65, member C	CCDS13431.2	chr20_49209724-49209724_C_A	737R>M	Substitution	Nonsynonymous coding	42%
MM12T	FAM65C	family with sequence similarity 65, member C	CCDS13431.2	chr20_49227741-49227741_C_T	113A>T	Substitution	Nonsynonymous coding	36%
MM12T	FAM69C	family with sequence similarity 69, member C	CCDS42445.2	chr18_72114245-72114245_C_T	158A>T	Substitution	Nonsynonymous coding	28%
MM12T	FAM71A	family with sequence similarity 71, member A	CCDS1507.1	chr1_212799706-212799706_C_T	496T>M	Substitution	Nonsynonymous coding	28%
MM12T	FAM71F2	family with sequence similarity 71, member F2	CCDS47701.1	chr7_128323071-128323071_C_A	263S>Y	Substitution	Nonsynonymous coding	23%
MM12T	FAM81A	family with sequence similarity 81, member A	CCDS45269.1	chr15_59799425-59799425_A_G	143I>V	Substitution	Nonsynonymous coding	36%
MM12T	FAM83A	family with sequence similarity 83, member A	CCDS6340.1	chr8_124195199-124195199_G_A	35A>T	Substitution	Nonsynonymous coding	15%
MM12T	FAM83B	family with sequence similarity 83, member B	CCDS34479.1	chr6_54735062-54735062_G_A	6M>I	Substitution	Nonsynonymous coding	25%
MM12T	FAM83H	family with sequence similarity 83, member H	CCDS6410.2	chr8_144808806-144808806_G_A	942S>F	Substitution	Nonsynonymous coding	34%
MM12T	FAN1	FANCD2/FANCI-associated nuclease 1	CCDS32186.1	chr15_31217406-31217406_C_T	750A>V	Substitution	Nonsynonymous coding	29%
MM12T	FAN1	FANCD2/FANCI-associated nuclease 1	CCDS32186.1	chr15_31218056-31218056_C_T	801A>V	Substitution	Nonsynonymous coding	13%
MM12T	FANCI	Fanconi anemia, complementation group I	CCDS45346.1	chr15_89828346-89828346_G_A	573S>N	Substitution	Nonsynonymous coding	26%
MM12T	FASLG	Fas ligand (TNF superfamily, member 6)	CCDS1304.1	chr1_172634810-172634810_G_T	167G>V	Substitution	Nonsynonymous coding	30%
MM12T	FASLG	Fas ligand (TNF superfamily, member 6)	CCDS1304.1	chr1_172635129-172635129_G_T	273Q>H	Substitution	Nonsynonymous coding	14%
MM12T	FASN	fatty acid synthase	CCDS11801.1	chr17_80043001-80043001_G_T	1440P>H	Substitution	Nonsynonymous coding	21%
MM12T	FASN	fatty acid synthase	CCDS11801.1	chr17_80037337-80037337_G_A	2432Q>X	Substitution	Nonsense	23%
MM12T	FASTKD3	FAST kinase domains 3	CCDS3873.1	chr5_7867331-7867331_G_A	289S>F	Substitution	Nonsynonymous coding	19%
MM12T	FAT1	FAT tumor suppressor homolog 1 (Drosophila)	CCDS47177.1	chr4_187521072-187521072_C_T	4028C>Y	Substitution	Nonsynonymous coding	22%

MM12T	FAT1	FAT tumor suppressor homolog 1 (Drosophila)	CCDS47177.1	chr4_187535345-187535345_C_A	3077G>C	Substitution	Nonsynonymous coding	30%
MM12T	FAT1	FAT tumor suppressor homolog 1 (Drosophila)	CCDS47177.1	chr4_187540053-187540053_C_A	2563V>L	Substitution	Nonsynonymous coding	27%
MM12T	FAT1	FAT tumor suppressor homolog 1 (Drosophila)	CCDS47177.1	chr4_187630167-187630167_G_A	272A>V	Substitution	Nonsynonymous coding	24%
MM12T	FAT2	FAT tumor suppressor homolog 2 (Drosophila)	CCDS4317.1	chr5_150901233-150901233_C_T	3641E>K	Substitution	Nonsynonymous coding	12%
MM12T	FAT2	FAT tumor suppressor homolog 2 (Drosophila)	CCDS4317.1	chr5_150925497-150925497_G_A	1731R>X	Substitution	Nonsense	26%
MM12T	FAT4	FAT tumor suppressor homolog 4 (Drosophila)	CCDS3732.3	chr4_126355475-126355475_C_T	2365A>V	Substitution	Nonsynonymous coding	27%
MM12T	FAT4	FAT tumor suppressor homolog 4 (Drosophila)	CCDS3732.3	chr4_126398471-126398471_C_T	4152A>V	Substitution	Nonsynonymous coding	17%
MM12T	FBF1	Fas (TNFRSF6) binding factor 1	CCDS45779.1	chr17_73914084-73914084_C_T	757V>M	Substitution	Nonsynonymous coding	39%
MM12T	FBLL1	fibrillarin-like 1	ENST00000453461	chr5_167957276-167957276_G_A	256R>H	Substitution	Nonsynonymous coding	27%
MM12T	FBLN1	fibulin 1	CCDS14067.1	chr22_45943088-45943088_C_T	ISV+4>	Substitution	Splice site donor	19%
MM12T	FBLN2	fibulin 2	CCDS46761.1	chr3_13659779-13659779_C_T	645R>C	Substitution	Nonsynonymous coding	27%
MM12T	FBLN5	fibulin 5	CCDS9898.1	chr14_92343833-92343833_G_A	395R>W	Substitution	Nonsynonymous coding	11%
MM12T	FBN3	fibrillin 3	CCDS12196.1	chr19_8130912-8130912_C_T	2774R>Q	Substitution	Nonsynonymous coding	40%
MM12T	FBN3	fibrillin 3	CCDS12196.1	chr19_8186255-8186255_C_T	1033R>H	Substitution	Nonsynonymous coding	11%
MM12T	FBN3	fibrillin 3	CCDS12196.1	chr19_8191659-8191659_C_T	785G>D	Substitution	Nonsynonymous coding	36%
MM12T	FBN3	fibrillin 3	CCDS12196.1	chr19_8209800-8209800_C_T	168A>T	Substitution	Nonsynonymous coding	16%
MM12T	FBN3	fibrillin 3	CCDS12196.1	chr19_8162299-8162299_C_T	ISV-1>	Substitution	Splice site acceptor	34%
MM12T	FBXL14	F-box and leucine-rich repeat protein 14	CCDS8509.1	chr12_1702439-1702439_T_C	265N>S	Substitution	Nonsynonymous coding	31%
MM12T	FBXL16	F-box and leucine-rich repeat protein 16	CCDS10421.1	chr16_746993-746993_G_A	138T>M	Substitution	Nonsynonymous coding	31%
MM12T	FBXL18	F-box and leucine-rich repeat protein 18	CCDS43546.1	chr7_5540653-5540653_C_T	416R>H	Substitution	Nonsynonymous coding	10%
MM12T	FBXL18	F-box and leucine-rich repeat protein 18	CCDS43546.1	chr7_5540744-5540744_G_A	386R>C	Substitution	Nonsynonymous coding	35%
MM12T	FBXL21	F-box and leucine-rich repeat protein 21 (gene/pseudogene)	NM_012159	chr5_135276839-135276839_C_T	262R>X	Substitution	Nonsense	33%
MM12T	FBXL4	F-box and leucine-rich repeat protein 4	CCDS5041.1	chr6_99353417-99353417_C_T	330A>T	Substitution	Nonsynonymous coding	20%
MM12T	FBXL7	F-box and leucine-rich repeat protein 7	NM_012304	chr5_15928283-15928283_G_A	138A>T	Substitution	Nonsynonymous coding	30%
MM12T	FBXO17	F-box protein 17	CCDS12526.1	chr19_39435697-39435697_C_T	202R>H	Substitution	Nonsynonymous coding	29%
MM12T	FBXO2	F-box protein 2	CCDS130.1	chr1_11708887-11708887_T_C	ISV-2>	Substitution	Splice site acceptor	31%
MM12T	FBXO22	F-box protein 22	CCDS10287.1	chr15_76225331-76225331_G_A	367R>Q	Substitution	Nonsynonymous coding	13%
MM12T	FBXO32	F-box protein 32	CCDS6345.1	chr8_124516948-124516948_C_T	292D>N	Substitution	Nonsynonymous coding	25%
MM12T	FBXO40	F-box protein 40	CCDS33835.1	chr3_121341484-121341484_C_A	403A>D	Substitution	Nonsynonymous coding	29%
MM12T	FBXO45	F-box protein 45	CCDS46985.1	chr3_196311074-196311074_T_C	249F>S	Substitution	Nonsynonymous coding	43%
MM12T	FBXO7	F-box protein 7	CCDS13907.1	chr22_32891486-32891486_C_A	384T>N	Substitution	Nonsynonymous coding	28%
MM12T	FBXW2	F-box and WD repeat domain containing 2	CCDS43872.1	chr9_123533659-123533659_T_C	348Y>C	Substitution	Nonsynonymous coding	24%

MM12T	FBXW5	F-box and WD repeat domain containing 5	CCDS7014.1	chr9_139835799-139835799_C_T	454R>Q	Substitution	Nonsynonymous coding	13%
MM12T	FBXW9	F-box and WD repeat domain containing 9	ENST00000380339	chr19_12800974-12800974_C_	NA	Deletion	Frameshift	37%
MM12T	FCGBP	Fc fragment of IgG binding protein	CCDS12546.1	chr19_40424435-40424435_C_T	590D>N	Substitution	Nonsynonymous coding	32%
MM12T	FCHSD2	FCH and double SH3 domains 2	CCDS8218.2	chr11_72551927-72551927_G_A	712R>X	Substitution	Nonsense	11%
MM12T	FCRL1	Fc receptor-like 1	CCDS1170.1	chr1_157771332-157771332_C_A	308G>X	Substitution	Nonsense	25%
MM12T	FCRL2	Fc receptor-like 2	CCDS1168.1	chr1_157738374-157738374_G_A	238S>F	Substitution	Nonsynonymous coding	30%
MM12T	FCRL5	Fc receptor-like 5	CCDS1165.1	chr1_157508981-157508981_C_T	433A>T	Substitution	Nonsynonymous coding	14%
MM12T	FCRLB	Fc receptor-like B	CCDS30927.1	chr1_161693367-161693367_G_A	88R>Q	Substitution	Nonsynonymous coding	15%
MM12T	FCRLB	Fc receptor-like B	CCDS30927.1	chr1_161695781-161695781_G_A	160A>T	Substitution	Nonsynonymous coding	11%
MM12T	FDX1L	ferredoxin 1-like	CCDS32905.1	chr19_10426670-10426670_C_T	1M>I	Substitution	Nonsynonymous coding	20%
MM12T	FDXR	ferredoxin reductase	CCDS11707.1	chr17_72861071-72861071_C_T	204A>T	Substitution	Nonsynonymous coding	28%
MM12T	FER	fer (fps/fes related) tyrosine kinase	CCDS4098.1	chr5_108203589-108203589_T_A	201D>E	Substitution	Nonsynonymous coding	33%
MM12T	FER1L6	fer-1-like 6 (C. elegans)	CCDS43767.1	chr8_125078699-125078699_A_G	1196K>E	Substitution	Nonsynonymous coding	17%
MM12T	FERMT2	fermitin family member 2	CCDS45107.1	chr14_53348130-53348130_G_T	195A>D	Substitution	Nonsynonymous coding	36%
MM12T	FEV	FEV (ETS oncogene family)	CCDS2428.1	chr2_219846828-219846828_T_G	93K>T	Substitution	Nonsynonymous coding	33%
MM12T	FFAR1	free fatty acid receptor 1	CCDS12458.1	chr19_35842776-35842776_G_A	108A>T	Substitution	Nonsynonymous coding	16%
MM12T	FGA	fibrinogen alpha chain	CCDS3787.1	chr4_155506995-155506995_A_T	529F>Y	Substitution	Nonsynonymous coding	36%
MM12T	FGD2	FYVE, RhoGEF and PH domain containing 2	CCDS4829.1	chr6_36990120-36990120_C_T	478R>C	Substitution	Nonsynonymous coding	50%
MM12T	FGD2	FYVE, RhoGEF and PH domain containing 2	ENST00000394459	chr6_36990885-36990885_G_A	358G>D	Substitution	Nonsynonymous coding	19%
MM12T	FGD2	FYVE, RhoGEF and PH domain containing 2	CCDS4829.1	chr6_36995328-36995328_C_T	577L>F	Substitution	Nonsynonymous coding	11%
MM12T	FGD6	FYVE, RhoGEF and PH domain containing 6	CCDS31878.1	chr12_95602839-95602839_T_C	741T>A	Substitution	Nonsynonymous coding	29%
MM12T	FGF11	fibroblast growth factor 11	CCDS11105.1	chr17_7344793-7344793_C_A	66P>H	Substitution	Nonsynonymous coding	22%
MM12T	FGF12	fibroblast growth factor 12	CCDS3301.1	chr3_191888348-191888348_C_T	171R>Q	Substitution	Nonsynonymous coding	11%
MM12T	FGF13	fibroblast growth factor 13	CCDS14665.1	chrX_137717759-137717759_T_C	154T>A	Substitution	Nonsynonymous coding	12%
MM12T	FGF21	fibroblast growth factor 21	CCDS12734.1	chr19_49261307-49261307_C_T	154R>W	Substitution	Nonsynonymous coding	11%
MM12T	FGF3	fibroblast growth factor 3	CCDS8195.1	chr11_69631089-69631089_G_A	108S>L	Substitution	Nonsynonymous coding	12%
MM12T	FGF4	fibroblast growth factor 4	CCDS8194.1	chr11_69588123-69588123_C_T	192R>Q	Substitution	Nonsynonymous coding	38%
MM12T	FGFR3	fibroblast growth factor receptor 3	CCDS3353.1	chr4_1805510-1805510_C_T	341A>V	Substitution	Nonsynonymous coding	27%
MM12T	FHL2	four and a half LIM domains 2	CCDS2070.1	chr2_105984111-105984111_C_A	139K>N	Substitution	Nonsynonymous coding	35%
MM12T	FHOD3	formin homology 2 domain containing 3	CCDS32816.1	chr18_34261459-34261459__AG	NA	Insertion	Frameshift	27%
MM12T	FIBCD1	fibrinogen C domain containing 1	CCDS6937.1	chr9_133799153-133799153_C_T	276R>H	Substitution	Nonsynonymous coding	31%
MM12T	FIG4	FIG4 homolog, SAC1 lipid phosphatase domain containing (S. cerevisiae)	CCDS5078.1	chr6_110112716-110112716_C_A	773S>Y	Substitution	Nonsynonymous coding	25%

MM12T	FIGN	fidgetin	CCDS2221.2	chr2_164466775-164466775_G_T	523L>I	Substitution	Nonsynonymous coding	36%
MM12T	FIGN	fidgetin	CCDS2221.2	chr2_164467326-164467326_C_T	339G>D	Substitution	Nonsynonymous coding	19%
MM12T	FIGN	fidgetin	CCDS2221.2	chr2_164467354-164467354_C_T	330D>N	Substitution	Nonsynonymous coding	15%
MM12T	FIS1	fission 1 (mitochondrial outer membrane) homolog (S. cerevisiae)	CCDS43626.1	chr7_100883109-100883109_G_T	146A>D	Substitution	Nonsynonymous coding	40%
MM12T	FIZ1	FLT3-interacting zinc finger 1	CCDS12928.1	chr19_56104078-56104078_C_T	410G>D	Substitution	Nonsynonymous coding	28%
MM12T	FKBP10	FK506 binding protein 10, 65 kDa	CCDS11409.1	chr17_39975915-39975915_G_A	351E>K	Substitution	Nonsynonymous coding	18%
MM12T	FKBP5	FK506 binding protein 5	CCDS4808.1	chr6_35547913-35547913_G_A	309S>L	Substitution	Nonsynonymous coding	11%
MM12T	FKTN	fukutin	CCDS6766.1	chr9_108363547-108363547_G_A	96G>D	Substitution	Nonsynonymous coding	34%
MM12T	FLAD1	FAD1 flavin adenine dinucleotide synthetase homolog (S. cerevisiae)	CCDS1078.1	chr1_154962651-154962651_C_T	428P>S	Substitution	Nonsynonymous coding	11%
MM12T	FLCN	folliculin	CCDS32579.1	chr17_17125882-17125882_C_T	238A>T	Substitution	Nonsynonymous coding	36%
MM12T	FLCN	folliculin	CCDS32579.1	chr17_17131403-17131403_G_A	17R>C	Substitution	Nonsynonymous coding	34%
MM12T	FLG2	filaggrin family member 2	CCDS30861.1	chr1_152325097-152325097_C_T	1722G>E	Substitution	Nonsynonymous coding	14%
MM12T	FLII	flightless I homolog (Drosophila)	CCDS11192.1	chr17_18154755-18154755_G_A	474R>W	Substitution	Nonsynonymous coding	35%
MM12T	FLNA	filamin A, alpha	CCDS48194.1	chrX_153578116-153578116_C_T	2485V>I	Substitution	Nonsynonymous coding	32%
MM12T	FLNA	filamin A, alpha	CCDS48194.1	chrX_153582400-153582400_G_A	1857Q>X	Substitution	Nonsense	36%
MM12T	FLNB	filamin B, beta	CCDS2885.1	chr3_57994398-57994398_G_A	36R>H	Substitution	Nonsynonymous coding	15%
MM12T	FLNB	filamin B, beta	CCDS2885.1	chr3_58155457-58155457_G_A	2520A>T	Substitution	Nonsynonymous coding	38%
MM12T	FLNC	filamin C, gamma	CCDS43644.1	chr7_128488073-128488073_G_A	1511G>R	Substitution	Nonsynonymous coding	33%
MM12T	FLNC	filamin C, gamma	CCDS43644.1	chr7_128493077-128493077_G_A	2067R>H	Substitution	Nonsynonymous coding	15%
MM12T	FLT1	fms-related tyrosine kinase 1	CCDS9330.1	chr13_28877407-28877407_C_T	1305R>H	Substitution	Nonsynonymous coding	29%
MM12T	FLT1	fms-related tyrosine kinase 1	CCDS9330.1	chr13_29001363-29001363_A_G	457Y>H	Substitution	Nonsynonymous coding	30%
MM12T	FLVCR1	feline leukemia virus subgroup C cellular receptor 1	CCDS1510.1	chr1_213032467-213032467_G_A	225A>T	Substitution	Nonsynonymous coding	26%
MM12T	FLVCR2	feline leukemia virus subgroup C cellular receptor family, member 2	CCDS9844.1	chr14_76112789-76112789_A_G	519T>A	Substitution	Nonsynonymous coding	31%
MM12T	FMN2	formin 2	CCDS31069.2	chr1_240255559-240255559_G_	NA	Deletion	Frameshift	10%
MM12T	FMN2	formin 2	CCDS31069.2	chr1_240255562-240255565_GGGA_	NA	Deletion	Frameshift	49%
MM12T	FMNL3	formin-like 3	CCDS44874.1	chr12_50042017-50042017_C_A	879G>C	Substitution	Nonsynonymous coding	30%
MM12T	FMO3	flavin containing monooxygenase 3	CCDS1292.1	chr1_171076877-171076877_C_T	128T>I	Substitution	Nonsynonymous coding	29%
MM12T	FMO3	flavin containing monooxygenase 3	CCDS1292.1	chr1_171085413-171085413_C_T	417R>C	Substitution	Nonsynonymous coding	28%
MM12T	FMO6P	flavin containing monooxygenase 6 pseudogene	ENST00000236166	chr1_171116837-171116837_G_T	186R>M	Substitution	Nonsynonymous coding	19%
MM12T	FNBP1	formin binding protein 1	CCDS48040.1	chr9_132665275-132665275_G_T	434A>D	Substitution	Nonsynonymous coding	18%
MM12T	FNBP1	formin binding protein 1	CCDS48040.1	chr9_132686176-132686176_C_T	373E>K	Substitution	Nonsynonymous coding	16%
MM12T	FNBP1	formin binding protein 1	CCDS48040.1	chr9_132741618-132741618_G_A	59S>L	Substitution	Nonsynonymous coding	45%

MM12T	FNDC1	fibronectin type III domain containing 1	CCDS47512.1	chr6_159654279-159654279_G_A	912S>N	Substitution	Nonsynonymous coding	48%
MM12T	FNDC3A	fibronectin type III domain containing 3A	CCDS41886.1	chr13_49580426-49580426_G_A	ISV+1>	Substitution	Splice site donor	47%
MM12T	FNDC3B	fibronectin type III domain containing 3B	CCDS3217.1	chr3_172060852-172060852_C_T	675R>X	Substitution	Nonsense	22%
MM12T	FOLR3	folate receptor 3 (gamma)	ENST00000456237	chr11_71850829-71850829_C_T	273A>V	Substitution	Nonsynonymous coding	15%
MM12T	FOXD2	forkhead box D2	CCDS30708.1	chr1_47904985-47904985_C_T	393T>M	Substitution	Nonsynonymous coding	31%
MM12T	FOXH1	forkhead box H1	CCDS6428.1	chr8_145699929-145699929_G_A	264R>W	Substitution	Nonsynonymous coding	37%
MM12T	FOXK2	forkhead box K2	CCDS11813.1	chr17_80521428-80521428_C_T	ISV+4>	Substitution	Splice site donor	36%
MM12T	FOXN4	forkhead box N4	CCDS9126.2	chr12_109719446-109719446_G_T	354L>M	Substitution	Nonsynonymous coding	28%
MM12T	FOXO6	forkhead box O6	ENST00000372591	chr1_41847547-41847547_C_A	225P>H	Substitution	Nonsynonymous coding	15%
MM12T	FOXP4	forkhead box P4	CCDS34447.1	chr6_41565529-41565529_C_T	579A>V	Substitution	Nonsynonymous coding	20%
MM12T	FOXRED2	FAD-dependent oxidoreductase domain containing 2	CCDS13929.1	chr22_36892046-36892046_G_A	531P>L	Substitution	Nonsynonymous coding	27%
MM12T	FOXS1	forkhead box S1	CCDS13192.1	chr20_30433011-30433011_C_T	112R>H	Substitution	Nonsynonymous coding	28%
MM12T	FOXS1	forkhead box S1	CCDS13192.1	chr20_30433234-30433234_G_A	38R>W	Substitution	Nonsynonymous coding	10%
MM12T	FPR2	formyl peptide receptor 2	CCDS12840.1	chr19_52272465-52272465_G_A	185G>D	Substitution	Nonsynonymous coding	15%
MM12T	FPR3	formyl peptide receptor 3	CCDS12841.1	chr19_52327266-52327266_G_A	89E>K	Substitution	Nonsynonymous coding	15%
MM12T	FREM3	FRAS1 related extracellular matrix 3	NM_001168235	chr4_144618638-144618638_G_T	1064P>H	Substitution	Nonsynonymous coding	19%
MM12T	FRMD1	FERM domain containing 1	CCDS5306.1	chr6_168459818-168459818_C_T	465E>K	Substitution	Nonsynonymous coding	22%
MM12T	FRMD4B	FERM domain containing 4B	CCDS46863.1	chr3_69273803-69273803_G_A	207A>V	Substitution	Nonsynonymous coding	14%
MM12T	FRMPD1	FERM and PDZ domain containing 1	CCDS6612.1	chr9_37740128-37740128_G_A	535V>I	Substitution	Nonsynonymous coding	17%
MM12T	FRMPD1	FERM and PDZ domain containing 1	CCDS6612.1	chr9_37744832-37744832_G_A	935D>N	Substitution	Nonsynonymous coding	11%
MM12T	FRMPD3	FERM and PDZ domain containing 3	ENST00000276185	chrX_106840986-106840986_G_A	659R>H	Substitution	Nonsynonymous coding	15%
MM12T	FRMPD4	FERM and PDZ domain containing 4	CCDS35201.1	chrX_12734656-12734656_C_T	693A>V	Substitution	Nonsynonymous coding	15%
MM12T	FRMPD4	FERM and PDZ domain containing 4	ENST00000429478	chrX_12739340-12739340_C_T	1544A>V	Substitution	Nonsynonymous coding	10%
MM12T	FRMPD4	FERM and PDZ domain containing 4	CCDS35201.1	chrX_12724996-12724996_C_T	417R>X	Substitution	Nonsense	11%
MM12T	FRS2	fibroblast growth factor receptor substrate 2	CCDS41809.1	chr12_69962862-69962862_C_T	18R>W	Substitution	Nonsynonymous coding	30%
MM12T	FRS3	fibroblast growth factor receptor substrate 3	CCDS4860.1	chr6_41743271-41743271_G_T	47L>M	Substitution	Nonsynonymous coding	13%
MM12T	FRY	furry homolog (Drosophila)	CCDS41875.1	chr13_32709043-32709043_A_T	296E>D	Substitution	Nonsynonymous coding	47%
MM12T	FRYL	FRY-like	CCDS43227.1	chr4_48548124-48548124_C_T	1747D>N	Substitution	Nonsynonymous coding	21%
MM12T	FRYL	FRY-like	CCDS43227.1	chr4_48611030-48611030_C_A	182E>D	Substitution	Nonsynonymous coding	30%
MM12T	FSCB	fibrous sheath CABYR binding protein	CCDS9679.1	chr14_44975361-44975361_G_A	277A>V	Substitution	Nonsynonymous coding	19%
MM12T	FSD1	fibronectin type III and SPRY domain containing 1	CCDS12127.1	chr19_4323108-4323108_G_A	389A>T	Substitution	Nonsynonymous coding	37%
MM12T	FSHR	follicle stimulating hormone receptor	CCDS1843.1	chr2_49190291-49190291_G_A	557R>W	Substitution	Nonsynonymous coding	30%

MM12T	FSIP2	fibrous sheath interacting protein 2	ENST00000424728	chr2_186653913-186653913_G_A	684D>N	Substitution	Nonsynonymous coding	37%
MM12T	FSTL1	follistatin-like 1	CCDS2998.1	chr3_120123733-120123733_G_A	183T>M	Substitution	Nonsynonymous coding	30%
MM12T	FSTL5	follistatin-like 5	CCDS3802.1	chr4_162307546-162307546_T_G	633T>P	Substitution	Nonsynonymous coding	31%
MM12T	FTSJ2	FtsJ RNA methyltransferase homolog 2 (E. coli)	CCDS5328.1	chr7_2279133-2279133_C_T	73G>D	Substitution	Nonsynonymous coding	17%
MM12T	FUBP1	far upstream element (FUSE) binding protein 1	CCDS683.1	chr1_78426092-78426092_C_T	478G>E	Substitution	Nonsynonymous coding	29%
MM12T	FUOM	fucose mutarotase	CCDS44499.1	chr10_135169276-135169276_G_A	122R>W	Substitution	Nonsynonymous coding	32%
MM12T	FURIN	furin (paired basic amino acid cleaving enzyme)	CCDS10364.1	chr15_91421502-91421502_G_A	270A>T	Substitution	Nonsynonymous coding	29%
MM12T	FUS	fused in sarcoma	CCDS10707.1	chr16_31201719-31201719_C_T	431P>L	Substitution	Nonsynonymous coding	60%
MM12T	FUT9	fucosyltransferase 9 (alpha (1,3) fucosyltransferase)	CCDS5033.1	chr6_96651471-96651471_G_A	147G>D	Substitution	Nonsynonymous coding	10%
MM12T	FUZ	fuzzy homolog (Drosophila)	CCDS12781.1	chr19_50312688-50312688_G_A	213P>S	Substitution	Nonsynonymous coding	30%
MM12T	FXR1	fragile X mental retardation, autosomal homolog 1	CCDS3238.1	chr3_180666228-180666228_A_	NA	Deletion	Frameshift	22%
MM12T	FYCO1	FYVE and coiled-coil domain containing 1	CCDS2734.1	chr3_46003775-46003775_G_T	1127L>M	Substitution	Nonsynonymous coding	10%
MM12T	FYCO1	FYVE and coiled-coil domain containing 1	CCDS2734.1	chr3_46010057-46010057_C_T	257D>N	Substitution	Nonsynonymous coding	30%
MM12T	FZD2	frizzled family receptor 2	CCDS11484.1	chr17_42636639-42636639_C_T	528T>M	Substitution	Nonsynonymous coding	20%
MM12T	FZD3	frizzled family receptor 3	CCDS6069.1	chr8_28385058-28385058_C_T	261R>X	Substitution	Nonsense	28%
MM12T	FZD4	frizzled family receptor 4	CCDS8279.1	chr11_86663448-86663448_C_T	117C>Y	Substitution	Nonsynonymous coding	11%
MM12T	FZD7	frizzled family receptor 7	CCDS2351.1	chr2_202901042-202901042_C_T	558R>C	Substitution	Nonsynonymous coding	31%
MM12T	FZD9	frizzled family receptor 9	CCDS5548.1	chr7_72849295-72849295_C_T	320L>F	Substitution	Nonsynonymous coding	36%
MM12T	FZR1	fizzy/cell division cycle 20 related 1 (Drosophila)	CCDS45916.1	chr19_3523009-3523009_C_T	8R>C	Substitution	Nonsynonymous coding	26%
MM12T	G6PC	glucose-6-phosphatase, catalytic subunit	CCDS11446.1	chr17_41063381-41063381_G_A	338V>I	Substitution	Nonsynonymous coding	40%
MM12T	GABBR1	gamma-aminobutyric acid (GABA) B receptor, 1	CCDS4663.1	chr6_29571468-29571468_C_T	913R>H	Substitution	Nonsynonymous coding	15%
MM12T	GABBR1	gamma-aminobutyric acid (GABA) B receptor, 1	CCDS4663.1	chr6_29581117-29581117_C_T	490R>H	Substitution	Nonsynonymous coding	25%
MM12T	GABBR1	gamma-aminobutyric acid (GABA) B receptor, 1	CCDS4663.1	chr6_29581168-29581168_G_A	473A>V	Substitution	Nonsynonymous coding	42%
MM12T	GABBR2	gamma-aminobutyric acid (GABA) B receptor, 2	CCDS6736.1	chr9_101052916-101052916_G_A	926R>C	Substitution	Nonsynonymous coding	36%
MM12T	GABPA	GA binding protein transcription factor, alpha subunit 60kDa	CCDS13575.1	chr21_27136614-27136614_C_T	299A>V	Substitution	Nonsynonymous coding	16%
MM12T	GABRA5	gamma-aminobutyric acid (GABA) A receptor, alpha 5	CCDS45194.1	chr15_27182373-27182373_G_A	208G>S	Substitution	Nonsynonymous coding	28%
MM12T	GABRG2	gamma-aminobutyric acid (GABA) A receptor, gamma 2	CCDS47333.1	chr5_161580117-161580117_C_T	431R>C	Substitution	Nonsynonymous coding	14%
MM12T	GABRP	gamma-aminobutyric acid (GABA) A receptor, pi	CCDS4375.1	chr5_170216215-170216215_G_A	46A>T	Substitution	Nonsynonymous coding	17%
MM12T	GABRQ	gamma-aminobutyric acid (GABA) A receptor, theta	CCDS14707.1	chrX_151818250-151818250_G_A	219G>E	Substitution	Nonsynonymous coding	31%
MM12T	GABRQ	gamma-aminobutyric acid (GABA) A receptor, theta	CCDS14707.1	chrX_151806666-151806666_C_T	4R>X	Substitution	Nonsense	43%
MM12T	GAL3ST1	galactose-3-O-sulfotransferase 1	CCDS13879.1	chr22_30951146-30951146_C_T	356A>T	Substitution	Nonsynonymous coding	10%
MM12T	GAL3ST1	galactose-3-O-sulfotransferase 1	CCDS13879.1	chr22_30951193-30951193_C_T	340R>H	Substitution	Nonsynonymous coding	12%

MM12T	GAL3ST3	galactose-3-O-sulfotransferase 3	CCDS8128.1	chr11_65810697-65810697_C_T	193A>T	Substitution	Nonsynonymous coding	30%
MM12T	GALNS	galactosamine (N-acetyl)-6-sulfate sulfatase	CCDS10970.1	chr16_88909176-88909176_C_T	61R>Q	Substitution	Nonsynonymous coding	28%
MM12T	GALNS	galactosamine (N-acetyl)-6-sulfate sulfatase	CCDS10970.1	chr16_88908359-88908359_C_A	89G>X	Substitution	Nonsense	17%
MM12T	GALNT13	UDP-N-acetyl-alpha-D-galactosamine:polypeptide N-	CCDS2199.1	chr2_155158010-155158010_G_A	355G>D	Substitution	Nonsynonymous coding	11%
MM12T	GALNT15	UDP-N-acetyl-alpha-D-galactosamine:polypeptide N-	CCDS33711.1	chr3_16268882-16268882_C_T	599L>F	Substitution	Nonsynonymous coding	11%
MM12T	GALNT5	UDP-N-acetyl-alpha-D-galactosamine:polypeptide N-	CCDS2203.1	chr2_158114640-158114640_G_A	16A>T	Substitution	Nonsynonymous coding	12%
MM12T	GALNTL5	UDP-N-acetyl-alpha-D-galactosamine:polypeptide N-	CCDS5929.1	chr7_151704991-151704991_G_A	330D>N	Substitution	Nonsynonymous coding	33%
MM12T	GALR1	galanin receptor 1	CCDS12012.1	chr18_74962541-74962541_G_A	13A>T	Substitution	Nonsynonymous coding	35%
MM12T	GALR1	galanin receptor 1	CCDS12012.1	chr18_74962637-74962637_G_A	45A>T	Substitution	Nonsynonymous coding	24%
MM12T	GAN	gigaxonin	CCDS10935.1	chr16_81391505-81391505_G_A	314M>I	Substitution	Nonsynonymous coding	14%
MM12T	GANAB	glucosidase, alpha; neutral AB	CCDS41656.1	chr11_62393913-62393913_C_T	862A>T	Substitution	Nonsynonymous coding	33%
MM12T	GAPVD1	GTPase activating protein and VPS9 domains 1	CCDS35138.1	chr9_128064582-128064582_G_A	169R>Q	Substitution	Nonsynonymous coding	34%
MM12T	GAPVD1	GTPase activating protein and VPS9 domains 1	CCDS35138.1	chr9_128094231-128094231_C_T	734R>C	Substitution	Nonsynonymous coding	26%
MM12T	GAPVD1	GTPase activating protein and VPS9 domains 1	CCDS35138.1	chr9_128118072-128118072_G_A	1330V>M	Substitution	Nonsynonymous coding	21%
MM12T	GARS	glycyl-tRNA synthetase	CCDS43564.1	chr7_30673444-30673444_G_A	730E>K	Substitution	Nonsynonymous coding	24%
MM12T	GATA1	GATA binding protein 1 (globin transcription factor 1)	CCDS14305.1	chrX_48652263-48652263_A_C	312K>Q	Substitution	Nonsynonymous coding	31%
MM12T	GATA2	GATA binding protein 2	CCDS3049.1	chr3_128204954-128204954_C_T	163A>T	Substitution	Nonsynonymous coding	21%
MM12T	GATAD2A	GATA zinc finger domain containing 2A	CCDS12402.2	chr19_19603488-19603488_G_T	167Q>H	Substitution	Nonsynonymous coding	26%
MM12T	GATM	glycine amidinotransferase (L-arginine:glycine amidinotransferase)	CCDS10122.1	chr15_45668831-45668831_C_T	86A>T	Substitution	Nonsynonymous coding	28%
MM12T	GBF1	golgi brefeldin A resistant guanine nucleotide exchange factor 1	CCDS7533.1	chr10_104126873-104126873_A_G	821D>G	Substitution	Nonsynonymous coding	35%
MM12T	GBF1	golgi brefeldin A resistant guanine nucleotide exchange factor 1	CCDS7533.1	chr10_104128983-104128983_C_T	996P>S	Substitution	Nonsynonymous coding	10%
MM12T	GCAT	glycine C-acetyltransferase	CCDS13957.1	chr22_38211217-38211217_G_A	221A>T	Substitution	Nonsynonymous coding	25%
MM12T	GCK	glucokinase (hexokinase 4)	CCDS5480.1	chr7_44198674-44198674_G_T	16L>M	Substitution	Nonsynonymous coding	32%
MM12T	GCN1L1	GCN1 general control of amino-acid synthesis 1-like 1 (yeast)	CCDS41847.1	chr12_120591147-120591147_C_T	1311S>N	Substitution	Nonsynonymous coding	28%
MM12T	GCN1L1	GCN1 general control of amino-acid synthesis 1-like 1 (yeast)	CCDS41847.1	chr12_120594264-120594264_C_T	ISV+1>	Substitution	Splice site donor	39%
MM12T	GDAP1	ganglioside induced differentiation associated protein 1	CCDS34911.1	chr8_75263615-75263615_G_A	75G>E	Substitution	Nonsynonymous coding	15%
MM12T	GDF15	growth differentiation factor 15	CCDS12376.1	chr19_18499506-18499506_C_A	230L>M	Substitution	Nonsynonymous coding	43%
MM12T	GDF5	growth differentiation factor 5	CCDS13254.1	chr20_34022081-34022081_G_A	378R>X	Substitution	Nonsense	16%
MM12T	GDPD4	glycerophosphodiester phosphodiesterase domain containing 4	ENST00000376217	chr11_76928143-76928143_G_A	571P>S	Substitution	Nonsynonymous coding	28%
MM12T	GDPD4	glycerophosphodiester phosphodiesterase domain containing 4	CCDS8249.1	chr11_76969574-76969574_G_A	241P>S	Substitution	Nonsynonymous coding	15%
MM12T	GDPD5	glycerophosphodiester phosphodiesterase domain containing 5	CCDS8238.1	chr11_75168791-75168791_C_T	88V>I	Substitution	Nonsynonymous coding	27%
MM12T	GEMIN4	gem (nuclear organelle) associated protein 4	CCDS45559.1	chr17_650898-650898_G_T	129L>M	Substitution	Nonsynonymous coding	14%

MM12T	GEMIN5	gem (nuclear organelle) associated protein 5	CCDS4330.1	chr5_154271012-154271012_G_A	1351R>X	Substitution	Nonsense	36%
MM12T	gene110258	-	CCDS47277.1	chr5_139266946-139266946_C_T	284R>H	Substitution	Nonsynonymous coding	10%
MM12T	gene111969	-	CCDS47925.1	chr8_143762846-143762846_C_T	52R>C	Substitution	Nonsynonymous coding	14%
MM12T	gene111969	-	CCDS47925.1	chr8_143763372-143763372_G_A	65G>D	Substitution	Nonsynonymous coding	15%
MM12T	gene112101	-	CCDS47736.1	chr7_144098144-144098144_C_T	280R>H	Substitution	Nonsynonymous coding	32%
MM12T	gene112101	-	CCDS47736.1	chr7_144099047-144099047_C_T	ISV-4>	Substitution	Splice site acceptor	31%
MM12T	gene113070	-	CCDS47498.1	chr6_146978052-146978052_C_A	183A>D	Substitution	Nonsynonymous coding	23%
MM12T	gene115326	-	CCDS48188.1	chrX_152613366-152613366_G_A	408S>N	Substitution	Nonsynonymous coding	14%
MM12T	gene119468	-	CCDS47160.1	chr4_165981533-165981533_G_A	412A>T	Substitution	Nonsynonymous coding	10%
MM12T	gene119844	-	CCDS47516.1	chr6_168265307-168265307_G_A	61R>Q	Substitution	Nonsynonymous coding	12%
MM12T	gene119844	-	CCDS47516.1	chr6_168297651-168297651_A_G	438Q>R	Substitution	Nonsynonymous coding	50%
MM12T	gene119844	-	CCDS47516.1	chr6_168315863-168315863_C_T	764T>M	Substitution	Nonsynonymous coding	38%
MM12T	gene119844	-	CCDS47516.1	chr6_168276118-168276118_C_T	227Q>X	Substitution	Nonsense	39%
MM12T	gene13617	-	CCDS47950.1	chr9_5968798-5968798_C_T	478G>D	Substitution	Nonsynonymous coding	23%
MM12T	gene18417	-	CCDS47541.1	chr7_11091247-11091247_C_T	774S>L	Substitution	Nonsynonymous coding	46%
MM12T	gene20935	-	CCDS46766.1	chr3_14537983-14537983_C_T	1020R>Q	Substitution	Nonsynonymous coding	29%
MM12T	gene20935	-	CCDS46766.1	chr3_14551344-14551344_G_A	786R>C	Substitution	Nonsynonymous coding	15%
MM12T	gene25924	-	CCDS44078.1	chr1_20645133-20645133_C_T	254R>C	Substitution	Nonsynonymous coding	11%
MM12T	gene37297	-	CCDS45094.1	chr14_31841108-31841108_C_A	330K>N	Substitution	Nonsynonymous coding	21%
MM12T	gene37297	-	CCDS45094.1	chr14_31856490-31856490_G_A	49P>L	Substitution	Nonsynonymous coding	21%
MM12T	gene37652	-	CCDS47407.1	chr6_32035444-32035444_C_T	2180V>I	Substitution	Nonsynonymous coding	21%
MM12T	gene42698	-	CCDS46056.1	chr19_36256027-36256027_C_T	240S>L	Substitution	Nonsynonymous coding	14%
MM12T	gene42698	-	CCDS46056.1	chr19_36259082-36259082_C_T	416R>X	Substitution	Nonsense	29%
MM12T	gene49600	-	CCDS45242.1	chr15_42276814-42276814_G_T	707L>M	Substitution	Nonsynonymous coding	33%
MM12T	gene57233	-	CCDS46820.1	chr3_47950975-47950975_A_G	253S>P	Substitution	Nonsynonymous coding	29%
MM12T	gene66631	-	CCDS47441.1	chr6_56457095-56457095_G_T	2144A>D	Substitution	Nonsynonymous coding	11%
MM12T	gene66631	-	CCDS47441.1	chr6_56765370-56765370_G_A	89A>V	Substitution	Nonsynonymous coding	22%
MM12T	gene68399	-	CCDS45112.1	chr14_58030900-58030900_G_A	507R>W	Substitution	Nonsynonymous coding	29%
MM12T	gene68399	-	CCDS45112.1	chr14_58031047-58031047_C_T	458A>T	Substitution	Nonsynonymous coding	26%
MM12T	gene84609	-	CCDS44168.1	chr1_85016132-85016132_G_T	403Q>H	Substitution	Nonsynonymous coding	10%
MM12T	gene86832	-	CCDS45551.1	chr16_89723994-89723994_C_T	1M>I	Substitution	Nonsynonymous coding	30%
MM12T	gene87951	-	CCDS44706.1	chr11_92498091-92498091_G_A	1344R>H	Substitution	Nonsynonymous coding	56%

MM12T	gene87951	-	CCDS44706.1	chr11_92531331-92531331_C_T	1718P>S	Substitution	Nonsynonymous coding	29%
MM12T	gene87951	-	CCDS44706.1	chr11_92616148-92616148_C_T	4176R>C	Substitution	Nonsynonymous coding	32%
MM12T	gene98833	-	CCDS44978.1	chr12_112708248-112708248_G_A	221T>M	Substitution	Nonsynonymous coding	11%
MM12T	GFAP	glial fibrillary acidic protein	CCDS11491.1	chr17_42989078-42989078_G_A	290Q>X	Substitution	Nonsense	36%
MM12T	GFPT2	glutamine-fructose-6-phosphate transaminase 2	CCDS43411.1	chr5_179743820-179743820_G_A	366R>X	Substitution	Nonsense	28%
MM12T	GFRA1	GDNF family receptor alpha 1	CCDS44481.1	chr10_118030591-118030591_C_T	26R>H	Substitution	Nonsynonymous coding	29%
MM12T	GGA3	golgi-associated, gamma adaptin ear containing, ARF binding protein 3	CCDS11717.1	chr17_73235147-73235147_T_G	600T>P	Substitution	Nonsynonymous coding	50%
MM12T	GGA3	golgi-associated, gamma adaptin ear containing, ARF binding protein 3	CCDS11717.1	chr17_73236050-73236050_G_T	468P>H	Substitution	Nonsynonymous coding	18%
MM12T	GGN	gametogenetin	CCDS12516.1	chr19_38877535-38877535_G_A	123R>C	Substitution	Nonsynonymous coding	23%
MM12T	GHSR	growth hormone secretagogue receptor	CCDS3218.1	chr3_172163105-172163105_G_A	316A>V	Substitution	Nonsynonymous coding	34%
MM12T	GID4	GID complex subunit 4, VID24 homolog (S. cerevisiae)	CCDS11190.1	chr17_17965214-17965214_C_T	255A>V	Substitution	Nonsynonymous coding	27%
MM12T	GIGYF1	GRB10 interacting GYF protein 1	CCDS34708.1	chr7_100280085-100280085_G_A	874S>L	Substitution	Nonsynonymous coding	26%
MM12T	GIGYF1	GRB10 interacting GYF protein 1	CCDS34708.1	chr7_100281911-100281911_G_A	560A>V	Substitution	Nonsynonymous coding	34%
MM12T	GIMAP8	GTPase, IMAP family member 8	CCDS34777.1	chr7_150171186-150171186_G_A	257G>S	Substitution	Nonsynonymous coding	30%
MM12T	GJA3	gap junction protein, alpha 3, 46kDa	CCDS9289.1	chr13_20716779-20716779_G_A	217L>F	Substitution	Nonsynonymous coding	28%
MM12T	GJA5	gap junction protein, alpha 5, 40kDa	CCDS929.1	chr1_147230328-147230328_T_C	340D>G	Substitution	Nonsynonymous coding	34%
MM12T	GJA5	gap junction protein, alpha 5, 40kDa	CCDS929.1	chr1_147230701-147230701_C_T	216A>T	Substitution	Nonsynonymous coding	35%
MM12T	GJA5	gap junction protein, alpha 5, 40kDa	CCDS929.1	chr1_147231031-147231031_G_A	106R>C	Substitution	Nonsynonymous coding	36%
MM12T	GJA9	gap junction protein, alpha 9, 59kDa	CCDS432.1	chr1_39340248-39340248_C_T	508R>Q	Substitution	Nonsynonymous coding	27%
MM12T	GJB7	gap junction protein, beta 7, 25kDa	CCDS5008.1	chr6_87993986-87993986_T_A	215L>F	Substitution	Nonsynonymous coding	15%
MM12T	GKN2	gastrokine 2	CCDS33215.1	chr2_69177317-69177317_C_T	49A>T	Substitution	Nonsynonymous coding	11%
MM12T	GLA	galactosidase, alpha	CCDS14484.1	chrX_100653866-100653866_C_T	236W>X	Substitution	Nonsense	31%
MM12T	GLB1L3	galactosidase, beta 1-like 3	CCDS44780.1	chr11_134151966-134151966_G_A	160R>H	Substitution	Nonsynonymous coding	21%
MM12T	GLE1	GLE1 RNA export mediator homolog (yeast)	CCDS35154.1	chr9_131295863-131295863_C_T	462R>C	Substitution	Nonsynonymous coding	10%
MM12T	GLI2	GLI family zinc finger 2	CCDS33283.1	chr2_121740331-121740331_C_A	520L>M	Substitution	Nonsynonymous coding	35%
MM12T	GLI2	GLI family zinc finger 2	CCDS33283.1	chr2_121746078-121746078_C_T	863S>L	Substitution	Nonsynonymous coding	23%
MM12T	GLI3	GLI family zinc finger 3	CCDS5465.1	chr7_42012096-42012096_G_T	648P>H	Substitution	Nonsynonymous coding	29%
MM12T	GLIS3	GLIS family zinc finger 3	CCDS43784.1	chr9_4286193-4286193_C_T	78R>H	Substitution	Nonsynonymous coding	10%
MM12T	GLP1R	glucagon-like peptide 1 receptor	CCDS4839.1	chr6_39040654-39040654_A_T	176R>W	Substitution	Nonsynonymous coding	26%
MM12T	GLP1R	glucagon-like peptide 1 receptor	CCDS4839.1	chr6_39046907-39046907_T_G	325V>G	Substitution	Nonsynonymous coding	21%
MM12T	GLP2R	glucagon-like peptide 2 receptor	CCDS11150.1	chr17_9792882-9792882_C_T	508R>X	Substitution	Nonsense	33%
MM12T	GLS	glutaminase	CCDS2308.1	chr2_191760355-191760355_C_T	170T>M	Substitution	Nonsynonymous coding	22%

MM12T	GLS2	glutaminase 2 (liver, mitochondrial)	CCDS8921.1	chr12_56865926-56865926_G_T	522A>D	Substitution	Nonsynonymous coding	28%
MM12T	GLT8D1	glycosyltransferase 8 domain containing 1	CCDS2862.1	chr3_52734353-52734353_C_T	14A>T	Substitution	Nonsynonymous coding	20%
MM12T	GLTP	glycolipid transfer protein	CCDS9136.1	chr12_110293489-110293489_C_T	128A>T	Substitution	Nonsynonymous coding	12%
MM12T	GLTSCR1	glioma tumor suppressor candidate region gene 1	CCDS46134.1	chr19_48183629-48183629_C_T	401A>V	Substitution	Nonsynonymous coding	11%
MM12T	GLTSCR2	glioma tumor suppressor candidate region gene 2	CCDS12705.1	chr19_48258120-48258120_G_A	342R>Q	Substitution	Nonsynonymous coding	30%
MM12T	GMEB2	glucocorticoid modulatory element binding protein 2	CCDS13528.1	chr20_62223494-62223494_G_T	278A>D	Substitution	Nonsynonymous coding	12%
MM12T	GMEB2	glucocorticoid modulatory element binding protein 2	CCDS13528.1	chr20_62250746-62250746_G_A	2A>V	Substitution	Nonsynonymous coding	15%
MM12T	GMPPB	GDP-mannose pyrophosphorylase B	CCDS2802.1	chr3_49759931-49759931_A_G	192S>P	Substitution	Nonsynonymous coding	11%
MM12T	GMPR	guanosine monophosphate reductase	CCDS4537.1	chr6_16290836-16290836_G_T	281G>X	Substitution	Nonsense	27%
MM12T	GNA12	guanine nucleotide binding protein (G protein) alpha 12	CCDS5335.1	chr7_2834770-2834770_C_A	106R>M	Substitution	Nonsynonymous coding	34%
MM12T	GNA13	guanine nucleotide binding protein (G protein), alpha 13	CCDS11661.1	chr17_63010545-63010545_C_T	322V>I	Substitution	Nonsynonymous coding	13%
MM12T	GNAI2	guanine nucleotide binding protein (G protein), alpha inhibiting activity polypeptide 2	CCDS2813.1	chr3_50290538-50290538_G_A	129R>Q	Substitution	Nonsynonymous coding	30%
MM12T	GNAL	guanine nucleotide binding protein (G protein), alpha activating activity polypeptide, olfactory	CCDS11851.1	chr18_11880989-11880989_G_T	411R>M	Substitution	Nonsynonymous coding	33%
MM12T	GNAO1	guanine nucleotide binding protein (G protein), alpha activating activity polypeptide O	CCDS10756.1	chr16_56226510-56226510_C_T	48T>I	Substitution	Nonsynonymous coding	20%
MM12T	GNAQ	guanine nucleotide binding protein (G protein), q polypeptide	CCDS6658.1	chr9_80537237-80537237_G_A	54T>M	Substitution	Nonsynonymous coding	11%
MM12T	GNAS	GNAS complex locus	CCDS46622.1	chr20_57430289-57430289_G_A	657A>T	Substitution	Nonsynonymous coding	33%
MM12T	GNAT3	guanine nucleotide binding protein, alpha transducing 3	CCDS47625.1	chr7_80088000-80088000_C_T	351C>Y	Substitution	Nonsynonymous coding	24%
MM12T	GNB1L	guanine nucleotide binding protein (G protein), beta polypeptide 1-like	CCDS13768.1	chr22_19776480-19776480_G_A	246R>C	Substitution	Nonsynonymous coding	11%
MM12T	GNB4	guanine nucleotide binding protein (G protein), beta polypeptide 4	CCDS3230.1	chr3_179131551-179131551_C_T	150R>H	Substitution	Nonsynonymous coding	29%
MM12T	GNL1	guanine nucleotide binding protein-like 1	CCDS4680.1	chr6_30515230-30515230_C_T	393G>S	Substitution	Nonsynonymous coding	20%
MM12T	GNL2	guanine nucleotide binding protein-like 2 (nucleolar)	CCDS421.1	chr1_38041276-38041276_G_A	355R>C	Substitution	Nonsynonymous coding	23%
MM12T	GNPTG	N-acetylglucosamine-1-phosphate transferase, gamma subunit	CCDS10436.1	chr16_1411778-1411778_C_A	71C>X	Substitution	Nonsense	29%
MM12T	GOLGA2B	-	ENST00000397112	chr19_22785154-22785154_G_A	33R>H	Substitution	Nonsynonymous coding	26%
MM12T	GOLGA2B	-	ENST00000397112	chr19_22785696-22785696_C_T	ISV+3>	Substitution	Splice site donor	31%
MM12T	GOLGA4	golgin A4	CCDS2666.1	chr3_37365590-37365590_G_A	738S>N	Substitution	Nonsynonymous coding	12%
MM12T	GOLGA4	golgin A4	CCDS2666.1	chr3_37366217-37366217_C_T	947T>I	Substitution	Nonsynonymous coding	11%
MM12T	GOLGA5	golgin A5	CCDS9905.1	chr14_93264290-93264290_A_G	170T>A	Substitution	Nonsynonymous coding	24%
MM12T	GOLIM4	golgi integral membrane protein 4	CCDS3204.1	chr3_167747752-167747752_C_T	417A>T	Substitution	Nonsynonymous coding	29%
MM12T	GOLM1	golgi membrane protein 1	CCDS35054.1	chr9_88694216-88694216_C_T	7G>E	Substitution	Nonsynonymous coding	28%
MM12T	GOLT1A	golgi transport 1A	CCDS1443.1	chr1_204172119-204172119_C_T	ISV-4>	Substitution	Splice site acceptor	11%
MM12T	GON4L	gon-4-like (C. elegans)	CCDS44242.1	chr1_155823199-155823199_C_T	125A>T	Substitution	Nonsynonymous coding	37%
MM12T	GOPC	golgi-associated PDZ and coiled-coil motif containing	CCDS5117.1	chr6_117894670-117894670_C_T	259R>H	Substitution	Nonsynonymous coding	34%

MM12T	GOT1L1	glutamic-oxaloacetic transaminase 1-like 1	CCDS47839.1	chr8_37791834-37791834_T_	NA	Deletion	Frameshift	48%
MM12T	GP6	glycoprotein VI (platelet)	CCDS42626.1	chr19_55530062-55530062_C_T	228A>T	Substitution	Nonsynonymous coding	31%
MM12T	GPAT2	glycerol-3-phosphate acyltransferase 2, mitochondrial	CCDS42714.1	chr2_96688929-96688929_G_A	692R>C	Substitution	Nonsynonymous coding	12%
MM12T	GPATCH2L	G patch domain containing 2-like	CCDS9848.1	chr14_76620840-76620840_G_A	45R>Q	Substitution	Nonsynonymous coding	29%
MM12T	GPATCH8	G patch domain containing 8	CCDS32666.1	chr17_42478532-42478532_C_T	305A>T	Substitution	Nonsynonymous coding	16%
MM12T	GPBAR1	G protein-coupled bile acid receptor 1	CCDS46515.1	chr2_219128007-219128007_G_A	187R>H	Substitution	Nonsynonymous coding	15%
MM12T	GPBP1	GC-rich promoter binding protein 1	CCDS47212.1	chr5_56546850-56546850_G_A	368D>N	Substitution	Nonsynonymous coding	39%
MM12T	GPC5	glypican 5	CCDS9468.1	chr13_92101096-92101096_G_A	82R>H	Substitution	Nonsynonymous coding	11%
MM12T	GPCPD1	glycerophosphocholine phosphodiesterase GDE1 homolog (S. cerevisiae)	CCDS13090.1	chr20_5559103-5559103_G_A	210Q>X	Substitution	Nonsense	41%
MM12T	GPD1L	glycerol-3-phosphate dehydrogenase 1-like	CCDS33729.1	chr3_32201136-32201136_G_A	310R>H	Substitution	Nonsynonymous coding	29%
MM12T	GPI	glucose-6-phosphate isomerase	ENST00000392234	chr19_34856357-34856357_G_T	65G>W	Substitution	Nonsynonymous coding	39%
MM12T	GPKOW	G patch domain and KOW motifs	CCDS35251.1	chrX_48973481-48973481_C_T	272M>I	Substitution	Nonsynonymous coding	26%
MM12T	GPLD1	glycosylphosphatidylinositol specific phospholipase D1	CCDS4553.1	chr6_24454346-24454346_A_T	411I>N	Substitution	Nonsynonymous coding	21%
MM12T	GPR108	G protein-coupled receptor 108	CCDS42479.1	chr19_6732305-6732305_T_G	365K>T	Substitution	Nonsynonymous coding	32%
MM12T	GPR112	G protein-coupled receptor 112	CCDS35409.1	chrX_135432533-135432533_C_T	2223S>F	Substitution	Nonsynonymous coding	12%
MM12T	GPR112	G protein-coupled receptor 112	CCDS35409.1	chrX_135455155-135455155_C_T	2570R>W	Substitution	Nonsynonymous coding	20%
MM12T	GPR113	G protein-coupled receptor 113	CCDS46239.1	chr2_26533093-26533093_G_T	1017L>I	Substitution	Nonsynonymous coding	36%
MM12T	GPR114	G protein-coupled receptor 114	CCDS10785.1	chr16_57601943-57601943_G_T	333A>S	Substitution	Nonsynonymous coding	16%
MM12T	GPR114	G protein-coupled receptor 114	CCDS10785.1	chr16_57604375-57604375_C_T	379S>L	Substitution	Nonsynonymous coding	39%
MM12T	GPR115	G protein-coupled receptor 115	CCDS4922.2	chr6_47682266-47682266_C_T	429R>W	Substitution	Nonsynonymous coding	22%
MM12T	GPR116	G protein-coupled receptor 116	CCDS4919.1	chr6_46830795-46830795_C_T	677G>R	Substitution	Nonsynonymous coding	23%
MM12T	GPR123	G protein-coupled receptor 123	CCDS41580.1	chr10_134916342-134916342_C_T	133L>F	Substitution	Nonsynonymous coding	28%
MM12T	GPR125	G protein-coupled receptor 125	CCDS33964.1	chr4_22390451-22390451_C_T	948R>H	Substitution	Nonsynonymous coding	22%
MM12T	GPR126	G protein-coupled receptor 126	CCDS47489.1	chr6_142736136-142736136_G_A	858A>T	Substitution	Nonsynonymous coding	35%
MM12T	GPR128	G protein-coupled receptor 128	CCDS2938.1	chr3_100413627-100413627_G_A	726V>I	Substitution	Nonsynonymous coding	11%
MM12T	GPR135	G protein-coupled receptor 135	CCDS9738.1	chr14_59931026-59931026_A_G	307S>P	Substitution	Nonsynonymous coding	19%
MM12T	GPR142	G protein-coupled receptor 142	CCDS11698.1	chr17_72368117-72368117_G_A	256R>H	Substitution	Nonsynonymous coding	12%
MM12T	GPR144	G protein-coupled receptor 144	CCDS48016.1	chr9_127215368-127215368_C_T	131A>V	Substitution	Nonsynonymous coding	11%
MM12T	GPR148	G protein-coupled receptor 148	CCDS2163.1	chr2_131487232-131487232_G_A	170A>T	Substitution	Nonsynonymous coding	36%
MM12T	GPR15	G protein-coupled receptor 15	CCDS2931.1	chr3_98251002-98251002_C_A	42A>D	Substitution	Nonsynonymous coding	25%
MM12T	GPR152	G protein-coupled receptor 152	CCDS8165.1	chr11_67218934-67218934_G_T	421S>Y	Substitution	Nonsynonymous coding	35%
MM12T	GPR153	G protein-coupled receptor 153	CCDS64.1	chr1_6313863-6313863_T_A	234E>V	Substitution	Nonsynonymous coding	32%

MM12T	GPR155	G protein-coupled receptor 155	CCDS2259.1	chr2_175333696-175333696_C_T	376A>T	Substitution	Nonsynonymous coding	25%
MM12T	GPR158	G protein-coupled receptor 158	CCDS31166.1	chr10_25464743-25464743_G_A	132A>T	Substitution	Nonsynonymous coding	10%
MM12T	GPR158	G protein-coupled receptor 158	CCDS31166.1	chr10_25887532-25887532_G_T	993G>C	Substitution	Nonsynonymous coding	21%
MM12T	GPR158	G protein-coupled receptor 158	CCDS31166.1	chr10_25883328-25883328_T_C	ISV+2>	Substitution	Splice site donor	14%
MM12T	GPR171	G protein-coupled receptor 171	CCDS3155.1	chr3_150916578-150916578_T_A	199N>I	Substitution	Nonsynonymous coding	41%
MM12T	GPR179	G protein-coupled receptor 179	CCDS42308.1	chr17_36483547-36483547_C_T	1969V>I	Substitution	Nonsynonymous coding	36%
MM12T	GPR19	G protein-coupled receptor 19	CCDS8652.1	chr12_12814265-12814265_C_T	373G>D	Substitution	Nonsynonymous coding	11%
MM12T	GPR26	G protein-coupled receptor 26	CCDS7636.1	chr10_125426537-125426537_G_A	205R>H	Substitution	Nonsynonymous coding	16%
MM12T	GPR37	G protein-coupled receptor 37 (endothelin receptor type B-like)	CCDS5792.1	chr7_124387270-124387270_G_A	384A>V	Substitution	Nonsynonymous coding	35%
MM12T	GPR39	G protein-coupled receptor 39	CCDS2170.1	chr2_133175356-133175356_G_T	247Q>H	Substitution	Nonsynonymous coding	39%
MM12T	GPR4	G protein-coupled receptor 4	CCDS12669.1	chr19_46094809-46094809_C_T	106A>T	Substitution	Nonsynonymous coding	35%
MM12T	GPR42	G protein-coupled receptor 42 (gene/pseudogene)	ENST00000246538	chr19_35862398-35862398_G_A	46R>H	Substitution	Nonsynonymous coding	28%
MM12T	GPR68	G protein-coupled receptor 68	CCDS9894.1	chr14_91700710-91700710_G_T	239L>M	Substitution	Nonsynonymous coding	40%
MM12T	GPR78	G protein-coupled receptor 78	CCDS3403.1	chr4_8588803-8588803_G_A	269V>I	Substitution	Nonsynonymous coding	36%
MM12T	GPR98	G protein-coupled receptor 98	CCDS47246.1	chr5_90106505-90106505_C_T	5143A>V	Substitution	Nonsynonymous coding	11%
MM12T	GPR98	G protein-coupled receptor 98	CCDS47246.1	chr5_90144541-90144541_C_T	5703R>C	Substitution	Nonsynonymous coding	13%
MM12T	GPRASP1	G protein-coupled receptor associated sorting protein 1	CCDS35352.1	chrX_101909158-101909158_C_T	106S>F	Substitution	Nonsynonymous coding	32%
MM12T	GPRASP1	G protein-coupled receptor associated sorting protein 1	CCDS35352.1	chrX_101909765-101909765_G_A	308W>X	Substitution	Nonsense	24%
MM12T	GPRC5B	G protein-coupled receptor, family C, group 5, member B	CCDS10581.1	chr16_19883725-19883725_C_T	148R>Q	Substitution	Nonsynonymous coding	35%
MM12T	GPRC5B	G protein-coupled receptor, family C, group 5, member B	CCDS10581.1	chr16_19883777-19883777_C_T	131V>I	Substitution	Nonsynonymous coding	28%
MM12T	GPRC6A	G protein-coupled receptor, family C, group 6, member A	CCDS5112.1	chr6_117114231-117114231_G_T	619L>M	Substitution	Nonsynonymous coding	32%
MM12T	GPRIN2	G protein regulated inducer of neurite outgrowth 2	CCDS31192.1	chr10_46999125-46999125_C_T	82A>V	Substitution	Nonsynonymous coding	12%
MM12T	GPRIN3	GPRIN family member 3	CCDS34030.1	chr4_90170792-90170792_C_A	157R>I	Substitution	Nonsynonymous coding	28%
MM12T	GPSM1	G-protein signaling modulator 1	CCDS48055.1	chr9_139233032-139233032_G_A	280R>Q	Substitution	Nonsynonymous coding	28%
MM12T	GPSM2	G-protein signaling modulator 2	CCDS792.2	chr1_109472453-109472453_T_G	649L>R	Substitution	Nonsynonymous coding	31%
MM12T	GPX4	glutathione peroxidase 4	CCDS42457.1	chr19_1105213-1105213_C_T	38A>V	Substitution	Nonsynonymous coding	30%
MM12T	GRAMD2	GRAM domain containing 2	CCDS32283.1	chr15_72455745-72455745_G_A	273A>V	Substitution	Nonsynonymous coding	29%
MM12T	GRAMD4	GRAM domain containing 4	CCDS33672.1	chr22_47073086-47073086_G_A	559E>K	Substitution	Nonsynonymous coding	10%
MM12T	GREB1	growth regulation by estrogen in breast cancer 1	CCDS42655.1	chr2_11750918-11750918_C_T	924T>M	Substitution	Nonsynonymous coding	31%
MM12T	GREB1	growth regulation by estrogen in breast cancer 1	CCDS42655.1	chr2_11755296-11755296_G_A	1068A>T	Substitution	Nonsynonymous coding	14%
MM12T	GREB1L	growth regulation by estrogen in breast cancer-like	CCDS45836.1	chr18_19088427-19088427_C_A	1537A>D	Substitution	Nonsynonymous coding	35%
MM12T	GRIA1	glutamate receptor, ionotropic, AMPA 1	CCDS4322.1	chr5_153026599-153026599_C_T	111P>L	Substitution	Nonsynonymous coding	27%

MM12T	GRIA3	glutamate receptor, ionotropic, AMPA 3	NM_000828	chrX_122538652-122538652_G_A	463A>T	Substitution	Nonsynonymous coding	21%
MM12T	GRID1	glutamate receptor, ionotropic, delta 1	CCDS31236.1	chr10_87628938-87628938_C_A	ISV-1>	Substitution	Splice site acceptor	29%
MM12T	GRIK3	glutamate receptor, ionotropic, kainate 3	CCDS416.1	chr1_37319298-37319298_C_T	377R>Q	Substitution	Nonsynonymous coding	11%
MM12T	GRIK3	glutamate receptor, ionotropic, kainate 3	CCDS416.1	chr1_37324816-37324816_C_T	333V>M	Substitution	Nonsynonymous coding	11%
MM12T	GRIK4	glutamate receptor, ionotropic, kainate 4	CCDS8433.1	chr11_120827539-120827539_G_A	584C>Y	Substitution	Nonsynonymous coding	28%
MM12T	GRIN1	glutamate receptor, ionotropic, N-methyl D-aspartate 1	CCDS7031.1	chr9_140034149-140034149_G_A	71A>T	Substitution	Nonsynonymous coding	29%
MM12T	GRIN1	glutamate receptor, ionotropic, N-methyl D-aspartate 1	CCDS7031.1	chr9_140059693-140059693_C_T	882A>V	Substitution	Nonsynonymous coding	30%
MM12T	GRIN2A	glutamate receptor, ionotropic, N-methyl D-aspartate 2A	CCDS10539.1	chr16_9943797-9943797_T_C	382T>A	Substitution	Nonsynonymous coding	34%
MM12T	GRIN2A	glutamate receptor, ionotropic, N-methyl D-aspartate 2A	CCDS10539.1	chr16_10274037-10274037_C_T	78D>N	Substitution	Nonsynonymous coding	31%
MM12T	GRIN2B	glutamate receptor, ionotropic, N-methyl D-aspartate 2B	CCDS8662.1	chr12_13716489-13716489_G_A	1228T>M	Substitution	Nonsynonymous coding	32%
MM12T	GRIN2B	glutamate receptor, ionotropic, N-methyl D-aspartate 2B	CCDS8662.1	chr12_13724822-13724822_C_T	696R>H	Substitution	Nonsynonymous coding	38%
MM12T	GRIN2D	glutamate receptor, ionotropic, N-methyl D-aspartate 2D	CCDS12719.1	chr19_48908447-48908447_C_T	308R>C	Substitution	Nonsynonymous coding	31%
MM12T	GRIPAP1	GRIP1 associated protein 1	CCDS35248.1	chrX_48847125-48847125_G_A	221T>I	Substitution	Nonsynonymous coding	33%
MM12T	GRK1	G protein-coupled receptor kinase 1	NM_002929	chr13_114322315-114322315_C_T	205S>L	Substitution	Nonsynonymous coding	36%
MM12T	GRK6	G protein-coupled receptor kinase 6	CCDS47348.1	chr5_176858998-176858998_C_T	51R>C	Substitution	Nonsynonymous coding	47%
MM12T	GRK6	G protein-coupled receptor kinase 6	CCDS47348.1	chr5_176860543-176860543_G_A	202A>T	Substitution	Nonsynonymous coding	10%
MM12T	GRK6	G protein-coupled receptor kinase 6	CCDS47348.1	chr5_176863154-176863154_G_A	380A>T	Substitution	Nonsynonymous coding	12%
MM12T	GRK6	G protein-coupled receptor kinase 6	CCDS47348.1	chr5_176867941-176867941_C_T	522Q>X	Substitution	Nonsense	32%
MM12T	GRM6	glutamate receptor, metabotropic 6	CCDS4442.1	chr5_178413541-178413541_G_A	572R>C	Substitution	Nonsynonymous coding	34%
MM12T	GRM7	glutamate receptor, metabotropic 7	CCDS43042.1	chr3_6903497-6903497_C_T	141P>L	Substitution	Nonsynonymous coding	32%
MM12T	GRPEL2	GrpE-like 2, mitochondrial (E. coli)	CCDS4295.1	chr5_148725107-148725107_C_T	2A>V	Substitution	Nonsynonymous coding	29%
MM12T	GRTP1	growth hormone regulated TBC protein 1	CCDS9534.2	chr13_113980035-113980035_C_T	288D>N	Substitution	Nonsynonymous coding	15%
MM12T	GSC2	goosecoid homeobox 2	CCDS13757.1	chr22_19136536-19136536_C_T	196G>S	Substitution	Nonsynonymous coding	38%
MM12T	GSPT2	G1 to S phase transition 2	CCDS14336.1	chrX_51486942-51486942_C_T	74R>W	Substitution	Nonsynonymous coding	11%
MM12T	GSPT2	G1 to S phase transition 2	CCDS14336.1	chrX_51488476-51488476_G_A	585R>H	Substitution	Nonsynonymous coding	30%
MM12T	GSR	glutathione reductase	CCDS34877.1	chr8_30538444-30538444_C_T	466V>I	Substitution	Nonsynonymous coding	23%
MM12T	GSR	glutathione reductase	CCDS34877.1	chr8_30538502-30538502_G_T	446S>R	Substitution	Nonsynonymous coding	17%
MM12T	GSTP1	glutathione S-transferase pi 1	CCDS41679.1	chr11_67352222-67352222_C_T	71R>C	Substitution	Nonsynonymous coding	12%
MM12T	GTDC2	glycosyltransferase-like domain containing 2	CCDS2709.1	chr3_43121788-43121788_G_A	379T>M	Substitution	Nonsynonymous coding	34%
MM12T	GTF2B	general transcription factor IIB	CCDS715.1	chr1_89325627-89325627_G_A	158A>V	Substitution	Nonsynonymous coding	37%
MM12T	GTF3C2	general transcription factor IIIC, polypeptide 2, beta 110kDa	CCDS1749.1	chr2_27549573-27549573_G_A	902P>L	Substitution	Nonsynonymous coding	27%
MM12T	GTF3C4	general transcription factor IIIC, polypeptide 4, 90kDa	CCDS6953.1	chr9_135554285-135554285_G_A	427V>I	Substitution	Nonsynonymous coding	29%

MM12T	GTF3C5	general transcription factor IIIC, polypeptide 5, 63kDa	ENST00000372089	chr9_135930426-135930426_G_T	255R>M	Substitution	Nonsynonymous coding	29%
MM12T	GTPBP1	GTP binding protein 1	CCDS13977.2	chr22_39104964-39104964_G_A	ISV+3>	Substitution	Splice site donor	23%
MM12T	GTPBP3	GTP binding protein 3 (mitochondrial)	CCDS32950.1	chr19_17452467-17452467_G_T	510E>D	Substitution	Nonsynonymous coding	34%
MM12T	GTPBP5	GTP binding protein 5 (putative)	CCDS13492.1	chr20_60773864-60773864_C_T	215R>X	Substitution	Nonsense	13%
MM12T	GTPBP8	GTP-binding protein 8 (putative)	CCDS33820.1	chr3_112709920-112709920_G_A	25R>H	Substitution	Nonsynonymous coding	11%
MM12T	GUCY1B2	guanylate cyclase 1, soluble, beta 2 (pseudogene)	ENST00000378172	chr13_51598407-51598407_C_A	221G>X	Substitution	Nonsense	14%
MM12T	GUCY1B3	guanylate cyclase 1, soluble, beta 3	CCDS47154.1	chr4_156710956-156710956_G_T	130G>X	Substitution	Nonsense	14%
MM12T	GUCY2F	guanylate cyclase 2F, retinal	CCDS14545.1	chrX_108636233-108636233_G_A	826R>W	Substitution	Nonsynonymous coding	13%
MM12T	GUF1	GUF1 GTPase homolog (S. cerevisiae)	CCDS3468.1	chr4_44682800-44682800_G_T	123A>S	Substitution	Nonsynonymous coding	25%
MM12T	GZMB	granzyme B (granzyme 2, cytotoxic T-lymphocyte-associated serine esterase 1)	CCDS9633.1	chr14_25101100-25101100_G_T	188C>X	Substitution	Nonsense	35%
MM12T	H1FOO	H1 histone family, member O, oocyte-specific	CCDS3064.1	chr3_129266513-129266513_G_A	123G>D	Substitution	Nonsynonymous coding	40%
MM12T	H1FOO	H1 histone family, member O, oocyte-specific	CCDS3064.1	chr3_129268039-129268039_G_A	192A>T	Substitution	Nonsynonymous coding	28%
MM12T	H2AFY	H2A histone family, member Y	CCDS4185.1	chr5_134724734-134724734_G_A	17A>V	Substitution	Nonsynonymous coding	12%
MM12T	H6PD	hexose-6-phosphate dehydrogenase (glucose 1-dehydrogenase)	CCDS101.1	chr1_9323930-9323930_G_A	460V>I	Substitution	Nonsynonymous coding	35%
MM12T	HACL1	2-hydroxyacyl-CoA lyase 1	CCDS2627.1	chr3_15613280-15613280_A_	NA	Deletion	Splice site acceptor	18%
MM12T	HAL	histidine ammonia-lyase	CCDS9058.1	chr12_96387702-96387702_A_G	ISV+2>	Substitution	Splice site donor	13%
MM12T	HAPLN1	hyaluronan and proteoglycan link protein 1	CCDS4061.1	chr5_82969333-82969333_G_T	4L>I	Substitution	Nonsynonymous coding	34%
MM12T	HAPLN2	hyaluronan and proteoglycan link protein 2	CCDS1148.1	chr1_156593317-156593317_G_A	12R>H	Substitution	Nonsynonymous coding	31%
MM12T	HARBI1	harbinger transposase derived 1	CCDS7920.1	chr11_46637429-46637429_C_T	120R>H	Substitution	Nonsynonymous coding	12%
MM12T	HAS1	hyaluronan synthase 1	ENST00000376738	chr19_52217367-52217367_C_T	180A>T	Substitution	Nonsynonymous coding	13%
MM12T	HAUS5	HAUS augmin-like complex, subunit 5	CCDS42550.1	chr19_36109051-36109051_C_T	259R>W	Substitution	Nonsynonymous coding	31%
MM12T	HBA2	hemoglobin, alpha 2	CCDS10398.1	chr16_223565-223565_C_T	132S>F	Substitution	Nonsynonymous coding	10%
MM12T	HBE1	hemoglobin, epsilon 1	CCDS7756.1	chr11_5290794-5290794_G_T	69L>M	Substitution	Nonsynonymous coding	31%
MM12T	HBS1L	HBS1-like (S. cerevisiae)	CCDS5173.1	chr6_135307949-135307949_C_A	ISV-1>	Substitution	Splice site acceptor	23%
MM12T	HCFC2	host cell factor C2	CCDS9097.1	chr12_104496990-104496990_A_G	773Y>C	Substitution	Nonsynonymous coding	37%
MM12T	HCRT	hypocretin (orexin) neuropeptide precursor	CCDS11421.1	chr17_40337373-40337373_G_A	4P>L	Substitution	Nonsynonymous coding	21%
MM12T	HDAC10	histone deacetylase 10	CCDS14088.1	chr22_50686323-50686323_C_A	445A>S	Substitution	Nonsynonymous coding	12%
MM12T	HDAC6	histone deacetylase 6	CCDS14306.1	chrX_48674580-48674580_G_A	509R>Q	Substitution	Nonsynonymous coding	22%
MM12T	HDAC6	histone deacetylase 6	CCDS14306.1	chrX_48676758-48676758_G_A	709R>H	Substitution	Nonsynonymous coding	16%
MM12T	HDAC7	histone deacetylase 7	CCDS8756.2	chr12_48177971-48177971_C_T	941A>T	Substitution	Nonsynonymous coding	31%
MM12T	HDAC7	histone deacetylase 7	CCDS8756.2	chr12_48189390-48189390_G_A	356S>F	Substitution	Nonsynonymous coding	12%
MM12T	HDAC7	histone deacetylase 7	CCDS8756.2	chr12_48187203-48187203_G_A	543R>X	Substitution	Nonsense	38%

MM12T	HDAC9	histone deacetylase 9	CCDS47553.1	chr7_18767227-18767227_C_T	586R>C	Substitution	Nonsynonymous coding	25%
MM12T	HDAC9	histone deacetylase 9	CCDS47553.1	chr7_18833029-18833029_G_A	759G>D	Substitution	Nonsynonymous coding	30%
MM12T	HDGFRP2	Hepatoma-derived growth factor-related protein 2 [Source:UniProtKB/Swiss-	NM_001001520	chr19_4488764-4488764_C_T	127A>V	Substitution	Nonsynonymous coding	14%
MM12T	HDLBP	high density lipoprotein binding protein	CCDS2547.1	chr2_242170301-242170301_G_A	1116A>V	Substitution	Nonsynonymous coding	18%
MM12T	HDLBP	high density lipoprotein binding protein	CCDS2547.1	chr2_242179456-242179456_G_A	751R>C	Substitution	Nonsynonymous coding	11%
MM12T	HDLBP	high density lipoprotein binding protein	CCDS2547.1	chr2_242195708-242195708_C_T	255R>H	Substitution	Nonsynonymous coding	22%
MM12T	HEATR1	HEAT repeat containing 1	CCDS31066.1	chr1_236766620-236766620_C_T	67A>T	Substitution	Nonsynonymous coding	25%
MM12T	HEATR4	HEAT repeat containing 4	CCDS9815.1	chr14_73962003-73962003_C_T	858R>H	Substitution	Nonsynonymous coding	30%
MM12T	HEATR5B	HEAT repeat containing 5B	CCDS33181.1	chr2_37292000-37292000_T_G	489E>D	Substitution	Nonsynonymous coding	37%
MM12T	HEATR6	HEAT repeat containing 6	CCDS11623.1	chr17_58147094-58147094_G_T	306S>Y	Substitution	Nonsynonymous coding	24%
MM12T	HECTD1	HECT domain containing E3 ubiquitin protein ligase 1	CCDS41939.1	chr14_31583160-31583160_G_A	1927R>C	Substitution	Nonsynonymous coding	11%
MM12T	HECTD1	HECT domain containing E3 ubiquitin protein ligase 1	CCDS41939.1	chr14_31598492-31598492_C_A	1362S>I	Substitution	Nonsynonymous coding	31%
MM12T	HECW1	HECT, C2 and WW domain containing E3 ubiquitin protein ligase 1	CCDS5469.2	chr7_43546759-43546759_G_A	1219A>T	Substitution	Nonsynonymous coding	11%
MM12T	HECW1	HECT, C2 and WW domain containing E3 ubiquitin protein ligase 1	CCDS5469.2	chr7_43546790-43546790_C_T	1229A>V	Substitution	Nonsynonymous coding	23%
MM12T	HELZ2	helicase with zinc finger 2, transcriptional coactivator	CCDS13527.1	chr20_62198989-62198989_G_	NA	Deletion	Frameshift	13%
MM12T	HELZ2	helicase with zinc finger 2, transcriptional coactivator	CCDS33508.1	chr20_62194855-62194855_C_T	1774G>S	Substitution	Nonsynonymous coding	15%
MM12T	HEPACAM	hepatic and glial cell adhesion molecule	CCDS8456.1	chr11_124793754-124793754_G_A	194L>F	Substitution	Nonsynonymous coding	16%
MM12T	HEPHL1	hephaestin-like 1	CCDS44710.1	chr11_93800872-93800872_C_A	340P>H	Substitution	Nonsynonymous coding	18%
MM12T	HERC1	HECT and RLD domain containing E3 ubiquitin protein ligase family member 1	CCDS45277.1	chr15_63915053-63915053_G_A	4550S>F	Substitution	Nonsynonymous coding	33%
MM12T	HERC1	HECT and RLD domain containing E3 ubiquitin protein ligase family member 1	CCDS45277.1	chr15_64015450-64015450_C_T	1225A>T	Substitution	Nonsynonymous coding	32%
MM12T	HERC2	HECT and RLD domain containing E3 ubiquitin protein ligase 2	CCDS10021.1	chr15_28420699-28420699_C_T	3264V>I	Substitution	Nonsynonymous coding	14%
MM12T	HERC2	HECT and RLD domain containing E3 ubiquitin protein ligase 2	CCDS10021.1	chr15_28427555-28427555_C_A	2977G>C	Substitution	Nonsynonymous coding	13%
MM12T	HERC2	HECT and RLD domain containing E3 ubiquitin protein ligase 2	CCDS10021.1	chr15_28514428-28514428_C_T	471R>H	Substitution	Nonsynonymous coding	12%
MM12T	HERC2	HECT and RLD domain containing E3 ubiquitin protein ligase 2	CCDS10021.1	chr15_28519467-28519467_G_A	257A>V	Substitution	Nonsynonymous coding	33%
MM12T	HERC4	HECT and RLD domain containing E3 ubiquitin protein ligase 4	CCDS41533.1	chr10_69832837-69832837_C_T	10G>E	Substitution	Nonsynonymous coding	20%
MM12T	HERC5	HECT and RLD domain containing E3 ubiquitin protein ligase 5	CCDS3630.1	chr4_89381266-89381266_A_	NA	Deletion	Frameshift	44%
MM12T	HERC5	HECT and RLD domain containing E3 ubiquitin protein ligase 5	CCDS3630.1	chr4_89426859-89426859_G_A	969D>N	Substitution	Nonsynonymous coding	39%
MM12T	HESX1	HESX homeobox 1	CCDS2881.1	chr3_57232894-57232894_C_A	82E>X	Substitution	Nonsense	30%
MM12T	HGFAC	HGF activator	CCDS3369.1	chr4_3447850-3447850_G_A	395G>D	Substitution	Nonsynonymous coding	35%
MM12T	HGFAC	HGF activator	CCDS3369.1	chr4_3449275-3449275_C_T	471T>M	Substitution	Nonsynonymous coding	10%
MM12T	HGS	hepatocyte growth factor-regulated tyrosine kinase substrate	CCDS11784.1	chr17_79658518-79658518_T_G	193C>W	Substitution	Nonsynonymous coding	33%
MM12T	HGS	hepatocyte growth factor-regulated tyrosine kinase substrate	CCDS11784.1	chr17_79660781-79660781_T_C	280L>P	Substitution	Nonsynonymous coding	38%

MM12T	HGS	hepatocyte growth factor-regulated tyrosine kinase substrate	CCDS11784.1	chr17_79663417-79663417_C_T	475A>V	Substitution	Nonsynonymous coding	32%
MM12T	HGS	hepatocyte growth factor-regulated tyrosine kinase substrate	CCDS11784.1	chr17_79663743-79663743_C_T	558A>V	Substitution	Nonsynonymous coding	40%
MM12T	HHIPL1	HHIP-like 1	CCDS45162.1	chr14_100125791-100125791_G_A	358R>H	Substitution	Nonsynonymous coding	14%
MM12T	HHIPL2	HHIP-like 2	CCDS1530.2	chr1_222696957-222696957_C_T	629A>T	Substitution	Nonsynonymous coding	33%
MM12T	HID1	HID1 domain containing	CCDS32726.1	chr17_72958080-72958080_C_T	206E>K	Substitution	Nonsynonymous coding	12%
MM12T	HIF3A	hypoxia inducible factor 3, alpha subunit	CCDS12681.2	chr19_46834528-46834528_C_A	610L>M	Substitution	Nonsynonymous coding	26%
MM12T	HINFP	histone H4 transcription factor	CCDS8414.1	chr11_118997775-118997775_C_T	41Q>X	Substitution	Nonsense	37%
MM12T	HIP1R	huntingtin interacting protein 1 related	CCDS31922.1	chr12_123345246-123345246_T_C	894V>A	Substitution	Nonsynonymous coding	10%
MM12T	HIPK1	homeodomain interacting protein kinase 1	CCDS867.1	chr1_114514469-114514469_C_T	1013P>S	Substitution	Nonsynonymous coding	12%
MM12T	HIPK1	homeodomain interacting protein kinase 1	CCDS867.1	chr1_114515739-114515739_G_A	1080A>T	Substitution	Nonsynonymous coding	33%
MM12T	HIPK2	homeodomain interacting protein kinase 2	NM_022740	chr7_139258092-139258092_C_T	1060A>T	Substitution	Nonsynonymous coding	31%
MM12T	HIST1H2AH	histone cluster 1, H2ah	CCDS4622.1	chr6_27114908-27114908_A_T	1M>L	Substitution	Nonsynonymous coding	10%
MM12T	HIVEP2	human immunodeficiency virus type I enhancer binding protein 2	CCDS43510.1	chr6_143092148-143092148_C_T	1243G>D	Substitution	Nonsynonymous coding	11%
MM12T	HIVEP3	human immunodeficiency virus type I enhancer binding protein 3	CCDS463.1	chr1_42049427-42049427_G_C	348H>D	Substitution	Nonsynonymous coding	38%
MM12T	HJURP	Holliday junction recognition protein	CCDS33406.1	chr2_234750809-234750809_G_A	206A>V	Substitution	Nonsynonymous coding	26%
MM12T	HK1	hexokinase 1	CCDS7289.1	chr10_71158430-71158430_G_A	823V>I	Substitution	Nonsynonymous coding	30%
MM12T	HK2	hexokinase 2	CCDS1956.1	chr2_75105930-75105930_G_A	383A>T	Substitution	Nonsynonymous coding	34%
MM12T	HK3	hexokinase 3 (white cell)	CCDS4407.1	chr5_176314532-176314532_G_A	507A>V	Substitution	Nonsynonymous coding	37%
MM12T	HK3	hexokinase 3 (white cell)	CCDS4407.1	chr5_176318154-176318154_C_A	100G>W	Substitution	Nonsynonymous coding	35%
MM12T	HKDC1	hexokinase domain containing 1	CCDS7288.1	chr10_71025345-71025345_C_T	793R>W	Substitution	Nonsynonymous coding	31%
MM12T	HKR1	HKR1, GLI-Kruppel zinc finger family member	CCDS12502.1	chr19_37854163-37854163_G_T	489R>M	Substitution	Nonsynonymous coding	32%
MM12T	HLA-DOB	major histocompatibility complex, class II, DO beta	CCDS4754.1	chr6_32782988-32782988_C_T	65R>H	Substitution	Nonsynonymous coding	11%
MM12T	HLA-E	major histocompatibility complex, class I, E	CCDS34379.1	chr6_30458013-30458013_C_A	ISV-4>	Substitution	Splice site acceptor	25%
MM12T	HLX	H2.0-like homeobox	CCDS1527.1	chr1_221053738-221053738_G_A	180R>H	Substitution	Nonsynonymous coding	31%
MM12T	HLX	H2.0-like homeobox	CCDS1527.1	chr1_221055560-221055560_G_A	276R>H	Substitution	Nonsynonymous coding	12%
MM12T	HM13	histocompatibility (minor) 13	CCDS42861.1	chr20_30137888-30137888_G_A	230V>M	Substitution	Nonsynonymous coding	13%
MM12T	HMCN1	hemicentin 1	CCDS30956.1	chr1_186113368-186113368_C_T	4663T>I	Substitution	Nonsynonymous coding	17%
MM12T	HMCN2	hemicentin 2	ENST00000420499	chr9_133231606-133231606_C_T	455R>W	Substitution	Nonsynonymous coding	12%
MM12T	HMCN2	hemicentin 2	ENST00000428715	chr9_133298093-133298093_G_A	164R>H	Substitution	Nonsynonymous coding	32%
MM12T	HMGB4	high mobility group box 4	NM_145205	chr1_34329968-34329968_A_G	59K>R	Substitution	Nonsynonymous coding	32%
MM12T	HMGN5	high mobility group nucleosome binding domain 5	CCDS14448.1	chrX_80371786-80371786_C_A	62A>S	Substitution	Nonsynonymous coding	33%
MM12T	HMGXB3	HMG box domain containing 3	ENST00000421549	chr5_149380596-149380596_G_A	197R>H	Substitution	Nonsynonymous coding	12%

MM12T	HNF1A	HNF1 homeobox A	CCDS9209.1	chr12_121431482-121431482_G_A	229R>Q	Substitution	Nonsynonymous coding	29%
MM12T	HNRNPH1	heterogeneous nuclear ribonucleoprotein H1 (H)	CCDS4446.1	chr5_179044837-179044837_C_T	295G>E	Substitution	Nonsynonymous coding	55%
MM12T	HNRNPH2	heterogeneous nuclear ribonucleoprotein H2 (H')	CCDS14485.1	chrX_100667701-100667701_G_A	242G>D	Substitution	Nonsynonymous coding	12%
MM12T	HOMER1	homer homolog 1 (Drosophila)	CCDS43335.1	chr5_78693456-78693456_G_T	230T>N	Substitution	Nonsynonymous coding	33%
MM12T	HOMER2	homer homolog 2 (Drosophila)	CCDS45334.1	chr15_83523542-83523542_C_T	180V>M	Substitution	Nonsynonymous coding	31%
MM12T	HOXA11	homeobox A11	CCDS5411.1	chr7_27224102-27224102_C_T	221S>N	Substitution	Nonsynonymous coding	15%
MM12T	HOXA13	homeobox A13	CCDS5412.1	chr7_27238934-27238934_C_T	255V>M	Substitution	Nonsynonymous coding	32%
MM12T	HOXA3	homeobox A3	CCDS5404.1	chr7_27148105-27148105_C_T	254G>D	Substitution	Nonsynonymous coding	31%
MM12T	HOXA4	homeobox A4	CCDS5405.1	chr7_27169073-27169073_C_T	245R>H	Substitution	Nonsynonymous coding	34%
MM12T	HOXA5	homeobox A5	CCDS5406.1	chr7_27183222-27183222_C_T	2S>N	Substitution	Nonsynonymous coding	27%
MM12T	HOXC6	homeobox C6	CCDS8871.1	chr12_54422550-54422550_A_G	82D>G	Substitution	Nonsynonymous coding	13%
MM12T	HPCAL1	hippocalcin-like 1	CCDS1671.1	chr2_10559933-10559933_G_A	17R>Q	Substitution	Nonsynonymous coding	18%
MM12T	HPN	hepsin	CCDS32993.1	chr19_35551281-35551281_G_A	162R>H	Substitution	Nonsynonymous coding	30%
MM12T	HPRT1	hypoxanthine phosphoribosyltransferase 1	CCDS14641.1	chrX_133609314-133609314_G_T	80D>Y	Substitution	Nonsynonymous coding	39%
MM12T	HPS4	Hermansky-Pudlak syndrome 4	CCDS13835.1	chr22_26861492-26861492_C_A	244Q>H	Substitution	Nonsynonymous coding	28%
MM12T	HPS5	Hermansky-Pudlak syndrome 5	CCDS7836.1	chr11_18313554-18313554_C_A	625Q>H	Substitution	Nonsynonymous coding	30%
MM12T	HPS6	Hermansky-Pudlak syndrome 6	CCDS7527.1	chr10_103826900-103826900_C_	NA	Deletion	Frameshift	28%
MM12T	HPSE	heparanase	CCDS3602.1	chr4_84231985-84231985_T_G	244E>D	Substitution	Nonsynonymous coding	48%
MM12T	HPX	hemopexin	CCDS7763.1	chr11_6452970-6452970_G_A	344R>W	Substitution	Nonsynonymous coding	26%
MM12T	HR	hair growth associated	CCDS6022.1	chr8_21974470-21974470_C_T	1099R>Q	Substitution	Nonsynonymous coding	41%
MM12T	HR	hair growth associated	CCDS6022.1	chr8_21984685-21984685_C_T	424G>S	Substitution	Nonsynonymous coding	30%
MM12T	HRASLS5	HRAS-like suppressor family, member 5	CCDS8044.1	chr11_63233649-63233649_C_T	227S>N	Substitution	Nonsynonymous coding	20%
MM12T	HRCT1	histidine rich carboxyl terminus 1	CCDS35012.1	chr9_35906330-35906330_G_T	16G>C	Substitution	Nonsynonymous coding	32%
MM12T	HRG	histidine-rich glycoprotein	CCDS3280.1	chr3_186386754-186386754_G_A	72D>N	Substitution	Nonsynonymous coding	25%
MM12T	HS3ST4	heparan sulfate (glucosamine) 3-O-sulfotransferase 4	NM_006040	chr16_26147228-26147228_C_A	344L>I	Substitution	Nonsynonymous coding	37%
MM12T	HS6ST3	heparan sulfate 6-O-sulfotransferase 3	CCDS9481.1	chr13_96743184-96743184_A_G	23Y>C	Substitution	Nonsynonymous coding	25%
MM12T	HSD11B2	hydroxysteroid (11-beta) dehydrogenase 2	CCDS10837.1	chr16_67470575-67470575_G_A	296G>D	Substitution	Nonsynonymous coding	28%
MM12T	HSD17B3	hydroxysteroid (17-beta) dehydrogenase 3	CCDS6716.1	chr9_99007671-99007671_C_T	188A>T	Substitution	Nonsynonymous coding	32%
MM12T	HSF2BP	heat shock transcription factor 2 binding protein	CCDS13697.1	chr21_45053168-45053168_C_A	142K>N	Substitution	Nonsynonymous coding	37%
MM12T	HSF2BP	heat shock transcription factor 2 binding protein	CCDS13697.1	chr21_45077962-45077962_C_T	6A>T	Substitution	Nonsynonymous coding	13%
MM12T	HSF5	heat shock transcription factor family member 5	CCDS32690.1	chr17_56565518-56565518_G_A	40L>F	Substitution	Nonsynonymous coding	26%
MM12T	HSP90AA1	heat shock protein 90kDa alpha (cytosolic), class A member 1	CCDS32160.1	chr14_102549996-102549996_T_G	580K>T	Substitution	Nonsynonymous coding	35%

MM12T	HSPA13	heat shock protein 70kDa family, member 13	CCDS13567.1	chr21_15747984-15747984_C_A	246R>L	Substitution	Nonsynonymous coding	12%
MM12T	HSPA1A	heat shock 70kDa protein 1A	CCDS34414.1	chr6_31785269-31785269_C_T	579S>L	Substitution	Nonsynonymous coding	41%
MM12T	HSPA1L	heat shock 70kDa protein 1-like	CCDS34413.1	chr6_31778061-31778061_C_A	563K>N	Substitution	Nonsynonymous coding	20%
MM12T	HSPG2	heparan sulfate proteoglycan 2	NM_005529	chr1_22158266-22158266_G_A	3744A>V	Substitution	Nonsynonymous coding	15%
MM12T	HSPG2	heparan sulfate proteoglycan 2	NM_005529	chr1_22160386-22160386_G_A	3585R>C	Substitution	Nonsynonymous coding	29%
MM12T	HSPG2	heparan sulfate proteoglycan 2	NM_005529	chr1_22161303-22161303_C_T	3530R>Q	Substitution	Nonsynonymous coding	17%
MM12T	HSPG2	heparan sulfate proteoglycan 2	NM_005529	chr1_22174529-22174529_C_T	2599V>M	Substitution	Nonsynonymous coding	15%
MM12T	HSPG2	heparan sulfate proteoglycan 2	NM_005529	chr1_22179286-22179286_C_T	2211A>T	Substitution	Nonsynonymous coding	36%
MM12T	HSPH1	heat shock 105kDa/110kDa protein 1	CCDS9340.1	chr13_31722621-31722623_AAA_	NA	Deletion	Splice site acceptor	17%
MM12T	HSPH1	heat shock 105kDa/110kDa protein 1	CCDS9340.1	chr13_31725211-31725211_G_A	261R>X	Substitution	Nonsense	28%
MM12T	HTATSF1	HIV-1 Tat specific factor 1	CCDS14657.1	chrX_135593813-135593813_G_A	637V>I	Substitution	Nonsynonymous coding	12%
MM12T	HTR1A	5-hydroxytryptamine (serotonin) receptor 1A, G protein-coupled	CCDS34168.1	chr5_63256682-63256682_C_T	289A>T	Substitution	Nonsynonymous coding	30%
MM12T	HTR1B	5-hydroxytryptamine (serotonin) receptor 1B, G protein-coupled	CCDS4986.1	chr6_78172993-78172993_G_A	43S>F	Substitution	Nonsynonymous coding	24%
MM12T	HTR2B	5-hydroxytryptamine (serotonin) receptor 2B, G protein-coupled	CCDS2483.1	chr2_231974040-231974040_G_A	213R>C	Substitution	Nonsynonymous coding	44%
MM12T	HTR2B	5-hydroxytryptamine (serotonin) receptor 2B, G protein-coupled	CCDS2483.1	chr2_231978575-231978575_C_T	141A>T	Substitution	Nonsynonymous coding	18%
MM12T	HTR2B	5-hydroxytryptamine (serotonin) receptor 2B, G protein-coupled	CCDS2483.1	chr2_231988139-231988139_T_C	114T>A	Substitution	Nonsynonymous coding	11%
MM12T	HTR3B	5-hydroxytryptamine (serotonin) receptor 3B, ionotropic	CCDS8364.1	chr11_113816854-113816854_G_A	441V>M	Substitution	Nonsynonymous coding	33%
MM12T	HTR3C	5-hydroxytryptamine (serotonin) receptor 3C, ionotropic	CCDS3250.1	chr3_183772623-183772623_G_A	61S>N	Substitution	Nonsynonymous coding	13%
MM12T	HTRA3	HtrA serine peptidase 3	CCDS3400.1	chr4_8288325-8288325_G_A	175G>S	Substitution	Nonsynonymous coding	24%
MM12T	HTT	huntingtin	CCDS43206.1	chr4_3180065-3180065_G_A	1502V>I	Substitution	Nonsynonymous coding	20%
MM12T	HTT	huntingtin	CCDS43206.1	chr4_3235020-3235020_C_T	2799A>V	Substitution	Nonsynonymous coding	12%
MM12T	HUS1B	HUS1 checkpoint homolog b (S. pombe)	CCDS4470.1	chr6_656180-656180_C_T	255W>X	Substitution	Nonsense	11%
MM12T	HUWE1	HECT, UBA and WWE domain containing 1, E3 ubiquitin protein ligase	CCDS35301.1	chrX_53562435-53562435_G_A	4187R>C	Substitution	Nonsynonymous coding	13%
MM12T	HYAL2	hyaluronoglucosaminidase 2	CCDS2818.1	chr3_50355841-50355841_G_A	381P>S	Substitution	Nonsynonymous coding	29%
MM12T	HYDIN	HYDIN, axonemal central pair apparatus protein	CCDS42189.1	chr16_70975661-70975661_G_T	2243A>D	Substitution	Nonsynonymous coding	14%
MM12T	HYLS1	hydrolethalus syndrome 1	CCDS8467.1	chr11_125770101-125770101_C_T	280R>C	Substitution	Nonsynonymous coding	25%
MM12T	HYOU1	hypoxia up-regulated 1	CCDS8408.1	chr11_118919207-118919207_G_A	745A>V	Substitution	Nonsynonymous coding	11%
MM12T	IARS	isoleucyl-tRNA synthetase	CCDS6694.1	chr9_94973153-94973153_G_A	1242P>L	Substitution	Nonsynonymous coding	13%
MM12T	IARS	isoleucyl-tRNA synthetase	CCDS6694.1	chr9_95012539-95012539_G_T	821A>E	Substitution	Nonsynonymous coding	35%
MM12T	ICA1	islet cell autoantigen 1, 69kDa	CCDS34602.1	chr7_8257969-8257969_G_T	182P>Q	Substitution	Nonsynonymous coding	13%
MM12T	ICAM3	intercellular adhesion molecule 3	CCDS12235.1	chr19_10446011-10446011__G	NA	Insertion	Frameshift	23%
MM12T	ICAM3	intercellular adhesion molecule 3	CCDS12235.1	chr19_10446425-10446425_G_A	191R>C	Substitution	Nonsynonymous coding	22%

MM12T	ICAM5	intercellular adhesion molecule 5, telencephalin	CCDS12233.1	chr19_10402876-10402876_A_G	280D>G	Substitution	Nonsynonymous coding	33%
MM12T	IER3IP1	immediate early response 3 interacting protein 1	CCDS11933.1	chr18_44683893-44683893_T_C	36D>G	Substitution	Nonsynonymous coding	30%
MM12T	IER5	immediate early response 5	CCDS1343.1	chr1_181058316-181058316_C_T	93A>V	Substitution	Nonsynonymous coding	28%
MM12T	IFFO1	intermediate filament family orphan 1	CCDS41741.1	chr12_6664930-6664930_G_A	89A>V	Substitution	Nonsynonymous coding	33%
MM12T	IFI35	interferon-induced protein 35	CCDS11450.1	chr17_41164289-41164289_G_T	39D>Y	Substitution	Nonsynonymous coding	12%
MM12T	IFIT1B	interferon-induced protein with tetratricopeptide repeats 1B	CCDS31242.1	chr10_91143630-91143630_G_A	187R>H	Substitution	Nonsynonymous coding	32%
MM12T	IFNGR2	interferon gamma receptor 2 (interferon gamma transducer 1)	CCDS33544.1	chr21_34793851-34793851_G_T	91A>S	Substitution	Nonsynonymous coding	29%
MM12T	IFRD2	interferon-related developmental regulator 2	CCDS46831.1	chr3_50328051-50328051_C_A	104A>S	Substitution	Nonsynonymous coding	28%
MM12T	IFT140	intraflagellar transport 140 homolog (Chlamydomonas)	CCDS10439.1	chr16_1570334-1570334_G_A	1224A>V	Substitution	Nonsynonymous coding	24%
MM12T	IFT172	intraflagellar transport 172 homolog (Chlamydomonas)	CCDS1755.1	chr2_27681692-27681692_G_A	944A>V	Substitution	Nonsynonymous coding	23%
MM12T	IFT172	intraflagellar transport 172 homolog (Chlamydomonas)	CCDS1755.1	chr2_27693827-27693827_C_A	554A>S	Substitution	Nonsynonymous coding	29%
MM12T	IFT80	intraflagellar transport 80 homolog (Chlamydomonas)	CCDS3188.1	chr3_159998594-159998594_C_T	509V>M	Substitution	Nonsynonymous coding	32%
MM12T	IGDCC3	immunoglobulin superfamily, DCC subclass, member 3	CCDS10205.1	chr15_65623825-65623825_G_A	441R>C	Substitution	Nonsynonymous coding	31%
MM12T	IGFALS	insulin-like growth factor binding protein, acid labile subunit	CCDS10446.1	chr16_1840995-1840995_G_A	475A>V	Substitution	Nonsynonymous coding	34%
MM12T	IGFBP5	insulin-like growth factor binding protein 5	CCDS2405.1	chr2_217541593-217541593_G_A	234R>C	Substitution	Nonsynonymous coding	21%
MM12T	IGFBPL1	insulin-like growth factor binding protein-like 1	CCDS35017.1	chr9_38424346-38424346_G_T	26L>I	Substitution	Nonsynonymous coding	39%
MM12T	IGHA2	immunoglobulin heavy constant alpha 2 (A2m marker)	ENST00000390538	chr14_106054556-106054556_G_T	59P>H	Substitution	Nonsynonymous coding	25%
MM12T	IGJ	immunoglobulin J polypeptide, linker protein for immunoglobulin alpha and mu	CCDS3545.1	chr4_71527815-71527815_C_T	61R>Q	Substitution	Nonsynonymous coding	18%
MM12T	IGSF11	immunoglobulin superfamily, member 11	CCDS46891.1	chr3_118624560-118624560_C_T	196V>I	Substitution	Nonsynonymous coding	37%
MM12T	IGSF9	immunoglobulin superfamily, member 9	CCDS44254.1	chr1_159904069-159904069_G_A	339P>S	Substitution	Nonsynonymous coding	12%
MM12T	IHH	Indian hedgehog	CCDS33380.1	chr2_219925069-219925069_G_A	41P>S	Substitution	Nonsynonymous coding	17%
MM12T	IKBKAP	inhibitor of kappa light polypeptide gene enhancer in B-cells, kinase complex-associated	CCDS6773.1	chr9_111640345-111640345_G_A	1262T>M	Substitution	Nonsynonymous coding	29%
MM12T	IKBKAP	inhibitor of kappa light polypeptide gene enhancer in B-cells, kinase complex-associated	CCDS6773.1	chr9_111679862-111679862_G_A	277H>Y	Substitution	Nonsynonymous coding	37%
MM12T	IKBKB	inhibitor of kappa light polypeptide gene enhancer in B-cells, kinase beta	ENST00000379708	chr8_42176969-42176969_G_T	467D>Y	Substitution	Nonsynonymous coding	19%
MM12T	IKZF1	IKAROS family zinc finger 1 (Ikaros)	NM_006060	chr7_50468033-50468033_G_A	423R>H	Substitution	Nonsynonymous coding	13%
MM12T	IKZF2	IKAROS family zinc finger 2 (Helios)	CCDS2395.1	chr2_213921568-213921568_C_A	132R>M	Substitution	Nonsynonymous coding	27%
MM12T	IL10RA	interleukin 10 receptor, alpha	CCDS8388.1	chr11_117864716-117864716_C_A	180F>L	Substitution	Nonsynonymous coding	45%
MM12T	IL10RA	interleukin 10 receptor, alpha	CCDS8388.1	chr11_117869595-117869595_C_T	326P>S	Substitution	Nonsynonymous coding	10%
MM12T	IL15RA	interleukin 15 receptor, alpha	CCDS7074.1	chr10_6001734-6001734_C_T	200G>D	Substitution	Nonsynonymous coding	32%
MM12T	IL17RD	interleukin 17 receptor D	CCDS2880.2	chr3_57144334-57144334_C_T	106E>K	Substitution	Nonsynonymous coding	28%
MM12T	IL17RE	interleukin 17 receptor E	CCDS2589.1	chr3_9944726-9944726_G_A	37R>H	Substitution	Nonsynonymous coding	38%
MM12T	IL1B	interleukin 1, beta	CCDS2102.1	chr2_113588108-113588108_G_A	214R>X	Substitution	Nonsense	35%

MM12T	IL1RAPL1	interleukin 1 receptor accessory protein-like 1	CCDS14218.1	chrX_29973704-29973704_C_T	620R>C	Substitution	Nonsynonymous coding	10%
MM12T	IL1RL1	interleukin 1 receptor-like 1	CCDS2057.1	chr2_102955492-102955492_C_T	86T>I	Substitution	Nonsynonymous coding	14%
MM12T	IL21R	interleukin 21 receptor	CCDS10630.1	chr16_27448930-27448930_G_A	92D>N	Substitution	Nonsynonymous coding	12%
MM12T	IL22RA2	interleukin 22 receptor, alpha 2	CCDS5182.1	chr6_137466864-137466864_G_T	230A>D	Substitution	Nonsynonymous coding	39%
MM12T	IL23R	interleukin 23 receptor	CCDS637.1	chr1_67724549-67724549_C_A	543T>K	Substitution	Nonsynonymous coding	22%
MM12T	IL2RA	interleukin 2 receptor, alpha	CCDS7076.1	chr10_6067971-6067971_G_A	28P>S	Substitution	Nonsynonymous coding	13%
MM12T	IL33	interleukin 33	CCDS6468.1	chr9_6254556-6254556_A_	NA	Deletion	Splice site donor	31%
MM12T	IL4R	interleukin 4 receptor	CCDS10629.1	chr16_27356257-27356257_G_A	93V>M	Substitution	Nonsynonymous coding	27%
MM12T	ILKAP	integrin-linked kinase-associated serine/threonine phosphatase	CCDS2526.1	chr2_239079585-239079585_T_C	346E>G	Substitution	Nonsynonymous coding	30%
MM12T	IMP4	IMP4, U3 small nucleolar ribonucleoprotein, homolog (yeast)	CCDS2160.1	chr2_131103657-131103657_G_A	221A>T	Substitution	Nonsynonymous coding	14%
MM12T	INADL	InaD-like (Drosophila)	CCDS617.2	chr1_62253433-62253433_G_T	286R>I	Substitution	Nonsynonymous coding	13%
MM12T	INADL	InaD-like (Drosophila)	CCDS617.2	chr1_62503695-62503695_C_T	1336P>S	Substitution	Nonsynonymous coding	31%
MM12T	INCA1	inhibitor of CDK, cyclin A1 interacting protein 1	CCDS11064.1	chr17_4892133-4892133_C_T	153E>K	Substitution	Nonsynonymous coding	25%
MM12T	INCENP	inner centromere protein antigens 135/155kDa	CCDS44624.1	chr11_61908285-61908285_G_A	485A>T	Substitution	Nonsynonymous coding	11%
MM12T	INCENP	inner centromere protein antigens 135/155kDa	CCDS44624.1	chr11_61908508-61908508_G_A	529D>N	Substitution	Nonsynonymous coding	12%
MM12T	INO80	INO80 homolog (S. cerevisiae)	CCDS10071.1	chr15_41371957-41371957_C_T	358R>H	Substitution	Nonsynonymous coding	37%
MM12T	INO80C	INO80 complex subunit C	CCDS45853.1	chr18_33048676-33048676_G_A	196R>W	Substitution	Nonsynonymous coding	42%
MM12T	INO80D	INO80 complex subunit D	CCDS46500.1	chr2_206892937-206892937_C_T	399R>Q	Substitution	Nonsynonymous coding	36%
MM12T	INPP1	inositol polyphosphate-1-phosphatase	CCDS2305.1	chr2_191235811-191235811_C_A	295L>I	Substitution	Nonsynonymous coding	39%
MM12T	INPP4A	inositol polyphosphate-4-phosphatase, type I, 107kDa	CCDS46369.1	chr2_99170953-99170953_G_A	ISV+1>	Substitution	Splice site donor	27%
MM12T	INPP5D	inositol polyphosphate-5-phosphatase, 145kDa	ENST00000359570	chr2_234098518-234098518_C_T	794P>L	Substitution	Nonsynonymous coding	22%
MM12T	INPP5E	inositol polyphosphate-5-phosphatase, 72 kDa	CCDS7000.1	chr9_139329190-139329190_A_G	ISV+2>	Substitution	Splice site donor	32%
MM12T	INPP5K	inositol polyphosphate-5-phosphatase K	CCDS11004.1	chr17_1401384-1401384_C_T	270R>H	Substitution	Nonsynonymous coding	21%
MM12T	INPPL1	inositol polyphosphate phosphatase-like 1	CCDS8213.1	chr11_71941426-71941426_C_A	371Q>K	Substitution	Nonsynonymous coding	35%
MM12T	INSIG2	insulin induced gene 2	CCDS2122.1	chr2_118860856-118860856_C_T	110R>W	Substitution	Nonsynonymous coding	36%
MM12T	INSR	insulin receptor	CCDS12176.1	chr19_7117197-7117197_G_A	1340A>V	Substitution	Nonsynonymous coding	30%
MM12T	INSRR	insulin receptor-related receptor	CCDS1160.1	chr1_156816395-156816395_G_A	576R>W	Substitution	Nonsynonymous coding	31%
MM12T	INTS1	integrator complex subunit 1	CCDS47526.1	chr7_1512723-1512723_C_T	2019D>N	Substitution	Nonsynonymous coding	13%
MM12T	INTS1	integrator complex subunit 1	CCDS47526.1	chr7_1535091-1535091_C_T	604A>T	Substitution	Nonsynonymous coding	36%
MM12T	INTS10	integrator complex subunit 10	CCDS6011.2	chr8_19709194-19709194_G_A	671V>M	Substitution	Nonsynonymous coding	23%
MM12T	INTS12	integrator complex subunit 12	CCDS3671.1	chr4_106604107-106604107_C_A	391S>I	Substitution	Nonsynonymous coding	32%
MM12T	INTS12	integrator complex subunit 12	CCDS3671.1	chr4_106613258-106613258_C_T	178E>K	Substitution	Nonsynonymous coding	13%

MM12T	INTS2	integrator complex subunit 2	CCDS45750.1	chr17_59967242-59967242_C_T	638R>H	Substitution	Nonsynonymous coding	14%
MM12T	INTS2	integrator complex subunit 2	CCDS45750.1	chr17_59989401-59989401_C_T	235R>Q	Substitution	Nonsynonymous coding	19%
MM12T	INTS3	integrator complex subunit 3	CCDS1052.1	chr1_153701150-153701150_G_A	14A>T	Substitution	Nonsynonymous coding	37%
MM12T	INTS4	integrator complex subunit 4	CCDS31644.1	chr11_77692559-77692559_C_A	104G>X	Substitution	Nonsense	19%
MM12T	INTS5	integrator complex subunit 5	CCDS8027.1	chr11_62414641-62414641_G_A	971R>W	Substitution	Nonsynonymous coding	13%
MM12T	INTS5	integrator complex subunit 5	CCDS8027.1	chr11_62414836-62414836_C_T	906A>T	Substitution	Nonsynonymous coding	39%
MM12T	INTS6	integrator complex subunit 6	CCDS9428.1	chr13_51948517-51948517_C_T	644R>Q	Substitution	Nonsynonymous coding	36%
MM12T	INVS	inversin	CCDS6746.1	chr9_103046819-103046819_G_A	668G>R	Substitution	Nonsynonymous coding	15%
MM12T	INVS	inversin	CCDS6746.1	chr9_103055001-103055001_C_T	821A>V	Substitution	Nonsynonymous coding	28%
MM12T	IP6K2	inositol hexakisphosphate kinase 2	ENST00000443964	chr3_48732762-48732762_C_T	47R>H	Substitution	Nonsynonymous coding	12%
MM12T	IPO4	importin 4	CCDS9616.1	chr14_24656904-24656904_C_T	126S>N	Substitution	Nonsynonymous coding	34%
MM12T	IPO5	importin 5	CCDS31999.1	chr13_98673302-98673302_T_C	1059I>T	Substitution	Nonsynonymous coding	32%
MM12T	IPP	intracisternal A particle-promoted polypeptide	CCDS30702.1	chr1_46182596-46182596_C_T	427R>H	Substitution	Nonsynonymous coding	16%
MM12T	IPPK	inositol 1,3,4,5,6-pentakisphosphate 2-kinase	CCDS6699.1	chr9_95400400-95400400_G_A	267R>W	Substitution	Nonsynonymous coding	30%
MM12T	IQCB1	IQ motif containing B1	CCDS33837.1	chr3_121544924-121544924_G_C	123Q>E	Substitution	Nonsynonymous coding	13%
MM12T	IQCC	IQ motif containing C	CCDS355.1	chr1_32672702-32672702_C_T	180A>V	Substitution	Nonsynonymous coding	17%
MM12T	IQCE	IQ motif containing E	CCDS43542.1	chr7_2641075-2641075_A_	NA	Deletion	Frameshift	22%
MM12T	IQCE	IQ motif containing E	CCDS43542.1	chr7_2641075-2641075_A_G	537K>E	Substitution	Nonsynonymous coding	13%
MM12T	IQCE	IQ motif containing E	CCDS43542.1	chr7_2641073-2641073_A_C	ISV-2>	Substitution	Splice site acceptor	11%
MM12T	IQCF1	IQ motif containing F1	CCDS2836.1	chr3_51929096-51929096_C_T	143R>H	Substitution	Nonsynonymous coding	29%
MM12T	IQCF2	IQ motif containing F2	CCDS2835.1	chr3_51897136-51897136_C_T	82S>L	Substitution	Nonsynonymous coding	14%
MM12T	IQCF3	IQ motif containing F3	CCDS46837.1	chr3_51864572-51864572_C_T	74R>W	Substitution	Nonsynonymous coding	33%
MM12T	IQCF5	IQ motif containing F5	CCDS46838.1	chr3_51908023-51908023_C_A	58R>M	Substitution	Nonsynonymous coding	36%
MM12T	IQGAP1	IQ motif containing GTPase activating protein 1	CCDS10362.1	chr15_90984899-90984899_A_G	271T>A	Substitution	Nonsynonymous coding	13%
MM12T	IQGAP3	IQ motif containing GTPase activating protein 3	CCDS1144.1	chr1_156498397-156498397_G_A	1526S>F	Substitution	Nonsynonymous coding	26%
MM12T	IQGAP3	IQ motif containing GTPase activating protein 3	CCDS1144.1	chr1_156499985-156499985_C_T	1439R>H	Substitution	Nonsynonymous coding	16%
MM12T	IQSEC1	IQ motif and Sec7 domain 1	CCDS33703.1	chr3_12978121-12978121_C_T	146R>H	Substitution	Nonsynonymous coding	10%
MM12T	IRAK2	interleukin-1 receptor-associated kinase 2	CCDS33697.1	chr3_10276183-10276183_C_T	438S>L	Substitution	Nonsynonymous coding	31%
MM12T	IRAK3	interleukin-1 receptor-associated kinase 3	CCDS8975.1	chr12_66638893-66638893_G_A	389E>K	Substitution	Nonsynonymous coding	19%
MM12T	IREB2	iron-responsive element binding protein 2	CCDS10302.1	chr15_78768569-78768569_T_	NA	Deletion	Frameshift	39%
MM12T	IRF2BP1	interferon regulatory factor 2 binding protein 1	CCDS12678.1	chr19_46388467-46388467_C_T	189R>H	Substitution	Nonsynonymous coding	32%
MM12T	IRF4	interferon regulatory factor 4	CCDS4469.1	chr6_394873-394873_C_A	90P>H	Substitution	Nonsynonymous coding	22%

MM12T	IRF8	interferon regulatory factor 8	CCDS10956.1	chr16_85952190-85952190_G_A	257A>T	Substitution	Nonsynonymous coding	14%
MM12T	IRGQ	immunity-related GTPase family, Q	CCDS33040.1	chr19_44096710-44096710_C_T	447R>Q	Substitution	Nonsynonymous coding	36%
MM12T	IRGQ	immunity-related GTPase family, Q	CCDS33040.1	chr19_44097471-44097471_C_A	193E>D	Substitution	Nonsynonymous coding	31%
MM12T	IRS2	insulin receptor substrate 2	CCDS9510.1	chr13_110434905-110434905_C_T	1166A>T	Substitution	Nonsynonymous coding	11%
MM12T	IRS2	insulin receptor substrate 2	CCDS9510.1	chr13_110434788-110434788_G_A	1205R>X	Substitution	Nonsense	31%
MM12T	IRS4	insulin receptor substrate 4	CCDS14544.1	chrX_107977633-107977633_T_G	648K>Q	Substitution	Nonsynonymous coding	40%
MM12T	IRX1	iroquois homeobox 1	CCDS34132.1	chr5_3600027-3600027_C_T	322T>I	Substitution	Nonsynonymous coding	42%
MM12T	IRX1	iroquois homeobox 1	CCDS34132.1	chr5_3600194-3600194_G_A	378A>T	Substitution	Nonsynonymous coding	34%
MM12T	IRX2	iroquois homeobox 2	CCDS3868.1	chr5_2748698-2748698_G_A	375S>L	Substitution	Nonsynonymous coding	31%
MM12T	IRX2	iroquois homeobox 2	CCDS3868.1	chr5_2749014-2749014_C_T	270D>N	Substitution	Nonsynonymous coding	10%
MM12T	IRX4	iroquois homeobox 4	CCDS3867.1	chr5_1878124-1878124_G_A	507L>F	Substitution	Nonsynonymous coding	31%
MM12T	ISM1	isthmin 1 homolog (zebrafish)	CCDS46579.1	chr20_13279717-13279717_G_A	336D>N	Substitution	Nonsynonymous coding	13%
MM12T	ISOC2	isochorismatase domain containing 2	CCDS12925.1	chr19_55967059-55967059_G_A	98R>W	Substitution	Nonsynonymous coding	29%
MM12T	ISPD	isoprenoid synthase domain containing	CCDS47550.1	chr7_16341066-16341066_G_A	272A>V	Substitution	Nonsynonymous coding	39%
MM12T	ISY1	ISY1 splicing factor homolog (S. cerevisiae)	CCDS43149.1	chr3_128875703-128875703_G_A	47R>X	Substitution	Nonsense	34%
MM12T	ITCH	itchy E3 ubiquitin protein ligase	CCDS13234.1	chr20_33026292-33026292_G_T	179G>X	Substitution	Nonsense	18%
MM12T	ITFG2	integrin alpha FG-GAP repeat containing 2	CCDS8513.1	chr12_2929914-2929914_G_A	191G>R	Substitution	Nonsynonymous coding	33%
MM12T	ITGA10	integrin, alpha 10	CCDS918.1	chr1_145541907-145541907_C_A	1144L>M	Substitution	Nonsynonymous coding	25%
MM12T	ITGA11	integrin, alpha 11	CCDS45291.1	chr15_68657089-68657089_G_T	105L>I	Substitution	Nonsynonymous coding	23%
MM12T	ITGA2	integrin, alpha 2 (CD49B, alpha 2 subunit of VLA-2 receptor)	CCDS3957.1	chr5_52355728-52355728_G_A	400G>R	Substitution	Nonsynonymous coding	35%
MM12T	ITGA2	integrin, alpha 2 (CD49B, alpha 2 subunit of VLA-2 receptor)	CCDS3957.1	chr5_52356876-52356876_G_T	486Q>H	Substitution	Nonsynonymous coding	17%
MM12T	ITGA2	integrin, alpha 2 (CD49B, alpha 2 subunit of VLA-2 receptor)	CCDS3957.1	chr5_52370943-52370943_C_T	906A>V	Substitution	Nonsynonymous coding	15%
MM12T	ITGA2	integrin, alpha 2 (CD49B, alpha 2 subunit of VLA-2 receptor)	CCDS3957.1	chr5_52358762-52358763_AA_	NA	Deletion	Splice site donor	22%
MM12T	ITGA5	integrin, alpha 5 (fibronectin receptor, alpha polypeptide)	CCDS8880.1	chr12_54798672-54798672_G_A	411A>V	Substitution	Nonsynonymous coding	37%
MM12T	ITGA6	integrin, alpha 6	ENST00000264106	chr2_173337534-173337534_G_T	270D>Y	Substitution	Nonsynonymous coding	22%
MM12T	ITGA9	integrin, alpha 9	CCDS2669.1	chr3_37512557-37512557_G_T	82G>V	Substitution	Nonsynonymous coding	26%
MM12T	ITGAD	integrin, alpha D	CCDS32438.1	chr16_31422687-31422687_G_A	519R>H	Substitution	Nonsynonymous coding	28%
MM12T	ITGAD	integrin, alpha D	CCDS32438.1	chr16_31435510-31435510_T_G	1083F>V	Substitution	Nonsynonymous coding	29%
MM12T	ITGAD	integrin, alpha D	CCDS32438.1	chr16_31435516-31435516_A_G	1085R>G	Substitution	Nonsynonymous coding	36%
MM12T	ITGAL	integrin, alpha L (antigen CD11A (p180), lymphocyte function-associated antigen 1;	CCDS32433.1	chr16_30485582-30485582_A_G	43R>G	Substitution	Nonsynonymous coding	29%
MM12T	ITGAL	integrin, alpha L (antigen CD11A (p180), lymphocyte function-associated antigen 1;	CCDS32433.1	chr16_30500582-30500582_C_T	363A>V	Substitution	Nonsynonymous coding	33%
MM12T	ITGAL	integrin, alpha L (antigen CD11A (p180), lymphocyte function-associated antigen 1;	CCDS32433.1	chr16_30531217-30531217_C_T	1090L>F	Substitution	Nonsynonymous coding	11%

MM12T	ITGAM	integrin, alpha M (complement component 3 receptor 3 subunit)	CCDS45470.1	chr16_31277168-31277168_C_T	92S>F	Substitution	Nonsynonymous coding	30%
MM12T	ITGB1BP2	integrin beta 1 binding protein (melusin) 2	CCDS14411.1	chrX_70523685-70523685_G_A	188G>D	Substitution	Nonsynonymous coding	18%
MM12T	ITGB3	integrin, beta 3 (platelet glycoprotein IIIa, antigen CD61)	CCDS11511.1	chr17_45369758-45369758_G_A	505R>H	Substitution	Nonsynonymous coding	11%
MM12T	ITIH2	inter-alpha-trypsin inhibitor heavy chain 2	CCDS31141.1	chr10_7745410-7745410_A_T	5T>S	Substitution	Nonsynonymous coding	11%
MM12T	ITIH5	inter-alpha-trypsin inhibitor heavy chain family, member 5	CCDS31139.1	chr10_7621961-7621961_C_T	392G>D	Substitution	Nonsynonymous coding	28%
MM12T	ITIH6	inter-alpha-trypsin inhibitor heavy chain family, member 6	CCDS14361.1	chrX_54786350-54786350_G_A	305A>V	Substitution	Nonsynonymous coding	22%
MM12T	ITIH6	inter-alpha-trypsin inhibitor heavy chain family, member 6	CCDS14361.1	chrX_54817273-54817273_C_A	205A>S	Substitution	Nonsynonymous coding	24%
MM12T	ITM2A	integral membrane protein 2A	CCDS14444.1	chrX_78618523-78618523_C_A	119E>D	Substitution	Nonsynonymous coding	31%
MM12T	ITPR1	inositol 1,4,5-trisphosphate receptor, type 1	CCDS46740.1	chr3_4821252-4821252_C_T	2056L>F	Substitution	Nonsynonymous coding	13%
MM12T	ITPR1	inositol 1,4,5-trisphosphate receptor, type 1	CCDS46740.1	chr3_4703895-4703895_G_T	461E>X	Substitution	Nonsense	36%
MM12T	ITPR3	inositol 1,4,5-trisphosphate receptor, type 3	CCDS4783.1	chr6_33631323-33631323_G_A	334G>E	Substitution	Nonsynonymous coding	21%
MM12T	ITPR3	inositol 1,4,5-trisphosphate receptor, type 3	CCDS4783.1	chr6_33656438-33656438_C_T	2192P>S	Substitution	Nonsynonymous coding	41%
MM12T	ITPRIP	inositol 1,4,5-trisphosphate receptor interacting protein	CCDS7557.1	chr10_106074332-106074332_G_A	493A>V	Substitution	Nonsynonymous coding	21%
MM12T	ITPRIP	inositol 1,4,5-trisphosphate receptor interacting protein	CCDS7557.1	chr10_106074840-106074840_C_T	324A>T	Substitution	Nonsynonymous coding	35%
MM12T	ITSN2	intersectin 2	CCDS1710.2	chr2_24426500-24426500_G_T	1697L>M	Substitution	Nonsynonymous coding	29%
MM12T	ITSN2	intersectin 2	CCDS1710.2	chr2_24431142-24431142_C_T	1548D>N	Substitution	Nonsynonymous coding	11%
MM12T	ITSN2	intersectin 2	CCDS1710.2	chr2_24521687-24521687_A_	NA	Deletion	Splice site acceptor	20%
MM12T	JAK1	Janus kinase 1	CCDS41346.1	chr1_65335121-65335121_G_A	174R>X	Substitution	Nonsense	14%
MM12T	JAK3	Janus kinase 3	CCDS12366.1	chr19_17950394-17950394_G_A	445R>X	Substitution	Nonsense	37%
MM12T	JAKMIP1	janus kinase and microtubule interacting protein 1	CCDS47005.1	chr4_6050607-6050607_T_C	669T>A	Substitution	Nonsynonymous coding	28%
MM12T	JAKMIP1	janus kinase and microtubule interacting protein 1	CCDS47005.1	chr4_6087332-6087332_G_A	217R>C	Substitution	Nonsynonymous coding	15%
MM12T	JAKMIP2	janus kinase and microtubule interacting protein 2	CCDS4285.1	chr5_147030094-147030094_G_A	215A>V	Substitution	Nonsynonymous coding	13%
MM12T	JAKMIP3	Janus kinase and microtubule interacting protein 3	CCDS44494.1	chr10_133955443-133955443_C_T	498T>M	Substitution	Nonsynonymous coding	34%
MM12T	JARID2	jumonji, AT rich interactive domain 2	CCDS4533.1	chr6_15497029-15497029_C_T	525R>C	Substitution	Nonsynonymous coding	14%
MM12T	JARID2	jumonji, AT rich interactive domain 2	CCDS4533.1	chr6_15513614-15513614_G_T	1137R>S	Substitution	Nonsynonymous coding	14%
MM12T	JHDM1D	jumonji C domain containing histone demethylase 1 homolog D (S. cerevisiae)	CCDS43658.1	chr7_139826600-139826600_G_T	242P>H	Substitution	Nonsynonymous coding	27%
MM12T	JKAMP	JNK1/MAPK8-associated membrane protein	CCDS45116.1	chr14_59961929-59961929_G_A	149A>T	Substitution	Nonsynonymous coding	32%
MM12T	JMJD4	jumonji domain containing 4	CCDS1561.1	chr1_227920667-227920667_C_T	341R>H	Substitution	Nonsynonymous coding	32%
MM12T	JMJD7-PLA2G4B	JMJD7-PLA2G4B readthrough	CCDS32202.1	chr15_42133048-42133048_C_T	330A>V	Substitution	Nonsynonymous coding	17%
MM12T	JMJD7-PLA2G4B	JMJD7-PLA2G4B readthrough	CCDS32202.1	chr15_42134427-42134427_G_A	451A>T	Substitution	Nonsynonymous coding	34%
MM12T	JRK	jerky homolog (mouse) [Source:HGNC Symbol;Acc:6199]	ENST00000430360	chr8_143747243-143747243_G_A	79R>C	Substitution	Nonsynonymous coding	12%
MM12T	KAL1	Kallmann syndrome 1 sequence	CCDS14130.1	chrX_8504825-8504825_G_C	536C>W	Substitution	Nonsynonymous coding	38%

MM12T	KALRN	kalirin, RhoGEF kinase	CCDS3027.1	chr3_124132426-124132426_G_A	817R>Q	Substitution	Nonsynonymous coding	33%
MM12T	KALRN	kalirin, RhoGEF kinase	CCDS3028.1	chr3_124351314-124351314_G_A	45V>M	Substitution	Nonsynonymous coding	16%
MM12T	KANSL2	KAT8 regulatory NSL complex subunit 2	CCDS44869.1	chr12_49054403-49054403_C_A	ISV-1>	Substitution	Splice site acceptor	27%
MM12T	KANSL3	KAT8 regulatory NSL complex subunit 3	CCDS46361.1	chr2_97271186-97271186_G_A	602R>X	Substitution	Nonsense	16%
MM12T	KARS	lysyl-tRNA synthetase	CCDS45532.1	chr16_75674232-75674232_G_A	108R>C	Substitution	Nonsynonymous coding	10%
MM12T	KAT2B	K(lysine) acetyltransferase 2B	CCDS2634.1	chr3_20141399-20141399_G_A	208G>S	Substitution	Nonsynonymous coding	21%
MM12T	KAT6B	K(lysine) acetyltransferase 6B	CCDS7345.1	chr10_76789746-76789746_A_G	1722T>A	Substitution	Nonsynonymous coding	37%
MM12T	KATNB1	katanin p80 (WD repeat containing) subunit B 1	CCDS10788.1	chr16_57787330-57787330_G_A	359R>H	Substitution	Nonsynonymous coding	12%
MM12T	KAZN	kazrin, periplakin interacting protein	CCDS152.2	chr1_15430562-15430562_T_C	642V>A	Substitution	Nonsynonymous coding	31%
MM12T	KBTBD2	kelch repeat and BTB (POZ) domain containing 2	CCDS34614.1	chr7_32909708-32909708_G_A	374S>F	Substitution	Nonsynonymous coding	10%
MM12T	KBTBD3	kelch repeat and BTB (POZ) domain containing 3	CCDS8334.1	chr11_105924700-105924700_T_C	239E>G	Substitution	Nonsynonymous coding	19%
MM12T	KBTBD6	kelch repeat and BTB (POZ) domain containing 6	CCDS9376.1	chr13_41705170-41705170_C_T	493R>H	Substitution	Nonsynonymous coding	30%
MM12T	KBTBD6	kelch repeat and BTB (POZ) domain containing 6	CCDS9376.1	chr13_41705386-41705386_C_T	421R>H	Substitution	Nonsynonymous coding	33%
MM12T	KCNA1	potassium voltage-gated channel, shaker-related subfamily, member 1 (episodic ataxia	CCDS8535.1	chr12_5020587-5020587_G_A	15A>T	Substitution	Nonsynonymous coding	34%
MM12T	KCNA4	potassium voltage-gated channel, shaker-related subfamily, member 4	CCDS41629.1	chr11_30033012-30033012_T_C	405N>S	Substitution	Nonsynonymous coding	24%
MM12T	KCNAB2	potassium voltage-gated channel, shaker-related subfamily, beta member 2	CCDS55.1	chr1_6154549-6154549_C_T	ISV+4>	Substitution	Splice site donor	25%
MM12T	KCNB2	potassium voltage-gated channel, Shab-related subfamily, member 2	CCDS6209.1	chr8_73848788-73848788_C_A	400L>M	Substitution	Nonsynonymous coding	40%
MM12T	KCNC1	potassium voltage-gated channel, Shaw-related subfamily, member 1	CCDS44547.1	chr11_17757695-17757695_A_G	49D>G	Substitution	Nonsynonymous coding	14%
MM12T	KCNC1	potassium voltage-gated channel, Shaw-related subfamily, member 1	CCDS44547.1	chr11_17757923-17757923_C_T	125A>V	Substitution	Nonsynonymous coding	40%
MM12T	KCNC2	potassium voltage-gated channel, Shaw-related subfamily, member 2	CCDS9007.1	chr12_75444637-75444637_A_	NA	Deletion	Frameshift	24%
MM12T	KCNC3	potassium voltage-gated channel, Shaw-related subfamily, member 3	CCDS12793.1	chr19_50827047-50827047_G_A	388A>V	Substitution	Nonsynonymous coding	11%
MM12T	KCND1	potassium voltage-gated channel, Shal-related subfamily, member 1	CCDS14314.1	chrX_48826278-48826278_T_C	134E>G	Substitution	Nonsynonymous coding	36%
MM12T	KCNF1	potassium voltage-gated channel, subfamily F, member 1	CCDS1676.1	chr2_11053711-11053711_G_A	387A>T	Substitution	Nonsynonymous coding	30%
MM12T	KCNG1	potassium voltage-gated channel, subfamily G, member 1	CCDS13436.1	chr20_49626514-49626514_G_A	121P>L	Substitution	Nonsynonymous coding	32%
MM12T	KCNG2	potassium voltage-gated channel, subfamily G, member 2	CCDS12019.1	chr18_77623891-77623891_C_T	75P>L	Substitution	Nonsynonymous coding	39%
MM12T	KCNG2	potassium voltage-gated channel, subfamily G, member 2	CCDS12019.1	chr18_77623948-77623948_G_A	94R>Q	Substitution	Nonsynonymous coding	21%
MM12T	KCNG2	potassium voltage-gated channel, subfamily G, member 2	CCDS12019.1	chr18_77659208-77659208_T_A	265S>T	Substitution	Nonsynonymous coding	39%
MM12T	KCNG4	potassium voltage-gated channel, subfamily G, member 4	CCDS10945.1	chr16_84270389-84270389_C_T	235A>T	Substitution	Nonsynonymous coding	31%
MM12T	KCNH1	potassium voltage-gated channel, subfamily H (eag-related), member 1	CCDS1496.1	chr1_211192294-211192294_C_T	288R>H	Substitution	Nonsynonymous coding	12%
MM12T	KCNH2	potassium voltage-gated channel, subfamily H (eag-related), member 2	CCDS5910.1	chr7_150644551-150644551_C_T	1006G>D	Substitution	Nonsynonymous coding	16%
MM12T	KCNH3	potassium voltage-gated channel, subfamily H (eag-related), member 3	CCDS8786.1	chr12_49943259-49943259_G_A	502A>T	Substitution	Nonsynonymous coding	13%
MM12T	KCNH4	potassium voltage-gated channel, subfamily H (eag-related), member 4	CCDS11420.1	chr17_40315939-40315939_G_A	721T>M	Substitution	Nonsynonymous coding	31%

MM12T	KCNH4	potassium voltage-gated channel, subfamily H (eag-related), member 4	CCDS11420.1	chr17_40321647-40321647_G_A	480R>C	Substitution	Nonsynonymous coding	29%
MM12T	KCNH6	potassium voltage-gated channel, subfamily H (eag-related), member 6	CCDS11638.1	chr17_61607793-61607793_G_A	189V>M	Substitution	Nonsynonymous coding	22%
MM12T	KCNH7	potassium voltage-gated channel, subfamily H (eag-related), member 7	CCDS2219.1	chr2_163693235-163693235_G_A	40A>V	Substitution	Nonsynonymous coding	21%
MM12T	KCNH8	potassium voltage-gated channel, subfamily H (eag-related), member 8	CCDS2632.1	chr3_19479714-19479714_G_	NA	Deletion	Frameshift	23%
MM12T	KCNIP1	Kv channel interacting protein 1	CCDS34286.1	chr5_169931593-169931593_G_A	6G>D	Substitution	Nonsynonymous coding	18%
MM12T	KCNJ11	potassium inwardly-rectifying channel, subfamily J, member 11	CCDS31436.1	chr11_17408638-17408638_C_T	334G>D	Substitution	Nonsynonymous coding	27%
MM12T	KCNJ16	potassium inwardly-rectifying channel, subfamily J, member 16	CCDS11687.1	chr17_68128871-68128871_A_C	215T>P	Substitution	Nonsynonymous coding	33%
MM12T	KCNJ3	potassium inwardly-rectifying channel, subfamily J, member 3	CCDS2200.1	chr2_155555414-155555414_C_T	43R>W	Substitution	Nonsynonymous coding	19%
MM12T	KCNJ3	potassium inwardly-rectifying channel, subfamily J, member 3	CCDS2200.1	chr2_155555690-155555690_C_A	135L>I	Substitution	Nonsynonymous coding	27%
MM12T	KCNK10	potassium channel, subfamily K, member 10	CCDS9881.1	chr14_88729854-88729854_C_T	32A>T	Substitution	Nonsynonymous coding	35%
MM12T	KCNK17	potassium channel, subfamily K, member 17	CCDS4842.1	chr6_39271856-39271856_G_T	189L>I	Substitution	Nonsynonymous coding	23%
MM12T	KCNK5	potassium channel, subfamily K, member 5	CCDS4841.1	chr6_39163672-39163672_G_A	93A>V	Substitution	Nonsynonymous coding	16%
MM12T	KCNN2	potassium intermediate/small conductance calcium-activated channel, subfamily N,	CCDS4114.1	chr5_113829168-113829168_A_T	484K>M	Substitution	Nonsynonymous coding	14%
MM12T	KCNN3	potassium intermediate/small conductance calcium-activated channel, subfamily N,	CCDS30880.1	chr1_154841641-154841641_C_T	267G>D	Substitution	Nonsynonymous coding	29%
MM12T	KCNQ1	potassium voltage-gated channel, KQT-like subfamily, member 1	CCDS7736.1	chr11_2608803-2608803_G_A	378A>T	Substitution	Nonsynonymous coding	14%
MM12T	KCNQ3	potassium voltage-gated channel, KQT-like subfamily, member 3	CCDS34943.1	chr8_133141909-133141909_G_A	740T>M	Substitution	Nonsynonymous coding	34%
MM12T	KCNQ3	potassium voltage-gated channel, KQT-like subfamily, member 3	CCDS34943.1	chr8_133153549-133153549_C_T	431R>H	Substitution	Nonsynonymous coding	25%
MM12T	KCNV2	potassium channel, subfamily V, member 2	CCDS6447.1	chr9_2718511-2718511_G_A	258A>T	Substitution	Nonsynonymous coding	35%
MM12T	KCNV2	potassium channel, subfamily V, member 2	CCDS6447.1	chr9_2718566-2718566_C_T	276A>V	Substitution	Nonsynonymous coding	12%
MM12T	KCTD3	potassium channel tetramerisation domain containing 3	CCDS1515.1	chr1_215792322-215792322_C_T	553R>C	Substitution	Nonsynonymous coding	41%
MM12T	KCTD7	potassium channel tetramerisation domain containing 7	CCDS5534.1	chr7_66103383-66103383_G_A	153R>H	Substitution	Nonsynonymous coding	12%
MM12T	KDELR1	KDEL (Lys-Asp-Glu-Leu) endoplasmic reticulum protein retention receptor 1	CCDS12718.1	chr19_48887630-48887630_G_A	154A>V	Substitution	Nonsynonymous coding	33%
MM12T	KDM2B	lysine (K)-specific demethylase 2B	CCDS41850.1	chr12_121877786-121877786_G_A	1235R>C	Substitution	Nonsynonymous coding	39%
MM12T	KDM3B	lysine (K)-specific demethylase 3B	CCDS34242.1	chr5_137715358-137715358_G_T	222Q>H	Substitution	Nonsynonymous coding	29%
MM12T	KDM4B	lysine (K)-specific demethylase 4B	CCDS12138.1	chr19_5144150-5144150_C_T	908S>L	Substitution	Nonsynonymous coding	33%
MM12T	KDM5A	lysine (K)-specific demethylase 5A	CCDS41736.1	chr12_493313-493313_T_C	84T>A	Substitution	Nonsynonymous coding	37%
MM12T	KDM5C	lysine (K)-specific demethylase 5C	CCDS14351.1	chrX_53228289-53228289_G_A	705R>C	Substitution	Nonsynonymous coding	36%
MM12T	KDM5C	lysine (K)-specific demethylase 5C	CCDS14351.1	chrX_53239938-53239938_C_T	501W>X	Substitution	Nonsense	28%
MM12T	KDM6A	lysine (K)-specific demethylase 6A	CCDS14265.1	chrX_44966740-44966740_C_T	1322R>W	Substitution	Nonsynonymous coding	16%
MM12T	KDR	kinase insert domain receptor (a type III receptor tyrosine kinase)	CCDS3497.1	chr4_55968130-55968130_G_A	734L>F	Substitution	Nonsynonymous coding	13%
MM12T	KDSR	3-ketodihydrosphingosine reductase	CCDS11982.1	chr18_61018243-61018243_G_A	163R>C	Substitution	Nonsynonymous coding	28%
MM12T	KEAP1	kelch-like ECH-associated protein 1	CCDS12239.1	chr19_10602359-10602359_C_T	407A>T	Substitution	Nonsynonymous coding	24%

MM12T	KEL	Kell blood group, metallo-endopeptidase	CCDS34766.1	chr7_142658194-142658194_G_A	ISV-3>	Substitution	Splice site acceptor	39%
MM12T	KHDRBS1	KH domain containing, RNA binding, signal transduction associated 1	CCDS350.1	chr1_32495991-32495991_C_T	159R>X	Substitution	Nonsense	27%
MM12T	KHNYN	KH and NYN domain containing	CCDS32058.1	chr14_24900756-24900756_G_T	97G>C	Substitution	Nonsynonymous coding	29%
MM12T	KHSRP	KH-type splicing regulatory protein	CCDS45936.1	chr19_6415433-6415433_G_A	642Q>X	Substitution	Nonsense	12%
MM12T	KIAA0100	KIAA0100	CCDS32595.1	chr17_26960637-26960637_C_T	1132S>N	Substitution	Nonsynonymous coding	36%
MM12T	KIAA0141	KIAA0141	CCDS4268.1	chr5_141309152-141309152_C_T	140R>X	Substitution	Nonsense	21%
MM12T	KIAA0146	KIAA0146	CCDS43737.1	chr8_48511707-48511707_C_T	498T>I	Substitution	Nonsynonymous coding	34%
MM12T	KIAA0195	KIAA0195	CCDS32732.1	chr17_73482013-73482013_G_T	69G>V	Substitution	Nonsynonymous coding	33%
MM12T	KIAA0195	KIAA0195	CCDS32732.1	chr17_73485732-73485732_C_A	315L>I	Substitution	Nonsynonymous coding	35%
MM12T	KIAA0195	KIAA0195	CCDS32732.1	chr17_73487170-73487170_T_C	390L>P	Substitution	Nonsynonymous coding	25%
MM12T	KIAA0195	KIAA0195	CCDS32732.1	chr17_73491371-73491371_G_A	912R>Q	Substitution	Nonsynonymous coding	21%
MM12T	KIAA0196	KIAA0196	CCDS6355.1	chr8_126056330-126056330_G_T	892L>I	Substitution	Nonsynonymous coding	28%
MM12T	KIAA0226	KIAA0226	CCDS43195.1	chr3_197427592-197427592_G_A	385R>C	Substitution	Nonsynonymous coding	30%
MM12T	KIAA0232	KIAA0232	CCDS43209.1	chr4_6865672-6865672_C_T	1188A>V	Substitution	Nonsynonymous coding	19%
MM12T	KIAA0430	KIAA0430	CCDS10562.2	chr16_15727651-15727651__G	NA	Insertion	Frameshift	29%
MM12T	KIAA0556	KIAA0556	CCDS32415.1	chr16_27786311-27786311_G_A	1452G>D	Substitution	Nonsynonymous coding	33%
MM12T	KIAA0753	KIAA0753	CCDS42247.1	chr17_6524252-6524252_G_A	391R>W	Substitution	Nonsynonymous coding	29%
MM12T	KIAA0825	KIAA0825	NM_001145678	chr5_93731843-93731843_G_A	1087R>C	Substitution	Nonsynonymous coding	29%
MM12T	KIAA0907	KIAA0907	CCDS30885.1	chr1_155893481-155893481_G_A	ISV-3>	Substitution	Splice site acceptor	34%
MM12T	KIAA1009	KIAA1009	CCDS34494.2	chr6_84872875-84872875_C_T	834A>T	Substitution	Nonsynonymous coding	22%
MM12T	KIAA1109	KIAA1109	CCDS43267.1	chr4_123183995-123183995_C_T	2280A>V	Substitution	Nonsynonymous coding	19%
MM12T	KIAA1109	KIAA1109	CCDS43267.1	chr4_123109047-123109047_T_A	ISV-3>	Substitution	Splice site acceptor	33%
MM12T	KIAA1161	KIAA1161	ENST00000297625	chr9_34372347-34372347_G_A	165R>C	Substitution	Nonsynonymous coding	32%
MM12T	KIAA1199	KIAA1199	CCDS10315.1	chr15_81188327-81188327_A_G	446Y>C	Substitution	Nonsynonymous coding	16%
MM12T	KIAA1211	KIAA1211	CCDS43230.1	chr4_57181628-57181628_C_A	654P>T	Substitution	Nonsynonymous coding	32%
MM12T	KIAA1211	KIAA1211	CCDS43230.1	chr4_57181772-57181772_C_T	702R>W	Substitution	Nonsynonymous coding	16%
MM12T	KIAA1211L	KIAA1211-like	CCDS42720.1	chr2_99438905-99438905_C_T	611A>T	Substitution	Nonsynonymous coding	38%
MM12T	KIAA1211L	KIAA1211-like	CCDS42720.1	chr2_99439465-99439465_G_A	424A>V	Substitution	Nonsynonymous coding	33%
MM12T	KIAA1211L	KIAA1211-like	CCDS42720.1	chr2_99439804-99439804_C_T	311R>H	Substitution	Nonsynonymous coding	14%
MM12T	KIAA1217	KIAA1217	CCDS31165.1	chr10_24809089-24809089_T_G	739S>A	Substitution	Nonsynonymous coding	17%
MM12T	KIAA1244	KIAA1244	CCDS5189.2	chr6_138584623-138584623_C_T	668A>V	Substitution	Nonsynonymous coding	20%
MM12T	KIAA1279	KIAA1279	CCDS7284.1	chr10_70775815-70775815__A	NA	Insertion	Frameshift	15%

MM12T	KIAA1324L	KIAA1324-like	CCDS47632.1	chr7_86574345-86574345_C_T	175R>H	Substitution	Nonsynonymous coding	36%
MM12T	KIAA1429	KIAA1429	CCDS34923.1	chr8_95501010-95501010_C_T	1788G>D	Substitution	Nonsynonymous coding	26%
MM12T	KIAA1549	KIAA1549	CCDS47723.1	chr7_138596000-138596000_C_T	963V>I	Substitution	Nonsynonymous coding	10%
MM12T	KIAA1614	KIAA1614	CCDS41442.1	chr1_180897616-180897616_C_T	371A>V	Substitution	Nonsynonymous coding	32%
MM12T	KIAA1644	KIAA1644	CCDS43025.1	chr22_44696786-44696786_C_T	14V>I	Substitution	Nonsynonymous coding	13%
MM12T	KIAA1683	KIAA1683	CCDS46017.1	chr19_18377627-18377627_C_A	241Q>H	Substitution	Nonsynonymous coding	14%
MM12T	KIAA1731	KIAA1731	CCDS44708.1	chr11_93430347-93430347_G_A	757A>T	Substitution	Nonsynonymous coding	11%
MM12T	KIAA1731	KIAA1731	CCDS44708.1	chr11_93432841-93432841_G_T	1588R>I	Substitution	Nonsynonymous coding	12%
MM12T	KIAA1731	KIAA1731	CCDS44708.1	chr11_93436042-93436042_A_G	1851I>M	Substitution	Nonsynonymous coding	11%
MM12T	KIAA1755	KIAA1755	CCDS33467.1	chr20_36841598-36841598_C_T	1150R>H	Substitution	Nonsynonymous coding	21%
MM12T	KIAA1958	KIAA1958	CCDS35108.1	chr9_115337082-115337082_A_G	241H>R	Substitution	Nonsynonymous coding	38%
MM12T	KIAA2022	KIAA2022	CCDS35337.1	chrX_73960698-73960698_T_C	1232I>V	Substitution	Nonsynonymous coding	36%
MM12T	KIAA2022	KIAA2022	CCDS35337.1	chrX_73961202-73961202_G_A	1064R>C	Substitution	Nonsynonymous coding	25%
MM12T	KIF11	kinesin family member 11	CCDS7422.1	chr10_94408036-94408036_C_T	872A>V	Substitution	Nonsynonymous coding	11%
MM12T	KIF12	kinesin family member 12	CCDS6801.1	chr9_116855607-116855607_G_A	362P>S	Substitution	Nonsynonymous coding	27%
MM12T	KIF13B	kinesin family member 13B	NM_015254	chr8_28929413-28929413_C_T	1648V>I	Substitution	Nonsynonymous coding	27%
MM12T	KIF13B	kinesin family member 13B	NM_015254	chr8_28980935-28980935_C_T	1143A>T	Substitution	Nonsynonymous coding	25%
MM12T	KIF1A	kinesin family member 1A	CCDS46561.1	chr2_241666352-241666352_A_C	1237F>C	Substitution	Nonsynonymous coding	47%
MM12T	KIF1A	kinesin family member 1A	ENST00000373308	chr2_241696785-241696785_C_T	937G>S	Substitution	Nonsynonymous coding	23%
MM12T	KIF1B	kinesin family member 1B	CCDS111.1	chr1_10327507-10327507_C_T	167R>C	Substitution	Nonsynonymous coding	24%
MM12T	KIF1C	kinesin family member 1C	CCDS11065.1	chr17_4925544-4925544_G_A	723R>H	Substitution	Nonsynonymous coding	28%
MM12T	KIF20B	kinesin family member 20B	CCDS7407.1	chr10_91483748-91483748__A	NA	Insertion	Frameshift	29%
MM12T	KIF21A	kinesin family member 21A	CCDS31773.1	chr12_39734809-39734809_C_T	657R>K	Substitution	Nonsynonymous coding	22%
MM12T	KIF21A	kinesin family member 21A	CCDS31773.1	chr12_39735909-39735909_T_C	564D>G	Substitution	Nonsynonymous coding	19%
MM12T	KIF21B	kinesin family member 21B	CCDS30965.1	chr1_200954050-200954050_C_T	1247R>H	Substitution	Nonsynonymous coding	14%
MM12T	KIF21B	kinesin family member 21B	CCDS30965.1	chr1_200978078-200978078_G_A	89T>M	Substitution	Nonsynonymous coding	27%
MM12T	KIF22	kinesin family member 22	CCDS10653.1	chr16_29814110-29814110_G_A	434S>N	Substitution	Nonsynonymous coding	37%
MM12T	KIF26B	kinesin family member 26B	CCDS44342.1	chr1_245848720-245848720_C_T	812S>L	Substitution	Nonsynonymous coding	33%
MM12T	KIF26B	kinesin family member 26B	CCDS44342.1	chr1_245849098-245849098_G_A	938G>D	Substitution	Nonsynonymous coding	38%
MM12T	KIF26B	kinesin family member 26B	CCDS44342.1	chr1_245850940-245850940_G_A	1552S>N	Substitution	Nonsynonymous coding	31%
MM12T	KIF26B	kinesin family member 26B	CCDS44342.1	chr1_245861434-245861434_G_T	1951A>S	Substitution	Nonsynonymous coding	28%
MM12T	KIF27	kinesin family member 27	CCDS6665.1	chr9_86517974-86517974_C_T	ISV+1>	Substitution	Splice site donor	22%

MM12T	KIF3C	kinesin family member 3C	CCDS1719.1	chr2_26203558-26203558_C_T	410R>H	Substitution	Nonsynonymous coding	28%
MM12T	KIF4A	kinesin family member 4A	CCDS14401.1	chrX_69573637-69573637_C_A	552P>T	Substitution	Nonsynonymous coding	11%
MM12T	KIF5A	kinesin family member 5A	CCDS8945.1	chr12_57957469-57957469_C_T	95T>I	Substitution	Nonsynonymous coding	21%
MM12T	KIF5C	kinesin family member 5C	NM_004522	chr2_149803536-149803536_A_C	238K>T	Substitution	Nonsynonymous coding	21%
MM12T	KIF6	kinesin family member 6	CCDS4844.1	chr6_39507966-39507966_C_A	486K>N	Substitution	Nonsynonymous coding	21%
MM12T	KIF7	kinesin family member 7	CCDS32325.2	chr15_90192289-90192289_A_G	280L>P	Substitution	Nonsynonymous coding	38%
MM12T	KIFAP3	kinesin-associated protein 3	CCDS1288.1	chr1_169947226-169947226_G_A	633Q>X	Substitution	Nonsense	42%
MM12T	KIN	KIN, antigenic determinant of recA protein homolog (mouse)	CCDS7080.1	chr10_7804523-7804523_T_C	ISV-4>	Substitution	Splice site acceptor	27%
MM12T	KIRREL3	kin of IRRE like 3 (Drosophila)	NM_032531	chr11_126343265-126343265_G_T	177P>H	Substitution	Nonsynonymous coding	23%
MM12T	KIT	v-kit Hardy-Zuckerman 4 feline sarcoma viral oncogene homolog	CCDS3496.1	chr4_55561875-55561875_G_T	89A>S	Substitution	Nonsynonymous coding	23%
MM12T	KLC1	kinesin light chain 1	CCDS45168.1	chr14_104145821-104145821_T_A	530V>D	Substitution	Nonsynonymous coding	28%
MM12T	KLC2	kinesin light chain 2	CCDS8130.1	chr11_66031910-66031910_T_C	404V>A	Substitution	Nonsynonymous coding	13%
MM12T	KLC3	kinesin light chain 3	CCDS12660.2	chr19_45852074-45852074_G_A	ISV-4>	Substitution	Splice site acceptor	31%
MM12T	KLF10	Kruppel-like factor 10	CCDS6294.1	chr8_103663484-103663484_G_T	359P>H	Substitution	Nonsynonymous coding	38%
MM12T	KLF15	Kruppel-like factor 15	CCDS3036.1	chr3_126070822-126070822_G_A	315A>V	Substitution	Nonsynonymous coding	34%
MM12T	KLF4	Kruppel-like factor 4 (gut)	CCDS6770.2	chr9_110249621-110249621_G_T	352L>M	Substitution	Nonsynonymous coding	12%
MM12T	KLF8	Kruppel-like factor 8	CCDS14373.1	chrX_56296701-56296701_G_T	282S>I	Substitution	Nonsynonymous coding	34%
MM12T	KLHDC10	kelch domain containing 10	CCDS5815.1	chr7_129764295-129764295_C_T	262R>X	Substitution	Nonsense	13%
MM12T	KLHDC7B	kelch domain containing 7B	CCDS14097.2	chr22_50987010-50987010_G_A	139A>T	Substitution	Nonsynonymous coding	39%
MM12T	KLHDC7B	kelch domain containing 7B	CCDS14097.2	chr22_50987845-50987845_G_T	417S>I	Substitution	Nonsynonymous coding	27%
MM12T	KLHDC7B	kelch domain containing 7B	CCDS14097.2	chr22_50987887-50987887_G_A	431R>H	Substitution	Nonsynonymous coding	38%
MM12T	KLHL12	kelch-like family member 12	CCDS1429.1	chr1_202894137-202894137_C_T	52A>T	Substitution	Nonsynonymous coding	20%
MM12T	KLHL13	kelch-like family member 13	CCDS14571.1	chrX_117033092-117033092_C_T	583A>T	Substitution	Nonsynonymous coding	39%
MM12T	KLHL14	kelch-like family member 14	CCDS32813.1	chr18_30260411-30260411_G_T	464H>N	Substitution	Nonsynonymous coding	15%
MM12T	KLHL15	kelch-like family member 15	CCDS35217.1	chrX_24006075-24006075_G_A	593P>L	Substitution	Nonsynonymous coding	30%
MM12T	KLHL17	kelch-like family member 17	CCDS30550.1	chr1_900374-900374_T_C	578W>R	Substitution	Nonsynonymous coding	36%
MM12T	KLHL2	kelch-like family member 2	CCDS34094.1	chr4_166235226-166235226_G_A	506G>D	Substitution	Nonsynonymous coding	29%
MM12T	KLHL20	kelch-like family member 20	CCDS1310.1	chr1_173743577-173743577_G_A	477V>M	Substitution	Nonsynonymous coding	38%
MM12T	KLHL22	kelch-like family member 22	CCDS13779.1	chr22_20783827-20783827_G_A	474R>C	Substitution	Nonsynonymous coding	30%
MM12T	KLHL24	kelch-like 24 (Drosophila)	CCDS3246.1	chr3_183396974-183396974_C_T	568A>V	Substitution	Nonsynonymous coding	31%
MM12T	KLHL24	kelch-like 24 (Drosophila)	CCDS3246.1	chr3_183397046-183397046_G_A	592R>K	Substitution	Nonsynonymous coding	12%
MM12T	KLHL29	kelch-like 29 (Drosophila)	NM_052920	chr2_23918956-23918956_G_A	626R>H	Substitution	Nonsynonymous coding	27%

MM12T	KLHL3	kelch-like family member 3	CCDS4192.1	chr5_137056154-137056154_C_A	45S>I	Substitution	Nonsynonymous coding	42%
MM12T	KLHL31	kelch-like 31 (Drosophila)	CCDS34478.1	chr6_53519199-53519199_G_A	291A>V	Substitution	Nonsynonymous coding	45%
MM12T	KLHL34	kelch-like 34 (Drosophila)	CCDS14199.1	chrX_21674826-21674826_C_T	361V>M	Substitution	Nonsynonymous coding	31%
MM12T	KLHL35	kelch-like 35 (Drosophila)	CCDS44685.1	chr11_75133742-75133742_C_A	325S>I	Substitution	Nonsynonymous coding	21%
MM12T	KLHL38	kelch-like 38 (Drosophila)	CCDS43766.1	chr8_124659190-124659190_G_A	472A>V	Substitution	Nonsynonymous coding	32%
MM12T	KLHL38	kelch-like 38 (Drosophila)	CCDS43766.1	chr8_124664522-124664522_C_T	215M>I	Substitution	Nonsynonymous coding	24%
MM12T	KLHL4	kelch-like family member 4	CCDS14456.1	chrX_86873010-86873010_T_C	268I>T	Substitution	Nonsynonymous coding	22%
MM12T	KLHL4	kelch-like family member 4	CCDS14456.1	chrX_86888814-86888814_A_G	539T>A	Substitution	Nonsynonymous coding	29%
MM12T	KLHL40	kelch-like 40 (Drosophila)	CCDS2703.1	chr3_42729686-42729686_G_A	402R>H	Substitution	Nonsynonymous coding	12%
MM12T	KLK14	kallikrein-related peptidase 14	CCDS12823.2	chr19_51581280-51581280_G_A	263T>M	Substitution	Nonsynonymous coding	32%
MM12T	KLK9	kallikrein-related peptidase 9	CCDS12816.1	chr19_51506426-51506426_C_T	232A>T	Substitution	Nonsynonymous coding	27%
MM12T	KLRC1	killer cell lectin-like receptor subfamily C, member 1	CCDS8625.1	chr12_10603606-10603606_C_A	50Q>H	Substitution	Nonsynonymous coding	28%
MM12T	KLRG2	killer cell lectin-like receptor subfamily G, member 2	CCDS5854.1	chr7_139168226-139168226_C_A	55E>X	Substitution	Nonsense	29%
MM12T	KNDC1	kinase non-catalytic C-lobe domain (KIND) containing 1	CCDS7674.1	chr10_135015371-135015371_C_T	1119A>V	Substitution	Nonsynonymous coding	16%
MM12T	KPNA6	karyopherin alpha 6 (importin alpha 7)	CCDS352.1	chr1_32620245-32620245_G_A	21A>T	Substitution	Nonsynonymous coding	30%
MM12T	KPNB1	karyopherin (importin) beta 1	CCDS11513.1	chr17_45741584-45741584_C_A	282F>L	Substitution	Nonsynonymous coding	29%
MM12T	KPNB1	karyopherin (importin) beta 1	CCDS11513.1	chr17_45750485-45750485_C_T	550T>M	Substitution	Nonsynonymous coding	24%
MM12T	KREMEN2	kringle containing transmembrane protein 2	CCDS10483.1	chr16_3016979-3016979_G_A	237D>N	Substitution	Nonsynonymous coding	30%
MM12T	KRI1	KRI1 homolog (S. cerevisiae)	CCDS12242.1	chr19_10670064-10670064_C_T	395D>N	Substitution	Nonsynonymous coding	40%
MM12T	KRT10	keratin 10	CCDS11377.1	chr17_38978491-38978491_C_T	116S>N	Substitution	Nonsynonymous coding	38%
MM12T	KRT28	keratin 28	CCDS11376.1	chr17_38953238-38953238_C_T	303R>Q	Substitution	Nonsynonymous coding	12%
MM12T	KRT35	keratin 35	CCDS11394.2	chr17_39637124-39637124_C_A	76G>C	Substitution	Nonsynonymous coding	14%
MM12T	KRT7	keratin 7	CCDS8822.1	chr12_52629117-52629117_G_A	168S>N	Substitution	Nonsynonymous coding	11%
MM12T	KRT71	keratin 71	CCDS8831.1	chr12_52946660-52946660_C_A	68G>C	Substitution	Nonsynonymous coding	29%
MM12T	KRT73	keratin 73	CCDS8834.1	chr12_53012245-53012245_C_T	22A>T	Substitution	Nonsynonymous coding	16%
MM12T	KRT82	keratin 82	CCDS8826.1	chr12_52794378-52794378_G_A	237A>V	Substitution	Nonsynonymous coding	12%
MM12T	KRTAP16-1	keratin associated protein 16-1	ENST00000391352	chr17_39465202-39465202_A_G	102S>P	Substitution	Nonsynonymous coding	34%
MM12T	KRTAP5-1	keratin associated protein 5-1	CCDS31330.1	chr11_1605763-1605763_G_T	239C>X	Substitution	Nonsense	21%
MM12T	KRTCAP3	keratinocyte associated protein 3	CCDS1754.1	chr2_27665613-27665613_G_A	66V>I	Substitution	Nonsynonymous coding	33%
MM12T	KSR2	kinase suppressor of ras 2	NM_173598	chr12_118105333-118105333_C_T	344A>T	Substitution	Nonsynonymous coding	17%
MM12T	KSR2	kinase suppressor of ras 2	NM_173598	chr12_118198873-118198873_C_T	281R>Q	Substitution	Nonsynonymous coding	10%
MM12T	KTN1	kinectin 1 (kinesin receptor)	CCDS41957.1	chr14_56103987-56103987_G_A	541V>I	Substitution	Nonsynonymous coding	27%

MM12T	L3HYPDH	L-3-hydroxyproline dehydratase (trans-)	CCDS9739.1	chr14_59950677-59950677_G_A	120L>F	Substitution	Nonsynonymous coding	11%
MM12T	L3MBTL1	l(3)mbt-like 1 (Drosophila)	CCDS13319.1	chr20_42144821-42144821_C_T	ISV+4>	Substitution	Splice site donor	28%
MM12T	L3MBTL2	l(3)mbt-like 2 (Drosophila)	CCDS14011.1	chr22_41620741-41620741_A_G	396D>G	Substitution	Nonsynonymous coding	40%
MM12T	L3MBTL3	l(3)mbt-like 3 (Drosophila)	CCDS34537.1	chr6_130387570-130387570_G_A	313A>T	Substitution	Nonsynonymous coding	27%
MM12T	L3MBTL4	l(3)mbt-like 4 (Drosophila)	CCDS11839.2	chr18_6243388-6243388_A_G	122L>S	Substitution	Nonsynonymous coding	10%
MM12T	LAG3	lymphocyte-activation gene 3	CCDS8561.1	chr12_6884477-6884477_G_A	274A>T	Substitution	Nonsynonymous coding	10%
MM12T	LAMA1	laminin, alpha 1	CCDS32787.1	chr18_6943232-6943232_G_A	3005T>I	Substitution	Nonsynonymous coding	34%
MM12T	LAMA2	laminin, alpha 2	CCDS5138.1	chr6_129513940-129513940_G_A	575R>Q	Substitution	Nonsynonymous coding	11%
MM12T	LAMA2	laminin, alpha 2	CCDS5138.1	chr6_129486720-129486720_G_T	ISV-1>	Substitution	Splice site acceptor	29%
MM12T	LAMA2	laminin, alpha 2	CCDS5138.1	chr6_129621928-129621928_C_T	1029R>X	Substitution	Nonsense	49%
MM12T	LAMA3	laminin, alpha 3	CCDS42419.1	chr18_21451564-21451564_G_A	1646R>H	Substitution	Nonsynonymous coding	15%
MM12T	LAMA5	laminin, alpha 5	CCDS33502.1	chr20_60887336-60887336_G_T	3133L>I	Substitution	Nonsynonymous coding	32%
MM12T	LAMA5	laminin, alpha 5	CCDS33502.1	chr20_60893526-60893526_C_T	2408R>H	Substitution	Nonsynonymous coding	30%
MM12T	LAMA5	laminin, alpha 5	CCDS33502.1	chr20_60900562-60900562_C_T	1780R>H	Substitution	Nonsynonymous coding	12%
MM12T	LAMA5	laminin, alpha 5	CCDS33502.1	chr20_60903938-60903938_C_T	1470R>H	Substitution	Nonsynonymous coding	28%
MM12T	LAMA5	laminin, alpha 5	CCDS33502.1	chr20_60905634-60905634_C_T	1297G>S	Substitution	Nonsynonymous coding	34%
MM12T	LAMA5	laminin, alpha 5	CCDS33502.1	chr20_60906088-60906088_C_T	1217S>N	Substitution	Nonsynonymous coding	24%
MM12T	LAMA5	laminin, alpha 5	CCDS33502.1	chr20_60908142-60908142_G_A	ISV+4>	Substitution	Splice site donor	18%
MM12T	LAMB1	laminin, beta 1	CCDS5750.1	chr7_107580533-107580533_T_C	1221D>G	Substitution	Nonsynonymous coding	29%
MM12T	LAMB2	laminin, beta 2 (laminin S)	CCDS2789.1	chr3_49166548-49166548_G_A	546H>Y	Substitution	Nonsynonymous coding	26%
MM12T	LAMB2	laminin, beta 2 (laminin S)	CCDS2789.1	chr3_49169032-49169032_C_T	195R>Q	Substitution	Nonsynonymous coding	36%
MM12T	LAMB3	laminin, beta 3	CCDS1487.1	chr1_209807808-209807808_C_T	183R>H	Substitution	Nonsynonymous coding	11%
MM12T	LAMC1	laminin, gamma 1 (formerly LAMB2)	CCDS1351.1	chr1_183102642-183102642_C_T	1269A>V	Substitution	Nonsynonymous coding	30%
MM12T	LAMC3	laminin, gamma 3	CCDS6938.1	chr9_133960983-133960983_G_T	1368R>I	Substitution	Nonsynonymous coding	13%
MM12T	LAMP5	lysosomal-associated membrane protein family, member 5	CCDS13106.1	chr20_9496933-9496933_G_A	134A>T	Substitution	Nonsynonymous coding	37%
MM12T	LANCL1	LanC lantibiotic synthetase component C-like 1 (bacterial)	CCDS2392.1	chr2_211302551-211302551_G_A	246P>S	Substitution	Nonsynonymous coding	14%
MM12T	LANCL1	LanC lantibiotic synthetase component C-like 1 (bacterial)	CCDS2392.1	chr2_211320042-211320042_A_	NA	Deletion	Splice site acceptor	13%
MM12T	LARP4B	La ribonucleoprotein domain family, member 4B	CCDS31131.1	chr10_863685-863685_T_A	559I>L	Substitution	Nonsynonymous coding	30%
MM12T	LARP4B	La ribonucleoprotein domain family, member 4B	CCDS31131.1	chr10_909820-909820_G_A	98S>L	Substitution	Nonsynonymous coding	19%
MM12T	LARP4B	La ribonucleoprotein domain family, member 4B	CCDS31131.1	chr10_930446-930446_T_C	28M>V	Substitution	Nonsynonymous coding	17%
MM12T	LARP7	La ribonucleoprotein domain family, member 7	CCDS3701.2	chr4_113574380-113574380_G_A	522E>K	Substitution	Nonsynonymous coding	23%
MM12T	LAX1	lymphocyte transmembrane adaptor 1	CCDS1441.2	chr1_203740006-203740006_C_T	47A>V	Substitution	Nonsynonymous coding	33%

MM12T	LAX1	lymphocyte transmembrane adaptor 1	CCDS1441.2	chr1_203741220-203741220_G_A	112R>H	Substitution	Nonsynonymous coding	36%
MM12T	LCAT	lecithin-cholesterol acyltransferase	CCDS10854.1	chr16_67977938-67977938_C_T	23A>T	Substitution	Nonsynonymous coding	32%
MM12T	LCE3C	late cornified envelope 3C	CCDS1015.1	chr1_152573407-152573407_G_A	67R>H	Substitution	Nonsynonymous coding	14%
MM12T	LCK	lymphocyte-specific protein tyrosine kinase	CCDS359.1	chr1_32740365-32740365_C_T	45R>W	Substitution	Nonsynonymous coding	15%
MM12T	LCK	lymphocyte-specific protein tyrosine kinase	CCDS359.1	chr1_32745797-32745797_G_A	438R>H	Substitution	Nonsynonymous coding	39%
MM12T	LCMT1	leucine carboxyl methyltransferase 1	CCDS45445.1	chr16_25172440-25172440_T_C	162S>P	Substitution	Nonsynonymous coding	21%
MM12T	LCMT1	leucine carboxyl methyltransferase 1	CCDS45445.1	chr16_25182080-25182080_G_A	280D>N	Substitution	Nonsynonymous coding	13%
MM12T	LCN15	lipocalin 15	CCDS7006.1	chr9_139657865-139657865_A_G	121V>A	Substitution	Nonsynonymous coding	23%
MM12T	LCN9	lipocalin 9	ENST00000277526	chr9_138556099-138556099_T_C	63I>T	Substitution	Nonsynonymous coding	23%
MM12T	LCN9	lipocalin 9	ENST00000277526	chr9_138557754-138557754_C_T	189R>C	Substitution	Nonsynonymous coding	20%
MM12T	LCP1	lymphocyte cytosolic protein 1 (L-plastin)	CCDS9403.1	chr13_46732656-46732656_C_T	ISV+1>	Substitution	Splice site donor	38%
MM12T	LCT	lactase	CCDS2178.1	chr2_136579651-136579651_C_T	309V>M	Substitution	Nonsynonymous coding	43%
MM12T	LCTL	lactase-like	CCDS10220.1	chr15_66855937-66855937_C_T	133E>K	Substitution	Nonsynonymous coding	39%
MM12T	LCTL	lactase-like	CCDS10220.1	chr15_66844110-66844110_C_A	472G>X	Substitution	Nonsense	29%
MM12T	LDHD	lactate dehydrogenase D	CCDS10913.1	chr16_75147459-75147459_C_A	377A>S	Substitution	Nonsynonymous coding	15%
MM12T	LEFTY1	left-right determination factor 1	CCDS1548.1	chr1_226075193-226075193_C_T	215A>T	Substitution	Nonsynonymous coding	12%
MM12T	LENG8	leukocyte receptor cluster (LRC) member 8	CCDS12894.1	chr19_54966652-54966652_G_A	311D>N	Substitution	Nonsynonymous coding	16%
MM12T	LENG8	leukocyte receptor cluster (LRC) member 8	CCDS12894.1	chr19_54967374-54967374_C_A	418D>E	Substitution	Nonsynonymous coding	29%
MM12T	LEPREL2	leprecan-like 2	ENST00000290510	chr12_6939863-6939863_C_A	304P>H	Substitution	Nonsynonymous coding	30%
MM12T	LETM1	leucine zipper-EF-hand containing transmembrane protein 1	CCDS3355.1	chr4_1825505-1825505_G_A	ISV-3>	Substitution	Splice site acceptor	16%
MM12T	LGALS13	lectin, galactoside-binding, soluble, 13	CCDS33024.1	chr19_40097882-40097882_G_A	108R>H	Substitution	Nonsynonymous coding	16%
MM12T	LGI3	leucine-rich repeat LGI family, member 3	CCDS6025.1	chr8_22005810-22005810_G_A	504R>W	Substitution	Nonsynonymous coding	10%
MM12T	LGI3	leucine-rich repeat LGI family, member 3	CCDS6025.1	chr8_22005884-22005884_C_T	479R>H	Substitution	Nonsynonymous coding	33%
MM12T	LGSN	lengsin, lens protein with glutamine synthetase domain	CCDS4964.1	chr6_63990492-63990492_C_T	322D>N	Substitution	Nonsynonymous coding	50%
MM12T	LHFP	lipoma HMGIC fusion partner	CCDS9369.1	chr13_40175145-40175145_C_T	70R>H	Substitution	Nonsynonymous coding	31%
MM12T	LHFPL4	lipoma HMGIC fusion partner-like 4	CCDS33691.1	chr3_9594291-9594291_G_T	25L>M	Substitution	Nonsynonymous coding	30%
MM12T	LHFPL5	lipoma HMGIC fusion partner-like 5	CCDS4812.1	chr6_35782470-35782470_G_A	187G>D	Substitution	Nonsynonymous coding	21%
MM12T	LHPP	phospholysine phosphohistidine inorganic pyrophosphate phosphatase	CCDS7640.1	chr10_126186615-126186615_G_A	181G>D	Substitution	Nonsynonymous coding	11%
MM12T	LHX1	LIM homeobox 1	CCDS11316.1	chr17_35297615-35297615_G_A	67A>T	Substitution	Nonsynonymous coding	11%
MM12T	LHX3	LIM homeobox 3	CCDS6995.1	chr9_139091668-139091668_G_A	109R>C	Substitution	Nonsynonymous coding	18%
MM12T	LHX4	LIM homeobox 4	CCDS1338.1	chr1_180241031-180241031_G_A	223R>H	Substitution	Nonsynonymous coding	12%
MM12T	LIAS	lipoic acid synthetase	CCDS3453.1	chr4_39466943-39466943_G_A	197V>I	Substitution	Nonsynonymous coding	13%

MM12T	LIAS	lipoic acid synthetase	CCDS3453.1	chr4_39466962-39466962_G_A	203R>K	Substitution	Nonsynonymous coding	19%
MM12T	LIG3	ligase III, DNA, ATP-dependent	CCDS11284.2	chr17_33326830-33326830_G_T	762K>N	Substitution	Nonsynonymous coding	22%
MM12T	LILRB3	leukocyte immunoglobulin-like receptor, subfamily B (with TM and ITIM domains),	ENST00000407860	chr19_54723778-54723778_C_A	440E>D	Substitution	Nonsynonymous coding	46%
MM12T	LIM2	lens intrinsic membrane protein 2, 19kDa	CCDS12831.1	chr19_51885738-51885738_C_T	129A>T	Substitution	Nonsynonymous coding	12%
MM12T	LIMA1	LIM domain and actin binding 1	CCDS44877.1	chr12_50570879-50570879_G_A	751R>W	Substitution	Nonsynonymous coding	33%
MM12T	LIMA1	LIM domain and actin binding 1	CCDS44877.1	chr12_50642415-50642415_C_T	ISV+1>	Substitution	Splice site donor	31%
MM12T	LIMCH1	LIM and calponin homology domains 1	CCDS33977.1	chr4_41648209-41648209_A_	NA	Deletion	Frameshift	19%
MM12T	LIMCH1	LIM and calponin homology domains 1	ENST00000313875	chr4_41635752-41635752_T_A	673F>I	Substitution	Nonsynonymous coding	14%
MM12T	LIMD2	LIM domain containing 2	CCDS11641.1	chr17_61776229-61776229_G_A	52R>W	Substitution	Nonsynonymous coding	14%
MM12T	LIMK2	LIM domain kinase 2	CCDS33637.1	chr22_31658728-31658728_C_A	248L>M	Substitution	Nonsynonymous coding	35%
MM12T	LIN37	lin-37 homolog (C. elegans)	NM_019104	chr19_36245172-36245172_C_A	204S>Y	Substitution	Nonsynonymous coding	26%
MM12T	LINGO2	leucine rich repeat and Ig domain containing 2	CCDS6524.1	chr9_27949443-27949443__T	NA	Insertion	Frameshift	25%
MM12T	LIPG	lipase, endothelial	CCDS11938.1	chr18_47107944-47107944_G_T	318S>I	Substitution	Nonsynonymous coding	30%
MM12T	LIPG	lipase, endothelial	CCDS11938.1	chr18_47110141-47110141_G_A	458R>Q	Substitution	Nonsynonymous coding	17%
MM12T	LIPH	lipase, member H	CCDS3272.1	chr3_185229354-185229354_C_A	409R>M	Substitution	Nonsynonymous coding	23%
MM12T	LLGL1	lethal giant larvae homolog 1 (Drosophila)	CCDS32586.1	chr17_18140899-18140899_G_T	572E>D	Substitution	Nonsynonymous coding	12%
MM12T	LLGL1	lethal giant larvae homolog 1 (Drosophila)	CCDS32586.1	chr17_18141417-18141417_G_T	647E>D	Substitution	Nonsynonymous coding	15%
MM12T	LLGL1	lethal giant larvae homolog 1 (Drosophila)	CCDS32586.1	chr17_18141840-18141840_G_A	708R>H	Substitution	Nonsynonymous coding	19%
MM12T	LLGL1	lethal giant larvae homolog 1 (Drosophila)	CCDS32586.1	chr17_18144167-18144167_G_A	828A>T	Substitution	Nonsynonymous coding	25%
MM12T	LMAN2	lectin, mannose-binding 2	CCDS4417.1	chr5_176765509-176765509_G_A	138T>I	Substitution	Nonsynonymous coding	26%
MM12T	LMAN2L	lectin, mannose-binding 2-like	CCDS46365.1	chr2_97377437-97377437_C_T	258R>H	Substitution	Nonsynonymous coding	35%
MM12T	LMCD1	LIM and cysteine-rich domains 1	CCDS33688.1	chr3_8609231-8609231_G_A	349V>I	Substitution	Nonsynonymous coding	35%
MM12T	LMF2	lipase maturation factor 2	CCDS14093.1	chr22_50941873-50941873_C_T	666A>T	Substitution	Nonsynonymous coding	40%
MM12T	LMLN	leishmanolysin-like (metallopeptidase M8 family)	CCDS46988.1	chr3_197707272-197707272_G_A	209A>T	Substitution	Nonsynonymous coding	18%
MM12T	LMLN	leishmanolysin-like (metallopeptidase M8 family)	CCDS46988.1	chr3_197707197-197707197_C_T	184Q>X	Substitution	Nonsense	20%
MM12T	LMNB1	lamin B1	CCDS4140.1	chr5_126156747-126156747_G_A	436A>T	Substitution	Nonsynonymous coding	20%
MM12T	LMNB2	lamin B2	CCDS12090.1	chr19_2434878-2434878_C_T	277D>N	Substitution	Nonsynonymous coding	26%
MM12T	LMO7	LIM domain 7	CCDS9454.1	chr13_76415878-76415878_C_T	982R>X	Substitution	Nonsense	17%
MM12T	LMTK2	lemur tyrosine kinase 2	CCDS5654.1	chr7_97800888-97800888_G_T	231E>D	Substitution	Nonsynonymous coding	30%
MM12T	LMTK3	lemur tyrosine kinase 3	CCDS46136.1	chr19_49001946-49001946_C_T	823G>S	Substitution	Nonsynonymous coding	41%
MM12T	LNPEP	leucyl/cystinyl aminopeptidase	CCDS4087.1	chr5_96315077-96315077_C_A	85S>R	Substitution	Nonsynonymous coding	30%
MM12T	LONP2	lon peptidase 2, peroxisomal	CCDS10734.1	chr16_48303983-48303983_G_A	347E>K	Substitution	Nonsynonymous coding	12%

MM12T	LONRF1	LON peptidase N-terminal domain and ring finger 1	CCDS5987.2	chr8_12586477-12586477_C_A	648R>M	Substitution	Nonsynonymous coding	20%
MM12T	LOXL1	lysyl oxidase-like 1	CCDS10253.1	chr15_74238819-74238819_C_T	425R>C	Substitution	Nonsynonymous coding	32%
MM12T	LOXL1	lysyl oxidase-like 1	CCDS10253.1	chr15_74241809-74241809_A_G	538N>D	Substitution	Nonsynonymous coding	26%
MM12T	LOXL2	lysyl oxidase-like 2	CCDS34864.1	chr8_23225726-23225726_G_A	47P>S	Substitution	Nonsynonymous coding	31%
MM12T	LOXL4	lysyl oxidase-like 4	CCDS7473.1	chr10_100022545-100022545_C_T	78A>T	Substitution	Nonsynonymous coding	27%
MM12T	LPAR4	lysophosphatidic acid receptor 4	CCDS14441.1	chrX_78010773-78010773_G_A	136R>H	Substitution	Nonsynonymous coding	33%
MM12T	LPCAT1	lysophosphatidylcholine acyltransferase 1	CCDS3864.1	chr5_1463833-1463833_A_G	513F>S	Substitution	Nonsynonymous coding	31%
MM12T	LPCAT3	lysophosphatidylcholine acyltransferase 3	CCDS8572.1	chr12_7087545-7087545_G_T	333T>N	Substitution	Nonsynonymous coding	32%
MM12T	LPCAT4	lysophosphatidylcholine acyltransferase 4	CCDS32191.1	chr15_34654520-34654520_G_A	296A>V	Substitution	Nonsynonymous coding	37%
MM12T	LPHN1	latrophilin 1	CCDS32928.1	chr19_14263401-14263401_C_T	1155D>N	Substitution	Nonsynonymous coding	35%
MM12T	LPHN2	latrophilin 2	CCDS689.1	chr1_82456679-82456679_G_A	1354M>I	Substitution	Nonsynonymous coding	34%
MM12T	LPHN2	latrophilin 2	CCDS689.1	chr1_82372776-82372776_C_T	50R>X	Substitution	Nonsense	40%
MM12T	LPHN3	latrophilin 3	NM_015236	chr4_62778475-62778475_G_T	636Q>H	Substitution	Nonsynonymous coding	19%
MM12T	LPO	lactoperoxidase	CCDS32689.1	chr17_56343598-56343598_G_A	535R>H	Substitution	Nonsynonymous coding	13%
MM12T	LRBA	LPS-responsive vesicle trafficking, beach and anchor containing	CCDS3773.1	chr4_151773632-151773632_T_C	1077E>G	Substitution	Nonsynonymous coding	14%
MM12T	LRCH1	leucine-rich repeats and calponin homology (CH) domain containing 1	CCDS31972.1	chr13_47315910-47315910_G_A	705G>D	Substitution	Nonsynonymous coding	26%
MM12T	LRCH2	leucine-rich repeats and calponin homology (CH) domain containing 2	CCDS48155.1	chrX_114384396-114384396_A_C	563Y>X	Substitution	Nonsense	36%
MM12T	LRCH3	leucine-rich repeats and calponin homology (CH) domain containing 3	CCDS3330.1	chr3_197557664-197557664_G_A	304R>H	Substitution	Nonsynonymous coding	16%
MM12T	LRCH4	leucine-rich repeats and calponin homology (CH) domain containing 4	CCDS34706.1	chr7_100173537-100173537_C_T	578R>Q	Substitution	Nonsynonymous coding	14%
MM12T	LRFN1	leucine rich repeat and fibronectin type III domain containing 1	CCDS46071.1	chr19_39799024-39799024_G_A	522A>V	Substitution	Nonsynonymous coding	31%
MM12T	LRFN1	leucine rich repeat and fibronectin type III domain containing 1	CCDS46071.1	chr19_39805838-39805838_G_C	47L>V	Substitution	Nonsynonymous coding	27%
MM12T	LRFN2	leucine rich repeat and fibronectin type III domain containing 2	CCDS34443.1	chr6_40399677-40399677__G	NA	Insertion	Frameshift	23%
MM12T	LRGUK	leucine-rich repeats and guanylate kinase domain containing	CCDS5830.1	chr7_133861753-133861753_G_A	349V>I	Substitution	Nonsynonymous coding	13%
MM12T	LRGUK	leucine-rich repeats and guanylate kinase domain containing	CCDS5830.1	chr7_133881831-133881831_G_A	507A>T	Substitution	Nonsynonymous coding	14%
MM12T	LRGUK	leucine-rich repeats and guanylate kinase domain containing	CCDS5830.1	chr7_133943059-133943059_C_T	750P>L	Substitution	Nonsynonymous coding	17%
MM12T	LRIG1	leucine-rich repeats and immunoglobulin-like domains 1	CCDS33783.1	chr3_66550660-66550660_C_T	58G>R	Substitution	Nonsynonymous coding	41%
MM12T	LRIG3	leucine-rich repeats and immunoglobulin-like domains 3	CCDS8960.1	chr12_59271617-59271617_G_A	701R>W	Substitution	Nonsynonymous coding	13%
MM12T	LRIT1	leucine-rich repeat, immunoglobulin-like and transmembrane domains 1	CCDS7373.1	chr10_85997258-85997258_G_T	103L>M	Substitution	Nonsynonymous coding	32%
MM12T	LRIT2	leucine-rich repeat, immunoglobulin-like and transmembrane domains 2	CCDS31234.1	chr10_85981836-85981836_C_T	498R>H	Substitution	Nonsynonymous coding	31%
MM12T	LRIT3	leucine-rich repeat, immunoglobulin-like and transmembrane domains 3	CCDS3688.2	chr4_110773069-110773069_T_C	131W>R	Substitution	Nonsynonymous coding	32%
MM12T	LRP1	low density lipoprotein receptor-related protein 1	CCDS8932.1	chr12_57572284-57572284_G_A	1502A>T	Substitution	Nonsynonymous coding	32%
MM12T	LRP1	low density lipoprotein receptor-related protein 1	CCDS8932.1	chr12_57589879-57589879_G_A	2904C>Y	Substitution	Nonsynonymous coding	35%

MM12T	LRP1	low density lipoprotein receptor-related protein 1	CCDS8932.1	chr12_57604623-57604623_C_T	4293R>X	Substitution	Nonsense	28%
MM12T	LRP10	low density lipoprotein receptor-related protein 10	CCDS9578.1	chr14_23342579-23342579_G_A	47V>I	Substitution	Nonsynonymous coding	11%
MM12T	LRP1B	low density lipoprotein receptor-related protein 1B	CCDS2182.1	chr2_141459401-141459401_C_T	2106G>R	Substitution	Nonsynonymous coding	25%
MM12T	LRP1B	low density lipoprotein receptor-related protein 1B	CCDS2182.1	chr2_141709492-141709492_G_A	969P>S	Substitution	Nonsynonymous coding	44%
MM12T	LRP1B	low density lipoprotein receptor-related protein 1B	CCDS2182.1	chr2_141004651-141004651_T_G	ISV+4>	Substitution	Splice site donor	15%
MM12T	LRP2	low density lipoprotein receptor-related protein 2	CCDS2232.1	chr2_170063445-170063445_C_T	2262R>H	Substitution	Nonsynonymous coding	37%
MM12T	LRP2	low density lipoprotein receptor-related protein 2	CCDS2232.1	chr2_170058336-170058336_G_A	2752R>X	Substitution	Nonsense	15%
MM12T	LRP4	low density lipoprotein receptor-related protein 4	CCDS31478.1	chr11_46889596-46889596_G_A	1674T>I	Substitution	Nonsynonymous coding	24%
MM12T	LRP4	low density lipoprotein receptor-related protein 4	CCDS31478.1	chr11_46916247-46916247_C_T	478R>H	Substitution	Nonsynonymous coding	13%
MM12T	LRP5	low density lipoprotein receptor-related protein 5	CCDS8181.1	chr11_68115615-68115615_G_A	131R>H	Substitution	Nonsynonymous coding	36%
MM12T	LRP5	low density lipoprotein receptor-related protein 5	CCDS8181.1	chr11_68170979-68170979_G_A	538R>Q	Substitution	Nonsynonymous coding	32%
MM12T	LRP5	low density lipoprotein receptor-related protein 5	CCDS8181.1	chr11_68183796-68183796_C_T	943P>L	Substitution	Nonsynonymous coding	30%
MM12T	LRP5	low density lipoprotein receptor-related protein 5	CCDS8181.1	chr11_68191155-68191155_G_A	1076A>T	Substitution	Nonsynonymous coding	40%
MM12T	LRP5	low density lipoprotein receptor-related protein 5	CCDS8181.1	chr11_68201270-68201270_G_A	1322D>N	Substitution	Nonsynonymous coding	16%
MM12T	LRP5	low density lipoprotein receptor-related protein 5	CCDS8181.1	chr11_68204387-68204387_C_T	1344A>V	Substitution	Nonsynonymous coding	13%
MM12T	LRP6	low density lipoprotein receptor-related protein 6	CCDS8647.1	chr12_12339985-12339985_C_A	239W>L	Substitution	Nonsynonymous coding	24%
MM12T	LRPPRC	leucine-rich pentatricopeptide repeat containing	CCDS33189.1	chr2_44132917-44132917_G_A	1093A>V	Substitution	Nonsynonymous coding	39%
MM12T	LRPPRC	leucine-rich pentatricopeptide repeat containing	CCDS33189.1	chr2_44190740-44190740_C_T	492R>Q	Substitution	Nonsynonymous coding	26%
MM12T	LRPPRC	leucine-rich pentatricopeptide repeat containing	CCDS33189.1	chr2_44176750-44176750_C_A	576G>X	Substitution	Nonsense	27%
MM12T	LRR1	leucine rich repeat protein 1	CCDS9686.1	chr14_50074262-50074262_A_	NA	Deletion	Frameshift	29%
MM12T	LRRC16A	leucine rich repeat containing 16A	ENST00000332168	chr6_25452454-25452454_T_	NA	Deletion	Frameshift	45%
MM12T	LRRC16A	leucine rich repeat containing 16A	NM_017640	chr6_25538159-25538159_A_T	715E>V	Substitution	Nonsynonymous coding	11%
MM12T	LRRC20	leucine rich repeat containing 20	CCDS7302.1	chr10_72061130-72061130_C_T	179A>T	Substitution	Nonsynonymous coding	34%
MM12T	LRRC34	leucine rich repeat containing 34	CCDS3208.1	chr3_169514669-169514669_C_T	213V>I	Substitution	Nonsynonymous coding	31%
MM12T	LRRC36	leucine rich repeat containing 36	CCDS32467.1	chr16_67401279-67401279_G_A	372A>T	Substitution	Nonsynonymous coding	27%
MM12T	LRRC39	leucine rich repeat containing 39	CCDS766.1	chr1_100618060-100618060_T_C	278N>S	Substitution	Nonsynonymous coding	16%
MM12T	LRRC41	leucine rich repeat containing 41	CCDS533.1	chr1_46763306-46763306_C_A	ISV-1>	Substitution	Splice site acceptor	40%
MM12T	LRRC42	leucine rich repeat containing 42	CCDS585.1	chr1_54426078-54426078_C_T	219R>W	Substitution	Nonsynonymous coding	27%
MM12T	LRRC49	leucine rich repeat containing 49	CCDS32282.1	chr15_71272452-71272452_C_T	312R>C	Substitution	Nonsynonymous coding	11%
MM12T	LRRC4B	leucine rich repeat containing 4B	CCDS42595.1	chr19_51021034-51021034_C_T	646G>R	Substitution	Nonsynonymous coding	35%
MM12T	LRRC4C	leucine rich repeat containing 4C	CCDS31464.1	chr11_40137472-40137472_G_A	124A>V	Substitution	Nonsynonymous coding	50%
MM12T	LRRC56	leucine rich repeat containing 56	CCDS7700.1	chr11_549907-549907_T_C	111L>S	Substitution	Nonsynonymous coding	33%

MM12T	LRRC66	leucine rich repeat containing 66	CCDS43229.1	chr4_52861860-52861860_C_T	443R>H	Substitution	Nonsynonymous coding	36%
MM12T	LRRC66	leucine rich repeat containing 66	CCDS43229.1	chr4_52861995-52861995_C_A	398R>M	Substitution	Nonsynonymous coding	15%
MM12T	LRRC8A	leucine rich repeat containing 8 family, member A	CCDS35155.1	chr9_131670168-131670168_C_T	242A>V	Substitution	Nonsynonymous coding	34%
MM12T	LRRC8E	leucine rich repeat containing 8 family, member E	CCDS12189.1	chr19_7964763-7964763_C_	NA	Deletion	Frameshift	35%
MM12T	LRRC8E	leucine rich repeat containing 8 family, member E	CCDS12189.1	chr19_7965584-7965584_C_T	726T>M	Substitution	Nonsynonymous coding	16%
MM12T	LRRD1	leucine-rich repeats and death domain containing 1	NM_001161528	chr7_91794442-91794442_C_A	25Q>H	Substitution	Nonsynonymous coding	28%
MM12T	LRRK2	leucine-rich repeat kinase 2	CCDS31774.1	chr12_40677776-40677776_A_G	781K>E	Substitution	Nonsynonymous coding	36%
MM12T	LRRN1	leucine rich repeat neuronal 1	CCDS33685.1	chr3_3887377-3887377_C_T	351A>V	Substitution	Nonsynonymous coding	32%
MM12T	LRRN4	leucine rich repeat neuronal 4	CCDS13097.1	chr20_6022374-6022374_G_C	506T>R	Substitution	Nonsynonymous coding	35%
MM12T	LRRTM3	leucine rich repeat transmembrane neuronal 3	CCDS7270.1	chr10_68687143-68687143_C_T	157R>W	Substitution	Nonsynonymous coding	33%
MM12T	LRTOMT	leucine rich transmembrane and 0-methyltransferase domain containing	CCDS8208.1	chr11_71805994-71805994_G_A	97V>I	Substitution	Nonsynonymous coding	18%
MM12T	LSM14B	LSM14B, SCD6 homolog B (S. cerevisiae)	CCDS46626.1	chr20_60708387-60708387_C_T	343T>I	Substitution	Nonsynonymous coding	31%
MM12T	LTBP1	latent transforming growth factor beta binding protein 1	CCDS33177.1	chr2_33588493-33588493_G_A	1437C>Y	Substitution	Nonsynonymous coding	31%
MM12T	LTBP1	latent transforming growth factor beta binding protein 1	CCDS33177.1	chr2_33623602-33623602_A_T	1720D>V	Substitution	Nonsynonymous coding	26%
MM12T	LTBP2	latent transforming growth factor beta binding protein 2	CCDS9831.1	chr14_75078170-75078170_G_A	160R>X	Substitution	Nonsense	29%
MM12T	LTBP3	latent transforming growth factor beta binding protein 3	CCDS44647.1	chr11_65310961-65310961_C_A	805G>X	Substitution	Nonsense	27%
MM12T	LTN1	listerin E3 ubiquitin protein ligase 1	CCDS33527.1	chr21_30331783-30331783_G_A	864P>S	Substitution	Nonsynonymous coding	16%
MM12T	LUC7L	LUC7-like (S. cerevisiae)	CCDS32348.1	chr16_256026-256026_G_A	163R>C	Substitution	Nonsynonymous coding	23%
MM12T	LUM	lumican	CCDS9038.1	chr12_91497971-91497971_G_A	330R>C	Substitution	Nonsynonymous coding	29%
MM12T	LUZP2	leucine zipper protein 2	CCDS31446.1	chr11_24927544-24927544_G_T	135S>I	Substitution	Nonsynonymous coding	19%
MM12T	LY6G5C	lymphocyte antigen 6 complex, locus G5C	ENST00000375863	chr6_31651020-31651020_C_T	51R>H	Substitution	Nonsynonymous coding	18%
MM12T	LYAR	Ly1 antibody reactive	CCDS3374.1	chr4_4283595-4283595_C_T	51C>Y	Substitution	Nonsynonymous coding	31%
MM12T	LYG1	lysozyme G-like 1	CCDS2043.1	chr2_99912106-99912106_G_T	10L>I	Substitution	Nonsynonymous coding	20%
MM12T	LYN	v-yes-1 Yamaguchi sarcoma viral related oncogene homolog	CCDS6162.1	chr8_56863088-56863088_G_A	119A>T	Substitution	Nonsynonymous coding	29%
MM12T	LYPD6	LY6/PLAUR domain containing 6	CCDS2188.1	chr2_150294272-150294272_C_T	17A>V	Substitution	Nonsynonymous coding	33%
MM12T	LYSMD4	LysM, putative peptidoglycan-binding, domain containing 4	CCDS10381.1	chr15_100269338-100269338_G_A	295A>V	Substitution	Nonsynonymous coding	19%
MM12T	LYZL4	lysozyme-like 4	CCDS2697.1	chr3_42448362-42448362_T_C	90N>D	Substitution	Nonsynonymous coding	35%
MM12T	LZTR1	leucine-zipper-like transcription regulator 1	CCDS33606.1	chr22_21345925-21345925_G_A	267R>H	Substitution	Nonsynonymous coding	32%
MM12T	LZTR1	leucine-zipper-like transcription regulator 1	CCDS33606.1	chr22_21347108-21347108_C_T	392A>V	Substitution	Nonsynonymous coding	34%
MM12T	LZTS1	leucine zipper, putative tumor suppressor 1	CCDS6015.1	chr8_20107475-20107475_C_A	517G>C	Substitution	Nonsynonymous coding	35%
MM12T	LZTS2	leucine zipper, putative tumor suppressor 2	CCDS7507.1	chr10_102765279-102765279_C_T	378A>V	Substitution	Nonsynonymous coding	10%
MM12T	MAATS1	MYCBP-associated, testis expressed 1	CCDS2994.1	chr3_119426264-119426264_G_T	72R>M	Substitution	Nonsynonymous coding	23%

MM12T	MAB21L2	mab-21-like 2 (C. elegans)	CCDS3774.1	chr4_151504473-151504473_G_A	98A>T	Substitution	Nonsynonymous coding	32%
MM12T	MAB21L2	mab-21-like 2 (C. elegans)	CCDS3774.1	chr4_151504731-151504731_G_A	184A>T	Substitution	Nonsynonymous coding	11%
MM12T	MACC1	metastasis associated in colon cancer 1	CCDS5369.1	chr7_20199686-20199686_G_A	100P>S	Substitution	Nonsynonymous coding	21%
MM12T	MACF1	microtubule-actin crosslinking factor 1	CCDS435.1	chr1_39550053-39550053_C_T	55R>W	Substitution	Nonsynonymous coding	30%
MM12T	MACF1	microtubule-actin crosslinking factor 1	CCDS436.1	chr1_39826575-39826575_C_A	2559L>M	Substitution	Nonsynonymous coding	41%
MM12T	MACF1	microtubule-actin crosslinking factor 1	CCDS436.1	chr1_39906729-39906729_C_A	4611L>I	Substitution	Nonsynonymous coding	34%
MM12T	MADCAM1	mucosal vascular addressin cell adhesion molecule 1	CCDS12028.1	chr19_498681-498681_C_T	175P>S	Substitution	Nonsynonymous coding	38%
MM12T	MADD	MAP-kinase activating death domain	CCDS7930.1	chr11_47331083-47331083_C_T	1360R>W	Substitution	Nonsynonymous coding	19%
MM12T	MAGEB3	melanoma antigen family B, 3	CCDS14220.1	chrX_30254817-30254817_A_G	259E>G	Substitution	Nonsynonymous coding	33%
MM12T	MAGEB5	melanoma antigen family B, 5	ENST00000379029	chrX_26235540-26235540_A_C	41N>T	Substitution	Nonsynonymous coding	29%
MM12T	MAGEB5	melanoma antigen family B, 5	ENST00000379029	chrX_26236215-26236215_C_T	266A>V	Substitution	Nonsynonymous coding	25%
MM12T	MAGEC1	melanoma antigen family C, 1	CCDS35417.1	chrX_140995910-140995910_C_T	907T>I	Substitution	Nonsynonymous coding	27%
MM12T	MAGEC2	melanoma antigen family C, 2	CCDS14678.1	chrX_141291287-141291287_T_A	163T>S	Substitution	Nonsynonymous coding	13%
MM12T	MAGEC2	melanoma antigen family C, 2	CCDS14678.1	chrX_141291292-141291292_G_T	161P>H	Substitution	Nonsynonymous coding	28%
MM12T	MAGED1	melanoma antigen family D, 1	CCDS35279.1	chrX_51637772-51637772_C_A	32A>D	Substitution	Nonsynonymous coding	30%
MM12T	MAGED1	melanoma antigen family D, 1	CCDS35279.1	chrX_51639710-51639710_G_A	376R>H	Substitution	Nonsynonymous coding	34%
MM12T	MAGED1	melanoma antigen family D, 1	CCDS35279.1	chrX_51639640-51639640_C_T	353Q>X	Substitution	Nonsense	34%
MM12T	MAGED2	melanoma antigen family D, 2	CCDS14362.1	chrX_54841970-54841970_C_T	559A>V	Substitution	Nonsynonymous coding	11%
MM12T	MAGEE2	melanoma antigen family E, 2	CCDS14431.1	chrX_75004823-75004823_C_T	22G>S	Substitution	Nonsynonymous coding	15%
MM12T	MAGEF1	melanoma antigen family F, 1	CCDS3269.1	chr3_184429241-184429241_C_A	123E>D	Substitution	Nonsynonymous coding	34%
MM12T	MAGI2	membrane associated guanylate kinase, WW and PDZ domain containing 2	CCDS5594.1	chr7_77755049-77755049_C_T	1177A>T	Substitution	Nonsynonymous coding	26%
MM12T	MAGI3	membrane associated guanylate kinase, WW and PDZ domain containing 3	CCDS44196.1	chr1_114137153-114137153_C_T	330A>V	Substitution	Nonsynonymous coding	31%
MM12T	MAGT1	magnesium transporter 1	CCDS14436.2	chrX_77112357-77112357_C_A	214R>I	Substitution	Nonsynonymous coding	12%
MM12T	MAGT1	magnesium transporter 1	CCDS14436.2	chrX_77126340-77126340_C_T	151V>M	Substitution	Nonsynonymous coding	28%
MM12T	MAK16	MAK16 homolog (S. cerevisiae)	CCDS6089.1	chr8_33346300-33346300_G_A	75R>Q	Substitution	Nonsynonymous coding	18%
MM12T	MALT1	mucosa associated lymphoid tissue lymphoma translocation gene 1	CCDS11967.1	chr18_56376682-56376682_G_T	241G>V	Substitution	Nonsynonymous coding	14%
MM12T	MAMDC2	MAM domain containing 2	CCDS6631.1	chr9_72783701-72783701_C_A	496C>X	Substitution	Nonsense	13%
MM12T	MAMDC4	MAM domain containing 4	CCDS7010.1	chr9_139747607-139747607_G_A	34A>T	Substitution	Nonsynonymous coding	32%
MM12T	MAN2A1	mannosidase, alpha, class 2A, member 1	CCDS34209.1	chr5_109026234-109026234_G_A	39R>H	Substitution	Nonsynonymous coding	36%
MM12T	MAN2A2	mannosidase, alpha, class 2A, member 2	CCDS32332.1	chr15_91455412-91455412_C_T	750A>V	Substitution	Nonsynonymous coding	26%
MM12T	MAN2B1	mannosidase, alpha, class 2B, member 1	CCDS32919.1	chr19_12759021-12759021_C_T	878A>T	Substitution	Nonsynonymous coding	15%
MM12T	MAN2B2	mannosidase, alpha, class 2B, member 2	CCDS33951.1	chr4_6612671-6612671_C_T	775S>L	Substitution	Nonsynonymous coding	27%

MM12T	MAN2B2	mannosidase, alpha, class 2B, member 2	CCDS33951.1	chr4_6612939-6612939_G_A	833A>T	Substitution	Nonsynonymous coding	36%
MM12T	MAN2C1	mannosidase, alpha, class 2C, member 1	CCDS32298.1	chr15_75656507-75656507_C_T	208R>H	Substitution	Nonsynonymous coding	35%
MM12T	MANBA	mannosidase, beta A, lysosomal	CCDS3658.1	chr4_103595095-103595095_C_T	365D>N	Substitution	Nonsynonymous coding	12%
MM12T	MAP1A	microtubule-associated protein 1A	CCDS42031.1	chr15_43814278-43814278_C_A	203L>I	Substitution	Nonsynonymous coding	16%
MM12T	MAP1A	microtubule-associated protein 1A	CCDS42031.1	chr15_43821109-43821109_C_T	2480R>C	Substitution	Nonsynonymous coding	33%
MM12T	MAP1B	microtubule-associated protein 1B	CCDS4012.1	chr5_71495196-71495196_G_A	2005S>N	Substitution	Nonsynonymous coding	36%
MM12T	MAP1S	microtubule-associated protein 1S	CCDS32954.1	chr19_17837043-17837043_C_T	284R>C	Substitution	Nonsynonymous coding	36%
MM12T	MAP2	microtubule-associated protein 2	CCDS2384.1	chr2_210561667-210561667__A	NA	Insertion	Frameshift	20%
MM12T	MAP2	microtubule-associated protein 2	CCDS2384.1	chr2_210560646-210560646_G_A	1251R>H	Substitution	Nonsynonymous coding	26%
MM12T	MAP2	microtubule-associated protein 2	CCDS2384.1	chr2_210574869-210574869_C_T	1655A>V	Substitution	Nonsynonymous coding	12%
MM12T	MAP3K1	mitogen-activated protein kinase kinase kinase 1, E3 ubiquitin protein ligase	CCDS43318.1	chr5_56161221-56161221_C_T	364R>W	Substitution	Nonsynonymous coding	23%
MM12T	MAP3K10	mitogen-activated protein kinase kinase kinase 10	CCDS12549.1	chr19_40710421-40710421_C_T	298T>M	Substitution	Nonsynonymous coding	24%
MM12T	MAP3K10	mitogen-activated protein kinase kinase kinase 10	CCDS12549.1	chr19_40719501-40719501_C_T	639P>S	Substitution	Nonsynonymous coding	34%
MM12T	MAP3K12	mitogen-activated protein kinase kinase kinase 12	CCDS8860.1	chr12_53877433-53877433_G_A	445A>V	Substitution	Nonsynonymous coding	11%
MM12T	MAP3K14	mitogen-activated protein kinase kinase kinase 14	ENST00000344686	chr17_43342550-43342550_C_T	886A>T	Substitution	Nonsynonymous coding	13%
MM12T	MAP3K4	mitogen-activated protein kinase kinase kinase 4	CCDS34565.1	chr6_161494612-161494612_G_A	689A>T	Substitution	Nonsynonymous coding	18%
MM12T	MAP3K5	mitogen-activated protein kinase kinase kinase 5	CCDS5179.1	chr6_137019666-137019666_C_T	256R>H	Substitution	Nonsynonymous coding	41%
MM12T	MAP3K7	mitogen-activated protein kinase kinase kinase 7	CCDS5028.1	chr6_91257806-91257806_C_T	347R>H	Substitution	Nonsynonymous coding	16%
MM12T	MAP3K7	mitogen-activated protein kinase kinase kinase 7	CCDS5028.1	chr6_91271364-91271364_G_T	107A>D	Substitution	Nonsynonymous coding	25%
MM12T	MAP4	microtubule-associated protein 4	CCDS33750.1	chr3_47958547-47958547_T_C	257E>G	Substitution	Nonsynonymous coding	36%
MM12T	MAP4K3	mitogen-activated protein kinase kinase kinase kinase 3	CCDS1803.1	chr2_39552733-39552733_C_T	282A>T	Substitution	Nonsynonymous coding	31%
MM12T	MAP4K5	mitogen-activated protein kinase kinase kinase kinase 5	NM_006575	chr14_50930796-50930796__T	NA	Insertion	Frameshift	31%
MM12T	MAP6	microtubule-associated protein 6	CCDS31641.1	chr11_75298840-75298840_G_T	569P>H	Substitution	Nonsynonymous coding	27%
MM12T	MAP7	microtubule-associated protein 7	CCDS5178.1	chr6_136687523-136687523_G_A	311R>X	Substitution	Nonsense	22%
MM12T	MAP7D3	MAP7 domain containing 3	CCDS44004.1	chrX_135333460-135333460_G_A	20R>W	Substitution	Nonsynonymous coding	42%
MM12T	MAPK1	mitogen-activated protein kinase 1	CCDS13795.1	chr22_22142570-22142570_G_T	278L>M	Substitution	Nonsynonymous coding	28%
MM12T	MAPK10	mitogen-activated protein kinase 10	CCDS34026.1	chr4_87019681-87019681_C_A	266R>S	Substitution	Nonsynonymous coding	35%
MM12T	MAPK13	mitogen-activated protein kinase 13	CCDS4818.1	chr6_36106832-36106832_C_T	340Q>X	Substitution	Nonsense	11%
MM12T	MAPK4	mitogen-activated protein kinase 4	CCDS42437.1	chr18_48248440-48248440_G_A	275R>H	Substitution	Nonsynonymous coding	14%
MM12T	MARK2	MAP/microtubule affinity-regulating kinase 2	CCDS41665.1	chr11_63670587-63670587_C_T	457R>W	Substitution	Nonsynonymous coding	11%
MM12T	MARS2	methionyl-tRNA synthetase 2, mitochondrial	CCDS33358.1	chr2_198571112-198571112_G_A	328G>D	Substitution	Nonsynonymous coding	18%
MM12T	MASP1	mannan-binding lectin serine peptidase 1 (C4/C2 activating component of Ra-reactive	CCDS33908.1	chr3_186953596-186953596_C_	NA	Deletion	Frameshift	32%

MM12T	MAST1	microtubule associated serine/threonine kinase 1	CCDS32921.1	chr19_12949465-12949465_A_G	27K>E	Substitution	Nonsynonymous coding	29%
MM12T	MAST2	microtubule associated serine/threonine kinase 2	CCDS41326.1	chr1_46290181-46290181_T_G	85V>G	Substitution	Nonsynonymous coding	30%
MM12T	MAST2	microtubule associated serine/threonine kinase 2	CCDS41326.1	chr1_46497185-46497185_C_T	1039R>C	Substitution	Nonsynonymous coding	38%
MM12T	MAST2	microtubule associated serine/threonine kinase 2	CCDS41326.1	chr1_46501116-46501116_C_A	1592A>D	Substitution	Nonsynonymous coding	28%
MM12T	MASTL	microtubule associated serine/threonine kinase-like	CCDS7153.1	chr10_27462119-27462119_G_A	732V>I	Substitution	Nonsynonymous coding	26%
MM12T	MAT2A	methionine adenosyltransferase II, alpha	CCDS1977.1	chr2_85769051-85769051_C_G	169R>G	Substitution	Nonsynonymous coding	23%
MM12T	MAT2A	methionine adenosyltransferase II, alpha	CCDS1977.1	chr2_85769760-85769760_G_A	281A>T	Substitution	Nonsynonymous coding	10%
MM12T	MAU2	MAU2 chromatid cohesion factor homolog (C. elegans)	CCDS32969.2	chr19_19456116-19456116_C_A	387L>M	Substitution	Nonsynonymous coding	36%
MM12T	MAX	MYC associated factor X	ENST00000441116	chr14_65568305-65568305_A_	NA	Deletion	Frameshift	56%
MM12T	MBD5	methyl-CpG binding domain protein 5	CCDS33302.1	chr2_149226977-149226977_C_T	489P>S	Substitution	Nonsynonymous coding	12%
MM12T	MBL2	mannose-binding lectin (protein C) 2, soluble	CCDS7247.1	chr10_54531365-54531365_G_A	11L>F	Substitution	Nonsynonymous coding	22%
MM12T	MBTPS1	membrane-bound transcription factor peptidase, site 1	CCDS10941.1	chr16_84092952-84092952_C_T	929R>H	Substitution	Nonsynonymous coding	31%
MM12T	MBTPS1	membrane-bound transcription factor peptidase, site 1	CCDS10941.1	chr16_84120995-84120995_C_T	368A>T	Substitution	Nonsynonymous coding	11%
MM12T	MCCC1	methylcrotonoyl-CoA carboxylase 1 (alpha)	CCDS3241.1	chr3_182755057-182755057_G_A	515L>F	Substitution	Nonsynonymous coding	32%
MM12T	MCCC1	methylcrotonoyl-CoA carboxylase 1 (alpha)	CCDS3241.1	chr3_182789122-182789122_G_T	172A>D	Substitution	Nonsynonymous coding	36%
MM12T	MCEE	methylmalonyl CoA epimerase	CCDS1915.1	chr2_71337204-71337204_G_A	143R>C	Substitution	Nonsynonymous coding	16%
MM12T	MCEE	methylmalonyl CoA epimerase	CCDS1915.1	chr2_71351631-71351631_G_A	28A>V	Substitution	Nonsynonymous coding	31%
MM12T	MCF2	MCF.2 cell line derived transforming sequence	CCDS48175.1	chrX_138699776-138699776_T_C	359R>G	Substitution	Nonsynonymous coding	41%
MM12T	MCF2L	MCF.2 cell line derived transforming sequence-like	CCDS45070.1	chr13_113729393-113729393_C_T	457R>C	Substitution	Nonsynonymous coding	31%
MM12T	MCF2L	MCF.2 cell line derived transforming sequence-like	ENST00000375591	chr13_113749347-113749347_A_G	840H>R	Substitution	Nonsynonymous coding	28%
MM12T	MCF2L	MCF.2 cell line derived transforming sequence-like	CCDS45070.1	chr13_113679076-113679076_C_T	ISV+4>	Substitution	Splice site donor	13%
MM12T	MCF2L	MCF.2 cell line derived transforming sequence-like	CCDS45070.1	chr13_113731375-113731375_C_T	561R>X	Substitution	Nonsense	29%
MM12T	MCF2L2	MCF.2 cell line derived transforming sequence-like 2	CCDS3243.1	chr3_182913016-182913016_C_T	971S>N	Substitution	Nonsynonymous coding	22%
MM12T	MCF2L2	MCF.2 cell line derived transforming sequence-like 2	CCDS3243.1	chr3_183056623-183056623_T_C	151K>E	Substitution	Nonsynonymous coding	31%
MM12T	MCF2L2	MCF.2 cell line derived transforming sequence-like 2	CCDS3243.1	chr3_183097186-183097186_C_A	58E>D	Substitution	Nonsynonymous coding	34%
MM12T	MCM10	minichromosome maintenance complex component 10	CCDS7096.1	chr10_13251120-13251120_A_G	844D>G	Substitution	Nonsynonymous coding	31%
MM12T	MCM2	minichromosome maintenance complex component 2	CCDS3043.1	chr3_127325138-127325138_G_A	284R>H	Substitution	Nonsynonymous coding	12%
MM12T	MCM3AP	minichromosome maintenance complex component 3 associated protein	CCDS13734.1	chr21_47660801-47660801_G_A	1853R>X	Substitution	Nonsense	18%
MM12T	MCM5	minichromosome maintenance complex component 5	CCDS13915.1	chr22_35804534-35804534_C_T	244H>Y	Substitution	Nonsynonymous coding	46%
MM12T	MCM5	minichromosome maintenance complex component 5	CCDS13915.1	chr22_35812354-35812354_C_G	457A>G	Substitution	Nonsynonymous coding	32%
MM12T	MCM7	minichromosome maintenance complex component 7	CCDS5683.1	chr7_99693535-99693535_G_A	486A>V	Substitution	Nonsynonymous coding	11%
MM12T	MCM9	minichromosome maintenance complex component 9	ENST00000316316	chr6_119137101-119137101_G_A	773A>V	Substitution	Nonsynonymous coding	10%

MM12T	MCOLN1	mucolipin 1	ENST00000394321	chr19_7592623-7592623_G_A	150R>H	Substitution	Nonsynonymous coding	10%
MM12T	MCOLN1	mucolipin 1	CCDS12180.1	chr19_7592790-7592790_A_G	241N>D	Substitution	Nonsynonymous coding	16%
MM12T	MCOLN3	mucolipin 3	CCDS701.1	chr1_85506800-85506800_C_T	97A>T	Substitution	Nonsynonymous coding	39%
MM12T	MCPH1	microcephalin 1	CCDS43689.1	chr8_6266841-6266841_G_T	22E>X	Substitution	Nonsense	31%
MM12T	MCTP2	multiple C2 domains, transmembrane 2	CCDS32338.1	chr15_94901790-94901790_A_G	417D>G	Substitution	Nonsynonymous coding	30%
MM12T	MDC1	mediator of DNA-damage checkpoint 1	CCDS34384.1	chr6_30670539-30670539_C_T	1994R>K	Substitution	Nonsynonymous coding	22%
MM12T	MDC1	mediator of DNA-damage checkpoint 1	CCDS34384.1	chr6_30671203-30671203_C_A	1892A>S	Substitution	Nonsynonymous coding	20%
MM12T	MDH1B	malate dehydrogenase 1B, NAD (soluble)	CCDS33365.1	chr2_207619795-207619795_G_A	283A>V	Substitution	Nonsynonymous coding	37%
MM12T	MDN1	MDN1, midasin homolog (yeast)	CCDS5024.1	chr6_90385891-90385891_C_T	4192C>Y	Substitution	Nonsynonymous coding	18%
MM12T	MDN1	MDN1, midasin homolog (yeast)	CCDS5024.1	chr6_90388327-90388327_C_T	4135A>T	Substitution	Nonsynonymous coding	41%
MM12T	MDN1	MDN1, midasin homolog (yeast)	CCDS5024.1	chr6_90405345-90405345_C_A	3250K>N	Substitution	Nonsynonymous coding	61%
MM12T	MEA1	male-enhanced antigen 1	CCDS4879.1	chr6_42981113-42981113_C_T	15A>T	Substitution	Nonsynonymous coding	52%
MM12T	MECOM	MDS1 and EVI1 complex locus	CCDS3205.1	chr3_168833367-168833367_G_A	577P>S	Substitution	Nonsynonymous coding	30%
MM12T	MECP2	methyl CpG binding protein 2 (Rett syndrome)	CCDS48193.1	chrX_153295900-153295900_T_C	472D>G	Substitution	Nonsynonymous coding	11%
MM12T	MECP2	methyl CpG binding protein 2 (Rett syndrome)	CCDS48193.1	chrX_153297785-153297785_G_A	96R>W	Substitution	Nonsynonymous coding	16%
MM12T	MED12	mediator complex subunit 12	CCDS43970.1	chrX_70340896-70340896_C_T	210A>V	Substitution	Nonsynonymous coding	31%
MM12T	MED12	mediator complex subunit 12	CCDS43970.1	chrX_70346966-70346966_G_A	945A>T	Substitution	Nonsynonymous coding	14%
MM12T	MED12	mediator complex subunit 12	CCDS43970.1	chrX_70354962-70354962_G_T	1628Q>H	Substitution	Nonsynonymous coding	27%
MM12T	MED12L	mediator complex subunit 12-like	CCDS33876.1	chr3_150845721-150845721_C_T	169A>V	Substitution	Nonsynonymous coding	28%
MM12T	MED12L	mediator complex subunit 12-like	CCDS33876.1	chr3_151075148-151075148_G_A	902A>T	Substitution	Nonsynonymous coding	29%
MM12T	MED13L	mediator complex subunit 13-like	CCDS9177.1	chr12_116446448-116446448_C_A	590Q>H	Substitution	Nonsynonymous coding	13%
MM12T	MED13L	mediator complex subunit 13-like	CCDS9177.1	chr12_116446494-116446494_G_A	575S>L	Substitution	Nonsynonymous coding	20%
MM12T	MED14	mediator complex subunit 14	CCDS14254.1	chrX_40513713-40513713_C_A	1398Q>H	Substitution	Nonsynonymous coding	27%
MM12T	MED15	mediator complex subunit 15	CCDS33602.1	chr22_20891439-20891439_G_T	35S>I	Substitution	Nonsynonymous coding	38%
MM12T	MED16	mediator complex subunit 16	CCDS12047.1	chr19_886195-886195_C_T	152A>T	Substitution	Nonsynonymous coding	35%
MM12T	MED19	mediator complex subunit 19	CCDS7966.1	chr11_57472557-57472557_G_A	121S>F	Substitution	Nonsynonymous coding	18%
MM12T	MED22	mediator complex subunit 22	CCDS6963.1	chr9_136213427-136213427_C_T	31D>N	Substitution	Nonsynonymous coding	12%
MM12T	MED23	mediator complex subunit 23	CCDS5147.1	chr6_131941863-131941863_C_A	168A>S	Substitution	Nonsynonymous coding	35%
MM12T	MED24	mediator complex subunit 24	CCDS11359.1	chr17_38178959-38178959_G_T	791L>M	Substitution	Nonsynonymous coding	16%
MM12T	MED24	mediator complex subunit 24	CCDS11359.1	chr17_38187463-38187463_C_T	368G>D	Substitution	Nonsynonymous coding	14%
MM12T	MED8	mediator complex subunit 8	CCDS486.2	chr1_43850143-43850143_T_	NA	Deletion	Frameshift	18%
MM12T	MEF2C	myocyte enhancer factor 2C	CCDS47245.1	chr5_88100467-88100467_T_C	69Y>C	Substitution	Nonsynonymous coding	15%

MM12T	MEGF10	multiple EGF-like-domains 10	CCDS4142.1	chr5_126790302-126790302_G_T	1009D>Y	Substitution	Nonsynonymous coding	13%
MM12T	MEGF6	multiple EGF-like-domains 6	CCDS41237.1	chr1_3422790-3422790_C_A	600K>N	Substitution	Nonsynonymous coding	31%
MM12T	MEGF6	multiple EGF-like-domains 6	CCDS41237.1	chr1_3417615-3417615_G_T	830C>X	Substitution	Nonsense	32%
MM12T	MEIS1	Meis homeobox 1	CCDS46309.1	chr2_66664959-66664959_C_T	35P>S	Substitution	Nonsynonymous coding	13%
MM12T	MEIS1	Meis homeobox 1	ENST00000407092	chr2_66798505-66798505_G_A	414M>I	Substitution	Nonsynonymous coding	10%
MM12T	MELK	maternal embryonic leucine zipper kinase	CCDS6606.1	chr9_36589629-36589629_A_	NA	Deletion	Frameshift	18%
MM12T	MEP1A	meprin A, alpha (PABA peptide hydrolase)	CCDS4918.1	chr6_46797216-46797216_C_T	351T>M	Substitution	Nonsynonymous coding	15%
MM12T	MEP1B	meprin A, beta	CCDS45846.1	chr18_29793468-29793468_C_T	509R>X	Substitution	Nonsense	11%
MM12T	MESP2	mesoderm posterior 2 homolog (mouse)	CCDS42078.1	chr15_90320226-90320226_C_T	213A>V	Substitution	Nonsynonymous coding	12%
MM12T	MET	met proto-oncogene (hepatocyte growth factor receptor)	CCDS47689.1	chr7_116435978-116435978_G_A	1343E>K	Substitution	Nonsynonymous coding	21%
MM12T	METTL1	methyltransferase like 1	CCDS8955.3	chr12_58163106-58163106_G_A	219R>C	Substitution	Nonsynonymous coding	15%
MM12T	METTL14	methyltransferase like 14	CCDS34053.1	chr4_119610559-119610559_C_T	64R>C	Substitution	Nonsynonymous coding	28%
MM12T	METTL14	methyltransferase like 14	CCDS34053.1	chr4_119618369-119618369_T_C	179I>T	Substitution	Nonsynonymous coding	11%
MM12T	METTL17	methyltransferase like 17	CCDS41913.1	chr14_21458205-21458205_G_A	15C>Y	Substitution	Nonsynonymous coding	13%
MM12T	METTL17	methyltransferase like 17	CCDS41913.1	chr14_21463358-21463358_G_T	304S>I	Substitution	Nonsynonymous coding	17%
MM12T	METTL23	methyltransferase like 23	CCDS45787.1	chr17_74729220-74729220_G_A	82G>D	Substitution	Nonsynonymous coding	16%
MM12T	METTL6	methyltransferase like 6	CCDS43056.1	chr3_15452786-15452786_G_T	278L>M	Substitution	Nonsynonymous coding	21%
MM12T	METTL8	methyltransferase like 8	NM_024770	chr2_172180740-172180740_G_T	389P>Q	Substitution	Nonsynonymous coding	45%
MM12T	MFAP3L	microfibrillar-associated protein 3-like	CCDS34103.1	chr4_170912961-170912961_C_A	266Q>H	Substitution	Nonsynonymous coding	36%
MM12T	MFF	mitochondrial fission factor	CCDS2465.1	chr2_228205057-228205057_C_A	160A>D	Substitution	Nonsynonymous coding	32%
MM12T	MFGE8	milk fat globule-EGF factor 8 protein	CCDS10347.1	chr15_89449105-89449105_C_T	190A>T	Substitution	Nonsynonymous coding	31%
MM12T	MFHAS1	malignant fibrous histiocytoma amplified sequence 1	CCDS34844.1	chr8_8747686-8747686__T	NA	Insertion	Frameshift	21%
MM12T	MFI2	antigen p97 (melanoma associated) identified by monoclonal antibodies 133.2 and 96.5	CCDS3325.1	chr3_196735716-196735716_C_A	549S>I	Substitution	Nonsynonymous coding	32%
MM12T	MFI2	antigen p97 (melanoma associated) identified by monoclonal antibodies 133.2 and 96.5	CCDS3325.1	chr3_196742244-196742244_G_A	409R>W	Substitution	Nonsynonymous coding	28%
MM12T	MFNG	MFNG O-fucosylpeptide 3-beta-N-acetylglucosaminyltransferase	CCDS13947.1	chr22_37870629-37870629_C_T	245G>D	Substitution	Nonsynonymous coding	37%
MM12T	MFRP	membrane frizzled-related protein	CCDS8421.1	chr11_119212269-119212269_C_T	577A>T	Substitution	Nonsynonymous coding	32%
MM12T	MFSD2B	major facilitator superfamily domain containing 2B	CCDS46228.1	chr2_24236251-24236251_C_A	65L>M	Substitution	Nonsynonymous coding	35%
MM12T	MFSD6	major facilitator superfamily domain containing 6	CCDS2306.1	chr2_191300954-191300954_G_A	67D>N	Substitution	Nonsynonymous coding	13%
MM12T	MFSD6L	major facilitator superfamily domain containing 6-like	CCDS11146.1	chr17_8701874-8701874_G_A	189P>S	Substitution	Nonsynonymous coding	27%
MM12T	MGA	MGA, MAX dimerization protein	NM_001164273	chr15_41989002-41989002_G_T	598K>N	Substitution	Nonsynonymous coding	26%
MM12T	MGA	MGA, MAX dimerization protein	NM_001164273	chr15_42041026-42041026_A_G	1802R>G	Substitution	Nonsynonymous coding	34%
MM12T	MGA	MGA, MAX dimerization protein	NM_001164273	chr15_42042505-42042505_G_A	2234A>T	Substitution	Nonsynonymous coding	24%

MM12T	MGAM	maltase-glucoamylase (alpha-glucosidase)	CCDS47727.1	chr7_141722139-141722139_C_G	261T>S	Substitution	Nonsynonymous coding	30%
MM12T	MGAM	maltase-glucoamylase (alpha-glucosidase)	CCDS47727.1	chr7_141724872-141724872_C_T	302A>V	Substitution	Nonsynonymous coding	11%
MM12T	MGAM	maltase-glucoamylase (alpha-glucosidase)	CCDS47727.1	chr7_141797430-141797430_G_A	1681R>H	Substitution	Nonsynonymous coding	33%
MM12T	MGAM	maltase-glucoamylase (alpha-glucosidase)	CCDS47727.1	chr7_141736778-141736778_G_A	ISV+1>	Substitution	Splice site donor	11%
MM12T	MGARP	mitochondria-localized glutamic acid-rich protein	CCDS43269.1	chr4_140188177-140188177_G_A	100A>V	Substitution	Nonsynonymous coding	16%
MM12T	MGAT4B	mannosyl (alpha-1,3-)-glycoprotein beta-1,4-N-acetylglucosaminyltransferase, isozyme B	CCDS4449.1	chr5_179228804-179228804_C_T	103R>H	Substitution	Nonsynonymous coding	11%
MM12T	MGAT4B	mannosyl (alpha-1,3-)-glycoprotein beta-1,4-N-acetylglucosaminyltransferase, isozyme B	CCDS4449.1	chr5_179228846-179228846_G_A	89A>V	Substitution	Nonsynonymous coding	37%
MM12T	MGAT4C	mannosyl (alpha-1,3-)-glycoprotein beta-1,4-N-acetylglucosaminyltransferase, isozyme C	CCDS9030.1	chr12_86373677-86373677_A_	NA	Deletion	Frameshift	22%
MM12T	MGAT5	mannosyl (alpha-1,6-)-glycoprotein beta-1,6-N-acetyl-glucosaminyltransferase	CCDS2171.1	chr2_135206222-135206222_A_G	677Y>C	Substitution	Nonsynonymous coding	41%
MM12T	MGST1	microsomal glutathione S-transferase 1	CCDS8677.1	chr12_16510545-16510545_G_A	45A>T	Substitution	Nonsynonymous coding	35%
MM12T	MIA3	melanoma inhibitory activity family, member 3	CCDS41470.1	chr1_222794539-222794539_G_A	58G>S	Substitution	Nonsynonymous coding	30%
MM12T	MIB1	mindbomb E3 ubiquitin protein ligase 1	CCDS11871.1	chr18_19321602-19321602_C_T	20R>C	Substitution	Nonsynonymous coding	36%
MM12T	MIB1	mindbomb E3 ubiquitin protein ligase 1	CCDS11871.1	chr18_19345871-19345871_G_A	123R>H	Substitution	Nonsynonymous coding	37%
MM12T	MIB2	mindbomb E3 ubiquitin protein ligase 2	CCDS41224.1	chr1_1563938-1563938_C_A	738H>N	Substitution	Nonsynonymous coding	30%
MM12T	MICAL1	microtubule associated monoxygenase, calponin and LIM domain containing 1	CCDS5076.1	chr6_109767434-109767434_G_T	829P>H	Substitution	Nonsynonymous coding	25%
MM12T	MICALL2	MICAL-like 2	CCDS5324.1	chr7_1477748-1477748_G_A	766R>W	Substitution	Nonsynonymous coding	10%
MM12T	MIIP	migration and invasion inhibitory protein	CCDS143.1	chr1_12090096-12090096_C_T	286P>L	Substitution	Nonsynonymous coding	32%
MM12T	MINK1	misshapen-like kinase 1	CCDS45588.1	chr17_4789858-4789858_C_T	296R>W	Substitution	Nonsynonymous coding	21%
MM12T	MINK1	misshapen-like kinase 1	CCDS45588.1	chr17_4793989-4793989_C_T	509R>W	Substitution	Nonsynonymous coding	25%
MM12T	MIR1307	microRNA 1307	hsa-mir-1307	chr10_105154104-105154104_T_G	19T>P	Substitution	Nonsynonymous coding	33%
MM12T	MIR331	microRNA 331	hsa-mir-331	chr12_95702218-95702218_G_A	8C>Y	Substitution	Nonsynonymous coding	38%
MM12T	MIR411	microRNA 411	hsa-mir-411	chr14_101489692-101489692_C_T	11R>C	Substitution	Nonsynonymous coding	15%
MM12T	MIR425	microRNA 425	hsa-mir-425	chr3_49057595-49057595_C_T	25A>T	Substitution	Nonsynonymous coding	16%
MM12T	MIR429	microRNA 429	hsa-mir-429	chr1_1104389-1104389_G_A	2R>Q	Substitution	Nonsynonymous coding	29%
MM12T	MIR448	microRNA 448	hsa-mir-448	chrX_114058123-114058123_C_T	36S>L	Substitution	Nonsynonymous coding	15%
MM12T	MIR573	microRNA 573	hsa-mir-573	chr4_24521841-24521841_G_A	25R>C	Substitution	Nonsynonymous coding	44%
MM12T	MIR675	microRNA 675	hsa-mir-675	chr11_2018019-2018019_G_A	15R>C	Substitution	Nonsynonymous coding	13%
MM12T	MIR885	microRNA 885	hsa-mir-885	chr3_10436209-10436209_T_C	13H>R	Substitution	Nonsynonymous coding	33%
MM12T	MIXL1	Mix paired-like homeobox	CCDS1552.1	chr1_226413271-226413271_G_A	153A>T	Substitution	Nonsynonymous coding	36%
MM12T	MKI67	antigen identified by monoclonal antibody Ki-67	CCDS7659.1	chr10_129904502-129904502_C_T	1868D>N	Substitution	Nonsynonymous coding	43%
MM12T	MKI67	antigen identified by monoclonal antibody Ki-67	CCDS7659.1	chr10_129905210-129905210_G_A	1632R>W	Substitution	Nonsynonymous coding	31%
MM12T	MKLN1	muskelin 1, intracellular mediator containing kelch motifs	CCDS34754.1	chr7_131082131-131082131_G_T	169S>I	Substitution	Nonsynonymous coding	21%

MM12T	MKNK1	MAP kinase interacting serine/threonine kinase 1	CCDS538.1	chr1_47027182-47027182_C_T	366A>T	Substitution	Nonsynonymous coding	34%
MM12T	MKRN2	makorin ring finger protein 2	CCDS33702.1	chr3_12611712-12611712_G_A	100G>R	Substitution	Nonsynonymous coding	33%
MM12T	MLIP	muscular LMNA-interacting protein	CCDS4954.1	chr6_54066922-54066922_G_A	335R>H	Substitution	Nonsynonymous coding	13%
MM12T	MLKL	mixed lineage kinase domain-like	CCDS32487.1	chr16_74708978-74708978_G_A	421R>C	Substitution	Nonsynonymous coding	15%
MM12T	MLL	myeloid/lymphoid or mixed-lineage leukemia (trithorax homolog, Drosophila)	CCDS31686.1	chr11_118372555-118372555_G_A	2160R>Q	Substitution	Nonsynonymous coding	31%
MM12T	MLL	myeloid/lymphoid or mixed-lineage leukemia (trithorax homolog, Drosophila)	CCDS31686.1	chr11_118342376-118342376_G_A	ISV-1>	Substitution	Splice site acceptor	31%
MM12T	MLL2	myeloid/lymphoid or mixed-lineage leukemia 2	CCDS44873.1	chr12_49431726-49431726_G_A	3138T>I	Substitution	Nonsynonymous coding	10%
MM12T	MLL2	myeloid/lymphoid or mixed-lineage leukemia 2	CCDS44873.1	chr12_49448112-49448112_G_A	163A>V	Substitution	Nonsynonymous coding	32%
MM12T	MLL2	myeloid/lymphoid or mixed-lineage leukemia 2	CCDS44873.1	chr12_49431178-49431178_G_A	3321R>X	Substitution	Nonsense	34%
MM12T	MLL3	myeloid/lymphoid or mixed-lineage leukemia 3	CCDS5931.1	chr7_151845314-151845314_C_G	4566M>I	Substitution	Nonsynonymous coding	33%
MM12T	MLL5	myeloid/lymphoid or mixed-lineage leukemia 5 (trithorax homolog, Drosophila)	CCDS34723.1	chr7_104753679-104753679_G_T	1826G>X	Substitution	Nonsense	26%
MM12T	MLLT10	myeloid/lymphoid or mixed-lineage leukemia (trithorax homolog, Drosophila); translocated	CCDS7135.1	chr10_22019826-22019826_C_T	ISV-3>	Substitution	Splice site acceptor	31%
MM12T	MLLT6	myeloid/lymphoid or mixed-lineage leukemia (trithorax homolog, Drosophila); translocated	CCDS11327.1	chr17_36878343-36878343__G	NA	Insertion	Frameshift	26%
MM12T	MLXIPL	MLX interacting protein-like	CCDS5553.1	chr7_73010593-73010593_G_A	650R>W	Substitution	Nonsynonymous coding	30%
MM12T	MLXIPL	MLX interacting protein-like	CCDS5553.1	chr7_73010971-73010971_C_T	607S>N	Substitution	Nonsynonymous coding	32%
MM12T	MMP1	matrix metallopeptidase 1 (interstitial collagenase)	CCDS8322.1	chr11_102665974-102665974_G_A	277A>V	Substitution	Nonsynonymous coding	29%
MM12T	MMP11	matrix metallopeptidase 11 (stromelysin 3)	CCDS13816.1	chr22_24124523-24124523_G_A	396E>K	Substitution	Nonsynonymous coding	32%
MM12T	MMP11	matrix metallopeptidase 11 (stromelysin 3)	CCDS13816.1	chr22_24125687-24125687_C_T	475P>S	Substitution	Nonsynonymous coding	37%
MM12T	MMP12	matrix metallopeptidase 12 (macrophage elastase)	ENST00000326227	chr11_102743635-102743635_G_A	104P>S	Substitution	Nonsynonymous coding	14%
MM12T	MMP12	matrix metallopeptidase 12 (macrophage elastase)	ENST00000326227	chr11_102737997-102737997_C_A	ISV+4>	Substitution	Splice site donor	39%
MM12T	MMP14	matrix metallopeptidase 14 (membrane-inserted)	CCDS9577.1	chr14_23312973-23312973_G_A	302R>Q	Substitution	Nonsynonymous coding	32%
MM12T	MMP17	matrix metallopeptidase 17 (membrane-inserted)	CCDS31927.1	chr12_132325257-132325257_G_A	188D>N	Substitution	Nonsynonymous coding	32%
MM12T	MMP25	matrix metallopeptidase 25	CCDS10492.1	chr16_3100415-3100415_G_A	177A>T	Substitution	Nonsynonymous coding	27%
MM12T	MMP25	matrix metallopeptidase 25	CCDS10492.1	chr16_3107058-3107058_C_T	229A>V	Substitution	Nonsynonymous coding	16%
MM12T	MMP26	matrix metallopeptidase 26	CCDS7752.1	chr11_5010915-5010915_C_T	46S>L	Substitution	Nonsynonymous coding	13%
MM12T	MMP28	matrix metallopeptidase 28	ENST00000338839	chr17_34100317-34100317_C_T	157A>T	Substitution	Nonsynonymous coding	10%
MM12T	MMP28	matrix metallopeptidase 28	ENST00000338839	chr17_34100375-34100375_C_T	138R>H	Substitution	Nonsynonymous coding	13%
MM12T	MMP9	matrix metallopeptidase 9 (gelatinase B, 92kDa gelatinase, 92kDa type IV collagenase)	CCDS13390.1	chr20_44642002-44642002_G_A	480R>H	Substitution	Nonsynonymous coding	18%
MM12T	MMRN1	multimerin 1	CCDS3635.1	chr4_90816409-90816409_G_T	96R>I	Substitution	Nonsynonymous coding	21%
MM12T	MMS22L	MMS22-like, DNA repair protein	CCDS5039.1	chr6_97720606-97720606_C_A	194A>S	Substitution	Nonsynonymous coding	23%
MM12T	MN1	meningioma (disrupted in balanced translocation) 1	CCDS42998.1	chr22_28194145-28194145_C_T	796R>H	Substitution	Nonsynonymous coding	42%
MM12T	MN1	meningioma (disrupted in balanced translocation) 1	CCDS42998.1	chr22_28194622-28194622_G_A	637P>L	Substitution	Nonsynonymous coding	12%

MM12T	MNS1	meiosis-specific nuclear structural 1	CCDS10158.1	chr15_56726535-56726535_T_C	357Q>R	Substitution	Nonsynonymous coding	25%
MM12T	MNT	MNT, MAX dimerization protein	CCDS11018.1	chr17_2290542-2290542_C_T	468A>T	Substitution	Nonsynonymous coding	18%
MM12T	MNT	MNT, MAX dimerization protein	CCDS11018.1	chr17_2298377-2298377_G_T	149L>I	Substitution	Nonsynonymous coding	37%
MM12T	MNT	MNT, MAX dimerization protein	CCDS11018.1	chr17_2291271-2291271_G_A	294Q>X	Substitution	Nonsense	18%
MM12T	MOB2	MOB kinase activator 2	ENST00000399693	chr11_1491484-1491484_G_A	242A>V	Substitution	Nonsynonymous coding	27%
MM12T	MOB3C	MOB kinase activator 3C	CCDS539.1	chr1_47075751-47075751_C_T	234V>I	Substitution	Nonsynonymous coding	29%
MM12T	MOCS3	molybdenum cofactor synthesis 3	CCDS13435.1	chr20_49575725-49575725_G_A	116V>M	Substitution	Nonsynonymous coding	24%
MM12T	MOCS3	molybdenum cofactor synthesis 3	CCDS13435.1	chr20_49576517-49576517_C_T	380R>C	Substitution	Nonsynonymous coding	15%
MM12T	MON1A	MON1 homolog A (yeast)	CCDS2808.2	chr3_49948233-49948233_G_A	330A>V	Substitution	Nonsynonymous coding	11%
MM12T	MORC4	MORC family CW-type zinc finger 4	CCDS14525.2	chrX_106186344-106186344_G_A	593P>S	Substitution	Nonsynonymous coding	32%
MM12T	MOSPD2	motile sperm domain containing 2	CCDS14162.1	chrX_14937819-14937819_G_T	482R>M	Substitution	Nonsynonymous coding	22%
MM12T	MOXD1	monooxygenase, DBH-like 1	CCDS5152.2	chr6_132618353-132618353_G_T	594S>Y	Substitution	Nonsynonymous coding	35%
MM12T	MPEG1	macrophage expressed 1	CCDS41650.1	chr11_58979047-58979047_T_G	431N>T	Substitution	Nonsynonymous coding	11%
MM12T	MPEG1	macrophage expressed 1	CCDS41650.1	chr11_58979924-58979924_G_T	139L>I	Substitution	Nonsynonymous coding	18%
MM12T	MPHOSPH8	M-phase phosphoprotein 8	CCDS9287.1	chr13_20244433-20244433_C_T	796A>V	Substitution	Nonsynonymous coding	29%
MM12T	MPHOSPH9	M-phase phosphoprotein 9	CCDS9243.1	chr12_123706143-123706143_C_A	64K>N	Substitution	Nonsynonymous coding	29%
MM12T	MPO	myeloperoxidase	CCDS11604.1	chr17_56356680-56356680_C_T	219R>H	Substitution	Nonsynonymous coding	13%
MM12T	MPPE1	metallophosphoesterase 1	CCDS11853.1	chr18_11886569-11886569_C_T	266A>T	Substitution	Nonsynonymous coding	34%
MM12T	MRAP	melanocortin 2 receptor accessory protein	CCDS13613.1	chr21_33671333-33671333_C_A	17Y>X	Substitution	Nonsense	17%
MM12T	MRC2	mannose receptor, C type 2	CCDS11634.1	chr17_60769794-60769794_G_A	1474M>I	Substitution	Nonsynonymous coding	34%
MM12T	MROH1	maestro heat-like repeat family member 1	CCDS47938.1	chr8_145235222-145235222_G_A	148A>T	Substitution	Nonsynonymous coding	39%
MM12T	MROH2A	maestro heat-like repeat family member 2A	ENST00000396517	chr2_234713698-234713698_C_T	696R>C	Substitution	Nonsynonymous coding	18%
MM12T	MROH7	maestro heat-like repeat family member 7	CCDS41342.2	chr1_55158166-55158166_G_T	927E>D	Substitution	Nonsynonymous coding	19%
MM12T	MROH9	maestro heat-like repeat family member 9	CCDS41436.1	chr1_170952602-170952602_G_T	219S>I	Substitution	Nonsynonymous coding	30%
MM12T	MRPL1	mitochondrial ribosomal protein L1	CCDS3583.2	chr4_78804521-78804521_G_A	90R>H	Substitution	Nonsynonymous coding	12%
MM12T	MRPL10	mitochondrial ribosomal protein L10	CCDS11516.1	chr17_45908830-45908830_C_A	16Q>H	Substitution	Nonsynonymous coding	10%
MM12T	MRPL16	mitochondrial ribosomal protein L16	CCDS7976.1	chr11_59573827-59573827_C_T	250R>H	Substitution	Nonsynonymous coding	47%
MM12T	MRPL37	mitochondrial ribosomal protein L37	ENST00000329505	chr1_54686467-54686467_C_T	397R>W	Substitution	Nonsynonymous coding	32%
MM12T	MRPS11	mitochondrial ribosomal protein S11	CCDS10342.1	chr15_89011000-89011000_C_T	18P>S	Substitution	Nonsynonymous coding	35%
MM12T	MRPS27	mitochondrial ribosomal protein S27	CCDS4013.1	chr5_71593475-71593475_G_T	69S>Y	Substitution	Nonsynonymous coding	20%
MM12T	MRPS30	mitochondrial ribosomal protein S30	CCDS3951.1	chr5_44811248-44811248_C_T	247L>F	Substitution	Nonsynonymous coding	19%
MM12T	MRPS35	mitochondrial ribosomal protein S35	CCDS8714.1	chr12_27888446-27888446_C_T	197R>X	Substitution	Nonsense	34%

MM12T	MRVI1	murine retrovirus integration site 1 homolog	CCDS44538.1	chr11_10631290-10631290_C_A	510R>M	Substitution	Nonsynonymous coding	47%
MM12T	MS4A12	membrane-spanning 4-domains, subfamily A, member 12	CCDS7988.1	chr11_60271208-60271208_C_A	169S>Y	Substitution	Nonsynonymous coding	31%
MM12T	MS4A6A	membrane-spanning 4-domains, subfamily A, member 6A	CCDS7981.1	chr11_59940505-59940505_G_T	216P>H	Substitution	Nonsynonymous coding	23%
MM12T	MS4A8B	membrane-spanning 4-domains, subfamily A, member 8B	CCDS7990.1	chr11_60476210-60476210_C_A	164H>N	Substitution	Nonsynonymous coding	19%
MM12T	MS4A8B	membrane-spanning 4-domains, subfamily A, member 8B	CCDS7990.1	chr11_60482610-60482610_G_A	ISV+3>	Substitution	Splice site donor	35%
MM12T	MSC	musculin	CCDS43746.1	chr8_72756124-72756124_G_A	97A>V	Substitution	Nonsynonymous coding	38%
MM12T	MSH3	mutS homolog 3 (E. coli)	CCDS34195.1	chr5_79970915-79970915_A_	NA	Deletion	Frameshift	36%
MM12T	MSH5	mutS homolog 5 (E. coli)	CCDS34409.1	chr6_31721134-31721134_T_C	307V>A	Substitution	Nonsynonymous coding	46%
MM12T	MSH6	mutS homolog 6 (E. coli)	CCDS1836.1	chr2_48026666-48026666_G_	NA	Deletion	Frameshift	25%
MM12T	MSH6	mutS homolog 6 (E. coli)	CCDS1836.1	chr2_48027453-48027453_G_A	777W>X	Substitution	Nonsense	23%
MM12T	MSL2	male-specific lethal 2 homolog (Drosophila)	CCDS33861.1	chr3_135870534-135870534_T_	NA	Deletion	Frameshift	17%
MM12T	MSL3	male-specific lethal 3 homolog (Drosophila)	CCDS14147.1	chrX_11777931-11777931_G_A	41G>E	Substitution	Nonsynonymous coding	15%
MM12T	MSLN	mesothelin	CCDS32356.1	chr16_816801-816801_G_A	463S>N	Substitution	Nonsynonymous coding	27%
MM12T	MSLNL	mesothelin-like	CCDS42093.1	chr16_830304-830304_G_A	233P>S	Substitution	Nonsynonymous coding	12%
MM12T	MSS51	MSS51 mitochondrial translational activator	CCDS31221.1	chr10_75187501-75187501_C_T	83V>I	Substitution	Nonsynonymous coding	29%
MM12T	MST4	Serine/threonine-protein kinase MST4 [Source:UniProtKB/Swiss-Prot;Acc:Q9P289]	CCDS14631.1	chrX_131203585-131203585_G_A	226R>K	Substitution	Nonsynonymous coding	43%
MM12T	MSX1	msh homeobox 1	CCDS3378.2	chr4_4862093-4862093_C_T	156A>V	Substitution	Nonsynonymous coding	29%
MM12T	MT1G	metallothionein 1G	ENST00000245183	chr16_56700749-56700749_C_T	77R>H	Substitution	Nonsynonymous coding	15%
MM12T	MTA1	metastasis associated 1	CCDS32169.1	chr14_105931075-105931075_C_A	470A>D	Substitution	Nonsynonymous coding	25%
MM12T	MTA2	metastasis associated 1 family, member 2	CCDS8022.1	chr11_62361478-62361478_G_A	626R>W	Substitution	Nonsynonymous coding	14%
MM12T	MTBP	Mdm2, transformed 3T3 cell double minute 2, p53 binding protein (mouse) binding protein,	CCDS6333.1	chr8_121514790-121514790_G_A	556W>X	Substitution	Nonsense	11%
MM12T	MTDH	metadherin	CCDS6274.1	chr8_98703366-98703366_G_A	333G>E	Substitution	Nonsynonymous coding	12%
MM12T	MTERFD1	MTERF domain containing 1	CCDS6270.1	chr8_97251893-97251893_A_	NA	Deletion	Frameshift	16%
MM12T	MTHFD1	methylenetetrahydrofolate dehydrogenase (NADP+ dependent) 1,	CCDS9763.1	chr14_64921540-64921540_C_T	889R>C	Substitution	Nonsynonymous coding	30%
MM12T	MTHFD1L	methylenetetrahydrofolate dehydrogenase (NADP+ dependent) 1-like	CCDS5228.1	chr6_151243387-151243387_G_T	344R>M	Substitution	Nonsynonymous coding	12%
MM12T	MTHFR	methylenetetrahydrofolate reductase (NAD(P)H)	CCDS137.1	chr1_11862998-11862998_C_A	59W>L	Substitution	Nonsynonymous coding	30%
MM12T	MTMR4	myotubularin related protein 4	CCDS11608.1	chr17_56584615-56584615_C_T	244R>H	Substitution	Nonsynonymous coding	35%
MM12T	MTMR7	myotubularin related protein 7	CCDS34851.1	chr8_17170806-17170806_C_T	324A>T	Substitution	Nonsynonymous coding	22%
MM12T	MTMR8	myotubularin related protein 8	CCDS14379.1	chrX_63569893-63569893_C_T	176V>M	Substitution	Nonsynonymous coding	12%
MM12T	MTNR1B	melatonin receptor 1B	CCDS8290.1	chr11_92703061-92703061_G_A	57G>D	Substitution	Nonsynonymous coding	22%
MM12T	MTO1	mitochondrial translation optimization 1 homolog (S. cerevisiae)	CCDS47452.1	chr6_74210304-74210304_C_T	682A>V	Substitution	Nonsynonymous coding	21%
MM12T	MTOR	mechanistic target of rapamycin (serine/threonine kinase)	CCDS127.1	chr1_11168260-11168260_G_T	2538L>I	Substitution	Nonsynonymous coding	11%

MM12T	MTOR	mechanistic target of rapamycin (serine/threonine kinase)	CCDS127.1	chr1_11288849-11288849_G_A	969T>I	Substitution	Nonsynonymous coding	36%
MM12T	MTOR	mechanistic target of rapamycin (serine/threonine kinase)	CCDS127.1	chr1_11294225-11294225_C_T	769R>H	Substitution	Nonsynonymous coding	34%
MM12T	MTOR	mechanistic target of rapamycin (serine/threonine kinase)	CCDS127.1	chr1_11307714-11307714_C_T	398R>H	Substitution	Nonsynonymous coding	12%
MM12T	MTR	5-methyltetrahydrofolate-homocysteine methyltransferase	CCDS1614.1	chr1_237044081-237044081_C_T	874P>L	Substitution	Nonsynonymous coding	14%
MM12T	MTRF1	mitochondrial translational release factor 1	CCDS9378.1	chr13_41826834-41826834_G_A	215S>L	Substitution	Nonsynonymous coding	28%
MM12T	MTSS1	metastasis suppressor 1	CCDS6353.1	chr8_125577954-125577954_G_A	258T>M	Substitution	Nonsynonymous coding	29%
MM12T	MTUS2	microtubule associated tumor suppressor candidate 2	CCDS45022.1	chr13_30075325-30075325_T_C	1307V>A	Substitution	Nonsynonymous coding	10%
MM12T	MUC1	mucin 1, cell surface associated	CCDS1098.1	chr1_155159936-155159936_C_T	198A>T	Substitution	Nonsynonymous coding	13%
MM12T	MUC16	mucin 16, cell surface associated	NM_024690	chr19_9075824-9075824__T	NA	Insertion	Frameshift	22%
MM12T	MUC16	mucin 16, cell surface associated	NM_024690	chr19_9046679-9046679_G_T	11651P>H	Substitution	Nonsynonymous coding	26%
MM12T	MUC16	mucin 16, cell surface associated	NM_024690	chr19_9057766-9057766_T_G	9894T>P	Substitution	Nonsynonymous coding	30%
MM12T	MUC16	mucin 16, cell surface associated	NM_024690	chr19_9059020-9059020_C_A	9476D>Y	Substitution	Nonsynonymous coding	40%
MM12T	MUC16	mucin 16, cell surface associated	NM_024690	chr19_9068968-9068968_C_T	6160G>S	Substitution	Nonsynonymous coding	14%
MM12T	MUC16	mucin 16, cell surface associated	NM_024690	chr19_9083702-9083702_G_A	2705P>S	Substitution	Nonsynonymous coding	38%
MM12T	MUC16	mucin 16, cell surface associated	NM_024690	chr19_9088046-9088046_C_T	1257A>T	Substitution	Nonsynonymous coding	28%
MM12T	MUC16	mucin 16, cell surface associated	NM_024690	chr19_9088617-9088617_C_A	1066E>D	Substitution	Nonsynonymous coding	40%
MM12T	MUC19	mucin 19, oligomeric	ENST00000398702	chr12_40912808-40912808_C_T	127A>V	Substitution	Nonsynonymous coding	33%
MM12T	MUC19	mucin 19, oligomeric	ENST00000440207	chr12_40935432-40935432_G_T	153E>D	Substitution	Nonsynonymous coding	36%
MM12T	MUC19	mucin 19, oligomeric	ENST00000380816	chr12_40941582-40941582_A_T	210D>V	Substitution	Nonsynonymous coding	26%
MM12T	MUC20	mucin 20, cell surface associated	NM_152673	chr3_195460039-195460039_G_A	523A>T	Substitution	Nonsynonymous coding	28%
MM12T	MUC3A	mucin 3A, cell surface associated	ENST00000319509	chr7_100551889-100551889_G_A	214E>K	Substitution	Nonsynonymous coding	19%
MM12T	MUC4	mucin 4, cell surface associated	CCDS3310.1	chr3_195481222-195481222_C_T	828G>R	Substitution	Nonsynonymous coding	41%
MM12T	MUC4	mucin 4, cell surface associated	CCDS3310.1	chr3_195493582-195493582_G_A	288P>S	Substitution	Nonsynonymous coding	29%
MM12T	MUC4	mucin 4, cell surface associated	NM_018406	chr3_195517227-195517227_C_A	408E>D	Substitution	Nonsynonymous coding	35%
MM12T	MUC5B	mucin 5B, oligomeric mucus/gel-forming	CCDS44515.1	chr11_1253903-1253903_G_T	659E>D	Substitution	Nonsynonymous coding	16%
MM12T	MUC5B	mucin 5B, oligomeric mucus/gel-forming	CCDS44515.1	chr11_1253992-1253992_G_A	689G>D	Substitution	Nonsynonymous coding	10%
MM12T	MUC5B	mucin 5B, oligomeric mucus/gel-forming	CCDS44515.1	chr11_1275427-1275427_G_A	5111R>H	Substitution	Nonsynonymous coding	36%
MM12T	MUC6	mucin 6, oligomeric mucus/gel-forming	CCDS44513.1	chr11_1019329-1019329_G_A	1326R>W	Substitution	Nonsynonymous coding	32%
MM12T	MUC6	mucin 6, oligomeric mucus/gel-forming	CCDS44513.1	chr11_1026040-1026040_C_T	883R>H	Substitution	Nonsynonymous coding	23%
MM12T	MUC6	mucin 6, oligomeric mucus/gel-forming	CCDS44513.1	chr11_1021264-1021264_G_T	1180C>X	Substitution	Nonsense	24%
MM12T	MUL1	mitochondrial E3 ubiquitin protein ligase 1	CCDS208.1	chr1_20827670-20827670_A_G	191V>A	Substitution	Nonsynonymous coding	36%
MM12T	MVP	major vault protein	CCDS10656.1	chr16_29853019-29853019_G_A	432D>N	Substitution	Nonsynonymous coding	23%

MM12T	MX2	myxovirus (influenza virus) resistance 2 (mouse)	CCDS13672.1	chr21_42751962-42751962_C_T	154P>L	Substitution	Nonsynonymous coding	21%
MM12T	MX2	myxovirus (influenza virus) resistance 2 (mouse)	CCDS13672.1	chr21_42780068-42780068_G_A	686A>T	Substitution	Nonsynonymous coding	11%
MM12T	MXRA5	matrix-remodelling associated 5	CCDS14124.1	chrX_3238487-3238487_G_A	1747R>W	Substitution	Nonsynonymous coding	16%
MM12T	MXRA5	matrix-remodelling associated 5	CCDS14124.1	chrX_3242828-3242828_C_A	300E>X	Substitution	Nonsense	36%
MM12T	MXRA8	matrix-remodelling associated 8	CCDS24.1	chr1_1289466-1289466_T_C	389E>G	Substitution	Nonsynonymous coding	31%
MM12T	MYBL2	v-myb myeloblastosis viral oncogene homolog (avian)-like 2	CCDS13322.1	chr20_42343802-42343802_G_T	618S>I	Substitution	Nonsynonymous coding	27%
MM12T	MYBPC1	myosin binding protein C, slow type	CCDS9083.1	chr12_102067274-102067274_C_T	895R>C	Substitution	Nonsynonymous coding	16%
MM12T	MYBPC2	myosin binding protein C, fast type	CCDS46152.1	chr19_50939104-50939104_G_A	61V>I	Substitution	Nonsynonymous coding	31%
MM12T	MYBPC3	myosin binding protein C, cardiac	ENST00000399249	chr11_47353710-47353710_G_T	1243P>T	Substitution	Nonsynonymous coding	39%
MM12T	MYC	v-myc myelocytomatosis viral oncogene homolog (avian)	CCDS6359.2	chr8_128753200-128753200_C_T	454A>V	Substitution	Nonsynonymous coding	28%
MM12T	MYCBP2	MYC binding protein 2, E3 ubiquitin protein ligase	CCDS31994.1	chr13_77792087-77792087_C_T	944C>Y	Substitution	Nonsynonymous coding	14%
MM12T	MYCBPAP	MYCBP associated protein	CCDS32680.2	chr17_48594799-48594799_C_A	160P>H	Substitution	Nonsynonymous coding	39%
MM12T	MYF6	myogenic factor 6 (herculin)	CCDS9019.1	chr12_81101850-81101850_C_T	118R>W	Substitution	Nonsynonymous coding	35%
MM12T	MYH10	myosin, heavy chain 10, non-muscle	CCDS11144.1	chr17_8402670-8402670_G_A	1259A>V	Substitution	Nonsynonymous coding	34%
MM12T	MYH11	myosin, heavy chain 11, smooth muscle	CCDS45423.1	chr16_15811079-15811079_C_T	1815V>I	Substitution	Nonsynonymous coding	27%
MM12T	MYH13	myosin, heavy chain 13, skeletal muscle	CCDS45613.1	chr17_10210266-10210266_G_A	1762A>V	Substitution	Nonsynonymous coding	41%
MM12T	MYH13	myosin, heavy chain 13, skeletal muscle	CCDS45613.1	chr17_10216024-10216024_G_A	1411A>V	Substitution	Nonsynonymous coding	11%
MM12T	MYH13	myosin, heavy chain 13, skeletal muscle	CCDS45613.1	chr17_10216055-10216055_G_A	1401L>F	Substitution	Nonsynonymous coding	24%
MM12T	MYH13	myosin, heavy chain 13, skeletal muscle	CCDS45613.1	chr17_10216675-10216675_G_A	ISV-4>	Substitution	Splice site acceptor	12%
MM12T	MYH15	myosin, heavy chain 15	CCDS43127.1	chr3_108189052-108189052_C_T	484S>N	Substitution	Nonsynonymous coding	44%
MM12T	MYH2	myosin, heavy chain 2, skeletal muscle, adult	CCDS11156.1	chr17_10429087-10429087_C_T	1432E>K	Substitution	Nonsynonymous coding	14%
MM12T	MYH2	myosin, heavy chain 2, skeletal muscle, adult	CCDS11156.1	chr17_10436717-10436717_G_T	776L>M	Substitution	Nonsynonymous coding	22%
MM12T	MYH3	myosin, heavy chain 3, skeletal muscle, embryonic	CCDS11157.1	chr17_10544635-10544635_G_A	672R>C	Substitution	Nonsynonymous coding	25%
MM12T	MYH3	myosin, heavy chain 3, skeletal muscle, embryonic	CCDS11157.1	chr17_10549143-10549143_C_T	341G>D	Substitution	Nonsynonymous coding	10%
MM12T	MYH4	myosin, heavy chain 4, skeletal muscle	CCDS11154.1	chr17_10354116-10354116_C_T	1321R>K	Substitution	Nonsynonymous coding	17%
MM12T	MYH4	myosin, heavy chain 4, skeletal muscle	CCDS11154.1	chr17_10368893-10368893_A_T	124V>D	Substitution	Nonsynonymous coding	28%
MM12T	MYH6	myosin, heavy chain 6, cardiac muscle, alpha	CCDS9600.1	chr14_23852502-23852502_G_A	1865R>W	Substitution	Nonsynonymous coding	31%
MM12T	MYH6	myosin, heavy chain 6, cardiac muscle, alpha	CCDS9600.1	chr14_23858909-23858909_G_A	1251S>F	Substitution	Nonsynonymous coding	10%
MM12T	MYH6	myosin, heavy chain 6, cardiac muscle, alpha	CCDS9600.1	chr14_23866769-23866769_C_T	649V>M	Substitution	Nonsynonymous coding	11%
MM12T	MYH6	myosin, heavy chain 6, cardiac muscle, alpha	CCDS9600.1	chr14_23874479-23874479_G_A	152P>L	Substitution	Nonsynonymous coding	36%
MM12T	MYH7B	myosin, heavy chain 7B, cardiac muscle, beta	CCDS42869.1	chr20_33588167-33588167_C_T	1660A>V	Substitution	Nonsynonymous coding	10%
MM12T	MYH8	myosin, heavy chain 8, skeletal muscle, perinatal	CCDS11153.1	chr17_10293864-10293864_C_A	1907E>D	Substitution	Nonsynonymous coding	17%

MM12T	MYH8	myosin, heavy chain 8, skeletal muscle, perinatal	CCDS11153.1	chr17_10304100-10304100_G_A	ISV-4>	Substitution	Splice site acceptor	28%
MM12T	MYH9	myosin, heavy chain 9, non-muscle	CCDS13927.1	chr22_36684456-36684456_G_A	1592R>W	Substitution	Nonsynonymous coding	12%
MM12T	MYH9	myosin, heavy chain 9, non-muscle	CCDS13927.1	chr22_36696246-36696246_G_C	968T>S	Substitution	Nonsynonymous coding	10%
MM12T	MYH9	myosin, heavy chain 9, non-muscle	CCDS13927.1	chr22_36694965-36694965_C_A	1034E>X	Substitution	Nonsense	18%
MM12T	MYLK	myosin light chain kinase	CCDS46896.1	chr3_123385149-123385149_G_A	1250R>C	Substitution	Nonsynonymous coding	29%
MM12T	MYO10	myosin X	NM_012334	chr5_16680187-16680187_G_A	1471R>C	Substitution	Nonsynonymous coding	12%
MM12T	MYO15A	myosin XVA	CCDS42271.1	chr17_18045430-18045430_G_T	1896S>I	Substitution	Nonsynonymous coding	38%
MM12T	MYO15A	myosin XVA	CCDS42271.1	chr17_18053762-18053762_C_T	2411A>V	Substitution	Nonsynonymous coding	34%
MM12T	MYO15B	myosin XVB pseudogene	ENST00000293201	chr17_73612658-73612658_C_T	1040A>V	Substitution	Nonsynonymous coding	33%
MM12T	MYO16	myosin XVI	CCDS32008.1	chr13_109438038-109438038_C_A	166A>D	Substitution	Nonsynonymous coding	36%
MM12T	MYO18A	myosin XVIIIA	CCDS45642.1	chr17_27448135-27448135_C_T	489R>H	Substitution	Nonsynonymous coding	21%
MM12T	MYO18A	myosin XVIIIA	CCDS45642.1	chr17_27425843-27425843_C_T	ISV+1>	Substitution	Splice site donor	18%
MM12T	MYO18B	myosin XVIIIB	NM_032608	chr22_26164082-26164082_A_G	67I>V	Substitution	Nonsynonymous coding	27%
MM12T	MYO18B	myosin XVIIIB	NM_032608	chr22_26165324-26165324_C_T	481R>W	Substitution	Nonsynonymous coding	26%
MM12T	MYO1B	myosin IB	CCDS46477.1	chr2_192160877-192160877_G_A	59R>Q	Substitution	Nonsynonymous coding	12%
MM12T	MYO1B	myosin IB	CCDS46477.1	chr2_192206238-192206238_C_T	133A>V	Substitution	Nonsynonymous coding	15%
MM12T	MYO1C	myosin IC	CCDS42226.1	chr17_1373566-1373566_T_C	810D>G	Substitution	Nonsynonymous coding	15%
MM12T	MYO1D	myosin ID	CCDS32615.1	chr17_31039035-31039035_G_T	698L>I	Substitution	Nonsynonymous coding	49%
MM12T	MYO3A	myosin IIIA	CCDS7148.1	chr10_26432398-26432398_G_A	762V>M	Substitution	Nonsynonymous coding	26%
MM12T	MYO3B	myosin IIIB	CCDS42773.1	chr2_171323173-171323173_G_A	989R>H	Substitution	Nonsynonymous coding	28%
MM12T	MYO5A	myosin VA (heavy chain 12, myoxin)	CCDS42037.1	chr15_52720685-52720685_G_A	74L>F	Substitution	Nonsynonymous coding	27%
MM12T	MYO5B	myosin VB	CCDS42436.1	chr18_47364169-47364169_G_A	1619S>F	Substitution	Nonsynonymous coding	50%
MM12T	MYO5B	myosin VB	CCDS42436.1	chr18_47367799-47367799_G_A	1546T>M	Substitution	Nonsynonymous coding	25%
MM12T	MYO5B	myosin VB	CCDS42436.1	chr18_47402163-47402163_G_A	1144T>M	Substitution	Nonsynonymous coding	31%
MM12T	MYO5B	myosin VB	CCDS42436.1	chr18_47405401-47405401_G_A	1064R>X	Substitution	Nonsense	28%
MM12T	MYO6	myosin VI	CCDS34487.1	chr6_76599858-76599858__A	NA	Insertion	Frameshift	10%
MM12T	MYO6	myosin VI	CCDS34487.1	chr6_76550344-76550344_G_A	199R>H	Substitution	Nonsynonymous coding	19%
MM12T	MYO6	myosin VI	CCDS34487.1	chr6_76570770-76570770_G_T	502G>C	Substitution	Nonsynonymous coding	24%
MM12T	MYO7A	myosin VIIA	NM_000260	chr11_76890850-76890850_G_A	813A>T	Substitution	Nonsynonymous coding	16%
MM12T	MYO7A	myosin VIIA	NM_000260	chr11_76909653-76909653_G_A	1519V>M	Substitution	Nonsynonymous coding	50%
MM12T	MYO7B	myosin VIIB	CCDS46405.1	chr2_128363489-128363489_A_G	804R>G	Substitution	Nonsynonymous coding	22%
MM12T	MYO7B	myosin VIIB	CCDS46405.1	chr2_128366343-128366343_C_T	902R>C	Substitution	Nonsynonymous coding	12%

MM12T	MYO7B	myosin VIIB	CCDS46405.1	chr2_128381878-128381878_G_A	1318E>K	Substitution	Nonsynonymous coding	25%
MM12T	MYO9A	myosin IXA	CCDS10239.1	chr15_72180441-72180441_G_A	1720S>L	Substitution	Nonsynonymous coding	32%
MM12T	MYO9B	myosin IXB	CCDS46010.1	chr19_17305733-17305733_T_C	1166I>T	Substitution	Nonsynonymous coding	42%
MM12T	MYOCD	myocardin	CCDS11163.1	chr17_12656041-12656041_C_	NA	Deletion	Frameshift	28%
MM12T	MYOCD	myocardin	CCDS11163.1	chr17_12620711-12620711_G_A	76D>N	Substitution	Nonsynonymous coding	26%
MM12T	MYOD1	myogenic differentiation 1	CCDS7826.1	chr11_17742823-17742823_C_T	244A>V	Substitution	Nonsynonymous coding	14%
MM12T	MYOD1	myogenic differentiation 1	CCDS7826.1	chr11_17742873-17742873_A_G	261T>A	Substitution	Nonsynonymous coding	22%
MM12T	MYOM1	myomesin 1	CCDS45824.1	chr18_3126723-3126723_C_G	989K>N	Substitution	Nonsynonymous coding	13%
MM12T	MYOM1	myomesin 1	CCDS45824.1	chr18_3129458-3129458_C_T	856A>T	Substitution	Nonsynonymous coding	33%
MM12T	MYRF	myelin regulatory factor	CCDS44622.1	chr11_61533626-61533626_C_A	111P>T	Substitution	Nonsynonymous coding	33%
MM12T	MYRF	myelin regulatory factor	CCDS44622.1	chr11_61544914-61544914_G_A	590R>H	Substitution	Nonsynonymous coding	11%
MM12T	MYRF	myelin regulatory factor	CCDS44622.1	chr11_61551072-61551072_G_T	1040R>M	Substitution	Nonsynonymous coding	32%
MM12T	MYRF	myelin regulatory factor	CCDS44622.1	chr11_61550969-61550969_G_A	ISV-1>	Substitution	Splice site acceptor	15%
MM12T	MYRFL	myelin regulatory factor-like	ENST00000299350	chr12_70329946-70329946_G_A	22R>H	Substitution	Nonsynonymous coding	36%
MM12T	MYRFL	myelin regulatory factor-like	ENST00000299350	chr12_70330257-70330257_C_T	55T>I	Substitution	Nonsynonymous coding	26%
MM12T	MYSM1	Myb-like, SWIRM and MPN domains 1	CCDS41343.1	chr1_59147985-59147985_T_C	244D>G	Substitution	Nonsynonymous coding	49%
MM12T	MYT1L	myelin transcription factor 1-like	CCDS46222.1	chr2_1926459-1926459_A_G	361V>A	Substitution	Nonsynonymous coding	12%
MM12T	N4BP2	NEDD4 binding protein 2	CCDS3457.1	chr4_40127885-40127885_A_G	1488T>A	Substitution	Nonsynonymous coding	12%
MM12T	N4BP2L2	NEDD4 binding protein 2-like 2	CCDS45024.1	chr13_33017998-33017998__T	NA	Insertion	Frameshift	15%
MM12T	NAA25	N(alpha)-acetyltransferase 25, NatB auxiliary subunit	CCDS9159.1	chr12_112480966-112480966_G_A	767S>F	Substitution	Nonsynonymous coding	12%
MM12T	NAA35	N(alpha)-acetyltransferase 35, NatC auxiliary subunit	CCDS6673.1	chr9_88632448-88632448_G_A	581A>T	Substitution	Nonsynonymous coding	13%
MM12T	NAAA	N-acylethanolamine acid amidase	CCDS43239.1	chr4_76842239-76842239_C_T	235G>D	Substitution	Nonsynonymous coding	12%
MM12T	NAAA	N-acylethanolamine acid amidase	CCDS43239.1	chr4_76861193-76861193_G_A	111A>V	Substitution	Nonsynonymous coding	23%
MM12T	NAALADL1	N-acetylated alpha-linked acidic dipeptidase-like 1	CCDS31604.1	chr11_64813726-64813726_C_T	597R>H	Substitution	Nonsynonymous coding	13%
MM12T	NABP1	nucleic acid binding protein 1	ENST00000307849	chr2_192547240-192547240_G_A	109A>T	Substitution	Nonsynonymous coding	25%
MM12T	NADSYN1	NAD synthetase 1	CCDS8201.1	chr11_71169583-71169583_G_A	86V>M	Substitution	Nonsynonymous coding	29%
MM12T	NAT16	N-acetyltransferase 16 (GCN5-related, putative)	CCDS5713.1	chr7_100815493-100815493_C_T	326G>D	Substitution	Nonsynonymous coding	12%
MM12T	NAV2	neuron navigator 2	CCDS7851.2	chr11_20066528-20066528_G_A	1072A>T	Substitution	Nonsynonymous coding	22%
MM12T	NAV2	neuron navigator 2	CCDS7851.2	chr11_20122600-20122600_G_A	2103S>N	Substitution	Nonsynonymous coding	22%
MM12T	NAV3	neuron navigator 3	CCDS41815.1	chr12_78444657-78444657_C_T	749P>L	Substitution	Nonsynonymous coding	31%
MM12T	NAV3	neuron navigator 3	CCDS41815.1	chr12_78452792-78452792_G_A	845G>R	Substitution	Nonsynonymous coding	36%
MM12T	NBAS	neuroblastoma amplified sequence	CCDS1685.1	chr2_15432713-15432713_G_A	1659R>W	Substitution	Nonsynonymous coding	24%

MM12T	NBAS	neuroblastoma amplified sequence	CCDS1685.1	chr2_15607532-15607532_C_A	ISV-1>	Substitution	Splice site acceptor	21%
MM12T	NBAS	neuroblastoma amplified sequence	CCDS1685.1	chr2_15608502-15608502_C_T	ISV+4>	Substitution	Splice site donor	11%
MM12T	NBEA	neurobeachin	CCDS45026.1	chr13_35517203-35517203_G_T	82Q>H	Substitution	Nonsynonymous coding	35%
MM12T	NBEAL1	neurobeachin-like 1	CCDS46495.1	chr2_204016238-204016238_G_A	1809R>H	Substitution	Nonsynonymous coding	17%
MM12T	NBEAL2	neurobeachin-like 2	CCDS46817.1	chr3_47041428-47041428_G_A	1280R>Q	Substitution	Nonsynonymous coding	31%
MM12T	NBEAL2	neurobeachin-like 2	CCDS46817.1	chr3_47041772-47041772_C_T	1395P>S	Substitution	Nonsynonymous coding	16%
MM12T	NBEAL2	neurobeachin-like 2	CCDS46817.1	chr3_47043417-47043417_C_T	1597A>V	Substitution	Nonsynonymous coding	28%
MM12T	NBEAL2	neurobeachin-like 2	CCDS46817.1	chr3_47049921-47049921_C_A	2623P>H	Substitution	Nonsynonymous coding	30%
MM12T	NCAM1	neural cell adhesion molecule 1	ENST00000401611	chr11_113105833-113105833_G_A	463G>D	Substitution	Nonsynonymous coding	23%
MM12T	NCAPD2	non-SMC condensin I complex, subunit D2	CCDS8548.1	chr12_6639102-6639102_G_A	1272R>H	Substitution	Nonsynonymous coding	37%
MM12T	NCAPD3	non-SMC condensin II complex, subunit D3	CCDS31723.1	chr11_134090543-134090543_C_A	48E>X	Substitution	Nonsense	29%
MM12T	NCAPH2	non-SMC condensin II complex, subunit H2	CCDS14094.2	chr22_50960636-50960636_G_A	397V>M	Substitution	Nonsynonymous coding	38%
MM12T	NCDN	neurochondrin	CCDS392.1	chr1_36030899-36030899_G_A	609A>T	Substitution	Nonsynonymous coding	29%
MM12T	NCKAP5	NCK-associated protein 5	CCDS46418.1	chr2_133540381-133540381_G_A	1335L>F	Substitution	Nonsynonymous coding	32%
MM12T	NCKAP5L	NCK-associated protein 5-like	CCDS41781.2	chr12_50185794-50185794_C_T	1278R>H	Substitution	Nonsynonymous coding	10%
MM12T	NCKIPSD	NCK interacting protein with SH3 domain	CCDS2776.1	chr3_48720448-48720448__G	NA	Insertion	Splice site acceptor	27%
MM12T	NCLN	nicalin	CCDS32869.1	chr19_3204066-3204066_G_T	318S>I	Substitution	Nonsynonymous coding	36%
MM12T	NCOA2	nuclear receptor coactivator 2	CCDS47872.1	chr8_71036314-71036314_C_T	1366M>I	Substitution	Nonsynonymous coding	24%
MM12T	NCOA2	nuclear receptor coactivator 2	CCDS47872.1	chr8_71037104-71037104_A_	NA	Deletion	Splice site acceptor	13%
MM12T	NCOA6	nuclear receptor coactivator 6	CCDS13241.1	chr20_33338326-33338326_A_	NA	Deletion	Splice site acceptor	11%
MM12T	NCOA6	nuclear receptor coactivator 6	CCDS13241.1	chr20_33338326-33338326__A	NA	Insertion	Splice site acceptor	13%
MM12T	NCOA7	nuclear receptor coactivator 7	CCDS5132.1	chr6_126210338-126210338_A_G	380T>A	Substitution	Nonsynonymous coding	46%
MM12T	NCOA7	nuclear receptor coactivator 7	CCDS5132.1	chr6_126210339-126210339_C_T	380T>I	Substitution	Nonsynonymous coding	17%
MM12T	NCOR1	nuclear receptor corepressor 1	CCDS11175.1	chr17_15965542-15965542_C_T	1755R>H	Substitution	Nonsynonymous coding	16%
MM12T	NCOR1	nuclear receptor corepressor 1	CCDS11175.1	chr17_16004572-16004572_C_A	894E>D	Substitution	Nonsynonymous coding	31%
MM12T	NCOR1	nuclear receptor corepressor 1	CCDS11175.1	chr17_16024565-16024565_C_A	551K>N	Substitution	Nonsynonymous coding	40%
MM12T	NCOR1	nuclear receptor corepressor 1	CCDS11175.1	chr17_16021201-16021201_C_T	ISV+1>	Substitution	Splice site donor	13%
MM12T	NCOR2	nuclear receptor corepressor 2	CCDS41858.1	chr12_124846684-124846684_C_T	1029A>T	Substitution	Nonsynonymous coding	13%
MM12T	NCSTN	nicastrin	CCDS1203.1	chr1_160322989-160322989_G_T	381D>Y	Substitution	Nonsynonymous coding	25%
MM12T	NDFIP2	Nedd4 family interacting protein 2	CCDS31998.1	chr13_80122529-80122529_G_T	303G>C	Substitution	Nonsynonymous coding	29%
MM12T	NDRG1	N-myc downstream regulated 1	CCDS34945.1	chr8_134296540-134296540_C_T	5M>I	Substitution	Nonsynonymous coding	27%
MM12T	NDRG3	NDRG family member 3	CCDS13285.1	chr20_35293702-35293702_C_T	232R>H	Substitution	Nonsynonymous coding	33%

MM12T	NDUFA10	NADH dehydrogenase (ubiquinone) 1 alpha subcomplex, 10, 42kDa	CCDS2531.1	chr2_240960685-240960685_C_T	130R>H	Substitution	Nonsynonymous coding	11%
MM12T	NDUFAF1	NADH dehydrogenase (ubiquinone) complex I, assembly factor 1	CCDS10075.1	chr15_41679647-41679647_T_C	327K>E	Substitution	Nonsynonymous coding	43%
MM12T	NDUFS7	NADH dehydrogenase (ubiquinone) Fe-S protein 7, 20kDa (NADH-coenzyme Q	ENST00000382445	chr19_1389545-1389545_C_T	279A>V	Substitution	Nonsynonymous coding	24%
MM12T	NEB	nebulin	CCDS46424.1	chr2_152350311-152350311_C_T	6361R>H	Substitution	Nonsynonymous coding	19%
MM12T	NEB	nebulin	CCDS46424.1	chr2_152374846-152374846_C_T	5895E>K	Substitution	Nonsynonymous coding	11%
MM12T	NEB	nebulin	CCDS46424.1	chr2_152382501-152382501_C_T	5677V>I	Substitution	Nonsynonymous coding	10%
MM12T	NEB	nebulin	CCDS46424.1	chr2_152408350-152408350_G_A	4915H>Y	Substitution	Nonsynonymous coding	31%
MM12T	NEB	nebulin	CCDS46424.1	chr2_152424934-152424934_A_	NA	Deletion	Splice site acceptor	34%
MM12T	NEB	nebulin	CCDS46424.1	chr2_152402516-152402516__A	NA	Insertion	Splice site acceptor	13%
MM12T	NEDD4L	neural precursor cell expressed, developmentally down-regulated 4-like, E3	CCDS45872.1	chr18_56035063-56035063_G_A	717A>T	Substitution	Nonsynonymous coding	28%
MM12T	NEDD8	neural precursor cell expressed, developmentally down-regulated 8	CCDS9621.1	chr14_24701471-24701471_C_T	1M>I	Substitution	Nonsynonymous coding	32%
MM12T	NEFL	neurofilament, light polypeptide	ENST00000221169	chr8_24813348-24813348_C_T	228E>K	Substitution	Nonsynonymous coding	34%
MM12T	NEFM	neurofilament, medium polypeptide	CCDS6046.1	chr8_24771602-24771602_C_T	99S>F	Substitution	Nonsynonymous coding	33%
MM12T	NEIL2	nei endonuclease VIII-like 2 (E. coli)	CCDS5984.1	chr8_11637332-11637332_G_A	122A>T	Substitution	Nonsynonymous coding	16%
MM12T	NEK1	NIMA-related kinase 1	CCDS47162.1	chr4_170429410-170429410_G_A	580R>C	Substitution	Nonsynonymous coding	19%
MM12T	NEK2	NIMA-related kinase 2	CCDS1500.1	chr1_211843735-211843735_G_T	218A>D	Substitution	Nonsynonymous coding	33%
MM12T	NELL1	NEL-like 1 (chicken)	CCDS7855.1	chr11_20950009-20950009_C_A	327C>X	Substitution	Nonsense	22%
MM12T	NEO1	neogenin 1	CCDS10247.1	chr15_73581554-73581554_G_A	1239M>I	Substitution	Nonsynonymous coding	27%
MM12T	NES	nestin	CCDS1151.1	chr1_156646294-156646294_G_A	255R>W	Substitution	Nonsynonymous coding	13%
MM12T	NETO2	neuropilin (NRP) and tolloid (TLL)-like 2	CCDS10727.1	chr16_47117434-47117434_G_A	426R>W	Substitution	Nonsynonymous coding	14%
MM12T	NEURL	neuralized homolog (Drosophila)	CCDS7551.1	chr10_105344640-105344640_C_T	333R>C	Substitution	Nonsynonymous coding	16%
MM12T	NEURL4	neuralized homolog 4 (Drosophila)	CCDS42251.1	chr17_7226290-7226290_C_T	857C>Y	Substitution	Nonsynonymous coding	35%
MM12T	NF1	neurofibromin 1	CCDS42292.1	chr17_29508792-29508792_C_T	240T>I	Substitution	Nonsynonymous coding	17%
MM12T	NF1	neurofibromin 1	CCDS42292.1	chr17_29661966-29661966_C_A	1975L>I	Substitution	Nonsynonymous coding	34%
MM12T	NF1	neurofibromin 1	CCDS42292.1	chr17_29533315-29533315_C_T	440R>X	Substitution	Nonsense	35%
MM12T	NF1	neurofibromin 1	CCDS42292.1	chr17_29533378-29533378_C_T	461R>X	Substitution	Nonsense	23%
MM12T	NFAT5	nuclear factor of activated T-cells 5, tonicity-responsive	CCDS45518.1	chr16_69729182-69729182_G_A	1520A>T	Substitution	Nonsynonymous coding	14%
MM12T	NFATC1	nuclear factor of activated T-cells, cytoplasmic, calcineurin-dependent 1	CCDS32850.1	chr18_77246904-77246904_C_T	904P>S	Substitution	Nonsynonymous coding	10%
MM12T	NFATC4	nuclear factor of activated T-cells, cytoplasmic, calcineurin-dependent 4	CCDS45089.1	chr14_24839029-24839029_C_T	205S>F	Substitution	Nonsynonymous coding	12%
MM12T	NFIL3	nuclear factor, interleukin 3 regulated	CCDS6690.1	chr9_94172221-94172221_G_A	266R>X	Substitution	Nonsense	36%
MM12T	NFKB1	nuclear factor of kappa light polypeptide gene enhancer in B-cells 1	CCDS3657.1	chr4_103488171-103488171_G_A	96V>I	Substitution	Nonsynonymous coding	25%
MM12T	NFKBIE	nuclear factor of kappa light polypeptide gene enhancer in B-cells inhibitor, epsilon	CCDS34463.1	chr6_44227847-44227847_C_T	457C>Y	Substitution	Nonsynonymous coding	17%

MM12T	NFKBIE	nuclear factor of kappa light polypeptide gene enhancer in B-cells inhibitor, epsilon	CCDS34463.1	chr6_44229509-44229509_G_C	321A>G	Substitution	Nonsynonymous coding	41%
MM12T	NFKBIZ	nuclear factor of kappa light polypeptide gene enhancer in B-cells inhibitor, zeta	CCDS2946.1	chr3_101570979-101570979_G_A	114V>I	Substitution	Nonsynonymous coding	32%
MM12T	NFX1	nuclear transcription factor, X-box binding 1	CCDS6538.1	chr9_33342751-33342751_A_G	708E>G	Substitution	Nonsynonymous coding	24%
MM12T	NGEF	neuronal guanine nucleotide exchange factor	CCDS2500.1	chr2_233756106-233756106_G_A	412P>S	Substitution	Nonsynonymous coding	40%
MM12T	NGFR	nerve growth factor receptor	CCDS11549.1	chr17_47583813-47583813_C_T	121R>C	Substitution	Nonsynonymous coding	23%
MM12T	NGLY1	N-glycanase 1	CCDS33719.1	chr3_25778830-25778830_T_C	333Y>C	Substitution	Nonsynonymous coding	33%
MM12T	NHS	Nance-Horan syndrome (congenital cataracts and dental anomalies)	CCDS14181.1	chrX_17744495-17744495_G_A	736A>T	Substitution	Nonsynonymous coding	31%
MM12T	NHS	Nance-Horan syndrome (congenital cataracts and dental anomalies)	CCDS14181.1	chrX_17744995-17744995_G_T	902K>N	Substitution	Nonsynonymous coding	33%
MM12T	NIN	ninein (GSK3B interacting protein)	CCDS41952.1	chr14_51190213-51190213_G_A	2124P>S	Substitution	Nonsynonymous coding	31%
MM12T	NINL	ninein-like	CCDS33452.1	chr20_25472100-25472100_G_A	458R>X	Substitution	Nonsense	33%
MM12T	NIPAL3	NIPA-like domain containing 3	ENST00000437117	chr1_24784307-24784307_C_A	64S>Y	Substitution	Nonsynonymous coding	39%
MM12T	NIPBL	Nipped-B homolog (Drosophila)	CCDS3920.1	chr5_37064899-37064899_A_	NA	Deletion	Frameshift	24%
MM12T	NIPSNAP3B	nipsnap homolog 3B (C. elegans)	CCDS6761.1	chr9_107531205-107531205_A_C	111E>D	Substitution	Nonsynonymous coding	10%
MM12T	NKAP	NFKB activating protein	CCDS14592.1	chrX_119059350-119059350_G_A	361R>X	Substitution	Nonsense	29%
MM12T	NKIRAS2	NFKB inhibitor interacting Ras-like 2	CCDS11415.1	chr17_40175859-40175859_C_A	175S>Y	Substitution	Nonsynonymous coding	25%
MM12T	NKPD1	NTPase, KAP family P-loop domain containing 1	CCDS42577.1	chr19_45656251-45656251_C_T	260D>N	Substitution	Nonsynonymous coding	13%
MM12T	NKPD1	NTPase, KAP family P-loop domain containing 1	CCDS42577.1	chr19_45656781-45656781_C_T	83R>H	Substitution	Nonsynonymous coding	13%
MM12T	NKPD1	NTPase, KAP family P-loop domain containing 1	CCDS42577.1	chr19_45656883-45656883_C_T	49R>H	Substitution	Nonsynonymous coding	21%
MM12T	NKRF	NFKB repressing factor	CCDS35375.1	chrX_118723587-118723587_T_A	601I>F	Substitution	Nonsynonymous coding	27%
MM12T	NKRF	NFKB repressing factor	CCDS35375.1	chrX_118724850-118724850_T_C	180T>A	Substitution	Nonsynonymous coding	44%
MM12T	NKRF	NFKB repressing factor	CCDS35375.1	chrX_118725105-118725105_C_A	95D>Y	Substitution	Nonsynonymous coding	33%
MM12T	NLGN2	neuroligin 2	CCDS11103.1	chr17_7312019-7312019_C_T	149P>S	Substitution	Nonsynonymous coding	24%
MM12T	NLRC4	NLR family, CARD domain containing 4	CCDS33174.1	chr2_32475581-32475581_G_A	451A>V	Substitution	Nonsynonymous coding	21%
MM12T	NLRP14	NLR family, pyrin domain containing 14	CCDS7776.1	chr11_7064627-7064627_T_C	457V>A	Substitution	Nonsynonymous coding	36%
MM12T	NLRP3	NLR family, pyrin domain containing 3	CCDS1632.1	chr1_247588376-247588376_C_T	544T>M	Substitution	Nonsynonymous coding	27%
MM12T	NLRP8	NLR family, pyrin domain containing 8	CCDS12937.1	chr19_56459475-56459475_G_A	69M>I	Substitution	Nonsynonymous coding	11%
MM12T	NLRX1	NLR family member X1	CCDS8416.1	chr11_119050459-119050459_G_A	577A>T	Substitution	Nonsynonymous coding	15%
MM12T	NLRX1	NLR family member X1	CCDS8416.1	chr11_119050985-119050985_C_T	752A>V	Substitution	Nonsynonymous coding	30%
MM12T	NLRX1	NLR family member X1	CCDS8416.1	chr11_119052946-119052946_G_A	833R>H	Substitution	Nonsynonymous coding	15%
MM12T	NM_001013690	-	NM_001013690	chr12_52215276-52215276_C_T	308G>R	Substitution	Nonsynonymous coding	31%
MM12T	NM_001102659	-	NM_001102659	chr2_207509151-207509151_G_A	64R>H	Substitution	Nonsynonymous coding	22%
MM12T	NM_001145073	-	NM_001145073	chrX_49645473-49645473_C_T	188A>V	Substitution	Nonsynonymous coding	32%

MM12T	NM_002248	-	NM_002248	chr19_18104364-18104364_C_T	458A>V	Substitution	Nonsynonymous coding	27%
MM12T	NM_213655	-	NM_213655	chr12_977864-977864_G_A	290R>Q	Substitution	Nonsynonymous coding	35%
MM12T	NMU	neuromedin U	CCDS3501.1	chr4_56471464-56471464_C_A	138R>I	Substitution	Nonsynonymous coding	13%
MM12T	NMUR1	neuromedin U receptor 1	CCDS2486.1	chr2_232393572-232393572_G_A	54P>S	Substitution	Nonsynonymous coding	26%
MM12T	NNT	nicotinamide nucleotide transhydrogenase	CCDS3949.1	chr5_43613209-43613209_G_T	117K>N	Substitution	Nonsynonymous coding	39%
MM12T	NNT	nicotinamide nucleotide transhydrogenase	CCDS3949.1	chr5_43675623-43675623_G_A	882R>H	Substitution	Nonsynonymous coding	13%
MM12T	NOB1	NIN1/RPN12 binding protein 1 homolog (S. cerevisiae)	CCDS10884.1	chr16_69788607-69788607_G_A	29T>I	Substitution	Nonsynonymous coding	16%
MM12T	NOC4L	nucleolar complex associated 4 homolog (S. cerevisiae)	CCDS9277.1	chr12_132636066-132636066_C_T	371R>W	Substitution	Nonsynonymous coding	10%
MM12T	NOD2	nucleotide-binding oligomerization domain containing 2	CCDS10746.1	chr16_50745497-50745497_G_T	559G>C	Substitution	Nonsynonymous coding	31%
MM12T	NOL11	nucleolar protein 11	CCDS11671.1	chr17_65735053-65735053_C_T	590A>V	Substitution	Nonsynonymous coding	42%
MM12T	NOL11	nucleolar protein 11	CCDS11671.1	chr17_65739626-65739626_C_T	656A>V	Substitution	Nonsynonymous coding	31%
MM12T	NOL3	nucleolar protein 3 (apoptosis repressor with CARD domain)	ENST00000445652	chr16_67208977-67208977_C_T	213R>W	Substitution	Nonsynonymous coding	31%
MM12T	NOL8	nucleolar protein 8	CCDS47993.1	chr9_95077784-95077784_C_T	375E>K	Substitution	Nonsynonymous coding	24%
MM12T	NOM1	nucleolar protein with MIF4G domain 1	CCDS34787.1	chr7_156759790-156759790_C_T	ISV+4>	Substitution	Splice site donor	30%
MM12T	NOP16	NOP16 nucleolar protein	CCDS43403.1	chr5_175812256-175812256_C_T	120R>H	Substitution	Nonsynonymous coding	37%
MM12T	NOP56	NOP56 ribonucleoprotein	CCDS13030.1	chr20_2637737-2637737_C_T	431A>V	Substitution	Nonsynonymous coding	13%
MM12T	NOS1	nitric oxide synthase 1 (neuronal)	CCDS41842.1	chr12_117768300-117768300_G_T	192T>N	Substitution	Nonsynonymous coding	32%
MM12T	NOS2	nitric oxide synthase 2, inducible	CCDS11223.1	chr17_26100217-26100217_C_A	510R>I	Substitution	Nonsynonymous coding	23%
MM12T	NOTCH1	notch 1	CCDS43905.1	chr9_139413115-139413115_C_T	343A>T	Substitution	Nonsynonymous coding	36%
MM12T	NOTCH1	notch 1	CCDS43905.1	chr9_139417601-139417601_G_A	148P>L	Substitution	Nonsynonymous coding	33%
MM12T	NOTCH1	notch 1	CCDS43905.1	chr9_139391011-139391011_G_A	2394Q>X	Substitution	Nonsense	28%
MM12T	NOTCH3	notch 3	CCDS12326.1	chr19_15291040-15291040_G_A	1057A>V	Substitution	Nonsynonymous coding	15%
MM12T	NOTCH4	notch 4	CCDS34420.1	chr6_32188854-32188854_G_A	234R>W	Substitution	Nonsynonymous coding	10%
MM12T	NOX1	NADPH oxidase 1	ENST00000372961	chrX_100098360-100098360_A_C	185F>V	Substitution	Nonsynonymous coding	16%
MM12T	NOX1	NADPH oxidase 1	ENST00000372961	chrX_100098361-100098361_T_A	184L>F	Substitution	Nonsynonymous coding	15%
MM12T	NOX5	NADPH oxidase, EF-hand calcium binding domain 5	CCDS32276.1	chr15_69329449-69329449_C_A	406L>M	Substitution	Nonsynonymous coding	22%
MM12T	NOXA1	NADPH oxidase activator 1	CCDS7042.1	chr9_140328429-140328429_C_T	398R>W	Substitution	Nonsynonymous coding	25%
MM12T	NPAS2	neuronal PAS domain protein 2	ENST00000427413	chr2_101437597-101437597_G_T	37Q>H	Substitution	Nonsynonymous coding	27%
MM12T	NPAS3	neuronal PAS domain protein 3	CCDS9645.1	chr14_34269029-34269029_G_A	474A>T	Substitution	Nonsynonymous coding	11%
MM12T	NPAS3	neuronal PAS domain protein 3	CCDS9645.1	chr14_34269138-34269138_C_T	510A>V	Substitution	Nonsynonymous coding	29%
MM12T	NPAT	nuclear protein, ataxia-telangiectasia locus	CCDS41710.1	chr11_108043331-108043331_C_T	794E>K	Substitution	Nonsynonymous coding	17%
MM12T	NPAT	nuclear protein, ataxia-telangiectasia locus	CCDS41710.1	chr11_108093238-108093238_C_T	9R>Q	Substitution	Nonsynonymous coding	38%

MM12T	NPBWR2	neuropeptides B/W receptor 2	CCDS13557.1	chr20_62737419-62737419_G_A	256R>W	Substitution	Nonsynonymous coding	12%
MM12T	NPFFR1	neuropeptide FF receptor 1	ENST00000277942	chr10_72015006-72015006_C_T	334E>K	Substitution	Nonsynonymous coding	38%
MM12T	NPFFR1	neuropeptide FF receptor 1	ENST00000277942	chr10_72015179-72015179_G_A	276A>V	Substitution	Nonsynonymous coding	12%
MM12T	NPFFR2	neuropeptide FF receptor 2	CCDS3551.1	chr4_72897770-72897770_G_T	51S>I	Substitution	Nonsynonymous coding	15%
MM12T	NPHP4	nephronophthisis 4	CCDS44052.1	chr1_5925190-5925190_G_A	1263A>V	Substitution	Nonsynonymous coding	38%
MM12T	NPHP4	nephronophthisis 4	CCDS44052.1	chr1_6007168-6007168_C_T	372G>D	Substitution	Nonsynonymous coding	10%
MM12T	NPHS1	nephrosis 1, congenital, Finnish type (nephrin)	CCDS32996.1	chr19_36335278-36335278_C_T	672A>T	Substitution	Nonsynonymous coding	35%
MM12T	NPL	N-acetylneuraminate pyruvate lyase (dihydrodipicolinate synthase)	CCDS1350.1	chr1_182787787-182787787_G_A	190R>H	Substitution	Nonsynonymous coding	36%
MM12T	NPNT	nephronectin	CCDS34046.1	chr4_106858208-106858208_G_A	103R>K	Substitution	Nonsynonymous coding	21%
MM12T	NPRL3	nitrogen permease regulator-like 3 (S. cerevisiae)	ENST00000399953	chr16_180531-180531_C_T	60G>S	Substitution	Nonsynonymous coding	39%
MM12T	NPTX1	neuronal pentraxin I	CCDS32762.1	chr17_78450197-78450197_A_G	17L>P	Substitution	Nonsynonymous coding	38%
MM12T	NPY	neuropeptide Y	CCDS5387.1	chr7_24324942-24324942_C_T	28A>V	Substitution	Nonsynonymous coding	12%
MM12T	NPY1R	neuropeptide Y receptor Y1	CCDS34089.1	chr4_164247198-164247198_G_A	170S>F	Substitution	Nonsynonymous coding	11%
MM12T	NPY2R	neuropeptide Y receptor Y2	CCDS3791.1	chr4_156136152-156136152_C_T	354S>F	Substitution	Nonsynonymous coding	12%
MM12T	NR0B1	nuclear receptor subfamily 0, group B, member 1	CCDS14223.1	chrX_30322856-30322856_G_A	418T>M	Substitution	Nonsynonymous coding	27%
MM12T	NR0B1	nuclear receptor subfamily 0, group B, member 1	CCDS14223.1	chrX_30327348-30327348_C_T	45D>N	Substitution	Nonsynonymous coding	14%
MM12T	NR1D1	nuclear receptor subfamily 1, group D, member 1	CCDS11361.1	chr17_38251869-38251869_C_T	359R>H	Substitution	Nonsynonymous coding	22%
MM12T	NR2F1	nuclear receptor subfamily 2, group F, member 1	CCDS4068.1	chr5_92929447-92929447_G_A	391V>I	Substitution	Nonsynonymous coding	34%
MM12T	NR2F2	nuclear receptor subfamily 2, group F, member 2	CCDS10375.1	chr15_96877812-96877812_C_T	317A>V	Substitution	Nonsynonymous coding	37%
MM12T	NR3C1	nuclear receptor subfamily 3, group C, member 1 (glucocorticoid receptor)	CCDS34258.1	chr5_142779249-142779249_G_A	386R>X	Substitution	Nonsense	14%
MM12T	NRDE2	NRDE-2, necessary for RNA interference, domain containing	CCDS9890.1	chr14_90778852-90778852_C_T	148R>H	Substitution	Nonsynonymous coding	13%
MM12T	NRG1	neuregulin 1	CCDS6083.1	chr8_32616830-32616830_G_A	318V>I	Substitution	Nonsynonymous coding	12%
MM12T	NRG3	neuregulin 3	CCDS31233.1	chr10_84745345-84745345_C_T	692S>F	Substitution	Nonsynonymous coding	13%
MM12T	NRK	Nik related kinase	NM_198465	chrX_105153391-105153391_G_T	586Q>H	Substitution	Nonsynonymous coding	11%
MM12T	NRXN2	neurexin 2	CCDS8077.1	chr11_64435006-64435006_G_A	505A>V	Substitution	Nonsynonymous coding	27%
MM12T	NRXN3	neurexin 3	CCDS9870.1	chr14_80164060-80164060_G_A	862G>E	Substitution	Nonsynonymous coding	11%
MM12T	NSD1	nuclear receptor binding SET domain protein 1	CCDS4412.1	chr5_176637231-176637231_C_T	611R>X	Substitution	Nonsense	26%
MM12T	NSDHL	NAD(P) dependent steroid dehydrogenase-like	CCDS14717.1	chrX_152031162-152031162_C_T	146A>V	Substitution	Nonsynonymous coding	21%
MM12T	NSMF	NMDA receptor synaptonuclear signaling and neuronal migration factor	CCDS48069.1	chr9_140347223-140347223_G_A	366R>C	Substitution	Nonsynonymous coding	34%
MM12T	NSUN2	NOP2/Sun RNA methyltransferase family, member 2	CCDS3869.1	chr5_6604332-6604332_C_A	626D>Y	Substitution	Nonsynonymous coding	14%
MM12T	NSUN2	NOP2/Sun RNA methyltransferase family, member 2	CCDS3869.1	chr5_6604788-6604788_G_A	583T>M	Substitution	Nonsynonymous coding	13%
MM12T	NT5C1A	5'-nucleotidase, cytosolic IA	CCDS440.1	chr1_40131301-40131301_G_A	109R>W	Substitution	Nonsynonymous coding	61%

MM12T	NT5C1B	5'-nucleotidase, cytosolic IB	CCDS33150.1	chr2_18768783-18768783_G_A	36R>C	Substitution	Nonsynonymous coding	14%
MM12T	NT5DC2	5'-nucleotidase domain containing 2	CCDS46843.1	chr3_52562687-52562687_C_T	172A>T	Substitution	Nonsynonymous coding	25%
MM12T	NT5M	5',3'-nucleotidase, mitochondrial	CCDS32581.1	chr17_17207015-17207015_G_A	51G>S	Substitution	Nonsynonymous coding	31%
MM12T	NTHL1	nth endonuclease III-like 1 (E. coli)	CCDS10457.1	chr16_2093639-2093639_G_A	213A>V	Substitution	Nonsynonymous coding	10%
MM12T	NTNG1	netrin G1	CCDS44180.1	chr1_107867537-107867537_C_T	294R>X	Substitution	Nonsense	33%
MM12T	NTNG2	netrin G2	CCDS6946.1	chr9_135073993-135073993_G_A	285G>D	Substitution	Nonsynonymous coding	11%
MM12T	NTRK3	neurotrophic tyrosine kinase, receptor, type 3	CCDS32323.1	chr15_88522589-88522589_C_T	609R>H	Substitution	Nonsynonymous coding	33%
MM12T	NUCB2	nucleobindin 2	CCDS41623.1	chr11_17316998-17316998_C_T	43A>V	Substitution	Nonsynonymous coding	38%
MM12T	NUDT10	nudix (nucleoside diphosphate linked moiety X)-type motif 10	CCDS35278.1	chrX_51076278-51076278_C_T	154S>F	Substitution	Nonsynonymous coding	43%
MM12T	NUDT14	nudix (nucleoside diphosphate linked moiety X)-type motif 14	CCDS10000.1	chr14_105644038-105644038_G_A	39T>M	Substitution	Nonsynonymous coding	10%
MM12T	NUDT18	nudix (nucleoside diphosphate linked moiety X)-type motif 18	ENST00000309188	chr8_21966701-21966701_C_T	ISV-1>	Substitution	Splice site acceptor	29%
MM12T	NUDT22	nudix (nucleoside diphosphate linked moiety X)-type motif 22	CCDS8061.1	chr11_63994252-63994252_C_T	43A>V	Substitution	Nonsynonymous coding	10%
MM12T	NUDT9	nudix (nucleoside diphosphate linked moiety X)-type motif 9	CCDS3620.1	chr4_88378991-88378991_C_A	ISV-4>	Substitution	Splice site acceptor	16%
MM12T	NUFIP2	nuclear fragile X mental retardation protein interacting protein 2	CCDS32600.1	chr17_27613076-27613076_C_A	646G>C	Substitution	Nonsynonymous coding	36%
MM12T	NUMA1	nuclear mitotic apparatus protein 1	CCDS31633.1	chr11_71718312-71718312_C_T	1796V>I	Substitution	Nonsynonymous coding	28%
MM12T	NUMA1	nuclear mitotic apparatus protein 1	CCDS31633.1	chr11_71725413-71725413_C_T	1046A>T	Substitution	Nonsynonymous coding	11%
MM12T	NUP107	nucleoporin 107kDa	CCDS8985.1	chr12_69084446-69084446_G_T	75D>Y	Substitution	Nonsynonymous coding	12%
MM12T	NUP155	nucleoporin 155kDa	CCDS3921.1	chr5_37310731-37310731_C_T	851A>T	Substitution	Nonsynonymous coding	40%
MM12T	NUP155	nucleoporin 155kDa	CCDS3921.1	chr5_37352911-37352911_G_A	162R>X	Substitution	Nonsense	22%
MM12T	NUP210L	nucleoporin 210kDa-like	CCDS41399.1	chr1_154112383-154112383__TA	NA	Insertion	Frameshift	18%
MM12T	NUP210L	nucleoporin 210kDa-like	CCDS41399.1	chr1_154098893-154098893_T_C	411Q>R	Substitution	Nonsynonymous coding	31%
MM12T	NUP35	nucleoporin 35kDa	CCDS2290.1	chr2_183995269-183995269_G_A	112R>K	Substitution	Nonsynonymous coding	12%
MM12T	NUP43	nucleoporin 43kDa	CCDS5218.1	chr6_150063531-150063531_G_A	166T>I	Substitution	Nonsynonymous coding	58%
MM12T	NUP62	nucleoporin 62kDa	CCDS12788.1	chr19_50412826-50412826_G_A	80T>I	Substitution	Nonsynonymous coding	35%
MM12T	NUP98	nucleoporin 98kDa	CCDS7746.1	chr11_3797197-3797197_G_T	137S>Y	Substitution	Nonsynonymous coding	29%
MM12T	NWD1	NACHT and WD repeat domain containing 1	CCDS32945.1	chr19_16910916-16910916_G_A	1092V>I	Substitution	Nonsynonymous coding	12%
MM12T	NXN	nucleoredoxin	CCDS10998.1	chr17_708440-708440_G_A	290R>W	Substitution	Nonsynonymous coding	17%
MM12T	NXPE2	neurexophilin and PC-esterase domain family, member 2	CCDS44738.1	chr11_114550402-114550402_C_T	17A>V	Substitution	Nonsynonymous coding	21%
MM12T	NXPH2	neurexophilin 2	CCDS46421.1	chr2_139429072-139429072_G_A	72A>V	Substitution	Nonsynonymous coding	13%
MM12T	NXPH4	neurexophilin 4	CCDS8933.1	chr12_57618661-57618661_G_A	20A>T	Substitution	Nonsynonymous coding	53%
MM12T	NYNRIN	NYN domain and retroviral integrase containing	CCDS45090.1	chr14_24886261-24886261_C_T	1769T>M	Substitution	Nonsynonymous coding	29%
MM12T	NYX	nyctalopin	CCDS14256.1	chrX_41333520-41333520_G_A	272A>T	Substitution	Nonsynonymous coding	18%

MM12T	OAS1	2'-5'-oligoadenylate synthetase 1, 40/46kDa	CCDS31905.1	chr12_113355473-113355473_G_A	336D>N	Substitution	Nonsynonymous coding	14%
MM12T	OAS2	2'-5'-oligoadenylate synthetase 2, 69/71kDa	CCDS31906.1	chr12_113435362-113435362_C_T	222P>L	Substitution	Nonsynonymous coding	33%
MM12T	OAS2	2'-5'-oligoadenylate synthetase 2, 69/71kDa	CCDS31906.1	chr12_113444353-113444353_G_A	535R>Q	Substitution	Nonsynonymous coding	36%
MM12T	OBSCN	obscurin, cytoskeletal calmodulin and titin-interacting RhoGEF	NM_001098623	chr1_228404743-228404743_G_A	803A>T	Substitution	Nonsynonymous coding	32%
MM12T	OBSCN	obscurin, cytoskeletal calmodulin and titin-interacting RhoGEF	ENST00000366707	chr1_228486400-228486400_G_A	1088A>T	Substitution	Nonsynonymous coding	11%
MM12T	OBSCN	obscurin, cytoskeletal calmodulin and titin-interacting RhoGEF	ENST00000366707	chr1_228491603-228491603_G_A	1346G>R	Substitution	Nonsynonymous coding	40%
MM12T	OBSCN	obscurin, cytoskeletal calmodulin and titin-interacting RhoGEF	NM_001098623	chr1_228528934-228528934_G_A	5946V>M	Substitution	Nonsynonymous coding	34%
MM12T	OBSCN	obscurin, cytoskeletal calmodulin and titin-interacting RhoGEF	NM_001098623	chr1_228538592-228538592_G_A	6123E>K	Substitution	Nonsynonymous coding	29%
MM12T	OBSCN	obscurin, cytoskeletal calmodulin and titin-interacting RhoGEF	NM_052843	chr1_228548296-228548296_C_T	6568T>M	Substitution	Nonsynonymous coding	33%
MM12T	OBSCN	obscurin, cytoskeletal calmodulin and titin-interacting RhoGEF	NM_001098623	chr1_228504522-228504522_G_A	4466W>X	Substitution	Nonsense	31%
MM12T	ODF2	outer dense fiber of sperm tails 2	CCDS6903.1	chr9_131231610-131231610_C_T	109A>V	Substitution	Nonsynonymous coding	28%
MM12T	ODF2L	outer dense fiber of sperm tails 2-like	CCDS30763.1	chr1_86842104-86842104__A	NA	Insertion	Splice site acceptor	11%
MM12T	OGDH	oxoglutarate (alpha-ketoglutarate) dehydrogenase (lipoamide)	CCDS34627.1	chr7_44737349-44737349_G_A	776V>M	Substitution	Nonsynonymous coding	28%
MM12T	OGDHL	oxoglutarate dehydrogenase-like	CCDS7234.1	chr10_50952060-50952060_T_A	614E>V	Substitution	Nonsynonymous coding	27%
MM12T	OIT3	oncoprotein induced transcript 3	CCDS7318.1	chr10_74660162-74660162_G_A	155C>Y	Substitution	Nonsynonymous coding	32%
MM12T	OLFM1	olfactomedin 1	CCDS6986.1	chr9_138011632-138011632_G_A	338D>N	Substitution	Nonsynonymous coding	32%
MM12T	OLFM2	olfactomedin 2	CCDS12221.1	chr19_9971361-9971361_C_T	58R>Q	Substitution	Nonsynonymous coding	35%
MM12T	OLFM4	olfactomedin 4	CCDS9440.1	chr13_53624424-53624424_G_A	351V>I	Substitution	Nonsynonymous coding	30%
MM12T	OLIG2	oligodendrocyte lineage transcription factor 2	CCDS13620.1	chr21_34399786-34399786_G_A	206A>T	Substitution	Nonsynonymous coding	17%
MM12T	OPA1	optic atrophy 1 (autosomal dominant)	CCDS33917.1	chr3_193384964-193384964_C_T	942R>X	Substitution	Nonsense	12%
MM12T	OPCML	opioid binding protein/cell adhesion molecule-like	CCDS8492.1	chr11_132306087-132306087_C_T	277R>H	Substitution	Nonsynonymous coding	11%
MM12T	OPCML	opioid binding protein/cell adhesion molecule-like	CCDS8492.1	chr11_132527042-132527042_T_C	114T>A	Substitution	Nonsynonymous coding	29%
MM12T	OPLAH	5-oxoprolinase (ATP-hydrolysing)	ENST00000426825	chr8_145107739-145107739_C_T	1028R>Q	Substitution	Nonsynonymous coding	31%
MM12T	OPLAH	5-oxoprolinase (ATP-hydrolysing)	ENST00000426825	chr8_145109512-145109512_C_T	880V>I	Substitution	Nonsynonymous coding	29%
MM12T	OPRD1	opioid receptor, delta 1	CCDS329.1	chr1_29189718-29189718_G_A	348A>T	Substitution	Nonsynonymous coding	27%
MM12T	OR10A2	olfactory receptor, family 10, subfamily A, member 2	CCDS31415.1	chr11_6891137-6891137_G_A	51R>K	Substitution	Nonsynonymous coding	12%
MM12T	OR10C1	olfactory receptor, family 10, subfamily C, member 1	ENST00000383554	chr6_29385483-29385483_G_A	143A>T	Substitution	Nonsynonymous coding	21%
MM12T	OR10C1	olfactory receptor, family 10, subfamily C, member 1	CCDS34364.1	chr6_29408484-29408484_C_T	231A>V	Substitution	Nonsynonymous coding	10%
MM12T	OR10J3	olfactory receptor, family 10, subfamily J, member 3	CCDS30909.1	chr1_159284446-159284446_G_A	2P>S	Substitution	Nonsynonymous coding	16%
MM12T	OR10X1	olfactory receptor, family 10, subfamily X, member 1	CCDS30900.1	chr1_158549160-158549160_G_A	177A>V	Substitution	Nonsynonymous coding	36%
MM12T	OR11L1	olfactory receptor, family 11, subfamily L, member 1	CCDS31098.1	chr1_248004904-248004904_C_T	99A>T	Substitution	Nonsynonymous coding	11%
MM12T	OR12D2	olfactory receptor, family 12, subfamily D, member 2	CCDS4659.1	chr6_29364862-29364862_G_A	129R>H	Substitution	Nonsynonymous coding	14%

MM12T	OR13D1	olfactory receptor, family 13, subfamily D, member 1	CCDS35094.1	chr9_107457103-107457103_T_G	134V>G	Substitution	Nonsynonymous coding	22%
MM12T	OR13H1	olfactory receptor, family 13, subfamily H, member 1	CCDS35396.1	chrX_130678052-130678052_C_T	2A>V	Substitution	Nonsynonymous coding	15%
MM12T	OR1B1	olfactory receptor, family 1, subfamily B, member 1	CCDS35126.1	chr9_125391117-125391117_C_T	233R>H	Substitution	Nonsynonymous coding	30%
MM12T	OR1C1	olfactory receptor, family 1, subfamily C, member 1	CCDS41481.1	chr1_247921417-247921417_G_T	98L>I	Substitution	Nonsynonymous coding	33%
MM12T	OR1G1	olfactory receptor, family 1, subfamily G, member 1	CCDS11020.1	chr17_3029963-3029963_G_T	295Q>K	Substitution	Nonsynonymous coding	12%
MM12T	OR1M1	olfactory receptor, family 1, subfamily M, member 1	CCDS32896.1	chr19_9204579-9204579_G_A	220R>H	Substitution	Nonsynonymous coding	12%
MM12T	OR2AJ1	olfactory receptor, family 2, subfamily AJ, member 1	ENST00000318244	chr1_248097830-248097830_C_T	254P>S	Substitution	Nonsynonymous coding	24%
MM12T	OR2B6	olfactory receptor, family 2, subfamily B, member 6	CCDS4642.1	chr6_27925374-27925374_C_T	119S>F	Substitution	Nonsynonymous coding	14%
MM12T	OR2D2	olfactory receptor, family 2, subfamily D, member 2	CCDS31416.1	chr11_6913368-6913368_G_A	122R>C	Substitution	Nonsynonymous coding	39%
MM12T	OR2F1	olfactory receptor, family 2, subfamily F, member 1	CCDS5887.1	chr7_143657472-143657472_C_T	137H>Y	Substitution	Nonsynonymous coding	24%
MM12T	OR2J2	olfactory receptor, family 2, subfamily J, member 2	CCDS43434.1	chr6_29141422-29141422_A_	NA	Deletion	Frameshift	17%
MM12T	OR2K2	olfactory receptor, family 2, subfamily K, member 2	CCDS6778.1	chr9_114090553-114090553_C_T	54R>H	Substitution	Nonsynonymous coding	32%
MM12T	OR2T11	olfactory receptor, family 2, subfamily T, member 11	CCDS31122.1	chr1_248789613-248789613_C_T	273A>T	Substitution	Nonsynonymous coding	13%
MM12T	OR4B1	olfactory receptor, family 4, subfamily B, member 1	CCDS31485.1	chr11_48238587-48238587_G_A	76A>T	Substitution	Nonsynonymous coding	12%
MM12T	OR4C3	olfactory receptor, family 4, subfamily C, member 3	CCDS31489.1	chr11_48347010-48347010_C_T	173A>V	Substitution	Nonsynonymous coding	11%
MM12T	OR4M2	olfactory receptor, family 4, subfamily M, member 2	CCDS32172.1	chr15_22369024-22369024_G_	NA	Deletion	Frameshift	22%
MM12T	OR4S2	olfactory receptor, family 4, subfamily S, member 2	CCDS31505.1	chr11_55418488-55418488_C_T	37L>F	Substitution	Nonsynonymous coding	19%
MM12T	OR52E8	olfactory receptor, family 52, subfamily E, member 8	CCDS31400.1	chr11_5878155-5878155_C_A	260A>S	Substitution	Nonsynonymous coding	28%
MM12T	OR52M1	olfactory receptor, family 52, subfamily M, member 1	CCDS31353.1	chr11_4566629-4566629_C_T	70A>V	Substitution	Nonsynonymous coding	11%
MM12T	OR52M1	olfactory receptor, family 52, subfamily M, member 1	CCDS31353.1	chr11_4567036-4567036_G_A	206G>S	Substitution	Nonsynonymous coding	36%
MM12T	OR52M1	olfactory receptor, family 52, subfamily M, member 1	CCDS31353.1	chr11_4567334-4567334_G_A	305S>N	Substitution	Nonsynonymous coding	32%
MM12T	OR52N1	olfactory receptor, family 52, subfamily N, member 1	CCDS31398.1	chr11_5809325-5809325_C_A	241S>I	Substitution	Nonsynonymous coding	29%
MM12T	OR52N2	olfactory receptor, family 52, subfamily N, member 2	CCDS31399.1	chr11_5842064-5842064_C_T	167R>C	Substitution	Nonsynonymous coding	13%
MM12T	OR56B4	olfactory receptor, family 56, subfamily B, member 4	CCDS31406.1	chr11_6129486-6129486_C_A	160L>M	Substitution	Nonsynonymous coding	32%
MM12T	OR5AP2	olfactory receptor, family 5, subfamily AP, member 2	CCDS31534.1	chr11_56409258-56409258_C_T	220V>I	Substitution	Nonsynonymous coding	18%
MM12T	OR5B21	olfactory receptor, family 5, subfamily B, member 21	CCDS31552.1	chr11_58275313-58275313_G_A	89A>V	Substitution	Nonsynonymous coding	12%
MM12T	OR5D13	olfactory receptor, family 5, subfamily D, member 13	CCDS31507.1	chr11_55541274-55541274_G_A	121A>T	Substitution	Nonsynonymous coding	15%
MM12T	OR5D13	olfactory receptor, family 5, subfamily D, member 13	CCDS31507.1	chr11_55541761-55541761_C_T	283A>V	Substitution	Nonsynonymous coding	33%
MM12T	OR5K4	olfactory receptor, family 5, subfamily K, member 4	CCDS33802.1	chr3_98073181-98073181_C_A	162L>I	Substitution	Nonsynonymous coding	21%
MM12T	OR5L2	olfactory receptor, family 5, subfamily L, member 2	CCDS31511.1	chr11_55595206-55595206_C_A	171S>Y	Substitution	Nonsynonymous coding	39%
MM12T	OR5M10	olfactory receptor, family 5, subfamily M, member 10	NM_001004741	chr11_56344878-56344878_A_G	107L>P	Substitution	Nonsynonymous coding	25%
MM12T	OR6A2	olfactory receptor, family 6, subfamily A, member 2	CCDS7772.1	chr11_6816101-6816101_G_T	280S>Y	Substitution	Nonsynonymous coding	11%

MM12T	OR6C75	olfactory receptor, family 6, subfamily C, member 75	CCDS31820.1	chr12_55759102-55759102_T_C	70S>P	Substitution	Nonsynonymous coding	29%
MM12T	OR6C76	olfactory receptor, family 6, subfamily C, member 76	CCDS31823.1	chr12_55820959-55820959__A	NA	Insertion	Frameshift	15%
MM12T	OR6K2	olfactory receptor, family 6, subfamily K, member 2	CCDS30902.1	chr1_158669781-158669781_C_T	221G>D	Substitution	Nonsynonymous coding	34%
MM12T	OR6M1	olfactory receptor, family 6, subfamily M, member 1	CCDS31696.1	chr11_123676786-123676786_G_T	91S>Y	Substitution	Nonsynonymous coding	32%
MM12T	OR6Q1	olfactory receptor, family 6, subfamily Q, member 1	CCDS31541.1	chr11_57798690-57798690_G_A	89G>E	Substitution	Nonsynonymous coding	23%
MM12T	OR8D1	olfactory receptor, family 8, subfamily D, member 1	CCDS31706.1	chr11_124179992-124179992_C_A	224S>I	Substitution	Nonsynonymous coding	47%
MM12T	OR8H2	olfactory receptor, family 8, subfamily H, member 2	CCDS31518.1	chr11_55872926-55872926_G_A	136M>I	Substitution	Nonsynonymous coding	12%
MM12T	ORC1	origin recognition complex, subunit 1	CCDS566.1	chr1_52838903-52838903_G_A	846R>W	Substitution	Nonsynonymous coding	30%
MM12T	ORC6	origin recognition complex, subunit 6	CCDS10722.1	chr16_46729525-46729525_A_	NA	Deletion	Frameshift	30%
MM12T	OSBPL3	oxysterol binding protein-like 3	CCDS5390.1	chr7_24901346-24901346_T_C	305N>D	Substitution	Nonsynonymous coding	26%
MM12T	OSBPL5	oxysterol binding protein-like 5	CCDS31344.1	chr11_3114884-3114884_C_T	607V>M	Substitution	Nonsynonymous coding	11%
MM12T	OSBPL6	oxysterol binding protein-like 6	CCDS2278.1	chr2_179214002-179214002_A_G	351N>D	Substitution	Nonsynonymous coding	13%
MM12T	OSBPL7	oxysterol binding protein-like 7	CCDS11515.1	chr17_45897356-45897356_G_T	61P>H	Substitution	Nonsynonymous coding	20%
MM12T	OSBPL9	oxysterol binding protein-like 9	CCDS44145.1	chr1_52246907-52246907_C_T	453A>V	Substitution	Nonsynonymous coding	15%
MM12T	OSGIN1	oxidative stress induced growth inhibitor 1	CCDS10938.1	chr16_83998725-83998725_G_A	266V>M	Substitution	Nonsynonymous coding	13%
MM12T	OSMR	oncostatin M receptor	CCDS3928.1	chr5_38884115-38884115_G_A	202S>N	Substitution	Nonsynonymous coding	39%
MM12T	OTOF	otoferlin	CCDS1725.1	chr2_26700057-26700057_G_A	836R>C	Substitution	Nonsynonymous coding	14%
MM12T	OTOG	otogelin	ENST00000399391	chr11_17627457-17627457_G_A	1323V>I	Substitution	Nonsynonymous coding	10%
MM12T	OTOG	otogelin	ENST00000399391	chr11_17662609-17662609_T_C	2733S>P	Substitution	Nonsynonymous coding	12%
MM12T	OTOP3	otopetrin 3	CCDS11709.1	chr17_72937646-72937646_C_T	78R>W	Substitution	Nonsynonymous coding	13%
MM12T	OTOP3	otopetrin 3	CCDS11709.1	chr17_72939724-72939724_C_T	237A>V	Substitution	Nonsynonymous coding	14%
MM12T	OTUB1	OTU domain, ubiquitin aldehyde binding 1	CCDS8055.1	chr11_63764867-63764867_C_T	222A>V	Substitution	Nonsynonymous coding	14%
MM12T	OTUD1	OTU domain containing 1	CCDS44366.1	chr10_23729764-23729764_G_A	460E>K	Substitution	Nonsynonymous coding	25%
MM12T	OTUD7A	OTU domain containing 7A	CCDS10026.1	chr15_31775580-31775580_G_A	900R>C	Substitution	Nonsynonymous coding	15%
MM12T	OTUD7A	OTU domain containing 7A	CCDS10026.1	chr15_31775735-31775735_G_A	848S>L	Substitution	Nonsynonymous coding	38%
MM12T	OTUD7A	OTU domain containing 7A	CCDS10026.1	chr15_31776405-31776405_G_T	625L>M	Substitution	Nonsynonymous coding	31%
MM12T	OTUD7A	OTU domain containing 7A	CCDS10026.1	chr15_31776492-31776492_C_T	596A>T	Substitution	Nonsynonymous coding	33%
MM12T	OTX2	orthodenticle homeobox 2	CCDS9728.1	chr14_57270953-57270953_G_A	76R>X	Substitution	Nonsense	11%
MM12T	OXCT1	3-oxoacid CoA transferase 1	CCDS3937.1	chr5_41739521-41739521_C_T	498V>I	Substitution	Nonsynonymous coding	18%
MM12T	OXCT2	3-oxoacid CoA transferase 2	CCDS445.1	chr1_40236054-40236054_C_T	292A>T	Substitution	Nonsynonymous coding	23%
MM12T	OXCT2	3-oxoacid CoA transferase 2	CCDS445.1	chr1_40236290-40236290_C_A	213W>L	Substitution	Nonsynonymous coding	40%
MM12T	P2RX5	purinergic receptor P2X, ligand-gated ion channel, 5	CCDS11034.1	chr17_3593422-3593422_C_T	186E>K	Substitution	Nonsynonymous coding	37%

MM12T	P2RY10	purinergic receptor P2Y, G-protein coupled, 10	CCDS14442.1	chrX_78216132-78216132_A_G	39T>A	Substitution	Nonsynonymous coding	17%
MM12T	P2RY14	purinergic receptor P2Y, G-protein coupled, 14	CCDS3156.1	chr3_150932029-150932029_G_A	26P>S	Substitution	Nonsynonymous coding	30%
MM12T	P2RY4	pyrimidinergic receptor P2Y, G-protein coupled, 4	CCDS14398.1	chrX_69479315-69479315_C_T	54A>T	Substitution	Nonsynonymous coding	11%
MM12T	P4HA3	prolyl 4-hydroxylase, alpha polypeptide III	CCDS8230.1	chr11_73978342-73978342_T_C	529E>G	Substitution	Nonsynonymous coding	46%
MM12T	P4HTM	prolyl 4-hydroxylase, transmembrane (endoplasmic reticulum)	CCDS2781.2	chr3_49042451-49042451_G_A	349E>K	Substitution	Nonsynonymous coding	30%
MM12T	PA2G4	proliferation-associated 2G4, 38kDa	CCDS8902.1	chr12_56500503-56500503_A_	NA	Deletion	Splice site donor	20%
MM12T	PABPC4	poly(A) binding protein, cytoplasmic 4 (inducible form)	CCDS44115.1	chr1_40030200-40030200_G_A	450P>S	Substitution	Nonsynonymous coding	31%
MM12T	PACRG	PARK2 co-regulated	CCDS5284.1	chr6_163149275-163149275_C_T	3A>V	Substitution	Nonsynonymous coding	50%
MM12T	PADI6	peptidyl arginine deiminase, type VI	ENST00000358481	chr1_17708468-17708468_C_T	187T>M	Substitution	Nonsynonymous coding	18%
MM12T	PAEP	progestagen-associated endometrial protein	CCDS35173.1	chr9_138453678-138453678_G_A	11A>T	Substitution	Nonsynonymous coding	11%
MM12T	PAEP	progestagen-associated endometrial protein	CCDS35173.1	chr9_138456131-138456131_G_A	118D>N	Substitution	Nonsynonymous coding	10%
MM12T	PAFAH1B1	platelet-activating factor acetylhydrolase 1b, regulatory subunit 1 (45kDa)	CCDS32528.1	chr17_2573487-2573487_C_T	144R>X	Substitution	Nonsense	24%
MM12T	PAG1	phosphoprotein associated with glycosphingolipid microdomains 1	CCDS6227.1	chr8_81888912-81888912_G_A	389A>V	Substitution	Nonsynonymous coding	30%
MM12T	PAG1	phosphoprotein associated with glycosphingolipid microdomains 1	CCDS6227.1	chr8_81897261-81897261_G_A	209A>V	Substitution	Nonsynonymous coding	27%
MM12T	PALLD	palladin, cytoskeletal associated protein	CCDS34098.1	chr4_169845445-169845445_T_A	1016L>H	Substitution	Nonsynonymous coding	22%
MM12T	PALMD	palmdelphin	CCDS758.1	chr1_100154394-100154394_C_A	193S>Y	Substitution	Nonsynonymous coding	10%
MM12T	PAM	peptidylglycine alpha-amidating monooxygenase	CCDS43348.1	chr5_102285605-102285605_G_T	ISV-1>	Substitution	Splice site acceptor	24%
MM12T	PANK4	pantothenate kinase 4	CCDS42.1	chr1_2449631-2449631_C_T	397G>D	Substitution	Nonsynonymous coding	12%
MM12T	PANX1	pannexin 1	CCDS8296.1	chr11_93886708-93886708_C_T	78A>V	Substitution	Nonsynonymous coding	33%
MM12T	PANX2	pannexin 2	CCDS14085.2	chr22_50617600-50617600_G_A	643R>H	Substitution	Nonsynonymous coding	45%
MM12T	PANX3	pannexin 3	CCDS8447.1	chr11_124487203-124487203_C_T	120L>F	Substitution	Nonsynonymous coding	22%
MM12T	PAPD5	PAP associated domain containing 5	NM_001040284	chr16_50263102-50263102_T_G	654F>V	Substitution	Nonsynonymous coding	35%
MM12T	PAPLN	papilin, proteoglycan-like sulfated glycoprotein	CCDS32114.1	chr14_73718368-73718368_G_A	ISV-4>	Substitution	Splice site acceptor	28%
MM12T	PAPPA	pregnancy-associated plasma protein A, pappalysin 1	CCDS6813.1	chr9_118950086-118950086_G_A	357V>M	Substitution	Nonsynonymous coding	10%
MM12T	PAPPA	pregnancy-associated plasma protein A, pappalysin 1	CCDS6813.1	chr9_119115098-119115098_G_A	1360A>T	Substitution	Nonsynonymous coding	12%
MM12T	PAPPA2	pappalysin 2	CCDS41438.1	chr1_176526239-176526239_C_T	261P>S	Substitution	Nonsynonymous coding	31%
MM12T	PAPPA2	pappalysin 2	CCDS41438.1	chr1_176668479-176668479_G_A	997G>D	Substitution	Nonsynonymous coding	31%
MM12T	PAQR4	progestin and adipoQ receptor family member IV	CCDS10485.1	chr16_3021605-3021605_G_A	160A>T	Substitution	Nonsynonymous coding	35%
MM12T	PAQR7	progestin and adipoQ receptor family member VII	CCDS267.1	chr1_26189660-26189660_C_T	224R>H	Substitution	Nonsynonymous coding	30%
MM12T	PAQR8	progestin and adipoQ receptor family member VIII	CCDS4941.1	chr6_52268021-52268021_G_A	4A>T	Substitution	Nonsynonymous coding	59%
MM12T	PARD3B	par-3 partitioning defective 3 homolog B (C. elegans)	CCDS42806.1	chr2_205990379-205990379_C_T	451A>V	Substitution	Nonsynonymous coding	10%
MM12T	PARD6B	par-6 partitioning defective 6 homolog beta (C. elegans)	CCDS33485.1	chr20_49366904-49366904_G_A	333G>D	Substitution	Nonsynonymous coding	33%

MM12T	PARN	poly(A)-specific ribonuclease	CCDS45419.1	chr16_14540748-14540748_C_T	621A>T	Substitution	Nonsynonymous coding	26%
MM12T	PARP1	poly (ADP-ribose) polymerase 1	CCDS1554.1	chr1_226556021-226556021_G_A	719A>V	Substitution	Nonsynonymous coding	29%
MM12T	PARP10	poly (ADP-ribose) polymerase family, member 10 [Source:HGNC Symbol;Acc:25895]	CCDS34960.1	chr8_145058803-145058803_C_T	456R>H	Substitution	Nonsynonymous coding	30%
MM12T	PARP10	poly (ADP-ribose) polymerase family, member 10 [Source:HGNC Symbol;Acc:25895]	CCDS34960.1	chr8_145060056-145060056_C_T	90R>H	Substitution	Nonsynonymous coding	15%
MM12T	PARP14	poly (ADP-ribose) polymerase family, member 14	CCDS46894.1	chr3_122418394-122418394_G_A	331W>X	Substitution	Nonsense	12%
MM12T	PARP6	poly (ADP-ribose) polymerase family, member 6	CCDS10241.2	chr15_72545777-72545777_C_T	ISV+1>	Substitution	Splice site donor	19%
MM12T	PARP9	poly (ADP-ribose) polymerase family, member 9	CCDS3014.1	chr3_122274536-122274536_G_T	196A>D	Substitution	Nonsynonymous coding	28%
MM12T	PARP9	poly (ADP-ribose) polymerase family, member 9	CCDS3014.1	chr3_122277287-122277287_T_G	15K>Q	Substitution	Nonsynonymous coding	29%
MM12T	PARVG	parvin, gamma	CCDS14057.1	chr22_44583716-44583716_G_T	69E>X	Substitution	Nonsense	25%
MM12T	PASK	PAS domain containing serine/threonine kinase	CCDS2545.1	chr2_242076585-242076585_G_A	324A>V	Substitution	Nonsynonymous coding	15%
MM12T	PATL1	protein associated with topoisomerase II homolog 1 (yeast)	CCDS44613.1	chr11_59425058-59425058_G_T	189A>D	Substitution	Nonsynonymous coding	50%
MM12T	PAX1	paired box 1	CCDS13146.2	chr20_21687487-21687487_C_T	233P>L	Substitution	Nonsynonymous coding	27%
MM12T	PBRM1	polybromo 1	CCDS2859.1	chr3_52713694-52713694_A_G	12S>P	Substitution	Nonsynonymous coding	28%
MM12T	PBRM1	polybromo 1	CCDS2859.1	chr3_52651532-52651532_G_A	490R>X	Substitution	Nonsense	13%
MM12T	PC	pyruvate carboxylase	CCDS8152.1	chr11_66616447-66616447_C_T	1154E>K	Substitution	Nonsynonymous coding	27%
MM12T	PC	pyruvate carboxylase	CCDS8152.1	chr11_66617918-66617918_C_T	831V>M	Substitution	Nonsynonymous coding	25%
MM12T	PC	pyruvate carboxylase	CCDS8152.1	chr11_66617920-66617920_C_T	830R>H	Substitution	Nonsynonymous coding	14%
MM12T	PC	pyruvate carboxylase	CCDS8152.1	chr11_66638318-66638318_C_T	227A>T	Substitution	Nonsynonymous coding	28%
MM12T	PCDH1	protocadherin 1	CCDS4267.1	chr5_141233995-141233995_C_T	1109R>H	Substitution	Nonsynonymous coding	50%
MM12T	PCDH1	protocadherin 1	CCDS4267.1	chr5_141248454-141248454_G_A	195R>C	Substitution	Nonsynonymous coding	15%
MM12T	PCDH10	protocadherin 10	CCDS34063.1	chr4_134071536-134071536_C_A	81R>S	Substitution	Nonsynonymous coding	40%
MM12T	PCDH11X	protocadherin 11 X-linked	CCDS14461.1	chrX_91090574-91090574_A_G	24Q>R	Substitution	Nonsynonymous coding	25%
MM12T	PCDH12	protocadherin 12	CCDS4269.1	chr5_141325089-141325089_G_A	1138R>W	Substitution	Nonsynonymous coding	29%
MM12T	PCDH12	protocadherin 12	CCDS4269.1	chr5_141335012-141335012_G_A	802A>V	Substitution	Nonsynonymous coding	28%
MM12T	PCDH12	protocadherin 12	CCDS4269.1	chr5_141336975-141336975_C_T	148A>T	Substitution	Nonsynonymous coding	28%
MM12T	PCDH18	protocadherin 18	CCDS34064.1	chr4_138442520-138442520_G_A	1024S>F	Substitution	Nonsynonymous coding	36%
MM12T	PCDH18	protocadherin 18	CCDS34064.1	chr4_138452728-138452728_T_C	172H>R	Substitution	Nonsynonymous coding	46%
MM12T	PCDH19	protocadherin 19	CCDS43976.1	chrX_99661749-99661749_T_C	616E>G	Substitution	Nonsynonymous coding	30%
MM12T	PCDH19	protocadherin 19	CCDS43976.1	chrX_99661860-99661860_C_T	579R>H	Substitution	Nonsynonymous coding	15%
MM12T	PCDH19	protocadherin 19	CCDS43976.1	chrX_99661984-99661984_C_T	538G>S	Substitution	Nonsynonymous coding	12%
MM12T	PCDH19	protocadherin 19	CCDS43976.1	chrX_99663111-99663111_C_T	162G>D	Substitution	Nonsynonymous coding	12%
MM12T	PCDH7	protocadherin 7	CCDS43220.1	chr4_30724965-30724965_C_T	594Q>X	Substitution	Nonsense	40%

MM12T	PCDHA1	protocadherin alpha 1	CCDS34246.1	chr5_140166977-140166977_G_A	368V>I	Substitution	Nonsynonymous coding	26%
MM12T	PCDHA12	protocadherin alpha 12	CCDS47285.1	chr5_140256757-140256757_C_T	567A>V	Substitution	Nonsynonymous coding	42%
MM12T	PCDHA4	protocadherin alpha 4	CCDS34249.1	chr5_140189083-140189083_G_A	771A>T	Substitution	Nonsynonymous coding	34%
MM12T	PCDHA5	protocadherin alpha 5	CCDS34250.1	chr5_140202504-140202504_G_A	382G>R	Substitution	Nonsynonymous coding	30%
MM12T	PCDHA6	protocadherin alpha 6	CCDS34255.1	chr5_140236345-140236345_G_A	238A>T	Substitution	Nonsynonymous coding	30%
MM12T	PCDHA6	protocadherin alpha 6	CCDS4240.1	chr5_140262845-140262845_C_T	331A>V	Substitution	Nonsynonymous coding	15%
MM12T	PCDHA6	protocadherin alpha 6	CCDS4240.1	chr5_140264045-140264045_C_T	731A>V	Substitution	Nonsynonymous coding	13%
MM12T	PCDHA6	protocadherin alpha 6	CCDS4242.1	chr5_140346677-140346677_C_T	109A>V	Substitution	Nonsynonymous coding	13%
MM12T	PCDHA6	protocadherin alpha 6	CCDS4242.1	chr5_140347709-140347709_G_A	453R>H	Substitution	Nonsynonymous coding	11%
MM12T	PCDHA7	protocadherin alpha 7	CCDS34252.1	chr5_140216159-140216159_G_A	731A>T	Substitution	Nonsynonymous coding	31%
MM12T	PCDHB1	protocadherin beta 1	CCDS4243.1	chr5_140432615-140432615_G_T	520M>I	Substitution	Nonsynonymous coding	30%
MM12T	PCDHB13	protocadherin beta 13	CCDS4255.1	chr5_140594897-140594897_A_G	401Y>C	Substitution	Nonsynonymous coding	12%
MM12T	PCDHB15	protocadherin beta 15	CCDS4257.1	chr5_140625528-140625528_G_A	128D>N	Substitution	Nonsynonymous coding	11%
MM12T	PCDHB15	protocadherin beta 15	CCDS4257.1	chr5_140625880-140625880_C_T	245A>V	Substitution	Nonsynonymous coding	33%
MM12T	PCDHB16	protocadherin beta 16	CCDS4251.1	chr5_140562237-140562237_G_A	35V>I	Substitution	Nonsynonymous coding	28%
MM12T	PCDHB4	protocadherin beta 4	CCDS4246.1	chr5_140502512-140502512_T_A	311I>N	Substitution	Nonsynonymous coding	33%
MM12T	PCDHGA10	protocadherin gamma subfamily A, 10	CCDS47292.1	chr5_140793371-140793371_C_A	210A>D	Substitution	Nonsynonymous coding	12%
MM12T	PCDHGA2	protocadherin gamma subfamily A, 2	CCDS4260.1	chr5_140810619-140810619_G_A	98G>E	Substitution	Nonsynonymous coding	10%
MM12T	PCDHGA8	protocadherin gamma subfamily A, 8	CCDS47291.1	chr5_140774001-140774001_C_T	541P>S	Substitution	Nonsynonymous coding	28%
MM12T	PCDHGB1	protocadherin gamma subfamily B, 1	NM_018922	chr5_140730125-140730125_G_A	100A>T	Substitution	Nonsynonymous coding	30%
MM12T	PCED1B	PC-esterase domain containing 1B	CCDS8752.1	chr12_47629403-47629403_C_T	186A>V	Substitution	Nonsynonymous coding	30%
MM12T	PCF11	PCF11, cleavage and polyadenylation factor subunit, homolog (S. cerevisiae)	CCDS44689.1	chr11_82879847-82879847_C_T	824P>S	Substitution	Nonsynonymous coding	13%
MM12T	PCIF1	PDX1 C-terminal inhibiting factor 1	CCDS13388.1	chr20_44571766-44571766_G_A	235R>H	Substitution	Nonsynonymous coding	28%
MM12T	PCMTD2	protein-L-isoaspartate (D-aspartate) O-methyltransferase domain containing 2	CCDS13559.1	chr20_62891433-62891433_G_A	39A>T	Substitution	Nonsynonymous coding	11%
MM12T	PCNT	pericentrin	CCDS33592.1	chr21_47766688-47766688_G_A	251R>H	Substitution	Nonsynonymous coding	17%
MM12T	PCNXL2	pecanex-like 2 (Drosophila)	CCDS44335.1	chr1_233275511-233275511_G_A	1203A>V	Substitution	Nonsynonymous coding	22%
MM12T	PCNXL2	pecanex-like 2 (Drosophila)	CCDS44335.1	chr1_233297019-233297019_G_A	1122R>X	Substitution	Nonsense	11%
MM12T	PCP2	Purkinje cell protein 2	CCDS32893.1	chr19_7697325-7697325_C_T	82R>H	Substitution	Nonsynonymous coding	37%
MM12T	PCSK1	proprotein convertase subtilisin/kexin type 1	CCDS4081.1	chr5_95757618-95757618_G_A	196P>S	Substitution	Nonsynonymous coding	33%
MM12T	PCSK2	proprotein convertase subtilisin/kexin type 2	CCDS13125.1	chr20_17462633-17462633_C_T	612A>V	Substitution	Nonsynonymous coding	39%
MM12T	PCYT1B	phosphate cytidylyltransferase 1, choline, beta	CCDS14213.1	chrX_24625946-24625946_T_G	84I>L	Substitution	Nonsynonymous coding	30%
MM12T	PDCD1	programmed cell death 1	CCDS33428.1	chr2_242794496-242794496_G_A	149A>V	Substitution	Nonsynonymous coding	10%

MM12T	PDE10A	phosphodiesterase 10A	CCDS47513.1	chr6_165809932-165809932_C_T	432R>H	Substitution	Nonsynonymous coding	45%
MM12T	PDE1A	phosphodiesterase 1A, calmodulin-dependent	CCDS2285.1	chr2_183387076-183387076_C_T	10E>K	Substitution	Nonsynonymous coding	11%
MM12T	PDE4B	phosphodiesterase 4B, cAMP-specific	CCDS632.1	chr1_66833662-66833662_C_A	526L>M	Substitution	Nonsynonymous coding	23%
MM12T	PDE4DIP	phosphodiesterase 4D interacting protein	CCDS30824.1	chr1_144866642-144866642_C_T	1867R>H	Substitution	Nonsynonymous coding	12%
MM12T	PDE4DIP	phosphodiesterase 4D interacting protein	CCDS30824.1	chr1_144922015-144922015_C_A	338Q>H	Substitution	Nonsynonymous coding	13%
MM12T	PDE5A	phosphodiesterase 5A, cGMP-specific	CCDS3713.1	chr4_120422375-120422375_G_A	814P>S	Substitution	Nonsynonymous coding	11%
MM12T	PDE6A	phosphodiesterase 6A, cGMP-specific, rod, alpha	CCDS4299.1	chr5_149264055-149264055_C_T	672A>T	Substitution	Nonsynonymous coding	33%
MM12T	PDE6B	phosphodiesterase 6B, cGMP-specific, rod, beta	CCDS33932.1	chr4_647722-647722_C_T	265R>W	Substitution	Nonsynonymous coding	11%
MM12T	PDE7B	phosphodiesterase 7B	CCDS5175.1	chr6_136512931-136512931_G_A	436A>T	Substitution	Nonsynonymous coding	28%
MM12T	PDE9A	phosphodiesterase 9A	CCDS13690.1	chr21_44180951-44180951_C_T	340T>M	Substitution	Nonsynonymous coding	39%
MM12T	PDGFRB	platelet-derived growth factor receptor, beta polypeptide	CCDS4303.1	chr5_149513527-149513527_G_A	226R>C	Substitution	Nonsynonymous coding	29%
MM12T	PDHA1	pyruvate dehydrogenase (lipoamide) alpha 1	CCDS14192.1	chrX_19373555-19373555_C_T	231T>M	Substitution	Nonsynonymous coding	28%
MM12T	PDHA2	pyruvate dehydrogenase (lipoamide) alpha 2	CCDS3644.1	chr4_96762303-96762303_G_T	334K>N	Substitution	Nonsynonymous coding	22%
MM12T	PDIA2	protein disulfide isomerase family A, member 2	CCDS42089.1	chr16_335175-335175_G_A	257R>H	Substitution	Nonsynonymous coding	33%
MM12T	PDIA3	protein disulfide isomerase family A, member 3	CCDS10101.1	chr15_44062751-44062751_C_G	459N>K	Substitution	Nonsynonymous coding	10%
MM12T	PDIA4	protein disulfide isomerase family A, member 4	CCDS5893.1	chr7_148709258-148709258_C_T	270R>Q	Substitution	Nonsynonymous coding	36%
MM12T	PDIA4	protein disulfide isomerase family A, member 4	CCDS5893.1	chr7_148709292-148709292_G_A	259R>C	Substitution	Nonsynonymous coding	12%
MM12T	PDILT	protein disulfide isomerase-like, testis expressed	CCDS10584.1	chr16_20387410-20387410_C_T	175V>I	Substitution	Nonsynonymous coding	12%
MM12T	PDLIM5	PDZ and LIM domain 5	CCDS3641.1	chr4_95561579-95561579_G_A	421A>T	Substitution	Nonsynonymous coding	37%
MM12T	PDPN	podoplanin	CCDS30602.1	chr1_13933722-13933722_T_C	117V>A	Substitution	Nonsynonymous coding	14%
MM12T	PDPR	pyruvate dehydrogenase phosphatase regulatory subunit	CCDS45520.1	chr16_70190685-70190685_G_A	848R>H	Substitution	Nonsynonymous coding	11%
MM12T	PDRG1	p53 and DNA-damage regulated 1	CCDS13194.1	chr20_30534344-30534344_G_A	92R>W	Substitution	Nonsynonymous coding	31%
MM12T	PDS5A	PDS5, regulator of cohesion maintenance, homolog A (S. cerevisiae)	CCDS47045.1	chr4_39929664-39929664_G_A	87R>C	Substitution	Nonsynonymous coding	10%
MM12T	PDS5B	PDS5, regulator of cohesion maintenance, homolog B (S. cerevisiae)	CCDS41878.1	chr13_33233388-33233388_G_A	199V>I	Substitution	Nonsynonymous coding	31%
MM12T	PDS5B	PDS5, regulator of cohesion maintenance, homolog B (S. cerevisiae)	CCDS41878.1	chr13_33327528-33327528_G_A	932R>H	Substitution	Nonsynonymous coding	36%
MM12T	PDS5B	PDS5, regulator of cohesion maintenance, homolog B (S. cerevisiae)	CCDS41878.1	chr13_33334791-33334791_C_T	1151R>X	Substitution	Nonsense	34%
MM12T	PDSS2	prenyl (decaprenyl) diphosphate synthase, subunit 2	CCDS5059.1	chr6_107533420-107533420_C_T	257A>T	Substitution	Nonsynonymous coding	47%
MM12T	PDZD2	PDZ domain containing 2	CCDS34137.1	chr5_32089802-32089802_G_A	2083R>H	Substitution	Nonsynonymous coding	12%
MM12T	PDZD4	PDZ domain containing 4	CCDS14732.1	chrX_153069544-153069544_G_A	525A>V	Substitution	Nonsynonymous coding	37%
MM12T	PDZD4	PDZ domain containing 4	CCDS14732.1	chrX_153072751-153072751_C_T	124D>N	Substitution	Nonsynonymous coding	27%
MM12T	PDZD7	PDZ domain containing 7	CCDS31269.1	chr10_102783715-102783715_C_T	113V>I	Substitution	Nonsynonymous coding	31%
MM12T	PEAR1	platelet endothelial aggregation receptor 1	CCDS30892.1	chr1_156878608-156878608_C_T	426A>V	Substitution	Nonsynonymous coding	26%

MM12T	PEG3	paternally expressed 3	CCDS33123.1	chr19_57286891-57286891_G_T	250S>Y	Substitution	Nonsynonymous coding	60%
MM12T	PELI1	pellino E3 ubiquitin protein ligase 1	CCDS1876.1	chr2_64321886-64321886_C_T	403A>T	Substitution	Nonsynonymous coding	14%
MM12T	PEPD	peptidase D	CCDS42544.1	chr19_33892638-33892638_G_A	319A>V	Substitution	Nonsynonymous coding	38%
MM12T	PER2	period circadian clock 2	CCDS2528.1	chr2_239180144-239180144_G_A	194A>V	Substitution	Nonsynonymous coding	16%
MM12T	PER3	period circadian clock 3	CCDS89.1	chr1_7845620-7845620_C_T	83A>V	Substitution	Nonsynonymous coding	43%
MM12T	PET112	PET112 homolog (yeast)	CCDS3776.1	chr4_152638096-152638096_T_C	191E>G	Substitution	Nonsynonymous coding	39%
MM12T	PEX14	peroxisomal biogenesis factor 14	CCDS30582.1	chr1_10684484-10684484_C_T	192A>V	Substitution	Nonsynonymous coding	32%
MM12T	PEX5L	peroxisomal biogenesis factor 5-like	CCDS3236.1	chr3_179615979-179615979_G_A	50P>L	Substitution	Nonsynonymous coding	28%
MM12T	PFAS	phosphoribosylformylglycinamidine synthase	CCDS11136.1	chr17_8160195-8160195_G_A	330R>Q	Substitution	Nonsynonymous coding	16%
MM12T	PFDN5	prefoldin subunit 5	CCDS8853.1	chr12_53691931-53691931_C_T	129Q>X	Substitution	Nonsense	16%
MM12T	PFKFB2	6-phosphofructo-2-kinase/fructose-2,6-biphosphatase 2	CCDS31004.1	chr1_207235986-207235986_G_A	78R>Q	Substitution	Nonsynonymous coding	26%
MM12T	PFKL	phosphofructokinase, liver	CCDS33582.1	chr21_45739268-45739268_G_T	366R>M	Substitution	Nonsynonymous coding	28%
MM12T	PFKL	phosphofructokinase, liver	CCDS33582.1	chr21_45741670-45741670_C_T	417A>V	Substitution	Nonsynonymous coding	13%
MM12T	PFKL	phosphofructokinase, liver	CCDS33582.1	chr21_45745122-45745122_C_T	655T>I	Substitution	Nonsynonymous coding	31%
MM12T	PFN3	profilin 3	CCDS34301.1	chr5_176827169-176827169_C_T	137A>T	Substitution	Nonsynonymous coding	18%
MM12T	PGBD2	piggyBac transposable element derived 2	CCDS31128.1	chr1_249211572-249211572_A_C	263E>D	Substitution	Nonsynonymous coding	32%
MM12T	PGBD5	piggyBac transposable element derived 5	CCDS1583.1	chr1_230493051-230493051_C_T	146M>I	Substitution	Nonsynonymous coding	16%
MM12T	PGLS	6-phosphogluconolactonase	CCDS12361.1	chr19_17631792-17631792_G_A	227A>T	Substitution	Nonsynonymous coding	21%
MM12T	PGLYRP3	peptidoglycan recognition protein 3	CCDS1035.1	chr1_153277444-153277444_C_T	119A>T	Substitution	Nonsynonymous coding	24%
MM12T	PGM2	phosphoglucomutase 2	CCDS3443.1	chr4_37831590-37831590_C_T	29S>F	Substitution	Nonsynonymous coding	19%
MM12T	PGS1	phosphatidylglycerophosphate synthase 1	CCDS42391.1	chr17_76396795-76396795_G_T	247D>Y	Substitution	Nonsynonymous coding	33%
MM12T	PHACTR1	phosphatase and actin regulator 1	NM_030948	chr6_13283703-13283703_T_C	520I>T	Substitution	Nonsynonymous coding	30%
MM12T	PHACTR3	phosphatase and actin regulator 3	CCDS13480.1	chr20_58322834-58322834_G_T	101G>V	Substitution	Nonsynonymous coding	14%
MM12T	PHEX	phosphate regulating endopeptidase homolog, X-linked	CCDS14204.1	chrX_22117199-22117199_G_A	337E>K	Substitution	Nonsynonymous coding	11%
MM12T	PHF13	PHD finger protein 13	CCDS85.1	chr1_6676856-6676856_G_T	27D>Y	Substitution	Nonsynonymous coding	13%
MM12T	PHF17	PHD finger protein 17	CCDS34062.1	chr4_129792734-129792734_G_T	616D>Y	Substitution	Nonsynonymous coding	12%
MM12T	PHF2	PHD finger protein 2	CCDS35069.1	chr9_96422493-96422493_G_A	450R>Q	Substitution	Nonsynonymous coding	31%
MM12T	PHF20L1	PHD finger protein 20-like 1	CCDS6367.2	chr8_133816947-133816947_C_T	270S>L	Substitution	Nonsynonymous coding	35%
MM12T	PHF21A	PHD finger protein 21A	CCDS44578.1	chr11_45992798-45992798_C_T	161A>T	Substitution	Nonsynonymous coding	27%
MM12T	PHF21B	PHD finger protein 21B	CCDS14061.1	chr22_45279115-45279115_G_A	483R>W	Substitution	Nonsynonymous coding	13%
MM12T	PHF8	PHD finger protein 8	CCDS14355.1	chrX_54013548-54013548_C_T	653R>Q	Substitution	Nonsynonymous coding	22%
MM12T	PHGDH	phosphoglycerate dehydrogenase	CCDS904.1	chr1_120285647-120285647_C_T	476A>V	Substitution	Nonsynonymous coding	25%

MM12T	PHIP	pleckstrin homology domain interacting protein	CCDS4987.1	chr6_79727258-79727258_C_T	346R>Q	Substitution	Nonsynonymous coding	14%
MM12T	PHLDB3	pleckstrin homology-like domain, family B, member 3	CCDS12621.2	chr19_44001926-44001926_C_T	213E>K	Substitution	Nonsynonymous coding	28%
MM12T	PHLDB3	pleckstrin homology-like domain, family B, member 3	CCDS12621.2	chr19_44006027-44006027_C_T	ISV-4>	Substitution	Splice site acceptor	13%
MM12T	PHTF1	putative homeodomain transcription factor 1	CCDS861.1	chr1_114248697-114248697_G_A	496L>F	Substitution	Nonsynonymous coding	26%
MM12T	PHYH	phytanoyl-CoA 2-hydroxylase	CCDS7097.1	chr10_13320332-13320332_C_T	329R>Q	Substitution	Nonsynonymous coding	23%
MM12T	PHYHD1	phytanoyl-CoA dioxygenase domain containing 1	CCDS6914.1	chr9_131702694-131702694_C_T	161A>V	Substitution	Nonsynonymous coding	12%
MM12T	PI4K2A	phosphatidylinositol 4-kinase type 2 alpha	CCDS7467.1	chr10_99359876-99359876_T_C	219V>A	Substitution	Nonsynonymous coding	33%
MM12T	PIBF1	progesterone immunomodulatory binding factor 1	CCDS31991.1	chr13_73409509-73409509_A_	NA	Deletion	Splice site donor	19%
MM12T	PIDD	p53-induced death domain protein	CCDS7716.1	chr11_800377-800377_C_T	706V>M	Substitution	Nonsynonymous coding	12%
MM12T	PIEZO1	piezo-type mechanosensitive ion channel component 1	NM_001142864	chr16_88782461-88782461_C_T	2399G>D	Substitution	Nonsynonymous coding	32%
MM12T	PIEZO1	piezo-type mechanosensitive ion channel component 1	NM_001142864	chr16_88787640-88787640_G_A	1868R>C	Substitution	Nonsynonymous coding	25%
MM12T	PIEZO1	piezo-type mechanosensitive ion channel component 1	NM_001142864	chr16_88799092-88799092_G_T	905L>M	Substitution	Nonsynonymous coding	23%
MM12T	PIF1	PIF1 5'-to-3' DNA helicase homolog (S. cerevisiae)	CCDS10195.2	chr15_65112130-65112130_G_A	417R>X	Substitution	Nonsense	23%
MM12T	PIGO	phosphatidylinositol glycan anchor biosynthesis, class O	CCDS6575.1	chr9_35095305-35095305_C_A	86Q>H	Substitution	Nonsynonymous coding	29%
MM12T	PIGS	phosphatidylinositol glycan anchor biosynthesis, class S	CCDS11235.1	chr17_26885574-26885574_C_T	285R>H	Substitution	Nonsynonymous coding	30%
MM12T	PIGT	phosphatidylinositol glycan anchor biosynthesis, class T	CCDS13353.1	chr20_44054210-44054210_C_T	ISV-4>	Substitution	Splice site acceptor	50%
MM12T	PIK3AP1	phosphoinositide-3-kinase adaptor protein 1	CCDS31259.1	chr10_98405263-98405263_C_T	448A>T	Substitution	Nonsynonymous coding	35%
MM12T	PIK3C2A	phosphatidylinositol-4-phosphate 3-kinase, catalytic subunit type 2 alpha	CCDS7824.1	chr11_17113176-17113176_C_T	1557G>D	Substitution	Nonsynonymous coding	17%
MM12T	PIK3C2A	phosphatidylinositol-4-phosphate 3-kinase, catalytic subunit type 2 alpha	CCDS7824.1	chr11_17118815-17118815_C_A	ISV-4>	Substitution	Splice site acceptor	13%
MM12T	PIK3C2B	phosphatidylinositol-4-phosphate 3-kinase, catalytic subunit type 2 beta	CCDS1446.1	chr1_204434384-204434384_C_A	333D>Y	Substitution	Nonsynonymous coding	25%
MM12T	PIK3C2G	phosphatidylinositol-4-phosphate 3-kinase, catalytic subunit type 2 gamma	CCDS44839.1	chr12_18435082-18435082_G_A	23E>K	Substitution	Nonsynonymous coding	11%
MM12T	PIK3C2G	phosphatidylinositol-4-phosphate 3-kinase, catalytic subunit type 2 gamma	CCDS44839.1	chr12_18716329-18716329_A_G	1226T>A	Substitution	Nonsynonymous coding	24%
MM12T	PIK3C3	phosphatidylinositol 3-kinase, catalytic subunit type 3	CCDS11920.1	chr18_39609380-39609380_G_A	561R>H	Substitution	Nonsynonymous coding	15%
MM12T	PIK3CA	phosphatidylinositol-4,5-bisphosphate 3-kinase, catalytic subunit alpha	CCDS43171.1	chr3_178936082-178936082_G_A	542E>K	Substitution	Nonsynonymous coding	30%
MM12T	PIK3CD	phosphatidylinositol-4,5-bisphosphate 3-kinase, catalytic subunit delta	CCDS104.1	chr1_9776550-9776550_C_T	218A>V	Substitution	Nonsynonymous coding	15%
MM12T	PIK3CD	phosphatidylinositol-4,5-bisphosphate 3-kinase, catalytic subunit delta	CCDS104.1	chr1_9783298-9783298_G_A	848A>T	Substitution	Nonsynonymous coding	37%
MM12T	PIK3CG	phosphatidylinositol-4,5-bisphosphate 3-kinase, catalytic subunit gamma	CCDS5739.1	chr7_106508746-106508746_G_A	247S>N	Substitution	Nonsynonymous coding	11%
MM12T	PIK3CG	phosphatidylinositol-4,5-bisphosphate 3-kinase, catalytic subunit gamma	CCDS5739.1	chr7_106509676-106509676_C_T	557A>V	Substitution	Nonsynonymous coding	15%
MM12T	PIK3R2	phosphoinositide-3-kinase, regulatory subunit 2 (beta)	CCDS12371.1	chr19_18266771-18266771_G_A	28G>S	Substitution	Nonsynonymous coding	27%
MM12T	PIK3R4	phosphoinositide-3-kinase, regulatory subunit 4	CCDS3067.1	chr3_130463686-130463686_C_T	126R>H	Substitution	Nonsynonymous coding	32%
MM12T	PIK3R5	phosphoinositide-3-kinase, regulatory subunit 5	CCDS11147.1	chr17_8791666-8791666_G_A	480P>S	Substitution	Nonsynonymous coding	28%
MM12T	PINK1	PTEN induced putative kinase 1	CCDS211.1	chr1_20975543-20975543_C_T	436A>V	Substitution	Nonsynonymous coding	13%

MM12T	PIP5K1C	phosphatidylinositol-4-phosphate 5-kinase, type I, gamma	CCDS32872.1	chr19_3662002-3662002_G_A	ISV-3>	Substitution	Splice site acceptor	43%
MM12T	PISD	phosphatidylserine decarboxylase	CCDS13899.1	chr22_32021777-32021777_G_A	9Q>X	Substitution	Nonsense	23%
MM12T	PITHD1	PITH (C-terminal proteasome-interacting domain of thioredoxin-like) domain containing	CCDS240.1	chr1_24112219-24112219_G_T	125Q>H	Substitution	Nonsynonymous coding	12%
MM12T	PITPNM1	phosphatidylinositol transfer protein, membrane-associated 1	CCDS31620.1	chr11_67262552-67262552_C_T	876R>H	Substitution	Nonsynonymous coding	16%
MM12T	PITPNM2	phosphatidylinositol transfer protein, membrane-associated 2	CCDS9242.1	chr12_123472783-123472783_G_A	999R>W	Substitution	Nonsynonymous coding	38%
MM12T	PITPNM2	phosphatidylinositol transfer protein, membrane-associated 2	ENST00000280562	chr12_123476343-123476343_G_A	836A>V	Substitution	Nonsynonymous coding	29%
MM12T	PITPNM2	phosphatidylinositol transfer protein, membrane-associated 2	CCDS9242.1	chr12_123494476-123494476_C_T	188M>I	Substitution	Nonsynonymous coding	32%
MM12T	PIWIL1	piwi-like 1 (Drosophila)	CCDS9268.1	chr12_130839432-130839432_G_T	ISV-1>	Substitution	Splice site acceptor	42%
MM12T	PIWIL3	piwi-like 3 (Drosophila)	CCDS33623.1	chr22_25145702-25145702_G_A	392R>C	Substitution	Nonsynonymous coding	22%
MM12T	PKD1L1	polycystic kidney disease 1 like 1	CCDS34633.1	chr7_47847859-47847859_C_T	2605A>T	Substitution	Nonsynonymous coding	24%
MM12T	PKD1L1	polycystic kidney disease 1 like 1	CCDS34633.1	chr7_47867033-47867033_G_A	2257R>C	Substitution	Nonsynonymous coding	26%
MM12T	PKD1L3	polycystic kidney disease 1-like 3	ENST00000335106	chr16_71976606-71976606_C_T	1392R>H	Substitution	Nonsynonymous coding	11%
MM12T	PKD2L1	polycystic kidney disease 2-like 1	CCDS7492.1	chr10_102052798-102052798_C_T	596R>H	Substitution	Nonsynonymous coding	16%
MM12T	PKDREJ	polycystic kidney disease (polycystin) and REJ homolog (sperm receptor for egg jelly	CCDS14073.1	chr22_46652461-46652463_AAC_	NA	Deletion	In-frame deletion	33%
MM12T	PKDREJ	polycystic kidney disease (polycystin) and REJ homolog (sperm receptor for egg jelly	CCDS14073.1	chr22_46653320-46653320_A_G	1967L>S	Substitution	Nonsynonymous coding	27%
MM12T	PKDREJ	polycystic kidney disease (polycystin) and REJ homolog (sperm receptor for egg jelly	CCDS14073.1	chr22_46655714-46655714_G_T	1169P>H	Substitution	Nonsynonymous coding	21%
MM12T	PKHD1	polycystic kidney and hepatic disease 1 (autosomal recessive)	CCDS4935.1	chr6_51768489-51768489_A_G	2301V>A	Substitution	Nonsynonymous coding	40%
MM12T	PKHD1	polycystic kidney and hepatic disease 1 (autosomal recessive)	CCDS4935.1	chr6_51915066-51915066_C_T	723R>H	Substitution	Nonsynonymous coding	49%
MM12T	PKN1	protein kinase N1	CCDS42514.1	chr19_14578727-14578727_G_A	648A>T	Substitution	Nonsynonymous coding	35%
MM12T	PKN3	protein kinase N3	CCDS6908.1	chr9_131482097-131482097_T_C	753L>P	Substitution	Nonsynonymous coding	31%
MM12T	PKP3	plakophilin 3	CCDS7695.1	chr11_399058-399058_C_T	379R>C	Substitution	Nonsynonymous coding	11%
MM12T	PLA2G6	phospholipase A2, group VI (cytosolic, calcium-independent)	CCDS13967.1	chr22_38541454-38541454_C_T	139R>H	Substitution	Nonsynonymous coding	32%
MM12T	PLA2R1	phospholipase A2 receptor 1, 180kDa	CCDS33309.1	chr2_160825844-160825844_T_A	896Y>F	Substitution	Nonsynonymous coding	38%
MM12T	PLAT	plasminogen activator, tissue	CCDS6126.1	chr8_42036494-42036494_C_A	484R>I	Substitution	Nonsynonymous coding	30%
MM12T	PLB1	phospholipase B1	CCDS33168.1	chr2_28843808-28843808_G_A	1164A>T	Substitution	Nonsynonymous coding	30%
MM12T	PLBD2	phospholipase B domain containing 2	CCDS9168.1	chr12_113812282-113812282_C_T	183R>W	Substitution	Nonsynonymous coding	17%
MM12T	PLCB1	phospholipase C, beta 1 (phosphoinositide-specific)	CCDS13102.1	chr20_8689399-8689399_C_T	417S>F	Substitution	Nonsynonymous coding	37%
MM12T	PLCB1	phospholipase C, beta 1 (phosphoinositide-specific)	CCDS13102.1	chr20_8717805-8717805_C_A	725P>H	Substitution	Nonsynonymous coding	27%
MM12T	PLCB2	phospholipase C, beta 2	CCDS42020.1	chr15_40582827-40582827_G_A	1054R>W	Substitution	Nonsynonymous coding	32%
MM12T	PLCB3	phospholipase C, beta 3 (phosphatidylinositol-specific)	CCDS8064.1	chr11_64021917-64021917_C_T	44R>C	Substitution	Nonsynonymous coding	11%
MM12T	PLCB3	phospholipase C, beta 3 (phosphatidylinositol-specific)	CCDS8064.1	chr11_64026048-64026048_G_T	372E>D	Substitution	Nonsynonymous coding	24%
MM12T	PLCB3	phospholipase C, beta 3 (phosphatidylinositol-specific)	CCDS8064.1	chr11_64026070-64026070_G_A	380G>S	Substitution	Nonsynonymous coding	12%

MM12T	PLCB3	phospholipase C, beta 3 (phosphatidylinositol-specific)	CCDS8064.1	chr11_64031499-64031499_C_T	856A>V	Substitution	Nonsynonymous coding	33%
MM12T	PLCB3	phospholipase C, beta 3 (phosphatidylinositol-specific)	CCDS8064.1	chr11_64035010-64035010_G_T	1229E>D	Substitution	Nonsynonymous coding	25%
MM12T	PLCD1	phospholipase C, delta 1	CCDS46793.1	chr3_38052939-38052939_G_A	ISV-3>	Substitution	Splice site acceptor	44%
MM12T	PLCD3	phospholipase C, delta 3	ENST00000322765	chr17_43190300-43190300_C_A	737D>Y	Substitution	Nonsynonymous coding	39%
MM12T	PLCD4	phospholipase C, delta 4	CCDS46516.1	chr2_219480690-219480690_C_T	29S>F	Substitution	Nonsynonymous coding	17%
MM12T	PLCE1	phospholipase C, epsilon 1	CCDS41552.1	chr10_96066260-96066260_C_A	1900P>H	Substitution	Nonsynonymous coding	31%
MM12T	PLCG2	phospholipase C, gamma 2 (phosphatidylinositol-specific)	CCDS42204.1	chr16_81942130-81942130_G_A	556G>D	Substitution	Nonsynonymous coding	12%
MM12T	PLCH2	phospholipase C, eta 2	NM_014638	chr1_2409940-2409940_A_C	50Q>H	Substitution	Nonsynonymous coding	26%
MM12T	PLCH2	phospholipase C, eta 2	ENST00000449969	chr1_2434146-2434146_C_A	1059P>H	Substitution	Nonsynonymous coding	35%
MM12T	PLD1	phospholipase D1, phosphatidylcholine-specific	CCDS3216.1	chr3_171455452-171455452_A_	NA	Deletion	Splice site acceptor	45%
MM12T	PLD3	phospholipase D family, member 3	CCDS33027.1	chr19_40872541-40872541_G_A	18A>T	Substitution	Nonsynonymous coding	17%
MM12T	PLD5	phospholipase D family, member 5	CCDS1621.1	chr1_242271010-242271010_G_A	309A>V	Substitution	Nonsynonymous coding	41%
MM12T	PLEC	plectin	CCDS43772.1	chr8_144991580-144991580_G_A	4274R>C	Substitution	Nonsynonymous coding	15%
MM12T	PLEC	plectin	CCDS43772.1	chr8_144992015-144992015_G_A	4129R>C	Substitution	Nonsynonymous coding	34%
MM12T	PLEC	plectin	CCDS43772.1	chr8_144999828-144999828_C_A	1560Q>H	Substitution	Nonsynonymous coding	30%
MM12T	PLEC	plectin	CCDS43772.1	chr8_144999940-144999940_G_A	1523A>V	Substitution	Nonsynonymous coding	31%
MM12T	PLEC	plectin	CCDS43772.1	chr8_145000972-145000972_G_A	1479R>W	Substitution	Nonsynonymous coding	31%
MM12T	PLEC	plectin	CCDS43772.1	chr8_145004457-145004457_G_A	960H>Y	Substitution	Nonsynonymous coding	29%
MM12T	PLEC	plectin	CCDS43772.1	chr8_145004619-145004619_C_T	934R>H	Substitution	Nonsynonymous coding	26%
MM12T	PLEC	plectin	CCDS43772.1	chr8_145007212-145007212_C_T	633V>M	Substitution	Nonsynonymous coding	34%
MM12T	PLEKHA1	pleckstrin homology domain containing, family A (phosphoinositide binding specific) member	CCDS7629.1	chr10_124159888-124159888_G_T	76K>N	Substitution	Nonsynonymous coding	22%
MM12T	PLEKHA1	pleckstrin homology domain containing, family A (phosphoinositide binding specific) member	CCDS7629.1	chr10_124189218-124189218_G_T	327A>S	Substitution	Nonsynonymous coding	26%
MM12T	PLEKHA4	pleckstrin homology domain containing, family A (phosphoinositide binding specific) member	CCDS12737.1	chr19_49362227-49362227_G_A	288R>C	Substitution	Nonsynonymous coding	26%
MM12T	PLEKHA5	pleckstrin homology domain containing, family A member 5	CCDS8682.1	chr12_19511352-19511352_A_T	944N>I	Substitution	Nonsynonymous coding	39%
MM12T	PLEKHA5	pleckstrin homology domain containing, family A member 5	CCDS8682.1	chr12_19410475-19410476_TT_	NA	Deletion	Splice site donor	13%
MM12T	PLEKHA6	pleckstrin homology domain containing, family A member 6	CCDS1444.1	chr1_204230473-204230473_G_A	162P>L	Substitution	Nonsynonymous coding	10%
MM12T	PLEKHA8P1	pleckstrin homology domain containing, family A member 8 pseudogene 1	CCDS8747.1	chr12_45567136-45567136_G_A	338A>V	Substitution	Nonsynonymous coding	35%
MM12T	PLEKHG1	pleckstrin homology domain containing, family G (with RhoGef domain) member 1	CCDS34552.1	chr6_151130343-151130343_C_T	339R>W	Substitution	Nonsynonymous coding	11%
MM12T	PLEKHG1	pleckstrin homology domain containing, family G (with RhoGef domain) member 1	CCDS34552.1	chr6_151153329-151153329_G_T	1028G>W	Substitution	Nonsynonymous coding	18%
MM12T	PLEKHG2	pleckstrin homology domain containing, family G (with RhoGef domain) member 2	CCDS33022.2	chr19_39915047-39915047_G_A	1092D>N	Substitution	Nonsynonymous coding	10%
MM12T	PLEKHG3	pleckstrin homology domain containing, family G (with RhoGef domain) member 3	CCDS32098.1	chr14_65198167-65198167_G_A	257R>H	Substitution	Nonsynonymous coding	30%
MM12T	PLEKHG4	pleckstrin homology domain containing, family G (with RhoGef domain) member 4	CCDS32466.1	chr16_67320490-67320490_G_A	892R>Q	Substitution	Nonsynonymous coding	26%

MM12T	PLEKHG4B	pleckstrin homology domain containing, family G (with RhoGef domain) member 4B	CCDS34124.1	chr5_143284-143284_G_A	178G>S	Substitution	Nonsynonymous coding	39%
MM12T	PLEKHG4B	pleckstrin homology domain containing, family G (with RhoGef domain) member 4B	CCDS34124.1	chr5_171491-171491_G_A	972V>I	Substitution	Nonsynonymous coding	32%
MM12T	PLEKHG4B	pleckstrin homology domain containing, family G (with RhoGef domain) member 4B	CCDS34124.1	chr5_182166-182166_G_A	1182A>T	Substitution	Nonsynonymous coding	12%
MM12T	PLEKHG5	pleckstrin homology domain containing, family G (with RhoGef domain) member 5	CCDS41240.1	chr1_6531690-6531690_G_A	457A>V	Substitution	Nonsynonymous coding	12%
MM12T	PLEKHH1	pleckstrin homology domain containing, family H (with MyTH4 domain) member 1	CCDS45128.1	chr14_68042685-68042685_G_A	772S>N	Substitution	Nonsynonymous coding	12%
MM12T	PLEKHO1	pleckstrin homology domain containing, family O member 1	ENST00000441340	chr1_150129573-150129573_C_A	20L>I	Substitution	Nonsynonymous coding	39%
MM12T	PLEKHS1	pleckstrin homology domain containing, family S member 1	CCDS7583.1	chr10_115534048-115534048_G_T	245Q>H	Substitution	Nonsynonymous coding	34%
MM12T	PLG	plasminogen	CCDS5279.1	chr6_161139801-161139801_C_T	343R>W	Substitution	Nonsynonymous coding	43%
MM12T	PLG	plasminogen	CCDS5279.1	chr6_161135825-161135825_G_A	ISV-1>	Substitution	Splice site acceptor	33%
MM12T	PLIN3	perilipin 3	CCDS12137.1	chr19_4847789-4847789_C_T	250A>T	Substitution	Nonsynonymous coding	44%
MM12T	PLIN5	perilipin 5	CCDS42473.1	chr19_4525766-4525766_C_T	200R>H	Substitution	Nonsynonymous coding	28%
MM12T	PLOD1	procollagen-lysine, 2-oxoglutarate 5-dioxygenase 1	CCDS142.1	chr1_12030833-12030833_C_T	621A>V	Substitution	Nonsynonymous coding	32%
MM12T	PLOD3	procollagen-lysine, 2-oxoglutarate 5-dioxygenase 3	CCDS5715.1	chr7_100860495-100860495_C_T	21A>T	Substitution	Nonsynonymous coding	33%
MM12T	PLRG1	pleiotropic regulator 1	CCDS34083.1	chr4_155470090-155470090__A	NA	Insertion	Splice site acceptor	31%
MM12T	PLTP	phospholipid transfer protein	CCDS13386.1	chr20_44533446-44533446_A_G	312V>A	Substitution	Nonsynonymous coding	33%
MM12T	PLTP	phospholipid transfer protein	CCDS13386.1	chr20_44538288-44538288_C_T	118A>T	Substitution	Nonsynonymous coding	27%
MM12T	PLXNA2	plexin A2	CCDS31013.1	chr1_208202377-208202377_G_A	1746R>C	Substitution	Nonsynonymous coding	26%
MM12T	PLXNA4	plexin A4	CCDS43646.1	chr7_132192323-132192323_C_T	377G>D	Substitution	Nonsynonymous coding	12%
MM12T	PLXNB1	plexin B1	CCDS2765.1	chr3_48461099-48461099_A_G	866S>P	Substitution	Nonsynonymous coding	27%
MM12T	PLXNC1	plexin C1	CCDS9049.1	chr12_94694746-94694746_G_T	1433K>N	Substitution	Nonsynonymous coding	14%
MM12T	PLXND1	plexin D1	CCDS33854.1	chr3_129281680-129281680_C_T	1592C>Y	Substitution	Nonsynonymous coding	10%
MM12T	PMCH	pro-melanin-concentrating hormone	CCDS31885.1	chr12_102591318-102591318_C_A	77E>D	Substitution	Nonsynonymous coding	17%
MM12T	PML	promyelocytic leukemia	CCDS10255.1	chr15_74315681-74315681_G_A	372R>H	Substitution	Nonsynonymous coding	37%
MM12T	PML	promyelocytic leukemia	CCDS10255.1	chr15_74325615-74325615_C_T	506P>L	Substitution	Nonsynonymous coding	25%
MM12T	PNCK	pregnancy up-regulated non-ubiquitously expressed CaM kinase	ENST00000438984	chrX_152935298-152935298_G_A	73P>L	Substitution	Nonsynonymous coding	15%
MM12T	PNCK	pregnancy up-regulated non-ubiquitously expressed CaM kinase	CCDS35503.2	chrX_152939452-152939452_G_A	60A>V	Substitution	Nonsynonymous coding	34%
MM12T	PNISR	PNN-interacting serine/arginine-rich protein	CCDS5043.1	chr6_99858807-99858807_G_	NA	Deletion	Frameshift	16%
MM12T	PNISR	PNN-interacting serine/arginine-rich protein	CCDS5043.1	chr6_99862455-99862455_C_A	27Q>H	Substitution	Nonsynonymous coding	27%
MM12T	PNLDC1	poly(A)-specific ribonuclease (PARN)-like domain containing 1	CCDS5271.1	chr6_160222182-160222182_C_T	47R>C	Substitution	Nonsynonymous coding	20%
MM12T	PNLDC1	poly(A)-specific ribonuclease (PARN)-like domain containing 1	CCDS5271.1	chr6_160240290-160240290_C_T	469R>W	Substitution	Nonsynonymous coding	20%
MM12T	PNLDC1	poly(A)-specific ribonuclease (PARN)-like domain containing 1	CCDS5271.1	chr6_160240304-160240304_G_T	473K>N	Substitution	Nonsynonymous coding	25%
MM12T	PNLIP	pancreatic lipase	CCDS7594.1	chr10_118313341-118313341_C_T	188R>C	Substitution	Nonsynonymous coding	38%

MM12T	PNMAL1	paraneoplastic Ma antigen family-like 1	CCDS33059.1	chr19_46973155-46973155_C_T	380E>K	Substitution	Nonsynonymous coding	32%
MM12T	PNN	pinin, desmosome associated protein	CCDS9671.1	chr14_39647101-39647101_C_T	160S>F	Substitution	Nonsynonymous coding	17%
MM12T	PNN	pinin, desmosome associated protein	CCDS9671.1	chr14_39649827-39649827_C_T	305A>V	Substitution	Nonsynonymous coding	13%
MM12T	PNN	pinin, desmosome associated protein	CCDS9671.1	chr14_39650210-39650210_A_T	433I>F	Substitution	Nonsynonymous coding	31%
MM12T	PNPLA4	patatin-like phospholipase domain containing 4	CCDS14129.1	chrX_7870046-7870046_G_A	205A>V	Substitution	Nonsynonymous coding	12%
MM12T	PNPLA6	patatin-like phospholipase domain containing 6	CCDS32891.1	chr19_7625936-7625936_G_A	1247A>T	Substitution	Nonsynonymous coding	29%
MM12T	PNPLA6	patatin-like phospholipase domain containing 6	CCDS32891.1	chr19_7606406-7606406_A_G	ISV-2>	Substitution	Splice site acceptor	27%
MM12T	PNPLA7	patatin-like phospholipase domain containing 7	CCDS48070.1	chr9_140435178-140435178_C_T	ISV-4>	Substitution	Splice site acceptor	11%
MM12T	PNPO	pyridoxamine 5'-phosphate oxidase	CCDS11522.1	chr17_46019133-46019133_C_T	31A>V	Substitution	Nonsynonymous coding	41%
MM12T	PNPT1	polyribonucleotide nucleotidyltransferase 1	CCDS1856.1	chr2_55883510-55883511_AA_	NA	Deletion	Splice site acceptor	10%
MM12T	PODXL2	podocalyxin-like 2	CCDS3044.1	chr3_127390401-127390401_C_T	517A>V	Substitution	Nonsynonymous coding	31%
MM12T	POGLUT1	protein O-glucosyltransferase 1	CCDS2988.1	chr3_119209414-119209414_C_T	272R>X	Substitution	Nonsense	11%
MM12T	POLD1	polymerase (DNA directed), delta 1, catalytic subunit	CCDS12795.1	chr19_50912075-50912075_C_A	603F>L	Substitution	Nonsynonymous coding	15%
MM12T	POLE	polymerase (DNA directed), epsilon, catalytic subunit	CCDS9278.1	chr12_133219168-133219168_G_A	1626R>C	Substitution	Nonsynonymous coding	10%
MM12T	POLE	polymerase (DNA directed), epsilon, catalytic subunit	CCDS9278.1	chr12_133249851-133249851_A_G	458Y>H	Substitution	Nonsynonymous coding	31%
MM12T	POLE	polymerase (DNA directed), epsilon, catalytic subunit	CCDS9278.1	chr12_133249853-133249853_G_A	457T>M	Substitution	Nonsynonymous coding	32%
MM12T	POLE2	polymerase (DNA directed), epsilon 2, accessory subunit	CCDS32073.1	chr14_50122403-50122403_C_T	305R>H	Substitution	Nonsynonymous coding	31%
MM12T	POLQ	polymerase (DNA directed), theta	CCDS33833.1	chr3_121154982-121154982_T_G	2510Q>H	Substitution	Nonsynonymous coding	15%
MM12T	POLR1A	polymerase (RNA) I polypeptide A, 194kDa	CCDS42706.1	chr2_86269168-86269168_G_A	1139A>V	Substitution	Nonsynonymous coding	17%
MM12T	POLR1A	polymerase (RNA) I polypeptide A, 194kDa	CCDS42706.1	chr2_86305292-86305292_A_G	401V>A	Substitution	Nonsynonymous coding	12%
MM12T	POLR1E	polymerase (RNA) I polypeptide E, 53kDa	CCDS6611.1	chr9_37495252-37495252_G_A	212D>N	Substitution	Nonsynonymous coding	11%
MM12T	POLR2A	polymerase (RNA) II (DNA directed) polypeptide A, 220kDa	CCDS32548.1	chr17_7407038-7407038_G_T	1056E>D	Substitution	Nonsynonymous coding	33%
MM12T	POLR3B	polymerase (RNA) III (DNA directed) polypeptide B	CCDS9105.1	chr12_106895160-106895160_T_C	1015M>T	Substitution	Nonsynonymous coding	30%
MM12T	POLR3B	polymerase (RNA) III (DNA directed) polypeptide B	CCDS9105.1	chr12_106799668-106799668_C_T	294Q>X	Substitution	Nonsense	12%
MM12T	POLR3E	polymerase (RNA) III (DNA directed) polypeptide E (80kD)	CCDS10605.1	chr16_22326450-22326450_G_A	188R>H	Substitution	Nonsynonymous coding	36%
MM12T	POLR3E	polymerase (RNA) III (DNA directed) polypeptide E (80kD)	CCDS10605.1	chr16_22335966-22335966_C_T	424T>M	Substitution	Nonsynonymous coding	30%
MM12T	POLRMT	polymerase (RNA) mitochondrial (DNA directed)	CCDS12036.1	chr19_620423-620423_G_A	902A>V	Substitution	Nonsynonymous coding	32%
MM12T	POM121	POM121 transmembrane nucleoporin	ENST00000434423	chr7_72396233-72396233_C_T	192H>Y	Substitution	Nonsynonymous coding	41%
MM12T	POM121L2	POM121 transmembrane nucleoporin-like 2	NM_033482	chr6_27276881-27276881_C_A	1023E>D	Substitution	Nonsynonymous coding	21%
MM12T	POM121L2	POM121 transmembrane nucleoporin-like 2	NM_033482	chr6_27277699-27277699_C_A	751A>S	Substitution	Nonsynonymous coding	22%
MM12T	POMC	proopiomelanocortin	CCDS1717.1	chr2_25384390-25384390_C_T	122E>K	Substitution	Nonsynonymous coding	36%
MM12T	POMGNT1	protein O-linked mannose beta1,2-N-acetylglucosaminyltransferase	CCDS531.1	chr1_46657799-46657799_C_T	504V>I	Substitution	Nonsynonymous coding	38%

MM12T	POMT1	protein-O-mannosyltransferase 1	CCDS6943.1	chr9_134382806-134382806_C_T	111A>V	Substitution	Nonsynonymous coding	25%
MM12T	POU1F1	POU class 1 homeobox 1	CCDS46873.1	chr3_87325600-87325600_C_T	5A>T	Substitution	Nonsynonymous coding	13%
MM12T	POU3F4	POU class 3 homeobox 4	CCDS14450.1	chrX_82764233-82764233_C_A	301P>T	Substitution	Nonsynonymous coding	34%
MM12T	POU5F1	POU class 5 homeobox 1	CCDS34391.1	chr6_31133064-31133064_C_A	ISV-1>	Substitution	Splice site acceptor	24%
MM12T	POU5F2	POU domain class 5, transcription factor 2	NM_153216	chr5_93076531-93076531_G_T	247L>I	Substitution	Nonsynonymous coding	31%
MM12T	POU6F2	POU class 6 homeobox 2	CCDS34620.2	chr7_39379582-39379582_C_T	285P>S	Substitution	Nonsynonymous coding	14%
MM12T	PPAN-P2RY11	PPAN-P2RY11 readthrough	CCDS42498.1	chr19_10225311-10225311_G_T	761S>I	Substitution	Nonsynonymous coding	12%
MM12T	PPAP2A	phosphatidic acid phosphatase type 2A	CCDS34160.1	chr5_54786907-54786907_T_G	32I>L	Substitution	Nonsynonymous coding	28%
MM12T	PPEF1	protein phosphatase, EF-hand calcium binding domain 1	CCDS14188.1	chrX_18748305-18748305_G_A	18R>K	Substitution	Nonsynonymous coding	26%
MM12T	PPFIA4	protein tyrosine phosphatase, receptor type, f polypeptide (PTPRF), interacting protein	ENST00000367238	chr1_203013227-203013227_C_T	ISV+4>	Substitution	Splice site donor	30%
MM12T	PPFIA4	protein tyrosine phosphatase, receptor type, f polypeptide (PTPRF), interacting protein	ENST00000367238	chr1_203017801-203017801_C_T	439Q>X	Substitution	Nonsense	27%
MM12T	PPFIBP2	PTPRF interacting protein, binding protein 2 (liprin beta 2)	CCDS31419.1	chr11_7570710-7570710_G_A	2A>T	Substitution	Nonsynonymous coding	13%
MM12T	PPIE	peptidylprolyl isomerase E (cyclophilin E)	CCDS443.1	chr1_40214689-40214689_G_A	208G>D	Substitution	Nonsynonymous coding	12%
MM12T	PPIL2	peptidylprolyl isomerase (cyclophilin)-like 2	CCDS46670.1	chr22_22035630-22035630_A_G	113N>S	Substitution	Nonsynonymous coding	23%
MM12T	PPL	periplakin	CCDS10526.1	chr16_4945721-4945721__G	NA	Insertion	Splice site acceptor	31%
MM12T	PPM1A	protein phosphatase, Mg2+/Mn2+ dependent, 1A	CCDS45120.1	chr14_60749518-60749518_C_T	106R>C	Substitution	Nonsynonymous coding	30%
MM12T	PPM1F	protein phosphatase, Mg2+/Mn2+ dependent, 1F	CCDS13796.1	chr22_22300290-22300290_G_A	44A>V	Substitution	Nonsynonymous coding	35%
MM12T	PPM1J	protein phosphatase, Mg2+/Mn2+ dependent, 1J	CCDS855.2	chr1_113253892-113253892_C_T	348G>S	Substitution	Nonsynonymous coding	17%
MM12T	PPP1R12C	protein phosphatase 1, regulatory subunit 12C	CCDS12916.1	chr19_55604185-55604185_C_T	626R>H	Substitution	Nonsynonymous coding	32%
MM12T	PPP1R13L	protein phosphatase 1, regulatory subunit 13 like	CCDS33050.1	chr19_45885868-45885868_G_A	789R>W	Substitution	Nonsynonymous coding	32%
MM12T	PPP1R13L	protein phosphatase 1, regulatory subunit 13 like	CCDS33050.1	chr19_45899018-45899018_G_A	344R>C	Substitution	Nonsynonymous coding	33%
MM12T	PPP1R13L	protein phosphatase 1, regulatory subunit 13 like	CCDS33050.1	chr19_45901357-45901357_G_A	35A>V	Substitution	Nonsynonymous coding	35%
MM12T	PPP1R15B	protein phosphatase 1, regulatory subunit 15B	CCDS1445.1	chr1_204379243-204379243_C_T	433A>T	Substitution	Nonsynonymous coding	29%
MM12T	PPP1R16B	protein phosphatase 1, regulatory subunit 16B	CCDS13309.1	chr20_37464809-37464809_G_A	81A>T	Substitution	Nonsynonymous coding	28%
MM12T	PPP1R16B	protein phosphatase 1, regulatory subunit 16B	CCDS13309.1	chr20_37546854-37546854_C_T	417R>X	Substitution	Nonsense	39%
MM12T	PPP1R3B	protein phosphatase 1, regulatory subunit 3B	CCDS5973.1	chr8_8998755-8998755_T_A	136E>V	Substitution	Nonsynonymous coding	14%
MM12T	PPP1R3F	protein phosphatase 1, regulatory subunit 3F	CCDS35254.1	chrX_49143400-49143400_G_A	750E>K	Substitution	Nonsynonymous coding	11%
MM12T	PPP1R7	protein phosphatase 1, regulatory subunit 7	CCDS2546.1	chr2_242097921-242097921_G_A	87R>H	Substitution	Nonsynonymous coding	39%
MM12T	PPP1R9B	protein phosphatase 1, regulatory subunit 9B	ENST00000316878	chr17_48213382-48213382_C_T	750E>K	Substitution	Nonsynonymous coding	11%
MM12T	PPP2R5B	protein phosphatase 2, regulatory subunit B', beta	CCDS8085.1	chr11_64695559-64695559_C_T	174R>C	Substitution	Nonsynonymous coding	29%
MM12T	PPP2R5E	protein phosphatase 2, regulatory subunit B', epsilon isoform	CCDS9758.1	chr14_63920453-63920453_C_A	103S>I	Substitution	Nonsynonymous coding	21%
MM12T	PPP4R1L	protein phosphatase 4, regulatory subunit 1-like	ENST00000334187	chr20_56826875-56826875_G_A	161R>C	Substitution	Nonsynonymous coding	26%

MM12T	PPP4R4	protein phosphatase 4, regulatory subunit 4	CCDS9921.1	chr14_94712745-94712745_G_A	494A>T	Substitution	Nonsynonymous coding	15%
MM12T	PPP6C	protein phosphatase 6, catalytic subunit	CCDS48018.1	chr9_127920643-127920643_C_A	123G>C	Substitution	Nonsynonymous coding	31%
MM12T	PPP6R1	protein phosphatase 6, regulatory subunit 1	CCDS46186.1	chr19_55750846-55750846_G_A	560R>C	Substitution	Nonsynonymous coding	29%
MM12T	PPP6R3	protein phosphatase 6, regulatory subunit 3	CCDS8182.1	chr11_68355426-68355426_C_T	475T>M	Substitution	Nonsynonymous coding	18%
MM12T	PPP6R3	protein phosphatase 6, regulatory subunit 3	CCDS8182.1	chr11_68377458-68377458_C_T	766A>V	Substitution	Nonsynonymous coding	31%
MM12T	PPRC1	peroxisome proliferator-activated receptor gamma, coactivator-related 1	CCDS7529.1	chr10_103906723-103906723_G_A	1325R>H	Substitution	Nonsynonymous coding	14%
MM12T	PQBP1	polyglutamine binding protein 1	CCDS14309.1	chrX_48759573-48759573_G_A	119R>H	Substitution	Nonsynonymous coding	27%
MM12T	PRAF2	PRA1 domain family, member 2	CCDS14317.1	chrX_48931573-48931573_G_A	25P>L	Substitution	Nonsynonymous coding	12%
MM12T	PRDM11	PR domain containing 11	CCDS7912.1	chr11_45204460-45204460_G_A	125G>D	Substitution	Nonsynonymous coding	15%
MM12T	PRDM16	PR domain containing 16	CCDS41236.1	chr1_3328479-3328479_C_T	573A>V	Substitution	Nonsynonymous coding	29%
MM12T	PRDM6	PR domain containing 6	CCDS47259.1	chr5_122522836-122522836_C_T	577R>W	Substitution	Nonsynonymous coding	12%
MM12T	PRDM9	PR domain containing 9	CCDS43307.1	chr5_23510140-23510140_A_C	ISV+4>	Substitution	Splice site donor	31%
MM12T	PRDX4	peroxiredoxin 4	ENST00000418872	chrX_23682667-23682667_G_T	11G>C	Substitution	Nonsynonymous coding	27%
MM12T	PREB	prolactin regulatory element binding	CCDS1738.1	chr2_27355721-27355721_G_T	206P>H	Substitution	Nonsynonymous coding	15%
MM12T	PREP	prolyl endopeptidase	CCDS5053.1	chr6_105726048-105726048_G_A	702R>W	Substitution	Nonsynonymous coding	21%
MM12T	PREX2	phosphatidylinositol-3,4,5-trisphosphate-dependent Rac exchange factor 2	CCDS6201.1	chr8_69046462-69046462_C_T	1312A>V	Substitution	Nonsynonymous coding	42%
MM12T	PRG3	proteoglycan 3	CCDS7954.1	chr11_57147239-57147239_C_T	35D>N	Substitution	Nonsynonymous coding	15%
MM12T	PRH1	proline-rich protein HaeIII subfamily 1	NM_006250	chr12_11034896-11034896_G_A	147Q>X	Substitution	Nonsense	19%
MM12T	PRICKLE1	prickle homolog 1 (Drosophila)	CCDS8742.1	chr12_42858833-42858833_G_A	335R>X	Substitution	Nonsense	15%
MM12T	PRKAG1	protein kinase, AMP-activated, gamma 1 non-catalytic subunit	CCDS8777.1	chr12_49399094-49399094_C_T	102A>T	Substitution	Nonsynonymous coding	37%
MM12T	PRKAG2	protein kinase, AMP-activated, gamma 2 non-catalytic subunit	CCDS5928.1	chr7_151372621-151372621_C_T	190R>H	Substitution	Nonsynonymous coding	25%
MM12T	PRKCQ	protein kinase C, theta	CCDS7079.1	chr10_6498668-6498668_A_T	539F>I	Substitution	Nonsynonymous coding	36%
MM12T	PRKCQ	protein kinase C, theta	CCDS7079.1	chr10_6525416-6525416_C_A	389G>X	Substitution	Nonsense	30%
MM12T	PRKD2	protein kinase D2	CCDS12689.1	chr19_47217236-47217236_C_T	88G>D	Substitution	Nonsynonymous coding	13%
MM12T	PRKDC	protein kinase, DNA-activated, catalytic polypeptide	ENST00000314191	chr8_48690359-48690359_G_A	3976T>M	Substitution	Nonsynonymous coding	26%
MM12T	PRKDC	protein kinase, DNA-activated, catalytic polypeptide	ENST00000314191	chr8_48736460-48736460_C_A	2960A>S	Substitution	Nonsynonymous coding	32%
MM12T	PRKDC	protein kinase, DNA-activated, catalytic polypeptide	ENST00000314191	chr8_48855909-48855909_C_T	276A>T	Substitution	Nonsynonymous coding	14%
MM12T	PRKDC	protein kinase, DNA-activated, catalytic polypeptide	ENST00000314191	chr8_48869984-48869984_C_A	54Q>H	Substitution	Nonsynonymous coding	32%
MM12T	PRKDC	protein kinase, DNA-activated, catalytic polypeptide	ENST00000314191	chr8_48691288-48691288_C_T	ISV+1>	Substitution	Splice site donor	14%
MM12T	PRL	prolactin	CCDS4548.1	chr6_22292835-22292835_C_T	82A>T	Substitution	Nonsynonymous coding	21%
MM12T	PRMT2	protein arginine methyltransferase 2	CCDS13737.1	chr21_48081814-48081814_C_A	355P>T	Substitution	Nonsynonymous coding	31%
MM12T	PRMT8	protein arginine methyltransferase 8	CCDS8521.2	chr12_3649841-3649841_G_A	49V>M	Substitution	Nonsynonymous coding	27%

MM12T	PRND	prion protein 2 (dublet)	CCDS13081.1	chr20_4705388-4705388_G_A	64R>H	Substitution	Nonsynonymous coding	11%
MM12T	PROC	protein C (inactivator of coagulation factors Va and VIIIa)	CCDS2145.1	chr2_128186299-128186299_C_T	388A>V	Substitution	Nonsynonymous coding	11%
MM12T	PRODH	proline dehydrogenase (oxidase) 1	CCDS13754.1	chr22_18912662-18912662_C_T	190R>H	Substitution	Nonsynonymous coding	12%
MM12T	PROSER1	proline and serine rich 1	CCDS9368.2	chr13_39588550-39588550_G_A	280A>V	Substitution	Nonsynonymous coding	11%
MM12T	PROX2	prospero homeobox 2	CCDS45136.1	chr14_75330186-75330186_C_A	118A>S	Substitution	Nonsynonymous coding	18%
MM12T	PRPF18	PRP18 pre-mRNA processing factor 18 homolog (S. cerevisiae)	CCDS7100.1	chr10_13642268-13642268_A_T	57K>X	Substitution	Nonsense	28%
MM12T	PRPF38B	PRP38 pre-mRNA processing factor 38 (yeast) domain containing B	CCDS788.1	chr1_109235421-109235421_C_T	70P>S	Substitution	Nonsynonymous coding	32%
MM12T	PRPF40B	PRP40 pre-mRNA processing factor 40 homolog B (S. cerevisiae)	CCDS31796.1	chr12_50035736-50035736_G_A	583A>T	Substitution	Nonsynonymous coding	11%
MM12T	PRPF8	PRP8 pre-mRNA processing factor 8 homolog (S. cerevisiae)	CCDS11010.1	chr17_1562055-1562055_G_A	1714A>V	Substitution	Nonsynonymous coding	10%
MM12T	PRPF8	PRP8 pre-mRNA processing factor 8 homolog (S. cerevisiae)	CCDS11010.1	chr17_1582684-1582684_G_A	437A>V	Substitution	Nonsynonymous coding	23%
MM12T	PRPF8	PRP8 pre-mRNA processing factor 8 homolog (S. cerevisiae)	CCDS11010.1	chr17_1587790-1587790_A_G	26S>P	Substitution	Nonsynonymous coding	21%
MM12T	PRR12	proline rich 12	CCDS46143.1	chr19_50098878-50098878_C_A	429T>N	Substitution	Nonsynonymous coding	11%
MM12T	PRR12	proline rich 12	CCDS46143.1	chr19_50099792-50099792_C_T	734R>W	Substitution	Nonsynonymous coding	16%
MM12T	PRR12	proline rich 12	CCDS46143.1	chr19_50100809-50100809_C_T	1073L>F	Substitution	Nonsynonymous coding	33%
MM12T	PRR14L	proline rich 14-like	CCDS13900.2	chr22_32134581-32134581_G_T	89P>H	Substitution	Nonsynonymous coding	25%
MM12T	PRR3	proline rich 3	CCDS43440.1	chr6_30525192-30525192_A_G	24E>G	Substitution	Nonsynonymous coding	23%
MM12T	PRR5	proline rich 5 (renal)	CCDS14058.1	chr22_45110503-45110503_G_A	56R>H	Substitution	Nonsynonymous coding	22%
MM12T	PRRC2A	proline-rich coiled-coil 2A	CCDS4708.1	chr6_31600342-31600342_G_T	1298A>S	Substitution	Nonsynonymous coding	25%
MM12T	PRRC2A	proline-rich coiled-coil 2A	CCDS4708.1	chr6_31602070-31602070_G_T	1593G>C	Substitution	Nonsynonymous coding	21%
MM12T	PRRC2B	proline-rich coiled-coil 2B	CCDS48044.1	chr9_134314436-134314436_G_T	153G>X	Substitution	Nonsense	17%
MM12T	PRRG3	proline rich Gla (G-carboxyglutamic acid) 3 (transmembrane)	CCDS14699.1	chrX_150868547-150868547_G_T	29Q>H	Substitution	Nonsynonymous coding	28%
MM12T	PRRT4	proline-rich transmembrane protein 4	CCDS47698.1	chr7_127999913-127999913_C_T	45A>T	Substitution	Nonsynonymous coding	13%
MM12T	PRSS22	protease, serine, 22	CCDS10481.1	chr16_2906837-2906837_G_A	32A>V	Substitution	Nonsynonymous coding	14%
MM12T	PRSS23	protease, serine, 23	CCDS8278.1	chr11_86519669-86519669_G_T	328Q>H	Substitution	Nonsynonymous coding	31%
MM12T	PRSS36	protease, serine, 36	CCDS32436.1	chr16_31154285-31154285_T_C	377D>G	Substitution	Nonsynonymous coding	32%
MM12T	PRSS37	protease, serine, 37	CCDS34764.1	chr7_141536278-141536278_G_A	209H>Y	Substitution	Nonsynonymous coding	17%
MM12T	PRSS38	protease, serine, 38	CCDS1563.1	chr1_228003563-228003563_T_C	49V>A	Substitution	Nonsynonymous coding	29%
MM12T	PRSS45	protease, serine, 45	CCDS46815.1	chr3_46785536-46785536_G_A	70H>Y	Substitution	Nonsynonymous coding	16%
MM12T	PRSS58	protease, serine, 58	CCDS5871.1	chr7_141952159-141952159_C_T	203G>E	Substitution	Nonsynonymous coding	15%
MM12T	PRTG	protogenin	CCDS42040.1	chr15_55912281-55912281_G_A	1128R>W	Substitution	Nonsynonymous coding	29%
MM12T	PRTG	protogenin	CCDS42040.1	chr15_55965835-55965835_C_A	529S>I	Substitution	Nonsynonymous coding	26%
MM12T	PRUNE2	prune homolog 2 (Drosophila)	CCDS47982.1	chr9_79318626-79318626_A_C	2635F>V	Substitution	Nonsynonymous coding	27%

MM12T	PRX	periaxin	CCDS33028.1	chr19_40903609-40903609_G_A	217A>V	Substitution	Nonsynonymous coding	30%
MM12T	PSAP	prosaposin	CCDS7311.1	chr10_73579489-73579489_G_A	392R>W	Substitution	Nonsynonymous coding	14%
MM12T	PSAP	prosaposin	CCDS7311.1	chr10_73579554-73579554_G_T	370S>Y	Substitution	Nonsynonymous coding	15%
MM12T	PSD2	pleckstrin and Sec7 domain containing 2	CCDS4216.1	chr5_139216791-139216791_G_A	545V>M	Substitution	Nonsynonymous coding	21%
MM12T	PSD3	pleckstrin and Sec7 domain containing 3	CCDS43720.1	chr8_18393443-18393443_C_A	985S>I	Substitution	Nonsynonymous coding	30%
MM12T	PSD4	pleckstrin and Sec7 domain containing 4	CCDS33276.1	chr2_113943833-113943833_G_A	ISV+1>	Substitution	Splice site donor	11%
MM12T	PSKH1	protein serine kinase H1	CCDS10851.1	chr16_67942674-67942674_G_A	8V>I	Substitution	Nonsynonymous coding	30%
MM12T	PSKH2	protein serine kinase H2	CCDS6240.1	chr8_87081839-87081839_C_T	5A>T	Substitution	Nonsynonymous coding	12%
MM12T	PSMB11	proteasome (prosome, macropain) subunit, beta type, 11	CCDS41923.1	chr14_23512147-23512147_G_A	238R>H	Substitution	Nonsynonymous coding	12%
MM12T	PSMB8	proteasome (prosome, macropain) subunit, beta type, 8 (large multifunctional peptidase 7)	CCDS4757.1	chr6_32811770-32811770_C_A	2A>S	Substitution	Nonsynonymous coding	17%
MM12T	PSMB9	proteasome (prosome, macropain) subunit, beta type, 9 (large multifunctional peptidase 2)	CCDS4759.1	chr6_32826229-32826229_C_T	160A>V	Substitution	Nonsynonymous coding	16%
MM12T	PSMC3	proteasome (prosome, macropain) 26S subunit, ATPase, 3	CCDS7935.1	chr11_47444216-47444216_C_T	265A>T	Substitution	Nonsynonymous coding	24%
MM12T	PSMD1	proteasome (prosome, macropain) 26S subunit, non-ATPase, 1	CCDS2482.1	chr2_231940259-231940259_G_A	306A>T	Substitution	Nonsynonymous coding	37%
MM12T	PSMD2	proteasome (prosome, macropain) 26S subunit, non-ATPase, 2	CCDS3258.1	chr3_184023669-184023669_G_T	551K>N	Substitution	Nonsynonymous coding	28%
MM12T	PSMD3	proteasome (prosome, macropain) 26S subunit, non-ATPase, 3	CCDS11356.1	chr17_38140676-38140676_T_C	117V>A	Substitution	Nonsynonymous coding	38%
MM12T	PSMD6	proteasome (prosome, macropain) 26S subunit, non-ATPase, 6	CCDS2901.1	chr3_64004517-64004517_C_T	232E>K	Substitution	Nonsynonymous coding	30%
MM12T	PSMD7	proteasome (prosome, macropain) 26S subunit, non-ATPase, 7	CCDS10910.1	chr16_74335485-74335485_G_T	98G>C	Substitution	Nonsynonymous coding	26%
MM12T	PSME4	proteasome (prosome, macropain) activator subunit 4	CCDS33197.2	chr2_54149665-54149665_G_A	679L>F	Substitution	Nonsynonymous coding	11%
MM12T	PSPN	persephin	CCDS12164.1	chr19_6375607-6375607_G_A	57R>C	Substitution	Nonsynonymous coding	24%
MM12T	PSTPIP2	proline-serine-threonine phosphatase interacting protein 2	CCDS32820.2	chr18_43577742-43577742_C_T	205W>X	Substitution	Nonsense	31%
MM12T	PTBP1	polypyrimidine tract binding protein 1	CCDS42456.1	chr19_806468-806468_C_T	344A>V	Substitution	Nonsynonymous coding	16%
MM12T	PTCH2	patched 2	CCDS516.1	chr1_45288829-45288829_G_A	1090A>V	Substitution	Nonsynonymous coding	31%
MM12T	PTCH2	patched 2	CCDS516.1	chr1_45293561-45293561_A_G	671F>S	Substitution	Nonsynonymous coding	35%
MM12T	PTCHD1	patched domain containing 1	CCDS35215.2	chrX_23353155-23353155_C_A	55L>M	Substitution	Nonsynonymous coding	32%
MM12T	PTCHD4	patched domain containing 4	NM_001013732	chr6_48036025-48036025_T_C	123N>D	Substitution	Nonsynonymous coding	10%
MM12T	PTDSS2	phosphatidylserine synthase 2	CCDS7696.1	chr11_460209-460209_C_A	69L>I	Substitution	Nonsynonymous coding	32%
MM12T	PTEN	phosphatase and tensin homolog	NM_000314	chr10_89720812-89720812__A	NA	Insertion	Frameshift	54%
MM12T	PTEN	phosphatase and tensin homolog	NM_000314	chr10_89692967-89692967_G_A	151A>T	Substitution	Nonsynonymous coding	35%
MM12T	PTEN	phosphatase and tensin homolog	NM_000314	chr10_89711900-89711900_G_A	173R>H	Substitution	Nonsynonymous coding	17%
MM12T	PTGDR2	prostaglandin D2 receptor 2	CCDS7994.1	chr11_60620204-60620204_C_A	331S>I	Substitution	Nonsynonymous coding	11%
MM12T	PTGDS	prostaglandin D2 synthase 21kDa (brain)	CCDS7019.1	chr9_139874679-139874679_G_A	165A>T	Substitution	Nonsynonymous coding	11%
MM12T	PTGER1	prostaglandin E receptor 1 (subtype EP1), 42kDa	CCDS12309.1	chr19_14584322-14584322_A_G	271S>P	Substitution	Nonsynonymous coding	36%

MM12T	PTGER4	prostaglandin E receptor 4 (subtype EP4)	CCDS3930.1	chr5_40691884-40691884_C_T	291R>X	Substitution	Nonsense	17%
MM12T	PTGFRN	prostaglandin F2 receptor inhibitor	CCDS890.1	chr1_117527352-117527352_G_A	740D>N	Substitution	Nonsynonymous coding	26%
MM12T	PTGFRN	prostaglandin F2 receptor inhibitor	CCDS890.1	chr1_117529431-117529431_G_A	828A>T	Substitution	Nonsynonymous coding	13%
MM12T	PTGIR	prostaglandin I2 (prostacyclin) receptor (IP)	CCDS12686.1	chr19_47126878-47126878_C_T	202C>Y	Substitution	Nonsynonymous coding	11%
MM12T	PTGIR	prostaglandin I2 (prostacyclin) receptor (IP)	CCDS12686.1	chr19_47127134-47127134_G_A	117R>C	Substitution	Nonsynonymous coding	29%
MM12T	PTGIS	prostaglandin I2 (prostacyclin) synthase	CCDS13419.1	chr20_48129637-48129637_C_A	396E>X	Substitution	Nonsense	11%
MM12T	PTGS1	prostaglandin-endoperoxide synthase 1 (prostaglandin G/H synthase and	CCDS6842.1	chr9_125154482-125154482_G_A	487A>T	Substitution	Nonsynonymous coding	16%
MM12T	PTH2R	parathyroid hormone 2 receptor	CCDS2383.1	chr2_209358273-209358273_G_T	514K>N	Substitution	Nonsynonymous coding	30%
MM12T	PTK2B	PTK2B protein tyrosine kinase 2 beta	CCDS6057.1	chr8_27292043-27292043_C_T	387R>W	Substitution	Nonsynonymous coding	27%
MM12T	PTK2B	PTK2B protein tyrosine kinase 2 beta	CCDS6057.1	chr8_27311740-27311740_C_T	889R>W	Substitution	Nonsynonymous coding	12%
MM12T	PTK7	PTK7 protein tyrosine kinase 7	CCDS4884.1	chr6_43097540-43097540_T_G	148L>R	Substitution	Nonsynonymous coding	28%
MM12T	PTPLA	protein tyrosine phosphatase-like (proline instead of catalytic arginine), member A	CCDS7121.1	chr10_17641349-17641349_C_T	182R>H	Substitution	Nonsynonymous coding	11%
MM12T	PTPN14	protein tyrosine phosphatase, non-receptor type 14	CCDS1514.1	chr1_214546057-214546057_C_A	1011E>D	Substitution	Nonsynonymous coding	43%
MM12T	PTPN14	protein tyrosine phosphatase, non-receptor type 14	CCDS1514.1	chr1_214638112-214638112_C_T	12R>H	Substitution	Nonsynonymous coding	30%
MM12T	PTPN21	protein tyrosine phosphatase, non-receptor type 21	CCDS9884.1	chr14_88946099-88946099_C_T	559R>Q	Substitution	Nonsynonymous coding	43%
MM12T	PTPN23	protein tyrosine phosphatase, non-receptor type 23	CCDS2754.1	chr3_47451731-47451731_G_A	815V>I	Substitution	Nonsynonymous coding	10%
MM12T	PTPN7	protein tyrosine phosphatase, non-receptor type 7	CCDS1422.1	chr1_202119449-202119449_G_A	366R>W	Substitution	Nonsynonymous coding	33%
MM12T	PTPN7	protein tyrosine phosphatase, non-receptor type 7	CCDS1422.1	chr1_202119484-202119484_C_T	354G>E	Substitution	Nonsynonymous coding	13%
MM12T	PTPRA	protein tyrosine phosphatase, receptor type, A	CCDS13038.1	chr20_3002824-3002824_C_T	429A>V	Substitution	Nonsynonymous coding	11%
MM12T	PTPRC	protein tyrosine phosphatase, receptor type, C	CCDS1397.1	chr1_198671606-198671606_C_A	175S>Y	Substitution	Nonsynonymous coding	14%
MM12T	PTPRG	protein tyrosine phosphatase, receptor type, G	CCDS2895.1	chr3_62204644-62204644_C_T	759L>F	Substitution	Nonsynonymous coding	11%
MM12T	PTPRJ	protein tyrosine phosphatase, receptor type, J	CCDS7945.1	chr11_48168450-48168450_T_G	978F>L	Substitution	Nonsynonymous coding	12%
MM12T	PTPRN	protein tyrosine phosphatase, receptor type, N	CCDS2440.1	chr2_220162785-220162785_C_A	570S>I	Substitution	Nonsynonymous coding	36%
MM12T	PTPRN2	protein tyrosine phosphatase, receptor type, N polypeptide 2	CCDS5947.1	chr7_157449122-157449122_C_T	708R>H	Substitution	Nonsynonymous coding	30%
MM12T	PTPRN2	protein tyrosine phosphatase, receptor type, N polypeptide 2	CCDS5947.1	chr7_157959919-157959919_G_A	205P>L	Substitution	Nonsynonymous coding	33%
MM12T	PTPRQ	protein tyrosine phosphatase, receptor type, Q	NM_001145026	chr12_81063246-81063246_A_	NA	Deletion	Splice site donor	19%
MM12T	PTPRT	protein tyrosine phosphatase, receptor type, T	CCDS42874.1	chr20_41419965-41419965_G_A	119A>V	Substitution	Nonsynonymous coding	29%
MM12T	PTPRU	protein tyrosine phosphatase, receptor type, U	CCDS334.1	chr1_29611366-29611366_T_C	768V>A	Substitution	Nonsynonymous coding	31%
MM12T	PTPRZ1	protein tyrosine phosphatase, receptor-type, Z polypeptide 1	CCDS34740.1	chr7_121653434-121653434_G_A	1445C>Y	Substitution	Nonsynonymous coding	34%
MM12T	PUS7	pseudouridylate synthase 7 homolog (S. cerevisiae)	CCDS34725.1	chr7_105105834-105105834_T_C	518D>G	Substitution	Nonsynonymous coding	33%
MM12T	PWP1	PWP1 homolog (S. cerevisiae)	CCDS9114.1	chr12_108086811-108086811_C_T	147P>L	Substitution	Nonsynonymous coding	22%
MM12T	PWP1	PWP1 homolog (S. cerevisiae)	CCDS9114.1	chr12_108105990-108105990_A_T	500E>V	Substitution	Nonsynonymous coding	32%

MM12T	PWP2	PWP2 periodic tryptophan protein homolog (yeast)	CCDS33579.1	chr21_45539340-45539340_G_T	375S>I	Substitution	Nonsynonymous coding	29%
MM12T	PWP2	PWP2 periodic tryptophan protein homolog (yeast)	CCDS33579.1	chr21_45550531-45550531_C_T	880R>W	Substitution	Nonsynonymous coding	14%
MM12T	PWWP2A	PWWP domain containing 2A	CCDS47332.1	chr5_159520116-159520116_C_T	514R>H	Substitution	Nonsynonymous coding	33%
MM12T	PWWP2A	PWWP domain containing 2A	CCDS47332.1	chr5_159520164-159520164_C_T	498R>Q	Substitution	Nonsynonymous coding	33%
MM12T	PWWP2A	PWWP domain containing 2A	CCDS47332.1	chr5_159520348-159520348_C_T	437E>K	Substitution	Nonsynonymous coding	16%
MM12T	PWWP2B	PWWP domain containing 2B	CCDS7667.2	chr10_134218518-134218518_C_T	172R>W	Substitution	Nonsynonymous coding	27%
MM12T	PXDN	peroxidasin homolog (Drosophila)	CCDS46221.1	chr2_1691440-1691440_C_A	127R>M	Substitution	Nonsynonymous coding	13%
MM12T	PXDNL	peroxidasin homolog (Drosophila)-like	CCDS47855.1	chr8_52321596-52321596_G_A	863A>V	Substitution	Nonsynonymous coding	32%
MM12T	PXMP2	peroxisomal membrane protein 2, 22kDa	CCDS9279.1	chr12_133277870-133277870__G	NA	Insertion	Frameshift	23%
MM12T	PXN	paxillin	CCDS44997.1	chr12_120662026-120662026_C_A	56E>D	Substitution	Nonsynonymous coding	38%
MM12T	PYGM	phosphorylase, glycogen, muscle	CCDS8079.1	chr11_64519094-64519094_G_T	601P>H	Substitution	Nonsynonymous coding	18%
MM12T	PYGM	phosphorylase, glycogen, muscle	CCDS8079.1	chr11_64521057-64521057_C_T	446C>Y	Substitution	Nonsynonymous coding	23%
MM12T	QPRT	quinolinate phosphoribosyltransferase	CCDS10651.1	chr16_29706503-29706503_G_A	178A>T	Substitution	Nonsynonymous coding	26%
MM12T	QRICH2	glutamine rich 2	CCDS32741.1	chr17_74288805-74288805_G_T	502P>H	Substitution	Nonsynonymous coding	27%
MM12T	QRICH2	glutamine rich 2	CCDS32741.1	chr17_74289918-74289918_C_A	131G>V	Substitution	Nonsynonymous coding	37%
MM12T	QTRT1	queuine tRNA-ribosyltransferase 1	CCDS12248.1	chr19_10812796-10812796_G_T	106S>I	Substitution	Nonsynonymous coding	28%
MM12T	RAB11FIP2	RAB11 family interacting protein 2 (class I)	CCDS7602.1	chr10_119799882-119799882_G_C	183T>R	Substitution	Nonsynonymous coding	31%
MM12T	RAB11FIP3	RAB11 family interacting protein 3 (class II)	CCDS32351.1	chr16_570201-570201_G_A	647S>N	Substitution	Nonsynonymous coding	13%
MM12T	RAB25	RAB25, member RAS oncogene family	CCDS41413.1	chr1_156038179-156038179_G_A	120V>I	Substitution	Nonsynonymous coding	29%
MM12T	RAB33A	RAB33A, member RAS oncogene family	CCDS14621.1	chrX_129306160-129306160_A_G	42I>V	Substitution	Nonsynonymous coding	13%
MM12T	RAB35	RAB35, member RAS oncogene family	CCDS41846.1	chr12_120541733-120541733_C_T	42G>R	Substitution	Nonsynonymous coding	29%
MM12T	RAB35	RAB35, member RAS oncogene family	CCDS41846.1	chr12_120546239-120546239_C_T	29A>T	Substitution	Nonsynonymous coding	28%
MM12T	RAB3C	RAB3C, member RAS oncogene family	CCDS3976.1	chr5_58120900-58120900_C_T	136A>V	Substitution	Nonsynonymous coding	33%
MM12T	RAB3GAP1	RAB3 GTPase activating protein subunit 1 (catalytic)	CCDS33294.1	chr2_135870731-135870731_C_T	125R>C	Substitution	Nonsynonymous coding	21%
MM12T	RAB40C	RAB40C, member RAS oncogene family	CCDS10413.1	chr16_667204-667204_G_A	ISV-4>	Substitution	Splice site acceptor	34%
MM12T	RAB41	RAB41, member RAS oncogene family	CCDS35322.1	chrX_69503127-69503127_C_T	83Q>X	Substitution	Nonsense	29%
MM12T	RAB4B-EGLN2	RAB4B-EGLN2 readthrough (non-protein coding)	CCDS12567.1	chr19_41306488-41306488_C_T	4P>L	Substitution	Nonsynonymous coding	43%
MM12T	RAB4B-EGLN2	RAB4B-EGLN2 readthrough (non-protein coding)	CCDS12567.1	chr19_41306941-41306941_G_A	155S>N	Substitution	Nonsynonymous coding	17%
MM12T	RABEP2	rabaptin, RAB GTPase binding effector protein 2	CCDS42140.1	chr16_28935906-28935906_G_A	31A>V	Substitution	Nonsynonymous coding	11%
MM12T	RABEP2	rabaptin, RAB GTPase binding effector protein 2	CCDS42140.1	chr16_28936420-28936420_G_A	ISV+4>	Substitution	Splice site donor	33%
MM12T	RABL5	RAB, member RAS oncogene family-like 5	CCDS5719.1	chr7_100961403-100961403_A_G	ISV+2>	Substitution	Splice site donor	11%
MM12T	RABL6	RAB, member RAS oncogene family-like 6	CCDS48058.1	chr9_139731926-139731926_G_A	313S>N	Substitution	Nonsynonymous coding	16%

MM12T	RABL6	RAB, member RAS oncogene family-like 6	CCDS48058.1	chr9_139734924-139734924_G_A	714G>R	Substitution	Nonsynonymous coding	42%
MM12T	RAC2	ras-related C3 botulinum toxin substrate 2 (rho family, small GTP binding protein Rac2)	CCDS13945.1	chr22_37628873-37628873_C_T	65D>N	Substitution	Nonsynonymous coding	10%
MM12T	RACGAP1	Rac GTPase activating protein 1	CCDS8795.1	chr12_50396060-50396060_C_A	173K>N	Substitution	Nonsynonymous coding	35%
MM12T	RAD51C	RAD51 homolog C (S. cerevisiae)	CCDS11611.1	chr17_56774103-56774103_G_A	152A>T	Substitution	Nonsynonymous coding	26%
MM12T	RADIL	Ras association and DIL domains	CCDS43544.1	chr7_4839327-4839327_C_T	1019R>H	Substitution	Nonsynonymous coding	31%
MM12T	RAE1	RAE1 RNA export 1 homolog (S. pombe)	CCDS13458.1	chr20_55940486-55940486_A_G	121I>M	Substitution	Nonsynonymous coding	20%
MM12T	RAET1G	retinoic acid early transcript 1G	CCDS43514.1	chr6_150239437-150239437_G_A	239L>F	Substitution	Nonsynonymous coding	10%
MM12T	RAG1	recombination activating gene 1	CCDS7902.1	chr11_36596320-36596320_G_A	489R>K	Substitution	Nonsynonymous coding	14%
MM12T	RAI1	retinoic acid induced 1	CCDS11188.1	chr17_17697290-17697290_G_A	343S>N	Substitution	Nonsynonymous coding	19%
MM12T	RAI1	retinoic acid induced 1	CCDS11188.1	chr17_17699740-17699740_C_T	1160R>W	Substitution	Nonsynonymous coding	32%
MM12T	RAI1	retinoic acid induced 1	CCDS11188.1	chr17_17701670-17701670_G_A	1803R>H	Substitution	Nonsynonymous coding	21%
MM12T	RAI2	retinoic acid induced 2	CCDS14183.1	chrX_17819214-17819214_G_A	306T>M	Substitution	Nonsynonymous coding	29%
MM12T	RALB	v-ral simian leukemia viral oncogene homolog B (ras related; GTP binding protein)	CCDS2131.1	chr2_121047263-121047263_G_T	144R>M	Substitution	Nonsynonymous coding	30%
MM12T	RALGAPA2	Ral GTPase activating protein, alpha subunit 2 (catalytic)	CCDS46584.1	chr20_20565513-20565513_C_A	842Q>H	Substitution	Nonsynonymous coding	24%
MM12T	RALGAPA2	Ral GTPase activating protein, alpha subunit 2 (catalytic)	CCDS46584.1	chr20_20601192-20601192_G_T	439P>H	Substitution	Nonsynonymous coding	25%
MM12T	RALGDS	ral guanine nucleotide dissociation stimulator	CCDS6959.1	chr9_135981407-135981407_T_G	ISV-4>	Substitution	Splice site acceptor	26%
MM12T	RANBP10	RAN binding protein 10	CCDS32469.1	chr16_67763727-67763727_G_A	335R>W	Substitution	Nonsynonymous coding	26%
MM12T	RANBP10	RAN binding protein 10	CCDS32469.1	chr16_67763844-67763844_A_G	332L>P	Substitution	Nonsynonymous coding	30%
MM12T	RANBP6	RAN binding protein 6	CCDS6467.1	chr9_6014757-6014757_C_T	284R>H	Substitution	Nonsynonymous coding	33%
MM12T	RAPGEF3	Rap guanine nucleotide exchange factor (GEF) 3	CCDS41775.1	chr12_48131828-48131828_C_A	877S>I	Substitution	Nonsynonymous coding	20%
MM12T	RARA	retinoic acid receptor, alpha	CCDS11366.1	chr17_38508601-38508601_C_T	217R>C	Substitution	Nonsynonymous coding	16%
MM12T	RARA	retinoic acid receptor, alpha	ENST00000319149	chr17_38512928-38512928_G_A	605R>Q	Substitution	Nonsynonymous coding	28%
MM12T	RARG	retinoic acid receptor, gamma	CCDS8850.1	chr12_53621312-53621312_C_A	6E>D	Substitution	Nonsynonymous coding	33%
MM12T	RASA1	RAS p21 protein activator (GTPase activating protein) 1	CCDS34200.1	chr5_86672333-86672333_A_G	712Y>C	Substitution	Nonsynonymous coding	33%
MM12T	RASA2	RAS p21 protein activator 2	CCDS3117.1	chr3_141292891-141292891_G_A	489A>T	Substitution	Nonsynonymous coding	24%
MM12T	RASA3	RAS p21 protein activator 3	ENST00000389544	chr13_114793308-114793308_C_T	182R>H	Substitution	Nonsynonymous coding	15%
MM12T	RASAL1	RAS protein activator like 1 (GAP1 like)	CCDS9165.1	chr12_113565961-113565961_G_T	49L>M	Substitution	Nonsynonymous coding	34%
MM12T	RASAL2	RAS protein activator like 2	CCDS1321.1	chr1_178410760-178410760_G_A	284R>H	Substitution	Nonsynonymous coding	15%
MM12T	RASAL3	RAS protein activator like 3	CCDS46006.1	chr19_15564994-15564994_G_A	749R>C	Substitution	Nonsynonymous coding	25%
MM12T	RASD2	RASD family, member 2	CCDS13916.1	chr22_35947834-35947834_C_T	186L>F	Substitution	Nonsynonymous coding	13%
MM12T	RASGEF1A	RasGEF domain family, member 1A	CCDS7202.2	chr10_43701551-43701551_G_T	5S>Y	Substitution	Nonsynonymous coding	30%
MM12T	RASGRF1	Ras protein-specific guanine nucleotide-releasing factor 1	CCDS10309.1	chr15_79324556-79324556_C_T	354R>H	Substitution	Nonsynonymous coding	12%

MM12T	RASGRF1	Ras protein-specific guanine nucleotide-releasing factor 1	CCDS10309.1	chr15_79277345-79277345_G_A	ISV+4>	Substitution	Splice site donor	31%
MM12T	RASGRF2	Ras protein-specific guanine nucleotide-releasing factor 2	CCDS4052.1	chr5_80476048-80476048_C_T	914A>V	Substitution	Nonsynonymous coding	13%
MM12T	RASIP1	Ras interacting protein 1	CCDS12731.1	chr19_49228130-49228130_C_T	739A>T	Substitution	Nonsynonymous coding	31%
MM12T	RASIP1	Ras interacting protein 1	CCDS12731.1	chr19_49232268-49232268_C_T	587A>T	Substitution	Nonsynonymous coding	28%
MM12T	RASIP1	Ras interacting protein 1	CCDS12731.1	chr19_49242741-49242741_G_A	100T>I	Substitution	Nonsynonymous coding	26%
MM12T	RASSF9	Ras association (RalGDS/AF-6) domain family (N-terminal) member 9	CCDS44950.1	chr12_86198644-86198644_C_T	382A>T	Substitution	Nonsynonymous coding	11%
MM12T	RAVER1	ribonucleoprotein, PTB-binding 1	CCDS45960.1	chr19_10434238-10434238_G_A	271A>V	Substitution	Nonsynonymous coding	32%
MM12T	RB1	retinoblastoma 1	CCDS31973.1	chr13_48953760-48953760_C_T	455R>X	Substitution	Nonsense	33%
MM12T	RB1	retinoblastoma 1	CCDS31973.1	chr13_48955573-48955573_G_A	563W>X	Substitution	Nonsense	39%
MM12T	RBBP7	retinoblastoma binding protein 7	CCDS14179.1	chrX_16871880-16871880_A_G	228V>A	Substitution	Nonsynonymous coding	34%
MM12T	RBBP8NL	RBBP8 N-terminal like	CCDS13498.1	chr20_60988517-60988517_G_A	539P>S	Substitution	Nonsynonymous coding	37%
MM12T	RBFOX1	RNA binding protein, fox-1 homolog (C. elegans) 1	CCDS10531.1	chr16_7721602-7721602_G_A	ISV+1>	Substitution	Splice site donor	34%
MM12T	RBFOX3	RNA binding protein, fox-1 homolog (C. elegans) 3	CCDS45805.1	chr17_77111662-77111662_T_C	46T>A	Substitution	Nonsynonymous coding	22%
MM12T	RBL1	retinoblastoma-like 1 (p107)	CCDS13289.1	chr20_35696592-35696592_A_	NA	Deletion	Splice site acceptor	43%
MM12T	RBL2	retinoblastoma-like 2 (p130)	CCDS10748.1	chr16_53496511-53496511_G_A	502V>I	Substitution	Nonsynonymous coding	38%
MM12T	RBL2	retinoblastoma-like 2 (p130)	CCDS10748.1	chr16_53524171-53524171_C_T	1127R>C	Substitution	Nonsynonymous coding	10%
MM12T	RBM10	RNA binding motif protein 10	CCDS14274.1	chrX_47044548-47044548_A_G	682D>G	Substitution	Nonsynonymous coding	13%
MM12T	RBM15	RNA binding motif protein 15	CCDS822.1	chr1_110884837-110884837_C_A	937A>D	Substitution	Nonsynonymous coding	35%
MM12T	RBM15B	RNA binding motif protein 15B	CCDS33764.1	chr3_51428964-51428964_C_T	45A>V	Substitution	Nonsynonymous coding	44%
MM12T	RBM19	RNA binding motif protein 19	CCDS9172.1	chr12_114400045-114400045_G_A	71R>W	Substitution	Nonsynonymous coding	27%
MM12T	RBM27	RNA binding motif protein 27	CCDS43378.1	chr5_145616986-145616986_G_A	424V>I	Substitution	Nonsynonymous coding	19%
MM12T	RBM43	RNA binding motif protein 43	CCDS2191.1	chr2_152108088-152108088_T_	NA	Deletion	Frameshift	34%
MM12T	RBM44	RNA binding motif protein 44	CCDS46554.1	chr2_238725777-238725777_A_G	73D>G	Substitution	Nonsynonymous coding	26%
MM12T	RBM46	RNA binding motif protein 46	CCDS3790.1	chr4_155719122-155719122_C_T	104A>V	Substitution	Nonsynonymous coding	30%
MM12T	RBM4B	RNA binding motif protein 4B	CCDS8149.1	chr11_66436538-66436538_C_T	213D>N	Substitution	Nonsynonymous coding	37%
MM12T	RBM5	RNA binding motif protein 5	CCDS2810.1	chr3_50145738-50145738_G_A	ISV+1>	Substitution	Splice site donor	28%
MM12T	RBMX2	RNA binding motif protein, X-linked 2	CCDS43993.1	chrX_129545424-129545424_T_C	136C>R	Substitution	Nonsynonymous coding	28%
MM12T	RBP3	retinol binding protein 3, interstitial	CCDS7218.1	chr10_48390057-48390057_G_A	274S>F	Substitution	Nonsynonymous coding	13%
MM12T	RBPJ	recombination signal binding protein for immunoglobulin kappa J region	CCDS3437.1	chr4_26431638-26431638_C_T	349A>V	Substitution	Nonsynonymous coding	24%
MM12T	RBPMS	RNA binding protein with multiple splicing	CCDS34876.1	chr8_30361858-30361858_C_T	101A>V	Substitution	Nonsynonymous coding	27%
MM12T	RC3H1	ring finger and CCCH-type domains 1	CCDS30940.1	chr1_173933998-173933998_G_A	532A>V	Substitution	Nonsynonymous coding	30%
MM12T	RC3H1	ring finger and CCCH-type domains 1	CCDS30940.1	chr1_173952790-173952790_C_A	120G>C	Substitution	Nonsynonymous coding	24%

MM12T	RCAN1	regulator of calcineurin 1	ENST00000381135	chr21_35897457-35897457_C_T	51D>N	Substitution	Nonsynonymous coding	14%
MM12T	RCN1	reticulocalbin 1, EF-hand calcium binding domain	CCDS7876.1	chr11_32119904-32119904_G_A	153A>T	Substitution	Nonsynonymous coding	42%
MM12T	RCSD1	RCSD domain containing 1	CCDS1263.1	chr1_167666450-167666450_G_A	197G>S	Substitution	Nonsynonymous coding	33%
MM12T	RD3	retinal degeneration 3	CCDS1498.1	chr1_211652610-211652610_C_T	119R>H	Substitution	Nonsynonymous coding	31%
MM12T	RECQL4	RecQ protein-like 4	ENST00000428558	chr8_145737702-145737702_G_A	1021R>W	Substitution	Nonsynonymous coding	33%
MM12T	RECQL5	RecQ protein-like 5	CCDS42380.1	chr17_73625542-73625542_C_T	654R>Q	Substitution	Nonsynonymous coding	11%
MM12T	RECQL5	RecQ protein-like 5	CCDS42380.1	chr17_73654395-73654395_C_T	378E>K	Substitution	Nonsynonymous coding	15%
MM12T	REEP2	receptor accessory protein 2	CCDS4205.1	chr5_137781274-137781274_C_T	226A>V	Substitution	Nonsynonymous coding	32%
MM12T	REEP6	receptor accessory protein 6	CCDS12070.1	chr19_1495338-1495338_C_T	54A>V	Substitution	Nonsynonymous coding	35%
MM12T	REL	v-rel reticuloendotheliosis viral oncogene homolog (avian)	CCDS1864.1	chr2_61149071-61149071_C_T	421R>C	Substitution	Nonsynonymous coding	24%
MM12T	RELA	v-rel reticuloendotheliosis viral oncogene homolog A (avian)	CCDS31609.1	chr11_65422018-65422018_T_C	496Y>C	Substitution	Nonsynonymous coding	26%
MM12T	RELB	v-rel reticuloendotheliosis viral oncogene homolog B	CCDS46110.1	chr19_45515205-45515205_G_A	59E>K	Substitution	Nonsynonymous coding	30%
MM12T	RELB	v-rel reticuloendotheliosis viral oncogene homolog B	CCDS46110.1	chr19_45535860-45535860_C_T	354H>Y	Substitution	Nonsynonymous coding	29%
MM12T	RELB	v-rel reticuloendotheliosis viral oncogene homolog B	CCDS46110.1	chr19_45540956-45540956_G_A	550A>T	Substitution	Nonsynonymous coding	13%
MM12T	RELN	reelin	CCDS47680.1	chr7_103301977-103301977_A_	NA	Deletion	Splice site acceptor	13%
MM12T	RELT	RELT tumor necrosis factor receptor	CCDS8222.1	chr11_73103393-73103393_G_A	169V>I	Substitution	Nonsynonymous coding	28%
MM12T	RENBP	renin binding protein	CCDS14738.2	chrX_153209575-153209575_C_T	57R>Q	Substitution	Nonsynonymous coding	35%
MM12T	RERE	arginine-glutamic acid dipeptide (RE) repeats	CCDS95.1	chr1_8421537-8421537_G_T	677P>H	Substitution	Nonsynonymous coding	34%
MM12T	RESP18	regulated endocrine-specific protein 18	NM_001007089	chr2_220197434-220197434_G_A	15A>V	Substitution	Nonsynonymous coding	12%
MM12T	RET	ret proto-oncogene	CCDS7200.1	chr10_43598018-43598018_G_A	189R>H	Substitution	Nonsynonymous coding	33%
MM12T	RETN	resistin	CCDS12182.1	chr19_7734246-7734246_C_A	12L>M	Substitution	Nonsynonymous coding	36%
MM12T	REV1	REV1, polymerase (DNA directed)	CCDS2045.1	chr2_100055425-100055425_C_T	284R>K	Substitution	Nonsynonymous coding	27%
MM12T	REV3L	REV3-like, polymerase (DNA directed), zeta, catalytic subunit	CCDS5091.2	chr6_111634552-111634552_T_C	ISV+3>	Substitution	Splice site donor	15%
MM12T	REXO1	REX1, RNA exonuclease 1 homolog (S. cerevisiae)	CCDS32866.1	chr19_1827654-1827654_C_A	378K>N	Substitution	Nonsynonymous coding	35%
MM12T	RFPL3	ret finger protein-like 3	CCDS43011.1	chr22_32756703-32756703_C_T	280R>C	Substitution	Nonsynonymous coding	22%
MM12T	RFT1	RFT1 homolog (S. cerevisiae)	CCDS2869.1	chr3_53126488-53126488_C_T	452R>H	Substitution	Nonsynonymous coding	29%
MM12T	RFX1	regulatory factor X, 1 (influences HLA class II expression)	CCDS12301.1	chr19_14073978-14073978_C_T	894V>I	Substitution	Nonsynonymous coding	39%
MM12T	RFX1	regulatory factor X, 1 (influences HLA class II expression)	CCDS12301.1	chr19_14074749-14074749_G_A	761A>V	Substitution	Nonsynonymous coding	39%
MM12T	RFX8	RFX family member 8, lacking RFX DNA binding domain	CCDS46376.1	chr2_102022446-102022446_G_T	296A>D	Substitution	Nonsynonymous coding	26%
MM12T	RFXAP	regulatory factor X-associated protein	CCDS9359.1	chr13_37393714-37393714_C_A	74L>M	Substitution	Nonsynonymous coding	25%
MM12T	RGAG1	retrotransposon gag domain containing 1	NM_020769	chrX_109695058-109695058_G_A	405A>T	Substitution	Nonsynonymous coding	30%
MM12T	RGMA	RGM domain family, member A	CCDS45357.1	chr15_93595602-93595602_G_A	89T>M	Substitution	Nonsynonymous coding	34%

MM12T	RGMB	RGM domain family, member B	CCDS47251.1	chr5_98115755-98115755_C_A	244P>H	Substitution	Nonsynonymous coding	26%
MM12T	RGP1	RGP1 retrograde golgi transport homolog (S. cerevisiae)	CCDS47964.1	chr9_35752663-35752663_G_T	363R>I	Substitution	Nonsynonymous coding	27%
MM12T	RGS11	regulator of G-protein signaling 11	CCDS42088.1	chr16_321398-321398_G_A	250A>V	Substitution	Nonsynonymous coding	27%
MM12T	RGS12	regulator of G-protein signaling 12	CCDS3366.1	chr4_3319227-3319227_G_T	444G>C	Substitution	Nonsynonymous coding	34%
MM12T	RGS12	regulator of G-protein signaling 12	CCDS3366.1	chr4_3424680-3424680_C_T	1028R>C	Substitution	Nonsynonymous coding	28%
MM12T	RGS6	regulator of G-protein signaling 6	CCDS9808.1	chr14_72985207-72985207_G_A	414V>I	Substitution	Nonsynonymous coding	27%
MM12T	RHBDL3	rhomboid, veinlet-like 3 (Drosophila)	CCDS32613.1	chr17_30615823-30615823_C_T	103R>C	Substitution	Nonsynonymous coding	34%
MM12T	RHBDL3	rhomboid, veinlet-like 3 (Drosophila)	CCDS32613.1	chr17_30643288-30643288_G_A	307R>Q	Substitution	Nonsynonymous coding	29%
MM12T	RHBG	Rh family, B glycoprotein (gene/pseudogene)	ENST00000255013	chr1_156354348-156354348_C_	NA	Deletion	Splice site acceptor	12%
MM12T	RHOBTB1	Rho-related BTB domain containing 1	CCDS7261.1	chr10_62631293-62631293_G_A	680H>Y	Substitution	Nonsynonymous coding	34%
MM12T	RHOF	ras homolog family member F (in filopodia)	CCDS9222.1	chr12_122219045-122219045_C_T	94V>M	Substitution	Nonsynonymous coding	31%
MM12T	RHOG	ras homolog family member G	CCDS7748.1	chr11_3849035-3849035_G_T	112L>M	Substitution	Nonsynonymous coding	28%
MM12T	RHOT1	ras homolog family member T1	CCDS32610.1	chr17_30530949-30530949_C_T	458A>V	Substitution	Nonsynonymous coding	18%
MM12T	RHOU	ras homolog family member U	CCDS1575.1	chr1_228879404-228879404_C_T	232P>S	Substitution	Nonsynonymous coding	31%
MM12T	RIC8A	resistance to inhibitors of cholinesterase 8 homolog A (C. elegans)	CCDS7690.1	chr11_209809-209809_C_T	179R>C	Substitution	Nonsynonymous coding	31%
MM12T	RIF1	RAP1 interacting factor homolog (yeast)	CCDS2194.1	chr2_152320139-152320139_G_A	1369E>K	Substitution	Nonsynonymous coding	14%
MM12T	RIMS1	regulating synaptic membrane exocytosis 1	CCDS47449.1	chr6_72892114-72892114_C_T	314R>W	Substitution	Nonsynonymous coding	11%
MM12T	RIMS4	regulating synaptic membrane exocytosis 4	CCDS13338.1	chr20_43386308-43386308_G_A	ISV+3>	Substitution	Splice site donor	13%
MM12T	RIOK1	RIO kinase 1	CCDS4500.1	chr6_7410620-7410620_C_T	402A>V	Substitution	Nonsynonymous coding	17%
MM12T	RIOK3	RIO kinase 3	CCDS11877.1	chr18_21044050-21044050_A_	NA	Deletion	Frameshift	19%
MM12T	RIOK3	RIO kinase 3	CCDS11877.1	chr18_21043043-21043043_C_T	60A>V	Substitution	Nonsynonymous coding	32%
MM12T	RIOK3	RIO kinase 3	CCDS11877.1	chr18_21054975-21054975_G_A	359M>I	Substitution	Nonsynonymous coding	29%
MM12T	RIPK1	receptor (TNFRSF)-interacting serine-threonine kinase 1	CCDS4482.1	chr6_3113365-3113365_G_A	603R>H	Substitution	Nonsynonymous coding	21%
MM12T	RIT2	Ras-like without CAAX 2	CCDS11921.1	chr18_40503620-40503620_C_T	115E>K	Substitution	Nonsynonymous coding	13%
MM12T	RNASE9	ribonuclease, RNase A family, 9 (non-active)	CCDS32036.1	chr14_21024693-21024693_G_T	179P>H	Substitution	Nonsynonymous coding	48%
MM12T	RNASEL	ribonuclease L (2',5'-oligoisoadenylate synthetase-dependent)	CCDS1347.1	chr1_182555743-182555743_C_T	67V>I	Substitution	Nonsynonymous coding	10%
MM12T	RND1	Rho family GTPase 1	CCDS8771.1	chr12_49251840-49251840_C_T	213R>H	Substitution	Nonsynonymous coding	12%
MM12T	RNF111	ring finger protein 111	CCDS10169.1	chr15_59384761-59384761_G_A	922G>E	Substitution	Nonsynonymous coding	15%
MM12T	RNF112	ring finger protein 112	NM_007148	chr17_19316270-19316270_C_T	134A>V	Substitution	Nonsynonymous coding	19%
MM12T	RNF114	ring finger protein 114	CCDS33482.1	chr20_48565860-48565860_C_T	197R>C	Substitution	Nonsynonymous coding	29%
MM12T	RNF123	ring finger protein 123	ENST00000389066	chr3_49758847-49758847_C_T	1296S>F	Substitution	Nonsynonymous coding	32%
MM12T	RNF169	ring finger protein 169	CCDS41691.1	chr11_74547680-74547680_C_T	678R>C	Substitution	Nonsynonymous coding	33%

MM12T	RNF20	ring finger protein 20, E3 ubiquitin protein ligase	CCDS35084.1	chr9_104312979-104312979_G_T	395S>I	Substitution	Nonsynonymous coding	30%
MM12T	RNF20	ring finger protein 20, E3 ubiquitin protein ligase	CCDS35084.1	chr9_104323422-104323422_G_T	853L>F	Substitution	Nonsynonymous coding	31%
MM12T	RNF212	ring finger protein 212	ENST00000333673	chr4_1087643-1087643_G_A	136R>W	Substitution	Nonsynonymous coding	28%
MM12T	RNF213	ring finger protein 213	CCDS11772.1	chr17_78265581-78265581_G_A	476V>I	Substitution	Nonsynonymous coding	28%
MM12T	RNF213	ring finger protein 213	CCDS32761.1	chr17_78320404-78320404_G_A	830V>I	Substitution	Nonsynonymous coding	25%
MM12T	RNF213	ring finger protein 213	CCDS32761.1	chr17_78327405-78327405_C_A	1579A>D	Substitution	Nonsynonymous coding	14%
MM12T	RNF214	ring finger protein 214	CCDS41720.1	chr11_117153241-117153241_G_A	612A>T	Substitution	Nonsynonymous coding	13%
MM12T	RNF219	ring finger protein 219	CCDS31997.1	chr13_79191198-79191198_G_A	233T>I	Substitution	Nonsynonymous coding	13%
MM12T	RNF219	ring finger protein 219	CCDS31997.1	chr13_79191220-79191220_C_T	226V>I	Substitution	Nonsynonymous coding	27%
MM12T	RNF25	ring finger protein 25	CCDS2420.1	chr2_219529140-219529140_G_T	307P>H	Substitution	Nonsynonymous coding	20%
MM12T	RNF39	ring finger protein 39	CCDS4673.1	chr6_30040952-30040952_T_	NA	Deletion	Frameshift	22%
MM12T	RNF4	ring finger protein 4	CCDS47001.1	chr4_2498785-2498785_C_T	21R>W	Substitution	Nonsynonymous coding	27%
MM12T	RNF40	ring finger protein 40, E3 ubiquitin protein ligase	CCDS10691.1	chr16_30779231-30779231_G_T	482E>D	Substitution	Nonsynonymous coding	13%
MM12T	RNF41	ring finger protein 41	CCDS8909.1	chr12_56601955-56601955_C_T	164A>T	Substitution	Nonsynonymous coding	14%
MM12T	RNF44	ring finger protein 44	CCDS4404.1	chr5_175956649-175956649_C_T	ISV-4>	Substitution	Splice site acceptor	29%
MM12T	RNF8	ring finger protein 8, E3 ubiquitin protein ligase	CCDS4834.1	chr6_37336779-37336779_T_C	254S>P	Substitution	Nonsynonymous coding	23%
MM12T	RNFT1	ring finger protein, transmembrane 1	CCDS11622.2	chr17_58040311-58040311_C_T	131A>T	Substitution	Nonsynonymous coding	11%
MM12T	RNMTL1	RNA methyltransferase like 1	CCDS10997.1	chr17_694821-694821_G_A	259A>T	Substitution	Nonsynonymous coding	42%
MM12T	ROBO1	roundabout, axon guidance receptor, homolog 1 (Drosophila)	CCDS46872.1	chr3_78683103-78683103_G_A	1116R>W	Substitution	Nonsynonymous coding	24%
MM12T	ROBO1	roundabout, axon guidance receptor, homolog 1 (Drosophila)	CCDS46872.1	chr3_78717195-78717195_C_T	563A>T	Substitution	Nonsynonymous coding	21%
MM12T	ROBO1	roundabout, axon guidance receptor, homolog 1 (Drosophila)	CCDS46872.1	chr3_78988044-78988044_C_T	30R>H	Substitution	Nonsynonymous coding	22%
MM12T	ROBO2	roundabout, axon guidance receptor, homolog 2 (Drosophila)	CCDS43109.1	chr3_77147399-77147399_G_A	99R>H	Substitution	Nonsynonymous coding	15%
MM12T	ROBO2	roundabout, axon guidance receptor, homolog 2 (Drosophila)	CCDS43109.1	chr3_77617566-77617566_C_T	651T>M	Substitution	Nonsynonymous coding	22%
MM12T	ROR2	receptor tyrosine kinase-like orphan receptor 2	CCDS6691.1	chr9_94488950-94488950_C_T	420C>Y	Substitution	Nonsynonymous coding	27%
MM12T	ROS1	c-ros oncogene 1 , receptor tyrosine kinase	CCDS5116.1	chr6_117658370-117658370_T_	NA	Deletion	Frameshift	14%
MM12T	ROS1	c-ros oncogene 1 , receptor tyrosine kinase	CCDS5116.1	chr6_117704649-117704649_G_A	776T>M	Substitution	Nonsynonymous coding	20%
MM12T	RP1	retinitis pigmentosa 1 (autosomal dominant)	CCDS6160.1	chr8_55533531-55533531_G_T	2S>I	Substitution	Nonsynonymous coding	34%
MM12T	RP1	retinitis pigmentosa 1 (autosomal dominant)	CCDS6160.1	chr8_55538993-55538993_G_A	851A>T	Substitution	Nonsynonymous coding	11%
MM12T	RP11-158D1.1	-	ENST00000402782	chr8_36746190-36746190_C_T	53T>M	Substitution	Nonsynonymous coding	29%
MM12T	RP11-218M22.1	-	ENST00000318291	chr12_752358-752358_G_T	71G>W	Substitution	Nonsynonymous coding	39%
MM12T	RP11-286H14.4	-	ENST00000423578	chr7_128767415-128767415_G_A	282V>M	Substitution	Nonsynonymous coding	14%
MM12T	RP1-139D8.6	-	ENST00000372963	chr6_42130833-42130833_G_T	64R>M	Substitution	Nonsynonymous coding	26%

MM12T	RP11-408E5.4	-	ENST00000240700	chr13_19759474-19759474_C_A	9L>M	Substitution	Nonsynonymous coding	11%
MM12T	RP11-616M22.6	-	ENST00000334002	chr16_1312460-1312460_G_A	199A>V	Substitution	Nonsynonymous coding	12%
MM12T	RP1L1	retinitis pigmentosa 1-like 1	CCDS43708.1	chr8_10480498-10480498_C_T	72V>M	Substitution	Nonsynonymous coding	36%
MM12T	RP2	retinitis pigmentosa 2 (X-linked recessive)	CCDS14270.1	chrX_46736976-46736976_C_T	307A>V	Substitution	Nonsynonymous coding	12%
MM12T	RPA4	replication protein A4, 30kDa	CCDS35345.1	chrX_96140070-96140070_G_A	254R>Q	Substitution	Nonsynonymous coding	33%
MM12T	RPH3AL	rabphilin 3A-like (without C2 domains)	CCDS10994.1	chr17_65506-65506_G_A	272T>M	Substitution	Nonsynonymous coding	24%
MM12T	RPIA	ribose 5-phosphate isomerase A	CCDS2004.2	chr2_89036135-89036135_G_A	227R>Q	Substitution	Nonsynonymous coding	27%
MM12T	RPL3	ribosomal protein L3	CCDS13988.1	chr22_39710177-39710177_C_T	296G>S	Substitution	Nonsynonymous coding	48%
MM12T	RPN1	ribophorin I	CCDS3051.1	chr3_128348989-128348989_G_A	ISV-3>	Substitution	Splice site acceptor	40%
MM12T	RPP25L	ribonuclease P/MRP 25kDa subunit-like	CCDS6559.1	chr9_34610872-34610872_C_T	141G>D	Substitution	Nonsynonymous coding	14%
MM12T	RPP30	ribonuclease P/MRP 30kDa subunit	CCDS44458.1	chr10_92638842-92638842_G_A	98R>Q	Substitution	Nonsynonymous coding	25%
MM12T	RPP38	ribonuclease P/MRP 38kDa subunit	CCDS7108.1	chr10_15145436-15145436_G_A	41M>I	Substitution	Nonsynonymous coding	34%
MM12T	RPP38	ribonuclease P/MRP 38kDa subunit	CCDS7108.1	chr10_15146010-15146010_G_A	233D>N	Substitution	Nonsynonymous coding	34%
MM12T	RPRD1A	regulation of nuclear pre-mRNA domain containing 1A	CCDS11917.1	chr18_33573253-33573253_C_T	267R>H	Substitution	Nonsynonymous coding	30%
MM12T	RPS10	ribosomal protein S10	CCDS4792.1	chr6_34386157-34386157_C_T	149E>K	Substitution	Nonsynonymous coding	13%
MM12T	RPS27L	ribosomal protein S27-like	CCDS42048.1	chr15_63447933-63447934_AA_	NA	Deletion	Splice site acceptor	13%
MM12T	RPS29	ribosomal protein S29	CCDS9685.1	chr14_50050397-50050397_A_	NA	Deletion	Splice site acceptor	35%
MM12T	RPS6KC1	ribosomal protein S6 kinase, 52kDa, polypeptide 1	CCDS1513.1	chr1_213436139-213436139_G_A	1024A>T	Substitution	Nonsynonymous coding	22%
MM12T	RPTOR	regulatory associated protein of MTOR, complex 1	CCDS11773.1	chr17_78867626-78867626_C_T	788R>C	Substitution	Nonsynonymous coding	52%
MM12T	RPTOR	regulatory associated protein of MTOR, complex 1	CCDS11773.1	chr17_78897477-78897477_C_T	ISV+4>	Substitution	Splice site donor	43%
MM12T	RPUSD3	RNA pseudouridylate synthase domain containing 3	CCDS2586.2	chr3_9879720-9879720_G_T	346L>M	Substitution	Nonsynonymous coding	24%
MM12T	RRAD	Ras-related associated with diabetes	CCDS10824.1	chr16_66956073-66956073_G_A	278A>V	Substitution	Nonsynonymous coding	14%
MM12T	RRBP1	ribosome binding protein 1	ENST00000246043	chr20_17639530-17639530_C_A	541E>D	Substitution	Nonsynonymous coding	29%
MM12T	RREB1	ras responsive element binding protein 1	CCDS34335.1	chr6_7230755-7230755_A_G	808H>R	Substitution	Nonsynonymous coding	19%
MM12T	RRM1	ribonucleotide reductase M1	CCDS7750.1	chr11_4132840-4132840_C_T	153R>W	Substitution	Nonsynonymous coding	25%
MM12T	RRP8	ribosomal RNA processing 8, methyltransferase, homolog (yeast)	CCDS31411.1	chr11_6621781-6621781_G_A	396R>C	Substitution	Nonsynonymous coding	16%
MM12T	RSAD2	radical S-adenosyl methionine domain containing 2	CCDS1656.1	chr2_7023597-7023597_C_T	148R>W	Substitution	Nonsynonymous coding	27%
MM12T	RSF1	remodeling and spacing factor 1	CCDS8253.1	chr11_77387970-77387970_C_T	1070E>K	Substitution	Nonsynonymous coding	13%
MM12T	RTDR1	rhabdoid tumor deletion region gene 1	CCDS13803.1	chr22_23481133-23481133_G_	NA	Deletion	Splice site acceptor	15%
MM12T	RTEL1-TNFRSF6B	RTEL1-TNFRSF6B readthrough (non-protein coding)	CCDS13530.2	chr20_62324312-62324312_G_A	936G>D	Substitution	Nonsynonymous coding	15%
MM12T	RTL1	retrotransposon-like 1	NM_001134888	chr14_101347828-101347828_G_A	1100R>C	Substitution	Nonsynonymous coding	33%
MM12T	RTN4RL1	reticulon 4 receptor-like 1	CCDS45569.1	chr17_1840943-1840943_C_T	58R>H	Substitution	Nonsynonymous coding	32%

MM12T	RTP3	receptor (chemosensory) transporter protein 3	CCDS2740.1	chr3_46539703-46539703_G_A	51A>T	Substitution	Nonsynonymous coding	34%
MM12T	RUFY1	RUN and FYVE domain containing 1	CCDS4445.2	chr5_178987025-178987025_G_A	ISV-1>	Substitution	Splice site acceptor	38%
MM12T	RUFY2	RUN and FYVE domain containing 2	CCDS41534.1	chr10_70141029-70141029_T_C	391E>G	Substitution	Nonsynonymous coding	27%
MM12T	RUNDC3B	RUN domain containing 3B	CCDS5609.1	chr7_87258194-87258194_G_T	19G>C	Substitution	Nonsynonymous coding	47%
MM12T	RUNX1T1	runt-related transcription factor 1; translocated to, 1 (cyclin D-related)	CCDS6256.1	chr8_92983013-92983013_G_A	471A>V	Substitution	Nonsynonymous coding	34%
MM12T	RUNX1T1	runt-related transcription factor 1; translocated to, 1 (cyclin D-related)	CCDS6256.1	chr8_93029555-93029555_G_A	42P>L	Substitution	Nonsynonymous coding	20%
MM12T	RUNX3	runt-related transcription factor 3	CCDS30633.1	chr1_25254068-25254068_G_A	160R>X	Substitution	Nonsense	30%
MM12T	RUSC2	RUN and SH3 domain containing 2	CCDS35008.1	chr9_35547974-35547974_C_	NA	Deletion	Frameshift	30%
MM12T	RXFP4	relaxin/insulin-like family peptide receptor 4	CCDS1124.1	chr1_155912143-155912143_G_A	215V>M	Substitution	Nonsynonymous coding	38%
MM12T	RXRA	retinoid X receptor, alpha	CCDS35172.1	chr9_137325971-137325971_G_A	387A>T	Substitution	Nonsynonymous coding	10%
MM12T	RYR1	ryanodine receptor 1 (skeletal)	CCDS33011.1	chr19_38955326-38955326_C_T	945A>V	Substitution	Nonsynonymous coding	31%
MM12T	RYR1	ryanodine receptor 1 (skeletal)	CCDS33011.1	chr19_38956813-38956813_G_A	985D>N	Substitution	Nonsynonymous coding	19%
MM12T	RYR1	ryanodine receptor 1 (skeletal)	CCDS33011.1	chr19_38964299-38964299_G_T	1350G>W	Substitution	Nonsynonymous coding	30%
MM12T	RYR1	ryanodine receptor 1 (skeletal)	CCDS33011.1	chr19_38995416-38995416_C_T	2699A>V	Substitution	Nonsynonymous coding	24%
MM12T	RYR2	ryanodine receptor 2 (cardiac)	NM_001035	chr1_237754125-237754125_G_T	1331K>N	Substitution	Nonsynonymous coding	33%
MM12T	RYR2	ryanodine receptor 2 (cardiac)	NM_001035	chr1_237777701-237777701_G_T	1758R>M	Substitution	Nonsynonymous coding	32%
MM12T	RYR2	ryanodine receptor 2 (cardiac)	NM_001035	chr1_237863590-237863590_G_A	3064A>T	Substitution	Nonsynonymous coding	36%
MM12T	RYR3	ryanodine receptor 3	CCDS45210.1	chr15_33858978-33858978_G_T	416A>S	Substitution	Nonsynonymous coding	26%
MM12T	RYR3	ryanodine receptor 3	CCDS45210.1	chr15_33872308-33872308_G_A	467R>H	Substitution	Nonsynonymous coding	13%
MM12T	RYR3	ryanodine receptor 3	CCDS45210.1	chr15_33916084-33916084_G_T	812A>S	Substitution	Nonsynonymous coding	12%
MM12T	RYR3	ryanodine receptor 3	CCDS45210.1	chr15_33952600-33952600_C_T	1533A>V	Substitution	Nonsynonymous coding	15%
MM12T	RYR3	ryanodine receptor 3	CCDS45210.1	chr15_34105762-34105762_C_T	3495P>L	Substitution	Nonsynonymous coding	34%
MM12T	RYR3	ryanodine receptor 3	CCDS45210.1	chr15_34113005-34113005_A_C	3603T>P	Substitution	Nonsynonymous coding	39%
MM12T	RYR3	ryanodine receptor 3	CCDS45210.1	chr15_34118889-34118889_G_T	3727Q>H	Substitution	Nonsynonymous coding	12%
MM12T	RYR3	ryanodine receptor 3	CCDS45210.1	chr15_34130273-34130273_G_A	4031R>H	Substitution	Nonsynonymous coding	26%
MM12T	S100A4	S100 calcium binding protein A4	CCDS1042.1	chr1_153516336-153516336_C_T	69E>K	Substitution	Nonsynonymous coding	35%
MM12T	S100A8	S100 calcium binding protein A8	CCDS1038.1	chr1_153362927-153362927_C_T	29V>I	Substitution	Nonsynonymous coding	12%
MM12T	S100PBP	S100P binding protein	CCDS30666.1	chr1_33291910-33291910_G_T	70E>D	Substitution	Nonsynonymous coding	30%
MM12T	SAC3D1	SAC3 domain containing 1	CCDS41668.1	chr11_64811935-64811935_G_T	271Q>H	Substitution	Nonsynonymous coding	17%
MM12T	SACS	spastic ataxia of Charlevoix-Saguenay (sacsin)	CCDS9300.2	chr13_23910773-23910773_C_A	2414K>N	Substitution	Nonsynonymous coding	29%
MM12T	SACS	spastic ataxia of Charlevoix-Saguenay (sacsin)	CCDS9300.2	chr13_23913201-23913201_C_A	1605S>I	Substitution	Nonsynonymous coding	38%
MM12T	SACS	spastic ataxia of Charlevoix-Saguenay (sacsin)	CCDS9300.2	chr13_23913213-23913213_T_C	1601K>R	Substitution	Nonsynonymous coding	22%

MM12T	SACS	spastic ataxia of Charlevoix-Saguenay (sacsin)	CCDS9300.2	chr13_23929628-23929628_C_T	375V>I	Substitution	Nonsynonymous coding	40%
MM12T	SAFB2	scaffold attachment factor B2	CCDS32879.1	chr19_5590343-5590343_C_	NA	Deletion	Frameshift	34%
MM12T	SAG	S-antigen; retina and pineal gland (arrestin)	CCDS46545.1	chr2_234229332-234229332_G_A	80G>S	Substitution	Nonsynonymous coding	10%
MM12T	SALL4	sal-like 4 (Drosophila)	CCDS13438.1	chr20_50408036-50408036_C_T	329R>H	Substitution	Nonsynonymous coding	15%
MM12T	SAMD15	sterile alpha motif domain containing 15	CCDS32126.1	chr14_77844579-77844579_G_A	273S>N	Substitution	Nonsynonymous coding	31%
MM12T	SAMD15	sterile alpha motif domain containing 15	CCDS32126.1	chr14_77857406-77857406_G_A	615R>H	Substitution	Nonsynonymous coding	26%
MM12T	SAMD15	sterile alpha motif domain containing 15	CCDS32126.1	chr14_77857466-77857466_C_T	635T>I	Substitution	Nonsynonymous coding	37%
MM12T	SAMD4A	sterile alpha motif domain containing 4A	CCDS32084.1	chr14_55218188-55218188_G_T	369R>I	Substitution	Nonsynonymous coding	16%
MM12T	SAMD9L	sterile alpha motif domain containing 9-like	CCDS34681.1	chr7_92762115-92762115_G_A	1057P>L	Substitution	Nonsynonymous coding	19%
MM12T	SARM1	sterile alpha and TIR motif containing 1	ENST00000379061	chr17_26711876-26711876_G_A	439D>N	Substitution	Nonsynonymous coding	10%
MM12T	SARNP	SAP domain containing ribonucleoprotein	CCDS8892.1	chr12_56182900-56182900_C_T	154A>T	Substitution	Nonsynonymous coding	12%
MM12T	SART1	squamous cell carcinoma antigen recognized by T cells	CCDS31611.1	chr11_65731993-65731993_C_T	127R>W	Substitution	Nonsynonymous coding	27%
MM12T	SART1	squamous cell carcinoma antigen recognized by T cells	CCDS31611.1	chr11_65734851-65734851_C_T	437S>F	Substitution	Nonsynonymous coding	14%
MM12T	SART1	squamous cell carcinoma antigen recognized by T cells	CCDS31611.1	chr11_65735239-65735239_C_T	507R>C	Substitution	Nonsynonymous coding	35%
MM12T	SART1	squamous cell carcinoma antigen recognized by T cells	CCDS31611.1	chr11_65743942-65743942_C_T	550A>V	Substitution	Nonsynonymous coding	29%
MM12T	SART1	squamous cell carcinoma antigen recognized by T cells	CCDS31611.1	chr11_65745258-65745258_G_A	687R>Q	Substitution	Nonsynonymous coding	16%
MM12T	SART1	squamous cell carcinoma antigen recognized by T cells	CCDS31611.1	chr11_65735248-65735248_C_T	510R>X	Substitution	Nonsense	28%
MM12T	SATB1	SATB homeobox 1	CCDS2631.1	chr3_18390691-18390691_T_G	755T>P	Substitution	Nonsynonymous coding	18%
MM12T	SATB1	SATB homeobox 1	CCDS2631.1	chr3_18436124-18436124_A_T	346Y>N	Substitution	Nonsynonymous coding	11%
MM12T	SATB1	SATB homeobox 1	CCDS2631.1	chr3_18462281-18462281_G_A	60S>L	Substitution	Nonsynonymous coding	15%
MM12T	SAYSD1	SAYSVFN motif domain containing 1	CCDS4840.1	chr6_39082757-39082757_C_T	37A>T	Substitution	Nonsynonymous coding	24%
MM12T	SBF2	SET binding factor 2	CCDS31427.1	chr11_10051426-10051426_C_T	ISV-4>	Substitution	Splice site acceptor	32%
MM12T	SBK1	SH3-binding domain kinase 1	CCDS32416.1	chr16_28328800-28328800_C_T	30P>S	Substitution	Nonsynonymous coding	27%
MM12T	SBK2	SH3-binding domain kinase family, member 2	CCDS42631.1	chr19_56041302-56041302_G_A	282A>V	Substitution	Nonsynonymous coding	20%
MM12T	SBK2	SH3-binding domain kinase family, member 2	CCDS42631.1	chr19_56041618-56041618_G_A	177R>C	Substitution	Nonsynonymous coding	38%
MM12T	SBNO1	strawberry notch homolog 1 (Drosophila)	CCDS9246.1	chr12_123805365-123805365__T	NA	Insertion	Frameshift	15%
MM12T	SBNO2	strawberry notch homolog 2 (Drosophila)	CCDS45894.1	chr19_1123541-1123541_G_A	207P>L	Substitution	Nonsynonymous coding	26%
MM12T	SCAF11	SR-related CTD-associated factor 11	CCDS8748.2	chr12_46321116-46321116_G_A	790R>X	Substitution	Nonsense	29%
MM12T	SCAF8	SR-related CTD-associated factor 8	CCDS5247.1	chr6_155154099-155154099_G_A	1129R>Q	Substitution	Nonsynonymous coding	40%
MM12T	SCAMP2	secretory carrier membrane protein 2	CCDS10271.1	chr15_75143713-75143713_C_T	151M>I	Substitution	Nonsynonymous coding	33%
MM12T	SCAND3	SCAN domain containing 3	CCDS34355.1	chr6_28543263-28543263_G_A	407R>W	Substitution	Nonsynonymous coding	45%
MM12T	SCARB2	scavenger receptor class B, member 2	CCDS3577.1	chr4_77134578-77134578_A_G	ISV+2>	Substitution	Splice site donor	28%

MM12T	SCARF1	scavenger receptor class F, member 1	CCDS11007.1	chr17_1547201-1547201_C_T	109G>D	Substitution	Nonsynonymous coding	37%
MM12T	SCARF1	scavenger receptor class F, member 1	CCDS11007.1	chr17_1547222-1547222_C_T	102S>N	Substitution	Nonsynonymous coding	13%
MM12T	SCD	stearoyl-CoA desaturase (delta-9-desaturase)	CCDS7493.1	chr10_102112174-102112174_G_A	121R>H	Substitution	Nonsynonymous coding	18%
MM12T	SCFD1	sec1 family domain containing 1	CCDS9639.1	chr14_31097426-31097426_C_T	25R>C	Substitution	Nonsynonymous coding	23%
MM12T	SCG2	secretogranin II	CCDS2457.1	chr2_224462179-224462179_G_A	608H>Y	Substitution	Nonsynonymous coding	23%
MM12T	SCG2	secretogranin II	CCDS2457.1	chr2_224462425-224462425_G_A	526R>X	Substitution	Nonsense	28%
MM12T	SCG3	secretogranin III	CCDS10142.1	chr15_51980558-51980558_G_A	167V>M	Substitution	Nonsynonymous coding	14%
MM12T	SCIN	scinderin	CCDS47545.1	chr7_12620722-12620722_C_T	131T>M	Substitution	Nonsynonymous coding	28%
MM12T	SCIN	scinderin	CCDS47545.1	chr7_12666362-12666362_C_T	379H>Y	Substitution	Nonsynonymous coding	17%
MM12T	SCML4	sex comb on midleg-like 4 (Drosophila)	CCDS5060.2	chr6_108066164-108066164_C_T	224R>K	Substitution	Nonsynonymous coding	34%
MM12T	SCN11A	sodium channel, voltage-gated, type XI, alpha subunit	CCDS33737.1	chr3_38913709-38913709_G_A	1157A>V	Substitution	Nonsynonymous coding	11%
MM12T	SCN1A	sodium channel, voltage-gated, type I, alpha subunit	CCDS33316.1	chr2_166900484-166900484_G_A	580R>X	Substitution	Nonsense	15%
MM12T	SCN4A	sodium channel, voltage-gated, type IV, alpha subunit	CCDS45761.1	chr17_62043502-62043502_C_T	401R>H	Substitution	Nonsynonymous coding	16%
MM12T	SCN4A	sodium channel, voltage-gated, type IV, alpha subunit	CCDS45761.1	chr17_62050177-62050177_G_T	9L>M	Substitution	Nonsynonymous coding	27%
MM12T	SCN5A	sodium channel, voltage-gated, type V, alpha subunit	CCDS46796.1	chr3_38598029-38598029_T_C	1447Y>C	Substitution	Nonsynonymous coding	12%
MM12T	SCN5A	sodium channel, voltage-gated, type V, alpha subunit	CCDS46796.1	chr3_38628895-38628895_C_T	811R>H	Substitution	Nonsynonymous coding	46%
MM12T	SCN5A	sodium channel, voltage-gated, type V, alpha subunit	CCDS46796.1	chr3_38628905-38628905_G_A	808R>C	Substitution	Nonsynonymous coding	38%
MM12T	SCN7A	sodium channel, voltage-gated, type VII, alpha subunit	CCDS46442.1	chr2_167262742-167262742_C_T	1466C>Y	Substitution	Nonsynonymous coding	18%
MM12T	SCN8A	sodium channel, voltage gated, type VIII, alpha subunit	CCDS44891.1	chr12_52200119-52200119_C_T	1617R>X	Substitution	Nonsense	10%
MM12T	SCN9A	sodium channel, voltage-gated, type IX, alpha subunit	CCDS46441.1	chr2_167138321-167138321_A_	NA	Deletion	Splice site acceptor	21%
MM12T	SCNN1B	sodium channel, non-voltage-gated 1, beta subunit	CCDS10609.1	chr16_23382745-23382745_G_A	336A>T	Substitution	Nonsynonymous coding	12%
MM12T	SCNN1G	sodium channel, non-voltage-gated 1, gamma subunit	CCDS10608.1	chr16_23197815-23197815_G_A	75V>I	Substitution	Nonsynonymous coding	36%
MM12T	SCO2	SCO2 cytochrome c oxidase assembly protein	CCDS14095.1	chr22_50962113-50962113_C_T	243R>Q	Substitution	Nonsynonymous coding	13%
MM12T	SCRIB	scribbled homolog (Drosophila)	CCDS6412.1	chr8_144891044-144891044_G_A	617S>F	Substitution	Nonsynonymous coding	33%
MM12T	SCRIB	scribbled homolog (Drosophila)	CCDS6412.1	chr8_144893174-144893174_G_A	392A>V	Substitution	Nonsynonymous coding	36%
MM12T	SCUBE1	signal peptide, CUB domain, EGF-like 1	CCDS14048.1	chr22_43625120-43625120_A_G	348C>R	Substitution	Nonsynonymous coding	25%
MM12T	SCUBE2	signal peptide, CUB domain, EGF-like 2	CCDS7797.1	chr11_9047267-9047267_G_A	917P>S	Substitution	Nonsynonymous coding	12%
MM12T	SCYL2	SCY1-like 2 (S. cerevisiae)	CCDS9076.1	chr12_100707266-100707266_C_A	307L>M	Substitution	Nonsynonymous coding	25%
MM12T	SCYL3	SCY1-like 3 (S. cerevisiae)	CCDS1287.1	chr1_169845129-169845129_G_A	152A>V	Substitution	Nonsynonymous coding	36%
MM12T	SDK2	sidekick cell adhesion molecule 2	CCDS45769.1	chr17_71348665-71348665_G_A	1902A>V	Substitution	Nonsynonymous coding	31%
MM12T	SDK2	sidekick cell adhesion molecule 2	CCDS45769.1	chr17_71383996-71383996_G_T	1458A>D	Substitution	Nonsynonymous coding	25%
MM12T	SDK2	sidekick cell adhesion molecule 2	CCDS45769.1	chr17_71429979-71429979_G_A	402P>S	Substitution	Nonsynonymous coding	15%

MM12T	SEC13	SEC13 homolog (S. cerevisiae)	CCDS2599.1	chr3_10345810-10345810_G_A	252T>M	Substitution	Nonsynonymous coding	13%
MM12T	SEC13	SEC13 homolog (S. cerevisiae)	CCDS2599.1	chr3_10347322-10347322_C_T	169D>N	Substitution	Nonsynonymous coding	32%
MM12T	SEC22A	SEC22 vesicle trafficking protein homolog A (S. cerevisiae)	CCDS3021.1	chr3_122978437-122978437_G_A	ISV+1>	Substitution	Splice site donor	42%
MM12T	SEC24D	SEC24 family, member D (S. cerevisiae)	CCDS3710.1	chr4_119652636-119652636_C_T	901M>I	Substitution	Nonsynonymous coding	13%
MM12T	SEC31B	SEC31 homolog B (S. cerevisiae)	CCDS7495.1	chr10_102250005-102250005_G_A	909P>S	Substitution	Nonsynonymous coding	25%
MM12T	SEC61A1	Sec61 alpha 1 subunit (S. cerevisiae)	CCDS3046.1	chr3_127783842-127783842_G_A	247A>T	Substitution	Nonsynonymous coding	12%
MM12T	SEC63	SEC63 homolog (S. cerevisiae)	CCDS5061.1	chr6_108279210-108279210_C_T	2A>T	Substitution	Nonsynonymous coding	20%
MM12T	SEH1L	SEH1-like (S. cerevisiae)	CCDS32791.1	chr18_12963285-12963285_G_T	146D>Y	Substitution	Nonsynonymous coding	21%
MM12T	SEMA3F	sema domain, immunoglobulin domain (Ig), short basic domain, secreted, (semaphorin) 3F	CCDS2811.1	chr3_50225291-50225291_G_A	701A>T	Substitution	Nonsynonymous coding	10%
MM12T	SEMA3G	sema domain, immunoglobulin domain (Ig), short basic domain, secreted, (semaphorin) 3G	CCDS2856.1	chr3_52476767-52476767_C_A	91R>L	Substitution	Nonsynonymous coding	29%
MM12T	SEMA4A	sema domain, immunoglobulin domain (Ig), transmembrane domain (TM) and short	CCDS1132.1	chr1_156130294-156130294_G_A	210R>H	Substitution	Nonsynonymous coding	34%
MM12T	SEMA4C	sema domain, immunoglobulin domain (Ig), transmembrane domain (TM) and short	CCDS2029.1	chr2_97527504-97527504_C_T	524S>N	Substitution	Nonsynonymous coding	32%
MM12T	SEMA4D	sema domain, immunoglobulin domain (Ig), transmembrane domain (TM) and short	CCDS6685.1	chr9_92001359-92001359_C_T	457A>T	Substitution	Nonsynonymous coding	33%
MM12T	SEMA4G	sema domain, immunoglobulin domain (Ig), transmembrane domain (TM) and short	CCDS7501.1	chr10_102738986-102738986_G_A	314S>N	Substitution	Nonsynonymous coding	34%
MM12T	SENP2	SUMO1/sentrin/SMT3 specific peptidase 2	CCDS33902.1	chr3_185335310-185335310_C_T	385Q>X	Substitution	Nonsense	26%
MM12T	SENP6	SUMO1/sentrin specific peptidase 6	CCDS47454.1	chr6_76405530-76405530_C_T	696L>F	Substitution	Nonsynonymous coding	16%
MM12T	SEPSECS	Sep (O-phosphoserine) tRNA:Sec (selenocysteine) tRNA synthase	CCDS3432.1	chr4_25125692-25125692_C_A	377R>M	Substitution	Nonsynonymous coding	31%
MM12T	SERINC2	serine incorporator 2	CCDS30662.1	chr1_31905867-31905867_G_A	356C>Y	Substitution	Nonsynonymous coding	26%
MM12T	SERPINA1	serpin peptidase inhibitor, clade A (alpha-1 antiproteinase, antitrypsin), member 1	CCDS9925.1	chr14_94845910-94845910_C_T	319G>E	Substitution	Nonsynonymous coding	11%
MM12T	SERPINB6	serpin peptidase inhibitor, clade B (ovalbumin), member 6	CCDS4479.1	chr6_2954883-2954883_T_C	125S>G	Substitution	Nonsynonymous coding	38%
MM12T	SERPINF2	serpin peptidase inhibitor, clade F (alpha-2 antiplasmin, pigment epithelium derived	CCDS11011.1	chr17_1651916-1651916_C_T	247P>L	Substitution	Nonsynonymous coding	35%
MM12T	SERPING1	serpin peptidase inhibitor, clade G (C1 inhibitor), member 1	CCDS7962.1	chr11_57367823-57367823_G_A	175A>T	Substitution	Nonsynonymous coding	20%
MM12T	SETBP1	SET binding protein 1	CCDS11923.2	chr18_42281598-42281598_C_T	96A>V	Substitution	Nonsynonymous coding	34%
MM12T	SETD2	SET domain containing 2	CCDS2749.2	chr3_47129719-47129719_C_A	1721A>S	Substitution	Nonsynonymous coding	12%
MM12T	SETD7	SET domain containing (lysine methyltransferase) 7	CCDS3748.1	chr4_140432878-140432878_G_T	347P>H	Substitution	Nonsynonymous coding	21%
MM12T	SETX	senataxin	CCDS6947.1	chr9_135139846-135139846_C_T	2605R>Q	Substitution	Nonsynonymous coding	31%
MM12T	SETX	senataxin	CCDS6947.1	chr9_135202634-135202634_C_T	1451V>I	Substitution	Nonsynonymous coding	37%
MM12T	SEZ6	seizure related 6 homolog (mouse)	CCDS45639.1	chr17_27308823-27308823_T_C	97D>G	Substitution	Nonsynonymous coding	44%
MM12T	SEZ6L	seizure related 6 homolog (mouse)-like	CCDS13833.1	chr22_26688446-26688446_C_T	57P>S	Substitution	Nonsynonymous coding	13%
MM12T	SEZ6L	seizure related 6 homolog (mouse)-like	CCDS13833.1	chr22_26688962-26688962_G_A	229A>T	Substitution	Nonsynonymous coding	12%
MM12T	SEZ6L2	seizure related 6 homolog (mouse)-like 2	CCDS10659.1	chr16_29906718-29906718_C_A	239G>C	Substitution	Nonsynonymous coding	11%
MM12T	SF3A1	splicing factor 3a, subunit 1, 120kDa	CCDS13875.1	chr22_30737692-30737692_C_A	354D>Y	Substitution	Nonsynonymous coding	29%

MM12T	SF3B3	splicing factor 3b, subunit 3, 130kDa	CCDS10894.1	chr16_70563046-70563046_G_A	114R>H	Substitution	Nonsynonymous coding	12%
MM12T	SF3B3	splicing factor 3b, subunit 3, 130kDa	CCDS10894.1	chr16_70566412-70566412_G_A	201A>T	Substitution	Nonsynonymous coding	35%
MM12T	SF3B4	splicing factor 3b, subunit 4, 49kDa	CCDS941.1	chr1_149898573-149898573_C_T	134R>Q	Substitution	Nonsynonymous coding	55%
MM12T	SFMBT2	Scm-like with four mbt domains 2	CCDS31138.1	chr10_7327896-7327896_C_A	153D>Y	Substitution	Nonsynonymous coding	11%
MM12T	SFPQ	splicing factor proline/glutamine-rich	CCDS388.1	chr1_35652837-35652837_G_A	611R>X	Substitution	Nonsense	29%
MM12T	SFTPA1	surfactant protein A1	CCDS44445.1	chr10_81373804-81373804_T_A	228Y>N	Substitution	Nonsynonymous coding	11%
MM12T	SFXN3	sideroflexin 3	CCDS7508.2	chr10_102795840-102795840_A_G	142D>G	Substitution	Nonsynonymous coding	13%
MM12T	SFXN5	sideroflexin 5	CCDS1922.1	chr2_73227601-73227601_G_T	143H>N	Substitution	Nonsynonymous coding	11%
MM12T	SGCA	sarcoglycan, alpha (50kDa dystrophin-associated glycoprotein)	CCDS32679.1	chr17_48246476-48246476_C_T	203A>V	Substitution	Nonsynonymous coding	12%
MM12T	SGK110	putative uncharacterized serine/threonine-protein kinase SgK110 [Source:RefSeq	ENST00000429736	chr19_56052351-56052351_C_A	314R>I	Substitution	Nonsynonymous coding	31%
MM12T	SGOL1	shugoshin-like 1 (S. pombe)	CCDS33716.1	chr3_20216050-20216050_T_	NA	Deletion	Frameshift	23%
MM12T	SGOL2	shugoshin-like 2 (S. pombe)	CCDS42796.1	chr2_201436926-201436926_G_T	619K>N	Substitution	Nonsynonymous coding	24%
MM12T	SGPL1	sphingosine-1-phosphate lyase 1	CCDS31216.1	chr10_72629616-72629616_C_T	258R>W	Substitution	Nonsynonymous coding	14%
MM12T	SGPP1	sphingosine-1-phosphate phosphatase 1	CCDS9760.1	chr14_64153188-64153188_C_T	321E>K	Substitution	Nonsynonymous coding	27%
MM12T	SGSM2	small G protein signaling modulator 2	CCDS32526.1	chr17_2265006-2265006_C_T	70A>V	Substitution	Nonsynonymous coding	22%
MM12T	SGSM2	small G protein signaling modulator 2	ENST00000424187	chr17_2266206-2266206_C_A	155P>H	Substitution	Nonsynonymous coding	37%
MM12T	SGSM2	small G protein signaling modulator 2	CCDS32526.1	chr17_2276766-2276766_G_A	687V>M	Substitution	Nonsynonymous coding	14%
MM12T	SGSM3	small G protein signaling modulator 3	CCDS14002.1	chr22_40802604-40802604_G_A	375A>T	Substitution	Nonsynonymous coding	14%
MM12T	SGSM3	small G protein signaling modulator 3	ENST00000322069	chr22_40802702-40802702_G_T	350Q>H	Substitution	Nonsynonymous coding	31%
MM12T	SH2B3	SH2B adaptor protein 3	CCDS9153.1	chr12_111856125-111856125_G_A	59R>H	Substitution	Nonsynonymous coding	11%
MM12T	SH2D1A	SH2 domain containing 1A	CCDS14608.1	chrX_123499657-123499657_G_A	62G>S	Substitution	Nonsynonymous coding	30%
MM12T	SH2D3A	SH2 domain containing 3A	CCDS12173.1	chr19_6754150-6754150_G_A	433R>C	Substitution	Nonsynonymous coding	13%
MM12T	SH2D3A	SH2 domain containing 3A	CCDS12173.1	chr19_6760769-6760769_C_T	100R>H	Substitution	Nonsynonymous coding	26%
MM12T	SH2D3C	SH2 domain containing 3C	CCDS6877.1	chr9_130536578-130536578_C_T	69R>H	Substitution	Nonsynonymous coding	23%
MM12T	SH2D7	SH2 domain containing 7	CCDS45315.1	chr15_78390312-78390312_G_A	103R>Q	Substitution	Nonsynonymous coding	14%
MM12T	SH2D7	SH2 domain containing 7	CCDS45315.1	chr15_78390423-78390423_C_A	140A>D	Substitution	Nonsynonymous coding	12%
MM12T	SH3BP5L	SH3-binding domain protein 5-like	CCDS31126.1	chr1_249110750-249110750_G_A	120R>W	Substitution	Nonsynonymous coding	20%
MM12T	SH3GLB2	SH3-domain GRB2-like endophilin B2	CCDS6916.1	chr9_131774523-131774523_C_T	206A>T	Substitution	Nonsynonymous coding	37%
MM12T	SH3GLB2	SH3-domain GRB2-like endophilin B2	CCDS6916.1	chr9_131783499-131783499_C_T	ISV-1>	Substitution	Splice site acceptor	39%
MM12T	SH3PXD2A	SH3 and PX domains 2A	CCDS31278.1	chr10_105363249-105363249_C_T	548V>M	Substitution	Nonsynonymous coding	35%
MM12T	SH3RF1	SH3 domain containing ring finger 1	CCDS34099.1	chr4_170042069-170042069_A_	NA	Deletion	Frameshift	13%
MM12T	SH3RF2	SH3 domain containing ring finger 2	CCDS4280.1	chr5_145428646-145428646_C_T	387A>V	Substitution	Nonsynonymous coding	43%

MM12T	SH3TC1	SH3 domain and tetratricopeptide repeats 1	CCDS3399.1	chr4_8221193-8221193_G_A	350A>T	Substitution	Nonsynonymous coding	17%
MM12T	SH3TC1	SH3 domain and tetratricopeptide repeats 1	CCDS3399.1	chr4_8235213-8235213_G_T	1085Q>H	Substitution	Nonsynonymous coding	38%
MM12T	SHANK1	SH3 and multiple ankyrin repeat domains 1	CCDS12799.1	chr19_51170740-51170740_G_A	1493R>W	Substitution	Nonsynonymous coding	13%
MM12T	SHANK1	SH3 and multiple ankyrin repeat domains 1	CCDS12799.1	chr19_51206817-51206817_C_T	498R>H	Substitution	Nonsynonymous coding	17%
MM12T	SHANK1	SH3 and multiple ankyrin repeat domains 1	CCDS12799.1	chr19_51206944-51206944_C_T	456A>T	Substitution	Nonsynonymous coding	15%
MM12T	SHANK2	SH3 and multiple ankyrin repeat domains 2	NM_133266	chr11_70333378-70333378_G_A	419A>V	Substitution	Nonsynonymous coding	18%
MM12T	SHANK3	SH3 and multiple ankyrin repeat domains 3	CCDS46737.1	chr22_51143268-51143268_G_A	641R>H	Substitution	Nonsynonymous coding	11%
MM12T	SHANK3	SH3 and multiple ankyrin repeat domains 3	CCDS46737.1	chr22_51159283-51159283_G_A	1024A>T	Substitution	Nonsynonymous coding	28%
MM12T	SHANK3	SH3 and multiple ankyrin repeat domains 3	CCDS46737.1	chr22_51169712-51169712_G_A	1739R>H	Substitution	Nonsynonymous coding	13%
MM12T	SHC2	SHC (Src homology 2 domain containing) transforming protein 2	CCDS45891.1	chr19_436412-436412_G_A	265A>V	Substitution	Nonsynonymous coding	36%
MM12T	SHC3	SHC (Src homology 2 domain containing) transforming protein 3	CCDS6681.1	chr9_91692786-91692786_G_T	193P>H	Substitution	Nonsynonymous coding	26%
MM12T	SHCBP1L	SHC SH2-domain binding protein 1-like	CCDS30955.1	chr1_182921884-182921884_C_T	129E>K	Substitution	Nonsynonymous coding	32%
MM12T	SHCBP1L	SHC SH2-domain binding protein 1-like	ENST00000287709	chr1_182922342-182922342_C_A	46A>S	Substitution	Nonsynonymous coding	34%
MM12T	SHH	sonic hedgehog	CCDS5942.1	chr7_155595610-155595610_G_A	458A>V	Substitution	Nonsynonymous coding	28%
MM12T	SHISA3	shisa homolog 3 (Xenopus laevis)	CCDS33979.1	chr4_42403338-42403338_A_G	196Y>C	Substitution	Nonsynonymous coding	46%
MM12T	SHMT1	serine hydroxymethyltransferase 1 (soluble)	ENST00000329968	chr17_18251613-18251613_C_T	108G>D	Substitution	Nonsynonymous coding	21%
MM12T	SHMT2	serine hydroxymethyltransferase 2 (mitochondrial)	CCDS8934.1	chr12_57624757-57624757_C_T	69R>C	Substitution	Nonsynonymous coding	12%
MM12T	SHMT2	serine hydroxymethyltransferase 2 (mitochondrial)	CCDS8934.1	chr12_57627406-57627406_G_A	362A>T	Substitution	Nonsynonymous coding	27%
MM12T	SHROOM2	shroom family member 2	CCDS14135.1	chrX_9900244-9900244_G_A	974S>N	Substitution	Nonsynonymous coding	30%
MM12T	SHROOM3	shroom family member 3	CCDS3579.2	chr4_77659960-77659960_C_T	212R>W	Substitution	Nonsynonymous coding	15%
MM12T	SHROOM3	shroom family member 3	CCDS3579.2	chr4_77700055-77700055_C_A	1906L>M	Substitution	Nonsynonymous coding	37%
MM12T	SHROOM4	shroom family member 4	CCDS35277.1	chrX_50557008-50557008_C_T	4R>Q	Substitution	Nonsynonymous coding	28%
MM12T	SIDT1	SID1 transmembrane family, member 1	CCDS2974.1	chr3_113331029-113331029_C_T	652R>C	Substitution	Nonsynonymous coding	20%
MM12T	SIGLEC1	sialic acid binding Ig-like lectin 1, sialoadhesin	CCDS13060.1	chr20_3687730-3687730_C_A	16A>S	Substitution	Nonsynonymous coding	30%
MM12T	SIGLEC16	sialic acid binding Ig-like lectin 16 (gene/pseudogene)	ENST00000417280	chr19_50476305-50476305_G_T	488R>M	Substitution	Nonsynonymous coding	32%
MM12T	SIGLEC5	sialic acid binding Ig-like lectin 5	CCDS33088.1	chr19_52132326-52132326_G_A	235A>V	Substitution	Nonsynonymous coding	24%
MM12T	SIGLEC9	sialic acid binding Ig-like lectin 9	CCDS12825.1	chr19_51629047-51629047_G_T	205Q>H	Substitution	Nonsynonymous coding	32%
MM12T	SIK2	salt-inducible kinase 2	CCDS8347.1	chr11_111571609-111571609_G_T	ISV-1>	Substitution	Splice site acceptor	40%
MM12T	SIK3	SIK family kinase 3	CCDS8379.1	chr11_116746131-116746131_G_A	368P>S	Substitution	Nonsynonymous coding	14%
MM12T	SIL1	SIL1 homolog, endoplasmic reticulum chaperone (S. cerevisiae)	CCDS4209.1	chr5_138356971-138356971_G_A	219A>V	Substitution	Nonsynonymous coding	13%
MM12T	SIN3A	SIN3 transcription regulator homolog A (yeast)	CCDS10279.1	chr15_75668133-75668133_G_A	1155T>I	Substitution	Nonsynonymous coding	32%
MM12T	SIN3B	SIN3 transcription regulator homolog B (yeast)	CCDS32946.1	chr19_16980568-16980568_G_A	734A>T	Substitution	Nonsynonymous coding	31%

MM12T	SIN3B	SIN3 transcription regulator homolog B (yeast)	CCDS32946.1	chr19_16987313-16987313_G_A	960R>H	Substitution	Nonsynonymous coding	12%
MM12T	SIPA1	signal-induced proliferation-associated 1	CCDS8108.1	chr11_65415014-65415014_G_A	731V>M	Substitution	Nonsynonymous coding	11%
MM12T	SIPA1L1	signal-induced proliferation-associated 1 like 1	CCDS9807.1	chr14_72191430-72191430_C_T	1519R>C	Substitution	Nonsynonymous coding	15%
MM12T	SIPA1L3	signal-induced proliferation-associated 1 like 3	CCDS33007.1	chr19_38591735-38591735_G_A	633G>D	Substitution	Nonsynonymous coding	13%
MM12T	SIRPB2	signal-regulatory protein beta 2	CCDS42849.1	chr20_1460678-1460678_G_A	40Q>X	Substitution	Nonsense	35%
MM12T	SIRT6	sirtuin 6	CCDS12122.1	chr19_4175908-4175908_C_T	155G>D	Substitution	Nonsynonymous coding	22%
MM12T	SKA1	spindle and kinetochore associated complex subunit 1	CCDS11946.1	chr18_47917558-47917558_G_A	172V>I	Substitution	Nonsynonymous coding	46%
MM12T	SKIL	SKI-like oncogene	CCDS33890.1	chr3_170108188-170108188_G_T	536R>I	Substitution	Nonsynonymous coding	36%
MM12T	SKIV2L	superkiller viralicidic activity 2-like (S. cerevisiae)	CCDS4731.1	chr6_31934558-31934558_G_A	759A>T	Substitution	Nonsynonymous coding	18%
MM12T	SKIV2L	superkiller viralicidic activity 2-like (S. cerevisiae)	CCDS4731.1	chr6_31936247-31936247_C_T	1001R>W	Substitution	Nonsynonymous coding	45%
MM12T	SLAIN2	SLAIN motif family, member 2	CCDS47051.1	chr4_48422403-48422403_C_T	541A>V	Substitution	Nonsynonymous coding	35%
MM12T	SLC11A2	solute carrier family 11 (proton-coupled divalent metal ion transporters), member 2	CCDS8805.1	chr12_51389441-51389441_C_A	321A>S	Substitution	Nonsynonymous coding	21%
MM12T	SLC12A5	solute carrier family 12 (potassium/chloride transporter), member 5	CCDS46610.1	chr20_44650444-44650444_G_T	14G>W	Substitution	Nonsynonymous coding	18%
MM12T	SLC12A5	solute carrier family 12 (potassium/chloride transporter), member 5	CCDS13391.1	chr20_44658005-44658005_T_C	8C>R	Substitution	Nonsynonymous coding	30%
MM12T	SLC12A5	solute carrier family 12 (potassium/chloride transporter), member 5	CCDS46610.1	chr20_44684911-44684911_G_T	993E>D	Substitution	Nonsynonymous coding	33%
MM12T	SLC12A9	solute carrier family 12 (potassium/chloride transporters), member 9	CCDS5707.1	chr7_100456537-100456537_G_T	280G>C	Substitution	Nonsynonymous coding	29%
MM12T	SLC13A3	solute carrier family 13 (sodium-dependent dicarboxylate transporter), member 3	CCDS13400.1	chr20_45188833-45188833_C_T	546R>Q	Substitution	Nonsynonymous coding	18%
MM12T	SLC13A4	solute carrier family 13 (sodium/sulfate symporters), member 4	CCDS5840.1	chr7_135376373-135376373_G_A	414A>V	Substitution	Nonsynonymous coding	10%
MM12T	SLC15A2	solute carrier family 15 (H+/peptide transporter), member 2	CCDS3007.1	chr3_121658260-121658260_C_T	609A>V	Substitution	Nonsynonymous coding	24%
MM12T	SLC15A3	solute carrier family 15, member 3	CCDS7998.1	chr11_60718864-60718864_C_T	54A>T	Substitution	Nonsynonymous coding	12%
MM12T	SLC15A3	solute carrier family 15, member 3	CCDS7998.1	chr11_60718882-60718882_C_T	48A>T	Substitution	Nonsynonymous coding	20%
MM12T	SLC16A2	solute carrier family 16, member 2 (thyroid hormone transporter)	CCDS14426.1	chrX_73744222-73744222_G_A	276G>R	Substitution	Nonsynonymous coding	42%
MM12T	SLC16A2	solute carrier family 16, member 2 (thyroid hormone transporter)	CCDS14426.1	chrX_73745669-73745669_C_T	445R>C	Substitution	Nonsynonymous coding	23%
MM12T	SLC16A8	solute carrier family 16, member 8 (monocarboxylic acid transporter 3)	CCDS13966.1	chr22_38477590-38477590_C_T	152G>D	Substitution	Nonsynonymous coding	12%
MM12T	SLC16A9	solute carrier family 16, member 9 (monocarboxylic acid transporter 9)	CCDS7256.1	chr10_61413948-61413948_C_T	279C>Y	Substitution	Nonsynonymous coding	12%
MM12T	SLC17A1	solute carrier family 17 (sodium phosphate), member 1	CCDS4565.1	chr6_25826742-25826742_C_A	52D>Y	Substitution	Nonsynonymous coding	16%
MM12T	SLC17A3	solute carrier family 17 (sodium phosphate), member 3	CCDS47385.1	chr6_25850360-25850360_C_A	347G>C	Substitution	Nonsynonymous coding	42%
MM12T	SLC17A4	solute carrier family 17 (sodium phosphate), member 4	CCDS4564.1	chr6_25762201-25762201_G_A	4G>E	Substitution	Nonsynonymous coding	21%
MM12T	SLC18A3	solute carrier family 18 (vesicular acetylcholine), member 3	CCDS7231.1	chr10_50819436-50819436_C_T	217A>V	Substitution	Nonsynonymous coding	10%
MM12T	SLC19A1	solute carrier family 19 (folate transporter), member 1	ENST00000380014	chr21_46918460-46918460_C_T	201A>T	Substitution	Nonsynonymous coding	12%
MM12T	SLC1A1	solute carrier family 1 (neuronal/epithelial high affinity glutamate transporter, system Xag),	CCDS6452.1	chr9_4561494-4561494_G_A	93R>H	Substitution	Nonsynonymous coding	17%
MM12T	SLC1A2	solute carrier family 1 (glial high affinity glutamate transporter), member 2	CCDS31459.1	chr11_35339032-35339032_G_A	17R>X	Substitution	Nonsense	22%

MM12T	SLC1A3	solute carrier family 1 (glial high affinity glutamate transporter), member 3	CCDS3919.1	chr5_36608694-36608694_G_A	57A>T	Substitution	Nonsynonymous coding	13%
MM12T	SLC1A4	solute carrier family 1 (glutamate/neutral amino acid transporter), member 4	CCDS1879.1	chr2_65248251-65248251_G_A	524E>K	Substitution	Nonsynonymous coding	12%
MM12T	SLC22A12	solute carrier family 22 (organic anion/urate transporter), member 12	CCDS8075.1	chr11_64359249-64359249_C_T	74A>V	Substitution	Nonsynonymous coding	18%
MM12T	SLC22A13	solute carrier family 22 (organic anion transporter), member 13	CCDS2676.1	chr3_38316982-38316982_G_A	ISV+1>	Substitution	Splice site donor	27%
MM12T	SLC22A14	solute carrier family 22, member 14	CCDS2677.1	chr3_38350480-38350480_G_A	271A>T	Substitution	Nonsynonymous coding	14%
MM12T	SLC22A2	solute carrier family 22 (organic cation transporter), member 2	CCDS5276.1	chr6_160679669-160679669_C_T	41V>I	Substitution	Nonsynonymous coding	43%
MM12T	SLC22A23	solute carrier family 22, member 23	CCDS47363.1	chr6_3287102-3287102_G_T	513L>I	Substitution	Nonsynonymous coding	33%
MM12T	SLC22A3	solute carrier family 22 (extraneuronal monoamine transporter), member 3	CCDS5277.1	chr6_160858054-160858054_G_T	367G>X	Substitution	Nonsense	19%
MM12T	SLC23A2	solute carrier family 23 (nucleobase transporters), member 2	CCDS13085.1	chr20_4850569-4850569__G	NA	Insertion	Frameshift	23%
MM12T	SLC23A3	solute carrier family 23 (nucleobase transporters), member 3	CCDS46517.1	chr2_220034352-220034352_G_T	71L>I	Substitution	Nonsynonymous coding	44%
MM12T	SLC24A5	solute carrier family 24, member 5	CCDS10128.1	chr15_48429140-48429140_G_A	284G>D	Substitution	Nonsynonymous coding	19%
MM12T	SLC24A6	solute carrier family 24 (sodium/lithium/calcium exchanger), member	CCDS31909.1	chr12_113758883-113758883_G_T	114L>M	Substitution	Nonsynonymous coding	34%
MM12T	SLC24A6	solute carrier family 24 (sodium/lithium/calcium exchanger), member	CCDS31909.1	chr12_113759104-113759104_C_T	69R>Q	Substitution	Nonsynonymous coding	30%
MM12T	SLC25A10	solute carrier family 25 (mitochondrial carrier; dicarboxylate transporter), member 10	CCDS11786.1	chr17_79683073-79683073_C_T	138R>W	Substitution	Nonsynonymous coding	13%
MM12T	SLC25A10	solute carrier family 25 (mitochondrial carrier; dicarboxylate transporter), member 10	CCDS11786.1	chr17_79684448-79684448_C_T	185A>V	Substitution	Nonsynonymous coding	12%
MM12T	SLC25A11	solute carrier family 25 (mitochondrial carrier; oxoglutarate carrier), member 11	CCDS11059.1	chr17_4842151-4842151_G_T	123A>D	Substitution	Nonsynonymous coding	22%
MM12T	SLC25A15	solute carrier family 25 (mitochondrial carrier; ornithine transporter) member 15	CCDS9373.1	chr13_41373421-41373421_T_C	95V>A	Substitution	Nonsynonymous coding	22%
MM12T	SLC25A2	solute carrier family 25 (mitochondrial carrier; ornithine transporter) member 2	CCDS4258.1	chr5_140683408-140683408_C_T	9A>T	Substitution	Nonsynonymous coding	11%
MM12T	SLC25A22	solute carrier family 25 (mitochondrial carrier: glutamate), member 22	CCDS7715.1	chr11_791983-791983_C_T	302A>T	Substitution	Nonsynonymous coding	31%
MM12T	SLC25A31	solute carrier family 25 (mitochondrial carrier; adenine nucleotide translocator), member 31	CCDS3733.1	chr4_128694557-128694557_G_A	259R>Q	Substitution	Nonsynonymous coding	14%
MM12T	SLC25A33	solute carrier family 25 (pyrimidine nucleotide carrier), member 33	CCDS103.1	chr1_9630381-9630381_G_T	127S>I	Substitution	Nonsynonymous coding	35%
MM12T	SLC25A37	solute carrier family 25 (mitochondrial iron transporter), member 37	CCDS47828.1	chr8_23428918-23428918_G_A	189W>X	Substitution	Nonsense	29%
MM12T	SLC25A42	solute carrier family 25, member 42	CCDS32966.1	chr19_19212600-19212600_G_T	31R>M	Substitution	Nonsynonymous coding	29%
MM12T	SLC26A1	solute carrier family 26 (sulfate transporter), member 1	CCDS33934.1	chr4_985409-985409_C_T	28R>H	Substitution	Nonsynonymous coding	29%
MM12T	SLC26A5	solute carrier family 26, member 5 (prestin)	CCDS5733.1	chr7_103029497-103029497_G_A	491A>V	Substitution	Nonsynonymous coding	18%
MM12T	SLC26A8	solute carrier family 26, member 8	CCDS4813.1	chr6_35927290-35927290_C_T	604G>R	Substitution	Nonsynonymous coding	11%
MM12T	SLC26A8	solute carrier family 26, member 8	CCDS4813.1	chr6_35943175-35943175_C_T	422D>N	Substitution	Nonsynonymous coding	12%
MM12T	SLC26A9	solute carrier family 26, member 9	CCDS30989.1	chr1_205893572-205893572_G_A	479P>S	Substitution	Nonsynonymous coding	31%
MM12T	SLC27A5	solute carrier family 27 (fatty acid transporter), member 5	CCDS12983.1	chr19_59010523-59010523_G_A	578Q>X	Substitution	Nonsense	23%
MM12T	SLC29A3	solute carrier family 29 (nucleoside transporters), member 3	CCDS7310.1	chr10_73111521-73111521_A_G	196R>G	Substitution	Nonsynonymous coding	15%
MM12T	SLC2A11	solute carrier family 2 (facilitated glucose transporter), member 11	CCDS13818.1	chr22_24224651-24224651_C_T	238R>W	Substitution	Nonsynonymous coding	24%
MM12T	SLC2A11	solute carrier family 2 (facilitated glucose transporter), member 11	CCDS13818.1	chr22_24224711-24224711_G_A	258A>T	Substitution	Nonsynonymous coding	13%

MM12T	SLC2A11	solute carrier family 2 (facilitated glucose transporter), member 11	CCDS13818.1	chr22_24226169-24226169_G_A	381A>T	Substitution	Nonsynonymous coding	11%
MM12T	SLC2A11	solute carrier family 2 (facilitated glucose transporter), member 11	CCDS13818.1	chr22_24226491-24226491_G_A	396G>R	Substitution	Nonsynonymous coding	25%
MM12T	SLC30A6	solute carrier family 30 (zinc transporter), member 6	CCDS1780.1	chr2_32434629-32434629_T_C	295L>S	Substitution	Nonsynonymous coding	27%
MM12T	SLC32A1	solute carrier family 32 (GABA vesicular transporter), member 1	CCDS13307.1	chr20_37356749-37356749_G_A	349V>M	Substitution	Nonsynonymous coding	36%
MM12T	SLC34A1	solute carrier family 34 (sodium phosphate), member 1	CCDS4418.1	chr5_176814874-176814874_G_A	215R>Q	Substitution	Nonsynonymous coding	35%
MM12T	SLC35A4	solute carrier family 35, member A4	ENST00000432254	chr5_139946719-139946719_C_A	34P>H	Substitution	Nonsynonymous coding	12%
MM12T	SLC35B1	solute carrier family 35, member B1	CCDS11552.1	chr17_47783615-47783615_G_T	97A>D	Substitution	Nonsynonymous coding	29%
MM12T	SLC35C1	solute carrier family 35, member C1	CCDS7914.1	chr11_45832872-45832872_G_A	361A>T	Substitution	Nonsynonymous coding	23%
MM12T	SLC35D3	solute carrier family 35, member D3	CCDS34544.1	chr6_137245534-137245534_G_T	317Q>H	Substitution	Nonsynonymous coding	43%
MM12T	SLC35E2	solute carrier family 35, member E2	CCDS33.1	chr1_1663923-1663923_G_T	258L>M	Substitution	Nonsynonymous coding	29%
MM12T	SLC35F3	solute carrier family 35, member F3	CCDS1600.1	chr1_234444852-234444852_G_A	205C>Y	Substitution	Nonsynonymous coding	29%
MM12T	SLC35F3	solute carrier family 35, member F3	CCDS1600.1	chr1_234454650-234454650_C_A	370P>T	Substitution	Nonsynonymous coding	27%
MM12T	SLC36A4	solute carrier family 36 (proton/amino acid symporter), member 4	CCDS8291.1	chr11_92882002-92882002_C_T	406A>T	Substitution	Nonsynonymous coding	20%
MM12T	SLC38A10	solute carrier family 38, member 10	CCDS11780.1	chr17_79225281-79225281_C_T	693A>T	Substitution	Nonsynonymous coding	33%
MM12T	SLC38A10	solute carrier family 38, member 10	CCDS42397.1	chr17_79257295-79257295_C_T	91G>R	Substitution	Nonsynonymous coding	30%
MM12T	SLC38A7	solute carrier family 38, member 7	CCDS10800.1	chr16_58705015-58705015_G_A	389P>S	Substitution	Nonsynonymous coding	15%
MM12T	SLC39A14	solute carrier family 39 (zinc transporter), member 14	CCDS47823.1	chr8_22273701-22273701_G_A	352G>D	Substitution	Nonsynonymous coding	11%
MM12T	SLC39A3	solute carrier family 39 (zinc transporter), member 3	CCDS12093.1	chr19_2732930-2732930_C_T	255G>D	Substitution	Nonsynonymous coding	28%
MM12T	SLC39A4	solute carrier family 39 (zinc transporter), member 4	CCDS6424.1	chr8_145638307-145638307_C_T	551A>T	Substitution	Nonsynonymous coding	35%
MM12T	SLC39A4	solute carrier family 39 (zinc transporter), member 4	CCDS6424.1	chr8_145639424-145639424_C_T	402R>H	Substitution	Nonsynonymous coding	32%
MM12T	SLC44A3	solute carrier family 44, member 3	CCDS44176.1	chr1_95356734-95356734_G_A	511A>T	Substitution	Nonsynonymous coding	32%
MM12T	SLC44A4	solute carrier family 44, member 4	CCDS4724.2	chr6_31837015-31837015_G_A	384Q>X	Substitution	Nonsense	19%
MM12T	SLC44A5	solute carrier family 44, member 5	CCDS667.1	chr1_75702681-75702681_C_T	244W>X	Substitution	Nonsense	37%
MM12T	SLC45A1	solute carrier family 45, member 1	CCDS30577.1	chr1_8384693-8384693_G_A	102A>T	Substitution	Nonsynonymous coding	13%
MM12T	SLC45A4	solute carrier family 45, member 4	CCDS34948.1	chr8_142228211-142228211_G_A	459R>W	Substitution	Nonsynonymous coding	12%
MM12T	SLC47A1	solute carrier family 47, member 1	CCDS11209.1	chr17_19451350-19451350_C_T	120A>V	Substitution	Nonsynonymous coding	12%
MM12T	SLC47A1	solute carrier family 47, member 1	CCDS11209.1	chr17_19480757-19480757_C_T	535A>V	Substitution	Nonsynonymous coding	36%
MM12T	SLC4A1	solute carrier family 4, anion exchanger, member 1 (erythrocyte membrane protein	CCDS11481.1	chr17_42328912-42328912_C_T	786A>T	Substitution	Nonsynonymous coding	15%
MM12T	SLC4A11	solute carrier family 4, sodium borate transporter, member 11	CCDS13052.1	chr20_3211375-3211375_C_T	ISV+3>	Substitution	Splice site donor	13%
MM12T	SLC4A3	solute carrier family 4, anion exchanger, member 3	CCDS2446.1	chr2_220494796-220494796_C_T	205P>L	Substitution	Nonsynonymous coding	21%
MM12T	SLC4A3	solute carrier family 4, anion exchanger, member 3	ENST00000413743	chr2_220496084-220496084_G_A	42E>K	Substitution	Nonsynonymous coding	40%
MM12T	SLC4A3	solute carrier family 4, anion exchanger, member 3	CCDS2446.1	chr2_220500569-220500569_A_G	743Y>C	Substitution	Nonsynonymous coding	38%

MM12T	SLC4A8	solute carrier family 4, sodium bicarbonate cotransporter, member 8	CCDS44890.1	chr12_51847370-51847370_G_A	154S>N	Substitution	Nonsynonymous coding	37%
MM12T	SLC52A1	solute carrier family 52, riboflavin transporter, member 1	CCDS11066.1	chr17_4936283-4936283_C_T	439R>K	Substitution	Nonsynonymous coding	11%
MM12T	SLC52A1	solute carrier family 52, riboflavin transporter, member 1	CCDS11066.1	chr17_4937480-4937480_C_T	102V>M	Substitution	Nonsynonymous coding	33%
MM12T	SLC52A1	solute carrier family 52, riboflavin transporter, member 1	CCDS11066.1	chr17_4937507-4937507_C_T	93A>T	Substitution	Nonsynonymous coding	28%
MM12T	SLC52A2	solute carrier family 52, riboflavin transporter, member 2 [Source:HGNC Symbol;Acc:30224]	CCDS6423.1	chr8_145584478-145584478_C_A	381L>M	Substitution	Nonsynonymous coding	26%
MM12T	SLC5A1	solute carrier family 5 (sodium/glucose cotransporter), member 1	CCDS13902.1	chr22_32487710-32487710_G_A	414R>H	Substitution	Nonsynonymous coding	28%
MM12T	SLC5A10	solute carrier family 5 (sodium/glucose cotransporter), member 10	CCDS11201.2	chr17_18872684-18872684_G_A	197A>T	Substitution	Nonsynonymous coding	22%
MM12T	SLC5A10	solute carrier family 5 (sodium/glucose cotransporter), member 10	CCDS11201.2	chr17_18874506-18874506_C_T	274A>V	Substitution	Nonsynonymous coding	37%
MM12T	SLC5A10	solute carrier family 5 (sodium/glucose cotransporter), member 10	CCDS11201.2	chr17_18922861-18922861_C_T	472A>V	Substitution	Nonsynonymous coding	25%
MM12T	SLC5A12	solute carrier family 5 (sodium/glucose cotransporter), member 12	CCDS7860.2	chr11_26718735-26718735_G_A	339A>V	Substitution	Nonsynonymous coding	17%
MM12T	SLC5A12	solute carrier family 5 (sodium/glucose cotransporter), member 12	CCDS7860.2	chr11_26743015-26743015_C_A	83V>F	Substitution	Nonsynonymous coding	14%
MM12T	SLC5A4	solute carrier family 5 (low affinity glucose cotransporter), member 4	CCDS13903.1	chr22_32620389-32620389_A_C	510S>R	Substitution	Nonsynonymous coding	33%
MM12T	SLC5A9	solute carrier family 5 (sodium/glucose cotransporter), member 9	CCDS44136.1	chr1_48688517-48688517_G_A	37D>N	Substitution	Nonsynonymous coding	36%
MM12T	SLC5A9	solute carrier family 5 (sodium/glucose cotransporter), member 9	CCDS44136.1	chr1_48708154-48708154_G_A	593R>Q	Substitution	Nonsynonymous coding	26%
MM12T	SLC6A11	solute carrier family 6 (neurotransmitter transporter, GABA), member 11	CCDS2602.1	chr3_10858201-10858201_G_A	84G>E	Substitution	Nonsynonymous coding	11%
MM12T	SLC6A13	solute carrier family 6 (neurotransmitter transporter, GABA), member 13	CCDS8502.1	chr12_331753-331753_C_A	487R>M	Substitution	Nonsynonymous coding	22%
MM12T	SLC6A17	solute carrier family 6, member 17	CCDS30799.1	chr1_110740055-110740055_G_T	ISV-4>	Substitution	Splice site acceptor	36%
MM12T	SLC6A19	solute carrier family 6 (neutral amino acid transporter), member 19	CCDS34130.1	chr5_1213663-1213663_G_A	250G>D	Substitution	Nonsynonymous coding	29%
MM12T	SLC6A19	solute carrier family 6 (neutral amino acid transporter), member 19	CCDS34130.1	chr5_1214064-1214064_G_A	ISV-4>	Substitution	Splice site acceptor	40%
MM12T	SLC6A2	solute carrier family 6 (neurotransmitter transporter, noradrenalin), member 2	CCDS10754.1	chr16_55690785-55690785_G_A	60G>D	Substitution	Nonsynonymous coding	13%
MM12T	SLC6A2	solute carrier family 6 (neurotransmitter transporter, noradrenalin), member 2	CCDS10754.1	chr16_55732421-55732421_C_T	477A>V	Substitution	Nonsynonymous coding	27%
MM12T	SLC6A6	solute carrier family 6 (neurotransmitter transporter, taurine), member 6	ENST00000428884	chr3_14485478-14485478__C	NA	Insertion	Frameshift	21%
MM12T	SLC7A11	solute carrier family 7 (anionic amino acid transporter light chain, xc- system), member	CCDS3742.1	chr4_139101945-139101945_C_A	ISV-1>	Substitution	Splice site acceptor	24%
MM12T	SLC7A2	solute carrier family 7 (cationic amino acid transporter, y+ system), member 2	CCDS6002.2	chr8_17419560-17419560_G_A	577V>I	Substitution	Nonsynonymous coding	26%
MM12T	SLC7A3	solute carrier family 7 (cationic amino acid transporter, y+ system), member 3	CCDS14404.1	chrX_70148340-70148340_C_T	225E>K	Substitution	Nonsynonymous coding	24%
MM12T	SLC7A3	solute carrier family 7 (cationic amino acid transporter, y+ system), member 3	CCDS14404.1	chrX_70149828-70149828_C_T	7R>H	Substitution	Nonsynonymous coding	18%
MM12T	SLC7A4	solute carrier family 7 (orphan transporter), member 4	CCDS33608.1	chr22_21383697-21383697_C_T	554V>I	Substitution	Nonsynonymous coding	18%
MM12T	SLC7A4	solute carrier family 7 (orphan transporter), member 4	CCDS33608.1	chr22_21385306-21385306_G_A	266R>W	Substitution	Nonsynonymous coding	12%
MM12T	SLC7A8	solute carrier family 7 (amino acid transporter light chain, L system), member 8	CCDS9590.1	chr14_23607127-23607127_C_T	ISV+3>	Substitution	Splice site donor	28%
MM12T	SLC8A1	solute carrier family 8 (sodium/calcium exchanger), member 1	CCDS1806.1	chr2_40342707-40342707_C_T	870V>I	Substitution	Nonsynonymous coding	12%
MM12T	SLC9A3	solute carrier family 9, subfamily A (NHE3, cation proton antiporter 3), member 3	CCDS3855.1	chr5_524284-524284_C_T	52V>M	Substitution	Nonsynonymous coding	32%
MM12T	SLC9A3	solute carrier family 9, subfamily A (NHE3, cation proton antiporter 3), member 3	CCDS3855.1	chr5_474997-474997_C_T	ISV+1>	Substitution	Splice site donor	43%

MM12T	SLC9A5	solute carrier family 9, subfamily A (NHE5, cation proton antiporter 5), member 5	CCDS42178.1	chr16_67298418-67298418_G_A	669R>H	Substitution	Nonsynonymous coding	14%
MM12T	SLC9A8	solute carrier family 9, subfamily A (NHE8, cation proton antiporter 8), member 8	CCDS13421.1	chr20_48500445-48500445_C_T	445R>W	Substitution	Nonsynonymous coding	32%
MM12T	SLC9C2	solute carrier family 9, member C2 (putative)	CCDS1308.1	chr1_173517623-173517623_C_A	456V>L	Substitution	Nonsynonymous coding	16%
MM12T	SLCO1C1	solute carrier organic anion transporter family, member 1C1	CCDS8683.1	chr12_20868126-20868126_C_A	195L>I	Substitution	Nonsynonymous coding	35%
MM12T	SLCO2A1	solute carrier organic anion transporter family, member 2A1	CCDS3084.1	chr3_133666230-133666230_G_A	389R>C	Substitution	Nonsynonymous coding	28%
MM12T	SLCO3A1	solute carrier organic anion transporter family, member 3A1	CCDS10371.1	chr15_92706160-92706160_C_T	643T>I	Substitution	Nonsynonymous coding	32%
MM12T	SLCO4A1	solute carrier organic anion transporter family, member 4A1	CCDS13501.1	chr20_61288426-61288426_C_T	207T>M	Substitution	Nonsynonymous coding	37%
MM12T	SLCO6A1	solute carrier organic anion transporter family, member 6A1	CCDS34206.1	chr5_101755708-101755708_C_T	432G>R	Substitution	Nonsynonymous coding	15%
MM12T	SLFN11	schlafen family member 11	CCDS11294.1	chr17_33679635-33679635_C_T	816A>T	Substitution	Nonsynonymous coding	12%
MM12T	SLFN5	schlafen family member 5	CCDS32619.1	chr17_33586586-33586586_G_A	293V>I	Substitution	Nonsynonymous coding	23%
MM12T	SLFN5	schlafen family member 5	CCDS32619.1	chr17_33586718-33586718_C_T	337P>S	Substitution	Nonsynonymous coding	50%
MM12T	SLITRK2	SLIT and NTRK-like family, member 2	CCDS14680.1	chrX_144904451-144904451_C_A	170L>M	Substitution	Nonsynonymous coding	30%
MM12T	SLITRK3	SLIT and NTRK-like family, member 3	CCDS3197.1	chr3_164908116-164908116_G_A	168A>V	Substitution	Nonsynonymous coding	29%
MM12T	SLITRK4	SLIT and NTRK-like family, member 4	CCDS14679.1	chrX_142718108-142718108_G_A	273R>C	Substitution	Nonsynonymous coding	28%
MM12T	SLK	STE20-like kinase	CCDS7553.1	chr10_105770629-105770629_G_A	947R>H	Substitution	Nonsynonymous coding	27%
MM12T	SLMAP	sarcolemma associated protein	CCDS33774.1	chr3_57743509-57743509_C_T	44A>V	Substitution	Nonsynonymous coding	12%
MM12T	SLMAP	sarcolemma associated protein	CCDS33774.1	chr3_57908701-57908701_T_A	765L>H	Substitution	Nonsynonymous coding	25%
MM12T	SLX4	SLX4 structure-specific endonuclease subunit homolog (S. cerevisiae)	CCDS10506.2	chr16_3641270-3641270_G_T	790P>H	Substitution	Nonsynonymous coding	32%
MM12T	SLX4	SLX4 structure-specific endonuclease subunit homolog (S. cerevisiae)	CCDS10506.2	chr16_3642754-3642754_G_A	758A>V	Substitution	Nonsynonymous coding	33%
MM12T	SLX4	SLX4 structure-specific endonuclease subunit homolog (S. cerevisiae)	CCDS10506.2	chr16_3646198-3646198_G_A	627P>L	Substitution	Nonsynonymous coding	31%
MM12T	SLX4IP	SLX4 interacting protein	CCDS33439.1	chr20_10603686-10603686_C_T	296R>C	Substitution	Nonsynonymous coding	25%
MM12T	SMAD4	SMAD family member 4	CCDS11950.1	chr18_48581203-48581203_G_T	169Q>H	Substitution	Nonsynonymous coding	32%
MM12T	SMAD6	SMAD family member 6	CCDS10221.1	chr15_67073337-67073337_G_A	319A>T	Substitution	Nonsynonymous coding	53%
MM12T	SMAD7	SMAD family member 7	CCDS11936.1	chr18_46468866-46468866_G_T	243P>T	Substitution	Nonsynonymous coding	33%
MM12T	SMARCA1	SWI/SNF related, matrix associated, actin dependent regulator of chromatin, subfamily	CCDS14612.1	chrX_128640113-128640113_G_A	348H>Y	Substitution	Nonsynonymous coding	22%
MM12T	SMARCA4	SWI/SNF related, matrix associated, actin dependent regulator of chromatin, subfamily	CCDS45971.1	chr19_11134251-11134251_C_T	973R>W	Substitution	Nonsynonymous coding	28%
MM12T	SMARCA4	SWI/SNF related, matrix associated, actin dependent regulator of chromatin, subfamily	CCDS45971.1	chr19_11143994-11143994_G_A	1192R>H	Substitution	Nonsynonymous coding	34%
MM12T	SMARCA4	SWI/SNF related, matrix associated, actin dependent regulator of chromatin, subfamily	CCDS45971.1	chr19_11144522-11144522_T_C	1285L>S	Substitution	Nonsynonymous coding	37%
MM12T	SMARCA4	SWI/SNF related, matrix associated, actin dependent regulator of chromatin, subfamily	CCDS45971.1	chr19_11144847-11144847_C_T	1308R>W	Substitution	Nonsynonymous coding	13%
MM12T	SMARCA5	SWI/SNF related, matrix associated, actin dependent regulator of chromatin, subfamily	CCDS3761.1	chr4_144449106-144449106_T_C	296V>A	Substitution	Nonsynonymous coding	27%
MM12T	SMARCC1	SWI/SNF related, matrix associated, actin dependent regulator of chromatin, subfamily c,	CCDS2758.1	chr3_47787435-47787435_G_A	122L>F	Substitution	Nonsynonymous coding	21%
MM12T	SMARCC2	SWI/SNF related, matrix associated, actin dependent regulator of chromatin, subfamily c,	CCDS8907.1	chr12_56566406-56566406_C_T	578R>H	Substitution	Nonsynonymous coding	15%

MM12T	SMC1A	structural maintenance of chromosomes 1A	CCDS14352.1	chrX_53407587-53407587_T_A	1191E>V	Substitution	Nonsynonymous coding	19%
MM12T	SMC1A	structural maintenance of chromosomes 1A	CCDS14352.1	chrX_53430549-53430549_C_T	790R>Q	Substitution	Nonsynonymous coding	32%
MM12T	SMC1A	structural maintenance of chromosomes 1A	CCDS14352.1	chrX_53432057-53432057_C_A	695E>X	Substitution	Nonsense	29%
MM12T	SMC1B	structural maintenance of chromosomes 1B	CCDS43027.1	chr22_45755738-45755738_C_A	937D>Y	Substitution	Nonsynonymous coding	11%
MM12T	SMC2	structural maintenance of chromosomes 2	CCDS35086.1	chr9_106873998-106873998_G_A	386G>D	Substitution	Nonsynonymous coding	16%
MM12T	SMC2	structural maintenance of chromosomes 2	CCDS35086.1	chr9_106901462-106901462_A_T	1154N>Y	Substitution	Nonsynonymous coding	28%
MM12T	SMCR7	Smith-Magenis syndrome chromosome region, candidate 7	CCDS45624.1	chr17_18167182-18167182_G_A	168A>T	Substitution	Nonsynonymous coding	25%
MM12T	SMCR7L	Smith-Magenis syndrome chromosome region, candidate 7-like	CCDS13995.1	chr22_39907349-39907349_G_A	17G>D	Substitution	Nonsynonymous coding	14%
MM12T	SMEK2	SMEK homolog 2, suppressor of mek1 (Dictyostelium)	CCDS46289.1	chr2_55831169-55831169_G_T	81A>D	Substitution	Nonsynonymous coding	10%
MM12T	SMG1	smg-1 homolog, phosphatidylinositol 3-kinase-related kinase (C. elegans)	CCDS45430.1	chr16_18853743-18853743_G_A	2085R>C	Substitution	Nonsynonymous coding	22%
MM12T	SMG7	smg-7 homolog, nonsense mediated mRNA decay factor (C. elegans)	CCDS1355.1	chr1_183502894-183502894_G_A	364V>I	Substitution	Nonsynonymous coding	27%
MM12T	SMO	smoothened, frizzled family receptor	CCDS5811.1	chr7_128846088-128846088_C_T	340H>Y	Substitution	Nonsynonymous coding	30%
MM12T	SMOX	spermine oxidase	CCDS13075.1	chr20_4158003-4158003_G_A	72A>T	Substitution	Nonsynonymous coding	33%
MM12T	SMPD4	sphingomyelin phosphodiesterase 4, neutral membrane (neutral sphingomyelinase-3)	CCDS42751.1	chr2_130911948-130911948_C_T	560V>I	Substitution	Nonsynonymous coding	10%
MM12T	SMPDL3B	sphingomyelin phosphodiesterase, acid-like 3B	CCDS30655.1	chr1_28282307-28282307_G_A	268R>H	Substitution	Nonsynonymous coding	29%
MM12T	SMTN	smoothelin	CCDS13887.1	chr22_31486821-31486821_G_A	298R>H	Substitution	Nonsynonymous coding	10%
MM12T	SMURF1	SMAD specific E3 ubiquitin protein ligase 1	CCDS34690.1	chr7_98655044-98655044_C_T	112G>R	Substitution	Nonsynonymous coding	29%
MM12T	SMYD4	SET and MYND domain containing 4	CCDS11013.1	chr17_1703966-1703966_G_T	241P>H	Substitution	Nonsynonymous coding	31%
MM12T	SMYD5	SMYD family member 5	CCDS33221.2	chr2_73447910-73447910_G_A	156R>K	Substitution	Nonsynonymous coding	11%
MM12T	SNAP47	synaptosomal-associated protein, 47kDa	CCDS1562.1	chr1_227935616-227935616_C_T	105A>V	Substitution	Nonsynonymous coding	10%
MM12T	SNAP91	synaptosomal-associated protein, 91kDa	CCDS47455.1	chr6_84326684-84326684_G_A	ISV+4>	Substitution	Splice site donor	14%
MM12T	SNAPC4	small nuclear RNA activating complex, polypeptide 4, 190kDa	CCDS6998.1	chr9_139275306-139275306_C_T	ISV-4>	Substitution	Splice site acceptor	10%
MM12T	SND1	staphylococcal nuclease and tudor domain containing 1	CCDS34747.1	chr7_127714723-127714723_C_T	650A>V	Substitution	Nonsynonymous coding	23%
MM12T	SNRK	SNF related kinase	CCDS43075.1	chr3_43389650-43389650_G_A	633M>I	Substitution	Nonsynonymous coding	31%
MM12T	SNRNP27	small nuclear ribonucleoprotein 27kDa (U4/U6.U5)	CCDS33219.1	chr2_70122271-70122271_G_A	27R>H	Substitution	Nonsynonymous coding	11%
MM12T	SNRNP35	small nuclear ribonucleoprotein 35kDa (U11/U12)	CCDS45005.1	chr12_123950688-123950688_C_T	206R>X	Substitution	Nonsense	33%
MM12T	SNRPA	small nuclear ribonucleoprotein polypeptide A	CCDS12565.1	chr19_41270935-41270935_C_T	238R>C	Substitution	Nonsynonymous coding	15%
MM12T	SNTG2	syntrophin, gamma 2	CCDS46220.1	chr2_1204826-1204826_C_T	210P>L	Substitution	Nonsynonymous coding	43%
MM12T	SNTG2	syntrophin, gamma 2	CCDS46220.1	chr2_1204861-1204861_G_A	222V>M	Substitution	Nonsynonymous coding	21%
MM12T	SNX14	sorting nexin 14	CCDS5004.1	chr6_86259447-86259447_T_C	262D>G	Substitution	Nonsynonymous coding	66%
MM12T	SNX18	sorting nexin 18	CCDS3962.1	chr5_53814044-53814044_C_T	88P>S	Substitution	Nonsynonymous coding	12%
MM12T	SNX19	sorting nexin 19	CCDS31721.1	chr11_130780269-130780269_C_	NA	Deletion	Splice site acceptor	50%

MM12T	SNX20	sorting nexin 20	CCDS10745.1	chr16_50707697-50707697_C_T	191E>K	Substitution	Nonsynonymous coding	30%
MM12T	SNX8	sorting nexin 8	CCDS5331.1	chr7_2314807-2314807_G_A	120L>F	Substitution	Nonsynonymous coding	30%
MM12T	SOAT1	sterol O-acyltransferase 1	CCDS1330.1	chr1_179312069-179312069_G_A	311S>N	Substitution	Nonsynonymous coding	32%
MM12T	SOCS4	suppressor of cytokine signaling 4	CCDS9722.1	chr14_55510313-55510313_T_C	185I>T	Substitution	Nonsynonymous coding	28%
MM12T	SOLH	small optic lobes homolog (Drosophila)	CCDS10410.1	chr16_597515-597515_C_T	226P>L	Substitution	Nonsynonymous coding	28%
MM12T	SOLH	small optic lobes homolog (Drosophila)	CCDS10410.1	chr16_598073-598073_G_A	412R>H	Substitution	Nonsynonymous coding	21%
MM12T	SOLH	small optic lobes homolog (Drosophila)	CCDS10410.1	chr16_599161-599161_C_T	540R>W	Substitution	Nonsynonymous coding	26%
MM12T	SORBS2	sorbin and SH3 domain containing 2	CCDS3845.1	chr4_186544848-186544848_C_T	575A>T	Substitution	Nonsynonymous coding	12%
MM12T	SORBS2	sorbin and SH3 domain containing 2	CCDS3845.1	chr4_186545595-186545595_C_T	326G>S	Substitution	Nonsynonymous coding	42%
MM12T	SORBS3	sorbin and SH3 domain containing 3	CCDS6031.1	chr8_22414269-22414269_C_T	88P>S	Substitution	Nonsynonymous coding	30%
MM12T	SORCS1	sortilin-related VPS10 domain containing receptor 1	CCDS31283.1	chr10_108459081-108459081_G_A	435T>M	Substitution	Nonsynonymous coding	29%
MM12T	SORCS1	sortilin-related VPS10 domain containing receptor 1	CCDS31283.1	chr10_108923830-108923830_C_T	152R>H	Substitution	Nonsynonymous coding	14%
MM12T	SORCS2	sortilin-related VPS10 domain containing receptor 2	CCDS47008.1	chr4_7640121-7640121_G_A	239A>T	Substitution	Nonsynonymous coding	13%
MM12T	SORCS3	sortilin-related VPS10 domain containing receptor 3	CCDS7558.1	chr10_106401468-106401468_C_T	128A>V	Substitution	Nonsynonymous coding	10%
MM12T	SORCS3	sortilin-related VPS10 domain containing receptor 3	CCDS7558.1	chr10_106907418-106907418_C_T	449A>V	Substitution	Nonsynonymous coding	11%
MM12T	SORCS3	sortilin-related VPS10 domain containing receptor 3	CCDS7558.1	chr10_106976859-106976859_G_A	905V>I	Substitution	Nonsynonymous coding	12%
MM12T	SORCS3	sortilin-related VPS10 domain containing receptor 3	CCDS7558.1	chr10_107015554-107015554_C_T	1111T>M	Substitution	Nonsynonymous coding	25%
MM12T	SORL1	sortilin-related receptor, L(DLR class) A repeats containing	CCDS8436.1	chr11_121323237-121323237_C_T	66A>V	Substitution	Nonsynonymous coding	12%
MM12T	SORL1	sortilin-related receptor, L(DLR class) A repeats containing	CCDS8436.1	chr11_121340771-121340771_G_A	114S>N	Substitution	Nonsynonymous coding	11%
MM12T	SOST	sclerostin	CCDS11468.1	chr17_41832976-41832976_G_A	126R>X	Substitution	Nonsense	11%
MM12T	SOWAHA	sosondowah ankyrin repeat domain family member A	CCDS43361.1	chr5_132149972-132149972_C_T	220P>L	Substitution	Nonsynonymous coding	46%
MM12T	SOWAHC	sosondowah ankyrin repeat domain family member C	CCDS33270.1	chr2_110372503-110372503_C_T	146P>L	Substitution	Nonsynonymous coding	31%
MM12T	SOWAHD	sosondowah ankyrin repeat domain family member D	CCDS43984.1	chrX_118892650-118892650_C_T	7A>V	Substitution	Nonsynonymous coding	33%
MM12T	SOX10	SRY (sex determining region Y)-box 10	CCDS13964.1	chr22_38374089-38374089_C_T	161R>H	Substitution	Nonsynonymous coding	15%
MM12T	SOX10	SRY (sex determining region Y)-box 10	CCDS13964.1	chr22_38374108-38374108_C_T	155E>K	Substitution	Nonsynonymous coding	25%
MM12T	SOX3	SRY (sex determining region Y)-box 3	CCDS14669.1	chrX_139587182-139587182_C_T	15R>Q	Substitution	Nonsynonymous coding	26%
MM12T	SOX30	SRY (sex determining region Y)-box 30	CCDS4339.1	chr5_157073704-157073704_C_T	443R>H	Substitution	Nonsynonymous coding	22%
MM12T	SOX30	SRY (sex determining region Y)-box 30	CCDS4339.1	chr5_157078524-157078524_G_A	188A>V	Substitution	Nonsynonymous coding	34%
MM12T	SOX5	SRY (sex determining region Y)-box 5	CCDS8699.1	chr12_23689427-23689427_G_A	650R>C	Substitution	Nonsynonymous coding	28%
MM12T	SOX6	SRY (sex determining region Y)-box 6	CCDS7821.1	chr11_16036587-16036587_G_A	545R>C	Substitution	Nonsynonymous coding	16%
MM12T	SP100	SP100 nuclear antigen	CCDS42832.1	chr2_231368952-231368952_C_A	606P>H	Substitution	Nonsynonymous coding	26%
MM12T	SP6	Sp6 transcription factor	CCDS11520.1	chr17_45925003-45925003_A_G	265Y>H	Substitution	Nonsynonymous coding	21%

MM12T	SP7	Sp7 transcription factor	CCDS44897.1	chr12_53722544-53722544_C_T	228V>I	Substitution	Nonsynonymous coding	27%
MM12T	SPAG16	sperm associated antigen 16	CCDS2396.1	chr2_214727290-214727290_C_A	384C>X	Substitution	Nonsense	10%
MM12T	SPAG4	sperm associated antigen 4	CCDS13259.1	chr20_34206016-34206016_G_A	182W>X	Substitution	Nonsense	10%
MM12T	SPAG5	sperm associated antigen 5	CCDS32594.1	chr17_26919165-26919165_G_A	366P>L	Substitution	Nonsynonymous coding	11%
MM12T	SPAG8	sperm associated antigen 8	CCDS6592.1	chr9_35811669-35811669_G_A	125T>I	Substitution	Nonsynonymous coding	36%
MM12T	SPAG9	sperm associated antigen 9	CCDS45740.1	chr17_49075896-49075896_A_G	583S>P	Substitution	Nonsynonymous coding	26%
MM12T	SPAG9	sperm associated antigen 9	CCDS45740.1	chr17_49124752-49124752_C_T	192A>T	Substitution	Nonsynonymous coding	19%
MM12T	SPAM1	sperm adhesion molecule 1 (PH-20 hyaluronidase, zona pellucida binding)	CCDS5790.1	chr7_123594460-123594460_G_A	279R>H	Substitution	Nonsynonymous coding	12%
MM12T	SPARC	secreted protein, acidic, cysteine-rich (osteonectin)	CCDS4318.1	chr5_151047030-151047030_G_A	195R>W	Substitution	Nonsynonymous coding	36%
MM12T	SPARC	secreted protein, acidic, cysteine-rich (osteonectin)	CCDS4318.1	chr5_151047092-151047092_A_G	174V>A	Substitution	Nonsynonymous coding	11%
MM12T	SPARCL1	SPARC-like 1 (hevin)	CCDS3622.1	chr4_88403695-88403695_C_T	517D>N	Substitution	Nonsynonymous coding	24%
MM12T	SPATA13	spermatogenesis associated 13	NM_001166271	chr13_24798688-24798688_G_A	541A>T	Substitution	Nonsynonymous coding	12%
MM12T	SPATA13	spermatogenesis associated 13	NM_001166271	chr13_24823718-24823718_G_T	586R>M	Substitution	Nonsynonymous coding	35%
MM12T	SPATA16	spermatogenesis associated 16	CCDS3221.1	chr3_172694817-172694817_A_G	292F>L	Substitution	Nonsynonymous coding	10%
MM12T	SPATA2	spermatogenesis associated 2	CCDS13422.1	chr20_48523129-48523129_C_T	197R>H	Substitution	Nonsynonymous coding	32%
MM12T	SPATA20	spermatogenesis associated 20	CCDS11571.1	chr17_48626648-48626648_G_T	221W>L	Substitution	Nonsynonymous coding	38%
MM12T	SPATA21	spermatogenesis associated 21	CCDS172.1	chr1_16748027-16748027_C_A	41A>S	Substitution	Nonsynonymous coding	31%
MM12T	SPATA2L	spermatogenesis associated 2-like	CCDS10985.1	chr16_89763869-89763869_G_A	383A>V	Substitution	Nonsynonymous coding	36%
MM12T	SPATA31D1	SPATA31 subfamily D, member 1	CCDS47986.1	chr9_84610019-84610019_G_A	1545S>N	Substitution	Nonsynonymous coding	28%
MM12T	SPATA31E1	SPATA31 subfamily E, member 1	CCDS6676.1	chr9_90500483-90500483_G_A	361V>M	Substitution	Nonsynonymous coding	36%
MM12T	SPATA31E1	SPATA31 subfamily E, member 1	CCDS6676.1	chr9_90500586-90500586_G_T	395G>V	Substitution	Nonsynonymous coding	31%
MM12T	SPATA31E1	SPATA31 subfamily E, member 1	CCDS6676.1	chr9_90501786-90501786_C_A	795A>D	Substitution	Nonsynonymous coding	30%
MM12T	SPATA31E1	SPATA31 subfamily E, member 1	CCDS6676.1	chr9_90502844-90502844_G_T	1148A>S	Substitution	Nonsynonymous coding	14%
MM12T	SPATA5	spermatogenesis associated 5	CCDS3730.1	chr4_123855860-123855860_C_T	372P>S	Substitution	Nonsynonymous coding	41%
MM12T	SPATA5L1	spermatogenesis associated 5-like 1	CCDS10123.1	chr15_45706841-45706841_C_A	503L>I	Substitution	Nonsynonymous coding	15%
MM12T	SPATA6	spermatogenesis associated 6	CCDS551.1	chr1_48877222-48877222_C_T	107D>N	Substitution	Nonsynonymous coding	19%
MM12T	SPATA6L	spermatogenesis associated 6-like	CCDS43785.1	chr9_4629171-4629171__AAAA	NA	Insertion	Splice site acceptor	50%
MM12T	SPATA7	spermatogenesis associated 7	CCDS9883.1	chr14_88892912-88892912_C_T	237Q>X	Substitution	Nonsense	30%
MM12T	SPEF2	sperm flagellar 2	CCDS43309.1	chr5_35697801-35697801_C_T	683R>C	Substitution	Nonsynonymous coding	22%
MM12T	SPEG	SPEG complex locus	CCDS42824.1	chr2_220315912-220315912_T_C	723V>A	Substitution	Nonsynonymous coding	19%
MM12T	SPEG	SPEG complex locus	CCDS42824.1	chr2_220355664-220355664_C_T	3124T>M	Substitution	Nonsynonymous coding	39%
MM12T	SPEM1	spermatid maturation 1	CCDS42254.1	chr17_7324682-7324682_G_A	230A>T	Substitution	Nonsynonymous coding	30%

MM12T	SPEN	spen homolog, transcriptional regulator (Drosophila)	CCDS164.1	chr1_16242743-16242743_G_A	455R>H	Substitution	Nonsynonymous coding	10%
MM12T	SPERT	spermatid associated	CCDS9399.1	chr13_46287386-46287386_G_A	76A>T	Substitution	Nonsynonymous coding	12%
MM12T	SPERT	spermatid associated	CCDS9399.1	chr13_46287966-46287966_C_T	269A>V	Substitution	Nonsynonymous coding	26%
MM12T	SPG7	spastic paraplegia 7 (pure and complicated autosomal recessive)	CCDS10977.1	chr16_89611163-89611163_G_A	478D>N	Substitution	Nonsynonymous coding	13%
MM12T	SPHK2	sphingosine kinase 2	CCDS12727.1	chr19_49132189-49132189_G_A	375R>H	Substitution	Nonsynonymous coding	14%
MM12T	SPHKAP	SPHK1 interactor, AKAP domain containing	CCDS46537.1	chr2_228883658-228883658_C_A	638D>Y	Substitution	Nonsynonymous coding	18%
MM12T	SPN	sialophorin	CCDS10650.1	chr16_29675116-29675116_G_T	23A>S	Substitution	Nonsynonymous coding	29%
MM12T	SPNS3	spinster homolog 3 (Drosophila)	CCDS11045.1	chr17_4389519-4389519_G_A	ISV-4>	Substitution	Splice site acceptor	12%
MM12T	SPOCK3	sparc/osteonectin, cwcv and kazal-like domains proteoglycan (testican) 3	CCDS34095.1	chr4_167675659-167675659_C_A	311D>Y	Substitution	Nonsynonymous coding	26%
MM12T	SPON1	spondin 1, extracellular matrix protein	ENST00000310358	chr11_14287120-14287120_G_A	770G>R	Substitution	Nonsynonymous coding	27%
MM12T	SPRED1	sprouty-related, EVH1 domain containing 1	CCDS32193.1	chr15_38632038-38632038_C_T	175P>L	Substitution	Nonsynonymous coding	24%
MM12T	SPRED3	sprouty-related, EVH1 domain containing 3	CCDS42560.1	chr19_38886167-38886167_G_T	205E>D	Substitution	Nonsynonymous coding	17%
MM12T	SPTA1	spectrin, alpha, erythrocytic 1 (elliptocytosis 2)	CCDS41423.1	chr1_158607971-158607971_C_T	1681V>I	Substitution	Nonsynonymous coding	19%
MM12T	SPTAN1	spectrin, alpha, non-erythrocytic 1	CCDS48036.1	chr9_131331175-131331175_G_A	121R>Q	Substitution	Nonsynonymous coding	27%
MM12T	SPTAN1	spectrin, alpha, non-erythrocytic 1	CCDS48036.1	chr9_131339422-131339422_C_T	267T>I	Substitution	Nonsynonymous coding	31%
MM12T	SPTAN1	spectrin, alpha, non-erythrocytic 1	CCDS48036.1	chr9_131339764-131339764_C_T	355A>V	Substitution	Nonsynonymous coding	35%
MM12T	SPTAN1	spectrin, alpha, non-erythrocytic 1	CCDS48036.1	chr9_131395555-131395555_G_A	2459R>H	Substitution	Nonsynonymous coding	34%
MM12T	SPTB	spectrin, beta, erythrocytic	CCDS32099.1	chr14_65237714-65237714_C_T	1896R>Q	Substitution	Nonsynonymous coding	14%
MM12T	SPTB	spectrin, beta, erythrocytic	CCDS32099.1	chr14_65253362-65253362_C_A	1107K>N	Substitution	Nonsynonymous coding	31%
MM12T	SPTBN2	spectrin, beta, non-erythrocytic 2	CCDS8150.1	chr11_66460504-66460504_G_A	1641A>V	Substitution	Nonsynonymous coding	35%
MM12T	SPTBN5	spectrin, beta, non-erythrocytic 5	NM_016642	chr15_42144411-42144411_C_T	3483G>E	Substitution	Nonsynonymous coding	17%
MM12T	SPTBN5	spectrin, beta, non-erythrocytic 5	NM_016642	chr15_42174166-42174166_G_A	774R>W	Substitution	Nonsynonymous coding	18%
MM12T	SQLE	squalene epoxidase	CCDS47918.1	chr8_126011860-126011860_G_A	72G>D	Substitution	Nonsynonymous coding	10%
MM12T	SQSTM1	sequestosome 1	CCDS34317.1	chr5_179251300-179251300_G_A	217R>H	Substitution	Nonsynonymous coding	26%
MM12T	SQSTM1	sequestosome 1	CCDS34317.1	chr5_179263445-179263445_C_A	392P>Q	Substitution	Nonsynonymous coding	12%
MM12T	SRC	v-src sarcoma (Schmidt-Ruppin A-2) viral oncogene homolog (avian)	CCDS13294.1	chr20_36031642-36031642_C_T	491P>S	Substitution	Nonsynonymous coding	24%
MM12T	SRCAP	Snf2-related CREBBP activator protein	CCDS10689.2	chr16_30740771-30740771_G_A	2002R>H	Substitution	Nonsynonymous coding	35%
MM12T	SRD5A2	steroid-5-alpha-reductase, alpha polypeptide 2 (3-oxo-5 alpha-steroid delta 4-dehydrogenase	ENST00000233139	chr2_31758684-31758684_C_T	144R>Q	Substitution	Nonsynonymous coding	39%
MM12T	SREBF1	sterol regulatory element binding transcription factor 1	CCDS32583.1	chr17_17718056-17718056_C_T	900D>N	Substitution	Nonsynonymous coding	13%
MM12T	SREBF1	sterol regulatory element binding transcription factor 1	CCDS32583.1	chr17_17723791-17723791_G_T	76L>I	Substitution	Nonsynonymous coding	11%
MM12T	SREK1	splicing regulatory glutamine/lysine-rich protein 1	CCDS43323.1	chr5_65449296-65449296_C_T	56A>V	Substitution	Nonsynonymous coding	28%
MM12T	SRF	serum response factor (c-fos serum response element-binding transcription factor)	CCDS4889.1	chr6_43146861-43146861_G_A	487S>N	Substitution	Nonsynonymous coding	19%

MM12T	SRPK1	SRSF protein kinase 1	CCDS47415.1	chr6_35810384-35810384_A_	NA	Deletion	Splice site acceptor	35%
MM12T	SRRM2	serine/arginine repetitive matrix 2	CCDS32373.1	chr16_2809656-2809656_G_A	276R>H	Substitution	Nonsynonymous coding	68%
MM12T	SRRM2	serine/arginine repetitive matrix 2	CCDS32373.1	chr16_2814674-2814674_G_A	1382S>N	Substitution	Nonsynonymous coding	19%
MM12T	SRRM2	serine/arginine repetitive matrix 2	CCDS32373.1	chr16_2816519-2816519_G_A	1997R>H	Substitution	Nonsynonymous coding	34%
MM12T	SRRM3	serine/arginine repetitive matrix 3	ENST00000388802	chr7_75914934-75914934__A	NA	Insertion	Frameshift	21%
MM12T	SRRM4	serine/arginine repetitive matrix 4	CCDS44994.1	chr12_119583185-119583185_G_A	ISV-1>	Substitution	Splice site acceptor	25%
MM12T	SRRT	serrate RNA effector molecule homolog (Arabidopsis)	CCDS34709.1	chr7_100478937-100478937_C_T	52R>W	Substitution	Nonsynonymous coding	39%
MM12T	SRRT	serrate RNA effector molecule homolog (Arabidopsis)	CCDS34709.1	chr7_100479421-100479421_G_T	131Q>H	Substitution	Nonsynonymous coding	35%
MM12T	SRRT	serrate RNA effector molecule homolog (Arabidopsis)	CCDS34709.1	chr7_100483914-100483914_G_A	502R>Q	Substitution	Nonsynonymous coding	44%
MM12T	SRRT	serrate RNA effector molecule homolog (Arabidopsis)	CCDS34709.1	chr7_100485033-100485033_C_T	690R>C	Substitution	Nonsynonymous coding	19%
MM12T	SRSF10	serine/arginine-rich splicing factor 10	CCDS30630.1	chr1_24298091-24298091_G_A	155R>C	Substitution	Nonsynonymous coding	33%
MM12T	SRSF5	serine/arginine-rich splicing factor 5	CCDS32109.1	chr14_70237227-70237227_C_T	137A>V	Substitution	Nonsynonymous coding	10%
MM12T	SRSF6	serine/arginine-rich splicing factor 6	CCDS13318.1	chr20_42089213-42089213_C_T	217R>X	Substitution	Nonsense	33%
MM12T	SSFA2	sperm specific antigen 2	CCDS46467.1	chr2_182767188-182767188_G_A	470E>K	Substitution	Nonsynonymous coding	13%
MM12T	SSH1	slingshot homolog 1 (Drosophila)	CCDS9121.1	chr12_109246447-109246447_G_A	26A>V	Substitution	Nonsynonymous coding	32%
MM12T	SSPN	sarcospan	CCDS8707.1	chr12_26383695-26383695_G_A	140V>M	Substitution	Nonsynonymous coding	48%
MM12T	SSPO	SCO-spondin homolog (Bos taurus)	ENST00000378016	chr7_149473608-149473608_C_A	75P>H	Substitution	Nonsynonymous coding	43%
MM12T	SSPO	SCO-spondin homolog (Bos taurus)	ENST00000378016	chr7_149479281-149479281_G_T	598K>N	Substitution	Nonsynonymous coding	10%
MM12T	SSPO	SCO-spondin homolog (Bos taurus)	ENST00000378016	chr7_149482682-149482682_C_T	1033P>L	Substitution	Nonsynonymous coding	30%
MM12T	SSPO	SCO-spondin homolog (Bos taurus)	ENST00000378016	chr7_149486412-149486412_G_A	1463R>H	Substitution	Nonsynonymous coding	24%
MM12T	SSPO	SCO-spondin homolog (Bos taurus)	ENST00000378016	chr7_149489009-149489009_C_T	1784R>C	Substitution	Nonsynonymous coding	31%
MM12T	SSSCA1	Sjogren syndrome/scleroderma autoantigen 1	CCDS8104.1	chr11_65338915-65338915_G_A	104A>T	Substitution	Nonsynonymous coding	16%
MM12T	SSTR1	somatostatin receptor 1	CCDS9666.1	chr14_38678847-38678847_C_T	85R>C	Substitution	Nonsynonymous coding	13%
MM12T	SSTR2	somatostatin receptor 2	CCDS11691.1	chr17_71165931-71165931_C_T	158T>M	Substitution	Nonsynonymous coding	34%
MM12T	SSU72	SSU72 RNA polymerase II CTD phosphatase homolog (S. cerevisiae)	ENST00000378725	chr1_1480140-1480140_G_A	156A>V	Substitution	Nonsynonymous coding	33%
MM12T	ST14	suppression of tumorigenicity 14 (colon carcinoma)	CCDS8487.1	chr11_130064600-130064600_C_A	361P>T	Substitution	Nonsynonymous coding	24%
MM12T	ST18	suppression of tumorigenicity 18 (breast carcinoma) (zinc finger protein)	CCDS6149.1	chr8_53126764-53126764_C_A	18E>D	Substitution	Nonsynonymous coding	55%
MM12T	ST3GAL1	ST3 beta-galactoside alpha-2,3-sialyltransferase 1	CCDS6373.1	chr8_134478251-134478251_G_A	130P>L	Substitution	Nonsynonymous coding	30%
MM12T	ST3GAL1	ST3 beta-galactoside alpha-2,3-sialyltransferase 1	CCDS6373.1	chr8_134488066-134488066_G_A	68R>C	Substitution	Nonsynonymous coding	11%
MM12T	ST5	suppression of tumorigenicity 5	CCDS7791.1	chr11_8734265-8734265_C_T	669V>I	Substitution	Nonsynonymous coding	23%
MM12T	ST5	suppression of tumorigenicity 5	CCDS7791.1	chr11_8752500-8752500_C_T	113A>T	Substitution	Nonsynonymous coding	22%
MM12T	ST6GAL2	ST6 beta-galactosamide alpha-2,6-sialyltranferase 2	CCDS2073.1	chr2_107450522-107450522_G_A	342R>C	Substitution	Nonsynonymous coding	33%

MM12T	STAB1	stabilin 1	CCDS33768.1	chr3_52543952-52543952_C_T	805T>M	Substitution	Nonsynonymous coding	28%
MM12T	STAB1	stabilin 1	CCDS33768.1	chr3_52553967-52553967_G_A	1748G>D	Substitution	Nonsynonymous coding	14%
MM12T	STAB1	stabilin 1	CCDS33768.1	chr3_52554898-52554898_G_A	1929V>I	Substitution	Nonsynonymous coding	10%
MM12T	STAB1	stabilin 1	CCDS33768.1	chr3_52556610-52556610_G_A	2217G>D	Substitution	Nonsynonymous coding	11%
MM12T	STAB2	stabilin 2	CCDS31888.1	chr12_104125410-104125410_A_G	1760I>M	Substitution	Nonsynonymous coding	13%
MM12T	STAB2	stabilin 2	CCDS31888.1	chr12_104140477-104140477_G_A	2080C>Y	Substitution	Nonsynonymous coding	16%
MM12T	STAB2	stabilin 2	CCDS31888.1	chr12_104149237-104149237_G_T	2291R>L	Substitution	Nonsynonymous coding	26%
MM12T	STAC2	SH3 and cysteine rich domain 2	CCDS11335.1	chr17_37371055-37371055_G_A	278P>S	Substitution	Nonsynonymous coding	44%
MM12T	STAG2	stromal antigen 2	CCDS43990.1	chrX_123200109-123200109_G_T	727E>D	Substitution	Nonsynonymous coding	25%
MM12T	STAM2	signal transducing adaptor molecule (SH3 domain and ITAM motif) 2	CCDS2196.1	chr2_152992050-152992050_T_C	258T>A	Substitution	Nonsynonymous coding	22%
MM12T	STARD10	StAR-related lipid transfer (START) domain containing 10	CCDS41688.1	chr11_72466018-72466018_G_A	267A>V	Substitution	Nonsynonymous coding	17%
MM12T	STARD10	StAR-related lipid transfer (START) domain containing 10	CCDS41688.1	chr11_72469649-72469649_C_T	129D>N	Substitution	Nonsynonymous coding	26%
MM12T	STARD13	StAR-related lipid transfer (START) domain containing 13	CCDS9348.1	chr13_33684173-33684173_G_A	962P>S	Substitution	Nonsynonymous coding	20%
MM12T	STARD13	StAR-related lipid transfer (START) domain containing 13	CCDS9348.1	chr13_33686985-33686985_G_A	789L>F	Substitution	Nonsynonymous coding	14%
MM12T	STARD3	StAR-related lipid transfer (START) domain containing 3	CCDS11341.1	chr17_37814233-37814233_C_T	128T>M	Substitution	Nonsynonymous coding	31%
MM12T	STARD8	StAR-related lipid transfer (START) domain containing 8	CCDS48134.1	chrX_67936250-67936250_G_A	92E>K	Substitution	Nonsynonymous coding	11%
MM12T	STARD9	StAR-related lipid transfer (START) domain containing 9	NM_020759	chr15_42875595-42875595__A	NA	Insertion	Frameshift	31%
MM12T	STARD9	StAR-related lipid transfer (START) domain containing 9	NM_020759	chr15_42953423-42953423_G_T	257S>I	Substitution	Nonsynonymous coding	30%
MM12T	STAT1	signal transducer and activator of transcription 1, 91kDa	CCDS2309.1	chr2_191841592-191841592_C_T	678G>E	Substitution	Nonsynonymous coding	13%
MM12T	STAT3	signal transducer and activator of transcription 3 (acute-phase response factor)	CCDS32656.1	chr17_40497671-40497671_C_T	93R>K	Substitution	Nonsynonymous coding	20%
MM12T	STAT4	signal transducer and activator of transcription 4	CCDS2310.1	chr2_192011486-192011486_A_	NA	Deletion	Splice site acceptor	44%
MM12T	STAT5A	signal transducer and activator of transcription 5A	CCDS11424.1	chr17_40457713-40457713_C_T	489A>V	Substitution	Nonsynonymous coding	21%
MM12T	STAT6	signal transducer and activator of transcription 6, interleukin-4 induced	CCDS8931.1	chr12_57501408-57501408_G_T	79Q>K	Substitution	Nonsynonymous coding	30%
MM12T	STBD1	starch binding domain 1	CCDS3578.1	chr4_77230510-77230510_G_A	145S>N	Substitution	Nonsynonymous coding	40%
MM12T	STIM2	stromal interaction molecule 2	CCDS3440.1	chr4_27024217-27024217_C_A	701L>I	Substitution	Nonsynonymous coding	13%
MM12T	STK10	serine/threonine kinase 10	CCDS34290.1	chr5_171583776-171583776_G_A	58T>M	Substitution	Nonsynonymous coding	12%
MM12T	STK17B	serine/threonine kinase 17b	CCDS2315.1	chr2_197021253-197021253_G_T	82A>D	Substitution	Nonsynonymous coding	37%
MM12T	STK24	serine/threonine kinase 24	CCDS9488.1	chr13_99112687-99112687_T_A	366D>V	Substitution	Nonsynonymous coding	13%
MM12T	STK25	serine/threonine kinase 25	CCDS2549.1	chr2_242440893-242440893_C_A	87K>N	Substitution	Nonsynonymous coding	35%
MM12T	STK32C	serine/threonine kinase 32C	CCDS7666.1	chr10_134036399-134036399_C_A	362S>I	Substitution	Nonsynonymous coding	30%
MM12T	STK36	serine/threonine kinase 36	CCDS2421.1	chr2_219544363-219544363_G_A	287E>K	Substitution	Nonsynonymous coding	30%
MM12T	STK38L	serine/threonine kinase 38 like	CCDS31761.1	chr12_27470338-27470338_T_A	303L>M	Substitution	Nonsynonymous coding	26%

MM12T	STK39	serine threonine kinase 39	CCDS42770.1	chr2_169038547-169038547_G_A	88R>C	Substitution	Nonsynonymous coding	26%
MM12T	STON1-GTF2A1L	STON1-GTF2A1L readthrough	CCDS1840.1	chr2_48809434-48809434_G_T	554Q>H	Substitution	Nonsynonymous coding	25%
MM12T	STOX2	storkhead box 2	CCDS47167.1	chr4_184930617-184930617_C_T	209T>M	Substitution	Nonsynonymous coding	33%
MM12T	STRIP1	striatin interacting protein 1	CCDS30798.1	chr1_110596347-110596347_A_G	776E>G	Substitution	Nonsynonymous coding	28%
MM12T	STRIP2	striatin interacting protein 2	CCDS34752.1	chr7_129122741-129122741_C_T	703A>V	Substitution	Nonsynonymous coding	29%
MM12T	STX12	syntaxin 12	ENST00000373939	chr1_28138553-28138553_G_A	174S>N	Substitution	Nonsynonymous coding	25%
MM12T	STX19	syntaxin 19	CCDS33793.1	chr3_93734032-93734032_T_G	28T>P	Substitution	Nonsynonymous coding	35%
MM12T	STX1A	syntaxin 1A (brain)	CCDS34655.1	chr7_73118514-73118514_G_A	144R>C	Substitution	Nonsynonymous coding	41%
MM12T	STX1B	syntaxin 1B	CCDS10699.1	chr16_31012261-31012261_A_G	90S>P	Substitution	Nonsynonymous coding	30%
MM12T	STXBP2	syntaxin binding protein 2	CCDS12181.1	chr19_7703933-7703933_G_A	39R>H	Substitution	Nonsynonymous coding	11%
MM12T	STXBP5L	syntaxin binding protein 5-like	CCDS43137.1	chr3_121132156-121132156_T_C	1058C>R	Substitution	Nonsynonymous coding	29%
MM12T	SUDS3	suppressor of defective silencing 3 homolog (S. cerevisiae)	CCDS44993.1	chr12_118829075-118829075_G_A	169E>K	Substitution	Nonsynonymous coding	31%
MM12T	SULF2	sulfatase 2	CCDS13408.1	chr20_46331315-46331315_T_C	172Y>C	Substitution	Nonsynonymous coding	13%
MM12T	SULT2B1	sulfotransferase family, cytosolic, 2B, member 1	CCDS12723.1	chr19_49100018-49100018_G_A	223R>H	Substitution	Nonsynonymous coding	17%
MM12T	SUN2	Sad1 and UNC84 domain containing 2	ENST00000414211	chr22_39134146-39134146_G_A	193A>V	Substitution	Nonsynonymous coding	39%
MM12T	SUN2	Sad1 and UNC84 domain containing 2	CCDS13978.1	chr22_39147007-39147007_G_A	107T>M	Substitution	Nonsynonymous coding	13%
MM12T	SUOX	sulfite oxidase	CCDS8901.2	chr12_56398119-56398119_G_A	316V>I	Substitution	Nonsynonymous coding	33%
MM12T	SUPT20H	suppressor of Ty 20 homolog (S. cerevisiae)	CCDS31959.1	chr13_37618298-37618298_G_A	105P>S	Substitution	Nonsynonymous coding	13%
MM12T	SUPT5H	suppressor of Ty 5 homolog (S. cerevisiae)	CCDS12536.1	chr19_39960816-39960816_G_A	478V>M	Substitution	Nonsynonymous coding	31%
MM12T	SUPT5H	suppressor of Ty 5 homolog (S. cerevisiae)	CCDS12536.1	chr19_39964107-39964107_G_A	813R>H	Substitution	Nonsynonymous coding	13%
MM12T	SUPT5H	suppressor of Ty 5 homolog (S. cerevisiae)	CCDS12536.1	chr19_39949652-39949652_C_T	133R>X	Substitution	Nonsense	30%
MM12T	SUPV3L1	suppressor of var1, 3-like 1 (S. cerevisiae)	CCDS7287.1	chr10_70962635-70962635_G_A	538D>N	Substitution	Nonsynonymous coding	11%
MM12T	SUPV3L1	suppressor of var1, 3-like 1 (S. cerevisiae)	CCDS7287.1	chr10_70962756-70962756_C_A	578P>H	Substitution	Nonsynonymous coding	24%
MM12T	SURF4	surfeit 4	CCDS6968.1	chr9_136231730-136231730_C_T	177A>T	Substitution	Nonsynonymous coding	30%
MM12T	SURF6	surfeit 6	CCDS6962.1	chr9_136198895-136198895_C_T	299R>H	Substitution	Nonsynonymous coding	16%
MM12T	SUSD5	sushi domain containing 5	CCDS46787.1	chr3_33255429-33255429_C_T	94G>D	Substitution	Nonsynonymous coding	10%
MM12T	SV2B	synaptic vesicle glycoprotein 2B	CCDS10370.1	chr15_91801710-91801710_G_A	282V>I	Substitution	Nonsynonymous coding	16%
MM12T	SVEP1	sushi, von Willebrand factor type A, EGF and pentraxin domain containing 1	CCDS48004.1	chr9_113137728-113137728_G_A	3507P>L	Substitution	Nonsynonymous coding	20%
MM12T	SVEP1	sushi, von Willebrand factor type A, EGF and pentraxin domain containing 1	CCDS48004.1	chr9_113233719-113233719_C_T	975D>N	Substitution	Nonsynonymous coding	14%
MM12T	SVIL	supervillin	CCDS7164.1	chr10_29769523-29769523_G_A	1774Q>X	Substitution	Nonsense	32%
MM12T	SYDE1	synapse defective 1, Rho GTPase, homolog 1 (C. elegans)	CCDS12324.1	chr19_15223239-15223239_C_T	554A>V	Substitution	Nonsynonymous coding	25%
MM12T	SYN3	synapsin III	CCDS13908.1	chr22_32924931-32924931_T_C	387D>G	Substitution	Nonsynonymous coding	29%

MM12T	SYNE1	spectrin repeat containing, nuclear envelope 1	CCDS5236.1	chr6_152457805-152457805_G_A	8536S>F	Substitution	Nonsynonymous coding	10%
MM12T	SYNE1	spectrin repeat containing, nuclear envelope 1	CCDS5236.1	chr6_152565728-152565728_G_A	6546R>C	Substitution	Nonsynonymous coding	51%
MM12T	SYNE1	spectrin repeat containing, nuclear envelope 1	CCDS5236.1	chr6_152683324-152683324_G_A	3427T>M	Substitution	Nonsynonymous coding	30%
MM12T	SYNE2	spectrin repeat containing, nuclear envelope 2	CCDS9761.2	chr14_64460647-64460647_C_A	914S>Y	Substitution	Nonsynonymous coding	18%
MM12T	SYNE3	spectrin repeat containing, nuclear envelope family member 3	CCDS9935.1	chr14_95934305-95934305_C_T	ISV-1>	Substitution	Splice site acceptor	20%
MM12T	SYNGAP1	synaptic Ras GTPase activating protein 1	CCDS34434.2	chr6_33406335-33406335_C_A	509A>D	Substitution	Nonsynonymous coding	13%
MM12T	SYNJ1	synaptojanin 1	CCDS33539.1	chr21_34003439-34003439_A_C	1530F>V	Substitution	Nonsynonymous coding	31%
MM12T	SYNJ1	synaptojanin 1	CCDS33539.1	chr21_34012040-34012040_C_T	1213R>Q	Substitution	Nonsynonymous coding	32%
MM12T	SYNJ2	synaptojanin 2	CCDS5254.1	chr6_158492660-158492660_C_T	656T>M	Substitution	Nonsynonymous coding	14%
MM12T	SYNM	synemin, intermediate filament protein	ENST00000336292	chr15_99672977-99672977_C_T	1470A>V	Substitution	Nonsynonymous coding	15%
MM12T	SYNPO	synaptopodin	CCDS4308.1	chr5_150028555-150028555_G_A	240A>T	Substitution	Nonsynonymous coding	32%
MM12T	SYT12	synaptotagmin XII	CCDS8154.1	chr11_66807630-66807630_C_T	193R>C	Substitution	Nonsynonymous coding	15%
MM12T	SYT14	synaptotagmin XIV	CCDS31014.1	chr1_210329100-210329100_C_T	400S>L	Substitution	Nonsynonymous coding	20%
MM12T	SYTL2	synaptotagmin-like 2	CCDS31651.1	chr11_85409029-85409029_C_T	1154V>I	Substitution	Nonsynonymous coding	38%
MM12T	SZT2	seizure threshold 2 homolog (mouse)	CCDS30694.1	chr1_43903337-43903337_G_A	1222R>Q	Substitution	Nonsynonymous coding	27%
MM12T	TAAR1	trace amine associated receptor 1	CCDS5158.1	chr6_132966494-132966494_C_T	217A>T	Substitution	Nonsynonymous coding	18%
MM12T	TAB1	TGF-beta activated kinase 1/MAP3K7 binding protein 1	CCDS13993.1	chr22_39822718-39822718_C_T	311A>V	Substitution	Nonsynonymous coding	26%
MM12T	TAB1	TGF-beta activated kinase 1/MAP3K7 binding protein 1	CCDS13993.1	chr22_39826067-39826067_G_A	452S>N	Substitution	Nonsynonymous coding	14%
MM12T	TAB3	TGF-beta activated kinase 1/MAP3K7 binding protein 3	CCDS14226.1	chrX_30872776-30872776_C_A	336G>X	Substitution	Nonsense	34%
MM12T	TACC3	transforming, acidic coiled-coil containing protein 3	CCDS3352.1	chr4_1729620-1729620_C_A	164S>Y	Substitution	Nonsynonymous coding	11%
MM12T	TACR3	tachykinin receptor 3	CCDS3664.1	chr4_104640501-104640501_A_G	111L>P	Substitution	Nonsynonymous coding	15%
MM12T	TADA2B	transcriptional adaptor 2B	CCDS47007.1	chr4_7056450-7056450_C_T	311A>V	Substitution	Nonsynonymous coding	31%
MM12T	TAF1	TAF1 RNA polymerase II, TATA box binding protein (TBP)-associated factor, 250kDa	CCDS14412.1	chrX_70597016-70597016__T	NA	Insertion	Frameshift	15%
MM12T	TAF12	TAF12 RNA polymerase II, TATA box binding protein (TBP)-associated factor, 20kDa	CCDS326.1	chr1_28931969-28931969_C_T	122R>H	Substitution	Nonsynonymous coding	34%
MM12T	TAL1	T-cell acute lymphocytic leukemia 1	CCDS547.1	chr1_47685444-47685444_C_T	315R>H	Substitution	Nonsynonymous coding	12%
MM12T	TAMM41	TAM41, mitochondrial translocator assembly and maintenance protein, homolog (S.	CCDS2607.1	chr3_11885643-11885643_C_T	60A>T	Substitution	Nonsynonymous coding	14%
MM12T	TAPBP	TAP binding protein (tapasin)	CCDS34426.1	chr6_33272100-33272100_C_T	395S>N	Substitution	Nonsynonymous coding	24%
MM12T	TAS1R3	taste receptor, type 1, member 3	CCDS30556.1	chr1_1268403-1268403_G_A	460V>M	Substitution	Nonsynonymous coding	34%
MM12T	TAS1R3	taste receptor, type 1, member 3	CCDS30556.1	chr1_1269268-1269268_G_T	661E>D	Substitution	Nonsynonymous coding	28%
MM12T	TBC1D10B	TBC1 domain family, member 10B	CCDS10676.2	chr16_30370531-30370531_G_A	535A>V	Substitution	Nonsynonymous coding	36%
MM12T	TBC1D2	TBC1 domain family, member 2	CCDS35080.1	chr9_100971286-100971286_C_T	605R>H	Substitution	Nonsynonymous coding	33%
MM12T	TBC1D22B	TBC1 domain family, member 22B	CCDS4832.1	chr6_37237402-37237402_C_A	30P>H	Substitution	Nonsynonymous coding	21%

MM12T	TBC1D30	TBC1 domain family, member 30	ENST00000229088	chr12_65225909-65225909_G_A	303R>H	Substitution	Nonsynonymous coding	36%
MM12T	TBC1D5	TBC1 domain family, member 5	CCDS46770.1	chr3_17300086-17300086_A_	NA	Deletion	Splice site acceptor	21%
MM12T	TBC1D7	TBC1 domain family, member 7	CCDS4523.1	chr6_13321234-13321234_C_T	96R>H	Substitution	Nonsynonymous coding	20%
MM12T	TBC1D8	TBC1 domain family, member 8 (with GRAM domain)	CCDS46375.1	chr2_101670764-101670764_G_A	131A>V	Substitution	Nonsynonymous coding	26%
MM12T	TBC1D9B	TBC1 domain family, member 9B (with GRAM domain)	CCDS43408.1	chr5_179306142-179306142_C_T	491R>H	Substitution	Nonsynonymous coding	28%
MM12T	TBCC	tubulin folding cofactor C	CCDS4872.1	chr6_42712880-42712880_C_T	311S>N	Substitution	Nonsynonymous coding	46%
MM12T	TBCD	tubulin folding cofactor D	CCDS45818.1	chr17_80899282-80899282_G_A	1163E>K	Substitution	Nonsynonymous coding	11%
MM12T	TBK1	TANK-binding kinase 1	CCDS8968.1	chr12_64849706-64849706_C_T	19A>V	Substitution	Nonsynonymous coding	21%
MM12T	TBKBP1	TBK1 binding protein 1	CCDS45722.1	chr17_45776730-45776730_C_T	227R>W	Substitution	Nonsynonymous coding	36%
MM12T	TBL1X	transducin (beta)-like 1X-linked	CCDS14133.1	chrX_9622261-9622261_G_A	37G>D	Substitution	Nonsynonymous coding	29%
MM12T	TBL3	transducin (beta)-like 3	CCDS10453.1	chr16_2024551-2024551_G_A	84A>T	Substitution	Nonsynonymous coding	10%
MM12T	TBL3	transducin (beta)-like 3	CCDS10453.1	chr16_2024785-2024785_G_A	134R>H	Substitution	Nonsynonymous coding	10%
MM12T	TBL3	transducin (beta)-like 3	CCDS10453.1	chr16_2024842-2024842_T_C	153V>A	Substitution	Nonsynonymous coding	33%
MM12T	TBL3	transducin (beta)-like 3	CCDS10453.1	chr16_2028594-2028594_G_A	779A>T	Substitution	Nonsynonymous coding	10%
MM12T	TBPL2	TATA box binding protein like 2	CCDS9724.1	chr14_55903726-55903726_G_A	54A>V	Substitution	Nonsynonymous coding	19%
MM12T	TBRG4	transforming growth factor beta regulator 4	CCDS5501.1	chr7_45148784-45148784_C_T	18R>H	Substitution	Nonsynonymous coding	27%
MM12T	TBX15	T-box 15	CCDS30816.1	chr1_119466078-119466078_G_A	175T>M	Substitution	Nonsynonymous coding	29%
MM12T	TBX15	T-box 15	ENST00000369429	chr1_119530322-119530322_G_A	33R>X	Substitution	Nonsense	28%
MM12T	TBX2	T-box 2	CCDS11627.2	chr17_59480510-59480510_G_A	251R>H	Substitution	Nonsynonymous coding	14%
MM12T	TBX4	T-box 4	CCDS11629.1	chr17_59534959-59534959_C_T	83A>V	Substitution	Nonsynonymous coding	38%
MM12T	TBX4	T-box 4	CCDS11629.1	chr17_59556115-59556115_C_A	226S>Y	Substitution	Nonsynonymous coding	39%
MM12T	TBX6	T-box 6	CCDS10670.1	chr16_30100478-30100478_C_T	136R>H	Substitution	Nonsynonymous coding	10%
MM12T	TBXA2R	thromboxane A2 receptor	ENST00000397896	chr19_3595643-3595643_A_G	229L>P	Substitution	Nonsynonymous coding	29%
MM12T	TBXAS1	thromboxane A synthase 1 (platelet)	CCDS5855.1	chr7_139529239-139529239_C_T	18T>M	Substitution	Nonsynonymous coding	13%
MM12T	TCF12	transcription factor 12	CCDS10160.1	chr15_57574709-57574709_C_T	682S>L	Substitution	Nonsynonymous coding	14%
MM12T	TCF7L2	transcription factor 7-like 2 (T-cell specific, HMG-box)	CCDS7576.1	chr10_114925604-114925604_C_T	555A>V	Substitution	Nonsynonymous coding	31%
MM12T	TCOF1	Treacher Collins-Franceschetti syndrome 1	CCDS47305.1	chr5_149754950-149754950_G_T	513G>C	Substitution	Nonsynonymous coding	38%
MM12T	TCP10L2	t-complex 10-like 2	CCDS47514.1	chr6_167591954-167591954_G_A	194R>H	Substitution	Nonsynonymous coding	21%
MM12T	TCP11	t-complex 11, testis-specific	ENST00000455706	chr6_35109157-35109157_C_	NA	Deletion	Frameshift	20%
MM12T	TCTEX1D4	Tctex1 domain containing 4	CCDS30699.1	chr1_45271805-45271805_C_T	179R>H	Substitution	Nonsynonymous coding	31%
MM12T	TDRD1	tudor domain containing 1	CCDS7588.1	chr10_115947662-115947662_G_T	24E>D	Substitution	Nonsynonymous coding	22%
MM12T	TDRD15	tudor domain containing 15	ENST00000405799	chr2_21364100-21364100_A_C	1254K>T	Substitution	Nonsynonymous coding	13%

MM12T	TDRD6	tudor domain containing 6	CCDS34470.1	chr6_46658172-46658172_A_T	769E>D	Substitution	Nonsynonymous coding	12%
MM12T	TECRL	trans-2,3-enoyl-CoA reductase-like	CCDS33990.1	chr4_65180394-65180394_C_T	175A>T	Substitution	Nonsynonymous coding	41%
MM12T	TECTA	tectorin alpha	CCDS8434.1	chr11_120998985-120998985_C_T	767R>W	Substitution	Nonsynonymous coding	10%
MM12T	TEK	TEK tyrosine kinase, endothelial	CCDS6519.1	chr9_27190676-27190676_C_A	493Q>K	Substitution	Nonsynonymous coding	10%
MM12T	TELO2	TEL2, telomere maintenance 2, homolog (S. cerevisiae)	CCDS32363.1	chr16_1557610-1557610_G_A	767R>H	Substitution	Nonsynonymous coding	40%
MM12T	TENM1	teneurin transmembrane protein 1	CCDS14609.1	chrX_123870848-123870848__A	NA	Insertion	Frameshift	12%
MM12T	TENM1	teneurin transmembrane protein 1	CCDS14609.1	chrX_123657290-123657290_G_T	986P>H	Substitution	Nonsynonymous coding	29%
MM12T	TENM1	teneurin transmembrane protein 1	CCDS14609.1	chrX_124097529-124097529_C_A	25S>I	Substitution	Nonsynonymous coding	13%
MM12T	TENM2	teneurin transmembrane protein 2	NM_001122679	chr5_167675119-167675119_C_T	2383T>M	Substitution	Nonsynonymous coding	38%
MM12T	TENM3	teneurin transmembrane protein 3	CCDS47165.1	chr4_183245223-183245223_G_A	17R>Q	Substitution	Nonsynonymous coding	31%
MM12T	TENM3	teneurin transmembrane protein 3	CCDS47165.1	chr4_183658160-183658160_C_T	1056A>V	Substitution	Nonsynonymous coding	14%
MM12T	TENM3	teneurin transmembrane protein 3	CCDS47165.1	chr4_183659641-183659641_C_T	1108A>V	Substitution	Nonsynonymous coding	12%
MM12T	TENM4	teneurin transmembrane protein 4	CCDS44688.1	chr11_78440528-78440528_C_T	1100R>H	Substitution	Nonsynonymous coding	19%
MM12T	TENM4	teneurin transmembrane protein 4	CCDS44688.1	chr11_78369498-78369498_G_A	2639R>X	Substitution	Nonsense	31%
MM12T	TEP1	telomerase-associated protein 1	CCDS9548.1	chr14_20842589-20842589_A_G	2157V>A	Substitution	Nonsynonymous coding	15%
MM12T	TEP1	telomerase-associated protein 1	CCDS9548.1	chr14_20848577-20848577_C_T	1607R>H	Substitution	Nonsynonymous coding	30%
MM12T	TEP1	telomerase-associated protein 1	CCDS9548.1	chr14_20852291-20852291_C_T	1147R>H	Substitution	Nonsynonymous coding	19%
MM12T	TEP1	telomerase-associated protein 1	CCDS9548.1	chr14_20856115-20856115_C_T	878R>Q	Substitution	Nonsynonymous coding	17%
MM12T	TEPP	testis, prostate and placenta expressed	CCDS10790.1	chr16_58010391-58010391_G_A	6A>T	Substitution	Nonsynonymous coding	13%
MM12T	TES	testis derived transcript (3 LIM domains)	CCDS5763.1	chr7_115874609-115874609_G_T	17G>X	Substitution	Nonsense	33%
MM12T	TESK1	testis-specific kinase 1	CCDS6580.1	chr9_35609489-35609489_C_T	544A>V	Substitution	Nonsynonymous coding	10%
MM12T	TET1	tet methylcytosine dioxygenase 1	CCDS7281.1	chr10_70426928-70426928_G_A	1530A>T	Substitution	Nonsynonymous coding	24%
MM12T	TET2	tet methylcytosine dioxygenase 2	CCDS47120.1	chr4_106196631-106196631_C_T	1655P>L	Substitution	Nonsynonymous coding	24%
MM12T	TET2	tet methylcytosine dioxygenase 2	CCDS47120.1	chr4_106197590-106197590_A_G	1975T>A	Substitution	Nonsynonymous coding	26%
MM12T	TET3	tet methylcytosine dioxygenase 3	CCDS46339.1	chr2_74273531-74273531_G_A	28A>T	Substitution	Nonsynonymous coding	11%
MM12T	TET3	tet methylcytosine dioxygenase 3	CCDS46339.1	chr2_74317140-74317140_G_A	867R>H	Substitution	Nonsynonymous coding	12%
MM12T	TEX2	testis expressed 2 [Source:HGNC Symbol;Acc:30884]	CCDS11658.1	chr17_62290700-62290700_C_T	293R>H	Substitution	Nonsynonymous coding	21%
MM12T	TEX33	testis expressed 33	CCDS13937.1	chr22_37397927-37397927_C_T	62R>H	Substitution	Nonsynonymous coding	11%
MM12T	TF	transferrin	CCDS3080.1	chr3_133486900-133486900_C_A	505P>H	Substitution	Nonsynonymous coding	24%
MM12T	TFAP2B	transcription factor AP-2 beta (activating enhancer binding protein 2 beta)	CCDS4934.2	chr6_50791372-50791372_C_T	112Q>X	Substitution	Nonsense	50%
MM12T	TFCP2L1	transcription factor CP2-like 1	CCDS2134.1	chr2_122038771-122038771_G_T	47L>M	Substitution	Nonsynonymous coding	37%
MM12T	TFE3	transcription factor binding to IGHM enhancer 3	CCDS14315.3	chrX_48887952-48887952_C_	NA	Deletion	Frameshift	23%

MM12T	TFEB	transcription factor EB	CCDS4858.1	chr6_41652348-41652348_C_T	474D>N	Substitution	Nonsynonymous coding	19%
MM12T	TFIP11	tuftelin interacting protein 11	CCDS13838.1	chr22_26895194-26895194_C_T	402R>H	Substitution	Nonsynonymous coding	11%
MM12T	TFR2	transferrin receptor 2	CCDS34707.1	chr7_100225731-100225731_G_A	501A>V	Substitution	Nonsynonymous coding	30%
MM12T	TG	thyroglobulin	CCDS34944.1	chr8_133923730-133923730_C_T	1371R>W	Substitution	Nonsynonymous coding	34%
MM12T	TGDS	TDP-glucose 4,6-dehydratase	CCDS9471.1	chr13_95235475-95235475_C_T	110R>H	Substitution	Nonsynonymous coding	15%
MM12T	TGFB1	transforming growth factor, beta 1	CCDS33031.1	chr19_41847939-41847939_C_A	ISV-4>	Substitution	Splice site acceptor	29%
MM12T	TGFB2	transforming growth factor, beta 2	CCDS44318.1	chr1_218520232-218520232_C_	NA	Deletion	Frameshift	10%
MM12T	TGFBI	transforming growth factor, beta-induced, 68kDa	CCDS47266.1	chr5_135382096-135382096_G_A	124R>H	Substitution	Nonsynonymous coding	11%
MM12T	TGM2	transglutaminase 2 (C polypeptide, protein-glutamine-gamma-glutamyltransferase)	CCDS13302.1	chr20_36767988-36767988_C_T	390A>T	Substitution	Nonsynonymous coding	12%
MM12T	TGM3	transglutaminase 3 (E polypeptide, protein-glutamine-gamma-glutamyltransferase)	CCDS33435.1	chr20_2312855-2312855_C_T	514T>I	Substitution	Nonsynonymous coding	34%
MM12T	TH	tyrosine hydroxylase	CCDS7731.1	chr11_2186997-2186997_C_T	ISV-4>	Substitution	Splice site acceptor	24%
MM12T	THAP10	THAP domain containing 10	CCDS10237.1	chr15_71175115-71175115_G_A	188R>C	Substitution	Nonsynonymous coding	21%
MM12T	THAP7	THAP domain containing 7	CCDS13787.1	chr22_21354312-21354312_G_A	263R>W	Substitution	Nonsynonymous coding	31%
MM12T	THBD	thrombomodulin	CCDS13148.1	chr20_23028541-23028541_G_A	534A>V	Substitution	Nonsynonymous coding	38%
MM12T	THBS2	thrombospondin 2	CCDS34574.1	chr6_169621619-169621619_G_A	1093R>X	Substitution	Nonsense	47%
MM12T	THEM5	thioesterase superfamily member 5	CCDS1005.1	chr1_151820264-151820264_G_T	217S>Y	Substitution	Nonsynonymous coding	31%
MM12T	THG1L	tRNA-histidine guanylyltransferase 1-like (S. cerevisiae)	CCDS4341.1	chr5_157160007-157160007_G_T	108S>I	Substitution	Nonsynonymous coding	25%
MM12T	THNSL2	threonine synthase-like 2 (S. cerevisiae)	CCDS2002.2	chr2_88474908-88474908_C_T	187H>Y	Substitution	Nonsynonymous coding	11%
MM12T	THOC2	THO complex 2	CCDS43988.1	chrX_122801051-122801051_C_T	366D>N	Substitution	Nonsynonymous coding	24%
MM12T	THOC2	THO complex 2	CCDS43988.1	chrX_122866845-122866845_C_T	10A>T	Substitution	Nonsynonymous coding	28%
MM12T	THOC5	THO complex 5	CCDS13859.1	chr22_29913299-29913299_C_T	516V>I	Substitution	Nonsynonymous coding	11%
MM12T	THOC6	THO complex 6 homolog (Drosophila)	CCDS10491.1	chr16_3077037-3077037_G_A	221R>H	Substitution	Nonsynonymous coding	14%
MM12T	THOC6	THO complex 6 homolog (Drosophila)	CCDS10491.1	chr16_3077247-3077247_C_T	259A>V	Substitution	Nonsynonymous coding	29%
MM12T	THOC6	THO complex 6 homolog (Drosophila)	CCDS10491.1	chr16_3077487-3077487_G_A	311A>T	Substitution	Nonsynonymous coding	32%
MM12T	THOP1	thimet oligopeptidase 1	CCDS12095.1	chr19_2807449-2807449_C_T	299A>V	Substitution	Nonsynonymous coding	27%
MM12T	THRAP3	thyroid hormone receptor associated protein 3	CCDS405.1	chr1_36748232-36748232_G_A	23R>H	Substitution	Nonsynonymous coding	32%
MM12T	THRB	thyroid hormone receptor, beta	CCDS2641.1	chr3_24231784-24231784_C_T	22D>N	Substitution	Nonsynonymous coding	26%
MM12T	THRSP	thyroid hormone responsive	CCDS8256.1	chr11_77774946-77774946_C_T	7R>C	Substitution	Nonsynonymous coding	30%
MM12T	THSD1	thrombospondin, type I, domain containing 1	CCDS9432.1	chr13_52951856-52951856_C_T	750G>E	Substitution	Nonsynonymous coding	31%
MM12T	THSD7B	thrombospondin, type I, domain containing 7B	ENST00000272643	chr2_137928475-137928475_C_T	564R>C	Substitution	Nonsynonymous coding	10%
MM12T	THSD7B	thrombospondin, type I, domain containing 7B	ENST00000272643	chr2_138030202-138030202_G_A	789C>Y	Substitution	Nonsynonymous coding	21%
MM12T	THSD7B	thrombospondin, type I, domain containing 7B	ENST00000272643	chr2_138163309-138163309_C_A	876A>D	Substitution	Nonsynonymous coding	40%

MM12T	THUMPD1	THUMP domain containing 1	CCDS10588.1	chr16_20748527-20748527_G_T	246A>D	Substitution	Nonsynonymous coding	26%
MM12T	TIA1	TIA1 cytotoxic granule-associated RNA binding protein	CCDS1901.1	chr2_70439858-70439858_G_C	385T>S	Substitution	Nonsynonymous coding	30%
MM12T	TIA1	TIA1 cytotoxic granule-associated RNA binding protein	CCDS1901.1	chr2_70442548-70442548_G_T	281C>X	Substitution	Nonsense	25%
MM12T	TIAM1	T-cell lymphoma invasion and metastasis 1	CCDS13609.1	chr21_32526578-32526578_C_T	1053R>H	Substitution	Nonsynonymous coding	18%
MM12T	TIAM2	T-cell lymphoma invasion and metastasis 2	CCDS34558.1	chr6_155465779-155465779_C_T	557S>F	Substitution	Nonsynonymous coding	17%
MM12T	TICAM1	toll-like receptor adaptor molecule 1	CCDS12136.1	chr19_4817289-4817289__AGG	NA	Insertion	In-frame insertion	22%
MM12T	TICAM1	toll-like receptor adaptor molecule 1	CCDS12136.1	chr19_4817666-4817666_G_A	242P>S	Substitution	Nonsynonymous coding	26%
MM12T	TICRR	TOPBP1-interacting checkpoint and replication regulator	CCDS10352.2	chr15_90129009-90129009_C_A	416A>D	Substitution	Nonsynonymous coding	30%
MM12T	TICRR	TOPBP1-interacting checkpoint and replication regulator	CCDS10352.2	chr15_90144763-90144763_C_T	764A>V	Substitution	Nonsynonymous coding	35%
MM12T	TIMM17B	translocase of inner mitochondrial membrane 17 homolog B (yeast)	CCDS14308.1	chrX_48751095-48751095_G_A	146P>S	Substitution	Nonsynonymous coding	31%
MM12T	TIMM17B	translocase of inner mitochondrial membrane 17 homolog B (yeast)	CCDS14308.1	chrX_48751189-48751189_G_A	142R>X	Substitution	Nonsense	28%
MM12T	TIMM9	translocase of inner mitochondrial membrane 9 homolog (yeast)	CCDS9735.1	chr14_58875834-58875834_A_G	63I>T	Substitution	Nonsynonymous coding	19%
MM12T	TIMP4	TIMP metallopeptidase inhibitor 4	CCDS2608.1	chr3_12195877-12195877_C_T	143V>M	Substitution	Nonsynonymous coding	25%
MM12T	TINAG	tubulointerstitial nephritis antigen	CCDS4955.1	chr6_54219311-54219311_C_T	376A>V	Substitution	Nonsynonymous coding	26%
MM12T	TJP3	tight junction protein 3	CCDS32873.1	chr19_3748054-3748054_G_A	895R>K	Substitution	Nonsynonymous coding	17%
MM12T	TKT	transketolase	CCDS2871.1	chr3_53267282-53267282_G_A	213A>V	Substitution	Nonsynonymous coding	11%
MM12T	TLE2	transducin-like enhancer of split 2 (E(sp1) homolog, Drosophila)	CCDS45911.1	chr19_3006492-3006492_C_T	476G>S	Substitution	Nonsynonymous coding	47%
MM12T	TLE2	transducin-like enhancer of split 2 (E(sp1) homolog, Drosophila)	CCDS45911.1	chr19_3013746-3013746_C_T	265R>H	Substitution	Nonsynonymous coding	31%
MM12T	TLE2	transducin-like enhancer of split 2 (E(sp1) homolog, Drosophila)	CCDS45911.1	chr19_3015709-3015709_C_T	207S>N	Substitution	Nonsynonymous coding	31%
MM12T	TLE2	transducin-like enhancer of split 2 (E(sp1) homolog, Drosophila)	CCDS45911.1	chr19_3019308-3019308_C_T	175A>T	Substitution	Nonsynonymous coding	35%
MM12T	TLE6	transducin-like enhancer of split 6 (E(sp1) homolog, Drosophila)	CCDS45910.1	chr19_2989725-2989725_G_A	396A>T	Substitution	Nonsynonymous coding	26%
MM12T	TLE6	transducin-like enhancer of split 6 (E(sp1) homolog, Drosophila)	CCDS45910.1	chr19_2994070-2994070_C_T	531P>S	Substitution	Nonsynonymous coding	35%
MM12T	TLK1	tousled-like kinase 1	ENST00000360843	chr2_171916481-171916481_G_A	141P>L	Substitution	Nonsynonymous coding	20%
MM12T	TLL1	tolloid-like 1	CCDS3811.1	chr4_166963254-166963254_G_A	446S>N	Substitution	Nonsynonymous coding	21%
MM12T	TLL1	tolloid-like 1	CCDS3811.1	chr4_166986977-166986977_C_T	717T>I	Substitution	Nonsynonymous coding	14%
MM12T	TLL2	tolloid-like 2	CCDS7449.1	chr10_98188479-98188479_C_T	183A>T	Substitution	Nonsynonymous coding	33%
MM12T	TLN1	talin 1	CCDS35009.1	chr9_35697907-35697907_C_T	2503A>T	Substitution	Nonsynonymous coding	32%
MM12T	TLN1	talin 1	CCDS35009.1	chr9_35707831-35707831_C_T	1510R>H	Substitution	Nonsynonymous coding	29%
MM12T	TLN1	talin 1	CCDS35009.1	chr9_35707882-35707882_G_A	1493S>F	Substitution	Nonsynonymous coding	11%
MM12T	TLN1	talin 1	CCDS35009.1	chr9_35720837-35720837_G_A	393A>V	Substitution	Nonsynonymous coding	19%
MM12T	TLN1	talin 1	CCDS35009.1	chr9_35724015-35724015_G_T	239A>D	Substitution	Nonsynonymous coding	27%
MM12T	TLN2	talin 2	CCDS32261.1	chr15_63004167-63004167_C_T	842A>V	Substitution	Nonsynonymous coding	30%

MM12T	TLN2	talin 2	CCDS32261.1	chr15_63032895-63032895_C_A	1318L>M	Substitution	Nonsynonymous coding	38%
MM12T	TLR1	toll-like receptor 1	CCDS33973.1	chr4_38798270-38798270_A_G	728L>P	Substitution	Nonsynonymous coding	39%
MM12T	TLR2	toll-like receptor 2	CCDS3784.1	chr4_154624296-154624296_G_T	79Q>H	Substitution	Nonsynonymous coding	23%
MM12T	TLR5	toll-like receptor 5	CCDS31033.1	chr1_223285767-223285767_G_T	203L>I	Substitution	Nonsynonymous coding	27%
MM12T	TLR8	toll-like receptor 8	CCDS14152.1	chrX_12938673-12938673_C_A	505P>H	Substitution	Nonsynonymous coding	29%
MM12T	TLR8	toll-like receptor 8	CCDS14152.1	chrX_12938717-12938717_G_A	520V>M	Substitution	Nonsynonymous coding	27%
MM12T	TLR8	toll-like receptor 8	CCDS14152.1	chrX_12939408-12939408_C_T	750T>I	Substitution	Nonsynonymous coding	28%
MM12T	TLR9	toll-like receptor 9	CCDS2848.1	chr3_52256539-52256539_G_A	598T>M	Substitution	Nonsynonymous coding	19%
MM12T	TLR9	toll-like receptor 9	CCDS2848.1	chr3_52257970-52257970_G_A	121A>V	Substitution	Nonsynonymous coding	31%
MM12T	TLX2	T-cell leukemia homeobox 2	CCDS1947.1	chr2_74742187-74742187_C_T	85A>V	Substitution	Nonsynonymous coding	14%
MM12T	TM2D3	TM2 domain containing 3	CCDS10393.1	chr15_102182798-102182798_G_A	210R>W	Substitution	Nonsynonymous coding	19%
MM12T	TM9SF1	transmembrane 9 superfamily member 1	CCDS9617.1	chr14_24661548-24661548_C_T	328A>T	Substitution	Nonsynonymous coding	31%
MM12T	TM9SF2	transmembrane 9 superfamily member 2	CCDS9493.1	chr13_100211648-100211648_C_T	595T>M	Substitution	Nonsynonymous coding	30%
MM12T	TM9SF3	transmembrane 9 superfamily member 3	CCDS7450.1	chr10_98311104-98311104_C_A	286S>I	Substitution	Nonsynonymous coding	34%
MM12T	TMC2	transmembrane channel-like 2	CCDS13029.2	chr20_2539135-2539135_G_A	39R>Q	Substitution	Nonsynonymous coding	34%
MM12T	TMC2	transmembrane channel-like 2	CCDS13029.2	chr20_2542613-2542613_C_T	171R>W	Substitution	Nonsynonymous coding	29%
MM12T	TMC2	transmembrane channel-like 2	CCDS13029.2	chr20_2592875-2592875_G_T	544W>C	Substitution	Nonsynonymous coding	29%
MM12T	TMC4	transmembrane channel-like 4	CCDS46174.1	chr19_54669235-54669235_C_T	294R>Q	Substitution	Nonsynonymous coding	27%
MM12T	TMC5	transmembrane channel-like 5	CCDS45431.1	chr16_19492774-19492774_C_T	784Q>X	Substitution	Nonsense	13%
MM12T	TMC6	transmembrane channel-like 6	CCDS32748.1	chr17_76122692-76122692_C_T	32D>N	Substitution	Nonsynonymous coding	11%
MM12T	TMC6	transmembrane channel-like 6	CCDS32748.1	chr17_76113589-76113589_C_A	720E>X	Substitution	Nonsense	11%
MM12T	TMC6	transmembrane channel-like 6	CCDS32748.1	chr17_76113617-76113617_C_T	710W>X	Substitution	Nonsense	36%
MM12T	TMCO3	transmembrane and coiled-coil domains 3	CCDS9537.1	chr13_114193756-114193756_G_A	542E>K	Substitution	Nonsynonymous coding	14%
MM12T	TMED1	transmembrane emp24 protein transport domain containing 1	CCDS12249.1	chr19_10945630-10945630_C_T	149V>I	Substitution	Nonsynonymous coding	30%
MM12T	TMED6	transmembrane emp24 protein transport domain containing 6	CCDS10878.1	chr16_69377539-69377539_C_T	165G>D	Substitution	Nonsynonymous coding	13%
MM12T	TMEFF2	transmembrane protein with EGF-like and two follistatin-like domains 2	CCDS2314.1	chr2_192863795-192863795_G_A	226R>X	Substitution	Nonsense	13%
MM12T	TMEM129	transmembrane protein 129	CCDS46998.1	chr4_1719346-1719346_C_A	246R>M	Substitution	Nonsynonymous coding	43%
MM12T	TMEM132A	transmembrane protein 132A	CCDS7997.1	chr11_60702054-60702054_G_A	553A>T	Substitution	Nonsynonymous coding	23%
MM12T	TMEM132B	transmembrane protein 132B	CCDS41859.1	chr12_125834486-125834486_G_A	181G>R	Substitution	Nonsynonymous coding	30%
MM12T	TMEM132E	transmembrane protein 132E	CCDS11283.1	chr17_32964613-32964613_G_A	773V>I	Substitution	Nonsynonymous coding	51%
MM12T	TMEM14C	transmembrane protein 14C	CCDS4514.1	chr6_10728953-10728953_G_T	94G>C	Substitution	Nonsynonymous coding	30%
MM12T	TMEM163	transmembrane protein 163	CCDS2172.1	chr2_135260483-135260483_G_A	182L>F	Substitution	Nonsynonymous coding	11%

MM12T	TMEM168	transmembrane protein 168	CCDS5757.1	chr7_112407412-112407412_C_T	645R>H	Substitution	Nonsynonymous coding	33%
MM12T	TMEM171	transmembrane protein 171	CCDS4017.1	chr5_72419385-72419385_C_T	62A>V	Substitution	Nonsynonymous coding	30%
MM12T	TMEM180	transmembrane protein 180	CCDS7535.1	chr10_104235565-104235565_G_A	460A>T	Substitution	Nonsynonymous coding	10%
MM12T	TMEM181	transmembrane protein 181	CCDS43520.1	chr6_159029368-159029368_C_T	363P>L	Substitution	Nonsynonymous coding	20%
MM12T	TMEM181	transmembrane protein 181	CCDS43520.1	chr6_159046206-159046206_C_T	479A>V	Substitution	Nonsynonymous coding	10%
MM12T	TMEM184B	transmembrane protein 184B	CCDS13969.2	chr22_38641959-38641959_C_T	114V>I	Substitution	Nonsynonymous coding	62%
MM12T	TMEM186	transmembrane protein 186	CCDS10535.1	chr16_8891468-8891468_C_T	ISV+1>	Substitution	Splice site donor	11%
MM12T	TMEM198	transmembrane protein 198	CCDS33385.1	chr2_220412657-220412657_G_A	199R>H	Substitution	Nonsynonymous coding	11%
MM12T	TMEM199	transmembrane protein 199	CCDS11228.1	chr17_26687869-26687869_C_T	177S>L	Substitution	Nonsynonymous coding	39%
MM12T	TMEM2	transmembrane protein 2	CCDS6638.1	chr9_74324305-74324305_T_C	952D>G	Substitution	Nonsynonymous coding	26%
MM12T	TMEM2	transmembrane protein 2	CCDS6638.1	chr9_74345102-74345102_C_A	614R>M	Substitution	Nonsynonymous coding	26%
MM12T	TMEM2	transmembrane protein 2	CCDS6638.1	chr9_74361153-74361153_C_T	146D>N	Substitution	Nonsynonymous coding	23%
MM12T	TMEM209	transmembrane protein 209	CCDS47712.1	chr7_129815360-129815360_C_T	446D>N	Substitution	Nonsynonymous coding	16%
MM12T	TMEM229A	transmembrane protein 229A	CCDS47694.1	chr7_123672061-123672061_C_T	333A>T	Substitution	Nonsynonymous coding	26%
MM12T	TMEM229B	transmembrane protein 229B	CCDS9783.1	chr14_67940219-67940219_C_T	141R>H	Substitution	Nonsynonymous coding	22%
MM12T	TMEM231	transmembrane protein 231	CCDS45531.1	chr16_75579818-75579818_G_A	144T>M	Substitution	Nonsynonymous coding	33%
MM12T	TMEM232	transmembrane protein 232	CCDS47253.1	chr5_109960993-109960993_C_A	248R>I	Substitution	Nonsynonymous coding	23%
MM12T	TMEM232	transmembrane protein 232	CCDS47253.1	chr5_109904327-109904327_C_A	ISV-1>	Substitution	Splice site acceptor	26%
MM12T	TMEM237	transmembrane protein 237	ENST00000409099	chr2_202496944-202496944_C_T	133R>H	Substitution	Nonsynonymous coding	29%
MM12T	TMEM239	transmembrane protein 239	NM_001167670	chr20_2797534-2797534_C_T	112L>F	Substitution	Nonsynonymous coding	33%
MM12T	TMEM240	transmembrane protein 240	ENST00000378733	chr1_1470664-1470664_G_A	197R>W	Substitution	Nonsynonymous coding	13%
MM12T	TMEM240	transmembrane protein 240	CCDS44040.1	chr1_1470807-1470807_C_T	152A>T	Substitution	Nonsynonymous coding	10%
MM12T	TMEM240	transmembrane protein 240	CCDS44040.1	chr1_1470975-1470975_G_A	123R>C	Substitution	Nonsynonymous coding	33%
MM12T	TMEM245	transmembrane protein 245	CCDS43858.1	chr9_111800351-111800351_A_G	724Y>H	Substitution	Nonsynonymous coding	31%
MM12T	TMEM245	transmembrane protein 245	CCDS43858.1	chr9_111853198-111853198_C_T	ISV+4>	Substitution	Splice site donor	23%
MM12T	TMEM247	transmembrane protein 247	ENST00000343465	chr2_46707890-46707890_C_T	155A>V	Substitution	Nonsynonymous coding	36%
MM12T	TMEM30A	transmembrane protein 30A	CCDS4983.1	chr6_75970575-75970575_G_A	169A>V	Substitution	Nonsynonymous coding	18%
MM12T	TMEM44	transmembrane protein 44	CCDS3308.1	chr3_194337922-194337922_G_A	230S>L	Substitution	Nonsynonymous coding	31%
MM12T	TMEM44	transmembrane protein 44	CCDS3308.1	chr3_194344327-194344327_C_T	152W>X	Substitution	Nonsense	25%
MM12T	TMEM47	transmembrane protein 47	CCDS14235.1	chrX_34675088-34675088_T_G	20K>T	Substitution	Nonsynonymous coding	27%
MM12T	TMEM63A	transmembrane protein 63A	CCDS31042.1	chr1_226037619-226037619_G_A	689R>C	Substitution	Nonsynonymous coding	15%
MM12T	TMEM63A	transmembrane protein 63A	CCDS31042.1	chr1_226053660-226053660_C_T	228R>Q	Substitution	Nonsynonymous coding	36%

MM12T	TMEM63C	transmembrane protein 63C	CCDS45141.1	chr14_77698065-77698065_C_T	162S>F	Substitution	Nonsynonymous coding	29%
MM12T	TMEM68	transmembrane protein 68	CCDS6161.1	chr8_56652717-56652717_T_C	250S>G	Substitution	Nonsynonymous coding	33%
MM12T	TMEM79	transmembrane protein 79	CCDS1138.1	chr1_156255277-156255277_C_T	87P>L	Substitution	Nonsynonymous coding	11%
MM12T	TMEM9	transmembrane protein 9	CCDS1408.1	chr1_201120981-201120981_C_A	ISV-1>	Substitution	Splice site acceptor	24%
MM12T	TMEM95	transmembrane protein 95	CCDS32546.1	chr17_7259169-7259169_C_A	80A>D	Substitution	Nonsynonymous coding	13%
MM12T	TMEM99	transmembrane protein 99	CCDS42319.1	chr17_38991037-38991037_G_A	90R>Q	Substitution	Nonsynonymous coding	10%
MM12T	TMOD1	tropomodulin 1	CCDS6726.1	chr9_100328232-100328232_G_A	241A>T	Substitution	Nonsynonymous coding	28%
MM12T	TMPO	thymopoietin	CCDS9064.1	chr12_98927863-98927863_C_T	610R>C	Substitution	Nonsynonymous coding	21%
MM12T	TMPRSS11D	transmembrane protease, serine 11D	CCDS3518.1	chr4_68693168-68693168_C_A	255V>L	Substitution	Nonsynonymous coding	18%
MM12T	TMPRSS11E	transmembrane protease, serine 11E	CCDS33993.1	chr4_69343236-69343236_C_A	286P>H	Substitution	Nonsynonymous coding	29%
MM12T	TMPRSS11F	transmembrane protease, serine 11F	CCDS3520.1	chr4_68930513-68930513_C_T	302G>E	Substitution	Nonsynonymous coding	13%
MM12T	TMPRSS11F	transmembrane protease, serine 11F	CCDS3520.1	chr4_68925163-68925163_G_A	347Q>X	Substitution	Nonsense	31%
MM12T	TMPRSS12	transmembrane (C-terminal) protease, serine 12	CCDS44881.1	chr12_51237705-51237705_C_T	90P>S	Substitution	Nonsynonymous coding	10%
MM12T	TMPRSS13	transmembrane protease, serine 13	CCDS41721.1	chr11_117780668-117780668_G_A	321A>V	Substitution	Nonsynonymous coding	19%
MM12T	TMPRSS9	transmembrane protease, serine 9	CCDS12088.1	chr19_2424155-2424155_C_T	839P>S	Substitution	Nonsynonymous coding	32%
MM12T	TMPRSS9	transmembrane protease, serine 9	CCDS12088.1	chr19_2425415-2425415_G_A	981R>H	Substitution	Nonsynonymous coding	10%
MM12T	TMTC4	transmembrane and tetratricopeptide repeat containing 4	CCDS9497.2	chr13_101289850-101289850_G_A	314P>L	Substitution	Nonsynonymous coding	30%
MM12T	TMX2	thioredoxin-related transmembrane protein 2	CCDS7967.1	chr11_57505132-57505132_G_A	81R>H	Substitution	Nonsynonymous coding	28%
MM12T	TNFAIP2	tumor necrosis factor, alpha-induced protein 2	CCDS9979.1	chr14_103592871-103592871_C_T	26A>V	Substitution	Nonsynonymous coding	34%
MM12T	TNFRSF11B	tumor necrosis factor receptor superfamily, member 11b	CCDS6326.1	chr8_119936834-119936834_G_A	329L>F	Substitution	Nonsynonymous coding	27%
MM12T	TNFRSF11B	tumor necrosis factor receptor superfamily, member 11b	CCDS6326.1	chr8_119941144-119941144_C_T	142C>Y	Substitution	Nonsynonymous coding	43%
MM12T	TNFRSF1B	tumor necrosis factor receptor superfamily, member 1B	CCDS145.1	chr1_12252978-12252978_T_C	204S>P	Substitution	Nonsynonymous coding	33%
MM12T	TNFRSF1B	tumor necrosis factor receptor superfamily, member 1B	CCDS145.1	chr1_12253156-12253156_G_A	ISV+1>	Substitution	Splice site donor	14%
MM12T	TNFRSF21	tumor necrosis factor receptor superfamily, member 21	CCDS4921.1	chr6_47221120-47221120_C_T	461A>T	Substitution	Nonsynonymous coding	11%
MM12T	TNFRSF21	tumor necrosis factor receptor superfamily, member 21	CCDS4921.1	chr6_47252003-47252003_T_C	305H>R	Substitution	Nonsynonymous coding	27%
MM12T	TNFRSF8	tumor necrosis factor receptor superfamily, member 8	CCDS144.1	chr1_12183425-12183425_C_T	344A>V	Substitution	Nonsynonymous coding	28%
MM12T	TNKS	tankyrase, TRF1-interacting ankyrin-related ADP-ribose polymerase	CCDS5974.1	chr8_9623793-9623793_C_T	1200R>X	Substitution	Nonsense	14%
MM12T	TNKS1BP1	tankyrase 1 binding protein 1, 182kDa	CCDS7951.1	chr11_57069677-57069677_C_T	1569A>T	Substitution	Nonsynonymous coding	23%
MM12T	TNMD	tenomodulin	CCDS14469.1	chrX_99854571-99854571_C_T	271R>X	Substitution	Nonsense	22%
MM12T	TNPO1	transportin 1	CCDS43329.1	chr5_72168492-72168492_A_G	208Q>R	Substitution	Nonsynonymous coding	33%
MM12T	TNPO2	transportin 2	CCDS45991.1	chr19_12822202-12822202_G_A	342T>M	Substitution	Nonsynonymous coding	26%
MM12T	TNR	tenascin R	CCDS1318.1	chr1_175328798-175328798_G_A	975A>V	Substitution	Nonsynonymous coding	36%

MM12T	TNRC18	trinucleotide repeat containing 18	CCDS47534.1	chr7_5385222-5385222_C_T	1897R>Q	Substitution	Nonsynonymous coding	10%
MM12T	TNRC18	trinucleotide repeat containing 18	CCDS47534.1	chr7_5413872-5413872_C_T	1015V>M	Substitution	Nonsynonymous coding	40%
MM12T	TNRC6A	trinucleotide repeat containing 6A	CCDS10624.2	chr16_24788620-24788620_C_A	177A>D	Substitution	Nonsynonymous coding	12%
MM12T	TNRC6A	trinucleotide repeat containing 6A	CCDS10624.2	chr16_24830006-24830006_G_A	1689A>T	Substitution	Nonsynonymous coding	17%
MM12T	TNRC6B	trinucleotide repeat containing 6B	CCDS46713.1	chr22_40696519-40696519_G_A	1147V>M	Substitution	Nonsynonymous coding	35%
MM12T	TNRC6C	trinucleotide repeat containing 6C	CCDS45799.1	chr17_76045499-76045499_C_T	119A>V	Substitution	Nonsynonymous coding	35%
MM12T	TNS1	tensin 1	CCDS2407.1	chr2_218674664-218674664_G_A	1615P>S	Substitution	Nonsynonymous coding	23%
MM12T	TNS1	tensin 1	CCDS2407.1	chr2_218747113-218747113_C_T	298G>D	Substitution	Nonsynonymous coding	32%
MM12T	TNS3	tensin 3	CCDS5506.2	chr7_47474969-47474969_G_A	79P>S	Substitution	Nonsynonymous coding	29%
MM12T	TOB1	transducer of ERBB2, 1	CCDS11576.1	chr17_48941234-48941234_G_A	49P>S	Substitution	Nonsynonymous coding	14%
MM12T	TOB2	transducer of ERBB2, 2	CCDS14015.1	chr22_41833183-41833183_C_T	56R>H	Substitution	Nonsynonymous coding	38%
MM12T	TOMM40L	translocase of outer mitochondrial membrane 40 homolog (yeast)-like	CCDS1227.1	chr1_161197728-161197728_G_A	145D>N	Substitution	Nonsynonymous coding	25%
MM12T	TOMM70A	translocase of outer mitochondrial membrane 70 homolog A (S. cerevisiae)	CCDS33807.1	chr3_100119643-100119643_G_T	51L>M	Substitution	Nonsynonymous coding	33%
MM12T	TOP2B	topoisomerase (DNA) II beta 180kDa	CCDS46776.1	chr3_25668727-25668727_C_T	651R>H	Substitution	Nonsynonymous coding	19%
MM12T	TOPAZ1	testis and ovary specific PAZ domain containing 1	CCDS46809.1	chr3_44285883-44285883_A_	NA	Deletion	Frameshift	33%
MM12T	TOPBP1	topoisomerase (DNA) II binding protein 1	CCDS46919.1	chr3_133376731-133376731_G_A	92R>X	Substitution	Nonsense	31%
MM12T	TOR1AIP1	torsin A interacting protein 1	CCDS1335.1	chr1_179883139-179883139_G_A	305S>N	Substitution	Nonsynonymous coding	11%
MM12T	TOR1AIP2	torsin A interacting protein 2	CCDS1334.1	chr1_179815464-179815464_C_A	385E>D	Substitution	Nonsynonymous coding	32%
MM12T	TP53	tumor protein p53	CCDS11118.1	chr17_7577538-7577538_C_T	248R>Q	Substitution	Nonsynonymous coding	31%
MM12T	TP53	tumor protein p53	CCDS11118.1	chr17_7574003-7574003_G_A	342R>X	Substitution	Nonsense	35%
MM12T	TP53INP1	tumor protein p53 inducible nuclear protein 1	CCDS6265.1	chr8_95952091-95952091_G_A	157P>L	Substitution	Nonsynonymous coding	31%
MM12T	TP73	tumor protein p73	CCDS49.1	chr1_3649575-3649575_G_A	615D>N	Substitution	Nonsynonymous coding	14%
MM12T	TP73	tumor protein p73	CCDS49.1	chr1_3645894-3645894_C_T	360R>X	Substitution	Nonsense	11%
MM12T	TPO	thyroid peroxidase	CCDS1643.1	chr2_1481101-1481101_C_T	355R>C	Substitution	Nonsynonymous coding	31%
MM12T	TPO	thyroid peroxidase	CCDS1643.1	chr2_1488494-1488494_G_A	489A>T	Substitution	Nonsynonymous coding	27%
MM12T	TPO	thyroid peroxidase	CCDS1643.1	chr2_1546214-1546214_C_T	924R>W	Substitution	Nonsynonymous coding	11%
MM12T	TPP1	tripeptidyl peptidase I	ENST00000428886	chr11_6638494-6638494_C_A	182Q>H	Substitution	Nonsynonymous coding	32%
MM12T	TPP2	tripeptidyl peptidase II	CCDS9502.1	chr13_103289452-103289452_C_T	567L>F	Substitution	Nonsynonymous coding	13%
MM12T	TPST2	tyrosylprotein sulfotransferase 2	CCDS13839.1	chr22_26937295-26937295_C_T	101R>H	Substitution	Nonsynonymous coding	46%
MM12T	TRAC	T cell receptor alpha constant	ENST00000431063	chr14_22957623-22957623_G_A	19S>N	Substitution	Nonsynonymous coding	19%
MM12T	TRAF1	TNF receptor-associated factor 1	CCDS6825.1	chr9_123675896-123675896_T_C	139M>V	Substitution	Nonsynonymous coding	32%
MM12T	TRAF2	TNF receptor-associated factor 2	CCDS7013.1	chr9_139815644-139815644_G_A	372R>H	Substitution	Nonsynonymous coding	35%

MM12T	TRAF4	TNF receptor-associated factor 4	CCDS11243.1	chr17_27076269-27076269_C_A	363L>I	Substitution	Nonsynonymous coding	14%
MM12T	TRAF5	TNF receptor-associated factor 5	CCDS1497.1	chr1_211538773-211538773_G_A	251R>H	Substitution	Nonsynonymous coding	28%
MM12T	TRAPPC10	trafficking protein particle complex 10	CCDS13704.1	chr21_45499447-45499447_G_T	491R>M	Substitution	Nonsynonymous coding	19%
MM12T	TRAPPC10	trafficking protein particle complex 10	CCDS13704.1	chr21_45499956-45499956_G_T	557K>N	Substitution	Nonsynonymous coding	13%
MM12T	TRAPPC12	trafficking protein particle complex 12	CCDS1652.1	chr2_3483047-3483047_C_T	675R>W	Substitution	Nonsynonymous coding	29%
MM12T	TRAPPC12	trafficking protein particle complex 12	ENST00000304601	chr2_3464015-3464015_G_T	ISV-1>	Substitution	Splice site acceptor	32%
MM12T	TRAPPC2	trafficking protein particle complex 2	CCDS48082.1	chrX_13734658-13734658_G_A	78A>V	Substitution	Nonsynonymous coding	29%
MM12T	TRAPPC8	trafficking protein particle complex 8	CCDS11901.1	chr18_29450445-29450445_C_T	760A>T	Substitution	Nonsynonymous coding	15%
MM12T	TRAPPC9	trafficking protein particle complex 9	CCDS34946.1	chr8_140743441-140743441_C_T	1202G>R	Substitution	Nonsynonymous coding	35%
MM12T	TRAPPC9	trafficking protein particle complex 9	CCDS34946.1	chr8_141285904-141285904_G_A	809Q>X	Substitution	Nonsense	67%
MM12T	TRBV30	T cell receptor beta variable 30 (gene/pseudogene)	ENST00000417977	chr7_142510300-142510300_G_T	102D>E	Substitution	Nonsynonymous coding	13%
MM12T	TRBV4-1	T cell receptor beta variable 4-1	ENST00000390357	chr7_142013329-142013329_C_T	62P>S	Substitution	Nonsynonymous coding	40%
MM12T	TRBV9	T cell receptor beta variable 9	ENST00000390363	chr7_142239712-142239712_C_A	56Q>H	Substitution	Nonsynonymous coding	38%
MM12T	TREX1	three prime repair exonuclease 1	CCDS43086.1	chr3_48508395-48508395_G_A	169R>H	Substitution	Nonsynonymous coding	11%
MM12T	TREX2	three prime repair exonuclease 2	CCDS35437.1	chrX_152710741-152710741_C_T	50A>T	Substitution	Nonsynonymous coding	11%
MM12T	TRHDE	thyrotropin-releasing hormone degrading enzyme	CCDS9004.1	chr12_72666763-72666763_G_A	69G>R	Substitution	Nonsynonymous coding	14%
MM12T	TRIM10	tripartite motif containing 10	CCDS34375.1	chr6_30128329-30128329_C_T	103G>R	Substitution	Nonsynonymous coding	11%
MM12T	TRIM28	tripartite motif containing 28	CCDS12985.1	chr19_59059163-59059163_C_T	282R>C	Substitution	Nonsynonymous coding	34%
MM12T	TRIM29	tripartite motif containing 29	CCDS8428.1	chr11_120007939-120007939_C_A	267K>N	Substitution	Nonsynonymous coding	31%
MM12T	TRIM3	tripartite motif containing 3	CCDS7764.1	chr11_6472230-6472230_G_A	588H>Y	Substitution	Nonsynonymous coding	22%
MM12T	TRIM3	tripartite motif containing 3	CCDS7764.1	chr11_6486922-6486922_C_T	2A>T	Substitution	Nonsynonymous coding	47%
MM12T	TRIM32	tripartite motif containing 32	CCDS6817.1	chr9_119460035-119460035_C_T	5A>V	Substitution	Nonsynonymous coding	33%
MM12T	TRIM32	tripartite motif containing 32	CCDS6817.1	chr9_119460302-119460302_C_T	94A>V	Substitution	Nonsynonymous coding	14%
MM12T	TRIM33	tripartite motif containing 33	CCDS872.1	chr1_114967347-114967347_G_A	576Q>X	Substitution	Nonsense	53%
MM12T	TRIM35	tripartite motif containing 35	CCDS6056.2	chr8_27145186-27145186_C_T	455A>T	Substitution	Nonsynonymous coding	12%
MM12T	TRIM4	tripartite motif containing 4	CCDS5679.1	chr7_99490304-99490304_C_T	329V>M	Substitution	Nonsynonymous coding	25%
MM12T	TRIM4	tripartite motif containing 4	CCDS5679.1	chr7_99500850-99500850_G_A	304R>X	Substitution	Nonsense	16%
MM12T	TRIM42	tripartite motif containing 42	CCDS3113.1	chr3_140397235-140397235_G_A	55C>Y	Substitution	Nonsynonymous coding	12%
MM12T	TRIM47	tripartite motif containing 47	CCDS32737.1	chr17_73872824-73872824_G_A	249S>F	Substitution	Nonsynonymous coding	35%
MM12T	TRIM54	tripartite motif containing 54	CCDS1745.2	chr2_27529128-27529128_G_A	347G>D	Substitution	Nonsynonymous coding	15%
MM12T	TRIM56	tripartite motif containing 56	CCDS43625.1	chr7_100732377-100732377_C_T	595A>V	Substitution	Nonsynonymous coding	34%
MM12T	TRIM59	tripartite motif containing 59	CCDS3190.1	chr3_160156368-160156368_T_	NA	Deletion	Frameshift	15%

MM12T	TRIM77	tripartite motif containing 77	ENST00000398290	chr11_89447496-89447496_G_T	216S>I	Substitution	Nonsynonymous coding	15%
MM12T	TRIM8	tripartite motif containing 8	CCDS31274.1	chr10_104414867-104414867_C_T	233R>W	Substitution	Nonsynonymous coding	32%
MM12T	TRIM9	tripartite motif containing 9	CCDS9703.1	chr14_51561375-51561375_A_G	95S>P	Substitution	Nonsynonymous coding	27%
MM12T	TRIML1	tripartite motif family-like 1	CCDS3851.1	chr4_189063589-189063589_G_A	230A>T	Substitution	Nonsynonymous coding	36%
MM12T	TRIML1	tripartite motif family-like 1	CCDS3851.1	chr4_189068441-189068441_T_G	441L>R	Substitution	Nonsynonymous coding	26%
MM12T	TRIO	trio Rho guanine nucleotide exchange factor	CCDS3883.1	chr5_14304635-14304635_G_T	478Q>H	Substitution	Nonsynonymous coding	28%
MM12T	TRIO	trio Rho guanine nucleotide exchange factor	CCDS3883.1	chr5_14405964-14405964_G_T	1575S>I	Substitution	Nonsynonymous coding	30%
MM12T	TRIO	trio Rho guanine nucleotide exchange factor	CCDS3883.1	chr5_14488001-14488001_G_A	2422A>T	Substitution	Nonsynonymous coding	13%
MM12T	TRIOBP	TRIO and F-actin binding protein	CCDS43015.1	chr22_38097475-38097475_G_A	35A>T	Substitution	Nonsynonymous coding	49%
MM12T	TRIOBP	TRIO and F-actin binding protein	CCDS43015.1	chr22_38121084-38121084_C_T	841R>X	Substitution	Nonsense	36%
MM12T	TRIOBP	TRIO and F-actin binding protein	CCDS43015.1	chr22_38161773-38161773_C_T	2141Q>X	Substitution	Nonsense	15%
MM12T	TRIP10	thyroid hormone receptor interactor 10	CCDS12172.1	chr19_6750420-6750420_G_A	449A>T	Substitution	Nonsynonymous coding	26%
MM12T	TRIP11	thyroid hormone receptor interactor 11	CCDS9899.1	chr14_92436069-92436069_G_A	1963A>V	Substitution	Nonsynonymous coding	35%
MM12T	TRIP12	thyroid hormone receptor interactor 12	CCDS33391.1	chr2_230632378-230632378_C_T	1957M>I	Substitution	Nonsynonymous coding	17%
MM12T	TRIP12	thyroid hormone receptor interactor 12	CCDS33391.1	chr2_230655840-230655840_C_T	1440D>N	Substitution	Nonsynonymous coding	39%
MM12T	TRMT10A	tRNA methyltransferase 10 homolog A (S. cerevisiae)	CCDS3650.1	chr4_100480433-100480433_T_C	21I>M	Substitution	Nonsynonymous coding	12%
MM12T	TRMT2A	tRNA methyltransferase 2 homolog A (S. cerevisiae)	CCDS13774.1	chr22_20100985-20100985_C_T	468E>K	Substitution	Nonsynonymous coding	17%
MM12T	TRMT5	tRNA methyltransferase 5 homolog (S. cerevisiae)	CCDS32092.1	chr14_61444318-61444318_C_T	236A>T	Substitution	Nonsynonymous coding	23%
MM12T	TRMT61B	tRNA methyltransferase 61 homolog B (S. cerevisiae)	CCDS1768.1	chr2_29074130-29074130_G_A	374R>C	Substitution	Nonsynonymous coding	12%
MM12T	TROAP	trophinin associated protein	CCDS8784.1	chr12_49725008-49725008_C_A	704L>M	Substitution	Nonsynonymous coding	27%
MM12T	TROAP	trophinin associated protein	CCDS8784.1	chr12_49719406-49719406_C_T	154R>X	Substitution	Nonsense	15%
MM12T	TRPC5	transient receptor potential cation channel, subfamily C, member 5	CCDS14561.1	chrX_111097007-111097007_A_G	410W>R	Substitution	Nonsynonymous coding	50%
MM12T	TRPC5	transient receptor potential cation channel, subfamily C, member 5	CCDS14561.1	chrX_111097254-111097254_C_T	327W>X	Substitution	Nonsense	12%
MM12T	TRPC6	transient receptor potential cation channel, subfamily C, member 6	CCDS8311.1	chr11_101375261-101375261_C_A	147E>X	Substitution	Nonsense	16%
MM12T	TRPM3	transient receptor potential cation channel, subfamily M, member 3	CCDS43835.1	chr9_73152199-73152199_G_A	1265T>M	Substitution	Nonsynonymous coding	35%
MM12T	TRPM4	transient receptor potential cation channel, subfamily M, member 4	CCDS33073.1	chr19_49685952-49685952_G_A	461A>T	Substitution	Nonsynonymous coding	20%
MM12T	TRPM5	transient receptor potential cation channel, subfamily M, member 5	CCDS31340.1	chr11_2443404-2443404_G_A	89R>C	Substitution	Nonsynonymous coding	28%
MM12T	TRPM6	transient receptor potential cation channel, subfamily M, member 6	CCDS6647.1	chr9_77343217-77343217_G_A	1958A>V	Substitution	Nonsynonymous coding	16%
MM12T	TRPM8	transient receptor potential cation channel, subfamily M, member 8	CCDS33407.1	chr2_234891793-234891793_T_C	896W>R	Substitution	Nonsynonymous coding	28%
MM12T	TRPS1	trichorhinophalangeal syndrome I	CCDS6318.2	chr8_116430586-116430586_G_A	932A>V	Substitution	Nonsynonymous coding	11%
MM12T	TRPV1	transient receptor potential cation channel, subfamily V, member 1	CCDS45576.1	chr17_3475494-3475494_C_T	718R>K	Substitution	Nonsynonymous coding	12%
MM12T	TRPV1	transient receptor potential cation channel, subfamily V, member 1	CCDS45576.1	chr17_3494600-3494600_C_A	111R>M	Substitution	Nonsynonymous coding	34%

MM12T	TRPV2	transient receptor potential cation channel, subfamily V, member 2	CCDS32576.1	chr17_16326863-16326863_G_A	236A>T	Substitution	Nonsynonymous coding	25%
MM12T	TRPV3	transient receptor potential cation channel, subfamily V, member 3	CCDS11029.1	chr17_3436061-3436061_G_A	319R>C	Substitution	Nonsynonymous coding	15%
MM12T	TRPV6	transient receptor potential cation channel, subfamily V, member 6	CCDS5874.1	chr7_142569522-142569522_C_A	706D>Y	Substitution	Nonsynonymous coding	32%
MM12T	TRRAP	transformation/transcription domain-associated protein	CCDS5659.1	chr7_98524917-98524917_C_T	1035R>W	Substitution	Nonsynonymous coding	34%
MM12T	TRRAP	transformation/transcription domain-associated protein	CCDS5659.1	chr7_98527702-98527702_C_A	1089P>H	Substitution	Nonsynonymous coding	31%
MM12T	TRRAP	transformation/transcription domain-associated protein	CCDS5659.1	chr7_98562327-98562327_G_A	2277R>H	Substitution	Nonsynonymous coding	13%
MM12T	TRRAP	transformation/transcription domain-associated protein	CCDS5659.1	chr7_98592334-98592334_C_T	3348T>M	Substitution	Nonsynonymous coding	28%
MM12T	TRRAP	transformation/transcription domain-associated protein	CCDS5659.1	chr7_98606086-98606086_C_T	3571R>C	Substitution	Nonsynonymous coding	34%
MM12T	TRUB2	TruB pseudouridine (psi) synthase homolog 2 (E. coli)	CCDS6897.1	chr9_131072055-131072055_C_T	257R>H	Substitution	Nonsynonymous coding	13%
MM12T	TRUB2	TruB pseudouridine (psi) synthase homolog 2 (E. coli)	CCDS6897.1	chr9_131083905-131083905_C_T	72V>I	Substitution	Nonsynonymous coding	35%
MM12T	TSC1	tuberous sclerosis 1	CCDS6956.1	chr9_135773001-135773004_AAAA_	NA	Deletion	Splice site acceptor	14%
MM12T	TSC2	tuberous sclerosis 2	CCDS10458.1	chr16_2126544-2126544_G_A	932S>N	Substitution	Nonsynonymous coding	26%
MM12T	TSC22D1	TSC22 domain family, member 1	CCDS31966.1	chr13_45008790-45008790_G_A	1065A>V	Substitution	Nonsynonymous coding	15%
MM12T	TSC22D2	TSC22 domain family, member 2	CCDS3149.1	chr3_150127556-150127556_C_T	140A>V	Substitution	Nonsynonymous coding	28%
MM12T	TSEN54	tRNA splicing endonuclease 54 homolog (S. cerevisiae)	CCDS11724.1	chr17_73513243-73513243_G_A	96G>D	Substitution	Nonsynonymous coding	43%
MM12T	TSGA10	testis specific, 10	CCDS2037.1	chr2_99688246-99688246_C_T	344A>T	Substitution	Nonsynonymous coding	24%
MM12T	TSGA10IP	testis specific, 10 interacting protein	NM_152762	chr11_65726388-65726388_G_A	453A>T	Substitution	Nonsynonymous coding	30%
MM12T	TSHR	thyroid stimulating hormone receptor	CCDS9872.1	chr14_81610021-81610021_C_T	540A>V	Substitution	Nonsynonymous coding	32%
MM12T	TSNARE1	t-SNARE domain containing 1	CCDS6384.1	chr8_143365807-143365807_C_T	444A>T	Substitution	Nonsynonymous coding	28%
MM12T	TSPAN10	tetraspanin 10	ENST00000328585	chr17_79615197-79615197_G_A	314R>Q	Substitution	Nonsynonymous coding	42%
MM12T	TSPAN11	tetraspanin 11	CCDS31765.1	chr12_31144821-31144821_C_T	245H>Y	Substitution	Nonsynonymous coding	34%
MM12T	TSPAN15	tetraspanin 15	CCDS7294.1	chr10_71265928-71265928_G_A	223V>M	Substitution	Nonsynonymous coding	23%
MM12T	TSPAN17	tetraspanin 17	ENST00000414405	chr5_176081482-176081482_G_T	35A>S	Substitution	Nonsynonymous coding	20%
MM12T	TSPEAR	thrombospondin-type laminin G domain and EAR repeats	CCDS13712.1	chr21_45953644-45953644_G_A	156R>C	Substitution	Nonsynonymous coding	28%
MM12T	TSPO	translocator protein (18kDa)	CCDS33661.1	chr22_43555296-43555296_G_T	18G>W	Substitution	Nonsynonymous coding	34%
MM12T	TSPYL1	TSPY-like 1	CCDS34518.1	chr6_116599778-116599778_G_A	406Q>X	Substitution	Nonsense	16%
MM12T	TSSC1	tumor suppressing subtransferable candidate 1	CCDS1651.1	chr2_3196221-3196221_T_C	318D>G	Substitution	Nonsynonymous coding	29%
MM12T	TSSK2	testis-specific serine kinase 2	CCDS13755.1	chr22_19119445-19119445_C_T	178S>L	Substitution	Nonsynonymous coding	13%
MM12T	TSSK2	testis-specific serine kinase 2	CCDS13755.1	chr22_19119589-19119589_G_A	226R>H	Substitution	Nonsynonymous coding	25%
MM12T	TSSK4	testis-specific serine kinase 4	CCDS9618.1	chr14_24675239-24675239_G_A	37G>E	Substitution	Nonsynonymous coding	32%
MM12T	TSTD2	thiosulfate sulfurtransferase (rhodanese)-like domain containing 2	CCDS6727.2	chr9_100372737-100372737_G_A	ISV-4>	Substitution	Splice site acceptor	35%
MM12T	TTBK2	tau tubulin kinase 2	CCDS42029.1	chr15_43045118-43045118_C_T	776E>K	Substitution	Nonsynonymous coding	23%

MM12T	TTBK2	tau tubulin kinase 2	CCDS42029.1	chr15_43122137-43122137_G_A	144P>L	Substitution	Nonsynonymous coding	12%
MM12T	TTC21A	tetratricopeptide repeat domain 21A	CCDS43068.1	chr3_39154001-39154001_C_T	163A>V	Substitution	Nonsynonymous coding	30%
MM12T	TTC21A	tetratricopeptide repeat domain 21A	CCDS43068.1	chr3_39170371-39170371_G_A	623R>Q	Substitution	Nonsynonymous coding	33%
MM12T	TTC21B	tetratricopeptide repeat domain 21B	CCDS33315.1	chr2_166789518-166789518_G_A	254L>F	Substitution	Nonsynonymous coding	15%
MM12T	TTC22	tetratricopeptide repeat domain 22	CCDS44152.1	chr1_55266373-55266373_T_C	155Y>C	Substitution	Nonsynonymous coding	23%
MM12T	TTC23	tetratricopeptide repeat domain 23	CCDS10379.2	chr15_99740129-99740129_G_T	252L>I	Substitution	Nonsynonymous coding	26%
MM12T	TTC23L	tetratricopeptide repeat domain 23-like	NM_144725	chr5_34863159-34863159_G_A	179R>H	Substitution	Nonsynonymous coding	13%
MM12T	TTC23L	tetratricopeptide repeat domain 23-like	NM_144725	chr5_34866993-34866993_G_A	ISV-4>	Substitution	Splice site acceptor	37%
MM12T	TTC27	tetratricopeptide repeat domain 27	CCDS33176.1	chr2_32875262-32875262_C_T	201P>L	Substitution	Nonsynonymous coding	13%
MM12T	TTC29	tetratricopeptide repeat domain 29	CCDS47141.1	chr4_147628706-147628706_A_	NA	Deletion	Splice site acceptor	29%
MM12T	TTC37	tetratricopeptide repeat domain 37	CCDS4072.1	chr5_94842719-94842719_A_	NA	Deletion	Splice site acceptor	45%
MM12T	TTC39A	tetratricopeptide repeat domain 39A	CCDS44143.1	chr1_51760073-51760073_A_G	423V>A	Substitution	Nonsynonymous coding	29%
MM12T	TTC40	tetratricopeptide repeat domain 40	ENST00000368582	chr10_134693009-134693009_G_A	1309A>V	Substitution	Nonsynonymous coding	34%
MM12T	TTC40	tetratricopeptide repeat domain 40	ENST00000368582	chr10_134730116-134730116_C_T	686S>N	Substitution	Nonsynonymous coding	19%
MM12T	TTC4P1	tetratricopeptide repeat domain 4 pseudogene 1	CCDS596.1	chr1_55194097-55194097_G_A	225A>T	Substitution	Nonsynonymous coding	14%
MM12T	TTC5	tetratricopeptide repeat domain 5	CCDS9546.1	chr14_20774090-20774090_C_T	3A>T	Substitution	Nonsynonymous coding	10%
MM12T	TTC7B	tetratricopeptide repeat domain 7B	CCDS32140.1	chr14_91110542-91110542_G_A	534A>V	Substitution	Nonsynonymous coding	18%
MM12T	TTC7B	tetratricopeptide repeat domain 7B	CCDS32140.1	chr14_91161849-91161849_G_A	258R>X	Substitution	Nonsense	16%
MM12T	TTC9C	tetratricopeptide repeat domain 9C	CCDS8033.1	chr11_62502916-62502916_C_A	101L>M	Substitution	Nonsynonymous coding	27%
MM12T	TTF1	transcription termination factor, RNA polymerase I	CCDS6948.1	chr9_135276936-135276936_C_T	425D>N	Substitution	Nonsynonymous coding	34%
MM12T	TTF2	transcription termination factor, RNA polymerase II	CCDS892.1	chr1_117618210-117618210_C_T	335A>V	Substitution	Nonsynonymous coding	13%
MM12T	TTI1	TELO2 interacting protein 1	CCDS13300.1	chr20_36640651-36640651_G_A	523A>V	Substitution	Nonsynonymous coding	19%
MM12T	TTI1	TELO2 interacting protein 1	CCDS13300.1	chr20_36641881-36641881_G_A	113A>V	Substitution	Nonsynonymous coding	13%
MM12T	TTI2	TELO2 interacting protein 2	CCDS6090.1	chr8_33369951-33369951_C_T	61A>T	Substitution	Nonsynonymous coding	31%
MM12T	TTK	TTK protein kinase	CCDS4993.1	chr6_80720630-80720630__A	NA	Insertion	Frameshift	45%
MM12T	TTL	tubulin tyrosine ligase	CCDS2096.1	chr2_113239953-113239953_G_A	11S>N	Substitution	Nonsynonymous coding	12%
MM12T	TTLL10	tubulin tyrosine ligase-like family, member 10	CCDS44036.1	chr1_1118431-1118431_C_T	ISV+4>	Substitution	Splice site donor	39%
MM12T	TTLL11	tubulin tyrosine ligase-like family, member 11	CCDS6834.2	chr9_124751418-124751418_G_A	532A>V	Substitution	Nonsynonymous coding	20%
MM12T	TTLL4	tubulin tyrosine ligase-like family, member 4	CCDS2422.1	chr2_219611918-219611918_G_T	ISV+1>	Substitution	Splice site donor	28%
MM12T	TTN	titin	ENST00000356127	chr2_179404312-179404312_T_C	30257D>G	Substitution	Nonsynonymous coding	36%
MM12T	TTN	titin	ENST00000356127	chr2_179410711-179410711_C_T	29181S>N	Substitution	Nonsynonymous coding	13%
MM12T	TTN	titin	ENST00000356127	chr2_179411796-179411796_G_A	28916L>F	Substitution	Nonsynonymous coding	38%

MM12T	TTN	titin	ENST00000356127	chr2_179429978-179429978_C_T	24391A>T	Substitution	Nonsynonymous coding	29%
MM12T	TTN	titin	ENST00000356127	chr2_179432858-179432858_C_T	23431A>T	Substitution	Nonsynonymous coding	14%
MM12T	TTN	titin	ENST00000356127	chr2_179439512-179439512_G_A	21213R>C	Substitution	Nonsynonymous coding	18%
MM12T	TTN	titin	ENST00000356127	chr2_179444893-179444893_G_A	19804T>I	Substitution	Nonsynonymous coding	19%
MM12T	TTN	titin	ENST00000356127	chr2_179466262-179466262_C_T	15920V>I	Substitution	Nonsynonymous coding	38%
MM12T	TTN	titin	ENST00000356127	chr2_179548802-179548802_C_A	9666E>D	Substitution	Nonsynonymous coding	13%
MM12T	TTN	titin	ENST00000356127	chr2_179553484-179553484_A_G	9462V>A	Substitution	Nonsynonymous coding	25%
MM12T	TTN	titin	ENST00000356127	chr2_179600592-179600592_C_T	3617D>N	Substitution	Nonsynonymous coding	11%
MM12T	TTN	titin	ENST00000342175	chr2_179605095-179605095_G_A	4118P>S	Substitution	Nonsynonymous coding	30%
MM12T	TTN	titin	NM_133379	chr2_179612149-179612149_G_T	4993S>Y	Substitution	Nonsynonymous coding	26%
MM12T	TTN	titin	NM_133379	chr2_179612744-179612744_G_A	4795P>S	Substitution	Nonsynonymous coding	38%
MM12T	TTN	titin	NM_133379	chr2_179641361-179641361_G_A	1744P>S	Substitution	Nonsynonymous coding	41%
MM12T	TTN	titin	NM_133379	chr2_179647077-179647077_G_A	1081A>V	Substitution	Nonsynonymous coding	18%
MM12T	TTYH2	tweety homolog 2 (Drosophila)	CCDS32717.1	chr17_72240181-72240181_C_T	266A>V	Substitution	Nonsynonymous coding	14%
MM12T	TUB	tubby homolog (mouse)	CCDS7786.1	chr11_8111688-8111688_C_T	110R>W	Substitution	Nonsynonymous coding	23%
MM12T	TUB	tubby homolog (mouse)	CCDS7786.1	chr11_8111714-8111714_G_T	118Q>H	Substitution	Nonsynonymous coding	24%
MM12T	TUB	tubby homolog (mouse)	CCDS7786.1	chr11_8123062-8123062_G_A	528V>I	Substitution	Nonsynonymous coding	34%
MM12T	TUBB1	tubulin, beta 1 class VI	CCDS13475.1	chr20_57599135-57599135_C_T	218T>M	Substitution	Nonsynonymous coding	31%
MM12T	TUBB6	tubulin, beta 6 class V	CCDS11858.1	chr18_12325117-12325117_C_T	110A>V	Substitution	Nonsynonymous coding	11%
MM12T	TUBGCP2	tubulin, gamma complex associated protein 2	CCDS7676.1	chr10_135101802-135101802_C_T	518R>H	Substitution	Nonsynonymous coding	37%
MM12T	TUBGCP6	tubulin, gamma complex associated protein 6	CCDS14087.1	chr22_50656526-50656526_G_A	1730A>V	Substitution	Nonsynonymous coding	11%
MM12T	TUBGCP6	tubulin, gamma complex associated protein 6	CCDS14087.1	chr22_50660914-50660914_C_T	793R>H	Substitution	Nonsynonymous coding	37%
MM12T	TUBGCP6	tubulin, gamma complex associated protein 6	CCDS14087.1	chr22_50662636-50662636_G_A	735A>V	Substitution	Nonsynonymous coding	18%
MM12T	TUSC5	tumor suppressor candidate 5	CCDS42225.1	chr17_1183567-1183567_C_T	91T>I	Substitution	Nonsynonymous coding	14%
MM12T	TUT1	terminal uridylyl transferase 1, U6 snRNA-specific	CCDS8021.1	chr11_62342592-62342592_G_A	867P>S	Substitution	Nonsynonymous coding	13%
MM12T	TWF1	twinfilin, actin-binding protein, homolog 1 (Drosophila)	CCDS31780.1	chr12_44194334-44194334_C_A	ISV-1>	Substitution	Splice site acceptor	39%
MM12T	TXLNG	taxilin gamma	CCDS14178.1	chrX_16859845-16859845_C_T	515P>S	Substitution	Nonsynonymous coding	29%
MM12T	TXN2	thioredoxin 2	CCDS13928.1	chr22_36876621-36876621_C_T	ISV+1>	Substitution	Splice site donor	11%
MM12T	TXNDC16	thioredoxin domain containing 16	CCDS32083.1	chr14_53004330-53004330_C_A	102A>S	Substitution	Nonsynonymous coding	24%
MM12T	TXNDC2	thioredoxin domain containing 2 (spermatozoa)	CCDS42414.1	chr18_9887924-9887924_G_A	483C>Y	Substitution	Nonsynonymous coding	30%
MM12T	TXNL1	thioredoxin-like 1	CCDS11961.1	chr18_54278226-54278226_G_A	280R>X	Substitution	Nonsense	23%
MM12T	TXNRD2	thioredoxin reductase 2	ENST00000334361	chr22_19906396-19906396_C_T	121V>M	Substitution	Nonsynonymous coding	63%

MM12T	TYMS	thymidylate synthetase	CCDS11821.1	chr18_670725-670725_C_T	197A>V	Substitution	Nonsynonymous coding	32%
MM12T	TYMS	thymidylate synthetase	CCDS11821.1	chr18_669163-669163_G_A	182W>X	Substitution	Nonsense	22%
MM12T	TYRP1	tyrosinase-related protein 1	CCDS34990.1	chr9_12708979-12708979_C_T	471R>W	Substitution	Nonsynonymous coding	12%
MM12T	TYSND1	trypsin domain containing 1	CCDS31213.1	chr10_71906320-71906320_G_A	8A>V	Substitution	Nonsynonymous coding	14%
MM12T	TYW1B	tRNA-yW synthesizing protein 1 homolog B (S. cerevisiae)	ENST00000438904	chr7_72277853-72277853_C_T	134G>S	Substitution	Nonsynonymous coding	40%
MM12T	TYW1B	tRNA-yW synthesizing protein 1 homolog B (S. cerevisiae)	ENST00000438904	chr7_72277903-72277903_C_T	117R>H	Substitution	Nonsynonymous coding	38%
MM12T	U2AF1L4	U2 small nuclear RNA auxiliary factor 1-like 4	CCDS12473.1	chr19_36233654-36233654_C_T	152G>S	Substitution	Nonsynonymous coding	29%
MM12T	U2AF2	U2 small nuclear RNA auxiliary factor 2	CCDS12933.1	chr19_56180147-56180147_G_A	312V>I	Substitution	Nonsynonymous coding	15%
MM12T	U2SURP	U2 snRNP-associated SURP domain containing	CCDS46928.1	chr3_142753780-142753780_G_A	635R>H	Substitution	Nonsynonymous coding	31%
MM12T	UBA1	ubiquitin-like modifier activating enzyme 1	CCDS14275.1	chrX_47065510-47065510_C_T	580A>V	Substitution	Nonsynonymous coding	28%
MM12T	UBA3	ubiquitin-like modifier activating enzyme 3	CCDS2909.1	chr3_69112658-69112658_C_	NA	Deletion	Splice site acceptor	20%
MM12T	UBA7	ubiquitin-like modifier activating enzyme 7	CCDS2805.1	chr3_49850093-49850093_C_A	182Q>H	Substitution	Nonsynonymous coding	11%
MM12T	UBAP2L	ubiquitin associated protein 2-like	CCDS1063.1	chr1_154226471-154226471_C_T	587A>V	Substitution	Nonsynonymous coding	28%
MM12T	UBE2B	ubiquitin-conjugating enzyme E2B	CCDS4174.1	chr5_133725989-133725989_G_A	135R>Q	Substitution	Nonsynonymous coding	14%
MM12T	UBE2D2	ubiquitin-conjugating enzyme E2D 2	CCDS43369.1	chr5_138994515-138994515_C_T	90R>X	Substitution	Nonsense	23%
MM12T	UBE2O	ubiquitin-conjugating enzyme E2O	CCDS32742.1	chr17_74388095-74388095_G_A	1016P>S	Substitution	Nonsynonymous coding	18%
MM12T	UBE2Z	ubiquitin-conjugating enzyme E2Z	CCDS11540.2	chr17_46990214-46990214_G_A	137G>S	Substitution	Nonsynonymous coding	23%
MM12T	UBE4B	ubiquitination factor E4B	CCDS41245.1	chr1_10205077-10205077_C_T	815R>C	Substitution	Nonsynonymous coding	28%
MM12T	UBE4B	ubiquitination factor E4B	CCDS41245.1	chr1_10239470-10239470_G_A	ISV-4>	Substitution	Splice site acceptor	15%
MM12T	UBL3	ubiquitin-like 3	CCDS9334.1	chr13_30346279-30346279_G_A	65R>X	Substitution	Nonsense	35%
MM12T	UBLCP1	ubiquitin-like domain containing CTD phosphatase 1	CCDS4345.1	chr5_158705280-158705280_C_T	240S>L	Substitution	Nonsynonymous coding	14%
MM12T	UBN2	ubinuclein 2	CCDS43655.1	chr7_138977990-138977990_G_T	1145A>S	Substitution	Nonsynonymous coding	34%
MM12T	UBQLN3	ubiquilin 3	CCDS7758.1	chr11_5530086-5530086_G_A	235R>C	Substitution	Nonsynonymous coding	27%
MM12T	UBR3	ubiquitin protein ligase E3 component n-recognin 3 (putative)	CCDS2238.2	chr2_170783910-170783910_G_A	807D>N	Substitution	Nonsynonymous coding	15%
MM12T	UBR4	ubiquitin protein ligase E3 component n-recognin 4	CCDS189.1	chr1_19407898-19407898_G_A	5060R>W	Substitution	Nonsynonymous coding	36%
MM12T	UBR4	ubiquitin protein ligase E3 component n-recognin 4	CCDS189.1	chr1_19413279-19413279_G_A	4861R>W	Substitution	Nonsynonymous coding	10%
MM12T	UBR4	ubiquitin protein ligase E3 component n-recognin 4	CCDS189.1	chr1_19420543-19420543_G_A	4613L>F	Substitution	Nonsynonymous coding	34%
MM12T	UBR4	ubiquitin protein ligase E3 component n-recognin 4	CCDS189.1	chr1_19421428-19421428_G_A	4573S>F	Substitution	Nonsynonymous coding	33%
MM12T	UBR4	ubiquitin protein ligase E3 component n-recognin 4	CCDS189.1	chr1_19499451-19499451_G_T	1143A>D	Substitution	Nonsynonymous coding	33%
MM12T	UBR4	ubiquitin protein ligase E3 component n-recognin 4	CCDS189.1	chr1_19524176-19524176_G_T	294A>D	Substitution	Nonsynonymous coding	14%
MM12T	UBTD1	ubiquitin domain containing 1	CCDS7465.1	chr10_99330089-99330089_G_A	165A>T	Substitution	Nonsynonymous coding	11%
MM12T	UBXN1	UBX domain protein 1	CCDS8029.1	chr11_62445523-62445523_G_A	120R>W	Substitution	Nonsynonymous coding	23%

MM12T	UCKL1	uridine-cytidine kinase 1-like 1	CCDS13547.1	chr20_62572556-62572556_G_A	310A>V	Substitution	Nonsynonymous coding	17%
MM12T	UCKL1	uridine-cytidine kinase 1-like 1	CCDS13547.1	chr20_62576006-62576006_C_T	246G>S	Substitution	Nonsynonymous coding	35%
MM12T	UGGT1	UDP-glucose glycoprotein glucosyltransferase 1	CCDS2154.1	chr2_128885017-128885017_A_C	406D>A	Substitution	Nonsynonymous coding	26%
MM12T	UGGT2	UDP-glucose glycoprotein glucosyltransferase 2	CCDS9480.1	chr13_96665659-96665659_C_T	188A>T	Substitution	Nonsynonymous coding	35%
MM12T	UGT2A1	UDP glucuronosyltransferase 2 family, polypeptide A1, complex locus	CCDS3529.1	chr4_70513136-70513136_A_G	76V>A	Substitution	Nonsynonymous coding	43%
MM12T	UHRF1BP1L	UHRF1 binding protein 1-like	CCDS31882.1	chr12_100453807-100453807_G_T	522L>I	Substitution	Nonsynonymous coding	26%
MM12T	ULK1	unc-51-like kinase 1 (C. elegans)	CCDS9274.1	chr12_132393189-132393189_C_T	106A>V	Substitution	Nonsynonymous coding	44%
MM12T	ULK1	unc-51-like kinase 1 (C. elegans)	CCDS9274.1	chr12_132393282-132393282_G_A	137R>H	Substitution	Nonsynonymous coding	31%
MM12T	ULK2	unc-51-like kinase 2 (C. elegans)	CCDS11213.1	chr17_19750061-19750061_C_T	153R>H	Substitution	Nonsynonymous coding	14%
MM12T	ULK4	unc-51-like kinase 4 (C. elegans)	CCDS43071.1	chr3_41877403-41877403_A_C	573C>G	Substitution	Nonsynonymous coding	38%
MM12T	UMODL1	uromodulin-like 1	CCDS42935.1	chr21_43496356-43496356_C_T	107P>S	Substitution	Nonsynonymous coding	12%
MM12T	UNC119B	unc-119 homolog B (C. elegans)	CCDS31914.1	chr12_121154485-121154485_G_A	138R>H	Substitution	Nonsynonymous coding	22%
MM12T	UNC13A	unc-13 homolog A (C. elegans)	CCDS46013.1	chr19_17753753-17753753_T_G	879K>N	Substitution	Nonsynonymous coding	32%
MM12T	UNC13C	unc-13 homolog C (C. elegans)	CCDS45264.1	chr15_54307637-54307637_C_T	846T>I	Substitution	Nonsynonymous coding	31%
MM12T	UNC13C	unc-13 homolog C (C. elegans)	CCDS45264.1	chr15_54707232-54707232_C_A	1634L>I	Substitution	Nonsynonymous coding	21%
MM12T	UNC45B	unc-45 homolog B (C. elegans)	CCDS11292.1	chr17_33495337-33495337_A_G	470Y>C	Substitution	Nonsynonymous coding	10%
MM12T	UNC45B	unc-45 homolog B (C. elegans)	CCDS11292.1	chr17_33496947-33496947_G_A	515R>H	Substitution	Nonsynonymous coding	36%
MM12T	UNC50	unc-50 homolog (C. elegans)	CCDS2035.1	chr2_99232993-99232993_A_C	214S>R	Substitution	Nonsynonymous coding	33%
MM12T	UNC5B	unc-5 homolog B (C. elegans)	CCDS7309.1	chr10_73056446-73056446_C_T	813R>W	Substitution	Nonsynonymous coding	23%
MM12T	UNC5C	unc-5 homolog C (C. elegans)	CCDS3643.1	chr4_96222873-96222873_T_G	125E>A	Substitution	Nonsynonymous coding	20%
MM12T	UNC5CL	unc-5 homolog C (C. elegans)-like	CCDS4847.1	chr6_41000845-41000845_C_A	243A>S	Substitution	Nonsynonymous coding	11%
MM12T	UNC79	unc-79 homolog (C. elegans)	CCDS9911.2	chr14_94088132-94088132_G_A	1341R>H	Substitution	Nonsynonymous coding	31%
MM12T	UNC93B1	unc-93 homolog B1 (C. elegans)	ENST00000227471	chr11_67764155-67764155_C_T	335R>H	Substitution	Nonsynonymous coding	26%
MM12T	UNK	unkempt homolog (Drosophila)	CCDS45778.1	chr17_73819534-73819534_G_T	812E>D	Substitution	Nonsynonymous coding	19%
MM12T	UPF1	UPF1 regulator of nonsense transcripts homolog (yeast)	CCDS12386.1	chr19_18976507-18976507_G_A	1053A>T	Substitution	Nonsynonymous coding	29%
MM12T	UPK2	uroplakin 2	CCDS8404.1	chr11_118828884-118828884_G_A	166A>T	Substitution	Nonsynonymous coding	10%
MM12T	UPK3A	uroplakin 3A	CCDS14064.1	chr22_45681896-45681896_G_A	43E>K	Substitution	Nonsynonymous coding	12%
MM12T	UQCRC2	ubiquinol-cytochrome c reductase core protein II	CCDS10601.1	chr16_21968593-21968593_C_T	24A>V	Substitution	Nonsynonymous coding	43%
MM12T	URB1	URB1 ribosome biogenesis 1 homolog (S. cerevisiae)	CCDS46645.1	chr21_33717849-33717849_G_T	1302L>M	Substitution	Nonsynonymous coding	34%
MM12T	URB2	URB2 ribosome biogenesis 2 homolog (S. cerevisiae)	CCDS31052.1	chr1_229773692-229773692_G_A	1111R>H	Substitution	Nonsynonymous coding	12%
MM12T	URB2	URB2 ribosome biogenesis 2 homolog (S. cerevisiae)	CCDS31052.1	chr1_229790035-229790035_G_A	1426R>H	Substitution	Nonsynonymous coding	34%
MM12T	URB2	URB2 ribosome biogenesis 2 homolog (S. cerevisiae)	CCDS31052.1	chr1_229768102-229768102_G_A	71W>X	Substitution	Nonsense	41%

MM12T	UROC1	urocanate hydratase 1	CCDS3038.1	chr3_126218249-126218249_G_A	416A>V	Substitution	Nonsynonymous coding	32%
MM12T	UROD	uroporphyrinogen decarboxylase	CCDS518.1	chr1_45481018-45481018_G_A	318G>R	Substitution	Nonsynonymous coding	53%
MM12T	USF1	upstream transcription factor 1	CCDS1214.1	chr1_161011504-161011504_C_T	137A>T	Substitution	Nonsynonymous coding	11%
MM12T	USH1C	Usher syndrome 1C (autosomal recessive, severe)	CCDS7825.1	chr11_17526205-17526205_C_A	725A>S	Substitution	Nonsynonymous coding	19%
MM12T	USH1C	Usher syndrome 1C (autosomal recessive, severe)	CCDS7825.1	chr11_17531123-17531123_C_T	598R>H	Substitution	Nonsynonymous coding	35%
MM12T	USH1C	Usher syndrome 1C (autosomal recessive, severe)	CCDS7825.1	chr11_17565827-17565827_G_A	10R>W	Substitution	Nonsynonymous coding	16%
MM12T	USH2A	Usher syndrome 2A (autosomal recessive, mild)	CCDS31025.1	chr1_216062028-216062028_C_T	2655E>K	Substitution	Nonsynonymous coding	26%
MM12T	USO1	USO1 vesicle transport factor	NM_003715	chr4_76711833-76711833_G_A	324M>I	Substitution	Nonsynonymous coding	18%
MM12T	USP10	ubiquitin specific peptidase 10	CCDS45537.1	chr16_84812515-84812515_G_A	742G>S	Substitution	Nonsynonymous coding	28%
MM12T	USP11	ubiquitin specific peptidase 11	CCDS14277.1	chrX_47100229-47100229_G_A	310G>D	Substitution	Nonsynonymous coding	17%
MM12T	USP14	ubiquitin specific peptidase 14 (tRNA-guanine transglycosylase)	CCDS32780.1	chr18_178960-178960_G_A	75A>T	Substitution	Nonsynonymous coding	26%
MM12T	USP15	ubiquitin specific peptidase 15	CCDS8963.1	chr12_62777759-62777759_G_A	381A>T	Substitution	Nonsynonymous coding	25%
MM12T	USP19	ubiquitin specific peptidase 19	CCDS43090.1	chr3_49149389-49149389_C_T	850R>H	Substitution	Nonsynonymous coding	23%
MM12T	USP19	ubiquitin specific peptidase 19	CCDS43090.1	chr3_49151620-49151620_C_A	694S>I	Substitution	Nonsynonymous coding	14%
MM12T	USP2	ubiquitin specific peptidase 2	CCDS8422.1	chr11_119244177-119244177_G_A	5S>F	Substitution	Nonsynonymous coding	30%
MM12T	USP20	ubiquitin specific peptidase 20	CCDS43892.1	chr9_132630515-132630515_G_A	308E>K	Substitution	Nonsynonymous coding	32%
MM12T	USP21	ubiquitin specific peptidase 21	CCDS30920.1	chr1_161130827-161130827_G_A	133G>R	Substitution	Nonsynonymous coding	31%
MM12T	USP21	ubiquitin specific peptidase 21	CCDS30920.1	chr1_161134825-161134825_C_A	500P>H	Substitution	Nonsynonymous coding	14%
MM12T	USP22	ubiquitin specific peptidase 22	CCDS42285.1	chr17_20924496-20924496_C_A	116Q>H	Substitution	Nonsynonymous coding	35%
MM12T	USP22	ubiquitin specific peptidase 22	CCDS42285.1	chr17_20931866-20931866_C_T	98R>Q	Substitution	Nonsynonymous coding	19%
MM12T	USP24	ubiquitin specific peptidase 24	CCDS44154.1	chr1_55547094-55547094_G_A	2167R>C	Substitution	Nonsynonymous coding	12%
MM12T	USP26	ubiquitin specific peptidase 26	CCDS14635.1	chrX_132160816-132160816_G_T	478S>Y	Substitution	Nonsynonymous coding	11%
MM12T	USP28	ubiquitin specific peptidase 28	CCDS31680.1	chr11_113672244-113672244_T_C	1007R>G	Substitution	Nonsynonymous coding	20%
MM12T	USP28	ubiquitin specific peptidase 28	CCDS31680.1	chr11_113704982-113704982_G_A	204R>X	Substitution	Nonsense	24%
MM12T	USP35	ubiquitin specific peptidase 35	CCDS41693.1	chr11_77907680-77907680_G_A	130R>H	Substitution	Nonsynonymous coding	50%
MM12T	USP35	ubiquitin specific peptidase 35	CCDS41693.1	chr11_77921591-77921591_G_A	897R>Q	Substitution	Nonsynonymous coding	23%
MM12T	USP4	ubiquitin specific peptidase 4 (proto-oncogene)	CCDS2793.1	chr3_49339841-49339841_C_A	421K>N	Substitution	Nonsynonymous coding	47%
MM12T	USP4	ubiquitin specific peptidase 4 (proto-oncogene)	ENST00000415188	chr3_49354915-49354915__T	NA	Insertion	Splice site acceptor	20%
MM12T	USP43	ubiquitin specific peptidase 43	CCDS45610.1	chr17_9596537-9596537_G_A	483A>T	Substitution	Nonsynonymous coding	33%
MM12T	USP47	ubiquitin specific peptidase 47	CCDS41619.1	chr11_11924837-11924837_T_C	188L>P	Substitution	Nonsynonymous coding	30%
MM12T	USP8	ubiquitin specific peptidase 8	CCDS10137.1	chr15_50784990-50784990_T_C	776L>P	Substitution	Nonsynonymous coding	23%
MM12T	USPL1	ubiquitin specific peptidase like 1	CCDS9336.1	chr13_31232745-31232745_C_A	844A>D	Substitution	Nonsynonymous coding	30%

MM12T	USPL1	ubiquitin specific peptidase like 1	CCDS9336.1	chr13_31232906-31232906_G_A	898A>T	Substitution	Nonsynonymous coding	11%
MM12T	UTP18	UTP18 small subunit (SSU) processome component homolog (yeast)	CCDS42362.1	chr17_49343617-49343617_A_G	177N>S	Substitution	Nonsynonymous coding	35%
MM12T	UTP20	UTP20, small subunit (SSU) processome component, homolog (yeast)	CCDS9081.1	chr12_101748645-101748645_G_A	1715A>T	Substitution	Nonsynonymous coding	13%
MM12T	UTP20	UTP20, small subunit (SSU) processome component, homolog (yeast)	CCDS9081.1	chr12_101773199-101773199_G_A	2483A>T	Substitution	Nonsynonymous coding	24%
MM12T	UTP3	UTP3, small subunit (SSU) processome component, homolog (S. cerevisiae)	CCDS3546.1	chr4_71555596-71555596_C_T	401A>V	Substitution	Nonsynonymous coding	32%
MM12T	UTRN	utrophin	ENST00000367526	chr6_144904630-144904630_T_C	13L>P	Substitution	Nonsynonymous coding	26%
MM12T	UTRN	utrophin	CCDS34547.1	chr6_145093060-145093060_C_T	2838T>I	Substitution	Nonsynonymous coding	17%
MM12T	UTRN	utrophin	CCDS34547.1	chr6_145103201-145103201_G_A	2926V>I	Substitution	Nonsynonymous coding	34%
MM12T	VANGL2	vang-like 2 (van gogh, Drosophila)	CCDS30915.1	chr1_160388873-160388873_G_A	92A>T	Substitution	Nonsynonymous coding	14%
MM12T	VARS2	valyl-tRNA synthetase 2, mitochondrial	CCDS34387.1	chr6_30883146-30883146_C_A	100L>M	Substitution	Nonsynonymous coding	19%
MM12T	VASN	vasorin	CCDS10514.1	chr16_4431203-4431203_G_A	109A>T	Substitution	Nonsynonymous coding	31%
MM12T	VAV1	vav 1 guanine nucleotide exchange factor	CCDS12174.1	chr19_6820777-6820777_G_A	90R>Q	Substitution	Nonsynonymous coding	29%
MM12T	VAV1	vav 1 guanine nucleotide exchange factor	CCDS12174.1	chr19_6828455-6828455_C_T	350A>V	Substitution	Nonsynonymous coding	13%
MM12T	VAX1	ventral anterior homeobox 1	CCDS7597.1	chr10_118891976-118891976_C_T	148E>K	Substitution	Nonsynonymous coding	19%
MM12T	VAX2	ventral anterior homeobox 2	CCDS1911.1	chr2_71159963-71159963_C_T	168R>W	Substitution	Nonsynonymous coding	12%
MM12T	VCAN	versican	CCDS4060.1	chr5_82816074-82816074_G_A	650R>H	Substitution	Nonsynonymous coding	36%
MM12T	VCL	vinculin	CCDS7341.1	chr10_75864880-75864880_G_A	735D>N	Substitution	Nonsynonymous coding	13%
MM12T	VCP	valosin containing protein	CCDS6573.1	chr9_35059665-35059665_C_T	610G>D	Substitution	Nonsynonymous coding	14%
MM12T	VIL1	villin 1	CCDS2417.1	chr2_219301879-219301879_G_T	668E>D	Substitution	Nonsynonymous coding	20%
MM12T	VILL	villin-like	CCDS2670.2	chr3_38040860-38040860_G_A	371G>D	Substitution	Nonsynonymous coding	30%
MM12T	VIM	vimentin	CCDS7120.1	chr10_17271528-17271528_G_A	36R>H	Substitution	Nonsynonymous coding	14%
MM12T	VIM	vimentin	CCDS7120.1	chr10_17275887-17275887_C_A	280A>D	Substitution	Nonsynonymous coding	38%
MM12T	VMAC	vimentin-type intermediate filament associated coiled-coil protein	CCDS32881.1	chr19_5904953-5904953_G_T	18A>S	Substitution	Nonsynonymous coding	43%
MM12T	VNN3	vanin 3	CCDS5160.1	chr6_133052667-133052667_C_A	ISV-1>	Substitution	Splice site acceptor	21%
MM12T	VPREB1	pre-B lymphocyte 1	CCDS13798.1	chr22_22599405-22599405_G_A	32A>T	Substitution	Nonsynonymous coding	12%
MM12T	VPS13B	vacuolar protein sorting 13 homolog B (yeast)	CCDS6280.1	chr8_100108599-100108599_A_C	117K>N	Substitution	Nonsynonymous coding	23%
MM12T	VPS13B	vacuolar protein sorting 13 homolog B (yeast)	CCDS6280.1	chr8_100673636-100673636_C_T	2013T>I	Substitution	Nonsynonymous coding	29%
MM12T	VPS13B	vacuolar protein sorting 13 homolog B (yeast)	CCDS6280.1	chr8_100729458-100729458_C_A	2197L>I	Substitution	Nonsynonymous coding	38%
MM12T	VPS13B	vacuolar protein sorting 13 homolog B (yeast)	CCDS6280.1	chr8_100729573-100729573_C_T	2235S>F	Substitution	Nonsynonymous coding	13%
MM12T	VPS13C	vacuolar protein sorting 13 homolog C (S. cerevisiae)	CCDS32257.1	chr15_62253984-62253984_G_A	1238R>C	Substitution	Nonsynonymous coding	14%
MM12T	VPS13D	vacuolar protein sorting 13 homolog D (S. cerevisiae)	CCDS30588.1	chr1_12520387-12520387_G_A	4200A>T	Substitution	Nonsynonymous coding	24%
MM12T	VPS35	vacuolar protein sorting 35 homolog (S. cerevisiae)	CCDS10721.1	chr16_46715289-46715289_A_C	108L>R	Substitution	Nonsynonymous coding	25%

MM12T	VPS35	vacuolar protein sorting 35 homolog (S. cerevisiae)	CCDS10721.1	chr16_46715288-46715288_C_A	ISV+1>	Substitution	Splice site donor	27%
MM12T	VPS37A	vacuolar protein sorting 37 homolog A (S. cerevisiae)	CCDS6001.1	chr8_17137612-17137612_A_T	263K>N	Substitution	Nonsynonymous coding	12%
MM12T	VPS39	vacuolar protein sorting 39 homolog (S. cerevisiae)	CCDS10083.1	chr15_42458416-42458416_G_A	541H>Y	Substitution	Nonsynonymous coding	18%
MM12T	VPS41	vacuolar protein sorting 41 homolog (S. cerevisiae)	CCDS5457.1	chr7_38812198-38812198_G_A	351P>L	Substitution	Nonsynonymous coding	42%
MM12T	VPS51	vacuolar protein sorting 51 homolog (S. cerevisiae)	CCDS8093.1	chr11_64875352-64875352_G_A	195A>T	Substitution	Nonsynonymous coding	20%
MM12T	VPS51	vacuolar protein sorting 51 homolog (S. cerevisiae)	CCDS8093.1	chr11_64879052-64879052_G_A	781R>H	Substitution	Nonsynonymous coding	37%
MM12T	VPS53	vacuolar protein sorting 53 homolog (S. cerevisiae)	CCDS45558.1	chr17_505051-505051_T_C	401K>R	Substitution	Nonsynonymous coding	13%
MM12T	VPS53	vacuolar protein sorting 53 homolog (S. cerevisiae)	CCDS45558.1	chr17_530540-530540_T_C	283K>R	Substitution	Nonsynonymous coding	39%
MM12T	VPS53	vacuolar protein sorting 53 homolog (S. cerevisiae)	CCDS45558.1	chr17_600713-600713_T_C	77D>G	Substitution	Nonsynonymous coding	25%
MM12T	VPS72	vacuolar protein sorting 72 homolog (S. cerevisiae)	CCDS989.1	chr1_151149371-151149371_G_A	282R>W	Substitution	Nonsynonymous coding	35%
MM12T	VPS72	vacuolar protein sorting 72 homolog (S. cerevisiae)	CCDS989.1	chr1_151158300-151158300_G_A	82R>C	Substitution	Nonsynonymous coding	29%
MM12T	VPS9D1	VPS9 domain containing 1	ENST00000261625	chr16_89785621-89785621_C_A	26D>Y	Substitution	Nonsynonymous coding	12%
MM12T	VSIG1	V-set and immunoglobulin domain containing 1	CCDS14535.1	chrX_107316479-107316479_G_T	ISV-1>	Substitution	Splice site acceptor	36%
MM12T	VSIG4	V-set and immunoglobulin domain containing 4	ENST00000427538	chrX_65241945-65241945_G_A	269P>S	Substitution	Nonsynonymous coding	34%
MM12T	VSX1	visual system homeobox 1	CCDS13168.1	chr20_25062402-25062402_G_A	111P>S	Substitution	Nonsynonymous coding	23%
MM12T	VWA3A	von Willebrand factor A domain containing 3A	CCDS45441.1	chr16_22111790-22111790_G_A	135R>H	Substitution	Nonsynonymous coding	18%
MM12T	VWA3B	von Willebrand factor A domain containing 3B	CCDS42718.1	chr2_98928416-98928416_C_T	1219A>V	Substitution	Nonsynonymous coding	27%
MM12T	VWA5B2	von Willebrand factor A domain containing 5B2	NM_138345	chr3_183953061-183953061_C_T	356L>F	Substitution	Nonsynonymous coding	30%
MM12T	VWA5B2	von Willebrand factor A domain containing 5B2	NM_138345	chr3_183955183-183955183_G_A	568R>Q	Substitution	Nonsynonymous coding	33%
MM12T	VWA5B2	von Willebrand factor A domain containing 5B2	NM_138345	chr3_183956420-183956420_G_A	654R>H	Substitution	Nonsynonymous coding	14%
MM12T	VWA7	von Willebrand factor A domain containing 7	CCDS4721.2	chr6_31735243-31735243_C_T	562R>H	Substitution	Nonsynonymous coding	10%
MM12T	VWA7	von Willebrand factor A domain containing 7	CCDS4721.2	chr6_31737572-31737572_G_A	ISV-4>	Substitution	Splice site acceptor	46%
MM12T	VWA8	von Willebrand factor A domain containing 8	CCDS41881.1	chr13_42264313-42264313_C_T	1322G>E	Substitution	Nonsynonymous coding	26%
MM12T	VWC2	von Willebrand factor C domain containing 2	CCDS5508.1	chr7_49815623-49815623_C_T	198R>C	Substitution	Nonsynonymous coding	10%
MM12T	VWF	von Willebrand factor	CCDS8539.1	chr12_6094249-6094249_C_T	2313R>H	Substitution	Nonsynonymous coding	20%
MM12T	WAPAL	wings apart-like homolog (Drosophila)	CCDS7375.1	chr10_88230805-88230805_C_A	696A>S	Substitution	Nonsynonymous coding	41%
MM12T	WARS	tryptophanyl-tRNA synthetase	CCDS9960.1	chr14_100801237-100801237_C_T	464R>Q	Substitution	Nonsynonymous coding	27%
MM12T	WBSCR27	Williams Beuren syndrome chromosome region 27	CCDS5561.1	chr7_73249123-73249123_C_T	230A>T	Substitution	Nonsynonymous coding	34%
MM12T	WDFY3	WD repeat and FYVE domain containing 3	CCDS3609.1	chr4_85634384-85634384_G_A	2657A>V	Substitution	Nonsynonymous coding	38%
MM12T	WDFY3	WD repeat and FYVE domain containing 3	CCDS3609.1	chr4_85711022-85711022_G_A	1176L>F	Substitution	Nonsynonymous coding	11%
MM12T	WDFY4	WDFY family member 4	CCDS44385.1	chr10_49986761-49986761_C_T	1094T>M	Substitution	Nonsynonymous coding	31%
MM12T	WDFY4	WDFY family member 4	ENST00000416831	chr10_50031236-50031236_C_T	526P>S	Substitution	Nonsynonymous coding	45%

MM12T	WDFY4	WDFY family member 4	CCDS44385.1	chr10_49994884-49994884_T_G	1259Y>X	Substitution	Nonsense	25%
MM12T	WDR11	WD repeat domain 11	CCDS7619.1	chr10_122662563-122662563_C_T	ISV-4>	Substitution	Splice site acceptor	39%
MM12T	WDR20	WD repeat domain 20	CCDS9968.1	chr14_102675733-102675733_C_T	409P>L	Substitution	Nonsynonymous coding	30%
MM12T	WDR24	WD repeat domain 24	CCDS10420.1	chr16_735343-735343_C_T	645A>T	Substitution	Nonsynonymous coding	29%
MM12T	WDR3	WD repeat domain 3	CCDS898.1	chr1_118476060-118476060_G_A	40A>T	Substitution	Nonsynonymous coding	13%
MM12T	WDR3	WD repeat domain 3	CCDS898.1	chr1_118497237-118497237_A_G	799Y>C	Substitution	Nonsynonymous coding	28%
MM12T	WDR43	WD repeat domain 43	CCDS46251.1	chr2_29160863-29160863_G_A	ISV+3>	Substitution	Splice site donor	30%
MM12T	WDR55	WD repeat domain 55	CCDS4235.1	chr5_140049199-140049199_C_A	371A>D	Substitution	Nonsynonymous coding	12%
MM12T	WDR59	WD repeat domain 59	CCDS32488.1	chr16_74937943-74937943_G_A	590R>C	Substitution	Nonsynonymous coding	30%
MM12T	WDR59	WD repeat domain 59	CCDS32488.1	chr16_74972152-74972152_C_A	183A>S	Substitution	Nonsynonymous coding	27%
MM12T	WDR6	WD repeat domain 6	CCDS2782.2	chr3_49049535-49049535_G_A	220A>T	Substitution	Nonsynonymous coding	19%
MM12T	WDR66	WD repeat domain 66	CCDS41853.1	chr12_122441654-122441654_G_A	1145G>D	Substitution	Nonsynonymous coding	14%
MM12T	WDR69	WD repeat domain 69	CCDS2470.1	chr2_228786245-228786245_C_T	394A>V	Substitution	Nonsynonymous coding	39%
MM12T	WDR72	WD repeat domain 72	CCDS10151.1	chr15_53998211-53998211_C_T	339V>I	Substitution	Nonsynonymous coding	10%
MM12T	WDR74	WD repeat domain 74	ENST00000453708	chr11_62603127-62603127_T_C	186R>G	Substitution	Nonsynonymous coding	34%
MM12T	WDR81	WD repeat domain 81	NM_001163809	chr17_1629452-1629452_G_A	400R>H	Substitution	Nonsynonymous coding	12%
MM12T	WDR83	WD repeat domain 83	CCDS12275.1	chr19_12784068-12784068_C_T	246R>C	Substitution	Nonsynonymous coding	24%
MM12T	WDR86	WD repeat domain 86	CCDS5925.2	chr7_151078732-151078732_C_T	356R>H	Substitution	Nonsynonymous coding	19%
MM12T	WDR86	WD repeat domain 86	CCDS5925.2	chr7_151092903-151092903_G_A	229R>W	Substitution	Nonsynonymous coding	35%
MM12T	WDR90	WD repeat domain 90	CCDS42092.1	chr16_710091-710091_G_A	1073R>H	Substitution	Nonsynonymous coding	12%
MM12T	WDR96	WD repeat domain 96	CCDS31281.1	chr10_105924013-105924013_C_T	1029A>T	Substitution	Nonsynonymous coding	33%
MM12T	WDR96	WD repeat domain 96	CCDS31281.1	chr10_105928513-105928513_G_A	894R>X	Substitution	Nonsense	11%
MM12T	WEE2	WEE1 homolog 2 (S. pombe)	CCDS43660.1	chr7_141424004-141424004_G_A	384A>T	Substitution	Nonsynonymous coding	13%
MM12T	WFIKKN1	WAP, follistatin/kazal, immunoglobulin, kunitz and netrin domain containing 1	CCDS10414.1	chr16_683672-683672_G_A	421R>H	Substitution	Nonsynonymous coding	32%
MM12T	WFIKKN2	WAP, follistatin/kazal, immunoglobulin, kunitz and netrin domain containing 2	CCDS11575.1	chr17_48918256-48918256_G_A	536G>D	Substitution	Nonsynonymous coding	16%
MM12T	WFS1	Wolfram syndrome 1 (wolframin)	CCDS3386.1	chr4_6290744-6290744_G_A	116D>N	Substitution	Nonsynonymous coding	10%
MM12T	WFS1	Wolfram syndrome 1 (wolframin)	CCDS3386.1	chr4_6304124-6304124_C_T	868R>C	Substitution	Nonsynonymous coding	32%
MM12T	WHSC1	Wolf-Hirschhorn syndrome candidate 1	CCDS33940.1	chr4_1902658-1902658_G_A	93A>T	Substitution	Nonsynonymous coding	22%
MM12T	WHSC1	Wolf-Hirschhorn syndrome candidate 1	CCDS33940.1	chr4_1957714-1957714_T_C	894W>R	Substitution	Nonsynonymous coding	45%
MM12T	WHSC1	Wolf-Hirschhorn syndrome candidate 1	CCDS33940.1	chr4_1980581-1980581_C_T	1348P>L	Substitution	Nonsynonymous coding	10%
MM12T	WIPF1	WAS/WASL interacting protein family, member 1	CCDS2260.1	chr2_175427303-175427303_G_A	495A>V	Substitution	Nonsynonymous coding	35%
MM12T	WIPI2	WD repeat domain, phosphoinositide interacting 2	CCDS5339.1	chr7_5239239-5239239_A_G	54K>R	Substitution	Nonsynonymous coding	29%

MM12T	WIZ	widely interspaced zinc finger motifs	CCDS42516.1	chr19_15536559-15536559_G_A	368R>C	Substitution	Nonsynonymous coding	10%
MM12T	WNK1	WNK lysine deficient protein kinase 1	CCDS8506.1	chr12_1017928-1017928_C_	NA	Deletion	Frameshift	29%
MM12T	WNK2	WNK lysine deficient protein kinase 2	NM_006648	chr9_96061481-96061481_C_T	2018A>V	Substitution	Nonsynonymous coding	27%
MM12T	WNK3	WNK lysine deficient protein kinase 3	CCDS14357.1	chrX_54263780-54263780_T_C	1407K>E	Substitution	Nonsynonymous coding	30%
MM12T	WNK3	WNK lysine deficient protein kinase 3	CCDS14357.1	chrX_54224999-54224999_C_A	1721G>X	Substitution	Nonsense	36%
MM12T	WNK4	WNK lysine deficient protein kinase 4	CCDS11439.1	chr17_40947694-40947694_C_T	1025P>L	Substitution	Nonsynonymous coding	36%
MM12T	WNT2B	wingless-type MMTV integration site family, member 2B	CCDS847.1	chr1_113059807-113059807_G_A	249R>H	Substitution	Nonsynonymous coding	31%
MM12T	WNT4	wingless-type MMTV integration site family, member 4	CCDS223.1	chr1_22446993-22446993_C_T	202M>I	Substitution	Nonsynonymous coding	35%
MM12T	WNT5A	wingless-type MMTV integration site family, member 5A	CCDS46850.1	chr3_55508450-55508450_C_T	200R>H	Substitution	Nonsynonymous coding	29%
MM12T	WNT5A	wingless-type MMTV integration site family, member 5A	CCDS46850.1	chr3_55508622-55508622_C_T	143A>T	Substitution	Nonsynonymous coding	13%
MM12T	WNT5A	wingless-type MMTV integration site family, member 5A	CCDS46850.1	chr3_55513543-55513543_C_T	64G>R	Substitution	Nonsynonymous coding	14%
MM12T	WNT9A	wingless-type MMTV integration site family, member 9A	CCDS31045.1	chr1_228109463-228109463_C_T	285R>H	Substitution	Nonsynonymous coding	38%
MM12T	WRN	Werner syndrome, RecQ helicase-like	CCDS6082.1	chr8_30999014-30999014_G_T	1012E>D	Substitution	Nonsynonymous coding	28%
MM12T	WWC2	WW and C2 domain containing 2	CCDS34109.2	chr4_184182656-184182656_C_A	627A>D	Substitution	Nonsynonymous coding	11%
MM12T	WWC3	WWC family member 3	CCDS14136.1	chrX_10085597-10085597_C_T	500R>W	Substitution	Nonsynonymous coding	17%
MM12T	WWC3	WWC family member 3	CCDS14136.1	chrX_10090683-10090683_C_A	552A>D	Substitution	Nonsynonymous coding	13%
MM12T	WWC3	WWC family member 3	CCDS14136.1	chrX_10085366-10085366_C_T	423R>X	Substitution	Nonsense	24%
MM12T	WWP2	WW domain containing E3 ubiquitin protein ligase 2	CCDS10885.1	chr16_69959337-69959337_C_T	395S>L	Substitution	Nonsynonymous coding	32%
MM12T	XDH	xanthine dehydrogenase	CCDS1775.1	chr2_31562359-31562359_G_A	1257S>L	Substitution	Nonsynonymous coding	16%
MM12T	XDH	xanthine dehydrogenase	CCDS1775.1	chr2_31589857-31589857_T_C	734E>G	Substitution	Nonsynonymous coding	36%
MM12T	XDH	xanthine dehydrogenase	CCDS1775.1	chr2_31610723-31610723_G_A	202T>M	Substitution	Nonsynonymous coding	23%
MM12T	XIRP1	xin actin-binding repeat containing 1	CCDS2683.1	chr3_39230597-39230597_C_T	114A>T	Substitution	Nonsynonymous coding	11%
MM12T	XIRP1	xin actin-binding repeat containing 1	CCDS2683.1	chr3_39230840-39230840_G_T	33L>M	Substitution	Nonsynonymous coding	38%
MM12T	XIRP2	xin actin-binding repeat containing 2	CCDS42769.1	chr2_168100601-168100601_C_A	900T>N	Substitution	Nonsynonymous coding	25%
MM12T	XKR3	XK, Kell blood group complex subunit-related family, member 3	CCDS42975.1	chr22_17265116-17265116_G_A	258A>V	Substitution	Nonsynonymous coding	42%
MM12T	XKR5	XK, Kell blood group complex subunit-related family, member 5	ENST00000409172	chr8_6669684-6669684_C_A	365G>W	Substitution	Nonsynonymous coding	29%
MM12T	XPNPEP2	X-prolyl aminopeptidase (aminopeptidase P) 2, membrane-bound	CCDS14613.1	chrX_128884528-128884528_C_T	241A>V	Substitution	Nonsynonymous coding	17%
MM12T	XPO4	exportin 4	CCDS41872.1	chr13_21375038-21375038_C_A	637D>Y	Substitution	Nonsynonymous coding	12%
MM12T	XPO4	exportin 4	CCDS41872.1	chr13_21442745-21442745_C_A	55K>N	Substitution	Nonsynonymous coding	16%
MM12T	XRCC2	X-ray repair complementing defective repair in Chinese hamster cells 2	CCDS5933.1	chr7_152346063-152346063_C_A	169Q>H	Substitution	Nonsynonymous coding	23%
MM12T	XXYLT1	xyloside xylosyltransferase 1	CCDS43188.1	chr3_194947462-194947462_C_T	210A>T	Substitution	Nonsynonymous coding	44%
MM12T	XYLT1	xylosyltransferase I	CCDS10569.1	chr16_17564622-17564622_G_A	11A>V	Substitution	Nonsynonymous coding	23%

MM12T	YAP1	Yes-associated protein 1	CCDS44716.1	chr11_102076651-102076651_C_A	277A>D	Substitution	Nonsynonymous coding	19%
MM12T	YARS2	tyrosyl-tRNA synthetase 2, mitochondrial	CCDS31770.1	chr12_32903729-32903729_G_A	343R>W	Substitution	Nonsynonymous coding	17%
MM12T	YBX2	Y box binding protein 2	CCDS11098.1	chr17_7195359-7195359_C_T	119V>I	Substitution	Nonsynonymous coding	24%
MM12T	YOD1	YOD1 OTU deubiquinating enzyme 1 homolog (S. cerevisiae)	CCDS31002.1	chr1_207222988-207222988_C_T	142V>I	Substitution	Nonsynonymous coding	25%
MM12T	YPEL1	yippee-like 1 (Drosophila)	CCDS13794.1	chr22_22057711-22057711_G_A	73A>V	Substitution	Nonsynonymous coding	10%
MM12T	YTHDF2	YTH domain family, member 2	CCDS41296.1	chr1_29069426-29069426_C_T	215S>F	Substitution	Nonsynonymous coding	15%
MM12T	YY1	YY1 transcription factor	CCDS9957.1	chr14_100728730-100728730_G_A	257G>R	Substitution	Nonsynonymous coding	11%
MM12T	YY2	YY2 transcription factor	CCDS14202.1	chrX_21875564-21875564_G_A	321R>H	Substitution	Nonsynonymous coding	28%
MM12T	ZBED3	zinc finger, BED-type containing 3	CCDS4036.1	chr5_76373576-76373576_G_A	43A>V	Substitution	Nonsynonymous coding	15%
MM12T	ZBED4	zinc finger, BED-type containing 4	CCDS33677.1	chr22_50279276-50279276_A_	NA	Deletion	Frameshift	12%
MM12T	ZBTB1	zinc finger and BTB domain containing 1	CCDS45126.1	chr14_64989156-64989156_G_A	312A>T	Substitution	Nonsynonymous coding	33%
MM12T	ZBTB1	zinc finger and BTB domain containing 1	CCDS45126.1	chr14_64990218-64990218_G_C	666V>L	Substitution	Nonsynonymous coding	32%
MM12T	ZBTB16	zinc finger and BTB domain containing 16	CCDS8367.1	chr11_114112982-114112982_G_A	516R>H	Substitution	Nonsynonymous coding	32%
MM12T	ZBTB17	zinc finger and BTB domain containing 17	CCDS165.1	chr1_16268898-16268898_C_T	686A>T	Substitution	Nonsynonymous coding	31%
MM12T	ZBTB17	zinc finger and BTB domain containing 17	CCDS165.1	chr1_16269201-16269201_G_A	621R>C	Substitution	Nonsynonymous coding	36%
MM12T	ZBTB20	zinc finger and BTB domain containing 20	CCDS2981.1	chr3_114058003-114058003_G_	NA	Deletion	Frameshift	24%
MM12T	ZBTB20	zinc finger and BTB domain containing 20	CCDS2981.1	chr3_114069198-114069198__T	NA	Insertion	Frameshift	24%
MM12T	ZBTB20	zinc finger and BTB domain containing 20	CCDS2981.1	chr3_114069181-114069181_G_A	509L>F	Substitution	Nonsynonymous coding	34%
MM12T	ZBTB3	zinc finger and BTB domain containing 3	CCDS8034.1	chr11_62520686-62520686_G_A	201R>C	Substitution	Nonsynonymous coding	29%
MM12T	ZBTB32	zinc finger and BTB domain containing 32	CCDS12471.1	chr19_36205815-36205815_C_T	96A>V	Substitution	Nonsynonymous coding	26%
MM12T	ZBTB33	zinc finger and BTB domain containing 33	CCDS14596.1	chrX_119387313-119387313_T_C	15S>P	Substitution	Nonsynonymous coding	31%
MM12T	ZBTB38	zinc finger and BTB domain containing 38	CCDS43157.1	chr3_141163434-141163434_C_T	735A>V	Substitution	Nonsynonymous coding	13%
MM12T	ZBTB44	zinc finger and BTB domain containing 44	CCDS44776.1	chr11_130131584-130131584_C_T	62G>D	Substitution	Nonsynonymous coding	23%
MM12T	ZBTB7A	zinc finger and BTB domain containing 7A	CCDS12119.1	chr19_4047913-4047913_C_T	531R>H	Substitution	Nonsynonymous coding	31%
MM12T	ZBTB8A	zinc finger and BTB domain containing 8A	CCDS30664.1	chr1_33058626-33058626_G_T	32G>W	Substitution	Nonsynonymous coding	21%
MM12T	ZBTB8B	zinc finger and BTB domain containing 8B	CCDS44104.1	chr1_32937025-32937025_C_T	267A>V	Substitution	Nonsynonymous coding	24%
MM12T	ZC3H13	zinc finger CCCH-type containing 13	CCDS9400.1	chr13_46559465-46559465_T_C	563S>G	Substitution	Nonsynonymous coding	11%
MM12T	ZC3H4	zinc finger CCCH-type containing 4	CCDS42582.1	chr19_47571000-47571000_C_T	842S>N	Substitution	Nonsynonymous coding	27%
MM12T	ZC3H6	zinc finger CCCH-type containing 6	CCDS46393.1	chr2_113089907-113089907_A_G	1138T>A	Substitution	Nonsynonymous coding	37%
MM12T	ZC3H7A	zinc finger CCCH-type containing 7A	CCDS10550.1	chr16_11857466-11857466_G_A	624R>C	Substitution	Nonsynonymous coding	33%
MM12T	ZC3HAV1	zinc finger CCCH-type, antiviral 1	CCDS5851.1	chr7_138764711-138764711_T_G	326T>P	Substitution	Nonsynonymous coding	35%
MM12T	ZCCHC14	zinc finger, CCHC domain containing 14	CCDS10961.1	chr16_87446661-87446661_G_A	445R>C	Substitution	Nonsynonymous coding	30%

MM12T	ZCCHC5	zinc finger, CCHC domain containing 5	CCDS14440.1	chrX_77913136-77913136_T_C	261Y>C	Substitution	Nonsynonymous coding	12%
MM12T	ZCCHC8	zinc finger, CCHC domain containing 8	NM_017612	chr12_122958288-122958288_C_T	627G>D	Substitution	Nonsynonymous coding	12%
MM12T	ZDBF2	zinc finger, DBF-type containing 2	CCDS46501.1	chr2_207171048-207171048_C_T	599T>I	Substitution	Nonsynonymous coding	41%
MM12T	ZDBF2	zinc finger, DBF-type containing 2	CCDS46501.1	chr2_207174069-207174069_G_A	1606R>Q	Substitution	Nonsynonymous coding	13%
MM12T	ZDBF2	zinc finger, DBF-type containing 2	CCDS46501.1	chr2_207174748-207174748_G_T	1832W>C	Substitution	Nonsynonymous coding	15%
MM12T	ZDHHC20	zinc finger, DHHC-type containing 20	CCDS45017.1	chr13_22033193-22033193_A_G	40F>L	Substitution	Nonsynonymous coding	27%
MM12T	ZEB2	zinc finger E-box binding homeobox 2	CCDS2186.1	chr2_145187573-145187573_C_T	32V>M	Substitution	Nonsynonymous coding	26%
MM12T	ZFAT	zinc finger and AT hook domain containing	CCDS47924.1	chr8_135614731-135614731_G_A	411R>C	Substitution	Nonsynonymous coding	32%
MM12T	ZFC3H1	zinc finger, C3H1-type containing	CCDS41813.1	chr12_72024433-72024433_G_T	1224S>Y	Substitution	Nonsynonymous coding	23%
MM12T	ZFC3H1	zinc finger, C3H1-type containing	CCDS41813.1	chr12_72038712-72038712_C_A	408Q>H	Substitution	Nonsynonymous coding	21%
MM12T	ZFC3H1	zinc finger, C3H1-type containing	CCDS41813.1	chr12_72038713-72038713_T_A	408Q>L	Substitution	Nonsynonymous coding	20%
MM12T	ZFC3H1	zinc finger, C3H1-type containing	CCDS41813.1	chr12_72038714-72038714_G_T	408Q>K	Substitution	Nonsynonymous coding	20%
MM12T	ZFHX3	zinc finger homeobox 3	CCDS10908.1	chr16_72827372-72827372_G_A	3070A>V	Substitution	Nonsynonymous coding	33%
MM12T	ZFHX3	zinc finger homeobox 3	CCDS10908.1	chr16_72993288-72993288_T_G	253S>R	Substitution	Nonsynonymous coding	36%
MM12T	ZFHX3	zinc finger homeobox 3	CCDS10908.1	chr16_72993925-72993925_C_A	40E>D	Substitution	Nonsynonymous coding	12%
MM12T	ZFHX4	zinc finger homeobox 4	CCDS47878.1	chr8_77619946-77619946_C_T	893A>V	Substitution	Nonsynonymous coding	12%
MM12T	ZFHX4	zinc finger homeobox 4	CCDS47878.1	chr8_77762550-77762550_C_T	1261R>W	Substitution	Nonsynonymous coding	12%
MM12T	ZFHX4	zinc finger homeobox 4	CCDS47878.1	chr8_77766705-77766705_C_A	2471F>L	Substitution	Nonsynonymous coding	38%
MM12T	ZFHX4	zinc finger homeobox 4	CCDS47878.1	chr8_77768414-77768414_G_A	3041S>N	Substitution	Nonsynonymous coding	31%
MM12T	ZFHX4	zinc finger homeobox 4	CCDS47878.1	chr8_77776779-77776779_G_A	3565R>Q	Substitution	Nonsynonymous coding	42%
MM12T	ZFP28	ZFP28 zinc finger protein	CCDS12946.1	chr19_57065632-57065632_G_A	493R>H	Substitution	Nonsynonymous coding	32%
MM12T	ZFP64	ZFP64 zinc finger protein	CCDS13440.1	chr20_50781299-50781299__A	NA	Insertion	Splice site acceptor	37%
MM12T	ZFP82	ZFP82 zinc finger protein	CCDS12493.1	chr19_36884085-36884085_C_A	386R>I	Substitution	Nonsynonymous coding	21%
MM12T	ZFP82	ZFP82 zinc finger protein	CCDS12493.1	chr19_36884291-36884291_C_A	317K>N	Substitution	Nonsynonymous coding	37%
MM12T	ZFPM1	zinc finger protein, FOG family member 1	CCDS32502.1	chr16_88552448-88552448_G_A	48A>T	Substitution	Nonsynonymous coding	41%
MM12T	ZFPM2	zinc finger protein, FOG family member 2	CCDS47908.1	chr8_106801017-106801017_T_G	202F>V	Substitution	Nonsynonymous coding	30%
MM12T	ZFPM2	zinc finger protein, FOG family member 2	CCDS47908.1	chr8_106815470-106815470_C_T	1054P>S	Substitution	Nonsynonymous coding	12%
MM12T	ZFR2	zinc finger RNA binding protein 2	CCDS45921.1	chr19_3831531-3831531_C_T	208A>T	Substitution	Nonsynonymous coding	25%
MM12T	ZFYVE1	zinc finger, FYVE domain containing 1	CCDS9811.1	chr14_73490873-73490873_C_A	115R>I	Substitution	Nonsynonymous coding	17%
MM12T	ZFYVE26	zinc finger, FYVE domain containing 26	CCDS9788.1	chr14_68233074-68233074_G_A	1961L>F	Substitution	Nonsynonymous coding	32%
MM12T	ZFYVE27	zinc finger, FYVE domain containing 27	CCDS31262.1	chr10_99512845-99512845_G_T	321Q>H	Substitution	Nonsynonymous coding	32%
MM12T	ZFYVE9	zinc finger, FYVE domain containing 9	CCDS563.1	chr1_52704262-52704262_G_T	391E>D	Substitution	Nonsynonymous coding	35%

MM12T	ZGLP1	zinc finger, GATA-like protein 1	CCDS45959.1	chr19_10419192-10419192_C_T	56A>T	Substitution	Nonsynonymous coding	26%
MM12T	ZIC1	Zic family member 1	CCDS3136.1	chr3_147128293-147128293_C_T	132H>Y	Substitution	Nonsynonymous coding	44%
MM12T	ZIC3	Zic family member 3	CCDS14663.1	chrX_136649495-136649495_G_T	215Q>H	Substitution	Nonsynonymous coding	29%
MM12T	ZIC3	Zic family member 3	CCDS14663.1	chrX_136649559-136649559_G_A	237G>S	Substitution	Nonsynonymous coding	33%
MM12T	ZIC3	Zic family member 3	CCDS14663.1	chrX_136649580-136649580_C_T	244R>W	Substitution	Nonsynonymous coding	35%
MM12T	ZMAT5	zinc finger, matrin-type 5	CCDS13868.1	chr22_30136711-30136711_C_T	67D>N	Substitution	Nonsynonymous coding	36%
MM12T	ZMYM3	zinc finger, MYM-type 3	CCDS14409.1	chrX_70470303-70470303_T_	NA	Deletion	Frameshift	29%
MM12T	ZMYM3	zinc finger, MYM-type 3	CCDS14409.1	chrX_70461141-70461141_G_A	1286R>C	Substitution	Nonsynonymous coding	14%
MM12T	ZMYM3	zinc finger, MYM-type 3	CCDS14409.1	chrX_70462110-70462110_G_A	1238R>W	Substitution	Nonsynonymous coding	39%
MM12T	ZMYM3	zinc finger, MYM-type 3	CCDS14409.1	chrX_70466442-70466442_G_A	ISV+3>	Substitution	Splice site donor	28%
MM12T	ZMYM6	zinc finger, MYM-type 6	CCDS387.2	chr1_35457837-35457837_G_A	715T>I	Substitution	Nonsynonymous coding	35%
MM12T	ZNF101	zinc finger protein 101	CCDS32971.1	chr19_19789582-19789582_G_A	60G>R	Substitution	Nonsynonymous coding	24%
MM12T	ZNF106	zinc finger protein 106	CCDS32208.1	chr15_42742022-42742022_C_A	793Q>H	Substitution	Nonsynonymous coding	31%
MM12T	ZNF124	zinc finger protein 124	CCDS31089.1	chr1_247319871-247319871_C_T	289M>I	Substitution	Nonsynonymous coding	21%
MM12T	ZNF124	zinc finger protein 124	ENST00000366499	chr1_247320641-247320641_T_A	56T>S	Substitution	Nonsynonymous coding	17%
MM12T	ZNF131	zinc finger protein 131	CCDS43313.1	chr5_43161532-43161532_G_A	185E>K	Substitution	Nonsynonymous coding	15%
MM12T	ZNF135	zinc finger protein 135	CCDS12970.1	chr19_58578434-58578434_A_	NA	Deletion	Frameshift	25%
MM12T	ZNF142	zinc finger protein 142	CCDS42817.1	chr2_219507509-219507509_G_A	1244R>C	Substitution	Nonsynonymous coding	13%
MM12T	ZNF16	zinc finger protein 16	CCDS6437.1	chr8_146157749-146157749_C_T	142G>R	Substitution	Nonsynonymous coding	37%
MM12T	ZNF17	zinc finger protein 17	CCDS42636.1	chr19_57929276-57929276_G_A	ISV-4>	Substitution	Splice site acceptor	35%
MM12T	ZNF18	zinc finger protein 18	CCDS32568.1	chr17_11895766-11895766_C_T	127W>X	Substitution	Nonsense	16%
MM12T	ZNF180	zinc finger protein 180	CCDS12639.1	chr19_44980761-44980761_G_A	646A>V	Substitution	Nonsynonymous coding	14%
MM12T	ZNF197	zinc finger protein 197	CCDS2717.1	chr3_44684045-44684045_C_T	475R>C	Substitution	Nonsynonymous coding	26%
MM12T	ZNF2	zinc finger protein 2	NM_021088	chr2_95847297-95847297_C_T	242R>C	Substitution	Nonsynonymous coding	28%
MM12T	ZNF211	zinc finger protein 211	CCDS12956.1	chr19_58152510-58152510_C_T	232A>V	Substitution	Nonsynonymous coding	44%
MM12T	ZNF214	zinc finger protein 214	CCDS31418.1	chr11_7021892-7021892_C_A	341R>I	Substitution	Nonsynonymous coding	14%
MM12T	ZNF219	zinc finger protein 219	CCDS9568.1	chr14_21560303-21560303_G_A	385R>X	Substitution	Nonsense	12%
MM12T	ZNF235	zinc finger protein 235	CCDS33048.1	chr19_44792330-44792330_C_T	420A>T	Substitution	Nonsynonymous coding	13%
MM12T	ZNF236	zinc finger protein 236	CCDS42447.1	chr18_74622087-74622087_C_A	870P>H	Substitution	Nonsynonymous coding	31%
MM12T	ZNF264	zinc finger protein 264	CCDS33127.1	chr19_57723880-57723880_G_A	472R>H	Substitution	Nonsynonymous coding	28%
MM12T	ZNF267	zinc finger protein 267	CCDS32440.1	chr16_31926606-31926606_G_A	346E>K	Substitution	Nonsynonymous coding	21%
MM12T	ZNF276	zinc finger protein 276	CCDS45554.1	chr16_89800423-89800423_G_A	484R>H	Substitution	Nonsynonymous coding	32%

MM12T	ZNF277	zinc finger protein 277	CCDS5755.2	chr7_111981105-111981105_C_T	ISV+4>	Substitution	Splice site donor	11%
MM12T	ZNF280A	zinc finger protein 280A	CCDS13800.1	chr22_22868772-22868772_C_T	395V>M	Substitution	Nonsynonymous coding	42%
MM12T	ZNF280C	zinc finger protein 280C	CCDS14622.1	chrX_129370292-129370292_T_C	223T>A	Substitution	Nonsynonymous coding	25%
MM12T	ZNF280D	zinc finger protein 280D	CCDS32245.1	chr15_56923854-56923854_C_T	928E>K	Substitution	Nonsynonymous coding	25%
MM12T	ZNF280D	zinc finger protein 280D	CCDS32245.1	chr15_56974531-56974531_C_A	309G>X	Substitution	Nonsense	25%
MM12T	ZNF282	zinc finger protein 282	CCDS5895.1	chr7_148907707-148907707_C_T	288A>V	Substitution	Nonsynonymous coding	22%
MM12T	ZNF283	zinc finger protein 283	CCDS46097.1	chr19_44351661-44351661_G_A	303G>D	Substitution	Nonsynonymous coding	25%
MM12T	ZNF283	zinc finger protein 283	CCDS46097.1	chr19_44351871-44351871_A_C	373K>T	Substitution	Nonsynonymous coding	27%
MM12T	ZNF286A	zinc finger protein 286A	CCDS11172.1	chr17_15609780-15609780_T_C	69V>A	Substitution	Nonsynonymous coding	21%
MM12T	ZNF300	zinc finger protein 300	NM_052860	chr5_150276021-150276021_C_A	260Q>H	Substitution	Nonsynonymous coding	28%
MM12T	ZNF304	zinc finger protein 304	CCDS12950.1	chr19_57868910-57868910_C_A	558P>H	Substitution	Nonsynonymous coding	34%
MM12T	ZNF317	zinc finger protein 317	CCDS12210.1	chr19_9271409-9271409_C_T	363A>V	Substitution	Nonsynonymous coding	12%
MM12T	ZNF318	zinc finger protein 318	CCDS4895.2	chr6_43322625-43322625_G_A	816P>L	Substitution	Nonsynonymous coding	48%
MM12T	ZNF324	zinc finger protein 324	CCDS12981.1	chr19_58982124-58982124_G_T	89D>Y	Substitution	Nonsynonymous coding	35%
MM12T	ZNF324	zinc finger protein 324	CCDS12981.1	chr19_58982773-58982773_G_A	305R>H	Substitution	Nonsynonymous coding	11%
MM12T	ZNF337	zinc finger protein 337	CCDS13174.1	chr20_25656637-25656637_C_A	429E>D	Substitution	Nonsynonymous coding	14%
MM12T	ZNF33A	zinc finger protein 33A	CCDS44372.1	chr10_38344125-38344125_A_C	358Q>P	Substitution	Nonsynonymous coding	23%
MM12T	ZNF341	zinc finger protein 341	CCDS13227.1	chr20_32328780-32328780_G_A	35G>D	Substitution	Nonsynonymous coding	28%
MM12T	ZNF35	zinc finger protein 35	CCDS2718.2	chr3_44701042-44701042_G_A	396G>E	Substitution	Nonsynonymous coding	29%
MM12T	ZNF358	zinc finger protein 358	CCDS32890.2	chr19_7585227-7585227_C_T	367H>Y	Substitution	Nonsynonymous coding	31%
MM12T	ZNF365	zinc finger protein 365	CCDS7264.1	chr10_64136124-64136124_C_A	58L>I	Substitution	Nonsynonymous coding	13%
MM12T	ZNF385B	zinc finger protein 385B	CCDS33339.1	chr2_180383346-180383346_G_A	139A>V	Substitution	Nonsynonymous coding	30%
MM12T	ZNF398	zinc finger protein 398	CCDS5894.1	chr7_148876640-148876640_G_A	559R>H	Substitution	Nonsynonymous coding	12%
MM12T	ZNF404	zinc finger protein 404	NM_001033719	chr19_44378166-44378166_G_A	64A>V	Substitution	Nonsynonymous coding	13%
MM12T	ZNF414	zinc finger protein 414	CCDS12205.1	chr19_8576525-8576525_C_T	284A>T	Substitution	Nonsynonymous coding	28%
MM12T	ZNF418	zinc finger protein 418	CCDS42642.1	chr19_58438881-58438881_C_A	223R>I	Substitution	Nonsynonymous coding	29%
MM12T	ZNF420	zinc finger protein 420	CCDS12498.1	chr19_37618319-37618319_G_T	142K>N	Substitution	Nonsynonymous coding	13%
MM12T	ZNF423	zinc finger protein 423	CCDS32445.1	chr16_49559361-49559361_C_T	1208D>N	Substitution	Nonsynonymous coding	16%
MM12T	ZNF423	zinc finger protein 423	CCDS32445.1	chr16_49669864-49669864_C_T	1067A>T	Substitution	Nonsynonymous coding	31%
MM12T	ZNF423	zinc finger protein 423	CCDS32445.1	chr16_49670344-49670344_G_A	907R>W	Substitution	Nonsynonymous coding	31%
MM12T	ZNF423	zinc finger protein 423	CCDS32445.1	chr16_49671985-49671985_C_T	360V>I	Substitution	Nonsynonymous coding	11%
MM12T	ZNF425	zinc finger protein 425	CCDS34773.1	chr7_148801188-148801188_G_A	592T>M	Substitution	Nonsynonymous coding	32%

MM12T	ZNF426	zinc finger protein 426	CCDS12215.1	chr19_9640313-9640313_C_A	ISV-1>	Substitution	Splice site acceptor	34%
MM12T	ZNF426	zinc finger protein 426	CCDS12215.1	chr19_9645956-9645956_C_A	ISV-1>	Substitution	Splice site acceptor	13%
MM12T	ZNF44	zinc finger protein 44	CCDS45988.1	chr19_12384679-12384679_G_A	131R>W	Substitution	Nonsynonymous coding	29%
MM12T	ZNF441	zinc finger protein 441	CCDS12266.2	chr19_11890970-11890970_G_A	111V>I	Substitution	Nonsynonymous coding	18%
MM12T	ZNF446	zinc finger protein 446	CCDS12982.1	chr19_58988674-58988674_G_A	30R>Q	Substitution	Nonsynonymous coding	35%
MM12T	ZNF462	zinc finger protein 462	CCDS35096.1	chr9_109701319-109701319_C_T	2120L>F	Substitution	Nonsynonymous coding	31%
MM12T	ZNF467	zinc finger protein 467	CCDS5899.1	chr7_149463011-149463011_C_T	194G>S	Substitution	Nonsynonymous coding	38%
MM12T	ZNF474	zinc finger protein 474	CCDS4130.1	chr5_121488388-121488388_T_C	235S>P	Substitution	Nonsynonymous coding	28%
MM12T	ZNF483	zinc finger protein 483	CCDS35106.1	chr9_114304071-114304071_G_A	286A>T	Substitution	Nonsynonymous coding	10%
MM12T	ZNF483	zinc finger protein 483	CCDS35106.1	chr9_114304842-114304842_C_T	543R>X	Substitution	Nonsense	37%
MM12T	ZNF484	zinc finger protein 484	CCDS35066.1	chr9_95609920-95609920_G_T	383H>Q	Substitution	Nonsynonymous coding	17%
MM12T	ZNF487P	zinc finger protein 487, pseudogene	ENST00000431662	chr10_43971561-43971561_G_A	79C>Y	Substitution	Nonsynonymous coding	29%
MM12T	ZNF497	zinc finger protein 497	CCDS12977.1	chr19_58867893-58867893_C_T	370R>H	Substitution	Nonsynonymous coding	24%
MM12T	ZNF497	zinc finger protein 497	CCDS12977.1	chr19_58868037-58868037_C_T	322R>H	Substitution	Nonsynonymous coding	21%
MM12T	ZNF497	zinc finger protein 497	CCDS12977.1	chr19_58868347-58868347_C_T	219E>K	Substitution	Nonsynonymous coding	12%
MM12T	ZNF497	zinc finger protein 497	CCDS12977.1	chr19_58868373-58868373_C_T	210R>H	Substitution	Nonsynonymous coding	11%
MM12T	ZNF503	zinc finger protein 503	CCDS7350.1	chr10_77158926-77158926_G_A	508P>S	Substitution	Nonsynonymous coding	11%
MM12T	ZNF503-AS2	ZNF503 antisense RNA 2	ENST00000425916	chr10_77167197-77167197_A_G	94S>G	Substitution	Nonsynonymous coding	43%
MM12T	ZNF507	zinc finger protein 507	CCDS32985.1	chr19_32844473-32844473_G_A	246G>D	Substitution	Nonsynonymous coding	11%
MM12T	ZNF512B	zinc finger protein 512B	CCDS13548.1	chr20_62595691-62595691_C_A	439G>W	Substitution	Nonsynonymous coding	30%
MM12T	ZNF513	zinc finger protein 513	CCDS1751.1	chr2_27600731-27600731_C_T	436R>H	Substitution	Nonsynonymous coding	13%
MM12T	ZNF516	zinc finger protein 516	ENST00000443185	chr18_74091039-74091039_T_C	1011R>G	Substitution	Nonsynonymous coding	36%
MM12T	ZNF518A	zinc finger protein 518A	ENST00000371192	chr10_97919602-97919602_C_T	1175P>S	Substitution	Nonsynonymous coding	15%
MM12T	ZNF530	zinc finger protein 530	CCDS12955.1	chr19_58118445-58118445_C_T	518P>S	Substitution	Nonsynonymous coding	20%
MM12T	ZNF541	zinc finger protein 541	CCDS46133.1	chr19_48036208-48036208_C_T	1058G>D	Substitution	Nonsynonymous coding	13%
MM12T	ZNF541	zinc finger protein 541	CCDS46133.1	chr19_48059100-48059100_C_T	5S>N	Substitution	Nonsynonymous coding	39%
MM12T	ZNF547	zinc finger protein 547	CCDS33131.1	chr19_57883174-57883174_G_A	17A>T	Substitution	Nonsynonymous coding	29%
MM12T	ZNF550	zinc finger protein 550	CCDS35500.1	chr19_58058566-58058566_C_T	308G>E	Substitution	Nonsynonymous coding	31%
MM12T	ZNF551	zinc finger protein 551	CCDS12959.1	chr19_58199257-58199257_A_C	522K>N	Substitution	Nonsynonymous coding	24%
MM12T	ZNF557	zinc finger protein 557	CCDS42485.1	chr19_7082968-7082968_G_A	169R>Q	Substitution	Nonsynonymous coding	22%
MM12T	ZNF560	zinc finger protein 560	CCDS12214.1	chr19_9577275-9577275_C_T	783R>H	Substitution	Nonsynonymous coding	17%
MM12T	ZNF566	zinc finger protein 566	CCDS46061.1	chr19_36940252-36940252_C_T	296G>D	Substitution	Nonsynonymous coding	19%

MM12T	ZNF567	zinc finger protein 567	CCDS12495.1	chr19_37203712-37203712_C_A	25L>I	Substitution	Nonsynonymous coding	28%
MM12T	ZNF57	zinc finger protein 57	CCDS12098.1	chr19_2917568-2917568_T_A	317F>I	Substitution	Nonsynonymous coding	23%
MM12T	ZNF571	zinc finger protein 571	CCDS12505.1	chr19_38056003-38056003_C_A	443G>C	Substitution	Nonsynonymous coding	15%
MM12T	ZNF577	zinc finger protein 577	CCDS12842.2	chr19_52375811-52375811_A_G	478Y>H	Substitution	Nonsynonymous coding	41%
MM12T	ZNF587B	zinc finger protein 587B	ENST00000442832	chr19_58352985-58352985_C_T	315R>C	Substitution	Nonsynonymous coding	12%
MM12T	ZNF592	zinc finger protein 592	CCDS32317.1	chr15_85333980-85333980_C_A	755F>L	Substitution	Nonsynonymous coding	11%
MM12T	ZNF592	zinc finger protein 592	CCDS32317.1	chr15_85341125-85341125_C_A	809L>M	Substitution	Nonsynonymous coding	34%
MM12T	ZNF592	zinc finger protein 592	CCDS32317.1	chr15_85343144-85343144_G_A	1070G>D	Substitution	Nonsynonymous coding	39%
MM12T	ZNF596	zinc finger protein 596	CCDS5951.2	chr8_195827-195827_G_A	327R>K	Substitution	Nonsynonymous coding	26%
MM12T	ZNF598	zinc finger protein 598	ENST00000431526	chr16_2052674-2052674_C_A	175Q>H	Substitution	Nonsynonymous coding	34%
MM12T	ZNF609	zinc finger protein 609	CCDS32270.1	chr15_64967247-64967247_A_	NA	Deletion	Frameshift	22%
MM12T	ZNF609	zinc finger protein 609	CCDS32270.1	chr15_64967551-64967551_C_A	833A>D	Substitution	Nonsynonymous coding	34%
MM12T	ZNF609	zinc finger protein 609	CCDS32270.1	chr15_64970645-64970645_C_T	1245R>W	Substitution	Nonsynonymous coding	37%
MM12T	ZNF614	zinc finger protein 614	CCDS12847.1	chr19_52519269-52519269_C_T	528V>I	Substitution	Nonsynonymous coding	26%
MM12T	ZNF615	zinc finger protein 615	CCDS12846.1	chr19_52496498-52496498_G_A	611L>F	Substitution	Nonsynonymous coding	24%
MM12T	ZNF618	zinc finger protein 618	CCDS48008.1	chr9_116750605-116750605_C_T	28R>C	Substitution	Nonsynonymous coding	33%
MM12T	ZNF618	zinc finger protein 618	CCDS48008.1	chr9_116811788-116811788_G_A	643A>T	Substitution	Nonsynonymous coding	10%
MM12T	ZNF638	zinc finger protein 638	CCDS1917.1	chr2_71645753-71645753_G_A	1095E>K	Substitution	Nonsynonymous coding	18%
MM12T	ZNF638	zinc finger protein 638	CCDS1917.1	chr2_71650903-71650903_C_T	1420S>F	Substitution	Nonsynonymous coding	13%
MM12T	ZNF641	zinc finger protein 641	ENST00000448928	chr12_48741009-48741009_G_A	101S>F	Substitution	Nonsynonymous coding	15%
MM12T	ZNF645	zinc finger protein 645	CCDS14205.1	chrX_22291269-22291269_T_A	54I>N	Substitution	Nonsynonymous coding	26%
MM12T	ZNF646	zinc finger protein 646	CCDS10702.1	chr16_31089593-31089593_G_A	650A>T	Substitution	Nonsynonymous coding	18%
MM12T	ZNF646	zinc finger protein 646	CCDS10702.1	chr16_31090236-31090236_G_A	864R>H	Substitution	Nonsynonymous coding	18%
MM12T	ZNF648	zinc finger protein 648	CCDS30952.1	chr1_182026427-182026427_C_T	240R>H	Substitution	Nonsynonymous coding	40%
MM12T	ZNF668	zinc finger protein 668	CCDS10701.1	chr16_31073434-31073434_C_T	272R>H	Substitution	Nonsynonymous coding	16%
MM12T	ZNF668	zinc finger protein 668	CCDS10701.1	chr16_31075395-31075395_A_G	129V>A	Substitution	Nonsynonymous coding	14%
MM12T	ZNF674	zinc finger protein 674	CCDS48099.1	chrX_46360669-46360669_T_G	119S>R	Substitution	Nonsynonymous coding	33%
MM12T	ZNF680	zinc finger protein 680	CCDS34644.1	chr7_63981547-63981547_T_A	529N>Y	Substitution	Nonsynonymous coding	25%
MM12T	ZNF689	zinc finger protein 689	CCDS10686.1	chr16_30616060-30616060_C_T	343R>H	Substitution	Nonsynonymous coding	13%
MM12T	ZNF711	zinc finger protein 711	CCDS35344.1	chrX_84510624-84510624_G_T	147D>Y	Substitution	Nonsynonymous coding	27%
MM12T	ZNF74	zinc finger protein 74	CCDS42982.1	chr22_20759760-20759760_C_T	146A>V	Substitution	Nonsynonymous coding	18%
MM12T	ZNF74	zinc finger protein 74	CCDS42982.1	chr22_20759925-20759925_G_T	201R>L	Substitution	Nonsynonymous coding	13%

MM12T	ZNF749	zinc finger protein 749	CCDS33132.2	chr19_57955378-57955378_C_T	288H>Y	Substitution	Nonsynonymous coding	35%
MM12T	ZNF764	zinc finger protein 764	CCDS10683.1	chr16_30566909-30566909_C_T	278R>Q	Substitution	Nonsynonymous coding	48%
MM12T	ZNF770	zinc finger protein 770	CCDS10042.1	chr15_35273765-35273765_A_G	624L>P	Substitution	Nonsynonymous coding	26%
MM12T	ZNF770	zinc finger protein 770	CCDS10042.1	chr15_35273843-35273843_G_A	598P>L	Substitution	Nonsynonymous coding	32%
MM12T	ZNF770	zinc finger protein 770	CCDS10042.1	chr15_35274733-35274733_C_A	301K>N	Substitution	Nonsynonymous coding	24%
MM12T	ZNF771	zinc finger protein 771	CCDS45460.1	chr16_30419515-30419515_G_T	47E>D	Substitution	Nonsynonymous coding	23%
MM12T	ZNF772	zinc finger protein 772	CCDS33133.1	chr19_57985738-57985738_G_T	125S>Y	Substitution	Nonsynonymous coding	27%
MM12T	ZNF777	zinc finger protein 777	CCDS43675.1	chr7_149152453-149152453_C_T	221A>T	Substitution	Nonsynonymous coding	27%
MM12T	ZNF778	zinc finger protein 778	CCDS45550.1	chr16_89293954-89293954_C_T	392P>S	Substitution	Nonsynonymous coding	30%
MM12T	ZNF778	zinc finger protein 778	CCDS45550.1	chr16_89294281-89294281_G_A	501G>R	Substitution	Nonsynonymous coding	29%
MM12T	ZNF782	zinc finger protein 782	NM_001001662	chr9_99580954-99580954_C_T	451E>K	Substitution	Nonsynonymous coding	28%
MM12T	ZNF784	zinc finger protein 784	CCDS12930.1	chr19_56133152-56133152_C_	NA	Deletion	Frameshift	31%
MM12T	ZNF784	zinc finger protein 784	CCDS12930.1	chr19_56133973-56133973_G_A	39P>L	Substitution	Nonsynonymous coding	32%
MM12T	ZNF805	zinc finger protein 805	CCDS46207.1	chr19_57765391-57765391_C_A	402L>I	Substitution	Nonsynonymous coding	27%
MM12T	ZNF814	zinc finger protein 814	CCDS46212.1	chr19_58385188-58385188_C_A	524E>X	Substitution	Nonsense	25%
MM12T	ZNF829	zinc finger protein 829	NM_001037232	chr19_37383070-37383070_C_T	208G>D	Substitution	Nonsynonymous coding	14%
MM12T	ZNF835	zinc finger protein 835	ENST00000342088	chr19_57175489-57175489_G_A	382R>W	Substitution	Nonsynonymous coding	38%
MM12T	ZNF837	zinc finger protein 837	CCDS46216.1	chr19_58879928-58879928_G_A	258P>S	Substitution	Nonsynonymous coding	27%
MM12T	ZNF837	zinc finger protein 837	CCDS46216.1	chr19_58880336-58880336_C_T	122G>R	Substitution	Nonsynonymous coding	16%
MM12T	ZNF839	zinc finger protein 839	CCDS45164.1	chr14_102807753-102807753_C_T	674A>V	Substitution	Nonsynonymous coding	11%
MM12T	ZNF860	zinc finger protein 860	CCDS46784.1	chr3_32030787-32030787_G_T	72K>N	Substitution	Nonsynonymous coding	10%
MM12T	ZNF862	zinc finger protein 862	CCDS47741.1	chr7_149556556-149556556_G_T	388R>M	Substitution	Nonsynonymous coding	30%
MM12T	ZNFX1	zinc finger, NFX1-type containing 1	ENST00000371754	chr20_47855693-47855693_C_T	1191A>T	Substitution	Nonsynonymous coding	38%
MM12T	ZNRF3	zinc and ring finger 3	CCDS42999.1	chr22_29445509-29445509_G_A	347R>H	Substitution	Nonsynonymous coding	25%
MM12T	ZNRF4	zinc and ring finger 4	CCDS42475.1	chr19_5455987-5455987_G_A	162R>H	Substitution	Nonsynonymous coding	40%
MM12T	ZNRF4	zinc and ring finger 4	CCDS42475.1	chr19_5456047-5456047_C_T	182A>V	Substitution	Nonsynonymous coding	37%
MM12T	ZP4	zona pellucida glycoprotein 4	CCDS1615.1	chr1_238048809-238048809_C_T	348V>M	Substitution	Nonsynonymous coding	34%
MM12T	ZP4	zona pellucida glycoprotein 4	CCDS1615.1	chr1_238050080-238050080_C_T	277S>N	Substitution	Nonsynonymous coding	14%
MM12T	ZPBP2	zona pellucida binding protein 2	CCDS11352.1	chr17_38031658-38031661_GTCT_	NA	Deletion	Frameshift	14%
MM12T	ZPLD1	zona pellucida-like domain containing 1	CCDS2947.1	chr3_102176660-102176660_G_A	198G>D	Substitution	Nonsynonymous coding	28%
MM12T	ZSCAN22	zinc finger and SCAN domain containing 22	CCDS12975.1	chr19_58849890-58849890_C_A	225A>D	Substitution	Nonsynonymous coding	12%
MM12T	ZSCAN25	zinc finger and SCAN domain containing 25	CCDS5671.2	chr7_99217330-99217330_A_G	34E>G	Substitution	Nonsynonymous coding	31%

MM12T	ZSCAN32	zinc finger and SCAN domain containing 32	CCDS10503.1	chr16_3434723-3434723_G_A	112R>C	Substitution	Nonsynonymous coding	27%
MM12T	ZSWIM2	zinc finger, SWIM-type containing 2	CCDS33348.1	chr2_187693018-187693018_C_T	532C>Y	Substitution	Nonsynonymous coding	12%
MM12T	ZSWIM4	zinc finger, SWIM-type containing 4	CCDS32924.1	chr19_13930227-13930227_A_G	544T>A	Substitution	Nonsynonymous coding	11%
MM12T	ZSWIM5	zinc finger, SWIM-type containing 5	CCDS41319.1	chr1_45525828-45525828__A	NA	Insertion	Frameshift	27%
MM12T	ZSWIM6	zinc finger, SWIM-type containing 6	CCDS47215.1	chr5_60827525-60827525_C_T	740R>C	Substitution	Nonsynonymous coding	39%
MM12T	ZSWIM8	zinc finger, SWIM-type containing 8	CCDS44440.1	chr10_75551790-75551790_G_A	498S>N	Substitution	Nonsynonymous coding	32%
MM12T	ZW10	zw10 kinetochore protein	CCDS8363.1	chr11_113614602-113614602_C_T	478R>H	Substitution	Nonsynonymous coding	14%
MM12T	ZXDA	zinc finger, X-linked, duplicated A	CCDS14376.1	chrX_57934750-57934750_A_G	702L>S	Substitution	Nonsynonymous coding	57%
MM12T	ZXDA	zinc finger, X-linked, duplicated A	CCDS14376.1	chrX_57935816-57935816_G_T	347L>I	Substitution	Nonsynonymous coding	13%
MM12T	ZYG11A	zyg-11 family member A, cell cycle regulator	CCDS44148.1	chr1_53329665-53329665_G_A	388G>R	Substitution	Nonsynonymous coding	27%
MM12T	ZZEF1	zinc finger, ZZ-type with EF-hand domain 1	CCDS11043.1	chr17_3912990-3912990_C_T	2881G>S	Substitution	Nonsynonymous coding	26%
MM13T	ABCD1	ATP-binding cassette, sub-family D (ALD), member 1	ENST00000370129	chrX_152995000-152995000_C_A	220A>D	Substitution	Nonsynonymous coding	36%
MM13T	AIFM1	apoptosis-inducing factor, mitochondrion-associated, 1	CCDS14618.1	chrX_129270133-129270133_C_T	398V>M	Substitution	Nonsynonymous coding	30%
MM13T	ALDH3B2	aldehyde dehydrogenase 3 family, member B2	CCDS31622.1	chr11_67434396-67434396_T_G	4E>A	Substitution	Nonsynonymous coding	29%
MM13T	ALX4	ALX homeobox 4	CCDS31468.1	chr11_44286451-44286451_G_A	397R>C	Substitution	Nonsynonymous coding	20%
MM13T	ANK3	ankyrin 3, node of Ranvier (ankyrin G)	CCDS7258.1	chr10_61833119-61833119_G_C	2507A>G	Substitution	Nonsynonymous coding	23%
MM13T	APLP2	amyloid beta (A4) precursor-like protein 2	CCDS8486.1	chr11_129999959-129999959__GC	NA	Insertion	Frameshift	10%
MM13T	ARC	activity-regulated cytoskeleton-associated protein	CCDS34950.1	chr8_143694459-143694459_G_A	392R>W	Substitution	Nonsynonymous coding	68%
MM13T	ARHGAP35	Rho GTPase activating protein 35	CCDS46127.1	chr19_47422518-47422519_GT_	NA	Deletion	Frameshift	33%
MM13T	ARHGEF16	Rho guanine nucleotide exchange factor (GEF) 16	CCDS46.2	chr1_3390015-3390015_G_A	412G>R	Substitution	Nonsynonymous coding	33%
MM13T	ATP1A1	ATPase, Na+/K+ transporting, alpha 1 polypeptide	CCDS887.1	chr1_116933411-116933411_T_	NA	Deletion	Frameshift	23%
MM13T	AUTS2	autism susceptibility candidate 2	CCDS5539.1	chr7_69599558-69599558_G_C	NA	Substitution	Splice site donor	12%
MM13T	BACH2	BTB and CNC homology 1, basic leucine zipper transcription factor 2	CCDS5026.1	chr6_90642510-90642510_G_A	715R>X	Substitution	Nonsense	35%
MM13T	BAZ1A	bromodomain adjacent to zinc finger domain, 1A	CCDS9651.1	chr14_35295298-35295298_C_A	153V>F	Substitution	Nonsynonymous coding	15%
MM13T	BEND3	BEN domain containing 3	CCDS34507.1	chr6_107390525-107390525_G_A	624R>C	Substitution	Nonsynonymous coding	36%
MM13T	C3orf79	chromosome 3 open reading frame 79	CCDS46937.1	chr3_153202386-153202386_T_A	14L>H	Substitution	Nonsynonymous coding	21%
MM13T	CHMP5	charged multivesicular body protein 5	CCDS6537.1	chr9_33265101-33265101__A	NA	Insertion	Frameshift	36%
MM13T	CLTB	clathrin, light chain B	NM_007097	chr5_175824647-175824647_C_T	142R>H	Substitution	Nonsynonymous coding	14%
MM13T	CNTNAP2	contactin associated protein-like 2	CCDS5889.1	chr7_146536893-146536893_C_A	100T>N	Substitution	Nonsynonymous coding	20%
MM13T	CNTNAP4	contactin associated protein-like 4	NM_138994	chr16_76555193-76555193_G_A	768R>Q	Substitution	Nonsynonymous coding	24%
MM13T	CRMP1	collapsin response mediator protein 1	CCDS33950.1	chr4_5857939-5857939_G_T	251L>I	Substitution	Nonsynonymous coding	16%
MM13T	CTCF	CCCTC-binding factor (zinc finger protein)	CCDS10841.1	chr16_67645363-67645366_AGCA_	NA	Deletion	Frameshift	24%

MM13T	DACH1	dachshund homolog 1 (Drosophila)	ENST00000359684	chr13_72147624-72147624_C_T	405R>H	Substitution	Nonsynonymous coding	21%
MM13T	DHX38	DEAH (Asp-Glu-Ala-His) box polypeptide 38	CCDS10907.1	chr16_72134988-72134988_G_C	428E>Q	Substitution	Nonsynonymous coding	42%
MM13T	DLL1	delta-like 1 (Drosophila)	CCDS5313.1	chr6_170592410-170592410_C_T	653A>T	Substitution	Nonsynonymous coding	39%
MM13T	DOCK2	dedicator of cytokinesis 2	CCDS4371.1	chr5_169230068-169230068_G_A	854R>Q	Substitution	Nonsynonymous coding	51%
MM13T	ELFN1	extracellular leucine-rich repeat and fibronectin type III domain containing 1	NM_001128636	chr7_1784787-1784787_C_A	185C>X	Substitution	Nonsense	24%
MM13T	ERAP1	endoplasmic reticulum aminopeptidase 1	CCDS4085.1	chr5_96139199-96139199_G_A	144P>L	Substitution	Nonsynonymous coding	25%
MM13T	FAM135B	family with sequence similarity 135, member B	CCDS6375.2	chr8_139209874-139209874_G_C	236H>Q	Substitution	Nonsynonymous coding	63%
MM13T	FANCM	Fanconi anemia, complementation group M	CCDS32070.1	chr14_45650909-45650909_G_A	NA	Substitution	Splice site donor	33%
MM13T	FGD1	FYVE, RhoGEF and PH domain containing 1	CCDS14359.1	chrX_54497764-54497764_G_A	155P>L	Substitution	Nonsynonymous coding	44%
MM13T	FICD	FIC domain containing	CCDS9116.1	chr12_108913130-108913130_G_A	419A>T	Substitution	Nonsynonymous coding	45%
MM13T	FOXA1	forkhead box A1	CCDS9665.1	chr14_38061844-38061845_TC_	NA	Deletion	Frameshift	19%
MM13T	FOXA2	forkhead box A2	CCDS46585.1	chr20_22563567-22563567__C	NA	Insertion	Frameshift	31%
MM13T	GAB3	GRB2-associated binding protein 3	CCDS48198.1	chrX_153906480-153906480_G_A	580T>M	Substitution	Nonsynonymous coding	22%
MM13T	GABRE	gamma-aminobutyric acid (GABA) A receptor, epsilon	CCDS14703.1	chrX_151138176-151138176_C_T	103V>I	Substitution	Nonsynonymous coding	34%
MM13T	GALNT18	UDP-N-acetyl-alpha-D-galactosamine:polypeptide N-	CCDS7807.1	chr11_11394141-11394141_C_T	338R>Q	Substitution	Nonsynonymous coding	43%
MM13T	GREB1	growth regulation by estrogen in breast cancer 1	CCDS42655.1	chr2_11716524-11716524__A	NA	Insertion	Frameshift	36%
MM13T	HCRTR2	hypocretin (orexin) receptor 2	CCDS4956.1	chr6_55142277-55142277_G_A	288A>T	Substitution	Nonsynonymous coding	38%
MM13T	HVCN1	hydrogen voltage-gated channel 1	CCDS31900.1	chr12_111089018-111089018_G_A	NA	Substitution	Splice site donor	65%
MM13T	IL13RA2	interleukin 13 receptor, alpha 2	CCDS14565.1	chrX_114249124-114249124_T_C	87K>R	Substitution	Nonsynonymous coding	24%
MM13T	IRS4	insulin receptor substrate 4	CCDS14544.1	chrX_107978999-107978999_C_A	192L>F	Substitution	Nonsynonymous coding	37%
MM13T	KCNG4	potassium voltage-gated channel, subfamily G, member 4	CCDS10945.1	chr16_84270841-84270841_C_T	84R>H	Substitution	Nonsynonymous coding	32%
MM13T	KCNK5	potassium channel, subfamily K, member 5	CCDS4841.1	chr6_39159259-39159259_C_T	303D>N	Substitution	Nonsynonymous coding	37%
MM13T	KCTD19	potassium channel tetramerisation domain containing 19	CCDS42179.1	chr16_67327811-67327812_GA_	NA	Deletion	Frameshift	30%
MM13T	KCTD8	potassium channel tetramerisation domain containing 8	CCDS3467.1	chr4_44450429-44450429_C_T	38A>T	Substitution	Nonsynonymous coding	27%
MM13T	KIAA1109	Uncharacterized protein	CCDS43267.1	chr4_123141510-123141510_A_G	833E>G	Substitution	Nonsynonymous coding	13%
MM13T	LEPRE1	leucine proline-enriched proteoglycan (leprecan) 1	ENST00000236040	chr1_43212896-43212896_G_A	701P>L	Substitution	Nonsynonymous coding	33%
MM13T	LRIT1	leucine-rich repeat, immunoglobulin-like and transmembrane domains 1	CCDS7373.1	chr10_85992410-85992410_T_C	382H>R	Substitution	Nonsynonymous coding	75%
MM13T	LRP1B	low density lipoprotein receptor-related protein 1B	CCDS2182.1	chr2_141812742-141812742_G_A	499R>W	Substitution	Nonsynonymous coding	26%
MM13T	MAST3	microtubule associated serine/threonine kinase 3	CCDS46014.1	chr19_18245498-18245498_G_T	532A>S	Substitution	Nonsynonymous coding	47%
MM13T	ME2	malic enzyme 2, NAD(+)-dependent, mitochondrial	CCDS11948.1	chr18_48473489-48473489_T_A	564S>T	Substitution	Nonsynonymous coding	17%
MM13T	MED12L	mediator complex subunit 12-like	CCDS33876.1	chr3_150877641-150877641_C_T	287S>L	Substitution	Nonsynonymous coding	37%
MM13T	MLL3	myeloid/lymphoid or mixed-lineage leukemia 3	CCDS5931.1	chr7_151845228-151845228_C_T	4595R>H	Substitution	Nonsynonymous coding	22%

MM13T	MRPL28	mitochondrial ribosomal protein L28	CCDS32349.1	chr16_420119-420119_C_A	34E>X	Substitution	Nonsense	35%
MM13T	MTA2	metastasis associated 1 family, member 2	CCDS8022.1	chr11_62367678-62367678_G_T	50D>E	Substitution	Nonsynonymous coding	10%
MM13T	MTSS1L	metastasis suppressor 1-like	CCDS32476.1	chr16_70699395-70699395_C_T	368V>I	Substitution	Nonsynonymous coding	27%
MM13T	MYH6	myosin, heavy chain 6, cardiac muscle, alpha	CCDS9600.1	chr14_23866412-23866412_G_A	673R>C	Substitution	Nonsynonymous coding	40%
MM13T	MYOF	myoferlin	CCDS41551.1	chr10_95111278-95111279_CA_	NA	Deletion	Frameshift	20%
MM13T	NFIC	nuclear factor I/C (CCAAT-binding transcription factor)	CCDS45914.1	chr19_3452578-3452578_C_T	386R>C	Substitution	Nonsynonymous coding	22%
MM13T	NPY1R	neuropeptide Y receptor Y1	CCDS34089.1	chr4_164247631-164247631_G_T	26L>M	Substitution	Nonsynonymous coding	35%
MM13T	NT5DC4	5'-nucleotidase domain containing 4	ENST00000327581	chr2_113479589-113479589_C_G	38L>V	Substitution	Nonsynonymous coding	30%
MM13T	NUP160	nucleoporin 160kDa	CCDS31484.1	chr11_47814444-47814448_AGAGT_	NA	Deletion	Frameshift	26%
MM13T	OR11L1	olfactory receptor, family 11, subfamily L, member 1	CCDS31098.1	chr1_248004543-248004543_A_T	219V>D	Substitution	Nonsynonymous coding	20%
MM13T	OR8G1	olfactory receptor, family 8, subfamily G, member 1	NM_001002905	chr11_124121273-124121273_T_G	284M>R	Substitution	Nonsynonymous coding	11%
MM13T	PAFAH2	platelet-activating factor acetylhydrolase 2, 40kDa	CCDS270.1	chr1_26288563-26288563_C_G	366D>H	Substitution	Nonsynonymous coding	33%
MM13T	PDLIM5	PDZ and LIM domain 5	CCDS3641.1	chr4_95376489-95376489_G_A	17R>Q	Substitution	Nonsynonymous coding	42%
MM13T	PDZRN4	PDZ domain containing ring finger 4	CCDS8739.1	chr12_41967456-41967456_C_T	701R>C	Substitution	Nonsynonymous coding	23%
MM13T	PIK3R1	phosphoinositide-3-kinase, regulatory subunit 1 (alpha)	CCDS3993.1	chr5_67591132-67591134_GAC_	575KT>K	Deletion	In-frame deletion	45%
MM13T	PIK3R1	phosphoinositide-3-kinase, regulatory subunit 1 (alpha)	CCDS3993.1	chr5_67588168-67588168_G_	NA	Deletion	Frameshift	30%
MM13T	PPP1R18	protein phosphatase 1, regulatory subunit 18	CCDS43444.1	chr6_30652397-30652397_G_A	467R>C	Substitution	Nonsynonymous coding	35%
MM13T	PRKDC	protein kinase, DNA-activated, catalytic polypeptide	ENST00000314191	chr8_48743181-48743181__T	NA	Insertion	Frameshift	69%
MM13T	PTEN	phosphatase and tensin homolog	NM_000314	chr10_89692904-89692904_C_G	130R>G	Substitution	Nonsynonymous coding	75%
MM13T	PTGDR	prostaglandin D2 receptor (DP)	CCDS9707.1	chr14_52735046-52735046_G_A	172G>R	Substitution	Nonsynonymous coding	43%
MM13T	QRFPR	pyroglutamylated RFamide peptide receptor	CCDS3719.1	chr4_122258006-122258006_C_T	173A>T	Substitution	Nonsynonymous coding	16%
MM13T	RAPGEF6	Rap guanine nucleotide exchange factor (GEF) 6	NM_001164389	chr5_130771876-130771876_G_C	1315P>R	Substitution	Nonsynonymous coding	56%
MM13T	RBBP7	retinoblastoma binding protein 7	CCDS14179.1	chrX_16870879-16870879_G_A	293A>V	Substitution	Nonsynonymous coding	38%
MM13T	RP5-944M2.3	Known lincRNA	ENST00000397346	chr12_126942660-126942660_C_A	118A>D	Substitution	Nonsynonymous coding	22%
MM13T	SALL1	sal-like 1 (Drosophila)	CCDS10747.1	chr16_51173432-51173432__C	NA	Insertion	Frameshift	29%
MM13T	SPEG	SPEG complex locus	CCDS42824.1	chr2_220312959-220312959_C_T	360S>L	Substitution	Nonsynonymous coding	35%
MM13T	SUN1	Sad1 and UNC84 domain containing 1	CCDS47525.1	chr7_912960-912960_G_A	784V>I	Substitution	Nonsynonymous coding	29%
MM13T	TELO2	TEL2, telomere maintenance 2, homolog (S. cerevisiae)	CCDS32363.1	chr16_1544365-1544365_T_G	28I>S	Substitution	Nonsynonymous coding	38%
MM13T	TGFBRAP1	transforming growth factor, beta receptor associated protein 1	CCDS2067.1	chr2_105897062-105897062_T_A	414T>S	Substitution	Nonsynonymous coding	31%
MM13T	TM4SF18	transmembrane 4 L six family member 18	CCDS3142.1	chr3_149042713-149042713_G_A	122R>C	Substitution	Nonsynonymous coding	26%
MM13T	TMEM174	transmembrane protein 174	CCDS4018.1	chr5_72469593-72469593_G_A	175A>T	Substitution	Nonsynonymous coding	26%
MM13T	TP53	tumor protein p53	CCDS11118.1	chr17_7577539-7577539_G_A	248R>W	Substitution	Nonsynonymous coding	72%

MM13T	TXNIP	thioredoxin interacting protein	CCDS913.1	chr1_145440910-145440910__T	NA	Insertion	Frameshift	29%
MM13T	UBE2J2	ubiquitin-conjugating enzyme E2, J2	ENST00000450390	chr1_1201553-1201553_C_T	52R>Q	Substitution	Nonsynonymous coding	34%
MM13T	URB1	URB1 ribosome biogenesis 1 homolog (S. cerevisiae)	CCDS46645.1	chr21_33709719-33709719_C_T	1539V>I	Substitution	Nonsynonymous coding	31%
MM14T	ARHGEF4	Rho guanine nucleotide exchange factor (GEF) 4	ENST00000409359	chr2_131674634-131674634_G_C	709G>R	Substitution	Nonsynonymous coding	61%
MM14T	ASH1L	ash1 (absent, small, or homeotic)-like (Drosophila)	CCDS1113.2	chr1_155451916-155451920_CCAAT_	NA	Deletion	Frameshift	41%
MM14T	ATOH8	atonal homolog 8 (Drosophila)	CCDS1985.1	chr2_85991262-85991262_G_A	306C>Y	Substitution	Nonsynonymous coding	61%
MM14T	BAHCC1	BAH domain and coiled-coil containing 1 [Source:HGNC Symbol;Acc:29279]	ENST00000436173	chr17_79409975-79409975_G_A	534G>S	Substitution	Nonsynonymous coding	13%
MM14T	BIRC6	baculoviral IAP repeat containing 6	CCDS33175.2	chr2_32667409-32667409_A_T	1375E>V	Substitution	Nonsynonymous coding	26%
MM14T	C12orf40	chromosome 12 open reading frame 40	CCDS41770.1	chr12_40040191-40040191_A_T	88K>M	Substitution	Nonsynonymous coding	41%
MM14T	CCDC42B	coiled-coil domain containing 42B	CCDS44983.1	chr12_113591080-113591080_G_A	112R>Q	Substitution	Nonsynonymous coding	31%
MM14T	CD40	CD40 molecule, TNF receptor superfamily member 5	CCDS13393.1	chr20_44756835-44756835_T_G	206F>L	Substitution	Nonsynonymous coding	25%
MM14T	CHDH	choline dehydrogenase	CCDS2873.1	chr3_53857533-53857533_C_T	168R>H	Substitution	Nonsynonymous coding	58%
MM14T	CNKSR2	connector enhancer of kinase suppressor of Ras 2	CCDS14198.1	chrX_21545105-21545105_C_A	360P>T	Substitution	Nonsynonymous coding	36%
MM14T	COL7A1	collagen, type VII, alpha 1	CCDS2773.1	chr3_48627937-48627937_C_T	621V>I	Substitution	Nonsynonymous coding	58%
MM14T	CUX1	cut-like homeobox 1	CCDS5721.1	chr7_101838860-101838860_G_A	400R>H	Substitution	Nonsynonymous coding	35%
MM14T	CYLC1	cylicin, basic protein of sperm head cytoskeleton 1	CCDS35341.1	chrX_83129621-83129621_A_T	635K>N	Substitution	Nonsynonymous coding	33%
MM14T	DFFB	DNA fragmentation factor, 40kDa, beta polypeptide (caspase-activated DNase)	CCDS52.1	chr1_3800067-3800067_G_A	NA	Substitution	Splice site acceptor	37%
MM14T	FAM32A	family with sequence similarity 32, member A	CCDS12341.1	chr19_16296505-16296505_G_A	49E>K	Substitution	Nonsynonymous coding	72%
MM14T	FUOM	fucose mutarotase	CCDS44499.1	chr10_135170181-135170181_G_A	96T>M	Substitution	Nonsynonymous coding	47%
MM14T	IGBP1	immunoglobulin (CD79A) binding protein 1	CCDS14396.1	chrX_69354579-69354579_A_T	132N>I	Substitution	Nonsynonymous coding	32%
MM14T	LEMD1	LEM domain containing 1	CCDS30986.1	chr1_205388376-205388376_C_T	63D>N	Substitution	Nonsynonymous coding	42%
MM14T	MDM1	Mdm1 nuclear protein homolog (mouse)	CCDS8983.1	chr12_68690766-68690766_A_	NA	Deletion	Splice site acceptor	14%
MM14T	NES	nestin	CCDS1151.1	chr1_156640613-156640613_G_A	1123P>S	Substitution	Nonsynonymous coding	25%
MM14T	OR6C68	olfactory receptor, family 6, subfamily C, member 68	CCDS31826.1	chr12_55886545-55886545_G_T	133L>F	Substitution	Nonsynonymous coding	49%
MM14T	OR8H2	olfactory receptor, family 8, subfamily H, member 2	CCDS31518.1	chr11_55872883-55872883_G_A	122R>H	Substitution	Nonsynonymous coding	91%
MM14T	OXT	oxytocin/neurophysin I prepropeptide	CCDS13044.1	chr20_3052335-3052335_G_A	12G>S	Substitution	Nonsynonymous coding	54%
MM14T	RANBP2	RAN binding protein 2	CCDS2079.1	chr2_109392300-109392300_C_T	2802S>F	Substitution	Nonsynonymous coding	35%
MM14T	RPS6KA2	ribosomal protein S6 kinase, 90kDa, polypeptide 2	CCDS34570.1	chr6_166952172-166952172_T_A	75Q>L	Substitution	Nonsynonymous coding	29%
MM14T	SBK2	SH3-binding domain kinase family, member 2	CCDS42631.1	chr19_56041641-56041641_G_T	169S>Y	Substitution	Nonsynonymous coding	82%
MM14T	SMG7	smg-7 homolog, nonsense mediated mRNA decay factor (C. elegans)	CCDS1355.1	chr1_183518896-183518896_C_A	NA	Substitution	Splice site acceptor	13%
MM14T	SMG7	smg-7 homolog, nonsense mediated mRNA decay factor (C. elegans)	CCDS1355.1	chr1_183519915-183519915_C_T	1005H>Y	Substitution	Nonsynonymous coding	12%
MM14T	SMG7	smg-7 homolog, nonsense mediated mRNA decay factor (C. elegans)	CCDS1355.1	chr1_183520950-183520950_C_T	NA	Substitution	Splice site acceptor	15%

MM14T	SYNGAP1	synaptic Ras GTPase activating protein 1	CCDS34434.2	chr6_33405458-33405458_G_	NA	Deletion	Frameshift	29%
MM14T	TAB1	TGF-beta activated kinase 1/MAP3K7 binding protein 1	CCDS13993.1	chr22_39815617-39815617_C_T	253T>M	Substitution	Nonsynonymous coding	91%
MM14T	TMC2	transmembrane channel-like 2	CCDS13029.2	chr20_2593864-2593864_G_A	590D>N	Substitution	Nonsynonymous coding	42%
MM14T	TP53	tumor protein p53	CCDS11118.1	chr17_7577556-7577556_C_A	242C>F	Substitution	Nonsynonymous coding	90%
MM14T	TPSG1	tryptase gamma 1	CCDS10430.1	chr16_1272663-1272663_G_A	167T>M	Substitution	Nonsynonymous coding	38%
MM14T	TPX2	TPX2, microtubule-associated, homolog (Xenopus laevis)	CCDS13190.1	chr20_30359385-30359385_G_A	170E>K	Substitution	Nonsynonymous coding	19%
MM14T	TRBV5-1	T cell receptor beta variable 5-1	ENST00000390381	chr7_142021217-142021217_T_C	66F>S	Substitution	Nonsynonymous coding	33%
MM14T	TROVE2	TROVE domain family, member 2	CCDS1379.1	chr1_193038621-193038621_G_A	146R>H	Substitution	Nonsynonymous coding	31%
MM14T	TRRAP	transformation/transcription domain-associated protein	CCDS5659.1	chr7_98579562-98579562_C_G	2910H>Q	Substitution	Nonsynonymous coding	36%
MM14T	TTN	titin	ENST00000356127	chr2_179440435-179440435_C_T	20905R>H	Substitution	Nonsynonymous coding	65%
MM14T	TTN	titin	ENST00000356127	chr2_179498311-179498311_G_C	11691P>A	Substitution	Nonsynonymous coding	37%
MM14T	UBR5	ubiquitin protein ligase E3 component n-recognin 5	CCDS34933.1	chr8_103289345-103289345_C_G	2122G>R	Substitution	Nonsynonymous coding	41%
MM14T	UFL1	UFM1-specific ligase 1	CCDS5034.1	chr6_97001177-97001177_T_C	728L>S	Substitution	Nonsynonymous coding	28%
MM14T	WDFY3	WD repeat and FYVE domain containing 3	CCDS3609.1	chr4_85711005-85711015_GCGAGCCACGC_	NA	Deletion	Frameshift	36%
MM14T	ZAP70	zeta-chain (TCR) associated protein kinase 70kDa	CCDS33254.1	chr2_98354542-98354542_G_A	570A>T	Substitution	Nonsynonymous coding	48%
MM14T	ZNF57	zinc finger protein 57	CCDS12098.1	chr19_2916207-2916207_T_C	88C>R	Substitution	Nonsynonymous coding	94%
MM15T	AMER1	APC membrane recruitment protein 1	CCDS14377.2	chrX_63410804-63410804_C_T	788C>Y	Substitution	Nonsynonymous coding	21%
MM15T	COL21A1	collagen, type XXI, alpha 1	NM_030820	chr6_56033015-56033015_T_G	369Q>H	Substitution	Nonsynonymous coding	14%
MM15T	CPAMD8	C3 and PZP-like, alpha-2-macroglobulin domain containing 8	CCDS42519.1	chr19_17086920-17086920_C_G	647E>D	Substitution	Nonsynonymous coding	27%
MM15T	DPP10	dipeptidyl-peptidase 10 (non-functional)	CCDS46400.1	chr2_116599913-116599913_G_A	795D>N	Substitution	Nonsynonymous coding	18%
MM15T	ETV2	ets variant 2	NM_014209	chr19_36133516-36133516_G_C	_ISV-1>	Substitution	Splice site acceptor	27%
MM15T	FBXW7	F-box and WD repeat domain containing 7, E3 ubiquitin protein ligase	CCDS3777.1	chr4_153250924-153250924_T_G	379H>P	Substitution	Nonsynonymous coding	50%
MM15T	GLI3	GLI family zinc finger 3	CCDS5465.1	chr7_42262788-42262788_T_A	22K>M	Substitution	Nonsynonymous coding	28%
MM15T	HK3	hexokinase 3 (white cell)	CCDS4407.1	chr5_176317822-176317822_C_T	_ISV+1>	Substitution	Splice site donor	40%
MM15T	ITFG3	integrin alpha FG-GAP repeat containing 3	CCDS10402.1	chr16_304668-304668_T_C	86Y>H	Substitution	Nonsynonymous coding	32%
MM15T	KRAS	v-Ki-ras2 Kirsten rat sarcoma viral oncogene homolog	CCDS8703.1	chr12_25398284-25398284_C_T	12G>D	Substitution	Nonsynonymous coding	59%
MM15T	MITF	microphthalmia-associated transcription factor	CCDS43106.1	chr3_69928509-69928509_C_G	110T>R	Substitution	Nonsynonymous coding	77%
MM15T	NOTCH2	notch 2	CCDS908.1	chr1_120612003-120612004_GG_	NA	Deletion	Frameshift	25%
MM15T	PRDM11	PR domain containing 11	CCDS7912.1	chr11_45203391-45203391_C_G	59T>R	Substitution	Nonsynonymous coding	49%
MM15T	SAP130	Sin3A-associated protein, 130kDa	CCDS2153.1	chr2_128712584-128712584_T_C	791M>V	Substitution	Nonsynonymous coding	54%
MM15T	SCP2D1	SCP2 sterol-binding domain containing 1	CCDS13139.1	chr20_18794541-18794541_G_T	28V>F	Substitution	Nonsynonymous coding	28%
MM15T	SEC24C	SEC24 family, member C (S. cerevisiae)	CCDS7332.1	chr10_75519456-75519456_C_T	129P>S	Substitution	Nonsynonymous coding	29%

MM15T	SLC26A3	solute carrier family 26, member 3	CCDS5748.1	chr7_107431671-107431671_G_A	131P>L	Substitution	Nonsynonymous coding	11%
MM15T	TG	thyroglobulin	CCDS34944.1	chr8_134042140-134042140_C_T	2371R>X	Substitution	Nonsense	28%
MM15T	TP53	tumor protein p53	CCDS11118.1	chr17_7578406-7578406_C_T	175R>H	Substitution	Nonsynonymous coding	69%
MM15T	WDFY3	WD repeat and FYVE domain containing 3	CCDS3609.1	chr4_85594083-85594083_G_T	3507Q>K	Substitution	Nonsynonymous coding	22%
MM16T	ADAMTS9	ADAM metallopeptidase with thrombospondin type 1 motif, 9	CCDS2903.1	chr3_64672264-64672264_T_G	166I>L	Substitution	Nonsynonymous coding	10%
MM16T	ANKS3	ankyrin repeat and sterile alpha motif domain containing 3	CCDS10520.1	chr16_4755225-4755225_C_T	247E>K	Substitution	Nonsynonymous coding	51%
MM16T	AR	androgen receptor	CCDS14387.1	chrX_66943546-66943546_C_G	876Q>E	Substitution	Nonsynonymous coding	24%
MM16T	BCL11B	B-cell CLL/lymphoma 11B (zinc finger protein)	CCDS9950.1	chr14_99697816-99697816_G_	NA	Deletion	Frameshift	17%
MM16T	CCDC129	coiled-coil domain containing 129	CCDS5435.2	chr7_31614356-31614356_G_A	200G>S	Substitution	Nonsynonymous coding	26%
MM16T	CDH19	cadherin 19, type 2	CCDS11994.1	chr18_64172366-64172366_G_A	668R>C	Substitution	Nonsynonymous coding	86%
MM16T	CPT1C	carnitine palmitoyltransferase 1C	ENST00000295404	chr19_50213770-50213770_C_T	425P>L	Substitution	Nonsynonymous coding	45%
MM16T	FAM118A	family with sequence similarity 118, member A	ENST00000405673	chr22_45724286-45724286_C_G	179A>G	Substitution	Nonsynonymous coding	21%
MM16T	FBXW7	F-box and WD repeat domain containing 7, E3 ubiquitin protein ligase	CCDS3777.1	chr4_153244131-153244131__T	NA	Insertion	Frameshift	49%
MM16T	FRRS1	ferric-chelate reductase 1	CCDS30780.1	chr1_100194079-100194079_T_C	326I>V	Substitution	Nonsynonymous coding	38%
MM16T	GGA3	golgi-associated, gamma adaptin ear containing, ARF binding protein 3	CCDS11717.1	chr17_73236132-73236132_C_T	441A>T	Substitution	Nonsynonymous coding	31%
MM16T	GPR115	G protein-coupled receptor 115	CCDS4922.2	chr6_47682367-47682367_C_A	462H>Q	Substitution	Nonsynonymous coding	28%
MM16T	IL23R	interleukin 23 receptor	CCDS637.1	chr1_67633851-67633851_A_	NA	Deletion	Frameshift	27%
MM16T	KCNA7	potassium voltage-gated channel, shaker-related subfamily, member 7	CCDS12755.1	chr19_49573379-49573379_G_C	438P>A	Substitution	Nonsynonymous coding	31%
MM16T	KEL	Kell blood group, metallo-endopeptidase	CCDS34766.1	chr7_142658931-142658931_G_A	11P>L	Substitution	Nonsynonymous coding	49%
MM16T	KIAA1239	uncharacterized protein	CCDS47040.1	chr4_37446254-37446254_A_C	882N>H	Substitution	Nonsynonymous coding	34%
MM16T	KIAA1383	uncharacterized protein	CCDS44334.1	chr1_232941227-232941227_C_T	153S>L	Substitution	Nonsynonymous coding	57%
MM16T	KIF26B	kinesin family member 26B	CCDS44342.1	chr1_245851227-245851227_G_A	1648A>T	Substitution	Nonsynonymous coding	23%
MM16T	KIF27	kinesin family member 27	CCDS6665.1	chr9_86452358-86452360_CCT_	NA	Deletion	In-frame deletion	11%
MM16T	KRAS	v-Ki-ras2 Kirsten rat sarcoma viral oncogene homolog	CCDS8703.1	chr12_25398285-25398285_C_A	12G>C	Substitution	Nonsynonymous coding	49%
MM16T	LARP4B	La ribonucleoprotein domain family, member 4B	CCDS31131.1	chr10_890938-890938_G_	NA	Deletion	Frameshift	39%
MM16T	LRP2	low density lipoprotein receptor-related protein 2	CCDS2232.1	chr2_169989109-169989109_G_A	4568T>I	Substitution	Nonsynonymous coding	69%
MM16T	MYH7	myosin, heavy chain 7, cardiac muscle, beta	CCDS9601.1	chr14_23889403-23889403_C_T	1126R>H	Substitution	Nonsynonymous coding	45%
MM16T	OR4A16	olfactory receptor, family 4, subfamily A, member 16	CCDS31499.1	chr11_55111454-55111454_G_A	260V>I	Substitution	Nonsynonymous coding	83%
MM16T	PARP14	poly (ADP-ribose) polymerase family, member 14	CCDS46894.1	chr3_122446760-122446760_C_	NA	Deletion	Frameshift	35%
MM16T	PLAGL1	pleiomorphic adenoma gene-like 1	CCDS5202.1	chr6_144263744-144263744_G_A	70T>M	Substitution	Nonsynonymous coding	32%
MM16T	PLD3	phospholipase D family, member 3	CCDS33027.1	chr19_40883776-40883781_ACGCGC_	NA	Deletion	In-frame deletion	14%
MM16T	PPIE	peptidylprolyl isomerase E (cyclophilin E)	CCDS443.1	chr1_40204599-40204599_C_G	9Y>X	Substitution	Nonsense	13%

MM16T	PPP2R1A	protein phosphatase 2, regulatory subunit A, alpha	CCDS12849.1	chr19_52715974-52715974_T_G	180M>R	Substitution	Nonsynonymous coding	48%
MM16T	PPP4R4	protein phosphatase 4, regulatory subunit 4	CCDS9921.1	chr14_94700023-94700023_T_C	184S>P	Substitution	Nonsynonymous coding	54%
MM16T	SMG9	smg-9 homolog, nonsense mediated mRNA decay factor (C. elegans)	CCDS33043.2	chr19_44241806-44241806_G_A	313T>M	Substitution	Nonsynonymous coding	39%
MM16T	SPEN	spen homolog, transcriptional regulator	CCDS164.1	chr1_16256454-16256454_A_C	1240K>T	Substitution	Nonsynonymous coding	32%
MM16T	SRPK2	SRSF protein kinase 2	CCDS34724.1	chr7_104782615-104782615_G_T	461F>L	Substitution	Nonsynonymous coding	37%
MM16T	STARD9	StAR-related lipid transfer (START) domain containing 9	NM_020759	chr15_42981079-42981079_A_G	2435R>G	Substitution	Nonsynonymous coding	29%
MM16T	TENM1	teneurin transmembrane protein 1	CCDS14609.1	chrX_123518394-123518394_C_G	2122W>C	Substitution	Nonsynonymous coding	84%
MM16T	TLR10	toll-like receptor 10	CCDS3445.1	chr4_38775719-38775719_T_A	498N>I	Substitution	Nonsynonymous coding	49%
MM16T	TP53	tumor protein p53	CCDS11118.1	chr17_7577153-7577153_C_A	262G>V	Substitution	Nonsynonymous coding	94%
MM16T	XYLT2	xylosyltransferase II	CCDS11563.1	chr17_48433239-48433239_G_T	396D>Y	Substitution	Nonsynonymous coding	14%
MM16T	ZP3	zona pellucida glycoprotein 3 (sperm receptor)	CCDS47618.1	chr7_76069661-76069663_GAG_	NA	Deletion	Splice site donor	16%
MM17T	ACIN1	apoptotic chromatin condensation inducer 1	CCDS9587.1	chr14_23549151-23549151_C_T	523D>N	Substitution	Nonsynonymous coding	48%
MM17T	ACSBG1	acyl-CoA synthetase bubblegum family member 1	CCDS10298.1	chr15_78470955-78470955_C_A	NA	Substitution	Splice site donor	21%
MM17T	AKAP8L	A kinase (PRKA) anchor protein 8-like	CCDS46005.1	chr19_15508058-15508058_T_G	480E>A	Substitution	Nonsynonymous coding	11%
MM17T	ARID5B	AT rich interactive domain 5B (MRF1-like)	CCDS31208.1	chr10_63851941-63851941_G_T	907D>Y	Substitution	Nonsynonymous coding	45%
MM17T	AXIN2	axin 2	CCDS11662.1	chr17_63554009-63554009_A_G	244S>P	Substitution	Nonsynonymous coding	48%
MM17T	BHLHE40	basic helix-loop-helix family, member e40	CCDS2565.1	chr3_5024758-5024758_C_T	207S>F	Substitution	Nonsynonymous coding	48%
MM17T	BRCA2	breast cancer 2, early onset	CCDS9344.1	chr13_32914647-32914647_C_A	2052S>X	Substitution	Nonsense	94%
MM17T	CCDC74A	coiled-coil domain containing 74A	ENST00000434330	chr2_132287094-132287094_G_A	37G>E	Substitution	Nonsynonymous coding	16%
MM17T	CELSR3	cadherin, EGF LAG seven-pass G-type receptor 3 (flamingo homolog, Drosophila)	CCDS2775.1	chr3_48677585-48677585_C_G	3145D>H	Substitution	Nonsynonymous coding	17%
MM17T	CLK3	CDC-like kinase 3	CCDS45304.1	chr15_74918270-74918270_G_T	408V>F	Substitution	Nonsynonymous coding	24%
MM17T	COL6A3	collagen, type VI, alpha 3	CCDS33412.1	chr2_238272044-238272044_G_T	NA	Substitution	Splice site acceptor	86%
MM17T	CTNNA2	catenin (cadherin-associated protein), alpha 2	CCDS42703.2	chr2_80782821-80782821_A_G	515N>S	Substitution	Nonsynonymous coding	57%
MM17T	CUL9	cullin 9	CCDS4890.1	chr6_43156381-43156381_C_T	703S>F	Substitution	Nonsynonymous coding	49%
MM17T	DMGDH	dimethylglycine dehydrogenase	CCDS4044.1	chr5_78326750-78326750_G_A	530A>V	Substitution	Nonsynonymous coding	14%
MM17T	DNAJC6	DnaJ (Hsp40) homolog, subfamily C, member 6	CCDS30739.1	chr1_65831857-65831857_G_A	117R>Q	Substitution	Nonsynonymous coding	40%
MM17T	DZANK1	double zinc ribbon and ankyrin repeat domains 1	CCDS46582.1	chr20_18440911-18440911_C_G	50S>T	Substitution	Nonsynonymous coding	39%
MM17T	DZIP3	DAZ interacting protein 3, zinc finger	CCDS2952.1	chr3_108363508-108363508_A_G	547I>V	Substitution	Nonsynonymous coding	28%
MM17T	EEPD1	endonuclease/exonuclease/phosphatase family domain containing 1	CCDS34619.1	chr7_36194279-36194279_C_G	116R>G	Substitution	Nonsynonymous coding	50%
MM17T	EPS8	epidermal growth factor receptor pathway substrate 8	CCDS31753.1	chr12_15814976-15814976_C_T	265R>H	Substitution	Nonsynonymous coding	49%
MM17T	FAM174A	family with sequence similarity 174, member A	CCDS4090.1	chr5_99871445-99871445_G_T	71A>S	Substitution	Nonsynonymous coding	43%
MM17T	FOXK1	forkhead box K1	CCDS34591.1	chr7_4800743-4800743_G_T	582G>V	Substitution	Nonsynonymous coding	52%

MM17T	HLA-DRB1	major histocompatibility complex, class II, DR beta 1	CCDS47409.1	chr6_32548544-32548544_T_G	248I>L	Substitution	Nonsynonymous coding	19%
MM17T	HMCN1	hemicentin 1	CCDS30956.1	chr1_186114995-186114995_G_A	4850G>S	Substitution	Nonsynonymous coding	44%
MM17T	IMPA2	inositol(myo)-1(or 4)-monophosphatase 2	CCDS11855.1	chr18_11981733-11981733_C_G	22A>G	Substitution	Nonsynonymous coding	17%
MM17T	ITPR2	inositol 1,4,5-trisphosphate receptor, type 2	CCDS41764.1	chr12_26775240-26775240_G_A	1074S>F	Substitution	Nonsynonymous coding	47%
MM17T	ITPR3	inositol 1,4,5-trisphosphate receptor, type 3	CCDS4783.1	chr6_33654232-33654232_C_A	1972A>E	Substitution	Nonsynonymous coding	40%
MM17T	MDN1	MDN1, midasin homolog (yeast)	CCDS5024.1	chr6_90361988-90361988_C_G	5280D>H	Substitution	Nonsynonymous coding	39%
MM17T	MTHFSD	methenyltetrahydrofolate synthetase domain containing	CCDS42211.1	chr16_86575799-86575799_C_G	154E>Q	Substitution	Nonsynonymous coding	48%
MM17T	MYO10	myosin X	NM_012334	chr5_16764364-16764364_A_T	441F>I	Substitution	Nonsynonymous coding	21%
MM17T	NDUFS3	NADH dehydrogenase (ubiquinone) Fe-S protein 3, 30kDa (NADH-coenzyme Q	CCDS7941.1	chr11_47602517-47602517_C_G	121T>S	Substitution	Nonsynonymous coding	30%
MM17T	NPFFR1	neuropeptide FF receptor 1	ENST00000277942	chr10_72015528-72015528_C_G	160V>L	Substitution	Nonsynonymous coding	20%
MM17T	OR5R1	olfactory receptor, family 5, subfamily R, member 1	CCDS31530.1	chr11_56185462-56185462_C_T	83V>M	Substitution	Nonsynonymous coding	22%
MM17T	OTOF	otoferlin	CCDS1725.1	chr2_26698238-26698238_G_T	1039Q>K	Substitution	Nonsynonymous coding	34%
MM17T	PCDH15	protocadherin-related 15	CCDS44400.1	chr10_55570388-55570388_C_A	1494C>F	Substitution	Nonsynonymous coding	35%
MM17T	PCNXL2	pecanex-like 2 (Drosophila)	CCDS44335.1	chr1_233275584-233275584_A_G	1179W>R	Substitution	Nonsynonymous coding	31%
MM17T	PDE1C	phosphodiesterase 1C, calmodulin-dependent 70kDa	CCDS5437.1	chr7_31918723-31918723_T_A	104D>V	Substitution	Nonsynonymous coding	32%
MM17T	PDE4DIP	phosphodiesterase 4D interacting protein	CCDS30826.1	chr1_144931168-144931168_C_G	181E>Q	Substitution	Nonsynonymous coding	23%
MM17T	PEX11B	peroxisomal biogenesis factor 11 beta	CCDS917.1	chr1_145522815-145522815_G_A	226G>S	Substitution	Nonsynonymous coding	56%
MM17T	PIK3R1	phosphoinositide-3-kinase, regulatory subunit 1 (alpha)	CCDS3993.1	chr5_67593395-67593395__T	NA	Insertion	Frameshift	41%
MM17T	PLCL2	phospholipase C-like 2	CCDS33713.1	chr3_17051959-17051959__CA	NA	Insertion	Frameshift	31%
MM17T	PP2D1	protein phosphatase 2C-like domain containing 1	ENST00000389050	chr3_20043147-20043147_C_A	155R>S	Substitution	Nonsynonymous coding	18%
MM17T	PRR12	proline rich 12	CCDS46143.1	chr19_50099424-50099424_G_T	611G>V	Substitution	Nonsynonymous coding	33%
MM17T	PTEN	phosphatase and tensin homolog	NM_000314	chr10_89711993-89711993_C_G	204P>R	Substitution	Nonsynonymous coding	85%
MM17T	QSOX1	quiescin Q6 sulfhydryl oxidase 1	CCDS1337.1	chr1_180158780-180158780_T_C	371F>L	Substitution	Nonsynonymous coding	14%
MM17T	RAD51C	RAD51 homolog C (S. cerevisiae)	CCDS11611.1	chr17_56770090-56770090_C_T	29S>F	Substitution	Nonsynonymous coding	55%
MM17T	RARB	retinoic acid receptor, beta	ENST00000404969	chr3_25216037-25216037_C_T	50P>L	Substitution	Nonsynonymous coding	67%
MM17T	RGL2	ral guanine nucleotide dissociation stimulator-like 2	CCDS4774.1	chr6_33260971-33260971_G_A	610P>L	Substitution	Nonsynonymous coding	19%
MM17T	SARNP	SAP domain containing ribonucleoprotein	CCDS8892.1	chr12_56182893-56182893_C_T	156R>K	Substitution	Nonsynonymous coding	32%
MM17T	SELENBP1	selenium binding protein 1	CCDS995.1	chr1_151341497-151341497_C_	NA	Deletion	Frameshift	59%
MM17T	SERPINB5	serpin peptidase inhibitor, clade B (ovalbumin), member 5	CCDS32839.1	chr18_61170668-61170668_A_T	281M>L	Substitution	Nonsynonymous coding	26%
MM17T	SIGLEC5	sialic acid binding Ig-like lectin 5	CCDS33088.1	chr19_52115595-52115595_T_A	515Q>H	Substitution	Nonsynonymous coding	28%
MM17T	ST3GAL1	ST3 beta-galactoside alpha-2,3-sialyltransferase 1	CCDS6373.1	chr8_134477117-134477117_C_T	196R>Q	Substitution	Nonsynonymous coding	22%
MM17T	SUPV3L1	suppressor of var1, 3-like 1 (S. cerevisiae)	CCDS7287.1	chr10_70946225-70946225_A_T	126S>C	Substitution	Nonsynonymous coding	45%

MM17T	TAPBPL	TAP binding protein-like	CCDS8546.1	chr12_6566767-6566767_C_T	254T>I	Substitution	Nonsynonymous coding	14%
MM17T	TBATA	thymus, brain and testes associated	CCDS7308.1	chr10_72541623-72541623_G_A	71R>C	Substitution	Nonsynonymous coding	54%
MM17T	TCF7	transcription factor 7 (T-cell specific, HMG-box)	CCDS4169.1	chr5_133473813-133473813_C_G	169P>A	Substitution	Nonsynonymous coding	33%
MM17T	TCF7	transcription factor 7 (T-cell specific, HMG-box)	CCDS4169.1	chr5_133478567-133478567_G_A	304G>D	Substitution	Nonsynonymous coding	36%
MM17T	TIMELESS	timeless circadian clock	CCDS8918.1	chr12_56817237-56817237_G_A	705R>X	Substitution	Nonsense	52%
MM17T	TP53	tumor protein p53	CCDS11118.1	chr17_7577570-7577570_C_A	237M>I	Substitution	Nonsynonymous coding	82%
MM17T	TTC29	tetratricopeptide repeat domain 29	CCDS47141.1	chr4_147830331-147830331_C_A	83E>X	Substitution	Nonsense	86%
MM17T	URB1	URB1 ribosome biogenesis 1 homolog (S. cerevisiae)	CCDS46645.1	chr21_33706593-33706593_C_G	1579S>T	Substitution	Nonsynonymous coding	42%
MM17T	URB1	URB1 ribosome biogenesis 1 homolog (S. cerevisiae)	CCDS46645.1	chr21_33721687-33721687_C_G	980E>Q	Substitution	Nonsynonymous coding	43%
MM17T	WDR72	WD repeat domain 72	CCDS10151.1	chr15_53998261-53998261_C_T	322R>H	Substitution	Nonsynonymous coding	47%
MM17T	ZBTB48	zinc finger and BTB domain containing 48	CCDS84.1	chr1_6647696-6647696_C_G	NA	Substitution	Splice site donor	16%
MM17T	ZNF17	zinc finger protein 17	CCDS42636.1	chr19_57932079-57932079__GT	NA	Insertion	Frameshift	21%
MM17T	ZNF41	zinc finger protein 41	CCDS14279.1	chrX_47315674-47315674_C_T	66V>M	Substitution	Nonsynonymous coding	51%
MM18T	MARCH3	membrane-associated ring finger (C3HC4) 3, E3 ubiquitin protein ligase	CCDS4141.1	chr5_126213945-126213945_G_A	179R>W	Substitution	Nonsynonymous coding	23%
MM18T	MARCH3	membrane-associated ring finger (C3HC4) 3, E3 ubiquitin protein ligase	CCDS4141.1	chr5_126253842-126253842_G_T	8H>N	Substitution	Nonsynonymous coding	13%
MM18T	MARCH6	membrane-associated ring finger (C3HC4) 6, E3 ubiquitin protein ligase	CCDS34135.1	chr5_10405770-10405770_G_A	478R>Q	Substitution	Nonsynonymous coding	17%
MM18T	MARCH6	membrane-associated ring finger (C3HC4) 6, E3 ubiquitin protein ligase	CCDS34135.1	chr5_10400949-10400949_G_T	323E>X	Substitution	Nonsense	13%
MM18T	MARCH8	membrane-associated ring finger (C3HC4) 8, E3 ubiquitin protein ligase	CCDS7213.1	chr10_45953743-45953743_T_G	274N>H	Substitution	Nonsynonymous coding	11%
MM18T	MARCH10	membrane-associated ring finger (C3HC4) 10, E3 ubiquitin protein ligase	CCDS11635.1	chr17_60814328-60814328_G_T	301L>M	Substitution	Nonsynonymous coding	11%
MM18T	SEPT3	septin 3	CCDS14027.2	chr22_42390736-42390736_G_T	343E>D	Substitution	Nonsynonymous coding	14%
MM18T	SEPT8	septin 8	CCDS43358.1	chr5_132099413-132099413_C_A	173K>N	Substitution	Nonsynonymous coding	13%
MM18T	SEPT11	septin 11	CCDS34018.1	chr4_77917591-77917591_G_A	14R>Q	Substitution	Nonsynonymous coding	15%
MM18T	SEPT12	septin 12	CCDS10522.1	chr16_4837574-4837574_C_T	25E>K	Substitution	Nonsynonymous coding	19%
MM18T	A2M	alpha-2-macroglobulin	CCDS44827.1	chr12_9254181-9254181_G_T	452F>L	Substitution	Nonsynonymous coding	11%
MM18T	A3GALT2P	alpha 1,3-galactosyltransferase 2, pseudogene	ENST00000330379	chr1_33772467-33772467_G_A	308S>L	Substitution	Nonsynonymous coding	10%
MM18T	A4GNT	alpha-1,4-N-acetylglucosaminyltransferase	CCDS3097.1	chr3_137849765-137849765_C_A	112D>Y	Substitution	Nonsynonymous coding	11%
MM18T	AACS	acetoacetyl-CoA synthetase	CCDS9263.1	chr12_125576071-125576071_T_C	ISV+2>	Substitution	Splice site donor	19%
MM18T	AADAT	aminoadipate aminotransferase	CCDS3814.1	chr4_171009605-171009605_T_C	60T>A	Substitution	Nonsynonymous coding	14%
MM18T	AARS2	alanyl-tRNA synthetase 2, mitochondrial	CCDS34464.1	chr6_44280914-44280914_G_T	49F>L	Substitution	Nonsynonymous coding	17%
MM18T	ABAT	4-aminobutyrate aminotransferase	CCDS10534.1	chr16_8866677-8866677_C_T	286T>M	Substitution	Nonsynonymous coding	15%
MM18T	ABCA1	ATP-binding cassette, sub-family A (ABC1), member 1	ENST00000423487	chr9_107618122-107618122_C_A	323R>I	Substitution	Nonsynonymous coding	17%
MM18T	ABCA12	ATP-binding cassette, sub-family A (ABC1), member 12	ENST00000412081	chr2_215940284-215940284_A_	NA	Deletion	Frameshift	14%

MM18T	ABCA12	ATP-binding cassette, sub-family A (ABC1), member 12	CCDS33372.1	chr2_215882767-215882767_A_C	583L>V	Substitution	Nonsynonymous coding	11%
MM18T	ABCA13	ATP-binding cassette, sub-family A (ABC1), member 13	CCDS47584.1	chr7_48314131-48314131_A_C	1623E>A	Substitution	Nonsynonymous coding	13%
MM18T	ABCA13	ATP-binding cassette, sub-family A (ABC1), member 13	CCDS47584.1	chr7_48318453-48318453_C_A	2554F>L	Substitution	Nonsynonymous coding	11%
MM18T	ABCA13	ATP-binding cassette, sub-family A (ABC1), member 13	CCDS47584.1	chr7_48391981-48391981_G_T	3529D>Y	Substitution	Nonsynonymous coding	13%
MM18T	ABCA13	ATP-binding cassette, sub-family A (ABC1), member 13	CCDS47584.1	chr7_48428697-48428697_T_C	3845V>A	Substitution	Nonsynonymous coding	10%
MM18T	ABCA13	ATP-binding cassette, sub-family A (ABC1), member 13	CCDS47584.1	chr7_48494730-48494730_G_A	4221R>H	Substitution	Nonsynonymous coding	22%
MM18T	ABCA2	ATP-binding cassette, sub-family A (ABC1), member 2	CCDS43909.1	chr9_139906995-139906995_G_A	1710S>L	Substitution	Nonsynonymous coding	27%
MM18T	ABCA3	ATP-binding cassette, sub-family A (ABC1), member 3	CCDS10466.1	chr16_2374412-2374412_G_T	147P>Q	Substitution	Nonsynonymous coding	10%
MM18T	ABCA4	ATP-binding cassette, sub-family A (ABC1), member 4	CCDS747.1	chr1_94461744-94461744_A_G	2246V>A	Substitution	Nonsynonymous coding	16%
MM18T	ABCA5	ATP-binding cassette, sub-family A (ABC1), member 5	CCDS11685.1	chr17_67282420-67282420_T_G	692K>Q	Substitution	Nonsynonymous coding	10%
MM18T	ABCA6	ATP-binding cassette, sub-family A (ABC1), member 6	CCDS11683.1	chr17_67129949-67129949_C_A	208K>N	Substitution	Nonsynonymous coding	12%
MM18T	ABCA7	ATP-binding cassette, sub-family A (ABC1), member 7	CCDS12055.1	chr19_1056372-1056372_T_C	1487V>A	Substitution	Nonsynonymous coding	12%
MM18T	ABCA7	ATP-binding cassette, sub-family A (ABC1), member 7	CCDS12055.1	chr19_1058181-1058181_T_G	1688F>V	Substitution	Nonsynonymous coding	10%
MM18T	ABCA8	ATP-binding cassette, sub-family A (ABC1), member 8	CCDS11680.1	chr17_66899515-66899515_G_A	802R>X	Substitution	Nonsense	11%
MM18T	ABCA9	ATP-binding cassette, sub-family A (ABC1), member 9	CCDS11681.1	chr17_66982315-66982315_C_T	1400A>T	Substitution	Nonsynonymous coding	16%
MM18T	ABCA9	ATP-binding cassette, sub-family A (ABC1), member 9	CCDS11681.1	chr17_67039853-67039853_C_T	193A>T	Substitution	Nonsynonymous coding	11%
MM18T	ABCA9	ATP-binding cassette, sub-family A (ABC1), member 9	CCDS11681.1	chr17_67047181-67047181_C_A	29Q>H	Substitution	Nonsynonymous coding	11%
MM18T	ABCB4	ATP-binding cassette, sub-family B (MDR/TAP), member 4	CCDS5606.1	chr7_87041263-87041263_C_T	957R>Q	Substitution	Nonsynonymous coding	13%
MM18T	ABCB4	ATP-binding cassette, sub-family B (MDR/TAP), member 4	CCDS5606.1	chr7_87047909-87047909_T_G	808N>H	Substitution	Nonsynonymous coding	11%
MM18T	ABCB5	ATP-binding cassette, sub-family B (MDR/TAP), member 5	NM_001163941	chr7_20685623-20685623__A	NA	Insertion	Frameshift	11%
MM18T	ABCB5	ATP-binding cassette, sub-family B (MDR/TAP), member 5	CCDS5371.1	chr7_20721180-20721180_G_A	142R>Q	Substitution	Nonsynonymous coding	15%
MM18T	ABCB5	ATP-binding cassette, sub-family B (MDR/TAP), member 5	CCDS5371.1	chr7_20721269-20721269_T_G	172Y>D	Substitution	Nonsynonymous coding	12%
MM18T	ABCB7	ATP-binding cassette, sub-family B (MDR/TAP), member 7	CCDS14428.1	chrX_74291502-74291502_C_A	351R>I	Substitution	Nonsynonymous coding	11%
MM18T	ABCB8	ATP-binding cassette, sub-family B (MDR/TAP), member 8	CCDS5913.1	chr7_150739111-150739111_G_A	561D>N	Substitution	Nonsynonymous coding	11%
MM18T	ABCB9	ATP-binding cassette, sub-family B (MDR/TAP), member 9	CCDS9241.1	chr12_123424795-123424795_C_T	536A>T	Substitution	Nonsynonymous coding	17%
MM18T	ABCC13	ATP-binding cassette, sub-family C (CFTR/MRP), member 13, pseudogene	ENST00000400574	chr21_15651966-15651966_C_A	39S>Y	Substitution	Nonsynonymous coding	13%
MM18T	ABCC2	ATP-binding cassette, sub-family C (CFTR/MRP), member 2	CCDS7484.1	chr10_101559106-101559106_T_G	337F>C	Substitution	Nonsynonymous coding	11%
MM18T	ABCC2	ATP-binding cassette, sub-family C (CFTR/MRP), member 2	CCDS7484.1	chr10_101595975-101595975_G_A	1181R>Q	Substitution	Nonsynonymous coding	15%
MM18T	ABCC2	ATP-binding cassette, sub-family C (CFTR/MRP), member 2	CCDS7484.1	chr10_101579014-101579014_G_T	870E>X	Substitution	Nonsense	21%
MM18T	ABCC3	ATP-binding cassette, sub-family C (CFTR/MRP), member 3	CCDS32681.1	chr17_48761085-48761085_G_T	1308D>Y	Substitution	Nonsynonymous coding	12%
MM18T	ABCC4	ATP-binding cassette, sub-family C (CFTR/MRP), member 4	CCDS9474.1	chr13_95686931-95686931_C_A	1266E>D	Substitution	Nonsynonymous coding	10%
MM18T	ABCC4	ATP-binding cassette, sub-family C (CFTR/MRP), member 4	CCDS9474.1	chr13_95724037-95724037_C_T	1030R>H	Substitution	Nonsynonymous coding	16%

MM18T	ABCC5	ATP-binding cassette, sub-family C (CFTR/MRP), member 5	CCDS43176.1	chr3_183670876-183670876_C_T	889G>R	Substitution	Nonsynonymous coding	37%
MM18T	ABCC6	ATP-binding cassette, sub-family C (CFTR/MRP), member 6	CCDS10568.1	chr16_16259697-16259697_C_T	1030R>Q	Substitution	Nonsynonymous coding	15%
MM18T	ABCC6	ATP-binding cassette, sub-family C (CFTR/MRP), member 6	CCDS10568.1	chr16_16276663-16276663_C_T	690E>K	Substitution	Nonsynonymous coding	26%
MM18T	ABCC6	ATP-binding cassette, sub-family C (CFTR/MRP), member 6	CCDS10568.1	chr16_16276663-16276663_C_A	690E>X	Substitution	Nonsense	14%
MM18T	ABCC8	ATP-binding cassette, sub-family C (CFTR/MRP), member 8	CCDS31437.1	chr11_17416722-17416722_G_T	1470L>I	Substitution	Nonsynonymous coding	14%
MM18T	ABCC9	ATP-binding cassette, sub-family C (CFTR/MRP), member 9	CCDS8693.1	chr12_21960410-21960410_G_A	1440A>V	Substitution	Nonsynonymous coding	11%
MM18T	ABCF3	ATP-binding cassette, sub-family F (GCN20), member 3	CCDS3254.1	chr3_183911466-183911466_C_T	704R>C	Substitution	Nonsynonymous coding	20%
MM18T	ABHD1	abhydrolase domain containing 1	CCDS1736.1	chr2_27351935-27351935_G_A	133G>D	Substitution	Nonsynonymous coding	10%
MM18T	ABHD10	abhydrolase domain containing 10	CCDS2963.1	chr3_111710335-111710335_T_G	230L>V	Substitution	Nonsynonymous coding	11%
MM18T	ABHD12	abhydrolase domain containing 12	CCDS13172.1	chr20_25282930-25282930_A_C	361F>C	Substitution	Nonsynonymous coding	11%
MM18T	ABHD12	abhydrolase domain containing 12	CCDS13172.1	chr20_25288634-25288634_G_A	279R>C	Substitution	Nonsynonymous coding	13%
MM18T	ABHD14A	abhydrolase domain containing 14A	CCDS2843.1	chr3_52014990-52014990_G_T	258D>Y	Substitution	Nonsynonymous coding	13%
MM18T	ABHD3	abhydrolase domain containing 3	CCDS32802.1	chr18_19236851-19236851_G_T	343S>Y	Substitution	Nonsynonymous coding	19%
MM18T	ABHD4	abhydrolase domain containing 4	CCDS9572.1	chr14_23072581-23072581_G_T	133E>D	Substitution	Nonsynonymous coding	10%
MM18T	ABI3	ABI family, member 3	CCDS11546.1	chr17_47299506-47299506_G_T	286D>Y	Substitution	Nonsynonymous coding	16%
MM18T	ABI3BP	ABI family, member 3 (NESH) binding protein	CCDS46880.1	chr3_100570788-100570788__AA	NA	Insertion	Splice site acceptor	21%
MM18T	ABLIM1	actin binding LIM protein 1	CCDS31289.1	chr10_116245085-116245085_G_A	50R>X	Substitution	Nonsense	14%
MM18T	ABTB1	ankyrin repeat and BTB (POZ) domain containing 1	CCDS3045.1	chr3_127399260-127399260_C_T	460A>V	Substitution	Nonsynonymous coding	17%
MM18T	AC006547.14	uncharacterized protein LOC388849 [Source:RefSeq peptide;Acc:NP_001230466]	ENST00000439765	chr22_20136278-20136278_G_T	320R>S	Substitution	Nonsynonymous coding	15%
MM18T	AC007431.1	Coiled-coil domain-containing protein ENSP00000299415 [Source:UniProtKB/Swiss-	ENST00000299415	chr17_55822561-55822561_T_A	25S>C	Substitution	Nonsynonymous coding	12%
MM18T	AC007557.1	HCG1816075; Uncharacterized protein [Source:UniProtKB/TrEMBL;Acc:C9J1J8]	ENST00000456163	chr2_217736269-217736269_G_T	75K>N	Substitution	Nonsynonymous coding	11%
MM18T	AC011239.1	Uncharacterized protein [Source:UniProtKB/TrEMBL;Acc:C9JNN6]	ENST00000430988	chr2_23746898-23746898__T	NA	Insertion	Frameshift	11%
MM18T	AC011239.1	Uncharacterized protein [Source:UniProtKB/TrEMBL;Acc:C9JNN6]	ENST00000430988	chr2_23746948-23746948__T	NA	Insertion	Frameshift	15%
MM18T	AC016251.1	-	ENST00000378936	chr15_96831553-96831553__T	NA	Insertion	Frameshift	13%
MM18T	AC016251.1	-	ENST00000378936	chr15_96831440-96831440_A_G	74V>A	Substitution	Nonsynonymous coding	14%
MM18T	AC092535.1	Uncharacterized protein [Source:UniProtKB/TrEMBL;Acc:E7EUI4]	ENST00000314552	chr4_1194815-1194815_C_A	77Q>H	Substitution	Nonsynonymous coding	15%
MM18T	AC103681.1	Uncharacterized protein [Source:UniProtKB/TrEMBL;Acc:H3BTB2]	ENST00000399269	chr11_45234685-45234685_C_T	17S>L	Substitution	Nonsynonymous coding	12%
MM18T	AC114273.1	-	ENST00000397365	chr19_16146303-16146303_C_T	115A>V	Substitution	Nonsynonymous coding	14%
MM18T	AC114273.1	-	ENST00000397365	chr19_16146384-16146384_C_A	142S>Y	Substitution	Nonsynonymous coding	13%
MM18T	AC131971.1	HCG1782999; PRO0943; Uncharacterized protein	ENST00000332881	chr11_3012326-3012326_A_T	21N>Y	Substitution	Nonsynonymous coding	13%
MM18T	ACAA1	acetyl-CoA acyltransferase 1	CCDS2673.1	chr3_38164573-38164573_C_A	414G>X	Substitution	Nonsense	15%
MM18T	ACACA	acetyl-CoA carboxylase alpha	CCDS42302.1	chr17_35518703-35518703_G_A	1781P>S	Substitution	Nonsynonymous coding	12%

MM18T	ACACA	acetyl-CoA carboxylase alpha	CCDS42302.1	chr17_35536280-35536280_T_C	1667D>G	Substitution	Nonsynonymous coding	14%
MM18T	ACACB	acetyl-CoA carboxylase beta	CCDS31898.1	chr12_109675039-109675039_C_T	1506R>C	Substitution	Nonsynonymous coding	10%
MM18T	ACAD10	acyl-CoA dehydrogenase family, member 10	CCDS44973.1	chr12_112143554-112143554_G_A	117V>M	Substitution	Nonsynonymous coding	11%
MM18T	ACAD10	acyl-CoA dehydrogenase family, member 10	CCDS44973.1	chr12_112165918-112165918_C_A	436A>D	Substitution	Nonsynonymous coding	13%
MM18T	ACAD9	acyl-CoA dehydrogenase family, member 9	CCDS3053.1	chr3_128622947-128622947_A_C	334K>T	Substitution	Nonsynonymous coding	11%
MM18T	ACADL	acyl-CoA dehydrogenase, long chain	CCDS2389.1	chr2_211082806-211082806_A_G	85V>A	Substitution	Nonsynonymous coding	12%
MM18T	ACAN	aggrecan	NM_013227	chr15_89417638-89417638_A_G	2507T>A	Substitution	Nonsynonymous coding	11%
MM18T	ACAP2	ArfGAP with coiled-coil, ankyrin repeat and PH domains 2	CCDS33924.1	chr3_195009904-195009904_C_T	707G>E	Substitution	Nonsynonymous coding	13%
MM18T	ACAT1	acetyl-CoA acetyltransferase 1	CCDS8339.1	chr11_108018049-108018049_G_T	406E>X	Substitution	Nonsense	15%
MM18T	ACBD3	acyl-CoA binding domain containing 3	CCDS1551.1	chr1_226340165-226340165_G_T	416L>I	Substitution	Nonsynonymous coding	12%
MM18T	ACIN1	apoptotic chromatin condensation inducer 1	CCDS9587.1	chr14_23530594-23530594_G_A	1171R>X	Substitution	Nonsense	18%
MM18T	ACMSD	aminocarboxymuconate semialdehyde decarboxylase	CCDS2173.2	chr2_135616903-135616903_C_T	59R>C	Substitution	Nonsynonymous coding	12%
MM18T	ACO2	aconitase 2, mitochondrial	CCDS14017.1	chr22_41919904-41919904_G_A	481D>N	Substitution	Nonsynonymous coding	15%
MM18T	ACOT11	acyl-CoA thioesterase 11	CCDS592.1	chr1_55073625-55073625_G_T	505V>F	Substitution	Nonsynonymous coding	20%
MM18T	ACOT8	acyl-CoA thioesterase 8	CCDS13378.1	chr20_44483804-44483804_G_A	86R>W	Substitution	Nonsynonymous coding	18%
MM18T	ACOX1	acyl-CoA oxidase 1, palmitoyl	CCDS11734.1	chr17_73947534-73947534_C_T	357E>K	Substitution	Nonsynonymous coding	17%
MM18T	ACPP	acid phosphatase, prostate	CCDS46916.1	chr3_132063884-132063884_G_T	254E>D	Substitution	Nonsynonymous coding	18%
MM18T	ACSBG2	acyl-CoA synthetase bubblegum family member 2	CCDS12159.1	chr19_6161277-6161277_C_T	187P>S	Substitution	Nonsynonymous coding	14%
MM18T	ACSF3	acyl-CoA synthetase family member 3	CCDS10974.1	chr16_89180770-89180770_C_T	334A>V	Substitution	Nonsynonymous coding	17%
MM18T	ACSL4	acyl-CoA synthetase long-chain family member 4	CCDS14548.1	chrX_108906520-108906520_G_T	542S>Y	Substitution	Nonsynonymous coding	12%
MM18T	ACSM1	acyl-CoA synthetase medium-chain family member 1	CCDS10587.1	chr16_20648741-20648741_C_A	383K>N	Substitution	Nonsynonymous coding	18%
MM18T	ACSM1	acyl-CoA synthetase medium-chain family member 1	CCDS10587.1	chr16_20673185-20673185_T_G	308K>T	Substitution	Nonsynonymous coding	15%
MM18T	ACSM4	acyl-CoA synthetase medium-chain family member 4	CCDS44825.1	chr12_7469857-7469857_G_T	249G>W	Substitution	Nonsynonymous coding	11%
MM18T	ACSS3	acyl-CoA synthetase short-chain family member 3	CCDS9022.1	chr12_81472080-81472080_G_A	61E>K	Substitution	Nonsynonymous coding	13%
MM18T	ACSS3	acyl-CoA synthetase short-chain family member 3	CCDS9022.1	chr12_81472195-81472195_C_T	99S>L	Substitution	Nonsynonymous coding	10%
MM18T	ACTC1	actin, alpha, cardiac muscle 1	CCDS10041.1	chr15_35086927-35086927_G_A	28A>V	Substitution	Nonsynonymous coding	13%
MM18T	ACTC1	actin, alpha, cardiac muscle 1	CCDS10041.1	chr15_35086973-35086973_C_T	13D>N	Substitution	Nonsynonymous coding	25%
MM18T	ACTN2	actinin, alpha 2	CCDS1613.1	chr1_236902707-236902707_C_T	328R>W	Substitution	Nonsynonymous coding	25%
MM18T	ACTN3	actinin, alpha 3 (gene/pseudogene)	NM_001104	chr11_66325538-66325538_A_C	390K>T	Substitution	Nonsynonymous coding	10%
MM18T	ACTR3	ARP3 actin-related protein 3 homolog (yeast)	CCDS33277.1	chr2_114714968-114714968_A_C	398K>T	Substitution	Nonsynonymous coding	14%
MM18T	ACTR5	ARP5 actin-related protein 5 homolog (yeast)	CCDS13308.1	chr20_37396225-37396225_C_T	518R>W	Substitution	Nonsynonymous coding	10%
MM18T	ACTR6	ARP6 actin-related protein 6 homolog (yeast)	CCDS9074.1	chr12_100617678-100617678_G_T	392E>D	Substitution	Nonsynonymous coding	14%

MM18T	ACTR8	ARP8 actin-related protein 8 homolog (yeast)	CCDS2875.1	chr3_53908375-53908375_C_T	310G>R	Substitution	Nonsynonymous coding	13%
MM18T	ACVR2A	activin A receptor, type IIA	CCDS33301.1	chr2_148684694-148684694_G_A	465D>N	Substitution	Nonsynonymous coding	14%
MM18T	ADAL	adenosine deaminase-like	CCDS32214.1	chr15_43639253-43639253_G_T	198A>S	Substitution	Nonsynonymous coding	16%
MM18T	ADAM12	ADAM metallopeptidase domain 12	CCDS7653.1	chr10_127789672-127789672_G_A	297R>C	Substitution	Nonsynonymous coding	11%
MM18T	ADAM12	ADAM metallopeptidase domain 12	CCDS7653.1	chr10_127967524-127967524_G_A	74R>W	Substitution	Nonsynonymous coding	12%
MM18T	ADAM12	ADAM metallopeptidase domain 12	CCDS7653.1	chr10_127727906-127727906_G_A	736R>X	Substitution	Nonsense	11%
MM18T	ADAM19	ADAM metallopeptidase domain 19	CCDS4338.1	chr5_156923927-156923927_C_A	523E>D	Substitution	Nonsynonymous coding	17%
MM18T	ADAM32	ADAM metallopeptidase domain 32	CCDS47846.1	chr8_39022331-39022331_G_A	215E>K	Substitution	Nonsynonymous coding	18%
MM18T	ADAM8	ADAM metallopeptidase domain 8	NM_001109	chr10_135085771-135085771_C_T	295D>N	Substitution	Nonsynonymous coding	18%
MM18T	ADAMTS12	ADAM metallopeptidase with thrombospondin type 1 motif, 12	CCDS34140.1	chr5_33588853-33588853_G_A	906R>X	Substitution	Nonsense	10%
MM18T	ADAMTS13	ADAM metallopeptidase with thrombospondin type 1 motif, 13	CCDS6970.1	chr9_136302931-136302931_G_T	500D>Y	Substitution	Nonsynonymous coding	26%
MM18T	ADAMTS17	ADAM metallopeptidase with thrombospondin type 1 motif, 17	CCDS10383.1	chr15_100882091-100882091_G_A	5A>V	Substitution	Nonsynonymous coding	20%
MM18T	ADAMTS2	ADAM metallopeptidase with thrombospondin type 1 motif, 2	CCDS4444.1	chr5_178541161-178541161_C_T	1115D>N	Substitution	Nonsynonymous coding	16%
MM18T	ADAMTS2	ADAM metallopeptidase with thrombospondin type 1 motif, 2	CCDS4444.1	chr5_178555079-178555079_T_G	833K>T	Substitution	Nonsynonymous coding	19%
MM18T	ADAMTS2	ADAM metallopeptidase with thrombospondin type 1 motif, 2	CCDS4444.1	chr5_178557092-178557092_C_A	766K>N	Substitution	Nonsynonymous coding	12%
MM18T	ADAMTS20	ADAM metallopeptidase with thrombospondin type 1 motif, 20	ENST00000389420	chr12_43822063-43822063_C_T	1309R>K	Substitution	Nonsynonymous coding	16%
MM18T	ADAMTS20	ADAM metallopeptidase with thrombospondin type 1 motif, 20	ENST00000389420	chr12_43840473-43840473_C_A	708D>Y	Substitution	Nonsynonymous coding	13%
MM18T	ADAMTS20	ADAM metallopeptidase with thrombospondin type 1 motif, 20	ENST00000389420	chr12_43944735-43944735_C_T	144V>I	Substitution	Nonsynonymous coding	21%
MM18T	ADAMTS4	ADAM metallopeptidase with thrombospondin type 1 motif, 4	CCDS1223.1	chr1_161166436-161166436_G_A	290R>C	Substitution	Nonsynonymous coding	10%
MM18T	ADAMTS5	ADAM metallopeptidase with thrombospondin type 1 motif, 5	CCDS13579.1	chr21_28296881-28296881_G_C	762Q>E	Substitution	Nonsynonymous coding	18%
MM18T	ADAMTS5	ADAM metallopeptidase with thrombospondin type 1 motif, 5	CCDS13579.1	chr21_28302208-28302208_T_G	741K>T	Substitution	Nonsynonymous coding	11%
MM18T	ADAMTS5	ADAM metallopeptidase with thrombospondin type 1 motif, 5	CCDS13579.1	chr21_28304330-28304330_G_T	681S>Y	Substitution	Nonsynonymous coding	11%
MM18T	ADAMTS5	ADAM metallopeptidase with thrombospondin type 1 motif, 5	CCDS13579.1	chr21_28306895-28306895_T_G	527M>L	Substitution	Nonsynonymous coding	12%
MM18T	ADAMTS6	ADAM metallopeptidase with thrombospondin type 1 motif, 6	CCDS3983.2	chr5_64769445-64769445_G_A	19S>L	Substitution	Nonsynonymous coding	15%
MM18T	ADAMTS9	ADAM metallopeptidase with thrombospondin type 1 motif, 9	CCDS2903.1	chr3_64607910-64607910_G_T	884H>N	Substitution	Nonsynonymous coding	16%
MM18T	ADAMTSL3	ADAMTS-like 3	CCDS10326.1	chr15_84683336-84683336_C_A	1339S>Y	Substitution	Nonsynonymous coding	13%
MM18T	ADAMTSL4	ADAMTS-like 4	CCDS955.1	chr1_150526083-150526083_G_A	206A>T	Substitution	Nonsynonymous coding	15%
MM18T	ADCY1	adenylate cyclase 1 (brain)	CCDS34631.1	chr7_45717801-45717801_G_A	613V>I	Substitution	Nonsynonymous coding	13%
MM18T	ADCY10	adenylate cyclase 10 (soluble)	CCDS1265.1	chr1_167793907-167793907_C_A	1313D>Y	Substitution	Nonsynonymous coding	13%
MM18T	ADCY10	adenylate cyclase 10 (soluble)	CCDS1265.1	chr1_167794068-167794068_G_A	1259S>L	Substitution	Nonsynonymous coding	12%
MM18T	ADCY6	adenylate cyclase 6	CCDS8767.1	chr12_49171008-49171008_G_T	419H>N	Substitution	Nonsynonymous coding	16%
MM18T	ADCY7	adenylate cyclase 7	CCDS10741.1	chr16_50322217-50322217_G_A	43A>T	Substitution	Nonsynonymous coding	11%

MM18T	ADCY9	adenylate cyclase 9	CCDS32382.1	chr16_4027612-4027612_G_A	900S>L	Substitution	Nonsynonymous coding	14%
MM18T	ADCY9	adenylate cyclase 9	CCDS32382.1	chr16_4057515-4057515_G_A	580R>C	Substitution	Nonsynonymous coding	18%
MM18T	ADCY9	adenylate cyclase 9	CCDS32382.1	chr16_4164759-4164759_C_T	229E>K	Substitution	Nonsynonymous coding	15%
MM18T	ADCY9	adenylate cyclase 9	CCDS32382.1	chr16_4165103-4165103_C_T	114R>H	Substitution	Nonsynonymous coding	23%
MM18T	ADCYAP1R1	adenylate cyclase activating polypeptide 1 (pituitary) receptor type I	CCDS5433.1	chr7_31125035-31125035_G_A	216S>N	Substitution	Nonsynonymous coding	11%
MM18T	ADD2	adducin 2 (beta)	CCDS1906.1	chr2_70922908-70922908_T_C	167H>R	Substitution	Nonsynonymous coding	15%
MM18T	ADH1A	alcohol dehydrogenase 1A (class I), alpha polypeptide	CCDS3648.1	chr4_100208784-100208784_C_A	19K>N	Substitution	Nonsynonymous coding	24%
MM18T	ADH1C	alcohol dehydrogenase 1C (class I), gamma polypeptide	NM_000669	chr4_100266053-100266053_G_A	178S>L	Substitution	Nonsynonymous coding	20%
MM18T	ADNP	activity-dependent neuroprotector homeobox	CCDS13433.1	chr20_49509883-49509883__A	NA	Insertion	Frameshift	12%
MM18T	ADNP	activity-dependent neuroprotector homeobox	CCDS13433.1	chr20_49510974-49510974_A_T	93F>I	Substitution	Nonsynonymous coding	13%
MM18T	ADNP2	ADNP homeobox 2	CCDS32853.1	chr18_77894001-77894001_G_T	235L>F	Substitution	Nonsynonymous coding	11%
MM18T	ADNP2	ADNP homeobox 2	CCDS32853.1	chr18_77895649-77895649_C_A	785L>I	Substitution	Nonsynonymous coding	18%
MM18T	ADORA2B	adenosine A2b receptor	CCDS11173.1	chr17_15878406-15878406_T_C	250V>A	Substitution	Nonsynonymous coding	14%
MM18T	ADORA3	adenosine A3 receptor	CCDS839.1	chr1_112042939-112042939_T_C	197Y>C	Substitution	Nonsynonymous coding	14%
MM18T	ADPGK	ADP-dependent glucokinase	CCDS42057.1	chr15_73045218-73045218_T_C	319T>A	Substitution	Nonsynonymous coding	12%
MM18T	ADPRH	ADP-ribosylarginine hydrolase	CCDS2990.1	chr3_119301022-119301022_G_T	2E>D	Substitution	Nonsynonymous coding	16%
MM18T	ADPRHL1	ADP-ribosylhydrolase like 1	CCDS9535.1	chr13_114107563-114107563_C_T	64A>T	Substitution	Nonsynonymous coding	21%
MM18T	ADPRM	ADP-ribose/CDP-alcohol diphosphatase, manganese-dependent	CCDS11159.2	chr17_10608701-10608701_T_C	153V>A	Substitution	Nonsynonymous coding	10%
MM18T	ADRA1B	adrenoceptor alpha 1B	CCDS4347.1	chr5_159399131-159399131_C_T	399R>C	Substitution	Nonsynonymous coding	16%
MM18T	ADRA2B	adrenoceptor alpha 2B	NM_000682	chr2_96781146-96781146_C_T	248G>E	Substitution	Nonsynonymous coding	19%
MM18T	ADRB2	adrenoceptor beta 2, surface	CCDS4292.1	chr5_148207005-148207005_C_T	204S>F	Substitution	Nonsynonymous coding	12%
MM18T	ADRB3	adrenoceptor beta 3	CCDS6099.1	chr8_37823695-37823695_G_A	98A>V	Substitution	Nonsynonymous coding	11%
MM18T	ADRBK2	adrenergic, beta, receptor kinase 2	CCDS13832.1	chr22_26118295-26118295_G_A	649E>K	Substitution	Nonsynonymous coding	18%
MM18T	AFAP1	actin filament associated protein 1	CCDS47010.1	chr4_7840334-7840334_G_C	215H>D	Substitution	Nonsynonymous coding	22%
MM18T	AFAP1	actin filament associated protein 1	CCDS47010.1	chr4_7873800-7873800_T_G	2E>D	Substitution	Nonsynonymous coding	15%
MM18T	AFF2	AF4/FMR2 family, member 2	CCDS14684.1	chrX_147733596-147733596_G_A	42D>N	Substitution	Nonsynonymous coding	12%
MM18T	AFF2	AF4/FMR2 family, member 2	CCDS14684.1	chrX_147743630-147743630_G_T	128D>Y	Substitution	Nonsynonymous coding	14%
MM18T	AFF2	AF4/FMR2 family, member 2	CCDS14684.1	chrX_148037779-148037779_C_T	735P>L	Substitution	Nonsynonymous coding	14%
MM18T	AFP	alpha-fetoprotein	CCDS3556.1	chr4_74306410-74306410_G_T	121R>I	Substitution	Nonsynonymous coding	13%
MM18T	AGAP1	ArfGAP with GTPase domain, ankyrin repeat and PH domain 1	CCDS33408.1	chr2_236653427-236653427_G_A	161R>Q	Substitution	Nonsynonymous coding	23%
MM18T	AGAP2	ArfGAP with GTPase domain, ankyrin repeat and PH domain 2	CCDS44932.1	chr12_58122154-58122154_C_T	855R>H	Substitution	Nonsynonymous coding	17%
MM18T	AGBL1	ATP/GTP binding protein-like 1	NM_152336	chr15_86697688-86697688_A_C	51E>A	Substitution	Nonsynonymous coding	15%

MM18T	AGBL1	ATP/GTP binding protein-like 1	NM_152336	chr15_86822871-86822871_A_T	647N>Y	Substitution	Nonsynonymous coding	12%
MM18T	AGBL3	ATP/GTP binding protein-like 3	CCDS47718.1	chr7_134678284-134678284_G_T	55Q>H	Substitution	Nonsynonymous coding	10%
MM18T	AGBL4	ATP/GTP binding protein-like 4	CCDS44137.1	chr1_50317178-50317178_C_A	16G>V	Substitution	Nonsynonymous coding	15%
MM18T	AGFG1	ArfGAP with FG repeats 1	CCDS46533.1	chr2_228395827-228395827_G_T	239D>Y	Substitution	Nonsynonymous coding	11%
MM18T	AGFG2	ArfGAP with FG repeats 2	CCDS5697.1	chr7_100161838-100161838_T_C	ISV-3>	Substitution	Splice site acceptor	16%
MM18T	AGGF1	angiogenic factor with G patch and FHA domains 1	CCDS4035.1	chr5_76330279-76330279_A_G	83N>D	Substitution	Nonsynonymous coding	11%
MM18T	AGL	amylo-alpha-1, 6-glucosidase, 4-alpha-glucanotransferase	CCDS759.1	chr1_100343220-100343220_G_T	483E>X	Substitution	Nonsense	21%
MM18T	AGPAT3	1-acylglycerol-3-phosphate O-acyltransferase 3	CCDS13703.1	chr21_45401032-45401032_C_T	336L>F	Substitution	Nonsynonymous coding	13%
MM18T	AGPAT4	1-acylglycerol-3-phosphate O-acyltransferase 4	CCDS5280.1	chr6_161587363-161587363_T_G	89N>H	Substitution	Nonsynonymous coding	15%
MM18T	AGTR1	angiotensin II receptor, type 1	CCDS3137.1	chr3_148459061-148459061_C_A	80T>N	Substitution	Nonsynonymous coding	11%
MM18T	AGTR1	angiotensin II receptor, type 1	CCDS3137.1	chr3_148459769-148459769_T_G	316L>R	Substitution	Nonsynonymous coding	11%
MM18T	AGTRAP	angiotensin II receptor-associated protein	ENST00000376627	chr1_11804618-11804618_A_G	19H>R	Substitution	Nonsynonymous coding	21%
MM18T	AHNAK	AHNAK nucleoprotein	CCDS31584.1	chr11_62286374-62286374_T_C	5172D>G	Substitution	Nonsynonymous coding	15%
MM18T	AHNAK	AHNAK nucleoprotein	CCDS31584.1	chr11_62285424-62285424_C_A	5489E>X	Substitution	Nonsense	14%
MM18T	AHNAK2	AHNAK nucleoprotein 2	CCDS45177.1	chr14_105420360-105420360_C_A	476Q>H	Substitution	Nonsynonymous coding	13%
MM18T	AHNAK2	AHNAK nucleoprotein 2	CCDS45177.1	chr14_105407021-105407021_C_A	4923E>X	Substitution	Nonsense	14%
MM18T	AHR	aryl hydrocarbon receptor	CCDS5366.1	chr7_17378642-17378642_G_A	398R>Q	Substitution	Nonsynonymous coding	24%
MM18T	AHR	aryl hydrocarbon receptor	CCDS5366.1	chr7_17378793-17378793_G_T	448K>N	Substitution	Nonsynonymous coding	16%
MM18T	AHSA2	AHA1, activator of heat shock 90kDa protein ATPase homolog 2 (yeast)	CCDS1868.1	chr2_61413802-61413802_G_T	99E>X	Substitution	Nonsense	18%
MM18T	AICDA	activation-induced cytidine deaminase	CCDS41747.1	chr12_8757943-8757943_G_A	99R>X	Substitution	Nonsense	17%
MM18T	AIF1	allograft inflammatory factor 1	CCDS4706.1	chr6_31583916-31583916_G_A	64D>N	Substitution	Nonsynonymous coding	12%
MM18T	AIM1L	absent in melanoma 1-like	ENST00000374207	chr1_26671971-26671971__T	NA	Insertion	Splice site acceptor	12%
MM18T	AIMP2	aminoacyl tRNA synthetase complex-interacting multifunctional protein 2	CCDS5344.1	chr7_6057640-6057640_G_T	180D>Y	Substitution	Nonsynonymous coding	14%
MM18T	AJAP1	adherens junctions associated protein 1	CCDS54.1	chr1_4832561-4832561_A_C	380Y>S	Substitution	Nonsynonymous coding	19%
MM18T	AK7	adenylate kinase 7	CCDS9945.1	chr14_96875212-96875212_A_C	144E>D	Substitution	Nonsynonymous coding	11%
MM18T	AK8	adenylate kinase 8	CCDS6954.1	chr9_135601125-135601125_C_T	464E>K	Substitution	Nonsynonymous coding	22%
MM18T	AK8	adenylate kinase 8	CCDS6954.1	chr9_135690035-135690035_C_A	323K>N	Substitution	Nonsynonymous coding	21%
MM18T	AK8	adenylate kinase 8	CCDS6954.1	chr9_135702409-135702409_C_T	197E>K	Substitution	Nonsynonymous coding	19%
MM18T	AKAP13	A kinase (PRKA) anchor protein 13	CCDS32320.1	chr15_86064750-86064750_G_A	42R>H	Substitution	Nonsynonymous coding	11%
MM18T	AKAP14	A kinase (PRKA) anchor protein 14	CCDS14591.1	chrX_119037322-119037322_G_A	46D>N	Substitution	Nonsynonymous coding	13%
MM18T	AKAP14	A kinase (PRKA) anchor protein 14	CCDS14591.1	chrX_119054485-119054485_G_A	170R>H	Substitution	Nonsynonymous coding	13%
MM18T	AKAP3	A kinase (PRKA) anchor protein 3	CCDS8531.1	chr12_4737307-4737307_G_A	254A>V	Substitution	Nonsynonymous coding	10%

MM18T	AKAP4	A kinase (PRKA) anchor protein 4	CCDS14329.1	chrX_49957082-49957082_A_G	761V>A	Substitution	Nonsynonymous coding	11%
MM18T	AKAP4	A kinase (PRKA) anchor protein 4	CCDS14329.1	chrX_49958696-49958696_C_T	223R>Q	Substitution	Nonsynonymous coding	11%
MM18T	AKAP6	A kinase (PRKA) anchor protein 6	CCDS9644.1	chr14_33165275-33165275_G_A	987D>N	Substitution	Nonsynonymous coding	19%
MM18T	AKAP6	A kinase (PRKA) anchor protein 6	CCDS9644.1	chr14_33293647-33293647_A_G	2210T>A	Substitution	Nonsynonymous coding	13%
MM18T	AKAP9	A kinase (PRKA) anchor protein (yotiao) 9	CCDS5622.1	chr7_91632526-91632526_C_A	1099L>I	Substitution	Nonsynonymous coding	14%
MM18T	AKAP9	A kinase (PRKA) anchor protein (yotiao) 9	CCDS5622.1	chr7_91726195-91726195_C_T	3308R>X	Substitution	Nonsense	11%
MM18T	AKD1	adenylate kinase domain containing 1	NM_001145128	chr6_109830545-109830545_A_C	1440L>V	Substitution	Nonsynonymous coding	16%
MM18T	AKD1	adenylate kinase domain containing 1	NM_001145128	chr6_109854609-109854609_G_A	1139R>C	Substitution	Nonsynonymous coding	13%
MM18T	AKR1B10	aldo-keto reductase family 1, member B10 (aldose reductase)	CCDS5832.1	chr7_134223753-134223753_G_A	298A>T	Substitution	Nonsynonymous coding	13%
MM18T	AKR1C2	aldo-keto reductase family 1, member C2	CCDS7062.1	chr10_5032201-5032201_G_T	320S>Y	Substitution	Nonsynonymous coding	21%
MM18T	AKR1C4	aldo-keto reductase family 1, member C4	CCDS7064.1	chr10_5258731-5258731_A_C	302N>H	Substitution	Nonsynonymous coding	13%
MM18T	AKT1	v-akt murine thymoma viral oncogene homolog 1	CCDS9994.1	chr14_105243030-105243030_C_T	85E>K	Substitution	Nonsynonymous coding	17%
MM18T	AKT2	v-akt murine thymoma viral oncogene homolog 2	CCDS12552.1	chr19_40741894-40741894_C_T	360E>K	Substitution	Nonsynonymous coding	13%
MM18T	AL359878.1	Uncharacterized protein [Source:UniProtKB/TrEMBL;Acc:E7EPV4]	ENST00000381466	chr10_1018696-1018696_G_T	59S>Y	Substitution	Nonsynonymous coding	14%
MM18T	ALAS1	aminolevulinate, delta-, synthase 1	CCDS2847.1	chr3_52233438-52233438_A_G	61T>A	Substitution	Nonsynonymous coding	17%
MM18T	ALDH1A2	aldehyde dehydrogenase 1 family, member A2	CCDS10163.1	chr15_58257949-58257949_G_T	292P>H	Substitution	Nonsynonymous coding	14%
MM18T	ALDH1A3	aldehyde dehydrogenase 1 family, member A3	CCDS10389.1	chr15_101445875-101445875_C_T	406R>W	Substitution	Nonsynonymous coding	11%
MM18T	ALDH1B1	aldehyde dehydrogenase 1 family, member B1	CCDS6615.1	chr9_38396571-38396571_G_A	276D>N	Substitution	Nonsynonymous coding	20%
MM18T	ALDH1L1	aldehyde dehydrogenase 1 family, member L1	CCDS3034.1	chr3_125824715-125824715_G_T	836S>Y	Substitution	Nonsynonymous coding	11%
MM18T	ALDH1L1	aldehyde dehydrogenase 1 family, member L1	CCDS3034.1	chr3_125879797-125879797_C_T	9S>N	Substitution	Nonsynonymous coding	10%
MM18T	ALDH1L2	aldehyde dehydrogenase 1 family, member L2	CCDS31891.1	chr12_105467784-105467784_C_A	ISV-1>	Substitution	Splice site acceptor	22%
MM18T	ALDH3A1	aldehyde dehydrogenase 3 family, member A1	CCDS11212.1	chr17_19641680-19641680_T_C	435N>D	Substitution	Nonsynonymous coding	17%
MM18T	ALDH3B2	aldehyde dehydrogenase 3 family, member B2	CCDS31622.1	chr11_67432881-67432881_T_C	194D>G	Substitution	Nonsynonymous coding	13%
MM18T	ALDH4A1	aldehyde dehydrogenase 4 family, member A1	CCDS188.1	chr1_19209899-19209899_C_A	159E>D	Substitution	Nonsynonymous coding	17%
MM18T	ALDOA	aldolase A, fructose-bisphosphate	CCDS10668.1	chr16_30078808-30078808_G_T	50E>D	Substitution	Nonsynonymous coding	14%
MM18T	ALG12	asparagine-linked glycosylation 12, alpha-1,6-mannosyltransferase homolog (S. cerevisiae)	CCDS14081.1	chr22_50298024-50298024_C_T	375A>T	Substitution	Nonsynonymous coding	14%
MM18T	ALG13	asparagine-linked glycosylation 13 homolog (S. cerevisiae)	ENST00000394780	chrX_110970087-110970087_C_T	594R>X	Substitution	Nonsense	19%
MM18T	ALG8	asparagine-linked glycosylation 8, alpha-1,3-glucosyltransferase homolog (S. cerevisiae)	CCDS8258.1	chr11_77825024-77825024_G_A	229R>X	Substitution	Nonsense	11%
MM18T	ALK	anaplastic lymphoma receptor tyrosine kinase	CCDS33172.1	chr2_29541180-29541180_G_A	546S>F	Substitution	Nonsynonymous coding	11%
MM18T	ALKBH1	alkB, alkylation repair homolog 1 (E. coli)	CCDS32127.1	chr14_78161109-78161109_G_T	143L>M	Substitution	Nonsynonymous coding	13%
MM18T	ALMS1	Alstrom syndrome 1	CCDS42697.1	chr2_73646425-73646425_C_A	209L>I	Substitution	Nonsynonymous coding	13%
MM18T	ALOX12B	arachidonate 12-lipoxygenase, 12R type	CCDS11129.1	chr17_7983234-7983234_C_A	260E>D	Substitution	Nonsynonymous coding	22%

MM18T	ALOX15	arachidonate 15-lipoxygenase	CCDS11049.1	chr17_4544895-4544895_A_G	18S>P	Substitution	Nonsynonymous coding	11%
MM18T	ALOX5AP	arachidonate 5-lipoxygenase-activating protein	CCDS9337.1	chr13_31309752-31309752_G_T	4E>X	Substitution	Nonsense	10%
MM18T	ALOXE3	arachidonate lipoxygenase 3	CCDS11130.1	chr17_8015456-8015456_C_A	247D>Y	Substitution	Nonsynonymous coding	11%
MM18T	ALOXE3	arachidonate lipoxygenase 3	CCDS11130.1	chr17_8019002-8019002_T_G	119R>S	Substitution	Nonsynonymous coding	14%
MM18T	ALPK2	alpha-kinase 2	CCDS11966.2	chr18_56203244-56203244_A_C	1392F>C	Substitution	Nonsynonymous coding	10%
MM18T	ALPK2	alpha-kinase 2	CCDS11966.2	chr18_56149272-56149272_C_A	ISV-1>	Substitution	Splice site acceptor	15%
MM18T	ALS2	amyotrophic lateral sclerosis 2 (juvenile)	CCDS42800.1	chr2_202609040-202609040_C_T	704R>Q	Substitution	Nonsynonymous coding	11%
MM18T	ALS2	amyotrophic lateral sclerosis 2 (juvenile)	CCDS42800.1	chr2_202626209-202626209_C_A	170E>X	Substitution	Nonsense	10%
MM18T	ALS2CL	ALS2 C-terminal like	CCDS2743.1	chr3_46723555-46723555_T_G	380N>T	Substitution	Nonsynonymous coding	17%
MM18T	ALS2CR11	amyotrophic lateral sclerosis 2 (juvenile) chromosome region, candidate 11	NM_001168221	chr2_202357715-202357715_A_C	1117F>V	Substitution	Nonsynonymous coding	15%
MM18T	AMBP	alpha-1-microglobulin/bikunin precursor	CCDS6800.1	chr9_116823712-116823712_C_T	282R>Q	Substitution	Nonsynonymous coding	10%
MM18T	AMER1	APC membrane recruitment protein 1	CCDS14377.2	chrX_63411216-63411216_C_T	651V>I	Substitution	Nonsynonymous coding	12%
MM18T	AMER1	APC membrane recruitment protein 1	CCDS14377.2	chrX_63411287-63411287_T_C	627E>G	Substitution	Nonsynonymous coding	14%
MM18T	AMER1	APC membrane recruitment protein 1	CCDS14377.2	chrX_63410358-63410358_C_A	937E>X	Substitution	Nonsense	12%
MM18T	AMER2	APC membrane recruitment protein 2	CCDS9312.1	chr13_25744461-25744461_C_T	314D>N	Substitution	Nonsynonymous coding	15%
MM18T	AMER3	APC membrane recruitment protein 3	CCDS2164.1	chr2_131521457-131521457_G_T	604E>D	Substitution	Nonsynonymous coding	14%
MM18T	AMIGO1	adhesion molecule with Ig-like domain 1	CCDS30795.1	chr1_110050105-110050105_G_A	477S>L	Substitution	Nonsynonymous coding	15%
MM18T	AMMECR1	Alport syndrome, mental retardation, midface hypoplasia and elliptocytosis chromosomal	CCDS14551.1	chrX_109507787-109507787_A_C	172L>V	Substitution	Nonsynonymous coding	12%
MM18T	AMOT	angiomotin	CCDS48154.1	chrX_112025831-112025831_C_T	726R>H	Substitution	Nonsynonymous coding	12%
MM18T	AMOT	angiomotin	CCDS48154.1	chrX_112048242-112048242_C_T	570R>Q	Substitution	Nonsynonymous coding	16%
MM18T	ANGEL1	angel homolog 1 (Drosophila)	CCDS9852.1	chr14_77272994-77272994_G_A	382S>L	Substitution	Nonsynonymous coding	13%
MM18T	ANGPTL4	angiopoietin-like 4	CCDS12200.1	chr19_8429458-8429458_G_T	85G>W	Substitution	Nonsynonymous coding	16%
MM18T	ANK1	ankyrin 1, erythrocytic	CCDS47849.1	chr8_41519453-41519453_G_A	1870R>C	Substitution	Nonsynonymous coding	11%
MM18T	ANK1	ankyrin 1, erythrocytic	CCDS47849.1	chr8_41566399-41566399_G_A	665S>L	Substitution	Nonsynonymous coding	19%
MM18T	ANK1	ankyrin 1, erythrocytic	CCDS47849.1	chr8_41573337-41573337_G_A	512R>C	Substitution	Nonsynonymous coding	12%
MM18T	ANK1	ankyrin 1, erythrocytic	CCDS47849.1	chr8_41547849-41547849_G_A	1375R>X	Substitution	Nonsense	12%
MM18T	ANK2	ankyrin 2, neuronal	CCDS3702.1	chr4_114277794-114277794__A	NA	Insertion	Frameshift	23%
MM18T	ANK2	ankyrin 2, neuronal	CCDS3702.1	chr4_114117612-114117612_C_A	92S>Y	Substitution	Nonsynonymous coding	21%
MM18T	ANK2	ankyrin 2, neuronal	CCDS3702.1	chr4_114274297-114274297_C_A	1508S>Y	Substitution	Nonsynonymous coding	21%
MM18T	ANK2	ankyrin 2, neuronal	CCDS3702.1	chr4_114275124-114275124_C_T	1784R>X	Substitution	Nonsense	19%
MM18T	ANK3	ankyrin 3, node of Ranvier (ankyrin G)	CCDS7258.1	chr10_61833191-61833191_G_A	2483S>L	Substitution	Nonsynonymous coding	14%
MM18T	ANK3	ankyrin 3, node of Ranvier (ankyrin G)	CCDS7258.1	chr10_61833429-61833429_G_A	2404P>S	Substitution	Nonsynonymous coding	14%

MM18T	ANKDD1A	ankyrin repeat and death domain containing 1A	CCDS10197.2	chr15_65235765-65235765_A_G	351N>S	Substitution	Nonsynonymous coding	13%
MM18T	ANKDD1A	ankyrin repeat and death domain containing 1A	ENST00000395720	chr15_65243168-65243168_G_T	ISV-1>	Substitution	Splice site acceptor	11%
MM18T	ANKHD1	ankyrin repeat and KH domain containing 1	CCDS4224.1	chr5_139908926-139908926_G_A	2132R>Q	Substitution	Nonsynonymous coding	11%
MM18T	ANKIB1	ankyrin repeat and IBR domain containing 1	CCDS47639.1	chr7_91957205-91957205_C_T	260R>X	Substitution	Nonsense	15%
MM18T	ANKK1	ankyrin repeat and kinase domain containing 1	CCDS44734.1	chr11_113270301-113270301_G_A	537R>Q	Substitution	Nonsynonymous coding	10%
MM18T	ANKK1	ankyrin repeat and kinase domain containing 1	CCDS44734.1	chr11_113270436-113270436_A_G	582Y>C	Substitution	Nonsynonymous coding	12%
MM18T	ANKRA2	ankyrin repeat, family A (RFXANK-like), 2	CCDS4020.1	chr5_72858681-72858681_A_C	9I>S	Substitution	Nonsynonymous coding	13%
MM18T	ANKRD11	ankyrin repeat domain 11	CCDS32513.1	chr16_89355030-89355030_G_A	217A>V	Substitution	Nonsynonymous coding	12%
MM18T	ANKRD13A	ankyrin repeat domain 13A	CCDS9140.1	chr12_110456221-110456221_G_A	158D>N	Substitution	Nonsynonymous coding	13%
MM18T	ANKRD17	ankyrin repeat domain 17	CCDS34004.1	chr4_73957962-73957962_C_T	1795D>N	Substitution	Nonsynonymous coding	11%
MM18T	ANKRD17	ankyrin repeat domain 17	CCDS34004.1	chr4_73985899-73985899_G_T	1335F>L	Substitution	Nonsynonymous coding	10%
MM18T	ANKRD24	ankyrin repeat domain 24	CCDS45925.1	chr19_4217303-4217303_G_T	716E>X	Substitution	Nonsense	23%
MM18T	ANKRD27	ankyrin repeat domain 27 (VPS9 domain)	CCDS32986.1	chr19_33089229-33089229_G_T	992S>Y	Substitution	Nonsynonymous coding	14%
MM18T	ANKRD27	ankyrin repeat domain 27 (VPS9 domain)	CCDS32986.1	chr19_33135253-33135253_C_A	168R>I	Substitution	Nonsynonymous coding	13%
MM18T	ANKRD32	ankyrin repeat domain 32	CCDS4071.2	chr5_93985303-93985303_G_T	ISV+1>	Substitution	Splice site donor	12%
MM18T	ANKRD33B	ankyrin repeat domain 33B	CCDS47191.1	chr5_10564856-10564856_G_A	93A>T	Substitution	Nonsynonymous coding	17%
MM18T	ANKRD35	ankyrin repeat domain 35	CCDS919.1	chr1_145562459-145562459_G_T	716R>M	Substitution	Nonsynonymous coding	18%
MM18T	ANKRD40	ankyrin repeat domain 40	CCDS11572.1	chr17_48774308-48774308_A_G	318L>S	Substitution	Nonsynonymous coding	20%
MM18T	ANKRD50	ankyrin repeat domain 50	CCDS34060.1	chr4_125631336-125631336_G_T	111H>N	Substitution	Nonsynonymous coding	15%
MM18T	ANKRD6	ankyrin repeat domain 6	CCDS47460.1	chr6_90315749-90315749_A_C	114E>D	Substitution	Nonsynonymous coding	12%
MM18T	ANKRD6	ankyrin repeat domain 6	CCDS47460.1	chr6_90334305-90334305_G_T	422E>D	Substitution	Nonsynonymous coding	15%
MM18T	ANKRD6	ankyrin repeat domain 6	CCDS47460.1	chr6_90340299-90340299_G_A	582R>Q	Substitution	Nonsynonymous coding	13%
MM18T	ANKS3	ankyrin repeat and sterile alpha motif domain containing 3	CCDS10520.1	chr16_4748037-4748037_C_T	584R>Q	Substitution	Nonsynonymous coding	19%
MM18T	ANKS6	ankyrin repeat and sterile alpha motif domain containing 6	CCDS43856.1	chr9_101518753-101518753_G_A	759R>W	Substitution	Nonsynonymous coding	23%
MM18T	ANKS6	ankyrin repeat and sterile alpha motif domain containing 6	CCDS43856.1	chr9_101533304-101533304_G_A	616P>S	Substitution	Nonsynonymous coding	25%
MM18T	ANKS6	ankyrin repeat and sterile alpha motif domain containing 6	CCDS43856.1	chr9_101552565-101552565_G_A	228P>L	Substitution	Nonsynonymous coding	24%
MM18T	ANLN	anillin, actin binding protein	CCDS5447.1	chr7_36446147-36446147_T_G	282L>W	Substitution	Nonsynonymous coding	13%
MM18T	ANO1	anoctamin 1, calcium activated chloride channel	CCDS44663.1	chr11_69978163-69978163_C_A	412F>L	Substitution	Nonsynonymous coding	20%
MM18T	ANO1	anoctamin 1, calcium activated chloride channel	CCDS44663.1	chr11_70031756-70031756_C_A	883F>L	Substitution	Nonsynonymous coding	18%
MM18T	ANO2	anoctamin 2	CCDS44807.1	chr12_5672609-5672609_C_A	951E>D	Substitution	Nonsynonymous coding	15%
MM18T	ANO5	anoctamin 5	CCDS31444.1	chr11_22291888-22291888_A_C	643K>N	Substitution	Nonsynonymous coding	11%
MM18T	ANO6	anoctamin 6	CCDS44865.1	chr12_45695862-45695862_C_T	46R>X	Substitution	Nonsense	13%

MM18T	ANTXR2	anthrax toxin receptor 2	CCDS47086.1	chr4_80954664-80954664_A_G	253V>A	Substitution	Nonsynonymous coding	11%
MM18T	AOX1	aldehyde oxidase 1	CCDS33360.1	chr2_201462156-201462156_T_G	79C>W	Substitution	Nonsynonymous coding	16%
MM18T	AOX1	aldehyde oxidase 1	CCDS33360.1	chr2_201469430-201469430_G_T	227E>D	Substitution	Nonsynonymous coding	13%
MM18T	AOX1	aldehyde oxidase 1	CCDS33360.1	chr2_201485455-201485455_A_C	596E>A	Substitution	Nonsynonymous coding	15%
MM18T	AP000322.54	Uncharacterized protein ENSP00000386791 [Source:UniProtKB/Swiss-Prot;Acc:B9A014]	ENST00000410005	chr21_35773287-35773287_T_G	28Q>H	Substitution	Nonsynonymous coding	18%
MM18T	AP1G2	adaptor-related protein complex 1, gamma 2 subunit	CCDS9602.1	chr14_24033573-24033573_G_A	316R>C	Substitution	Nonsynonymous coding	14%
MM18T	AP2M1	adaptor-related protein complex 2, mu 1 subunit	CCDS43177.1	chr3_183894799-183894799_C_A	6F>L	Substitution	Nonsynonymous coding	17%
MM18T	AP4M1	adaptor-related protein complex 4, mu 1 subunit	CCDS5685.1	chr7_99702696-99702696_T_G	234F>L	Substitution	Nonsynonymous coding	13%
MM18T	AP5M1	adaptor-related protein complex 5, mu 1 subunit	CCDS9729.1	chr14_57741311-57741311_C_A	142L>I	Substitution	Nonsynonymous coding	12%
MM18T	APAF1	apoptotic peptidase activating factor 1	CCDS9069.1	chr12_99042156-99042156_A_C	7N>H	Substitution	Nonsynonymous coding	13%
MM18T	APAF1	apoptotic peptidase activating factor 1	CCDS9069.1	chr12_99042224-99042224_G_A	29M>I	Substitution	Nonsynonymous coding	12%
MM18T	APBB1	amyloid beta (A4) precursor protein-binding, family B, member 1 (Fe65)	CCDS31410.1	chr11_6432178-6432178_C_A	134G>X	Substitution	Nonsense	16%
MM18T	APC	adenomatous polyposis coli	CCDS4107.1	chr5_112102945-112102945_C_T	94R>C	Substitution	Nonsynonymous coding	12%
MM18T	APC	adenomatous polyposis coli	CCDS4107.1	chr5_112174802-112174802_C_T	1171R>C	Substitution	Nonsynonymous coding	12%
MM18T	APC	adenomatous polyposis coli	CCDS4107.1	chr5_112179153-112179153_C_T	2621S>F	Substitution	Nonsynonymous coding	14%
MM18T	APC2	adenomatosis polyposis coli 2	CCDS12068.1	chr19_1453120-1453120_G_T	40E>D	Substitution	Nonsynonymous coding	13%
MM18T	APCDD1	adenomatosis polyposis coli down-regulated 1	CCDS11849.1	chr18_10485735-10485735_G_A	351V>I	Substitution	Nonsynonymous coding	12%
MM18T	APH1B	anterior pharynx defective 1 homolog B (C. elegans)	CCDS10184.1	chr15_63597847-63597847_C_T	214A>V	Substitution	Nonsynonymous coding	13%
MM18T	APOB	apolipoprotein B (including Ag(x) antigen)	CCDS1703.1	chr2_21225092-21225092_T_C	4401Y>C	Substitution	Nonsynonymous coding	14%
MM18T	APOB	apolipoprotein B (including Ag(x) antigen)	CCDS1703.1	chr2_21225917-21225917_A_C	4126I>S	Substitution	Nonsynonymous coding	10%
MM18T	APOB	apolipoprotein B (including Ag(x) antigen)	CCDS1703.1	chr2_21235214-21235214_G_T	1509P>H	Substitution	Nonsynonymous coding	15%
MM18T	APOBR	apolipoprotein B receptor	NM_018690	chr16_28507917-28507917_G_A	510D>N	Substitution	Nonsynonymous coding	17%
MM18T	APOF	apolipoprotein F	CCDS44923.1	chr12_56755611-56755611_G_T	127L>I	Substitution	Nonsynonymous coding	12%
MM18T	APOL4	apolipoprotein L, 4	ENST00000352371	chr22_36587520-36587520_T_C	219D>G	Substitution	Nonsynonymous coding	14%
MM18T	APPBP2	amyloid beta precursor protein (cytoplasmic tail) binding protein 2	CCDS32699.1	chr17_58525055-58525055_G_A	549R>W	Substitution	Nonsynonymous coding	11%
MM18T	APPL1	adaptor protein, phosphotyrosine interaction, PH domain and leucine zipper containing 1	CCDS2882.1	chr3_57274571-57274571_G_A	122D>N	Substitution	Nonsynonymous coding	14%
MM18T	APPL1	adaptor protein, phosphotyrosine interaction, PH domain and leucine zipper containing 1	CCDS2882.1	chr3_57283566-57283566_G_A	348D>N	Substitution	Nonsynonymous coding	21%
MM18T	APPL1	adaptor protein, phosphotyrosine interaction, PH domain and leucine zipper containing 1	CCDS2882.1	chr3_57303587-57303587_C_T	668R>W	Substitution	Nonsynonymous coding	16%
MM18T	APPL2	adaptor protein, phosphotyrosine interaction, PH domain and leucine zipper containing 2	CCDS9101.1	chr12_105583854-105583854_C_A	441E>X	Substitution	Nonsense	13%
MM18T	AQP6	aquaporin 6, kidney specific	CCDS31798.1	chr12_50369333-50369333_C_T	243A>V	Substitution	Nonsynonymous coding	14%
MM18T	AQR	aquarius homolog (mouse)	CCDS42013.1	chr15_35178842-35178842_A_G	901V>A	Substitution	Nonsynonymous coding	19%
MM18T	AQR	aquarius homolog (mouse)	CCDS42013.1	chr15_35210560-35210560_C_T	414R>Q	Substitution	Nonsynonymous coding	17%

MM18T	ARAP3	ArfGAP with RhoGAP domain, ankyrin repeat and PH domain 3	CCDS4266.1	chr5_141059977-141059977_C_T	26R>Q	Substitution	Nonsynonymous coding	11%
MM18T	ARF3	ADP-ribosylation factor 3	CCDS8774.1	chr12_49333438-49333438_C_A	128Q>H	Substitution	Nonsynonymous coding	13%
MM18T	ARFGEF1	ADP-ribosylation factor guanine nucleotide-exchange factor 1 (brefeldin A-inhibited)	CCDS6199.1	chr8_68163639-68163639_C_A	915M>I	Substitution	Nonsynonymous coding	11%
MM18T	ARFGEF1	ADP-ribosylation factor guanine nucleotide-exchange factor 1 (brefeldin A-inhibited)	CCDS6199.1	chr8_68213528-68213528_G_T	81F>L	Substitution	Nonsynonymous coding	15%
MM18T	ARHGAP10	Rho GTPase activating protein 10	CCDS34075.1	chr4_148743880-148743880_C_A	53L>M	Substitution	Nonsynonymous coding	18%
MM18T	ARHGAP17	Rho GTPase activating protein 17	CCDS32409.1	chr16_24980032-24980032_C_T	112E>K	Substitution	Nonsynonymous coding	15%
MM18T	ARHGAP17	Rho GTPase activating protein 17	CCDS32409.1	chr16_24981862-24981862_C_A	80E>X	Substitution	Nonsense	11%
MM18T	ARHGAP18	Rho GTPase activating protein 18	CCDS34535.1	chr6_129932722-129932722_T_C	360T>A	Substitution	Nonsynonymous coding	10%
MM18T	ARHGAP26	Rho GTPase activating protein 26	CCDS4277.1	chr5_142150451-142150451_A_G	42D>G	Substitution	Nonsynonymous coding	17%
MM18T	ARHGAP29	Rho GTPase activating protein 29	CCDS748.1	chr1_94652141-94652141_C_T	565R>Q	Substitution	Nonsynonymous coding	11%
MM18T	ARHGAP31	Rho GTPase activating protein 31	CCDS43135.1	chr3_119128609-119128609_C_T	638L>F	Substitution	Nonsynonymous coding	11%
MM18T	ARHGAP35	Rho GTPase activating protein 35	CCDS46127.1	chr19_47422062-47422062_C_T	44R>C	Substitution	Nonsynonymous coding	11%
MM18T	ARHGAP4	Rho GTPase activating protein 4	CCDS14736.1	chrX_153175280-153175280_C_T	777E>K	Substitution	Nonsynonymous coding	15%
MM18T	ARHGAP4	Rho GTPase activating protein 4	CCDS14736.1	chrX_153184284-153184284_A_G	ISV+2>	Substitution	Splice site donor	11%
MM18T	ARHGAP5	Rho GTPase activating protein 5	CCDS32062.1	chr14_32560623-32560623_C_T	250R>C	Substitution	Nonsynonymous coding	11%
MM18T	ARHGAP6	Rho GTPase activating protein 6	CCDS14140.1	chrX_11157461-11157461_G_A	816S>L	Substitution	Nonsynonymous coding	12%
MM18T	ARHGAP6	Rho GTPase activating protein 6	CCDS14140.1	chrX_11204422-11204422_G_T	403L>M	Substitution	Nonsynonymous coding	22%
MM18T	ARHGAP9	Rho GTPase activating protein 9	CCDS8941.2	chr12_57867923-57867923_T_C	607D>G	Substitution	Nonsynonymous coding	14%
MM18T	ARHGEF10	Rho guanine nucleotide exchange factor (GEF) 10	CCDS34794.1	chr8_1904965-1904965_G_A	1191V>I	Substitution	Nonsynonymous coding	22%
MM18T	ARHGEF17	Rho guanine nucleotide exchange factor (GEF) 17	CCDS8221.1	chr11_73021803-73021803_G_A	707R>H	Substitution	Nonsynonymous coding	11%
MM18T	ARHGEF26	Rho guanine nucleotide exchange factor (GEF) 26	CCDS46938.1	chr3_153840707-153840707_G_A	309R>Q	Substitution	Nonsynonymous coding	11%
MM18T	ARHGEF4	Rho guanine nucleotide exchange factor (GEF) 4	CCDS2165.1	chr2_131785535-131785535_C_A	149L>M	Substitution	Nonsynonymous coding	16%
MM18T	ARHGEF40	Rho guanine nucleotide exchange factor (GEF) 40	CCDS32041.1	chr14_21542204-21542204_C_A	105F>L	Substitution	Nonsynonymous coding	14%
MM18T	ARHGEF9	Cdc42 guanine nucleotide exchange factor (GEF) 9	CCDS35315.1	chrX_62893976-62893976_C_T	289R>Q	Substitution	Nonsynonymous coding	16%
MM18T	ARID1A	AT rich interactive domain 1A (SWI-like)	CCDS285.1	chr1_27087961-27087961_C_T	750R>X	Substitution	Nonsense	19%
MM18T	ARID2	AT rich interactive domain 2 (ARID, RFX-like)	CCDS31783.1	chr12_46245343-46245343_C_T	1146S>L	Substitution	Nonsynonymous coding	10%
MM18T	ARID3C	AT rich interactive domain 3C (BRIGHT-like)	CCDS35006.1	chr9_34623982-34623982_G_A	152R>C	Substitution	Nonsynonymous coding	15%
MM18T	ARID3C	AT rich interactive domain 3C (BRIGHT-like)	CCDS35006.1	chr9_34624014-34624014_G_A	141A>V	Substitution	Nonsynonymous coding	26%
MM18T	ARID4A	AT rich interactive domain 4A (RBP1-like)	CCDS9732.1	chr14_58831999-58831999_G_T	1064E>D	Substitution	Nonsynonymous coding	11%
MM18T	ARID4B	AT rich interactive domain 4B (RBP1-like)	CCDS31061.1	chr1_235345059-235345059_G_A	1059R>X	Substitution	Nonsense	11%
MM18T	ARID5B	AT rich interactive domain 5B (MRF1-like)	CCDS31208.1	chr10_63817051-63817051_G_A	341R>Q	Substitution	Nonsynonymous coding	19%
MM18T	ARL13B	ADP-ribosylation factor-like 13B	CCDS2925.1	chr3_93758725-93758725_A_C	231K>Q	Substitution	Nonsynonymous coding	24%

MM18T	ARMC10	armadillo repeat containing 10	CCDS5728.1	chr7_102724203-102724203_T_C	107S>P	Substitution	Nonsynonymous coding	13%
MM18T	ARMC10	armadillo repeat containing 10	CCDS5728.1	chr7_102737788-102737788_G_A	257R>H	Substitution	Nonsynonymous coding	11%
MM18T	ARMC2	armadillo repeat containing 2	CCDS5069.2	chr6_109294603-109294603_A_C	830K>N	Substitution	Nonsynonymous coding	10%
MM18T	ARMC8	armadillo repeat containing 8	NM_015396	chr3_137991953-137991953_C_T	528R>C	Substitution	Nonsynonymous coding	13%
MM18T	ARMCX2	armadillo repeat containing, X-linked 2	CCDS14490.1	chrX_100912562-100912562_G_A	5R>W	Substitution	Nonsynonymous coding	19%
MM18T	ARNT2	aryl-hydrocarbon receptor nuclear translocator 2	CCDS32307.1	chr15_80762626-80762626_G_A	88D>N	Substitution	Nonsynonymous coding	15%
MM18T	ARRB1	arrestin, beta 1	CCDS44684.1	chr11_74985252-74985252_G_C	260D>E	Substitution	Nonsynonymous coding	15%
MM18T	ARSD	arylsulfatase D	ENST00000458014	chrX_2823097-2823097_G_T	148L>M	Substitution	Nonsynonymous coding	15%
MM18T	ARSD	arylsulfatase D	CCDS35196.1	chrX_2836164-2836164_T_A	182N>Y	Substitution	Nonsynonymous coding	10%
MM18T	ARSI	arylsulfatase family, member I	CCDS34275.1	chr5_149676944-149676944_A_C	515F>V	Substitution	Nonsynonymous coding	14%
MM18T	ARSI	arylsulfatase family, member I	CCDS34275.1	chr5_149676878-149676878_G_A	537R>X	Substitution	Nonsense	18%
MM18T	ARVCF	armadillo repeat gene deleted in velocardiofacial syndrome	CCDS13771.1	chr22_19978248-19978248_C_T	24E>K	Substitution	Nonsynonymous coding	21%
MM18T	AS3MT	arsenic (+3 oxidation state) methyltransferase	CCDS41567.1	chr10_104629955-104629955_G_T	53E>X	Substitution	Nonsense	11%
MM18T	ASAP1	ArfGAP with SH3 domain, ankyrin repeat and PH domain 1	CCDS6362.1	chr8_131072826-131072826_G_A	1064T>M	Substitution	Nonsynonymous coding	15%
MM18T	ASAP1	ArfGAP with SH3 domain, ankyrin repeat and PH domain 1	CCDS6362.1	chr8_131193023-131193023_G_A	212R>C	Substitution	Nonsynonymous coding	14%
MM18T	ASAP2	ArfGAP with SH3 domain, ankyrin repeat and PH domain 2	CCDS1661.1	chr2_9498929-9498929_G_A	458E>K	Substitution	Nonsynonymous coding	12%
MM18T	ASB15	ankyrin repeat and SOCS box containing 15	CCDS34742.1	chr7_123276892-123276892_C_T	542R>W	Substitution	Nonsynonymous coding	10%
MM18T	ASB16	ankyrin repeat and SOCS box containing 16	CCDS11478.1	chr17_42254320-42254320_C_T	262R>X	Substitution	Nonsense	22%
MM18T	ASB7	ankyrin repeat and SOCS box containing 7	CCDS10387.1	chr15_101169792-101169792__A	NA	Insertion	Frameshift	12%
MM18T	ASCC2	activating signal cointegrator 1 complex subunit 2	CCDS13869.1	chr22_30200645-30200645_C_A	445E>D	Substitution	Nonsynonymous coding	17%
MM18T	ASCC3	activating signal cointegrator 1 complex subunit 3	CCDS5046.1	chr6_101086541-101086541_G_A	1353S>L	Substitution	Nonsynonymous coding	15%
MM18T	ASCC3	activating signal cointegrator 1 complex subunit 3	CCDS5046.1	chr6_101296046-101296046_C_A	260S>I	Substitution	Nonsynonymous coding	15%
MM18T	ASCC3	activating signal cointegrator 1 complex subunit 3	CCDS5046.1	chr6_101296131-101296131_T_G	232N>H	Substitution	Nonsynonymous coding	11%
MM18T	ASCC3	activating signal cointegrator 1 complex subunit 3	CCDS5046.1	chr6_100988267-100988267__A	NA	Insertion	Splice site acceptor	13%
MM18T	ASGR1	asialoglycoprotein receptor 1	CCDS11089.1	chr17_7077055-7077055_C_T	267D>N	Substitution	Nonsynonymous coding	12%
MM18T	ASIC2	acid-sensing (proton-gated) ion channel 2	CCDS11276.1	chr17_31355268-31355268_T_C	377H>R	Substitution	Nonsynonymous coding	15%
MM18T	ASPHD2	aspartate beta-hydroxylase domain containing 2	CCDS13834.2	chr22_26829870-26829870_G_A	97D>N	Substitution	Nonsynonymous coding	17%
MM18T	ASPM	asp (abnormal spindle) homolog, microcephaly associated (Drosophila)	CCDS1389.1	chr1_197061173-197061173_C_A	3103R>I	Substitution	Nonsynonymous coding	11%
MM18T	ASPSCR1	alveolar soft part sarcoma chromosome region, candidate 1	CCDS11796.1	chr17_79954361-79954361_C_A	191A>D	Substitution	Nonsynonymous coding	18%
MM18T	ATAD2	ATPase family, AAA domain containing 2	CCDS6343.1	chr8_124346560-124346560_G_A	1072R>C	Substitution	Nonsynonymous coding	17%
MM18T	ATAD2	ATPase family, AAA domain containing 2	CCDS6343.1	chr8_124359422-124359422_G_T	708L>I	Substitution	Nonsynonymous coding	11%
MM18T	ATAD2B	ATPase family, AAA domain containing 2B	CCDS46227.1	chr2_24021174-24021174_C_A	825R>I	Substitution	Nonsynonymous coding	11%

MM18T	ATAD2B	ATPase family, AAA domain containing 2B	CCDS46227.1	chr2_24103565-24103565_C_A	282R>I	Substitution	Nonsynonymous coding	14%
MM18T	ATAD2B	ATPase family, AAA domain containing 2B	CCDS46227.1	chr2_24108655-24108655_C_T	206R>Q	Substitution	Nonsynonymous coding	13%
MM18T	ATAD5	ATPase family, AAA domain containing 5	CCDS11260.1	chr17_29162886-29162886_G_T	596R>I	Substitution	Nonsynonymous coding	12%
MM18T	ATAD5	ATPase family, AAA domain containing 5	CCDS11260.1	chr17_29196283-29196283_G_T	1111D>Y	Substitution	Nonsynonymous coding	16%
MM18T	ATE1	arginyltransferase 1	CCDS31299.1	chr10_123670557-123670557_C_A	149E>D	Substitution	Nonsynonymous coding	17%
MM18T	ATE1	arginyltransferase 1	CCDS31299.1	chr10_123687384-123687384_C_A	27K>N	Substitution	Nonsynonymous coding	12%
MM18T	ATF6B	activating transcription factor 6 beta	CCDS4737.1	chr6_32083629-32083629_G_A	667R>X	Substitution	Nonsense	13%
MM18T	ATG2A	autophagy related 2A	CCDS31602.1	chr11_64675039-64675039_A_T	869W>R	Substitution	Nonsynonymous coding	10%
MM18T	ATG4A	autophagy related 4A, cysteine peptidase	CCDS14538.1	chrX_107374531-107374531_C_A	79L>I	Substitution	Nonsynonymous coding	13%
MM18T	ATG4B	autophagy related 4B, cysteine peptidase	CCDS46564.1	chr2_242598558-242598558_C_T	165A>V	Substitution	Nonsynonymous coding	14%
MM18T	ATG9A	autophagy related 9A	CCDS42820.1	chr2_220089154-220089154_G_T	313F>L	Substitution	Nonsynonymous coding	14%
MM18T	ATG9A	autophagy related 9A	CCDS42820.1	chr2_220092742-220092742_G_A	2A>V	Substitution	Nonsynonymous coding	11%
MM18T	ATL1	atlastin GTPase 1	CCDS9700.1	chr14_51079995-51079995_C_T	217R>X	Substitution	Nonsense	10%
MM18T	ATM	ataxia telangiectasia mutated	CCDS31669.1	chr11_108172519-108172519_C_A	ISV+3>	Substitution	Splice site donor	15%
MM18T	ATOH1	atonal homolog 1 (Drosophila)	CCDS3638.1	chr4_94750934-94750934_C_T	286S>L	Substitution	Nonsynonymous coding	23%
MM18T	ATP10B	ATPase, class V, type 10B	CCDS43394.1	chr5_160044894-160044894_G_A	835R>C	Substitution	Nonsynonymous coding	13%
MM18T	ATP11C	ATPase, class VI, type 11C	CCDS14668.1	chrX_138856897-138856897_C_T	726R>H	Substitution	Nonsynonymous coding	13%
MM18T	ATP11C	ATPase, class VI, type 11C	CCDS14668.1	chrX_138856924-138856924_C_T	717R>Q	Substitution	Nonsynonymous coding	12%
MM18T	ATP11C	ATPase, class VI, type 11C	CCDS14668.1	chrX_138879362-138879362_C_A	330K>N	Substitution	Nonsynonymous coding	11%
MM18T	ATP12A	ATPase, H+/K+ transporting, nongastric, alpha polypeptide	CCDS31948.1	chr13_25281329-25281329_G_T	780G>C	Substitution	Nonsynonymous coding	13%
MM18T	ATP12A	ATPase, H+/K+ transporting, nongastric, alpha polypeptide	CCDS31948.1	chr13_25255697-25255697_C_A	ISV-3>	Substitution	Splice site acceptor	15%
MM18T	ATP13A3	ATPase type 13A3	CCDS43187.1	chr3_194169210-194169210_C_T	376V>I	Substitution	Nonsynonymous coding	10%
MM18T	ATP13A3	ATPase type 13A3	CCDS43187.1	chr3_194180243-194180243_T_G	146K>Q	Substitution	Nonsynonymous coding	10%
MM18T	ATP2A1	ATPase, Ca++ transporting, cardiac muscle, fast twitch 1	CCDS10643.1	chr16_28911947-28911947_C_T	604R>C	Substitution	Nonsynonymous coding	11%
MM18T	ATP2B3	ATPase, Ca++ transporting, plasma membrane 3	CCDS35440.1	chrX_152814190-152814190_T_C	406C>R	Substitution	Nonsynonymous coding	17%
MM18T	ATP2B4	ATPase, Ca++ transporting, plasma membrane 4	CCDS1440.1	chr1_203682345-203682345_G_A	755R>Q	Substitution	Nonsynonymous coding	10%
MM18T	ATP2C1	ATPase, Ca++ transporting, type 2C, member 1	CCDS33856.1	chr3_130659476-130659476_A_C	121E>D	Substitution	Nonsynonymous coding	12%
MM18T	ATP2C1	ATPase, Ca++ transporting, type 2C, member 1	CCDS33856.1	chr3_130715617-130715617_T_G	740I>M	Substitution	Nonsynonymous coding	11%
MM18T	ATP4A	ATPase, H+/K+ exchanging, alpha polypeptide	CCDS12467.1	chr19_36046152-36046152_C_T	748A>T	Substitution	Nonsynonymous coding	17%
MM18T	ATP5O	ATP synthase, H+ transporting, mitochondrial F1 complex, O subunit	CCDS13634.1	chr21_35281461-35281461_G_A	85R>C	Substitution	Nonsynonymous coding	13%
MM18T	ATP6V0A1	ATPase, H+ transporting, lysosomal V0 subunit a1	CCDS45683.1	chr17_40642656-40642656_G_A	ISV+1>	Substitution	Splice site donor	11%
MM18T	ATP6V1B1	ATPase, H+ transporting, lysosomal 56/58kDa, V1 subunit B1	CCDS1912.1	chr2_71187083-71187083_C_A	154P>T	Substitution	Nonsynonymous coding	12%

MM18T	ATP7B	ATPase, Cu++ transporting, beta polypeptide	CCDS41892.1	chr13_52518279-52518279_G_A	1070P>L	Substitution	Nonsynonymous coding	17%
MM18T	ATP7B	ATPase, Cu++ transporting, beta polypeptide	ENST00000418097	chr13_52549510-52549510_T_G	7L>F	Substitution	Nonsynonymous coding	12%
MM18T	ATP8A2	ATPase, aminophospholipid transporter, class I, type 8A, member 2	CCDS41873.1	chr13_26129135-26129135_G_A	398D>N	Substitution	Nonsynonymous coding	10%
MM18T	ATP8A2	ATPase, aminophospholipid transporter, class I, type 8A, member 2	CCDS41873.1	chr13_26413750-26413750_G_T	982A>S	Substitution	Nonsynonymous coding	16%
MM18T	ATP8B2	ATPase, aminophospholipid transporter, class I, type 8B, member 2	CCDS1066.1	chr1_154316634-154316634_G_T	680A>S	Substitution	Nonsynonymous coding	12%
MM18T	ATP9A	ATPase, class II, type 9A	CCDS33489.1	chr20_50225134-50225134_A_G	890F>L	Substitution	Nonsynonymous coding	15%
MM18T	ATP9A	ATPase, class II, type 9A	CCDS33489.1	chr20_50273518-50273518_C_T	489E>K	Substitution	Nonsynonymous coding	13%
MM18T	ATPBD4	ATP binding domain 4	CCDS10043.1	chr15_35834638-35834638_T_G	32N>H	Substitution	Nonsynonymous coding	11%
MM18T	ATRNL1	attractin-like 1	CCDS7592.1	chr10_117045811-117045811_A_C	773K>N	Substitution	Nonsynonymous coding	12%
MM18T	ATRX	alpha thalassemia/mental retardation syndrome X-linked	CCDS14434.1	chrX_76949354-76949354_C_T	148S>N	Substitution	Nonsynonymous coding	15%
MM18T	ATXN1	ataxin 1	CCDS34342.1	chr6_16327415-16327415_G_A	376S>L	Substitution	Nonsynonymous coding	10%
MM18T	ATXN1	ataxin 1	CCDS34342.1	chr6_16328523-16328523_G_A	7R>W	Substitution	Nonsynonymous coding	12%
MM18T	ATXN10	ataxin 10	CCDS14070.1	chr22_46136386-46136386_C_A	381L>M	Substitution	Nonsynonymous coding	11%
MM18T	ATXN7L1	ataxin 7-like 1	CCDS47682.1	chr7_105255055-105255055_C_T	576A>T	Substitution	Nonsynonymous coding	16%
MM18T	ATXN7L1	ataxin 7-like 1	CCDS47682.1	chr7_105260693-105260693_C_T	491A>T	Substitution	Nonsynonymous coding	16%
MM18T	ATXN7L2	ataxin 7-like 2	CCDS30794.1	chr1_110029632-110029632_G_T	101R>I	Substitution	Nonsynonymous coding	15%
MM18T	AUTS2	autism susceptibility candidate 2	CCDS5539.1	chr7_70227938-70227938_G_T	275E>D	Substitution	Nonsynonymous coding	14%
MM18T	AUTS2	autism susceptibility candidate 2	ENST00000418686	chr7_70250444-70250444_C_T	17S>F	Substitution	Nonsynonymous coding	12%
MM18T	AWAT1	acyl-CoA wax alcohol acyltransferase 1	CCDS35321.1	chrX_69458071-69458071_C_A	157S>Y	Substitution	Nonsynonymous coding	18%
MM18T	AWAT2	acyl-CoA wax alcohol acyltransferase 2	NM_001002254	chrX_69263793-69263793_C_T	84D>N	Substitution	Nonsynonymous coding	11%
MM18T	AXDND1	axonemal dynein light chain domain containing 1	CCDS30948.1	chr1_179339165-179339165_A_C	109K>T	Substitution	Nonsynonymous coding	10%
MM18T	AXDND1	axonemal dynein light chain domain containing 1	CCDS30948.1	chr1_179414278-179414278_A_T	579E>D	Substitution	Nonsynonymous coding	13%
MM18T	AXIN2	axin 2	CCDS11662.1	chr17_63533512-63533512_C_T	548E>K	Substitution	Nonsynonymous coding	15%
MM18T	B3GNT3	UDP-GlcNAc:betaGal beta-1,3-N-acetylglucosaminyltransferase 3	CCDS12364.1	chr19_17922546-17922546_T_C	245I>T	Substitution	Nonsynonymous coding	12%
MM18T	B3GNT3	UDP-GlcNAc:betaGal beta-1,3-N-acetylglucosaminyltransferase 3	CCDS12364.1	chr19_17922765-17922765_G_A	318R>H	Substitution	Nonsynonymous coding	14%
MM18T	B3GNTL1	UDP-GlcNAc:betaGal beta-1,3-N-acetylglucosaminyltransferase-like 1	CCDS32778.1	chr17_81006600-81006600_A_G	41L>S	Substitution	Nonsynonymous coding	14%
MM18T	B4GALNT4	beta-1,4-N-acetyl-galactosaminyl transferase 4	CCDS7694.1	chr11_376474-376474_G_A	451E>K	Substitution	Nonsynonymous coding	11%
MM18T	B4GALT6	UDP-Gal:betaGlcNAc beta 1,4-galactosyltransferase, polypeptide 6	CCDS11900.1	chr18_29211016-29211016_C_T	228D>N	Substitution	Nonsynonymous coding	15%
MM18T	BACE1	beta-site APP-cleaving enzyme 1	CCDS8383.1	chr11_117161630-117161630_T_C	360T>A	Substitution	Nonsynonymous coding	23%
MM18T	BACH2	BTB and CNC homology 1, basic leucine zipper transcription factor 2	CCDS5026.1	chr6_90647965-90647965_A_C	647I>M	Substitution	Nonsynonymous coding	11%
MM18T	BAG1	BCL2-associated athanogene	ENST00000379700	chr9_33255071-33255071_C_A	239E>X	Substitution	Nonsense	18%
MM18T	BAHD1	bromo adjacent homology domain containing 1	CCDS10058.1	chr15_40751906-40751906_C_T	415L>F	Substitution	Nonsynonymous coding	15%

MM18T	BAI1	brain-specific angiogenesis inhibitor 1	NM_001702	chr8_143545998-143545998_C_T	147R>C	Substitution	Nonsynonymous coding	19%
MM18T	BAI1	brain-specific angiogenesis inhibitor 1	NM_001702	chr8_143546142-143546142_G_A	195A>T	Substitution	Nonsynonymous coding	18%
MM18T	BAI1	brain-specific angiogenesis inhibitor 1	NM_001702	chr8_143562960-143562960_C_A	673S>Y	Substitution	Nonsynonymous coding	11%
MM18T	BAI1	brain-specific angiogenesis inhibitor 1	NM_001702	chr8_143592337-143592337_G_A	907R>H	Substitution	Nonsynonymous coding	17%
MM18T	BAIAP2L2	BAI1-associated protein 2-like 2	CCDS43018.1	chr22_38494097-38494097_C_A	146K>N	Substitution	Nonsynonymous coding	20%
MM18T	BAIAP3	BAI1-associated protein 3	CCDS10434.1	chr16_1396066-1396066_C_G	798A>G	Substitution	Nonsynonymous coding	11%
MM18T	BAIAP3	BAI1-associated protein 3	CCDS10434.1	chr16_1396486-1396486_C_T	881S>L	Substitution	Nonsynonymous coding	14%
MM18T	BAP1	BRCA1 associated protein-1 (ubiquitin carboxy-terminal hydrolase)	CCDS2853.1	chr3_52438565-52438565_C_T	385R>Q	Substitution	Nonsynonymous coding	11%
MM18T	BARHL1	BarH-like homeobox 1	CCDS6950.1	chr9_135458378-135458378_G_T	65R>L	Substitution	Nonsynonymous coding	11%
MM18T	BARX2	BARX homeobox 2	CCDS8481.1	chr11_129306861-129306861_C_T	135R>W	Substitution	Nonsynonymous coding	15%
MM18T	BAZ1A	bromodomain adjacent to zinc finger domain, 1A	CCDS9651.1	chr14_35231013-35231013_G_T	1398S>Y	Substitution	Nonsynonymous coding	11%
MM18T	BAZ1B	bromodomain adjacent to zinc finger domain, 1B	CCDS5549.1	chr7_72865213-72865213_C_T	1182A>T	Substitution	Nonsynonymous coding	12%
MM18T	BAZ1B	bromodomain adjacent to zinc finger domain, 1B	CCDS5549.1	chr7_72892322-72892322_T_C	490D>G	Substitution	Nonsynonymous coding	14%
MM18T	BAZ2B	bromodomain adjacent to zinc finger domain, 2B	CCDS2209.2	chr2_160205276-160205276_G_T	1736L>I	Substitution	Nonsynonymous coding	10%
MM18T	BAZ2B	bromodomain adjacent to zinc finger domain, 2B	CCDS2209.2	chr2_160287570-160287570_C_A	666E>D	Substitution	Nonsynonymous coding	14%
MM18T	BAZ2B	bromodomain adjacent to zinc finger domain, 2B	CCDS2209.2	chr2_160295210-160295210_G_T	ISV-4>	Substitution	Splice site acceptor	13%
MM18T	BCAM	basal cell adhesion molecule (Lutheran blood group)	CCDS12644.1	chr19_45315790-45315790_G_T	163E>D	Substitution	Nonsynonymous coding	17%
MM18T	BCAM	basal cell adhesion molecule (Lutheran blood group)	CCDS12644.1	chr19_45322052-45322052_G_A	417D>N	Substitution	Nonsynonymous coding	13%
MM18T	BCAR3	breast cancer anti-estrogen resistance 3	CCDS745.1	chr1_94054613-94054613_C_T	284D>N	Substitution	Nonsynonymous coding	19%
MM18T	BCAS3	breast carcinoma amplified sequence 3	CCDS45749.1	chr17_59067401-59067401_G_A	431V>I	Substitution	Nonsynonymous coding	10%
MM18T	BCAS3	breast carcinoma amplified sequence 3	CCDS45749.1	chr17_59118161-59118161_A_G	661Q>R	Substitution	Nonsynonymous coding	15%
MM18T	BCHE	butyrylcholinesterase	CCDS3198.1	chr3_165547696-165547696_T_G	376K>Q	Substitution	Nonsynonymous coding	15%
MM18T	BCHE	butyrylcholinesterase	CCDS3198.1	chr3_165547828-165547828_C_A	332D>Y	Substitution	Nonsynonymous coding	10%
MM18T	BCL11A	B-cell CLL/lymphoma 11A (zinc finger protein)	CCDS1861.1	chr2_60679729-60679729_G_A	768S>L	Substitution	Nonsynonymous coding	12%
MM18T	BCL11B	B-cell CLL/lymphoma 11B (zinc finger protein)	CCDS9950.1	chr14_99640520-99640520_C_T	885D>N	Substitution	Nonsynonymous coding	17%
MM18T	BCL11B	B-cell CLL/lymphoma 11B (zinc finger protein)	CCDS9950.1	chr14_99641885-99641885_C_T	430E>K	Substitution	Nonsynonymous coding	10%
MM18T	BCMO1	beta-carotene 15,15'-monooxygenase 1	CCDS10934.1	chr16_81324156-81324156_G_A	540D>N	Substitution	Nonsynonymous coding	10%
MM18T	BCMO1	beta-carotene 15,15'-monooxygenase 1	CCDS10934.1	chr16_81319192-81319192_C_T	419R>X	Substitution	Nonsense	13%
MM18T	BCOR	BCL6 corepressor	CCDS48093.1	chrX_39914741-39914741_C_A	1541D>Y	Substitution	Nonsynonymous coding	14%
MM18T	BCOR	BCL6 corepressor	CCDS48093.1	chrX_39916545-39916545_C_A	1486K>N	Substitution	Nonsynonymous coding	14%
MM18T	BCOR	BCL6 corepressor	CCDS48093.1	chrX_39932663-39932663_A_C	646F>V	Substitution	Nonsynonymous coding	18%
MM18T	BCORL1	BCL6 corepressor-like 1	CCDS14616.1	chrX_129147028-129147028_G_A	94D>N	Substitution	Nonsynonymous coding	14%

MM18T	BCORL1	BCL6 corepressor-like 1	CCDS14616.1	chrX_129147479-129147479_C_T	244S>L	Substitution	Nonsynonymous coding	16%
MM18T	BCORL1	BCL6 corepressor-like 1	CCDS14616.1	chrX_129147629-129147629_C_T	294P>L	Substitution	Nonsynonymous coding	14%
MM18T	BCORL1	BCL6 corepressor-like 1	CCDS14616.1	chrX_129149171-129149171_C_T	808T>M	Substitution	Nonsynonymous coding	18%
MM18T	BCORL1	BCL6 corepressor-like 1	CCDS14616.1	chrX_129149995-129149995_C_T	1083R>W	Substitution	Nonsynonymous coding	12%
MM18T	BCORL1	BCL6 corepressor-like 1	CCDS14616.1	chrX_129159284-129159284_G_T	1336K>N	Substitution	Nonsynonymous coding	15%
MM18T	BCORL1	BCL6 corepressor-like 1	CCDS14616.1	chrX_129190082-129190082_G_A	1703E>K	Substitution	Nonsynonymous coding	11%
MM18T	BCR	breakpoint cluster region	CCDS13806.1	chr22_23613771-23613771_C_A	638S>Y	Substitution	Nonsynonymous coding	12%
MM18T	BCS1L	BC1 (ubiquinol-cytochrome c reductase) synthesis-like	CCDS2419.1	chr2_219526239-219526239_G_A	144R>Q	Substitution	Nonsynonymous coding	18%
MM18T	BEST3	bestrophin 3	CCDS8992.2	chr12_70087681-70087681_T_C	85Y>C	Substitution	Nonsynonymous coding	10%
MM18T	BFSP1	beaded filament structural protein 1, filensin	CCDS13126.1	chr20_17479512-17479512_C_A	303K>N	Substitution	Nonsynonymous coding	11%
MM18T	BFSP1	beaded filament structural protein 1, filensin	CCDS13126.1	chr20_17479597-17479597_C_T	275R>H	Substitution	Nonsynonymous coding	15%
MM18T	BFSP2	beaded filament structural protein 2, phakinin	CCDS33859.1	chr3_133166164-133166164_G_A	165G>S	Substitution	Nonsynonymous coding	14%
MM18T	BGN	biglycan	CCDS14721.1	chrX_152770773-152770773_C_T	106R>C	Substitution	Nonsynonymous coding	12%
MM18T	BHLHB9	basic helix-loop-helix domain containing, class B, 9	CCDS14502.1	chrX_102004767-102004767_C_T	282R>C	Substitution	Nonsynonymous coding	11%
MM18T	BHLHE22	basic helix-loop-helix family, member e22	CCDS6179.1	chr8_65494125-65494125_G_A	260D>N	Substitution	Nonsynonymous coding	20%
MM18T	BHLHE41	basic helix-loop-helix family, member e41	CCDS8706.1	chr12_26277054-26277054_T_C	62E>G	Substitution	Nonsynonymous coding	11%
MM18T	BICD1	bicaudal D homolog 1 (Drosophila)	CCDS8726.1	chr12_32481443-32481443_G_A	685R>Q	Substitution	Nonsynonymous coding	14%
MM18T	BICD2	bicaudal D homolog 2 (Drosophila)	CCDS35064.1	chr9_95481546-95481546_C_T	461E>K	Substitution	Nonsynonymous coding	29%
MM18T	BIN1	bridging integrator 1	CCDS2138.1	chr2_127864483-127864483_T_G	13K>Q	Substitution	Nonsynonymous coding	13%
MM18T	BIRC6	baculoviral IAP repeat containing 6	CCDS33175.2	chr2_32582533-32582533_C_A	102L>M	Substitution	Nonsynonymous coding	12%
MM18T	BLACE	B-cell acute lymphoblastic leukemia expressed	ENST00000378120	chr7_155150415-155150415_A_G	154S>P	Substitution	Nonsynonymous coding	13%
MM18T	BLM	Bloom syndrome, RecQ helicase-like	CCDS10363.1	chr15_91292957-91292957_T_G	153D>E	Substitution	Nonsynonymous coding	11%
MM18T	BLMH	bleomycin hydrolase	CCDS32604.1	chr17_28576142-28576142_C_T	421E>K	Substitution	Nonsynonymous coding	14%
MM18T	BLVRA	biliverdin reductase A	CCDS5472.1	chr7_43827506-43827506_G_A	6E>K	Substitution	Nonsynonymous coding	17%
MM18T	BLZF1	basic leucine zipper nuclear factor 1	CCDS1278.1	chr1_169356403-169356403_G_T	396E>X	Substitution	Nonsense	15%
MM18T	BMP1	bone morphogenetic protein 1	CCDS6026.1	chr8_22059429-22059429_G_A	741D>N	Substitution	Nonsynonymous coding	18%
MM18T	BMP10	bone morphogenetic protein 10	CCDS1890.1	chr2_69093047-69093047_C_T	331D>N	Substitution	Nonsynonymous coding	14%
MM18T	BMP15	bone morphogenetic protein 15	CCDS14334.1	chrX_50654009-50654009_C_T	76R>C	Substitution	Nonsynonymous coding	13%
MM18T	BMP15	bone morphogenetic protein 15	CCDS14334.1	chrX_50658940-50658940_C_A	171S>Y	Substitution	Nonsynonymous coding	11%
MM18T	BMP2K	BMP2 inducible kinase	CCDS47083.1	chr4_79754829-79754829_G_A	131E>K	Substitution	Nonsynonymous coding	11%
MM18T	BMP2K	BMP2 inducible kinase	CCDS47083.1	chr4_79832192-79832192_G_T	831D>Y	Substitution	Nonsynonymous coding	14%
MM18T	BMP3	bone morphogenetic protein 3	CCDS3588.1	chr4_81967011-81967011_T_C	146C>R	Substitution	Nonsynonymous coding	11%

MM18T	BMP3	bone morphogenetic protein 3	CCDS3588.1	chr4_81974568-81974568_C_T	433P>S	Substitution	Nonsynonymous coding	11%
MM18T	BMP5	bone morphogenetic protein 5	CCDS4958.1	chr6_55623865-55623865_C_A	385E>X	Substitution	Nonsense	16%
MM18T	BMP6	bone morphogenetic protein 6	CCDS4503.1	chr6_7880279-7880279_C_T	446S>F	Substitution	Nonsynonymous coding	11%
MM18T	BMP7	bone morphogenetic protein 7	CCDS13455.1	chr20_55841010-55841010_C_T	57E>K	Substitution	Nonsynonymous coding	17%
MM18T	BMPER	BMP binding endothelial regulator	CCDS5442.1	chr7_33976908-33976908_A_G	76E>G	Substitution	Nonsynonymous coding	13%
MM18T	BMPR1B	bone morphogenetic protein receptor, type IB	CCDS3642.1	chr4_96044973-96044973_G_A	121G>E	Substitution	Nonsynonymous coding	17%
MM18T	BNC2	basonuclin 2	CCDS6482.2	chr9_16436067-16436067_C_T	709D>N	Substitution	Nonsynonymous coding	11%
MM18T	BNC2	basonuclin 2	CCDS6482.2	chr9_16436736-16436736_G_A	486R>C	Substitution	Nonsynonymous coding	18%
MM18T	BOD1L1	biorientation of chromosomes in cell division 1-like 1	CCDS3411.2	chr4_13601673-13601673_G_A	2284A>V	Substitution	Nonsynonymous coding	17%
MM18T	BOP1	block of proliferation 1	CCDS6418.1	chr8_145512798-145512798_T_	NA	Deletion	Frameshift	16%
MM18T	BPGM	2,3-bisphosphoglycerate mutase	CCDS5833.1	chr7_134346662-134346662_G_T	135E>X	Substitution	Nonsense	15%
MM18T	BPHL	biphenyl hydrolase-like (serine hydrolase)	CCDS4483.2	chr6_3127510-3127510_G_T	82K>N	Substitution	Nonsynonymous coding	11%
MM18T	BPIFA1	BPI fold containing family A, member 1	CCDS13217.1	chr20_31828179-31828179_C_A	190S>Y	Substitution	Nonsynonymous coding	13%
MM18T	BPIFA3	BPI fold containing family A, member 3	CCDS13216.2	chr20_31812268-31812268_C_T	129P>L	Substitution	Nonsynonymous coding	15%
MM18T	BPIFB2	BPI fold containing family B, member 2	CCDS13210.1	chr20_31607497-31607497_G_A	341V>M	Substitution	Nonsynonymous coding	11%
MM18T	BPIFB4	BPI fold containing family B, member 4	CCDS13213.2	chr20_31671359-31671359_G_A	119R>Q	Substitution	Nonsynonymous coding	18%
MM18T	BPIFB4	BPI fold containing family B, member 4	CCDS13213.2	chr20_31677359-31677359_T_C	349V>A	Substitution	Nonsynonymous coding	16%
MM18T	BPIFB4	BPI fold containing family B, member 4	CCDS13213.2	chr20_31680349-31680349_C_A	410P>H	Substitution	Nonsynonymous coding	20%
MM18T	BPIFB4	BPI fold containing family B, member 4	CCDS13213.2	chr20_31685589-31685589_A_G	522D>G	Substitution	Nonsynonymous coding	13%
MM18T	BPIFC	BPI fold containing family C	CCDS13906.1	chr22_32811935-32811935_G_T	456F>L	Substitution	Nonsynonymous coding	22%
MM18T	BPTF	bromodomain PHD finger transcription factor	CCDS11673.1	chr17_65850859-65850859_T_G	473L>V	Substitution	Nonsynonymous coding	21%
MM18T	BPTF	bromodomain PHD finger transcription factor	CCDS11673.1	chr17_65942263-65942263_C_T	2480A>V	Substitution	Nonsynonymous coding	13%
MM18T	BPTF	bromodomain PHD finger transcription factor	CCDS11673.1	chr17_65971920-65971920_C_T	2858R>X	Substitution	Nonsense	12%
MM18T	BRAF	v-raf murine sarcoma viral oncogene homolog B1	CCDS5863.1	chr7_140434522-140434522_G_A	726R>C	Substitution	Nonsynonymous coding	13%
MM18T	BRAP	BRCA1 associated protein	CCDS9154.1	chr12_112117032-112117032_C_T	186E>K	Substitution	Nonsynonymous coding	10%
MM18T	BRCA2	breast cancer 2, early onset	CCDS9344.1	chr13_32905125-32905125_A_G	251T>A	Substitution	Nonsynonymous coding	14%
MM18T	BRCC3	BRCA1/BRCA2-containing complex, subunit 3	NM_024332	chrX_154305491-154305491_G_A	81R>Q	Substitution	Nonsynonymous coding	11%
MM18T	BRD1	bromodomain containing 1	CCDS14080.1	chr22_50169245-50169245_C_A	996R>I	Substitution	Nonsynonymous coding	13%
MM18T	BRD1	bromodomain containing 1	CCDS14080.1	chr22_50217672-50217672_T_G	98K>N	Substitution	Nonsynonymous coding	10%
MM18T	BRD3	bromodomain containing 3	ENST00000357885	chr9_136901714-136901714_C_A	ISV-1>	Substitution	Splice site acceptor	11%
MM18T	BRD4	bromodomain containing 4	CCDS12328.1	chr19_15378278-15378278_C_T	170E>K	Substitution	Nonsynonymous coding	11%
MM18T	BRDT	bromodomain, testis-specific	CCDS735.1	chr1_92445205-92445205_T_C	393I>T	Substitution	Nonsynonymous coding	13%

MM18T	BRE	brain and reproductive organ-expressed (TNFRSF1A modulator)	CCDS1764.1	chr2_28117449-28117449_G_A	9R>Q	Substitution	Nonsynonymous coding	11%
MM18T	BRIP1	BRCA1 interacting protein C-terminal helicase 1	CCDS11631.1	chr17_59793411-59793411_C_T	798R>Q	Substitution	Nonsynonymous coding	12%
MM18T	BRIX1	BRX1, biogenesis of ribosomes, homolog (S. cerevisiae)	CCDS34143.1	chr5_34925016-34925016_T_G	243I>S	Substitution	Nonsynonymous coding	16%
MM18T	BRSK1	BR serine/threonine kinase 1	CCDS12921.1	chr19_55816099-55816099_C_T	510R>C	Substitution	Nonsynonymous coding	11%
MM18T	BRWD3	bromodomain and WD repeat domain containing 3	CCDS14447.1	chrX_79978252-79978252_C_T	562R>H	Substitution	Nonsynonymous coding	11%
MM18T	BSN	bassoon presynaptic cytomatrix protein	CCDS2800.1	chr3_49693024-49693024_C_T	2012S>L	Substitution	Nonsynonymous coding	13%
MM18T	BSN	bassoon presynaptic cytomatrix protein	CCDS2800.1	chr3_49694869-49694869_G_A	2627R>H	Substitution	Nonsynonymous coding	26%
MM18T	BSN	bassoon presynaptic cytomatrix protein	CCDS2800.1	chr3_49697926-49697926_C_T	2883A>V	Substitution	Nonsynonymous coding	10%
MM18T	BSND	Bartter syndrome, infantile, with sensorineural deafness (Barttin)	CCDS602.1	chr1_55464947-55464947_C_T	30R>W	Substitution	Nonsynonymous coding	16%
MM18T	BTBD17	BTB (POZ) domain containing 17	CCDS32719.1	chr17_72353397-72353397_C_T	279G>D	Substitution	Nonsynonymous coding	22%
MM18T	BTBD3	BTB (POZ) domain containing 3	CCDS13113.1	chr20_11903641-11903641_G_A	299R>Q	Substitution	Nonsynonymous coding	13%
MM18T	BTD	biotinidase	CCDS2628.1	chr3_15686160-15686160_T_C	266V>A	Substitution	Nonsynonymous coding	14%
MM18T	BTF3	basic transcription factor 3	CCDS34185.1	chr5_72794527-72794527_C_A	12S>Y	Substitution	Nonsynonymous coding	15%
MM18T	BTG4	B-cell translocation gene 4	CCDS8346.1	chr11_111369417-111369417_C_A	29D>Y	Substitution	Nonsynonymous coding	21%
MM18T	BTK	Bruton agammaglobulinemia tyrosine kinase	CCDS14482.1	chrX_100611842-100611842_C_T	427V>M	Substitution	Nonsynonymous coding	14%
MM18T	BTNL3	butyrophilin-like 3	CCDS47358.1	chr5_180424336-180424336_A_G	174D>G	Substitution	Nonsynonymous coding	14%
MM18T	BTNL8	butyrophilin-like 8	CCDS43413.1	chr5_180335716-180335716_C_A	60F>L	Substitution	Nonsynonymous coding	17%
MM18T	BTNL9	butyrophilin-like 9	CCDS4460.2	chr5_180477348-180477348_G_A	239E>K	Substitution	Nonsynonymous coding	11%
MM18T	BZRAP1	benzodiazapine receptor (peripheral) associated protein 1	CCDS11605.1	chr17_56386344-56386344_C_T	1430R>Q	Substitution	Nonsynonymous coding	21%
MM18T	BZW1	basic leucine zipper and W2 domains 1	NM_014670	chr2_201678000-201678000_A_C	20K>N	Substitution	Nonsynonymous coding	13%
MM18T	C10orf137	chromosome 10 open reading frame 137	CCDS7646.1	chr10_127436523-127436523_G_A	988R>Q	Substitution	Nonsynonymous coding	15%
MM18T	C10orf54	chromosome 10 open reading frame 54	CCDS31218.1	chr10_73511609-73511609_T_G	238Q>H	Substitution	Nonsynonymous coding	12%
MM18T	C10orf67	chromosome 10 open reading frame 67	CCDS44365.1	chr10_23611384-23611384_G_T	138S>Y	Substitution	Nonsynonymous coding	19%
MM18T	C10orf76	chromosome 10 open reading frame 76	CCDS41563.1	chr10_103769157-103769157_G_A	310R>C	Substitution	Nonsynonymous coding	17%
MM18T	C10orf90	chromosome 10 open reading frame 90	CCDS31310.1	chr10_128193630-128193630_T_G	47K>Q	Substitution	Nonsynonymous coding	11%
MM18T	C11orf63	chromosome 11 open reading frame 63	CCDS8438.1	chr11_122828081-122828081_T_G	674L>X	Substitution	Nonsense	17%
MM18T	C11orf87	chromosome 11 open reading frame 87	CCDS31672.1	chr11_109294681-109294681_G_A	108G>S	Substitution	Nonsynonymous coding	24%
MM18T	C11orf87	chromosome 11 open reading frame 87	CCDS31672.1	chr11_109294802-109294802_C_T	148S>L	Substitution	Nonsynonymous coding	12%
MM18T	C12orf44	chromosome 12 open reading frame 44	CCDS8820.1	chr12_52467514-52467514_C_T	27T>M	Substitution	Nonsynonymous coding	18%
MM18T	C12orf55	chromosome 12 open reading frame 55	ENST00000298953	chr12_96883544-96883544_G_A	53E>K	Substitution	Nonsynonymous coding	12%
MM18T	C12orf66	chromosome 12 open reading frame 66	CCDS41803.1	chr12_64609464-64609464_C_A	172S>I	Substitution	Nonsynonymous coding	13%
MM18T	C12orf68	chromosome 12 open reading frame 68	CCDS31785.1	chr12_48578305-48578305_G_A	134D>N	Substitution	Nonsynonymous coding	23%

MM18T	C14orf159	chromosome 14 open reading frame 159	ENST00000298858	chr14_91633932-91633932_G_T	126R>I	Substitution	Nonsynonymous coding	16%
MM18T	C14orf37	chromosome 14 open reading frame 37	CCDS32089.1	chr14_58471800-58471800_C_T	741R>Q	Substitution	Nonsynonymous coding	15%
MM18T	C14orf37	chromosome 14 open reading frame 37	CCDS32089.1	chr14_58604792-58604792_C_T	429D>N	Substitution	Nonsynonymous coding	11%
MM18T	C15orf26	chromosome 15 open reading frame 26	CCDS42068.1	chr15_81430472-81430472_A_T	158K>I	Substitution	Nonsynonymous coding	14%
MM18T	C15orf39	chromosome 15 open reading frame 39	CCDS10276.1	chr15_75498695-75498695_G_T	102E>D	Substitution	Nonsynonymous coding	12%
MM18T	C15orf55	chromosome 15 open reading frame 55	CCDS32190.1	chr15_34640325-34640325_G_A	58D>N	Substitution	Nonsynonymous coding	11%
MM18T	C15orf59	chromosome 15 open reading frame 59	CCDS32289.1	chr15_74032947-74032947_C_T	65D>N	Substitution	Nonsynonymous coding	17%
MM18T	C16orf71	chromosome 16 open reading frame 71	CCDS10521.1	chr16_4790455-4790455_C_A	193S>Y	Substitution	Nonsynonymous coding	17%
MM18T	C16orf91	chromosome 16 open reading frame 91	CCDS32360.1	chr16_1479248-1479248_G_T	33A>D	Substitution	Nonsynonymous coding	14%
MM18T	C17orf104	chromosome 17 open reading frame 104	ENST00000409122	chr17_42750761-42750761_C_T	829R>C	Substitution	Nonsynonymous coding	11%
MM18T	C17orf104	chromosome 17 open reading frame 104	ENST00000409122	chr17_42750767-42750767_C_T	831R>W	Substitution	Nonsynonymous coding	11%
MM18T	C17orf112	chromosome 17 open reading frame 112	ENST00000441889	chr17_51063822-51063822_G_A	22M>I	Substitution	Nonsynonymous coding	13%
MM18T	C17orf112	chromosome 17 open reading frame 112	ENST00000441889	chr17_51064041-51064041_G_A	59G>D	Substitution	Nonsynonymous coding	15%
MM18T	C17orf112	chromosome 17 open reading frame 112	ENST00000441889	chr17_51064127-51064127_G_A	88G>R	Substitution	Nonsynonymous coding	17%
MM18T	C17orf47	chromosome 17 open reading frame 47	CCDS32691.1	chr17_56621153-56621153_C_T	132R>Q	Substitution	Nonsynonymous coding	16%
MM18T	C17orf47	chromosome 17 open reading frame 47	CCDS32691.1	chr17_56621281-56621281_C_A	89E>D	Substitution	Nonsynonymous coding	11%
MM18T	C17orf66	chromosome 17 open reading frame 66	CCDS11299.1	chr17_34190039-34190039_C_T	239R>Q	Substitution	Nonsynonymous coding	10%
MM18T	C17orf75	chromosome 17 open reading frame 75	NM_022344	chr17_30660476-30660476_G_T	312P>H	Substitution	Nonsynonymous coding	12%
MM18T	C17orf78	chromosome 17 open reading frame 78	CCDS45655.1	chr17_35743045-35743045_G_A	164E>K	Substitution	Nonsynonymous coding	14%
MM18T	C18orf54	chromosome 18 open reading frame 54	ENST00000382911	chr18_51888648-51888648_G_T	307D>Y	Substitution	Nonsynonymous coding	16%
MM18T	C19orf44	chromosome 19 open reading frame 44	CCDS12345.1	chr19_16611923-16611923_G_A	107R>Q	Substitution	Nonsynonymous coding	18%
MM18T	C19orf44	chromosome 19 open reading frame 44	CCDS12345.1	chr19_16620764-16620764_C_T	535T>M	Substitution	Nonsynonymous coding	19%
MM18T	C19orf68	chromosome 19 open reading frame 68	ENST00000328759	chr19_48699128-48699128_C_T	603R>C	Substitution	Nonsynonymous coding	15%
MM18T	C1orf109	chromosome 1 open reading frame 109	CCDS423.1	chr1_38151951-38151951_C_T	150R>Q	Substitution	Nonsynonymous coding	11%
MM18T	C1orf116	chromosome 1 open reading frame 116	CCDS1475.1	chr1_207198298-207198298_C_T	73E>K	Substitution	Nonsynonymous coding	14%
[truncated: 955,736 more chars]
